
JOURNAL INFORMATION
==============================
NLM Title Abbreviation: Insights Imaging
Journal ID: Insights Imaging
NLM Journal ID: 101532453

Insights into Imaging

EISSN: 1869-4101
Publisher: Springer

ARTICLE INFORMATION
==============================
PMCID: PMC10754764
DOI: 10.1186/s13244-023-01522-6
Article ID: 1522
Article version: 1
Subjects: Meeting Abstracts

ECR 2023 Book of Abstracts

Electronic publication date: 2023 Dec 21
Publication date: 2023 Dec
Volume: 14
Issue: Suppl 4
Issue sponsor: Publication of this supplement was funded by the European Congress of Radiology.
Electronic Location ID: 217
Copyright: © The Author(s) 2023
License: Open Access This article is licensed under a Creative Commons Attribution 4.0 International License, which permits use, sharing, adaptation, distribution and reproduction in any medium or format, as long as you give appropriate credit to the original author(s) and the source, provide a link to the Creative Commons licence, and indicate if changes were made. The images or other third party material in this article are included in the article's Creative Commons licence, unless indicated otherwise in a credit line to the material. If material is not included in the article's Creative Commons licence and your intended use is not permitted by statutory regulation or exceeds the permitted use, you will need to obtain permission directly from the copyright holder. To view a copy of this licence, visit http://creativecommons.org/licenses/by/4.0/.
License URL: https://creativecommons.org/licenses/by/4.0/

Conference name: European Congress of Radiology 2023
Conference location: Vienna, Austria
Conference date: 1-5 March 2023
issue-copyright-statement: © European Society of Radiology (ESR) 2023

==============================

INSIGHTS INTO IMAGING
VOLUME 14
SUPPLEMENT 4

EUROPEAN CONGRESS OF RADIOLOGY

ECR 2023

### Book of Abstracts

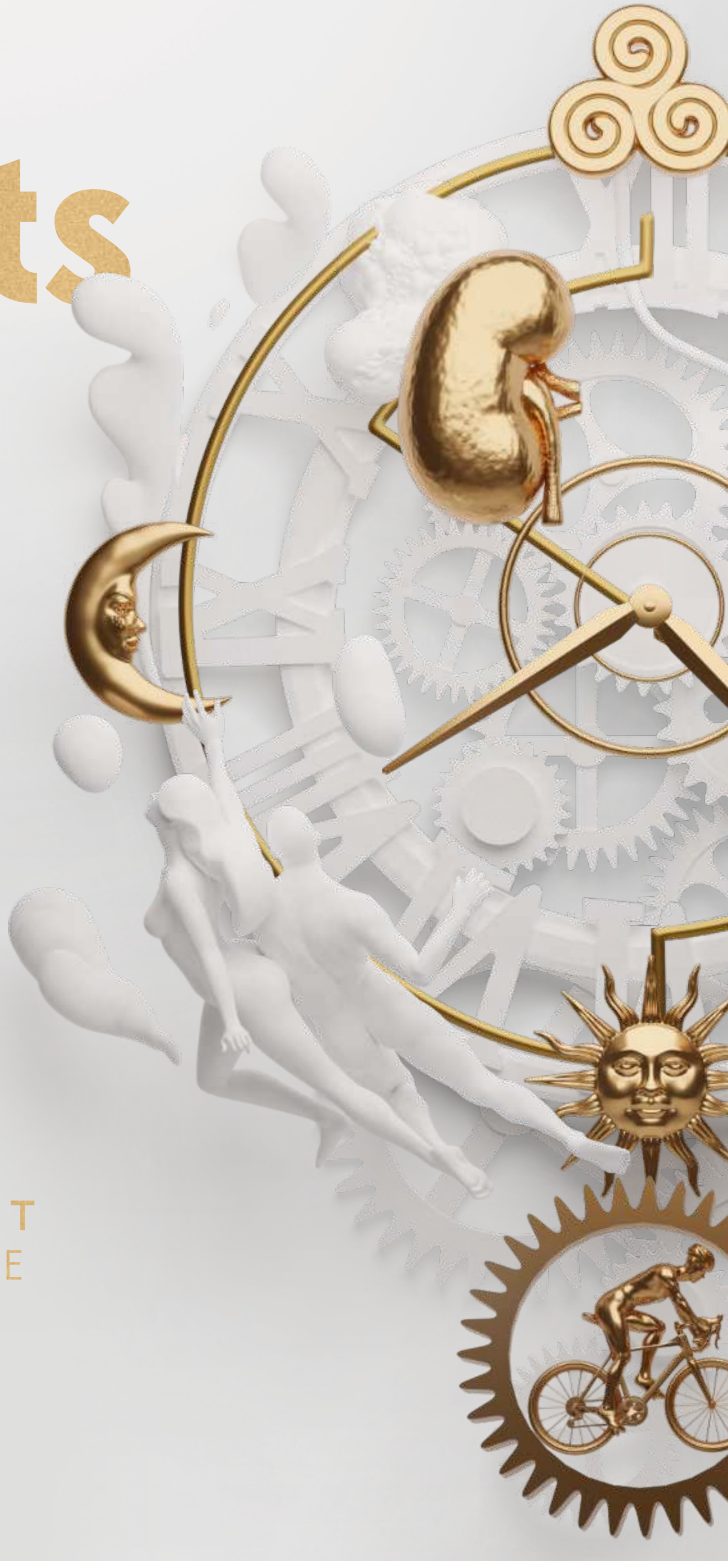

Contents

3 – 274 ECR 2023 ABSTRACT BASED PROGRAMME MARCH 1-5, 2023

EUROPEAN SOCIETY OF RADIOLOGY

Springer

**Disclaimer**

The ECR 2023 Book of Abstracts is published by the European Society of Radiology (ESR) and summarises the presentations accepted to be held at the European Congress of Radiology 2023 (Vienna, Austria, March 1 - March 5, 2023).

Abstracts were submitted by the authors warranting that good scientific practice, copyrights and data privacy regulations have been observed and relevant conflicts of interest declared.
Abstracts reflect the authors' opinions and knowledge. The ESR does not give any warranty about the accuracy or completeness of medical procedures, diagnostic procedures or treatments contained in the material included in this publication. The views and opinions presented in ECR abstracts and presentations, including scientific, educational and professional matters, do not necessarily reflect the views and opinions of the ESR.

In no event will the ESR be liable for any direct or indirect, special, incidental, consequential, punitive or exemplary damages arising from the use of these abstracts.

The Book of Abstracts and all of its component elements are for general educational purposes for health care professionals only and must not take the place of professional medical advice. Those seeking medical advice should always consult their physician or other medical professional.

In preparing this publication, every effort has been made to provide the most current, accurate, and clearly expressed information possible. Nevertheless, inadvertent errors in information can occur. The ESR is not responsible for typographical errors, accuracy, completeness or timeliness of the information contained in this publication.

The ECR 2023 Book of Abstracts is a supplement to Insights into Imaging (1869-4101) and published under Creative Commons Attribution 4.0 International License.

### ECR 2023
### Abstract-based Programme

Research Presentation Sessions (RPS)

**Wednesday, March 1 .................... 3**
**Thursday, March 2...................... 63**
**Friday, March 3 .........................118**
**Saturday, March 4 ......................173**
**Sunday, March 5........................232**

Insights Imaging (2023) Vol 14 (Suppl 4)
https://doi.org/10.1186/s13244-023-01522-6

### Wednesday, March 1

08:00-09:00 **Research Stage 1**

### Research Presentation Session: Artificial Intelligence & Machine Learning & Imaging Informatics

### RPS 105

### AI-enabled workflow optimisation in cardiac imaging

**Moderator**
T. Emrich; Mainz/DE
*(tilman.emrich@gmail.com)*
**Author Disclosures:**
Tilman Stephan Emrich: Advisory Board: "Photon Counting CT", Siemens Healthineers; Research Grant/Support: Siemens Healthineers; Speaker: Siemens Healthineers

### RPS 105-2

**Analysis of an AI-based prototype for user-independent automated cardiac MRI (CMR) scanning**

*A. S. Haenel*[1], J. Wetzl[2], S. S. Yoon[2], M. Schmidt[2], A-B. Weißgerber[1], J. Barkhausen[1], A. Frydrychowicz[1]; [1]Luebeck/DE, [2]Erlangen/DE
*(Alexander.Haenel@uksh.de)*

**Purpose:** Cardiac MRI (CMR) necessitates highly trained technicians, which are not available in all centres. Artificial intelligence- (AI) based automation could demonstrate a potential solution. The aim of this study was to introduce and evaluate a prototype for automated CMR data acquisition (AI cardiac scan companion; AICSC).
**Methods or Backround:** A total of 44 patients (52±16 years, 25f) referred to CMR for myocarditis (n=20), cardiomyopathy (n=21), or arrhythmogenic foci (n=2) were included. AICSC was initiated after positioning in a 1.5T MR scanner (MAGNETOM Sola, Siemens Healthcare, Erlangen, GER), placement of ECG-leads and entry of basic data (breathhold duration, choice of exam protocol). The exam protocol contained cine-bSSFP- and delayed-enhancement-(DGE)-sequences in longitudinal (2ch, 3ch, 4ch) and continuous short axes (SAX), T2w STIR SAX, T1-mapping before and after contrast administration (4ch and representative SAX). Safety-related user interaction (table motion, SAR limits) was not documented. AICSC scan success, necessary user interaction, and scan results were recorded.
**Results or Findings:** AICSC was successfully performed in 91% (n=40) exams without mandatory user interaction. In the heterogeneous patient collective (LVEDV 194±111ml [85-652ml], LVEF 48±15% [17-67], n=3 sternal wires, n=2 artificial heart valves, n=1 event recorder), mean heart rate was 75±19bpm at scan start. Total table time was 40±9 min (24-59). AICSC was stopped in n=1 exam due to large myocardial scarring and erroneous TI-scout and in n=3 exams due to aberrant scan axes following deviating cardiac anatomy. Erroneous breathing or ECG-registration resulted in optional interaction by the technicians.
**Conclusion:** AICSC was successfully introduced for automated CMR. It seems well-suited for scanning in situations without specifically trained technicians or for follow-up scanning such as in clinical scientific studies.
**Limitations:** Expectedly, there is room for further improvement, i.e., in situations with extensive myocardial scarring and in presence of complex cardiac anatomy.
**Ethics committee approval:** No information provided by the submitter.
**Funding for this study:** No funding was received for this study.
**Author Disclosures:**
Seung Su Yoon: Employee: Siemens Healthcare GmbH
Alexander Stephan Haenel: Nothing to disclose
Alex Frydrychowicz: Research/Grant Support: Siemens Healthcare GmbH
Jörg Barkhausen: Nothing to disclose
Michaela Schmidt: Employee: Siemens Healthcare GmbH
Alexandra-Bianca Weißgerber: Nothing to disclose
Jens Wetzl: Employee: Siemens Healthcare GmbH

### RPS 105-3

**Evaluation of zero-click whole-workflow of CMR cardiac function and strain analyses pipeline on cardio-oncology patients**

M. Watkins[1], A. Atteberry[1], V. Sridhar[2], X. Chen[3], A. Innanje[3], S. Sun[3], T. Chen[3], *G. M. Lanza*[1]; [1]St. Louis, MO/US, [2]Houston, TX/US, [3]Cambridge, MA/US
*(greg.lanza@mac.com)*

**Purpose:** The accuracy and reproducibility of CMR cardiac function and strain analyses from cine images are directly related to observer's experience. We evaluated a fully-automated deep-learning-powered analysis pipeline that starts from DICOMs and generates final report with no human intervention. The results were compared with human observers.
**Methods or Backround:** A total of 10 cardio-oncology patients were scanned on a 1.5T scanner. Routine clinical protocols were used to acquire the multiple sequences and images on a uMR 570 scanner (United Imaging Healthcare Co. Ltd, Shanghai). The zero-click analysis pipeline uses various neural networks to automate the whole process: detects cine images from other sequences; categorises short-axis and long-axis views; detects LV range from long-axis view and RV-LV insertion points to setup AHA 17 segment model; segments heart chambers and myocardium and calculates cardiac functional parameters; tracks LV myocardium and calculates myocardium strain parameters. Two experienced medical practitioners performed the analyses with manual editing.
**Results or Findings:** Agreement between human observers and automated pipeline on cardiac function was excellent (ICC 0.99, 0.98, 0.98; Pearson 0.99, 0.98, 0.98; for LV EDV, LV ESV, LV EF, respectively). The agreement on global circumferential, radial and longitudinal strain was also excellent (ICC 0.92, 0.85, 0.86; Pearson 0.90, 0.85, 0.87 for GCS, GRS, GLS, respectively). Within-subject coefficient of variations (WSCV) for human observers was 4.7%-16.7% for function analysis, and 14.1%-32.2% for strain analysis. WSCV for automated pipeline was 0%.
**Conclusion:** The proposed fully-automated pipeline, without any intervention, provides highly accurate and reproducible cardiac function and myocardium strain analyses compared to human observations. The proposed workflow shows the promise to be implemented into routine clinical practice to assist medical practitioners.
**Limitations:** Larger scale studies can help fortify the primary results.
**Ethics committee approval:** Ethics committee approval was received from the Washington University Institutional Review Board.
**Funding for this study:** No funding was received for this study.
**Author Disclosures:**
Shanhui Sun: Employee: United Imaging Intelligence
Arun Innanje: Employee: United Imaging Intelligence
Anne Atteberry: Employee: Washington University School of Medicine
Gregory Mark Lanza: Employee: Washington University School of Medicine
Xiao Chen: Employee: United Imaging Intelligence
Vidya Sridhar: Employee: United Imaging Healthcare America, Inc.
Terrence Chen: Employee: United Imaging Intelligence
Mary Watkins: Employee: Washington University School of Medicine

### RPS 105-4

**A deep learning-based approach to automatically choose a protocol of cardiothoracic CT examinations: understanding the decision process using a rate-distortion framework**

*M. Segeroth*[1], J. Haldimann[2], J. Vosshenrich[1], D. J. Winkel[1], J. Wasserthal[1], T. Heye[1]; [1]Basel/CH, [2]Hagen/DE
*(martin.segeroth@gmail.com)*

**Purpose:** To automate the protocolling process of cardiothoracic CT examinations based on clinical questions and to identify the most relevant terms used for the decision process by a neural network.
**Methods or Backround:** A total of 66,173 consecutive CT examinations performed in our cardiothoracic imaging section from January 2010 to October 2021 were included. Protocol assignment performed during clinical routine as one of 30 standardised cardiothoracic imaging protocols served as ground-truth. The 1,000 most common terms were extracted from all clinical questions, serving as input for a deep neuronal network. This was trained for 100 epochs on 80% of the examinations. The remaining 20% were used for validation. A rate-distortion framework, explaining neuronal network decisions, was employed to assess the most important terms for automated protocol assignment. A term was regarded as important if distortion (change of the neuronal network prediction using squared distance) increased strongly when ignoring it.
**Results or Findings:** The trained neuronal network had an 87% accuracy on the training dataset and a 61% accuracy on the validation dataset. Accuracy of automated CT protocol assignment by the neural network in the validation dataset was as follows: non-contrast chest CT (n=3,639) = 79%; CT pulmonary angiogram (n=2,897) = 88%; contrast-enhanced chest CT in arterial phase (n=2,650) = 44%; contrast-enhanced CT chest/abdomen in venous phase (n=549) = 48%; ECG-gated CT-angiogram chest/abdomen (n=619) = 66%. Using a rate-distortion framework, the clinical question "rule out infiltrate" led to the assignment of a non-contrast chest CT protocol, and "infiltrate" was marked as important. The terms "angiography", "exclusion" and "pulmonary embolism" were marked as important for the assignment of the CT pulmonary angiogram protocol.
**Conclusion:** Preliminary results from this neuronal network may support the CT protocolling process given its comprehensible decision process based on important key terms.
**Limitations:** The retrospective nature of this study was an identified limitation.
**Ethics committee approval:** No information provided by the submitter.

**Funding for this study:** No funding was received for this study.
**Author Disclosures:**
Tobias Heye: Nothing to disclose
David Jean Winkel: Nothing to disclose
Jacob Wasserthal: Nothing to disclose
Martin Segeroth: Nothing to disclose
Jonas Haldimann: Nothing to disclose
Jan Vosshenrich: Nothing to disclose

### RPS 105-5

**Automated segment-level coronary artery calcium scoring on non-contrast CT using deep learning**

*B. Föllmer*, F. Biavati, M. Dewey; Berlin/DE
*(bernhard.foellmer@charite.de)*

**Purpose:** To develop and evaluate, a deep learning model for automated segment-level coronary artery calcium (CAC) scoring in non-contrast enhanced cardiac CT for precise localisation and quantification of calcifications in the coronary artery tree.
**Methods or Backround:** Current automated CAC scoring methods detect calcifications only on the vessel level. However, recent works suggest integrating information about precise location and distribution of calcification in the proximal, medial and distal segments improves the prognostic value. We developed a multi-task neural network performing three tasks: 1) coronary artery region segmentation, 2) segmentation of CAC on the vessel level, and 3) segmentation on the segment level. We randomly selected 400 non-contrast CTs from the multicentre DISCHARGE trial, excluding patients with metal artifacts, and randomly divided the patients into a training/validation set (200) and a test set (200) for evaluation.
**Results or Findings:** Of 694 correctly identified calcifications located in 114 (57%) patients of the test set, 647 (93.2%) were assigned to the correct vessel and 451 (65.0%) were assigned to the correct segment. The agreement between the model and the reference standard was excellent (weighted κ: 0.90 [95% CI 0.88-0.91]) for classifying calcification into the correct vessel class and good (weighted κ: 0.83 [95% CI 0.82-0.85]) for the correct segment class.
**Conclusion:** Automated segment-level CAC scoring using a multi-task neural network showed good agreement with human reference readings, indicating that deep learning holds promise to facilitate quantification of calcifications on the segment level.
**Limitations:** An identified limitation was that the reference standard was created by only one observer.
**Ethics committee approval:** No information provided by the submitter.
**Funding for this study:** This work was funded by the German Research Foundation through the graduate program BIOQIC (GRK2260, project-ID: 289347353), the priority program SPP-Radiomics (SPP2177, project-ID: 402688427) and the DISCHARGE project (603266-2, HEALTH-2012.2.4.-2) funded by the FP7 Program of the European Commission.
**Author Disclosures:**
Marc Dewey: Other: Per the guiding principles of ESR, the work as Research and Publications Chair is on a voluntary basis and only remuneration of travel expenses occurs. Prof. Dewey is also the editor of Cardiac CT, published by Springer Nature, and offers hands-on courses on CT imaging (www.ct-kurs.de). Research/Grant Support: Fractal analysis of myocardial perfusion (DE 1361/18-1, DFG project number: 392304398), DFG Priority Programme Radiomics (DFG project number: 402688427) for the investigation of coronary plaque and coronary flow (DE 1361/19-1 [DFG project number: 428222922] and DE 1361/20-1 [DFG project number: 428223139] in SPP 2177/1). Research/Grant Support: FP7 Program of the European Commission for the randomized multicenter DISCHARGE trial (EC-GA 603266 in HEALTH.2013.2.4.2-2), German Research Foundation (DFG) in the Heisenberg Program (DE 1361/14-1, DFG project number: 213705389), graduate program on quantitative biomedical imaging (BIOQIC, GRK 2260/1, DFG project number: 289347353), Equipment Support Recipient: Institutional master research agreements exist with Siemens, General Electric, Philips, and Canon. The terms of these arrangements are managed by the legal department of Charité – Universitätsmedizin Berlin. Professor Patent Holder: Dewey holds a joint patent with Florian Michallek on dynamic perfusion analysis using fractal analysis (PCT/EP2016/071551 and USPTO 2021 10,991,109 approved).
Federico Biavati: Nothing to disclose
Bernhard Föllmer: Nothing to disclose

### RPS 105-6

**Diagnostic performance of a deep-learning model to detect coronary stenoses on CTA images in emergency patients presenting with acute chest pain**

*C. G. Glessgen*[1], J-P. Vallee[1], A. Platon[1], P-A. A. Poletti[1], J-F. Paul[2], J-F. Deux[1]; [1]Geneva/CH, [2]Paris/FR
*(carl.glessgen@hcuge.ch)*

**Purpose:** Aortic computed tomography angiography (CTA) is a critical tool in the workup of patients with acute chest pain (ACP). Our goal was to evaluate the performances of a deep-learning model (DLM) for the detection of significant coronary stenoses in emergency patients presenting with ACP and explored with CTA.
**Methods or Backround:** One hundred and eighty-one patients presenting with ACP and examined by ECG-gated CTA of the aorta between January 2021 and May 2022 were retrospectively included. With regard to the emergency setting, no premedication (beta-blockers or nitrates) was administered. For each of the 3 coronary arteries, 9 radially acquired curvilinear MPR images 40° apart were classified by the DLM in 2 groups: CAD-RADS 0-1-2 (<50%) or 3-4-5 (≥50% stenosis). Two readers in consensus separated the patients in two groups depending on the overall presence of motion artefacts and read the cases regarding significant stenoses (<50% vs. ≥50% stenosis).
**Results or Findings:** In patients without artefacts on CT images (n=89, 49%), all significant stenoses (CAD RADS 3-4-5) detected by the human readers were correctly identified by the DLM. Diagnostic performances of the DLM were: sensitivity 100%, specificity 66%, positive predictive value (PPV) 56%, negative predictive value (NPV) 100%. Among patients with artifacts (n=92, 51%), 19 (27%) had significant stenoses following human analysis but were misdiagnosed by the DLM resulting in poorer diagnostic performances: sensitivity 78%, specificity 75%, PPV 98%, NPV 14%.
**Conclusion:** The DLM showed superior performance for the identification of significant stenoses on CT images without artefacts, advocating the potential of such a system to help radiologists in their daily routine in patients with ACP. The presence of artefacts reduced performance and required human supervision.
**Limitations:** An identified limitation was that the proportion of patients with artefacts was relatively high.
**Ethics committee approval:** No information provided by the submitter.
**Funding for this study:** No funding was received for this study.
**Author Disclosures:**
Jean-Paul Vallee: Nothing to disclose
Alexandra Platon: Nothing to disclose
Carl Guillaume Glessgen: Nothing to disclose
Pierre-Alexandre Aloïs Poletti: Nothing to disclose
Jean-Francois Paul: Founder: SPIMED-AI CEO: SPIMED-AI
Jean-François Deux: Nothing to disclose

### RPS 105-7

**A direct deep-learning approach for prediction of patient survival in patients undergoing transcatheter aortic valve replacement based on CT data without the need for muscle segmentation**

*M. Theis*, S. Nowak, W. Block, J. A. Luetkens, U. I. Attenberger, A. M. Sprinkart; Bonn/DE
*(Maike.Theis@ukbonn.de)*

**Purpose:** Previous studies have demonstrated the prognostic value of the fatty muscle fraction (FMF) derived from skeletal muscle segmentations from 2D CT data for prediction of survival time in patients undergoing transcatheter aortic valve replacement (TAVR). We aimed to develop a direct deep-learning model for prediction of survival time directly on unsegmented CT images.
**Methods or Backround:** A total of 760 patients undergoing TAVR were included. Clinical follow-up data were available for at least 1 year. Mean survival time of non-censored patients (54%) was 871±763 days. A baseline Cox proportional hazard model was trained using the muscle segmentation based frailty marker FMF as predictor (CPHM_FMF). This baseline approach was compared to a convolutional neural network (CNN) trained on unsegmented images at L3/L4 lumbar level. The encoder of this model was pre-trained by training of an autoencoder conditioned on preserving paraspinal musculature. Pre-trained weights were first kept frozen (SurvNet_Frozen) and subsequently unfrozen (SurvNet_Unfrozen). C-index and area under the curve (AUC) for prediction of one year survival was assessed with 95% confidence intervals. All methods were trained with five-fold cross validation and evaluated on 152 hold-out test cases.
**Results or Findings:** The direct deep-learning approach leads to higher performance on the hold-out test set than the baseline approach (CPHM_FMF: c-index: 0.573 [0.506,0.640], AUC: 0.578 [0.459,0.684]; SurvNet_Frozen: c-index: 0.658 [0.593,0.723], AUC: 0.728 [0.624,0.831]). Unfreezing the weights did not lead to further improvements (SurvNet_Unfrozen: c-index: 0.654 [0.586,0.718], AUC: 0.711 [0.599,0.816]).
**Conclusion:** Direct prediction of patient survival from the entire CT slice is feasible, eliminating the need for segmentations of muscle tissue for FMF calculation.

**Limitations:** Currently, neither approach can provide perfect risk stratification. Therefore, the addition of clinical parameters to both models is planned.
**Ethics committee approval:** This retrospective study was approved by the institutional review board with waiver of written informed consent.
**Funding for this study:** There was no funding for this study.
**Author Disclosures:**
Julian Alexander Luetkens: Nothing to disclose
Alois M. Sprinkart: Nothing to disclose
Wolfgang Block: Nothing to disclose
Sebastian Nowak: Nothing to disclose
Ulrike I. Attenberger: Nothing to disclose
Maike Theis: Nothing to disclose

### RPS 105-8

**Radiomics based on steady-state free precession (SSFP) cine sequences for predicting major adverse cardiac events in patients with dilated cardiomyopathy**

*X. Li*[1], Y. Xu[1], L. Peng[1], X. Chen[1], J. Liu[1], H. Yin[2], Y. Chen[1]; [1]Chengdu/CN, [2]Shanghai/CN

**Purpose:** We aimed to use a cardiac magnetic resonance imaging (CMR) radiomics approach in predicting major adverse cardiac events in dilated cardiomyopathy (DCM) patients.
**Methods or Backround:** We enrolled 416 patients diagnosed with DCM who underwent CMR from September 2013 to March 2018. The study endpoint was defined as all-cause mortality and heart transplantation. These patients were allocated to a training dataset (n=291) and a validation dataset (n=125). Radiomics features were extracted from mid-left ventricle end-diastole myocardium using cine short-axis sequences. Four models based on radiomics features, clinical parameters and CMR tissue characteristics were built by the multivariable logistic regression algorithm and compared by the area under the receiver operating characteristic curves (AUCs). Kaplan–Meier analysis was used to evaluate the prognostic value of radiomics features.
**Results or Findings:** During the median follow-up of 48.6 months (interquartile range: 29.9–62.8 months), 73 patients experienced the clinical endpoints. Eight radiomics features, nine clinical parameters and five CMR tissue characteristics were found to be independent predictors. Of all the four models, the radiomics + clinical + CMR model reached the best AUC of 0.77, 0.75 in training and validation dataset, respectively (p<0.05). In the comparison of ROC curves of multiple groups, radiomics features were better than RI, LGE, ECV, LVEF at predicting the prognosis of DCM. Kaplan–Meier analysis of radiomics features revealed that the hazard ratio between the high-risk group and low-risk group was 4.002 (95%CI, 2.310-6.933) and 3.488 (95%CI, 1.448-8.402) in the training dataset and validation dataset, respectively (logrank p<0.001, p＝0.01).
**Conclusion:** CMR-based radiomics analysis shows potential as an effective tool for risk stratification in DCM patients.
**Limitations:** An identifed limitation was that radiomics feature extraction was limited to mid-slices.
**Ethics committee approval:** No information provided by the submitter.
**Funding for this study:** This study was funded by the National Natural Science Foundation of China (No. 81601462).
**Author Disclosures:**
Yucheng Chen: Nothing to disclose
Liqing Peng: Nothing to disclose
Jing Liu: Nothing to disclose
Hongkun Yin: Nothing to disclose
Yuanwei Xu: Nothing to disclose
Xue Li: Nothing to disclose
Xiaoyi Chen: Nothing to disclose

### RPS 105-9

**Transfer learning from cine to late gadolinium enhancement MRI for myocardial segmentation in patients with acute myocardial infarction**

*S. A. Khan*, K. Bouazizi, M. Prigent, J. Silvain, A. Redheuil, G. Montalescot, T. Dietenbeck, N. Kachenoura; Paris/FR
*(saudahmadkhan@hotmail.com)*

**Purpose:** Deep-learning models have shown good performances in segmenting Cine-bSSFP MRI, a widely used sequence in clinical routine for cardiac volumes quantification. While late gadolinium enhancement (LGE) images offer a powerful imaging tool for myocardial infarction (MI) characterisation, their processing remains tedious and widely relies on the operator experience. The aim of this study was to present the added-value of a transfer learning (TL) approach from cine-bSSFP towards LGE short-axis images for left ventricular (LV) myocardial segmentation, depending on the ratio between annotated data for both sequences.
**Methods or Backround:** We studied 192 patients of the QUORUM study (NCT03715998, 4264 images) with acute anterior MI who had cine-bSSFP MRI images annotated in end-systole and end-diastole. Among them, 127 patients (1511 images) also had annotated LGE images. Preprocessing comprised centering on the LV and cropping excess background. U-Net++ network was trained on 63, 31, 15 and 7 LGE datasets either from scratch or using TL from cine-bSSFP, while the 64 remaining LGE datasets were used for testing.
**Results or Findings:** The model achieved high performances for myocardial segmentation, with dice scores of 90.2%, 89.8%, 87.1%, 85.1% without TL and 90.7%, 90.5%, 88.6% and 87.7% with TL when trained respectively on 63, 31, 15 and 7 LGE datasets. One might also note the usefulness of preprocessing revealed by a substantial drop in dice scores (86.98%, 86.27%, 82.04%, 74.24%) for a network trained using TL and no preprocessing.
**Conclusion:** TL enhanced performances in all training configurations with a higher myocardial dice score boost when smaller LGE datasets are available for training. Preprocessing has a major impact on segmentation performances and should not be overlooked.
**Limitations:** The fact that this was not tested on LGE patterns other than those of MI, was an identified limitation.
**Ethics committee approval:** Ethics committee approval reference: NCT03715998.
**Funding for this study:** This study was funded by: H2020 Maestria (965286) for funding S.A.K.
**Author Disclosures:**
Johanne Silvain: Nothing to disclose
Alban Redheuil: Nothing to disclose
Thomas Dietenbeck: Nothing to disclose
Khaoula Bouazizi: Nothing to disclose
Gilles Montalescot: Nothing to disclose
Mikaël Prigent: Nothing to disclose
Saud Ahmad Khan: Nothing to disclose
Nadjia Kachenoura: Nothing to disclose

**08:00-09:00** **Research Stage 2**

### Research Presentation Session: Head and Neck

### RPS 108

### Thyroid imaging and treatment

**Moderator**
P.-Y. Marcy; Ollioules/FR
*(brozpy@gmail.com)*

### RPS 108-2

**The TIRADS System**

*S. Mortellaro*[1], G. Carrafiello[2]; [1]Stradella/IT, [2]Milan/IT
*(sveva.mortellaro@unimi.it)*

**Purpose:** The objective of this work was to evaluate in an outpatient, real-world setting the cytology results of the European Thyroid Association (EU-TIRADS), the Korean Society of Thyroyd Radiology (K-TIRADS) and the American College of Radiology (ACR-TIRADS).
**Methods or Backround:** Thyroid Imaging Reporting and Data Systems (TIRADS) are ultrasound risk stratification systems designed to exclude thyroid nodules that can be safely deferred, in order to identify, instead, those that deserve further diagnostic investigation by FNAC (Cytologic Examination by Fine Needle Aspirate). 467 nodules underwent (June 2019-April 2021) FNAC. Before FNAC was performed, nodules were classified with EU-TIRADS, ACR-TIRADS and K-TIRADS. A total of 168 nodules with TIR1 (non-diagnostic) or TIR3 (indeterminate cytology) cytology and 22 sub-centimeter nodules with no suspicious features were excluded from the analysis.
**Results or Findings:** The application of TIRADS criteria reduced the number of needle-aspirations by 61.5% for ACR-TIRADS (287 nodules/467 total), 14.3% for KTIRADS (67 nodules/467 total) and 35.1% (164 nodules/467 total) for EU-TIRADS.
**Conclusion:** The systematic application of the TIRADS Systems would allow a significant reduction in the number of FNAs needed. However, a not insignificant number of nodules could be missed that are malignant on cytology.
**Limitations:** Since 31.2% of TIR4 / TIR5 in our cohort were <10 mm, it will be interesting to evaluate whether a cytologic diagnosis of malignancy in micro-nodules would have clinical benefits. Further studies are needed to also establish the clinical outcomes of TIRADS in the context of the actual population.
**Ethics committee approval:** The ethics committee of the university of Milan approved this work.
**Funding for this study:** The polyclinic of Milan provided funding for this study.
**Author Disclosures:**
Gianpaolo Carrafiello: Nothing to disclose
Sveva Mortellaro: Nothing to disclose

### RPS 108-3

**Predictive US findings between malignant and benign thyroid nodules: Doppler and microvascular imaging according to the K-TIRADS and pathological subtypes of malignancy**

H. S. Ahn, M. J. Hong, H. J. Park, Y. Yim, S. B. Park, *Y. Kim*; Seoul/KR
*(woooook@caumc.or.kr)*

**Purpose:** This study aimed to investigate the utility of Doppler and microvascular imaging for distinguishing between benign and malignant thyroid nodules according to the TI-RADS and pathologic subtypes of thyroid malignancy.
**Methods or Backround:** From February 2022 to September 2022, a total of consecutive 113 thyroid nodules (≥ 0.5 cm) in 103 patients (mean age, 51.0 ± 15.1 years) with final diagnoses were included in this study. Two radiologists retrospectively reviewed the ultrasonographic findings include colour Doppler and microvascular score using 4-scale visual analysis in consensus. It was compared between benign and malignant nodules and analysed according to the US patterns of thyroid nodules based on the Korean thyroid imaging reporting and data system (K-TIRADS).
**Results or Findings:** Among 113 thyroid nodules, 72 were benign (63.7%) and 41 were malignant (36.3%). The malignant nodules comprised 32 papillary carcinomas, 8 follicular carcinomas and 1 lymphoma. The lesions were assigned according to the K-TIRADS as follows: benign (n = 1, 0.9%), low suspicion (n = 39, 34.5%), intermediate suspicion (n = 43, 38.1%), high suspicion nodules (n = 30, 26.5%). In low suspicion nodules, hypoechogenicity of the nodule was more common in malignancy compared with benign (p = 0.023), but there were no other significant findings in low to intermediate suspicion nodules include doppler and microvascular score to predict malignancy. In highly suspicion nodules, Doppler score (p = 0.007) and microvascular score (p = 0.048) were sole significant finding between benign and malignancy. Malignant nodules showed significantly lower Doppler (p = 0.000) and microvascular score (p = 0.006) compared with benign, especially in papillary carcinomas.
**Conclusion:** Doppler and microvascular imaging can be helpful US finding in differentiating between benign status and malignancy in highly suspicion thyroid nodules.
**Limitations:** No limitations were identified.
**Ethics committee approval:** No information provided by the submitter.

**Funding for this study:** No funding was received for this study.

**Author Disclosures:**
Sung Bin Park: Nothing to disclose
Hyun Jeong Park: Nothing to disclose
Min Ji Hong: Nothing to disclose
Younguk Kim: Nothing to disclose
Younghee Yim: Nothing to disclose
Hye Shin Ahn: Nothing to disclose

### RPS 108-4

**Superb microvascular imaging (SMI) and elastosonography in thyroid nodule: diagnostic value in a real-time cohort**

*D. Negroni*, S. Cesano, G. Maddalena, F. Abruzzese, P. Conte, P. Neri, R. L. Boldorini, A. Carriero; Novara/IT
*(dvdngr@gmail.com)*

**Purpose:** In clinical practice, thyroid nodules are classified according to TI-RADS by B-mode and colour-flow Doppler study. The aim of the study is to evaluate the possible added value of superb microvascular imaging (SMI) and elastosonography in the stratification of malignancy risk of thyroid nodules.
**Methods or Backround:** All patients with thyroid nodules who were candidates for needle aspiration were enrolled. Experienced operators performed a standard examination with TI-RADS calculation, followed by SMI and elastosonography on the nodules. The needle aspiration outcome was used as the gold standard. Statistical analysis calculated the ROC curves of the techniques applied individually and serially.
**Results or Findings:** In this prospective study, we analysed 260 nodules found in 251 patients (mean age 58.6 years ± 14). 11.2% were TI-RADS 1, 18.9% TI-RADS 2, 41.1% TI-RADS 3, 28.1% TI-RADS 4, and 0.8% TI-RADS 5. The SMI technique showed an AUC of 0.57 (95%CI 0.49; 0.66) while elastosonography had an AUC of 0.58 (95%CI 0.49; 0.67) when used individually. SMI and elastosonography together had an AUC of 0.62 (95% CI 0.52; 0.71). TI-RADS had an AUC of 0.67 (95%CI 0.59; 0.75). SMI and elastosonography applied together with TI-RADS had an AUC of 0.69 (95% CI 0.61; 0.77).
**Conclusion:** In the real-world cohort of patients, the SMI technique and elastosonography slightly increase the AUC of TI-RADS. Taken individually, SMI and elastosonography do not have a very strong AUC.
**Limitations:** The sample is limited to a single centre, and anotomo-patholoigic post-operative data of high-grade TI-RADS are currently absent.
**Ethics committee approval:** Ethics committee approval was received from Comitato Etico Novara.

**Funding for this study:** No funding was received for this study.

**Author Disclosures:**
Pierluigi Neri: Nothing to disclose
Patrizio Conte: Nothing to disclose
Renzo Luciano Boldorini: Nothing to disclose
Davide Negroni: Nothing to disclose
Sara Cesano: Nothing to disclose
Alessandro Carriero: Nothing to disclose
Gaetano Maddalena: Nothing to disclose
Flavia Abruzzese: Nothing to disclose

### RPS 108-5

**Feasibility study on multi-phase virtual non-contrast images derived from dual-layer spectral detector CT substitute for true non-contrast images in patients with thyroid carcinoma**

*X. Shiyu*, W. Deng, Y. Gu; Shanghai/CN
*(xiangshiyu0803@163.com)*

**Purpose:** The purpose of this study was to explore the feasibility of the multi-phase virtual non-contrast (VNC) images from dual-layer spectral detector CT (DLSCT) substitute for true non-contrast (TNC) images in patients with thyroid carcinoma.
**Methods or Backround:** 24 pathology-confirmed thyroid carcinoma patients, who underwent unenhanced and enhanced scans by DLSCT were retrospectively analysed. VNC images were generated from arterial (VNC_a) and venous (VNC_v) phases. The CT attenuation values of thyroid lesion, thyroid paraenchyma, carotid artery, jugular vein, muscle, bone, subcutaneous fat on the three groups were compared. The dose length product (DLP) of all scans were recorded.
**Results or Findings:** The CT attenuation values in TNC were all significant different with the values in both VNC_a and VNC_v for all the organs (all P<0.05), except for muscle between TNC and VNC_a (P=0.609). While there was no significant difference for the attenuation values of thyroid lesion, thyroid parenchyma, muscle, bone, subcutaneous fat between VNC_a and VNC_v groups (all P>0.05). The attenuation difference of lesions between TNC and VNC_a, TNC and VNC_v, VNC_a and VNC_v were 11.6±8.8, 9.0±7.6 and -2.6±6.5, respectively. Bland-Altman plots showed that the mean bias (95% limits of agreement) of lesions between TNC and VNC_a, TNC and VNC_v, TNC_a and TNC_v were 11.6(-5.9 to 29.20), 9.0(-6.2 to 24.2), and -2.6(-15 to 10), respectively. The DLP of TNC accounted for 26.6% (261.5/984.6) of the total DLP.
**Conclusion:** The CT attenuation values in VNC_a and VNC_v has very low attenuation differences and good consistency. The VNC images cannot directly substitute for TNC in carcinoma patients, but may reduce radiation dose.
**Limitations:** First, this was a retrospective study with a limited patient population. Second, the parameters of VNC images might be influenced by different scanning protocols and image post-processing algorithms.
**Ethics committee approval:** No information provided by the submitter.

**Funding for this study:** No funding was received for this study.

**Author Disclosures:**
Yajia Gu: Author: None Author: None
Xiang Shiyu: Speaker: None Author: None
Weiwei Deng: Author: None Author: None

### RPS 108-6

**Long-term outcomes of ultrasound-guided radiofrequency ablation in patients with intrathoracic goiter: a case series with an average follow-up of 20 months.**

*Y-H. Wang*, P-L. Chiang, C-K. Wang, A-N. Lin, W-C. Lin; Kaohsiung City/TW
*(ff10wyh@gmail.com)*

**Purpose:** This study aims to evaluate the long-term efficacy and safety of patient with intrathoracic goiter (ITG) in a single medical center after receiving radiofrequency ablation (RFA) over a follow-up period of more than six months.
**Methods or Backround:** Twenty-two patients (6 men and 16 women; mean age 52.9 years; range 37-90 years) with twenty-four ITGs treated by RFA from 2017 to 2021 were evaluated. All patients underwent computed tomography (CT) or magnetic resonance imaging (MRI) before RFA. Follow-up CT/MRI was performed 6 months after initial RFA and then every 6-12 months. Main outcomes of RFA measures degree of extension using the cross-section imaging CT system, goiter volume, volume reduction rate (VRR), trachea deviation, and trachea lumen by using ultrasonography, CT or MRI. Complications during the procedure and follow-up period were evaluated. We also compared outcomes for goiters treated with single session RFA (n = 16) and those treated with multiple session RFA (n = 8).
**Results or Findings:** The mean follow-up period was 20.6 ± 15.8 months (range 6–51 months). At the last follow-up visit, volume of the nodule measured by CT/MRI had decreased significantly (90.1± 50.7 mL vs 31.5 ± 27.8 mL; P < 0.05), whereas volume reduction rate was 67.4% ± 15.3%. The mean number of ablation sessions was 1.38 ± 0.6 (range 1–3 sessions). Volume reduction rate in patients underwent multiple sessions were significantly higher than single session subgroup. (61% and 77%, P < 0.05) was noted.

**Conclusion:** The results suggest long-term clinical efficacy and the substantial safety of RFA for the treatment of intrathoracic goiters. Multiple sessions of RFA may be helpful to achieve better clinical outcome in patients with larger goiters.
**Limitations:** No gross pathology could be obtained to exclude the possibility of malignancy.
**Ethics committee approval:** No information provided by the submitter.
**Funding for this study:** No funding was received for this study.
**Author Disclosures:**
Cheng-Kang Wang: Nothing to disclose
Wei-Che Lin: Nothing to disclose
Pi-Ling Chiang: Nothing to disclose
Yu-Hsin Wang: Nothing to disclose
An-Ni Lin: Nothing to disclose

### RPS 108-7

**Reverse encoding distortion correction (RDC) on diffusion-weighted imaging (DWI): capability for image quality improvement and ADC evaluation accuracy in head and neck tumours**

H. Ikeda[1], *Y. Ohno*[1], K. Yamamoto[2], M. Shinohara[2], M. Yui[2], M. Ikedo[2], H. Nagata[1], T. Ueda[1], H. Toyama[1]; [1]Toyoake/JP, [2]Otawara/JP
*(yohno@fujita-hu.ac.jp)*

**Purpose:** To directly compare the capability of reverse encoding distortion correction (RDC) method for image quality improvement and ADC evaluation accuracy on diffusion-weighted imaging (DWI) in head and neck tumours.
**Methods or Backround:** Forty-seven patients with head and neck tumours underwent DWIs with and without RDC on 3T systems and pathological examinations. Then, all tumours were divided into malignant (n=16) and benign (n=31) tumour groups. To determine the utility of RDC for DWI, the deformation ratio (DR) of each tumour was determined from each tumour area difference between DWI and T2WI. To compare ADC measurement accuracy between both DWIs, ADC values of tumour and spinal cord were determined by ROI measurements in each patient. Moreover, overall image quality, lesion distortion and lesion conspicuity of each tumour were assessed by 5-point scales. For image quality comparisons, DR and ADC were compared between DWIs with and without RDC via paired t-test. Each qualitative score within the DWIs was also compared using Wilcoxon's signed rank test. Moreover, ADC values were correlated with Spearman's correlations. To determine each feasible threshold value in the differentiation of malignant from benign tumours, a ROC-based positive test was performed. Finally, sensitivity (SE), specificity (SP) and accuracy (AC) were compared to each other via McNemar's test.
**Results or Findings:** The DR of DWI with RDC was significantly smaller than the DR of DWI without RDC ($p<0.05$). Each qualitative score of DWI with RDC was significantly higher than the ones without RDC ($p<0.05$). When applying each feasible threshold value, there were no significant difference of SE, SP and AC between two DWIs ($p>0.05$).
**Conclusion:** RDC has the potential to improve image quality without any influence to ADC measurement and differentiation capability in head and neck tumours.
**Limitations:** The small sample size was identified as a limitation.
**Ethics committee approval:** The IRB of Fujita Health University Hospital approved this study.
**Funding for this study:** Funding for this study was provided through a research grant from Canon Medical Systems Corporation.
**Author Disclosures:**
Maiko Shinohara: Employee: Canon Medical Systems Corporation
Masato Ikedo: Employee: Canon Medical Systems Corporation
Kaori Yamamoto: Employee: Canon Medical Systems Corporation
Hirotaka Ikeda: Nothing to disclose
Hiroshi Toyama: Research/Grant Support: Canon Medical Systems Corporation
Takahiro Ueda: Nothing to disclose
Hiroyuki Nagata: Research/Grant Support: Canon Medical Systems Corporation
Masao Yui: Employee: Canon Medical Systems Corporation
Yoshiharu Ohno: Research/Grant Support: Canon Medical Systems Corporation

**08:00-09:00** **Research Stage 3**

### Research Presentation Session: Oncologic Imaging

### RPS 116

### Predicting and monitoring response in oncology

**Moderator**
M. D'Anastasi; Msida/MT
**Author Disclosures:**
Melvin D'Anastasi: Advisory Board: Keosys Medical Imaging

### RPS 116-2

**Early prediction of pathological response to neoadjuvant chemoradiotherapy for oesophageal cancer using fully hybrid PET/MR: updated results from interim analysis**

*A. E. Pellegrini*, D. Palumbo, C. Fiorino, M. Mori, C. Canevari, S. Steidler, N. Di Muzio, R. Rosati, F. De Cobelli; Milan/IT
*(alessandro.pellegrini94@gmail.com)*

**Purpose:** Neoadjuvant chemoradiotherapy (nCRT) double the median overall survival of patients with locally advanced oesophageal cancer. This subgroup of patients (representing almost one third of the entire population) does not benefit from additional surgery; on the other hand 18% of patients who underwent nCRT were non-responders, only suffering from nCRT side effects. In this respect, our aim was to identify novel biomarkers allowing early prediction of pathological tumour response.
**Methods or Backround:** From 01/2020, all consecutive patients with biopsy-proven potentially resectable oesophageal cancer scheduled to receive nCRT were prospectively enrolled. In addition to standard of care imaging, a fully integrated hybrid PET/MR was performed at three time points (prior to, during and six weeks after treatment completion). For each patient, an early regression index (ERI) was computed according to the formula: ERI= ln [1–(tumour volumemid/tumour volumepre)tumour volume pre]. Tumour volumes were contoured on axial high-resolution T2 weighted images by an experienced radiologist. Finally, after surgery, these data were systematically correlated with the pathological outcome in terms of Tumour Regression Grade (TRG). TRG=1 refers to pathological complete response status.
**Results or Findings:** At present, of 25 patients enrolled, 10 underwent surgery after completion of nCRT; of these, five patients had TRG=1. Interestingly, patients with a pathological complete response demonstrated significantly lower ERI values when compared with those patients with TRG≥2 (6.05vs.19.83,$p<0.001$). No PET parameter was shown to significantly correlate to TRG; however, patients with TRG=1 had lower values of mean Standardised Uptake Value at baseline imaging.
**Conclusion:** Interim results confirm the existence of a novel MR-based biomarker (ERI), able to early predict pathological tumour response during nCRT, thus providing an actual aid for patients' personalised therapeutic path.
**Limitations:** Small sample size.
**Ethics committee approval:** 07/INT/2017
**Funding for this study:** Funded by grant AIRC IG 23015.
**Author Disclosures:**
Alessandro Eugenio Pellegrini: Nothing to disclose
Nadia Di Muzio: Nothing to disclose
Claudio Fiorino: Nothing to disclose
Diego Palumbo: Nothing to disclose
Stephanie Steidler: Nothing to disclose
Francesco De Cobelli: Nothing to disclose
Martina Mori: Nothing to disclose
Carla Canevari: Nothing to disclose
Riccardo Rosati: Nothing to disclose

### RPS 116-3

**Reasons for discordance among imaging response criteria in lymphoma under CAR T-cell therapy**

*M. Winkelmann*, V. Blumenberg, K. Rejeski, F. Dekorsy, P. Bartenstein, J. Ricke, M. Bergwelt-Baildon, M. Subklewe, W. Kunz; Munich/DE

**Purpose:** Chimeric antigen receptor T-cell therapy (CART) prolongs survival for patients with refractory or relapsed (r/r) lymphoma. Discrepancies among different response criteria for lymphoma under CART were recently shown. We evaluated reasons for discordance among different response criteria and their relation to overall survival (OS).
**Methods or Backround:** Consecutive patients with baseline and follow-up imaging 30 (FU1) and 90 days (FU2) after CART were included. Overall response was determined based on Lugano criteria, Cheson criteria, response evaluation criteria in lymphoma (RECIL) and lymphoma response to immunomodulatory therapy criteria (LYRIC). Overall response rate (ORR) and rates of progressive disease (PD) were determined. For each criterion reasons for PD were analysed in detail.
**Results or Findings:** 41 patients were included (median age: 64 years, 41% female). ORR was 68%, 68%, 63%, and 68% at FU2 by Lugano, Cheson, RECIL, and LYRIC, respectively. PD rates differed among criteria with 32% by Lugano, 27% by Cheson, 17% by RECIL, and 17% by LYRIC. Dominant reasons for PD according to Lugano were target lesion (TL) progression (84.6%), new appearing lesions (NL; 53.8%), non-TL progression (27.3%), and progressive metabolic disease (PMD; 15.4%). Deviations among the criteria were largely explained by PMD of pre-existing lesions that are defined as PD only by Lugano and non-TL progression, which is not defined as PD by RECIL and in some cases classified as indeterminate response by LYRIC. Interestingly, grouping patients according to focality of progressive target lesions showed a significant trend for OS stratification (p=0.036).
**Conclusion:** Following CART, lymphoma response criteria show differences in imaging endpoints, especially in defining PD. The response criteria must be considered when interpreting imaging endpoints and outcomes from clinical trials.
**Limitations:** Single centre study with limited patient population.
**Ethics committee approval:** All medical records and imaging studies were reviewed with the approval of the LMU Munich Institutional Review Board (LMU Ethics Committee, project number 19-817). Informed consent was obtained from all individual participants included in the study.
**Funding for this study:** FöFoLe LMU München (1147)
**Author Disclosures:**
Marion Subklewe: Consultant: Amgen, Bristol Myers Squibb, Celgene, Gilead, Pfizer, Novartis, and Roche Advisory Board: Amgen, Celgene, Gilead, Janssen, Novartis, Pfizer, and Seattle Genetics Research/Grant Support: Amgen, Gilead, Miltenyi Biotec, MorphoSys, Roche, and Seattle Genetics Speaker: Amgen, Celgene, Gilead, Janssen, and Pfizer
Viktoria Blumenberg: Research/Grant Support: Gilead, Novartis, Celgene, and Roche
Michael Bergwelt-Baildon: Research/Grant Support: Novartis, Kite Pharma, Miltenyi Biotec, Mologen, MSD, Astellas, and Roche
Peter Bartenstein: Nothing to disclose
Michael Winkelmann: Nothing to disclose
Wolfgang Kunz: Consultant: Bristol Myers Squibb
Kai Rejeski: Research/Grant Support: Kite/Gilead and Novartis
Franziska Dekorsy: Nothing to disclose
Jens Ricke: Nothing to disclose

### RPS 116-4

### Monitoring patients with metastatic castration resistant prostate cancer using whole-body magnetic resonance imaging: comparison with conventional imaging

*F. Zugni*, S. Alessi, P. Motta, P. Pricolo, M. Belmonte, A. Colombo, G. Petralia; Milan/IT

**Purpose:** To evaluate whether whole-body magnetic resonance imaging (WB-MRI) allows earlier identification of progressive disease (PD) compared to conventional imaging (CT and bone scintigraphy), in patients with bone metastatic castration-resistant prostate cancer (mCRPC).
**Methods or Backround:** This is an interim analysis of an ongoing prospective trial enrolling patients with mCRPC with bone metastases candidate to systemic treatment. Patients underwent baseline imaging assessment with WB-MRI, bone scintigraphy, and CT. Monitoring was performed every 12 weeks with all three modalities, until the occurrence of PD. Radiologists reporting each modality were blinded to the results of the other two. The performance of WB-MRI vs conventional imaging in identifying PD was evaluated.
**Results or Findings:** Of the 35 patients enrolled (aged 68.5 ± 8.5), 5 were excluded (drop out) and 30 were included in the analysis. Out of 30 patients, 28 showed PD at imaging; in 13 of them (46.4%, confidence interval 28.7%-68.1%), PD was detected only by WB-MRI; while in the remaining 14 patients, PD was reported contemporarily by WB-MRI and conventional imaging. The difference in the detection of PD was statistically significant (p= 0.0009, Mc Nemar test). All cases of PD were confirmed by further imaging evaluations. Bone was the most frequent site of disease, having occurred in 26 out of 28 cases; of these only four were detected by scintigraphy. PD occurred most frequently at 12 weeks (range 1-60 weeks).
**Conclusion:** WB-MRI was superior to conventional imaging in monitoring patients with mCRPC, allowing an earlier identification of PD in nearly half of all cases.
**Limitations:** Impact on survival not evaluated; interim results.
**Ethics committee approval:** No information provided by the submitter.
**Funding for this study:** This work was partially supported by the Italian Ministry of Health with Ricerca Corrente and 5x1000 funds.
**Author Disclosures:**
Sarah Alessi: Nothing to disclose
Fabio Zugni: Nothing to disclose
Giuseppe Petralia: Nothing to disclose
Paolo Motta: Nothing to disclose
Maddalena Belmonte: Nothing to disclose
Paola Pricolo: Nothing to disclose
Alberto Colombo: Nothing to disclose

### RPS 116-5

### Role of radiomics to predict patient outcome in metastatic gastroenteropancreatic neuroendocrine neoplasms

*M. Polici*, B. Masci, M. Zerunian, A. Del Gaudio, D. De Santis, G. Tremamunno, F. Pucciarelli, D. Caruso, A. Laghi; Rome/IT
*(policimichela28@gmail.com)*

**Purpose:** The evaluation of radiomic approach to predict progression in patients with metastatic gastroenteoropancreatic neuroendocrine neoplasms (GEP-NENs).
**Methods or Backround:** Fourty-six patients affected by GEP-NENs with liver metastases and availability of baseline CT scans, were retrospectively enrolled, between June 2011 and August 2019. For each patient were collected data about histological diagnosis, tumour grading, progression free survival (PFS), overall survival (OS), progression, death, and Ki67. Population was divided into two groups progressive and non-progressive. An expert radiologist manually drawn 3D segmentation of whole liver on baseline CT scans in arterial phase, by using a dedicated software (3DSlicer v4.10.2). 107 radiomic features were extracted and compared between two groups (T-test or Mann-Whitney); radiomics performance was assessed with receiver operating characteristic curve, Kaplan-Meyer curves used for survival analysis, univariate and multivariate logistic regression were performed to build three different predictive models (clinical, radiomic, and combined model). P<0.05 considered significant.
**Results or Findings:** 32/46 patients were classified as progressive (PFS and OS median: 13.5 and 36 months, respectively) and 14/46 as non-progressive (PFS and OS median: 51 and 51 months, respectively). Among radiomic parameters, Elongation and SurfaceVolumeRatio showed significant differences between two groups (P<0.05) with the best performance (AUC 0.70-0.67, P<0.05). Elongation>0.07 and SurfaceVolumeRatio<0.06 resulted to be correlated with progressive disease (P<0.03). Univariate analysis showed seven radiomic features independently correlated with progression, and in multivariate analysis the combined model showed the best performance with AUC 0.83 (P=0.03), followed by radiomic model AUC 0.80 (P=0.04). Clinical model did not show significant results (P=0.43).
**Conclusion:** Radiomics could be used as imaging biomarker to predict progressive disease in patients affected by metastatic GEP-NENs.
**Limitations:** Small sample, retrospective nature.
**Ethics committee approval:** Written informed consent obtained from all participants.
**Funding for this study:** No funding was received by this study.
**Author Disclosures:**
Damiano Caruso: Nothing to disclose
Benedetta Masci: Nothing to disclose
Francesco Pucciarelli: Nothing to disclose
Marta Zerunian: Nothing to disclose
Domenico De Santis: Nothing to disclose
Giuseppe Tremamunno: Nothing to disclose
Andrea Laghi: Nothing to disclose
Antonella Del Gaudio: Nothing to disclose
Michela Polici: Nothing to disclose

### RPS 116-6

### Utility of 18F-FDG PET and MR-DWI datasets for the evaluation of treatment response of lymphoma patients to chemotherapy

*J. Grueneisen*[1], M. Chodyla[1], J. Kirchner[2], B. M. Schaarschmidt[1], K. Herrmann[1], J. M. Ludwig[1], M. Forsting[1], L. Umutlu[1]; [1]Essen/DE, [2]Dusseldorf/DE

**Purpose:** To assess the clinical applicability of MR-DWI datasets as a radiation-free alternative to 18F-FDG PET for the assessment of treatment response of lymphoma under chemotherapy.
**Methods or Backround:** A total of 29 patients with the diagnosis of lymphoma underwent a simultaneous 18F-FDG PET/MR examination before induction chemotherapy and a second 18F-FDG PET/MR scan for the evaluation of treatment response. For each patient, the tumour stage was determined according to the Ann-Arbor staging classification and disease activity according to the Deauville-criteria. Therefore, the metabolic activity (SUV) of lymphoma manifestations was measured and in addition, diffusion restriction (ADC values) of the lymphoma lesions was determined. For statistical analysis, a Mann-Whitney-U test was used and ROC-analysis was performed to compare the results of the different quantitative imaging parameters.

**Results or Findings:** A total of n=67 lymphoma manifestations were evaluated and according to the Deauville-criteria, n=18 patients were defined as treatment responders and the remaining n=11 patients as non-responders. Lesions in the group of treatment responders showed a mean reduction in metabolic activity (SUVmax: -85.9%) and an increase of the ADC-values (ADCmean: +67.2%). Percentage changes in the non-responder group amounted to -56.6% (SUVmax) and +43.7% (ADCmean). Differences of the parameters between responders and non-responders were significantly different (SUVmax: p-value <0.001, ADCmean: p-value <0.05). The corresponding AUC values for the differentiation between responders and non-responders were 0.94 (SUVmax) and 0.79 (ADCmean), respectively.
**Conclusion:** The present study shows promising results using MR-DWI data for the discrimination between treatment response or non-response of lymphoma patients to chemotherapy. While the metabolic information from 18F-FDG PET provides a higher accuracy for response evaluation, MR-DWI could be useful as a radiation-free alternative for therapy assessment, especially in non-FDG avid lymphoma subtypes.
**Limitations:** Limited patient cohort.
**Ethics committee approval:** The study was conducted in conformance with the Declaration of Helsinki and approved by the Ethics Commission of the Medical Faculty of the University Duisburg-Essen (study number 11–4822-BO).
**Funding for this study:** No funding was received for this study.
**Author Disclosures:**
Ken Herrmann: Nothing to disclose
Michael Forsting: Nothing to disclose
Johannes Grueneisen: Nothing to disclose
Benedikt Michael Schaarschmidt: Nothing to disclose
Johannes Maximilian Ludwig: Nothing to disclose
Lale Umutlu: Nothing to disclose
Julian Kirchner: Nothing to disclose
Michal Chodyla: Nothing to disclose

### RPS 116-7

**Predicting pathological response to neoadjuvant chemotherapy in osteosarcoma using dynamic contrast-enhanced MRI: internal and external validation of a semi-quantitative model**

*T. Van Den Berghe*[1], G. M. Kalisvaart[2], W. Grootjans[2], M. Lejoly[1], W. Huysse[1], J. V. Bovée[2], D. Creytens[1], K. Verstraete[1], J. L. Bloem[2]; [1]Ghent/BE, [2]Leiden/NL
*(thomasdanae@hotmail.com)*

**Purpose:** Osteosarcoma (OS) patients with poor pathological response (p-PR) to neoadjuvant chemotherapy (NACT) have an impaired prognosis. Accurate preoperative p-PR identification with MRI enables early treatment adaptations, better patient outcomes and less invasive surgery. We developed and validated a predictive p-PR model with dynamic contrast-enhanced (DCE)-MRI. Two segmentation methods and the added value to tumour volume changes were assessed.
**Methods or Backround:** In this retrospective study, patients with OS underwent DCE-MRI pre- and post-NACT with identical scanning protocols and resection (two centres; 2005–2020), forming consecutive training and external validation cohorts. Regions-of-interest were drawn in a blinded way according to the largest tumour slab and the rapid enhancing area method. Relative changes in time-intensity curve parameters and tumour volume were calculated. Patients with >10% viable tumour on pathology post-NACT were labelled p-PR. Logistic regression models for p-PR prediction were trained and cross-validated for both segmentation methods and compared to tumour volume.
**Results or Findings:** In the training and validation cohorts, 55 (27p-PR) and 30 (19p-PR) patients were included. The intraclass correlation coefficient was 0.68 for tumour volume change and ranged 0.82-0.97 and 0.56-0.85 for relative parameters of the slab and area method. A model solely based on tumour volume had a mean accuracy of 0.70 in cross-validation. The best performing model was based on the slab method with a mean accuracy and area under the curve (AUC) of 0.85 and 0.92. A 50% wash-in rate reduction cut-off was deducted as optimal single parameter threshold. In external validation, mean accuracy and AUC were 0.77 and 0.80.
**Conclusion:** Changes in DCE-MRI perfusion characteristics reflect biological changes in OS, induced by NACT. These parameters are predictive for p-PR assessment in external validation.
**Limitations:** No limitations were identified.
**Ethics committee approval:** Both centres.
**Funding for this study:** No funding was received for this study.
**Author Disclosures:**
Gijsbert M. Kalisvaart: Nothing to disclose
Koenraad Verstraete: Nothing to disclose
Maryse Lejoly: Nothing to disclose
Wouter Huysse: Nothing to disclose
Willem Grootjans: Nothing to disclose
Johan L. Bloem: Nothing to disclose
David Creytens: Nothing to disclose
Thomas Van Den Berghe: Nothing to disclose
Judith V.M.G. Bovée: Nothing to disclose

### RPS 116-8

**Lugano versus RECIL criteria in the assessment of treatment response in patients with large-cell B-diffuse lymphoma: preliminary single-centre results**

*N. Di Meglio*, A. Perrella, G. Bagnacci, I. Monteleone, G. Pinton, E. Cencini, A. Fabbri, L. Volterrani, M. A. Mazzei; Siena/IT
*(dimeglionunzia@gmail.com)*

**Purpose:** To verify the concordance of RECIL and Lugano criteria in the evaluation of treatment response in patients with newly diagnosed diffuse large B-cell lymphoma (DLBCL).
**Methods or Backround:** The assessment therapy response in lymphomas is a relevant issue for the correct therapeutic management. Currently, the 2014 Lugano criteria use is recognised at initial staging, early assessment (EA) and restaging at the end of therapy (EOT). To simplify the radiological assessment, RECIL criteria were proposed in 2017, in which only the longest diameter of the target lesions is considered and the number of lesions to be assessed in CT is reduced (maximum 3 instead of 6). However, good concordance has been shown in the review of radiological images from clinical trials and there is a lack of real-life data. Fifty-five newly diagnosed DLBCL patients undergoing first-line chemoimmunotherapy were analysed. CT images were retrospectively evaluated according to RECIL criteria and compared with the Lugano criteria for both EA and EOT assessment. For each patient, target lesions were reviewed and the disease burden was determined.
**Results or Findings:** At EA, the response to therapy (complete and partial, CR and PR) was concordant in 54/55 cases. The only discordant case showed stable disease (SD) according to Lugano and a PR according to RECIL (K-index 0.652). At EOT, 48/55 patients were in CR according to Lugano e and 47/55 according to RECIL (K-index 0.841); between discordant cases, 1 patient had a progression disease (PD) according to RECIL, but not according to Lugano.
**Conclusion:** Treatment assessment according to Lugano and RECIL showed good concordance in our cohort of patients with DLBCL, both at EA and at EOT, supporting the use of the easier RECIL criteria in radiological practice.
**Limitations:** Single-centre, retrospective study.
**Ethics committee approval:** No information provided by the submitter.
**Funding for this study:** No funding was received for this study.
**Author Disclosures:**
Alberto Fabbri: Nothing to disclose
Maria Antonietta Mazzei: Nothing to disclose
Armando Perrella: Nothing to disclose
Emanuele Cencini: Nothing to disclose
Ilaria Monteleone: Nothing to disclose
Nunzia Di Meglio: Nothing to disclose
Luca Volterrani: Nothing to disclose
Gianluca Pinton: Nothing to disclose
Giulio Bagnacci: Nothing to disclose

**08:00-09:00** **Research Stage 4**

### Research Presentation Session: Radiographers

### RPS 114

### Advancements and challenges in radiography education

**Moderators**
L. J. O. C. Lança; Lisbon/PT
D. Mulholland; Dublin/IE

### RPS 114-3

**Academic ultrasound education for radiographers: a survey by the European Federation of Radiographer Societies' (EFRS)**

*G. Harrison*[1], B. Kraus[2], R. Martins Dos Santos[3], M. R. V. Pedersen[4]; [1]London/UK, [2]Wolkersdorf/AT, [3]Coimbra/PT, [4]Vejle/DK
*(GillH@sor.org)*

**Purpose:** Research has shown that ultrasound practice by radiographers varies across Europe. In 2020 the EFRS surveyed education providers to explore academic ultrasound education, accreditation, curricula and course delivery across EFRS countries to identify priorities for professional development and role extension of radiographers in ultrasound.
**Methods or Backround:** Participants were recruited to an online cross-sectional survey by participating national societies of the EFRS (n=43). Questions were predominantly closed questions with options for comments. Descriptive statistics were used to assess the results.
**Results or Findings:** Responses were received from 14 EFRS countries (n=45), predominantly the UK (30%), the Netherlands (12%) and Switzerland (9%). Of these respondents, 28 (62.2%) had involvement in courses leading to a qualification to practise ultrasound. Courses varied from short courses without competency (n=1) or focused on a narrow area of competence (n=6) to undergraduate programmes in ultrasound only (n=2) or as part of the radiography award (n=7) and various postgraduate level awards. Curricula and teaching methods varied and only three countries had external course approval mechanisms. Face-to-face teaching methods were most commonly used (n=21), with four courses being distance learning. Teaching was predominantly by radiologists and radiographers.
**Conclusion:** Academic ultrasound education for radiographers varies across and within EFRS countries, similar to the variation in ultrasound practice. Course type and length differs, as do educational levels. Priorities identified by participants echo previous survey findings, including the development of education and funding streams for radiographers in ultrasound, regulation of sonographers, breaking down barriers and medical protectionism.
**Limitations:** Limitations include the survey language, lack of formal ultrasound education in many countries. Non-responses from some countries may be related to lack of ultrasound education and/or practice.
**Ethics committee approval:** No information provided by the submitter.
**Funding for this study:** No funding was received for this study.
**Author Disclosures:**
Malene Roland Vils Pedersen: Nothing to disclose
Rute Martins Dos Santos: Nothing to disclose
Barbara Kraus: Nothing to disclose
Gill Harrison: Nothing to disclose

### RPS 114-4

**The impact of the COVID-19 pandemic on radiography education: perspective of students and educators**

*L. Ferrari*[1], S. S. Ghotra[1], M. Davis[2], A. Čekada[3], T. Dijkstra TW[4], S. G. McGrath[2], C. Repryntseva[5], M. O'Connor[2]; [1]Lausanne/CH, [2]Dublin/IE, [3]Ljubljana/SI, [4]Groningen/NL, [5]Lisbon/PT
*(Lucaferraritm@gmail.com)*

**Purpose:** The COVID-19 pandemic has affected healthcare education around the globe. This study aimed to investigate the impact of the pandemic on diagnostic radiography education from the perspective of radiography students and educators internationally.
**Methods or Backround:** A qualitative approach was used to explore radiography students' and academics' experiences of radiography education during the pandemic. Sixteen students and three educators were purposively recruited during an international radiography summer school. Four semi-structured focus groups allowed data collection from numerous participants in a rich discussion group setting. Thematic analysis based on Braun & Clarke's six-phase framework was used to identify themes from the participant's experiences of radiography education during the pandemic.
**Results or Findings:** Common themes emerged for both students and educators concerning challenges in pedagogy methods adaptation, technology and internet connectivity issues, vaccination requirements and university support for logistic and mental health problems. Furthermore, educators indicated an increased workload and stress during the pandemic. The sudden conversion of face-to-face lectures to hybrid or online classes was indicated as a big challenge for educators who lacked remote learning experience and time. Students indicated that they felt isolated during online teaching and had a decreased interest in learning. Some participants indicated that the university offered psychological support, while others highlighted the lack of help.
**Conclusion:** This study highlighted some key topics which should be taken into consideration by radiography education institutions to implement adequate and supportive teaching and learning environments for radiography students and educators.
**Limitations:** Possible language barrier for non-native English speakers.
**Ethics committee approval:** No information provided by the submitter.
**Funding for this study:** No funding was received for this study.
**Author Disclosures:**
Michelle O'Connor: Nothing to disclose
Catarina Repryntseva: Nothing to disclose
Michaela Davis: Nothing to disclose
Alja Čekada: Nothing to disclose
Sarah Grace McGrath: Nothing to disclose
Switinder Singh Ghotra: Nothing to disclose
Luca Ferrari: Nothing to disclose
Tim Dijkstra TW: Nothing to disclose

### RPS 114-5

**Radiography students' performance in clinical assessments enhanced through virtual reality (VR) practice**

*M. O'Connor*, L. Rainford; Dublin/IE
*(michelle.oconnor@ucd.ie)*

**Purpose:** Simulation-based learning plays an integral role in preparing students for clinical practice. This study investigated the impact of immersive three-dimensional (3D) virtual reality (VR) simulation-based learning on first-year radiography students' performance in the clinical setting.
**Methods or Backround:** A retrospective analysis of first-year radiography clinical assessments was carried out to compare performance pre-and post-introduction of VR. The stage one cohort with no VR education was considered the control group (n=93). The VR group (n=98) had seven hours of practice in the immersive VR suite (Virtual Medical Coaching). Experienced clinical tutors assessed first-year students performing an extremity radiographic examination in the clinical setting. Assessment criteria were ranked on a 5-point Likert scale from poor to excellent. Mann Whitney U Tests were applied to compare performance across cohorts.
**Results or Findings:** Students trained with VR performed better across 20 of the 22 assessment criteria. VR-trained students performed significantly better (more ranked as 'very good' or 'excellent') than the control group in the following criteria: positioning patients for X-rays (19% difference) ($U = 3525$, $z=-2.66$, $p<0.05$), selecting exposure factors (12% difference) ($U = 3680$, $z=-3.13$, $p<0.05$), image appraisal of patient positioning (27% difference) ($U = 3448$, $z=-2.9$, $p<0.05$) and image appraisal of image quality (18% difference) ($U = 3514$, $z=-2.6$, $p<0.05$).
**Conclusion:** This is the first study to investigate the translation of VR learning into radiography clinical practice. VR learning had an overwhelmingly positive impact on the performance of first year students in their clinical assessment, especially with respect to patient positioning, exposure parameter selection and image appraisal.
**Limitations:** The VR cohort undertook placement and clinical assessment during the pandemic which may have impacted performance.
**Ethics committee approval:** No information provided by the submitter.
**Funding for this study:** No funding was received for this study.
**Author Disclosures:**
Michelle O'Connor: Nothing to disclose
Louise Rainford: Nothing to disclose

### RPS 114-6

**Education and training in radiation protection in Europe: results from the EURAMED Rocc-n-Roll project survey**

*J. Santos*[1], S. J. Foley[2], J. S. Andersson[3], J. P. Figueiredo[1], C. Hoeschen[4], J. Damilakis[5], G. Frija[6], F. Alves[1], G. Paulo[1]; [1]Coimbra/PT, [2]Dublin/IE, [3]Umea/SE, [4]Magdeburg/DE, [5]Iraklion/GR, [6]Paris/FR
*(joanasantos@estescoimbra.pt)*

**Purpose:** To analyse the existing radiation protection (RP) education and training (E&T) capabilities in the European Union and identify associated needs, problems and challenges.
**Methods or Backround:** An online survey was disseminated via the EURAMED Rocc-n-Roll consortium network and prominent medical societies in the field of radiological research. The survey sections analyse the RP E&T during undergraduate, residency/internship and continuous professional

Wednesday development; RP E&T problems and legal implementation. Differences were analysed by European geographic regions, profession, years of professional experience and main area of practice/research.
**Results or Findings:** The majority of the 550 respondents indicated that RP topics are part of undergraduate curricula in all courses for their profession and country (55%), however hands-on practical training is not included according to 30% of the respondents. The lack of E&T, practical aspects in current E&T, and mandatory continuing E&T were considered the major problems. The legal requirement that obtained higher implementation score was the inclusion of the practical aspects of medical radiological procedures on education (86%) and lower score was obtained for the inclusion of RP E&T on medical and dental school curriculums (61%).
**Conclusion:** A heterogeneity in RP E&T during undergraduate, residency/internship and continuous professional development is evident across Europe. Differences were noted per area of practice/research, profession, and European geographic region. A large variation in RP E&T problem rating was also obtained.
**Limitations:** The low frequency of responses and a higher number of responses from 5 countries, can be consider a limitation of the present European survey, that affected the data analyses per EU regions and for some professional groups.
**Ethics committee approval:** No information provided by the submitter.
**Funding for this study:** Funding was received from the Euratom research and training programme 2019-2020 under grant agreement No. 899995.
**Author Disclosures:**
Graciano Paulo: Nothing to disclose
Guy Frija: Nothing to disclose
Christoph Hoeschen: Nothing to disclose
Joana Santos: Nothing to disclose
João Paulo Figueiredo: Nothing to disclose
Shane J Foley: Nothing to disclose
Jonas Seth Andersson: Nothing to disclose
John Damilakis: Nothing to disclose
Francisco Alves: Nothing to disclose

### RPS 114-7

**Practice educators' experiences of supporting radiography clinical placements during the COVID-19 pandemic**

*M. O'Connor*, A. Lunney, J. Potočnik, D. Kearney, J. M. Grehan; Dublin/IE
*(michelle.oconnor@ucd.ie)*

**Purpose:** The COVID-19 pandemic has significantly impacted healthcare services and the clinical learning environment. Several studies have investigated radiography students' experiences of clinical placement during the pandemic; however, few have investigated the Clinical Practice Educator's (CPEs) perspective. CPEs play a pivotal role in supporting clinical education.
**Methods or Backround:** A qualitative study was conducted using a purposeful sample of twenty-two CPEs, each working in a different Irish hospital. Four semi-structured focus groups were used to gather data. To maintain reasonable homogeneity, CPEs who were new to the role (n=8) were assigned a separate focus group from experienced CPEs (n=14). Inductive thematic analysis was applied.
**Results or Findings:** CPEs experienced role expansion, particularly in managerial and administrative aspects of the role. They described arranging COVID-19 vaccinations locally for radiography students and the complexities of student rostering during the pandemic. CPEs perceived the pandemic to have impacted students' emotional well-being with 'high anxiety levels' and 'loneliness' as issue. They also perceived issues with clinical readiness and the student transition to clinical practice. Many challenges were faced by CPEs including arranging clinical recovery time for numerous students when sites were already at full capacity, fewer learning opportunities due to decreased patient throughput and range of imaging examinations, social distancing constraints, resistance from staff to student placements, and a shortage of staff for student supervision. Flexibility, communication, and multi-level support helped CPEs to fulfill their role.
**Conclusion:** The results provide insight into how CPEs supported radiography clinical placements during the pandemic and into the challenges faced by CPEs in their role. Lessons learned from supporting radiography clinical placements during the pandemic were outlined.
**Limitations:** Focus group facilitators work with participants which may impact openness and honesty of CPE responses.
**Ethics committee approval:** No information provided by the submitter.
**Funding for this study:** No funding was received for this study.
**Author Disclosures:**
Michelle O'Connor: Nothing to disclose
Aine Lunney: Nothing to disclose
Jennifer Mary Grehan: Nothing to disclose
Jaka Potočnik: Nothing to disclose
Dearbhla Kearney: Nothing to disclose

### RPS 114-8

**Is radiography ready for a new level of graduate professional?**

*A. Gill*, M. Hardy, E. Wilkinson; Bradford/UK
*(a.gill8@bradford.ac.uk)*

**Purpose:** The UK radiography regulatory body (Health and Care Professions Council, HCPC) have published the new Standards of Proficiency (SoP) for radiographers applicable to all students graduating from September 2023. The standards have been expanded to include wider personal and professional competencies in addition to multi-modality technical competencies across projection, computed tomography and magnetic resonance modalities, emphasising the professional and technical skills necessary to meet future service delivery demands. However, it is uncertain whether clinical radiographers and placement providers are ready to support the new learner requirements. It is also unclear if and how employers are reviewing radiographer roles and responsibilities, including upskilling of existing staff, to ensure that they radiographer skills are fully utilised.
**Methods or Backround:** Thematic analysis of the phrasing of the new HCPC SoP for radiographers was undertaken to identify themes and sub-categories. Clinical radiographers from across a large geographic region in Northern England were invited to participate in a series of focus groups to explore these themes and consider level of competence expectation of students, new graduates and experienced radiographers. Approaches to determining competence and assessment of skills were also explored.
**Results or Findings:** Analysis of the new HCPC SoP identified 6 clear themes: "communication", "person-centred care", "key concepts of radiography", "legislation", "development" and "innovation and leadership". The focus groups are currently ongoing and simultaneous analysis undertaken.
**Conclusion:** The new HCPC SoP challenge current thinking of radiographer education, roles and responsibilities. Work is required to ensure that the skills of the current workforce, as well as new graduates, are optimised and new models of working devised if the future workforce is to fulfil its potential.
**Limitations:** Only covered opinions of radiographers in Northern England.
**Ethics committee approval:** No information provided by the submitter.
**Funding for this study:** Health Education England have funded this project.
**Author Disclosures:**
Elaine Wilkinson: Nothing to disclose
Maryann Hardy: Nothing to disclose
Avneet Gill: Nothing to disclose

### RPS 114-9

**The future of continuing professional development (CPD) for diagnostic radiographers in Ireland**

*J. M. Grehan*, L. Rainford, M-L. Ryan; Dublin/IE

**Purpose:** CPD is mandatory for state registration for Irish radiographers since October 2015. Two nationwide surveys had been undertaken, one at the introduction of mandatory CPD and a second two years later. This focus group study investigated the experiences of different grades of radiographer to mandatory CPD through themes around CPD participation that had resulted from these two earlier surveys, in order to establish potential future direction for radiographers in this area.
**Methods or Backround:** A discussion guide of seven areas covering time constraints, the state regulator, delivery methods, personal/professional drivers, relevance and responsibility was developed. Participants were recruited through email, social media posts, professional body websites, snowball and purposive sampling and opinion was captured through online focus groups.
**Results or Findings:** 12 online focus groups (48 radiographers) were undertaken with all grades of represented. Focus groups were grade and environment specific (i.e. public or private). All involved offered a range of opinion across areas discussed. Themes emerged from the analysis around: (1) Leadership and support; unilaterally there was a desire for more protected time and better support. (2) Structure of CPD programmes; all groups craved a solid structure under which to accrue and acquire CPD credits. (3) Delivery methods; a hybrid method of delivery allows ongoing flexibility. (4) Protecting time; personal time must be protected therefore work time must be allocated. (5) Progression: little or no progression around CPD has been made since its introduction.
**Conclusion:** This study identified radiographers' continued desire to undertake CPD, a lack of standardisation across sites and the need for more hybrid offerings. An overall positive perception towards CPD was noted with an aspiration to find solutions to perceived barriers going forward.
**Limitations:** Recruitment and issues around bias were experienced and mitigated.
**Ethics committee approval:** No information provided by the submitter.
**Funding for this study:** No funding was received for this study.
**Author Disclosures:**
Louise Rainford: Nothing to disclose
Marie-Louise Ryan: Nothing to disclose
Jennifer Mary Grehan: Nothing to disclose

**RPS 114-10**

**COVID-19's impact on the recent graduate in health professions**

*A. Devetti*[1], G. Santucci[2], I. G. Rigott[3], A. Paccone[4], A. Attanasio[5], P. Cornacchione[6], S. Da Dalt[1], M. Zanardo[7], C. Martini[8]; [1]Udine/IT, [2]Cesena/IT, [3]Bolzano/IT, [4]Naples/IT, [5]Sassari/IT, [6]Rome/IT, [7]Milan/IT, [8]Parma/IT

**Purpose:** The purpose of this study was to evaluate the impact of COVID-19 on recent health profession graduates by assessing the intensity of anxiety and depression symptoms, as well as their impact on clinical placement and academic career.
**Methods or Backround:** A 23-question online survey was given to students participating in bachelor's degree programs in health professions (except nurses and obstetricians). We gathered sociodemographic data as well as responses to a 5-point scale on anxiety, depressive symptoms, clinical placement, and academic career.
**Results or Findings:** The survey was completed online by 132 undergraduates. Almost two-thirds are female and mostly aged up to 24 years, so they were within the prescribed time. 60% were aspiring radiographers or radiotherapy technicians, 14% were physiotherapists, 11% were medical laboratory technicians, and 20 were pursuing other health professions. Since the beginning of the COVID-19 pandemic, 72% of students report that less than 10% of their practical training hours has been replaced by online training, with 14% experiencing more than 6 months of practical training disruption. 70% of students claim that the pandemic has hampered their academic progress, although just 23% think their graduation date has been delayed. During clinical training, 25% of respondents reported feeling anxious, and 22% reported feeling depressed. Only 11% of students regret taking a health professions college course after the COVID-19 pandemic began.
**Conclusion:** The COVID-19 pandemic had almost no effect on practical training, but it had a significant impact on university careers and graduation rates among healthcare students. Undergraduates occasionally expressed anxiety and depressive states, but generally they did not regret the academic path they had chosen.
**Limitations:** The study had a limited data collection period of 9 months (24/09/2021–07/07/2022) and a limited number of respondents.
**Ethics committee approval:** University of Parma
**Funding for this study:** No funding was received for this study.
**Author Disclosures:**
Gioele Santucci: Nothing to disclose
Patrizia Cornacchione: Nothing to disclose
Stefano Da Dalt: Nothing to disclose
Angie Devetti: Nothing to disclose
Antonio Attanasio: Nothing to disclose
Moreno Zanardo: Nothing to disclose
Chiara Martini: Nothing to disclose
Antonella Paccone: Nothing to disclose
Irene Gertrud Rigott: Nothing to disclose

**09:30-11:00** **Research Stage 1**

### Research Presentation Session: Abdominal Viscera & GI Tract

### RPS 201

### Advances in imaging of hepatocellular carcinoma

**Moderator**
F. Vernuccio; Padova/IT
*(federicavernuccio@gmail.com)*

**RPS 201-2**

**Risk factors of hepatocellular carcinoma after direct-acting antiviral treatment: a prospective multicentre study**

*S. Ichikawa*[1], U. Motosugi[2], Y. Sawai[3], K. Kozaka[4], M. Tsurusaki[5], K. Sofue[6], N. Kawai[7], Y. Fukukura[8], S. Goshima[1]; [1]Hamamatsu/JP, [2]Kofu/JP, [3]Ikeda/JP, [4]Kanazawa/JP, [5]Osakasayama/JP, [6]Kobe/JP, [7]Gifu/JP, [8]Kagoshima/JP

**Purpose:** To determine the risk factors of hepatocellular carcinoma (HCC) development after direct-acting antiviral (DAA) treatment.
**Methods or Backround:** This multicentre study was approved by the institutional review boards of the seven participating centres. We enrolled patients with chronic hepatitis C who were treated with DAAs and who achieved SVR at 12 weeks (SVR12) between 2012 and 2018. Patients were followed up prospectively after SVR12 was achieved. The main outcome was HCC development or the date of the follow-up at which the absence of HCC was confirmed. Univariate and multivariate Cox proportional hazards models were used to identify factors, including gadoxetic acid-enhanced magnetic resonance imaging findings related to HCC development. The cumulative incidence rates of HCC development were calculated using the Kaplan–Meier method, and group differences in the curves were assessed using the log-rank test.
**Results or Findings:** The final study cohort comprised 482 patients (mean age, 69.5 ± 10.6 [range, 31–90] years). The mean follow-up period was 34.8 ± 13.1 (median, 36.8) months. Of the 482 patients, 96 developed HCC (19.9%). The 1-, 3-, and 5-year cumulative incidence of HCC development was 4.9%, 18.6%, and 30.5%, respectively. Multivariate analysis revealed that age, male sex, history of HCC, hepatobiliary phase (HBP) hypointense nodule without arterial phase hyperenhancement (APHE), and albumin were independent risk factors significantly associated with HCC development ($P < 0.001$–$0.047$). The highest risk group comprised patients with both a history of HCC and an HBP hypointense nodule without APHE (the 1- and 3-year cumulative incidence of HCC development was 14.2% and 62.2%, respectively).
**Conclusion:** Both, history of HCC and HBP hypointense nodule without APHE were strong risk factors of HCC development after DAA treatment.
**Limitations:** No limitations were identified.
**Ethics committee approval:** This multicentre study was approved by the institutional review boards of the seven participating centres.
**Funding for this study:** Sponsored by Bayer Yakuhin, Ltd. without role regarding the study performance.
**Author Disclosures:**
Shintaro Ichikawa: Nothing to disclose
Nobuyuki Kawai: Nothing to disclose
Masakatsu Tsurusaki: Nothing to disclose
Kazuto Kozaka: Nothing to disclose
Satoshi Goshima: Nothing to disclose
Yoshiyuki Sawai: Nothing to disclose
Utaroh Motosugi: Nothing to disclose
Keitaro Sofue: Nothing to disclose
Yoshihiko Fukukura: Nothing to disclose

**RPS 201-3**

**The feasibility of early response evaluation using Superb Microvascular Imaging one day after transcatheter arterial chemoembolisation for hepatocellular carcinoma**

H. Kwon, K. Lim, J. Cho, *E. R. Jeong*, B. Goo; Busan/KR

**Purpose:** To determine the feasibility of early Superb Microvascular Imaging (SMI) for the prediction of HCC treatment effect after transcatheter arterial chemoembolisation (TACE).
**Methods or Backround:** Between September 2021 and May 2022, 100 HCCs (73 patients) treated with TACE were included in this study. SMI, Color Doppler imaging (CDI) and Power Doppler imaging (PDI) were performed the day after TACE for the evaluation intratumoural vascularity of the lesion by using Aplio500 ultrasound scanner (Toshiba Medical Systems, Corporation, Tochigi, Japan). The vascular presence was graded on a five-point scale. The dynamic CT after 1-month image was used to compare sensitivity, specificity, and accuracy for detecting tumour vascularity between SMI, CDI and PDI. Factors affecting intratumoural vascularity had assessed by univariate and multivariate analysis.
**Results or Findings:** 60 lesions (60%) had complete remission (CR) and 40 lesions (40%) had partial (PR) or no response at 4 weeks MDCT after TACE. SMI showed 82.5% sensitivity for intratumoural flow detection, which was significantly higher than that of CDI (10%, $p<0.001$) or PDI (37.5%, $p<0.001$). On multivariate analysis, tumour size and tumour depth from the skin were significant factors of blood flow detection.
**Conclusion:** Early SMI may be utilised as an adjunctive diagnostic test for evaluation of treated lesions after TACE, especially when the tumours are located in an area of the liver with a good sonic window.
**Limitations:** (1) CDI, PDI, and SMI in a sequential manner in each patient: recall bias. (2) Only one microvascular imaging technique from a single vendor: comparisons of different microvascular imaging data from several vendors are warranted.
**Ethics committee approval:** No information provided by the submitter.
**Funding for this study:** This study was funded by the Dong-A university research fund.
**Author Disclosures:**
Heejin Kwon: Nothing to disclose
Bonjae Goo: Nothing to disclose
Eo Ram Jeong: Nothing to disclose
Jinhan Cho: Nothing to disclose
Kyungjae Lim: Nothing to disclose

### RPS 201-4

**Predicting overall survival after hepatocellular carcinoma resection using the COMA score: a dual-institutional study**

*H. Wei*, H. Jiang, B. Song; Chengdu/CN
*(weih_cat@163.com)*

**Purpose:** To develop and validate a risk score based on preoperative clinical-radiological parameters for predicting overall survival (OS) in patients undergoing hepatectomy for hepatocellular carcinoma (HCC).
**Methods or Backround:** From July 2010 to December 2021, consecutive patients with surgically-proven HCC who underwent preoperative contrast-enhanced MRI were retrospectively enrolled. A preoperative OS risk score was constructed in the training cohort using a Cox regression model and validated in a propensity score-matched internal validation cohort and an external validation cohort.
**Results or Findings:** A total of 520 patients were enrolled, among whom 210, 210, and 100 patients were from the training, internal validation, and external validation cohorts, respectively. Independent predictors for OS included incomplete tumour "capsule", mosaic architecture, tumour multiplicity and serum alpha-fetoprotein (AFP), which were incorporated into the risk score (COMA score). The C-index for the COMA score was 0.85, 0.81 and 0.62 in the training, internal and external validation cohorts, respectively. Using 32 as the cut-off point, the COMA score stratified patients into prognostically distinct low- and high-risk groups in all cohorts (all P <0.05).
**Conclusion:** Incorporating three MRI features serum AFP, the COMA score enabled accurate prediction of postsurgical OS in HCC patients.
**Limitations:** This study was limited by the retrospective design, and further prospective studies are warranted to confirm the presented results.
**Ethics committee approval:** This retrospective dual-institutional study was approved by the institutional review boards of participating institutions. The requirements for informed consent were waived.
**Funding for this study:** This study has received funding by the National Natural Science Foundation of China (No. 81971571, 82101997)
**Author Disclosures:**
Hong Wei: Nothing to disclose
Hanyu Jiang: Nothing to disclose
Bin Song: Nothing to disclose

### RPS 201-5

**Transversal psoas muscle thickness measurement is associated with response and survival in patients with hepatocellular carcinoma undergoing immunotherapy**

*K. Lampichler*[1], B. Scheiner[1], L. Beer[1], M. Trauner[1], M. Scharitzer[1], R. Sartoris[2], D. Pinato[3], M. Ronot[2], M. Pinter[1]; [1]Vienna/AT, [2]Clichy/FR, [3]London/UK

**Purpose:** Sarcopenia is a common problem in patients with hepatocellular carcinoma (HCC) and may be diagnosed using clinical or imaging-based assessments, including the measurement of the transversal psoas muscle thickness (TPMT). The aim of this study was to evaluate the prognostic and predictive value of TPMT at baseline in patients with hepatocellular carcinoma undergoing immunotherapy (IT).
**Methods or Backround:** Patients with HCC treated with PD-(L)1-based therapies between 6/2016 and 10/2022 at two university hospitals (Centre 1, n=80 and Centre 2, n=96) were included. TPMT measurements at the level of the third lumbar vertebrae were performed by two independent readers in each centre. TPMT <12 mm/m in men and <8 mm/m in women indicated sarcopenia. Inter-reader reliability was assessed in the cohort from Centre 1 with the Cohen’s Kappa statistics. The influence of sarcopenia on overall survival (OS) was assessed by computing Kaplan Meyer curves. Radiological response was evaluated by mRECIST.
**Results or Findings:** 176 patients were included (age: 66.3±11.7 years, 81% male), of which 131 (74%) also had liver cirrhosis. Inter-reader reliability for TPMT-based sarcopenia was 'substantial' (kappa = 0.795). Sarcopenia was present in 34% of patients (n=59) and was significantly more frequent in men than in women (39% vs. 9%, p=0.001). OS was significantly shorter in sarcopenic patients than in non-sarcopenic patients (median 8.0 [95%CI:4.1-11.9] vs. 24.7 [95%CI:19.1-30.4]; p<0.001). Radiological response was evaluable in 161 subjects (91.5%). The objective response rate (ORR) was lower in patients with sarcopenia (24.5% (n=12/49)) compared to those without sarcopenia (41.1% (n=46/112; p=0.044)).
**Conclusion:** The assessment of sarcopenia using TPMT measurement is reliable and identifies HCC patients with a dismal prognosis and lower response rate to immunotherapy.
**Limitations:** Retrospective analysis.
**Ethics committee approval:** No information provided by the submitter.
**Funding for this study:** No funding was received for this study.
**Author Disclosures:**
David Pinato: Consultant: Mina Therapeutics, DaVolterra, Mursla, IPSEN, Exact Sciences, Avamune, EISAI, Roche, and Astra Zeneca Other: DJP received lecture fees from ViiV Healthcare, Bayer Healthcare, EISAI, BMS, Roche and travel expenses from BMS and Bayer Healthcare Research/Grant Support: MSD, GSK and BMS
Bernhard Scheiner: Other: Travel grants from AbbVie, Ipsen, Gilead
Matthias Pinter: Other: Travel support from Bayer and Bristol-Myers Squibb Consultant: Bayer, Bristol-Myers Squibb, Eisai, Ipsen, Lilly, MSD, and Roche
Maxime Ronot: Nothing to disclose
Lucian Beer: Nothing to disclose
Michael Trauner: Other: Speaker fees by Bristol-Myers Squibb, Falk, Gilead, Intercept and MSD; travel support by AbbVie, Falk, Gilead, and Intercept Grant Recipient: Albireo, Almylam, Cymabay, Falk, Gilead, Intercept, MSD, Takeda, and Ultragenyx Consultant: Albireo, Boehringer Ingelheim, BiomX, Falk, Genfit, Gilead, Intercept, Janssen, MSD, Novartis, Phenex, Pliant and Regulus
Katharina Lampichler: Nothing to disclose
Riccardo Sartoris: Nothing to disclose
Martina Scharitzer: Nothing to disclose

### RPS 201-6

**Prognostic value of baseline MRI features in patients treated with thermal ablation for hepatocellular carcinoma**

*O. Öcal*[1], S. Deniz[1], C. Loewe[2], O. M. Van Delden[3], M. Pech[4], J. Ricke[1], M. Seidensticker[1]; [1]Munich/DE, [2]Vienna/AT, [3]Amsterdam/NL, [4]Magdeburg/DE
*(osman.oecal@med.uni-muenchen.de)*

**Purpose:** To investigate prognostic value of baseline MRI features for time-to-recurrence (TTR) in patients with early hepatocellular carcinoma (HCC).
**Methods or Backround:** Baseline and follow-up images of the 88 patients treated with thermal ablation due to HCC in this multicentre study retrospectively evaluated. Baseline MRI images were evaluated in terms of atypical enhancement), lesion diameter, tumour capsule, peritumoral enhancement on arterial phase, intratumoural fat, irregular margin, and satellite lesions. Prognostic value of these features for TTR was assessed with univariable and multivariable Cox proportional hazard models.
**Results or Findings:** Recurrence was diagnosed during follow-up in 32 patients, and the median time-to-recurrence was 16 (95% CI, 14.8 – NA) months. Presence of more than one lesion (p=0.028) and peritumoural hypointensity on hepatobiliary phase images (p=0.012) were significantly associated with shorter TTR in univariable analysis. Lesion diameter (>25 mm; p=0.068), AFP > 15mg/dL (p=0.084), and history of cirrhosis (p=0.099) were marginally non-significant. Peritumoural hypointensity on hepatobiliary phase images was the only significant risk factor in multivariable analysis.
**Conclusion:** Peritumoural hypointensity on hepatobiliary phase can serve as imaging biomarkers to identify increased recurrence risk in patients undergo thermal ablation due to early-stage HCC.
**Limitations:** The retrospective nature of image read and the single reader for baseline MRI analysis.
**Ethics committee approval:** No information provided by the submitter.
**Funding for this study:** This study received financial support from Sirtex Medical and Bayer Healthcare.
**Author Disclosures:**
Otto M Van Delden: Nothing to disclose
Max Seidensticker: Grant Recipient: Bayer Healthcare Grant Recipient: Sirtex
Sinan Deniz: Nothing to disclose
Maciej Pech: Nothing to disclose
Osman Öcal: Nothing to disclose
Christian Loewe: Nothing to disclose
Jens Ricke: Grant Recipient: Sirtex Grant Recipient: Bayer Healthcare

### RPS 201-7

**The future of hepatocellular carcinoma screening: non-contrast abbreviated MRI versus dynamic abbreviated MRI**

*R. D. Girardet*, G. Manasseh, M. Dubois, C. Du Pasquier, E. Canniff, Y. Aleman, M. Fraga Christinet, C. Dromain, N. Vietti Violi; Lausanne/CH
*(girardet.raphael@gmail.com)*

**Purpose:** To compare the diagnostic performance of two abbreviated MRI sets [non-contrast (NC)-AMRI, and dynamic (Dyn)-AMRI] to complete MRI for HCC detection in an at-risk population.
**Methods or Backround:** This retrospective study included 351 patients (M/F 264/87, mean age 58 y.o.) with chronic liver disease, who underwent extracellular contrast-enhanced MRI between 2014 and 2020 at our institution, corresponding to 631 MRIs. Two reconstructed AMRI sets, NC-AMRI [T2-weighted imaging (T2wi) + diffusion-weighted imaging (DWI)], Dyn-AMRI [T2wi + dynamic T1wi] and complete MRI were assessed by 2 independent radiologists. Lesions were categorised using a composite scoring system for NC-AMRI [negative, subthreshold (<10mm), positive] and LI-RADS v2018 algorithm for Dyn-AMRI and complete MRI. Each exam was classified as HCC-positive or HCC-negative according to the reference standard, based on all available patient data including pathology and 6-months follow-up for the negative exams. Interreader agreement was assessed. Diagnostic performance of the AMRI sets and complete MRI were compared.
**Results or Findings:** The reference standard found 83/351 HCC positive patients (incidence 23.6%, median size 22 mm, range 10-142mm), thus 83/641

Wednesday positive MRIs. Interreader agreement was substantial for all sets; k=0.68 (NC-AMRI), 0.67 (Dyn-AMRI) and 0.68 (complete MRI). Sensitivities of Dyn-AMRI [92.8% (77/83); 95% confidence interval (CI) 90.4-94.6%] and complete MRI [92.8% (77/83); 90.4-94.6%)] were significantly higher than NC-AMRI [72.3% (60/83); 68.6-75.7%] when considering 95% CIs. Specificity was not significantly different between sets: NC-AMRI [94.5%; 92.4-96.1%], Dyn-AMRI [96.3%; 94.5-97.6%] and complete MRI [96%; 94.1-97.4%].
**Conclusion:** In this large retrospective study performed with extracellular contrast, Dyn-AMRI was highly sensitive for HCC screening as it presented similar diagnostic performance to complete MRI, with higher sensitivity compared to NC-AMRI.
**Limitations:** Retrospective design. Selected patient population rather than a true screening population (HCC rate: 23.6%).
**Ethics committee approval:** ID CER-VD 2020-00680
**Funding for this study:** No funding was received for this study.
**Author Disclosures:**
Raphaël Dominique Girardet: Nothing to disclose
Gibran Manasseh: Nothing to disclose
Montserrat Fraga Christinet: Nothing to disclose
Yasser Aleman: Nothing to disclose
Emma Canniff: Nothing to disclose
Céline Du Pasquier: Nothing to disclose
Naïk Vietti Violi: Nothing to disclose
Margaux Dubois: Nothing to disclose
Clarisse Dromain: Nothing to disclose

### RPS 201-8

**Y-90 radioembolisation for intermediate to advanced hepatocellular carcinoma: AFP response patterns and correlation with radiologic disease progression**
J. Wen, L. Shen, *J. R. Tse*; Stanford, CA/US
*(justinrtse8@gmail.com)*

**Purpose:** To describe alpha-fetoprotein (AFP) response patterns to Barcelona Clinic Liver Cancer (BCLC) stage B or C hepatocellular carcinoma (HCC).
**Methods or Backround:** This single-centre, retrospective study evaluated 78 adult patients from 2015-2022 with treatment-naïve, AFP producing HCC (AFP>20 ng/mL) without extrahepatic metastases who received Y-90 radioembolisation. Pre-treatment and post-treatment multiphasic CT and/or MRI at 3- and 6- months were evaluated for progression, defined as new HCC, new local invasion (e.g. tumour in vein), extrahepatic metastases, or death.
**Results or Findings:** 78 patients were included: 38 BCLC Stage B and 40 BCLC Stage C; mean age 68 years. The AFP treatment response was categorised into 3 patterns: 1) sustained response (sustained post-procedure AFP decrease by ≥20%; n=21), 2) transient response (AFP decrease by ≥20%, followed by increase back to or above baseline; n=26), and 3) no response (<20% AFP decrease or increase; n=31). Of patients with no response, 27/31 (87%) progressed at 3-month follow-up and 29/31 (94%) progressed by 6-month follow-up. Of patients with transient response, 16/26 (62%) progressed at 3-month follow-up and 24/26 (92%) progressed at 6-month follow-up. Of patients with sustained response, 4/21 (19%) progressed at 3-month follow-up and 5/21 (24%) progressed at 6-month follow-up (p<0.001). Among all patients, the most common cause for progression was new HCC (n=53), followed by new local invasion (n=21), nodal (n=17), lung (n=13), adrenal (n=10), osseous (n=6), and peritoneal metastases (n=5).
**Conclusion:** No AFP response and transient AFP response are associated with early and delayed progression, respectively. Only about 1/4 of patients have sustained AFP response, which is associated with progression-free survival. The most common cause of progression was increased HCC burden or local invasion. The most common extrahepatic metastases were to lymph nodes and lungs.
**Limitations:** Retrospective, single-centre.
**Ethics committee approval:** This study was approved by Stanford University Institutional Review Board
**Funding for this study:** No funding was received for this study.
**Author Disclosures:**
Luyao Shen: Nothing to disclose
Justin Ruey Tse: Nothing to disclose
Jessica Wen: Nothing to disclose

### RPS 201-9

**Deep learning model with convolutional neural network for detecting and segmenting hepatocellular carcinoma in CT: a preliminary study**
D. Vo Tan, *C. C. Phan*, et al.; Ho Chi Minh/VN

**Purpose:** This study aimed to develop a convolutional neural network-based model to identify and segment hepatocellular carcinoma (HCC) lesions utilising dynamic contrast agent-enhanced computed tomography (CT).
**Methods or Backround:** This retrospective study used CT image sets of histopathology-confirmed hepatocellular carcinoma over three phases (arterial, venous, and delayed). The proposed convolutional neural network (CNN) segmentation method was based on the U-Net architecture and trained using the domain adaptation technique. The proposed method was evaluated using 115 liver masses of 110 patients (87 men and 23 women; mean age, 56.9 years ± 11.9 (SD); mean mass size, 6.0 cm ± 3.6). The sensitivity for identifying the HCC of the model and Dice score for segmentation of liver masses between radiologists and the CNN model were calculated for the test set.
**Results or Findings:** The sensitivity for HCC identification of the model was 100%. The median Dice score for HCC segmenting between radiologists and the CNN model was 0.81 for the test set.
**Conclusion:** Deep learning with CNN had high performance in the identification and segmentation of HCC on dynamic CT.
**Limitations:** There are some drawbacks to this study. First, we only used a dataset from one institute to validate the model. Second, because we only employed CT scanners from one vendor, a decrease in performance may occur when applying the algorithm to different CT systems.
**Ethics committee approval:** No information provided by the submitter.
**Funding for this study:** No funding was received for this study.
**Author Disclosures:**
Chien Cong Phan: Nothing to disclose
et al.: Nothing to disclose
Duc Vo Tan: Nothing to disclose

### RPS 201-10

**Magnetic resonance elastography (MRE)-based nomogram for predicting proliferative hepatocellular carcinoma**
*G. Liu*, J. Zhou, R. Li, F. Yan; Shanghai/CN

**Purpose:** To investigate the viscoelastic signatures of proliferative hepatocellular carcinoma (HCC) using three-dimensional (3D) magnetic resonance elastography (MRE) and establish corresponding prediction models.
**Methods or Backround:** This prospective study included 121 patients with 124 pathologically confirmed HCCs (63 proliferative, 61 non-proliferative) who underwent preoperative conventional magnetic resonance imaging and 3D MRE from July 2020 to November 2021. Viscoelastic parameters of the tumour and liver were quantified as shear wave speed (c, m/s) and loss angle (φ, rad), representing stiffness and fluidity, respectively. Major imaging features (rim arterial phase hyperenhancement [APHE], non-peripheral washout, capsule, peritumoural enhancement, tumour margin) were evaluated. Multivariate logistic regression analyses were used to determine predictors of proliferative HCC to construct corresponding nomograms.
**Results or Findings:** Tumour c, tumour φ, cirrhosis, hepatitis virus, rim APHE, peritumoural enhancement, and tumour margin (all p <0.05) were independent predictors of proliferative HCC. Model-1 was established (cirrhosis, hepatitis virus, rim APHE, peritumoural enhancement, tumour margin) yielded an area under the curve (AUC), sensitivity, specificity, accuracy of 0.72 (95%CI: 0.64–0.80), 58.73 (95%CI:45.6–71.0), 78.69(95%CI:66.3–88.1), 67.74 (95%CI:58.76–75.85), respectively. When adding MRE properties (tumour c and tumour φ) , established model-2, the AUC increased to 0.81 (95%CI: 0.72–0.87), with sensitivity, specificity, accuracy of 71.43 (95%CI:58.7–82.1), 81.97 (95%CI:70.0–90.6), 75 (95%CI: 66.43–82.34), respectively. The C-index of the nomogram of model-2 was 0.81, showing good performance for proliferative HCC.
**Conclusion:** Proliferative HCC exhibits low stiffness and high fluidity. Combining MRE properties can improve performance of preoperative diagnosis of proliferative HCC.
**Limitations:** First, this was a single-centre exploratory study. Second, most patients had viral hepatitis. Finally, proliferative HCCs and non-proliferative HCCs were classified based on histopathological analyses.
**Ethics committee approval:** No information provided by the submitter
**Funding for this study:** This study has received funding by Shanghai Science and Technology Fundation (21TS1400600), and the Deutsche Forschungsgemeinschaft (BIOQIC GRK 2260 and SFB1340 Matrix-In-Vision).
**Author Disclosures:**
Ruokun Li: Nothing to disclose
Guixue Liu: Nothing to disclose
Fuhua Yan: Nothing to disclose
Jiahao Zhou: Nothing to disclose

### RPS 201-11

**Risk stratification of solitary hepatocellular carcinoma ≤5 cm without microvascular invasion: prognostic values of MR imaging features and clinical parameters**
*W. Leyao*, X. Ma, X. Zhao; Beijing/CN

**Purpose:** To estimate the potential of preoperative MR imaging features and clinical parameters in the risk stratification of patients with solitary hepatocellular carcinoma (HCC) ≤5 cm without microvascular invasion (MVI) after hepatectomy.
**Methods or Backround:** The study enrolled 166 patients with histopathologically confirmed MVI-negative HCC retrospectively. The MR imaging features were evaluated by two radiologists independently. The independent risk factors associated with recurrence-free survival (RFS) were identified by univariate Cox analysis and the least absolute shrinkage and selection operator (LASSO) Cox regression analysis. A predictive nomogram was developed based on these predictors and the performance was tested in the validation cohort. The RFS was analysed by using the Kaplan–Meier survival curves and log-rank test.
**Results or Findings:** Among the 166 patients with solitary MVI-negative HCC, 86 (51.81%) presented with postoperative recurrence. Multivariate Cox analysis indicated that cirrhosis, tumour size, hepatitis, albumin, arterial phase hyperenhancement (APHE), washout, and mosaic architecture were independent predictors of poor RFS and were incorporated into the nomogram. The nomogram achieved good performance with C-index value of 0.713 and 0.707 in the development and validation cohorts, respectively. Furthermore, patients were stratified into high- and low-risk subgroups based on the median value of the nomogram score, and significant prognostic differences were found between the different subgroups in both cohorts (P < 0.001 and P = 0.024, respectively).
**Conclusion:** The nomogram incorporated preoperative MR imaging features and clinical parameters can be a simple and reliable tool for predicting RFS and achieving risk stratification in patients with solitary MVI-negative HCC.
**Limitations:** Not applicable.
**Ethics committee approval:** This study approved by the Ethics Committee of Cancer Hospital, Chinese Academy of Medical Sciences.
**Funding for this study:** Not applicable.
**Author Disclosures:**
Xiaohong Ma: Nothing to disclose
Xinming Zhao: Nothing to disclose
Wang Leyao: Nothing to disclose

### RPS 201-12

**CT radiomics for the prediction of microvascular invasion and survival in patients with HCC: is it a matter of chance or standardisation?**

*R. Cannella*[1], M. Dioguardi Burgio[2], J. Santinha[3], A. Beaufrère[2], N. Papanikolaou[3], V. Vilgrain[2]; [1]Palermo/IT, [2]Clichy/FR, [3]Lisbon/PT

**Purpose:** To test the performance of a radiomics-based model for the prediction of microvascular invasion (MVI) and survival by temporal splitting a cohort of patients with resected HCC acquired with different CT scanners. To evaluate the variability of radiomics performance by generating multiple random partition models.
**Methods or Backround:** This study selected 230 patients (181 men, mean age of 62.3±12.7 years) with 242 pathologically-proven HCCs who underwent CT, of which 73/230 (31.7%) were scanned in external centres with a variety of CT scanners. The study cohort was spitted into training set (158 patients, 165 HCCs) and held-out test set (72 patients, 77 HCCs), stratified by random partitioning, which was repeated 100 times, and by a temporal partitioning to simulate the sequential development and clinical use of the radiomics model. A machine learning model for the prediction of MVI was developed with least absolute shrinkage and selection operator (LASSO). The concordance Index (C-Index) was used to assess the recurrence-free (RFS) and overall survivals (OS).
**Results or Findings:** In the 100-repetition randomly stratified cohorts, the radiomics model demonstrated a mean AUC of 0.54 (range in the 100 partitions of 0.44-0.68) for the prediction of MVI, mean C-index of 0.59 (range 0.44-0.73) for RFS, and 0.65 (range 0.46-0.86) for OS in the held-out test set. In the temporal partitioning cohort, the radiomics model yielded an AUC of 0.50 for the prediction of MVI, a C-index of 0.61 for RFS and 0.61 for OS, in the held-out test set.
**Conclusion:** Radiomics models had a poor performance for MVI when attempting to simulate its sequential development and application in a multicentric cohort. Radiomic models by using random partitioning yields to large variability in the model performance.
**Limitations:** Inclusion of surgically-resected patients only.
**Ethics committee approval:** No information provided by the submitter.
**Funding for this study:** No funding was received for this study.
**Author Disclosures:**
Marco Dioguardi Burgio: Nothing to disclose
Aurélie Beaufrère: Nothing to disclose
Joao Santinha: Nothing to disclose
Nikolaos Papanikolaou: Nothing to disclose
Roberto Cannella: Other: Bracco, Bayer: support for attending meetings.
Valérie Vilgrain: Nothing to disclose

### RPS 201-13

**The value of Gd-EOB MRI for HCC subtype differentiation in a western population according to the 5th edition of the World Health Organization (WHO) classification**

T. A. Auer, *S. Halskov*, U. Fehrenbach, R. Mohr, B. Hamm, W. Schöning, D. Horst, J. Ihlow, D. Geisel; Berlin/DE

**Purpose:** To investigate the value of gadoxetic acid (Gd-EOB)-enhanced magnetic resonance imaging (MRI) for non-invasive subtype differentiation of hepatocellular carcinomas (HCCs) according to the 5th edition of the World Health Organization (WHO) classification of digestive system tumours.
**Methods or Backround:** The study included 262 surgically resected HCC lesions in 240 treatment-naïve patients with preoperative Gd-EOB-enhanced MRI. Specimens of all lesions were retrospectively classified into the subtypes defined by the WHO. Two readers blinded to clinical findings assessed Gd-EOB-enhanced MRI datasets using subjective and objective imaging features.
**Results or Findings:** High enhancement in the hepatobiliary phase (HBP) was present in the following subtypes: steatohepatitic (3/33), clear cell (3/13) and not otherwise specified (NOS) (17/174) (p=0.031). The following morphologic MRI parameters correlated with subtypes: macrovessel invasion (5/16 – p=0.033) and arterial phase hyperintensity (9/15 – p=0.018) with macrotrabecular-massive and intralesional steatosis (28/32 – p<0.001) with steatohepatitic subtypes. Furthermore, significant correlations were found for: age (p<0.001) and sex (p=0.023) as patients with the fibrolamellar subtype were younger (median 44 years) and female (4/5); alpha-fetoprotein (p<0.001) was elevated in the macrotrabecular-massive (median 397 µg/l), chromophobe (median 208 µg/l), and fibrolamellar subtypes (median 30 µg/l); diabetes mellitus type II was more common in the steatohepatitic subtype (20/33 - p=0.027).
**Conclusion:** While rare, strong HBP enhancement occurs in a subset of HCC subtypes. In addition, previously reported imaging features are reproducible with Gd-EOB-enhanced MRI, emphasising their validity.
**Limitations:** Basic MRI sequences were consistent but acquisition parameters differed in this retrospective analysis. Due to fixed delays after contrast agent injection for acquisition of postcontrast series, we may have missed optimal time windows for characterisation of some lesions. Readers were blinded but aware of the study design, which may have introduced detection bias.
**Ethics committee approval:** This study was approved by the institutional review board of Charité - Universitätsmedizin Berlin (internal registration number: EA1/323/20). The requirement for informed consent was waived due to the retrospective nature of the study. The study protocol conforms to the ethical guidelines of the 2002 Declaration of Helsinki.
**Funding for this study:** No funding was received for this study.
**Author Disclosures:**
Bernd Hamm: Nothing to disclose
Wenzel Schöning: Nothing to disclose
Uli Fehrenbach: Nothing to disclose
Dominik Geisel: Speaker: Bayer Schering Pharma, Berlin, Germany
David Horst: Nothing to disclose
Timo Alexander Auer: Research/Grant Support: Berlin Institute of Health
Raphael Mohr: Nothing to disclose
Sebastian Halskov: Nothing to disclose
Jana Ihlow: Nothing to disclose

**09:30-11:00 Research Stage 2**

### Research Presentation Session: Chest

### RPS 204

### Developments in chest CT

**Moderator**
T. Frauenfelder; Zurich/CH
*(thomas.frauenfelder@usz.ch)*

### RPS 204-2

**Comparison of ultra-low dose chest CT with SilverBeam and deep learning reconstruction vs. standard dose chest CT with hybrid reconstruction**

*C. Steveson*[1], J. Schuzer[2], S. Rollison[3], J. Moss[3], M. Y. Chen[3]; [1]Adelaide/AU, [2]Wheaton, IL/US, [3]Bethesda/US

**Purpose:** SilverBeam filter in conjunction with deep learning image reconstruction can reduce image noise and enable radiation dose reductions. The aim of this study is to investigate whether the use of SilverBeam and a deep learning reconstruction algorithm allows for reduced radiation dose chest CT scanning.
**Methods or Backround:** With institutional ethics approval, 60 consecutive patients with lymphangioleiomyomatosis (LAM) underwent chest CT at standard and ultra-low radiation doses on a 320-detector row CT scanner. The standard dose scan was performed with a standard filter and reconstructed using a soft tissue kernel with hybrid iterative reconstruction (AIDR3D) and the ultra-low dose scan was performed with a SilverBeam filter and reconstructed using a soft tissue kernel with deep learning reconstruction (AiCE) techniques. Cystic lung diseases can be quantified as a cyst score (% of lungs affected by cysts). Cyst scores were quantified by semi-automated software. Signal to noise ratio was calculated for each reconstruction. Data was analysed by linear correlation, paired t-test and Bland-Altman.

**Results or Findings:** Patients averaged 49.4 years (range 23-70yrs), 100% were female with mean BMI 25.1 ±6.0 kg/m2. Radiation dose was reduced by 85.3% (2.25 mSv ±1.4 mSv for standard filter vs. 0.33 mSv ±0.03 mSv for SilverBeam filter). The mean cyst score was 8.9±10.3% for standard filter with AIDR 3D reconstruction and 7.6±9.10.1% for SilverBeam filter with AiCE reconstruction, representing a difference of 1.3%. Linear correlation coefficient was excellent at 0.965 (p<0.001). Signal-to-noise for SilverBeam AiCE images was slightly lower than standard filter AIDR3D images (3.1 vs. 3.2, respectively, p=0.005) despite lower radiation doses.
**Conclusion:** SilverBeam filter and deep learning reconstruction in conjunction with 85.3% radiation reduction has a very good correlation with standard conventional chest CT.
**Limitations:** Single Centre Study.
**Ethics committee approval:** No information provided by the submitter.
**Funding for this study:** No funding was received for this study.
**Author Disclosures:**
Marcus Y Chen: Nothing to disclose
Chloe Steveson: Employee: Canon Medical Systems Corporation
John Schuzer: Employee: Canon Medical Research Unit
Joel Moss: Nothing to disclose
Shirley Rollison: Nothing to disclose

### RPS 204-3

**Economic evaluation of ultra-low dose chest CT versus chest X-ray for acute pulmonary disease**

*M. M. N. P. Kanglie*[1], I. A. H. Van Den Berk[1], T. van Engelen[1], W. de Monye[2], S. Bipat[1], P. M. M. Bossuyt[1], J. M. Prins[1], J. Stoker[1], M. G. Dijkgraaf[1]; [1]Amsterdam/NL, [2]Haarlem/NL

**Purpose:** To assess the cost-effectiveness of replacing chest X-ray (CXR) with ultra-low dose chest computed tomography (ULDCT) in patients with symptoms of non-traumatic pulmonary disease at the emergency department.
**Methods or Backround:** Between January 2017 and May 2018, participants were included in the OPTIMACT multicentre, randomised trial. They were followed for 4 weeks and completed patient questionnaires at day 28 on health status, use of health care resources, and health-related productivity loss. As reported elsewhere, short-term functional health was comparable between ULDCT and CXR, as were hospital admissions and length of hospital stay. For the current study primary outcomes were the costs per quality-adjusted life-year (QALY) gained and total 28-days healthcare costs and societal costs.
**Results or Findings:** Data of 2,393 OPTIMACT trial patients (1,199 ULDCT, 1,194 CXR) were included in the economic evaluation. Higher immediate imaging costs in the ULDCT group (+€61) did not result in higher mean hospital costs (€29). Overall 28-days health care costs were €4,655 (€4,018 to €5,375) in the ULDCT group and €5,176 (€4,306 to €6,133) in the CXR group, a difference of €521 (€1,665 to €638; p=0.38). Mean societal costs were €4,834 (€4,185 to €5,514) in the ULDCT group and €5,422 (€4,563 to €6,314) in the CXR group, the €588 difference was not significant (€1,795 to €587, p=0.32). ULDCT participants generated 0.051 (0.050 to 0.053) QALYs, CXR patients 0.050 (0.048 to 0.051), a non-significant gain of 0.0017 (0.0003 to 0.0039; p=0.10).
**Conclusion:** From a societal perspective ULDCT and CXR break about even in terms of costs and QALY.
**Limitations:** Reported data on resources other than hospital resources stem from a convenience sample of patients who responded to (re)invitations to complete patient questionnaires.
**Ethics committee approval:** (NL57923.018.16)
**Funding for this study:** Innovation grant of the Amsterdam UMC and ZonMW grant.
**Author Disclosures:**
Jan M. Prins: Nothing to disclose
Shandra Bipat: Nothing to disclose
Marcel G.W. Dijkgraaf: Nothing to disclose
Tjitske van Engelen: Nothing to disclose
Jaap Stoker: Nothing to disclose
Maadrika Meenakshi Nirvana Phoelmatie Kanglie: Nothing to disclose
Wouter de Monye: Nothing to disclose
Inge Alma Henrica Van Den Berk: Nothing to disclose
Patrick M. M. Bossuyt: Nothing to disclose

### RPS 204-4

**Chest CT angiography in daily practice with a photon-counting CT equipment: comparison of image quality with energy-integrating-detector (EID) CT in 142 patients**

L. Guiffault, I. Oufriche, A. Duhamel, J-B. Faivre, P. Felloni, J. Remy, *M. Rémy-Jardin*; Lille/FR
*(martine.remy@chru-lille.fr)*

**Purpose:** To compare CT angiographic (CTA) examinations obtained with photon-counting CT (PCCT) and dual-source CT (DSCT).
**Methods or Backround:** 71 consecutive patients scanned with PCCT (Naeotom Alpha; Siemens Healthineers) were compared to a paired population scanned with a 3rd-generation DSCT scanner (Somatom Force; Siemens Healthineers) using (a) for DSCT (group 1): collimation: 64x0.6x2 mm; pitch: 0.55; (b) for PCCT (group 2): collimation: 144 x 0.4mm; pitch: 1.5; single-source acquisition.
**Results or Findings:** Compared to group 1, group 2 examinations showed a: (a) a significantly lower DLP (172.6 ± 55.14 vs 339.4 ±75.64 mGy.cm; p<0.0001) and shorter duration of data acquisition (0.93 ± 0.1s vs 3.98 ± 0.35s; p<0.0001); (b) no significant difference in the attenuation values within central pulmonary arteries (group 1: 420.0 ± 131.3 HU; group 2: 398.0 ± 144.4 HU; p=0.32) and aorta (group 1: 394.7 ± 92.34 HU; group 2: 375.2 ± 90.34 HU; p=0.15) on 70 keV images; (c) a higher level of noise (group 2: 9.55 ± 1.86 HU; group 1: 6.88 ±1.56 HU; p<0.0001). On perfusion images (a) the mean level of attenuation did not differ (p=0.05) nor did the rating of subjective image noise (p=0.16); (b) the distribution of scores of fissure visualisation differed between the 2 groups (p<0.0001) with a higher proportion of fissures sharply delineated in group 2 (n=60; 84.5% versus n=26; 26.6%); (c) the rating of cardiac motion artifacts differed between the 2 groups (p<0.0001) with a higher percentage of mild artifacts in group 2 (n=69; 97.2%) than in group 1 (n=19; 26.8%).
**Conclusion:** PCCT acquisitions provided similar morphologic image quality and superior perfusion imaging at lower radiation dose.
**Limitations:** Single-source PCCT was the only option available at the time of this preliminary evaluation.
**Ethics committee approval:** Waiver of patient informed consent.
**Funding for this study:** No funding was received for this study.
**Author Disclosures:**
Lucas Guiffault: Nothing to disclose
Jean-Baptiste Faivre: Nothing to disclose
Paul Felloni: Nothing to disclose
Idir Oufriche: Nothing to disclose
Martine Rémy-Jardin: Research/Grant Support: Siemens Healthineers
Jacques Remy: Nothing to disclose
Alain Duhamel: Nothing to disclose

### RPS 204-5

**Evaluation of regional lung ventilation with xenon enhanced Dual Energy Computed Tomography (DECT)**

*L. J. Vandendorpe*, N. Buls; Jette/BE

**Purpose:** First, this study examined if a visual and quantitative regional evaluation of lung ventilation with a xenon enhanced DECT is feasible in rabbits. Second, the effects of broncho-constriction during wash-in (WI) and wash-out (WO) for all the rabbits and per rabbit separately were studied as well as the effects of broncho-constriction on a regional level. Finally, the study evaluated if the gathered results and insights could be useful for future clinical trials.
**Methods or Backround:** Six rabbits were anesthetised and placed under mechanical ventilation to undergo a dynamical xenon enhanced DECT. To study regional lung ventilation, xenon concentrations at ten different time points in six regions of interest (ROIs) were examined during the xenon WI and WO phases and compared in a normal state and in a broncho-constricted state. This resulted in a total of 88 comparisons.
**Results or Findings:** From the 88 comparisons, 45 showed significant changes in the xenon concentrations. Beyond significant differences, the percentual changes are visually observable and can be used when evaluating xenon DECT images. The effect of broncho-constriction during WI induces no change in the mean xenon concentration in the left lung, but shows a significant decrease in the right lung. When comparing height, broncho-constriction causes a decrease at every height during WI, only the decrease in the superior region is significant. Broncho-constriction causes a significant increase at every height during WO, the superior regions see a higher significant increase in the xenon concentrations compared to the inferior regions.
**Conclusion:** This study confirmed all the hypotheses and provided not only valuable information on regional lung ventilation with a xenon enhanced DECT, but also provided results and insights useful for future clinical trials.
**Limitations:** Occasional suboptimal gated ventilation of the rabbits.
**Ethics committee approval:** Ethics Commitee of the University Hospital Brussels (UZB)
**Funding for this study:** No funding was received for this study.
**Author Disclosures:**
Laurent Julien Vandendorpe: Nothing to disclose
Nico Buls: Nothing to disclose

### RPS 204-6

**Silver x-ray spectrum modulation filter and DLR: comparison of dose reduction capability for CT lung nodule detection with copper filter and other reconstruction methods at in vitro study**

Y. Oshima[1], *Y. Ohno*[1], Y. Ito[2], H. KImata[2], K. Fujii[2], H. Nagata[1], T. Ueda[1], Y. Ozawa[1], H. Toyama[1]; [1]Toyoake/JP, [2]Otawara/JP
*(yohno@fujita-hu.ac.jp)*

**Purpose:** To compare the capability of dose reduction for lung nodule detection among CTs obtained with silver x-ray beam spectral modulation and copper filters (Ag filter and Cu filter) and reconstructed with FBP, hybrid-type IR and deep learning reconstruction (DLR) at in vitro study.
**Methods or Backround:** A chest CT phantom including simulated ground-glass and part-solid nodules was scanned with a 320-detector row CT with Ag filter at 0.6, 1.6 and 2.4 mGys and Cu filters at 0.6, 1.6, 2.5 and 9.5mGys, and reconstructed with all methods. To compare image quality, SNR at each nodule was calculated. To compare nodule detection capability among all protocols, the probability of each nodule was assessed by 5-point scale. Then, Wilcoxon's signed rank test was performed for SNR comparison between Ag and Cu filters on each CT data. ROC analyses were performed to compare nodule detection capability between Ag and Cu filters on each CT data and between each CT data and CT obtained at 9.5mGy and reconstructed with FBP (i.e. standard protocol).
**Results or Findings:** SNR and area under the curve (AUC) of Ag filter were significantly higher than those of Cu filter on each CT data ($p<0.05$). AUC of standard protocol was significantly higher than that of CT data by Cu filter ($p<0.05$), when doses were set at ≤2.5mGy with FBP, ≤1.6mGy with hybrid-type IR and 0.6mGy with DLR. AUC of standard protocol was significantly higher that of CT data by Ag filter ($p<0.05$), when doses were set at ≤1.6mGy with FBP and hybrid-type IR and 0.6mGy with DLR.
**Conclusion:** Ag filter has better potential for dose reduction than Cu filter with improving image quality and detection capability on FBP.
**Limitations:** Phantom study.
**Ethics committee approval:** No information provided by the submitter.
**Funding for this study:** Research grant from Canon Medical Systems Corporation
**Author Disclosures:**
Yoshiyuki Ozawa: Nothing to disclose
Kenji Fujii: Employee: Canon Medical Systems Corporation
Hirona KImata: Employee: Canon Medical Systems Corporation
Hiroshi Toyama: Research/Grant Support: Canon Medical Systems Corporation
Yuya Ito: Employee: Canon Medical Systems Corporation
Yuka Oshima: Nothing to disclose
Takahiro Ueda: Nothing to disclose
Hiroyuki Nagata: Research/Grant Support: Canon Medical Systems Corporation
Yoshiharu Ohno: Research/Grant Support: Canon Medical Systems Corporation

### RPS 204-7

**Prospective evaluation of thoracic diseases using a compact flat-panel detector spiral computed tomographic scanner**

*D. S. Kim*[1], S. H. Yoon[2], S-J. Yoo[2], J. H. Hong[3]; [1]Busan/KR, [2]Seoul/KR, [3]Daegu/KR
*(dskim226@daum.net)*

**Purpose:** To prospectively evaluate the image quality and diagnostic performance of a compact flat-panel detector (FD) scanner for thoracic diseases compared to a clinical CT scanner.
**Methods or Backround:** From December 2020 to May 2021, 30 patients underwent two same-day low-dose chest CT scans using clinical state-of-art and compact FDCT scanners. Image quality was assessed visually and quantitatively. Two readers evaluated the diagnostic performance for nodules, parenchymal opacifications, bronchiectasis, linear opacities, and pleural abnormalities in 40 paired CT scans. The other 20 paired CT scans were used to examine the agreement of semi-quantitative CT scoring regarding bronchiectasis, bronchiolitis, nodules, airspace consolidations, and cavities.
**Results or Findings:** FDCT images had significantly lower visual image quality than clinical CT images (all $p<0.001$). The two CT image sets showed no significant differences in signal-to-noise and contrast-to-noise ratios (56.8 ± 12.5 vs. 57.3 ± 15.2; p=0.985 and 62.9 ± 11.7 vs. 60.7 ± 16.9; p=0.615). The pooled sensitivity was comparable for nodules, parenchymal opacifications, linear opacities, and pleural abnormalities (p=0.065 – 0.625), whereas the sensitivity was significantly lower in FDCT images than in clinical CT images for micronodules (p=0.007) and bronchiectasis (p=0.004). The specificity was mostly 1.0. Semi-quantitative CT scores were similar between the CT image sets ($p>0.05$), and intraclass correlation coefficients were around 0.950 or higher, except for bronchiectasis (0.869).
**Conclusion:** Compact FDCT images provided lower image quality but comparable diagnostic performance to clinical CT images for nodules, parenchymal opacifications, linear opacities, and pleural abnormalities.
**Limitations:** The number of patients was relatively small.
**Ethics committee approval:** This single-arm prospective trial was approved by Seoul National University Hospital institutional review board
**Funding for this study:** This work was supported by the Korea Medical Device Development Fund grant funded by the Korea government (the Ministry of Science and ICT, the Ministry of Trade Industry and Energy, the Ministry of Health & Welfare, Republic of Korea, the Ministry of Food and Drug Safety) (Project Number: 202011A03).
**Author Disclosures:**
Jung Hee Hong: Nothing to disclose
Seung-Jin Yoo: Nothing to disclose
Soon Ho Yoon: Shareholder: Medical IP
Da Som Kim: Nothing to disclose

### RPS 204-8

**Coronary artery calcium scoring on non-gated thoracic computed tomography: a systematic review and meta-analysis**

*M. Osborne-Grinter*[1], A. Ali[2], M. C. Williams[3]; [1]Bristol/UK, [2]Dundee/UK, [3]Edinburgh/UK

**Purpose:** Coronary artery calcification (CAC) is a marker of coronary artery disease and can be identified on thoracic CT performed for non-cardiac indications. In this systematic review and meta-analysis, we assessed reporting practices and clinical implications of incidental CAC on non-gated thoracic CT.
**Methods or Backround:** Online databases (e.g.PubMed and MEDLINE) were searched for research articles assessing the prevalence, associated demographic characteristics accuracy and prognosis of incidental CAC.
**Results or Findings:** Following a review of published articles, 108 studies were included in systematic review with meta-analysis performed in 96 studies assessing the prevalence of incidental CAC, 12 studies assessing the reporting practices for incidental CAC, 23 studies with consistent methodology on agreement between non-gated and electrocardiography gated CT, 33 studies on the characteristics on participants with present or absent incidental CAC and 41 studies assessing the prognosis of CAC. In 113,406 patients, the pooled prevalence of incidental CAC was 52%. On demographic analysis, patients with incidental CAC were older and more likely to be male with a history of diabetes, hypercholesterolaemia and hypertension. The agreement between non-gated and gated CT for continuous CAC scoring and for categorisation into four risk groups was very good. The presence of CAC associated with an increased likelihood of adverse cardiovascular events and all-cause mortality.
**Conclusion:** Incidental CAC is a common, but underreported, finding on non-gated CT. Non-gated CT is an accurate and reliable modality for identifying incidental CAC. The presence of incidental CAC is an adverse prognostic marker and should be reported regardless of age or clinical status.
**Limitations:** There was significant heterogeneity in the study due to a range of CT scanner types, radiation doses, slice thickness and calcium scoring methods.
**Ethics committee approval:** No information provided by the submitter.
**Funding for this study:** MCW (FS/ICRF/20/26002) is supported by the British Heart Foundation.
**Author Disclosures:**
Adnan Ali: Nothing to disclose
Maia Osborne-Grinter: Nothing to disclose
Michelle Claire Williams: Speaker: Canon Medical Systems Speaker: Siemens Healthineers

### RPS 204-9

**Impact of calcified plaques detected in preoperative chest CT on outcome after heart transplantation**

*C. S. Böttger*, H. Aubin, V. Hettlich, N. Kalampokas, D. Scheiber, G. Antoch, A. Lichtenberg, S. Reinartz, U. Boeken; Düsseldorf/DE
*(charlotte.boettger@med.uni-duesseldorf.de)*

**Purpose:** Heart transplantation (htx) is gold standard therapy in end stage heart failure. Calcified plaques in the ascending aorta or supra aortic branches may increase the risk of cerebrovascular events. Computed tomography (CT) of the chest may be performed prior to htx to detect calcified plaques and to plan potential cardiopulmonary bypass cannulation. The aim of this analysis therefore was to investigate the impact of arterial calcifications extracted from a routine chest computed tomography on outcome after heart transplantation.
**Methods or Backround:** 264 patients underwent htx in our department between 09/2010 and 10/2022. With regard to a preoperatively performed chest CT patients could retrospectively divided into 2 groups: without preoperative chest CT (group 1) and with preoperative chest CT (group 2). The recipients were comparable regarding the cardiac function, high urgent (HU) status on the waiting list and underlying disease. Further, donor criteria were comparable between the groups, i.e. age, gender, CMV-status and LV-EF.
**Results or Findings:** The majority in group 2 had preoperative VAD-support ($p<0.05$). Thirty-day-mortality was 11.5% in group 1 and 7.1% in group 2 ($p>0.05$). Stay on intensive care unit was prolonged in group 2, duration of mechanical ventilation was slightly prolonged in this group. No significant

Wednesday differences between the groups regarding incidence of rejection, postoperative renal failure or infections was evident. 1-year-survival was 77% in group 1 and 82.1% in group 2 (p>0.05).
**Conclusion:** Survival after htx was slightly better in patients with preoperative chest CT. Reasons could be better understanding of anatomic conditions and awareness of potential issues during aortic cannulation. An additional modification of CT technique for additional detection of soft plaques may even increase the impact of this screening.
**Limitations:** This study is a single centre study.
**Ethics committee approval:** No information provided by the submitter.
**Funding for this study:** No funding for this study.
**Author Disclosures:**
Charlotte Sonja Böttger: Nothing to disclose
Sebastian Reinartz: Nothing to disclose
Udo Boeken: Nothing to disclose
Vincent Hettlich: Nothing to disclose
Artur Lichtenberg: Nothing to disclose
Hug Aubin: Nothing to disclose
Gerald Antoch: Nothing to disclose
Nikolaos Kalampokas: Nothing to disclose
Daniel Scheiber: Nothing to disclose

### RPS 204-10

**Photon-counting detector CT in the evaluation of rheumatoid arthritis-related interstitial lung disease: a cross sectional study**

*N. Marton*, J. Gyebnar, K. Fritsch, J. Majnik, H. Balogh, T. Munkacsi, N. M. Kerkovits, J. Simon, P. Maurovich-Horvat; Budapest/HU
*(marton.nikol@gmail.com)*

**Purpose:** Interstitial lung disease (ILD) significantly accounts for the morbidity and mortality in rheumatoid arthritis (RA). The identification of pulmonary parenchymal involvement requires high spatial resolution CT scans. The aim of this cross-sectional study is to evaluate the performance of novel photon-counting detector CT (PCD-CT) protocols in the detection of RA-ILD.
**Methods or Backround:** 59 RA patients (age median [IQR] 69.9 [74.3; 63] years; 71% female) without antecedent diagnosis of ILD involvement underwent consecutive 0.4 mm slice-thickness high-resolution (HR) and 0.2 mm slice thickness ultra-high-resolution (UHR) PCD-CT examinations. Interstitial involvement (ground glass opacity [GGO], bronchiectasis, reticulation, and honeycombing) was scored in each lobe using a Likert-type scale. The total ILD score was calculated by the sum of the scores from all the lobes.
**Results or Findings:** The HR protocol had significantly lower (p<0.001) total dose length product (median [IQR] 29.01 [21.05; 35.09] mGy*cm) compared to the UHR scans (335.80 [223.00; 498.00] mGy*cm). Slightly lower total ILD scores (4 [2;7.5] vs 6 [4;9], p<0.05) were registered based on the HR scans, because elevated reticulation (0[0; 2.25] vs 2 [0; 3], p<0.05), bronchiectasis (0 [0; 2] vs 2 [0; 3] p<0.05) and honeycombing (0 [0; 0] vs 0 [0; 0], p<0.05) scores were registered based on the UHR protocol. GGO scores did not differ significantly (2.5 [2; 4] vs 2 [2; 5], p=ns).
**Conclusion:** UHR PCD-CT protocol provides more detailed information regarding the extent of interstitial involvement in patients with RA compared to HR PCD-CT measurement. A reduced dose HR PCD-CT scan may serve as preselection study for UHR PCD-CT protocol.
**Limitations:** The lack of comparison to energy-integrating-detector CT scans.
**Ethics committee approval:** This trial was registered under clinicaltrials.gov website (IV-2683-1/2022/EKU) and approved by the local ethical review board (National Scientific and Research Ethics Committee, Hungary). This work was carried out in accordance with the Helsinki Declaration (JAMA 2000; 284:3043–3049).
**Funding for this study:** Nikolett Marton MD, PhD receives Janos Bolyai Research Scholarship of the Hungarian Academy of Sciences. No other fundings.
**Author Disclosures:**
Judit Majnik: Nothing to disclose
Tamas Munkacsi: Nothing to disclose
Pál Maurovich-Horvat: Nothing to disclose
János Gyebnar: Nothing to disclose
Nora Melinda Kerkovits: Nothing to disclose
Judit Simon: Nothing to disclose
Hanna Balogh: Nothing to disclose
Kinga Fritsch: Nothing to disclose
Nikolett Marton: Nothing to disclose

### RPS 204-11

**Ultra-low-dose spectral-detector CT for the accurate quantification of pulmonary nodules: an anthropomorphic chest phantom study**

*X. Zhou*[1], H. Zhang[1], W. Huang[2], L. Fan[1]; [1]Shanghai/CN, [2]Weifang/CN

**Purpose:** To investigate the accuracy of the diameter and density of pulmonary nodules in an anthropomorphic chest phantom using a dual-layer detector CT with an ultra-low-dose scan protocol.
**Methods or Backround:** An anthropomorphic chest phantom contained 12 pulmonary nodules with 4 sizes (diameters of 5, 8, 10, and 12 mm) and 3 densities (CT values of - 800HU, - 630HU, and 100HU) was scanned on a dual-layer detector CT 3 times with 100 kVp/10 mAs, 100 kVp/20 mAs, 120 kVp/10 mAs, and 120 kVp/30 mAs, respectively. Each CT scan was reconstructed using 10 algorithms, such as FBP, iDose4, IMR, and VMIs of 40/50/60/70/80/90/100 keV. The inter-observer agreement was assessed, and the image quality and absolute percentage measurement errors (APEs) of the diameter and density were calculated and compared.
**Results or Findings:** With each fixed reconstruction algorithm, the four low-dose scanning schemes showed no significant differences in the measurement of APEs for the diameter and density of 12 lung nodules (P > 0.05). There were differences between different reconstruction algorithms under 120 kVp/30 mAs (p＞0.05). Under the protocol of 100 kVp/10 mAs, the APEs of density with IMR were the least (APE-mean=6.69), but no significant difference was found between 50 keV (APE-mean=11.69) and IMR (P=0.666). In the subgroup analysis, under 100 kVp/10 mAs, there were no significant differences between the 50 keV and IMR in the diameter and density of solid nodules (SNs) and ground-glass nodules (GGNs) (P > 0.05); moreover, the radiation dose was reduced by 78.1% in comparison with using 120kVp/30mAs.
**Conclusion:** The protocol of using 100kVp/10 mAs with 50keV exhibited greater image quality and more accurate quantification of SN and GGN, with a reduction of the radiation dose by 78.1%.
**Limitations:** Not applicable.
**Ethics committee approval:** No information provided by the submitter.
**Funding for this study:** Not applicable.
**Author Disclosures:**
Li Fan: Nothing to disclose
Hanxiao Zhang: Nothing to disclose
Xiuxiu Zhou: Nothing to disclose
Wenjun Huang: Nothing to disclose

**09:30-11:00** **Research Stage 3**

### Research Presentation Session: Neuro

### RPS 211

### CNS infection and inflammation

**Moderator**
J. Boban; Novi Sad/RS
*(jasmina.konstantinovic@gmail.com)*

### RPS 211-2

**Olfactory bulb MRI in mild to moderate COVID-19**

*M. Castor*, L. Haider, E. Tommasino, M. M. Thurnher; Vienna/AT

**Purpose:** While CNS involvement is indicated in individuals with severe COVID-19, evidence for CNS manifestations in cases with mild to moderate COVID-19 is still limited.
**Methods or Backround:** Brain-MRI was prospectively acquired in 112 individuals following mild-to-moderate COVID-19 on a Siemens Vida, 3T, using a 64-channel head coil from 11/2019 – 06/2021 and 50 controls. Olfactory bulb volume was manually segmented independently by two raters with itk-snap on 3D FLAIR images (voxel size: 0.75mm3) guided by expert placed anatomical landmarks, blinded to clinical data. Inter-and intra-rater agreement was quantified with inter-class correlation coefficients (two-way, mixed-effects, consistency), group comparisons are based on linear regression models adjusting for age and gender.
**Results or Findings:** Age in COVID-19: mean 45 years (sd: 15), controls: mean 43 (sd: 18) and gender, COVID-19: 63% female, controls: 65%, did not differ between the two groups (p= 0.336; p= 0.802). Individuals were imaged on mean 198 days (sd: 126) after the first PCR confirmed COVID-19 infection. Of the 30% hospitalized in the course of their infection, non-required intensive care unit admission or mechanical ventilation. Inter- and intra-rater agreement for olfactory bulb volume was high 0.964, 95% (CI: 0.949 - 0.975, p< 0.001). Olfactory bulb volume was significantly lower after COVID-19 (mean: 252mm3, sd: 96) compared to controls (mean: 295mm3, sd: 90), p= 0.028. We did not observe differences in the olfactory bulb volume between hospitalized and not-hospitalized COVID-19 subjects (p= 0.587), female vs male subjects (p= 0.978) and observed a weak negative associated with age (Pearson correlation coefficient: -0.22, p= 0.037).
**Conclusion:** Olfactory bulb volume is significantly decreased after mild-to-moderate COVID-19 suggesting potential CNS involvement.
**Limitations:** This study is limited by the lack of MRIs prior to COVID-19 and low sample size.
**Ethics committee approval:** This study was approved by 1693/2020 Medical university vienna
**Funding for this study:** No funding was received for this study.

**Author Disclosures:**
Emanuele Tommasino: Nothing to disclose
Moritz Castor: Nothing to disclose
Majda M. Thurnher: Nothing to disclose
Lukas Haider: Nothing to disclose

### RPS 211-3

**Signal intensity in cranial nerves in COVID-19**

*S. Gómez Peña*, J. A. Matias-Guiu, L. Gil, C. Delgado-Alonso, M. Díez-Cirarda, M. C. Polidura Arruga, N. Gomez Ruiz, M. Jorquera Moya, M. Yus Fuertes; Madrid/ES
*(sagompe@gmail.com)*

**Purpose:** Some reports have found signal hyperintensity in several cranial nerves during acute COVID-19. It has been suggested that this could be caused by nerve inflammation or viral invasion in the central nervous system. In addition, the persistence of neuroinflammation or viral reservoirs in the central nervous system could be associated with the persistence of symptoms in post-COVID syndrome. To our knowledge, there are no studies evaluating cranial nerve intensity in the post-acute stage of COVID-19. In this study, we aimed to evaluate the signal intensity in a large cohort of patients reporting cognitive complaints after COVID-19 in the context of post-COVID syndrome.
**Methods or Backround:** We enrolled 145 post-COVID patients and 36 control patients 14 +/- 6.9 months after the onset of acute infection. Patients underwent 3.0T MRI, including a 3D-FLAIR sequence. The signal intensities of three cranial pairs bilaterally (I, III and V), as well as the signal intensity in corpus callosum and pons, were measured in reformatted coronal images of 3D FLAIR sequences with the intention of normalising them with the latter as a ratio. Images were evaluated blinded to the clinical information.
**Results or Findings:** We found no statistically significant differences in the signal intensity of cranial nerved between post-COVID patients and controls. There was no association between intensity and time since the clinical onset to MRI. There were no differences between that required hospitalization, ventilatory assistance, intensive care unit admission or not. In addition, no associations were found with olfactory function or olfactory symptoms.
**Conclusion:** Our study suggests that cranial nerve intensity is not modified in the post-acute stages of COVID-19 and post-COVID syndrome. These findings, although negative, contribute to the knowledge of the pathophysiology of post-acute complications of COVID-19.
**Limitations:** The study included a cross-sectional design.
**Ethics committee approval:** No information provided by the submitter.
**Funding for this study:** No funding was received for this study.
**Author Disclosures:**
María Díez-Cirarda: Nothing to disclose
Sara Gómez Peña: Nothing to disclose
Lidia Gil: Nothing to disclose
Manuela Jorquera Moya: Nothing to disclose
Miguel Yus Fuertes: Nothing to disclose
Cristina Delgado-Alonso: Nothing to disclose
Mª Carmen Polidura Arruga: Nothing to disclose
Natividad Gomez Ruiz: Nothing to disclose
Jordi A. Matias-Guiu: Nothing to disclose

### RPS 211-4

**Quantitative T1 mapping reveals changes after COVID-19 in specific brain regions: a prospective observational case-control study**

M. Fahim, E. Hattingen, A. Jurcoane, J-R. Schüre, R. Deichmann, *C. Arendt*; Frankfurt am Main/DE

**Purpose:** Infections with SARS-CoV-2 are associated with a broad range of structural cerebral changes. Prolongation of proton relaxation times is indicative of microstructural alterations of brain tissue. The purpose was to evaluate quantitative T1 mapping in COVID-19-recovered patients.
**Methods or Backround:** Unselected patients with a previous SARS-CoV-2-positive test (≥3 months) and age- and sex-matched SARS-CoV-2-antibody-negative controls were included in this prospective 3T MRI study (September 2020-December 2021). Quantitative T1 maps were generated using the variable flip angle method. Structural segmentation was done on synthetic MP-RAGE images with custom-made programs for the following regions: both-sided grey and white matter, hippocampus, amygdala, putamen, pallidum, accumbens and caudate nucleus. Van Elteren test stratified by age and Bonferroni-corrected was used to analyze T1 differences between groups.
**Results or Findings:** 145 subjects (median age, 46 years; 73 women) were included: 69 formerly seropositive (43 treated at home, 26 in hospital) and 76 seronegative control subjects. Median duration between COVID-19 diagnosis and MRI was 157 days. Previously hospitalized patients aged 40 years and older (n=23/69; 13 men) had significantly higher T1 values than controls (n=47/76; 21 men) in the following locations only: right white matter (+14.0 ms; p=0.039), right hippocampus (+34.3 ms; p=0.039), left caudate (+44.0 ms; p=0.017) and accumbens (+69.2 ms; p=0.034) nucleus. None of these values correlated significantly with visual findings, pre-existing conditions, time from COVID-19 diagnosis, duration of inpatient treatment and results from dedicated neurological tests.
**Conclusion:** Quantitative T1 reveals subtle tissue changes in specific brain regions in previously hospitalized COVID-19 patients above the age of 40 years.
**Limitations:** This is a single-centre study; specific T1 sequence, heterogeneous virus variants, and (more severe) disease courses in pre-Omicron-era
**Ethics committee approval:** No information provided by the submitter.
**Funding for this study:** This study was approved by Deutsches Zentrum für Infektionsforschung; Goethe Corona Fonds
**Author Disclosures:**
Jan-Rüdiger Schüre: Nothing to disclose
Masia Fahim: Nothing to disclose
Elke Hattingen: Nothing to disclose
Christophe Arendt: Nothing to disclose
Alina Jurcoane: Nothing to disclose
Ralf Deichmann: Nothing to disclose

### RPS 211-5

**FDG PET CT in the neurological complications of COVID-19**

*N. Seniaray*, R. Verma, R. Ranjan, E. Belho, H. Mahajan; New Delhi/IN

**Purpose:** Severe acute respiratory syndrome coronavirus 2 (SARS- CoV-2) is now universally accepted as a multisystem disease, including the central nervous system (CNS) and the peripheral nervous system (PNS). Although MRI is the primary investigative tool for CNS, Fluorodeoxyglucose (FDG)_ Positron Emission Tomography Computed Tomography (PET-CT) may show additional areas of involvement in the brain in the form of regional hypometabolism or hypermetabolism, secondary to synaptic dysfunction, electrical or glial activation. This study elucidated the patterns of 18F-FDG PET/CT uptake in neurological complications of coronavirus 2 (SARS- CoV-2).
**Methods or Backround:** A retrospective analysis of 30 COVID-19 patients with CNS symptoms was done. All the subjects were clinically and radiologically (MRI) analyzed and were further segregated into MRI positive (n=13) and negative (n=17). All the subjects underwent 18F-FDG PET/CT scan on a separate day. The FDG uptake patterns were recorded, and areas of hypometabolism that were two standard deviations from the mean were considered abnormal.
**Results or Findings:** Out of 13 MRI-positive subjects, 8 had findings suggestive of ischemic stroke with diffusion restriction and characteristic angiographic abnormalities; three subjects had concurrent Rhino-cerebral Mucor mycosis with associated changes on MRI and FDG PET scan. 2 Subjects had space occupying T2 hyperintense lesions on MRI, diagnosed as focal demyelination which was mildly FDG and F-DOPA avid. 14 subjects with non-contributory MRI had patterns of pre-frontal-limbic-insular hypermetabolism with clusters of hypermetabolism in the prefrontal, dorsolateral frontal, insular and cingulate cortices, and 7 subjects had posterior cortical-subcortical and cerebellar hypermetabolism on FDG PET images.
**Conclusion:** FDG PET/CT may help in certain acute and sub-acute neurological manifestations of COVID-19, especially with non-contributory MRI, thereby expediting the subsequent clinical management.
**Limitations:** No
**Ethics committee approval:** No information provided by the submitter.
**Funding for this study:** No funding was received for this study.
**Author Disclosures:**
Nikhil Seniaray: Nothing to disclose
Ritu Verma: Nothing to disclose
Harsh Mahajan: Nothing to disclose
Rajiv Ranjan: Nothing to disclose
Ethel Belho: Nothing to disclose

### RPS 211-6

**Pre-and post-mortem MRI evaluation in COVID-19**

*S. Gerevini*[1], A. Napolitano[1], M. Cava[2], A. Caroli[3], A. Arrigoni[3], A. Gianatti[1], C. Parisi[4], S. Roosendaal[5], E. Aronica[5]; [1]Bergamo/IT, [2]Bosco Marengo/IT, [3]Ranica/IT, [4]Palermo/IT, [5]Amsterdam/NL
*(gerevini.simonetta@gmail.com)*

**Purpose:** The study aimed to compare neuropathological features of cerebral microbleeds (CMB) in pre-and post-mortem MRI by targeting specimens.
**Methods or Backround:** CMB extent has been evaluated via an in-house algorithm exploiting the susceptibility-weighted image (SWI) with the pertinent filtered phase sequence and minimum intensity projection. The technique combines different state-of-the-art solutions for CMB discrimination and segmentation. The final mask was refined manually before undergoing quantification. A simplified pipeline exploiting just the SWI scan and not involving markers detection was applied to the post-mortem images. To compare the modalities and the microbleeds segmentations performed, we localized the ex-vivo imaging of the specimens within the in-vivo brain volume and finally co-registered the brain portions by scaling, rotation and translation with mutual information as the metric.

**Results or Findings:** In a preliminary qualitative analysis, we reached a good co-registration accuracy. The comparison shows a visual correspondence between the microbleeds segmentations and suggests reliability of the analysis in SWI in-vivo scans.
**Conclusion:** In-vivo analysis of cerebral microbleeds using SWI imaging is suggested to be consistent with the ex-vivo findings, net of the technical and study limitations.
**Limitations:** Limitations of this explorative study include the retrospective nature and blind collection of the specimens, not planned on prior knowledge, and the different spatial resolution in the pre-and post-mortem images, which penalizes the comparison.
**Ethics committee approval:** No information provided by the submitter.
**Funding for this study:** No funding was received for this study.
**Author Disclosures:**
Stefan Roosendaal: Nothing to disclose
Alberto Arrigoni: Nothing to disclose
Andrea Gianatti: Nothing to disclose
Mariangela Cava: Nothing to disclose
Carmelo Parisi: Nothing to disclose
Anna Caroli: Nothing to disclose
Angela Napolitano: Nothing to disclose
Eleonora Aronica: Nothing to disclose
Simonetta Gerevini: Nothing to disclose

### RPS 211-7

### Yield of high-resolution vessel wall magnetic resonance imaging in patients with tuberculous meningitis: a prospective study from a tertiary care hospital

*S. Sonker*, S. Tiwari, P. S. Khera, P. K. Garg, T. Yadav, S. Panda, G. K. Bohra, B. Choudhary, V. Jain; Jodhpur/IN

**Purpose:** Tuberculous meningitis can cause inflammation in the cerebral arterial system, leading to arterial spasms and stenosis, causing ischemic brain injury. HR-VWI is a recently introduced technique in evaluating the severity of arterial involvement. Our study aims to evaluate vessel wall enhancement in intracranial arteries in tuberculous meningitis and correlate the vascular involvement with disease severity and the presence of infarction.
**Methods or Backround:** Prospective single-group observational study
Study duration: 15 months (July 2021 - September 2021) Study population: All patients aged between 5 years and 60 years diagnosed as possible, probable, and definite cases of tuberculous meningitis as per uniform case definition criteria, as proposed by Marais et al. Patients with HIV infection were excluded. Scan protocol: T2-axial, 3D-FLAIR, DWI, SWI, 3D–TOF MRA, Pre-contrast and post-contrast T1 CUBE/SPACE using 3-T Scanner MRI. Two radiologists (with 8 years and 1.5 years of experience) interpreted the patients' MRI images.
**Results or Findings:** Out of the 45 cases of tuberculous meningitis, infarcts were seen in 23 cases (51.1%), and vessel wall enhancement was seen in 27 cases (60.0%). Out of the 23 cases with infarcts, vessel wall enhancement was seen in 17 cases (73.9%). There was a significant correlation between vessel wall enhancement and the presence of infarcts. Out of the 23 cases with infarction, infarcts in anterior circulation were seen in 21 (91.3%) cases, posterior circulation in 10 (43.4%) cases and both anterior and posterior circulation in 8 (34.7%) cases. Cortical infarcts were seen in 10 (43.4%) cases, and cerebral perforator territory infarcts were seen in 21 (91.3%) cases.
**Conclusion:** HR-VWI provides detailed information on arterial involvement in tuberculous meningitis, which has prognostic and management implication.
**Limitations:** This is a single-centre study, and there are possibilities of spurious vessel wall enhancement contributed by the presence of vasa vasorum.
**Ethics committee approval:** No information provided by the submitter.
**Funding for this study:** No funding was received for this study.
**Author Disclosures:**
Sarbesh Tiwari: Nothing to disclose
Gopal Krishana Bohra: Nothing to disclose
Pawan Kumar Garg: Nothing to disclose
Pushpinder Singh Khera: Nothing to disclose
Bharat Choudhary: Nothing to disclose
Samhita Panda: Nothing to disclose
Vidhi Jain: Nothing to disclose
Taruna Yadav: Nothing to disclose
Shailly Sonker: Nothing to disclose

### RPS 211-8

### Diagnostic value of gadolinium-enhanced SWI sequences in multiple sclerosis

L. Pertici, *C. Fagotti*, S. Mancini, F. Bruno, A. Catalucci, M. Bellini, G. Caldarelli, C. Masciocchi, A. Splendiani; L'Aquila/IT
*(cristinafagotti1991@gmail.com)*

**Purpose:** MR imaging using SWI sequences after gadolinium administration has proved to have a high diagnostic yield in the evaluation of several neurologic disorders. Moreover, several literature studies highlighted the value of SWI sequences in the evaluation of several specific signs in multiple sclerosis imaging, including the central vein sign and slow-expanding lesions. The purpose of our study was to evaluate the diagnostic performance of gadolinium-enhanced SWI for the detection of MS plaques.
**Methods or Backround:** MR imaging studies of 52 MS patients (36 females, mean age 41.6 years, range: 28-64) were performed using a 3T scanner. Two neuroradiologists scrutinised the number and signal abnormalities of the demyelinating plaques on SWI sequences before and after gadolinium and compared them with pre-contrast FLAIR sequences and gadolinium-enhanced T1 images. Inter-and intraobserver agreements were evaluated, together with SNR and diagnostic confidence of the readers.
**Results or Findings:** Gadolinium-enhanced T1 sequences demonstrated the highest number of active demyelinating plaques. Gadolinium SWI increased the interobserver agreement on the lesion count of active plaques (κ coefficient = 0.86) without increasing, however, the overall detection rate of demyelinating plaques. Signal abnormalities in pre-contrast SWI sequences were detected by both readers (κ coefficient = 0.82) for the identification of “chronically active” plaques. No significant differences in readers' diagnostic confidence between gadolinium-enhances SWI and T1 were detected.
**Conclusion:** Gadolinium SWI is able to detect BBB dysfunction in active MS plaques without a significantly superior diagnostic performance than gadolinium T1 spin-echo. Gadolinium administration did not increase the diagnostic performance of SWI sequences in inactive or chronically-active lesions.
**Limitations:** Not applicable.
**Ethics committee approval:** The study was approved by the Institutional review board (IRB)
**Funding for this study:** No funding was received for this study.
**Author Disclosures:**
Giulia Caldarelli: Nothing to disclose
Alessandra Splendiani: Nothing to disclose
Carlo Masciocchi: Nothing to disclose
Sara Mancini: Nothing to disclose
Leonardo Pertici: Nothing to disclose
Cristina Fagotti: Nothing to disclose
Mattia Bellini: Nothing to disclose
Alessia Catalucci: Nothing to disclose
Federico Bruno: Nothing to disclose

### RPS 211-9

### Estimation of white matter hyperintensities with synthetic MRI myelin volume fraction in patients with multiple sclerosis and non-MS white matter hyperintensities

*N. Syed Nasser*, S. Rajan, V. K. Venugopal, V. Mahajan, H. Mahajan; Delhi/IN
*(nisha.nasser@caring-research.com)*

**Purpose:** The study aimed to evaluate the synthetic MRI (SyMRI) generated Myelin (MyCY) to White matter (WM) ratio performing normative brain volumetry to investigate MyCY loss in patients with Multiple Sclerosis (MS) in a clinical setting.
**Methods or Backround:** Fifteen patients with WMHs and 15 non-MS patients were included retrospectively. Synthetic MRI images were acquired using MAGiC, a customized version of SyntheticMR's SyMRI software on a 3T MRI scanner (Discovery MR750w; GE Healthcare; Milwaukee, USA). A fast multi-delay multi-echo acquisition (MDME) was performed with a 2D axial pulse sequence with different combinations of TEs and saturation delay times in 6 minutes. SyMRI image analysis was done using SyMRI software (SyMRI Prototype 21Q3 SP2; Synthetic MR, Linköping, Sweden). Synthetic MR imaging data were used to generate the MyCY partial maps and WM, GM and CSF volume fractions. MyCY-to-WM ratio was calculated to quantify the signal intensities of the normal-appearing white-matter (NAWM) in both groups, and their mean values were recorded. All the subjects also underwent conventional diffusion-weighted imaging, T1w and T2w imaging.
**Results or Findings:** Differences in the means of Myelin volume fraction (MVF) and MyCY-to-WM fraction were assessed using the Mann-Whitney U test. In subjects with WMHs, the MyCY-to-WM fraction was lower than in the control (3.20±0.16 vs 3.242±0.16, p=0.548). But, interestingly, we noted a significant difference in the mean Myelin volume between the WMH and the control group (158.66±32.31 vs 138.29± 29.28, p=0.044) and also the WMF was significantly lower in the WMHs group than in the control group (38.8% vs 33.2%, p<0.001).
**Conclusion:** The presence of WMHs can result in myelin loss that can be quantitatively evaluated using synthetic MRI.
**Limitations:** The study included a small sample size.
**Ethics committee approval:** This study was approved by IRB.
**Funding for this study:** No funding was received for this study.
**Author Disclosures:**
Nisha Syed Nasser: Employee: CARPL.ai
Sriram Rajan: Employee: Mahajan Imaging
Harsh Mahajan: Other: Research collaboration with General Electric, Oxipit.ai, Lunit.ai, Predible Health. Other: Director at Mahajan Imaging. Other: Research collaboration with Koninklijke Philips NV Research, Quibim, Subtle Medical, Qure.ai.
Vasantha Kumar Venugopal: Other: Research collaboration with Oxipit.ai, Lunit.ai, Subtle Medical. Employee: CARPL.ai Other: Research collaboration with Qure.ai, General Electric, Koninklijke Philips NV Research, Quibim, Predible Health.
Vidur Mahajan: Other: Research collaboration with Lunit.ai, Qure.ai. CEO: CARPL.ai Other: Research collaboration with Oxipit.ai, Subtle Medical, Quibim, General Electric, Predible Health, Koninklijke Philips NV Research

### RPS 211-10

**MR-Planimetry in multiple sclerosis: potential biomarker predicting clinical disease severity**

*S. Mangesius*[1], L. Lenhart[1], L. Haider[2], G. Bsteh[2], S. Wurth[1], F. Deisenhammer[1], E. R. Gizewski[1]; [1]Innsbruck/AT, [2]Vienna/AT
*(stephanie.mangesius@tirol-kliniken.at)*

**Purpose:** Brain volume change has been suggested as an MRI predictor of disability in multiple sclerosis (MS). However, volumetric measurements are not used routinely. Easily applicable planimetric measurements are proposed for different neurologic disease entities. In this study, the use of the more widely available and more easily applicable MR planimetry was examined as a potential alternative biomarker for predicting clinical disease severity scores and clinical progression of MS.
**Methods or Backround:** In this retrospective study, Spearman correlation coefficients between MR planimetric measurements and Expanded Disability Status Scale (EDSS) were calculated. Furthermore, to compare both methods of atrophy assessment, MR volumetric measurements were performed, and their correlations with EDSS were also investigated. Subgroup analyses for sex and MS phenotypes were further assessed.
**Results or Findings:** 279 MR examinations of 98 patients were analysed. Significant correlations between MR planimetry and EDSS were present. Corpus Callosum (CC) related MR planimetric measurements showed the strongest correlation with EDSS, e.g. anterior segment of CC ($r = -0.23$, $p = <0.001$). Overall, the strongest correlation with EDSS was found for cerebral spinal fluid MR volumetry ($r = 0.35$, $p = < 0.001$). Sex-specific differences were also present. In the secondary progressive MS (SPMS) phenotype, male patients showed significant correlations with EDSS, e.g. grey matter MR volumetry ($r = -0.33$, $p = 0.015$), but women did not ($r = -0.12$, $p = 0.186$).
**Conclusion:** MR planimetric measurements show moderate to good correlations with the EDSS. MR planimetry can hence be a valuable biomarker in the assessment of MS patients.
**Limitations:** Due to the retrospective design, follow-up MRI and clinical evaluation were performed as needed and not at fixed study intervals, therefore reflecting a real-life setting.
**Ethics committee approval:** The study was approved by the Ethics Committee of the Medical University of Innsbruck, Austria.
**Funding for this study:** No funding was received for this study.
**Author Disclosures:**
Lukas Lenhart: Nothing to disclose
Lukas Haider: Nothing to disclose
Gabriel Bsteh: Nothing to disclose
Sebastian Wurth: Nothing to disclose
Elke Ruth Gizewski: Nothing to disclose
Florian Deisenhammer: Nothing to disclose
Stephanie Mangesius: Nothing to disclose

### RPS 211-11

**Clinical validity of quantitative AI software-assisted monitoring in multiple sclerosis: comparison with neuroradiological visual assessment and clinical correlates**

*F. Bruno*, L. Pertici, S. Mancini, C. Fagotti, G. Caldarelli, F. Colarieti, A. Catalucci, A. Splendiani, C. Masciocchi; L'Aquila/IT
*(federico.bruno.1988@gmail.com)*

**Purpose:** MRI is a fundamental tool for diagnosis and disease monitoring in multiple sclerosis (MS). Quantitative evaluation using AI is gaining increased research interest; however, there are few studies assessing the real-world clinical validity and application of such a solution in the diagnosis and monitoring of lesion load in MS patients. The purpose of the present study is to compare the automated analysis using a dedicated software and the visual neuro-radiological evaluation of expert and inexperienced readers.
**Methods or Backround:** Brain MRI examinations of 56 MS patients (46 relapsing-remitting, 38 females, mean age 42.3 years) were retrieved from medical records in a single institution, selection follow-up MRI examinations at baseline and up to 1 year after diagnosis. Volumetric T1 and FLAIR sequences for all patients were analyzed using a dedicated AI-based software. Data regarding lesion count, localization, volume, and brain volumetric analysis were recorded and validated by two neuroradiologists. The same readers performed the same measurements with manual segmentation using the PACS tools and visual qualitative/semiquantitative assessment. Segmentation time and workload (using the NASA task-load index) were recorded and compared.
**Results or Findings:** Lesion count assessments by the software and by the two readers were significantly correlated with high inter- and intrareader agreement (k=0.92). Lower interobserver agreement (software-reader) was found for lesion volume calculation (k=0.73). Brain volumetric assessment was quantitatively feasible only using the software analysis. We found a significant positive correlation between lesion load measured by the software and clinical scores evaluated during the follow-up.
**Conclusion:** Software analysis showed high agreement with the expert radiologist reading. The artificial intelligence support for reporting allows to increase the consistency of the data in the report and the agreement between readers, with time-effective analysis.
**Limitations:** The study included a relatively limited population.
**Ethics committee approval:** This study was approved by the local IRB
**Funding for this study:** None
**Author Disclosures:**
Giulia Caldarelli: Nothing to disclose
Alessandra Splendiani: Nothing to disclose
Filippo Colarieti: Nothing to disclose
Carlo Masciocchi: Nothing to disclose
Sara Mancini: Nothing to disclose
Leonardo Pertici: Nothing to disclose
Cristina Fagotti: Nothing to disclose
Alessia Catalucci: Nothing to disclose
Federico Bruno: Nothing to disclose

### RPS 211-12

**Assessment of the agreement for detection of optic nerve lesions on 3D double inversion recovery sequence magnetic resonance imaging**

*H. Kalamou*; Lille/FR

**Purpose:** The study aimed to assess the agreement for the detection of optic nerve lesions on 3D-double inversion recovery sequence magnetic resonance imaging.
**Methods or Backround:** We conducted a retrospective study including all patients who underwent a brain/optic nerve magnetic resonance imaging (MRI), including a 3-dimensional (3D) double inversion recovery (DIR) sequence between March 18th 2013 and September 29th 2020. The study group included 786 patients suffering from: demyelinating diseases of the central nervous system (such as CIS, MS, NMOSD, and MOGAD), radiologically isolated syndrome (RIS), other neurological disorders, systemic inflammatory diseases, patients with non-inflammatory neurological disease and visual complaints finally due to psychogenic troubles. We assessed the optic nerve lesions on acquisitions that only included the anterior visual pathways. The primary outcome was the detection of optic nerve DIR hypersignal lesion. Secondary outcomes were the number of optic nerve lesion(s), the optic nerve lesion length and location.
**Results or Findings:** Intra-and inter-observer agreements for detection, counting the number of hypersignals, and the measure of hypersignal length on 3D DIR sequence were excellent with concordance values (ICC, CCC and Krippendorff's alpha) greater than 0.75.
**Conclusion:** The 3D DIR MRI is a promising sequence for the detection of optic nerve inflammatory lesions. With this larger study, we confirmed some previous results, excellent intra- and inter-observer agreements allowing for international multicenter use.
**Limitations:** Our study has some limitations; first, we didn't assess each optic nerve separately, and it could lead to bias in the detection of lesions on the second nerve analyzed. Second, we assessed the 3D double inversion-recovery MRI sequence of only one constructor.
**Ethics committee approval:** No information provided by the submitter.
**Funding for this study:** No funding was received for this study.
**Author Disclosures:**
Hawa Kalamou: Nothing to disclose

**09:30-11:00** **Research Stage 4**

### Research Presentation Session: Cardiac

### RPS 203

### Photon-counting CT: cardiac applications

**Moderator**
D. Beitzke; Vienna/AT
*(dietrich.beitzke@meduniwien.ac.at)*
**Author Disclosures:**
Dietrich Beitzke: Speaker: Siemens Healthineers; GE Healthcare

### RPS 203-2

**Intra-individual comparison of coronary artery stenosis measurements between photon counting and energy-integrating detector-based coronary computed tomography angiography**

*T. S. Emrich*[1], E. Wolf[1], M. C. Halfmann[1], E. Zsarnóczay[2], N. Fink[2], Y. Yang[1], J. O'Doherty[3], U. J. Schoepf[2], A. Varga-Szemes[2]; [1]Mainz/DE, [2]Charleston, SC/US, [3]Malvern, PA/US
*(emrich@musc.edu)*

**Purpose:** To evaluate the intra-individual difference in percentage diameter stenosis (PDS) measurements based on coronary computed tomography angiography (CCTA), comparing a photon-counting (PCD) with a conventional energy-integrating detector (EID) CT.
**Methods or Backround:** CCTA was prospectively acquired in 23 patients (16 men, 65±12.1 years, 42 coronary artery stenosis) who underwent PCD- and EID-CT scans within a median of 5.5(3.0-12.5) days between the two scans in the period from August 2021 to March 2022. The PCD-CT allows spectral CT data acquisition at high spatial (144x0.4mm) and temporal resolution, while the EID-CT (Somatom Force) is equipped with a collimation of 192x0.6mm. Tube voltage was set to 120 kVp for the PCD-CT and 110 kVp for the EID-CT. A sequential cardiac protocol was acquired on both CT systems with similar radiation doses. CCTA images were reconstructed as similar as possible in regard to slice thickness, iterative reconstruction level and kernel selection. PDS was quantified using a commercially available software solution by two readers.
**Results or Findings:** The PDS (PCD-CT) showed a very strong correlation to PDS (EID-CT) (r=0.97, p<0.0001, ICC=0.98). However, PDS measurements were significantly lower on the PCD-CT compared with the EID-CT (PCD-CT 44.9% (32.4%-60.7%), EID-CT: 46.4% (34.3%-62.6%), p<0.0001, mean bias=1.8, Limits of Agreement: -5.3/8.9,). This led to a reclassification of CAD-RADS categories in eight of 42 coronary stenoses (19.0%). There was an excellent agreement between both readers for PDS measurements (ICC=0.98).
**Conclusion:** PDS measurements from PCD-CCTA show excellent correlation to EID-CCTA but lead to different CAD-RADS categories in around 20% of lesions. Further studies are needed to evaluate the accuracy of PCD-CCTA imaging protocols and their potential effect on the accuracy of coronary stenosis measurements and downstream medical therapies.
**Limitations:** Single-centre study, missing invasive correlation.
**Ethics committee approval:** This study was approved by IRB Medical University of South Carolina
**Funding for this study:** Research support provided by Siemens Healthineers
**Author Disclosures:**
Emese Zsarnóczay: Nothing to disclose
Jim O'Doherty: Employee: Siemens Healthcare
Uwe Joseph Schoepf: Research/Grant Support: Bayer, Guerbet, Elucid, Siemens Healthcare, Bracco, HeartFlow
Tilman Stephan Emrich: Speaker: Siemens Healthineers Advisory Board: Siemens Healthineers Research/Grant Support: Siemens Healthineers
Moritz Christian Halfmann: Nothing to disclose
Elias Wolf: Nothing to disclose
Nicola Fink: Nothing to disclose
Yang Yang: Nothing to disclose
Akos Varga-Szemes: Research/Grant Support: Elucid, Siemens Healthcare

### RPS 203-3

**Ultra-high resolution dual-source photon-counting computed tomography for coronary artery assessment**

*S. P. Sharma*, J. van der Bie, M. van Straten, D. Bos, M. L. Dijkshoorn, R. Booij, A. Hirsch, R. P. J. Budde; Rotterdam/NL
*(s.sharma@erasmusmc.nl)*

**Purpose:** The study is intended to assess the image quality of ultra-high resolution (UHR) coronary computed tomography angiography (CCTA) performed on dual-source photon-counting computed tomography (PCCT).
**Methods or Backround:** Consecutive patients who underwent a clinically indicated calcium score scan followed by a UHR (120 x 0.2mm collimation) CCTA with PCCT were included. CTA images were reconstructed at 0.2mm slice thickness with Bv44, Bv48 and Bv56 kernels and quantum iterative reconstruction level 4. Contrast-to-noise (CNR) and signal-to-noise ratios (SNR) were measured on Bv56 images in the ascending aorta and subcutaneous adipose tissue. An experienced radiologist scored all reconstructions per coronary segment (18-segment model) for presence, image quality (graded with a 5-point Likert scale, 1=non diagnostic, 5=excellent), motion artefacts, stack artefacts, plaque presence and composition as well as stenosis degree.
**Results or Findings:** 32 patients were included (mean age 64±9 years, 56% male, mean BMI 26.8±4.5, median calcium score 91[38-507]). Mean heart rate during scanning was 72±10 bpm. The number of stacks used was 5 (n=5), 6 (n=16), 7 (n=8), 8 (n=3). Mean CTDIvol was 19.1±6.3mGy and mean DLP 257±106mGycm. The SNR was 9.5±2.2 and the CNR was 11.8±2.5. Motion artefacts were present in 34 segments, stack artefacts in 31 segments and other (metal/stent) artefacts in 5 segments. Of the potential 576 coronary segments (32 patients x 18 segments), 112 were absent and 21 non-evaluable due to size. The remaining 443 segments were graded as excellent 258(58%); very good 97(22%); good 51(12%); adequate 17(4%); 13(3%) as poor; and 7(2%) non-evaluable due to quality.
**Conclusion:** UHR dual-source PCCT CCTA provides excellent or very good image quality in 80% of coronary segments at high heart rates at moderate radiation dose with only limited stack artefacts.
**Limitations:** No comparison with conventional CCTA was done.
**Ethics committee approval:** Since this study is purely retrospective and observational, the need for ethics committee approval was waived by the institutional review board.
**Funding for this study:** No funding was received for this study.
**Author Disclosures:**
Ricardo P. J. Budde: Nothing to disclose
Alexander Hirsch: Nothing to disclose
Marcel van Straten: Nothing to disclose
Simran Priyanka Sharma: Nothing to disclose
Marcel L. Dijkshoorn: Nothing to disclose
Ronald Booij: Nothing to disclose
Daniel Bos: Nothing to disclose
Judith van der Bie: Nothing to disclose

### RPS 203-4

**Coronary stenosis quantification with ultra-high-resolution photon-counting detector CT angiography: preliminary experience**

*V. Mergen*, A. Candreva, B. Stähli, R. Manka, H. Alkadhi, M. Eberhard; Zurich/CH
*(victor.mergen@usz.ch)*

**Purpose:** To assess the accuracy of stenosis quantification using ultra-high-resolution coronary CT angiography (CCTA) with photon-counting detector CT (PCD-CT) compared with invasive quantitative coronary angiography (QCA).
**Methods or Backround:** In this IRB-approved, retrospective study, nineteen patients (7 women, mean age 78±9 years) undergoing ECG-gated ultra-high-resolution CCTA with a dual-source PCD-CT and QCA before transcatheter aortic valve replacement were included. CCTA images were reconstructed with a slice thickness of 0.6 mm using a smooth vascular kernel (Bv40) and with a slice thickness of 0.2 mm using a sharp vascular kernel (Bv64). The degree of stenosis was evaluated using QCA (reference standard) and CCTA. Stenoses were classified as minimal (1-24%), mild (25-49%), moderate (50-69%), and severe (70-99%).
**Results or Findings:** In total, 141 coronary artery segments were assessed. QCA showed minimal, mild, moderate and severe stenoses in 113, 20, 4, and 4 cases, respectively. Stenosis grading on CCTA showed small differences and strong correlation to QCA (mean error 0.42%, limits of agreement (LoA), -12%/13%, r=.94 and mean error -0.03%, LoA -14%/14%, r=.93 for reconstructions with a slice thickness of 0.6 mm using the smooth vascular kernel and with a slice thickness of 0.2 mm using the sharp vascular kernel, respectively). The agreement was almost perfect between stenoses categories determined on ICA and CCTA images reconstructed with a slice thickness of 0.6 mm using the smooth vascular kernel (κ=.864, reclassification of 18/141 segments) and improved slightly with a slice thickness of 0.2 mm using the sharp vascular kernel (κ=.895, reclassification of 15/141 segments).
**Conclusion:** Ultra-high-resolution coronary CT angiography with photon-counting detector CT allows for an accurate stenosis quantification compared with QCA.
**Limitations:** Limited number of moderate and severe stenoses.
**Ethics committee approval:** No information provided by the submitter.
**Funding for this study:** No funding was received for this study.
**Author Disclosures:**
Barbara Stähli: Nothing to disclose
Victor Mergen: Nothing to disclose
Matthias Eberhard: Nothing to disclose
Alessandro Candreva: Nothing to disclose
Robert Manka: Nothing to disclose
Hatem Alkadhi: Nothing to disclose

### RPS 203-5

**Ultra-high resolution photon-counting coronary CT angiography improves coronary stenosis quantification over a wide range of heart rates – a dynamic phantom study**

*E. Zsarnóczay*[1], N. Fink[1], J. O'Doherty[1], T. Allmendinger[2], E. Wolf[1], J. P. Griffith[1], A. Varga-Szemes[1], T. S. Emrich[1], U. J. Schoepf[1]; [1]Charleston, SC/US, [2]Forchheim/DE

**Purpose:** This study aimed to investigate the effect of using photon-counting detector (PCD)-CT with ultra-high resolution (UHR) on stenosis quantification accuracy and blooming artifacts at low, intermediate and high heart rates in a dynamic motion phantom.
**Methods or Backround:** Two vessel phantoms (4 mm in diameter), containing diameter stenoses of 25% and 50%, filled with different concentrations of iodine contrast material, were placed inside an anthropomorphic thorax CT phantom attached to a coronary 3D motion simulator. Scanning was performed on a dual-source PCD-CT system using an ECG-gated mode at UHR (0.2mm slice thickness) and standard resolution (SR) (0.6mm slice thickness). Images were reconstructed at 60, 80 and 100 beats per minute (bpm) (UHR: Bv56 kernel, quantum iterative reconstruction (QIR) at level 3, slice thickness 0.2mm; SR: virtual monoenergetic images at 55 keV, Bv40 kernel, QIR3, slice thickness 0.6mm). Percent diameter stenosis (PDS) and blooming artifact measurements were performed by two readers.
**Results or Findings:** The measured PDS values for UHR were more accurate compared to SR for both lesions at each heart rate (e.g. 50% lesion at 100 bpm; SR: 61.0% [58.6%-64.3%] vs UHR: 52.4% [51.3%-54.3%]; p<0.001). The overall mean difference across all heart rates and lesions compared to the nominal stenoses was 9.2% (Limit of Agreement, 2.4%/16.0%) for SR vs 2.4% (Limit of Agreement, -2.8%/7.5%) for UHR. Blooming artifacts decreased with UHR compared to SR for both lesions at every heart rate (e.g. 50% lesion at 100 bpm; SR: 70.2 [64.8-78.1] vs UHR: 56.1 [51.2-60.8]; p<0.001).
**Conclusion:** This motion phantom study demonstrated improved stenosis quantification accuracy and reduced blooming artifacts with UHR PCD-CT compared to SR, independent of heart rate.
**Limitations:** Transferability of the results to human application requires further investigation.
**Ethics committee approval:** No information provided by the submitter.
**Funding for this study:** Research grant provided by Siemens Healthineers.
**Author Disclosures:**
Emese Zsarnóczay: Nothing to disclose
Jim O'Doherty: Nothing to disclose
Joseph P. Griffith: Nothing to disclose
Thomas Allmendinger: Employee: Siemens Healthcare GmbH
Uwe Joseph Schoepf: Nothing to disclose
Tilman Stephan Emrich: Nothing to disclose
Elias Wolf: Nothing to disclose
Nicola Fink: Nothing to disclose
Akos Varga-Szemes: Nothing to disclose

### RPS 203-6

**Ultra-high resolution in photon-counting detector coronary CT angiographies leads to significant stenosis reclassification**

*M. C. Halfmann*, S. Bockius, Y. Yang, D. Graafen, L. Müller, M. Hell, T. S. Emrich; Mainz/DE

**Purpose:** This study is intended to assess the influence of photon-counting detector (PCD) CT-derived ultra-high resolution (UHR) reconstructions from coronary CT angiographies (CCTA) on the quantification of stenosis.
**Methods or Backround:** For this study, 20 patients (6 (30%) female) who had previously undergone UHR-CCTA on a first-generation dual-source PCD CT were retrospectively identified. CCTA images were reconstructed with slice thicknesses of 0.6mm, 0.4mm and 0.2mm (UHR), respectively. Subsequently, coronary stenoses were identified and quantified using dedicated semi-automatic software. Normal distribution was assessed using Shapiro-Wilk tests, and differences between reconstructions were assessed using one-way analysis of variance.
**Results or Findings:** A total of 41 coronary stenoses were identified (18 (44%) left anterior descending, 10 (24%) ramus circumflexus, 12 (30%) right coronary artery, and one left main coronary artery (2%)). Fully calcified plaques were the cause of stenosis in 18 (45%) cases, while mixed plaques accounted for 25 (55%). The median Agatston-score was 490 (interquartiles 180-1235). The luminal stenosis was significantly lower when quantified in UHR reconstructions (26±15%) when compared to reconstructions with 0.4mm (34±17%, p=0.03) and 0.6mm slice thickness (38±16%, p<0.001), respectively. This led to a reclassification of patients into a lower category of the Coronary Artery Disease-Reporting and Data System (CAD-RADS) for 14 (70%) and 13 (65%) cases for the 0.6mm and 0.4mm slice thicknesses, respectively.
**Conclusion:** Increased spatial resolution in UHR reconstructions from PCD-CCTA significantly influences stenosis quantification towards lower magnitudes of luminal stenosis in patients with partially or fully calcified coronary plaques. The resulting reclassification into the lower CAD-RADS category may have a relevant impact on downstream therapy management and indication for invasive procedures.
**Limitations:** This is a single-centre retrospective pilot study.
**Ethics committee approval:** This study was approved by IRB no. 2022-16359.
**Funding for this study:** No outside funding was received for this study.
**Author Disclosures:**
Dirk Graafen: Nothing to disclose
Michaela Hell: Nothing to disclose
Stefanie Bockius: Nothing to disclose
Tilman Stephan Emrich: Speaker: Siemens Healthineers
Moritz Christian Halfmann: Nothing to disclose
Lukas Müller: Nothing to disclose
Yang Yang: Nothing to disclose

### RPS 203-7

**Virtual monoenergetic reconstructions influence the accuracy of stenosis measurements of coronary computed tomography angiography using a first-generation, dual-source photon counting CT**

*T. S. Emrich*[1], E. Wolf[2], M. C. Halfmann[1], M. Hell[1], J. P. Griffith[2], N. Fink[2], E. Zsarnóczay[2], U. J. Schoepf[2], A. Varga-Szemes[2]; [1]Mainz/DE, [2]Charleston, SC/US
*(emrich@musc.edu)*

**Purpose:** Coronary CT angiography (CCTA) has emerged as a clinical first-line test for the assessment of stable coronary artery disease (CAD). However, conventional energy integrating systems suffer from certain limitations, including the overestimation of stenosis due to calcium blooming. With the recent introduction of clinical PCD-CT systems, the possibility of reconstructing virtual monoenergetic reconstructions (VMI) at different keV levels at the high temporal resolution needed for cardiac imaging became available. The purpose of this study was to evaluate the influence of virtual monoenergetic reconstructions on the accuracy of coronary artery stenosis measurements on a clinical first-generation dual-source photon-counting detector computed tomography (PCD-CT) system, compared to invasive coronary angiography.
**Methods or Backround:** Thirty-six patients (25 (69.4%) male, mean age 70.0±9.1 years) underwent PCD-CCTA and invasive coronary angiography as part of their clinical workup to evaluate stable CAD. Different keV levels from 40-140 keV in 10 keV steps were reconstructed with a slice thickness of 0.6 mm, Qr40 Kernel, and QIR strength level of 3 (Figure). CT-based stenosis quantification of 62 calcified, mixed or soft tissue lesions was compared to quantitative coronary angiography (QCA).
**Results or Findings:** Different keV reconstructions lead to significantly different stenosis measurements (e.g. mean CT-based stenosis: 67.5 ± 8.9% at 40 keV vs 53.9 ± 9.7% at 140 keV, p<0.001). The optimal VMI keV level depended on the tissue quality of the plaque. Compared to the reference, all CT-based stenosis measurements led to an overestimation of stenosis by 5 to 20%.
**Conclusion:** VMI reconstructions have the potential to improve the accuracy of stenosis quantification for PCD-CCTA, therefore potentially reducing the amount of unneeded invasive coronary angiographies.
**Limitations:** Small sample size, no outcome data.
**Ethics committee approval:** This study was approved by Ethics Committee Rheinland-Pfalz
**Funding for this study:** No funding was received for this study.
**Author Disclosures:**
Emese Zsarnóczay: Nothing to disclose
Michaela Hell: Nothing to disclose
Joseph P. Griffith: Nothing to disclose
Uwe Joseph Schoepf: Research/Grant Support: Bayer, Guerbet, Elucid, Siemens Healthcare, Bracco, HeartFlow
Tilman Stephan Emrich: Advisory Board: Siemens Healthineers Speaker: Siemens Healthineers Research/Grant Support: Siemens Healthineers
Moritz Christian Halfmann: Nothing to disclose
Elias Wolf: Nothing to disclose
Nicola Fink: Nothing to disclose
Akos Varga-Szemes: Research/Grant Support: Elucid, Siemens Healthcare

### RPS 203-8

**The impact of Virtual Non-Calcium CCTA reconstruction on CT cFFR values compared to standard CCTA images: a pilot study**

*D. Juskanic*[1], S. Hollý[1], A. Obert[1], J. Polakova Mistinova[2], Z. Berecova[2], L. Patrovic[1]; [1]Nitra/SK, [2]Bratislava/SK

**Purpose:** High coronary calcium is traditionally known to obstruct the clarity of coronary CT angiography (CCTA) images and increase measurement errors in assessing the severity of coronary artery stenoses. CT virtual non-calcium (VNCa) image reconstruction in CCTA is now available with the introduction of spectral detectors. We evaluated the impact of VNCa CCTA on CT cFFR values compared to standard CCTA images.
**Methods or Backround:** 24 CCTA studies acquired on a CT with photon counting detector (CT Naeotom Alpha, VA50, Siemens Healthineers) were

Wednesday included in the study. In VNCa reconstructions, all remaining parameters were unchanged compared to standard CCTA. For quantification of lesion hemodynamic relevance, we used s.via Frontier CT cFFR prototype. For consistency, at least two readers confirmed the correct segmentation of the coronary tree model, myocardial mass, as well as the measurements' localisation in the prototype. The difference was recorded if at least 0,05 cFFR value change was present.
**Results or Findings:** No statistically significant difference in cFFR values was found between the standard and VNCa groups (p=0.88). Out of 110 measurements, the value crossed the 0.8 threshold in 14 measurements (12.7%) and occurred mostly in the moderate calcification group. However, the changes in the number of lesions with cFFR < 0.8 were not statistically significant in any of the calcium categories (p = 0.776; p = 0.516; p = 0.696 for the mild, moderate and severe categories, respectively). The direction of the majority of changes (59.1%) was towards lower cFFR values.
**Conclusion:** Virtual non-calcium CCTA reconstructions showed a tendency to decrease CT cFFR values compared to standard CCTA images, although these were not statistically significant. Potential impact on different management occurred most often in the moderate calcification group.
**Limitations:** This is a single-centre pilot study.
**Ethics committee approval:** No information provided by the submitter.
**Funding for this study:** No funding was received for this study.
**Author Disclosures:**
Dominik Juskanic: Nothing to disclose
Lukas Patrovic: Nothing to disclose
Jana Polakova Mistinova: Nothing to disclose
Zuzana Berecova: Nothing to disclose
Adam Obert: Nothing to disclose
Samuel Hollý: Nothing to disclose

### RPS 203-9

**Optimal kernel type and sharpness level improve objective and subjective image quality for photon counting coronary CT angiography**
*Y. Yang*, M. C. Halfmann, D. Graafen, M. Hell, C. Düber, K-F. Kreitner, T. S. Emrich; Mainz/DE
*(yang.yang@unimedizin-mainz.de)*

**Purpose:** As a novel technique, Photon-Counting-Detector-CT (PCD-CT) offers a wide variety of possible kernels and sharpness levels for image reconstruction, which necessitate clinical evaluation. The aim of this study was to find optimal settings for coronary computed tomography angiography (CCTA) by assessing objective and subjective image quality.
**Methods or Backround:** This retrospective study included 30 patients (8(27%) female, mean age 63±13 years) who had undergone PCD-CCTA with an electrocardiography-gated high-pitch mode. Images were reconstructed using three different kernels and four sharpness levels (Br36/40/44/48, Bv36/40/44/48 and Qr36/40/44/48), quantum iterative reconstruction at a strength level of 3, at spectral Quantumplus-mode with a slice thickness of 0.4 mm. Vessel sharpness, mean attenuation, image noise, and contrast-to-noise ratio (CNR) were quantified in proximal and distal coronary arteries. For subjective image quality, two blinded readers assessed overall image quality, image noise and evaluation of coronary artery plaques and vessel walls using a 5-point Likert scale.
**Results or Findings:** Mean attenuation, image noise, CNR and vessel sharpness significantly differed across kernels (all p<0.001), with the Br-kernel reaching the highest mean attenuation. With increasing kernel sharpness, image noise and vessel sharpness increased while CNR continuously decreased. Generally, reconstruction with Br-kernel showed the highest CNR (Br>Bv>Qr) except at sharpness level 40; superior CNR was found for Bv-kernel in distal coronary arteries. Furthermore, Bv-kernel has significantly higher vessel sharpness than Br- and Qr-kernels (p<0.001). Subjective overall image quality, image noise and sharpness of plaques and vessel wall were rated best for Bv36- and Bv40-Kernels, followed by Br36- and Qr36-Kernels.
**Conclusion:** Considering objective analysis, especially in regard to vessel sharpness and subjective evaluation of coronary arteries, reconstructions with a Bv40 kernel are beneficial to achieve optimal image quality in spectral CCTA with PCD-CT.
**Limitations:** Retrospective single center study.
**Ethics committee approval:** This study was approved by Ethics Committee, State Medical Association Rheinland-Pfalz, Germany
**Funding for this study:** No funding was received for this study.
**Author Disclosures:**
Dirk Graafen: Nothing to disclose
Michaela Hell: Nothing to disclose
Christoph Düber: Nothing to disclose
Tilman Stephan Emrich: Speaker: Siemens
Moritz Christian Halfmann: Nothing to disclose
Karl-Friedrich Kreitner: Nothing to disclose
Yang Yang: Nothing to disclose

### RPS 203-10

**Radiation dose reduction in photon-counting-based coronary artery calcium scoring using a virtual non-iodine algorithm**
*J. P. Griffith*[1], N. Fink[1], E. Zsarnóczay[1], J. O'Doherty[2], D. Sanchez[1], M. J. Willemink[3], A. Varga-Szemes[1], U. J. Schoepf[1], T. S. Emrich[4];
[1]Charleston, SC/US, [2]Malvern, PA/US, [3]Stanford, CA/US, [4]Mainz/DE

**Purpose:** To evaluate the feasibility of radiation dose reduction in coronary artery calcium scoring (CACS) using a virtual non-iodine (VNI) reconstruction algorithm on a clinical first-generation photon counting detector (PCD) computed tomography (CT) system.
**Methods or Backround:** CACS was performed with an anthropomorphic chest phantom simulating three patient sizes by using different extension rings. The radiation dose was gradually reduced using tube current modulation, resulting in four radiation dose levels: 100, 75, 50 and 25%. VNI reconstructions (at 55keV, QIR1 and 60keV; QIR4) were evaluated regarding image quality (image noise [IN], contrast-to-noise ratio [CNR]) and CACS analysis. True non-contrast (TNC) reconstructions at 70keV and QIR “off” served as the reference.
**Results or Findings:** INTNC was significantly higher than INVNI. INVNI was significantly higher at 55keV, QIR1 than at 60keV, QIR4 (e.g., at standard dose: 16.7+/-1.9 vs. 12.8+/-1.7 vs. 7.7+/-0.9; p<0.001 for every radiation dose level). INVNI at 60keV, QIR4 met the recommended noise target at every radiation dose. CNRTNC was higher than CNRVNI but less reduced using 60keV, QIR4 (p< 0.001 for all). CACSVNI showed strong correlation and excellent agreement at every radiation dose level (p<0.001, r>0.9, ICC>0.9, for all) and deviated less than 5% compared to CACSTNC at standard radiation dose. Coefficients of variations of root-mean-squared error were less than 10%, and thus clinically non-relevant when comparing CACSVNI of every radiation dose level with CACSTNC at standard dose.
**Conclusion:** Radiation dose reduction using VNI-based CACS is feasible on PCD-CT without compromising image quality and CACS values. VNI reconstructions at 60keV, and QIR4 performed slightly better than those at 55keV and QIR1.
**Limitations:** Phantom study; four predefined radiation dose levels without investigating the lowest possible level; radiation dose levels lower than usually expected in CCTA.
**Ethics committee approval:** No information provided by the submitter.
**Funding for this study:** Funding was received from Research grant Siemens Healthineers.
**Author Disclosures:**
Emese Zsarnóczay: Nothing to disclose
Nicola Fink: Nothing to disclose
Jim O'Doherty: Employee: Siemens Medical Solutions
Daniel Sanchez: Nothing to disclose
Joseph P. Griffith: Nothing to disclose
Uwe Joseph Schoepf: Consultant: Siemens Medical Solutions Research/Grant Support: Bayer, Bracco, Elucid Bioimaging, Guerbet, HeartFlow Inc., Keya Medical, and Siemens
Tilman Stephan Emrich: Speaker: Siemens Medical Solutions
Martin J. Willemink: Nothing to disclose
Akos Varga-Szemes: Research/Grant Support: Bayer, Elucid Bioimaging, Siemens

### RPS 203-11

**Intra-individual comparison of coronary calcium scoring between photon counting detector- and energy integrating detector-CT: effects on risk reclassification**
*T. S. Emrich*[1], E. Wolf[1], M. C. Halfmann[1], E. Zsarnóczay[2], N. Fink[2], J. O'Doherty[3], U. J. Schoepf[2], A. Varga-Szemes[2]; [1]Mainz/DE, [2]Charleston, SC/US, [3]Malvern, PA/US
*(emrich@musc.edu)*

**Purpose:** The study is intended to compare coronary artery calcium score (CACS) between photon-counting (PCD) and conventional energy integrating detector (EID) CT in a phantom and prospective patient study.
**Methods or Backround:** A commercially available CACS phantom was scanned with a standard CACS protocol (120 kVp, slice thickness/increment 3/1.5 mm, Qr36 kernel), with filtered back projection on the EID-CT, and with monoenergetic reconstruction at 70 keV and quantum iterative reconstruction off on the PCD-CT. The same settings were used to prospectively acquire data in patients (n=23, 65±12.1 years) who underwent PCD- and EID-CT scans with a median of 5.5 (3.0-12.5) days between the two scans in the period from August 2021 to March 2022. CACS was quantified using a commercially available software. A regression formula was obtained from the aforementioned comparison and applied to simulate risk reclassification in a pre-existing cohort of 514 patients who underwent a cardiac EID-CT between January and December 2021.
**Results or Findings:** Based on the phantom experiment, CACS_PCD-CT showed a more accurate measurement of the reference CAC volumes (overestimation of physical volumes: PCD-CT 66.1±1.6% vs EID-CT: 77.2±0.5%). CACS_EID-CT and CACS_PCD-CT were strongly correlated; however, the latter measured significantly lower values in the phantom

(CACS_PCD-CT: 60.5(30.2-170.3) vs CACS_EID-CT 74.7(34.6-180.8), p=0.0015, r=0.99, mean bias=-9.7, Limits of Agreement (LoA) -36.6/17.3) and in patients (non-significant) (CACS_PCD-CT: 174.3 (11.1-872.7) vs CACSEID-CT 218.2 (18.5-876.4), p=0.10, r=0.94, mean bias=-41.1, LoA -315.3/232.5). The systematic lower measurements of CACS on the PCD-CT system led to the reclassification of 5.25% of the simulated patient cohort to a lower classification class.
**Conclusion:** CACS on PCD-CT is feasible and correlates strongly with CACS derived by EID-CT; however, it leads to lower CACS values. PCD-CT may provide results that are more accurate for CACS than EID-CT.
**Limitations:** Single-Center Study.
**Ethics committee approval:** This study was approved by IRB Medical University of South Carolina
**Funding for this study:** Research support provided by Siemens Healthineers
**Author Disclosures:**
Emese Zsarnóczay: Nothing to disclose
Jim O'Doherty: Employee: Siemens Healthcare
Uwe Joseph Schoepf: Research/Grant Support: Bayer, Guerbet, Elucid, Siemens Healthcare, Bracco, HeartFlow
Elias Wolf: Nothing to disclose
Tilman Stephan Emrich: Advisory Board: Siemens Healthineers Speaker: Siemens Healthineers Research/Grant Support: Siemens Healthineers
Moritz Christian Halfmann: Nothing to disclose
Nicola Fink: Nothing to disclose
Akos Varga-Szemes: Research/Grant Support: Elucid, Siemens Healthcare

### RPS 203-12

**Virtual non-iodine algorithm for coronary artery calcium scoring on photon-counting detector CT: impact of virtual monoenergetic and quantum iterative reconstructions**
*E. Zsarnóczay*, N. Fink, U. J. Schoepf, J. Griffith, E. Wolf, J. O'Doherty, A. Varga-Szemes, T. S. Emrich; Charleston, SC/US

**Purpose:** Virtual non-iodine (VNI) reconstructions have been shown to significantly underestimate coronary artery calcium scoring (CACS) on photon-counting detector (PCD) data. This study aimed to evaluate the impact of virtual monoenergetic image (VMI) and quantum iterative reconstructions (QIR) on CACS accuracy using this VNI algorithm on a clinical first-generation PCD-CT system.
**Methods or Backround:** CACS was evaluated in an anthropomorphic chest phantom simulating three different patient sizes and in 21 patients (64.3±9.3 years; 61.9% male) with a non-enhanced and contrast-enhanced cardiac PCD-CT scan. VNI reconstructions were performed at different VMI levels (55-80keV) and different QIR levels (strength 1-4). True non-contrast (TNC) scans at 70keV and QIR"off" served as reference for phantom and patient data.
**Results or Findings:** At every evaluated VMI and QIR level, in vitro and in vivo CACS-VNI showed strong correlation and excellent agreement (r>0.9, p<0.001, ICC>0.9 for all) with CACS-TNC. Phantom and patient CACS-VNI increased significantly with decreasing keV levels (in vitro: from 475.2±26.3 at 80keV up to 652.5±42.2 at 55keV; in vivo: from 140.2 [5.8/685.7] at 80keV up to 254.7 [27.4/1048] at 55keV; p<0.001 for all). In vitro CACS-VNI increased with increasing QIR at low VMI levels. In vivo, CACS-VNI were significantly higher at QIR 1 than at QIR 4 only at 80 keV (157.2 [8.8/710.5] vs 140.2 [5.8/685.7]; p<0.001). CACS-VNI closest to CACS-TNC was obtained at 55 keV, QIR 1 (+0.05%) in phantoms (averaged over phantom sizes) and at 55 keV, QIR 4 (+2.3%) in patients.
**Conclusion:** CACS-VNI can significantly be adjusted with VMI reconstructions which can be used to counteract the previously shown underestimation compared to CACS-TNC.
**Limitations:** Limited cohort size; the impact of body habitus and plaque characteristics were not evaluated; interscan variability was only assessed in the phantom study.
**Ethics committee approval:** The protocol of this HIPAA-compliant, prospective study was approved by the local Institutional Review Board. All participants provided written informed consent.
**Funding for this study:** Research grant received from Siemens Healthineers.
**Author Disclosures:**
Emese Zsarnóczay: Nothing to disclose
Jim O'Doherty: Nothing to disclose
Uwe Joseph Schoepf: Nothing to disclose
Elias Wolf: Nothing to disclose
Tilman Stephan Emrich: Nothing to disclose
Joseph Griffith: Nothing to disclose
Nicola Fink: Nothing to disclose
Akos Varga-Szemes: Nothing to disclose

### RPS 203-13

**Myocardial characterization with extracellular volume mapping on a first-generation, photon-counting detector CT with MRI reference**
*A. Varga-Szemes*, G. Aquino, U. J. Schoepf, N. Fink, E. Zsarnóczay, J. Griffith, T. S. Emrich; Charleston, SC/US

**Purpose:** The study is intended to compare extracellular volume (ECV) quantification by using a first-generation, photon counting-detector CT (PCD-CT) against MRI reference.
**Methods or Backround:** In this single-centre, prospective study, participants (n=30) underwent same-day cardiac PCD-CT and MRI with native and post-contrast T1 mapping and late gadolinium enhancement (LGE) for various clinical indications between July 2021 and January 2022. Global and midventricular ECV was assessed using three methods: single-energy CT (CT-ECVSE), dual-energy CT (CT-ECVDE), and MRI T1 mapping (MRI-ECV). Quantitative comparisons between all techniques were performed.
**Results or Findings:** The final cohort included 29 study participants (mean age 54 ± 17 years; 15 men). There was a strong correlation between global CT-ECVSE and CT-ECVDE (r = 0.91), with 40% lower radiation exposure using the dual-energy method (CTDIvol 16.8 vs 10.1 mGy, P < 0.001). In comparison to MRI-ECV, CT-ECVDE showed a strong correlation (global ECV, r = 0.91; midventricular ECV, r = 0.82; both P < 0.001), but slightly overestimated ECV by 1.9 ± 2.5% on global analysis (MRI 31.0 ± 5.8 vs CTDE 32.9 ± 5.4; P = 0.001) and 2.3 ± 3.2% on midventricular analysis (MRI 30.4 ± 5.5 vs CTDE 32.7 ± 4.7; P = 0.001).
**Conclusion:** Myocardial tissue characterization by PCD-CT-based quantitative ECV analysis showed a strong correlation to MRI as the reference standard.
**Limitations:** Parameters used for image reconstructions have not been validated as the most accurate settings for ECV assessment (recommendations by the vendor); no histology reference; MRI as a reference in the diastolic phase, CT-ECV in the systolic phase; MRI-ECV included only three slices, CT-ECV the whole heart; higher contrast media dose compared to standard CCTA examination.
**Ethics committee approval:** The protocol of this HIPAA-compliant, prospective study was approved by the local Institutional Review Board. All participants provided written informed consent.
**Funding for this study:** This work was supported by a research grant from Siemens Healthineers.
**Author Disclosures:**
Emese Zsarnóczay: Nothing to disclose
Uwe Joseph Schoepf: Consultant: Siemens, Bayer, Elucid Grant Recipient: Siemens, Bayer, Elucid, Keya, HeartFlow
Tilman Stephan Emrich: Speaker: Siemens
Joseph Griffith: Nothing to disclose
Nicola Fink: Nothing to disclose
Akos Varga-Szemes: Grant Recipient: Siemens Consultant: Bayer Consultant: Elucid Bioimaging
Gilberto Aquino: Nothing to disclose

**11:30-12:30** **Research Stage 1**

### Research Presentation Session: Breast

### RPS 302

### AI in breast imaging

**Moderator**
A. Hussien; Dallas, TX/US
*(Abdelmohsen.RadwanHussien@UTSouthwestern.edu)*

### RPS 302-2

**Performance comparison of artificial intelligence (AI) to double reading in the Australian BreastScreen Program**
*J. Waugh*, J. Evans, M. J. Miocevic, D. J. Lockie, R. Bell, P. Aminzadeh, A. M. Lynch; Melbourne/AU
*(jrwinoz@bigpond.net.au)*

**Purpose:** The study aimed to evaluate the performance of an AI algorithm using a ground truth data set of consecutive prevalent round digital mammograms with verified 3-year follow-up in a low cancer prevalence (~ 1%) screening setting.
**Methods or Backround:** The Monash BreastScreen service uses independent double reading with arbitration by a single expert reader. The AI system retrospectively analysed prevalent screens from 2017. The AI system (Transpara V1.7 from ScreenPoint Medical) assigned scores in deciles from 1 – 10, with higher scores indicating an increasing risk of malignancy. Interval cancers and next-round cancers were categorised at a blinded consensus review. Findings were used to develop a proposed protocol for the incorporation of AI into standard practice.

**Results or Findings:** Of the 7533 consecutive prevalent screens, 728 were recalled by the radiologists and included 54 cases of invasive breast cancer (IBC). Fifty-two of the 54 IBCs were within the 1611 cases scored as either 9 or 10 by Transpara. If the IBCs from the prevalent round were combined with interval IBCs and IBCs from the subsequent round considered after expert review to have had signs in 2017, then the radiologists identified 54/67 IBCs and Transpara identified 62/67 (sensitivity 80.6% radiologists vs 92.5% Transpara, p=0.07).
**Conclusion:** Only 2/54 prevalent IBCs were not scored 9 or 10 by Transpara and this AI group included some interval cancers and cancers from the subsequent round that were not identified by the radiologists. A proposed protocol for the incorporation of AI into a screening protocol will be discussed, which includes all images being read by a radiologist and Transpara, with no loss of sensitivity. Potential benefits include reducing the number of women recalled for benign lesions, interval cancers and workload.
**Limitations:** Limitation will be explained later.
**Ethics committee approval:** No information provided by the submitter.
**Funding for this study:** Funding was received from Monash University and BSA.
**Author Disclosures:**
Anne Margaret Lynch: Nothing to disclose
Jill Evans: Nothing to disclose
Robin Bell: Nothing to disclose
John Waugh: Nothing to disclose
Darren J. Lockie: Nothing to disclose
Miranda Jane Miocevic: Nothing to disclose
Parisa Aminzadeh: Nothing to disclose

### RPS 302-3

**Can an artificial intelligence system (AIS) reduce the workload of mammography readings in a population-based breast screening program (BSP)?**

*C. M. Weiss*, E. Di Gaetano, G. Soppelsa, E. Cattarin, R. Cerniato, L. Loeffelstiel, G. Morana; Treviso/IT

**Purpose:** To hypothesize possible alternative reading paths using AIS-aided blinded double human reading (AIS-BDHR) with real-world data from a breast screening program (BSP).
**Methods or Backround:** In the Treviso BSP (Italy), period November 2021 to August 2022, 38247 consecutive screening mammograms (SMs) were performed in 49-75years aged women (mean 61.84yrs). SMs were processed with AIS Lunit INSIGHT MMG, which provided a malignancy score (MS) in percentage (range 0-100; ≥10 considered positive) and then subdivided into three MS-groups: MS-group1(MS-G1) 0-5%; MS-group2(MS-G2) >5-<95%; MS-group3(MS-G3) ≥95%. Recall rate(RR), detection rate(DR) of biopsy-proven breast cancers (BCs), positive predictive values(PPV), negative predictive value(NPV), sensitivity(Sn), and specificity(Sp) were evaluated for each MS-group.
**Results or Findings:** MS-group distribution was: MS-G1 64.99% (24856/38247); MS-G2 34.58% (13225/38247); MS-G 30.43% (166/38247). In MS-G1, no BC was diagnosed or missed. In MS-G2, AIS-BHDR unidentified-BCs were 2/298 (0.65%), diagnosed during screening assessment in the contralateral breast of the recalled suspicious lesion, while AIS unidentified-BCs were 4/298 (1,34%:range 5.97-45.47%; median 8.75%). In MS-G3, a BC was diagnosed in 84.34% (140/166). So far, three interval BC has been recorded, all within the MS-G2 (range 13.84-49.58%; median 46.63%); 2/3 (66.67%) were correctly identified by AIS but ignored by BHDR. The different values of RR, DR, NPV, PPV, Sn, and Sp in AIS-BHDR in MS-G1, MS-G2 and MS-G3 were, respectively: RR 0.32% (80/24856), 5.84% (772/13225) and 90.36% (150/166); DR 0.00‰ (0/24856), 11.95‰ (158/13225) and 843.37‰ (140/166); PPV 0.00%, 20.47% and 93.33%; NPV 100%, 99.96% and 100%; Sn 0.00%, 96.93% and 100%; Sp 99.68%, 95.30% and 61.54%.
**Conclusion:** The high NPV (100%) of the MS-G1 could allow these cases to be sent directly to the next two-year screening round or selected for AIS-assisted single human reading, thus reducing the workload without losing BC.
**Limitations:** The study includes single-institution data, which makes it impossible to generalize the results.
**Ethics committee approval:** No information provided by the submitter.
**Funding for this study:** No funding for this study was received.
**Author Disclosures:**
Giorgia Soppelsa: Nothing to disclose
Eleonora Di Gaetano: Nothing to disclose
Elisa Cattarin: Nothing to disclose
Giovanni Morana: Nothing to disclose
Liat Loeffelstiel: Nothing to disclose
Claudia Maria Weiss: Nothing to disclose
Roberta Cerniato: Nothing to disclose

### RPS 302-4

**Artificial intelligence (AI) in Breast Cancer Screening Programs in Cordoba (AITIC): introduction and first interim results**

*E. Elías Cabot*[1], S. Romero Martin[1], J. L. Raya Povedano[1], A. Gubern Mérida[2], A-K. Brehl[2], M. Álvarez Benito[1]; [1]Cordoba/ES, [2]Nijmegen/NL
*(eeliascabot@gmail.com)*

**Purpose:** The study aimed to prospectively evaluate AI for safe workload reduction by applying double-reading only to high-risk cases in screening with 2D and 3D mammography.
**Methods or Backround:** Participants in a breast cancer screening programme in Córdoba (women, age 50-69) are included upon signed informed consent. Two reading strategies are independently applied to all exams: Double-blind and non-consensual reading of all exams (control arm) and an AI-based triaging (AI arm), where an AI system (Transpara, ScreenPoint Medical) evaluates the cancer risk of all exams. Cases identified by AI as low risk are automatically assessed as negative, while cases with intermediate to elevated risk are double-read with AI support. The operating point of the AI system was selected to identify approximately 70% of the exams with the lowest cancer risk. Readers are randomly assigned to each reading and blinded to other reading outcomes. We hypothesize that an AI-based screening workflow allows for substantial workload reduction without presenting inferiority in terms of cancer detection (CDR). (Clinicaltrials.gov NCT04949776).
**Results or Findings:** Between March and July 2022, 4852 women participated. In total, 42 cancers were detected. AI-based triaging led to equivalent screening performance with non-inferior CDR compared to double-reading of all cases (7.8/1000 (38/42 cancers) vs 7.2/1000 (35/42), p=1) and similar recall rates (5.7% vs 6.1%, p=0.5). In the AI arm, 3276 exams were automatically assessed as negative, resulting in 67% workload reduction.
**Conclusion:** AI-based triaging safely reduces workload and increases the overall effectiveness in breast cancer screening.
**Limitations:** The results are preliminary. Final results are expected after the inclusion of 27000 participants by March 2024.
**Ethics committee approval:** The study was approved by the Córdoba Ethics committee on March 30th 2021.
**Funding for this study:** No funding was received for this study.
**Author Disclosures:**
Sara Romero Martin: Nothing to disclose
Marina Álvarez Benito: Nothing to disclose
Esperanza Elías Cabot: Nothing to disclose
Albert Gubern Mérida: Employee: screen point medical group employee
Employee: screen poitn medical group employee
Anne-Kathrin Brehl: Employee: screen poitn medical group employee
Employee: screen point medical group employee
Jose Luis Raya Povedano: Nothing to disclose

### RPS 302-5

**A survey on Norwegian radiologists' attitudes towards artificial intelligence in mammography screening**

*M. A. Martiniussen*[1], S. Sagstad[2], Å. S. Holen[2], A. S. Larsen[1], J. E. Thy[2], S. Hofvind[2]; [1]Graalum/NO, [2]Oslo/NO
*(maritkarl@gmail.com)*

**Purpose:** This study aimed to investigate radiologists' attitudes towards the use of artificial intelligence (AI) in the screen-reading of mammograms.
**Methods or Backround:** In this cross-sectional study, we developed an anonymous electronic questionnaire and invited all radiologists working in BreastScreen Norway during 2021-2022 (n=98) to take part in the survey. AI was defined as computer programs trained to interpret mammograms based on deep learning. The questionnaire included background information and questions regarding the importance of knowledge about AI. Data were collected digitally and analyzed using descriptive statistics.
**Results or Findings:** The overall response rate was 61.2% (60/98), 66.7% (40/60) were women, and 33.3% (20/60) were men. Forty per cent (24/60) reported less than 10 years of experience with screen-reading, and 60% (36/60) reported 10 years or more. Eight out of ten radiologists reported no experience with AI reading radiological images or had only seen AI interpretation software demonstrated. Seventy-five per cent (45/60) of the respondents considered it important to have knowledge about the population used in training the AI systems and how AI systems reached their results. The majority of the respondents (88.0%, 51/58) answered that knowledge of the legal framework that would apply in case of discrepancy between AI systems and the radiologists is important, and 65% (39/60) answered that knowledge of how AI systems receive legal approvals is important. Thirty-nine per cent (23/59) considered it important to have knowledge of how AI systems store and handle data, while 43.3% (25/59) were neutral, and 15.1% (9/59) did not consider that information important.
**Conclusion:** Efforts to increase the radiologists' knowledge of technical and legal aspects of AI will probably be important in order to successfully implement and use AI in interpreting screening mammograms.
**Limitations:** Not applicable.

**Ethics committee approval:** No information provided by the submitter.
**Funding for this study:** Not applicable.
**Author Disclosures:**
Åsne Sørlien Holen: Nothing to disclose
Marit Almenning Martiniussen: Nothing to disclose
Silje Sagstad: Nothing to disclose
Solveig Hofvind: Nothing to disclose
Anne Sofie Larsen: Nothing to disclose
Jonas E. Thy: Nothing to disclose

### RPS 302-6

**Artificial intelligence (AI) improves breast cancer screening performance for both digital mammography and digital breast tomosynthesis**

*E. Elías Cabot*[1], S. Romero Martin[1], J. L. Raya Povedano[1], A. Gubern Mérida[2], A-K. Brehl[2], M. Álvarez Benito[1]; [1]Cordoba/ES, [2]Nijmegen/NL
*(eeliascabot@gmail.com)*

**Purpose:** The study aimed to evaluate the added value of AI for digital mammography (DM) and digital breast tomosynthesis (DBT) reading as support for human double reading in a breast cancer screening program.
**Methods or Backround:** We included all DM or DBT screening examinations (Selenia and Selenia Dimensions, Hologic) between March 2021 and March 2022. These were double-read (without consensus) by radiologists with concurrent AI support. We compared the screening performance in this period with the same 12 months period one year earlier, prior to the implementation of AI in clinical practice. The AI system (Transpara, ScreenPoint Medical) used in this study categorizes each exam representing the probability of cancer, and highlights suspicious areas. We compared the overall cancer detection rate (CDR), recall rate (RR) and positive predictive value (PPV) during the study period for DM and DBT separately. Chi2 test was used.
**Results or Findings:** We identified 12000 screening examinations (DM=5028, DBT=6972) that were double-read with concurrent AI support. Overall, 105 cancers were detected (DM=44, DBT=61). Before the introduction of AI, 92 cancers were detected (DM=37, DBT=55) in 16555 screening examinations (DM=7229 and DBT=9326). For DM, the CDR increased by 71% from 5.1 to 8.7/1000 ($p = 0.014$), with a concomitant decrease in RR from 8.3 to 6.7% ($p< 0.001$) and an increase in PPV from 6.1 to 13% ($p< 0.001$). For DBT, the CDR increased by 50% from 5.8 to 8.7/1000 ($p = 0.032$) with an increase in RR from 4.5% to 5.7% ($p < 0.001$) and in PPV from 13.1% to 15.3% ($p = 0.37$).
**Conclusion:** AI used concurrently in screening practice contributes to an increase in CDR and PPV for both DM and DBT. A larger improvement is observed for DM.
**Limitations:** A single institution.
**Ethics committee approval:** The study was approved by the Córdoba Ethics committe- March 30th 2021.
**Funding for this study:** No funding was received for this study.
**Author Disclosures:**
Sara Romero Martin: Nothing to disclose
Marina Álvarez Benito: Nothing to disclose
Esperanza Elías Cabot: Nothing to disclose
Albert Gubern Mérida: Employee: screen point medical group employee
Employee: screen poitn medical group employee
Anne-Kathrin Brehl: Employee: screen point medical group employee
Employee: screen poitn medical group employee
Jose Luis Raya Povedano: Nothing to disclose

### RPS 302-7

**Automated segmentation and classification of mammographic microcalcifications using a deep learning algorithm mimicking human decision-making**

*J. Kajüter*[1], C. Ruppert[2], P. Hejduk[2], K. Borkowski[2], J. Cyriac[1], S. Potthast[3], N. Schmidt[1]; [1]Basle/CH, [2]Zurich/CH, [3]Schlieren/CH
*(jonas.kajueter@usb.ch)*

**Purpose:** Microcalcifications are a typical mammography finding of breast cancer; however, they can also be associated with benign changes. The differentiation between suspicious and benign microcalcifications is a tedious task requiring expertise, experience, and routine. An AI algorithm mimicking human decision-making could be useful to differentiate between benign and suspicious morphology of microcalcifications. The aim of this study is to train and evaluate a multiclass U-Net for automated microcalcification segmentation and BI-RADS-based classification.
**Methods or Backround:** In our study, we include 1288 digital mammograms and tomosyntheses from the University Hospital Basel with a corresponding radiology report stating suspicious microcalcifications. An experienced radiologist, segmenting and classifying microcalcifications as benign or suspicious, manually annotated the dataset. A multiclass U-Net developed by the AI software company b-rayZ (b-rayZ AG, Wagistrasse 21, 8952 Schlieren, Switzerland) was trained with 904 images, validated with 258 images, and tested with 126 images, and predicts segmented masks for benign and suspicious calcifications.
**Results or Findings:** The results of the test dataset were evaluated by counting the network´s output and comparison to the human reading by an experienced radiologist, leading to the calculation of confusion matrices. An overall agreement of 76% was found between the network´s output and human reading for both benign and suspicious calcifications.
**Conclusion:** Our results demonstrate a robust automated multiclass U-Net-based segmentation and categorization of mammographic microcalcifications into benign and suspicious with an overall agreement of 76% between network and human reader, which may improve clinical workflow, quality and accuracy in reading mammograms.
**Limitations:** So far, neither an external validation with mammograms from other centres nor an interreader agreement between different human readers has been performed. Both are planned next steps to complete the study.
**Ethics committee approval:** The study was approved by Ethikkommission Nordwest- und Zentralschweiz
Projekt-ID 2021-01472
**Funding for this study:** No external funding was received.
**Author Disclosures:**
Noemi Schmidt: Nothing to disclose
Karol Borkowski: Nothing to disclose
Carlotta Ruppert: Nothing to disclose
Silke Potthast: Nothing to disclose
Patryk Hejduk: Nothing to disclose
Joshy Cyriac: Nothing to disclose
Jonas Kajüter: Nothing to disclose

### RPS 302-8

**Unsupervised contrastive domain adaptation for breast MRI segmentation**

*S. Kuang*[1], H. C. Woodruff[1], R. Granzier[1], T. van Nijnatten[1], M. Lobbes[1], M. Smidt[1], S. Mehrkanoon[2], P. Lambin[1]; [1]Maastricht/NL, [2]Utrecht/NL
*(sheng.kuang@maastrichtuniversity.nl)*

**Purpose:** A common obstacle to translating the achievements of AI solutions to clinical applications is domain shift, which arises from different vendors, acquisition, and reconstruction protocols, and biological heterogeneity. We aimed to overcome this problem for breast MRI by developing an unsupervised contrastive domain adaptation (UCDA) method that works with very small training datasets.
**Methods or Backround:** Two datasets were retrospectively collected: The source domain dataset comprised T1W and T2W breast MRI sequences with expert segmentation of the whole breast from 11 healthy volunteers. The target domain dataset comprised DCE-T1W and T2W breast MRI sequences from 134 patients histologically confirmed invasive breast cancer patients. We set up a bidirectional domain adaptation experiment: from source T2W image to target DCE-T1W image (T2W-to-T1W) and from source T1W image to target T2W image (T1W-to-T2W). Our proposed method incorporated a self-training framework with contrastive learning and used DeepLab-v3+ as the segmentation backbone. We further extended the contrastive loss by incorporating multi-level contrasts to integrate more breast semantic information. Dice similarity coefficients (DSCs) between expert and AI-generated contours were used to quantify the ability of our method to shift to a new domain and compared to source-only models (no domain adaptation) and supervised models (trained with expert annotations of the target domain).
**Results or Findings:** The proposed method achieved DSC of 0.89 and 0.84 in T2W-to-T1W and T1W-to-T2W, respectively, significantly outperforming the source-only models (T2W-to-T1W: 0.69, T1W-to-T2W: 0.74) and comparable to supervised models (T2W-to-T1W: 0.94, T1W-to-T2W: 0.93).
**Conclusion:** Our method can be successfully applied to improve the performance in cross-domain breast MRI segmentation. This approach may be applicable to other domain adaptation tasks, paving the road to more widely applicable AI tools.
**Limitations:** The performance in cross-domain breast lesion segmentation remains unclear.
**Ethics committee approval:** No information provided by the submitter.
**Funding for this study:** Not applicable
**Author Disclosures:**
Marjolein Smidt: Nothing to disclose
Sheng Kuang: Nothing to disclose
Siamak Mehrkanoon: Nothing to disclose
Thiemo van Nijnatten: Nothing to disclose
Marc Lobbes: Nothing to disclose
Henry Christian Woodruff: Nothing to disclose
Renée Granzier: Nothing to disclose
Philippe Lambin: Nothing to disclose

11:30-12:30 Research Stage 2

### Research Presentation Session: Interventional Radiology

### RPS 309

### Interventional radiology: abdominal and vascular

**Moderator**
M. C. Calcagno; Catania/IT
*(mc.calcagno@gmail.com)*

### RPS 309-2

**Endovascular embolisation as a treatment of patients with pelvic arteriovenous malformations**

*M. Szmygin*, K. K. Pyra, T. Jargiełło; Lublin/PL
*(mszmygin@gmail.com)*

**Purpose:** The study aimed to report our experience with pelvic AVMs treated with endovascular means and present a literature overview.
**Methods or Backround:** Pelvic arteriovenous malformations (pAVM) result from abnormal direct communication between an artery and a vein without an intervening capillary bed. They are either congenital or acquired (occurring after surgery or trauma) vascular abnormalities that present with hematuria, pelvic pain and varices but may be life-threatening in case of a rupture. A variety of therapies have been used to treat pAVM, but endovascular embolization has been suggested as the primary therapeutic modality.
**Results or Findings:** We describe four patients (3 female and one male) diagnosed with pAVM. In 2 cases, the malformation was congenital, and in the other two acquired (occurred after pregnancy). From this group, two patients were treated with transarterial embolization and 2 with percutaneous direct puncture embolization. In all cases, both satisfactory embolization and significant clinical improvement were achieved. No peri-procedural complications were observed. The long-term follow-up did not show a recurrence of pAVM in any case.
**Conclusion:** Hemodynamics of pelvic arteriovenous malformations are variable, and a thorough understanding of the vessel anatomy is crucial in planning and choosing proper treatment. Both transarterial embolization and percutaneous direct puncture embolization appear to be feasible, effective and safe methods and should be therefore considered as first-line treatment modalities for patients with pAVM.
**Limitations:** The study included a limited number of patients
**Ethics committee approval:** No information provided by the submitter.
**Funding for this study:** No funding was received for this study.
**Author Disclosures:**
Maciej Szmygin: Nothing to disclose
Tomasz Jargiełło: Nothing to disclose
Krzysztof K. Pyra: Nothing to disclose

### RPS 309-3

**Effectiveness of ultrasound-guided percutaneous transhepatic biliary drainage to reduce radiation exposure: a single-centre experience**

*I. C. Nam*[1], S. Park[2], J. Won[3], H. J. Baek[2], K. H. Ryu[4], D. R. Kim[1]; [1]Jeju/KR, [2]Changwon/KR, [3]Jinju/KR, [4]Busan/KR

**Purpose:** This study aimed to evaluate the effectiveness of US-guided PTBD, focusing on radiation exposure according to intrahepatic duct (IHD) dilatation degree, differences between right-and left-sided approaches and differences between benign and malignant biliary stenosis/obstruction.
**Methods or Backround:** We evaluated technical success, clinical success, procedural data (the number of liver capsule punctures, procedural time, fluoroscopy time and radiation dose), and procedure-related complications. During the study period, a total of 123 patients with biliary stenosis/obstruction or bile leakage were initially eligible. We excluded 76 patients treated with only ERCP or initially treated with ERCP followed underwent PTBD insertion. Finally, a total of 50 procedures were performed on 47 patients.
**Results or Findings:** The technical success rate was 100%, clinical success was 94%, and the complication rate was 10%. Fluoroscopy time and the reported radiation dose were significantly lower in patients with dilated bile ducts than in those with non-dilated bile ducts when biliary puncture under US guidance was performed initially. However, even in patients with non-dilated bile ducts undergoing initial trials of biliary puncture under US guidance, the fluoroscopy time and the reported radiation dose were low, based on current studies. No statistically significant differences were observed in terms of technical and dosimetry results according to right-sided and left-sided procedures and benign and malignant biliary stenosis/obstruction.
**Conclusion:** Thus, US-guided PTBD was found to be a safe and effective technique that significantly reduced fluoroscopy time and radiation doses.
**Limitations:** First, this study was retrospectively designed, which suggests selection bias. Second, the sample size was small. Third, there was no direct comparison in terms of fluoroscopy time and radiation dose between fluoroscopy-guided PTBD and US-guided PTBD because all biliary punctures were routinely performed under US guidance initially.
**Ethics committee approval:** This study was approved by the Institutional Review Board of Gyeongsang National University Changwon Hospital approved the study (No.: GNUCH 2021-07-037).
**Funding for this study:** No funding was received for this study.
**Author Disclosures:**
In Chul Nam: Nothing to disclose
Jungho Won: Nothing to disclose
SungEun Park: Nothing to disclose
Doo Ri Kim: Nothing to disclose
Kyeong Hwa Ryu: Nothing to disclose
Hye Jin Baek: Nothing to disclose

### RPS 309-4

**Value of TIPS reduction for the treatment of TIPS-related hepatic encephalopathy and liver dysfunction**

*R. Plajer*, J. M. Ludwig, M. Opitz, M. Forsting, Y. Li , B. M. Schaarschmidt, J. Theysohn; Essen/DE

**Purpose:** In cirrhotic liver patients, the transjugular intrahepatic portosystemic shunt (TIPS) procedure remains a mainstay treatment option. However, post-TIPS hepatic encephalopathy (HE) is a common and severe complication in which shunt reduction with a reduction stent typically formed in an hour-glass shaped configuration has become a viable treatment option. The aim of this study was to investigate the clinical benefit and liver function changes of patients undergoing TIPS reduction.
**Methods or Backround:** In this retrospective study, 14 patients (median age: 54,4 years, five men) who underwent TIPS reduction between 12/2012-09/2021 were analyzed. Indications were HE (n=12) and worsening liver function (n=2). The reduction was performed after a median of 9.5 months (95%CI:1.4-38.2 months) after the initial TIPS. Child-Pugh Score (CP) A/B/C was 5(36%)/5(36%)/4 (28%). Covered stents were used in 12/14 (86%) reductions. Mann-Whitney-U and ANOVA testing were used for statistical analysis.
**Results or Findings:** The portosystemic pressure gradient increased from a median of 8mmHg (range:0-12) to 16mmHg (range:5-17) (p=0.0003). Post-reduction ammonia laboratory value decreased from a median of 148 µg/dl [95%CI:103-251] to 79 µg/dl [62-171] (p=0.03). HE ameliorated in 7 of 14 patients (50%) (p=0.07). Recurrent ascites occurred in 6/14 patients overall with 3/4 CP C patients (p=0.03) and variceal haemorrhage in 3/14 patients with 1/4 CP C patients (p=0.25). Liver function parameters (median bilirubin, albumin and INR) were 1.3 mg/dl [0.7-3.2], 3.3g/dl [2.7-3.6] and 1.2 [1.1-1.3] showed no statistical changes after reduction (1.35 [0.8-4.7] (p=0.4), 3.2 [2.3-4.0] (p=0.73) and 1.2 [1.1-1.5] (p=0.97).
**Conclusion:** TIPS reduction is a valuable treatment option to reduce hepatic encephalopathy and ammonia serum levels without aggravating liver function.
**Limitations:** The study included a small sample size and is a single-centre study.
**Ethics committee approval:** No information provided by the submitter.
**Funding for this study:** Not applicable.
**Author Disclosures:**
Michael Forsting: Nothing to disclose
Jens Theysohn: Nothing to disclose
Yan Li : Nothing to disclose
Benedikt Michael Schaarschmidt: Nothing to disclose
Johannes Maximilian Ludwig: Nothing to disclose
Richard Plajer: Nothing to disclose
Marcel Opitz: Nothing to disclose

### RPS 309-5

**Resuscitative endovascular balloon occlusion of the aorta (REBOA) for controlling life-threatening postpartum haemorrhage: 14 years of experience in Norway**

*K. H. Stensaeth*[1], M. I. S. Carlsen[1], Y. Haig[2], T. S. Lovvik[1], J. R. Brede[1], O. Uleberg[1], E. Sovik[1]; [1]Trondheim/NO, [2]Oslo/NO
*(knutsten@gmail.com)*

**Purpose:** Postpartum haemorrhage (PPH) remains a global health problem, responsible for 8% of maternal deaths. PPH is often dramatic and may be life-threatening. Resuscitative Endovascular Balloon Occlusion of the Aorta (REBOA) has been established as a novel treatment for hemorrhagic shock. We present our second series (2016-2021) of REBOA for controlling life-threatening PPH.
**Methods or Backround:** In 2008 an 'aortic occlusion kit' was assembled in two Norwegian university hospitals. In cases of life-threatening PPH, the on-call interventional radiologist was to be contacted. Due to our experiences from the first series (2008-2015), the kit was revised in 2016, and a balloon size of 15 mm became standard. Insertion and placing of the aortic balloon have been done without fluoroscopy since 2008. Patient characteristics were noted from the medical records.
**Results or Findings:** This second series includes 29 patients treated with REBOA for life-threatening PPH in 2016–2021. The majority was due to uterus atony. No procedure-related complications were observed. Compared to the first period (2008-2015), there was a significant reduction of hysterectomies performed (47% versus 24%), the amount of total bleeding (5.2 versus 3.7 litres), units of blood transfusions (SAGMAN) used (9 versus 6), as well as uterine artery embolization performed (17 versus 3). All hysterectomies (n=7) in this latest series were performed due to known pathologic uterus conditions.
**Conclusion:** Our 14 years of experience in Norway have shown that performing REBOA for life-threatening PPH is a safe technique. One can rapidly achieve hemodynamic stability and, importantly, preserve the reproductive function of the patient. A close multidisciplinary collaboration between gynaecologists, anesthesiologists, and interventional radiologists, is essential to achieve the best possible outcome.
**Limitations:** The study included a small number of eligible patients and is a retrospective study.
**Ethics committee approval:** No information provided by the submitter.
**Funding for this study:** No funding was received for this study.
**Author Disclosures:**
Knut Haakon Stensaeth: Nothing to disclose
Oddvar Uleberg: Nothing to disclose
Jostein Rodseth Brede: Nothing to disclose
Marte Irene Skille Carlsen: Nothing to disclose
Edmund Sovik: Nothing to disclose
Tone Shetelig Lovvik: Nothing to disclose
Ylva Haig: Nothing to disclose

### RPS 309-6

**Spontaneous abdominal wall hematoma treated with percutaneous transarterial embolization, diagnostic findings, procedural outcome and efficacy: a multicentric study.**

*F. Tiralongo*[1], S. Di Pietro[1], F. Vacirca[1], P. Lucatelli[2], A. Contegiacomo[2], C. Mosconi[3], M. Venturini[4], F. Giurazza[5], A. Basile[1]; [1]Catania/IT, [2]Rome/IT, [3]Bologna/IT, [4]Segrate/IT, [5]Naples/IT
*(tiralongofrancesco91@hotmail.it)*

**Purpose:** Endovascular treatment of abdominal wall hematomas (AWHs) has been increasingly used when conservative treatments were not sufficiently effective, and it is often preferred to surgical interventions. The aim of our study was to evaluate the safety and technical and clinical success of percutaneous transarterial treatment of AWH and to evaluate the efficacy of blind embolisation compared to targeted embolisation, including also a group of patients affected by COVID-19.
**Methods or Backround:** This retrospective, multicentric study, included 112 patients (59men and 53females) with spontaneous AWH who underwent digital subtraction angiography (DSA) and embolization, focusing on the presence of signs of bleeding at pre-procedural CT-Angiography (CTA) and at DSA. Furthermore, we divided patients into two groups depending on blind or targeted embolization approaches. The patients were also divided into COVID-19 and non-COVID-19 groups.
**Results or Findings:** The mean age of the study population was 69 ± 16 years. CTA revealed signs of active bleeding in 99 patients (88%). DSA showed signs of active bleeding in 88 patients (79%). In 21 patients (19%), blind embolization was performed. The overall technical success rate was 99%. Clinical success was achieved in 96 patients (86%), while 16 patients (14%) rebled within 96 h. One patient reported a major peri-procedural complication. The comparison between blind and targeted embolization showed no statistically significant differences for characteristics of groups and for technical success rates (99% and 100%, respectively, –p = 0.42). The technical success was 86% in both groups. No statistical differences were also found between the COVID and non-COVID groups.
**Conclusion:** Our multicentric study confirms that transarterial embolization is a safe and effective option for the treatment of spontaneous AWHs, and it suggests that the efficacy and safety of blind embolization are comparable to non-blind.
**Limitations:** Not applicable.
**Ethics committee approval:** No information provided by the submitter.
**Funding for this study:** Not applicable.
**Author Disclosures:**
Andrea Contegiacomo: Nothing to disclose
Stefano Di Pietro: Nothing to disclose
Massimo Venturini: Nothing to disclose
Francesco Giurazza: Nothing to disclose
Cristina Mosconi: Nothing to disclose
Antonio Basile: Nothing to disclose
Pierleone Lucatelli: Nothing to disclose
Francesco Vacirca: Nothing to disclose
Francesco Tiralongo: Nothing to disclose

### RPS 309-7

**Interventional radiological management of arterial complications after paediatric liver transplant surgery: experience from a single tertiary centre**

*C. Capodaglio*[1], P. Marra[1], L. Dulcetta[1], F. S. Carbone[1], R. Muglia[1], P. A. Bonaffini[1], G. Muscogiuri[1], D. Ippolito[2], S. Sironi[1]; [1]Bergamo/IT, [2]Milan/IT
*(c.capodaglio@campus.unimib.it)*

**Purpose:** Paediatric liver transplant is burdened by a high rate of arterial complications due to the technical difficulties of performing vascular anastomoses on small vessels. Interventional radiological (IR) management is not yet standardized. We report the outcomes in a cohort of paediatric liver transplant patients with hepatic artery (HA) complications treated by IR
**Methods or Backround:** From December 2019 to April 2022, consecutive paediatric patients with deceased-donor liver transplants who underwent IR procedures due to suspected HA complications were prospectively collected and reviewed. Clinical and procedural data were analyzed, with a focus on: type of complication (pseudoaneurysm, thrombosis, stenosis), time of development (acute=< 15 days, subacute=15-30days, late=> 30 days), technique (angioplasty, stenting), technical success and clinical outcome.
**Results or Findings:** Sixteen paediatric patients (seven male; median age three years) underwent 21 right transfemoral hepatic arteriography procedures for the treatment of six thromboses, five acute or subacute stenosis, three acute pseudoaneurysms with stenosis, and two late stenoses. Three patients underwent more than one intervention. Primary technical success was obtained in 16/21 (76,2%) procedures, secondary technical success in one (overall technical success 17/21, 80,9%). Angioplasty was technically successful in 3 cases of stenosis; in all other treatments, arterial stenting was required using coronary devices in all but one case. Clinical outcome was good for 13/16 (81,2%) patients with patent HA at a median follow-up of eight months. Two patients underwent retransplantation. Two patients died of multiorgan failure: one complicated by hemorrhagic shock due to arterial rupture and one complicated by HA stent thrombosis and liver necrosis.
**Conclusion:** In paediatric patients who present HA complications after liver transplant, IR management often provides good clinical outcomes. In a cohort of predominantly acute and subacute conditions, arterial stenting was necessary in most cases, requiring coronary devices.
**Limitations:** Not applicable.
**Ethics committee approval:** No information provided by the submitter.
**Funding for this study:** Not applicable.
**Author Disclosures:**
Sandro Sironi: Nothing to disclose
Carlo Capodaglio: Nothing to disclose
Paolo Marra: Nothing to disclose
Pietro Andrea Bonaffini: Nothing to disclose
Francesco Saverio Carbone: Nothing to disclose
Davide Ippolito: Nothing to disclose
Riccardo Muglia: Nothing to disclose
Giuseppe Muscogiuri: Nothing to disclose
Ludovico Dulcetta: Nothing to disclose

**11:30-12:30** **Research Stage 3**

### Research Presentation Session: Neuro

### RPS 311

### New approaches and techniques in neuroradiology

**Moderator**
A. Krainik; Grenoble/FR

### RPS 311-2

**Intrinsic CSF outflow declines with age by spin-labelling MRI**

V. Malis[1], W. Bae[1], A. Yamamoto[2], M. McDonald[1], L. McEvoy[1], *M. Miyazaki*[1]; [1]San Diego/US, [2]Tokyo/JP
*(mimiyazaki@health.ucsd.edu)*

**Purpose:** Clearance of CSF outflow is important for the removal of toxins from the brain to maintain healthy brain and prevent neurogenerative diseases. However, the egress pathway and quantitative measures of intrinsic CSF drainage remain unknown. In general, glymphatic studies were performed in small animals with invasive tracers [1,2]. The purpose of this study is to develop non-contrast MRI techniques to depict the intrinsic CSF outflow, measure their quantitative metrics, and investigate an age effect in the human brain.
**Methods or Backround:** All MR imaging data were obtained with a clinical 3-T MR imager in healthy subjects without neurodegenerative diseases (10 males and 6 females; mean age, 47.6 ± 18.9 years; range, 19-71 years) who had given written informed consent. For 4D time-resolved MRI, both labelled and control were acquired and subtracted for quantitative analysis and visualisation of intrinsic CSF outflow [3].
**Results or Findings:** The subtracted colour maps of contiguous slices show marked CSF moved toward the SSS. Using spin-labelling MRI, we revealed that tagged CSF from PSD was purged into the SSS. Quantitative CSF outflow metrics show a decline of CSF outflow at the SSS in the human brains of older individuals. Furthermore, we observed a sharp decline of the outflow after over 60 years, with a significant correlation between young (19 to 59 years).
**Conclusion:** Our study demonstrates unambiguous visualisation of intrinsic CSF outflow at the SSS, and quantitative measures indicate a decline in CSF outflow metrics with age.
**Limitations:** Our study consists of only healthy individuals without known neurodegenerative disease, cardiovascular disease, and pulmonary disease.
**Ethics committee approval:** The study was approved by the Institutional Review Board of IRB (200335) of University of California, San Diego.
**Funding for this study:** This work was supported by an NIH grant RF1AG076692 (M.M.) and a grant by Canon Medical Systems, Japan (35938).
**Author Disclosures:**
Vadim Malis: Nothing to disclose
Linda McEvoy: Nothing to disclose
Asako Yamamoto: Nothing to disclose
Won Bae: Nothing to disclose
Marin McDonald: Nothing to disclose
Mitsue Miyazaki: Nothing to disclose

### RPS 311-3

**Tackling dataset deficiencies in medical AI: creating a multi-vendor, multi-site MRI dataset of brain metastases**
*Q. D. Strotzer*, T. Wagner, P. Angstwurm, K. Hense, J. Dinkel, E. Noeva, C. Fellner, A. Schicho, C. Wendl; Regensburg/DE
*(quirin.strotzer@web.de)*

**Purpose:** Most research on artificial intelligence in medicine is limited by the training data and methodological flaws (e.g., data leakage and bias). We aimed to create a comprehensive annotated dataset of MRI scans and accompanying clinical data of patients suffering from brain metastases to be used for further analysis, e.g., with methods from the field of artificial intelligence.
**Methods or Backround:** This bi-centre project included consecutive patients between 2004 and 2021 who presented for the workup of brain metastases. The pre-interventional routine MRI sequences T1, contrast-enhanced T1, T2, and FLAIR, and the histologically determined primary tumour had to be available. External and internal imaging studies were included. Where available, a post-contrast T1 3D magnetization-prepared rapid gradient-echo acquisition was also included. A preprocessing pipeline using open-source Python libraries included co-registration, normalisation, and automated tumour segmentation using a U-shaped convolutional neural network. Segmentations were manually refined where needed.
**Results or Findings:** Imaging studies of 248 patients (118 females) were included, resulting in 711 segmented brain metastases. The mean age was 60.67y (standard deviation 12.29). The most prevalent primary tumours were adenocarcinoma of the lung (number of metastases=183), melanoma (n=130), breast cancer (n=96), and colorectal cancer (n=54). Imaging was collected from fifteen different MRI scanner models (9.7% of studies at 3.0 Tesla, 89.0% at 1.5T, and 1.3% at 1.0T).
**Conclusion:** We present an annotated multi-site, multi-vendor dataset of pre-therapeutic MRI scans of brain metastases. This dataset represents the heterogeneous reality better than the datasets of most published studies related to artificial intelligence in medicine, and it can easily be used for further analysis.
**Limitations:** To better resemble the real-world scenario, datasets should cover primary brain tumours like astrocytoma and meningioma and tumour mimics like demyelinating disease, haemorrhage, or abscess.
**Ethics committee approval:** This study was approved by University of Regensburg ethics committee.
**Funding for this study:** No funding was received for this study.
**Author Disclosures:**
Andreas Schicho: Nothing to disclose
Thomas Wagner: Nothing to disclose
Claudia Fellner: Nothing to disclose
Pia Angstwurm: Nothing to disclose
Ekaterina Noeva: Nothing to disclose
Quirin D. Strotzer: Nothing to disclose
Katharina Hense: Nothing to disclose
Christina Wendl: Nothing to disclose
Johannes Dinkel: Nothing to disclose

### RPS 311-4

**Whole-brain vascular architecture mapping reveals distinct microvascular profiles for cortical and subcortical grey matter**
*A. Hohmann*, K. Zhang, C. Mooshage, J. Jende, L. T. Rotkopf, M. Bendszus, W. Wick, F. Kurz; Heidelberg/DE
*(anja.hohmann@med.uni-heidelberg.de)*

**Purpose:** Vessel architecture imaging (VAI) is a recently developed MR imaging technique that characterises changes in cerebral microvasculature in vivo; however, VAI parameter reference ranges in brain tissue are lacking, thus limiting interpretation and, eventually, application of VAI.
**Methods or Backround:** VAI is based on time-parametrised vortex curves from dynamic changes in T2 and T2* relaxation rates during contrast agent bolus administration, resulting in descriptors of the underlying vasculature, namely vessel type indicator (VTI) and vascular inflow calibre (VIC) as the signed area and the slope of the vortex curve, respectively. We scanned n=135 healthy brain hemispheres of patients with stable low-grade gliomas and no previous chemo- or radiotherapy, using a whole-brain high-resolution multiband echo-planar imaging combined spin and gradient echo sequence at 3.0 Tesla MRI (PRISMA, Siemens). MNI-atlas-based volumes of interest were extracted for cortical grey matter (cGM), supratentorial white matter (WM), caudate nucleus (CN), and putamen (Put) for both VTI and VIC.
**Results or Findings:** We found the following reference ranges across the patient collective for VTI [in s-2]: cGM -5.57±0.49, WM -2.65±0.22, CN -5.66±0.77, Put -3.68±0.51; and for VIC [unitless]: cGM 5.81±0.1, WM 4.25±0.1, CN 6.22±0.2, and Put 4.15±0.13. Pair-wise post-hoc comparisons revealed larger vessel calibres (VIC) and dominance of venous vessels (VTI) in cGM vs WM (p<0.001). Furthermore, both vascular parameters differed significantly between Put vs cGM and CN vs WM (both p<0.01), but not for Put vs WM and CN vs cGM (p=0.76 and p=0.99, respectively).
**Conclusion:** VAI mapping in the healthy adult brain reveals distinct (micro-)vascular profiles. The obtained parameter reference ranges may serve as a starting point for future VAI investigations of cerebrovascular pathologies.
**Limitations:** Further work will see dedicated age stratification of VAI reference ranges.
**Ethics committee approval:** Approved by the local ethics committee of the University of Heidelberg (ethics approval number: S-320/2012).
**Funding for this study:** Rahel Goitein-Straus Program of Medical Faculty Heidelberg to AH.
**Author Disclosures:**
Ke Zhang: Nothing to disclose
Johann Jende: Nothing to disclose
Felix Kurz: Nothing to disclose
Anja Hohmann: Nothing to disclose
Lukas Thomas Rotkopf: Nothing to disclose
Wolfgang Wick: Nothing to disclose
Martin Bendszus: Nothing to disclose
Christoph Mooshage: Nothing to disclose

### RPS 311-5

**Orthostatic variation of the fourth ventricular volume & cerebellar tonsillar position on open MR in normal healthy volunteers**
*N. Syed Nasser*[1], A. Suman[1], G. Nanda[2], V. K. Venugopal[1], V. Mahajan[1], H. Mahajan[1]; [1]Delhi/IN, [2]New Delhi/IN
*(nisha.nasser@caring-research.com)*

**Purpose:** To measure the orthostatic variation in cerebellar tonsillar position (CTP) and fourth ventricular volume between supine and upright scanning positions in normal healthy volunteers.
**Methods or Backround:** A prospective study was conducted among 42 normal healthy volunteers using sagittal T1-w images and 3D T1-w volumetric acquisition on a 0.25T G-scan Brio MRI in both supine and standing postures in 15 minutes. A neuroradiologist measured the vertical distance (in mm) from the tip of the cerebellar tonsils to a line drawn between the anterior and posterior margins of the foramen magnum (McRae line-BO line) in the midsagittal plane. A CTP above the BO line was assigned a positive value (no tonsillar descent), and below the BO line was assigned a negative value (tonsillar descent) on the BO line (zero). The fourth ventricular volume was measured (in mm3) using a 3D Slicer.
**Results or Findings:** Tonsillar descent was present in six of the 42 subjects in supine and eight subjects in an upright position. Paired t-tests revealed no significant change in fourth ventricular volume in the upright position (M=1214.088; SD=491.58) and supine position (M=1210.67; SD=422.62); t(41)=0.078, p=0.938). Although the mean CTP was 0.33mm lower in the upright (M=0.837; SD=2.82) than in the supine position (M=1.17; SD=2.64), this difference was not statistically significant (p=0.054). Bland-Altman difference plots were generated to assess the agreement between the volume measurements and the CTP in both positions. The correlation coefficient, r, of agreement between the methods was 0.17 (95% CI: 2.471–1.801; p=0.280) for the distance between the CTP and BO line, 0.25 (95% CI:559.99–553.163; p=0.104) for fourth ventricular volume. The tests were not statistically significant, and there was no proportional bias.

**Conclusion:** The CTP relative to the BO line and fourth ventricular volume does not change with position.
**Limitations:** Gender-bias.
**Ethics committee approval:** This study was approved by IRB.
**Funding for this study:** No funding was received for this study.
**Author Disclosures:**
Nisha Syed Nasser: Employee: CARPL.ai
Harsh Mahajan: Other: Research collaboration with Lunit.ai Other: Research collaboration with Predible Health Other: Research collaboration with Quibim Other: Research collaboration with Oxipit.ai Other: Research collaboration with Subtle Medical Other: Director at Mahajan Imaging Other: Research collaboration with Qure.ai Other: Research collaboration with General Electric Other: Research collaboration with Koninklijke Philips NV Research
Geethanjali Nanda: Employee: Mahajan Imaging and Diagnostics
Vasantha Kumar Venugopal: Other: Research collaboration with Qure.ai Other: Research collaboration with General Electric Employee: CARPL.ai Other: Research collaboration with Quibim Other: Research collaboration with Predible Health Other: Research collaboration with Subtle Medical Other: Research collaboration with Koninklijke Philips NV Research Other: Research collaboration with Oxipit.ai Other: Research collaboration with Lunit.ai
Abhishek Suman: Employee: Mahajan Imaging and Diagnostics
Vidur Mahajan: Other: Research collaboration with Lunit.ai Other: Research collaboration with Oxipit.ai CEO: CEO at CARPL.ai Other: Research collaboration with Quibim Other: Research collaboration with General Electric Other: Research collaboration with Koninklijke Philips NV Research Other: Research collaboration with Predible Health Other: Research collaboration with Qure.ai Other: Research collaboration with Subtle Medical

### RPS 311-6

**Looking beyond standard dosage in contrast-enhanced brain MRI using a pre-trained deep learning model**

*S. Pasumarthi Venkata*[1], A. Shankaranarayanan[1], G. Zaharchuk[2], R. Chamberlain[1]; [1]Menlo Park, CA/US, [2]Stanford, CA/US

**Purpose:** Gadolinium-based contrast agents (GBCAs) enhance the visibility of pathologies and are indispensable in many clinical applications. This work explores the feasibility of repurposing a pre-trained Deep Learning (DL) dose reduction model to synthesise a super-dose contrast-enhanced (CE) image with superior border delineation and lesion detection performance.
**Methods or Backround:** A DL model was trained in [1] with pre-contrast and 10%-dose images as input and 100%-dose images as the output. This pre-trained model was used with pre-contrast and 100%-dose images as input to predict the super-dose CE image. The super-dose synthesis was performed on 20 randomly selected test cases. In addition, DL-based denoising (DNE) and super-resolution (SRE) models from [2] were applied before and/or after the super-dose synthesis step in different combinations to further improve the overall image quality. Images were qualitatively assessed for improved enhancement and quantitatively evaluated using Contrast-to-Noise Ratio (CNR), Lesion-to-Brain Ratio (LBR) and Contrast-Enhancement Percentage (CEP) as described in [3]. The ROIs for quantitative assessment were manually chosen as bounding box around the largest enhancing lesion. The CNR and LBR were calculated for pre-contrast, post-contrast, and synthesised super-dose volumes, whereas the CEP was calculated (w.r.t the pre-contrast) for the post-contrast and the super-dose volumes.
**Results or Findings:** Through qualitative and quantitative assessment of the test cases, it was found that applying the DNE model before and after super-dose synthesis performed the best. The average CNR and LBR for pre-contrast/post-contrast/DNE+super-dose were 0.930/1.895/2.305 and 2.510/4.298/5.737. The average CEP(%) for post-contrast/DNE+super-dose 71.99/131.04.
**Conclusion:** Super-dose images obtained by applying the pre-trained dose-reduction DL model on pre/post-contrast images have superior border delineation and lesion detection performance.
**Limitations:** Detailed clinical evaluation is required.
**Ethics committee approval:** No information provided by the submitter.
**Funding for this study:** Grant was received from NIH R44EB027560.
**Author Disclosures:**
Srivathsa Pasumarthi Venkata: Grant Recipient: Subtle Medical Inc Shareholder: Subtle Medical Inc Employee: Subtle Medical Inc
Greg Zaharchuk: Founder: Subtle Medical Inc Shareholder: Subtle Medical Inc
Ryan Chamberlain: Employee: Subtle Medical Inc Research/Grant Support: Subtle Medical Inc
Ajit Shankaranarayanan: Employee: Subtle Medical Inc Shareholder: Subtle Medical Inc

### RPS 311-7

**A randomised, 4 x 4 cross-over study to investigate the dose-response of the new high relaxivity GBCA gadoquatrane in comparison to the standard dose of gadobutrol**

*B. M. Hofmann*, K. Riecke, S. Klein, J. Kahn, H. Posch, M. Berse, M. A. Klemens, G. Sutter, W. Ebert; Berlin/DE
*(birte.hofmann@bayer.com)*

**Purpose:** A randomised, single-blind, 4 x 4 cross-over MRI study in healthy men and women was conducted to investigate the signal enhancement properties as well as PK and safety of gadoquatrane at various dose levels.
**Methods or Backround:** Dose-response for gadoquatrane was assessed as a relative signal enhancement (RSE) and compared with the RSE after gadobutrol. Participants (N=43, age range 18-50 years) received IV injections of gadoquatrane (0.01, 0.03 and 0.06 mmol Gd/kg bw) and gadobutrol (0.1 mmol Gd/kg bw) in randomised order applying a Latin square design. RSEs were determined in 5 regions (head and neck area: carotid artery, parotid gland, sagittal sinus, sigmoidal sinus, submandibular gland) representing different degrees of vascularisation. Dose-response curves were established individually per region by linear regression at 5-, 10- and 15-minutes post-injection. Plasma Gd concentrations were determined by Inductively Coupled Plasma Mass Spectrometry at 3-time points p.i. (collection intervals: 20-60min, 2-4h and 6-8h). Safety was assessed based on adverse events (AE), clinical laboratory values and vital signs.
**Results or Findings:** For all five regions, gadoquatrane demonstrated a linear increase of RSE with dose. Using a Bayesian method of inverse regression, gadoquatrane doses with equivalent RSE as gadobutrol were obtained for each region. For each region, the equivalent dose was less than half the Gd dose of gadobutrol. Dose-normalized plasma Gd concentration for both GBCAs was almost equivalent, demonstrating similar PK. Gadoquatrane was safe and well tolerated with no apparent difference in the incidence of treatment-emergent AEs with increasing doses or compared to gadobutrol.
**Conclusion:** The dose-response study confirmed that - in line with its high relaxivity - gadoquatrane could be used at a substantially lower dose compared to marketed GBCAs.
**Limitations:** Study was conducted in healthy men and women.
**Ethics committee approval:** The protocol and the protocol amendment were reviewed and approved by the local Independent Ethics Committee (IEC) Landesamt für Gesundheit und Soziales, Berlin, Germany, before the start of the study. The conduct of this clinical study met all local legal and regulatory requirements. The study was conducted in accordance with the ethical principles that have their origin in the declaration of Helsinki and the International Council for Harmonisation (ICH) guideline E6: good clinical practice (GCP).
**Funding for this study:** Funding was received from Bayer AG
**Author Disclosures:**
Stefan Klein: Employee: Bayer AG
Johannes Kahn: Investigator: for Bayer AG Employee: Charité Universitätsmedizin
Gabriele Sutter: Employee: Bayer AG
Kai Riecke: Employee: Bayer AG
Matthias Berse: Employee: CRS Clinical Research Services Berlin GmbH Investigator: for Bayer AG
Birte Maria Hofmann: Employee: Employee of Bayer AG
Wolfgang Ebert: Employee: Bayer AG
Helena Posch: Investigator: for Bayer AG Employee: Charité Universitätsmedizin
Mark Alexander Klemens: Employee: Bayer AG

### RPS 311-8

**Initial experience using 3d quantitative synthetic MRI in the estimation of brain tissue volumes: normal & abnormal cases**

*N. Syed Nasser*, V. K. Venugopal, S. Rajan, V. Mahajan, H. Mahajan; Delhi/IN
*(nisha.nasser@caring-research.com)*

**Purpose:** To evaluate the clinical feasibility of synthetic MRI (SyMRI) in the estimation of brain tissue volumes (myelin content, Gray-matter (GM), white-matter (WM), Cerebro-spinal fluid (CSF), Intracranial Volume (ICV), Brain parenchymal fraction (BPF)) and to use age-stratified reference curves to identify the abnormal MRI studies and compare them with the conventional MRI findings in an Indian population.
**Methods or Backround:** 3D quantitative SyMRI was performed among 41 subjects of various age groups from 0-100 years using a prototype provided by GE. They underwent brain MRI using a 3T MRI scanner (Discovery MR750w; GE Healthcare; Milwaukee, USA) with a 32-channel GEM flex-medium flexible coil. Synthetic 3D T1WI, 3D T2WI, 3D FLAIR, 3D PSIR, and 3D DIR images were generated using a postprocessing synthetic MRI software (SyMRI Prototype 21Q3 SP2; Synthetic MR, Linköping, Sweden) by virtually setting TR, TE, and TI.
**Results or Findings:** The total brain tissue volumes were automatically quantified using the software. Age-stratified reference curves for the given population were obtained and compared with the normative value. For each tissue type, data were plotted against the patient's age. The automated segmentation maps for the given population were obtained with the normative reference curves for the different tissue types. Of the 41 clinically reported MRI studies, interpretation of the age-stratified reference curves and conventional images identified 16 studies to be structurally and synthetically normal (39%); 7 studies were structurally normal and synthetically abnormal (17%); 8 studies were structurally abnormal and synthetically normal (20%); 10 studies were structurally and synthetically abnormal (24%).
**Conclusion:** There is a need to create normogram values for each age group (10-year range) that can be used as a reference standard for the evaluation of neurodegenerative and demyelination disorders.
**Limitations:** No limitations were identified.
**Ethics committee approval:** This study was approved by IRB.
**Funding for this study:** No funding was received for this study.
**Author Disclosures:**
Nisha Syed Nasser: Employee: CARPL.ai
Sriram Rajan: Employee: Mahajan Imaging
Harsh Mahajan: Other: Research collaboration with Lunit.ai Other: Research collaboration with Quibim Other: Research collaboration with Qure.ai Other: Research collaboration with Predible Health Other: Director at Mahajan Imaging Other: Research collaboration with Subtle Medical Other: Research collaboration with Oxipit.ai Other: Research collaboration with Koninklijke Philips NV Research Other: Research collaboration with General Electric
Vasantha Kumar Venugopal: Other: Research collaboration with General Electric Other: Research collaboration with Subtle Medical Other: Research collaboration with Predible Health Employee: CARPL.ai Other: Research collaboration with Oxipit.ai Other: Research collaboration with Quibim Other: Research collaboration with Koninklijke Philips NV Research Other: Research collaboration with Lunit.ai Other: Research collaboration with Qure.ai
Vidur Mahajan: Other: Research collaboration with Koninklijke Philips NV Research Other: Research collaboration with Subtle Medical Other: Research collaboration with Qure.ai CEO: CARPL.ai Other: Research collaboration with Oxipit.ai Other: Research collaboration with General Electric Other: Research collaboration with Predible Health Other: Research collaboration with Lunit.ai Other: Research collaboration with Quibim

**11:30-12:30** **Research Stage 4**

### Research Presentation Session: Vascular

### RPS 315

### Pulmonary embolism and venous imaging

**Moderator**
K. Gruszczynska; Katowice/PL

### RPS 315-2

**Deep vein thrombosis at compressive ultrasound as screening to predict the onset of early pulmonary embolism in covid patients**

*C. Capodaglio*[1], D. Ippolito[1], C. Maino[1], R. Corso[1],
C. R. G. L. O. M. Talei Franzesi[1], S. Sironi[2]; [1]Monza/IT, [2]Bergamo/IT
*(c.capodaglio@campus.unimib.it)*

**Purpose:** To evaluate the usefulness of screening using compressive ultrasound (CUS) for the diagnosis of deep-vein thrombosis (DVT) in hospitalised SARS-CoV-2 patients.
**Methods or Backround:** From March 1st until May 31st, 2020, 112 COVID patients were prospectively enrolled. Evaluation with CUS of the proximal and deep veins of the arms and legs was performed within two days of admission. Lack of compressibility or direct identification of thrombus was used for diagnosing DVT. Acute pulmonary embolism (PE) was investigated at computed tomography within five days after CUS. Patients received a standard dosage of prophylactic anticoagulants (Enoxaparin 4000-6000 UI/die). Logistic binary regressions (HR= Hazard Ratio) to determine which clinical and radiological parameters were independently associated with PE.
**Results or Findings:** The incidence of DVT in our cohort was 43% (n=48). The most common district involved was the left lower limb (68.7%) compared to the right (58.3%). The upper limbs were less frequently involved (4.2% on the right and 2.1% on the left, respectively). The distal popliteal vein was the most frequently involved portion (50% right side, 45.8% left side). During the following five days, 20 patients (17.8) showed PE at CTPA, and only D-Dimer values were significantly higher than PE negative ones (p=0.030). The presence of DVT in the distal tract of the right popliteal vein (HR=2.444 95%CIs 1.084-16.624, p=0.038), in the distal tract of the left popliteal vein (HR=4.201 95%CIs 1.484-11.885, p=0.007), and D-dimer values (HR=2.122 95%CIs 1.030-5.495, p=0.003) were independently associated with PE. The number of districts involved significantly correlated with PE (r=0.655, p=0.005).
**Conclusion:** As it is easily performable and repeatable, CUS is highly recommended as a screening test during the initial management of critical and sub-critical Sars-CoV-2 patients due to the frequency of DVT events.
**Limitations:** No limitations were identified.
**Ethics committee approval:** No information provided by the submitter.
**Funding for this study:** No funding was received for this study.
**Author Disclosures:**
Sandro Sironi: Nothing to disclose
Cesare Maino: Nothing to disclose
Carlo Capodaglio: Nothing to disclose
Cammillo Roberto Giovanni Leopoldo Oreste Massimiliano Talei Franzesi: Nothing to disclose
Rocco Corso: Nothing to disclose
Davide Ippolito: Nothing to disclose

### RPS 315-3

**Value of spectral CT image-based multiparameter for increase detection rate of embolus and improving a risk stratification model in acute pulmonary embolism**

*Y. Zhou*, Z. Wang, S. Dong; Zheng Zhou/CN
*(1178033775@qq.com)*

**Purpose:** Spectral CT provides quantitative multiparameter besides conventional CT attenuation for diagnosis. This study aims to explore whether spectral CT-derived iodine density (ID) and effective atomic number (Zeff) could improve the detection of thrombi in acute pulmonary embolism (PE) and refine a clinical risk stratification model in PE.
**Methods or Backround:** 75 patients who were diagnosed with pulmonary embolism and underwent CTPA by dual-layer detector spectral CT were prospectively enrolled. The raw data of CT images were reconstructed and divided into two groups, conventional images (CI) and spectral images (combined ID and Zeff, ID-Zeff). The number of thrombi was recorded separately. According to the ESC 2019 guidelines, the distribution of thrombi was analysed by deep-learning lung segment segmentation. For different risk stratification, the pulmonary embolism load was evaluated with Qanadli and Mastora scores. The receiver operating characteristic curve (ROC) and the area under the curve (AUC) were used to determine the diagnostic efficiency. Qualitative data were performed by a chi-squared test. Quantitative data used a t-test and a Mann-Whitney U test.
**Results or Findings:** In 75 patients, 173 and 225 thrombi were detected in CI and ID-Zeff, respectively. ID-Zeff (100% specificity and 97% sensitivity) showed better embolus detection ability than CI (94% specificity and 84% sensitivity p <0.01). There was a significant difference in the pulmonary embolism index between the low-risk and medium-risk groups using the ID-Zeff image (Qanadli: 15.6% vs 38.8%, p <0.001; Mastora: 10.44% vs 30.68%, p <0.001). The pulmonary embolism index using Qanadli and Mastora scores effectively discriminates in low-/intermediate risk (Qanadli: AUC=0.863, Mastora: AUC=0.869).
**Conclusion:** Spectral CT parameters can improve the diagnostic efficiency for detecting acute pulmonary embolism. The pulmonary embolism index based on ID-Zeff can accurately predict the clinical risk stratification of acute pulmonary embolism.
**Limitations:** No limitations were identified.
**Ethics committee approval:** No information was provided by the submitter.
**Funding for this study:** No funding was received for this study.
**Author Disclosures:**
Shushan Dong: Nothing to disclose
Zhihao Wang: Nothing to disclose
Yuhan Zhou: Nothing to disclose

### RPS 315-4

**Can we distinguish acute from chronic pulmonary thromboembolism using dual-energy CT?**

*M. Dobrovie*, M. Baila, R. Enache, R-M. Enache, V. I. Gingu, S. Andreea, R. Capsa, I. G. Lupescu; Bucharest/RO
*(monica_dobrovie@yahoo.com)*

**Purpose:** With the help of dual-energy CT (DECT), certain substances, such as iodine, can be detected and selectively displayed in the form of so-called "iodine maps". Thus, it is possible to visualise not only intravascular thrombi but also peripheral perfusion defects. Iodine maps reflect pulmonary blood volume, but through serial acquisitions in the pulmonary artery - and delayed time, they can also provide additional data related to pulmonary perfusion. Our aim was to differentiate acute from chronic pulmonary embolism (PE) by comparing perfusion defects on iodine maps acquired in the pulmonary artery (PA) - and aortic (AO) time.
**Methods or Backround:** We retrospectively analysed eight patients who underwent pulmonary CT angiography for suspected PE between April 2021 and July 2022, using DECT GE Revolution - 256 slices, in which acute or chronic PE was detected and both PA and AO time acquisitions were performed. Perfusion defects on iodine maps for both acquisitions time were compared through qualitative visual assessment and quantitative iodine concentration measurements.
**Results or Findings:** DECT reveals triangular perfusion defects, with a wide base towards the periphery, in the vascular territories with occlusive thrombi. In the case of acute thrombi (n = 4), perfusion defects had a similar appearance in PA and AO time, with no difference in iodine concentration (p 0.08). In the case of chronic thrombi (N=23), perfusion defects are less obvious in AO compared to PA time, most likely due to collaterals that form over time; measurements of iodine concentration were significantly different between both acquisition time points (p>0.0001).
**Conclusion:** DECT provides data related to pulmonary perfusion and can differentiate between acute and chronic PE.
**Limitations:** A small number of patients and no dedicated software for measuring total lung blood volume on iodine maps.
**Ethics committee approval:** No information provided by the submitter.
**Funding for this study:** No funding was received for this study.
**Author Disclosures:**
Ioana Gabriela Lupescu: Nothing to disclose
Robert-Mihai Enache: Nothing to disclose
Monica Dobrovie: Nothing to disclose
Serban Andreea: Nothing to disclose
Mihnea Baila: Nothing to disclose
Roxana Enache: Nothing to disclose
Razvan Capsa: Nothing to disclose
Victor Ion Gingu: Nothing to disclose

### RPS 315-5

### CT imaging biomarker to predict the progression of pulmonary hypertension

*D. Yoon*, E. J. Chun; Gyeonggi-do, Republic of Korea/KP
*(podongski20@naver.com)*

**Purpose:** To evaluate computed tomography(CT) imaging biomarkers to predict the progression of pulmonary hypertension(PH), comparing to right heart catheterization (RHC) as a reference standard
**Methods or Backround:** We retrospectively enrolled 150 consecutive patients(57.3±15.3 years, 43% male) with suspected PH who underwent cardiac CT and RHC within three intervals. According to main pulmonary artery pressure, patients were classified into five grades: < 20 mmHg(G1, normal), 20-25 mmHg(G2), 25-35 mmHg(G3), 35-45 mmHg(G4), and > 45 mmHg(G5). To find parameters with good inter-observer agreement while reflecting PH grades well, two observers measured each diameter of ascending aorta(A), pulmonary artery(P), right ventricle(RV), left ventricle(LV), right atrium(RA), left atrium(LA), inferior vena cava(IVC), septal flattening and septal angle.
**Results or Findings:** Of a total of 150 patients, G1(n=28), G2(n=17), G3(n=41), G4(n=33) and G5(n=31) were classified, respectively. Among them, pulmonary thromboembolism(n=20, 13%), coronary artery disease(n=25, 17%) and congenital heart disease(n=30, 20%) were combined. Using one-way analysis of variance, P, P/A ratio, RV, RV/LV ratio, RA, septal flattening, and angle were positively correlated with the grading of PH on RHC(p trend <0.05). Using univariate and multivariate analysis, the P/A ratio and RV/LV ratio were strong predictors for pulmonary hypertension among CT imaging parameters(<0.001). In addition, A, P/A ratio shows excellent inter-observer agreement(concordance correlation coefficient [CCC] > 0.9). Other parameters, including P, LV, RV/LV ratio, and LA dimension, also show good inter-observer variability(CCC 0.83-0.89).
**Conclusion:** Among CT imaging parameters, the P/A ratio is an excellent imaging biomarker with excellent inter-observer agreement that can predict not only the detection of PH but also the progression of PH severity. In addition, RV/LV ratio is also another good imaging biomarker for PH, although the inter-observer agreement is slightly lower than the P/A ratio.
**Limitations:** This study was a retrospective-single institutional study.
**Ethics committee approval:** This study was approved by Seoul National University Bundang Hospital, Institutional Review Board (IRB)
**Funding for this study:** No funding was received for this study.
**Author Disclosures:**
Eun Ju Chun: Nothing to disclose
Dowon Yoon: Nothing to disclose

### RPS 315-6

### An audit on the completeness of the information provided on requests for ultrasound (US) Doppler querying deep venous thrombosis (DVT)

*Z. Tabba*, G. Allen, C. Redmond; Waterford/IE

**Purpose:** The study aimed to assess the completeness of information on deep venous thrombosis (DVT) requests to facilitate a more efficient use of resources.
**Methods or Backround:** This clinical audit project adopted the Royal College of Radiologists' suggested template titled: An audit on the appropriateness and accuracy of information provided on ultrasound (US) requests in the deep venous thrombosis (DVT) service for suitable vetting and justification. 50 patients were selected from PACS over a 4-week period. Patient demographics, including age and gender, were collected. Requests were examined for the following: 1. requesting department, 2. Wells score, 3. D-dimer, 4. sufficient clinical information, 5. specific clinical question, and 6. DVT detection. We compared the requested data between the DVT-positive and DVT-negative groups to provide education to the various departments through a teaching session. The re-audit was done 4 weeks later.
**Results or Findings:** Of the 50 patients, 23 were male, and 27 were female. The average age was 61.3 years. 6/50 were positive for DVT, 10/50 had d-dimer provided, 10/50 had Well's score provided, 49/50 had a specific clinical question, 30/50 had sufficient clinical information. In the DVT-positive group, 33% (2/6) of the patients had complete information on their requests. In the DVT-negative group, 13.6% (6/44) had complete information on their requests. Requests by departments (% of total requests, % of complete information on requests): emergency department (ED): 60% of all requests, 16.7% had complete information, general practice (GP): 2%, 0%, medical teams: 16%, 37.5%, surgical teams: 20%, 0%. Rates of DVT detection by department: ED 3.3% (n=1), GP 100% (n=1), medical teams 25% (n=2), surgical teams 20% (n=2).
**Conclusion:** The rate of complete information on requests for ultrasound Doppler query DVT was below the gold standard target of 100%.
**Limitations:** Small number of patients.
**Ethics committee approval:** No information was provided by the submitter.
**Funding for this study:** No funding was received for this study.
**Author Disclosures:**
Grainne Allen: Nothing to disclose
Ciaran Redmond: Nothing to disclose
Zaid Tabba: Nothing to disclose

### RPS 315-7

### Non-invasive evaluation of portosystemic pressure gradients after TIPS placement by 4D flow MRI and computational fluid dynamics

*C. Riedel*, M. Hoffmann, A. Lenz, F. Piecha, I. Ristow, M. Schlüter, G. Adam, B. Schönnagel, P. Bannas; Hamburg/DE

**Purpose:** The study aimed to evaluate the non-invasive assessment of portosystemic pressure gradients in patients with transjugular intrahepatic portosystemic shunts (TIPS) using four-dimensional flow magnetic resonance imaging (4D flow MRI) and computational fluid dynamics (CFD).
**Methods or Backround:** 4D flow MRI was performed in 20 patients with TIPS. The flow rates were measured within the TIPS stent and the inferior vena cava (IVC). The portal vein (PV), TIPS tract, right liver vein, and IVC were segmented to create a CFD mesh. PV and infrahepatic IVC were defined as inlets for 4D flow MRI-derived flow rates. The suprahepatic IVC was defined as an outlet. Steady-state CFD simulations were performed to non-invasively estimate the portosystemic pressure gradient (PSPG) as the difference between the simulated pressure in the suprahepatic IVC and the PV. Invasive angiographic measurements of PSPG served as the standard of reference and were compared with 4D flow MRI-/CFD-derived non-invasive estimations using Pearson's correlation.
**Results or Findings:** CFD simulations allowed an analysis of flow velocities and pressure distributions. The simulated PSPG showed a significant correlation with the invasive measurement of PSPG (r=0.770, p<0.001). Angiography revealed severe portal hypertension (PSPG > 12 mmHg) in 11 of 20 patients (17.5±4.5 mmHg). A PSPG ≤ 12 mmHg was observed in 9 of 20 patients (8.7±1.9 mmHg). CFD simulations based on 4D flow MRI-derived measurements correctly classified 16 of 20 patients (80%) regarding the presence or absence of severe portal hypertension.
**Conclusion:** The combination of 4D flow MRI and CFD might be used to non-invasively assess portosystemic pressure gradients following TIPS placement and has the potential to determine the need for invasive catheter angiography in case of suspected TIPS dysfunction.
**Limitations:** Simulated flows were assumed to be constant, and influences of, e.g. the other hepatic veins were considered to be negligible.
**Ethics committee approval:** This study was approved by the local ethics board.
**Funding for this study:** The study was funded by the Forschungszentrum Medizintechnik Hamburg.
**Author Disclosures:**
Christoph Riedel: Nothing to disclose
Marko Hoffmann: Nothing to disclose
Michael Schlüter: Nothing to disclose
Alexander Lenz: Nothing to disclose
Peter Bannas: Nothing to disclose
Björn Schönnagel: Nothing to disclose
Gerhard Adam: Nothing to disclose
Inka Ristow: Nothing to disclose
Felix Piecha: Nothing to disclose

13:00-14:30 Research Stage 1

### Research Presentation Session: Artificial Intelligence & Machine Learning & Imaging Informatics

### RPS 405

### AI applications in chest CT

**Moderator**
C. de Margerie-Mellon; Paris/FR
*(constance.de-margerie@aphp.fr)*
**Author Disclosures:**
Constance De Margerie-Mellon: Consultant: Boerhinger Ingelheim, Pfizer, Gilead Science, Bracco

### RPS 405-2

**Machine learning classification of mediastinal lymph node metastasis in NSCLC: a multicentre study in a Western European patient population**

S. Laros[1], D. Dickerscheid[1], S. Blazis[1], *J. Van Der Heide*[2]; [1]Dordrecht/NL, [2]Aachen/DE
*(j.vdheide@live.nl)*

**Purpose:** FDG PET-CT plays an important role in the initial staging of lung cancer, but accurate differentiation between activity in malignant and benign intrathoracic lymph nodes on PET-CT scans is challenging. The purpose of the current study was to investigate the effects of incorporating primary tumour data and clinical features to differentiate between FDG-avid malignant and benign intrathoracic lymph nodes.
**Methods or Backround:** We retrospectively selected lung cancer patients who underwent PET-CT for initial staging in two Dutch centres. The primary tumour and suspected lymph node metastases were annotated and cross-referenced with pathology results. Lymph nodes were classified as malignant or benign. From the image data, we extracted radiomic features and trained the classifier model using the extreme gradient boost algorithm. Various scenarios were defined by selecting different combinations of data input and clinical features. Data from centre one was used for training and validation of the models, and data form centre two was used for testing.
**Results or Findings:** Adding primary tumour data resulted in a significant gain in the performance of the trained classifier model. Adding clinical information about distant metastases did not lead to significant improvement. The performance of the model in the test set (centre 2) was slightly but statistically significantly lower than in the validation set (centre 1).
**Conclusion:** Using the extreme gradient boost algorithm potentially leads to an improved model for the classification of intrathoracic lymph nodes. The inclusion of primary tumour data improved the performance of the model; additional knowledge of distant metastases did not. In patients in whom metastases are limited to intrathoracic lymph nodes, this may reduce costly and invasive procedures such as bronchoscopy or mediastinoscopy.
**Limitations:** Pathology results were used as ground truth, but cytology from bronchoscopy procedures has approximately 95% sensitivity only.
**Ethics committee approval:** This retrospective study was approved by the institutional review board, which waived the need for informed consent.
**Funding for this study:** Funding was received from Albert Schweitzer Hospital.
**Author Disclosures:**
Johannes Van Der Heide: Nothing to disclose
Sara Laros: Nothing to disclose
Stephan Blazis: Nothing to disclose
Dennis Dickerscheid: Nothing to disclose

### RPS 405-3

**Different lung parenchyma quantification using dissimilar segmentation software: a multi-centre study for COVID-19 patients**

*C. Risoli*[1], M. Nicolò[2], D. Colombi[1], R. Ambrosini[2], L. Grazioli[2], A. Adraman[3], E. Michieletti[1], M. Moia[1], C. Martini[4]; [1]Piacenza/IT, [2]Brescia/IT, [3]Trento/IT, [4]Parma/IT
*(camilla.risoli11@gmail.com)*

**Purpose:** This study aims to compare the qualitative/quantitative pathological lung extension data on COVID-19 patients. Secondly, the quantitative data obtained were compared to verify their concordance since they were derived from three different lung segmentation software.
**Methods or Backround:** This double-centre study includes a total of 120 COVID-19 patients (60 from each center) with positive reverse-transcription polymerase chain reaction (RT-PCR) who underwent a chest CT scan from November 2020 to February 2021. CT scans were analysed retrospectively and independently in each centre. Specifically, CT images were examined manually by two different and experienced radiologists for each center, providing the qualitative extent score of lung involvement, whereas the quantitative analysis was performed by one trained radiographer for each centre using three different software: 3DSlicer, CT Lung Density Analysis, and CT Pulmo 3D.
**Results or Findings:** The agreement between radiologists for visual estimation of pneumonia at CT can be defined as good (ICC 0.79, 95% CI 0.73–0.84). The statistical tests show that 3DSlicer overestimates the measures assessed; however, ICC index returns a value of 0.92 (CI 0.90–0.94), indicating excellent reliability within the three-software employed. The statistical analysis underlines that the best agreement is between 3D Slicer “LungCTAnalyzer” and the median of the visual score (0.75 with a CI 0.67–82 and with a median value of 22% of disease extension for the software and 25% for the visual values).
**Conclusion:** This study provides for the first time a direct comparison between the actual gold standard, which is represented by the qualitative information described by radiologists, and novel quantitative AI-based techniques, here represented by three different commonly used lung segmentation software.
**Limitations:** The retrospective nature of the study and in having just one radiographer per centre analyzing patients with the segmentation software.
**Ethics committee approval:** Protocol approval number 553/2021/OSS/AUSLPC-LUNG-COVID-19.
**Funding for this study:** No funding was received for this study.
**Author Disclosures:**
Camilla Risoli: Nothing to disclose
Marco Moia: Nothing to disclose
Emanuele Michieletti: Nothing to disclose
Marco Nicolò: Nothing to disclose
Davide Colombi: Nothing to disclose
Roberta Ambrosini: Nothing to disclose
Luigi Grazioli: Nothing to disclose
Altin Adraman: Nothing to disclose
Chiara Martini: Nothing to disclose

### RPS 405-4

**Feature selection and prediction of resolving indeterminate lung nodules based on random forest**

*J. Wang*, N. G. van der Velden, G. J. Pelgrim, P. M. A. Van Ooijen, R. Vliegenthart; Groningen/NL
*(j.wang02@umcg.nl)*

**Purpose:** To identify important clinical features and make a prediction of the disappearance of indeterminate nodules detected at baseline CT screening by using the random forest method.
**Methods or Backround:** A considerable percentage of intermediate-sized nodules (volume, 50-500 mm3) resolve on follow-up imaging, but it’s difficult to predict which nodule disappears. The features of 724 indeterminate nodules (575 participants) of a baseline round in a lung cancer screening trial were used, divided into training (80%) and test (20%) sets. The indeterminate nodules were re-examined at a 3-month follow-up round. In machine learning methods, random forest is an ensemble algorithm with strong feature extraction and prediction ability with high dimension, redundancy, and correlation. Therefore, we applied a random forest model to predict the resolving and non-resolving of indeterminate nodules by using important features. To maximize the performance of the random forest, we performed a random search for better hyperparameters, evaluated the model using 5-fold cross-validation on the training data, and selected the best performance model. We also compared the prediction performance of the random forest model with three other machine learning models (decision tree, K-nearest neighbour, and extra trees).
**Results or Findings:** The top five-ranked important features were volume (0.298), maximum diameter (0.183), minimum diameter (0.160), age (0.093), and pack-years (0.086). The AUC score for the optimal random forest model was 91.4% for the test set, compared to 75.1% for the decision tree, 80.1% for the K-nearest neighbour, and 88.6% for extra trees.
**Conclusion:** This study shows that the random forest model has high accuracy in predicting short-term, intermediate lung nodule disappearance by using clinical features.
**Limitations:** The data came from multiple centers, but the amount of data in each center is not large enough. Data supplementation is the next step.
**Ethics committee approval:** NELSON data ethics committee
**Funding for this study:** Not applicable.
**Author Disclosures:**
Nils Gösta van der Velden: Nothing to disclose
Jingxuan Wang: Nothing to disclose
Peter M. A. Van Ooijen: Nothing to disclose
Rozemarijn Vliegenthart: Research/Grant Support: Institutional research grant from Siemens Healthineers
Gert Jan Pelgrim: Nothing to disclose

### RPS 405-5

**CT-based end-to-end deep learning approach for COVID-19 outcome prediction**

*X. Wei*; Beijing/CN

**Purpose:** Automatic detection through CT images provides a great help for rapid and accurate detection of COVID-19. We propose an end-to-end multimodal features fusion algorithm of deep learning for outcome prediction of COVID-19.
**Methods or Backround:** We used pulmonary AI software to process 769 chest CT data, including data desensitisation, denoising, segmentation of the lesion area, and extraction of geometric features after segmentation. We have collected time series CT data from every patient, and extract a variety of high-order geometric features. Then the multimodal features are input into the fusion module to obtain the characteristics of the time series, and the prognosis of the disease is predicted by Long Short-Term Memory network (LSTM). Each case of CT contains information about the outcome. Then input the processed features into the proposed deep neural network model to predict the outcome. We divide all data into 80% training set for training the deep neural network model, and 20% test set for testing the performance of the model. The training set contains 293 cases of pneumonia reduction CT and 316 cases of pneumonia exacerbation CT; the test set includes 78 cases of pneumonia reduction CT and 82 cases of pneumonia exacerbation CT. We extracted geometric features and finally built an outcome prediction model based on the volume of pneumonia.
**Results or Findings:** The results showed that the geometric fusion network provides 74% accuracy, 77% precision, 71% recall, 74 % F1 score and 76% specificity.
**Conclusion:** The method proposed can be a useful tool for radiologists to predict the outcome and the outcomes of patients with high accuracy and the patterns will aid in fighting COVID-19.
**Limitations:** We need to collect more patients for further analysis.
**Ethics committee approval:** This study was approved by the ethics committees of Beijing Friendship Hospital, 2022-P2-216-01.
**Funding for this study:** National Natural Science Foundation of China (62141110).
**Author Disclosures:**
Xuan Wei: Nothing to disclose

### RPS 405-6

**Vaccination effect on patients with Delta variant of COVID-19 pneumonia: a study of longitudinal dynamic chest CTs using artificial intelligence model**

*Y. Sun*, X. Xin, J. Hu, X. Peng, B. Zhang; Nanjing/CN

**Purpose:** The Delta variant of COVID-19 has recently emerged and spreads globally. Their vaccination status, symptom onset time, and imaging signatures have not been fully investigated and clarified.Therefore,we want to investigate these.
**Methods or Backround:** This study included 131 patients who were infected with the Delta variant of COVID-19. After screening, 106 patients with 458 follow-up CT scans were retrospectively selected and divided into complete and incomplete vaccination groups (66 and 40 patients, respectively). Imaging features were automatically extracted, and infection distribution in lung fields and progression pattern tendency were investigated. Furthermore, we extracted the most related clinical and imaging features to establish a vaccination-status classification model. An independent testing dataset with 55 patients from another inpatient ward was enrolled to evaluate the generalisability of the model.
**Results or Findings:** The severity of infection in lung and lung fields of the complete vaccination group was overall lower than those of the incomplete vaccination group. A relatively earlier peak CT abnormality was found on days 8-11 in the complete vaccination group than in incomplete vaccination group on days 12-15 after the first positive PCR time. The vaccination-status classification model achieved the highest performance with an AUC of 0.929, accuracy of 0.864 in the testing set, and an AUC of 0.858, accuracy of 0.727 in the independent testing set.
**Conclusion:** In summary, compared to the incomplete vaccination group, the fully vaccinated patients had milder CT abnormalities and earlier peak time for chest impairment. Also, the vaccination status shows determinable through imaging and clinical features.
**Limitations:** Not applicable.
**Ethics committee approval:** This study was approved by the ethics committees of the Second Affiliated Hospital of Nanjing University of Chinese Medicine, Nanjing, China.
**Funding for this study:** Not applicable.
**Author Disclosures:**
Bing Zhang: Nothing to disclose
Jun Hu: Nothing to disclose
Yi Sun: Nothing to disclose
Xiaoyan Xin: Nothing to disclose
Xin Peng: Nothing to disclose

### RPS 405-7

**Automated CT severity score analysis of COVID-19 pneumonia: a comparative analysis against radiologists' estimation during a pandemic surge**

*V. K. Venugopal*, S. S. Chennamsetty, A. Chunduru, K. C. Kaluva, S. Gupta, R. Takhar, P. Mehta, H. Mahajan; New Delhi/IN
*(vasanthdrv@gmail.com)*

**Purpose:** To compare the CT severity scores of COVID19 pneumonia as estimated by an automated deep learning-based system against the radiologists' estimation in a real-world workflow.
**Methods or Backround:** This is a retrospective study. 265 HRCT lung studies performed on RTPCR+ cases of COVID19 disease were included in this study. These cases were from three different centres, two outpatient clinics, and one hospital. These studies were read and reported by four chest radiologists, each with more than five years of experience. They also estimated the CT severity scores on a 25-point scale. These studies were then analysed by a deep-learning-based solution LungIQ (predible health inc.). The CT severity scores generated by this solution were then compared with radiologists' scores using Bland Altman analysis.
**Results or Findings:** On the Bland Altman plot, 95% of the data points fell within the 95% confidence interval indicating good agreement between the AI and radiologist scores. The overall mean average difference of scores was 2.6. 60% of the studies had <=2pt difference and 74% of the studies had<=3pt difference. The CT score-based severity categorisation was also compared. None of the severe cases were categorised as mild by the algorithm. In 35 cases, both the scores matched exactly. In 94 cases, the AI estimation was more than the radiology scores and in the remaining 136 cases, radiologist estimated scores were more than the AI estimated scores.
**Conclusion:** The deep learning system shows comparable performance to the experienced radiologists in predicting the CT severity scores. This solution can be used to effectively triage critical patients during pandemic surges.
**Limitations:** Limited number of cases.
**Ethics committee approval:** No information provided by the submitter.
**Funding for this study:** No funding was received for this study.
**Author Disclosures:**
Salil Gupta: Employee: CARPL.ai
Parv Mehta: Employee: CARPL.ai
Krishna Chaitanya Kaluva: Nothing to disclose
Rohit Takhar: Employee: CARPL.ai
Sai Saketh Chennamsetty: Nothing to disclose
Harsh Mahajan: Other: Research Collaboration with Subtle Medical Other: Research Collaboration with Lunit.ai Other: Research Collaboration with General Electric Other: Director at Mahajan Imaging Other: Research Collaboration with Qure.ai Other: Research Collaboration with Quibim Other: Research Collaboration with Oxipit.ai Other: Research Collaboration with Predible Health Other: Research Collaboration with Koninklijke Philips NV research
Vasantha Kumar Venugopal: Other: Director at Mahajan Imaging Other: Research Collaboration with Subtle Medical Other: Research Collaboration with Oxipit.ai Other: Research Collaboration with Qure.ai Other: Research Collaboration with General Electric Other: Research Collaboration with Koninklijke Philips NV research Other: Research Collaboration with Lunit.ai Other: Research Collaboration with Predible Health Other: Research Collaboration with Quibim
Abhijith Chunduru: Nothing to disclose

### RPS 405-8

**RespiNet: a deep learning model for multipurpose segmentation in patients with atypical pulmonary morphology**

*S. Bonte*[1], R. João[1], S. Verhulst[2], K. Van Hoorenbeeck[2], D-W. Perng[3], P-W. Hu[3], H-K. Ko[3], M. Lanclus[1], B. R. Lavon[1]; [1]Kontich/BE, [2]Antwerp/BE, [3]Taipei/TW
*(stijn.bonte@fluidda.com)*

**Purpose:** Quantitative CT measurements of airway volume and morphology have gained attention as potential tools for the diagnosis and management of lung disease. The time-consuming and observer-dependent nature of manual segmentation has led to the development of automated methods based on deep learning. The presence of interstitial fibrosis, bronchiectasis, and other findings; however, may confound automated methods. We present our deep learning model for the segmentation of the lobes and airway tree in volumetric, thin-slice CT scans called RespiNet.
**Methods or Backround:** RespiNet's architecture is based on UNet, adapted to have two separate encoder paths: one for airway segmentation, one for lobe segmentation. The training set includes over 2500 chest CT scans from subjects with a variety of diagnoses and manually annotated. We compared the output of RespiNet with manual segmentation on an independent set of scans acquired at TLC from 9 patients with cystic fibrosis (CF) and 11 with idiopathic pulmonary fibrosis (IPF) from centers in Belgium and Taiwan, respectively.

Wednesday

**Results or Findings:** The average dice similarity coefficient for the total airway is 0.940 (range 0.905-0.968) in CF and 0.946 (range 0.916-0.970) in IPF. For the distal airways, the values are 0.871 (range 0.789-0.917) and 0.836 (range 0.812-0.870) in CF and IPF, respectively.
**Conclusion:** In this study, we have shown robust performance of RespiNet airway segmentation in diseases where manual annotation is challenging. These segmentations may be used as is or as a basis for manual corrections to reduce processing time. Due to the size and variability of the training set, we expect similar results in other diseases such as asthma, COPD or COVID-19.
**Limitations:** Most training scans are reconstructed with sharp kernels, the model might show worse performance with other reconstruction settings.
**Ethics committee approval:** No information provided by the submitter.
**Funding for this study:** RespiNet is developed by Fluidda.
**Author Disclosures:**
Maarten Lanclus: Employee: Fluidda
Kim Van Hoorenbeeck: Nothing to disclose
Benjamin R. Lavon: Employee: Fluidda
Hsin-Kuo Ko: Nothing to disclose
Po-Wei Hu: Nothing to disclose
Rita João: Employee: Fluidda
Diahn-Warng Perng: Nothing to disclose
Stijn Verhulst: Nothing to disclose
Stijn Bonte: Employee: Fluidda

### RPS 405-9

**Comparing emphysema detection based on a threshold and deep learning**

*M. Perkonigg*, M. M. Pieler, J. Hofmanninger, L. Nicodeme, E. Jiménez Arroyo, R. Zhang, M. Krenn, G. Langs, A. Makropoulos; Vienna/AT

**Purpose:** Emphysema detection and volumetry in lung CT serves as an important factor in COPD detection and is relevant for timely and effective treatment, risk prediction for acute respiratory events, prognosis, and lung cancer surveillance. In this study we compare a deep learning (DL) based segmentation approach for quantification of emphysema in lung CT to a standard HU-based approach using a threshold of -950 HU.
**Methods or Backround:** We assessed the segmentation performance of DL-based emphysema segmentation in comparison to standard HU-based thresholding on 3617 2D CT slices drawn from a set of 494 emphysema-positive scans. Ground truth was established through pixel-wise labeling of emphysematous image regions performed by expert radiologists. Additionally case-level performance was assessed on the set of emphysema-positive and 559 emphysema-negative scans.
**Results or Findings:** For pixel-wise segmentation measurements the DL-approach reached a Dice score of 0.40 compared to 0.23 for the HU-thresholding approach. For case-level performance the sensitivity of both approaches is high with 0.97 and 1.0 for DL-based and HU-thresholding respectively. However, DL-based segmentation reaches a specificity of 0.56 compared to 0.002 for HU-thresholding. Precluding the impact of small predictions by removing segmentations below a coverage cut-off of 5% of total lung volume, the DL-approach reached a sensitivity of 0.95 and specificity of 0.68 while HU-thresholding reached 0.98 sensitivity and 0.09 specificity.
**Conclusion:** The DL approach detects the presence of emphysema more accurately compared to the HU-thresholding approach. Additionally, the DL approach is able to measure the extent of emphysema more accurately which may be an important step towards detecting emphysema subtypes on a variety of lung CT scanner types.
**Limitations:** The HU-thresholding method does not apply any post-processing steps, such as cluster analysis or noise-reduction.
**Ethics committee approval:** No information provided by the submitter.
**Funding for this study:** Not applicable.
**Author Disclosures:**
Georg Langs: Shareholder: contextflow GmbH Founder: contextflow GmbH
Johannes Hofmanninger: Nothing to disclose
Matthias Perkonigg: Nothing to disclose
Ester Jiménez Arroyo: Nothing to disclose
Rui Zhang: Nothing to disclose
Markus Krenn: Nothing to disclose
Luc Nicodeme: Nothing to disclose
Antonios Makropoulos: Nothing to disclose
Michael Martin Pieler: Nothing to disclose

### RPS 405-10

**Using artificial intelligence to interrogate multi-national imaging datasets to determine the mechanism of COVID-19 pneumothorax**

*I. A. Selby*, D. Driggs, V. Majcher, M. Roberts, L. Escudero Sanchez, E. Sala, C-B. Schönlieb, S. J. Marciniak, J. Babar; Cambridge/UK
*(ias49@cam.ac.uk)*

**Purpose:** Pneumothorax is a rare but important complication of COVID-19. Although barotrauma may account for some cases, many affected patients have not received positive-pressure ventilation (PPV). The pathophysiology is challenging to investigate because data exists in silos, and the incidence is only 1% in COVID-19 patients. To provide mechanistic insight, we used artificial intelligence (AI) at scale to identify cases from 4 imaging datasets across 26 centres in 7 countries.
**Methods or Backround:** A convolutional neural network was trained to detect pneumothorax on chest x-rays (CXRs). Predicted pneumothoraces on COVID-positive CXRs were reviewed, and the incidence of COVID-pneumothorax was estimated. Available CTs for patients with pneumothorax were assessed by radiologists.
**Results or Findings:** Pneumothoraces were confirmed for 55 patients, and the estimated incidence was 0.97%. Forty-five patients with CTs were identified; however, 13 were unrelated to COVID-19, and nine iatrogenic cases (except barotrauma) were excluded. Most pneumothoraces in patients on PPV were likely related to an interplay of barotrauma and COVID-19, with an acute lung injury pattern on CT. A high proportion demonstrated emphysema, and three patients developed cystic abnormalities. Patients not on PPV developed pneumothoraces later in the disease, with CT showing patterns consistent with the absorption stage of COVID-19, suggesting such pneumothoraces perhaps represent increased parenchymal resistance.
**Conclusion:** There are multiple mechanisms of COVID-pneumothorax. Barotrauma in patients with acute lung injury is most common, whilst pneumothorax in the absence of PPV most commonly occurs in the sub-acute absorption stage of the disease. This study highlights the utility of AI in investigating rare diseases.
**Limitations:** Not applicable.
**Ethics committee approval:** Original approval obtained in April 2020, with ‘substantial’ amendments approved on 9th August 2021. Approval was provided by the London - Brent Research Ethics Committee, and the Health Research Authority (HRA) and Health and Care Research Wales (HCRW) (IRAS ID: 282705, REC No.: 20/HRA/2504, R\&D No.: A095585).
**Funding for this study:** Funding was received from EU/EFPIA IMI2 Joint Undertaking: DRAGON (101005122); NIHR Cambridge Biomedical Research Centre (BRC-1215-20014); CRUK National Cancer Imaging Translational. Accelerator (C22479/A28667); Cambridge Mathematics of Information in Healthcare (EP/T017961/1); Intel Corporation.
**Author Disclosures:**
Carola-Bibiane Schönlieb: Nothing to disclose
Michael Roberts: Employee: AstraZeneca
Derek Driggs: Nothing to disclose
Lorena Escudero Sanchez: Nothing to disclose
Veronika Majcher: Nothing to disclose
Evis Sala: Shareholder: Lucida Medical Speaker: GSK Speaker: Siemens Consultant: Amazon
Ian Andrew Selby: Nothing to disclose
Judith Babar: Nothing to disclose
Stefan J. Marciniak: Nothing to disclose

### RPS 405-11

**Prediction of RECIST progression in NSCLC patients treated with immunotherapy using routine laboratory blood tests**

*M. Yeghaian*[1], T. Bucho[1], Z. Bodalal[1], E. Smit[2], R. G. H. Beets-Tan[1], D. van den Broek[1], S. Trebeschi[1]; [1]Amsterdam/NL, [2]Leiden/NL
*(m.yeghaian@nki.nl)*

**Purpose:** Tumour progression is quantitatively detected on follow-up imaging using RECIST criteria. Serial imaging is costly and exposes patients to radiation. In contrast, blood tests are routinely available and cost-effective. Our study aims to investigate whether routine blood tests can be employed for the prediction of progression-free survival (PFS) in a longitudinal cohort of non-small cell lung cancer (NSCLC) patients receiving immunotherapy.
**Methods or Backround:** We included n=170 stage-IV NSCLC patients and n=4184 blood tests and recorded progression according to RECIST. Associative and predictive analysis, via machine learning, were employed to predict whether progression had occurred at the time of blood withdrawal or whether it would happen within the next 1, 3, 6, 9 and 12 months.
**Results or Findings:** Our averaged PFS predictive results on 100 Monte-Carlo cross-validation splits were AUCs of 0.74 (CI: 0.68-0.80) for concurrent progression, with a downward performance trend for prediction of future progression, up to 0.71 (CI: 0.63-0.78) to predict progression in a year time. Alkaline phosphatase (ALP) and C-reactive protein (CRP) were observed to be among the most predictive features for progression. These markers were also present among the most important markers for overall survival (OS) prediction, demonstrating a potentially stronger predictive power (up to 0.87 AUC).
**Conclusion:** Our analysis showed that routine blood tests can moderately be predictive of radiological progression, suggesting a moderate biological basis of RECIST progression, yet weaker than overall survival.
**Limitations:** Further studies should be conducted on larger cohorts to improve the predictive performance of blood data and to be able to demonstrate whether we could potentially use it as an alternative to radiological imaging for the prediction of radiological progression.
**Ethics committee approval:** Approved by the ethical committee (IRBd21-142)
**Funding for this study:** Not applicable.

**Author Disclosures:**
Zuhir Bodalal: Nothing to disclose
Teresa Bucho: Nothing to disclose
Stefano Trebeschi: Nothing to disclose
Egbert Smit: Nothing to disclose
Regina G. H. Beets-Tan: Nothing to disclose
Melda Yeghaian: Nothing to disclose
Daan van den Broek: Nothing to disclose

### RPS 405-12

**A multi-centre study on detection of COVID-19 using distinct AI models**

*A. Jaiswal*[1], R. Shahzad[2], P. Fervers[1], F. Meng[3], H. Zhang[3], T. Persigehl[1], J. Kottlors[1]; [1]Cologne/DE, [2]Aachen/DE, [3]Changchun/CN
*(astha.jaiswal@uk-koeln.de)*

**Purpose:** To assess the performance of distinct AI approaches using a clinically relevant set of CT-scans in classifying COVID-19 and community-acquired pneumonia(CAP).
**Methods or Backround:** Retrospective IRB waived, multi-centre, multi-vendor study consisting of 1591 chest CT-scans of COVID-19 (n=762) and CAP (n=829) patients from China and Germany included. All COVID-19 cases are RT-PCR confirmed and were characterised by pulmonary infiltration. CAP cases include inflammatory infiltrations and were confirmed by a microbiological diagnosis or typical imaging finding and a negative RT-PCR test. Scans with lung tumours, tuberculosis, traumatic, and postoperative scarred lesions were excluded. For COVID-19 detection, we trained and validated three distinct AI models with different architectures: COVNet based on 2D-CNN, DeCoVNet based on 3D-CNN, and AD3D-MIL based on 3D-CNN with attention module. Input to these classifications models are lung masked CT-scans along with patient-level labels. Lung masks were obtained using Seg-net or U-net based lung segmentation models. 991 CT-scans were used for training the AI models using 5-fold-cross-validation. 600 CT-scans from 6 different centers were used for independent testing. Model's performance was thoroughly evaluated using accuracy (ACC), sensitivity (Se), and specificity (Sp).
**Results or Findings:** The average validation accuracies of COVNet, DeCoVNet, and AD3D-MIL models over the 5-folds are 80.9%, 82.0% and 84.3%, respectively. On the independent test set with 600 CT-scans, COVNet yielded ACC=76.6%, Se=67.8%, Sp=85.7%; DeCoVNet provided ACC=75.1%, Se=61.2%, Sp=89.7%; and AD3D-MIL resulted in ACC=73.9%, Se=57.7%, Sp=90.8%.
**Conclusion:** AI classification models are useful in detecting COVID-19. The detection performance is highly dependent on the training data rather than the architecture itself. Results demonstrating a higher specificity and a moderate sensitivity.
**Limitations:** This retrospective study design focuses on COVID-19 with exclusion of other classification options in daily clinical reporting. Moreover, human-machine interaction was not evaluated.
**Ethics committee approval:** This retrospective study received ethical approval and informed consent was waived at all participating hospitals (Jilin: 2020-595, Wuhan: [2020]17, Ningbo: PJ-NBEY-KY-2020-194-01, Cologne: 20-1676, Frankfurt: 20-719, and Heidelberg: S-293/2020).
**Funding for this study:** Sino-German Center for Research Promotion(C-0007); RACOON(NUM), BMBF(01KX2021).
**Author Disclosures:**
Rahil Shahzad: Employee: Philips Healthcare
Philipp Fervers: Nothing to disclose
Astha Jaiswal: Nothing to disclose
Jonathan Kottlors: Nothing to disclose
Fanyang Meng: Nothing to disclose
Thorsten Persigehl: Nothing to disclose
Huimao Zhang: Nothing to disclose

**13:00-14:30 Research Stage 2**

### Research Presentation Session: Breast

### RPS 402

### Interventions and diagnostic scenarios in breast imaging

**Moderator**
G. Ivanac; Zagreb/HR
*(gordana.augustan@gmail.com)*

### RPS 402-2

**Contrast-enhanced cone-beam breast CT-guided biopsies in breast phantoms: accuracy, rate of diagnostic success, and total intervention time**

*A. Hoxhaj*[1], A. A. D'Angelo[2], I. Sechopoulos[3], R. Mann[3]; [1]Amsterdam/NL, [2]Rome/IT, [3]Nijmegen/NL
*(a.hoxhaj@nki.nl)*

**Purpose:** The study aimed to evaluate the feasibility and the success rate of vacuum-assisted biopsy (VAB) guided by contrast-enhanced cone-beam breast CT (CE-bCT) with breast phantoms.
**Methods or Backround:** CE-bCT-guided VAB was performed in four different types of phantoms (chicken breast, kiwi, banana, bread) in order to show the targeting accuracy in breasts of different consistency. Iodinated contrast-enhanced olives (N=10) were introduced in the phantoms to simulate mass lesions. Biopsies were performed using a dedicated bCT (CBCT 1000, Koning Corp., Norcross, USA). The technical success rate (defined as olive in the sample) and the time for identification/targeting of lesions, tissue sampling, and total intervention time were documented. Targeting was deemed accurate when the centre of the biopsy chamber was ≤ 5 mm from the predetermined target.
**Results or Findings:** Ten 9G VABs were performed (12 samples) to biopsy 10 lesions. Technical success was achieved in all 10 lesions (100%). During the biopsy on a kiwi phantom, needle repositioning was needed twice as the distance to the target lesion was 7 mm and 6 mm in the first attempts. The location of the biopsy cavity was at the site of the lesion at the control scan for all 10 lesions, and the median size was 28.5 (27.25, 30) mm. The median total intervention time was 22.5 (19, 27.75) minutes. The identification/targeting of lesions and tissue sampling took 11.5 (10.25, 17=6.75) and 10.5 (9, 12.5) minutes.
**Conclusion:** The success rate of CEbCT-guided VAB in breast phantoms was comparable to that of MRI-guided VAB reported in the literature (87-99%). The median total intervention time was shorter as compared to that reported in the literature for MRI-guided VAB.
**Limitations:** Targeting lesions on breast phantoms does not always simulate real patients, and studies in real patients are needed.
**Ethics committee approval:** No information provided by the submitter.
**Funding for this study:** VIDI grant ZonMw.
**Author Disclosures:**
Alma Hoxhaj: Nothing to disclose
Ritse Mann: Consultant: Bayer Healthcare, Siemens Healthineers, BD, Transonic Imaging Research/Grant Support: Siemens Healthineers, Medtronic, Bayer Healthcare, BD, Screenpoint Medical, Seno Medical, and Koning
Ioannis Sechopoulos: Speaker: Siemens Healthcare Research/Grant Support: Siemens Healthcare, Canon Medical, ScreenPoint Medical, Sectra Benelux, Volpara Healthcare, Lunit, iCAD
Anna D'Angelo: Nothing to disclose

### RPS 402-3

**Efficacy and safety of vacuum-assisted excision (VAE) for fibroadenomas: a single tertiary centre experience**

*S. Carriero*, C. Depretto, E. D'Ascoli, G. Irmici, G. Della Pepa, C. De Berardinis, C. Tamburrano, S. Marziali, G. P. Scaperrotta; Milan/IT
*(serena.carriero@gmail.com)*

**Purpose:** Fibroadenoma (FAD) of the breast is a common cause of a benign breast lump in premenopausal women and has an incidence of 10% in a women's lifetime. Surgical excision of FAD is an established and safe procedure but has some disadvantages: high costs, the need for an operatory room and the presence of a scar. Nowadays, vacuum-assisted excision (VAE) is a minimally invasive technique for the excision of FAD with a reduction of costs, waiting time and better cosmetic results. Our study aims to evaluate the efficacy and safety of US-guided percutaneous VAB excision of FAD.
**Methods or Backround:** Our retrospective study included all patients with FAD who underwent US-guided VAE between 2011 and 2017. All procedures were performed under local anaesthesia with a 9G device ATEC SUROS (Hologic Inc.). All FAD with a previous histological diagnosis and a maximum diameter of 3 cm following the US Food and Drug Administration guidelines for the removal of benign lesions were included. We evaluated: technical success, technical efficacy, and follow-up with breast US and/or mammography after 6, 12, 24 and 32 months. We also evaluated major and minor complications. All patients fulfilled a satisfaction survey.
**Results or Findings:** We enrolled 109 patients with a total of 110 FAD. The technical success was 100%, and the technical efficacy was 98%. No major complications were found. Only 3 minor complications (haematomas) were noticed. All patients were satisfied with the procedure and the cosmetic result (100%).
**Conclusion:** US-guided VAE is a safe and effective procedure in the excision of FAD. It represents a valid and feasible alternative to surgery with lower costs, better compliance and better cosmetic results.
**Limitations:** The limitations of this study are its retrospective nature and the limited sample size.
**Ethics committee approval:** No information was provided by the submitter.
**Funding for this study:** No funding was received for this study.
**Author Disclosures:**
Gianmarco Della Pepa: Nothing to disclose
Claudia De Berardinis: Nothing to disclose
Elisa D'Ascoli: Nothing to disclose
Sara Marziali: Nothing to disclose
Serena Carriero: Nothing to disclose
Gianfranco Paride Scaperrotta: Nothing to disclose
Catherine Depretto: Nothing to disclose
Giovanni Irmici: Nothing to disclose
Chiara Tamburrano: Nothing to disclose

### RPS 402-4

**Evaluation of the safety and effectiveness of breast tumour cryoablation at our institution**

P. Arman Retana, *L. Abelairas López*, Á. Villares, L. Graña Lopez; Lugo/ES

**Purpose:** To evaluate the safety and effectiveness of cryoablation for the treatment of benign and malignant breast tumours and present our results.
**Methods or Backround:** Between 2019-2022 a total of 39 patients underwent percutaneous liquid nitrogen-based cryoablation of unifocal breast tumours (9 benign and 30 malignant) under ultrasound (US) guidance. Mammography and/or ultrasound were scheduled every six months to assess the response and recurrence.
**Results or Findings:** From 2019 to 2022, 9 benign breast tumours were treated with cryoablation at our institution. The analysis at a median follow-up of 39 months showed a high success rate. Only one phyllode tumour required surgery due to a residual tumour observed after 12 months of surveillance. Thirty biopsy-proven breast cancers (BC) (median size 24 mm) underwent US-guided cryoablation, 26 were Luminal BC, and 3 were triple-negative tumours. One patient presented stage IV BC. Candidates had either refused surgery or were not surgical candidates. The procedure was well-tolerated, and no major adverse effects were observed. The follow-up period was, on average, 14 months to draw reliable conclusions regarding the local tumour control. No residual disease or recurrences were detected in the 16 patients that received a follow-up for more than 12 months. Only one of the cases presented tumour progression that was likely related to the large tumour size.
**Conclusion:** Cryoablation is a well-tolerated outpatient procedure. Nowadays, it offers a safe, effective alternative to surgery for women with benign breast tumours or patients with BC who are not surgical or chemotherapy candidates or who refuse surgery. Nevertheless, ongoing trials intending to demonstrate that cryoablation is an acceptable treatment for women with small BC are showing promising results.
**Limitations:** The follow-up period was, on average, 14 months to draw reliable conclusions.
**Ethics committee approval:** Not applicable.
**Funding for this study:** Not applicable.
**Author Disclosures:**
Lucia Graña Lopez: Nothing to disclose
Laura Abelairas López: Nothing to disclose
Ángeles Villares: Nothing to disclose
Patricia Arman Retana: Nothing to disclose

### RPS 402-5

**ICE STUDY - To detect cryoimmunologic response induced by ultrasound-guided Cryoablation on early breast cancer: preliminary results**

F. Galati, M. Pasculli, V. Rizzo, G. Moffa, A. Della Valle, *R. Maroncelli*, F. Cicciarelli, F. Pediconi; Rome/IT
*(roberto.maroncelli@uniroma1.it)*

**Purpose:** This pilot, the prospective, case-control study aims to characterize the cryoimmunologic response induced by US-guided cryoablation and to evaluate cryoablation treatment efficacy and safety in patients with early-stage breast cancer (BC) (T1 N0), not eligible for neo-adjuvant therapy and scheduled to breast surgery.
**Methods or Backround:** From July 2022, we recruited 13 women, 6 underwent cryoablation (Cryo-Group), and 7 underwent the same therapeutic pathway for the treatment of BC without performing cryoablation (Control-Group). Patients with a clearly visible lesion on ultrasound with a minimum distance of 1.5 cm between the tumour and the skin and 2 cm between the tumour and the nipple were enrolled in the Cryo-Group. Patients who did not meet these inclusion criteria were enrolled in the Control-Group.
**Results or Findings:** Cryoablation treatment was successful and effective in 5 (83.3 %) cancers without residual enhancement on post-cryoablation breast MR, while 10% residual tumour was detected in 1 patient (16.7 %). Immunogenic tumour cell death was found on surgical specimens of all Cryo-group patients, while in none of the Control-group patients.
**Conclusion:** Our results suggest that cryoablation is safe and effective in patients with early BC; this is an important first step in possibly replacing lumpectomy with cryoablation for this subset of BC patients. Moreover, BC cryoablation demonstrates the potential to generate immunogenic tumour cell death on surgical specimens, and this will open new therapeutic strategies for BC treatment.
**Limitations:** Our study includes a small number of patients; therefore, results need to be further validated in larger population studies.
**Ethics committee approval:** No information provided by the submitter.
**Funding for this study:** The 2022 ESR Research Seed Grants were kindly supported by an unrestricted, non-exclusive grant from GE Healthcare.
**Author Disclosures:**
Roberto Maroncelli: Nothing to disclose
Giuliana Moffa: Nothing to disclose
Veronica Rizzo: Nothing to disclose
Adriana Della Valle: Nothing to disclose
Francesca Galati: Nothing to disclose
Federica Cicciarelli: Nothing to disclose
Federica Pediconi: Nothing to disclose
Marcella Pasculli: Nothing to disclose

### RPS 402-6

**Multiple unilateral magnetic seeds for localisation in breast surgery: a single-centre retrospective analysis of radiological and surgical outcomes**

*I. Welaratne*, M. Al Azzawi, O. Kelly, E. R. Stanley, A. Heeney, M. Barry, M. Kell, M. Stokes, S. Walsh; Dublin/IE

**Purpose:** Magnetic seeds (Magseeds) are increasingly used for localising non-palpable breast lesions pre-operatively. For radiologists, the cost-effectiveness and reduced scheduling conflicts in comparison to wire localisation have led to an increase in recent years. While the outcomes of single Magseed-guided wide local excisions have been reported extensively, there is limited data on the outcomes for multiple unilateral Magseeds. The aim is to assess the radiological, operative and pathological outcomes of inserting multiple Magseeds in the same breast.
**Methods or Backround:** This is a retrospective analysis of all Magseed-guided excisions between January 2020 and September 2022 from a single centre for breast imaging (symptomatic and screening). The inclusion criteria required >1 unilateral Magseed. The primary outcomes comprised non-migration of the Magseed, successful excision of the lesion(s) and retrieval of the magnetic seed. The secondary outcomes included the distance between the Magseeds and the re-excision rate.
**Results or Findings:** A total of 1598 Magseeds were inserted between January 2020 and September 2022 at MMUH. Of these, 40 patients were included in the analysis; the mean age was 58.6 years. US-guided insertion was more common than stereotactic guidance. The mean duration from insertion to excision was 20.9 days. The mean distance between Magseeds was 48.3 and 42.5 mm on mediolateral and craniocaudal views, respectively. Bracket Magseeds were required in 9 cases. The remainder were for separate unilateral lesions. There was no Magseed migration post-insertion. All Magseeds and clips were retrieved within the final specimen; there were no reported difficulties with identifying both Magseeds intra-operatively or in the specimen radiograph. The re-excision rate was 15%. Only a single patient proceeded to a complete mastectomy.

**Conclusion:** Inserting two Magseeds is a feasible, safe and cost/time-effective alternative to wire-localisation when utilised in bracketing or for separate lesion excisions in unilateral breast surgery, as demonstrated by excellent radiological and surgical outcomes.
**Limitations:** No limitations were identified.
**Ethics committee approval:** No information provided by the submitter.
**Funding for this study:** No funding was received for this study.
**Author Disclosures:**
Malcolm Kell: Nothing to disclose
Mitchell Barry: Nothing to disclose
Mohammed Al Azzawi: Nothing to disclose
Siun Walsh: Nothing to disclose
Ivan Welaratne: Nothing to disclose
Anna Heeney: Nothing to disclose
Maurice Stokes: Nothing to disclose
Emma R Stanley: Nothing to disclose
Orlaith Kelly: Nothing to disclose

### RPS 402-7

**Idiopathic granulomatous mastitis: follow-up results of patients who underwent US-guided steroid injection**

*Ö. Aslan*, A. Taghiyeva, A. Oktay Alfatli, B. Goktepe; Izmir/TR
*(ozgeaorbay@hotmail.com)*

**Purpose:** Idiopathic granulomatous mastitis (IGM) is a benign inflammatory chronic breast disease. Its radiological features are variable. As a new approach in the treatment of IGM, local steroid injections are used instead of systemic steroids with side effects. We aimed to share the results of patients who had local steroid injections under US guidance in IGM.
**Methods or Backround:** We evaluated patients who received US-guided steroid injections between January 2021 and September 2022. Initial patient complaints, age, number of births, breastfeeding history, family history, laboratory values and smoking information were recorded. We evaluated baseline and follow-up US findings and the response to treatment.
**Results or Findings:** The median age of 23 patients was 31.6 years. The lesion was in the left breast in 61% of the patients. Findings in more than one quadrant were observed in 74% (n=17) of the patients. In all patients, the pathology results of core needle biopsy performed before treatment were granulomatous mastitis. All patients were ARB negative in pre-treatment laboratory analysis. Eight patients had received systemic steroid therapy for 4-6 weeks before local therapy. However, oral treatment could not be continued due to complications. We had performed the steroid injection under US guidance more than once, depending on the patient's response to treatment, and it was performed an average of 3.3 times in all patients. According to the follow-up radiological and clinical findings, 91.3% of the patients regressed almost completely.
**Conclusion:** IGM is a recurrent, chronic disease that impairs patients' daily quality of life. Instead of systemic treatment, good responses can be obtained with local steroid treatment, which is easy to apply and side effects in systemic treatment are not observed.
**Limitations:** Low number of patients.
**Ethics committee approval:** No information was provided by the submitter.
**Funding for this study:** No funding was received for this study.
**Author Disclosures:**
Özge Aslan: Nothing to disclose
Ayşenur Oktay Alfatli: Nothing to disclose
Aida Taghiyeva: Nothing to disclose
Berk Goktepe: Nothing to disclose

### RPS 402-8

**MRI depicted pregnancy-related changes in the residual breast tissue of post-mastectomy BRCA carriers**

*L. Gorenstein*, O. Kaidar-Person, D. Anaby, D. Shavin, R. Faermann, O. Halshtok, D. Samoocha, A. Shalmon, M. Sklair-Levy; Ramat Gan/IL

**Purpose:** BRCA1,2 genes confer an increased lifetime risk for breast cancer, 5-6 times compared with the general population. Prophylactic mastectomy provides BRCA carriers with a 90%-95% risk reduction. During pregnancy, there is an increase in breast tissue volume. Our objective was to explore the effects of pregnancy on the volume of residual breast tissue in post-mastectomy BRCA carriers as seen on MRI.
**Methods or Backround:** After IRB approval, a retrospective review of all BRCA carriers who were followed at our institution between 20212-2021 was conducted. Only patients who became pregnant after mastectomy (prophylactic or therapeutic) were included in our study. Breast MRIs before and after pregnancy were reviewed. MRI residual breast tissue width (BTW) was measured by two radiologists, and volumetric calculation was performed using an AI-based algorithm. (T-test, $P<0.05$).
**Results or Findings:** A total of 33 women were included, 27 were BRCA 1, 20 women underwent a therapeutic mastectomy, and 13 had prophylactic mastectomies. The maximum post-pregnancy BTW was 48.4mm in the oncological cohort and 52.9mm in the prophylactic mastectomy group. In the prophylactic group, an average increase of $3.5 \pm 4.51$mm, $P<0.05$. In the breast cancer cohort, an average increase in BTW of $1.618 \pm 2.66$mm, $P<0.05$. The average volumetric increase in the oncological cohort was 57,389±110,761mm3, an average increase of 12%($P<0.05$).
**Conclusion:** Mastectomy aims to remove all breast glandular tissue. Our study shows that there is residual breast tissue after mastectomy, and the amount increases after pregnancy. The correlation with increased risk of breast cancer recurrence or occurrence should be further studied.
**Limitations:** A retrospective and single-centre study.
**Ethics committee approval:** No information provided by the submitter.
**Funding for this study:** No funding was received for this study.
**Author Disclosures:**
Debbie Anaby: Nothing to disclose
Larisa Gorenstein: Nothing to disclose
Miri Sklair-Levy: Nothing to disclose
David Shavin: Nothing to disclose
Orit Kaidar-Person: Nothing to disclose
David Samoocha: Nothing to disclose
Renata Faermann: Nothing to disclose
Osnat Halshtok: Nothing to disclose
Anat Shalmon: Nothing to disclose

### RPS 402-9

**Image quality of accelerated DWI in 3T breast MRI with deep learning reconstruction**

*C. Wilpert*, S. Hannah, M. Reisert, A. Rau, C. Neubauer, F. Bamberg, M. Windfuhr-Blum, M. Benndorf, J. Neubauer; Freiburg im Breisgau/DE
*(caroline.wilpert@uniklinik-freiburg.de)*

**Purpose:** Deep learning (DL) reconstruction of DWI is a novel method that allows for a substantially faster acquisition time than conventional DWI. In this study, we evaluate the image quality of DL-DWI in direct comparison to standard DWI.
**Methods or Backround:** A prospective pseudo-anonymized randomized study with the inclusion of 37 patients with a mean age of 53 years (range: 29–85) who underwent 3T breast MRI with an 18-channel breast coil. A standard DWI and DWI with DL reconstruction with b-values of 50 and 800 s/mm2 were performed in addition to a standard diagnostic breast protocol, yielding an interpolated 0.6x0.6x3.0 mm spatial resolution for DL-DWI. Image quality was evaluated ROI-based for SNR. Two radiologists rated overall image quality, artefacts and lesion conspicuity separately using 5-point Likert scales to assess qualitative image features.
**Results or Findings:** Acquisition time was 05:02 min for standard DWI and 02:44 min for DL-DWI. SNR was statistically significantly higher for standard DWI compared to DL-DWI ($p < 0.01$). Both raters were able to detect DL-DWI with high certainty. When both sequences were compared intra-individually, even though standard DWI was rated towards the higher image quality score, both sequences revealed a high image quality. A higher lesion conspicuity score was observed for DL-DWI. Artefacts were observed without statistically significant differences in both sequences similarly.
**Conclusion:** DL-DWI shows a drastically improved acquisition time of 46% compared to standard DWI in 3T breast MRI. Even though SNR was higher in standard DWI and standard DWI was rated towards the higher scores, image quality remained good for DL-DWI, and no additional artefacts were observed. Therefore, DL-DWI seems feasible.
**Limitations:** Only a small number of patients were evaluated. Motion artefacts might have influenced the outcome of qualitative analysis.
**Ethics committee approval:** EK 22-1185
**Funding for this study:** Internal funding.
**Author Disclosures:**
Caroline Wilpert: Nothing to disclose
Jakob Neubauer: Nothing to disclose
Marco Reisert: Nothing to disclose
Alexander Rau: Nothing to disclose
Claudia Neubauer: Nothing to disclose
Matthias Benndorf: Nothing to disclose
Fabian Bamberg: Nothing to disclose
Marisa Windfuhr-Blum: Nothing to disclose
Schneider Hannah: Nothing to disclose

### RPS 402-10

**Axillary disease extent on baseline 18F-FDG PET/CT and its influence on the accuracy of axillary surgical staging after NCT in clinically node-positive breast cancer**

C. M. de Mooij[1], J. Simons[2], C. Mitea[1], P. Nelemans[1], C. van der Pol[3], E. Luiten[4], L. Koppert[2], M. Smidt[1], *T. van Nijnatten*[1]; [1]Maastricht/NL, [2]Rotterdam/NL, [3]Leiderdorp/NL, [4]Al Ain/AE
*(Thiemo.nijnatten@mumc.nl)*

**Purpose:** Clinically node-positive (cN+) breast cancer patients increasingly undergo axillary surgical staging after neoadjuvant chemo(targeted) therapy (NCT). The RISAS procedure combines the sentinel lymph node biopsy

(SLNB) with the excision of axillary lymph nodes pre-NCT marked with a radioactive iodine seed (MARI) after NCT. The impact of axillary disease extent on 18F-FDG PET/CT prior to NCT on the false negative rate (FNR) and negative predictive value (NPV) of the RISAS procedure is investigated.
**Methods or Backround:** After NCT, pathologically confirmed cN+ patients underwent axillary surgical staging with the RISAS procedure (i.e. combined SLNB and MARI) followed by a completion axillary lymph node dissection. The FNR and NPV of the SLNB, MARI-procedure and RISAS procedure were compared between patients with limited and advanced axillary disease (1-3 vs ≥4 suspicious axillary lymph nodes) on baseline 18F-FDG PET/CT prior to NCT by means of Fisher's exact test.
**Results or Findings:** Axillary pathologic complete response occurred in 55/185 patients. Prior to NCT, 116 patients had limited and 69 advanced baseline axillary disease. The FNR of the RISAS procedure (1.3% vs 7.8%, P = 0.077), MARI-procedure (6.8% vs 9.8%, P = 0.739), and SLNB (16.4% vs 27.0%, P = 0.213) is lower, and the NPV of the RISAS-procedure (97.4% vs 81.8%, P = 0.056), MARI-procedure (87.5% vs 77.3%, P = 0.307), and SLNB (73.3% vs 63.0%, P = 0.431) higher, in limited baseline axillary disease compared to advanced baseline axillary disease.
**Conclusion:** Stratification of baseline axillary disease extent on 18F-FDG PET/CT has an insignificant effect on the accuracy of the RISAS procedure in cN+ patients after NCT.
**Limitations:** Not applicable.
**Ethics committee approval:** The local medical ethical review committees of all participating institutions waived the necessity to acquire informed consent due to the retrospective study design.
**Funding for this study:** Funding was received from Dutch Cancer Society (REFINE-trial; project 14055), University Fund Limburg (SWOL; project 20.048)
**Author Disclosures:**
Marjolein Smidt: Research/Grant Support: Servier
Cornelis Maarten de Mooij: Nothing to disclose
Patty Nelemans: Nothing to disclose
Cristina Mitea: Nothing to disclose
Janine Simons: Nothing to disclose
Linetta Koppert: Nothing to disclose
Thiemo van Nijnatten: Research/Grant Support: University Fund Limburg (SWOL; project 20.048) Research/Grant Support: Dutch Cancer Society (REFINE-trial; project 14055)
Ernest Luiten: Nothing to disclose
Carmen van der Pol: Nothing to disclose

### RPS 402-11

### Semiquantitative score of breast arterial calcifications on mammography (BAC-SS): reproducibility among 11 readers

*D. Capra*[1], R. M. Trimboli[1], L. A. Carbonaro[1], S. Schiaffino[2], C. G. Monaco[1], A. Cozzi[1], S. Carriero[1], C. B. Monti[1], F. Sardanelli[1]; [1]Milan/IT, [2]Lugano/CH

**Purpose:** Breast arterial calcifications (BAC) are associated with cardiovascular risk in the female population. We investigated the inter-reader reproducibility of a fast, semiquantitative score for BAC assessment on mammography.
**Methods or Backround:** Eleven readers with different experience in breast imaging (1-36 years) assessed BAC on a set of 60 four-view mammograms (30 BAC+ and 30 BAC-). The semiquantitative score for BAC (BAC-SS) was defined as the sum of 1) complete calcium coverage of the artery (0/1); and 2) length class of calcified vessels (0-4) on medio-lateral oblique views. The quartiles of BAC length distribution were set as threshold values, thus defining four length classes. A panel majority report (PMR, the mode of the scores for each case) was established as reference standard. Cohen and Fleiss κ statistics were used to evaluate pairwise and overall agreement, respectively. Correlations between each reader's agreement with PMR and their experience were assessed with Spearman's ρ test.
**Results or Findings:** Overall, readers showed substantial agreement on calcium density, length assessment, and overall score (κ=0.648, κ=0.713, and κ=0.663, respectively). The agreement was higher for higher BAC scores, reaching κ=0.714 for BAC-SS=5. The agreement of each reader with PMR ranged from κ=0.545 to κ=0.883, with a mean of κ=0.775 and a standard deviation (SD) of 0.096, whereas each reader pair ranged between κ=0.411 and κ=0.809, with a mean of κ=0.663 and an SD of 0.096. No correlation was found between readers' agreement and their experience (p=0.600). The mean application time for BAC+ cases was 143±104 s.
**Conclusion:** BAC-SS showed a substantial agreement over a panel of 11 readers with wide differences in experience, with assessment times compatible with everyday clinical practice.
**Limitations:** Single-centre study.
**Ethics committee approval:** Ethics Committee of IRCCS Ospedale San Raffaele; protocol code SenoRetro; approved on November 9th, 2017 and amended on July 18th, 2019.
**Funding for this study:** No funding was received for this study.
**Author Disclosures:**
Andrea Cozzi: Nothing to disclose
Luca Alessandro Carbonaro: Nothing to disclose
Francesco Sardanelli: Research/Grant Support: Bayer Research/Grant Support: General Electric Advisory Board: Bracco Speaker: Bayer Speaker: Bracco Advisory Board: General Electric Speaker: General Electric Advisory Board: Bayer Research/Grant Support: Bracco
Cristian Giuseppe Monaco: Nothing to disclose
Caterina Beatrice Monti: Nothing to disclose
Serena Carriero: Nothing to disclose
Simone Schiaffino: Nothing to disclose
Rubina Manuela Trimboli: Nothing to disclose
Davide Capra: Nothing to disclose

### RPS 402-12

### Is there a correlation between body composition parameters and prognosis in patients with metastatic breast cancer treated with cyclin-dependent kinase (CDK) 4/6 inhibitors?

*M. Pasculli*, F. Pediconi, F. Galati, V. Rizzo, G. Moffa, R. Maroncelli, F. Cicciarelli; Rome/IT
*(marcella1.pasculli@gmail.com)*

**Purpose:** To investigate the association between body composition parameters, sarcopenia, obesity and prognosis in patients with metastatic ER+/HER2- breast cancer treated with cyclin-dependent kinase (CDK) 4/6 inhibitors.
**Methods or Backround:** Patients with biopsy-proven metastatic ER+/HER2- breast cancer treated with CDK 4/6 inhibitors between 2018-2021 were included in this retrospective study. Visceral Adipose Tissue (VAT), Subcutaneous Adipose Tissue (SAT) and Skeletal Muscle Index (SMI) were measured before starting targeted therapy. Measurements were performed on computed tomography-derived abdominal imaging by dedicated software (Quantib body composition®, Rotterdam, Netherlands). Visceral obesity was defined as a VAT area > 130 cm2, and sarcopenia as SMI < 40 cm2/m2. Changes in breast lesion size were evaluated after six months of treatment, according to RECIST 1.1 criteria. Statistical analysis was performed using Spearman's correlation and χ2 tests.
**Results or Findings:** Thirty patients were included in the evaluation: 7 patients were sarcopenic, 16 were obese, while seven patients were neither sarcopenic nor obese. A good response to therapy was correlated with higher SMI values (p < 0.001), higher VAT values (p = 0.008) and obesity (p = 0.007); poor response to therapy was correlated to sarcopenia (p < 0.001). Moreover, there was a significant association between sarcopenia and menopause (p = 0.021) and between sarcopenia and the persistence of axillary lymphadenopathies after treatment (p = 0.003), while the disappearance of axillary lymphadenopathies was associated with obesity (p = 0.028).
**Conclusion:** Our results have shown an interesting correlation between sarcopenia and the progression of the disease and have demonstrated that VAT can positively influence the response to targeted therapy with CDK 4/6 inhibitors.
**Limitations:** Retrospective, single-center study, with a small population.
**Ethics committee approval:** No information provided by the submitter.
**Funding for this study:** No funding for this study.
**Author Disclosures:**
Roberto Maroncelli: Nothing to disclose
Giuliana Moffa: Nothing to disclose
Veronica Rizzo: Nothing to disclose
Francesca Galati: Nothing to disclose
Marcella Pasculli: Nothing to disclose
Federica Cicciarelli: Nothing to disclose
Federica Pediconi: Nothing to disclose

### RPS 402-13

### Comparing perceived breast lesion characterisation difficulty to actual radiologist performance: results from the ECR & EUSOBI perception labs

*J. Gommers*, S. D. Verboom, M. Caballo, M. Broeders, I. Sechopoulos; Nijmegen/NL
*(jessie.gommers@radboudumc.nl)*

**Purpose:** To investigate how the perceived difficulty of breast lesion characterization in mammography relates to the accuracy of lesion interpretation.
**Methods or Backround:** Observer studies were performed at the 2022 editions of ECR and EUSOBI. Each participating radiologist scored approximately 100 unilateral two-view screening mammograms, with every case containing one annotated mass, in terms of recall decision and the difficulty of making this decision (score 1-10). Every case was scored by at least nine radiologists to obtain a mean case-interpretation accuracy and perceived case difficulty. These two metrics were compared separately for benign and malignant masses. Furthermore, the correlation between mean perceived difficulty with recall rate and reading time was investigated.

**Results or Findings:** Forty-five radiologists, of which 33 had > 1-year mammography-reading experience, read a total of 396 cases (46.7% malignant). For benign cases, cases perceived easy (difficulty<4.0, N=85) resulted in a median accuracy of 40% (IQR: 20.0-68.6%), which was lower than that for cases perceived difficult (N=126), which had a median accuracy of 50% (IQR: 30.0-66.7%) (P=0.022). In contrast, for malignant cases, easy cases (difficulty<4.0, N=110) had a higher median accuracy (100%, IQR: 90.0-100.0%) compared to the difficult ones (N=75; 67%, IQR: 47.7-80.0%) (P<0.001). Overall, ROC analyses showed a higher AUC of 0.84 (95% CI: 0.79-0.88) for perceived-easy cases (difficulty<4.0, N=195), compared to perceived-difficult cases (N=201) with an AUC of 0.64 (95% CI: 0.57-0.72) (P<0.001). The perceived difficulty was negatively correlated with recall rate (r=-0.46, P<0.001) and positively correlated with reading time (r=0.43, P<0.001).
**Conclusion:** Perceived high mass characterization difficulty resulted in lower sensitivity and slightly higher specificity. Radiologists had longer reading times and a lower recall rate when cases were perceived as being difficult.
**Limitations:** Performed with enriched case sets and without priors.
**Ethics committee approval:** No information provided by the submitter.
**Funding for this study:** Funding was received from aiREAD financed by NWO, KWF, HH.
**Author Disclosures:**
Mireille Broeders: Research/Grant Support: with Lunit inc., Screenpoint Medical, Sectra Benelux, Hologic, Volpara Solutions, iCAD Speaker: Hologic, Siemens Healthcare
Marco Caballo: Nothing to disclose
Sarah Delaja Verboom: Nothing to disclose
Jessie Gommers: Nothing to disclose
Ioannis Sechopoulos: Research/Grant Support: Siemens Healthcare, Canon Medical, ScreenPoint Medical, Sectra Benelux, Volpara Healthcare, Lunit, iCAD Speaker: Siemens Healthcare

**13:00-14:30** **Research Stage 3**

### Research Presentation Session: Musculoskeletal

### RPS 410

### Musculoskeletal cutting edge: artificial intelligence, photon counting CT, and more

**Moderator**
M. Maas; Amsterdam/NL
*(m.maas@amsterdamumc.nl)*

### RPS 410-2

**Microstructural imaging of bone by photon-counting computed tomography**

*A. Korajac*[1], A. Burghardt[2], A. E. Strassl[1], J. M. Patsch[1]; [1]Vienna/AT, [2]San Francisco, CA/US
*(aida.korajac@meduniwien.ac.at)*

**Purpose:** To compare high-resolution peripheral quantitative computed tomography (HR-pQCT) and photon-counting computed tomography (PCCT) regarding image quality, quantitative parameters (geometry, density, microstructure, strength) and radiation dose in anthropomorphic microstructure-realistic imaging phantoms that have served as key-tool for the cross-calibration for multi-centre precision analysis, developed by A.J. Burghardt et al..
**Methods or Backround:** The UCSF bone structure phantoms were imaged by µCT (µCT-100, Scanco Medical), first and second-generation HR-pQCT (XtremeCT, XtremeCT-IIb, Scanco Medical), and PCCT (Naeotom Alpha, Siemens Healthineers). PCCT scans were acquired using 120kVp in ultra-high resolution (UHR) mode. A 15 mm FOV was reconstructed across 1024x1024 matrix using the Br92 kernel (146µm pixel size and 200µm slice thickness). To evaluate noise-dose performance, scans were collected with effective mAs spanning 50 to 450 mAs. Spatial resolution (10% MTF) and noise performance (SNR) were measured in idealised bone density and wire phantoms.
**Results or Findings:** PCCT noise performance (SNR=12.1-29.8) was superior to second generation HR-pQCT noise (SNR=7.1) at all dose levels, and equivalent to first-generation HR-pQCT (SNR=15.4) at 100mAs. The spatial resolution of PCCT (10% MTF = 132 µm) was comparable to first generation HR-pQCT (10% MTF = 138 µm), and inferior to second-generation HR-pQCT (10% MTF = 95 µm). Scan time for each phantom by PCCT was <10s, compared to 3.2 and 2.0 minutes for first and second-generation HR-pQCT.
**Conclusion:** PCCT provides substantial advantages over current cone-beam skeletal imaging systems, including superior noise performance and scan time, while spatial resolution is comparable to first-generation HR-pQCT. Given the potential for wide availability and existing regulatory approval, PCCT represents an exciting opportunity to finally translate quantitative microstructural skeletal imaging to a clinical platform.
**Limitations:** The impossibility to use mAs higher than 600, an internal limitation of PCCT itself.
**Ethics committee approval:** No information provided by the submitter.
**Funding for this study:** No funding was received for this study.
**Author Disclosures:**
Janina Maria Patsch: Nothing to disclose
Andreas Eduard Strassl: Nothing to disclose
Aida Korajac: Nothing to disclose
Andrew Burghardt: Nothing to disclose

### RPS 410-3

**Photon-counting computed tomography (PC-CT) of the spine: impact on diagnostic confidence and radiation dose**

A. Rau[1], J. Straehle[1], K. Nikolaou[2], S. O. Schönberg[3], J. Beck[1], H. Urbach[1], J-H. Klingler[1], F. Bamberg[1], *J. Weiß*[1]; [1]Freiburg/DE, [2]Tübingen/DE, [3]Mannheim/DE
*(j_b_weiss@me.com)*

**Purpose:** Computed tomography (CT) is the modality of choice to evaluate surgical outcome after spinal interventions. We investigate the potential of multispectral photon-counting computed tomography (PC-CT) on image quality, diagnostic confidence, and radiation dose compared to an energy-integrating CT (EID-CT).
**Methods or Backround:** In this prospective study, 32 patients were referred for PC-CT of the spine. Two series were reconstructed: 1) standard bone kernel (PC-CTstd), and 2) 130 keV monoenergetic images (PC-CT130keV). Prior EID-CT of the spine was available for 17 patients; for the remaining 15, a matched EID-CT cohort was identified. Image quality (overall, sharpness, artifacts, noise, diagnostic confidence) of PC-CTstd and EID-CT was assessed by four radiologists independently. If metallic implants were present (n=10), PC-CTstd and PC-CT130keV images were evaluated. HU were measured within artifact and compared between PC-CTstd and PC-CT130keV. The radiation dose (CTDIvol) was evaluated.
**Results or Findings:** Among the 32 patients (mean age 58.8 years; 18 male), sharpness was rated significantly higher (p=0.009) and noise significantly lower (p<0.001) in PC-CTstd. In the subset of patients with metallic implants, reading scores for PC-CT130keV revealed superior ratings vs. PC-CTstd for overall image quality, artifacts, noise and diagnostic confidence (all p<0.001) accompanied by a significant increase of HU values within the artifact (p<0.001). Radiation dose was significantly lower for PC-CT vs. EID-CT (mean CTDIvol: 8.83 vs. 15.7 mGy; p<0.001).
**Conclusion:** PC-CT of the spine with high keV reconstructions provides significantly sharper images and higher diagnostic confidence in patients with metallic implants while radiation dose was significantly reduced.
**Limitations:** One limitation is that an EID comparison examination is not available for the entire cohort.
**Ethics committee approval:** Ethics committee University of Freiburg.
**Funding for this study:** This project is funded by the Baden-Württemberg Ministry of Economic Affairs, Labor and Tourism as part of the “Forum Gesundheitsstandort Baden-Württemberg”, number: 35-4223.10/20.
**Author Disclosures:**
Jürgen Beck: Nothing to disclose
Jan-Helge Klingler: Nothing to disclose
Konstantin Nikolaou: Nothing to disclose
Stefan Oswald Schönberg: Nothing to disclose
Alexander Rau: Nothing to disclose
Horst Urbach: Nothing to disclose
Fabian Bamberg: Nothing to disclose
Jakob Weiß: Nothing to disclose
Jakob Straehle: Nothing to disclose

### RPS 410-4

**Earlier non-invasive diagnosis of gout and calcium pyrophosphate deposition disease with multi-energy photon-counting CT**

*A. Viry*[1], F. Becce[1], F. R. Verdun[1], G. Filippou[2], J. M. M. Froehlich[3], T. Pascart[4], J. Damet[1], L. Gallego Manzano[1]; [1]Lausanne/CH, [2]Milan/IT, [3]Zurich/CH, [4]Lille/FR

**Purpose:** We aimed to assess whether photon-counting CT (PCCT) can identify smaller deposits of monosodium urate (MSU) and calcium pyrophosphate (CPP) than dual-energy CT (DECT) for the diagnosis of gout and calcium pyrophosphate deposition (CPPD) disease.
**Methods or Backround:** We used a soft-tissue phantom with six synthetic crystal inserts with known concentrations of MSU (90-500 mg/mL) and CPP (26-109 mg/mL). Crystals were suspended in a crystal-free agar background densified with polysaccharides to mimic the attenuation of articular cartilage.

The phantom was scanned using a DECT (GE APEX) operated at 70 and 140 kVp and a PCCT (MARS Extremity 5X120, Mars Bioimaging Ltd.) at 118 kVp using a comparable dose level of 3 mGy. Images were reconstructed using a voxel size of 0.22×0.22×0.625 mm3 for DECT (GE APEX) and 0.1×0.1×0.1 mm3 for PCCT. The area under the receiver operating characteristic curves (AUCs) were computed for the MSU/CPP and MSU/crystal-free background pairs. We also calculated the minimum pixel sample size required to distinguish two crystal types within a crystal deposit.
**Results or Findings:** The performance of PCCT in identifying MSU and CPP was excellent (AUC=0.964+/-0.022) and comparable to DECT (AUC=1). For the identification of MSU in a crystal-free background, PCCT (AUC=0.979+/-0.013) outperformed DECT (AUC=0.799+/-0.16). The minimum pixel sample size required to distinguish MSU and CPP decreased from 3 pixels (0.6-mm diameter) with DECT to 2 pixels (0.2-mm diameter) with PCCT. To differentiate MSU from the crystal-free background, both systems needed minimum 4 pixels (0.8-mm diameter for DECT vs. 0.4-mm for PCCT).
**Conclusion:** PCCT can identify smaller volumes of MSU and CPP deposits with comparable accuracy to DECT. PCCT could improve the diagnosis of gout and CPPD with a higher spatial resolution.
**Limitations:** It remains a phantom study.
**Ethics committee approval:** No information provided by the submitter.
**Funding for this study:** Funded by SNFNS 320030_212764.
**Author Disclosures:**
Tristan Pascart: Nothing to disclose
Anaïs Viry: Nothing to disclose
Johannes Malte Maria Froehlich: Nothing to disclose
Lucia Gallego Manzano: Nothing to disclose
Georgios Filippou: Nothing to disclose
Fabio Becce: Nothing to disclose
Francis R. Verdun: Nothing to disclose
Jérôme Damet: Nothing to disclose

### RPS 410-5

**Protocol optimisation for metal artifact reduction in wrist imaging using photon-counting detector CT**

*N. F. Kämmerling*, M. Sandstedt, L. Henriksson, R. Booij, S. Farnebo, A. Persson, E. Tesselaar; Linkoping/SE

**Purpose:** The aim of this study was to optimise the source spectrum and reconstruction parameters with respect to metal artifact reduction (MAR) in wrist imaging using photon-counting detector CT (PCD-CT) and to compare the image quality with ultra-high-resolution (UHR) energy-integrating detector CT (EID-CT).
**Methods or Backround:** Two wrist specimens with commonly used implants containing titanium, stainless steel and cobalt-chrome-molybdenum were scanned with EID-CT and PCD-CT at equal radiation dose using different source spectra. Two experienced radiologists assessed the effect of reconstruction parameters including kernel strength, iterative metal artifact reduction (iMAR) and virtual monochromatic images (VMI) on the visibility of the bone-metal interface and the bone architecture in general using a 7-point Likert scale. Beam hardening and blooming artifacts were measured quantitatively in the image data.
**Results or Findings:** The visibility of the metal-bone interface and the trabecular bone structure was considered superior in images obtained with PCD-CT using 120kV (median rating 5.5) and 140kV (median 5.5) compared with EID-CT at 150kV with tin prefiltration (median 2.5, p=0.002). With PCD-CT, tin prefiltration reduced beam hardening and blooming artefacts, but image quality was rated lower by the radiologists (median 3, no difference from EID-CT, p=0.85). iMAR (median 1) and VMI (median 2) yielded lower image quality than energy-weighted reconstructions or EID-CT.
**Conclusion:** In PCD-CT of wrists, the best image quality in the presence of commonly used metal implants was obtained using 120kV or 140kV using a Br89 kernel and 0.2 mm slice thickness. Tin prefiltration, iMAR and VMI did not benefit image quality in the presence of metal, possibly due to the narrower source spectrum and the limitations of iMAR algorithm (limited kernel sharpness) and VMI (minimal slice thickness 0.4 mm).
**Limitations:** The effect of motion artifacts was not considered.
**Ethics committee approval:** No information provided by the submitter.
**Funding for this study:** Not applicable.
**Author Disclosures:**
Simon Farnebo: Nothing to disclose
Anders Persson: Research/Grant Support: Siemens Healthineers Advisory Board: Siemens Healthineers
Nina Fredäng Kämmerling: Nothing to disclose
Erik Tesselaar: Nothing to disclose
Mårten Sandstedt: Nothing to disclose
Ronald Booij: Nothing to disclose
Lilian Henriksson: Nothing to disclose

### RPS 410-6

**Automatic classification of the endplate lesions by machine learning approach**

*P. Glielmo*[1], T. Bassani[1], L. M. Sconfienza[1], D. Albano[1], A. Cina[2], F. Galbusera[2], A. Colombini[1], A. Luca[1], M. Brayda-Bruno[1]; [1]Milan/IT, [2]Zurich/CH
*(pierluigiglielmo@gmail.com)*

**Purpose:** An increasing number of studies have applied artificial intelligence techniques to predict parameters from biomedical images, with the aim of providing reproducible and reliable evaluation. As regards the MRI scan of the lumbar spine, a novel classification scheme for endplate lesions, based on T2-weighted images, has been recently introduced. In this scheme, the intervertebral spaces are classified as 'normal', 'wavy/irregular', 'notched', and 'Schmorl's node'. The present study exploits a machine learning application based on convolutional neural networks to automatically classify the type of lesion.
**Methods or Backround:** T2-weighted MRI scans of the sagittal thoraco-lumbar spine of 300 consecutive patients were retrospectively collected (age ranging from 18 to 65 years). The middle slice was manually processed to identify the intervertebral spaces from T12L1 to L5S1, with labelling of corresponding lesion type. A total number of 1559 gradable discs was obtained, distributed as follows: 'normal' (567 discs), 'wavy/irregular' (485), 'notched' (362), and 'Schmorl's node' (145). Training- and validation-set (1248 and 311 discs, respectively) were randomly defined. Pre-trained network for image classification was exploited. Fine-tuning by exploiting the training-set was performed. The validation-set was processed with the retrained net to evaluate the accuracy as percentage of correct predictions.
**Results or Findings:** Overall accuracy was 0.73. Accuracy for the specific lesion type was 0.96 (normal), 0.63 (wavy/irregular), 0.68 (notched), and 0.24 (Schmorl's node).
**Conclusion:** Automatic classification by machine learning approach provided moderate accuracy overall. The percentage of correct predictions was excellent for the most represented type (normal), but decreased in the other groups, with poor accuracy for the least represented one (Schmorl's node). Larger datasets with balanced groups need to be accounted for in the next steps to improve the performance of the net.
**Limitations:** Relatively limited cases of Schmorl's nodes.
**Ethics committee approval:** No information provided by the submitter.
**Funding for this study:** No funding was received for this study.
**Author Disclosures:**
Andrea Cina: Nothing to disclose
Pierluigi Glielmo: Nothing to disclose
Andrea Luca: Nothing to disclose
Luca Maria Sconfienza: Nothing to disclose
Fabio Galbusera: Nothing to disclose
Marco Brayda-Bruno: Nothing to disclose
Tito Bassani: Nothing to disclose
Alessandra Colombini: Nothing to disclose
Domenico Albano: Nothing to disclose

### RPS 410-7

**Deep learning MRI reconstruction to accelerate turbo spin-echo hip imaging: a comparison of image quality and visualisation of anatomy with standard imaging**

*J. Herrmann*, S. Gassenmaier, H. Almansour, S. Afat, S. Werner; Tübingen/DE
*(judith.herrmann@med.uni-tuebingen.de)*

**Purpose:** To implement a deep learning (DL) reconstruction for turbo spin echo (TSE) sequences of the hip and determine its feasibility in routine clinical practice, and to further evaluate image quality, delineation of anatomical structures, and diagnostic performance compared to standard TSE imaging.
**Methods or Backround:** First, the DL reconstruction for TSE (TSE_DL) was implemented for hip MRI and feasibility determined through a prospective volunteer study with 9 participants at 3 T (mean age 30±8; 5 women, 4 men). Second, the TSE_DL was implemented clinically and evaluated in 14 patients (mean age 46±13; 7 women, 7 men) undergoing a clinically indicated hip MRI at 1.5 and 3T between October 2020 and May 2021. Two radiologists assessed the TSE_DL and standard sequences (TSE_S) regarding overall image quality, noise, edge sharpness, artifacts, and diagnostic confidence as well as the delineation of anatomical structures using a Likert scale ranging from 1 to 5 (5 = best). Comparative analyses were conducted to assess the differences between TSE_DL and routinely used TSE_S.
**Results or Findings:** Overall image quality was evaluated to be superior in TSE_DL versus TSE_S (p<0.001). Noise and edge sharpness were evaluated to be significantly superior in TSE_DL versus TSE_S (p<0.05). No difference was found concerning qualitative diagnostic confidence and delineation of anatomical structures (p>0.05) when comparing the two sequences. Using TSE_DL can reduce scan time to 3:57 min compared to 13:08 min for a native four-plane, multi-contrast hip MRI at 1.5 T.

**Conclusion:** The fast TSE_DL MRI protocol of the hip is feasible in routine clinical practice, with comparable diagnostic performance, allowing a reduction in scan time of about 70 % compared to the standard TSE_S protocol.
**Limitations:** Single-centre study, small sample size.
**Ethics committee approval:** University of Tuebingen
**Funding for this study:** No external funding was recieved.
**Author Disclosures:**
Judith Herrmann: Nothing to disclose
Saif Afat: Nothing to disclose
Haidara Almansour: Nothing to disclose
Sebastian Gassenmaier: Nothing to disclose
Sebastian Werner: Nothing to disclose

### RPS 410-8

**Deep learning convolutional neural network reconstruction and radial k-space acquisition MR technique to increase detection of retropatellar cartilage lesions of the knee joint**

M. Kaniewska, E. Deininger-Czermak, M. Lohezic, F. Ensle, *R. Guggenberger*; Zurich/CH

**Purpose:** To assess diagnostic performance of standard radial k-space (PROPELLER) MRI sequences and compare with accelerated acquisitions combined with a deep learning-based convolutional neural network (DL-CNN) reconstruction for evaluation of the knee joint.
**Methods or Backround:** 35 MRIs of the knee at 1.5T were prospectively included. Two readers evaluated image quality and diagnostic confidence of standard and DL-CNN accelerated PROPELLER MR sequences using a four-point Likert scale. Pathological findings of bone, cartilage, cruciate and collateral ligaments, menisci, and joint space were analysed. Inter-reader agreement (IRA) for image quality and diagnostic confidence was assessed using intraclass correlation coefficients (ICC). Cohen's kappa statistic was applied for assessment of IRA and agreement between MR sequences for pathological findings. Image quality was quantitatively evaluated by signal-to-noise ratio (SNR) and contrast-to-noise ratio (CNR).
**Results or Findings:** Mean scan time of standard vs. DL-CNN sequences was 10 minutes 3 seconds vs. 4 minutes 45 seconds. DL-CNN sequences showed significantly superior image quality and diagnostic confidence compared to standard MR sequences. There was moderate and good IRA for assessment of image quality in standard and DL-CNN sequences with ICC of 0.524 and 0.830. Pathological findings of the knee joint could be equally well detected in both sequences (k=0.8). Retropatellar cartilage could be significantly better assessed on DL-CNN sequences. SNR and CNR was significantly higher for DL-CNN sequences ($p<0.05$).
**Conclusion:** DL-CNN sequences showed significantly higher image quality and diagnostic confidence compared to standard PROPELLER sequences while reducing scan time substantially. Both sequences perform comparably in the detection of knee joint pathologies, while DL-CNN sequences are superior for evaluation of retropatellar cartilage lesions.
**Limitations:** Small pilot study including 35 patients. No arthroscopic reference standard for the different joint pathologies detected by MRI.
**Ethics committee approval:** Kantonale Ethikkommission. Stampfenbachstrasse 121, 8090, Zürich, Switzerland.
**Funding for this study:** No funding was received for this study.
**Author Disclosures:**
Maelene Lohezic: Nothing to disclose
Falko Ensle: Nothing to disclose
Roman Guggenberger: Advisory Board: Aison Company
Eva Deininger-Czermak: Nothing to disclose
Malwina Kaniewska: Nothing to disclose

### RPS 410-9

**Image quality and lesion detectability of deep learning-accelerated dixon imaging in the cervical spine**

*G. Seo*, S. J. Lee; Busan/KR
*(soya1115@naver.com)*

**Purpose:** The purpose of this study was to validate the subjective image quality and lesion detectability of deep learning-accelerated Dixon (DL-Dixon) images in the cervical spine compared with standard Dixon images.
**Methods or Backround:** A total of 50 patients underwent sagittal standard Dixon and DL-Dixon images of the cervical spine. Acquisition parameters, including the acquisition time, were compared, and non-uniformity (NU) values were calculated. Two radiologists independently assessed the two imaging techniques for subjective image quality and lesion detectability. Interreader and intermethod agreements were estimated with weighted kappa values.
**Results or Findings:** Compared with the standard Dixon images, the DL-Dixon images reduced the acquisition time by 23.76% (from 181 s to 138 s). There was no significant difference in NU between Dixon images and DL-Dixon images (p value: 0.172). DL-Dixon images showed superior visibility of all four anatomic structures (spinal cord, disc margin, dorsal root ganglion, and facet joint) for both readers (p value< 0.001~0.002). Intermethod agreements were almost perfect for disc herniation, facet osteoarthritis, uncovertebral arthritis, central canal stenosis (kappa values ranged 0.830~0.980 for both readers; all p values < 0.001) and substantial to almost perfect for foraminal stenosis (kappa value: 0.955, 0.705 for each reader). There was an improvement in the interreader agreement of foraminal stenosis by DL-Dixon images, from moderate (kappa value: 0.596) to substantial agreement (kappa value: 0.760).
**Conclusion:** DL-Dixon images may improve overall image quality as well as the confidence of lesion detectability by improving interreader agreement, with a shortened acquisition time than that of standard Dixon images.
**Limitations:** First, the detection bias might be present. Second, the current study didn't consider the characteristic artifact in the DL-Dixon image. Last, we didn't compare our evaluation to gold standards, such as operation records.
**Ethics committee approval:** This study was approved by the institutional review board (IRB file No. BPIRB 2022-05-005) from the centre.
**Funding for this study:** No funding was received for this study.
**Author Disclosures:**
Sun Ju Lee: Nothing to disclose
Geojeong Seo: Nothing to disclose

### RPS 410-10

**The role of computer-aided analysis of lumbar spine parameters on kinetic MRI using a pattern-recognition software: inter-reader and performance evaluation in consensus**

*F. Bruno*, C. Fagotti, L. Pertici, S. Mancini, G. Caldarelli, C. Santobuono, F. Arrigoni, C. Masciocchi, A. Splendiani; L'Aquila/IT
*(federico.bruno.1988@gmail.com)*

**Purpose:** Our study aims to assess the value of a computer-aided detection tool (CAD) as a second reader in combination with experienced neuroradiologists and residents in training for the analysis of lumbar spine parameters on kinetic MRI images.
**Methods or Backround:** Lumbar spine MRI images in supine and upright position of 51 patients (27 females, mean age 66.2 years, range 34-89) with low back pain were analysed manually by and experienced neuroradiologists and a resident, and automatically using a dedicated AI-based software. Metrics of anatomical and biomechanical modifications between recumbent and weight-bearing positions, such as vertebral wedging, listhesis index, intervertebral translation and angle, spine canal section, thickness and curvature were calculated on the images, either manually by the human readers and automatically by the CAD. Results were compared with an independent reference standard. Inter-observer agreement was calculated by kappa statitistics.
**Results or Findings:** Inter-observer agreement was good (k= 0.79, 95% CI = 0.75-0.84). Extended inter-observer agreement was higher in the evaluation of Listhesis index (k= 0.84-0.86, P <0.05). Agreement between neuroradiologist and residents was improved by CAD (k = 0.63-0.65 and k = 0.70-0.74 before and after CAD consensus, respectively P < 0.05). CAD improved experienced readers' sensitivities in some parameters, without significant improvement of their overall performances.
**Conclusion:** Particularly inexperienced readers benefit from consensus with CAD data, greatly improving the analysis of biomechanical modifications between recumbent and weight-bearing positions.
**Limitations:** No gold standard examination.
**Ethics committee approval:** Local IRB
**Funding for this study:** No funding was received for this study.
**Author Disclosures:**
Giulia Caldarelli: Nothing to disclose
Alessandra Splendiani: Nothing to disclose
Chiara Santobuono: Nothing to disclose
Francesco Arrigoni: Nothing to disclose
Carlo Masciocchi: Nothing to disclose
Sara Mancini: Nothing to disclose
Leonardo Pertici: Nothing to disclose
Cristina Fagotti: Nothing to disclose
Federico Bruno: Nothing to disclose

### RPS 410-11

**Automated analysis of trapeziometacarpal joint kinematics with 4D-CT**

*B. Keelson*, K. Van Royen, A. Gutiérrez Ruiz, T. Scheerlinck, T. Jager, G. Van Gompel, E. Cattrysse, J. Vandemeulebroucke, N. Buls; Jette/BE

**Purpose:** To establish a fully automated image processing protocol for investigating in-vivo kinematics of the TMC joint using 4D-CT.
**Methods or Backround:** 4D-CT images of fifteen healthy volunteers (7 females, 8 males) were obtained during cyclic opposition reposition motion of the thumb. Tube rotation time was 0.28 s, tube voltage 80 kVp, tube current 50 mA, 12 cm collimation, CTDIvol was 13 mGy, DLP of 156 mGycm and scan duration of 6s. Segmentation of the trapezium (Tz), first metacarpal (MC1) and second metacarpal (MC2) were obtained using a multi-atlas segmentation framework. Anatomical landmarks automatically propagated onto each of the bones were used in the creation of bone embedded reference frames. A dynamic sequential rigid registration resulted in transformations from which cardan angles were computed as kinematic parameters. Proximity maps were also estimated as a surrogate for joint contact area.
**Results or Findings:** The predominant rotation of MC1 relative to Tz was around the flexion/extension axis 41.45°[36.63-46.26]. MC1 relative to MC2 showed predominant motion about the internal/external rotation axis of MC2 50.22° [45.11 – 55.33]. The average rotation between Tz and MC2 was below 5° across all axes. A gradual decrease in proximity areas was observed from reposition towards opposition, an indication that joint is more stable at beginning of reposition.
**Conclusion:** Our automated workflow successfully allowed the computation of TMC kinematics with significant time gain by minimising laborious user-interactions. In term, our proposed workflow and results obtained have the potential to be used as a tool for improving knowledge on TMC joint kinematics.
**Limitations:** The impact of potential errors in segmentation and/or registration was not evaluated.
**Ethics committee approval:** This study was supported by our institutional ethical review board.
**Funding for this study:** This study was funded by an interdisciplinary research grant.
**Author Disclosures:**
Kjell Van Royen: Nothing to disclose
Gert Van Gompel: Nothing to disclose
Tjeerd Jager: Nothing to disclose
Adrián Gutiérrez Ruiz: Nothing to disclose
Erik Cattrysse: Nothing to disclose
Nico Buls: Nothing to disclose
Thierry Scheerlinck: Nothing to disclose
Benyameen Keelson: Nothing to disclose
Jef Vandemeulebroucke: Nothing to disclose

### RPS 410-12

**Incremental diagnostic value of dual energy CT-based colored collagen maps for the assessment of the cruciate ligaments in patients with acute trauma**

*C. Booz*, L. D. Grünewald, V. Koch, M. Dimitrova, A. Gökduman, T. J. Vogl, S. Martin, I. Yel; Frankfurt/DE

**Purpose:** To evaluate the diagnostic accuracy and confidence of third-generation dual-source DECT color-coded collagen reconstructions based on material decomposition for the assessment of the cruciate ligaments compared to standard grayscale image reconstruction.
**Methods or Backround:** Patients were eligible for study inclusion in this retrospective study if they underwent third-generation dual-source DECT followed by either 3-T MRI or arthroscopy of the knee joint within 14 days between January 2016 and September 2022. Five radiologists blinded to clinical and MRI information independently evaluated conventional grayscale DECT for the presence of injury to the cruciate ligaments; after 4 weeks, readers re-evaluated the examinations using grayscale images and color-coded collagen reconstructions. A reference standard for MRI was provided by a consensus reading of two experienced readers, blinded to clinical data and DECT images or arthroscopy. Sensitivity and specificity were the primary metrics of diagnostic performance.
**Results or Findings:** A total of 124 patients (mean age, 42 years ± 16 years; 50 male) were enrolled in this study. Application of color-coded collagen reconstructions significantly increased overall sensitivity (91% vs 65%), specificity (79% vs 66%), PPV (58% vs 39%), NPV (96% vs 84%) and accuracy (82% vs 67%) for the detection of injury to the anterior cruciate ligament (all parameters, p<.001). For injury to the posterior cruciate ligament, incremental diagnostic accuracy was observed for complete tears (p<.001). Color-coded collagen maps achieved superior diagnostic confidence, image quality and noise scores compared to grayscale CT (all parameters, p<.001).
**Conclusion:** Color-coded collagen reconstructions derived from DECT yield substantially higher diagnostic accuracy and confidence for the assessment of the cruciate ligaments compared to standard grayscale CT in patients with acute trauma.
**Limitations:** Single-centre retrospective study design.-
**Ethics committee approval:** The IRB approved this study.
**Funding for this study:** No funding was received for this study.
**Author Disclosures:**
Simon Martin: Speaker: Siemens Healthineers
Christian Booz: Speaker: Siemens Healthineers
Ibrahim Yel: Speaker: Siemens Healthineers
Mirela Dimitrova: Nothing to disclose
Vitali Koch: Nothing to disclose
Thomas J. Vogl: Nothing to disclose
Aynur Gökduman: Nothing to disclose
Leon David Grünewald: Nothing to disclose

### RPS 410-13

**Radiofrequency Echographic Multi Spectrometry (REMS): a safe non-ionizing technique for the assessment of bone status during pregnancy**

V. A. Degennaro[1], M. L. Brandi[2], *G. Cagninelli*[1], S. Casciaro[3], F. Conversano[3], E. Di Pasquo[1], S. Gonnelli[4], F. A. Lombardi[3], T. Ghi[1]; [1]Parma/IT, [2]Florence/IT, [3]Lecce/IT, [4]Siena/IT

**Purpose:** The elevated calcium demand of foetuses and neonates across pregnancy and lactation renders women extremely susceptible to bone resorption and subsequent osteoporosis. Currently, no technique exists to assess the maternal bone status without harming the foetus. The present work aims to determine the bone health status in childbearing women versus a control group by means of the non-invasive Radiofrequency Echographic Multi Spectrometry (REMS) technology.
**Methods or Backround:** A cohort of 87 pregnant women at least at 37 weeks of gestation were matched with a reference group of non-pregnant women on the basis of ethnicity (caucasian and non-caucasian), age, body mass index (BMI) and parity. The bone mineral density (BMD) measurement was carried out at the femur in all the enrolled subjects using the REMS technology. A t-test was performed to assess the difference in femoral neck BMD between the two groups and regression analyses were used to investigate the association between the BMD and patients' characteristics.
**Results or Findings:** The mean femoral neck BMD measured in pregnant women was significantly lower than non-pregnant controls (0.771±0.09 g/ cm2 versus 0.834±0.10 g/cm2, p<0.0001). At univariate linear regression, femoral BMD appeared positively associated with BMI (p<0.0001) and negatively with age (p=0.013). Parity did not impact the femoral BMD.
**Conclusion:** A significant decline of bone loss has been quantitatively proved with REMS. Hence, this study demonstrates that REMS represents an appropriate method for BMD assessment which can be safely employed across gestation. Longitudinal follow-up studies are warranted to effectively determine the impact of pregnancy and lactation on maternal bone alterations, with the aim to monitor transient osteoporosis as well as to preserve long-term skeletal health.
**Limitations:** No limitations were identified.
**Ethics committee approval:** No information provided by the submitter.
**Funding for this study:** No funding was received for this study.
**Author Disclosures:**
Francesco Conversano: Nothing to disclose
Valentina Anna Degennaro: Nothing to disclose
Greta Cagninelli: Nothing to disclose
Fiorella Anna Lombardi: Nothing to disclose
Elvira Di Pasquo: Nothing to disclose
Tullio Ghi: Nothing to disclose
Sergio Casciaro: Nothing to disclose
Maria Luisa Brandi: Nothing to disclose
Stefano Gonnelli: Nothing to disclose

**13:00-14:30** **Research Stage 4**

### Research Presentation Session: Radiographers

### RPS 414

### Novelties in computed tomography imaging

**Moderators**
A. D. Annoni; Milan/IT
T. Stankovic; Zagreb/HR

### RPS 414-3

**Dual-energy computed tomography acquisition to ensure lower contrast media volume injection for oncologic imaging follow-up**

M. Gulizia, *Y. Marro*, C. Chevallier, A. Viry, C. Dromain, N. Vietti Violi; Lausanne/CH
*(yannickfcf@hotmail.com)*

**Purpose:** The aim of this study is to define the virtual monochromatic spectral imaging (VMSI) providing the best image quality. The second objective is to compare the image quality and radiation dose of SECT protocol with DECT protocol with reduced iodinated contrast media (ICM) injection.
**Methods or Backround:** We retrospectively included 44 adult patients requiring thoracoabdominopelvic (TAP) in the portal phase for oncologic indication. Two protocols were compared: a conventional single-energy CT (SECT) (protocol A) and a DECT with an added precontrast scan in the upper abdominal region (protocol B). In protocol A, CM dosage corresponded to body

Wednesday weight + 30 in mL and only to body weight for protocol B. VMSI sets from 40 to 80 keV were reconstructed. Quantitative analyses measured enhancement in the liver and portal vein to calculate contrast-to-noise (CNR) and signal-to-noise (SNR) ratios. A subjective analysis was done to assess enhancement, noise, and overall image quality to define the optimal VMSI reconstruction.
**Results or Findings:** CNR were higher in MonoE at 40 keV (6.67 ±1.92) and SNR at 80 keV (12.41 ± 2.33). Both raters assessed significantly higher enhancement at 40 keV (5.0 ± 0.0 vs. 4.83 ± 0.4 ; p > 0.01), noise at 80 keV (4.98 ± 0.15; 4.95 ± 0.21; p > 0.01) and overall image quality at 65 keV (4.59 ± 0.59; 4.55 0.71; p>0.01). CNR were significantly higher in DECT protocol than in SECT protocol (p =0.00).
**Conclusion:** 65 keV provided the optimal image quality between noise and enhancement. The GSI protocol provides just as good an enhancement as the standard one and saves a significant amount of ICM. Image quality is improved by monoenergetic images in DECT with reduced iodinated contrast protocol.
**Limitations:** The limitations of this study were the single-centred focus, the small sample and the standard BMI investigated.
**Ethics committee approval:** This study was approved by an ethics committee (CER-VD 2022-00564).
**Funding for this study:** Not applicable.
**Author Disclosures:**
Yannick Marro: Nothing to disclose
Anaïs Viry: Nothing to disclose
Marianna Gulizia: Nothing to disclose
Naïk Vietti Violi: Nothing to disclose
Clarisse Dromain: Nothing to disclose
Christine Chevallier: Nothing to disclose

### RPS 414-4

**Detection accuracy for COVID-19 in CT imaging**

D. O'Brien, *A. England*, N. Moore, R. Young, M. F. F. McEntee; Cork/IE
*(aengland@ucc.ie)*

**Purpose:** The novel coronavirus has proven challenging to detect radiologically. Studies have reported variations in detection accuracy ranging from 67-97%. Computed tomography (CT) imaging performed by radiographers is the modality of choice to assess the pathological presentations of COVID-19 pneumonitis. This study aimed to investigate any changes in radiographers' detection accuracy for appearances suggestive of coronavirus after exposure to interventional training sets of CT images.
**Methods or Backround:** In total nine radiographers completed a baseline test set, followed by three training sets and then a final test set. Upon completion of each set the radiographers were supplied with immediate feedback provided in the form of a radiology report which allowed participants learn from individual errors. Case sensitivity, specificity, Receiver Operating Characteristic (ROC), true positive values, true negative values, false positive values and false negative values were calculated for each case set. Statistical analysis was calculated based on results from the baseline line test and the final test set; values were then compared to assess any changes.
**Results or Findings:** The results for the final test set demonstrated significant improvement in radiographers performance for case sensitivity (+14.3%) and ROC (+6.1%) compared to the baseline test. The diagnostic accuracy of the radiographers improved in the detection of true positives for COVID-19 (+ 1.2) and true negatives for COVID-19 (+0.2).
**Conclusion:** This study indicated that improvements can be made in radiographers' detection accuracy for COVID-19 after training sets of CT images, as this aids radiographers in learning the radiological appearances of COVID-19 on CT.
**Limitations:** The sample size was limited (n=9 respondents). A wider multinational student of radiographer performance is warranted.
**Ethics committee approval:** University College Cork Social Research Ethics Committee.
**Funding for this study:** No funding was received for this study.
**Author Disclosures:**
Mark F. F. McEntee: Nothing to disclose
Niamh Moore: Nothing to disclose
Rena Young: Nothing to disclose
Daniel O'Brien: Nothing to disclose
Andrew England: Board Member: EFRS

### RPS 414-5

**Estimation of CTDIvol ratio between low and high tube voltage in dual-energy CT**

*K. Hirayama*[1], T. Kobayashi[2], S. Mochinaga[1], K. Nomura[1], K. Fujii[3], Y. Muramatsu[1]; [1]Kashiwa/JP, [2]Minatoku/JP, [3]Nagoya/JP
*(kehiraya@ncc.go.jp)*

**Purpose:** Dual-energy computed tomography (DECT) produces new imaging information such as virtual monochromatic images and basis material decomposition. The dose evaluation in DECT examinations is important and the dose management is performed with the volumed CT dose index (CTDIvol). Total CTDIvol in DECT is displayed on CT console, but the dose ratio between low-kV and high-kV is unknown. The purpose of this study is to estimate the CTDIvol ratio based on the dose measurements with dual-source and rapid kVp switching DECT.
**Methods or Backround:** CTDIvol were measured using a cylindrical ionization chamber (Radcal 10X5-0.6CT) and CTDI body phantom (320mmφ) with three DECT scanners, Aquilion ONE/PRISM edition (Canon Medical Systems, Inc.), Revolution HD (GE Healthcare), and SOMATOM Definition flash (Siemens Healthiness). When the CTDIvol ratio between low-kV and high-kV is a to b (a+b=1), and CTDIvol in low-kV, high-kV, and total CTDIvol in DECT with fixed mAs is CTDIvol^(Low kV), CTDIvol^(High kV), CTDIvol^DE, respectively, we assumed a×CTDIvol^(Low kV)+b×CTDIvol^(High kV)=CTDIvol^DE. The CTDIvol ratio was calculated from this formula and the measured and displayed CTDIvol^(Low kV), CTDIvol^(High kV), and CTDIvol^DE.
**Results or Findings:** The relative errors between measured and displayed CTDIvol were below 13%. The CTDIvol ratio a:b estimated from the displayed CTDIvol for Aquilion ONE/PRISM edition and Revolution HD CT scanners was 0.49:0.51 and 0.67:0.33. The ratio estimated from the measured CTDIvol for Aquilion ONE/PRISM edition and Revolution HD CT scanners was 0.51:0.49 and 0.65:0.35, respectively. This method could not be applied to SOMATOM Definition flash.
**Conclusion:** CTDIvol ratio between low-kV and high-kV estimated from the measured CTDIvol agreed well with that from the displayed CTDIvol. The dose ratio varied among the rapid kVp switching DECT scanners. The dose ratio will be useful for accurate dose verification and simulation in DECT.
**Limitations:** Measurement performed not considering CT-AEC.
**Ethics committee approval:** No information provided by the submitter.
**Funding for this study:** No funding was received for this study.
**Author Disclosures:**
Yoshihisa Muramatsu: Nothing to disclose
Keiichi Nomura: Nothing to disclose
Ken Hirayama: Nothing to disclose
Saeko Mochinaga: Nothing to disclose
Keisuke Fujii: Nothing to disclose
Takayuki Kobayashi: Nothing to disclose

### RPS 414-6

**Low dose CTPA using a low kV technique combined with high IR: a clinical study**

*A. Bellizzi*, P. Bezzina, F. Zarb; Msida/MT

**Purpose:** Optimisation of a computerised tomography pulmonary angiogram (CTPA) scan protocol in terms of radiation dose and image quality. Optimisation was achieved using a low kV technique together with high iterative reconstruction (IR) setting (>50%).
**Methods or Backround:** A phantom study was conducted to identify the optimal kV and IR settings. CTPA examinations were then conducted on 64 patients divided into control and experimental groups. Patients in the control group (n=32) were scanned using the current hospital CTPA protocol (100 kV with 50% IR) while patients in the experimental group (n=32) were scanned using the optimised CTPA protocol (80 kV with 60% IR). The volume computed tomography dose index (CTDIVOL), dose length product (DLP), size specific dose estimate (SSDE) and effective dose (ED) were calculated for both groups as an indication of the delivered radiation dose. Objective image quality was recorded in terms of contrast-to-noise-ratio (CNR) and signal-to-noise-ratio (SNR). Subjective image quality was evaluated by 3 radiologists performing absolute visual grading analysis (VGA). Resultant scores were used to plot Visual Grading Characteristics (VGC) curves with the area under the curve (AUC) indicating image quality.
**Results or Findings:** The application of the optimised CTPA protocol resulted in a significant reduction (p<0.05) in mean CTDIVOL (-49%), DLP (-48%), SSDE (-52%) and ED (-49%). Objective image quality showed significant (p<0.05) improvement in CNR (+32%) and SNR (+13%). VGCAUC values indicated insignificant (p>0.05) variations in image quality between the two protocols.
**Conclusion:** When applying a low kV technique (80kV) combined with high IR (60% IR), a significant dose reduction was achieved while maintaining diagnostic image quality.
**Limitations:** Differences in patient thickness was a variable, addressed by matching mean chest diameters for both control and experimental groups.
**Ethics committee approval:** Approval from the University of Malta Research and Ethics Committee (UREC).
**Funding for this study:** No funding was received for this work.
**Author Disclosures:**
Francis Zarb: Nothing to disclose
Andrea Bellizzi: Nothing to disclose
Paul Bezzina: Nothing to disclose

### RPS 414-7

**Extremely low-dose high-pitch helical CT with tin spectral filtration for planning of functional cardiac CT**

*M. W. Kusk*[1], O. Gerke[2], S. Hess[1], S. J. Foley[3]; [1]Esbjerg/DK, [2]Odense C/DK, [3]Dublin/IE
*(martin.weber.kusk@rsyd.dk)*

**Purpose:** Determining cardiac CT scan range on scan projection radiographs (SPR) can lead to overscanning and unnecessary radiation. Using Tin(Sn) beam filtration combined with high pitch enables doses below the minimum achievable using conventional techniques. We examine the potential for scan range and total dose reduction using this scan mode for planning functional cardiac CT contrast scans.
**Methods or Backround:** 40 patients undergoing CT measurement of left ventricular ejection fraction (LVEF) were examined on a 3rd generation dual source CT (DSCT). Using established standard criteria for cardiac CT (carina to cardiac apex), an ultra-low dose scan was planned on SPR's and performed with tube voltage 100(Sn) kVp, reference mAs of 10, pitch 3.2 and automatic exposure correction (AEC). This scan was used to locate the left ventricle and to plan the contrast scan to only include anatomy of interest. An ECG-gated helical CT of the entire cardiac cycle was then performed. Scan range, CTDI & DLP were recorded. Potential dose saving was based on extrapolation of contrast scan range to initial planned range.
**Results or Findings:** Mean CTDI and DLP for the plan scan was 0.32 mGy and 1.26 mGycm respectively, corresponding to an effective excess dose of 0.02 mSv. The mean scan length was 10.3 cm, compared to 14.7 cm when planning on SPR's. Mean calculated dose saving was 0.47 mSv. Relevant anatomy was included in all scans.
**Conclusion:** High-pitch tin-filtered planning scans is a feasible way to implement scan range reduction, with dose reduction far outweighing the excess dose imparted.
**Limitations:** The number of patients was low.
**Ethics committee approval:** "Ultra-lowdose CT for measurement of left ventricular ejection fraction (LVEF)" (Project ID: S-20210094) Approved by The Regional Committees on Health Research Ethics for Southern Denmark .
**Funding for this study:** Funded by the Esbjerg Fund, Karola Jørgensen Fund and the Danish Radiographer Association (Radiograf Rådet).
**Author Disclosures:**
Martin Weber Kusk: Nothing to disclose
Søren Hess: Nothing to disclose
Oke Gerke: Nothing to disclose
Shane J Foley: Nothing to disclose

### RPS 414-8

**The effect of contact radiation shielding on breast dose during CT abdomen-pelvis: a phantom study**

*M. Long*, A. England, N. Moore, R. Young, M. F. F. McEntee; Cork/IE

**Purpose:** In recent years, several professional bodies have reviewed the use of lead contact shielding outside the field of the radiation beam in computed tomography (CT), advocating that this practice be discontinued. This study investigates whether contact shielding reduces breast radiation dose during CT abdomen-pelvis examinations using automatic tube current modulation (ATCM) to protect one of the four most radiosensitive organs during one of the most performed CT examinations.
**Methods or Backround:** A radiation dosimeter was used to measure skin surface breast dose for a single CT abdomen-pelvis scan protocol of a Kyoto anthropomorphic torso phantom. ATCM was used for all acquisitions. Dose measurements were taken with and without contact shielding in place across the anterior and lateral aspects of the breasts and also with and without organ dose modulation (ODM) to quantify achievable dose reductions.
**Results or Findings:** The addition of lead contact shielding or ODM did not result in statistically significant reductions in breast surface dose. Comparing with and without shielding, the mean breast surface dose was reduced by 0.01µSv without ODM (1.92µSv to 1.91µSv, p=0.49) and increased by 0.03µSv with ODM (1.53µSv to 1.56µSv, p=0.44). Comparing with and without ODM, the mean breast surface dose was reduced by 0.35µSv with shielding (1.91µSv to 1.56µSv, p=0.24) and by 0.39µSv without shielding (1.92µSv to 1.53µSv, p=0.17).
**Conclusion:** The addition of lead contact shielding does not provide significant breast surface radiation dose reduction during CT abdomen-pelvis using ATCM.
**Limitations:** This study used a single adult anthropomorphic phantom and further studies which include a range of patient sizes and CT machines is warranted.
**Ethics committee approval:** No information provided by the submitter.
**Funding for this study:** No funding was received for this study.
**Author Disclosures:**
Mark F. F. McEntee: Nothing to disclose
Niamh Moore: Nothing to disclose
Rena Young: Nothing to disclose
Maria Long: Nothing to disclose
Andrew England: Board Member: EFRS

### RPS 414-9

**Image quality of model-based iterative reconstruction of 150 kVp tin-filtered high-resolution chest CT**

M. W. Kusk, *S. Lysdahlgaard*; Esbjerg/DK
*(Simon.f.l.89@gmail.com)*

**Purpose:** To test the influence of different levels of model-based iterative reconstruction on observed image quality in high-resolution chest CT, acquired at 150 kVp with spectral tin-filtration.
**Methods or Backround:** Informed consent was obtained for the use of 44 inspiratory HRCT scans for processing and analysis. Scans were performed on a 3rd generation Dual Source CT, with 150 kVp tin-filtered X-ray spectrum. All datasets were reconstructed using filtered back-projection (FBP) and 3 different levels of model-based iterative reconstruction (ADMIRE). All other recon parameters were identical. Two radiologists reviewed images blinded, independently, and in random order. Images were scored on a 5-point Likert scale according to 4 pre-defined criteria: (A) visualization of interlobular septae, (B) pleural and subpleural regions, (C) centrilobular region, and (D) bronchi and vessels. Results were analysed with visual grading characteristics (VGC), using a VGC analyser. The 3 ADMIRE levels were compared to FBP, with area under the curve (AUC) and 95% confidence intervals. Interreader reproducibility was assessed with weighted kappa statistics.
**Results or Findings:** For ADMIRE level 1, AUC did not differ significantly from 0.5 for all criteria. For ADMIRE levels 3 and 5, AUC were 0.64 and 0.91 for criterion A, 0.68 and 0.93 for criterion B, 0.65 and 0.86 for criterion C, and 0.62 and 0.83 for criterion D. For all criteria, 95% confidence intervals did not overlap, indicating the statistically significant difference. Weighted kappa was between 0.2 and 0.3 for all criteria.
**Conclusion:** ADMIRE level 5 provided superior visualization of structures, compared to FBP and other ADMIRE levels.
**Limitations:** We found a relatively low agreement and not all available ADMIRE levels were tested.
**Ethics committee approval:** No information provided by the submitter.
**Funding for this study:** No funding was received for this study.
**Author Disclosures:**
Martin Weber Kusk: Nothing to disclose
Simon Lysdahlgaard: Nothing to disclose

### RPS 414-10

**Teleradiography in stroke management**

*E. Kjelle*, A. M. Myklebust; Drammen/NO
*(elin.kjelle@usn.no)*

**Purpose:** To assess radiographers, paramedics, junior doctors and managers' experience of working with a novel teleradiography stroke treatment service in rural Norway.
**Methods or Backround:** Acute ischemic stroke requires early diagnostic computed tomography (CT) to rule out bleeding and immediate thrombolysis treatment to prevent major brain damage. In rural areas, travel time to the hospital is often a barrier for early imaging and thrombolysis treatment. In this study, ten semi-structured individual interviews were conducted. The interview guide covered the themes: experience of working with the service, training, quality, management, and challenges. Thematic content analysis was used to analyze the transcripts of the interviews.
**Results or Findings:** Ten interviews, including radiographers, junior doctors, paramedics, and managers, were conducted. The analysis found that the telemedicine stroke service was considered to be of good quality and high value for the local community. Regular training sessions were essential for the radiographers and junior doctors to guide and help the paramedics to position the patient for the CT, assess the patient clinically, and for thrombolysis to be administered correctly. Communication across professions and the lack of radiologists on call were the biggest challenges. Active management with follow-up of cases and organizing training sessions was considered essential for keeping the service going after an initial pilot period.
**Conclusion:** A teleradiography service for stroke evaluation and treatment in rural areas seem to be received well by managers and healthcare personnel. In task shifting from radiographer and junior doctor to paramedic, regular training sessions are essential. Training sessions should focus on both specific tasks and communication. A radiologist should be available for image reading.
**Limitations:** This study describes an ongoing project in one rural area of Norway. Thus, the transferability to other contexts might be limited.
**Ethics committee approval:** Norwegian center for research data

**Funding for this study:** Not applicable.
**Author Disclosures:**
Elin Kjelle: Nothing to disclose
Aud Mette Myklebust: Nothing to disclose

### RPS 414-11

**Feasibility study of quantitative analysis of thigh muscle mass-based on different CT threshold segments**

*F. Zhou*; Beijing/CN
*(zfy13063561620@163.com)*

**Purpose:** To explore the optimal threshold for quantifying thigh intermuscular fat by CT using MR Dixon images as the reference standard.
**Methods or Backround:** In total, 45 individuals were randomly selected from a cohort of patients with rheumatic immunity. Muscle images of the level of the disappearance of ischial tuberosity at the thigh root were acquired from axial MR Vibe-Dixon images and axial CT images. The thigh muscle compositions of all patients were measured using 2 commonly used image analysis programs: ImageJ and OsiriX. All measurements were repeated twice using each software program, at least 5 days apart. The assessor was blinded to all earlier measurements.
**Results or Findings:** In this study, it was found that the correlation between the muscle signal intensity obtained based on MR image threshold segmentation and the muscle density value obtained based on CT under different threshold segments was moderately negative in all threshold segments in 45 subjects. (-60~150HU, -50 ~150HU, -40 ~150HU, -30 ~150HU, -20 ~150HU, -10 ~150HU, 0~100HU, 0~150HU, r= -0.513 ~ -0.554, P < 0.001). Among them, -20~150HU has the best correlation with MR (r= -554, p＜0.001). The analysis of different genders showed that the best correlation with MR was found in males at 0-150HU (r= -0.610, p＜0.05), while the best correlation was found in females at -40~150HU (r= -0.583, p＜0.05). There was no significant correlation between each threshold segment and MR in patients younger than 40 years old. The best correlation coefficient was found between -20~150HU in ≥40 years old (r= -0.597, p＜0.05).
**Conclusion:** This study verified that -20 to 150HU was more reliable as the optimal threshold for muscle tissue and adipose tissue segmentation.
**Limitations:** There were fewer men and older adults in this study. Sail musculoskeletal imaging.
**Ethics committee approval:** No information provided by the submitter.
**Funding for this study:** Not applicable.
**Author Disclosures:**
Fengyun Zhou: Nothing to disclose

**15:00-16:00** **Research Stage 1**

### Research Presentation Session: Chest

### RPS 504

### The next frontier: radiomics and machine learning in thoracic imaging

**Moderator**
F. Gleeson; Oxford/UK
**Author Disclosures:**
Fergus Gleeson: Author: Publications on the development, testing and validation of various AI algorithms; Board Member: RAIQC Ltd; Founder: RAIQC Ltd; Grant Recepient: AI grants - from Innovate UK and CRUK; Investigator: CI for an Innovate UK grant; Research Grant/Support: AI grants - from Innovate UK and CRUK; Share Holder: Optellum Ltd and RAIAC Ltd

### RPS 504-2

**Evaluation of radiologists' performance with and without AI for the detection of thoracic abnormalities on chest x-ray**

S. Bennani[1], N-E. Regnard[2], L. Lassalle[2], T. Nguyen[1], C. Malandrin[3], H. Koulakian[1], P. Khafagy[4], *G. Chassagnon*[1], M-P. Revel[1]; [1]Paris/FR, [2]Lieusaint/FR, [3]Longjumeau/FR, [4]Fribourg/CH

**Purpose:** The study aimed to compare the performance of artificial intelligence (AI)-assisted radiologists to unassisted radiologists in detecting thoracic abnormalities on chest radiographs (CXR)
**Methods or Backround:** Thoracic images of patients who underwent CXR and thoracic CT scans within 72 hours at Cochin University Hospital were collected over a 10-year period. A senior thoracic radiologist annotated the CXR images for 5 main abnormalities (pneumothorax, pleural effusion, consolidation, mediastinum-hilar mass, and nodule) based on the corresponding CT scan results. Twelve readers (4 thoracic radiologists, 4 general radiologists and 4 radiology residents) read half of the dataset without AI and half with AI (ChestView, Gleamer).
**Results or Findings:** The study included 500 examinations, 240 with detectable abnormalities on CXR and 260 without. The increase in sensitivity for detecting visible pneumothorax was 6.8% (p=0.02) for chest radiologists, 36.5% (p<0.001) for general radiologists, and 32.5% (p<0.001) for radiology residents. For visible pleural effusions, the gain in sensitivity was 9.4% (p<0.001), 13.4% (p<0.001), and 7% (p<0.001) for thoracic radiologists, general radiologists, and radiology residents, respectively. The change in sensitivity for visible mediastinohilar masses was -0.6% (p=0.3), 5% (p=0.002), and 11.6% (p<0.001) for chest radiologists, general radiologists, and radiology residents, respectively. For consolidations, the sensitivity gain was 10.5% (p<0.001) for chest radiologists, 12.1% (p<0.001) for general radiologists, and 21.4% (p<0.001) for radiology residents. For pulmonary nodules, sensitivity increased by 5% (p<0.001), 15.8% (p<0.001) and 13.4% (p<0.001) for chest radiologists, general radiologists and radiology residents. The specificity for all readers did not decrease when assisted by AI.
**Conclusion:** AI can significantly increase the detection of chest abnormalities without decreasing the specificity.
**Limitations:** This study was retrospective with artificial prevalence of pathologies.
**Ethics committee approval:** No information was provided by the submitter.
**Funding for this study:** The study was funded by Gleamer.
**Author Disclosures:**
Guillaume Chassagnon: Nothing to disclose
Hasmik Koulakian: Nothing to disclose
Philippe Khafagy: Nothing to disclose
Nor-Eddine Regnard: Founder: Gleamer
Louis Lassalle: Consultant: Gleamer
Toan Nguyen: Consultant: Gleamer
Souhail Bennani: Consultant: Gleamer
Cecile Malandrin: Nothing to disclose
Marie-Pierre Revel: Nothing to disclose

### RPS 504-3

**Will I change nodule management recommendations if I change my CAD system? - Volumetry of artificial pulmonary nodules by different CAD systems**

*A. A. Peters*[1], A. Christe[1], O. v. Stackelberg[2], M. Pohl[2], H-U. Kauczor[2], C. P. Heussel[2], M. O. Wielpütz[2], L. Ebner[1]; [1]Bern/CH, [2]Heidelberg/DE
*(alan.peters@insel.ch)*

**Purpose:** This study aims to evaluate and compare the measurement accuracy of two different CAD systems regarding artificial pulmonary nodules and assess the clinical impact of volumetric inaccuracies in a phantom study.
**Methods or Backround:** In this phantom study, 59 different phantom arrangements with 326 artificial nodules (178 solid, 148 ground-glass) were scanned at 80 kV, 100 kV and 120 kV. Four different nodule diameters were used: 5 mm, 8 mm, 10 mm and 12 mm. Scans were analysed by a DL-based CAD and a standard CAD system. Relative volumetric errors (RVE) of each system vs ground truth and the relative volume difference (RVD) DL-based vs standard CAD were calculated. The Bland-Altman method was used to define the limits of agreement (LOA). The hypothetical impact on LungRADS classification was assessed for both systems.
**Results or Findings:** There was no difference between the three voltage groups regarding nodule volumetry. Regarding the solid nodules, the RVE of the 5 mm, 8 mm, 10 mm and 12 mm size group for the DL CAD/standard CAD were 12.2/2.8%, 1.3/-2.8%, -3.6/1.5% and -12.2/-0.3%, respectively. The corresponding values for the GGN were 25.6%/81.0%, 9.0%/28.0%, 7.6/20.6% and 6.8/21.2%. The mean RVD for solid nodules/GGN was 1.3/-15.2%. Regarding the LungRADS classification, 88.5% and 79.8% of all solid nodules were correctly assigned by the DL CAD and the standard CAD, respectively. 14.9% of the nodules were assigned differently between the systems.
**Conclusion:** Patient management may be affected by the volumetric inaccuracy of the CAD systems and demand supervision and/or manual correction by a radiologist.
**Limitations:** The protocol is not state-of-the-art and the phantom setting not realistic.
**Ethics committee approval:** No information provided by the submitter.
**Funding for this study:** Project conducted during ESOR Research Fellowship.
**Author Disclosures:**
Mark O. Wielpütz: Nothing to disclose
Claus Peter Heussel: Nothing to disclose
Alan Arthur Peters: Nothing to disclose
Hans-Ulrich Kauczor: Nothing to disclose
Oyunbileg von Stackelberg: Nothing to disclose
Moritz Pohl: Nothing to disclose
Lukas Ebner: Nothing to disclose
Andreas Christe: Nothing to disclose

### RPS 504-4

**Computed tomography embolus texture analysis as a prognostic marker of acute pulmonary embolism**

*H-J. Meyer*[1], J. Leonhardi[1], N. Bailis[1], T. Denecke[1], A. Surov[2]; [1]Leipzig/DE, [2]Magdeburg/DE
*(hans-jonas.meyer@medizin.uni-leipzig.de)*

**Purpose:** Texture analysis is a quantitative imaging analysis that provides novel biomarkers beyond conventional image reading. Our aim was to use texture analysis of pulmonary emboli derived from thoracic computed tomography for the prediction of mortality and prognosis of acute pulmonary embolism (PE).
**Methods or Backround:** Overall, 216 patients (116 female, 53.7%) were included in the analysis. Texture analysis was calculated on axial slices of the contrast-enhanced pulmonary angiography of the proximal embolus. Clinical scores, serological parameters, need for intubation, intensive care unit (ICU) admission, and mortality was assessed and correlated with the texture features.
**Results or Findings:** In the correlation analysis, there were several associations with mortality in days, the highest for the parameter S(0,5) SumVarnc ($r = -0.43$, $P < 0.001$). Another parameter, S(3,-3) AngScMom, correlated with sepsis-related organ failure assessment score (SOFA)-score ($r = 0.31$, $P < 0.001$). Several texture features correlated with venous lactate and glucose levels. In the discrimination analysis, there were significant differences regarding texture features between survivors and non-survivors and between patients with and without the need for ICU admission ($P = 0.02$, respectively).
**Conclusion:** These results highlight the potential clinical benefit of texture features in patients with acute PE as novel imaging biomarkers. Further studies are needed to validate these results.
**Limitations:** Retrospective single-centre study. Second, the patient sample is relatively small. A certain selection bias might have influenced the present results.
**Ethics committee approval:** No information was provided by the submitter.
**Funding for this study:** No funding was received for this study.
**Author Disclosures:**
Alexey Surov: Nothing to disclose
Timm Denecke: Nothing to disclose
Hans-Jonas Meyer: Nothing to disclose
Jakob Leonhardi: Nothing to disclose
Nikolaos Bailis: Nothing to disclose

### RPS 504-5

**Retrospective validation of nodule management based on deep learning-based malignancy thresholds in lung cancer screening**

*N. Antonissen*[1], K. V. Venkadesh[1], H. A. Piggelen-Gietema[2], R. Vliegenthart[3], Z. Saghir[4], E. T. T. Scholten[1], M. Prokop[1], C. M. Schaefer-Prokop[5], C. Jacobs[1]; [1]Nijmegen/NL, [2]Maastricht/NL, [3]Groningen/NL, [4]Copenhagen/DK, [5]Amersfoort/NL
*(noa.antonissen@radboudumc.nl)*

**Purpose:** We previously developed and validated a deep learning (DL) algorithm for malignancy risk estimation of screen-detected nodules. The nodule risk cut-off for a positive screen, triggering more intensive follow-up (either short-term follow-up, PET-CT or biopsy), varies in existing nodule management protocols; 1-2% for Lung-RADS (cat 3), 6% for PanCan2b (CAT3). In this study, we investigated two DL-based malignancy thresholds to define a positive screen, compared to existing nodule management protocols.
**Methods or Backround:** All baseline CT scans from the Danish Lung Cancer Screening Trial were linked to lung cancer diagnosis within 2 years, resulting in 2,019 non-cancer and 18 cancer cases. The DL-based malignancy risk was computed for all screen-detected nodules using two malignancy risk cut-off points (6% and 10%) as a threshold for a positive screen. For both Lung-RADS and PanCan2b, we used the published nodule-risk cut-offs for a positive screen. Sensitivity and false positive rate (FPR) were calculated for all baseline scans (n=2,037) using the risk dominant nodule per scan.
**Results or Findings:** At a threshold of 6%, DL achieved the highest sensitivity with 88.9% compared to 83.3% for Lung-RADS and 77.8% with PanCan2b. DL and PanCan2b yielded comparable FPRs of 3.6% and 4.1%, respectively, while Lung-RADS had a higher FPR of 8.7%. Increasing the DL threshold to ≥10% resulted in a sensitivity of 88.9% and an FPR of 2.5%.
**Conclusion:** DL-based nodule risk cut-offs achieved the highest sensitivity and lowest FPR for defining a positive screen, triggering a more intense diagnostic workup. Increasing the risk cut-off from 6% to 10% further decreased the FPR without alteration of the sensitivity.
**Limitations:** This study is a retrospective data analysis from one screening trial and one screening round. More external validation is needed, including validation for incidence screenings.
**Ethics committee approval:** No information was provided by the submitter.
**Funding for this study:** The study is funded by the Dutch Cancer Foundation.
**Author Disclosures:**
Cornelia M. Schaefer-Prokop: Nothing to disclose
Ernst Th. T Scholten: Nothing to disclose
Mathias Prokop: Research/Grant Support: Research grant and royalties to institution from MeVis Medical Solutions, Bremen, Germany
Kiran Vaidhya Venkadesh: Nothing to disclose
Zaigham Saghir: Nothing to disclose
Rozemarijn Vliegenthart: Nothing to disclose
Noa Antonissen: Nothing to disclose
Hester Alexandra Piggelen-Gietema: Nothing to disclose
Colin Jacobs: Research/Grant Support: Research grant and royalties to institution from MeVis Medical Solutions, Bremen, Germany

### RPS 504-6

**Correlation between CT radiomic features of lung parenchyma and baseline respiratory function in ES-NSCLC patients treated with SBRT: an exploratory analysis**

*F. Mastroleo*, S. Volpe, L. Berton, A. Gaeta, F. Bellerba, M. Zaffaroni, M. G. Vincini, S. Gandini, B. A. Jereckzek-Fossa; Milan/IT
*(fed.mastroleo@gmail.com)*

**Purpose:** The purpose of the study is to investigate the association between radiomic features of lung parenchyma and the diffusing capacity of carbon monoxide (DLCO) in early-stage non-small-cell lung cancer (ES-NSCLC) patient candidates to stereotactic body radiotherapy (SBRT). As respiratory comorbidities are common in this setting, the availability of non-invasive markers of lung functionality may assist the patients' follow-up and implement spirometry-derived information.
**Methods or Backround:** Patients treated at a single Italian cancer centre from 2014 to 2022 with SBRT for ES-NSCLC were considered; tumour recurrences were excluded. The gross tumour volume (GTV) and the entire lungs were delineated on the 4DCT, removing GTV from the ipsilateral lung. The feature extraction was performed by pyradiomics. The DLCO was used for lung functionality. Multivariable mixed-effects models for repeated measures analysis were adopted to analyse the association between radiomic features and DLCO abnormality, adjusting models for age at diagnosis and COPD status.
**Results or Findings:** The selected population included 23 patients. In the univariate logistic model, age was the only clinical variable with a significant association with abnormal DLCO (OR 1.33). At the multivariable mixed-effects model, 74 and 108 radiomics features were associated with DLCO ($p < 0.10$) in both lungs and ipsilateral lung segmentations: 12 and 17 first order, 22 and 23 GLDM, 24 and 29 GLRLM, 12 and 26 GLSZM, 4 and 3 NGTDM. Regarding the subdivision of features based on the wavelet, the log-sigma and square filter features had the highest association with DLCO.
**Conclusion:** The identification of stable features and clinical variables in ES-NSCLC patients with a strong association with DLCO are fundamental steps for the development of a radiomic signature able to describe the baseline lung functionality in clinical activity.
**Limitations:** It is a retrospective study with a small dataset.
**Ethics committee approval:** No information provided by the submitter.
**Funding for this study:** No funding was received for this study.
**Author Disclosures:**
Sara Gandini: Nothing to disclose
Federico Mastroleo: Nothing to disclose
Aurora Gaeta: Nothing to disclose
Maria Giulia Vincini: Nothing to disclose
Barba Alicja Jereckzek-Fossa: Nothing to disclose
Lucrezia Berton: Nothing to disclose
Federica Bellerba: Nothing to disclose
Stefania Volpe: Nothing to disclose
Mattia Zaffaroni: Nothing to disclose

15:00-16:00 Research Stage 2

### Research Presentation Session: Hybrid, Molecular and Translational Imaging

### RPS 506

### Hybrid imaging techniques at the brink of clinical implementation

**Moderator**
T. H. Helbich; Vienna/AT
**Author Disclosures:**
Thomas H. Helbich: Grant Recepient: siemens, bracco, guerbet, hologic, bard, novomed

### RPS 506-2

**Ultra-high resolution propagation-based lung CT imaging at clinically relevant x-ray dose levels**
*W. Wagner*[1], J. Albers[2], M. Fiedler[1], E. Baratella[3], J. Biederer[1], M. O. Wielpütz[1], H-U. Kauczor[1], G. Tromba[3], C. Dullin[1]; [1]Heidelberg/DE, [2]Hamburg/DE, [3]Trieste/IT

**Purpose:** Compared to conventional absorption-based clinical CT imaging, novel phase-sensitive x-ray imaging techniques harbour the potential of improved soft-tissue contrast and higher spatial resolutions at a relatively low radiation burden. Here, we present results from an improved propagation-based phase contrast imaging (BPI) setup for ultra-high-resolution local CT of fresh porcine lungs in a human-sized chest phantom at radiation dose levels below clinical chest CT.
**Methods or Backround:** We used the ARTIChest phantom (PRO Design, Germany) equipped with fresh porcine lungs. PBI was performed at the Italian synchrotron using the XCounter (Direct Conversion, Sweden) detector with a pixel size of 100µm, a sample-to-detector distance of 10.7m and a monochromatic x-ray beam at 40keV, resulting in an effective pixel size of 67µm. A comparative clinical CT was performed at the Cattinara Hospital Trieste using an iCT 256 (Philips, Germany) with a resolution of 0.45x0.45x0.9mm. PBI and iCT images were acquired with 10 and 13mGy respectively, measured with thermoluminescence dosimeters embedded in the phantom.
**Results or Findings:** The superiority of PBI local tomography was shown in the healthy lung, in artificially induced solid and subsolid pulmonary nodules, in ground glass depiction and in micro-tree-in-bud pattern depiction. Additionally, a porcine ARDS model was compared in both modalities. Objective image quality parameters such as pixel size, contrast-to-noise-ratio, edge-enhancement-to-noise-ratio, deviation-from-Heaviside-function-index and radial-average-power-spectra were determined, and subjective image quality evaluation by trained thoracic radiologists showed the advantages of PBI at comparable dose levels.
**Conclusion:** Local PBI CT of the lung is emerging as a promising tool for targeted ultra-high-resolution lung imaging in the sense of a "virtual biopsy" in cases microstructural information is needed to guide pulmonary disease diagnostics.
**Limitations:** Further developments regarding the acquisition protocol are required.
**Ethics committee approval:** No information provided by the submitter.
**Funding for this study:** Funding was received from the German Centre for Lung Research.
**Author Disclosures:**
Mark O. Wielpütz: Nothing to disclose
Mascha Fiedler: Nothing to disclose
Giuliana Tromba: Nothing to disclose
Jürgen Biederer: Nothing to disclose
Christian Dullin: Nothing to disclose
Jonas Albers: Nothing to disclose
Hans-Ulrich Kauczor: Nothing to disclose
Willi Wagner: Nothing to disclose
Elisa Baratella: Nothing to disclose

### RPS 506-3

**Ultrasound-unenhanced CT fusion for the follow-up of ureteral stones**
*E. Bercovich*, A. Zisman, A. Ilivitzki; Haifa/IL
*(eyalberc@gmail.com)*

**Purpose:** Computed tomography (CT) is the gold standard for detecting urinary tract stones. Ultrasound (US) has a lower sensitivity in detecting ureteral stones, particularly where the acoustic window is obscured by bowel gas. The increasing global use of CT for suspected urolithiasis has led to an increase in the population's exposure to ionizing radiation. We propose to reduce the need for serial CT scans to detect distal progression of urinary stones by creating a map of the ureters from the initial CT. Using 3d-coregistration, this CT map is then fused with ultrasound. By comparing US-CT fusion to ureteroscopy, we can determine if patients with ureteral stones can be followed-up with US-CT fusion.
**Methods or Backround:** Patients with a single ureteral stone detected by CT were referred for ureteroscopy. Before the procedure, US-CT fusion was conducted. A radiologist who was blind to the results of the ureteroscopy evaluated the fusion images. An abdominal plain film and/or a second NCCT were performed if the stone was not visible at fusion. The US-CT fusion outcomes were compared to the ureteroscopy.
**Results or Findings:** 31 patients were included in the study. The mean time from the NCCT to the US-CT fusion exam was 42.6±39 days. The mean time from the US-CT fusion exam until the ureteroscopy was 1.8±4.5 days. The pooled data had a sensitivity of 72.7% and a specificity of 100% for detecting a stone.
**Conclusion:** A US-CT fusion scan is not inferior to CT scans for the follow-up of ureteral stones found at initial NCCT. US-CT fusion might reduce the need for additional CT scans, but it cannot rule out a stone when one is not detected. We believe this alternative can reduce radiation exposure and can be cost-saving.
**Limitations:** Single-centre study and variability in fusion technique.
**Ethics committee approval:** No information was provided by the submitter.
**Funding for this study:** No funding was received for this study.
**Author Disclosures:**
Eyal Bercovich: Nothing to disclose
Ariel Zisman: Nothing to disclose
Anat Ilivitzki: Nothing to disclose

### RPS 506-4

**F-18-FDG-PET/CT imaging of COVID-19 in a prospective clinical study**
*S. Czibor*[1], L. Száraz[1], J. Simon[1], B. Dombai[1], D. Dey[2], L. Szakács[1], V. Müller[1], T. Györke[1], P. Maurovich-Horvat[1]; [1]Budapest/HU, [2]Los Angeles, CA/US

**Purpose:** The study aimed to evaluate morphological and metabolic findings in COVID-19 with FDG-PET/CT.
**Methods or Backround:** This was a single-centre, prospective clinical trial enrolling consecutive patients who required hospitalisation due to a COVID-19 infection. All patients underwent chest CT on admission and a follow-up FDG-PET/CT scan on the seventh day of hospitalisation. COVID-19-related lung alterations, such as GGO and consolidation were quantified with a semi-automated, deep-learning-enabled software, and metabolic parameters were expressed with PET-based metabolic inflammatory volume (MIV). The primary outcome was defined as the low or high inflammatory state on PET scan, with the median MIV being the cut-off value.
**Results or Findings:** Forty-four patients were enrolled (25 men; median [IQR] age: 52 [49-61] years). The median [IQR] MIV was 209 [73-517] ml. The percentage of GGO and total lung CT severity scores at baseline showed a weak correlation with MIV ($r=0.33-0.39$; $p=0.13-0.34$). At follow-up, we detected a strong correlation between all chest CT abnormalities and MIV ($r=0.77$; $p<0.01$), as well as between CT severity scores and MIV ($r=0.77$; $p<0.01$). Logistic regression analysis adjusted for demographics revealed that the extent of chest CT abnormalities on the follow-up was an independent predictor of a high inflammatory state (OR [by 1%change]=1.11; $p=0.018$). A model encompassing CT abnormalities, interleukin-6 and lactate-dehydrogenase levels at follow-up showed high predictive values for the inflammatory state, with an AUC on ROC analysis of 0.88.
**Conclusion:** The metabolic inflammatory volume and activity of COVID-19 pneumonia showed a good correlation with morphological changes on CT imaging performed 7 days after patient hospitalisation. Combining CT and laboratory data (lactate-dehydrogenase and interleukin-6 levels), the FDG-PET-based lung inflammatory status could be effectively predicted, which may play a role in microbial lung infections beyond COVID-19.
**Limitations:** Spread in the intervals between baseline and follow-up scanning.
**Ethics committee approval:** Hungarian Board of Ethics was the review board that approved the study (ID: 38829/2021). The COMPOSIT study is registered at www.clinicaltrials.gov (ID: NCT05009563).
**Funding for this study:** Semmelweis Scientific and Innovation Fund.
**Author Disclosures:**
Tamás Györke: Nothing to disclose
László Szakács: Nothing to disclose
Damini Dey: Nothing to disclose
Lili Száraz: Nothing to disclose
Pál Maurovich-Horvat: Nothing to disclose
Brigitta Dombai: Nothing to disclose
Sandor Czibor: Nothing to disclose
Veronika Müller: Nothing to disclose
Judit Simon: Nothing to disclose

### RPS 506-5

**Validation of the PSMA-RADS 1.0 system for the structured interpretation of F18-PSMA-PET/CT scans in diagnosing prostate cancer**

*T. Geyer*, J. Rübenthaler, M. P. Fabritius, F. Grawe; Munich/DE

**Purpose:** The standardised reporting and data system for PSMA-targeted PET imaging (PSMA-RADS 1.0) has shown promising results as an effective way to categorise these scans according to the likelihood of the presence of prostate cancer. This study aims to evaluate the inter- and intra-observer agreement in the interpretation of F18-PSMA-PET/CT scans according to the standardised PSMA-RADS 1.0.
**Methods or Backround:** The scans of 103 patients with prostate cancer who underwent F18-PSMA-PET/CT were retrospectively analysed. Two experienced (E1, E2) and two inexperienced (I1, I2) nuclear medicine and radiology physicians independently analysed the scans of these patients at two different time points. The interpretation was based on the PSMA-RADS 1.0 criteria, and a maximum of five lesions per scan were evaluated. Reader agreement was measured using Cohen's weighted kappa and the intraclass correlation coefficient (ICC).
**Results or Findings:** The intra-observer agreement was very high for reader E1 (cappa=0.8261), very high for reader E2 (cappa= 0.9139), substantial for reader I1 (cappa=0.6863), and very high for reader I2 (cappa= 0.9677). The inter-rater reliability was substantial for E1 and E2 at t1 (cappa= 0.6814), moderate for E1 and E2 at t2 (cappa=0.5970), moderate for I1 and I1 at t2 (cappa= 0.5188), and moderate for I1 and I2 at t1 (cappa=0.5101). The agreement between inexperienced and experienced readers was substantial (t1: cappa= 0.7196; t2: cappa=0.7245). The inter-rater agreement of all four readers was moderate (T1: cappa =0.46298; T2: k =0.48219).
**Conclusion:** PSMA-RADS 1.0 is a highly reproducible and accurate system with high intra-rater reliability and high agreement among readers. The system is a promising approach to standardise the diagnosis of prostate cancer in hybrid imaging.
**Limitations:** The retrospective setting must be considered a limitation, and further prospective validation studies are required.
**Ethics committee approval:** No information was provided by the submitter.
**Funding for this study:** No funding was received for this study.
**Author Disclosures:**
Matthias Philipp Fabritius: Nothing to disclose
Freba Grawe: Nothing to disclose
Thomas Geyer: Nothing to disclose
Johannes Rübenthaler: Nothing to disclose

### RPS 506-6

**Total body PET/CT vs conventional PET/CT in lymph node staging of non-small cell lung cancer**

*L. Nardo*, Y. Mohammad, Y. G. Abdelhafez, L. A. Godoy, T. Li, D. T. Cooke, B. A. Spencer, F. Sen, R. Badawi; Sacramento, CA/US
*(lnardo@ucdavis.edu)*

**Purpose:** The aims of this study were (1) to evaluate the accuracy of total-body PET/CT (TBP) and (2) to compare TBP to a conventional PET/CT scan (CP) in the assessment of mediastinal and hilar lymph nodes in the staging of non-small cell lung cancer (NSCLC) using only pathology as ground truth.
**Methods or Backround:** In this prospective study, 27 subjects (69.3±10.0 yrs; 18 female) presenting for initial PET/CT staging of NSCLC were enrolled. All subjects were scanned 120 minutes after injection of 296-444-MBq of 18F-FDG. Of them, 14 subjects were scanned both on a CP scanner (~22 cm axial field of view) and TBP (~194 cm axial field of view) at 60 and 90 minutes in a randomised fashion. A chart review was performed by a researcher, and all lymph nodes with pathologic data were recorded and mapped in all corresponding PET/CT scans. The mapped PET studies were then read and scored by a nuclear medicine-certified radiologist using a score from 1 to 5; being 1 = benign, 2 = likely benign, 3 = possibly malignant, 4 = likely malignant and 5 = malignant.
**Results or Findings:** 93 lymph node groups had a pathologic assessment (11/93 malignant). TBP sensitivity, specificity, negative predictive value and accuracy were 91%, 99%, 99% and 98%, respectively. In the subgroup scanned on both scanners, the corresponding values were, respectively, 100%, 98%, 100% and 98% for TBP, and 75%, 98%, 96% and 94% for CP. There were 2 false negative results on CP, which were correctly identified on TBP.
**Conclusion:** TBP has a high accuracy; the initial data suggests potential upstaging of nodal disease on TBP imaging.
**Limitations:** It was a relatively small cohort, and there were some difficulties in lymph node mapping on PET images using a surgical report.
**Ethics committee approval:** No information was provided by the submitter.
**Funding for this study:** The study was funded by the US National Institutes of Health R01-CA249422; R01-CA026187.
**Author Disclosures:**
Fatma Sen: Nothing to disclose
Luis A. Godoy: Nothing to disclose
Yasamin Mohammad: Nothing to disclose
Tianhong Li: Nothing to disclose
Benjamin A. Spencer: Nothing to disclose
Ramsey Badawi: Research/Grant Support: United IMaging Healthcare
Lorenzo Nardo: Research/Grant Support: Cerianna Research/Grant Support: Lantheus Research/Grant Support: Telix Grant Recipient: United Imaging Healthcare Investigator: Novartis
Yasser Gaber Abdelhafez: Nothing to disclose
Davis T Cooke: Nothing to disclose

### RPS 506-7

**Changes in tumour lesion glycolysis are associated with clinical endpoints in patients with advanced NSCLC receiving immune checkpoint inhibitor therapy: a prospective single-centre study**

*D. Kifjak*, A. Haug, M. E. Mayerhöfer, M. Hochmair, H. Prosch, L. Beer; Vienna/AT
*(daria.kifjak@umassmemorial.org)*

**Purpose:** To assess the association of metabolic tumour parameters using 18F-FDG-PET-CT and clinical endpoints in patients with advanced non-small-cell lung cancer (NSCLC) treated with adjuvant immune checkpoint inhibitor therapy (ICIT).
**Methods or Backround:** This prospective single-centre study included 68 patients who underwent 18F-FDG PET-CT before ICIT initiation and after 2-3 doses of various ICITs (median of 43 days). We semi-automatically extracted the total lesion glycolysis (TLG) at baseline and at the follow-up examinations. Patients were stratified into three groups: 1) metabolic response: reduction of ≥-30%; 2) stable disease: -30% to +20%; 3) progressive disease: >+20%. PD-L1 status and neutrophiles count were retrieved from patients' records. We evaluated the prognostic value of TLG for the overall survival (OS) and progression-free survival (PFS) using the Kaplan-Meier test and Cox regression analysis.
**Results or Findings:** Patients were followed up for a median of 11 months (range 1-63 months). TLG was associated with PFS and OS in the univariable analysis (p<0.001). Even after the correction for known risk factors such as the PD-L1 status and neutrophils count, TLG was associated with PFS (metabolic response vs metabolic progressive disease: 14 months vs 4 months, HR:3.7:95%CI: 1.82-7.53; p<0.001) and OS (metabolic response vs metabolic progressive disease: median not met vs 12 months, HR:5.07:95%CI: 1.93-13.31; p=001) between the first and the third group.
**Conclusion:** Changes in TLG are independently associated with progression-free and overall survival in patients with advanced NSCLC treated with immune-checkpoint inhibitors.
**Limitations:** Small sample size and single-centre study.
**Ethics committee approval:** EC No.:1521/2015
**Funding for this study:** Funding for this study was received by the Österreichische Nationalbank (grant 16886), "Fond für interdisziplinäre Krebsforschung der Stadt Wien".
**Author Disclosures:**
Maximilian Johannes Hochmair: Nothing to disclose
Alexander Haug: Nothing to disclose
Helmut Prosch: Nothing to disclose
Marius E. Mayerhöfer: Nothing to disclose
Lucian Beer: Nothing to disclose
Daria Kifjak: Nothing to disclose

### RPS 506-8

**Pre- to post-treatment evolution of PET/CT parameters in CAR-T cell treatment of lymphoma**

*M. Winkelmann*, K. Rejeski, V. Blumenberg, F. Dekorsy, P. Bartenstein, J. Ricke, M. Bergwelt-Baildon, M. Subklewe, W. Kunz; Munich/DE

**Purpose:** Chimeric antigen receptor T-cell therapy (CART) with patient-derived T cells is approved for relapsed or refractory (r/r) large B-cell lymphoma (LBCL). Anti-tumour activity differs from conventional treatment strategies with so far undefined response patterns. We evaluated changes in morphologic and metabolic parameters from baseline to 3 months follow-up PET/CT and their prognostic value for overall survival (OS).
**Methods or Backround:** Consecutive r/r LBCL patients with 18F-FDG PET/CT imaging at baseline and 3 months after CART were selected. The overall response was determined based on the Lugano criteria with up to 6 target lesions. The sum of the product of the diameters (SPD) was used to represent the tumour burden and its percent reduction over time (depth of response, DoR). Maximum standard uptake value percent reduction between baseline and 3 months (ΔSUV) was applied as a metabolic response parameter.
**Results or Findings:** 22 patients met the inclusion criteria (median age: 67 years, 36% female) with a median baseline SPD of 6,088 mm2 and median SUVmax of 25.8. According to the Lugano criteria, 12 patients (55%) were in complete metabolic remission at 3 months. A colour-coded waterfall plot shows DoR together with the Lugano response category. The median tumour burden and metabolism were reduced in DoR by 81.5% and in ΔSUV by 62.1%. Among the patients, DoR and ΔSUV showed a strong correlation (r=0.71, p<0.001). DoR≥50%, ΔSUV≥40% and complete metabolic response per Lugano showed significant stratification of OS (each with p=0.05).
**Conclusion:** For r/r LBCL patients undergoing CAR T-cell therapy, morphologic and metabolic parameters represent different properties. Both may have prognostic information. Further studies should determine which individual parameter or combination is most suitable for survival prognostication after CAR T-cell therapy.
**Limitations:** Single-centre study with a limited patient population.
**Ethics committee approval:** All medical records and imaging studies were reviewed with the approval of the LMU Munich Institutional Review Board (LMU Ethics Committee, project number 19-817). Informed consent was obtained from all individual participants included in the study.
**Funding for this study:** FöFoLe LMU München (1147).
**Author Disclosures:**
Marion Subklewe: Advisory Board: Amgen, Celgene, Gilead, Janssen, Novartis, Pfizer, and Seattle Genetics Consultant: Amgen, Bristol Myers Squibb, Celgene, Gilead, Pfizer, Novartis, and Roche Research/Grant Support: Amgen, Gilead, Miltenyi Biotec, MorphoSys, Roche, and Seattle Genetics Speaker: Amgen, Celgene, Gilead, Janssen, and Pfizer
Viktoria Blumenberg: Research/Grant Support: Gilead, Novartis, Celgene, and Roche
Michael Bergwelt-Baildon: Research/Grant Support: Novartis, Kite Pharma, Miltenyi Biotec, Mologen, MSD, Astellas, and Roche
Peter Bartenstein: Nothing to disclose
Michael Winkelmann: Nothing to disclose
Wolfgang Kunz: Consultant: Bristol Myers Squibb
Kai Rejeski: Research/Grant Support: Kite/Gilead and Novartis
Franziska Dekorsy: Nothing to disclose
Jens Ricke: Nothing to disclose

### RPS 506-9
**Hybrid photoacoustic-ultrasound breast tomography: first patient results**
*R. F. G. Bulthuis*[1], M. Dantuma[1], F. Lucka[2], A. Javaherian[3], B. De Santi[1], B. Cox[3], M. van der Schaaf[1], J. Veltman[4], S. Manohar[1]; [1]Enschede/NL, [2]Amsterdam/NL, [3]London/UK, [4]Almelo/NL
*(r.f.g.bulthuis@utwente.nl)*

**Purpose:** To present the first breast imaging results from an advanced hemispherical geometry photoacoustic-ultrasound tomography system. From the photoacoustic mode, we show the appearance of blood vessels in the breast and those associated with breast cancer in three cases of suspect breasts. From the ultrasound mode, we present the sound speed distributions in the breast. We discuss the potential and outlook for the hybrid technology from the initial results on 10 patients.
**Methods or Backround:** Photoacoustic (PA) tomography is a contrast agent-free functional imaging technique which has the ability to visualise blood vessels with high resolutions. The method uses pulsed light as incident energy while measuring ultrasound produced by transient thermal expansion. A sophisticated multiwavelength PA system in hemispherical geometry was recently developed to image the breast. The system also includes, for the first time the capability to image the speed of sound (SOS) through the breast. This knowledge is used to improve the accuracy of photoacoustic reconstructions while also providing structural information inside the breast. Ten patients have been included in the technology demonstration study. The results were compared to conventional imaging and histopathology.
**Results or Findings:** Three case reports are highlighted in this presentation. In general, PA can visualize blood vessels in the breast at superior fields-of-view and depths (around 48 mm) compared to state-of-the-art PA imaging systems. From the PA images, tumours can be revealed by unusual patterns in the blood vessel distribution. The SOS images also show contrast differences where tumours are located and help provide context to these blood vessel morphologies.
**Conclusion:** The combination of PA and SOS images can reveal tumour-specific characteristics and may have diagnostic potential.
**Limitations:** The study was limited by the number of cases.
**Ethics committee approval:** NL71091.100.19
**Funding for this study:** REACT-EU, Pioneers in Health Care, H2020.
**Author Disclosures:**
Srirang Manohar: Grant Recipient: University of Twente
Margreet van der Schaaf: Nothing to disclose
Felix Lucka: Nothing to disclose
Ashkan Javaherian: Nothing to disclose
Ben Cox: Nothing to disclose
Rianne Fredrika Gerritdina Bulthuis: Investigator: University of Twente
Bruno De Santi: Nothing to disclose
Jeroen Veltman: Nothing to disclose
Maura Dantuma: Nothing to disclose

**15:00-16:00** **Research Stage 3**

### Research Presentation Session: Paediatric

### RPS 512

### Paediatric musculoskeletal and oncologic imaging

**Moderator**
L. Riera; Barcelona/ES

### RPS 512-2
**Imaging features of posterior fossa ependymoma subgroups**
T. Leclerc[1], R. Levy[1], A. Tauziède-Espariat[1], C-J. Roux[1], K. Beccaria[1], J. Grill[2], P. Varlet[1], N. Boddaert[1], *V. Dangouloff-Ros*[1]; [1]Paris/FR, [2]Villejuif/FR

**Purpose:** Posterior fossa ependymoma group A (EPN_PFA) and group B (EPN_PFB) can be distinguished by their DNA methylation and give rise to different prognoses. We compared the MRI characteristics of EPN_PFA and EPN_PFB at the presentation.
**Methods or Backround:** Preoperative images of 68 patients with posterior fossa ependymoma were reviewed, blinded for histomolecular grouping. Location, tumour volume, calcifications, tissue component, enhancement or diffusion signal, and histopathological data were compared between the groups.
**Results or Findings:** Fifty-six patients were categorised as EPN_PFA and 12 as EPN_PFB, with median ages of 2 and 20 years, respectively (p=0.0008). The median EPN_PFA tumoural volume was larger (57 vs 29 cm3, p =0.003). EPN_PFA showed an exclusive central position within the 4th ventricle in 61% of patients vs 92% for EPN_PFB (p=0.047). Intratumour calcifications were found in 93% of EPN_PFA vs 40% of EPN_PFB (p=0.001). Invasion of the posterior fossa foramina was mostly found for EPN_PFA, particularly the foramina of Luschka (p=0.0008). EPN_PFA showed a whole and homogeneous tumour enhancement in 5% vs 75% of EPN_PFB(p=0.0008). All mainly cystic tumours were EPN_PFB(p=0.002). The minimum and maximum relative ADC was slightly lower in EPN_PFA (p=0.02 and p=0.01, respectively).
**Conclusion:** Morphological characteristics from imaging make it possible to distinguish between posterior fossa ependymoma subtypes. A median or lateral tissular calcified mass, with incomplete enhancement, affecting young children and responsible for pronounced hydrocephalus and invasion of the posterior fossa foramina suggests the EPN_PFA group. Conversely, a median non-calcified mass of adolescents and adults, predominantly cystic, and minimally invasive, with total and homogeneous enhancement, suggests the EPN_PFB group.
**Limitations:** Small number of EPN_PFB than EPN_PFA (expected since they are rarer). Heterogeneous imaging protocols for advanced sequences.
**Ethics committee approval:** EDRACT 2014-A-00541-46.
**Funding for this study:** No funding was received for this study.
**Author Disclosures:**
Kevin Beccaria: Nothing to disclose
Thomas Leclerc: Nothing to disclose
Volodia Dangouloff-Ros: Nothing to disclose
Charles-Joris Roux: Nothing to disclose
Raphael Levy: Nothing to disclose
Nathalie Boddaert: Nothing to disclose
Arnault Tauziède-Espariat: Nothing to disclose
Jacques Grill: Nothing to disclose
Pascale Varlet: Nothing to disclose

### RPS 512-3
**Forensic age estimation by MRI of the knee: comparison of two classifications for ossification stages**
*D. Vogele*, V. Malokaj, M. F. Wernsing, M. J. Beer; Ulm/DE

**Purpose:** In forensic age estimation, e.g. for judicial proceedings, surpassed age thresholds can be legally relevant. To examine age-related differences in skeletal development, the recommendations by the Study Group on Forensic Age Diagnostics (AGFAD) are based on ionizing radiation (among others, orthopantomograms and plain x-rays of the hand). Vieth et al. and Ottow et al. proposed MRI classifications for the epiphyseal-diaphyseal fusion of the knee joint to determine different age groups in healthy volunteers. The aim of the present study was to directly compare these two classifications in a large patient population.
**Methods or Backround:** MRI of the knee joint of 900 patients (405 female, 495 male) from 10 to 28 years of age was retrospectively analysed. Acquired T1-weighted turbo spin-echo sequence (TSE) and T2-weighted sequence with fat suppression by short tau inversion recovery (STIR) were analysed for the two classifications. The different bony fusion stages of the two classifications were determined, and the corresponding chronological ages were assigned.

Differences between the sexes were analysed. Intra- and interobserver agreements were determined using Cohen's kappa.
**Results or Findings:** With the classification of Ottow et al., it was possible to determine the completion of the 18th and 21st years of life in both sexes. With the classification of Vieth et al., completion of the 18th year of life in both sexes and the 14th and 21st year of life for female patients could be determined. The intra- and interobserver agreement levels were very good (κ > 0.85).
**Conclusion:** In the large patient cohort of this study, it was possible to determine the 18th year of life with both MRI classifications and the 21st year of life with the classification of Ottow et al..
**Limitations:** Retrospective single centre study.
**Ethics committee approval:** Ethics Committee University Ulm.
**Funding for this study:** No funding was received for this study.
**Author Disclosures:**
Maximilian Frederic Wernsing: Nothing to disclose
Valesa Malokaj: Nothing to disclose
Daniel Vogele: Nothing to disclose
Meinrad Johannes Beer: Nothing to disclose

### RPS 512-4

**MR angiography facilitates the differentiation of aneurysmal from unicameral bone cysts**

*L. D. Grünewald*, V. Koch, C. Booz, I. Yel, S. Martin, T. Vogl; Frankfurt am Main/DE
*(leonosddc@gmail.com)*

**Purpose:** The objective of this work was to evaluate the incremental value of magnetic resonance angiography over plain radiographs and MRI for the differentiation of aneurysmal bone cysts (ABC) from unicameral bone cysts (UBC).
**Methods or Backround:** Thirty-six juvenile patients with histologically secured ABCs or UBCs were included in this retrospective study. Two radiologists assessed all obtained images in a blinded fashion using a catalogue of previously suggested imaging findings. A second readout with supplementary MR angiography images was performed after eight weeks to prevent observer recall bias. Diagnostic accuracy parameters were calculated for individual imaging findings, and the overall diagnostic accuracy and diagnostic confidence were assessed for all readouts. ROC curve comparison was used to determine the incremental value of MR angiography.
**Results or Findings:** Of sixteen imaging features, only abnormal vascularisation in MR angiography provided a significant diagnostic accuracy for the identification of ABCs. Other imaging features, such as fluid-fluid levels and internal septations, were insufficient for the differentiation of UBCs from ABCs. The availability of MR angiography images significantly increased the diagnostic accuracy (94.4% vs 75.0% and 83.3% vs 69.4%, respectively, p< 0.05) and diagnostic confidence (4.5 vs 3.7, p<0.05) of reading radiologists.
**Conclusion:** The presence of arterial feeders in magnetic resonance angiography can accurately discriminate aneurysmal bone cysts from unicameral bone cysts and increases the diagnostic accuracy and diagnostic confidence of reporting radiologists.
**Limitations:** Small sample size. Exclusion of older patients.
**Ethics committee approval:** No information was provided by the submitter.
**Funding for this study:** No funding was received for this study.
**Author Disclosures:**
Simon Martin: Nothing to disclose
Christian Booz: Consultant: Siemens Healthineers
Ibrahim Yel: Speaker: Siemens Healthineers
Thomas Vogl: Nothing to disclose
Vitali Koch: Nothing to disclose
Leon David Grünewald: Nothing to disclose

### RPS 512-5

**Improved accuracy of a deep-learning model with carpal bone analysis for bone age assessment**

*J. H. Kim*, S-U. Kim, S. Oh, C. H. Kang; Seoul/KR

**Purpose:** The study aimed to evaluate the clinical significance of carpal bone analysis for improving bone age assessment (BAA) accuracy.
**Methods or Backround:** A pre-existing deep-learning BAA model was trained with an additional carpal bone analysis using an open dataset. A total of 153 hand radiographs (70 male, 83 female; mean age ± standard deviation [SD], 10.59±2.78 years) were selected for external validation. To build the reference standard, three human experts performed BAA based on the Greulich-Pyle atlas and the interobserver agreement was measured. Software performance was assessed by comparing the mean absolute difference (MAD) and root mean squared error (RMSE) of the two BAA models, one without (M1) and the other with (M2), the carpal bone analysis, with the reference standard. Considering the four major skeletal development stages, the absolute errors of the models were compared by age using repeated-measures analysis of variance.
**Results or Findings:** The values from the two BAA models did not differ significantly from the reference standard (p = 0.491 for M1, 0.995 for M2). The MAD between each model and the reference standard was 0.304 years (M1 model: 95% confidence interval [CI], 0.261–0.348) and 0.290 years (M2 model: 95% CI, 0.248–0.333). The RMSE vs the reference standard was 0.408 and 0.393 years for the M1 and M2 models, respectively. The M2 model showed better predictive ability than the M1 for males under 9 years of age (p = 0.016).
**Conclusion:** The addition of the carpal bone analysis to the BAA model improved bone age assessment accuracy in children of all ages.
**Limitations:** The reference standard for the external validation set was based on the GP atlas, which is inherently limited to children in this age group.
**Ethics committee approval:** This study was approved by the institutional review board and ethics committee of our institution, which waived the need for informed consent because the data were collected retrospectively and analysed anonymously.
**Funding for this study:** This study was financially and technically supported by Crescom Inc.
**Author Disclosures:**
Sang-Un Kim: Nothing to disclose
Joo Hui Kim: Nothing to disclose
Saelin Oh: Research/Grant Support: Crescom Inc.
Chang Ho Kang: Shareholder: Crescom Inc.

**15:00-16:00** **Research Stage 4**

### Research Presentation Session: Physics in Medical Imaging

### RPS 513

### Discovering the potential of spectral and photon-counting CT

**Moderator**
L. Sukupova; Prague/CZ
*(lucie.sukupova@ikem.cz)*

### RPS 513-2

**Image quality of a spectral photon-counting CT for ultra-high-resolution chest imaging compared to a dual-layer CT system**

*H. Lacombe*[1], J. Labour[1], J. Greffier[2], M. Villien[1], S. Boccalini[1], P. Douek[1], S. A. Si-Mohamed[1]; [1]Lyon/FR, [2]Nimes/FR
*(hugo.lacombe@philips.com)*

**Purpose:** The study aimed to assess the image quality of a standard and low-dose spectral photon counting CT (SPCCT) for ultra-high-resolution (UHR) chest imaging compared to a dual-layer CT (DLCT).
**Methods or Backround:** The 21-cm diameter section of the Mercury 4.0 phantom was scanned at 10 and 6 mGy using an SPCCT and a DLCT (CT7500) (Philips Healthcare). SPCCT images were reconstructed using a 0.29x0.29x0.29 mm voxel size, a Sharp kernel. DLCT images were reconstructed using a 0.58x0.58x0.67 mm voxel size, a YC kernel. An iterative algorithm at level 6 was used for both images. Noise power spectrum (NPS), task-based transfer function (TTF) and detectability index (d') were assessed for ground-glass nodule (GGN) and solid nodule of 2 mm using IQMetrix software.
**Results or Findings:** At 10 mGy, the noise with SPCCT was reduced by ~12% compared to DLCT. At 6 mGy, the noise was increased by ~12% compared to DLCT at 10 mGy. fmean of the NPS with SPCCT was higher at 10 mGy (0.82) as well at 6 mGy (0.77), compared to CT7500 at 10 mGy (0.63). For both inserts, TTF values at 50% (f50) with SPCCT were higher compared to EID-CT, of 0.79 vs 0.66 mm-1 for GGN and 0.98 vs 0.62 mm-1 for the solid nodule. At 6 mGy, f50 was higher compared to DLCT at 10 mGy, of 0.78 and 0.92 mm-1 for GGN and the solid nodule, respectively. Finally, at 10 mGy, d' with SPCCT was higher compared to DLCT for GGN (8.8vs5.5) and the solid nodule (14.4 vs 11.6). At 6 mGy, d' was higher with SPCCT compared to DLCT at 10 mGy for GGN (6.8vs5.5) and similar for the solid nodule (11.4 vs 11.6).
**Conclusion:** UHR imaging with SPCCT may improve the diagnosis of lung nodules while enabling a significant dose reduction.
**Limitations:** This is a phantom study.
**Ethics committee approval:** No information provided by the submitter.

**Funding for this study:** The study was funded by the European Union H2020 668142.
**Author Disclosures:**
Marjorie Villien: Employee: Philips Healthcare
Salim Aymeric Si-Mohamed: Nothing to disclose
Joey Labour: Nothing to disclose
Philippe Douek: Nothing to disclose
Hugo Lacombe: Research/Grant Support: Philips Healthcare
Sara Boccalini: Nothing to disclose
Joel Greffier: Nothing to disclose

### RPS 513-3

**Multi-contrast K-edge imaging with a whole-body edge-on silicon detector photon-counting CT scanner: initial experience**

T. W. Holmes[1], Z. Yin[2], R. Bujila[3], J. Yao[3], J. Maltz[3], *A. Pourmorteza*[1]; [1]Atlanta, GA/US, [2]Niskayuna, NY/US, [3]Waukesha, WI/US
*(amir.pourmorteza@gmail.com)*

**Purpose:** Multi-contrast imaging is one of the unique applications of photon-counting detector (PCD) CT technology. Simultaneous detection and quantification of two contrast agents in the body in an "inject twice, scan once" fashion reduces the radiation dose of multi-phasic CT scans by half while generating co-registered maps of the anatomy in the two phases. First-generation PCDs, with high-Z materials, are deployed commercially, while silicon-based PCDs for CT were introduced recently. Silicon detectors have desirable properties such as narrow photoelectric peaks, low charge sharing, and characteristics that can lead to good spectral fidelity. In this work, we investigate K-edge imaging capabilities of a prototype whole-body edge-on silicon PCD-CT (GE Healthcare, USA) offering 8 energy bins.
**Methods or Backround:** A multi-energy CT phantom (Gammex) with custom-made inserts of varying concentrations of iodine and gadolinium was scanned. The scan parameters were: 120 kVp, 350 mAs, and 1-second rotation time, with two of the thresholds set to bracket the k-edge of gadolinium at 50 keV. The last 5 thresholds were used for linear material decomposition. Four regions of interest were used for calibration, 4 other regions were used to test the algorithm.
**Results or Findings:** The k-edge effect of gadolinium was visible as a peak in HU of bin# 6. We used the data for calibrating the two-material decomposition algorithm. Maximum errors for estimating iodine and gadolinium concentrations were 0.09 mg I/mL and 0.23 mg Gd/mL, respectively, for 5-bin material decomposition.
**Conclusion:** Edge-on irradiated silicon PCD-CT can perform K-edge imaging and thus quantify concentrations of two contrast agents simultaneously. More experiments with denoising algorithms are warranted.
**Limitations:** This was a phantom study on a prototype CT scanner.
**Ethics committee approval:** No information provided by the submitter.
**Funding for this study:** This study was funded by a sponsored research agreement between GE Healthcare and Emory University.
**Author Disclosures:**
Jonathan Maltz: Employee: GE healthcare
Robert Bujila: Employee: GE healthcare
Amir Pourmorteza: Grant Recipient: GE healthcare
Thomas Wesley Holmes: Nothing to disclose
Jingwu Yao: Employee: GE healthcare
Zhye Yin: Employee: GE healthcare

### RPS 513-4

**Determination of iron content in soft tissues through fast kV switching dual-energy CT imaging: calibration data required**

*K. Perisinakis*, A. Ntoulis, T. G. Maris; Iraklion/GR
*(perisynk@uoc.gr)*

**Purpose:** To provide calibration data for converting the tissue virtual iron content (VIC) derived from material density images produced through fast-kV switching dual-energy (DE)CT imaging to true iron concentration (TIC).
**Methods or Backround:** Several water solutions with varying concentration of agarose or gelatin were tested to determine soft tissue-like solutions depicted with CT numbers 0 and 30 HU, respectively, in the monochromatic 70 keV CT images. Soft-tissue mimicking solutions were mixed with an increasing amount of a pharmaceutical compound of iron to produce iron concentrations 0-15 mg iron/ml. Vials containing prepared solutions were positioned around a standard head PMMA CT phantom and subjected to fast kV-switching dual-energy CT imaging. The VIC of each prepared solution was determined from the material density iron(water) images. TIC against VIC calibration curves were thus derived. Different DECT acquisition protocols were employed to investigate the effect of acquisition protocol on the derived calibration curve.
**Results or Findings:** TIC was found to be linearly correlated with VIC ($R2=0.99$) for both soft-tissue mimicking solutions imaged at 0 and 30 HU when iron free, respectively. Differences between TIC vs VIC calibration curves derived for soft tissues of different density were found to be up to 15%. The use of different DECT imaging acquisition protocols was found to result in essentially the same TIC-VIC curves since differences were found to be <5%.
**Conclusion:** TIC-VIC calibration curves determined herein may be used to measure iron content in soft tissues such as kidneys, pancreas, and myocardium through fast kV switching DECT imaging.
**Limitations:** Calibration data provided may be used to determine TIC in soft tissue depicted with a CT number 30HU (e.g. kidneys) only. Efforts are currently made to produce corresponding calibration data for liver (CT number 60HU).
**Ethics committee approval:** No information provided by the submitter.
**Funding for this study:** No funding was received for this study.
**Author Disclosures:**
Konstantinos Perisinakis: Nothing to disclose
Thomas G. Maris: Nothing to disclose
Aggeliki Ntoulis: Nothing to disclose

### RPS 513-5

**Dental imaging in the first clinical photon-counting CT system: comparison to digital volume tomography (DVT)**

S. Sawall[1], J. Maier[1], S. Sen[1], H. Gehrig[1], T-S. Kim[1], H-P. Schlemmer[1], S. O. Schönberg[2], *M. Kachelrieß*[1], M. Rütters[1]; [1]Heidelberg/DE, [2]Mannheim/DE
*(marc.kachelriess@dkfz.de)*

**Purpose:** The study aims to investigate potential benefits of the first clinical photon-counting computed tomography (PCCT) system for dental imaging in comparison to digital volume tomography (DVT).
**Methods or Backround:** Clinical PCCT systems offer an increased spatial resolution compared to previous generations of clinical systems that are in the order of dental DVT systems. The used photon-counting detectors offer a dynamic range higher than the flat detectors used in DVTs, and together with high-power x-ray tubes and tin prefiltration, this might allow for a similar image quality in clinical PCCTs compared to DVTs at a lower dose level. To test this, resolution- and dose-matched acquisitions of 10 porcine jaws were performed in a DVT (Orthophos XL, Dentsply Sirona) and a PCCT (Naeotom Alpha, Siemens Healthineers). A low-dose (LD, 0.34mGy) and a high-dose (HD, 4.7mGy) protocol is investigated in both systems. The DVT is operated using a tube voltage of 85kV, whereas the PCCT uses 100kV and a tin prefilter. Image reconstruction is performed using the standard algorithms of each system to a voxel size of $0.16\times0.16\times0.20$mm. Image noise in soft tissue and contrast-to-noise ratio (CNR) of relevant dental structures is evaluated.
**Results or Findings:** Noise measured in soft tissue is similar in both systems (DVT/PCCT-LD: ±67/±63HU, DVT/PCCT-HD: ±30/±29HU). PCCT acquisitions show a higher CNR for all investigated structures. E.g. for the HD protocol, the CNR between soft tissue and bone is 9.15 in DVT acquisitions and 19.55 in PCCT acquisitions. This increase in CNR corresponds to a dose reduction of up to 78%.
**Conclusion:** Clinical PCCT offers an advantage in terms of image quality and radiation dose efficacy in comparison to conventional DVTs for dental diagnostics.
**Limitations:** The study is limited to porcine ex-vivo samples since multiple measurements are required.
**Ethics committee approval:** Not applicable.
**Funding for this study:** No funding was received for this study.
**Author Disclosures:**
Ti-Sun Kim: Nothing to disclose
Stefan Oswald Schönberg: Nothing to disclose
Holger Gehrig: Nothing to disclose
Maurice Rütters: Nothing to disclose
Heinz-Peter Schlemmer: Nothing to disclose
Marc Kachelrieß: Nothing to disclose
Stefan Sawall: Nothing to disclose
Joscha Maier: Nothing to disclose
Sinan Sen: Nothing to disclose

### RPS 513-6

**Systematic evaluation of the impact of scan parameters and coexisting iron on dual-layer detector spectral-CT fat quantification**

*I. Molwitz*[1], G. Campbell[1], M. Grosser[1], G. B. Adam[1], J. Yamamura[2], T. Knopp[1], P. Szwargulski[1]; [1]Hamburg/DE, [2]Berlin/DE

**Purpose:** The study aimed to evaluate the impact of tube voltage, dose right index, and iron on fat quantification in dual-layer detector spectral CT (dslCT), for which spectral information is newly available below 120 kV.
**Methods or Backround:** In this experimental study, phantoms with 0, 4, and 8mg/cm3 iron; 0, 2.5, and 5mg/cm3 iodine; 0, 10, 20, 35, 50, and 100% fat and liver tissue, respectively, were scanned in a measurement set-up representative for human body size (Gammex, Sun Nuclear Corporation, Australia) by a dlsCT of the newest generation (CT7500, Philips, the Netherlands). Scan parameters were 100, 120, and 140kV with 16, 20, and 24 dose right index (DRI). Material decomposition was conducted using calibration data from 120kV with 20 DRI as standard clinical parameters. For decomposition reference vectors of fat, liver tissue, and 1. iodine, 2. iron, and

3. the combined iodine+iron vector was employed. Differences were analysed by the paired-t-test, agreement by the intraclass correlation coefficient (ICC).
**Results or Findings:** Fat quantification results varied between 120 kV and both 100 kV (-1.09±3.51%, P=.037) and 140 kV (1.30±1.69%, P<.001). At the same kV for different DRI, the fat fraction differed significantly only between 20 to 24 DRI at 100 kV (-1.07±1.57%, P=.015). In phantoms with coexisting iodine and iron fat material decomposition without iron underestimated fat (mean 4.9±0.1%), while results for the combined iodine+iron vector were excellent (ICC. 0.999 [0.991-1]). For phantoms with zero iron or zero iodine, all vectors (iodine, iron, iodine+iron) delivered excellent results (respective ICC >.9)
**Conclusion:** Fat quantification in dlsCT is feasible <120 kV. Calibration to tube voltage is necessary. Material decomposition assuming the presence of both iodine and iron delivers the most robust results of fat contents.
**Limitations:** Phantom study.
**Ethics committee approval:** Ärztekammer Hamburg PV7006-4406-BO.
**Funding for this study:** The study was funded by the Hamburg Research Center for Medical Technology (04fmthh2020).
**Author Disclosures:**
Isabel Molwitz: Research/Grant Support: Faculty funding
Graeme Campbell: Employee: Philips Healthcare. All data handling and analyzes were performed by the independent authors Molwitz I, Szwargulski P.
Tobias Knopp: Nothing to disclose
Gerhard B. Adam: Nothing to disclose
Patryk Szwargulski: Research/Grant Support: Faculty funding
Jin Yamamura: Employee: There is no connection of this work to the evidia group. Prof. Yamamura is also affiliated to the department of diagnostic and interventional radiology and nuclear medicine at the university hospital Hamburg-Eppendorf and in this position counseled this work.
Mirco Grosser: Nothing to disclose

### RPS 513-7

**Clinical photon-counting CT: the small pixel effect and its implications for dose reduction**

*M. Fix*, Y. P. Kayser, L. Klein, J. Maier, L. T. Rotkopf, H-P. Schlemmer, S. O. Schönberg, M. Kachelrieß, S. Sawall; Heidelberg/DE

**Purpose:** The study aims to assess the potential dose reduction achievable with clinical photon counting CT (PCCT) in ultra-high-resolution (UHR) mode compared to acquisitions using standard spatial resolution.
**Methods or Backround:** With smaller detector pixels, PCCT achieves far higher spatial resolution than energy-integrating CT systems. The reconstruction of UHR acquisitions to the lower spatial resolution of conventional systems results in an image noise and radiation dose reduction [InvestRadiol 55(2):111-19, 2020]. PCCT itself allows to select two different detector pixel sizes. We quantify the small pixel effect by measuring semi-anthropomorphic abdominal phantoms of different body sizes (S=20×30 cm, M=25×35 cm, L=30×40 cm) in the first clinical PCCT system (Naeotom Alpha, Siemens Healthineers, Forchheim, Germany) once using the UHR mode (S1, 0.2 mm pixel size at isocenter) and once using the standard resolution mode (M4, 0.4 mm, resulting from 2x2 binning of the S1 pixels). Using all available tube voltages and reconstruction kernels, FBP reconstruction was performed to the same target spatial resolution (same xyz-MTF). Image noise was evaluated, and the potential dose reduction was quantified.
**Results or Findings:** Images acquired using UHR pixels yield lower noise in comparison to acquisitions using standard spatial pixels at the same spatial reconstruction. This particularly holds for sharper convolution kernels at the spatial resolution limit of the standard mode (e.g. up to a factor 3.2 in noise reduction and potential dose reduction of 90.5% for the B76 kernel).
**Conclusion:** Using sharper convolution kernels, UHR acquisitions allow for a significant dose reduction compared to acquisitions with standard resolution.
**Limitations:** This study is limited to phantoms only.
**Ethics committee approval:** No information provided by the submitter.
**Funding for this study:** No funding was received for this study.
**Author Disclosures:**
Stefan Oswald Schönberg: Nothing to disclose
Yannick Paul Kayser: Nothing to disclose
Laura Klein: Nothing to disclose
Lukas Thomas Rotkopf: Nothing to disclose
Heinz-Peter Schlemmer: Nothing to disclose
Marc Kachelrieß: Nothing to disclose
Stefan Sawall: Nothing to disclose
Markel Fix: Nothing to disclose
Joscha Maier: Nothing to disclose

### RPS 513-8

**Quantitative multi-parameters analysis between 100-kVp and 120-kVp protocols in second-generation dual-layer spectral CT: a phantom study**

*Y. Wang*[1], Y. Zhang[1], S. Dong[2]; [1]Zhengzhou/CN, [2]Beijing/CN

**Purpose:** The study aimed to evaluate the feasibility of 100kVp on spectral CT imaging by comparing 120kVp spectral parameters in a phantom experiment.
**Methods or Backround:** We scanned a multi-energy CT phantom consisting of calibrated inserts. The 100kVp and 120kVp protocols both selected five tube current-time values at 10, 30, 50, 75, 100, and 150 mAs. Other scan and reconstruction parameters consisted of 128x0.6 collimation; and 0.5-second rotation time. Raw data were reconstructed with iDose in level4. Slice thickness and increment were 3mm×3mm. The effective atom number, iodine density, and electron density of the two protocols were compared. Virtual monoenergetic images (VMIs) from 40kev to 200kev were reconstructed for comparison with the actual attenuation values from the phantom instructions.
**Results or Findings:** The results of the quantitative analysis showed that multi-parameters were similar in the same dose levels under different kVp (11.22±0.0 vs 11.24±0.0 in effective atom number, 10.26±0.1 vs 10.38±0.1 in iodine density, 102.4% ±0.2% vs 102.7% ±0.2% in electron density, respectively). That is to say, the quantitative results of spectral CT multi-parameter analysis are minimally affected regardless of the tube voltage at 100kVp or 120kVp. In comparison with the standard references, 90keV VMIs in 100kVp protocols were closer to the values of calibrated inserts, while 120keV VMIs in 120kVp were closer to the actual values.
**Conclusion:** Spectral parameters in the different tube voltages were rarely impacted in the same radiation dose level. The multi-parameter in 90keV VMIs of 100 kVp protocols and 120keV VMIs of 120kVp protocols were closer to the reference standards.
**Limitations:** Phantom study only.
**Ethics committee approval:** Not applicable. This is a phantom study.
**Funding for this study:** No funding was received for this study.
**Author Disclosures:**
Shushan Dong: Nothing to disclose
Yiran Wang: Nothing to disclose
Yonggao Zhang: Nothing to disclose

**16:30-17:30** **Research Stage 1**

### Research Presentation Session: Abdominal Viscera & GI Tract

### RPS 601

### Upper GI tract and small bowel imaging

**Moderator**
S. K. Puri; New Delhi/IN
*(skpurigbph@yahoo.co.in)*

### RPS 601-2

**Electrical Impedance Tomography (EIT) Examination of Swallowing**

*A. Venugopal*, M. J. Kraśny, N. Colgan; Galway/IE
*(a.venugopal1@nuigalway.ie)*

**Purpose:** Evaluate the feasibility of an EIT-based investigation of swallowing in comparison to Ultrasound (US).
**Methods or Backround:** This study demonstrates the swallowing process in adult volunteers with both US and EIT modalities and evaluates the performance of the EIT system against the US. Bolus used was 25-30 ml of tap water. We used the SenTec EIT Pioneer system with 32 electrodes Neonatal EIT belt and 50 fps recording speed, and the Hitachi EUB-6500 HV system with L53S Probe in B mode Carotid Artery Doppler setting.
**Results or Findings:** The SenTec EIT system detects and records swallow events with evident consistency and repeatability. Key stages of the swallow cycle were identified in co-registered images of EIT and US. Highlights include: (i) initial position of anatomical landmarks, (ii) movement of tongue base, (iii) pharyngeal stage of swallowing, and (iv) bolus progression. US recordings are shown in colour Doppler mode. EIT, being a functional modality, records swallowing action as rapid dynamic variations in colour intensities denoting significant impedance changes during the swallowing process. Differential impedance images are obtained in default time-difference imaging mode, where white regions represent the strongest impedance changes, and brightness reduces as impedance change reduces.
**Conclusion:** Swallowing action is detected and recorded by the SenTec EIT system, with enough data to conclude that a swallow is occurring. The consistency and repeatability of various stages of swallowing are evident despite the temporal difference in EIT imaging relative to real-time activity. Hence EIT image provides reliable information for the assessment of swallowing.
**Limitations:** This project is a feasibility study of an EIT-based system to obtain reliable information to assess swallowing. As a real-time differential image is obtained as default, an in-depth analysis is necessary to develop the algorithm for image reconstruction suitable for specific applications.
**Ethics committee approval:** School of Natural Sciences, University of Galway.

**Funding for this study:** No funding was received for this study.
**Author Disclosures:**
Marcin Jan Kraśny: Nothing to disclose
Ardra Venugopal: Nothing to disclose
Niall Colgan: Nothing to disclose

### RPS 601-3

**The utility of conventional CT and CT perfusion in predicting the high grade of gastrointestinal stromal tumour of the stomach: A prospective comparison of classical CT features and CT perfusion values**

*M. Mitrovic*, A. Djuric-Stefanovic, J. Kovac, A. Jankovic, D. Šaponjski, L. Lazic, K. Stošić, V. Cvetic, D. Mašulović; Belgrade/RS
*(dr_milica@yahoo.com)*

**Purpose:** The role of advanced functional imaging techniques in the prediction of pathological risk categories of gastrointestinal stromal tumours (GIST) is still unknown. The purpose of this study was to evaluate classical CT features and CT-perfusion parameters in predicting the histopathologic grade of gastric GIST.
**Methods or Backround:** Sixty-two patients with histologically proven gastric GIST who underwent abdominal CT exams with CT perfusion were prospectively included. Morphological CT characteristics and CT-perfusion parameters of tumors were comparatively analyzed in the high-risk (HR) and low-risk (LR) GIST groups.
**Results or Findings:** Binary logistic regression analysis revealed that greater tumour diameter ($p<0.001$), cystic structure ($p<0.001$), irregular margins ($p=0.007$), irregular shape ($p<0.001$), disrupted mucosa ($p<0.001$), visible EFDV ($p<0.001$), and shorter time-to-peak ($p=0.006$) were significant predictors of HR GIST. Multivariate analysis extracted the irregular shape ($p=0.006$) and visible EFDV ($p=0.017$) as independent predictors of HR GIST (AUC of predicting model 0.869). Our results also show a tendency for higher values of perfusion parameters in high aggressive gastric GISTs, as well as shorter MTT. However, there was no statistically significant difference except for PS and TTP.
**Conclusion:** Although certain classical CT imaging features remain the most valuable, some functional imaging parameters may add diagnostic value in preoperative predicting the HR gastric GIST.
**Limitations:** Our study has several limitations, such as a relatively small patient sample, and our study did not include the follow-up of the involved patients.
**Ethics committee approval:** Ethics Committee Name: Ethical Committee of School of Medicine, University of Belgrade. Approval Code: 1322/II-6. Approval Date:20.02.2020.
**Funding for this study:** This research received no external funding.
**Author Disclosures:**
Dušan Šaponjski: Nothing to disclose
Aleksandra Jankovic: Nothing to disclose
Vladimir Cvetic: Nothing to disclose
Jelena Kovac: Nothing to disclose
Katarina Stošić: Nothing to disclose
Ljubica Lazic: Nothing to disclose
Milica Mitrovic: Nothing to disclose
Dragan Mašulović: Nothing to disclose
Aleksandra Djuric-Stefanovic: Nothing to disclose

### RPS 601-4

**Magnetic Resonance Texture Analysis for assessment of bowel wall fibrosis in Crohn's disease**

I. Mercante[1], *C. Zanon*[1], A. Toniolo[1], G. Cabrelle[1], C. Campi[2], M. Scarpa[1], C. Lacognata[1], E. Quaia[1], F. Crimì[1]; [1]Padua/IT, [2]Genoa/IT

**Purpose:** Crohn's disease (CD) is a multi-factorial chronic relapsing disease. Patients can develop intestinal strictures containing various degrees of fibrosis. This study aims to improve the diagnostic accuracy of MRI texture analysis (TA) in grading intestinal wall fibrosis for defining personalized therapeutic management.
**Methods or Backround:** Thirty-five patients (12 F) with a confirmed CD diagnosis were included in this retrospective study. All patients underwent surgery for stenotic bowel loop resection and consequent histopathological examination. Specific software was used to draw intestinal stenotic wall portion volumes of interest (VOIs) on pre-operative MRI scans (T2 HASTE, ADC, arterial VIBE, late VIBE sequence). For each VOI, 164 first and second-order textural features were extracted. Patients were divided into three groups according to the degree of fibrosis identified by the pathologist on the surgical specimen in grade 0 (no fibrosis), grade 1 (mild/moderate fibrosis), and grade 3 (severe fibrosis). Textural features of the three groups were compared with the Kruskal-Wallis test; a p-value of 0.05 was considered statistically significant.
**Results or Findings:** There were statistically significant differences among the three groups in 26 parameters for the late VIBE sequence, 23 parameters for the ADC sequence, 16 for the arterial VIBE, and 5 for the T2 HASTE. Moreover, some of these (15 in ADC sequence, 13 in arterial VIBE sequence, 1 in late VIBE sequence) have a statistically significant difference among patients with mild/moderate or severe degrees of fibrosis.
**Conclusion:** MRI TA may play a role in the quantification of fibrosis in the bowel affected by CD. New radiomics improvements are promising tools that could support clinicians in the management of CD, improving the diagnostic and therapeutic course of the disease.
**Limitations:** Small sample size; retrospective nature; involved only one centre.
**Ethics committee approval:** No information provided by the submitter
**Funding for this study:** No funding was received for this study.
**Author Disclosures:**
Carmelo Lacognata: Nothing to disclose
Giulio Cabrelle: Nothing to disclose
Irene Mercante: Nothing to disclose
Marco Scarpa: Nothing to disclose
Chiara Zanon: Nothing to disclose
Alessandro Toniolo: Nothing to disclose
Filippo Crimì: Nothing to disclose
Emilio Quaia: Nothing to disclose
Cristina Campi: Nothing to disclose

### RPS 601-5

**Abdominal Tuberculosis on 3T MR Enterography(MRE)- An underexplored territory in a resource-limited setting**

*S. Pandey*, A. K. Sharma, S. K. Puri, P. Aggarwal; New Delhi/IN
*(saumyapand3y@gmail.com)*

**Purpose:** To study the imaging appearances in patients of abdominal tuberculosis on MRE, with an emphasis on intestinal tuberculosis.
**Methods or Backround:** Thirty patients suspected of intestinal tuberculosis on clinical grounds or previous non-MR radiological imaging, attending Gastroenterology OPD at G.B. Pant Hospital, New Delhi, were enrolled. MRE was performed on 3 Tesla MRIs. Follow-up- Confirmation of diagnosis based on histopathological findings, nucleic acid detection or clinical improvement on ATT (anti-tubercular treatment) over the next three months. The total duration of study- 1 year.
**Results or Findings:** Out of the 30 patients imaged, 22 were diagnosed with cases of intestinal tuberculosis. Imaging characteristics of bowel lesions like the site, length of the lesion, degree of thickness, enhancement pattern and diffusion characteristics were noted. Extra-intestinal lesions like patterns of involvement of lymph nodes, greater omentum, peritoneum and adnexa were also noted.
**Conclusion:** MRE showed a high sensitivity(~100%) and positive predictive value(~88%) in detecting cases of abdominal tuberculosis, including intestinal involvement. MRE offered the advantage of a multi-parametric evaluation of suspected lesions, especially motility and diffusion characteristics, raising confidence while reporting bowel lesions. Thus, MRE has the potential to become a one-stop, cross-sectional imaging modality for diagnosing intestinal tuberculosis.
**Limitations:** Limited sample size. Insufficient time for follow-up due to time constraints. Lack of a comparison arm with other modalities like ultrasound and computed tomography.
**Ethics committee approval:** INSTITUTIONAL ETHICS COMMITTEE MAULANA AZAD MEDICAL COLLEGE AND ASSOCIATED HOSPITAL LokNayak, Govind Ballabh Pant Institute of Post Graduate Medical Education Research Hospital and Guru Nanak Eye Centre, Chacha Nehru Bal Chikitasalya, New Delhi-110002
**Funding for this study:** No funding has been received for this study.
**Author Disclosures:**
Saumya Pandey: Nothing to disclose
Piyush Aggarwal: Nothing to disclose
Sunil Kumar Puri: Nothing to disclose
Ashok Kumar Sharma: Nothing to disclose

**16:30-17:30** **Research Stage 2**

### Research Presentation Session: Genitourinary

### RPS 607

### Renal and excretory system pathology: advanced techniques and new approaches

**Moderator**
M.-F. Bellin; Le Kremlin-Bicêtre/FR

### RPS 607-2

**No gadolinium in renal allograft perfusion assessment: no problem**

*T. Radovic*, M. M. Jankovic, R. Stevic, B. Spasojevic, M. Kostic, M. Cvetkovic, I. Gojkovic, P. Pavicevic; Belgrade/RS

**Purpose:** Perfusion represents blood flow at the level of the tissue capillary bed and determines the delivery of nutrients and oxygen, while kidneys regulate glomerular filtration rate (GFR) as a central measure of renal function. The development of a non-invasive and reliable method for renal perfusion estimation without using an exogenous contrast agent that would reflect GFR would significantly improve the on-time identification of potential allograft injury. We aimed to discriminate renal allografts with impaired function in paediatric and young adult patients by measuring cortical renal blood flow (cRBF), as well as a comparison with cRBF values of healthy controls using magnetic resonance imaging arterial spin labelling (ASL-MRI).
**Methods or Backround:** We performed ASL-MRI in 20 allograft patients and 20 healthy controls to calculate cRBF on parameter maps. It was correlated to calculated GFR and compared between patient groups with good (GFR≥60 mL/min/1.73m2) and impaired allograft function (GFR<60 mL/min/1.73m2) as well as with healthy controls.
**Results or Findings:** cRBF in the patient group ranged between 85 and 335 mL/100 g/min (mean 190.05 ± 67.62 mL/100 g/min). Mean cRBF in patients with good allograft function was significantly higher than in patients with impaired function (225.91±64.38 vs 146.22±41.84 mL/min/100g, p=0.005), showing a highly significant correlation with GFR in all subjects (r=0.64, p=0.002). In healthy controls, mean cRBF was significantly higher than in patients with poor allograft function (322.00±121.36 vs 146.22±41.84 ml/100gr/min, p=0.002) and showed no difference to patients with stable function (322.00±121.36 vs 225.91±64.38 ml/100gr/min, p=0.056).
**Conclusion:** Cortical perfusion, as non-invasively measured by ASL-MRI, differs between patients with good and impaired allograft function and correlates significantly with its function.
**Limitations:** A low number of included participants and variability in the time interval from transplantation to study day.
**Ethics committee approval:** This prospective study was approved by the University Children's Hospital Ethics Committee (application numbers 14/303/22/08/2018) and informed consent was obtained from all participants and their legal guardians, if necessary.
**Funding for this study:** This is a non-profitable study.
**Author Disclosures:**
Brankica Spasojevic: Nothing to disclose
Polina Pavicevic: Nothing to disclose
Mirjana Cvetkovic: Nothing to disclose
Ruza Stevic: Nothing to disclose
Tijana Radovic: Nothing to disclose
Milica M. Jankovic: Nothing to disclose
Mirjana Kostic: Nothing to disclose
Ivana Gojkovic: Nothing to disclose

### RPS 607-3

**Assessment of renal allograft rejection with diffusion tensor imaging (DTI)**

*C. J. Das*, S. Agarwal; New Delhi/IN

**Purpose:** To investigate the value of DTI in differentiating renal allograft rejection from well-functioning stable allograft, using fractional anisotropy (FA) and apparent diffusion coefficient (ADC) values.
**Methods or Backround:** Renal biopsy is a gold standard method for assessing renal allograft dysfunction and graft rejection. Diffusion tensor imaging (DTI) has been evaluated as a non-invasive alternative to biopsy to assess graft rejection.
**Results or Findings:** Among 42 transplant recipients recruited in our study, 22 had well-functioning stable allograft (group A), 7 had acute allograft rejection (group B), and 13 had chronic allograft rejection (group C). A statistically significant difference between group A and renal allograft rejection (group B+C) was noted for cortical(p<0.001), medullary(p=0.003) FA values, and cortical(p=0.020), and medullary(p=0.046) ADC values. Cortical(p<0.001) and Medullary(p=0.020) FA values showed a statistically significant difference between group A and group C, and cortical FA value(p=0.012) also showed a statistically significant difference between group B and group C. AUC (to differentiate between renal allograft rejection and well-functioning stable allograft) for cortical, and medullary FA values, and cortical and medullary ADC values were 0.853(p<0.001), 0.757(p=0.004), 0.709(p=0.021), and 0.736(p=0.009), respectively.
**Conclusion:** DTI is a promising functional MRI technique for the non-invasive assessment of renal allograft function. Diffusion parameters, such as FA and ADC values, can be useful in the differentiation of renal allograft rejection from well-functioning stable allograft.
**Limitations:** The primary limitation of our study is the smaller sample size. Histopathological correlation was only available for patients with biochemical renal function impairment and graft rejection. Renal biopsy correlation was not done for stable allograft recipients as these patients do not require biopsy as per the standard of care.
**Ethics committee approval:** No information provided by the submitter.
**Funding for this study:** Funding was received from department of Science and Technology, Government of India.
**Author Disclosures:**
Chandan Jyoti Das: Nothing to disclose
SK Agarwal: Nothing to disclose

### RPS 607-4

**Percutaneous nephrostomy and ureteric stenting for malignant ureteric obstruction - is it justified?**

*O. Llewellyn*; Edinburgh/UK

**Purpose:** Malignant ureteric obstruction (MUO) occurs in advanced malignancy, but there is no standard approach to management. Renal decompression can be achieved with percutaneous nephrostomy (PCN) or ureteric stunting (US). These interventions may improve renal function, facilitating further oncological treatment, but they come with a quality of life implications.
**Methods or Backround:** Retrospective multicentre audit of patients who underwent PCN or US for MUO 2008-2020 across 6 Scottish health boards. Patients were identified by OPERA (theatre) and RIS (radiology) codes. Primary outcomes were overall survival (OS) following decompression and the development of a scoring tool to predict 3-month survival. Secondary outcomes changed in renal function three months post-intervention and receipt of further treatment. Cox and logistic regression were used to generate the Scottish MUO score. Missing variables were imputed. For validation, the entire cohort was split 70:30 into discovery: validation groups.
**Results or Findings:** In 905 patients, OS was poor, with 26% mortality at three months and 42% at six months. Increased age, potassium, WCC, CRP, lower haemoglobin and bilateral hydronephrosis were associated with poorer OS. The Scottish MUO Score was developed and validated, available at:(https://webapps.igmm.ed.ac.uk/world/research/muo_calculator/). It compares favourably with other scores with higher AUC and specificity. 85% of patients had eGFR<60 prior to intervention. 50% of these had a >20% improvement in creatinine at 3months. 43% of patients received further oncological treatment following an intervention.
**Conclusion:** This study demonstrates MUO as a marker of advanced disease. We developed the Scottish MUO score, which is valid for predicting low overall survival. We hope its use will guide decision-making around the intervention.
**Limitations:** Retrospective study design.
**Ethics committee approval:** No information provided by the submitter.
**Funding for this study:** Funding was received from BSIR Audit Bursary
**Author Disclosures:**
Oliver Llewellyn: Nothing to disclose

### RPS 607-5

**Utility of pre- and post-immunotherapy MRI to predict the pathological response in muscle-invasive bladder cancer (MIBC)**

*G. Brembilla*, G. Basile, D. Raggi, L. Marandino, M. Moschini, F. Montorsi, A. Briganti, F. De Cobelli, A. Necchi; Milan/IT
*(brembilla.giorgio@hsr.it)*

**Purpose:** To analyse the ability of VIRADS to predict the pT0 or pT≤1 response post-immunotherapy (IO) for muscle-invasive bladder cancer (MIBC).
**Methods or Backround:** In the PURE-01 trial, patients (pts) were staged with multiparametric MRI (mpMRI) of the bladder before and after neoadjuvant immunotherapy (Pembrolizumab) for MIBC. All patients underwent radical cystectomy. All mpMRI scans were centrally reviewed, and imaging parameters were recorded, including the VIRADS score. Logistic regression models analyzed pre- and post-pembro VIRADS against pT≤1 (primary endpoint) and pT0 (secondary endpoint). Covariates included cT-stage, age, gender, PD-L1 combined positive score (CPS) and tumour mutational burden (TMB).

**Results or Findings:** 58 pts were retrospectively reviewed (116 mpMRI). Pre-pembro: 8 pts (13.8%) had no measurable disease (VIRADS=0), 20 (34.5%) a VIRADS 1-3 score, and 30 (51.7%) had a VIRADS 4-5 score. Both pre-pembro and post-pembro VIRADS 0-3 scores were significantly associated with pT≤1 on multivariable analyses (MVA): the strongest effect was seen with post-pembro VIRADS 0-3 against pT≤1 response (OR: 30.2, 95%CI: 6.2-223.2, p<0.0001). The AUC of this model was 0.92. Regarding pre-pembro VIRADS 0-3: OR: 4.35, 95%CI: 1.1-19.7, p=0.04; AUC: 0.83. CPS was another significant variable for pT≤1 endpoint only in MVA using pre-pembro VIRADS 0-3 (OR: 1.02, 95%CI: 1-1.05, p=0.02). Post-pembro VIRADS 0-3 was also associated with pT0 on MVA (OR: 4.59, 95%CI: 1.2-19.6, p=0.02; AUC: 0.78), whereas pre-pembro VIRADS was not (p=0.13).
**Conclusion:** To our knowledge, this is the first evidence establishing the predictivity of the VIRADS score towards the pathological downstaging, both in the pre and post-IO settings. Post-pembro VIRADS, along with the combination of pre-pembro VIRADS and CPS, emerged as the strongest features based on which selecting pts for bladder-sparing strategies.
**Limitations:** Small sample size, retrospective analysis.
**Ethics committee approval:** No information provided by the submitter.
**Funding for this study:** No limitations were identified.
**Author Disclosures:**
Francesco Montorsi: Nothing to disclose
Marco Moschini: Nothing to disclose
Daniele Raggi: Nothing to disclose
Giuseppe Basile: Nothing to disclose
Laura Marandino: Nothing to disclose
Giorgio Brembilla: Nothing to disclose
Francesco De Cobelli: Nothing to disclose
Andrea Necchi: Nothing to disclose
Alberto Briganti: Nothing to disclose

### RPS 607-6

### Is the Excretory Phase Necessary to Identify Upper Tract Uroepithelial Neoplasms at CT Urography? A 10-Year Population-Based Study

*W. V. Limaye*, A. Fenwick, R. Mason, A. F. T. Costa; Halifax, NS/CA
*(warda.limaye@dal.ca)*

**Purpose:** To assess the proportion of upper tract uroepithelial carcinomas (UTUC) that are evident without the excretory phase of CT urography (CTU) based on the presence of secondary imaging findings.
**Methods or Backround:** Patients with a history of UTUC between January 2008-December 2017 were retrospectively identified from a population-based cancer registry. For each patient, US, non-CTU CT, and MRI exams were reviewed to assess for a primary abnormality (mass) and secondary findings: hydronephrosis; urinary tract thickening; focal luminal distention; fat stranding; lymphadenopathy; and distant metastasis. For patients who underwent CTU, the excretory phase was reviewed to assess if the tumour was evident as a filling defect (primary abnormality); non-excretory phases were assessed for secondary findings that would enable a diagnosis of UTUC.
**Results or Findings:** Three hundred twelve patients (mean age, 73+/-11 years, 176 males, 136 females) who underwent 585 imaging examinations were included. 209/312 (67.0%) patients underwent non-CTU imaging; in these, the primary mass was evident in 142/209 (67.9%) patients, secondary findings were evident in 176/209 (84.2%) patients, and primary or secondary findings were evident in 184/209 (88.0%) patients. Of 181/312 (58.0%) patients that underwent CTU, the UTUC was evident as a filling defect in 133/181 (73.5%) CTUs. The mass was evident on non-excretory phases in 149/181 (82.3%) CTUs. Secondary findings were present in 172/181 (95.0%) CTUs.
**Conclusion:** The vast majority of UTUCs is evident on imaging as either a primary mass or via secondary findings, without the need for an excretory phase. This finding should be considered when updating clinical practice guidelines and imaging pathways.
**Limitations:** Retrospective. Variable imaging quality over the study period. Only patients with UTUC were assessed; only the sensitivity of imaging findings was assessed. Specificity, predictive values, and overall accuracy were outside the scope of this study.
**Ethics committee approval:** No information provided by the submitter.
**Funding for this study:** Not applicable.
**Author Disclosures:**
Andreu Francesc Torre Costa: Advisory Board: Bayer Inc.
Ross Mason: Nothing to disclose
Warda Vidyadhar Limaye: Nothing to disclose
Andrew Fenwick: Nothing to disclose

### RPS 607-7

### Applicability of Bosniak 2019 for renal mass classification on portal venous phase in the era of spectral CT imaging using rapid kV-switching dual-energy CT

E. Reizine[1], *M. Blain*[1], L. C. Pescatori[1], B. Longere[2], A. Ingels[1], W. Boughanmi[3], M. Bouanane[3], S. Mule[4], A. Luciani[1]; [1]Paris/FR, [2]Lille/FR, [3]Creteil/FR, [4]Charenton-Le-Pont/FR

**Purpose:** Evaluation of renal masses characterization and applicability of Bosniak 2019 criteria on a monophasic portal venous phase using rapid kilovoltage-switching DECT (rsDECT).
**Methods or Backround:** In this retrospective IRB-approved study, 127 renal masses assessed on rsDECT were included. Each lesion was classified according to Bosniak 2019 classification using MRI as the reference standard. Using the portal venous phase, virtual monochromatic images at 40, 50 and 77 keV, virtual unenhanced (VUE) and iodine map images were reconstructed. Changes in attenuation values between VUE and 40 keV, 50 keV and 77 keV measurements were computed and respectively defined as ∆HU40keV, ∆HU50keV and ∆HU77keV. The values of ∆HU40keV, ∆HU50keV and ∆HU77keV thresholds providing the optimal diagnostic performance for the detection of solid renal masses were determined using the F1 score.
**Results or Findings:** The population study included 25 solid renal masses (25/127, 20%) and 102 cystic renal masses (102/127, 80%). To differentiate solid to cystic masses, the specificity of the predefined 20HU threshold reached 88% (95%CI: 82, 93) using ∆HU77keV and 21% (95%CI: 15, 28) using ∆HU40keV. The estimated optimal threshold of attenuation change was 22HU on ∆HU77keV, 76HU on ∆HU50eV and 147HU on ∆HU40eV. The rsDECT classification was highly similar to that of MRI for solid renal masses (23/25, 92%) and for Bosniak 1 masses (62/66, 94%). However, two hyperattenuating Bosniak 2 renal masses (2/26, 8%) were classified as solid renal masses on rsDECT.
**Conclusion:** 2019 Bosniak criteria can be used for renal masses characterization on monophasic portal venous phase acquired on rsDECT but should be applied with caution. Known enhancement thresholds must be adapted according to the types of reconstructions used and especially to the energy level of virtual monochromatic reconstructions.
**Limitations:** Retrospective study with mainly low-grade masses.
**Ethics committee approval:** This study was approved by IRB-n°CRM-2204-245
**Funding for this study:** No limitations were identified.
**Author Disclosures:**
Alexandre Ingels: Nothing to disclose
Lorenzo Carlo Pescatori: Nothing to disclose
Benjamin Longere: Nothing to disclose
Mohamed Bouanane: Nothing to disclose
Alain Luciani: Nothing to disclose
Edouard Reizine: Nothing to disclose
Wafa Boughanmi: Nothing to disclose
Sebastien Mule: Nothing to disclose
Maxime Blain: Nothing to disclose

### RPS 607-8

### One-phase computed tomography is sufficient for detecting urothelial cell carcinoma in patients with gross hematuria

*K. F. Galtung*[1], P. M. Lauritzen[1], G. Sandbaek[1], D. Bay[1], E. Ponzi[1], E. Baco[1], N. C. Cowan[2], P. E. Rud[1]; [1]Oslo/NO, [2]Portsmouth/UK
*(kriflor@hotmail.com)*

**Purpose:** In case of gross hematuria (GH) and suspicion of urothelial cell carcinoma (UCC), cystoscopy and multiphase computed tomography (CT) are recommended. However, previous studies have questioned the need for multiphase CT. This study aimed to evaluate if one-phase CT is sufficient for detecting UCC in patients with GH.
**Methods or Backround:** This prospective paired diagnostic non-inferiority study included 308 consecutive patients with GH. All patients underwent four-phase CT before cystoscopy. Randomly, one radiologist was assigned to score nephrographic CT only (experimental), while a second radiologist was assigned to score four-phase CT (control). Each CT phase was scored with Likert 1-5. Likert ≥3 defined a positive CT. UCC verified in histological specimen defined a positive reference standard. Results were stratified upon anatomical location (bladder, ureter, or renal pelvis). We tested for non-inferiority of the experimental CT in terms of accuracy, using a 7.5% non-inferiority margin.
**Results or Findings:** UCC was detected in 14.6% (45) patients, of which 89% (40) were in the bladder, 7% (3) were in the ureter, and 4% (2) were in the renal pelvis. No patients had UCC in more than one location. The sensitivity of the control and experimental CT was 93.3% vs. 91.1%, specificity was 83.7% vs. 81.8%, NPV was 98.7% vs. 98.2% and PPV 49.4% vs. 46.1%. The accuracy was 85.0% vs. 83.1%, with a difference of 1.9 % (upper boundary of the 95% CI: 7.1%), hence non-inferiority was demonstrated.
**Conclusion:** The accuracy of nephrographic CT is non-inferior to four-phase CT for detecting UCC.

**Limitations:** Although the prevalence of UCC in the study population is representative of patients with GH, the number of UCC cases was relatively low.
**Ethics committee approval:** No information provided by the submitter.
**Funding for this study:** No limitations were identified.
**Author Disclosures:**
Gunnar Sandbaek: Nothing to disclose
Peter Mæhre Lauritzen: Nothing to disclose
Nigel Christopher Cowan: Nothing to disclose
Eduard Baco: Nothing to disclose
Erica Ponzi: Nothing to disclose
Kristina Flor Galtung: Nothing to disclose
Per Erik Rud: Nothing to disclose
Dag Bay: Nothing to disclose

**16:30-17:30** **Research Stage 3**

### Research Presentation Session: Musculoskeletal

### RPS 610

### Musculoskeletal: tumours

**Moderator**
W. Rennie; Leicester/UK
*(wjrennie@yahoo.com)*

### RPS 610-2

**Apparent diffusion coefficient (ADC) first-order radiomic features differentiate pelvic chordomas and chondrosarcomas**
L. Chenglei, *R. F. Valenzuela*, W. Green, M. Canjirathinkal, M. Griggs, W-L. Wang, W. Murphy, B. Amini; Houston, TX/US
*(raulvp@estigia.cl)*

**Purpose:** Although most chordomas arise from the midline and chondrosarcoma from the lateral pelvis, approximately 10% of chondrosarcomas occur near the midline, displaying a similar degree of T2 hyperintensity than chondroma, causing a diagnostic challenge.
**Methods or Backround:** We explore the diagnostic accuracy of first-order radiomic features of ADC maps and two novel diffusion indices for the differentiation of sacral chordomas and midline chondrosarcomas.
**Results or Findings:** There was good to excellent inter-observer reliability for 8 of the 10 ADC metrics on the training dataset. There was a significant difference ($p<0.005$) between chordomas and chondrosarcomas for RI, FI, median and mean ADC, and skewness using the training dataset. Optimal cut-points for diagnosis of chordoma were RI > 0.015; FI < 0.25; Mean ADC < 1.7 × 10-3 mm2/s; Skewness > 0.177. The optimal decision tree relied only on FI. Testing cut points on the validation dataset yielded the highest diagnostic accuracy for FI.
**Conclusion:** FI reliably differentiates chordomas (FI <0.25) from chondrosarcomas. Mean ADC provides a practical alternative to FI for routine clinical use.
**Limitations:** The sample size was limited, which was 40 for Chordomas and 42 for Chondrosarcomas.
**Ethics committee approval:** No information provided by the submitter.
**Funding for this study:** The John S. Dunn, Sr. Distinguished Chair in Diagnostic Imaging and M.R Evelyn Hudson Foundation Endowed Professorship have financially supported this study.
**Author Disclosures:**
Wei-Lien Wang: Nothing to disclose
Moreko Griggs: Nothing to disclose
Behrang Amini: Nothing to disclose
Liu Chenglei: Nothing to disclose
Raul Fernando Valenzuela: Nothing to disclose
William Murphy: Nothing to disclose
William Green: Nothing to disclose
Mathew Canjirathinkal: Nothing to disclose

### RPS 610-3

**Clinico-radiological characterisation of osteoid osteomas treated in a single institution and comparison of two treatment modalities – a retrospective study**
*M. Feichtinger*, M. Smolle, J. Steiner, E. Talakic, M. Pohl, H. R. Portugaller, I. Brcic, M. Fuchsjäger, J. Igrec; Graz/AT
*(mira.feichtinger@medunigraz.at)*

**Purpose:** Our aim is to evaluate the efficacy of CT-guided percutaneous radiofrequency ablation (RFA) and surgical treatment in osteoid osteoma (OO) treated at the Medical University of Graz.
**Methods or Backround:** We retrospectively analysed the clinical and radiological data of all consecutive patients treated in our institution with either histological or radiological diagnoses of OO from January 2005 to January 2021. CT and MRI examinations performed during the diagnostic process were analysed for the presence of typical findings.
**Results or Findings:** In total, 119 patients (mean age: 21.6 ± 10.9 years; 63.9% males) with clinically and radiologically confirmed OO were retrospectively evaluated. 73 patients underwent RFA and 43 surgery, respectively. In three cases, RFA combined with surgery was performed. Pre-intervention, 103 patients (88.8%) had undergone CT and 101 MRI (87.1%) examinations. A nidus was confirmed in 82.5% of cases with CTs (85/103) and 63.4% with MRIs (64/101). The majority of nidi was situated cortically (n=96; 82.8%), most frequently in the femur in 38 patients (33.3%) with a median size of 8.0 mm (IQR: 5.0 – 12.0 mm). Median symptom duration prior to treatment was 6.0 (IQR: 4.0 – 13.0) months. The overall complication rate was 11.2% (13/116) (13.7% RFA vs 7% surgery; p=0.268). In total, 11.2% of patients had persistent symptoms after 1 week with clinical success rates of RFA and surgery, 86.3% and 90.7% (p=0.647), respectively.
**Conclusion:** Compared to surgical treatment, CT-guided percutaneous RFA is a safe, minimally invasive, reliable, and efficient treatment option for OO.
**Limitations:** Histological confirmation of OO is not always possible with CT-guided percutaneous RFA.
**Ethics committee approval:** This study was approved by Medical University Graz.
**Funding for this study:** No funding was received for this study.
**Author Disclosures:**
Jakob Steiner: Nothing to disclose
Michael Fuchsjäger: Nothing to disclose
Iva Brcic: Nothing to disclose
Horst Rupert Portugaller: Nothing to disclose
Maximilian Pohl: Nothing to disclose
Maria Smolle: Nothing to disclose
Mira Feichtinger: Nothing to disclose
Emina Talakic: Nothing to disclose
Jasminka Igrec: Nothing to disclose

### RPS 610-4

**Assessment of validity of plain film and magnetic resonance imaging in the evaluation of bone tumours**
*B. Surakutty*[1], S. Salim[1], J. Verghese[2], H. Nair G S[1]; [1]Alappuzha/IN, [2]Vandanam/IN
*(beenamols@gmail.com)*

**Purpose:** To assess the sensitivity, specificity, positive predictive value and negative predictive value of plain film, and MRI findings in bone tumors using pathological findings as a reference standard. To define a scoring system in plain film, MRI, and in combination, to diagnose a bone tumor as benign or malignant.
**Methods or Backround:** A descriptive study with diagnostic test evaluation done in the Department of Radiodiagnosis, Govt. T.D. Medical College, Alappuzha. The study population was clinically suspected bone tumor patients referred for MRI evaluation. Plain film findings were evaluated to assess the margin of lesion, zone of transition, cortical break, soft tissue involvement, and periosteal reaction. MRI findings were evaluated to look for marrow, soft tissue, joint, neurovascular bundle, and cortical involvement. The obtained data from plain film and MRI findings were compared with pathological findings by statistical analysis.
**Results or Findings:** Plain film assessment of tumor margin is the single important factor in the detection of malignancy with the highest sensitivity of 94.7% and good specificity of 71.7%. The nature of the lesion can be determined as malignant using a plain radiographic score of more than or equal to 2 with a sensitivity of 89.5% and a specificity of 71.7%. MRI score and combined score yield a higher specificity of 82.6%. Positive likelihood ratio of combined score is better than plain radiographic score or MRI score taken alone.
**Conclusion:** Plain film and MRI can reasonably characterize a bone lesion as benign or malignant using a plain radiographic score, MRI score and a combined score.
**Limitations:** Many important variables in the detection of malignancy like the size of lesion, the presence of hemorrhage, or necrosis were not taken into consideration. All assessments in this study were subjective also. Hence inter-observer variability is likely.
**Ethics committee approval:** No information provided by the submitter.
**Funding for this study:** No funding was received for this study.
**Author Disclosures:**
Safi Salim: Nothing to disclose
Harikumaran Nair G S: Nothing to disclose
Josey Verghese: Nothing to disclose
Beenamol Surakutty: Nothing to disclose

### RPS 610-5
**MRI criteria for lipomas and well-differentiated or dedifferentiated liposarcomas of soft tissue: analysis and decision tree proposal**
*A. Filliole*, H. Basselerie, F. Lapegue, H. Chiavassa, P. Bonnevialle, A. Gomez-Mascard, N. Sans, M. Faruch; Toulouse/FR
*(filliole.a@chu-toulouse.fr)*

**Purpose:** To describe the MRI characteristics of adipose tumors and define criteria that can differentiate LS from lipomas. To propose a new decision tree for biopsies, aiming for 100% sensitivity for the diagnosis of LS, while reducing the number of biopsies of benign tumors.
**Methods or Backround:** This retrospective and observational study included all patients with lipoma or LS diagnosed at our hospital between 2010 and 2019 for whom MRI data was available. The final diagnosis was made based on the cytogenetic status of the MDM2 oncogene by FISH (or if missing, by immunohistochemistry). Two radiologists read the MRI images. Finally, a comparison was made between our decision tree and the decision trees found in literature.
**Results or Findings:** Our sample consisted of 252 lipomas and 50 LS. Significant criteria (p<.05) to differentiate LS from lipomas were the presence of thick septa (>2.00mm), non-fatty nodules, unclean fat, and peritumoral infiltration. For deep locations, other criteria were associated with LS: location in the limbs, size >7cm, number of septa ≥3, and unclean fat ≥50mm. A new decision tree for biopsies was developed, with a sensitivity of 100% and specificity of 49% for LS diagnosis. For superficial tumors, a biopsy should be done if MRI shows thick septa (>2.00mm), non-fatty nodules, or unclean fat. For deep tumors, a biopsy should be done if MRI shows a size >7cm, ≥3 thick septa (>2.00mm), non-fatty nodules, and unclean fat ≥50mm. The performance of decision trees from other studies was not as good on our sample.
**Conclusion:** We propose a new decision tree for biopsies of fatty tumors that helps to reduce the number of unnecessary biopsies without missing any LS.
**Limitations:** The limitation of this study were the retrospective perspective, the fact that the cytogenetic status of the MDM2 oncogene by FISH did not include all lesions. In addition, the study has not been yet confirmed on other sample.
**Ethics committee approval:** This study was approved by an institutional review board (CRM-2106-173).
**Funding for this study:** No funding was received for this study.
**Author Disclosures:**
Nicolas Sans: Nothing to disclose
Marie Faruch Marie Faruch: Nothing to disclose
Helene Chiavassa: Nothing to disclose
Anne Gomez-Mascard: Nothing to disclose
Paul Bonnevialle: Nothing to disclose
Franck Lapegue: Nothing to disclose
Hubert Basselerie: Nothing to disclose
Antoine Filliole: Nothing to disclose

### RPS 610-6
**Focusing on tumour configuration can restrict the use of contrast agent in primary soft tissue sarcoma: a multicentred study**
*M. Sedaghat*[1], M. Grözinger[2], O. Jansen[1], S. Sedaghat[1]; [1]Kiel/DE, [2]Heidelberg/DE
*(maya.stange92@googlemail.com)*

**Purpose:** Soft-tissue sarcomas (STS) comprise a heterogeneous group of malignancies, accounting for only about 1% of all cancers. In this study, we evaluated whether by focusing on tumour configuration, the use of contrast agents could be restricted.
**Methods or Backround:** 64 primary STS were included. Patients with lipo-, angio- and retroperitoneal sarcomas were excluded. In the first step, all primary STS for configuration and borders (infiltrative or well-defined) were evaluated on non-contrast MRI. Then, contrast-enhanced MRI images were added and directly compared to the non-contrast MRI images. Finally, the need for a contrast agent for detecting the primary tumour and the tumour borders was retrospectively evaluated.
**Results or Findings:** The main configurations of primary STS were multilobulated (54%) and ovoid/nodular (30%), followed by streaky (10%). 59%, 28%, and 13% of the tumours presented with a malignancy grade 3, 2, and 1 (FNCLCC), respectively. 57% of the tumours showed infiltrative borders. All multilobulated primary STS were clearly detectable without the use of a contrast agent (p<0.001). Additionally, the borders of most of the multilobulated tumours could be well evaluated without contrast agents (90%; p<0.001). In non-multilobulated STS, the contrast agent was needed in 41% and 58% of the cases to detect the primary tumours and to evaluate the tumour borders, respectively.
**Conclusion:** Our findings suggest that with a focus on the configuration of STS on MRI, the use of contrast agents could be significantly decreased, especially in multilobulated STS. This contributes to patients' safety and minimizes the possibility of side effects.
**Limitations:** The main limitation is the retrospective design. Additionally, not all STS subtypes such as lipo-, angio- and retroperitoneal sarcomas were investigated.
**Ethics committee approval:** No information provided by the submitter.
**Funding for this study:** No funding was received for this study.
**Author Disclosures:**
Sam Sedaghat: Nothing to disclose
Martin Grözinger: Nothing to disclose
Maya Sedaghat: Nothing to disclose
Olav Jansen: Nothing to disclose

**16:30-17:30** **Research Stage 4**

### Research Presentation Session: Cardiac

### RPS 603
### CT imaging for TAVI planning

**Moderator**
G. Muscogiuri; Milan/IT
*(g.muscogiuri@gmail.com)*

### RPS 603-2
**Contrast medium reduction for computed tomography before transaortic valve replacement planning considering virtual monoenergetic reconstructions: a prospective systematic approach**
*I. L. Langenbach*[1], H. Wienemann[2], K. Klein[2], C. P. Naehle[2], D. Maintz[2], K. B. Krug[2], B. Foldyna[1], M. C. Langenbach[1]; [1]Boston, MA/US, [2]Cologne/DE
*(il.langenbach@gmail.com)*

**Purpose:** This prospective trial systematically investigated the reduction of iodized contrast medium (CM) for pre-transaortic valve replacement (TAVR) planning computed tomography (CT) without compromising the image quality using dual-energy spectral-detector CT and low-kV virtual monoenergetic imaging (VMI) reconstructions.
**Methods or Backround:** All patients received a standardized CT protocol, including a contrast-enhanced cardiac, aortic, and iliac scan for TAVR planning. Fifteen patients were scanned with 60 ml CM as a gold standard. In groups of 15 patients, CM was reduced stepwise (50 ml, 40 ml, 30 ml). The image data was calculated using the standard reconstruction algorithm and low-KV VMI reconstructions with 40 and 60 keV. Signal-to-noise ratio (SNR) and contrast-to-noise ratio (CNR) in several vessel sections were compared.
**Results or Findings:** This study investigated sixty consecutive patients (28 women; 81±7.5 years) for pre-TAVR assessment. Analysis of SNR and CNR revealed a significant decrease in the 30 ml group for the standard reconstruction (p=0.004, p=0.002) and the VMI 40 keV reconstruction (p=0.001, p<0.001) in the ascending aorta and the iliac arteries compared to 60 ml CM with no difference for 50 ml and 40 ml CM. Within each contrast group, results showed no difference between the standard and VMI 40 and 60 keV reconstructions for the ascending aorta. Only the VMI 40 keV reconstruction for the pelvic circulation showed no significant difference (p=0.810) compared to the standard.
**Conclusion:** The iodized contrast medium of the pre-TAVR CT can be reduced to 40 ml without limitations on the image quality. The VMI 40 keV showed comparable results to the standard reconstruction for SNR and CNR. The reduction to 30 ml contrast medium showed a significant loss of quality and is not recommended for clinical practice.
**Limitations:** Small study population, singular scanner type.
**Ethics committee approval:** No information provided by the submitter.
**Funding for this study:** Not applicable.
**Author Disclosures:**
Isabel Luisa Langenbach: Nothing to disclose
David Maintz: Nothing to disclose
Konstantin Klein: Nothing to disclose
Borek Foldyna: Nothing to disclose
Claas Philip Naehle: Nothing to disclose
Marcel Christian Langenbach: Nothing to disclose
Kathrin Barbara Krug: Nothing to disclose
Hendrik Wienemann: Nothing to disclose

### RPS 603-3

**Diagnostic accuracy of ultra-high-resolution photon counting coronary CT angiography for the detection of significant coronary artery disease in a high-risk TAVI population**

*M. T. Hagar*, R. Saffar, M. Soschynski, J. Taron, T. Stein, C. von zur Mühlen, C. L. Schlett, F. Bamberg, T. Krauß; Freiburg im Breisgau/DE

**Purpose:** To determine the diagnostic accuracy of ultra-high-resolution CT (UHR-CT) for the detection of obstructive coronary artery disease (CAD) in high-risk patients referred for trans-arterial valve implantation (TAVI).
**Methods or Backround:** Consecutive patients with severe aortic stenosis and clinically indicated CT for TAVI planning were prospectively enrolled. All patients were examined on a first-generation dual-source photon counting scanner, and a calcium scan, followed by UHR cardiac CT and high-pitch CT aortoiliacography scan (100 kV, 140 kV, and 120 kV, respectively), were performed. Two blinded radiologists analyzed UHR-CT images for the presence of obstructive CAD (diameter stenosis ≥50%). The diagnostic performance of UHR-CT for detecting significant CAD was determined using invasive coronary angiography (ICA) as the reference standard.
**Results or Findings:** Among 34 included patients (mean age 80.9±6.9 years, 50% female), 26% had severe coronary calcification (Agatston-Score ≥1000), 38% had significant coronary artery stenosis, and 26% had prior stent placement (n=9, n=13, and n=9, respectively). Overall image quality was good to excellent (1.54±0.5); the mean contrast-to-noise ratio was 10.7±3.0. Sensitivity, specificity, and accuracy were 67%, 97% and 95% on a segment-based analysis (n=480) and 92%, 95% and 94% for patient-based analysis (n=34). In ROC analysis, the AUC of UHR-CT for detecting obstructive CAD was 0.891 per segment level (95% CI:0.806-0.976), 0.948 per vessel-level (95% CI:2.902-0.994) and 0.99 per patient-level(95% CI:0.966-1.014).
**Conclusion:** Ultra-high-resolution cardiac in high-risk patients provides excellent performance in detecting obstructive CAD, indicating a potentially useful indication in this target population.
**Limitations:** The relatively small sample size from a single centre requires further confirmatory research to improve generalizability.
**Ethics committee approval:** IRB: 09/21/2021, (No. 21-2469). Informed written consent was obtained.
**Funding for this study:** No funding was received for this study.
**Author Disclosures:**
Martin Soschynski: Nothing to disclose
Tobias Krauß: Nothing to disclose
Muhammad Taha Hagar: Nothing to disclose
Christopher L. Schlett: Research/Grant Support: Siemens Healthineers
Ruben Saffar: Nothing to disclose
Jana Taron: Consultant: Core Lab Black Forrest GmbH Speaker: Bayer AG Speaker: Siemens Healthineers
Fabian Bamberg: Research/Grant Support: Siemens Healthineers Research/Grant Support: Bayer Healthcare Consultant: Bayer Healthcare Speaker: Bayer Healthcare Speaker: Siemens Healthineers
Constantin von zur Mühlen: Nothing to disclose
Thomas Stein: Nothing to disclose

### RPS 603-4

**Clinical Value of CT-based Fractional Flow Reserve in Pre-Procedural TAVR Planning**

*S. S. Martin*, V. Koch, C. Booz, L. D. Gruenewald, E. Nagel, T. Vogl; Frankfurt/DE

**Purpose:** To examine the clinical feasibility and potential gate-keeper role of workstation-based fractional flow reserve (CT-FFR) in pre-procedural CT planning in patients undergoing transcatheter aortic valve replacement (TAVR) with concomitant coronary artery disease (CAD).
**Methods or Backround:** Overall, 112 patients undergoing TAVR were included using standard CT data procured during TAVR planning. The employed software uses intricate fluid dynamic operations with recent developments of machine-learning algorithms enabling rapid computation of CT-FFR across the lesions of interest in the same institute. CAD burden was additionally assessed via coronary calcium scores (CACS) and stenosis quantification. All patients were observed for major adverse cardiac events (MACE) during 12-month follow-up succeeding TAVR implantation.
**Results or Findings:** The pre-specified endpoint was met in 17 patients (15.2%), with cardiovascular death being the most prevalent of the MACE composite. Of these, 13 patients had a positive CT-FFR (p=0.002), eventuating in a relative risk ratio of 4.33 (p=0.006, 95%CI 1.5-12.5). Thirty-five out of 46 patients (76.1%) with one or more significant stenosis (≥70%) showed hemodynamic relevance, as implied by CT-FFR. Compared to conventional CAD risk markers, CT-FFR performed best in predicting adverse outcomes (odds ratios 5.7 vs. 1.6).
**Conclusion:** At 12 months, there was a significantly greater occurrence of MACE in patients deemed to have CAD via consideration of cardiac CT and CT-FFR findings. Workstation-based CT-FFR demonstrates signs of viability in a realistic clinical setting with the possibility to distinguish patients with a potential benefit of adjusted CAD treatment.
**Limitations:** This common comorbidity continues to challenge clinicians, particularly when considering the to date, unclear viability and strategic timing of coronary intervention in this cohort. Further limitations of the current study include the retrospective study design and the lack of catheter-based FFR values.
**Ethics committee approval:** No information provided by the submitter.
**Funding for this study:** Not applicable.
**Author Disclosures:**
Christian Booz: Nothing to disclose
Leon D. Gruenewald: Nothing to disclose
Eike Nagel: Nothing to disclose
Thomas Vogl: Nothing to disclose
Vitali Koch: Nothing to disclose
Simon S. Martin: Nothing to disclose

### RPS 603-5

**Low-dose and Low-contrast Media Volume CT Angiography for Transcatheter Aortic Valve Replacement Planning as a Comprehensive Tool in the Assessment of Coronary Arteries Disease**

*C. Capodaglio*[1], M. Ragusi[1], C. Maino[1], C. Talei Franzesi[1], D. Ippolito[1], S. Sironi[2]; [1]Monza/IT, [2]Bergamo/IT
*(c.capodaglio@campus.unimib.it)*

**Purpose:** To evaluate the diagnostic performance of low radiation dose and low contrast media CT-angiography (CTA) protocol reconstructed with model-based iterative reconstruction (MBIR) algorithm in the assessment of coronary arteries during TAVI- planning.
**Methods or Backround:** One hundred fifteen patients from February 2017 to March 2021 who underwent contrast-enhanced CT angiography (CTA) prior to TAVI were retrospectively enrolled. A 256-MDCT scanner, 80 kVp, 50 ml of contrast media and retrospective ECG-gating were used. Images were reconstructed with a Model-Based Iterative algorithm (MBIR-IMR Level1). Objective assessment was performed measuring contrast enhancement expressed as mean Hounsfield Units (HU) and standard deviation (SD) as an indicator for image noise at subclavian, iliac, aortic and coronary arterial levels. Signal-to-noise and contrast-to-noise ratios were calculated. Subjective image quality was assessed for each coronary vessel using a 4-point Likert scale.
**Results or Findings:** The mean attenuation value in the thoracic aorta was 478 HU, and 447 HU in the abdominal aorta. Evaluation of the proximal and intermediate tract of the left descending coronary left circumflex and of the right coronary arteries was possible in all but one patient. The median grading for the right coronary artery was 3 and 2 for the proximal and intermediate segments, respectively. Median values for the proximal segment of the common trunk were 4. Median values attributed to the left anterior descending artery were 3 in proximal and median segments and 3 in proximal and median segments of the circumflex artery. The qualitative analysis did not show significant differences (p-Values <0.05) between patients stratified according to heart rate (HR), above or below 70 bpm.
**Conclusion:** CTA reconstructed with the MBIR algorithm is a valid tool for coronary tree evaluation and the assessment of coronary artery disease (CAD) during the diagnostic workup of TAVI candidates.
**Limitations:** Not applicable.
**Ethics committee approval:** No information provided by the submitter.
**Funding for this study:** Not applicable.
**Author Disclosures:**
Sandro Sironi: Nothing to disclose
Cesare Maino: Nothing to disclose
Carlo Capodaglio: Nothing to disclose
Maria Ragusi: Nothing to disclose
Davide Ippolito: Nothing to disclose
Cammillo Talei Franzesi: Nothing to disclose

### RPS 603-6

**Relevant pre-TAVI CT imaging parameters for patients requiring permanent pacemaker implantation**

*D. Puhr-Westerheide*, E. K. Gresser, C. Utz, F. Grathwohl, M. Onkes, K. Rizas, M. Ingrisch, A. T. Stüber, A. Curta; Munich/DE

**Purpose:** To evaluate differences in pre-transfemoral aortic valve implantation (TAVI) CTs for post-procedural conduction disturbances requiring permanent pacemaker implantation (PPI) compared to a no-PPI group.
**Methods or Backround:** Aortic valve stenosis leads to high morbidity and mortality. TAVI is a technique for minimally-invasive valve replacement. Post-interventional conduction disturbances requiring PPI are a common complication after TAVI in up to 10-15% of patients. This retrospective study includes 312 patients with conduction disturbances and PPI after TAVI compared to 305 age-matched TAVI patients without PPI who received pre-procedural CT and TAVI (Sapien 3 valve) between 2014 and 2019 at our institution.
**Results or Findings:** Valve and quantitative CT-imaging parameters were recorded. Univariate statistics (Chi2, Mann-Whitney-U) with correction for multiple testing (Benjamin-Hochberg) showed significant differences between

PPI- and no-PPI-group for max aortic annulus (AoA) diameter (p<0.001), min AoA diameter (p=0.012), AoA perimeter (p<0.001), length of sinotubular junction (p=0.0014), sinus of Valsalva diameter (p<0.001), left coronary cusp calcification (LCC Ca, p=0.009) and right coronary cusp calcification (RCC Ca, p=0.008). Five interaction terms of imaging and valve parameters were analyzed using a logit model to test for differences between groups. In this model, no significant differences were found for interactions between prosthesis size and aortic annulus (AoA) perimeter, the distance of annulus to the left coronary artery, the distance of annulus to the right coronary artery and AoA minimum and maximum diameter. Interaction of the length of the left ventricular outflow tract (LVOT) with prosthesis size showed significant differences (p=0.015).
**Conclusion:** We found significant differences in the PPI vs. no-PPI-group for a variety of imaging variables, including an interaction term of LVOT length with prosthesis size, which should be considered for multivariate risk stratification models for PPI prediction combined with clinical parameters.
**Limitations:** Retrospective design, age-matched comparison group.
**Ethics committee approval:** No information provided by the submitter.
**Funding for this study:** No funding was received for this study.
**Author Disclosures:**
Meike Onkes: Nothing to disclose
Konstantinos Rizas: Nothing to disclose
Daniel Puhr-Westerheide: Nothing to disclose
Michael Ingrisch: Nothing to disclose
Adrian Curta: Nothing to disclose
Eva Kristina Gresser: Nothing to disclose
Anna Theresa Stüber: Nothing to disclose
Christina Utz: Nothing to disclose
Fabian Grathwohl: Nothing to disclose

### RPS 603-7

**Clinical and imaging parameters associated with post-TAVI permanent pacemaker implantation in a multivariate prediction model**

*D. Puhr-Westerheide*, E. K. Gresser, C. Utz, F. Grathwohl, M. Onkes, K. Rizas, M. Ingrisch, A. T. Stüber, A. Curta; Munich/DE

**Purpose:** To evaluate the prognostic value of clinical and pre-Transfemoral Aortic Valve Implantation (TAVI) CT-imaging parameters for post-procedural conduction disturbances requiring permanent pacemaker implantation (PPI).
**Methods or Backround:** TAVI is a technique for minimally-invasive valve replacement in aortic valve stenosis, a potentially life-threatening condition. Common complications are post-interventional conduction disturbances requiring PPI in 10-15% of patients. This retrospective study includes 617 patients who received TAVI (Sapien 3 valve) and preprocedural CT at our institution. Three hundred twelve patients requiring PPI were age-matched with 305 non-PPI patients from our database. A scaled model with LASSO was used for variable selection, including clinical and imaging parameters, and to determine significant factors associated with PPI post-TAVI.
**Results or Findings:** The final scaled LASSO model included the following nine variables for PPI prediction: the presence of a right bundle branch block (RBBB), atrial fibrillation (AF), 1st-degree AV-block (AVB-I), coronary artery disease, arterial hypertension, prosthesis size, left coronary cusp calcification (LCCC) and the angle between left ventricle axis and the aortic root. The ratio of valve size to aortic annulus diameter did not show relevant predictive potential and was, therefore, not included in the final model. The strongest association was found for RBBB (OR 2.74), AVB-I (OR 2.09) and prosthesis size (OR 1.35) and outperformed imaging parameters for PPI prediction. The imaging parameter with the highest predictive value was the angle between the left ventricle axis and the aortic root (OR 1.03).
**Conclusion:** We found RBBB, AVB-I and prosthesis size as important predictive parameters for post-TAVI PPI requirements. Further, imaging parameters such as the angle between the left ventricle and the aortic root showed a predictive potential for PPI and should be further evaluated in multifactorial models for risk stratification.
**Limitations:** Retrospective design, age-matched comparison group.
**Ethics committee approval:** No information provided by the submitter.
**Funding for this study:** No funding was received for this study.
**Author Disclosures:**
Meike Onkes: Nothing to disclose
Konstantinos Rizas: Nothing to disclose
Daniel Puhr-Westerheide: Nothing to disclose
Michael Ingrisch: Nothing to disclose
Adrian Curta: Nothing to disclose
Eva Kristina Gresser: Nothing to disclose
Anna Theresa Stüber: Nothing to disclose
Christina Utz: Nothing to disclose
Fabian Grathwohl: Nothing to disclose

### RPS 603-8

**Extracellular volume fraction (ECV) derived from preoperative computed tomography predicts prognosis in patients undergoing transcatheter aortic valve implantation (TAVI).**

*D. Vignale*, C. Gnasso, A. Palmisano, S. Barbieri, D. Margonato, M. Montorfano, A. Esposito; Milan/IT
*(vignale.davide@hsr.it)*

**Purpose:** Transcatheter aortic valve implantation (TAVI) is the treatment of choice for high-risk patients with severe aortic stenosis (AS). A significant portion of TAVI recipients has no long-term clinical benefit, and myocardial fibrosis may contribute to unfavourable outcomes. We aimed to assess the prognostic value of an interstitial fibrosis marker, extracellular volume fraction (ECV), measured at CT performed for TAVI planning.
**Methods or Backround:** From October 2020 to July 2021, 113 consecutive patients (82 [79-85] years, 52% female) undergoing CT for TAVI planning were prospectively enrolled. ECV was calculated as the ratio of myocardium and blood pool differential attenuations before and 5 minutes after contrast administration, pondered for hematocrit. Follow-up was collected using electronic medical records or telehealth, or in-person visits. Cox proportional hazards model was used to assess the association between ECV and a composite end-point of heart failure hospitalization (HFH) and death.
**Results or Findings:** Median follow-up was 13 [11-15] months, during which 23/113 (20%) patients experienced death or HFH. These patients had lower aortic valve mean pressure gradient (39 [29-48] vs 46 [40-54] mmHg, p=0.002) and LV and RF ejection fraction (51 [37-69] vs 66 [54-74]%, p=0.014; 45 [31-53] vs 49 [44-55]%, p=0.010), and higher ECV (31.5 [26.9-34.3] vs 27.8 [25.3-30.2]%, p=0.006). At multivariable Cox analysis, ECV higher than 31.3% was associated with increased risk of death or HFH at follow-up (HR=5.92, 95% CI 2.37-14.75, p<0.001), together with reduced gradient (HR=0.95, 95% CI 0.92-0.98,p<0.001) and reduced AV area at ultrasound (HR=0.55, 95% CI 0.35-0.88, p=0.013).
**Conclusion:** In this prospective observational cohort of high-risk AS patients undergoing TAVI, ECV measured at planning CT was associated with increased risk of death or HFH at follow-up.
**Limitations:** Relatively small sample size. Standard of reference for ECV (CMR) not available.
**Ethics committee approval:** CTMyoC 112/INT/2019
**Funding for this study:** No funding was received for this study.
**Author Disclosures:**
Davide Margonato: Nothing to disclose
Davide Vignale: Nothing to disclose
Antonio Esposito: Nothing to disclose
Anna Palmisano: Nothing to disclose
Simone Barbieri: Nothing to disclose
Matteo Montorfano: Nothing to disclose
Chiara Gnasso: Nothing to disclose

### Thursday, March 2

**08:00-09:00** **Research Stage 1**

### Research Presentation Session: Artificial Intelligence & Machine Learning & Imaging Informatics

### RPS 705

### AI in trauma and ortho X-ray

**Moderator**
M. Klontzas; Iraklion/GR

### RPS 705-2

**A prospective approach to integrating AI fracture detection in radiographs into clinical workflow**
*S. Lüken*, J. Oppenheimer, B. Hamm, S. Niehues; Berlin/DE

**Purpose:** Most AI tools for radiologists are retrospectively tested, and integration into clinical workflow remains unclear. Gleamer BoneView is a commercially available AI algorithm for fracture detection in radiographs. We aim to test if the algorithm can assist in better sensitivity and specificity for fracture detection by residents with prospective integration into clinical workflow.
**Methods or Backround:** Radiographs with an inquiry for fracture reviewed by two residents were randomly assigned and included. A preliminary fracture diagnosis was made in a "pure human" report. After that, the AI decision on presence and location of fractures was shown, and changes to diagnosis could be made, resulting in an assisted report. The final diagnosis of fracture was made by a board-certified radiologist with over 8 years of experience, or if available, cross-sectional imaging. The sensitivity and specificity of the pure human report, AI diagnosis, and assisted report were calculated in comparison to the final expert diagnosis.
**Results or Findings:** 1163 exams in 735 patients were included, with a total of 367 fractures (31.56%). Pure human sensitivity was 84.74%, and AI sensitivity was 86.92%. 35 changes were made after showing AI results, 33 of which resulted in the correct diagnosis, resulting in 25 additionally found fractures. This resulted in a sensitivity of 91.28% for the assisted report. Specificity was 97.11, 84.67, and 97.36%, respectively.
**Conclusion:** AI assistance showed an increase in sensitivity for both residents without a loss of specificity. Stand-alone AI performance showed slightly superior sensitivity to the residents, albeit with lower specificity. The addition of AI assistance for residents resulted in a correction of 35 reports (2.4%), with only two false corrections due to the identification of a false-positive fracture by the AI.
**Limitations:** The review in this study was performed by only two residents.
**Ethics committee approval:** No information provided by the submitter.
**Funding for this study:** No funding was received for this study.
**Author Disclosures:**
Sophia Lüken: Nothing to disclose
Bernd Hamm: Nothing to disclose
Stefan Niehues: Nothing to disclose
Jonas Oppenheimer: Nothing to disclose

### RPS 705-3

**How can a deep learning algorithm for fracture detection accelerate patient management at the radiology department and the emergency room?**
*S. A. Sintzoff*, O. Segall, M. Sahin; Geneva/CH

**Purpose:** For the past year, we have integrated an artificial intelligence software (AIS) for the first reading of x-rays of patients admitted for trauma in the emergency room into our daily routine. We wanted to evaluate the benefits of this solution in terms of time management of patients, based on the delay in performing an additional CT motivated by the x-rays findings.
**Methods or Backround:** We performed a retrospective study of our data from July to September 2021, after the integration of the AIS (Rayvolve by Azmed), compared with the same period in 2020 for trauma x-ray cases that were followed up with an additional CT motivated by the x-rays findings and evaluating the delay between the procedures. The studied population included 582 patients in 2021, with x-rays processed by the AIS, and 525 patients in 2020, without this processing.
**Results or Findings:** In 2020, 73 out of the 525 patients required a CT-CBCT with an average delay of 1.50 days - 0 to 16 days, including 42 (57.5%) on day 0. In 2021, after the introduction of the AIS, 81 CT-CBCT were performed for the 582 patients with an average delay of 0.42 days - 0 to 5 days, including 68 (84%) on day 0. After the introduction of the AIS, the average time to complete the CT was reduced by 72%.
**Conclusion:** The study shows that this AI solution accelerates patient management at the radiology department and the emergency room, potentially leading to shorter treatment times and earlier recovery. Therefore, we believe that our results provide new elements supporting the idea that the use of AIS in traumatology will become a quality service standard in the future.
**Limitations:** Retrospective study.
**Ethics committee approval:** No information provided by the submitter.
**Funding for this study:** No funding was received for this study.
**Author Disclosures:**
Olivier Segall: Nothing to disclose
Serge A. Sintzoff: Nothing to disclose
Mehmet Sahin: Nothing to disclose

### RPS 705-4

**AI detection of fractures in radiographs in blast and shot injuries: helpful or hurtful?**
*J. Oppenheimer*, S. Lüken, B. Hamm, S. Niehues; Berlin/DE
*(jonas.oppenheimer@charite.de)*

**Purpose:** Patients with blast and shot injuries may present with severe trauma as well as smaller injuries that may then be missed. We propose to retrospectively test a commercially available AI software for fracture and dislocation detection in radiographs, Gleamer BoneView, on images of severe trauma patients. AI may help in the detection of smaller injuries or be hindered by the strong changes in anatomy.
**Methods or Backround:** A full-text search of radiographs performed in three maximum care centres was used to identify patients with either initial presentation due to shot or blast injury or in different stages of recovery from such an injury. Images were selected if severe trauma was shown. These images were then analyzed by the AI software. It was noted if the major and minor injuries (if present) were identified by the AI, as well as if any additional injuries not previously reported were identified.
**Results or Findings:** 62 patients were included, 35 (56.5%) with an acute injury, 27 in recovery. In 44 (71.0%) cases, the AI software correctly identified the major injury, in 37 (59.7%), it identified all minor injuries, in 8 (12.9%), it identified some of the minor injuries, and in 3 (4.8%) it identified fractures not previously reported. 3 (4.8%) cases were incorrectly not accepted by the AI software as being images of an unsupported anatomical region.
**Conclusion:** AI fracture detection in severe trauma was able to identify the major injuries in only 71% of cases; however, it found additional fractures in 3 cases. AI is often trained on standard images, which may not always be available in severe trauma settings or areas with less advanced medical training and equipment.
**Limitations:** This study represents a limited set of retrospectively selected cases.
**Ethics committee approval:** No information was provided by the submitter.
**Funding for this study:** No funding was received for this study.
**Author Disclosures:**
Sophia Lüken: Nothing to disclose
Bernd Hamm: Nothing to disclose
Stefan Niehues: Nothing to disclose
Jonas Oppenheimer: Nothing to disclose

### RPS 705-5

**Can artificial intelligence discriminate between recent and healing fractures?**
*A. Vanzulli*, A. Rizzo, L. A. Carbonaro, A. Vanzulli; Milan/IT
*(andrea.vanzulli@unimi.it)*

**Purpose:** The purpose of the study was to evaluate the ability of an artificial intelligence (AI) software to discriminate between recent and healing fractures.
**Methods or Backround:** We selected a cohort of patients who had received a diagnosis of bone fracture(s) at our emergency department and at least one follow-up x-ray at our hospital. We fed the images to the AI software for fracture identification BoneView by GLEAMER. An expert radiologist, blind to both the original report and timing of each image, was then asked to independently label each fracture as either "recent" or "healing". Identification rates of fractures by AI and the radiologist were stratified and compared at different timepoints.
**Results or Findings:** We evaluated 56 patients who received a diagnosis of bone fracture(s) for a total of 144 studies (56 diagnoses plus 88 follow-up x-rays) and 223 fracture sites. "Recent" fracture rates identified by the radiologist were 84%, 70%, 32%, 10%, and 0% at 0, 1-14, 15-28, 29-56, >56 days, respectively, with no significant difference between 0 and 1-14 days timepoints ($P = 0.116$) and statistically significant difference between 1-14 and 15-28, 29-56, >56 days ($P \leq 0.011$). Fracture rates identified by AI were 73%, 74%, 68%, 63%, and 51% at 0, 1-14, 15-28, 29-56, >56 days, respectively, with no statistically significant difference over time ($P \geq 0.072$). AI vs radiologist rates showed no significant differences at 1-14 days ($P = 1$), yet radiologist's rates were significantly lower at > 14 days timepoints ($P \leq 0.004$).
**Conclusion:** AI proved unreliable in discriminating recent and healing fractures. The radiologist is still needed to make such a distinction, which has

Thursday important implications both in terms of clinical and surgical planning and in forensic contexts.
**Limitations:** We challenged AI to perform a task it was not specifically trained for.
**Ethics committee approval:** No information provided by the submitter.
**Funding for this study:** No funding was received for this study.
**Author Disclosures:**
Luca Alessandro Carbonaro: Nothing to disclose
Angelo Vanzulli: Nothing to disclose
Aldo Rizzo: Nothing to disclose
Andrea Vanzulli: Nothing to disclose

### RPS 705-6

**Automated hip measurements using an artificial intelligence-based software**

*L. Lassalle*[1], N-E. Regnard[1], A. Tran[2], J. Ventre[2], V. Marty[2], L. Clovis[2], Z. Zhang[2], A. Guermazi[2], J-D. Laredo[2]; [1]Lieusaint/FR, [2]Paris/FR

**Purpose:** To assess the diagnostic performances of an artificial intelligence (AI)-based software to perform automatic anatomic measurements on anteroposterior and lateral hip radiographs.
**Methods or Backround:** We retrospectively collected 117 consecutive anteroposterior hip radiographs and 110 lateral hip radiographs from 3 imaging institutions. Two senior musculoskeletal radiologists independently annotated key points to calculate the femoral-neck-shaft (CC'D), the lateral-centre-edge (LCE), and acetabular roof angles, the pelvic obliquity on anteroposterior hip radiographs and the vertical-centre-anterior (VCA) angle on lateral hip radiographs. The gold standard was defined as the mean of their two measurements. Statistical analysis consisted of mean absolute error (MAE), bias assessed with Bland-Altman analysis between the gold standard and the AI prediction and intraclass coefficient (ICC) between the two manual ratings.
**Results or Findings:** 90 anteroposterior hip radiographs were included, and 27 radiographs were excluded. 83 lateral hip radiographs were included, and 27 were excluded. MAE for the CC'D angle, LCE angle, acetabular roof angle, and pelvic obliquity on anteroposterior hip radiographs were respectively 2.6° (95% CI: [2.3; 2.9], bias=-1.27°, ICC=0.8), 2.5° (95% CI: [2.2; 2.8], bias=1.21°, ICC=0.7), 1.9° (95% CI: [1.6; 2.1], bias=1.13°, ICC=0.8), 0.5mm (95% CI: [0.4; 0.6], bias=0.13mm, ICC=0.99). MAE for the VCA angle on the lateral hip radiograph was 3.7° (95% CI: [2.5; 4.8], bias=1.61°, ICC=0.89). Bias and MAE between the gold standard and the AI prediction were low across all measurements. ICC was good across all measurements and excellent for pelvic obliquity.
**Conclusion:** AI allows accurate and automatic anatomic measurements on anteroposterior and lateral hip radiographs.
**Limitations:** The study is retrospective, with a small number of radiographs and no comparison to an independent manual rating.
**Ethics committee approval:** No information was provided by the submitter.
**Funding for this study:** Funding was received from Gleamer.
**Author Disclosures:**
Zekun Zhang: Employee: Gleamer
Vincent Marty: Employee: Gleamer
Nor-Eddine Regnard: Founder: Gleamer
Lauryane Clovis: Employee: Gleamer
Louis Lassalle: Consultant: Gleamer
Jean-Denis Laredo: Employee: Gleamer
Jeanne Ventre: Employee: Gleamer
Ali Guermazi: Nothing to disclose
Alexia Tran: Nothing to disclose

### RPS 705-7

**Automated full-leg measurements using an artificial intelligence-based software**

*L. Lassalle*[1], N-E. Regnard[1], J. Ventre[2], V. Marty[2], L. Clovis[2], Z. Zhang[2], N. Nitche[2], A. Guermazi[3], J-D. Laredo[2]; [1]Lieusaint/FR, [2]Paris/FR, [3]West Roxbury, MA/US

**Purpose:** The study aimed to assess the diagnostic performances of an artificial intelligence (AI)-based software to perform automatic measurements on full-leg radiographs.
**Methods or Backround:** We retrospectively collected 393 consecutive full-leg radiographs, including 50 EOS radiographs and 343 conventional radiographs from 4 imaging institutions. Two senior musculoskeletal radiologists independently annotated key points to measure the Hip-Knee-Ankle (HKA) angle, femur, tibial, full-leg lengths, and pelvic obliquity. The gold standard was defined as the mean of their two measurements. Statistical analysis consisted of mean absolute error (MAE), bias assessed with Bland-Altman analysis between the gold standard and the AI prediction and intraclass coefficient (ICC) between the two manual ratings.
**Results or Findings:** 153 full-leg conventional radiographs and 23 full-leg EOS radiographs were included, 217 radiographs were excluded. MAE for the HKA angle, femur, tibial, and full-leg lengths, and pelvic obliquity were respectively 0.33° (95% CI: [0.29; 0.37], bias=0.28mm, ICC=0.99), 1.5mm (95% CI: [1.2; 1.9], bias=1.31mm, ICC=0.99), 1.5mm (95% CI: [1.2; 1.8], bias=0.33mm, ICC=0.99), 1.7mm (95% CI: [1.3; 2.1], bias=1.48mm, ICC=0.99), 0.7mm (95% CI: [0.6; 0.9], bias=-0.01mm, ICC=0.99). Bias and MAE between the gold standard and the AI prediction were low across all measurements. ICC between the two manual ratings was excellent across all measurements.
**Conclusion:** AI allows accurate and automatic anatomic measurements on full-leg conventional and EOS radiographs.
**Limitations:** The study is retrospective, with a small number of radiographs and no comparison to an independent manual rating.
**Ethics committee approval:** No information was provided by the submitter.
**Funding for this study:** The study was funded by Gleamer.
**Author Disclosures:**
Zekun Zhang: Employee: Gleamer
Vincent Marty: Employee: Gleamer
Nor-Eddine Regnard: Founder: Gleamer
Lauryane Clovis: Employee: Gleamer
Louis Lassalle: Consultant: Gleamer
Jean-Denis Laredo: Employee: Gleamer
Jeanne Ventre: Employee: Gleamer
Ali Guermazi: Nothing to disclose
Nicolas Nitche: Employee: Gleamer

### RPS 705-8

**Diagnosing adult hip dysplasia on radiographs using a deep learning model**

*J. Jensen*[1], O. Graumann[1], S. Overgaard[2], O. Gerke[1], M. J. Lundemann[2], M. H. Haubro[1], C. Varnum[3], J. Rasmussen[4], B. Rasmussen[1]; [1]Odense/DK, [2]Copenhagen/DK, [3]Vejle/DK, [4]Svendborg/DK
*(Janni.Jensen@rsyd.dk)*

**Purpose:** Hip dysplasia (HD) is a leading cause of hip pain in young patients and may lead to osteoarthritis. Initial diagnosis is typically based on radiographic measurements such as lateral centre edge angle (LCEA) and acetabular index angle (AIA). However, measurement variability between readers has previously been reported. Objectives were to assess the reliability of an algorithm designed to read pelvic radiographs and estimate the agreement between the algorithm and human readers for measuring LCEA and AIA.
**Methods or Backround:** 78 weight-bearing pelvic radiographs were retrospectively collected. Two radiologists, two orthopaedics and one radiographer measured LCEA and AA. The agreement between the algorithm and readers was estimated by Bland-Altman Limits of Agreement (BA LoA).
**Results or Findings:** The algorithm offered consistent measurement outputs. The agreement between readers and the algorithm was subject to variance. Bias (95% confidence-interval) and [BA LoA] for right LCEA ranged from 0.37° (-0.61 to 1.36) [-7.79 to 8.53] for the senior orthopaedic and to 9.56° (8.14 to 10.97) [-2.16 to 21.27] for the senior radiologist. Bias and LoA for right AIA ranged from -0.58° (-1.32 to 0.16) [-6.69 to 5.5] to 1.70° (0.88 to 2.53) [-5.11 to 8.53] for the junior radiologist and the senior orthopaedic respectively. The systematic discrepancy was evident for LCEA, where human readers reported higher values than the algorithm. Mean measured right LCEA for human readers ranged from 25.8 to 35.0° vs 25.4° as measured by the algorithm.
**Conclusion:** The agreement between the algorithm and readers was subject to variance. The algorithm was consistent and displayed the highest agreement with the senior orthopaedic. Human readers appeared to measure higher LCEA angles than the algorithm.
**Limitations:** The algorithm was consistent, but accuracy remains to be established. The variation between human readers and the algorithm needs to be explored further.
**Ethics committee approval:** Approved by the Danish National Committee on Health Research Ethics (Project-ID: 2103745).
**Funding for this study:** EIT Health Digital Sandbox Programme 2020 (DS20-12449).
**Author Disclosures:**
Benjamin Rasmussen: Nothing to disclose
Søren Overgaard: Nothing to disclose
Janni Jensen: Nothing to disclose
Michael Juncker Lundemann: Other: Mr Lundemann is Director of Clinical Operations Radiobotics. ML had no access to study data and was not involved in data analysis.
Martin Haagen Haubro: Nothing to disclose
Ole Graumann: Nothing to disclose
Oke Gerke: Nothing to disclose
Claus Varnum: Nothing to disclose
Janne Rasmussen: Nothing to disclose

**RPS 705-9**

**AI-enabled multi-institutional audit for the prevalence of missed spondylolisthesis on spine x-rays**

*V. K. Venugopal*, V. Mahajan, H. Mahajan, B. Bahl, S. Gupta, S. Pundir, R. Takhar; New Delhi/IN
*(vasanthdrv@gmail.com)*

**Purpose:** The purpose of the study was to perform a multi-institutional audit for the prevalence of missed spondylolisthesis and vertebral fractures on spine x-rays using a deep learning-based AI solution.
**Methods or Backround:** The retrospective study was performed at four different institutions on spine x-rays. From the database of clinical reports, 1000 consecutive spine x-rays performed between June 2016 and November 2020 without spondylolisthesis and/or fractures were identified. These x-rays were then analysed using a deep learning-based spine x-ray classification solution that identifies six types of abnormalities. The x-rays flagged by this solution as having fractures and/or spondylolisthesis were then reviewed by a neuroradiologist with ten years of experience specifically for the presence of these findings.
**Results or Findings:** Among the 1000 x-rays, the regional distribution was as follows: cervical, dorsal, lumbar, and whole spine - 215, 62, 575 and 148. There was a total of 63 cases with missed spondylolisthesis flagged by the AI, out of which 58 were agreed by the reviewer. Among these cases, there were 39,8 and 3 cases with grade 1, 2 and 3 anterolisthesis, respectively. There were 8 cases of retrolisthesis that were correctly identified by the AI solution.
**Conclusion:** Using AI, we were able to identify missed spondylolisthesis in approximately 5% of spine x-rays across four different institutions. Hence, AI-based tools can be very useful and efficient tools for quality audits of spine x-rays. We demonstrated the clinical evidence for that in this study.
**Limitations:** The number of studies was not very high for a definitive conclusion.
**Ethics committee approval:** No information provided by the submitter.
**Funding for this study:** No funding was received for this study.
**Author Disclosures:**
Salil Gupta: Employee: CARPL.ai
Shivam Pundir: Employee: CARPL.ai
Bhanushree Bahl: Employee: CARPL.ai
Rohit Takhar: Employee: CARPL.ai
Harsh Mahajan: Other: Research Collaboration with Lunit.ai, Predible Health, General Electric, Oxipit.ai, Koninklijke Philips NV research, Qure.ai. Other: Director at Mahajan Imaging Other: Research Collaboration with Quibim, Subtle Medical.
Vasantha Kumar Venugopal: Other: Research Collaboration with Subtle Medical, Qure.ai. Consultant: CARPL.ai Other: Research Collaboration with General Electric, Quibim, Oxipit.ai, Predible Health, Lunit.ai, Koninklijke Philips NV research.
Vidur Mahajan: Other: Research Collaboration with Oxipit.ai, Quibim, Qure.ai, Subtle Medical, Predible Health, Koninklijke Philips NV research. CEO: CARPL.ai Other: Research Collaboration with General Electric, Lunit.ai

**08:00-09:00** **Research Stage 2**

### Research Presentation Session: Breast

### RPS 702

### Advanced applications in breast imaging

**Moderator**
P. Clauser; Vienna/AT
*(clauser.p@hotmail.it)*
**Author Disclosures:**
Paola Clauser: Speaker: Siemens Healthineers

**RPS 702-2**

**Microwave breast imaging via MammoWave: assessment of young women (under 45) through a prospective study in a reference hospital**

D. Álvarez Sánchez-Bayuela[1], L. M. Cruz Hernandez[1], P. M. Aguilar Angulo[1], *R. Giovanetti González*[1], M. D. P. Sánchez Camacho González Carrato[1], A. Rodríguez Sánchez[1], L. Sani[2], G. Tiberi[2], C. Romero Castellano[1]; [1]Toledo/ES, [2]Perugia/IT

**Purpose:** The study aimed to assess the capability of microwave imaging in breast lesions detection in women below screening age in the framework of an international clinical study (ClinicalTrials.gov Identifier: NCT04253366).
**Methods or Backround:** MammoWave was introduced into the clinical routine of a reference hospital as a complementary technique for young women (below regular screening age: 45 years old). When coming to medical appointments or checking, they were offered to participate in the prospective study. Conventional imaging exams for each volunteer were considered the gold standard for this investigation. BI-RADS assessment was used to classify breasts with no radiological findings (NF) and breasts with radiological findings (WF) that could be either benign or malignant. MammoWave examination was performed with the patient lying down on an examination table in a comfortable position without breast compression. The MBI system used an automatic algorithm to classify scanned breasts based on different images' features (analysed with respect to threshold references calculated from a subset of NF breasts). Finally, sensitivity and specificity were evaluated vs the gold standard (statistical significance was set at $p<0.05$).
**Results or Findings:** MammoWave's performance was evaluated on 78 breasts from 49 young volunteers (38 ± 6.3 years old) with different breast densities (41 dense breasts ACR C and D) and radiological findings. Sensitivity, specificity, and accuracy of 80.4%, 52.4%, and 72.7% were achieved, respectively, in breast lesion detection. Additionally, MammoWave detected 87.5% of malignant lesions and 79.2% of benign findings.
**Conclusion:** New technologies such as MBI are promising to get safe, accurate, comfortable, and non-density limited screening, which may include younger women.
**Limitations:** We did not consider volunteers' pre-menstrual information.
**Ethics committee approval:** This study involved human participants and was approved by the following Ethics Committee: CEIm Gerencia de Atención Especializada de Toledo, Antigua Escuela Enfermería de Toledo, C/ Alicante, s/n 45005 TOLEDO, Spain (ID: 760/19/EC).
**Funding for this study:** This project has received funding from the European Union's Horizon 2020 research and innovation programme under grant agreements No 830265 and 872752.
**Author Disclosures:**
Lorenzo Sani: Shareholder: UBT srl Employee: UBT srl
Lina Marcela Cruz Hernandez: Nothing to disclose
Daniel Álvarez Sánchez-Bayuela: Nothing to disclose
Cristina Romero Castellano: Nothing to disclose
Ana Rodríguez Sánchez: Nothing to disclose
Paul Martin Aguilar Angulo: Nothing to disclose
Gianluigi Tiberi: Shareholder: UBT srl Founder: UBT srl
María Del Pilar Sánchez Camacho González Carrato: Nothing to disclose
Rubén Giovanetti González: Nothing to disclose

**RPS 702-3**

**Generation of a breast imaging repository and an AI breast imaging toolbox by INCISIVE 2020**

*M. Lelegianni*[1], I. Georgiou[2], T. Loncar Turukalo[3], Y. Roussakis[4], C. Barelle[5], A. Lalas[1], S. Sykiotis[2], G. Tsakou[6], I. Chouvarda[1]; [1]Thessaloniki/GR, [2]Athens/GR, [3]Novi Sad/RS, [4]Limassol/CY, [5]Luxembourg/LU, [6]Marousi/GR
*(marialelegianni@gmail.com)*

**Purpose:** The purpose of this study was to present the research work towards the creation of a breast cancer imaging repository and the development of an AI-based toolbox that aims to provide a solution to the current challenges a breast radiologist encounters. This research has been realised as a part of the INCISIVE 2020 programme.
**Methods or Backround:** A total of 5889 breast cancer patients with studies on various imaging modalities (mammography, ultrasound, CT, MRI, PET/CT) have been recruited. Clinical, laboratory and histopathological data were also collected. A data collection protocol is set, with a pre-specified structure of data and inclusion criteria of recruited patients. Data are de-identified, and breast lesions are being annotated by each hospital research team. Data are quality-checked for inconsistencies before being uploaded to the INCISIVE repository. Meanwhile, AI algorithms are developed based on retrospective data together with open databases and will be refined based on the prospective data collected.
**Results or Findings:** This data collection process will enable the creation of a breast imaging repository which will facilitate the accessibility and secure donation of data along with the increase of AI-based solutions. Moreover, the development of algorithms (using artificial intelligence and machine learning technologies) aims to address specified clinical challenges in breast cancer imaging, such as: discrimination between malignant and benign breast lesions or microcalcifications in mammography, grouping of patients by breast density in mammography, segmentation of malignant lesions and classification of various lesions in MRI images and a potential case-based prediction of response to treatment.
**Conclusion:** INCISIVE aims to provide an imaging repository to support research and an AI toolbox that will improve the accuracy of breast cancer diagnosis and facilitate the definition and evaluation of treatment plans.
**Limitations:** The study is limited by the lack of organised databases in certain centres.
**Ethics committee approval:** No information was provided by the submitter.
**Funding for this study:** The study was funded by Horizon 2020 EU.
**Author Disclosures:**
Ioanna Chouvarda: Nothing to disclose
Maria Lelegianni: Nothing to disclose
Antonios Lalas: Nothing to disclose
Irene Georgiou: Nothing to disclose

Stavros Sykiotis: Nothing to disclose
Caroline Barelle: Nothing to disclose
Gianna Tsakou: Nothing to disclose
Yannis Roussakis: Nothing to disclose
Tatiana Loncar Turukalo: Nothing to disclose

### RPS 702-4

**Presenting the MammoScreen project: innovative and safe microwave-based imaging technology to make breast cancer screening more accurate, inclusive, and female-friendly**

*G. Tiberi*[1], D. Álvarez Sánchez-Bayuela[2], N. Ghavami[3], C. Romero Castellano[2], T. Milagre[4], P. O'Brien[5], M. Riccaboni[6], M. Ghavami[3], S. Dudley[3], G. D'Errico[7], S. Freeney[8]; [1]Perugia/IT, [2]Toledo/ES, [3]London/UK, [4]Lisbon/PT, [5]Sheffield/UK, [6]Lucca/IT, [7]Siena/IT, [8]Gorey/IE
*(gianluigi@ubt-tech.com)*

**Purpose:** One of the goals of the HORIZON-MISS-2021-CANCER-02-01 scheme is to validate new methods and technologies for cancer screening and early detection, preferably non-invasive and more inclusive than current approaches. In this context, we present MammoScreen, one of the projects selected for funding. The aim of MammoScreen is to generate evidence regarding the use of MammoWave (a recently developed microwave imaging device) as a breast cancer (BC) screening technique in population-based programmes promoted by national or regional health systems.
**Methods or Backround:** The core technology to be used is MammoWave: a safe, non-ionizing microwave imaging device for breast lesion detection. The clinical validation of MammoWave was performed on 500 subjects thus far. In MammoScreen, a dedicated clinical protocol will be developed with the primary objective of verifying that MammoWave (augmented by Artificial Intelligence) reaches a specificity and sensitivity of 90% in a dedicated population-based trial. Participants will be women currently included in screening programmes who will also undergo MammoWave analysis. The trial (multicentric, prospective, double-blinded) will involve 10,000 women (sample size calculated considering a 2-tailed 95% confidence interval (CI width=10) and prevalence of female BCs of 0.5%) in at least 10 recruitment centres in Italy, Spain, Portugal, Germany, Poland and Ukraine. As a reference standard, we will use the output of conventional breast screening, i.e. mammogram, which will be integrated with other radiological/histological outputs.
**Results or Findings:** We will evaluate MammoWave specificity and sensitivity (also stratified by breast density).
**Conclusion:** MammoScreen will generate evidence regarding the use of MammoWave in BC screening. Expected impacts are many, including: (1) women will benefit from safe, more inclusive (no age-limitation), more comfortable (no breast compression), accessible and accurate BC screening, (2) health policymakers will have the evidence to review current population-based BC screening programmes.
**Limitations:** Not applicable.
**Ethics committee approval:** No information provided by the submitter.
**Funding for this study:** The study was funded by HORIZON-MISS-2021-Cancer-02-01 (GA.101097079).
**Author Disclosures:**
Sandra Dudley: Nothing to disclose
Daniel Álvarez Sánchez-Bayuela: Nothing to disclose
Tamara Milagre: Nothing to disclose
Siobhan Freeney: Founder: Beingdense.com
Paul O'Brien: CEO: ELAROS
Mohammad Ghavami: Nothing to disclose
Cristina Romero Castellano: Nothing to disclose
Gianluigi Tiberi: Founder: UBT
Massimo Riccaboni: Nothing to disclose
Gianni D'Errico: Nothing to disclose
Navid Ghavami: Founder: UBT

### RPS 702-5

**Shifting the position on breast MRI: development and testing of a 60-channel, ultra-flexible, supine breast coil**

*J. Vincent*[1], C. Follante[1], M. Giancola[1], V. Taracila[1], Y-J. Stickle[1], C. Moran[2], B. Hargreaves[2], B. Daniel[2], F. Robb[1]; [1]Aurora, OH/US, [2]Stanford, CA/US

**Purpose:** While breast MR scans are typically done in the prone position, advantages to screening in the supine position include an increase in patient comfort and more accurate positioning for surgical and radiation therapy planning. To enhance patient comfort and demonstrate the potential of supine breast imaging, a 60-channel, ultra-flexible, supine breast coil was developed and tested.
**Methods or Backround:** The 60-channel supine breast coil is comprised of two, 30-channel, separable halves to allow for unilateral or bilateral breast imaging. The elements are 7 cm in diameter, ultra-flexible, and highly overlapped on fabric materials with a medical grade sleeve based on the GE AIR™ Coil technology. The contoured cups allow for bust sizes up to a 40DD with coverage extending from the axilla, breast, supraclavicular lymph nodes, and lower torso. This coil was tested on a 3T GE Premier scanner on both phantom and human subjects with and without the additional 30 channels of the embedded posterior coil. Accelerations up to 6 in the R/L direction and 2 in the S/I direction were tested using sequences such as SSFSE, LAVA Flex, and 3D Cube Flex.
**Results or Findings:** Clear visualisation of the axilla was seen for both respiratory-gated and free-breathing scans, even in the presence of motion. The high-density, high-channel-count allowed for increased acceleration and faster imaging times that enabled mitigation of respiratory and cardiac motion.
**Conclusion:** The ultra-flexible coil and supine positioning provided high-resolution images, enhanced patient comfort, and a greater degree of breast coverage compared to conventional prone coils.
**Limitations:** Due to its flexible nature, the side of the coil can be lifted for biopsy, but stabilisation of the breast tissue will be more challenging than a conventional coil.
**Ethics committee approval:** IRB GE.
**Funding for this study:** The study was funded by GE Healthcare.
**Author Disclosures:**
Yun-Jeong Stickle: Employee: GE Healthcare
Fraser Robb: Employee: GE Healthcare
Jana Vincent: Employee: GE Healthcare
Clyve Follante: Employee: GE Healthcare
Catherine Moran: Research/Grant Support: GE Healthcare
Brian Hargreaves: Research/Grant Support: GE Healthcare
Bruce Daniel: Research/Grant Support: GE Healthcare
Mark Giancola: Employee: GE Healthcare
Victor Taracila: Employee: GE Healthcare

### RPS 702-6

**A panoramic view for supine breast MRI**

L. Nohava, M. Obermann, P. Clauser, P. A. Baltzer, *E. Laistler*; Vienna/AT

**Purpose:** A wearable coil worn like a vest (“BraCoil”) has recently been shown to improve the signal-to-noise ratio by a factor of up to three in supine breast MRI. However, the visualisation of supine breast images is inefficient when using cartesian views due to the bent shape of the breast along the chest wall. We introduce a novel panoramic view similar to a panoramic dental x-ray to make the reading of supine breast MRI more efficient.
**Methods or Backround:** On three healthy volunteers with breast volumes 657/1338/3020mL, isotropic 3D gradient-echo images were acquired using the BraCoil (supine) and a rigid breast coil (prone) on a 3T scanner. Breast tissue was manually segmented. Panoramas were created by applying two consecutive curved planar reformatting transforms along manually drawn curves (sagittally along the sternum and axially along the breast shape). The segmentations were transformed in the panoramic view, keeping slice thickness constant. Data were cropped to the extent of the breast tissue. The remaining slices in supine panoramic/coronal/axial and prone axial images were counted.
**Results or Findings:** The panoramic view resulted in considerably fewer slices containing breast tissue. Compared to the reference, slice numbers were reduced by 60-78%, depending on breast size. Similar results (68-80%) were obtained for the comparison to supine axial images. Against supine coronal data, the reduction was 45-47%.
**Conclusion:** We present an intuitive panoramic view of both breasts that could facilitate the description of the lesion position. The technique reduces the number of slices viewed by a factor of 3-4 compared to the current clinical standard.
**Limitations:** Manual curve drawing for flattening and limited flattening accuracy in image corners could be remedied with the automatic detection of the rib cage surface and 3D surface-based flattening.
**Ethics committee approval:** Medical University of Vienna, EK Nr. 2137/2021.
**Funding for this study:** FWF I-3618/ANR-17-CE19-0022 & ÖGS Forschungsförderung.
**Author Disclosures:**
Pascal A.T. Baltzer: Nothing to disclose
Lena Nohava: Nothing to disclose
Elmar Laistler: Founder: ALSIX GmbH
Michael Obermann: Nothing to disclose
Paola Clauser: Nothing to disclose

### RPS 702-7

**Initial results of the SOLUS clinical trial: non-invasive characterisation of breast lesions through a multimodal innovative device**

*E. Venturini*, G. Maffeis, C. Losio, M. Rodighiero, S. La Marca, N. Mule, C. Solus, P. Taroni, P. Panizza; Milan/IT
*(eleventurini86@gmail.com)*

**Purpose:** We present the first clinical results of a research project aimed at the development of a hybrid ultrasound (US) and diffuse optical tomography (DOT) device, the so-called SOLUS system. It was created by the SOLUS consortium, which unites 9 European partners, including expertise in DOT, US and image processing.

**Methods or Backround:** The SOLUS system consists of a Hologic, Supersonic Mach®30 US unit combined with an innovative DOT device. The examination includes B-mode ultrasound, colour-Doppler, Shear Wave Elastography and DOT through a probe with a standard US transducer in the centre, flanked by 8 optodes that emit and receive light in the red and near-infrared spectrum. DOT allows measuring water concentrations, lipids, collagen, total haemoglobin content and saturation. The aim of the feasibility study in the clinical setting is to compare the parameters obtained in vivo from the evaluation of 20 benign and 20 malignant breast lesions to verify the possibility of differentiating them with this multimodal non-invasive device.
**Results or Findings:** To date, we have enrolled 24 patients, 7 with malignant nodules and 17 with benign findings. Machine learning algorithms based on lesion composition data measure a 91% sensitivity and 75% specificity.
**Conclusion:** Very initial data, based for now only on DOT findings guided by US, suggest that a multiparametric analysis may have a role in the characterisation of breast lesions.
**Limitations:** The study is limited by a small data set.
**Ethics committee approval:** Hospital Ethical Committee and Italian Ministry of Health approved the study. Copies of the approvals, protocols and informed consent forms were submitted to the European Commission.
**Funding for this study:** The project has received funding from the European Union's Horizon 2020 research and innovation programme under grant agreement No 731877.
**Author Disclosures:**
Giulia Maffeis: Nothing to disclose
Paola Taroni: Nothing to disclose
Consortium Solus: Nothing to disclose
Salvatore La Marca: Nothing to disclose
Claudio Losio: Nothing to disclose
Elena Venturini: Nothing to disclose
Mariagrazia Rodighiero: Nothing to disclose
Nikhitha Mule: Nothing to disclose
Pietro Panizza: Nothing to disclose

### RPS 702-8

**Comparison of the accuracy of digital breast tomosynthesis with 3DQuorum™ Smart Slice**

J. C. Morel, *R. K. Wasan*, A. Iqbal, C. Peacock, N. Patel, R. Rahim, M. Michell, K. Satchithananda; London/UK
*(Rema.wasan@nhs.net)*

**Purpose:** Digital breast tomosynthesis (DBT) increases cancer detection compared with 2D digital mammography. DBT has drawbacks in reading times, data storage and retrieval. In this study, we evaluate a new version of DBT called 3DQuorum™, which employs an algorithm to reconstruct DBT slices to generate fewer slices while enhancing key mammographic features.
**Methods or Backround:** Ethical approval was not required for this study. Women recalled following routine screening, mammograms were included. Participants underwent DBT as standard; 3DQuorum™ were generated from this data set. Readers scored each lesion for the probability of malignancy on the 3DQuorum™ plus synthetic 2D, followed by standard DBT plus Synthetic 2D. In addition, a qualitative assessment of standard DBT vs 3DQuorum™ was made. Scores were compared with the assessment outcome.
**Results or Findings:** 487 women were included. 164 were diagnosed with malignancy (139 invasive and 25 non-invasive), 92 as benign and 231 as normal. 256 women underwent needle biopsies. The diagnostic accuracy was evaluated using receiving operating characteristic (ROC) analysis. For 3DQuorum™, the area under the curve (AUC) was 0.942, 95% confidence interval (CI) [0.916 - 0.962], and for DBT, the AUC was 0.937, 95% CI [0.911 - 0.958]; the difference in AUCs was 0.00454 with p-value 0.3211. The difference is statistically non-significant, suggesting that the accuracy of standard DBT and 3DQuorum™ is comparable. Qualitatively, 11.29% (55/487) reads observed that 3DQuorum™ was slightly better than standard DBT, while 81.5% (397/487) observed it was equal to DBT ($p<0.0001$).
**Conclusion:** This study suggests that 3DQuorum™ slices could be read in place of standard DBT leading to advantages in reading speed and reduced PACS storage. Further, more extensive studies are required.
**Limitations:** 3DQuorum™ images were read sequentially rather than blinded. Small sample size of the study.
**Ethics committee approval:** No information provided by the submitter.
**Funding for this study:** No funding was received for this study.
**Author Disclosures:**
Asif Iqbal: Nothing to disclose
Keshthra Satchithananda: Nothing to disclose
Michael Michell: Nothing to disclose
Nikhil Patel: Nothing to disclose
Juliet Clare Morel: Nothing to disclose
Clare Peacock: Nothing to disclose
Rema Kaur Wasan: Nothing to disclose
Rumana Rahim: Nothing to disclose

### RPS 702-9

**The effect of the SmartCurve compression system on pain experience and image quality: a randomised placebo-controlled trial in a screening population**

*D. van der Waal*[1], C. van Landsveld-Verhoeven[1], E. Tetteroo[2], R. M. Pijnappel[3], M. Broeders[1]; [1]Nijmegen/NL, [2]Breda/NL, [3]Utrecht/NL
*(d.vanderwaal@lrcb.nl)*

**Purpose:** To compare the SmartCurve and the standard compression system in terms of pain experience and image quality in mammography screening.
**Methods or Backround:** Dutch breast cancer screening participants (n=2499) were randomised to the SmartCurve or placebo group. One additional image, with the SmartCurve or placebo paddle, was made for each participant. Pain experience was measured on a numerical rating scale (NRS). Participants gave an NRS score after compression with the standard paddle and after the SmartCurve or placebo paddle, resulting in two pain scores for each participant. The difference in pain score (standard vs SmartCurve or placebo) was compared between the two groups using an ANCOVA. An unblinded paired comparison of SmartCurve vs standard images was performed by two radiographers (1246 image sets) and two radiologists (320 image sets), using standard criteria for image quality.
**Results or Findings:** On average, the pain score in the SmartCurve group improved by 0.19 points more (on a scale from 0 to 10) than in the control group (95% CI 0.09-0.28; $p<0.01$). Adjusting for screening unit and type of view (CC or MLO) had no effect on the estimates. Despite some interobserver variation, all observers agreed that there was either no preference or a preference for the standard paddle in terms of image quality. Decreased contrast in the dark areas and visibility of structures were the main concerns for the SmartCurve images.
**Conclusion:** The use of the SmartCurve system only seemed to result in a very marginal pain reduction. The visibility of structures was less than for images acquired with the standard compression system.
**Limitations:** Blinding of radiographers who made the mammograms and blinding of observers who assessed image quality was not possible.
**Ethics committee approval:** Institutional Review Board approval was not required because a permit for this study, equal to institutional review board approval, was obtained from the Minister of Health, Welfare and Sport under the Dutch Population Screening Act.
**Funding for this study:** The study was funded by LRCB and the National Institute for Public Health and the Environment (RIVM).
**Author Disclosures:**
Mireille Broeders: Speaker: Hologic, Siemens Healthcare Other: Research agreements with Hologic, Volpara, iCAD
Cary van Landsveld-Verhoeven: Nothing to disclose
Danielle van der Waal: Nothing to disclose
Ruud M Pijnappel: Nothing to disclose
Eric Tetteroo: Nothing to disclose

**08:00-09:00** **Research Stage 3**

### Research Presentation Session: Paediatric

### RPS 712

### Paediatric neuroradiology and foetal imaging

**Moderator**
R. Gunny; London/UK

### RPS 712-2

**Follow-up study of neuropsychological scores of infant patients with methylmalonic acidemia (cblC type) and influencing factors of cerebral MRI characteristics**

*Y. Wang*[1], T. Chen[2], L. Yang[2], C. Sui[2], X. Li[1], L. Guo[2]; [1]Yantai City/CN, [2]Jinan/CN
*(wyy19961107@163.com)*

**Purpose:** The purpose of this study was to investigate whether baseline cerebral MRI characteristics could predict therapeutic responsiveness in patients with methylmalonic acidemia (MMA)(cblC type).
**Methods or Backround:** The cerebral MRI results of 40 patients with MMA (cblC type) were evaluated by a neuroradiologist. Neuropsychological scores and imaging data were collected. Neuropsychological tests were performed before and after standardised treatment.
**Results or Findings:** Thirty-eight patients initially underwent neuropsychological testing [developmental quotient (DQ)]. MMA (cblC type) patients with cerebellar atrophy, corpus callosum thinning, and ventricular dilation had significantly lower DQs than those without ($P<0.05$). Through multivariate linear stepwise regression equation after univariate analysis, ventricular dilation was the most valuable predictor of lower DQs. Thirty-six patients (94.7%) underwent follow-up neuropsychological testing. The pre-and post-treatment DQ values were not significantly different (Z=-1.611, P=0.107). The post-treatment DQ classification (normal, moderately low, or extremely low) showed nearly no change compared to the pre-treatment DQ classification (k =0.790, P<0.001).
**Conclusion:** Ventricular dilation, cerebral atrophy and corpus callosum thinning are the main MRI abnormalities of MMA (cblC type) patients, and these manifestations are significantly correlated with delayed development in children. MRI findings can be considered an important tool for determining the severity of MMA (cblC type) in patients.
**Limitations:** The short follow-up period is a limitation of this study. In future studies, we should continue to follow up with the patients included in this study and obtain neuropsychological scores and MRI data at multiple time points to better explore the prognosis of MMA and its influencing factors.
**Ethics committee approval:** No information provided by the submitter.
**Funding for this study:** This work was supported by grants from the National Natural Science Foundation of China (81800840).
**Author Disclosures:**
Chaofan Sui: Nothing to disclose
Xianglin Li: Nothing to disclose
Lingfei Guo: Nothing to disclose
Tao Chen: Nothing to disclose
Yuanyuan Wang: Nothing to disclose
Linfeng Yang: Nothing to disclose

### RPS 712-3

**Magnetic resonance imaging findings of paediatric patients presenting with sudden visual impairment**
*E. Çıvgın*, I. S. Parlak, S. Duran, Ö. Dedeoğlu, N. B. Gülleroğlu, İ. E. Erbahçeci Timur, A. S. Ekşioğlu; Ankara/TR
*(esrayurduseven@gmail.com)*

**Purpose:** Sudden vision impairment (VI) is an extremely worrisome symptom that requires detailed investigation and quick diagnosis for appropriate management in paediatric patients. The objective of this study was to determine the most common non-traumatic cranial and orbital causes of VI in paediatric patients according to the age groups with magnetic resonance imaging (MRI) in children who presented with VI and to determine the frequency of common causes according to the age groups.
**Methods or Backround:** Electronic medical records of paediatric patients who underwent MRI for VI between June 2019 and June 2022 were retrospectively reviewed. The patients were divided into 3 groups according to their age preschoolers, school-aged and adolescents. Demographic features of patients, MRI results and final diagnosis were collected and analysed by age groups.
**Results or Findings:** The records of 298 children were analysed. The most common neurological causes were idiopathic intracranial hypertension (IIH) (12%), tumour (10.7%), infectious (7.7%), demyelinating disorders (DD) (7.3%), and optic neuritis (ON) (4%). MRI was abnormal in 130 children (43.6%), and the most common findings detected on MRI were IIH (26.1%), tumour (24.6%), DD (16.9%), infectious (10%), and ON (6.9%). The most common imaging abnormalities detected on MRI were tumours in preschool children, IHH in the school-aged, and DD in adolescents.
**Conclusion:** With the advantage of being able to visualise the afferent visual system, MRI plays an important role in diagnosing life-threatening underlying diseases, including infection, tumour, and DD in children with acute VI. Children presenting with acute VI had clinically significant MRI findings, including tumour, IHH, DD, and ON. Especially in young children, cranial and orbital MRI should be considered to detect important causes.
**Limitations:** Retrospective, the time between the onset of the VI and imaging was not standardised.
**Ethics committee approval:** No information provided by the submitter.
**Funding for this study:** No funding was received for this study.
**Author Disclosures:**
Izzet Selçuk Parlak: Nothing to disclose
Semra Duran: Nothing to disclose
Esra Çıvgın: Nothing to disclose
Nadide Başak Gülleroğlu: Nothing to disclose
İnci ELİF Erbahçeci Timur: Nothing to disclose
Özge Dedeoğlu: Nothing to disclose
Ayşe Seçil Ekşioğlu: Nothing to disclose

### RPS 712-4

**Assessment of fetoplacental diffusion and perfusion properties in intrauterine growth restriction: a preliminary study using Intravoxel Incoherent Motion (IVIM) MRI**
*M. Gennarini*, R. Ninkova, A. Cupertino, V. Miceli, G. Ercolani, V. Celli, S. Ciulla, C. Catalano, L. Manganaro; Rome/IT
*(marco.gennarini@hotmail.it)*

**Purpose:** To investigate the potential use of Intravoxel Incoherent Motion (IVIM) imaging in the study of microperfusion and microstructural characteristics of fetal lungs and placenta in IUGR fetuses, comparing IVIM parameters with those of a healthy control group.
**Methods or Backround:** Complex interactions between placental and fetal environments ensure normal fetal growth. Impairment of the fetoplacental unit may lead to Intrauterine Growth Restriction (IUGR), which is associated with perinatal morbidity and mortality, neonatal complications such as ARDS, and long-term complications, like cardiovascular disease and neurodevelopmental delay. The development of new non-invasive techniques for prompt and accurate prenatal diagnosis is critical to improving the prenatal and postnatal outcomes of IUGR fetuses.
**Results or Findings:** Preliminary results show that IVIM parameters, with special regard to perfusion fraction f, may be potential in vivo biomarkers to discriminate between IUGR and healthy fetuses and correlate with GA and BW.
**Conclusion:** Preliminary results show that IVIM parameters, with special regard to perfusion fraction f, may be potential in vivo biomarkers to discriminate between IUGR and healthy fetuses, and correlate with GA and BW.
**Limitations:** Small study sample; foetal movements; long acquisition of MRI sequences.
**Ethics committee approval:** No information provided by the submitter.
**Funding for this study:** No funding was received for this study.
**Author Disclosures:**
Marco Gennarini: Nothing to disclose
Valentina Miceli: Nothing to disclose
Roberta Ninkova: Nothing to disclose
Giada Ercolani: Nothing to disclose
Angelica Cupertino: Nothing to disclose
Sandra Ciulla: Nothing to disclose
Veronica Celli: Nothing to disclose
Carlo Catalano: Nothing to disclose
Lucia Manganaro: Nothing to disclose

### RPS 712-5

**Altered brain activity and childhood trauma in Chinese adolescents with borderline personality disorder**
*X. Yi*, Y. Fu, X. Wang, B. T. Chen; Changsha/CN
*(yixiaoping@csu.edu.cn)*

**Purpose:** The aim of this study was to assess functional alteration and its association with childhood trauma in Chinese adolescents with BPD.
**Methods or Backround:** Childhood trauma may cause borderline personality disorder (BPD). A total of 187 adolescents with BPD (12–17 years) and 207 matched healthy controls (HCs) were enrolled. A neuroimaging subgroup consisted of 50 adolescents with BPD and 21 HCs underwent brain resting-state functional magnetic resonance imaging (rs-fMRI). Both neural activity, as indicated by amplitude of low-frequency fluctuation (ALFF) and seed-based functional connectivity (FC), were performed. A correlative analysis of functional alterations with childhood trauma assessment was performed.
**Results or Findings:** Adolescents with BPD had a significantly higher rate of childhood trauma (P < 0.001). Compared with HCs, adolescents with BPD showed decreased ALFF in the cortical regions, including the left superior frontal gyrus and right middle occipital gyrus, and default mode network (DMN) regions, including the left angular gyrus and medial superior frontal gyrus. Adolescents with BPD also showed enhanced ALFF in the limbic system (left hippocampus, insula, thalamus) (P＜0.05). There were significant correlations between the insula ALFF and childhood trauma assessment for emotional neglect, physical abuse and physical neglect (P < 0.01). Moreover, adolescents with BPD showed increased FC between the left insula and right cortical regions (P < 0.05).
**Conclusion:** There were alterations of brain activity as indicated by ALFF in the limbic-cortical circuit and DMN regions in adolescents with BPD. The activity in the left insula was correlated with emotional neglect. The functional alterations of insula may serve as a potential neuroimaging biomarker for adolescents with BPD who suffered from childhood trauma.
**Limitations:** A small size study. Patients with more severe BPD symptoms and some had comorbidities.

**Ethics committee approval:** This study was approved by the ethics committee of Xiangya Hospital of Central South University.
**Funding for this study:** This study was approved by China Post-Doctoral Science Foundation (2022M713536).
**Author Disclosures:**
Yan Fu: Nothing to disclose
Xueying Wang: Nothing to disclose
Bihong T Chen: Nothing to disclose
Xiaoping Yi: Nothing to disclose

### RPS 712-6

**MRI evaluation of central tegmental tract hyperintensity in paediatric patients**
E. U. Büyükceran[1], *S. Kaynak Şahap*[1], S. Genç[2], Ö. S. Fitoz[1]; [1]Ankara/TR, [2]Izmir/TR
*(dr.seda@gmail.com)*

**Purpose:** The clinical significance of central tegmental tract hyperintensity (CTTH) is still unclear. Generally, these lesions are considered pathological, and they may be associated with age-related selective sensitivity of the dorsal brain stem. Our purpose is to reveal the clinical and radiological significance of CTTH on T2WI and to evaluate its relationship with other MRI sequences as well as their clinical associations.
**Methods or Backround:** Brain MRI examinations of paediatric patients were evaluated retrospectively. In 104 patients with STTH on T2WI, the equivalents of this finding on DWI, ADC map, and FLAIR sequences were also evaluated. In addition to the radiological findings, the patient's clinical information was recorded. As well as descriptive statistics, the Mann–Whitney U test was applied to compare the non-normal data. The Chi-squared test and Fisher exact test were used to analyse categorical variables.
**Results or Findings:** The clinical diagnoses of children with CTTH were epilepsy (24%), metabolic disease (15.4%), CP (14.4%), growth retardation (10.6%), and immunodeficiency (7.7%). CTTH was most commonly found in isolation. The median age was 17 months in those with diffusion restriction and 33 months in those without diffusion restriction (P=0.01). "Hyperintensity loss in FLAIR sequence" is at a statistically significant level in patients followed up with CP, epilepsy and metabolic diseases (p=0.020).
**Conclusion:** The smaller median age of patients with diffusion restriction, the correlation between diffusion restriction and FLAIR, and the relationship between FLAIR and clinical diagnoses suggested that CTTH may be a common finding of two different pathophysiological processes. MRI findings may be useful to differentiate axial diffusivity changes and axonal damage from more chronic involvement and myelin damage. Diffusivity studies with DTI in larger series will increase our knowledge about this phenomenon.
**Limitations:** No limitations were identified.
**Ethics committee approval:** This study was approved by the Ethics Committee of Ankara University.
IRB approval number: İ4-229-20.
**Funding for this study:** No funding was received for this study.
**Author Disclosures:**
Sinan Genç: Nothing to disclose
Ömer Suat Fitoz: Nothing to disclose
Seda Kaynak Şahap: Nothing to disclose
Emre Utkan Büyükceran: Nothing to disclose

### RPS 712-7

**Abnormalities of the corpus callosum: can prenatal imaging predict the genetic status and correlations between imaging phenotype and genotype**
*T. Nguyen*, S. Valence, S. Heide, B. Keren, C. Garel; Paris/FR
*(toan.nguyen@aphp.fr)*

**Purpose:** Recent studies have assessed prenatal exome sequencing (pES) in the management of abnormalities of the corpus callosum (AbnCC). The objective of our study was to compare genetic findings with prenatal imaging data.
**Methods or Backround:** This was a retrospective study between 2018 and 2020 of ultrasound and/or MRI review of fetuses with AbnCC from 28 French centres who had pES in our institution. AbnCC were classified as complete (cACC) or partial (pACC) agenesis, short CC (sCC), callosal dysgenesis (CD), interhemispheric cyst (IHC) or pericallosal lipoma (PL), isolated or not. Only class 4 (probably pathogenic) or class 5 (pathogenic) variants were considered.
**Results or Findings:** Of the 162 fetuses included, 49 were excluded (no imaging available). Results show (class 4 or 5 variants/number of cases): - 3/32 isolated cACC, - 3/19 isolated pACC, - 0/10 isolated sCC, - 8/16 non-isolated cACC, pACC or sCC, - 5/10 isolated CD, - 8/11 non-isolated CD, - 0/12 IHC and PL. We did not find any phenotype suggesting a particular genetic abnormality except for a case of mTOR pathway mutation and a case of tubulinopathy.
**Conclusion:** Prenatal imaging remains pivotal in the setting of prenatally diagnosed AbnCC as a key aid to prognosis. pES abnormalities were more frequent in cases of CD (especially in non-isolated cases) and absent in isolated sCC, IHC and PL.
**Limitations:** It was a retrospective multicentre study with sometimes poor imaging qualities. In thirty cases, ultrasound or MR imaging was missing.
**Ethics committee approval:** This study was approved by our institution (CER-2022-001).
**Funding for this study:** No funding was received for this study.
**Author Disclosures:**
Stéphanie Valence: Nothing to disclose
Catherine Garel: Nothing to disclose
Solveig Heide: Nothing to disclose
Boris Keren: Nothing to disclose
Toan Nguyen: Nothing to disclose

**08:00-09:00** **Research Stage 4**

### Research Presentation Session: Cardiac

### RPS 703

### Epicardial adipose tissue and obesity

**Moderator**
M. Pirnat; Maribor/SI
*(maja.pirnat@ukc-mb.si)*
**Author Disclosures:**
Maja Pirnat: Advisory Board: Bayer

### RPS 703-2

**The role of epicardial and pericoronary adipose tissue radiomics in identifying patients with non-ST-segment elevation myocardial infarction**
*Z. Wang*, B. Yang; Shenyang/CN

**Purpose:** This study aimed to ascertain if the radiomics features of epicardial adipose tissue (EAT) and pericoronary adipose tissue (PCAT) based on coronary computed tomography angiography (CCTA) could identify non-ST-segment elevation myocardial infarction (NSTEMI) from unstable angina (UA).
**Methods or Backround:** This retrospective case-control study included 83 patients with NSTEMI and 83 controls with UA who were matched based on gender, age, body mass index (BMI), cardiovascular risk factors, and medications. Randomly, all patients were separated into training (n = 116, 70%) and validation cohorts (n = 50, 30%). The EAT and PCAT radiomics features selected by maximum relevance minimum redundancy (mRMR) and least absolute shrinkage and selection operator (LASSO) were adopted to build logistic regression models. Finally, we developed an EAT radiomics model, a PCAT radiomics model, and a combined model by combining the two radiomics models. Discrimination, calibration, and clinical application were employed to assess the performance of all models.
**Results or Findings:** The area under the curves (AUCs) of EAT model, PCAT model, and combined model were 0.739 (95% CI: 0.650-0.828), 0.868 (95% CI:0.804-0.933), 0.905 (95% CI: 0.854-0.956) in the training cohort, and 0.717 (95% CI: 0.573-0.861), 0.843 (95% CI: 0.735-0.951), 0.882 (95% CI: 0.786-0.978) in the validation cohort, respectively.
**Conclusion:** The combination of EAT and PCAT radiomics may have the potential to distinguish between NSTEMI and UA.
**Limitations:** It was a single-centre retrospective case-control study limited by sample size, and all patients received CCTA examination employing the same equipment and procedure.
**Ethics committee approval:** NO. Y (2022)113
**Funding for this study:** The key research and development project of Liaoning Province of China (No. 2020JH2/10300119).
**Author Disclosures:**
Zhenguo Wang: Nothing to disclose
Bengqiang Yang: Nothing to disclose

### RPS 703-3

**Volumetric assessment of epicardial adipose tissue with cardiovascular CT imaging: association with coronary plaque morphology**
*E. Meltem*, S. Turgut Güneş, İ. T. Rakıcı, A. S. Mahmutoglu; Istanbul/TR
*(meltem_emine@hotmail.com)*

**Purpose:** Epicardial adipose tissue (EAT) is a local adipose tissue, located between the myocardium and visceral pericardium. Recent studies have shown that epicardial adipose volume (EAV) is associated with the development of atherosclerosis and metabolic syndrome. Cardiac CT provides a reliable assessment of EAV due to its high spatial resolution and accurate volumetric coverage of the heart. We aimed to assess the relation between EAV and coronary plaque morphology as well as other clinical findings.
**Methods or Backround:** This retrospective study included 49 patients who underwent ECG-gated non-contrast cardiac computed tomography (NCT) and coronary CT angiography (CTA) on the same day and were found to have coronary artery disease. Evaluation of plaque morphologies was done on CTA images with the help of the Toshiba Aquilion version 4.1 workstation. High-risk plaque was defined in the presence of two or more high-risk plaque features (positive remodelling, low < 30 Hounsfield Units, napkin-ring, spotty calcium) (CAD-RADS™ 2.0 - 2022). EAV was measured on NCT using a free, online, semi-automated segmentation tool (Medseg). The software was arranged to delimit areas with a density between -30 and – 200 HU, and the pericardium was manually outlined. EAV was calculated by software from marked epicardial fat. An independent sample t-test was used for the comparison of quantitative data.
**Results or Findings:** 27 participants (55,1%) were found to have high-risk plaque. The mean EAV was 140,2 ± 49,3 in women and 139,4 ± 36,8 in men. Participants with high-risk coronary plaques had significantly greater EAV (150,4 ± 49,6) than others (126,6 ± 26,1) (p<0.05). Diabetes, hypertension and smoking were associated with higher values of EAV (p>0.05).
**Conclusion:** This study revealed that EAV was significantly larger in patients with high-risk coronary artery plaque.
**Limitations:** Low number of cases and not including BMI values.
**Ethics committee approval:** Ethical approval was taken from 'Istanbul Training and Research Hospital' ethics comittee. (Decision/file number: 296 )
**Funding for this study:** No funding was received for this study.
**Author Disclosures:**
Abdullah Soydan Mahmutoglu: Nothing to disclose
İbrahim Taşkın Rakıcı: Nothing to disclose
Saime Turgut Güneş: Nothing to disclose
Emine Meltem: Nothing to disclose

### RPS 703-4

**Breast density and breast arterial calcifications as potential biomarkers of cardiovascular risk: correlations between mammographic data and cardiac computed tomography**

*C. B. Monti*, D. Capra, V. Magni, M. D. M. Galimberti Ortiz, F. Secchi, F. Sardanelli; Milan/IT
*(caterinab.monti@gmail.com)*

**Purpose:** Our purpose was to appraise a potential role for breast density and breast arterial calcifications (BAC) as biomarkers of cardiovascular risk, assessing their correlations with cardiovascular risk factors such as epicardial adipose tissue (EAT) features and coronary stenosis data.
**Methods or Backround:** We retrospectively reviewed all consecutive patients who had undergone a mammogram and a cardiac computed tomography (CT) within six months at our institution between January 2020 and December 2021. From each mammogram we recorded breast density, divided into two groups (non-dense breasts: categories a or b; dense breasts, categories c or d) and the presence of BAC. From each cardiac CT scan, we recorded each patients' CAD-RADS score, and manually segmented EAT to compute volume and density.
**Results or Findings:** We included 33 patients, all of which females, with a median age of 71 years (interquartile range, IQR, 58−77 years). The median interval between mammography and CT was 2 months (IQR 2–5 months). Patients with non-dense breasts displayed a greater EAT volume than those with dense breasts (104 cm3, IQR 87−139 cm3 vs 74 cm3, IQR 56−112 cm3, p=0.039). Similarly, patients with BAC displayed a trend towards greater EAT volume than those without BAC (106 cm3, IQR 89−151 cm3 vs 88 cm3, IQR 65−135 cm3, p=0.076). Breast density and presence of BAC did not correlate with EAT density (p=0.520 and p=0.759, respectively). The presence of BAC displayed a moderate correlation with the CAD-RADS score (ρ=0.431, p=0.012), whereas breast density did not (p=0.190).
**Conclusion:** Data concerning breast density and presence of BAC, obtained from conventional mammograms, could act as a potential surrogate biomarkers of cardiovascular risk in women.
**Limitations:** Retrospective, single centre, no data on cardiovascular adverse events or outcomes.
**Ethics committee approval:** No information was provided by the submitter.
**Funding for this study:** No funding was received for this study.
**Author Disclosures:**
Francesco Secchi: Nothing to disclose
Francesco Sardanelli: Research/Grant Support: Bracco Advisory Board: Bayer
Caterina Beatrice Monti: Nothing to disclose
Maria Del Mar Galimberti Ortiz: Nothing to disclose
Veronica Magni: Nothing to disclose
Davide Capra: Nothing to disclose

### RPS 703-5

**To assess the thickness of pericoronary adipose tissue and pericoronary fat attenuation index (FAI) as predictors for major adverse cardiac events (MACE)**

*B. N. Biradar*, J. Valakada, A. Ayyappan; Thiruvananthapuram/IN
*(basavarajnbiradar@gmail.com)*

**Purpose:** This study aimed to (1) determine the association between pericoronary adipose tissue thickness, coronary artery stenosis and vulnerable plaque characteristics and (2) to investigate the value of the pericoronary fat attenuation index (FAI) as a marker of adverse coronary events and residual cardiac risk.
**Methods or Backround:** This prospective cohort study involved patients who underwent CT coronary angiograms between 2012 to 2017 at our institute and were followed for 5 to 10 years. The sample size is 100. Patients' risk factors included diabetes mellitus, hypertension, dyslipidemia, smoking, obesity, and family h/o of CAD. They were assessed for the development of acute coronary syndrome (STEMI, NSTEMI), coronary revascularisation, heart failure and sudden cardiac death during the follow-up period. A hazard ratio of each risk factor was calculated. Multiple Logistic Regression analysis of variables was done. A logrank test of each variable for the coronary event was done.
**Results or Findings:** Pericoronary FAI is associated with an increased risk of major adverse coronary events and mortality. A significant association was noted between pericoronary adipose tissue thickness, coronary artery stenosis and vulnerable plaque characteristics.
**Conclusion:** Pericoronary FAI and fat thickness are significant predictors of coronary artery disease and MACE.
**Limitations:** Single centre study and small sample size.
**Ethics committee approval:** No information was provided by the submitter.
**Funding for this study:** No funding was received for this study.
**Author Disclosures:**
Jineesh Valakada: Nothing to disclose
Basavaraj N Biradar: Nothing to disclose
Anoop Ayyappan: Nothing to disclose

### RPS 703-6

**Coronary assessment in patients with severe aortic valve stenosis: invasive resting full-cycle ratio vs CT-fractional flow reserve, plaque composition, and peri-coronary adipose tissue density**

*M. C. Langenbach*[1], H. Wienemann[2], K. Klein[2], C. P. Naehle[2], D. Maintz[2], B. Foldyna[1], I. L. Langenbach[1]; [1]Boston, MA/US, [2]Cologne/DE
*(marcel.langenbach@me.com)*

**Purpose:** This study investigated the capability of the standard pre-interventional contrast-enhanced computed tomography (CT) for assessing ischaemic coronary artery disease (CAD) before transcatheter aortic valve replacement (TAVR). Therefore, the coronary artery stenosis severity, CT-fractional flow reserve (CT-FFR), plaque composition, and pericoronary adipose tissue (PCAT) density, a surrogate of coronary inflammation, were compared. The gold standard was the invasive coronary angiography (ICA) with resting full-cycle ratio (RFR) as a haemodynamic index.
**Methods or Backround:** All patients underwent a standardised contrast-enhanced ECG-gated high-pitch CT from the aortic arch to the common femoral arteries and a subsequent ICA with invasive RFR. Patients had a least one intermediate coronary stenosis. A dedicated heart reconstruction was used for semi-automated evaluation of the coronary lesions, on-site CT-FFR, plaque composition, and PCAT density. ICA with pressure wire measurement using RFR was assumed as the gold standard.
**Results or Findings:** Eighty-seven patients (mean age 77.8 ±7.5 years, 33 women) with 95 lesions were investigated. The CT-FFR significantly correlated with RFR (R=0.66, p<0.001). Further, CT-FFR demonstrated overall a high sensitivity and specificity in predicting RFR ≤ 0.89 (AUC 0.885). Coronary plaque characteristics (p=0.72), necrotic core volume >4% (p=0.46) and PCAT density (p=0.46) were not statistically different in lesions without ischaemia compared to ischaemic lesions. In a nested model analysis, adding these parameters to CT-FFR showed no incremental benefit in predicting ischaemic lesions.
**Conclusion:** Non-invasive CT-FFR was assessed by routine pre-TAVR CTA as a feasible method superior to qualitative analysis of the coronary arteries in CT and ICA in determining ischaemic coronary lesions in patients with relevant aortic valve stenosis. Plaque composition and PCAT density had no independent or incremental benefit in assessing ischaemic coronary lesions compared to or in addition to CT-FFR.
**Limitations:** Retrospective study and a small cohort.
**Ethics committee approval:** IRB, 22-1154.

**Funding for this study:** Not applicable.
**Author Disclosures:**
Isabel Luisa Langenbach: Nothing to disclose
David Maintz: Nothing to disclose
Konstantin Klein: Nothing to disclose
Borek Foldyna: Nothing to disclose
Claas Philip Naehle: Nothing to disclose
Marcel Christian Langenbach: Nothing to disclose
Hendrik Wienemann: Nothing to disclose

### RPS 703-7

**Association of epicardial adipose tissue dispersion at CT and recurrent atrial fibrillation after pulmonary vein isolation**

A. T. Huber, *S. Fankhauser*; Bern/CH

**Purpose:** We aimed to investigate the association of left atrial (LA) epicardial adipose tissue (EAT) dispersion with AF recurrence after pulmonary vein isolation (PVI).
**Methods or Backround:** In a secondary analysis of a prospective registry of consecutive patients undergoing a first PVI, mean EAT attenuation values were measured on contrast-enhanced cardiac CT scans in Hounsfield units (HU) within low (-195 to -45 HU) and high (-44 HU to -15 HU) threshold EAT compartments around the left atrium (LA) and the coronary arteries. EAT dispersion was defined as the difference between the mean HU values within the two EAT compartments, reflecting EAT heterogeneity. Continuous variables were compared between groups using the Mann-Whitney U test. Cox proportional hazard models were used to calculate hazard ratios of predictors of 1-year AF recurrence.
**Results or Findings:** A total of 208 patients were included, 135 with paroxysmal AF and 73 with persistent AF. Patients with persistent AF had a significantly lower LA EAT attenuation both in the low (-76.6 vs -79.2 HU; p<0.001) and high threshold EAT (-26.3 vs -26.6; p=0.004). LA EAT dispersion was significantly larger in patients with persistent compared to paroxysmal AF (52.6 HU vs 49.9 HU; p=0.001). After one year of follow-up, the AF recurrence rate was 76/208 (37%). LA EAT dispersion above the mean was associated with a higher risk of 1-year AF recurrence (HR 2.3, 95% CI 1.5-3.6; p<0.001). It retained its predictive value when corrected for age, sex, body mass index, LA volume and AF type (HR 2.8, 95% CI 1.6-4.6; p<0.001).
**Conclusion:** A larger LA EAT dispersion on contrast-enhanced cardiac CT scans is independently associated with AF recurrence.
**Limitations:** This was a single-centre study; therefore, it should be externally validated using the same methods.
**Ethics committee approval:** The study was approved by the Bern Cantonal Ethics Committee.
**Funding for this study:** No funding was received for this study.
**Author Disclosures:**
Severin Fankhauser: Nothing to disclose
Adrian Thomas Huber: Speaker: Speaker / Consulting honoraria or travel support from Bayer, Bracco and Siemen. All for work outside the submitted study. Grant Recipient: Swiss National Science Foundation, Helmut-Hartweg Foundation, Foundation to Fight against Cancer. All for work outside the submitted study.

### RPS 703-8

**Prognostic role of epicardial adipose tissue quantified with cardiac magnetic resonance in patients undergoing cryoablation for atrial fibrillation**

*F. Giorgino*, D. Tore, M. Gatti, F. P. Papa, F. Menchini, M. Anselmino, H. Xhakupi, R. Faletti, P. Fonio; Turin/IT

**Purpose:** To evaluate the role of epicardial adipose tissue (EAT) measured on preprocedural cardiac MRIs in the setting of transcatheter cryoablation of patients affected by atrial fibrillation (AF) as a possible predictor of arrhythmia recurrence.
**Methods or Backround:** The retrospective monocentric study was approved by the ethics committee on 50 patients with symptomatic AF who underwent transcatheter cryoablation. All patients underwent preprocedural cardiac MRI and transesophageal echocardiogram. The cardiac MRI atrial protocol consisted of non-gated angio-MRI sequences and gated T2-STIR, DWI and 3D inversion recovery (3D IR) late gadolinium-enhanced acquisitions. EAT volume and EAT maximal thickness were measured using manual segmentation and intensity thresholds on 3D IR images using an open-source software 3D slicer.
**Results or Findings:** Median EAT volume indexed on left atrial volume and BSA (EATIV) was 223.7 mm3/ml/m2 (95.6-623.5 mm3/ml/m2; IQR 180.2-292.1 mm3/ml/m2). 18 patients experienced AF recurrence during the follow-up (median time 15.5±7.3 months). EATIV was significantly larger in relapsing patients (269.24 mm3/ml/m2 vs 244.84, p=0.0471). EATIV quantiles differences were statistically significant (p=0.049), even on the logrank test (p=0.03), in patients experiencing recurrence. At Cox univariate regression analysis, EATIV and diabetes demonstrated to be risk factors for arrhythmia recurrence after cryoablation in this cohort of patients.
**Conclusion:** EATIV measured on MRI sequences may represent a predictor of AF recurrence after cryoablation. EATIV may have a role in recurrence risk stratification and clinical decision-making in the setting of preprocedural planning of catheter ablation of AF.
**Limitations:** Retrospective monocentric study with limited sample size.
**Ethics committee approval:** No information was provided by the submitter.
**Funding for this study:** No funding was received for this work.
**Author Disclosures:**
Francesco Pio Papa: Nothing to disclose
Paolo Fonio: Nothing to disclose
Francesca Menchini: Nothing to disclose
Fabio Giorgino: Nothing to disclose
Riccardo Faletti: Nothing to disclose
Henri Xhakupi: Nothing to disclose
Marco Gatti: Nothing to disclose
Matteo Anselmino: Nothing to disclose
Davide Tore: Nothing to disclose

### RPS 703-9

**The nomogram with epicardial adipose tissue parameters based on cardiac MRI shows superior performance for predicting myocardial fibrosis in Duchenne muscular dystrophy: a prospective cohort study**

*W. Yuan*, H-Y. Xu, Y-K. Guo; Chengdu/CN

**Purpose:** Epicardial adipose tissue (EAT) contributes to inflammation and fibrosis of the neighbouring myocardial tissue via paracrine signalling. We investigated the abnormal changes in the amount of EAT in boys with Duchenne muscular dystrophy (DMD) using cardiac magnetic resonance (CMR) imaging. Furthermore, we constructed and validated a nomogram including EAT-related CMR imaging parameters for predicting the occurrence of myocardial fibrosis in patients with DMD.
**Methods or Backround:** Two hundred and forty-two patients with DMD in the training (n=92) and the internal and external validation cohorts (n=67 and 83, respectively) and fifty-seven healthy participants were enrolled in this study. They underwent CMR acquisitions to measure the quantitative parameters of EAT and pericardial adipose tissue. Late gadolinium enhancement (LGE) CMR was performed for detecting myocardial fibrosis in the DMD cohorts.
**Results or Findings:** The cine MRI-based EAT parameters were significantly higher in patients with DMD compared with the healthy controls. Furthermore, the LGE-positive patients with DMD showed significantly higher EAT volume (mean, 48.53 ml; P=0.030) compared with the LGE-negative patients with DMD. Age (OR, 1.525; P=0.001), body fat percentage (OR, 1.090; P=0.001), and EAT volume (OR, 1.195; P=0.002) were independently associated with LGE in both the training and validation sets.
**Conclusion:** Our study demonstrated that compared with healthy controls, patients with DMD had higher EAT deposition. The onset of myocardial fibrosis was associated with an increased EAT volume in the DMD cohorts. The nomogram showed superior performance in the DMD cohorts for predicting the occurrence of myocardial fibrosis.
**Limitations:** First, we did not consider the cumulative dosing of medications such as glucocorticoids for our analysis. Second, our study did not account for differences in DMD genotypes.
**Ethics committee approval:** This prospective CMR study was approved by the China Ethics Committee of Registering Clinical Trials (ChiECRCT-20180107) and the Medical Ethics Committee of Sichuan University (K2019056).
**Funding for this study:** Study supported by National Natural Science Foundation of China (81971586, 82071874, 81901712, 821201080105).
**Author Disclosures:**
Ying-Kun Guo: Nothing to disclose
Hua-Yan Xu: Nothing to disclose
Weifeng Yuan: Nothing to disclose

Thursday

**10:30-12:00** **Research Stage 1**

### Research Presentation Session: Abdominal Viscera & GI Tract

### RPS 801

### Pancreatic cancer and inflammation

**Moderator**
M. A. Bali; Brussels/BE
*(mbali@ulb.ac.be)*

### RPS 801-2

**Pancreatic IPMN cysts detection and segmentation in multi-sequence MRI by deep learning**

N. Mazor, G. Dar, R. Lederman, J. Sosna, *L. Joskowicz*; Jerusalem/IL
*(josko@cs.huji.ac.il)*

**Purpose:** Radiological detection and follow-up of intraductal papillary mucinous neoplasm (IPMN) pancreatic cysts in MRI is a challenging task. The goal of this study was to evaluate the performance of a novel deep-learning automatic method for the accurate and reliable detection and volumetric evaluation of IPMN cysts in MRI.
**Methods or Backround:** Our method consists of three steps: 1) pancreas Region of Interest (ROI) segmentation in the MR scan acquired with the axial TSE protocol; 2) transfer and masking of the computed pancreas ROI to the MR scan acquired with the coronal MRCP protocol by affine transformation to remove unrelated voxels; 3) cysts detection and segmentation in the masked MRCP scan. Both steps 1 and 3 use 3D U-Net models. Our method consists of three steps: 1) pancreas Region of Interest (ROI) segmentation in the MR scan acquired with the axial TSE protocol; 2) transfer and masking of the computed pancreas ROI to the MR scan acquired with the coronal MRCP protocol by affine transformation to remove unrelated voxels; 3) cysts detection and segmentation in the masked MRCP scan. Both steps 1 and 3 use, 3D U-Net models. We retrospectively collected 158 MRI studies with TSE and MRCP sequences of patients with IPMN undergoing follow-up. The dataset was split into training/validation/test sets of 118/17/23 scans. The dataset contains a total of 840 cysts, of which 619 are >5 mm and 221 are >10 mm. The mean number of cysts per scan is 5.3(2.6), with a mean cyst diameter (volume) of 7.4mm (0.91cc). The computed test set results were then compared to their respective manual ground truth delineations.
**Results or Findings:** Our method achieved a cyst inclusion in the pancreas ROI recall=0.94(0.22). Of the cysts inside the pancreas ROI, the cyst detection recall = 0.82(0.27), precision=0.73(0.24) and cyst segmentation Dice=0.81(0.08).
**Conclusion:** Automatic IPMN cyst detection and segmentation in MRI may provide an accurate and reliable method for precise disease evaluation that allows radiologists to optimise the patient's examination time and prevent errors in distinguishing the cysts' size.
**Limitations:** Single observer annotation, one centre.
**Ethics committee approval:** No information provided by the submitter.
**Funding for this study:** This research was funded in part by the Shmuel Meitar Research Fund.
**Author Disclosures:**
Richard Lederman: Consultant: HighRAD Ltd
Leo Joskowicz: Consultant: HighRAD Ltd
Jacob Sosna: Consultant: HighRAD Ltd
Nir Mazor: Nothing to disclose
Gili Dar: Nothing to disclose

### RPS 801-3

**Anatomical carcinogenesis theory for pancreatic cancer: significant association between low insertion anomaly of the cystic duct and new-onset diabetes after the age of 50**

*B. Erkan*, A. Altan, S. Keçeoğlu, H. Suer, C. Tunçer, V. Adsay, M. M. Erkan; Istanbul/TR
*(burcu_akpinar@yahoo.com)*

**Purpose:** Pancreatic ductal-adenocarcinoma (PDAC) incidence is 9:100000, that rises to 1:150 among patients with new-onset diabetes (NOD) after 50 years. More than 80% of the PDAC arise at the periampullary region both in sporadic- and genetically-predisposed patients. This anatomical predilection cannot only be explained by genetic carcinogenesis theory hinting at yet unknown secondary factors.
**Methods or Backround:** We hypothesise that anatomical variations of the pancreatic-biliary ducts facilitate pancreatic carcinogenesis in genetically predisposed individuals. A prospective trial (315S290) was conducted to identify early PDAC cases among NOD patients. New-onset (within 6 months) diabetes (HbA1C>6.5%) and low-insertion anomaly (LIA) were defined as merging of the cystic duct at or below the pancreatic upper margin per study protocol. All patients underwent an MRI scan. MRI results of living-related liver donors (TXD) were taken as healthy controls. Radiological evaluation was made by consensus of two abdominal radiologists. The length of the extrahepatic bile ducts, patterns of union of lobar ducts, sector ducts and cystic ducts, as well as merging with the pancreatic duct, was recorded according to a predefined scheme.
**Results or Findings:** 388 NOD patients were compared with 150 TXD. The length of the extrahepatic bile ducts, the union of lobar and sectoral ducts, and the presence of pancreas divisum were not different between the groups. Extrahepatic bile duct diameter (4.4 vs 3.3mm, P<0.0001) and pancreatic duct diameter (2 vs 1.6mm, p<0.0001) were larger in NOD patients as they were more than 1.5 decades older than the TXD. LIA frequency was significantly higher among NOD patients compared to TXD (43.6% vs 17.3, p<0.0001).
**Conclusion:** LIA might be associated with PDAC in analogy with pancreaticobiliary maljunction causing reflux of biliary and pancreatic juices in reciprocal ducts.
**Limitations:** No limitations were identified.
**Ethics committee approval:** Study protocol was approved by the local IRB (315S290) and informed consent was obtained from all patients.
**Funding for this study:** Funding was received from Tübitak (The Scientific and Technological Research Council of Türkiye).
**Author Disclosures:**
Ceren Tunçer: Nothing to disclose
Selim Keçeoğlu: Investigator: . Author: .
Aylin Altan: Nothing to disclose
Volkan Adsay: Nothing to disclose
Burcu Erkan: Nothing to disclose
Murat Mert Erkan: Nothing to disclose
Hande Suer: Nothing to disclose

### RPS 801-4

**CT evaluation of ectasia of gastrocolic trunk, jejunal and colic veins as a secondary sign of venous infiltration after neoadjuvant therapy in patients with pancreatic ductal adenocarcinoma**

*M. Borzi*, E. Boffa, M. Bariani, G. Zamboni, G. Malleo, G. Mansueto; Verona/IT
*(marti.borzi@gmail.com)*

**Purpose:** To analyse the correlation between ectasia of gastrocolic trunk, jejunal and colic veins and infiltration of the portomesenteric veins after neoadjuvant therapy (NAT) in patients with pancreatic ductal adenocarcinoma.
**Methods or Backround:** Informed consent for the utilisation of clinical and radiologic data was provided by all patients (PAD-R registry, n1101CESC). Patients who underwent pancreaticoduodenectomy after NAT and had a preoperative contrast-enhanced CT available for review were included. Patients were divided into 2 groups: the first group underwent venous resection during PD, and the second group was not subjected to venous resection. The patients with venous resection were subdivided based on histological findings, positive or negative for neoplastic venous infiltration. Two readers in consensus reviewed preoperative CT and analysed: the location and size of the tumour, contact with peripancreatic vessels, resectability based on NCCN guidelines and the presence of ectasia of the gastrocolic trunk, jejunal and colic veins. Fisher’s exact test was used for statistical analysis.
**Results or Findings:** We included 135 patients (62 males and 73 females; mean age 63) who underwent NAT and PD between January 2013 and December 2021 with an available CT and whose resected specimens were re-evaluated in detail for venous invasion. 65 patients (48%) were subjected to venous resection during PD: 23/65 patients (35%) had ectasia of the gastrocolic trunk, jejunal and colic veins on CT. 20 of these 23 patients with venous ectasia (87%) presented venous neoplastic infiltration at pathology. 70 patients (52%) did not undergo venous resection, including 52 patients without venous ectasia on CT.
**Conclusion:** The presence of venous ectasia correlated with venous neoplastic infiltration at histology. This could help overcome the difficulty of differentiating between fibrosis post-NAT and neoplastic vascular infiltration.
**Limitations:** Retrospective study. Qualitative CT evaluation of venous ectasia.
**Ethics committee approval:** No information provided by the submitter.
**Funding for this study:** No funding was received for this study.
**Author Disclosures:**
Giulia Zamboni: Nothing to disclose
Matilde Bariani: Nothing to disclose
Giancarlo Mansueto: Nothing to disclose
Elisa Boffa: Nothing to disclose
Martina Borzi: Nothing to disclose
Malleo Giuseppe Malleo: Nothing to disclose

### RPS 801-5

**Efficacy, safety and accuracy of image-guided percutaneous biopsy of pancreatic masses: experience in a tertiary cancer centre**

*B. K. Choudhury*; Guwahati/IN

**Purpose:** The purpose of this study was to evaluate the efficacy, safety, and diagnostic accuracy of computed tomography (CT) and ultrasound (US) guided biopsy in pancreatic masses.
**Methods or Backround:** It was a retrospective study of patients referred for image-guided percutaneous biopsy of pancreatic masses at our hospital from January 2017 to December 2021. CT or US-guided core biopsy or fine needle aspiration cytology (FNAC) or both were performed in 55 patients having pancreatic masses based on CT and US image findings. FNAC was performed using a spinal or Chiba needle ( 20/22 G ). Core biopsy was performed using a coaxial system. A 17 or 19-gauge guiding needle was used, and an 18 or 20-gauge spring-loaded semiautomatic core biopsy gun was used to obtain adequate samples.
**Results or Findings:** A total of 55 patients underwent image guided pancreatic biopsy using CT or US as image guidance. US guidance was used in 20 cases (36.4%), and CT guidance was used in the rest 35 cases (63.6%). The mean pancreatic size was 3.1 cm. Majority of the lesions were located in the head (58.2%), followed by the body (23.6 %) and tail (18.2 %). Direct access was performed in 32 cases (58.2 %), and in the rest 23 cases (41.8 %) indirect approach (mostly trasgastric or transhepatic) was used. Only FNAC was performed in 15 cases (27.3%), and both core biopsy with FNAC were obtained in 40 cases (72.7%). A conclusive result was obtained in 49 cases (89%). There were no major complications in our study.
**Conclusion:** CT or US-guided percutaneous needle biopsies of pancreatic masses are safe and highly effective diagnostic techniques with excellent accuracy.
**Limitations:** Single center retrospective study with limited number (55) of patients.
**Ethics committee approval:** No information provided by the submitter.
**Funding for this study:** No funding was received for this study.
**Author Disclosures:**
Binoy Kumar Choudhury: Nothing to disclose

### RPS 801-6

**Comparison of radiomics and deep learning signature for the lymph node metastasis detection in pancreatic neuroendocrine tumour**

*W. Tang*[1], W. Gu[2]; [1]Shanghai/CN, [2]Tsukuba/JP

**Purpose:** To develop and compare the novel signatures, which are based on the radiomics and deep learning features for prediction of the lymph node metastasis (LNM) in pancreatic neuroendocrine tumour (pNET).
**Methods or Backround:** PNETs is a rare pancreatic tumour, and the accuracy of diagnostic for LNM is low. Therefore, the quantification of the radiomics and deep learning features (DLF) may help to elevate the accuracy of detection of the LNM. 220 patients from Fudan University Shanghai Cancer centre were enrolled. All the patients have enhanced Multislice computer tomography (MSCT) imaging and surgical and pathological information. The segmentation of tumours was in the arterial phase of CT imaging by two senior radiologists. The DLF was extracted from the DensenNet201 architecture based on deep convolutional neural networks. The weighted gene co-expression network analysis was used to select the features related to LNM. Then the least absolute shrinkage and selection operator method was used to determine the final features. Finally, the novel signature was constructed by the lightGMB. The clinical factors selection and model construction were based on univariate and multivariate logistic regression. The performance evaluation is based on the ROC curve.
**Results or Findings:** In the radiomics signature, the training and validation group show AUC of 0.89 and 0.81, respectively. The deep learning signature shows an AUC of 0.75 and 0.81, respectively. It is not significantly different between the radiomics features and deep learning features. However, the clinical model shows the lowest prediction as a 0.68 AUC value.
**Conclusion:** Both radiomics and deep learning signatures show the good prediction capability for detection the LMN.
**Limitations:** The combination of radiomics and deep learning signature may elevate the prediction of LNM in pNET. The multi-centre validation is necessary to further confirm the results.
**Ethics committee approval:** This retrospective study was approved by the Institutional Review Broad of Fudan University Shanghai Cancer Centre, and written informed consent was waived.
**Funding for this study:** No funding was received for this study.
**Author Disclosures:**
Wei Tang: Nothing to disclose
Wenchao Gu: Nothing to disclose

### RPS 801-7

**Comparison of the severity between recurrent acute pancreatitis and initial acute pancreatitis on Computed Tomography**

*J. Zhang*[1], H. Pu[1], X. M. Zhang[2]; [1]Chengdu/CN, [2]Nanchang/CN
*(zhangjuradiology@163.com)*

**Purpose:** To compare the severity of recurrent acute pancreatitis (RAP) with that of initial acute pancreatitis (AP) on CT in the early and late phases.
**Methods or Backround:** RAP and AP data were obtained over the past 5 years. The patients were categorised into RAP and AP groups based on recurrence and initial events. Both the RAP and AP groups were divided into early (first week) and late stages (after the first week) based on 2012 revised Atlanta classification (RAC). Patient demographic data, RAC, CT findings, CT severity index (CTSI) scores, and extrapancreatic inflammation on CT (EPIC) scores in the early and late phases were analysed between the two groups.
**Results or Findings:** In 683 RAP and 1831 AP patients, the most common etiologies were hypertriglyceridemia and cholelithiasis, respectively. Among the 683 patients with RAP, interstitial edematous pancreatitis (IEP) and necrotizing pancreatitis (NP) accounted for 62.23% (425/683) and 37.78% (258/683), respectively. Among the 1831 patients with AP, IEP and NP accounted for 64.66% (1184/1831) and 35.34% (647/1831), respectively. The RAP group had a higher proportion of NP than the AP group in both the early and late stages (both P=0.001). The RAP group had lower EPIC scores and APACHE Ⅱ scores than the AP group in the early phase (both P<0.005). The RAP group had higher CTSI scores than the AP group in the late stage (P<0.005).
**Conclusion:** The severity of RAP was lower than that of initial AP in the early stage and higher than that of initial AP in the late stage.
**Limitations:** No limitations were identified.
**Ethics committee approval:** This study was approved by the institutional review board of Affiliated Hospital of North Sichuan Medical College.
**Funding for this study:** The National Natural Science Foundation of China (Grant No. 81871440).
**Author Disclosures:**
Xiao M. Zhang: Nothing to disclose
Hong Pu: Nothing to disclose
Ju Zhang: Nothing to disclose

### RPS 801-8

**Collaborative efforts of radiologist and clinician in predicting prognosis of acute pancreatitis using revised Atlanta classification**

*V. Singh*[1], A. Jain[1], S. Tapadia[2], B. N. Lakhkar[2], A. K. Sahu[1]; [1]New Delhi/IN, [2]Sawangi/IN

**Purpose:** Prognostication of Acute pancreatitis (AP) is crucial for accurate planning and management for better outcomes, thus reducing morbidity, mortality, need for ICU stay, and healthcare costs. The Revised Atlanta's classification (RAC) predicts the severity of the patient using local complications and organ failure as tools. The severity ranges from self-limiting states (70-80%) to life-threatening conditions (20-30%), which ultimately require intervention. This classification is based on close coordination between the radiologist and the treating physician.
**Methods or Backround:** During the study period of 3 years, a total of 140 patients with a clinical diagnosis of AP underwent a contrast-enhanced computer-tomography scan to evaluate the disease process. These patients were followed up to assess for the presence/absence of organ failure. Severity stratification was done using RAC, and comparisons were drawn between severity, local complications, hospital/ICU stay and treatment plan.
**Results or Findings:** Among the 140 patients studied, 71.43% had interstitial edematous pancreatitis, and 28.57% had necrotising pancreatitis. Severity was as follows-mild pancreatitis (31.43%), moderately severe (51.43%), and severe in (17.43%) of the patients. Mean (hospital/ICU) stays were the highest in severe cases (13/6.75 days) and lowest in mild cases (4.5/0 days). Striking statistical significance (p<0.001) was seen between the need for ICU admission and the local fluid collection, 81.82 % of the patients with necrotic collection, 71.43% of patients with walled-off necrosis,28.95% of patients with acute peri-pancreatic collection and only 12.50% of patients with pseudocyst needed ICU admissions. The ultimate prognosis was assessed by Mortality, seen in 6 (4.29%) patients, all of which had severe pancreatitis with necrosis. Surgery was conducted in 12.6%, minimally-invasive procedures in 28.5%, and conservative treatment in 58.9% of the patients.
**Conclusion:** Collaborative efforts between treating physicians and radiologists improve prognostication and management planning of the patients using RAC.
**Limitations:** Follow-up imaging was not done to see the outcomes of fluid collection, as the study was based in a rural tertiary care centre.
**Ethics committee approval:** This study was approved by the institutional ethics committee of Datta meghe institute of medical sciences, wardha , Maharashtra , India
**Funding for this study:** No funding was received for this study.

**Author Disclosures:**
Abhinav Jain: Nothing to disclose
Bhushan N Lakhkar: Nothing to disclose
Varun Singh: Nothing to disclose
Shreya Tapadia: Nothing to disclose
Amit Kumar Sahu: Nothing to disclose

### RPS 801-9

**Can preoperative CT scan and bioimpedance vector analysis help to predict the development of postoperative pancreatic fistula?**

*C. Maino*, R. Corso, C. R. G. L. O. M. Talei Franzesi, D. G. Gandola, D. Ippolito; Monza/IT

**Purpose:** To evaluate the accuracy of computed tomography (CT) and bioimpedance vector analysis (BIVA) in predicting pancreatic steatosis (PS) and development of postoperative pancreatic fistula (POPF).
**Methods or Backround:** A total of 75 patients who underwent pancreatic resection having preoperative CT staging for pancreatic cancer were prospectively enrolled. All the unenhanced CT phases were analysed to determine the overall mean attenuation value of the pancreas, expressed as Hounsfield unit (HU), excluding focal lesions. A radiologist drew three different regions of interest (ROI), located in the head, body, and tail, to calculate the mean attenuation value. Histological data were used as a standard of reference. BIVA was performed the day before surgery, and pancreatic steatosis was assessed by calculating the fat mass index (FMI). Spearman correlation and ROC analysis were used to analyse and compare the different techniques with pathological specimens.
**Results or Findings:** The mean preoperative computed tomography pancreatic attenuation value was 18 (-3-39), and the mean FMI was 7.2 (3.4-11). Positive linear correlations were found between mean HU value and FMI when compared to histologic data (r= -0,852, p<0.001 and r= 0.652, p<0.001, respectively), and a good correlation was found between HU value and FMI (r= -0,659, p<0.001). All methods reported a good diagnostic accuracy in determining POPF, resulting in an AUC of 0.924 (95%CI: 0.844-1), 0.884 (95%CI: 0.778-0.990), and 0.942 (95%CI: 0.879-1) for mean HU value, FMI, and histology respectively.
**Conclusion:** Pre-operative fat mass evaluation by mean of unenhanced CT attenuation value and BIVA can be considered good predictors of POPF and also a reliable approach to quantify the pancreatic steatosis.
**Limitations:** Retrospective, single-center.
**Ethics committee approval:** Not applicable.
**Funding for this study:** No funding was received for this study.
**Author Disclosures:**
Davide Giacomo Gandola: Nothing to disclose
Cesare Maino: Nothing to disclose
Cammillo Roberto Giovanni Leopoldo Oreste Massimiliano Talei Franzesi: Nothing to disclose
Rocco Corso: Nothing to disclose
Davide Ippolito: Nothing to disclose

### RPS 801-10

**Correlation between visceral fat volume, sarcopenia and post-operative hyperamylasemia in patients who underwent major pancreatectomy**

*M. Brotto*, M. Bariani, M. Borzi, G. Marchegiani, G. Zamboni, G. Mansueto; Verona/IT

**Purpose:** Post-operative hyperamylasemia (POH) >48 h is one diagnostic criterion for post-pancreatectomy acute pancreatitis (PPAP), according to the ISGPS. Our purpose was to analyse the correlations between visceral obesity, sarcopenia, and POH, to evaluate if these parameters can predict PPAP development after a major pancreatectomy.
**Methods or Backround:** Informed consent for the utilisation of clinical and radiologic data was provided by all patients (PAD-R registry, n1101CESC). We selected 30 patients who underwent major pancreatectomy at the Verona Pancreas Institute in 2022 and had a CT scan between postoperative day (POD) 3 and 15. We divided the patients into 2 groups according to amylase values: group 1=normal values or POH<48 h; group 2= POH>48 h. We used commercially available software (Syngo.via Siemens) to segment visceral fat, paraspinal and psoas muscles at the umbilical level. For each, we logged volume (cm3) and mean density values (HU). Quantitative variables were described as mean and compared by Student's t-test. p values <0.05 were considered statistically significant.
**Results or Findings:** We enrolled 30 patients, 16 M and 14 F, mean age 56,7. Group 1 had visceral fat volume (VFV) of 15.4 cm3, with mean density (MD) values of -82.6 HU; muscles had volume of 8,2 cm3 and MD of 22.1 HU. Group 2 had VFV of 13.4, with MD of -83.0; muscles had volume of 7.4 cm3 and MD of 21 HU. No statistically significant differences were found comparing the findings in the 2 groups.
**Conclusion:** In our small series, markers of visceral obesity and sarcopenia do not appear to be correlated to persistent POH and, therefore, to the risk of PPAP.
**Limitations:** Retrospective, single-centre study. Preliminary evaluation to test the correlation between the variables in a small number of patients.
**Ethics committee approval:** Informed consent for the use of clinical and radiological data was provided by all patients (PAD_R registry, n1101CESC).
**Funding for this study:** No funding was received for this study.
**Author Disclosures:**
Giulia Zamboni: Nothing to disclose
Matilde Bariani: Nothing to disclose
Giancarlo Mansueto: Nothing to disclose
Marta Brotto: Nothing to disclose
Martina Borzi: Nothing to disclose
Giovanni Marchegiani: Nothing to disclose

**10:30-12:00** **Research Stage 2**

### Research Presentation Session: Breast

### RPS 802

### Image-based assessment in primary systemic treatment in breast cancer

**Moderator**
F. Kilburn-Toppin; Cambridge/UK

### RPS 802-2

**Imaging and pathologic complete response after neoadjuvant therapy in breast cancer: are we ready to avoid surgery? Results from a prospective single-institution study**

*F. Pesapane*[1], V. Dominelli[1], E. M. C. Rossi[1], C. Trentin[1], A. Rotili[1], V. Sacchini[2], V. Galimberti[1], P. Veronesi[1], E. Cassano[1]; [1]Milan/IT, [2]New York, NY/US
*(Filippopesapane@gmail.com)*

**Purpose:** Image-guided vacuum-assisted breast biopsy (VABB) of the tumour bed, performed after neoadjuvant therapy (NAT), is increasingly used to assess residual cancer and to potentially identify pathologic complete response. We assessed the concordance between presurgical VABB specimens and surgical specimens in patients with triple-negative or HER2+ invasive ductal breast cancer who had imaging complete response (iCR) after NAT.
**Methods or Backround:** This single-institution prospective pilot study enrolled patients with breast cancer who were treated with NAT from 04/2018 to 04/2021, had iCR after NAT and underwent image-guided VABB before surgery. We assessed the diagnostic accuracy of image-guided VABB in patients who had iCR on breast MRI and PET/CT. Final surgical pathologic findings served as the reference standard.
**Results or Findings:** Data on 22 patients were analysed. The median (range) age was 44 (36-51) years. There was concordance between the VABB and surgical specimens for 21 of 22 patients, equating to an accuracy of 95% (95%CI,77%-100%); there were no false-negative results. Accuracy was similar between MRI and PET (81% vs 82%, p=0.76).
**Conclusion:** Ultrasound-guided VABB of the residual lesions and tumour bed, combined with breast MRI and PET/CT, performed after NAT may identify candidates for de-escalation of therapy, avoiding potential overtreatment. Our results from patients with triple-negative or HER2+ breast cancer support this approach and should inform the design of future de-escalation trials in NAT responders.
**Limitations:** In addition to the single-centre nature of the study, other limitations include the population with only patients with triple-negative or HER2+, who account for only a quarter of patients with breast cancer, and that image-guided biopsy to assess the presence of residual disease is not routinely performed and is not included in standard breast cancer management pathways.
**Ethics committee approval:** This study was approved by R717/18-IEO 758.
**Funding for this study:** No funding was received for this study.
**Author Disclosures:**
Chiara Trentin: Nothing to disclose
Anna Rotili: Nothing to disclose
Virgilio Sacchini: Nothing to disclose
Valeria Dominelli: Nothing to disclose
Filippo Pesapane: Nothing to disclose
Elisabetta Maria Cristina Rossi: Nothing to disclose
Enrico Cassano: Nothing to disclose
Viviana Galimberti: Nothing to disclose
Paolo Veronesi: Nothing to disclose

### RPS 802-3

**Prognostic value of achieving metabolic complete response of the primary tumour on dedicated breast 18F-FDG PET/MRI post-neoadjuvant chemo(targeted) therapy in breast cancer patients**

*M. Lenaerts*[1], C. de Mooij[1], M. Panosetti[1], J. B. Houwers[1], C. Mitea[1], M. Lobbes[2], K. Keymeulen[1], M. Smidt[1], T. van Nijnatten[1]; [1]Maastricht/NL, [2]Sittard-Geleen/NL
*(melissa.lenaerts@maastrichtuniversity.nl)*

**Purpose:** To investigate the prognostic difference in terms of disease-free (DFS) and overall survival (OS) of breast cancer patients treated with neoadjuvant chemo(targeted) therapy (NCT) who achieved metabolic (mCR) complete response of the primary tumour on dedicated breast 18F-FDG PET/MRI as imaging modality post-NCT.
**Methods or Backround:** Between 2015-2017 patients with a recent breast cancer diagnosis underwent dedicated breast 18F-FDG PET/MRI post-NCT to evaluate response assessment. All breast 18F-FDG PET/MRI was evaluated to evaluate whether patients achieved mCR as opposed to non-mCR post-NCT. Follow-up data per patient were collected until October 1st 2022, to determine disease-free (DFS) and overall survival (OS). Regarding DFS, an event was defined as any locoregional, contralateral or metastatic recurrence. Kaplan Meier curves, including log-rank tests, provided information on DFS and OS, respectively.
**Results or Findings:** A total of 42 consecutive patients underwent dedicated breast 18F-FDG PET/MRI post-NCT for response assessment between 2015-2017. The mean follow-up time was 5.3 years for DFS and 5.8 years for OS. Regarding DFS, achieving mCR as opposed to non-mCR at dedicated breast 18F-FDG PET/MRI post-NCT significantly improved DFS (5.9 versus 4.8 years, p=0.021). Regarding OS, achieving mCR as opposed to non-mCR at dedicated breast 18F-FDG PET/MRI post-NCT significantly improved OS (6.0 versus 5.5 years, p=0.040).
**Conclusion:** Achieving complete metabolic response of the primary tumour on dedicated breast 18F-FDG PET/MRI post-neoadjuvant chemo(targeted) therapy in breast cancer patients seems prognostically relevant as an additional tool.
**Limitations:** Limited sample size of only 42 cases with a follow-up time of approximately 5.5 years.
**Ethics committee approval:** The necessity to obtain informed consent was waived by the medical ethical committee.
**Funding for this study:** M. Lenaerts received salary from "Kankeronderzoeksfonds" Limburg.
**Author Disclosures:**
Marjolein Smidt: Nothing to disclose
Thiemo van Nijnatten: Nothing to disclose
Cornelis de Mooij: Nothing to disclose
Kristien Keymeulen: Nothing to disclose
Marc Lobbes: Nothing to disclose
Cristina Mitea: Nothing to disclose
Melissa Lenaerts: Nothing to disclose
Janneke B Houwers: Nothing to disclose
Marco Panosetti: Nothing to disclose

### RPS 802-4

**Percentage functional tumour volume on pretreatment MRI within triple-negative breast cancer predicts pathologic complete response to combination neoadjuvant immunotherapy and chemotherapy**

*R. J. Weinfurtner*, M. Abdalah, O. Stringfield, N. Khakpour, M. Rosa, B. Czerniecki, H. Soliman, N. Raghunand; Tampa, FL/US
*(Tigerphage@yahoo.com)*

**Purpose:** To determine if functional tumour volume (FTV) analysis of triple-negative breast cancer (TNBC) on pre-treatment MRI can predict pathologic complete response (pCR) in patients treated with Talimogene Laherparepvec (TVEC) neoadjuvant immunotherapy followed by neoadjuvant chemotherapy (NAI/NAC).
**Methods or Backround:** Patients with TNBC in this pilot phase II trial underwent pretreatment MRI followed by ultrasound-guided intratumoral TVEC injections and then neoadjuvant chemotherapy prior to surgery. FTV was calculated on post-contrast T1-weighted images using a 70% enhancement threshold and signal enhancement ratio set to 0. The percentage of FTV within the segmented tumour was then calculated (%FTV = FT voxels / segmented tumour voxels) and correlated with pathologic response at the surgery. pCR was defined as no residual invasive malignancy in the breast or axillary lymph nodes. Statistical analyses were performed using unpaired t-tests, with $p<0.05$ considered statistically significant.
**Results or Findings:** Thirty-one patients were included in the study, and 15 (48%) achieved pCR. Patients achieving pCR had higher %FTV than those with partial pathologic response (pPR) (87% vs 68%, respectively, p=0.0002). Indeed, in the pCR group, all but one tumour demonstrated ≥85% FTV (13/14, 93%). Contrast this with only 4/16 (25%) patients in the pPR group showing %FTV ≥85%. If this is used as a diagnostic test where %FTV ≥85% positively predicts pCR, accuracy is 83% (95% Confidence Interval of 65-94%) with a sensitivity of 93%, specificity of 75%, PPV of 76%, and NPV 92%.
**Conclusion:** In this pilot study of combination NAI/NAC treatment for TNBC, tumours with ≥85% FTV on pre-treatment MRI demonstrated a more favourable response to treatment, achieving pCR in 76%. Conversely, only 2/14 (14%) tumours with <85% FTV achieved pCR, and this could be used to predict response to NAI/NAC in future studies.
**Limitations:** Pilot study and the sample size.
**Ethics committee approval:** This study was approved by Institutional Review Board.
**Funding for this study:** Funding was received from Moffitt Cancer Center.
**Author Disclosures:**
Robert Jared Weinfurtner: Nothing to disclose
Mahmoud Abdalah: Nothing to disclose
Hatem Soliman: Nothing to disclose
Brian Czerniecki: Nothing to disclose
Nazanin Khakpour: Nothing to disclose
Marilin Rosa: Nothing to disclose
Olya Stringfield: Nothing to disclose
Natarajan Raghunand: Nothing to disclose

### RPS 802-5

**MRI accurately predicts pCR in patients with HER2+ breast cancer treated with neoadjuvant immunotherapy followed by neoadjuvant chemotherapy**

*R. J. Weinfurtner*, M. Patel, J. Patel, N. Khakpour, H. Han, B. Czerniecki; Tampa, FL/US
*(Tigerphage@yahoo.com)*

**Purpose:** The purpose of this study was to correlate MRI and pathologic response in patients with HER2+ breast cancer treated with combination neoadjuvant immunotherapy/chemotherapy (NAI/NAC).
**Methods or Backround:** An IRB-approved retrospective analysis was performed on patients in two pilot NAI/NAC HER2+ breast cancer treatment studies using 1) subcutaneous interferon gamma (IFN-γ) and NAC or 2) HER2-pulsed dendritic cell vaccines (DC1) injected intratumorally/intranodally and NAC. Baseline and post neoadjuvant MRIs were reviewed to determine complete vs partial response to therapy (mCR vs mPR) and compared to pathologic complete vs partial response (pCR vs pPR), with pCR defined as no residual invasive tumour in the breast or axilla.
**Results or Findings:** Forty-five female patients were included, with a mean age of 56 (range 22-74). Most pretreatment MRIs demonstrated a dominate mass (39/45, 87%), with the remaining demonstrating non-mass enhancement (NME) only (6/45, 13%). Some patients presenting with masses also had satellite lesions (18/39) or associated NME (8/39). Prior to treatment, 36/45 patients (80%) had biopsy-proven axillary lymph node (LN) metastases. The average longest diameter of the dominant breast finding was 43mm (range 14-140mm). Post-treatment MRIs demonstrated mCR in 34/45 patients (76%). Surgical pathology yielded pCR in 27/45 (60%), without a significant difference between the IFN-γ (13/25, 52%) and DC1 studies (14/20, 70%, p=0.22). As a diagnostic test, mCR corresponded to pCR with an accuracy of 80% (65-90% confidence interval), a sensitivity of 96%, a specificity of 56%, a positive predictive value (PPV) of 76%, and a negative predictive value (NPV) 91%.
**Conclusion:** Post-NAI/NAC pre-surgical MRI in HER2+ breast cancer patients demonstrated reasonable accuracy in predicting pCR with high sensitivity and NPV, despite the potential for interpretation complications with the addition of immunotherapy.
**Limitations:** Pilot study and the sample size.
**Ethics committee approval:** This study was approved by Institutional Review Board.
**Funding for this study:** No funding was received for this study.
**Author Disclosures:**
Maulik Patel: Nothing to disclose
Robert Jared Weinfurtner: Nothing to disclose
Brian Czerniecki: Nothing to disclose
Nazanin Khakpour: Nothing to disclose
Hyo Han: Nothing to disclose
Jay Patel: Nothing to disclose

### RPS 802-6

**Positron emission mammography (PEM) evaluation in the assessment of response to neoadjuvant chemotherapy and prediction of residual disease in breast cancer**

A. F. Moustafa, *Y. M. Nada*, R. M. Kamal, E. Faker, M. Hassan, M. M. Gomaa; Cairo/EG

**Purpose:** The aim of the study is to evaluate the response to neoadjuvant chemotherapy (NAC) and the prediction of residual disease in breast cancer using positron emission mammography (PEM).
**Methods or Backround:** Thirty-six breast cancer patients scheduled for receiving NAC were included. All patients underwent two PEM examinations: pre and post-NAC, to assess the response to neoadjuvant chemotherapy. The PUV max and lesion to background (LTB) ratio are recorded from the mass of concern pre and post-neoadjuvant chemotherapy. Assessment of the response to chemotherapy by measuring the percentage of change in the FDG uptake pre and post-NAC with an evaluation of the residual viable tumoral tissue. In addition to a subjective visual evaluation, results were then correlated with the postoperative pathology evaluation using Miller–Payne grades. For statistical evaluation, patients were classified into responders and non-responders.
**Results or Findings:** According to the Miller–Payne criteria, 36/38 (94.7%) of the participants were responders (Miller–Payne grades III, IV, and V) and 2/38 (5.3%) were non-responders (Miller–Payne grades I and II). Out of the 36 responder cases, 11 were complete response (V), and 25 were partial response (III and IV). The diagnostic performance of PEM for assessment of partial response showing sensitivity, specificity, and positive and negative predictive values 96.00%, 100.00%, 100.00% and 91.67% with an accuracy of 97.22%. The diagnostic performance of PEM for assessment of complete response showing sensitivity, specificity, and positive and negative predictive values of 100%, 96%, 91.67% and 100.00% with an accuracy of 97.22%.
**Conclusion:** Positron emission mammography (PEM) allows accurate evaluation of the response of breast cancer to chemotherapy and residual viable tumoral tissue.
**Limitations:** No limitations were identified.
**Ethics committee approval:** No information provided by the submitter.
**Funding for this study:** No funding was received for this study.
**Author Disclosures:**
Mohammed Mohamed Gomaa: Nothing to disclose
Maher Hassan: Nothing to disclose
Yassmin Mohamed Nada: Nothing to disclose
Eman Faker: Nothing to disclose
Amr Farouk Moustafa: Nothing to disclose
Rasha Mohamed Kamal: Nothing to disclose

### RPS 802-7

**Neoadjuvant chemotherapy monitoring with contrast-enhanced ultrasound in patients with breast cancer**

*I. Biondić Špoljar*, G. Ivanac, E. Divjak, B. Brkljačić; Zagreb/HR

**Purpose:** Neoadjuvant chemotherapy is a treatment of choice in patients with certain subtypes of breast cancer or when cancer is not operable due to size or locally advanced spreading, with the aim of making it operable. When the complete pathologic response is achieved after the therapy, studies have shown that long-term survival increases. The golden standard imaging modality for NACT follow-up is magnetic resonance imaging. In this study, we followed patients on NACT with MRI and CEUS and compared the responses of both methods.
**Methods or Backround:** For the CEUS examination, an ultrasound-specific contrast agent was used. It is a liquid suspension of microbubbles containing the gas sulfur-hexafluoride in a phospholipid envelope, which remains in blood vessels only. After the CEUS examination, post-processing was performed to generate kinetic curves. Maximal intensity enhancement and peak time were measured with the region of interest placed inside the lesion. The size and grayscale features were analysed as well. Treatment responses were evaluated based on the RECIST criteria and kinetic parameters of both methods and then compared.
**Results or Findings:** 61 MRI and 61 CEUS follow-up examinations in 37 patients were analysed. The results showed high matching of the responses to the therapy with both methods. Cohen's kappa coefficient was calculated at 0.66, suggesting a high methods agreement. Kendall's Tau was calculated at 0.67, also pointing to a strong relationship.
**Conclusion:** The conclusion is that CEUS is a valuable method for breast cancer treatment follow-up, especially in patients with contraindications for MRI or where MRI is not available. CEUS examination is more pleasant for the patients, takes less time, and there are also less side effects from an ultrasound contrast agent.
**Limitations:** No limitations were identified.
**Ethics committee approval:** No information provided by the submitter.
**Funding for this study:** No funding was received for this study.
**Author Disclosures:**
Boris Brkljačić: Nothing to disclose
Iva Biondić Špoljar: Nothing to disclose
Gordana Ivanac: Nothing to disclose
Eugen Divjak: Nothing to disclose

### RPS 802-8

**The use of targeted axillary dissection after neoadjuvant treatment in breast cancer patients with axillary lymph node involvement**

*L. Bell*, A. Mariscal, A. P. Salazar, M. D. L. O. Perez Molina, A. Catanese, M. Negrini, J. C. Gómez Nova, A. Martínez Antón, M. Gener González; Barcelona/ES

**Purpose:** To evaluate the role of targeted axillary dissection (TAD) as a predictor of axillary lymph node involvement in N+ breast cancer patients after neoadjuvant treatment.
**Methods or Backround:** A prospective study was carried out that included patients with breast cancer and axillary lymph node involvement (N1). The most suspicious lymph node, according to the Bedi classification, was marked using a metallic marker. After receiving neoadjuvant chemotherapy, a magnetic seed (Magseed) was placed in the marked lymph node, and an axillary lymphadenectomy was performed. We reviewed the ultrasound features of the marked lymph nodes, the amount of magnetic seeds recovered after TAD, the anatomopathology result of the sentinel lymph node, the marked lymph node and lymphadenectomy, and calculated the percentage of false negative results.
**Results or Findings:** A total of 60 patients were included, with a mean age of 53.4 years (range 25 - 78). A false negative result was present in 8/37 (21.62 %) patients when evaluating the sentinel lymph node alone and 2/28 (7.14%) when combined with the marked lymph node (TAD). Metallic markers were identified on post-chemotherapy ultrasound in all patients, and the magnetic seed was recovered in all patients except one (intraoperative loss).
**Conclusion:** TAD is a reliable predictor of lymph node involvement in patients with breast cancer with axillary nodal metastasis that have received neoadjuvant treatment. TAD can therefore avoid axillary lymph node dissection and its associated morbidity in patients with good response to chemotherapy treatment.
**Limitations:** Large multicentre studies are needed to further evaluate the role of TAD as an alternative to axillary lymph node dissection. Difficulties in the ultrasonographic evaluation of lymph nodes after neoadjuvant treatment can limit whether patients would benefit from a TAD or an axillary lymph node dissection.
**Ethics committee approval:** No information provided by the submitter.
**Funding for this study:** No funding was received for this study.
**Author Disclosures:**
Angela Martínez Antón: Nothing to disclose
Miquel Gener González: Nothing to disclose
Jean Carlos Gómez Nova: Nothing to disclose
Angela Patricia Salazar: Nothing to disclose
Antonio Mariscal: Nothing to disclose
Mario Negrini: Nothing to disclose
Alessandro Catanese: Nothing to disclose
Liam Bell: Nothing to disclose
Maria De Las Olas Perez Molina: Nothing to disclose

### RPS 802-9

**Implementation of the MARI treatment protocol in clinically node-positive breast cancer patients: correlation with axillary ultrasound and 18F-FDG PET/CT nodal findings**

*T. van Nijnatten*[1], A. de Bruijn[2], M. van der Sangen[2], B. Vriens[2], E. Degreef[2], M. Roef[2], M. Gielens[2], A. Voogd[1], R-J. Schipper[2]; [1]Maastricht/NL, [2]Eindhoven/NL
*(Thiemo.nijnatten@mumc.nl)*

**Purpose:** The primary aim of the present study was to evaluate the implementation of the MARI (i.e. marking axillary lymph node with radioactive iodine seeds) treatment protocol for nodal restaging after neoadjuvant systemic therapy in clinically node-positive breast cancer patients in an external hospital. The secondary aim was to identify opportunities to tailor the protocol based on different intrinsic subtypes of breast cancer and axillary ultrasound findings.
**Methods or Backround:** Patients diagnosed with clinically node-positive breast cancer treated with neoadjuvant systemic therapy between October 2014 and December 2020 were retrospectively included. Data on diagnostic workup, including axillary ultrasound and 18F-FDG PET/CT nodal findings, were analysed. Histopathological results served as the gold standard.
**Results or Findings:** A total of 151 patients were included. The identification rate of the MARI procedure was 97.3%. Axillary pCR after neoadjuvant chemo(+/-target) therapy, according to MARI, was achieved in 40.5% of patients, and 4.0% of patients after neoadjuvant endocrine treatment achieved axillary pCR. According to 18F-FDG PET/CT findings, 66.9% of the patients had 1-3 suspicious axillary lymph nodes, and 33.1% of patients had 4 or more suspicious axillary lymph nodes. Suspicious axillary ultrasound findings among the different subtypes did not correlate with 18F-FDG PET/CT nodal findings regarding the differentiation of 1-3 versus 4 or more suspicious axillary lymph nodes. There was no association between the extent of nodal disease and the likelihood of achieving axillary pCR (according to MARI): 40.5% vs 40.4% (p=0.993). In 79.4% of all patients, a completion ALND was omitted.
**Conclusion:** The feasibility of implementation of the MARI protocol is validated in an external hospital. Furthermore, categorising patients with 1-3 versus 4 or more suspicious axillary lymph nodes based on axillary ultrasound findings as surrogates for 18F-FDG PET/CT seems not feasible.
**Limitations:** No limitations were identified.
**Ethics committee approval:** The necessity to obtain informed consent was waived by the medical ethical committee.
**Funding for this study:** No funding was received for this study.
**Author Disclosures:**
Maurice van der Sangen: Nothing to disclose
Birgit Vriens: Nothing to disclose

Mark Roef: Nothing to disclose
Robert-Jan Schipper: Nothing to disclose
Maaike Gielens: Nothing to disclose
Adri Voogd: Nothing to disclose
Anne de Bruijn: Nothing to disclose
Ellen Degreef: Nothing to disclose
Thiemo van Nijnatten: Nothing to disclose

**10:30-12:00 Research Stage 3**

### Research Presentation Session: Interventional Radiology

### RPS 809

### Head, neck and thoracic embolisation

**Moderator**
V. Bérczi; Budapest/HU
*(berczi@hotmail.com)*

### RPS 809-2

**A multicentre analysis of percutaneous sclerotherapies in venous malformations of the face**

*V. F. Schmidt*[1], M. Masthoff[2], P. Sporns[3], L. K. Segger[4], M. T-L. Takes[3], J. Ricke[1], B. Gebauer[4], W. A. A. Wohlgemuth[5], M. Wildgruber[1]; [1]Munich/DE, [2]Münster/DE, [3]Basel/CH, [4]Berlin/DE, [5]Halle/DE

**Purpose:** To evaluate the safety and outcome of image-guided sclerotherapy for treating venous malformations (VMs) of the face.
**Methods or Backround:** A multicentre cohort of 68 patients with VMs primarily affecting the face was retrospectively investigated. In total, 142 image-guided sclerotherapies were performed using gelified ethanol and/or polidocanol. Clinical and imaging findings were assessed to evaluate the clinical response, lesion size reduction, and complication rates. Sub-analyses of complication rates depending on the type and injected volume of the sclerosant as well as of paediatric vs adult patient groups were conducted.
**Results or Findings:** The mean number of procedures per patient was 2.1 (±1.7), and the mean follow-up consisted of 8.7 months (±6.8 months). Clinical response (n=58) revealed a partial relief of symptoms in 70.7% (41/58), 13/58 patients (22.4%) presented symptom-free, while only 4/58 patients (6.9%) reported no improvement. Post-treatment imaging (n=52) revealed an overall objective response rate of 86.5% (45/52). The total complication rate was 10.6% (15/142), including 4.2% (7/142) major complications, mostly (14/15, 93.3%) resolved by conservative means. In one case, a mild facial palsy persisted over time. The complication rate in the gelified ethanol subgroup (8/34, 23.5%) was significantly higher (p=0.01) compared to polidocanol (5/84, 6.0%) and to the combination of both sclerosants (2/22, 9.1%) while there were no significant differences in complications between the paediatric and adult subgroup (12.1% vs 9.2%, p=0.57). The clinical response did not correlate with lesion size reduction on MRI.
**Conclusion:** Image-guided sclerotherapy is effective for treating VMs of the face. Clinical response is not necessarily associated with size reduction on imaging. Despite the complex anatomy of this location, the procedures are safe for adults and children.
**Limitations:** This study represents a retrospective design, including a lack of standardized follow-up data for the reported cohort.
**Ethics committee approval:** Local ethics committee (University Hospital, LMU Munich, protocol No.: 21-0943, 10/06/2021).
**Funding for this study:** No funding was received for this study.
**Author Disclosures:**
Laura Katharina Segger: Nothing to disclose
Vanessa Franziska Schmidt: Nothing to disclose
Martin Thanh-Long Takes: Nothing to disclose
Bernhard Gebauer: Nothing to disclose
Peter Sporns: Nothing to disclose
Moritz Wildgruber: Nothing to disclose
Walter A. A. Wohlgemuth: Nothing to disclose
Max Masthoff: Nothing to disclose
Jens Ricke: Nothing to disclose

### RPS 809-3

**Establishment of an interdisciplinary vascular anomalies programme in Tanzania, East Africa**

*D. Puhr-Westerheide*[1], M. Masthoff[2], M. Shemwetta[3], A. Naif[3], O. N. Ukweh[3], E. M. Mbuguje[3], F. Minja[4], F. M. Laage-Gaupp[5], M. Wildgruber[1]; [1]Munich/DE, [2]Münster/DE, [3]Dar es Salaam/TZ, [4]Atlanta, GA/US, [5]New Haven, CT/US

**Purpose:** The aim of this project was the implementation of a sustainable vascular anomalies programme in Tanzania.
**Methods or Backround:** In 2021 the first interdisciplinary vascular anomalies (VA) programme was initiated at Muhimbili National Hospital (MNH). During the planning phase, the clinical need for minimally-invasive therapies of VAs and the preexisting structures were assessed by the local Interventional Radiology (IR) team at MNH. During the initiation phase, an IR team from two German VA centres joined the interdisciplinary team at MNH for clinical workup, image-guided procedures and follow-up. VA patients were recruited from existing patient records or seen at clinics as de-novo presentations following nationwide advertisement. In the post-processing phase, sustainability was ensured using joined online conferences for follow-up and support in the management of new patients. Further follow-up was supported by attending providers from other established VA centres supporting the MNH team in Tanzania.
**Results or Findings:** The first interdisciplinary VA programme was successfully launched in Tanzania. Minimally-invasive treatments were successfully trained by performing ultrasound-guided sclerotherapy with polidocanol and bleomycin in twelve patients with slow-flow malformations, one endovascular embolisation of a high-flow malformation, and medical treatment of an aggressive infantile haemangioma. Regular online follow-up presentations have been initiated. Follow-up evaluation and required treatment were sustained when appropriate.
**Conclusion:** The presented “hands-on” training set the ground for the first interdisciplinary VA programme in Tanzania. This framework is expected to establish comprehensive and sustainable care of patients with VAs in East Africa and can serve as a blueprint for other sites.
**Limitations:** Global outreach project for the establishment of a VA centre - no conventional study setting.
**Ethics committee approval:** No information provided by the submitter.
**Funding for this study:** RSNA Derek Harwood-Nash Grant, Doximity Foundation, Road2IR Foundation.
**Author Disclosures:**
Erick Michael Mbuguje: Nothing to disclose
Fabian M. Laage-Gaupp: Nothing to disclose
Mwivano Shemwetta: Nothing to disclose
Daniel Puhr-Westerheide: Nothing to disclose
Moritz Wildgruber: Nothing to disclose
Azza Naif: Nothing to disclose
Ofonime Nkechinyere Ukweh: Nothing to disclose
Frank Minja: Nothing to disclose
Max Masthoff: Nothing to disclose

### RPS 809-4

**Impact of mechanical thrombectomy on lung perfusion blood volume in acute pulmonary embolism**

S. A. Si-Mohamed, *L. Martineau*, A. Branchu, D. Gamondes, S. Boccalini, V. Cottin, P. Douek, D. Revel, L. Boussel; Lyon/FR

**Purpose:** Mechanical thrombectomy has been recently proposed as an alternative or complementary approach for relieving the right ventricle overload in acute pulmonary embolism (PE). The objective of this study was to compare the quantitative lung perfusion blood volume (PBV) acquired with a dual-layer dual-energy CT system (DECT) before and after percutaneous mechanical thrombectomy using the FlowTriever System in patients with acute PE.
**Methods or Backround:** Consecutive patients with acute PE who underwent DECT angiography immediately before and after FlowTriever thrombectomy in an expert centre were identified retrospectively. Normalized quantitative PBV, RV/LV ratio and Qanadli score were calculated and compared using the Wilcoxon rank sum test.
**Results or Findings:** Six patients (2 women, 68 ± 8.15 (SD) years [range: 60–81 years]) were identified retrospectively from February through September 2022. Thrombectomy was performed on average 23.2 hours after admission. All patients received anticoagulants and no fibrinolytic. Quantitative PBV was significantly different and 33.3 ± 34.4% higher after thrombectomy (5.9% ± 1.4 (1QR–3QR: 4.4–7.0) vs 8.1% ± 3.8 (1QR–3QR: 5.2–10.4), P=0.03). Qanadli score and RV/LV ratio were significantly lower (P<0.05) after thrombectomy, with an average reduction of 39.2% and 18.1%, respectively.
**Conclusion:** Percutaneous mechanical thrombectomy with the FlowTriever System enables immediate and significant improvement in lung perfusion in patients with acute intermediate-risk PE.
**Limitations:** Our study has several limitations, including the small number of patients and the retrospective design resulting in missing data. Further, the management of patients eligible for thrombectomy is not yet standardized, especially with regard to the performance of CT scans before and after thrombectomy.
**Ethics committee approval:** Approval by the local institutional review board (HCL, approval number 18-305).
**Funding for this study:** No funding was received for this study.
**Author Disclosures:**
Salim Aymeric Si-Mohamed: Nothing to disclose
Didier Revel: Nothing to disclose
Delphine Gamondes: Nothing to disclose
Loïc Boussel: Nothing to disclose
Philippe Douek: Nothing to disclose
Sara Boccalini: Nothing to disclose
Vincent Cottin: Nothing to disclose
Lorraine Martineau: Nothing to disclose
Arthur Branchu: Nothing to disclose

### RPS 809-5

**Flow diverter stents in the treatment of acutely ruptured aneurysms using single antiplatelet therapy: a retrospective analysis**

G. Guzzardi, *A. Siani*, C. Stanca, M. Spinetta, M. Cernigliaro, M. Sassone, C. Coda, R. Di Fiore, A. Carriero; Novara/IT

**Purpose:** The use of a flow diverter stent (FDS) has limitations in cases of subarachnoid haemorrhage caused by ruptured aneurysm due to the need for double antiplatelet therapy and the delay in the aneurysm occluding. The p48 MW and the p64 MW (Phenox) are available with Hydrophilic Polymer Coating (HPC), which reduces the risk of thrombus formation. The purpose of this study is to evaluate the safety and efficacy of p48 and p64 MW HPC with single antiplatelet therapy for the acute treatment of ruptured aneurysm.
**Methods or Backround:** We retrospectively evaluated all patients treated for acutely ruptured aneurysms, unfit for simple coiling, with a p48 MW HPC or p64 MW HPC from October 2019 to September 2022 using single antiplatelet therapy. In all cases, an aggregometry test was used. For each patient, we considered demographic and aneurysm-related data, clinical presentation, size and location of the implanted flow diverter stent, intra- and post-procedural complications and aneurysm occlusion according to the O'Kelly-Marotta scale (OKM).
**Results or Findings:** Eighteen patients were included (12 w, 6 m). Of the ruptured aneurysms, thirteen were saccular, three blister-like and two dissecting; fifteen were in the anterior and three in the posterior circulation. No intraprocedural stent thrombosis and rebleeding was observed. Adequate occlusion (OKM class C-D) was registered in eight cases. Four patients died of severe vasospasm. No patients needed retreatment in this series.
**Conclusion:** In our experience, FDS HPC appears to be a potential treatment option in selected cases. Our study is limited by a small population and short-term follow-up. We report our preliminary data, but further investigations are necessary.
**Limitations:** Small population and short-term follow-up.
**Ethics committee approval:** No information provided by the submitter.
**Funding for this study:** The author(s) received no financial support for the research, authorship, and/or publication of this article.
**Author Disclosures:**
Marco Spinetta: Nothing to disclose
Carmelo Stanca: Nothing to disclose
Massimiliano Cernigliaro: Nothing to disclose
Giuseppe Guzzardi: Nothing to disclose
Riccardo Di Fiore: Nothing to disclose
Agnese Siani: Nothing to disclose
Alessandro Carriero: Nothing to disclose
Miriana Sassone: Nothing to disclose
Carolina Coda: Nothing to disclose

### RPS 809-6

**Endovascular treatment of Chronic SubDural Haematoma (CSDH): distal embolisation of Middle Meningeal Artery (MMA) with a low viscosity embolic agent**

*C. Sicignano*, V. Piscitelli, F. Fasano, V. D'Agostino, L. Gemini, A. Generali, F. Frio, G. Di Nuzzo, G. Catapano; Naples/IT
*(carmine.sicignano@gmail.com)*

**Purpose:** We report our early experience with distal embolisation of MMA in patients with CSDH.
**Methods or Backround:** From January 2022, we started treating CSDH patients with endovascular embolisation of MMA. According to the neurosurgeons, we selected 15 patients with minimal/moderate neurological deficits or after surgery relapse. We chose to use a low viscosity non-adhesive liquid embolic agent (SQUID 12) to reach distal microvessels of the haematoma capsule and membrane; for the same reason, we preferred distal micro-catheterisation. CT scan controls were performed at about 30 days and 90/180 days; the clinical assessment was performed by a neurosurgeon.
**Results or Findings:** 14/15 patients underwent embolisation; 1 patient was not embolised because of an anatomical variant of the MMA recognised at angiography. 4/14 patients had bilateral CSDH (10/14 unilateral). In total, we embolised 18 MMA. The procedure was performed awake, under minimal conscious analgosedation during the embolisation. Only 1 patient was under general anaesthesia because of their conditions (disabled from birth). Anticoagulant/antiplatelet therapy was not interrupted. No clinical periprocedural complications were observed. 13/14 (93%) patients experienced clinical and radiological improvement at the 1-month control, which was stable or more improved at the 3-6 months controls. We observed a reduction of the thickness of the haematoma from 50% to complete resolution and the progressive realignment of the middle line. The only unresponsive patient had a reduction of thickness inferior to 50%, with mild clinical improvement, but she had a relapse after surgery (double burr-hole) and is affected by hydrocephalus. She is under a longer follow-up as a chronic care model.
**Conclusion:** The embolisation of the MMA for CSDH, with distal catheterisation and using SQUID 12, seems feasible, safe and effective. It may represent an alternative to surgery in selected cases.
**Limitations:** No limitations were identified.
**Ethics committee approval:** No information provided by the submitter.
**Funding for this study:** No funding was received for this study.
**Author Disclosures:**
Giuseppe Di Nuzzo: Nothing to disclose
Laura Gemini: Nothing to disclose
Carmine Sicignano: Nothing to disclose
Ada Generali: Nothing to disclose
Federico Frio: Nothing to disclose
Giuseppe Catapano: Nothing to disclose
Valeria Piscitelli: Nothing to disclose
Vincenzo D'Agostino: Nothing to disclose
Fabrizio Fasano: Nothing to disclose

### RPS 809-7

**Surpass evolve flow diverter in the treatment of intracranial aneurysms: a single centre initial experience study with angiography and clinical results**

*G. Raja*, S. Sekar, J. Ansan, J. Dhanasekaran, S. Joseph, V. Pothula; Chennai/IN
*(gokul91@gmail.com)*

**Purpose:** Flow diverters (FD) have become a safe treatment option for intracranial aneurysms (IA), especially in fusiform and blister-like aneurysms, which remained challenging. Newer generation Surpass Evolve (SE) is an FD that was approved for usage in 2019 in India. The aim of this study is to describe the initial experience and analyse the angiographic and clinical outcomes using SE in the treatment of IA.
**Methods or Backround:** Between December 2019 and July 2022, sixty-three IA in 44 patients (70.5% women, mean age 50 years) were treated with SE in our institute. Baseline patient, aneurysm demographics, and treatments were prospectively collected, and results were reviewed retrospectively.
**Results or Findings:** IA was noted as an incidental finding in 70.5% of patients; the majority (68.2%) were saccular, and 70% wide neck. Single FD was used in 77.3% of patients. Apart from re-sheathing of FD, two patients required angioplasty of deployed FD. Adjuvant coiling was performed in four aneurysms, and two aneurysms required an extra FD to achieve proper wall apposition. The Mean Aneurysm Flow Amplitude (MAFA) ratio obtained in 2 patients showed a superior flow diversion effect of the SE device. There was no thromboembolic complication; however, we had a few early reversible neurological complications and one mortality. Favourable aneurysm occlusion O'Kelly-Marotta (OKM) grading scale D was achieved in 27/33 (81%) follow-up patients. No FD deployment failure, intra-procedural mortality, or increase in the mRS was observed.
**Conclusion:** SE worked technically well in terms of its excellent navigability, re-sheathing, placement accuracy, and maximal flow diversion. Our initial clinical and angiographic results show a good efficacy comparable to other FD.
**Limitations:** This is a small sample size, and long-term clinical outcomes are necessary for evaluating long-term safety and efficacy. The study population was heterogeneous.
**Ethics committee approval:** Sri Ramachandra Institute of Higher Education and Research.
**Funding for this study:** No funding was received for this study.
**Author Disclosures:**
Sabarish Sekar: Nothing to disclose
Venkatesh Pothula: Nothing to disclose
Santhosh Joseph: Nothing to disclose
Gokul Raja: Nothing to disclose
Jagadeesan Dhanasekaran: Nothing to disclose
Joseph Ansan: Nothing to disclose

### RPS 809-8

**Radiological and clinical outcome of using Woven EndoBridge (WEB) device as an endovascular treatment for wide-neck intracranial aneurysms**

*R-E. Chung*, Y. Zaremba, C. K. Jadun, S. Nayak, Z. Hashim; Stoke-on-Trent/UK
*(cruien@gmail.com)*

**Purpose:** The challenges of endovascular treatment of wide-neck intracranial aneurysms have been well recognized. Our objective is to present the clinical and radiological outcomes of using the WEB device for the treatment of wide-necked intracerebral aneurysms.
**Methods or Backround:** Retrospective data collection was performed to identify patients treated with the WEB device between 2017 and 2022 for ruptured and unruptured aneurysms at a tertiary care centre in England.
**Results or Findings:** 39 cases of ruptured (79.5%) and unruptured (20.5%) aneurysms involving WEB embolisation were identified. Mean age of 57.6, with 28 (72%) female and 11 (28%) male patients. Femoral or radial access was performed (64% vs 36%). 42 individual aneurysms (mean neck diameter: 3.5 mm, mean height: 5.26 mm, mean width: 5.42 mm) were identified. The ACA was the most common location (42.9%), followed by the MCA (21.4%), basilar (19%), ICA (7.1%), pericallosal (.8%), PCOM (2.4%) and SCA (1, 2.4%). Successful deployment of the WEB was achieved in all aneurysms (mean procedure length 1 hour 42 minutes). 14 patients were excluded, with 1 lost to follow-up, 6 awaiting follow-up, and 7 passed away (all ruptured presentation). With the available 6-month and 2-year follow-up imaging, 20 (71.4%) aneurysms were occluded, and 8 (28.6%) aneurysms had a residual neck. 1 residual aneurysm was re-treated with a flow-diverting stent with confirmed occlusion on follow-up. The rest of the 7 residual aneurysms were either awaiting treatment or didn’t require further intervention. Upon follow-up, 24 out of 25 patients (96%) were functionally independent (modified Rankin scale ≤2).
**Conclusion:** Our centre’s experience supports the use of the WEB device in treating wide-neck aneurysms with good occlusion rates demonstrated in the acute and elective settings at 6-month and 2-year follow-ups.
**Limitations:** No limitations were identified.
**Ethics committee approval:** No information provided by the submitter.
**Funding for this study:** No funding was received for this study.
**Author Disclosures:**
Zafar Hashim: Nothing to disclose
Rui-En Chung: Nothing to disclose
Sanjeev Nayak: Nothing to disclose
Yasemin Zaremba: Nothing to disclose
Changez Khan Jadun: Nothing to disclose

### RPS 809-9

**Flow diverter treatment of posterior circulation fusiform, saccular and blister-like aneurysms: a single-centre experience of 35 patients**

*V. Garayeva*, S. G. Kandemirli, A. Alakbarov, O. Kızılkılıç, C. Işlak; Istanbul/TR
*(qarayeva_vusala@mail.ru)*

**Purpose:** It is well established that posterior circulation aneurysms have a higher risk of complications after FD treatment. Herein, we evaluated flow-diverting stent therapy's effectiveness and complication rates in posterior circulation aneurysms.
**Methods or Backround:** Our study comprised 35 patients with posterior circulation aneurysms treated with a flow-diverting stent between May 2008 and November 2019 in a single institution. They had at least one year of follow-up. Of the 35 aneurysms, 14 were saccular (40%), 18 were fusiform (51%), and 3 were blister type (9%). Eight aneurysms were treated with SILK stent, 13 aneurysms with Pipeline embolization device, and 14 aneurysms with FRED (Flow Re-direction Endoluminal Device).
**Results or Findings:** Only a single aneurysm was treated with FD in each patient. Total occlusion was observed in 24 (69%) aneurysms after a mean follow-up of 14 months. 51% (18 patients) of aneurysm occlusions occurred in the 0-6th month follow-up period. While there was no mortality (0%), morbidity was observed in 4 (11.4%) patients (mRS 1-4).
**Conclusion:** Despite technical difficulties and complications, flow-diverting stents can be considered an effective endovascular treatment option in treating posterior circulation aneurysms. The probability of total aneurysm sac occlusion increases as time passes after flow-diverting therapy. Our study has documented no recurrence or parent vessel occlusion with this therapeutic procedure, and the results are highly promising.
**Limitations:** The major limitations are retrospective design, small sample size, absence of a control group, and limited experience on the long-term behaviour of the FD stent. Different types and sizes of aneurysm were included in this cohort (i.e., saccular/blister/fusiform/dissecting), and subgroup analyses were not performed for the subset of blister/fusiform/dissecting aneurysms in this study due to the limited number of cases.
**Ethics committee approval:** No information was provided by the submitter.
**Funding for this study:** No funding was received for this study.
**Author Disclosures:**
Osman Kızılkılıç: Consultant: Flow diverter treatment of posterior circulation fusiform, saccular and blister-like aneurysms
Sedat Giray Kandemirli: Consultant: Flow diverter treatment of posterior circulation fusiform, saccular and blister-like aneurysms
Anar Alakbarov: Consultant: Flow diverter treatment of posterior circulation fusiform, saccular and blister-like aneurysms
Civan Işlak: Consultant: Flow diverter treatment of posterior circulation fusiform, saccular and blister-like aneurysms
Vusala Garayeva: Author: Flow diverter treatment of posterior circulation fusiform, saccular and blister-like aneurysms

### RPS 809-10

**Comparison between stent retriever (SR) and direct aspiration (DA) technique in the treatment of acute M2 segment occlusion of middle cerebral artery: a single centre randomized prospective study**

M. Cernigliaro, *D. Negroni*, M. Sassone, C. Coda, C. Stanca, S. M. Tettoni, A. Galbiati, M. Spinetta, G. Guzzardi; Novara/IT
*(dvdngr@gmail.com)*

**Purpose:** The aim of this study was to compare the efficacy of stent retriever (SR) vs direct aspiration (DA) in the treatment of acute M2 segment occlusion of the middle cerebral artery on the thrombolysis in cerebral infarction (TICI) scale.
**Methods or Backround:** All patients presenting with an ischaemic stroke and meeting the inclusion criteria for endovascular therapy were enrolled in the study and assigned to an arm of the study through a pre-randomized database. Demographic and clinical data were collected. TICI score was the primary outcome (successful revascularization if TICI2b or 3). NIHSS pre-treatment and at the discharge, mRS pre-treatment at 90 days were collected.
**Results or Findings:** A total of 36 patients met the inclusion criteria and were randomized into two groups: 19 (52.78%) in the stent retriever (SR) arm and 17 (47.22%) in the direct aspiration (DA) arm. Overall, thrombolysis in the cerebral infarction scale (TICI) 2b-3 was achieved in 25 patients (69,4%). In the SR group, 12 subjects out of 19 (63.16%) had a score of TICI 2b-3; in our direct aspiration (DA) group, 13 out of 17 (76.47%) had a score of TICI 2b-3 (p-value 0.48). There are no statistically significant differences between the NIHSS delta and mRS delta groups.
**Conclusion:** At present, we did not find superiority in vessel recanalization or outcome of either DA or SR, with a slight tendency for better results with DA. Nonetheless, the overall safety and efficacy of M2 treatment in our cohort were in line with the literature.
**Limitations:** This is a study from a single centre, and the sample size is big enough for a preliminary statistical analysis but still small.
**Ethics committee approval:** Comitato Etico Novara.
**Funding for this study:** No funding was received for this study.
**Author Disclosures:**
Serena Maria Tettoni: Nothing to disclose
Marco Spinetta: Nothing to disclose
Davide Negroni: Nothing to disclose
Andrea Galbiati: Nothing to disclose
Carmelo Stanca: Nothing to disclose
Massimiliano Cernigliaro: Nothing to disclose
Giuseppe Guzzardi: Nothing to disclose
Miriana Sassone: Nothing to disclose
Carolina Coda: Nothing to disclose

### RPS 809-11

**Setting up an endovascular intracranial thrombectomy service in a medical centre without neurointerventionists: challenges in building a multidisciplinary team and ensuring quality**

*D. L. Hykkerud*, M. Altmann; Lorenskog/NO
*(dahy@ahus.no)*

**Purpose:** To show the feasibility of establishing an intracranial thrombectomy service and building a multidisciplinary team in a medical centre without neurointerventionists.
**Methods or Backround:** Acute endovascular thrombectomy (ET) has proved to be a safe and highly efficient treatment for ischaemic stroke with large vessel occlusion. Traditional neurointerventional centres are few and may limit the rapid access to mechanical thrombectomy. An ongoing debate is how to train physicians without prior neuroendovascular experience to perform ET. The tools we have been using to meet guidelines and treatment standards are: iterative processes to improve stroke care algorithms, multidisciplinary team simulation training, endovascular simulator training, attending neurothrombectomy theoretical courses, hands-on training observership programme, internal quality assurance processes and adherence to a national quality improvement registry. Our hospital was already an experienced stroke centre with consistent door-to-CT time (mean 10.2min) and door-to-needle time (19.9min, thrombolysis). We aim to show that in an experienced interventional radiology centre, endovascular intracranial thrombectomy start-up is feasible and safe.

**Results or Findings:** After 77 procedures, we registered a TICI score of 94%; ≥2b, 86%; ≥2c and 75%; 3. Median groin to recanalization time improved from 72min to 44min after one year (range 15-170). Door-to-recanalization time: median 124min (range 20-237, all patients), and in 2022 median 113min (range 20-263). Door-to-groin puncture for transfer patients: 34min (all patients). Median NIHSS at the time of admission: 16 (range 4-30), at discharge 4 (range 0-22), 90 days MRS mean 2 (0-6). 90 days MRS 0-2: 50%, 0-3: 71%. Age: 72 years (range 38-97), >80 years: 31%. Thrombolysis in cerebral infarction (TICI), The Modified Rankin Scale (MRS), National Institutes of Health Stroke Scale (NIHSS).
**Conclusion:** Implementing ET as a new procedure in a medical centre without neurointerventional experience is feasible and safe.
**Limitations:** Single centre, retrospective study.
**Ethics committee approval:** No information provided by the submitter.
**Funding for this study:** No funding was received for this study.
**Author Disclosures:**
Dan Levi Hykkerud: Nothing to disclose
Marianne Altmann: Nothing to disclose

### RPS 809-12

**Shape modification of Woven EndoBridge in wide-neck bifurcation aneurysms: 3-year follow-up with digital subtraction angiography**

*D. Negroni*, A. Siani, C. Coda, R. G. Di Fiore, C. Stanca, M. Cernigliaro, G. Guzzardi, A. Carriero, M. Spinetta; Novara/IT
*(dvdngr@gmail.com)*

**Purpose:** The Woven EndoBridge (WEB) device is a self-expanding intrasaccular braided-wire device for the treatment of wide-neck bifurcation aneurysms (WNBAs). A change in the shape of this device has been observed over time. The aim of the study is to describe this phenomenon using long follow-up and digital subtracted angiography.
**Methods or Backround:** All patients treated with WEB due to ruptured WNBAs were subjected to follow-up digital subtraction angiography (DSA) 2 and 3 years after device deployment. The degree of the residual neck was assessed through BOSS, Lubicz, and WEBCAST scales. Data on WEB shape, modified Rankin scale (mRS), bleeding events, and ischaemic events occurring during this time period were collected as well.
**Results or Findings:** A total of 18 patients were checked by digital subtraction angiography 3 years after implantation of the WEB device. We observed a worsening of the scores (in the Lubicz and BOSS scales) in 17% of the patients at 3 years - from A to B and from 0 to 0', respectively. This change was due to the deformation of the shape of the WEB. On average, the WEB shortened by 1.7 ± 0.5 mm. No recurrence events or strokes occurred during the follow-up. No patients required retreatment.
**Conclusion:** The change in the shape of the WEB did not require further treatment. Follow-up by digital subtraction angiography showed no substantial differences from the angioCT study.
**Limitations:** The sample is monocentric and limited in number.
**Ethics committee approval:** No information provided by the submitter.
**Funding for this study:** No funding was received for this study.
**Author Disclosures:**
Marco Spinetta: Nothing to disclose
Davide Negroni: Nothing to disclose
Carmelo Stanca: Nothing to disclose
Massimiliano Cernigliaro: Nothing to disclose
Giuseppe Guzzardi: Nothing to disclose
Agnese Siani: Nothing to disclose
Alessandro Carriero: Nothing to disclose
Riccardo Giuseppe Di Fiore: Nothing to disclose
Carolina Coda: Nothing to disclose

### RPS 809-13

**Filter device for prevention of large embolism during repair of symptomatic superior vena cava occlusion**

I. Mundo, M. Uder, A. Schmid, C. Treutlein, *M. S. May*; Erlangen/DE
*(matthias.may@uk-erlangen.de)*

**Purpose:** Symptomatic superior vena cava occlusion is an emergency indication for interventional recanalization. We aimed to establish a safe treatment by combining an aspiration device and a large filter to prevent pulmonary embolism.
**Methods or Backround:** Consecutive patients (n=5) with a symptomatic thrombotic superior vena cava occlusion were treated by a combination of 10F aspiration and filtration devices via combined femoral and brachial percutaneous access. Mechanical aspiration thrombectomy was supported by catheter aspiration, balloon dilatation, catheter-based thrombus fragmentation and stent implantation whenever needed. Procedural success was defined by established antegrade flow and clinical reduction of the superior vena cava syndrome (Aspirex and Capturex).
**Results or Findings:** Thrombectomy was successful in all patients. Relevant thrombotic material was found in all filter devices after retrieval. Supportive stent implantation was required in all patients. No patient suffered from visible or clinically relevant pulmonary embolism. Symptoms were relieved within hours or days in all patients. Nine of the patients had complications from the procedure or required surgical support.
**Conclusion:** Superior vena cava syndrome can be safely treated by a combination of mechanical aspiration and filtration devices.
**Limitations:** We can only provide a small case series of this rare disease.
**Ethics committee approval:** No information provided by the submitter.
**Funding for this study:** No funding was received for this study.
**Author Disclosures:**
Matthias Stefan May: Speaker: Siemens Healthcare GmBH
Michael Uder: Speaker: Siemens Healthcare GmBH
Axel Schmid: Speaker: Siemens Healthcare GmBH
Christoph Treutlein: Speaker: Siemens Healthcare GmBH
Isabellle Mundo: Speaker: Siemens Healthcare GmBH

**10:30-12:00** **Research Stage 4**

### Research Presentation Session: Radiographers

### RPS 814

### General radiography: tips and tricks

**Moderators**
E. M. Fallenberg; Munich/DE
N. Mekis; Ljubljana/SI
*(nejc.mekis@zf.uni-lj.si)*
**Author Disclosures:**
Eva Maria M. Fallenberg: Advisory Board: GE, Siemens, Bayer, Guerbet; Board Member: EUSOBI, AGO Guidelines, ESMO Guidelins early BC, S3 Guidelines BC, AG Mamma DRG; Speaker: GE, Siemens, Bayer, Guerbet, Roche
Nejc Mekis: Author: Co-author of presentation entiteled - How does the increase in BMI affect the radiation dose in general radiography?

### RPS 814-3

**Optimisation of technical parameters for obese patients' bedside pelvis and knee radiographs**

*C. S. d. Reis*, M. Gulizia, P. Monnin, S. S. Ghotra, S. E. de Labouchere, D. Alex, S. Grabherr; Lausanne/CH

**Purpose:** This study's purpose was to optimise technical parameters for obese patients' bedside pelvis and knee radiographs.
**Methods or Backround:** Twenty-seven radiographs of 4 corps investigated in a forensic imaging unit (CURML) with a mean BMI of 36 were acquired varying the tube potential (Pelvis:80-120kVp/Knee:70-90kVp), beam intensity (Pelvis:5.1-39.2mAs/Knee:2.2-8.3mAs), use of grid/or no grid. Image quality (IQ) was assessed using a perceptual approach with observers (ViewDex). Effective Dose (ED) was estimated. Descriptive statistics, intraclass correlation coefficient (ICC) and Visual Grading Characteristics (VGC) were used to analyse data.
**Results or Findings:** VGC results obtained from pelvis radiographs showed that the low energy setting (80-90kVp/22.9-39.2mAs) performed statistically worse than low/medium beam energy (90-100kVp/ 14.3-16.48mAs), medium/high beam energy (100-110kVp/8.9–16.48mAs) and the high beam energy setting (110-120kVp/5.1-8.7mAs) (<0.05 p-value). These results indicate that pelvis radiographs with a mobile system on obese patients should be acquired with higher kVp and lower mAs. This allows a dose reduction for the patient and a better image quality. For knee radiographs, the VGC results showed that setting (90kVp/2.2mAs) performed statistically better than low beam energy setting (70kVp/8.3mAs) and medium energy (80kVp/4.1mAs) (<0.05 p-value).
**Conclusion:** This study's results indicate that higher beam energy and lower beam intensity allow for patient dose reduction and increased image quality to identify relevant anatomy for pelvis and knee radiographs adequately. Ideal parameters to image the pelvis of obese patients are 110-120kVp/5.1-8.7mAs and 90kVp/2.2mAs, respectively, for the knee while using mobile x-ray systems.
**Limitations:** Low availability of corps BMI>30.
**Ethics committee approval:** Vaud Ethics committee (Ref2020-00311).
**Funding for this study:** Funding for this study was received by HES-SO scientific commission.
**Author Disclosures:**
Claudia Sa dos Reis: Nothing to disclose
Stephanie Elaine de Labouchere: Nothing to disclose

Marianna Gulizia: Nothing to disclose
Switinder Singh Ghotra: Nothing to disclose
Dominguez Alex: Nothing to disclose
Silke Grabherr: Nothing to disclose
Pascal Monnin: Nothing to disclose

### RPS 814-4

**Dose and image quality optimisation for obese patients' bedside thorax and abdominal radiographs**

M. Gulizia, P. Monnin, D. Alex, *S. S. Ghotra*, S. Grabherr, S. E. de Labouchere, C. S. d. Reis; Lausanne/CH
*(switighotra93@gmail.com)*

**Purpose:** To identify the most adequate combination of exposure parameters to acquire thorax and abdominal radiographs of obese patients at bedside.
**Methods or Backround:** Twenty-nine radiographs of 4 corps investigated in a forensic imaging unit (CURML) with a mean BMI of 36 were acquired, varying the tube potential (Tx:90-130kVp/Abd:100-120kVp), beam intensity (TX:0.9-9.2mAs/Abd:10.9-22mAs), use of grid/or no grid considering the patients' BMI and the body thickness. Image quality (IQ) was assessed using a perceptual approach with observers (ViewDex). Effective Dose (ED) was estimated. Descriptive statistics, intraclass correlation coefficient (ICC) and Visual Grading Characteristics (VGC) were used to analyse data.
**Results or Findings:** For thorax radiographs, the VGC results showed that setting medium-energy (100-110kVp/1.2-6.9mAs) performed statistically better than setting low-energy (90-100kVp/1.7-9.2 mAs) (0.02p-value) or setting high-energy (120-130kVp/0.9-4.1mAs) (0.013 p-value). For the abdomen, VGC results showed that the high-energy setting (120kVp/10.9mAs) performed statistically better than low-energy (100kVp/22mAs) (0.01 p-value) or medium-energy (110kVp/15.8mAs) (<0.05 p-value). This indicates that abdominal x-ray of obese patients acquired with higher kVp/lower mAs reduced patient dose and offered better image quality.
**Conclusion:** For thorax radiographs, the exposure parameters setting 100-110kVp/1.2-6.9mAs presents the best IQ at a lower dose. Abdominal investigation with a higher kVp/lower mAs combination (120kVp/10.9mAs) is the most adequate method to keep image quality while reducing the dose.
**Limitations:** A limitation was the low availability of corps BMI>30.
**Ethics committee approval:** Vaud Ethics committee (Ref2020-00311).
**Funding for this study:** Funding for this study was received by the HES-SO scientific commission.
**Author Disclosures:**
Claudia Sa dos Reis: Nothing to disclose
Stephanie Elaine de Labouchere: Nothing to disclose
Marianna Gulizia: Nothing to disclose
Switinder Singh Ghotra: Nothing to disclose
Dominguez Alex: Nothing to disclose
Silke Grabherr: Nothing to disclose
Pascal Monnin: Nothing to disclose

### RPS 814-5

**How does the increase in BMI affect the radiation dose in general radiography?**

*L. Dolenc*, B. Petrinjak, D. Škrk, N. Mekis; Ljubljana/SI
*(laura.dolenc@zf.uni-lj.si)*

**Purpose:** This study examined the effect of increasing body mass index (BMI) on the dose-area product (DAP) and effective dose (ED) for general radiography procedures.
**Methods or Backround:** The following projections were included in the study: lumbar spine AP and lateral, pelvis AP, knee AP and lateral, shoulder AP, chest AP and lateral. In this study, the data from 597 patients (1014 examinations) was collected. During the study, patients' weight, height, and DAP values were recorded. The BMI of the patients was calculated, and the patients were divided into three groups (normal-weight, overweight, and obese) based on their BMI value. The effective dose was calculated using PCXMC 2.0 software.
**Results or Findings:** The study showed an increase in DAP and ED when comparing normal-weight patients with overweight or obese patients. When comparing the DAP values between normal-weight and overweight patients, the increase was 29%, 59%, 62%, 46%, and 69% for images of the chest PA, chest lateral, lumbar spine AP, lumbar spine lateral, and pelvis, respectively. The increase was even higher when normal-weight patients were compared with obese patients: 96%, 216%, 272%, 117%, and 291% for the same imaging procedures aforementioned. The ED increase in overweight patients compared with normal-weight patients was 40%, and in obese patients, it was as high as 245%.
**Conclusion:** Based on the study results, it can be concluded that BMI has a major impact on DAP and ED for imaging of the lumbar spine, pelvis, and thorax, while it has a lesser impact on imaging of the knee and shoulder. No automatic control was used in the last two mentioned examinations, which may be the main reason for this result.
**Limitations:** The study was performed only in one institution.
**Ethics committee approval:** National medical ethics committee.
**Funding for this study:** No funding was received for this study.
**Author Disclosures:**
Nejc Mekis: Nothing to disclose
Barbara Petrinjak: Nothing to disclose
Laura Dolenc: Nothing to disclose
Damijan Škrk: Nothing to disclose

### RPS 814-6

**The effect of breast shielding outside the field of view on breast entrance surface dose in axial x-ray examinations: a phantom study**

L. Hurley, A. England, *M. F. F. McEntee*; Cork/IE
*(mark.mcentee@ucc.ie)*

**Purpose:** This study aimed to evaluate the effect of outside-of-field-of-view (OFOV) lead shielding on an anthropomorphic x-ray phantom's breast surface dose (BSD) for various axial skeleton x-ray examinations.
**Methods or Backround:** Using an anthropomorphic phantom and radiation dosimeter, the ESD of the breast was measured with/without OFOV shielding in (1) anterior-posterior (AP) abdomen, (2) AP cervical spine, (3) occipito-mental 30o (OM30) facial bones, (4) AP lumbar spine, and (5) lateral lumbar spine radiography. The effect of several exposure parameters on BSD with/without OFOV was examined, including (1) a low mAs technique, (2) grid use, (3) automatic exposure control (AEC) use, (4) wrap-around lead (WAL) use, (5) trolley use, and (6) x-ray table use. The mean BSD (microSv) and standard deviation (SD) for each radiographic protocol were calculated. A student t-test was carried out to evaluate whether BSD was reduced with the use of OFOV shielding.
**Results or Findings:** A total of 920 breast BSD measurements were recorded across the different protocol parameters. The largest decrease (non-significant, p=0.084) in mean BSD with OFOV shielding was 0.002 microSv. There was no significant BSD reduction in any projection with any combination of exposure settings.
**Conclusion:** This study found no significant decrease in the breast dose with OFOV shielding across a range of examinations and imaging protocols.
**Limitations:** This was a phantom study with limited variation in phantom size. Clinical follow-up studies are advised.
**Ethics committee approval:** No information provided by the submitter.
**Funding for this study:** No funding was received for this study.
**Author Disclosures:**
Mark F. F. McEntee: Nothing to disclose
Lauren Hurley: Nothing to disclose
Andrew England: Board Member: EFRS

### RPS 814-7

**Air gap used for dose optimization in DR pelvis examinations: a phantom study**

*S. C. Lytzen*, N. Tollak Jensen, E. G. Smærup, J. Jensen, S. D. Moerup, H. Precht, K. Brage; Odense/DK
*(sofie.cecilie.lytzen@rsyd.dk)*

**Purpose:** To investigate if air gap provides dose reduction compared to anti-scatter-grid for pelvic examinations while maintaining diagnostic image quality.
**Methods or Backround:** An anthropomorphic pelvic phantom (sectional lower torso phantom, SK 250, US) and an Alderson phantom (male Alderson Rando therapy phantom, sections 0-35, ART-200, US) were used. Both phantoms were examined with the following setup: Air-gap distances ranged from 5 to 30 cm with 5 cm intervals, source-to-image-distance (SID) ranged from 230 to 260 cm and kVp was set at 67, 77 and 87 kVp, respectively. The standard setting was: an anti-scatter grid image with an air gap of 0 cm, 115 cm SID and 77 kVp. Radiation doses were measured using thermoluminescent dosimeters tablets, which were placed equivalent to the ovaries. The image quality was rated by one blinded reporting radiographer using the visual grading analysis method (VGA), including 5 quality criteria on a 5-point Likert scale.
**Results or Findings:** A total of 47 images were produced, of which 10 were duplicates from the VGA performed on the anthropomorphic pelvic. The reference image had a radiation dose of 548.5 µGy; the lowest dose was achieved with an air gap of 5 cm, 87 kVp and 235 cm SID, corresponding to a dose reduction of 108.5 µGy (80%). The results of the VGA showed that all images were approved for diagnostic use.
**Conclusion:** It is possible to reduce the radiation dose by up to 80% to the ovaries while maintaining diagnostic image quality when an air gap is used compared to a standard grid in pelvic radiographs.
**Limitations:** More observers could have been used in the VGA allowing for inter- and intra-rater analyses. Future studies should consider measurements of hip angles.
**Ethics committee approval:** No information provided by the submitter.
**Funding for this study:** No funding was received for this study.
**Author Disclosures:**
Helle Precht: Author: Co-author
Karen Brage: Author: Co-author

Janni Jensen: Consultant: VGA consultant
Nathalie Tollak Jensen: Author: Co-author
Svea Deppe Moerup: Author: Co-author
Emma Gertsen Smærup: Author: Co-author
Sofie Cecilie Lytzen: Speaker: Presenter for abstract and the group Author: Co-author

### RPS 814-8

**kVp and anthropomorphic phantom thickness do not affect nodule visibility in chest DR**

*G. de Vries*[1], A. van der Heij-Meijer[2], J. Thompson[3], K. Szczepura[3]; [1]Haarlem/NL, [2]Groningen/NL, [3]Manchester/UK
*(geert.devries@inholland.nl)*

**Purpose:** In digital chest x-ray imaging, tube potential (kVp) and patient thickness are considered factors affecting scatter and inherent contrast at the image detector and, consequently, image quality. Local visibility of structures can be optimized post-acquisition by adjusting window-level/window-width (WL/WW) settings to visually access the post-processed images. In this study, we investigated the effect of WL/WW on nodule visibility.
**Methods or Backround:** PA images of a PBU-60 anthropomorphic chest phantom with synthetic nodules attached to the posterior side at 3 different locations (primarily rib, vascular, and rib and vascular anatomical noise) were acquired using a DR system. The exposure factors were 70kVp, 90kVp, 110kVp and 130kVp without added filtration using AEC. SID was 180cm. 4 phantom thicknesses were used: 0cm, 2.5cm, 5cm and 7.5cm of added PMMA. 16 human observers ranked the visibility of nodules in sets of 4 image patches: full range of kVps at 0cm or 7.5cm PMMA, and full range of PMMAs at 70kVp or 130kVp. All sets were presented with WL/WW unadjusted and adjusted by contrast stretching the image patches. Kendall's coefficient of concordance W was estimated to assess differences in perceived nodule visibility.
**Results or Findings:** For unadjusted WL/WW, the concordance was perfect (W=0.98) for the nodule in primarily rib background at a range of PMMAs at 130kVp. It was moderate at a range of PMMAs at 70kVp for nodules in ribs and vascular (W=0.51) and primarily vascular background (W=0.49). In adjusted WL/WW sets, the concordance ranged from slight to fair (W<0.4).
**Conclusion:** These results suggest that the nodule visibility is affected by phantom thickness when WL/WW is unadjusted. Differences in visibility are not observed when WL/WW is adjusted to match local contrasts.
**Limitations:** Small group of observers and clear nodule visibility.
**Ethics committee approval:** University of Salford ethics committee ID 3402.
**Funding for this study:** No funding was received for this study.
**Author Disclosures:**
John Thompson: Nothing to disclose
Katy Szczepura: Nothing to disclose
Annemieke van der Heij-Meijer: Nothing to disclose
Geert de Vries: Nothing to disclose

### RPS 814-9

**An investigation into dose optimisation and image quality in digital radiography using the high kVp technique**

*T. Varga*[1], M. O'Connor[2], A. O'Brien[1]; [1]Waterford/IE, [2]Dublin/IE
*(tudor.varga@hse.ie)*

**Purpose:** The high kVp technique, such as the 15% kVp rule, is a well-known dose reduction method and is becoming more relevant with recent advances in digital radiography (DR) technology. This study investigated the optimisation of pelvis and lumbar spine pre-set DR protocols in a single clinical site by using the high kVp rule.
**Methods or Backround:** A series of radiographic images of an anthropomorphic phantom and quality control (QA) test tools were obtained. The phantom and test tools were exposed to clinical pre-set protocols with only kVp being varied. The AEC was used to determine the mAs for each kVp setting. All phantom radiographs were assessed by 17 radiographers using relative visual grading analysis (VGA). All test tools images were scored by 4 physicists using specifically designed manufacturer tests.
**Results or Findings:** The Spearman's rank correlation test intra-observer reliability indicates that there was a moderate to strong positive correlation across 11 observers with rs ranging from 0.434 to 0.621 (p<0.05). Five observers were omitted from the study due to insignificant intra-observer correlation (p>0.05). Up to approximately 100 kVp, all images were scored as being diagnostic, with most images being rated on average as "same as reference". Both the Huttner and TO12 results show that there is no real perceptible difference in spatial and contrast resolution with the increase of kVp.
**Conclusion:** The results show that contrast and image quality are not heavily affected by kVp on digital systems. This study validates the high kVp technique as a useful tool for reducing patient radiation doses whilst maintaining the diagnostic quality of digital pelvis and lumbar spine radiography.
**Limitations:** The study was limited by unavoidable artefacts present in the phantom.
**Ethics committee approval:** No information provided by the submitter.
**Funding for this study:** No funding was received for this study.
**Author Disclosures:**
Michelle O'Connor: Nothing to disclose
Alan O'Brien: Nothing to disclose
Tudor Varga: Nothing to disclose

### RPS 814-10

**Bone mineral density and body composition: a comparative study between soccer players and sedentary individuals**

R. Cataluna, *L. P. V. Ribeiro*, A. F. C. L. Abrantes, J. Pinheiro, S. I. Rodrigues; Faro/PT
*(luispedroribeiro@hotmail.com)*

**Purpose:** The purpose of this study was to assess bone mineral density (BMD), bone mineral content (BMC) and body composition (BC) in soccer players and sedentary normal-weighted individuals.
**Methods or Backround:** Whole-body dual-energy x-ray absorptiometry (DXA) was performed to assess BMD, BMC and BC in 54 individuals. Anthropometric measures such as stature, body mass and nutrient intake were also measured. Descriptive statistics comprised median, standard deviation, maximum, minimum, frequency and mode. Comparison between groups was performed using t-tests for independent samples.
**Results or Findings:** In view of the results of the "t-student" test in the analysis of BMD, we can say that sports practice positively influences its increase since the group of "athletes" presented total body BMD values higher by almost 0.138g/cm2 with "d-cohen" values greater than 1.5, that is, of moderate effect. These BMD values measured in the "athletes" group were higher than those obtained in the "sedentary" group, not only for the whole body but also for all anatomical segments analyzed.
**Conclusion:** Soccer has a positive accretion effect on the whole body when compared to sedentary individuals.
**Limitations:** The study was limited by the sample size.
**Ethics committee approval:** No information was provided by the submitter.
**Funding for this study:** No funding was received for this study.
**Author Disclosures:**
António Fernando Caldeira Lagem Abrantes: Nothing to disclose
Sónia Isabel Rodrigues: Nothing to disclose
Joao Pinheiro: Nothing to disclose
Rui Cataluna: Nothing to disclose
Luís Pedro Vieira Ribeiro: Nothing to disclose

### RPS 814-11

**Evaluating the knowledge and awareness of breast density amongst Maltese women undergoing mammography screening**

*S. L. Gauci*[1], J. G. Couto[2], D. Mizzi[2]; [1]Saint Paul's Bay/MT, [2]Msida/MT
*(samgaucii@gmail.com)*

**Purpose:** Breast density refers to the proportion of breast glandular tissue compared to fatty tissue. This study aimed to evaluate the knowledge and awareness of breast density amongst Maltese women undergoing mammography screening at the National Screening Unit.
**Methods or Backround:** A quantitative, prospective, cross-sectional, and descriptive study was performed using a validated closed-ended questionnaire, distributed to women aged 50-69 years, who were eligible for breast cancer screening at the National Screening Unit. The data was analysed using the Friedman test and Kruskal Wallis test, using IBM-SPSS (v28) software.
**Results or Findings:** 127 questionnaires were collected (maximum margin of error of 8.66%, assuming a 95% confidence interval). Knowledge and awareness of breast density and the associated breast cancer risks were low (2.80-3.34 out of 5), whilst awareness of supplementary screening was higher (3.65). Age, occupation, and level of education were related to participants' knowledge and awareness. Healthcare professionals (40%) and leaflets (40%) were the preferred sources of information.
**Conclusion:** Knowledge and awareness of breast density and the associated risks are lacking among the studied population. Further information regarding breast density needs to be provided to women. This knowledge will empower women to seek the best care.
**Limitations:** Some socio-demographic factors were related to women's knowledge and awareness; however, it is recommended that interviews and further studies with a larger sample should be carried out.
**Ethics committee approval:** No information was provided by the submitter.
**Funding for this study:** No funding was received for this study.
**Author Disclosures:**
Deborah Mizzi: Author: University of Malta
Samantha Leah Gauci: Nothing to disclose
Jose Guilherme Couto: Author: University of Malta

### RPS 814-12

**Pitfalls of mammography positioning: missed tissues at the chest wall edge due to improper compression**

*H. Yamashina*; Fukushima/JP
*(coco81h@gmail.com)*

**Purpose:** This study aimed to review the effect of breast compression and the pitfalls of the compression technique.
**Methods or Backround:** Compression is the most important aspect of mammographic positioning procedures. The mammographic quality control manual published in 2022 in Japan states that the maximum compression force should be 120 to 140 Newtons. In contrast, the mammography guideline has no longer stated the maximum compression force since 2010. A recent study revealed that compression force varies significantly even in the same patient. A compressive breast phantom (10 cm x 16.6 cm x 5 cm) containing simulated lesions was utilized in this study. Images with compression force ranging from 60 to 130N at intervals of 10N were obtained. The distance of two axes on a phantom image and the missed tissue at the chest wall edge were measured using ImageJ.
**Results or Findings:** The chest-to-nipple and medial-to-axilla distances were 93.78mm, 181.41 at a maximum when applying 130N, and 87.96mm, 173.33 at a minimum when applying 60N. The simulated lesion placed nearest to the chest wall edge was viewed with a complete circular appearance when applying 60 to 100N; however, a part of the round perimeter was missing with the compression force 110N or more.
**Conclusion:** The more the breast compressed, the more the projected area was viewed; however, it may increase the missed tissue area at the chest wall edge. This reflects that if the breast was compressed by the compression paddle rather than mammographers pulling up, spread out, and firmly holding its breast by hands, it will cause an increase in the missed tissue area.
**Limitations:** The phantom used in this study simulates a 45mm compressed breast. It requires further study using clinical images.
**Ethics committee approval:** No information provided by the submitter.
**Funding for this study:** No funding was received for this study.
**Author Disclosures:**
Hiroko Yamashina: Nothing to disclose

### RPS 814-13

**The diagnostic performance of supplementary imaging procedures for breast cancer screening in women with dense breasts: a systematic review and meta-analysis**

*D. Mizzi*[1], C. Allely[2], F. Zarb[1], J. F. Kelly[3], P. H. Hogg[2], M. F. F. McEntee[4], A. England[4], C. Mercer[2]; [1]Msida/MT, [2]Salford/UK, [3]Chester/UK, [4]Cork/IE
*(deborah.mizzi@um.edu.mt)*

**Purpose:** The purpose of this study was to evaluate and review the diagnostic test performance of supplementary imaging procedures for breast cancer screening in women with dense breasts.
**Methods or Backround:** A systematic search and quantitative synthesis of peer-reviewed publications in English (January 2000 to March 2021) were conducted. Diagnostic meta-analysis was performed to estimate the proportion of cancers detected, recalls and biopsies and to calculate sensitivity and specificity for all supplementary imaging. Subgroup meta-analysis was performed for each supplementary imaging to plot comparison summary data.
**Results or Findings:** A total of 3,764 studies underwent title and abstract screening. Two hundred twenty-one studies underwent full-text screening, and forty-two were included in the quantitative synthesis. Based on a fixed effects model (p=0.020), the sensitivity of supplementary imaging for breast cancer screening in women with dense breasts was 89% (95% CI: 81%-94%), while specificity was 91% (95% CI: 79%-96%). Supplementary imaging procedures in women with negative mammograms would lead to a detection of an additional 4 true cancers per 1,000 women (p< 0.001) (95% CI: 4.0-5.0 per 1,000 women). Recalls would increase to 109 per 1,000 women (p< 0.001) (95% CI: 94-125 per 1,000 women), and the proportion of biopsies would increase to 36 per 1,000 women (p< 0.001) (95% CI: 29-43 per 1,000 women).
**Conclusion:** Meta-analysis results determined that more women with dense breasts would be correctly diagnosed with breast cancer using supplementary imaging, but more women would undergo further imaging and biopsies, and this can lead to greater potential additional anxiety and greater utilisation of health resources.
**Limitations:** No limitations were identified.
**Ethics committee approval:** No information provided by the submitter.
**Funding for this study:** No funding was received for this study.
**Author Disclosures:**
Mark F. F. McEntee: Nothing to disclose
Peter Harry Hogg: Nothing to disclose
Deborah Mizzi: Nothing to disclose
Clare Allely: Nothing to disclose
Francis Zarb: Nothing to disclose
Claire Mercer: Nothing to disclose
Andrew England: Nothing to disclose
Judith Frances Kelly: Nothing to disclose

### RPS 814-14

**Characterisation of plain radiography performance for upper and lower limbs, thorax, abdomen, skull and spine in Western Switzerland**

C. S. d. Reis[1], M. Gulizia[1], *S. E. de Labouchere*[1], M. Champendal[1], Z. Sun[2], C. Silva[3]; [1]Lausanne/CH, [2]Perth/AU, [3]Lisboa/PT
*(stephanie.delabouchere@hesav.ch)*

**Purpose:** To investigate current practice regarding the use of plain radiography and the main justifications in Western Switzerland.
**Methods or Backround:** An online survey was designed and tested using LimeSurvey. Clinical tutors working in medical imaging departments that collaborate with "HES" institutions from Western Switzerland were invited to participate. The snowball sampling technique was employed, and the surveys were distributed through the schools' network and by the Swiss Association of Radiographers. Descriptive and inferential statistics were performed using Excel and IBM SPSS v2.0 software.
**Results or Findings:** The global response rate was 56.5% (26/46). The shoulder projections were the most commonly performed radiographic examinations, namely Neer (92.3%) and antero-posterior/(AP)/neutral position (80.8%) for trauma and degenerative disorders. For the pelvis region, the AP (92.3%), AP for surgical measurements (80.8%), and Lequesne (18/26) were also frequently referred to. Concerning the skull, the Hirtz, tangential nose bones, worms and Caldwell's projections were less commonly performed in clinical practice. The lateral view of the skull (30.8%) was still requested for oncological purposes, and the panoramic scanning dental x-ray (23.1%) was also used. Cervical AP and lateral views were identified as frequently requested (92.3%). The lumbar spine was typically assessed by 10 different projections, with lateral (88.5%) and PA upright (84.6%) views being the most frequently requested projections for the follow-up of degenerative disorders. Thorax PA projections are frequently performed for infections and follow-up purposes.
**Conclusion:** Plain radiography still plays a central role for upper and lower limbs and thorax examinations. Most skull projections are not being performed. Trauma is the main indication as indicated by the referrals for the limbs.
**Limitations:** The study was only conducted in Western Switzerland. The was a low response rate during the Covid-19 period.
**Ethics committee approval:** Vaud Ethics committee (Ref2020-00311).
**Funding for this study:** Funding for this study was received by the HES-SO scientific commission.
**Author Disclosures:**
Claudia Sa dos Reis: Nothing to disclose
Stephanie Elaine de Labouchere: Nothing to disclose
Zhonghua Sun: Nothing to disclose
Marianna Gulizia: Nothing to disclose
Carina Silva: Nothing to disclose
Mélanie Champendal: Nothing to disclose

**12:30-13:30** **Research Stage 1**

### Research Presentation Session: Artificial Intelligence & Machine Learning & Imaging Informatics

### RPS 905

### New approaches for sustainability and image optimisation

**Moderator**
A. Trianni; Trento/IT

### RPS 905-2

**Turn it off! Saving energy in radiology departments by implementing live dashboards showing idle imaging modalities, PCs and PACS workstations not powered down during off-hours**

*M. T. Meyer*, T. Heye, E. M. Merkle, J. Vosshenrich; Basel/CH

**Purpose:** To develop and implement a live dashboard monitoring the network status of all imaging modalities and PC workstations in a radiology department in order to reduce the energy consumption.
**Methods or Backround:** Our tertiary care radiology department operates around 40 medical imaging systems and 200 PC workstations. In order to identify devices running in idle mode rather than being switched off when not in use, a self-developed Python-based script querying the network status ("Online" vs "Offline") of all imaging modalities and PC workstations was implemented on an Ubuntu server. The status of all network clients is tracked automatically in 15-minute intervals by a ping signal to the respective IP addresses or DNS aliases. Client names, network status and timestamps are recorded in an SQL database. Data can be queried from the database and visualized with open-source or commercially available business intelligence software solutions (e.g. Redash or Tableau).
**Results or Findings:** Current network status, as well as trends over the last 24 hours, are visualized in workspace-specific live dashboards (e.g. information on MRI scanners and side panels for technologists or information on PACS workstations in the different imaging divisions for radiologists). Using the provided dashboard overviews, each team of technologists or radiologists may now identify unused imaging modalities or PC workstations running in idle mode in their environment (e.g. during off-hours) and can contribute to reducing the department's energy consumption by switching off currently unneeded consumers of energy.
**Conclusion:** Live dashboards can provide insights on the current status of imaging modalities and PC workstations at a glance. Radiology department staff members may now easily contribute to reducing the department's energy consumption by identifying and switching off unused machines, especially during off-hours. The script's source code will be published open-source.
**Limitations:** No limitations were identified.
**Ethics committee approval:** No information provided by the submitter.
**Funding for this study:** No funding was received for this study.
**Author Disclosures:**
Tobias Heye: Nothing to disclose
Elmar M. Merkle: Nothing to disclose
Jan Vosshenrich: Nothing to disclose
Manfred Tobias Meyer: Nothing to disclose

### RPS 905-3

### Reduction of Gadolinium-based contrast agents in magnetic resonance imaging: evaluation of a convolutional neural network in different multiparametric settings

*R. Haase*, T. Pinetz, Z. Bendella, E. Kobler, W. Block, A. Effland, A. Radbruch, K. Deike-Hofmann; Bonn/DE

**Purpose:** The purpose of the study was to reimplement a state-of-the-art convolutional neural network used to synthesize artificial T1-weighted (T1w) full-dose images from corresponding non-contrast and low-dose images and test its performance on a patient population acquired prospectively.
**Methods or Backround:** In this monocentric, prospective study, 138 participants were included. They received an imaging protocol with the acquisition of a T1w-low-dose after administration of 10% of the standard dose and a T1w-full-dose after administration of the remaining 90% of a gadolinium-based contrast agent. Four input settings were differentiated, and the output was tested using a reader study: T1w-non-contrast and T1w-low-dose (standard-setting), T1w-non-contrast and T1w-low-dose with a prolonged post-injection time of 5-min (5-min-setting), multiple non-contrast sequences and the T1w-low-dose (extended-setting), and only non-contrast sequences (T1w, T2w, diffusion) (zero-dose-setting).
**Results or Findings:** The proportion of scans scored as fully or mostly interchangeable with the original T1w-full-dose was 55, 58, 43, and 3% and the average counts of false positives per case were 0.42±0.83, 0.34±0.71, 0.82±1.15, and 2.00±1.07 for the standard, 5-min, extended, and zero-dose setting, respectively. Using a five-point Likert scale (0 to 4, 0 being the worst), all settings of synthesized full-dose images showed significantly poorer contrast enhancement of lesions compared to the original T1w-full-dose (difference of average degree of contrast enhancement: Standard: -0.97±0.83, p=<0.001; 5-min: -0.93±0.91, p=<0.001; Extended: -0.96±0.97, p=<0.001; Zero-dose: -2.39±1.14, p=<0.001). The average scores of conformity of the lesions compared to the original full-dose sequence were 2.25±1.21, 2.22±1.27, 2.24±1.25, and 0.73±0.93 for the standard, 5-min, extended and zero-dose setting.
**Conclusion:** Despite good image quality in all settings, both false-negative and false-positive signals resulted in significantly limited interchangeability of the synthesized T1w-full-dose with the respective original T1w-full-dose in regard to the clinical conclusion.
**Limitations:** A multicentric study would provide additional insights.
**Ethics committee approval:** No information was provided by the submitter.
**Funding for this study:** R.H.: Bonfor-Research-Grant (University Bonn). A.E./T.P.: DFG-Grant-EXC-2047/1--390685813/--EXC2151--390873048.
**Author Disclosures:**
Katerina Deike-Hofmann: Founder: relios.vision GmbH
Wolfgang Block: Nothing to disclose
Erich Kobler: Founder: relios.vision GmbH
Robert Haase: Founder: relios.vision GmbH
Alexander Effland: Founder: relios.vision GmbH
Zeynep Bendella: Nothing to disclose
Thomas Pinetz: Founder: relios.vision GmbH
Alexander Radbruch: Founder: relios.vision GmbH

### RPS 905-4

### Diffusion probabilistic models to reduce the need for contrast agents in breast MRI

G. Müller-Franzes, S. Nebelung, C. K. Kuhl, D. Truhn, *T. Nolte*; Aachen/DE
*(tnolte@ukaachen.de)*

**Purpose:** The study aims to explore diffusion probabilistic models (DPM) as a machine learning technique to recover the appearance of contrast-enhanced breast DCE-MRI subtraction images from virtual low-contrast (VLC) versions thereof.
**Methods or Backround:** We studied DPM denoise images by adding white Gaussian noise (WGN) to the training images in progressive steps and learning to reverse this process. We set up a DPM following Ho et al. [arXiv:2006.11239] and trained it on subtraction images of 9751 breast DCE-MRI examinations. For 50 test images (scaled between 0 and 1) of enhancing lesions, a series of increasingly noisy VLC subtraction images was obtained by adding WGN with increasing standard deviation σ. Denoised images were retrieved by applying the DPM. A medical imaging scientist judged from which σ onwards the gross location of enhancement, lesion structural details and background structural details were lost. Additionally, the multi-scale structural similarity index measure (MS-SSIM) was calculated between denoised and original subtraction images.
**Results or Findings:** On average, lesion and background structural details started to be lost from σ=0.09±0.02 (corresponding to MS-SSIM=0.78±0.03) and σ=0.08±0.02 (corresponding to MS-SSIM=0.79±0.03), respectively. The gross location of enhancement remained correct over the investigated range of σ (i.e., up to σ=0.32) for 12/50 test cases and started to change from σ=0.24±0.05 onwards for the remaining ones (corresponding to MS-SSIM=0.69±0.05).
**Conclusion:** DPM permit to recover the signal from input images with a high and flexible initial noise level. They may pave the way to breast DCE-MRI with drastically reduced contrast agent dose, and, more generally, to enhance signal-to-noise ratios of medical images.
**Limitations:** Limitations were the model being under active development and a thorough radiologic evaluation, which is still pending and will be presented at the conference.
**Ethics committee approval:** Local institutional review board approval was obtained (EK028/19).
**Funding for this study:** The 2022 ESR Research Seed Grants were kindly supported by an unrestricted, non-exclusive grant from GE Healthcare.
**Author Disclosures:**
Gustav Müller-Franzes: Nothing to disclose
Sven Nebelung: Nothing to disclose
Teresa Nolte: Grant Recipient: ESR Seed Grant Proramme (supported by GE Healthcare)
Christiane K. Kuhl: Nothing to disclose
Daniel Truhn: Nothing to disclose

### RPS 905-5

### Using machine learning to help reduce the need for contrast agents in breast MRI through image synthesis

*G. Müller-Franzes*, S. Nebelung, C. K. Kuhl, D. Truhn, T. Nolte; Aachen/DE
*(gumueller@ukaachen.de)*

**Purpose:** To investigate if Generative Neural Networks (GNNs) can recover the appearance of CE breast MR images from unenhanced as well as from virtual low-contrast contrast-enhanced (CE) subtraction images.
**Methods or Backround:** Virtual low-contrast subtraction images were calculated from 9751 breast MRI examinations by adding Gaussian noise to the existing CE subtraction images so that their signal-to-noise ratio corresponded to a four-fold reduced contrast agent dose. A GNN (Pix2PixHD with Adam optimizer, learning rate 2*10-4, batch size 48 and early stopping) was trained to recover the full-dose CE images from the virtual low-contrast images (approach A) or from unenhanced T1- and T2-weighted images only (approach B). Blinded to the true nature of 100 test images, two readers (>5 years of experience in breast MRI) first judged whether a full-dose CE subtraction image was real or synthetic. Unblinded, they rated the overall image appearance and lesion conspicuity of synthetic as compared to real full-dose CE subtraction images with 5-point Likert scales.
**Results or Findings:** Blinded readers were unable to distinguish real from synthetic subtraction images (average accuracy of differentiation: 52% [52/100] for approach A and 61% [61/100] for approach B). Equivalence of overall image appearance was rated significantly higher for approach A than for approach B (mean rating 4.6±0.1 vs 3.0±0.2;p<0.001). For approach A, lesion conspicuity in synthetic images was rated equivalent to the one in real images, while for approach B, it was scored significantly lower (mean rating 4.9±0.1 vs 1.8±0.1;p<0.001).
**Conclusion:** GNNs can use simulated low-contrast, but not unenhanced images to recover the full image contrast in CE breast MRI. GNNs may enable breast MRI with reduced contrast-agent dose.
**Limitations:** Our GNN is currently limited to a fixed input noise level.

**Ethics committee approval:** Local institutional review board approval was obtained (EK028/19).
**Funding for this study:** No funding was received for this study.
**Author Disclosures:**
Gustav Müller-Franzes: Nothing to disclose
Sven Nebelung: Nothing to disclose
Teresa Nolte: Nothing to disclose
Christiane K. Kuhl: Nothing to disclose
Daniel Truhn: Nothing to disclose

### RPS 905-6

**Faster MRI acquisition: the relationship between visual and diagnostic quality in prostate MRI reconstruction**

*Q. Y. van Lohuizen*, C. Roest, F. Simonis, S. Fransen, D. Yakar, T. Kwee, H. Huisman; Groningen/NL
*(q.y.van.lohuizen@umcg.nl)*

**Purpose:** Studies showed that AI reconstruction of accelerated MRI improves visual quality, but it is unclear whether this improves diagnostic value. We investigated a novel framework for accelerated MRI by assessing reconstruction quality (naïve- vs AI-reconstructed) by comparing diagnostic performance and visual similarity as an outcome for prostate cancer detection.
**Methods or Backround:** A retrospective multi-site study was performed on a cohort of 1535 patients who underwent bi-parametric prostate MRI between 2016-2020. An expert radiologist delineated all clinically significant prostate cancer (csPCa) lesions (PI-RADS ≥ 4). T2W scans were retrospectively undersampled in k-space, simulating four (R4) and eight (R8) times acceleration. A 3D U-Net was used to reconstruct undersampled images. The resulting images were fed to an existing state-of-the-art csPCa detection AI to evaluate the effect of AI reconstruction on diagnosis. Visual image quality (SSIM) was compared with a Wilcoxon test. Lesion level diagnostics were evaluated by comparing the partial area-under-the-FROC-curve over the false positive interval 0.1-2.5 (pAUC) using permutation tests.
**Results or Findings:** AI-based reconstruction significantly improved visual quality compared to naïve (IFFT) reconstruction MRI at R4 (SSIM 0.78±0.02 vs 0.68±0.03, p<0.001) and R8 (SSIM 0.67±0.03 vs 0.51±0.03, p<0.001), however, no significant improvements in diagnostic performance were observed for R4 (pAUC FROC 1.33 [CI 1.28-1.39] vs 1.29 [CI 1.23-1.35], p=0.37), nor R8 (pAUC FROC 1.12 [CI 1.07-1.17] vs 0.95 [CI 1.89-1.01], p=0.067). AI-based reconstruction resulted in 0.1 or more decrease in sensitivity compared to unaccelerated MRI.
**Conclusion:** Recovery of visual quality in reconstructions does not correlate with recovering diagnostic quality, emphasizing the importance of measuring diagnostic value rather than visual similarity. AI reconstruction tools should be approached with care because they might have been optimized to reconstruct visually appealing images instead of diagnostic images.
**Limitations:** No limitations were identified.
**Ethics committee approval:** No information was provided by the submitter.
**Funding for this study:** Grant: Heath~Holland.
**Author Disclosures:**
Henkjan Huisman: Nothing to disclose
Stefan Fransen: Grant Recipient: Siemens Healthineers
Quintin Yves van Lohuizen: Grant Recipient: Siemens Healthineers
Thomas Kwee: Nothing to disclose
Derya Yakar: Consultant: At Astellas
Christian Roest: Grant Recipient: Siemens Healthineers
Frank Simonis: Nothing to disclose

### RPS 905-7

**Effect of deep learning based denoising method on low count/reduced time protocol in low dose 18F-FDG scans with digital PET/CT**

*S. S. Singh*, S. Nigam; Lucknow/IN
*(rahul.sin880@gmail.com)*

**Purpose:** To investigate the effect of deep learning (DL) based denoising method on the image parameters of low dose 18F-FDG positron emission tomography (PET) with digital PET/CT and reconstructed with different time per bed position/percentage count.
**Methods or Backround:** We retrospectively included 90 patients undergoing an F-18 FDG PET/CT imaging on a digital PET-CT scanner (uMI550, United Imaging Healthcare) with an acquisition of 120 seconds per bed position. The data was rebinned into five datasets with 100%, 75%, 50%, 37.5% and 25% of the total counts, respectively. Images were reconstructed by OSEM algorithm followed by DL and Gaussian filter (GS). Standardized uptake values (SUVs) in liver, mediastinum, liver and mediastinal signal-to-noise ratio (SNR) were compared among different subsets.
**Results or Findings:** The average injected 18F-FDG dose was 3±0.5 MBq/kg body weight. The mean uptake time was 60±10 minutes. There was a significant difference between SUVmax of liver and mediastinum between all subsets of DL-based and GS-based denoising methods; however, the difference in SUVmean was non-significant. Significantly higher SNR and reduced image noise were found in the DL group compared to the GS cohort (p<0.05) in all the subsets. However, a significant difference in lesion contrast was only observed in the 45s and 30s image cohorts. In the DL group, SUV values, SNR, background noise and lesion contrast showed no significant difference between the reference (120s) and the 90s cohort.
**Conclusion:** DL-based denoising method results in a better reduction in image noise and signal-to-noise ratio compared to the GS method at low-dose FDG PET scans. Image acquisition time per bed position can be reduced to 90 seconds without compromising image quality and semi-quantitative data.
**Limitations:** BMI-based analysis was not done due to fewer patients in each BMI group.
**Ethics committee approval:** No information was provided by the submitter.
**Funding for this study:** No funding was received for this study.
**Author Disclosures:**
Satish Nigam: Nothing to disclose
Shashank Shekhar Singh: Nothing to disclose

### RPS 905-8

**Identification of repeat rates in pulmonary embolism exams: comparison of manual and automatic analyses**

A. Romanyukha[1], N. Fitousi[1], *C. R. Jeukens*[2]; [1]Leuven/BE, [2]Maastricht/NL
*(cecile.jeukens@mumc.nl)*

**Purpose:** Repeated acquisitions have been recognized in the literature as a source of increased patient radiation exposure and reduced department efficiency. The goal of this study was to identify and compare repeat rates in pulmonary embolism (PE) exams in a major European university hospital determined by manual analysis and automatic detection using a novel software.
**Methods or Backround:** Repeated scans in pulmonary embolism exams performed in the Maastricht University Medical Centre from July 2017 to December 2021 were first identified manually through the analysis of series descriptions corresponding to pulmonary embolism spiral acquisitions of 4481 studies. DICOM metadata was then extracted from the radiation dose management system (Radimetrics, Bayer, Germany) and processed using dedicated automated software (FOQAL-CT Repeat, Qaelum, Belgium). The latter uses a patented algorithm developed by the University of Wisconsin-Madison (Rose et al., 2020). First, the algorithm automatically identifies the base pattern of acquisitions normally present in the PE protocol. Next, repeated acquisitions were identified as deviations from the base pattern, and repeat rates of localizers, bolus tracking and PE scans were calculated.
**Results or Findings:** In the manual analysis, the repeat rate for the PE scans was quantified as 3.0%. The automatic algorithm found repeat rates of 2.0% for localizers, 4.7% for bolus tracking and 3.0% for PE scans.
**Conclusion:** Repeat rates of the PE scans were found to agree well between the manual analysis and the automated software. These results can be used to target protocols with the highest repeat rates and improve clinical practice to minimize unnecessary repetitions.
**Limitations:** No limitations were identified.
**Ethics committee approval:** No information provided by the submitter.
**Funding for this study:** No funding was received for this study.
**Author Disclosures:**
Anna Romanyukha: Employee: Qaelum
Cécile RLPN Jeukens: Nothing to disclose
Niki Fitousi: Employee: Qaelum

### RPS 905-9

**Deep learning-based image denoising in imaging of kidney stones: an assessment of image quality and comparison to state-of-the-art image reconstructions**

*R. A. Terzis*, R. P. Reimer, C. Nelles, E. Celik, L. Caldeira, D. Zopfs, A. Heidenreich, D. Maintz, N. Große Hokamp; Cologne/DE
*(robert.terzis@uk-koeln.de)*

**Purpose:** This study aimed to investigate the image quality and diagnostic accuracy of an artificial intelligence-based denoising image reconstruction technique (AIR) generated by low-dose non-contrast computed tomography (LDCT) in patients with suspected urolithiasis.
**Methods or Backround:** This retrospective IRB-approved study included LDCTs (CTDIvol, 2mGy) of 76 patients (age: 40.3±5.2years, M/W: 51/25) with suspected urolithiasis. Filtered-back projection (FBP), hybrid-iterative and model-based iterative reconstruction (HIR/MBIR, respectively) were reconstructed. FBP images were processed using an FDA-approved AIR. ROIs were placed in renal parenchyma, fat, muscle and urinary bladder. Signal- and contrast-to-noise ratios (SNR/CNR, respectively) were calculated. Two radiologists evaluated the image quality on 5-point Likert scales and the presence as well as the size of kidney stones. Statistics were performed using the Steel-Dwass test, contingency tables and correlation coefficients.
**Results or Findings:** Image noise gradually decreased from FBP, HIR, and AIR to MBIR with significant differences between each image reconstruction technique (p<0.05). SNR and CNR were comparable between MBIR and AIR, while it was significantly lower in HIR followed by FBP (e.g. SNR: 1.5±0.3; 1.4±0.4; 1.0±0.3; 0.7±0.2, p<0.05). Subjective analysis confirmed the best

Thursday image quality in MBIR, followed by AIR and HIR, both being superior to FBP ($p<0.05$). Diagnostic accuracy for kidney stone detection was best using MBIR (0.94), lowest using FBP (0.84) and comparable between AIR (0.90) and HIR (0.90). Stone size measurements were comparable between all reconstructions and showed excellent correlation ($r2=0.958-0.975$).
**Conclusion:** AIR increased the image quality of LDCT in suspected urolithiasis as compared to FBP and HIR, while diagnostic accuracy was comparable to HIR and superior to FBP. AIR allows radiation dose savings in LDCT in suspected urolithiasis while maintaining image quality.
**Limitations:** Retrospective single-centre study.
**Ethics committee approval:** No information was provided by the submitter.
**Funding for this study:** The study was supported by the Cologne Clinician Scientist Program and funded by the Deutsche Forschungsgemeinschaft (DFG) (Project No. 413543196).
**Author Disclosures:**
Axel Heidenreich: Nothing to disclose
Christian Nelles: Nothing to disclose
Liliana Caldeira: Nothing to disclose
David Maintz: Nothing to disclose
David Zopfs: Nothing to disclose
Erkan Celik: Nothing to disclose
Nils Große Hokamp: Nothing to disclose
Robert Angelo Terzis: Nothing to disclose
Robert Peter Reimer: Nothing to disclose

**12:30-13:30 Research Stage 2**

### Research Presentation Session: Emergency Imaging

### RPS 917

### Abdominal emergencies: nontraumatic and traumatic

**Moderator**
H. Ringl; Vienna/AT

### RPS 917-2

**Determination of the most suitable monoenergetic level of virtual monochromatic images in dual-source CT for the diagnosis of bowel obstruction and colitis**

L. Lahuna, J. Greffier, J. Frandon, J-P. Beregi, J. Goupil, *D. Dabli*; Nîmes/FR
*(Djamel.Dabli@chu-nimes.fr)*

**Purpose:** To determine the monoenergetic level with the best image quality for the diagnosis of colitis and bowel obstruction in an emergency context.
**Methods or Backround:** The images of 64 patients who benefited from an enhanced abdominal-pelvis scan in dual-energy CT (DECT) mode for the diagnosis of colitis or bowel obstruction were retrospectively analyzed. Acquisitions were performed on a third-generation dual-source CT (DSCT) scanner at portal phase. Acquisitions were performed at 100/Sn150kVp. Mixed images (simulating images at 120kVp) were generated as well as virtual monochromatic images (VMI) at 40/50/60/70keV. An objective image quality assessment was performed by measuring contrast, noise and contrast-to-noise ratio (CNR). Subjective analysis was performed by anonymous scoring of the images by two radiologists evaluating the noise, smoothing, overall quality and diagnostic quality on a Likert scale. The results were compared between the different images using the Mann-Whitney U test for paired samples.
**Results or Findings:** Of all the patients, 33 had intestinal obstruction, and 31 had colitis. The mean age was 65±20 and 49±22 years, respectively. The maximum CNR was measured in VMI at 60keV for both examination types, but the difference was significant only compared to 70keV for bowel obstruction and to 40keV, 70keV and mixed images for colitis. A good inter-observer agreement for all subjective criteria was found with a kappa index higher than 0.86. The VMI at 60keV presented higher scores for all criteria for bowel obstruction and colitis with no significant difference in smoothing score compared to mixed images ($p=0.119$ and $p=0.888$, respectively).
**Conclusion:** VMI at 60keV could provide better image quality compared with other low monoenergetic levels and mixed images for the diagnosis of bowel obstruction and colitis.
**Limitations:** Monocentric and retrospective study.
**Ethics committee approval:** No information was provided by the submitter.
**Funding for this study:** No funding was received for this study.
**Author Disclosures:**
Jean Goupil: Nothing to disclose
Loris Lahuna: Nothing to disclose
Jean-Paul Beregi: Nothing to disclose
Julien Frandon: Nothing to disclose
Joel Greffier: Nothing to disclose
Djamel Dabli: Nothing to disclose

### RPS 917-3

**Acute septic shock diagnostics using iodine uptake of adrenal glands on spectral CT**

*P. Langguth*[1], S. Aludin[1], A. Horr[1], G. Campbell[2], M. Salehi Ravesh[1], N. Larsen[1], M. Both[1], O. Jansen[1], S. Sedaghat[1]; [1]Kiel/DE, [2]Hamburg/DE
*(patrick.langguth@uksh.de)*

**Purpose:** Septic shock is a severe medical emergency with a potentially fatal outcome. The diagnosis is predominantly made clinically, but imaging techniques such as computed tomography can be an important diagnostic and prognostic tool in this regard. New techniques such as spectral-detector computed tomography (SDCT) allow imaging of organ perfusion based on quantification of iodine-containing contrast agent. In the present study, we investigated whether biomarkers for septic shock can be determined in patients.
**Methods or Backround:** Ninety-five patients who underwent venous abdominal CT on SDCT were divided into two groups: A - septic shock (27); B - no septic shock (68). Two blinded radiologists independently assessed density (Hounsfield Units, HU) and iodine concentrations (mg/ml) in defined abdominal organs.
**Results or Findings:** A total of 1520 measurements of iodine concentration and HU were performed. Iodine concentration and HU showed a significant correlation in all organs, with good inter-rater reliability for HU ($\kappa = 0.773$; $p<0.01$) and very good for iodine concentration ($\kappa = 0.8$; $p<0.01$). For adrenal glands, iodine concentration was significantly higher in group A compared to group B ($p<0.01$). In group A, patients showed a particularly high iodine concentration of >3.5 mg/ml (sensitivity=0.926, specificity=0.849, AUC=0.951) in one or/and of >7 mg/ml in both adrenal glands (sensitivity=0.889, specificity=0.836, AUC=0.928). Compared to the adrenal glands, there was no significant difference between groups for either HU or iodine concentration in the other organs.
**Conclusion:** Adrenal iodine concentration assessed by SDCT represents an imaging biomarker in the differentiation of patients with and without septic shock. An iodine concentration of >3.5 mg/ml in one adrenal gland or a total of >7 mg/ml in both adrenal glands can reliably differentiate between these two conditions.
**Limitations:** Small number of septic shock patients.
**Ethics committee approval:** No information was provided by the submitter.
**Funding for this study:** No funding was received for this study.
**Author Disclosures:**
Graeme Campbell: Nothing to disclose
Patrick Langguth: Nothing to disclose
Sam Sedaghat: Nothing to disclose
Marcus Both: Nothing to disclose
Mona Salehi Ravesh: Nothing to disclose
Schekeb Aludin: Nothing to disclose
Agreen Horr: Nothing to disclose
Olav Jansen: Nothing to disclose
Naomi Larsen: Nothing to disclose

### RPS 917-4

**Analysis of the utilisation of abdominal radiography in the emergency department: appropriateness, interpretation, radiation protection and costs**

*P. García García*, E. Paz Calzada, C. Alonso Rodríguez, L. Del Campo Del Val, I. Salmerón Béliz, P. Garcia Castañon, R. Rosado del Castillo, P. Rodríguez Carnero; Madrid/ES
*(patriciagarc@hotmail.com)*

**Purpose:** The purpose of the study was to audit the utilisation and appropriateness of AXR in the emergency department (ED) of a tertiary referral hospital.
**Methods or Backround:** We retrospectively reviewed all AXR performed in the ED of our centre in January 2020 (before the COVID-19 pandemic), including a rigorous review of the patient's medical records, AXR indication and technical quality, appropriateness according to ACR Appropriateness Criteria ® and the Spanish Society of Radiology Appropriateness Guidelines, presence of a formal radiology report, and impact on the patients' management. We also reviewed the radiation dose received by the patients in terms of kerma area product (KAP). The IRB of our centre approved the study.
**Results or Findings:** We obtained 429 AXRs (9.1% of all radiographs carried out in the ED). The most frequent indication was abdominal pain (40% n=176), followed by low back pain (21.4% n=92). 12.4% of the AXR did not have any clinical information in the request at all, and 79.6% had sufficient technical

Thursday quality. Appropriateness was inadequate in most AXR (60.8% n=261). In 84.6% of the cases (n=363), the information disclosed by the AXR did not produce any change in the patient's management. 5% of the inadequate AXR changed patient management, compared with 30% of adequate AXR (p<0.001). Only 3% of the AXR had a formal radiology report. Inappropriate AXR implied unjustified radiation (median KAP of 2.3 Gy.cm2), and also an estimated cost overrun of 6,500 €.
**Conclusion:** The AXR is still common in the emergency setting, although most of them might be inadequate and are not formally interpreted by a radiologist. Its use should be optimised to avoid unnecessary radiation and costs. Radiologists must have a more active participation in the management of plain radiographs.
**Limitations:** Retrospective study.
**Ethics committee approval:** Ethics committee Hospital Universitario La Princesa.
**Funding for this study:** No funding was received for this study.
**Author Disclosures:**
Isabel Salmerón Béliz: Nothing to disclose
Pablo Rodríguez Carnero: Nothing to disclose
Rodrigo Rosado del Castillo: Nothing to disclose
Celia Alonso Rodríguez: Nothing to disclose
Patricia García García: Nothing to disclose
Lourdes Del Campo Del Val: Nothing to disclose
Elena Paz Calzada: Nothing to disclose
Paula Garcia Castañon: Nothing to disclose

### RPS 917-5

**CT-angiography in acute bleeding: is a non-enhanced scan necessary?**

*J. Bremm*, J. Henze, D. Maintz, D. Pinto dos Santos; Köln/DE

**Purpose:** Active bleeding can result in life-threatening conditions. In clinical routine, CT-angiography is frequently used to locate its source. In many centres, the non-contrast scan is considered part of the clinical standard. The aim of this study was to clarify the added value of non-contrast CT in the detection of haemorrhage and provide evidence for or against its usage.
**Methods or Backround:** We retrospectively evaluated all patients that received CT angiography in case of suspected bleeding between 2014 and 2020 and had interventional angiography within 6 hours after the CT scan. CT scans were acquired with non-enhanced, arterial and portalvenous phase. Two experienced observers re-evaluated the CT scans and established a consensus on whether the bleeding could confidently be detected without the non-enhanced CT scan. Additionally, we recorded data on radiation exposure of the respective CT series.
**Results or Findings:** We were able to include 77 unique patients in the final dataset. Most detected bleedings were extraluminal (44 = 57.1%). After consensus reading, the non-enhanced CT scan was deemed necessary or desirable in only 4 cases (5.2%). Non-enhanced series contributed substantially to the overall radiation exposure (mean DLPunenhanced 934.7 mGy*cm, mean DLParterial 634.0 mGy*cm, mean DLPvenous 922.1 mGy*cm).
**Conclusion:** Our results suggest that in the vast majority of cases, non-enhanced CT is not necessary to detect haemorrhage. The omission of non-enhanced CT results in relevant dose reduction and therefore is beneficial for patients. Modern technology, such as dual energy or spectral imaging with the possibility of virtual non-contrast images, may provide additional possibilities to safely omit non-enhanced scans in all patients with suspected bleeding.
**Limitations:** In a few cases, a non-enhanced scan can be helpful to exclude false positive findings, particularly in suspected gastrointestinal bleeding. Careful patient selection should therefore be considered.
**Ethics committee approval:** No information provided by the submitter.
**Funding for this study:** No funding was received for this study.
**Author Disclosures:**
Daniel Pinto dos Santos: Research/Grant Support: Cook Medical
David Maintz: Nothing to disclose
Johannes Bremm: Nothing to disclose
Jörn Henze: Nothing to disclose

### RPS 917-6

**Abdominal CT in non-traumatic emergencies: is contrast material really necessary?**

*D. Poretti*[1], T. De Giorgi[1], G. Ferrillo[1], V. Pedicini[2], F. Mrakic-Sposta[2], M. Profili[2], E. Lanza[2], M. Francone[2], L. S. S. Politi[2]; [1]Milan/IT, [2]Rozzano/IT
*(daporetti@gmail.com)*

**Purpose:** We assessed the diagnostic performance of non-contrast CT compared to single-phase post-contrast CT images for the evaluation of acute traumatic abdominal pathologies.
**Methods or Backround:** 150 non-contrast CT scan images were independently reviewed by one senior radiologist with 20 years of experience and one senior radiology resident and compared with the diagnosis expressed by the reporting radiologist on a post-contrast CT scan. Each diagnosis was also compared to the final diagnosis (retrieved from the hospital database). Results were evaluated for the whole set of cases and also subdivided into different diagnosis groups to assess diagnostic performance in each subgroup.
**Results or Findings:** Concordance between SRR and RR was 95,33%, concordance between SRR and SR was 96%, concordance between SRR and RR was 94%, concordance of SRR and SR with FD was 94%, concordance between RR and FD was 96.8%. Accuracy for diverticulitis proved very high, with 87% sensitivity and 100% specificity, and for appendicitis, 90% sensitivity and 100% specificity. The main diagnoses in our group were diverticulitis, appendicitis, renal colic, and inflammatory bowel disease.
**Conclusion:** Non-contrast CT has proven very effective compared to post-contrast CT in achieving the correct diagnosis in the most frequent pathologies met in an average Emergency Department.
**Limitations:** This is a retrospective analysis based on a limited number of cases. The range of diagnoses in our group was rather limited and may reduce the significance of our conclusions when applied in different populations.
**Ethics committee approval:** No information provided by the submitter.
**Funding for this study:** No funding was received for this study.
**Author Disclosures:**
Vittorio Pedicini: Nothing to disclose
Teresa De Giorgi: Nothing to disclose
Letterio Salvatore Salvatore Politi: Nothing to disclose
Giuseppe Ferrillo: Nothing to disclose
Manuel Profili: Nothing to disclose
Ezio Lanza: Nothing to disclose
Marco Francone: Nothing to disclose
Dario Poretti: Nothing to disclose
Federica Mrakic-Sposta: Nothing to disclose

### RPS 917-7

**Abdominal computed tomography parameters for planning of endoscopic drainage of acute cholecystitis unfit for surgery**

*D. Colombi*[1], G. A. R. Adebanjo[2], A. Cominardi[1], M. Petrini[1], G. Aragona[1], N. Sverzellati[2], E. Michieletti[1]; [1]Piacenza/IT, [2]Parma/IT
*(colombidavide@gmail.com)*

**Purpose:** To detect possible predictors at abdominal computed tomography (CT) of transgastric or transduodenal endoscopic drainage of acute cholecystitis in patients unfit for surgery.
**Methods or Backround:** Patients admitted to the emergency department (ED) of our hospital between May 2020 and September 2022 with acute cholecystitis unfit for surgery who underwent previous abdominal CT within 5 days prior to endoscopic drainage were included in the study. Endoscopic drainage was performed by a gastroenterologist who positioned under EUS guidance a lumen apposing metal stent (LAMS) between gastric or duodenal lumen and gallbladder (GB) lumen. At CT we evaluated with the use of multiplanar reconstruction the minimum distance between gastric antrum or duodenum and GB, GB wall thickness, GB size (long axis and short axis), the stones presence, and the occurrence of collection or free fluid near the GB. Multivariate logistic regression was used to assess potential predictors of transgastric procedure.
**Results or Findings:** Twenty-eight patients (median age 84 years, IQR 78-88 years; females 15/28, 54%) were finally included in the study. Technical success was achieved in 26/28 (93%) cases. GB drainage with transgastric approach was used in 19/28 (68%) patients. At multivariate logistic regression analysis a distance between GB and gastric antrum<8mm (OR 31.5; 95% CI 1.6-619.5; P=0.023) and the presence of free fluid near GB (OR 15.9; 95% CI 1.06-240.8; P=0.045) were significant predictors of transgastric approach against transduodenal.
**Conclusion:** A distance between GB and gastric antrum <8mm and the presence of free fluid near GB at CT favours the transgastric approach for GB drainage in patients with acute cholecystitis unfit for surgery.
**Limitations:** Small sample, retrospective analysis from a single hospital.
**Ethics committee approval:** No information provided by the submitter.
**Funding for this study:** No funding was received for this study.
**Author Disclosures:**
Emanuele Michieletti: Nothing to disclose
Giovanni Aragona: Nothing to disclose
Ganiyat Adenike Ralitsa Adebanjo: Nothing to disclose
Marcello Petrini: Nothing to disclose
Davide Colombi: Nothing to disclose
Nicola Sverzellati: Nothing to disclose
Anna Cominardi: Nothing to disclose

**RPS 917-8**

**Quantification of adipose tissue as a predictor of major injury in blunt abdominal trauma: preliminary results**

*C. Valle*[1], P. A. Bonaffini[1], A. Arrigoni[2], M. Bonetti[3], G. Dizdari[3], G. Muscogiuri[4], P. Marra[1], R. Cosentini[5], S. Sironi[1]; [1]Bergamo/IT; Milan/IT, [2]Bergamo/IT, [3]Dalmine/IT, [4]Milan/IT, [5]Bergamo/IT, Milan/IT
*(clarissa.valle1987@icloud.com)*

**Purpose:** To test the possible correlation between the amount of abdominal adipose tissue and the occurrence of major abdominal lesions after blunt high-energy trauma.
**Methods or Backround:** 25 patients admitted to the Emergency Department due to blunt high-energy trauma were included in the study and clustered in 4 groups according to their abdominal injury grading based on The American Association for the Surgery of Trauma (AAST) injury scoring scales: 1) no lesions (n=6), 2) one organ with injury grade ≤3 (n=6), 3) multiple organs with injury grade ≤3 (n=7), 4) at least one organ with injury grade >3 (n=6). The adipose tissue was segmented on abdominal axial CECT scans slice-by-slice and classified into superficial (SAT), visceral (VAT), and muscular (MAT) using a graph-based algorithm. The adipose tissue masks were first generated by image intensity thresholding; left/right in-body ends were selected and connected following the shortest path computed by the Dijkstra algorithm, penalizing adipose tissue and the central body area. Manual editing was performed before quantification. Total adipose tissue, SAT, VAT and MAT were compared across groups by Wilcoxon Signed Rank (pairwise) and Kruskal-Wallis tests (overall comparison).
**Results or Findings:** Group 1 showed higher VAT and SAT than the other groups. The pairwise tests were significant in VAT between classes 1 vs 3 and 4 (p1-3=0.015, p1-4=0.014); and for SAT between classes 1 vs 2 and 3 (p1-2=0.009, p1-3=0.014).
**Conclusion:** Our results suggest a significant correlation between the abundance of abdominal adipose tissue and the lesser occurrence of major injuries after blunt high-energy trauma.
**Limitations:** Retrospective and monocentric design. Limited number of cases.
**Ethics committee approval:** The study was conducted following the Declaration of Helsinki. The patient's anonymity was granted.
**Funding for this study:** No funding was received for this study.
**Author Disclosures:**
Sandro Sironi: Nothing to disclose
Alberto Arrigoni: Nothing to disclose
Roberto Cosentini: Nothing to disclose
Giorgia Dizdari: Nothing to disclose
Clarissa Valle: Nothing to disclose
Paolo Marra: Nothing to disclose
Pietro Andrea Bonaffini: Nothing to disclose
Matteo Bonetti: Nothing to disclose
Giuseppe Muscogiuri: Nothing to disclose

**RPS 917-9**

**Importance of follow-up CT in blunt splenic trauma**

*J. Cuetos Fernandez*, G. Gabilondo, G. Arenaza Choperena, N. Lizarraga Oroz, L. Cavero Barreras, P. G. Aguinagalde Vives, O. Iñarra, M. I. Blanco, A. Ugarte; San Sebastian/ES
*(javier.cuetosfernandez@osakidetza.eus)*

**Purpose:** To establish a follow-up protocol in high-energy splenic trauma in order to identify late-onset injuries to minimize the risks of delayed splenic rupture and the high mortality associated with it.
**Methods or Backround:** A retrospective observational study was carried out identifying all the cases of splenic injury in blunt abdominal trauma admitted at our centre between 2012 and 2020. 116 patients were identified. After applying the exclusion criteria, the sample was reduced to 70 patients. These studies were reviewed by an expert radiologist. A new reading was made both of the admission CT as well as, if any, of the control CT. The appearance of acute new vascular lesions in the control CT was evaluated.
**Results or Findings:** After the initial analysis of the 116 cases by a specialist in the field, the diagnosis of splenic vascular injury increased by 11% (13 cases). In addition, the control CT demonstrated new vascular lesions in 20 of the 70 patients that met the inclusion criteria.
**Conclusion:** The spleen is the most frequently injured organ in blunt abdominal trauma. The natural evolution of splenic injuries is uneven, and late vascular injuries may appear. It is well known that these injuries can trigger delayed splenic rupture in up to 5% of the cases with an associated mortality of 5-15%. The interpretation of the studies by a professional with experience in the subject, as well as carrying out a control 48 hours after the initial study are key aspects in reducing the risk of delayed rupture and the adequate treatment of these injuries.
**Limitations:** The implementation of the control CT in our centre was carried out in 2015, so previously only those patients with poor evolution underwent a control CT.
**Ethics committee approval:** No information provided by the submitter.
**Funding for this study:** No funding was received for this study.
**Author Disclosures:**
Paula Gabriela Aguinagalde Vives: Nothing to disclose
Nahia Lizarraga Oroz: Nothing to disclose
Laura Cavero Barreras: Nothing to disclose
Gorka Arenaza Choperena: Nothing to disclose
Javier Cuetos Fernandez: Nothing to disclose
Marina Isabel Blanco: Nothing to disclose
Oihane Iñarra: Nothing to disclose
Ane Ugarte: Nothing to disclose
Gorka Gabilondo: Nothing to disclose

**12:30-13:30 Research Stage 3**

### Research Presentation Session: Musculoskeletal

### RPS 910

### Musculoskeletal: image guided interventions

**Moderator**
A. S. Klauser; Innsbruck/AT

**RPS 910-2**

**Effect of biopsy length on the success of the CT-guided core needle biopsy of the musculoskeletal lesions**

F. Inci, *K. Ghali Gataa*, P. Szaro, M. Geijer; Gothenburg/SE
*(khaldunghali@yahoo.com)*

**Purpose:** Computed tomography (CT)-guided percutaneous needle biopsy of the musculoskeletal lesions is a well-described technique for determining the nature of indeterminate lesions. The purpose of study is to investigate the effect of sample length on the biopsy success of CT-guided musculoskeletal core biopsies.
**Methods or Backround:** A total of 428 CT-guided biopsies for musculoskeletal lesions which are performed at Sahlgrenska University Hospital in the last six years were retrospectively analysed. The accuracy of the biopsies was evaluated by comparing the final diagnosis with the biopsy results for patients who underwent surgery or with six-month clinical and radiological follow-up findings for patients who did not have surgery. The radiological features of each lesion, the length of biopsy material, the pathology results, and the patient demographic data were statistically analysed with a chi-square test for their influence on the success of the biopsy.
**Results or Findings:** The total success rate of CT-guided percutaneous needle biopsy of the musculoskeletal lesions was 89 per cent (383/428). Biopsy samples varied från 2 mm to 109 mm. Sample length had no effect on the adequacy of the obtained material or the success of the biopsy (p =0.82).
**Conclusion:** CT-guided musculoskeletal core biopsies are an effective procedure for the diagnosis of suspicious musculoskeletal lesions. There is, however no association between the length of the sample taken and the success of the CT-guided musculoskeletal core biopsies.
**Limitations:** The major limitation is that the study has a retrospective methodology.
**Ethics committee approval:** Swedish Ethical Review Authority Dnr-00466
**Funding for this study:** ALF-Agreement.
**Author Disclosures:**
Fatih Inci: Nothing to disclose
Mats Geijer: Nothing to disclose
Khaldun Ghali Gataa: Nothing to disclose
Pawel Szaro: Nothing to disclose

**RPS 910-3**

**Relation between success rate and number of biopsy attempts of CT-guided bone core needle biopsy in the musculoskeletal system using a 13-gauge needle**

*K. Ghali Gataa*, F. Inci, P. Szaro, M. Geijer; Gothenburg/SE
*(khaldunghali@yahoo.com)*

**Purpose:** The study aims to determine if the number of biopsy attempts with a 13-G needle during a biopsy procedure affects the biopsy's success.
**Methods or Backround:** The retrospective analysis of CT-guided MSK biopsies was done at the Sahlgrenska University Hospital in Gothenburg between 2016 and 2021. Six months of clinical and radiological follow-up or results from a pathological anatomical analysis (PAD) served as outcome reference. The biopsy was classified as "diagnostic"' when a definitive diagnosis could be made by PAD and "accurate" when only the tumour's malignant or benign nature could be determined by PAD or at least a 6-month follow-up was done. Non-accurate biopsy was considered if neither the malignant nor the benign nature of the tumour could be determined. Procedures where a number of attempts were not registered, were excluded. A p-value <0.05 was considered statistically significant.
**Results or Findings:** The study included 428 biopsies of MSK-lesions; diagnostic biopsies n= 314 (73%), accurate biopsies n=69 (16%) and non-accurate biopsies n=45 (11%). In n=218 (51%) cases, only one biopsy was taken with n=149 (68%) diagnostic biopsies; n=41 (19%) with accurate biopsies. In n=123 (29%) cases, two biopsies were done with n=97 (79%) diagnostic biopsies and n=16 (13%) with accurate biopsies. In n=51 (12%) cases, three biopsies were done with n=40 (78%) diagnostic biopsies and n= 7(14%) with accurate biopsies. In n=24 (5%) cases, four biopsies were done with diagnostic biopsies n=21 (88%) and n=1 (4%) with accurate biopsies. In n=12 (3%) cases, the number of biopsies was not registered, and these biopsies were excluded. A Chi-square test was applied for a number of biopsy attempts vs the success rate, which showed that there is no statistically significant association between the number of biopsy attempts and diagnostic success.
**Conclusion:** The increased number of biopsy attempts per procedure does not increase the diagnostic value of the procedure or the total success rate.
**Limitations:** The study was limited by the few patients who went through 4 biopsies.
**Ethics committee approval:** The ethical approval was granted by the Swedish Ethical Review Authority Dnr 2021–00466.
**Funding for this study:** The study was funded by the ALF Agreement.
**Author Disclosures:**
Fatih Inci: Nothing to disclose
Mats Geijer: Nothing to disclose
Khaldun Ghali Gataa: Nothing to disclose
Pawel Szaro: Nothing to disclose

### RPS 910-4

### Combined ultrasound-guided platelet-rich plasma injection and genicular alcohol neurolysis in knee osteoarthritis cases: new technique, promising results and more to explore

*M. E. E. Mostafa Fouad*[1], A. E. Hanafy[2]; [1]Frankfurt/DE, [2]Cairo/EG
*(dr.mohamed_fouad@hotmail.com)*

**Purpose:** We assessed the combined ultrasound-guided platelet-rich plasma (PRP) injection and genicular alcohol neurolysis (GAN) procedure's efficacy and safety as a minimally invasive management technique for grade III and IV knee osteoarthritis (KOA).
**Methods or Backround:** Fifty-five clinically and radiologically (x-ray grading according to Kellgren and Lawrence system) confirmed KOA diagnosis patients were included. Each received a single session using an ultrasound transducer of 7-15 MHz for knee assessment and guidance. The PRP was prepared from withdrawn blood and then centrifugation in a particular manner. GAN mixture was 2 ml of 1% lidocaine added to 2 ml of ethyl alcohol (70%). Clinical and sonographic assessments were applied at pre, 2 weeks, 1 month and 6 months time intervals. It included VAS, Oxford, WOMAC and LYSHLOM knee scoring systems with knee ultrasound evaluating joint effusion, osteophytes, and synovial thickening. Finally, intra- and post-procedural complications were assessed.
**Results or Findings:** The VAS, OXFORD, WOMAC and LYSHLOM pre- and post-procedural scores showed statistically significant improvements from 80, 29, 63 and 54 to 50, 10, 25 and 81, respectively, throughout the whole duration of P value 0.001. Follow-up ultrasound showed only statistically significant positive changes in joint effusion, which was detected in 69.7% of patients and declined to 18.2% at 6 months of P value 0.001. In comparison, osteophytes and synovial thickening illustrated statistically insignificant changes of P value 0.07. The procedure showed skin burning, contusions and difficulty in walking as postprocedural complications in 33% of patients, which was resolved conservatively within a few days with no sustained or life-threatening events.
**Conclusion:** The procedure illustrated promising safe results regarding the patient's symptoms, lifestyle and satisfaction improvement. However, further studies are required to prove its efficacy in comparison to other treatment modalities.
**Limitations:** The study was limited by the longer follow-up duration and the lack of comparative studies.
**Ethics committee approval:** No information was provided by the submitter.
**Funding for this study:** No funding was received for this study.
**Author Disclosures:**
Aya Essam Hanafy: Nothing to disclose
Mohamed Elsayed Elbadawy Mostafa Fouad: Nothing to disclose

### RPS 910-5

### Dry needling on myofascial trigger points: a better way to treat iliotibial band syndrome

*S. Pasetto*; El Vendrell/ES
*(s.pasetto@gmail.com)*

**Purpose:** The aim of this study was to evaluate the outcomes of patients with iliotibial band syndrome with dry needling (DN) as a primary intervention strategy.
**Methods or Backround:** A total of nine patients with clinical symptoms of iliotibial band syndrome were enrolled for treatment. We examined and reported sonographic findings of all patients before DN treatment (t0), after 1 month (t1) and 2 months (t2). Iliotibial band syndrome manifests as a combination of thickening and hypoechogenicity of the iliotibial band itself or, eventually, discrete fluid collection, suggestive of bursitis, between the iliotibial band and the lateral femoral epicondyle.
**Results or Findings:** The efficacy of DN intervention was measured by the reduction of pain and disability levels and subjective reports of improvement in the subject's overall functional ability and quality of life. Ultrasound is helpful in managing the follow-up and demonstrating the remodelling of the tendinosis area in correlation to the healing process and clinical improvement.
**Conclusion:** DN is an effective treatment for reducing pain associated with ITBS in the short term. The research shows that DN restores blood flow to oxygen-deprived tissue and allows muscles to relax and return to normal functioning.
**Limitations:** Pregnant women, people who are unable to understand the purpose of dry needling and people who are very afraid of needles should not undergo dry needling.
**Ethics committee approval:** No information provided by the submitter.
**Funding for this study:** This research received no specific grant from any funding agency in the public, commercial, or "not-for-profit sectors."
**Author Disclosures:**
Stefano Pasetto: Nothing to disclose

### RPS 910-6

### Intramuscular paradigm shift: MRI shoulder anatomy analysis to establish safer intramuscular injection site

*J. Krawczyk*, D. Amiras, C. Lord, A. D. Wilson, A. Ladas, M. Sarvesvaran; London/UK
*(jejabig.psn@gmail.com)*

**Purpose:** Can we use MRI anatomy to predict the best approach to reduce the risk of shoulder injury related to vaccine administration (SIRVA)?
**Methods or Backround:** We reviewed existing literature to assess risks related to the traditional intramuscular injection site in the middle deltoid target, as well as included imaging examples of pathology related to complications. A cross-section of 80 adult shoulders MRI scans was reviewed. Studies with anatomical variations, fractures or bone destruction were excluded. Using anatomical landmarks, a circular region of interest was drawn on the axial sequences approximately 5 cm below the acromion to simulate a middle deltoid target and a further ROI was drawn in a similar fashion targeting the posterior deltoid. These were then compared. The volume of muscle, subcutaneous fat and volume of special structures, such as nerves, tendons etc., were included.
**Results or Findings:** A middle and posterior deltoid approach showed an anatomical difference in the volume of muscle in the region of interest.
**Conclusion:** A posterior deltoid target may reduce the risk of non-muscular injection, which is of particular importance in light of mass COVID-19 vaccination, as well as the widespread use of deltoid targets as a site for intramuscular injection in general.
**Limitations:** Paradigm might be difficult to change.
**Ethics committee approval:** -
**Funding for this study:** Not applicable.
**Author Disclosures:**
Jędrzej Krawczyk: Nothing to disclose
Dimitri Amiras: Nothing to disclose
Andreas Ladas: Nothing to disclose
Angus Douglas Wilson: Nothing to disclose
Christopher Lord: Nothing to disclose
Matthew Sarvesvaran: Nothing to disclose

**RPS 910-7**

**Randomised controlled trial comparing diagnostic adequacy of 8G versus 11G bone biopsy needle in patients of tubercular spondylitis (TBS)**

*P. Aggarwal*, D. N. Srivastava, K. S. Madhusudhan, A. Goyal, S. Pandey; New Delhi/IN
*(piyush.1994.pa@gmail.com)*

**Purpose:** To compare the diagnostic value of 8G and 11G bone biopsy needles in tuberculous spondylitis (TBS).
**Methods or Backround:** Fifty patients (mean age 40 years; males–27) suspected of TBS based on clinical and MRI features underwent transpedicular biopsies under fluoroscopic guidance with 8G or 11G needles after randomisation. The samples were evaluated on histology (chronic inflammation, granulomas, caseous necrosis and giant cells), GeneXpert, PCR and liquid cultures and categorised into diagnostic, non-specific and inadequate groups. Procedure-related complications were recorded.
**Results or Findings:** There were 25 patients in each group. One patient developed pneumothorax during the procedure in the 8G group. Of the three microbiological tests (GeneXpert, PCR, Liquid culture), at least one test was found to be positive in 32%(n=8) of the 8G group and 24%(n=60) of 11G group samples (p>0.05). The histology report was "Suggestive" of TB in 40%(n=10) of the 8G group and 36%(n=9) of the 11G group samples (p-value-0.88). Overall, the samples in 8G and 11G groups were diagnostic in 13 and 12 patients, non-specific in 10 and 9 patients, and inadequate in 2 and 4 patients, respectively (p=0.62).
**Conclusion:** Although biopsy samples were more frequently diagnostic in the 8G biopsy needle group, the difference recorded was not statistically different from the 11G biopsy needle group.
**Limitations:** More significant differences could have surfaced with a larger sample size.
**Ethics committee approval:** Institute Ethics Committee (AIIMS, New Delhi)
**Funding for this study:** The cost of the procedure and needle was borne by the patient. No financial funding was received from any pharmaceutical company.
**Author Disclosures:**
Saumya Pandey: Nothing to disclose
Piyush Aggarwal: Nothing to disclose
Ankur Goyal: Nothing to disclose
Deep Narayan Srivastava: Nothing to disclose
K S Madhusudhan: Nothing to disclose

**RPS 910-8**

**Is it possible to predict response to knee injections using Behavioral Activation and Behavioral Inhibition scales (BAS/BIS)?**

C. T. Rodrigues[1], *G. M. Allen*[2], D. Wilson[2]; [1]Coimbra/PT, [2]Oxford/UK
*(gina@slro.co.uk)*

**Purpose:** The aim of this study was to evaluate the response of patients to knee injections (steroid/hyaluronic acid/combined) using visual pain scales and clinical information regarding their individual scores in Behavioral Activation (BAS) and Behavioral Inhibition scales (BIS).
**Methods or Backround:** Behavioral Activation and Behavioral Inhibition Scales are internationally recognized measures of response to pain. This study included 37 patients who underwent knee injections between April 2021 and September 2022. All patients had BAS Drive, BAS Fun Seeking, BAS Reward, and BIS scores assessed. Participants also completed pain diaries, including visual pain scores and clinical information on their daily activity, sporting activities and painkiller use. Demographic data and previous treatments were also included.
**Results or Findings:** The average age of the group was 64.2, including 24 females (65%) and 13 males (35%). Patients who had a positive therapeutic response in visual pain scales after two weeks presented with average BAS scores higher than patients with no therapeutic response (BAS Reward: 15.5 vs 14.1; BAS Fun Seeking: 10.6 vs 9.3; BAS Drive: 9.82 vs 9). BIS scores were on average lower in patients with therapeutic response compared to patients with no therapeutic response (19.3 versus 20.9).
**Conclusion:** Integrating BAS and BIS scores into pretreatment evaluation of patients, for example through online questionnaires, could help decide which patients have a higher probability of therapeutic response to knee injections, integrating a personalized view of musculoskeletal interventional radiology procedures.
**Limitations:** The size sample was limited by non-compliance with pain diaries. Pain diaries were completed over a maximum of two weeks. There are no control patients.
**Ethics committee approval:** No information provided by the submitter.
**Funding for this study:** This study was funded by St Lukes Radiology Oxford Ltd.
**Author Disclosures:**
David Wilson: Nothing to disclose
Georgina Marian Allen: Nothing to disclose
Carolina Terra Rodrigues: Nothing to disclose

**12:30-13:30** **Research Stage 4**

### Research Presentation Session: Oncologic Imaging

### RPS 916

### Clinically relevant imaging for tumour detection or staging

**Moderator**
S. Gourtsoyianni; Athens/GR

**RPS 916-2**

**Radiomics analysis of whole-body low-dose CT in patients with multiple myeloma: correlation with bone marrow and patient outcome**

*R. Cannella*[1], A. Fidenco[1], E. Gigliotta[1], S. Siragusa[1], F. Vernuccio[2], C. Botta[1]; [1]Palermo/IT, [2]Padova/IT

**Purpose:** To assess the correlation between radiomics features extracted from whole-body low-dose CT (WBLDCT), disease stage and prognosis in patients with multiple myeloma.
**Methods or Backround:** This study included adult patients who underwent WBLDCT for the staging of multiple myeloma and bone marrow biopsy. A total of 35 radiomics features were extracted by placing a three-dimensional region of interest in the iliac bone on the same side and location of the bone marrow biopsy. Complete blood cells count, laboratory tests, disease stage as well as the percentage of clonal plasma cells in the bone marrow were included in the analysis. Progression-free survival (PFS) at first-line therapy was assessed for the survival analysis with Kaplan-Mayer curves.
**Results or Findings:** The study population included 109 patients (median age 72 years), of which 59 had newly diagnosed multiple myeloma. A total of five Grey Level Co-occurrence Matrix (GLCM) features were significantly different among disease stages, and they were able to discriminate between monoclonal gammopathy of undetermined significance, smouldering multiple myeloma and multiple myeloma (p<0.001). In the bone marrow, there was a significant difference in radiomics features distribution according to the type of T lymphocytes and monocytes (p<0.05). Unsupervised hierarchical clustering identified two different groups of patients according to the distribution of radiomic features with a significantly different PFS (p=0.021).
**Conclusion:** Radiomics analysis of whole-body low-dose CT could help to improve disease staging and predict treatment response in patients with multiple myeloma.
**Limitations:** This was a single-centre, retrospective study.
**Ethics committee approval:** No information was provided by the submitter.
**Funding for this study:** No funding was received for this study.
**Author Disclosures:**
Federica Vernuccio: Nothing to disclose
Sergio Siragusa: Nothing to disclose
Cirino Botta: Nothing to disclose
Alessandro Fidenco: Nothing to disclose
Roberto Cannella: Other: Bracco, Bayer: support for attending meetings.
Emilia Gigliotta: Nothing to disclose

**RPS 916-3**

**Value of new CT signs in T-staging of resectable oesophagal carcinoma**

*Y. Wang*, J-z. Wang, H. Zhong, X-s. Shang, Y. Huang, W-y. Zhang, S. Dong; Jinan/CN
*(kongbeixiaobao@163.com)*

**Purpose:** To investigate the contrast-enhanced computed tomography (CT) findings of resectable oesophagal squamous cell carcinoma (ESCC) in different T stages.
**Methods or Backround:** We retrospectively analyzed 1540 patients with ESCC who underwent surgical treatment in the Shandong Cancer Hospital and Institute between July 2014 and January 2022. Image evaluation and measurement of oesophagal wall thickness (EWT) of ESCC were performed by two thoracic radiologists in consensus. The contrast-enhanced CT manifestations of ESCC in different T stages were summarized by five signs, including invisible lesions, mucosal enhancement, non-circumferential wall thickening, circumferential wall thickening and external invasion.
**Results or Findings:** The CT findings of 1540 patients with ESCC were as follows: there were 317 cases with invisible lesions, of which 98% (310/317) were pTis-T1 stage; there were 39 cases with mucosal enhancement, of which 72% (28/39) were pT1b stage; there were 504 cases with non-circumferential wall thickening, of which 54% (270/504) were pT2 stage; there were 680 cases with circumferential wall thickening, of which 91% (622/680) were pT3-4a stage. Of the pTis-T1 cases, 75% (310/412) showed invisible lesions, 8% (33/412) showed mucosal enhancement, 17% (69/412) showed noncircumferential wall thickening, and 82% (338/412) had an EWT less than 5.5 mm. Of the pT2 cases, 79% (270/341) showed non-circumferential wall thickening, 17% (58/341) showed circumferential wall thickening, and 67% (230/341) had an EWT between 5.5 mm and 10.8 mm. Of the pT3-4a cases, 79% (622/787) showed circumferential wall thickening, 19% (150/787) showed non-circumferential wall thickening, 71% (560/787) had an EWT greater than 10.8 mm, and 46% (365/787) had an irregular outer contour.
**Conclusion:** The CT manifestations of stage pTis-T4a ESCC have their own characteristics, which provide useful clues for the T-staging of ESCC.
**Limitations:** Patients with stage T4b ESCC were not included in this study.
**Ethics committee approval:** The ethical approval number is 2020003027.
**Funding for this study:** This study is supported by the Natural Science Foundation of Shandong Province (ZR2019PH115).
**Author Disclosures:**
Shuai Dong: Nothing to disclose
Wen-yu Zhang: Nothing to disclose
Xiao-sha Shang: Nothing to disclose
Jin-zhi Wang: Nothing to disclose
Hui Zhong: Nothing to disclose
Yue Wang: Nothing to disclose
Yong Huang: Nothing to disclose

### RPS 916-4

**Preliminary results of 68Ga-FAPI PET CT and ADC comparison in patients with gastric cancer**

O. M. Topcuoğlu, G. Beydagi, A. Görmez, E. Demirci, N. Alan Selcuk, *T. Orhan*; Istanbul/TR

**Purpose:** To evaluate 68Ga-FAPI PET CT and apparent diffusion coefficient (ADC) in patients with gastric cancer.
**Methods or Backround:** Fibroblast activation protein inhibitors (FAPI) were developed to be used as radiotracers with DOTA-containing ligand (68Ga-FAPI). On PET images, increased 68Ga-FAPI uptake for tumour tissues and low background uptake for normal healthy tissues were identified, resulting in high contrast resolution and increased tumour detectability. Low background uptake is an important advantage of 68Ga-FAPI PET CT over 18 fluorodeoxyglucose (18F-FDG) PET CT. However, the uptake of non-tumoural healing tissues and matrix remodelling processes are major drawbacks. Patients with pathologically proven gastric cancer who accepted both FAPI and MR imaging methods were prospectively enrolled. Increased 68Ga FAPI uptake, accompanied by density change in the corresponding sites on CT, was accepted as positive. ROIs were drawn on the axial plane ADC maps for the corresponding lesions of increased 68Ga FAPI uptake, and SUVmax and ADC values were recorded for each tumour. Spearman's rank correlation was calculated for SUVmax and ADC.
**Results or Findings:** Eleven patients (5 men and 6 women) with a mean age of 57.9±11.7 years (range 37 to 81 years) met the inclusion criteria. Mean SUVmax and ADC were 13.4±4.6 and 0.79±0.13 s/mm2, respectively. All 68Ga FAPI positive lesions showed diffusion restriction. There was no significant positive or negative correlation between FAPI SUVmax and ADC values (P=0.212). Interobserver agreements were perfect and substantial for SUVmax and ADC, respectively (Kappa=0.83 and 0.75, respectively).
**Conclusion:** 68Ga-FAPI PET CT is a new and promising imaging method for conventional PET CT negative tumours. However, although there was no significant correlation between FAPI SUVmax and ADC, all FAPI positive gastric tumours show restricted diffusion in this series.
**Limitations:** The small sample size is the main limitation.
**Ethics committee approval:** No information was provided by the submitter.
**Funding for this study:** No funding was received for this study.
**Author Disclosures:**
Tolga Orhan: Nothing to disclose
Gamze Beydagi: Nothing to disclose
Emre Demirci: Nothing to disclose
Ayşegül Görmez: Nothing to disclose
Osman Melih Topcuoğlu: Nothing to disclose
Nalan Alan Selcuk: Nothing to disclose

### RPS 916-5

**The accuracy of surgeons predicting the extent of abdominal surgeries with MRI**

C. Rijsemus, N. Kok, A. Aalbers, B. Grotenhuis, D. M. J. Lambregts, R. G. H. Beets-Tan, *M. Lahaye*; Amsterdam/NL

**Purpose:** If colorectal cancer (CRC) patients are eligible for cytoreductive surgery and hyperthermic intraperitoneal chemotherapy (CRS-HIPEC) is discussed during a multidisciplinary meeting. Surgeons decide, based on clinical and radiological findings, what structures will need to be resected to achieve a complete resection. The accuracy of surgeons predicting the extent of surgery with MRI has not yet been investigated. Therefore this study aims to determine the accuracy of surgeons predicting the extent of surgery based on MR images.
**Methods or Backround:** CRC patients with peritoneal metastases (PM) who underwent a CRS-HIPEC and a pre-operative MRI were included. All MRIs were viewed during a meeting with three experienced surgeons who independently predicted the probability of the resection of 34 abdominal structures to achieve complete resection. Other predicted variables were additional specialists necessary and stoma post-CRS-HIPEC. Surgical/histopathological reports were reviewed to score the actual resected structures.
**Results or Findings:** In the 25 patients, the intraclass correlation between the surgeons was excellent (0·92 (0·82- 0·97)). In 75% of all resected structures (165), viable tumour tissue was found in the histopathological examination. Overall positive predictive values (PPV) for the 34 anatomical structures ranged between 54% and 100% [except for abdominal wall (40%) and spleen (20%)]. Ten of the 34 structures had a PPV of 100%. Overall, NPVs ranged between 55.6% and 100%. The PPV of stoma post-HIPEC was 80%, and additional specialists needed 75%.
**Conclusion:** Surgeons can accurately predict the resectability of certain anatomical structures and variables that could be useful in surgical planning and patient expectation management based on MRI findings. However, more tools are necessary perioperatively to distinguish benign from malignant lesions since 25% of the resected structures were not malignant.
**Limitations:** Limitations were the retrospective design and the sample size.
**Ethics committee approval:** IRbd20-226.
**Funding for this study:** No funding was received for this study.
**Author Disclosures:**
Max Lahaye: Nothing to disclose
Doenja Marina Johanna Lambregts: Nothing to disclose
Charlotte Rijsemus: Nothing to disclose
Niels Kok: Nothing to disclose
Brechtje Grotenhuis: Nothing to disclose
Regina G. H. Beets-Tan: Nothing to disclose
Arend Aalbers: Nothing to disclose

### RPS 916-6

**68Ga-PSMA uptake in PET-CT in prostate cancer lesions non-recognizable in conventional CT**

*M. C. Huerta Sanchez*, X. A. G. Ballesteros, A. Hernandez-Alejo, L. A. Alvarez-Lozada, G. Elizondo Riojas, A. Montemayor Martínez; Monterrey/MX
*(drmonicarad@gmail.com)*

**Purpose:** The purpose of the study was to compare the uptake of the radiotracer 68Ga-PSMA in primary, recurrent, or metastatic prostate cancer lesions identified on PET-CT with and without tomographic evidence.
**Methods or Backround:** A cross-sectional study was conducted from October 2017 to July 2021. We included patients diagnosed with prostate cancer with positive PSMA PET-CT scan. The study variables were the indication for PET-CT defined by the referring physician, a recent PSA determination, the SUVmax, and the anatomic location of lesions. Studies were classified as 68Ga-PSMA PET-CT with tomographic evidence and 68Ga-PSMA PET-CT without tomographic evidence.
**Results or Findings:** 340 lesions with tumoural activity were identified: 184 lesions were detected with PET-CT without tomographic evidence and with a mean SUVmax of 13.35 ± 12.29, in contrast to a mean SUVmax of 24.01 ± 25.54 in 156 lesions detected with PET-CT with tomographic evidence (p < 0.001). The mean SUVmax of tumour activity with PET-CT without tomographic evidence was lower than lesions detected with tomographic evidence, regardless of the study indication. Significant differences were seen in patients sent for staging and clinical suspicion of prostate cancer recurrence. 94.2% of the patients had PSA available values with a mean of 37.91 ± 152.04 ng/mL in patients with lesions detected by PET-CT without tomographic evidence, compared to a mean of 92.32 ± 268.87 in patients with lesions with tomographic evidence (p = 0.008).
**Conclusion:** PET-CT without tomographic evidence showed lower radiotracer uptake, suggesting lower tumour volume and earlier detection of incipient lesions in patients with prostate cancer. These results are relevant in the context of the limited availability of PET-CT equipment in Mexico and the urgency to invest resources in equipping medical facilities.
**Limitations:** Our main weaknesses were the cross-sectional design and the lack of histopathological correlation.
**Ethics committee approval:** Not applicable.
**Funding for this study:** No funding was required for this studay.
**Author Disclosures:**
Guillermo Elizondo Riojas: Nothing to disclose
Xavier Alejandro Gonzalez Ballesteros: Nothing to disclose
Alejandro Hernandez-Alejo: Nothing to disclose
Luis Adrian Alvarez-Lozada: Nothing to disclose
Alberto Montemayor Martínez: Nothing to disclose
Monica Catalina Huerta Sanchez: Nothing to disclose

### RPS 916-7

**Identifying pathways for recurrent disease in colorectal cancer patients after cytoreductive surgery and HIPEC**

C. Rijsemus, E. Berardi, N. Kok, A. Aalbers, P. Snaebjornsson, D. M. J. Lambregts, R. G. H. Beets-Tan, *M. Lahaye*; Amsterdam/NL

**Purpose:** Cytoreductive surgery (CRS) and hyperthermic intraperitoneal chemotherapy (HIPEC) patients have a high recurrence rate. No solutions have emerged in the literature to reduce recurrences. This study aims to identify pathways for recurrent disease after CRS-HIPEC in colorectal cancer (CRC) patients with peritoneal metastases (PM) by comparing MRIs before and after surgery.
**Methods or Backround:** CRC patients with recurrence after CRS-HIPEC who had an MRI at primary diagnosis (MRI1, before CRS-HIPEC) and at the time of recurrence (MRI2, after CRS-HIPEC)were included. The MRIs were compared by 2 abdominal radiologists in consensus and the recurrent metastases were categorized as follows: (1) the same site as before surgery and (2) new PM, including abdominal wall metastases and (3) extra-peritoneal metastases. The recurrent metastases on MRI were compared with the histopathological findings of CRS-HIPEC. Reference standards for recurrent metastases were histopathology or follow-up imaging.
**Results or Findings:** Thirty-three patients were included, of which 74 recurrent metastases were depicted on MRI2. Thirty-seven (50%) metastases were visible in the same site as the pre-operative MRI1, and 37 (50%) metastases were found at sites where no metastases were seen on MRI1. In total, 18/74 metastases were found in the abdominal wall, and 17/74 were extra-peritoneal metastases. Seven of the new recurrent metastases had histopathological proof that the lesion was previously present but was missed on MRI1.
**Conclusion:** Recurrent metastases are often found in the same site as pre-operatively and in the abdominal wall. This knowledge suggests that: (1) Surgeons must attempt to achieve a wider excision of metastases during CRS-HIPEC when possible. (2) Radiologists, when reporting follow-up scans, must evaluate in detail the abdominal wall and the sites where there was PM present before CRS-HIPEC.
**Limitations:** Limitations of the study were the retrospective design and the sample size.
**Ethics committee approval:** IRBd20-226.
**Funding for this study:** No funding was received for this study.
**Author Disclosures:**
Eva Berardi: Nothing to disclose
Max Lahaye: Nothing to disclose
Petur Snaebjornsson: Nothing to disclose
Doenja Marina Johanna Lambregts: Nothing to disclose
Charlotte Rijsemus: Nothing to disclose
Niels Kok: Nothing to disclose
Regina G. H. Beets-Tan: Nothing to disclose
Arend Aalbers: Nothing to disclose

### RPS 916-8

**Prognostic significance of FAPI PET/CT vs FDG PET/CT in ovarian neoplasms**

*N. G. A. Niyarah*, K. Kallur, S. Swamy Shivalingappa, M. Ashok Kumar, G. R. Prashanth, A. A. R. Kesari, S. Sampangi; Bangaluru/IN
*(niyarahalemao99@ymail.com)*

**Purpose:** The study aimed to (1) evaluate the efficacy between two tracers in real-time by identifying the SUV max associated with ovarian cancer subtypes and to (2) demonstrate the prognostic significance of FAPI and FDG-PET in ovarian cancer subtypes by calculating MTV, TLG and SUV max.
**Methods or Backround:** The study included 30 patients with ovarian neoplasms who had previously undergone a Ga 68 FAPI and 18 F FDG PET study and a histopathological confirmation to determine the subtype, which included mucinous and serous adenocarcinoma, teratoma and granulosa cell tumours. The SUV max, TLG and MTV of the primary tumour and metastases on baseline scan was obtained on a FAPI and FDG PET scan. The tumour size, lymph nodal, peritoneal and distant metastasis for each histological subtype was recorded on follow-up scans.
**Results or Findings:** Upon evaluation, it was found that the tumour SUVmax, MTV and TLG was significantly higher on the FAPI PET scan in comparison to the FDG PET scan ($p<0.005$), predominantly in mucinous adenocarcinoma. Also, higher SUV values on both scans represented a poor prognosis. It was also seen that the higher the SUV values, the higher was the peritoneal, lymph nodal and solid organ metastasis.
**Conclusion:** In conclusion, it was found that the prognosis of patients with high SUVmax at baseline is significantly worse than that of patients with low SUVmax. Ovarian neoplasm grades showed significant differences in SUVmax, MTV and TLG parameters on each scan.
**Limitations:** Limitations to the study were the case selection bias (institution-based study) and the fact that other PET parameters were not considered.
**Ethics committee approval:** We obtained approval from the institutional ethics committee.
**Funding for this study:** No funding was received for this study.
**Author Disclosures:**
Shivakumar Swamy Shivalingappa: Nothing to disclose
Kumar Kallur: Nothing to disclose
G R Prashanth: Nothing to disclose
Sudhakar Sampangi: Nothing to disclose
Avinash Arjun Rao Kesari: Nothing to disclose
Nadezhda Gloria Alemao Niyarah: Nothing to disclose
Mahesh Ashok Kumar: Nothing to disclose

### RPS 916-9

**The impact of the multidisciplinary tumour board (MDTB) on the management of focal liver lesions in a tertiary referral centre**

*E. Genco*, S. De Vizio, D. Carano, E. Gori, A. Bevere, M. R. P. Carafa, P. Testa, R. Iezzi, M. G. Brizi; Rome/IT

**Purpose:** The implementation of multidisciplinary tumour board (MDTB) meetings significantly improved the management of oncological diseases. However, few pieces of evidence are currently present on the characterization of focal liver lesions and the management of hepatocellular carcinoma (HCC). The aim of this study was to evaluate the impact of the MDTB on the diagnosis and characterization of focal hepatic lesions (in particular HCC), therapeutic choice (surgery, interventional and systemic treatment) and tumour response to oncological treatment compared with indications before the discussion.
**Methods or Backround:** We reviewed the initial diagnosis and multidisciplinary discussions of 758 patients between January 2019 and July 2022. Changes in diagnosis and treatment choice after MDTB discussions were analyzed.
**Results or Findings:** A total of 758 patients were included in the study. Concerning focal liver lesions, characterization diagnosis was changed/modified in 265 out of 758 (35%), of which 151 (20%) with different diagnosis and 114 (15%) with numerical discrepancy. Meanwhile, the diagnosis was confirmed in 493 out of 758 (65%). Regarding treatment choice: 85 (11,2%) patients underwent surgery, single or combined interventional procedures were chosen in 193 (25,4%) patients, and systemic chemotherapy was decided in 123 (16,2%) patients. A follow-up was programmed for 357 (47%) patients. The diagnostic assessment was changed in 265 patients (35%) with consequent modification of subsequent management.
**Conclusion:** MDTB discussion significantly impacts focal liver lesion characterization and HCC management, especially in high-volume centres, with consistent changes in diagnosis, treatment choice and tumour response assessment compared with diagnosis/indications before the discussion.
**Limitations:** No limitations were identified.
**Ethics committee approval:** No information was provided by the submitter.
**Funding for this study:** No funding was received for this study.
**Author Disclosures:**
Elisabetta Gori: Nothing to disclose
Roberto Iezzi: Nothing to disclose
Maria Gabriella Brizi: Nothing to disclose
Silvia De Vizio: Nothing to disclose
Maria Rachele Pia Carafa: Nothing to disclose
Antonio Bevere: Nothing to disclose
Davide Carano: Nothing to disclose
Enza Genco: Nothing to disclose
Priscilla Testa: Nothing to disclose

**14:00-15:30** **Research Stage 1**

### Research Presentation Session: Genitourinary

### RPS 1007

### Imaging role in the diagnosis and prognosis of GU tumours

**Moderator**
T. M. Cunha; Lisbon/PT
*(tmargarida@gmail.com)*

### RPS 1007-2

**Predictive quantitative MDCT models for characterisation of renal cell carcinoma subtypes and differentiation from renal oncocytoma: three phases nomogram approach analysis**

*T. El-Diasty*, H. M. Shebel, D. E. Sharaf, H. Abou El Atta; Mansoura/EG
*(teldiasty@hotmail.com)*

**Purpose:** Our objective is to develop an approach using algorithmic models to discriminate between common solid renal masses, including RCC subtypes and renal oncocytoma (RO), using multiphase computed tomography (CT) with a large sample size.
**Methods or Backround:** We retrospectively analysed a group of solid renal masses between January 2011 and June 2022 regarding the CT attenuation values using a CT scanner with 64 detector and clinical parameters. Inclusion criteria included patients who had four phases of CT with a partial or radical nephrectomy. Exclusion criteria were patients with biphasic or one-phase CT, poor quality, ablation, or indeterminate pathology.
**Results or Findings:** Our sample included 643 cases with a total number of 467 cases following our inclusion criteria, including 195 masses of clear RCC (CRCC), 81 of RO, 124 of chromophobe RCC (ChRCC), and 67 proved to be papillary RCC (PRCC). We analysed our sample size through three analytical phases. There was a significant difference between hypervascular (CRCC and RO), hypovascular (ChRCC and PRCC), AUC= 0.95. The predictive model for differentiation between CRCC from RO showed AUC=0.80. The discrimination of ChRCC from PRCC showed AUC= 0.94. Nomogram was developed for each phase of analysis. The most significant predictors in the first phase analysis were [age, arterial, and nephrographic phases]. The second phase analysis showed that noncontract and arterial phases were the most significant predictors. Finally, the third phase revealed the arterial and nephrographic phases were the most valuable predictors to discriminate between hypovascular masses.
**Conclusion:** Using a large sample, we developed a three-phase analysis to initiate a practical method to discriminate between different solid renal masses that can be used in daily clinical practice.
**Limitations:** Identified limitations were: (1) that this was a retrospective study prone to self-bias, and (2) this was a monocentre study, which needs more validation.
**Ethics committee approval:** -
**Funding for this study:** This study was self funded.
**Author Disclosures:**
Tarek El-Diasty: Nothing to disclose
Haytham Mohamed Shebel: Nothing to disclose
Heba Abou El Atta: Nothing to disclose
Doaa Elsayed Sharaf: Nothing to disclose

### RPS 1007-3

**The predictive role of the Node-RADS scoring system for assessing lymph node involvement in bladder cancer patients**

*A. Dehghanpour*, M. Pisciotti, A. Borrelli, S. Lucciola, C. Catalano, V. Panebianco; Rome/IT
*(ad48ad@yahoo.com)*

**Purpose:** To evaluate the diagnostic performance of the Node-RADS score in predicting lymph node invasion in patients with high grade non muscle invasive and muscle invasive bladder cancer.
**Methods or Backround:** Current cross-sectional imaging modalities have low sensitivity for assessing lymph node invasion (LNI) in bladder cancer (BCa) patients. In addition, diagnostic performance varies widely based on the evaluation criteria adopted. The Node-RADS score was recently introduced to provide a standardised and comprehensive assessment of the LNI based on a five-point scale that considers both size and configuration criteria. We retrospectively evaluated the CT scan of BCa patients treated with radical cystectomy (RC) plus bilateral extended pelvic lymph node dissection from January 2019 to June 2022 at our institution. Patients receiving preoperative systemic chemotherapy were excluded. Logistic regression analysis tested the correlation between Node-RADS score and LNI. ROC curves and AUC depicted overall diagnostic performance. Sensitivity, specificity, positive predictive value (PPV) and negative predicting value (NPV) were calculated for each Node-RADS assessment category.
**Results or Findings:** Overall, 49 patients were included. LNI ranged from 0 to 83.3% with increasing Node-RADS score ($p<0.001$). Node-RADS independently predicted LNI in the multivariable model (OR 3.36, 95%CI 1.68-9.40, p=0.004). Using a Node-RADS cut-off ≥3 the specificity, sensitivity, PPV and NPV were respectively 85.7, 57.1, 61.5, 83.3. Instead, using a cut-off value ≥ 4 the specificity, sensitivity, PPV and NPV were 97.1, 35.7, 83.3, 79.1. Therefore, Node-RADS ≥ 3 showed the best results and should be considered as the threshold to define nodal involvement.
**Conclusion:** Our study suggests that Node-RADS score could be considered a useful tool to predict lymph-node involvement in BCa patients with a moderate-to-high overall diagnostic accuracy.
**Limitations:** The retrospective study design and the small sample size were identified limitations.
**Ethics committee approval:** -
**Funding for this study:** No funding was received for this study.
**Author Disclosures:**
Sara Lucciola: Nothing to disclose
Valeria Panebianco: Nothing to disclose
Antonella Borrelli: Nothing to disclose
Ailin Dehghanpour: Nothing to disclose
Martina Pisciotti: Nothing to disclose
Carlo Catalano: Nothing to disclose

### RPS 1007-4

**Which is more accurate for predicting bladder cancer muscle invasion: artificial intelligence, VI-RADS, or a combination of the two?**

*M. Sam Özdemir*, S. Azamat, H. Özdemir, E. T. Keskin, M. Savun, A. H. Yardımcı, A. Şimşek; Istanbul/TR
*(mervesam@msn.com)*

**Purpose:** The presence of muscle invasion is the important factor in establishing the treatment strategy in bladder cancer. The VI-RADS criteria standardise the clinical use and reporting of mp-MRI in bladder cancer. The aim of this study was to evaluate the diagnostic performance of radiomics features in predicting bladder muscle invasion when evaluated separately and together with the VI-RADS scoring system.
**Methods or Backround:** Retrospectively, 58 patients with histologically confirmed bladder cancer who underwent preoperative MRI were recruited. Patients were assigned one of five scores according to the VI-RADS criteria. Following that, 3D Slicer version 4.8.1 was utilised to segment the entire tumour volumes on ADC maps. 960 features of the segmented images were extracted using filtration techniques and Pyradiomics. Recursive feature elimination with 5-fold cross-validation was used to select imaging features (CV). Selected radiomics features with and without VI-RADS were utilised to develop a classification model utilising a random forest classifier with a 10 fold CV. To evaluate diagnostic performance, the area under the curve (AUC) of the receiver operating characteristic analysis was used.
**Results or Findings:** The median age of the 58 patients was 63 years (50M/8F). A total of 31 bladder cancer patients had muscle invasion, while 27 had superficial disease. A total of 21 of 24 patients with ≥VI-RADS 4 scores had muscle invasion, compared to 10 of 34 patients with <VI-RADS 4 scores (AUC = 0.775).The performance of the combined VI-RADS score and radiomics model (AUC= 0.870.15) was significantly superior to that of a single radiomics model using ADC MRI (AUC = 0.770.25).
**Conclusion:** Both the radiomics properties and the VI-RADS scoring system can aid in predicting muscle invasion in bladder cancer, but their diagnostic performance is improved when evaluated together.
**Limitations:** Our study has limitations because it was conducted in a single location with a small sample size.
**Ethics committee approval:** -
**Funding for this study:** No funding was received for this study.
**Author Disclosures:**
Merve Sam Özdemir: Nothing to disclose
Aytül Hande Yardımcı: Nothing to disclose
Metin Savun: Nothing to disclose
Harun Özdemir: Nothing to disclose
Sena Azamat: Nothing to disclose
Emin Taha Keskin: Nothing to disclose
Abdulmuttalip Şimşek: Nothing to disclose

**RPS 1007-5**

**Role of 68Ga-PSMA PET radiomics for primary prostate cancer characterisation**

S. Ghezzo, G. Brembilla, *T. Russo*, C. Bezzi, A. M. Samanes Gajate, S. Paola, A. Briganti, F. De Cobelli, M. Picchio; Milan/IT
*(t.russo.1994@gmail.com)*

**Purpose:** Radiomics has been proposed for prostate cancer (PCa) characterisation. Results are encouraging, but often difficult to interpret. This study investigates 68Ga-PSMA PET radiomics to predict post-surgical International Society of Urological Pathology (ISUP) grade in primary PCa patients.
**Methods or Backround:** We retrospectively enrolled 47 PCa patients examined with 68Ga-PSMA PET at our institution prior to radical prostatectomy (RP). Images were acquired using PET/MR or PET/CT. ISUP scores were retrieved from pathology reports after RP. Whole prostate was used as volume of interest (VOI) to avoid limitations of radiomics for small volumes. An expert radiologist manually segmented VOIs on PET images using the co-registered CT or MRI for anatomical localisation on 3D Slicer. VOIs were resampled, normalised, discretised, and image biomarker standardisation initiative-compliant, radiomic features (RFs) were extracted. Combinations of the four most relevant RFs were used to train three different machine learning classifiers (logistic regression, support vector machine and K-nearest neighbour) for the prediction of ISUP≥4 vs. ISUP<4 that were validated by fivefold repeated stratified cross-validation. To ensure that results were not driven by spurious associations, two ad-hoc control models were generated respectively with SUVmax and VOI volume as input (radiomics baseline), while the other was made by setting to zero all voxel values prior to features extraction (PET zeros). Balanced accuracy, sensitivity, specificity positive and negative predictive values were collected.
**Results or Findings:** Thirty-two/47 patients had ISUP≥4, 15/47 ISUP<4. See Table 1 for the performance of the generated models.
**Conclusion:** This preliminary work provides evidence on the potential of 68Ga-PSMA PET radiomics in the prediction of post-surgical ISUP score, ruling out the possibility that results were due to spurious associations.
**Limitations:** The small cohort was an identified limitation.
**Ethics committee approval:** -
**Funding for this study:** This work was supported by grant IG 2017 Id.20571 and by PE-2016-02361273; EUDRACT number: 2018-001034-18.
**Author Disclosures:**
Maria Picchio: Nothing to disclose
Samuele Ghezzo: Nothing to disclose
Ana Maria Samanes Gajate: Nothing to disclose
Giorgio Brembilla: Nothing to disclose
Carolina Bezzi: Nothing to disclose
Scifo Paola: Nothing to disclose
Francesco De Cobelli: Nothing to disclose
Tommaso Russo: Speaker: Nothing to disclose
Alberto Briganti: Nothing to disclose

**RPS 1007-6**

**Benefit of dynamic contrast-enhanced (DCE) imaging for prostate cancer detection depending on readers' experience in prostate MRI**

*F. Ziayee*, L. Schimmöller, M. Boschheidgen, G. Antoch, T. Ullrich; Dusseldorf/DE

**Purpose:** The purpose of this study was to investigate the relevance of gadolinium-based contrast agent (GBCA) for the detection of prostate cancer (PCa).
**Methods or Backround:** Consecutive patients with 3T multiparametric (mp) MRI of the prostate and subsequent combined 12-core systematic and targeted MRI/US fusion-guided biopsy (SB and TB) from January to September 2019 were retrospectively evaluated blinded biparametic (bp) without DCE by readers with different experience (R1 with 2 versus R2 with 8-11 years of experience in prostate imaging) reading T2 and diffusion-weighted imaging (DWI). The study endpoint was the comparison of bp and mpMRI.
**Results or Findings:** The study included 124 male patients with 84 verified PCa (n=53; ISUP 2-5). BpMRI compared to mpMRI demonstrated a congruency of 55% (R1) and 65% (R2), respectively. Sensitivity of mpMRI was 90% for all PCA (98% for clinically significant (cs) PCa with ISUP 2-5). In bpMRI sensitivity was 70% (R1; 77% for csPCa) and 83% (R2; 87% for csPCa). Specificity was 80% for mpMRI (55% csPCa) and for bpMRI 60% (R1; csPCA 52%) and 78% (R2; csPCa 54%). PI-RADS detection rates for mp- vs. bp-MRI were 0% vs. 21%/13% (PIRADS 1/2), 25% vs. 63%/35% (PI-RADS 3), 84% vs. 76%/90% (PI-RADS 4) and 97% vs. 81%/88% (PI-RADS 5) and for csPCa 0% vs. 14%/0% (PIRADS 1/2), 3% vs. 28%/19% (PI-RADS 3), 44% vs. 46%/49% (PI-RADS 4) and 82% vs. 66%/68% (PI-RADS 5).
**Conclusion:** MpMRI demonstrated a higher sensitivity and detection rate compared to bpMRI for both experienced and less experienced readers. Evaluation of bpMRI was more accurate by experienced readers compared to less experienced readers. BpMRI would lead to more PCa detected in PI-RADS 1-3 and fewer PCa detected in Pi-RADS 4-5 lesions.
**Limitations:** The retrospective design represents the main limitation.
**Ethics committee approval:** -
**Funding for this study:** No funding was received for this study.
**Author Disclosures:**
Farid Ziayee: Nothing to disclose
Tim Ullrich: Nothing to disclose
Matthias Boschheidgen: Nothing to disclose
Lars Schimmöller: Nothing to disclose
Gerald Antoch: Nothing to disclose

**RPS 1007-7**

**Assessment of quantitative microflow vascular index for discrimination of testicular cancer**

*P. Spiesecke*, T. Fischer, C. Stephan, B. Hamm, M. H. Lerchbaumer; Berlin/DE

**Purpose:** Superb microvascular imaging (SMI) demonstrates an ultrasensitive Doppler/microflow modality allowing detection of small low-flow vessels. The amount of vascularisation can be quantified by a vascular index (VI) in percentage. The present study evaluates a comparison of the common testicular malignancies compared to healthy testicular vascularisation.
**Methods or Backround:** Ultrasound Doppler examinations using a high-end ultrasound-system (Canon Aplio i800/i900) between 2018 and 2022 were investigated. Inclusion criteria were i) age ≥ 18 years, ii) sufficient image quality and iii) testicular normal findings or solid testicular tumour with confirmed histopathological diagnosis. Vascularisation of solid testicular tumours were compared to healthy testicular parenchyma and divided into three main subgroups (seminoma, non-seminoma and lymphoma).
**Results or Findings:** Overall, 62 patients were included (31 normal findings and 31 solid tumours [16 seminomas, 9 non-seminomas and 6 lymphomas]). In normal findings, VI was in median 2.5% (IQR [interquartile range] 2.1–3.4%), whereas in tumours it was found to be 17.4% (IQR 11.1–25.2%) showing a highly significant difference (p<0.001). Subanalysis of VI among solid tumour categories showed an overall significant difference (p=0.029) between seminomas, non-seminomas and lymphomas (VI in mean 13.7%, 19.8% and 34.5%, respectively). Subgroup analysis showed especially high significance in differing seminomas and lymphomas (p=0.010).
**Conclusion:** Our results suggest a great potential of vascular quantification in further differentiation of testicular tumours – which can be especially helpful for non-subspecialised physicians.
**Limitations:** The present study data show a significant correlation that should be investigated by further researchers in order to possibly establish the VI in testicular sonography by merging the data.
**Ethics committee approval:** -
**Funding for this study:** No funding was received for this study.
**Author Disclosures:**
Bernd Hamm: Nothing to disclose
Paul Spiesecke: Nothing to disclose
Markus Herbert Lerchbaumer: Nothing to disclose
Carsten Stephan: Nothing to disclose
Thomas Fischer: Nothing to disclose

**RPS 1007-8**

**Impact of DWI and ADC values in ovarian-adnexal reporting and data system (O-RADS) MRI score**

V. Miceli, A. Cupertino, *M. Gennarini*, R. V. Ninkova, S. Ciulla, G. Ercolani, V. Celli, L. Manganaro, C. Catalano; Rome/IT
*(marco.gennarini@hotmail.it)*

**Purpose:** To introduce DWI and quantitative ADC evaluation in O-RADS MRI system and observe how diagnostic performance changes. To assess its validity and reproducibility between readers with different experience in female pelvic imaging. Finally, to evaluate any correlation between ADC value and histotype in malignant lesions.
**Methods or Backround:** A total of 173 patients with 213 indeterminate adnexal masses (AMs) on ultrasound were subjected to MRI examination, from which 140 patients with 172 AMs were included in the final analysis. Standardised MRI sequences were used, including DWI and DCE sequences. Two readers, blinded to histopathological data, retrospectively classified AMs according to the O-RADS MRI scoring system. A quantitative analysis method was applied by placing a ROI on the ADC maps obtained from single-exponential DWI sequences. AMs considered benign (O-RADS MRI score 2) were excluded from the ADC analysis.
**Results or Findings:** Excellent inter-reader agreement was found in the classification of lesions according to the O-RADS MRI score (K = 0.936; 95% CI). Two ROC curves were created to determine the optimal cut-off value for the ADC variable between O-RADS MRI categories 3-4 and 4-5: respectively 1.411 x 10-3 mm2/sec and 0.849 x 10-3 mm2/sec. Based on these ADC values, 3/45 and 22/62 AMs were upgraded respectively, to score 4 and 5, while 4/62 AMs were downgraded to score 3. ADC values correlated significantly with the ovarian carcinoma histotype (p value < 0.001).

**Conclusion:** Our study demonstrates the prognostic potential of DWI and ADC values in the O-RADS MRI classification for better radiological standardisation and characterisation of AMs.
**Limitations:** Firstly, it was single-centre retrospective study. Secondly, MRI examinations were performed by using two scanners operating at 1.5 T and 3.0 T. Thirdly, long-term follow-up was not available for patients who did not undergo surgery.
**Ethics committee approval:** -
**Funding for this study:** No funding was received for this study.
**Author Disclosures:**
Marco Gennarini: Nothing to disclose
Valentina Miceli: Nothing to disclose
Giada Ercolani: Nothing to disclose
Angelica Cupertino: Nothing to disclose
Sandra Ciulla: Nothing to disclose
Roberta Valerieva Ninkova: Nothing to disclose
Veronica Celli: Nothing to disclose
Carlo Catalano: Nothing to disclose
Lucia Manganaro: Nothing to disclose

### RPS 1007-9

**O-RADS MRI: a meta-analysis of diagnostic performance and of category-wise malignancy rates**

*A. Cozzi*[1], S. Rizzo[1], M. Dolciami[2], F. Del Grande[1], A. L. Scarano[1], B. Gui[2], N. Gandolfo[3], C. Catalano[2], L. Manganaro[2]; [1]Lugano/CH, [2]Rome/IT, [3]Genoa/IT
*(andrea.cozzi@gmail.com)*

**Purpose:** To systematically review and meta-analyse the diagnostic performance of pelvic MRI examinations performed to characterise US-indeterminate adnexal lesions and interpreted using the ADNEX-MR score and the Ovarian-Adnexal Imaging Reporting and Data System (O-RADS MRI), also meta-analysing malignancy rates in each ADNEX/O-RADS MRI category.
**Methods or Backround:** A systematic literature search from May 2013 (publication of the ADNEX-MR score) to September 2022 was performed. Studies reporting the use of pelvic MRI interpreted with the ADNEX/O-RADS MRI systems to characterise US-indeterminate adnexal lesions, with pathology and/or follow-up as reference standard, were included. Summary estimates of diagnostic performance were obtained with the bivariate random-effects model, while category-wise summary malignancy rates of ADNEX/O-RADS MRI 2, 3, 4, and 5 lesions were obtained with a random-effects model. Effects of covariates on diagnostic performance were investigated through meta-regression.
**Results or Findings:** Thirteen study parts from 12 studies (3731 women) met the inclusion criteria. Diagnostic performance meta-analysis on 4012 lesions found a 92% summary sensitivity (95% CI 88–95%) and a 91% summary specificity (95% CI 89–93%). The meta-analysis of malignancy rates on 3641 lesions showed a summary malignancy rate of 0.1% (95% CI 0–1%) among 1894 ADNEX/O-RADS MRI 2 lesions, of 6% (95% CI 3–9%) among 758 ADNEX/O-RADS MRI 3 lesions, of 60% (95% CI 52–67%) among 450 ADNEX/O-RADS MRI 4 lesions, and of 96% (95% CI 92–99%) among 539 ADNEX/O-RADS MRI 5 lesions.
**Conclusion:** The use of the ADNEX-MR/O-RADS systems to interpret pelvic MRI yields high diagnostic performance for the characterisation of US-indeterminate adnexal lesions. Summary estimates of malignancy rates in the ADNEX/O-RADS MRI 4 and ADNEX/O-RADS MRI 5 categories were higher than predicted ones.
**Limitations:** Identified limitations were: (1) early-stage diffusion of the O-RADS MRI system, and (2) sparse reporting of diagnostic performance data according to reader experience.
**Ethics committee approval:** Not applicable.
**Funding for this study:** No funding was received for this study.
**Author Disclosures:**
Andrea Cozzi: Nothing to disclose
Nicoletta Gandolfo: Nothing to disclose
Miriam Dolciami: Nothing to disclose
Benedetta Gui: Nothing to disclose
Filippo Del Grande: Nothing to disclose
Stefania Rizzo: Nothing to disclose
Carlo Catalano: Nothing to disclose
Angela Lia Scarano: Nothing to disclose
Lucia Manganaro: Nothing to disclose

### RPS 1007-10

**Association of ADC values and TP53 mutations in women with high grade serous ovarian cancer: preliminary results of an observational study**

C. Bourgioti, *M. A. Konidari*, D. Binas, F. Fostira, T. Economopoulos, R. Konstantopoulou, G. Lymperopoulou, G. Matsopoulos, L. A. Moulopoulos; Athens/GR

**Purpose:** Extensive intra-tumoural heterogeneity of high grade serous ovarian carcinoma (HGSOC) affects response to therapy and prognosis; the ability to obtain image-guided biopsies from different tumour clusters may guide more precise and personalised management of the disease.
**Methods or Backround:** Between July 2018-July 2022, 20 women with HGSOC underwent preoperative dedicated MRI of the abdomen and pelvis. In all 20 women, a three-dimensional (3-D) colour map of the tumour based on ADC values (selected ADC cut-off: 0.85x10-3mm2/sec; ADC ≤ 0.85x10-3mm2/sec colour-coded red; ADC>0.85x10-3mm2/sec color-coded blue) was obtained. All women underwent complete debulking surgery in a tertiary oncologic centre. Primary tumour specimen was appropriately oriented by two gynaecologic oncologists at surgery and targeted biopsies (2 from red areas/2 from blue areas) were obtained, based on preselected regions on the preoperative 3-D colour tumour model. DNA extracted from selected samples were analysed implementing oncomine comprehensive cancer panel with next-generation sequencing.
**Results or Findings:** Eleven of 20 women (mean age: 58 years) were included in the study; 9/20 women were excluded because of inadequate sampling (n=2) or refusal of genetic analysis (n=7). All samples obtained from "red areas" (ADC≤0.85x10-3mm2) harboured TP53 mutations whilst none of the "blue area" tissues (ADC>0.85x10-3mm2/sec) had TP53 mutations.
**Conclusion:** Association of low ADC values (ADC≤0.85X10-3mm2/s) with TP53 mutations in HGSOC tumours may potentially serve as an imaging biomarker for patient prognosis and treatment adaptations.
**Limitations:** Identified limitiations were: (1) the small number of patients, and (2) the inherent difficulties in precise identification of imaging-based target areas on actual macroscopic specimen.
**Ethics committee approval:** -
**Funding for this study:** This research has been co-financed by the European Union and Greek national funds through the Operational Program Competitiveness, Entrepreneurship and Innovation, under the call RESEARCH–CREATE–INNOVATE (project code: T1EDK-02886).
**Author Disclosures:**
Florentia Fostira: Nothing to disclose
Theodoros Economopoulos: Nothing to disclose
Reena Konstantopoulou: Nothing to disclose
Georgia Lymperopoulou: Nothing to disclose
George Matsopoulos: Nothing to disclose
Maria Anna Konidari: Nothing to disclose
Lia Angela Moulopoulos: Nothing to disclose
Dimitris Binas: Nothing to disclose
Charis Bourgioti: Nothing to disclose

### RPS 1007-11

**Can maximum, average or minimum ADC values of the cervix-parametrium boundary estimate parametrial invasion for cervical carcinoma?**

*M. Sorkun*, A. Durur Karakaya, H. Özen Atalay; Istanbul/TR

**Purpose:** Diffusion weighted imaging (DWI), which is quantified by apparent diffusion coefficient (ADC), can predict tissue microstructure. It has become an essential part of the gynaecological magnetic resonance imaging (MRI) protocol. In our study it was aimed to evaluate the value of maximum, average and minimum ADC values of the cervix-parametrium boundary to estimate parametrial invasion for cervix carcinoma.
**Methods or Backround:** A total of 50 patients with cervical carcinoma, 18 of which had no parametrial invasion (48±11 years) and 32 had parametrial invasion (58±12 years) according to conventional T2 weighted imaging were enrolled. Maximum, average, and minimum ADC values of the cervix-parametrium boundary of primary tumours were statistically compared between the groups without and with parametrial invasion. The diagnostic performances of the maximum, average, and minimum ADC values were evaluated by ROC analysis in terms of estimating parametrial invasion.
**Results or Findings:** The mean maximum, average, and minimum ADC values were lower for the patients with parametrial invasion. However, only the minimum ADC values had statistically significant differences between the groups. ROC analysis showed an AUC value of 0.726 for minimum ADC in estimating parametrial invasion. A minimum ADC cut-off value of 0.553x10-3 mm2/s had a sensitivity of 63%, specificity of 73%, negative predictive value of 52% and positive predictive value of 80%, and accuracy of 66%.
**Conclusion:** ADC values can be applied for the determination of parametrial invasion of cervical carcinoma. Lower minimum ADC values obtained from the cervix-parametrium boundary of primary cervical carcinoma may show parametrial invasion. Especially positive predictive value of the cervix-parametrium boundary ADC is remarkable.

**Limitations:** Limitation of this study were the retrospective design and the limited number of patients.
**Ethics committee approval:** -
**Funding for this study:** No funding was received for this study.
**Author Disclosures:**
Hande Özen Atalay: Nothing to disclose
Afak Durur Karakaya: Nothing to disclose
Mine Sorkun: Nothing to disclose

### RPS 1007-12

**MRI evaluation of vulvar cancer after chemoradiotherapy (CRT) for primary debulking and recurrences: preliminary results**

*M. Dolciami*[1], A. Marra[1], S. Persiani[1], E. Andreani[1], L. Russo[1], V. Lancellotta[1], E. Sala[2], R. Manfredi[1], B. Gui[1]; [1]Rome/IT, [2]Cambridge/UK
*(miriam.dolciami@gmail.com)*

**Purpose:** Vulvar cancer is a rare gynaecological tumour, which affects approximately 45000 new women per year. Chemoradiotherapy (CRT) is currently the treatment of choice in locally advanced primary tumours or in recurrences. The aim of this study was to evaluate the accuracy of magnetic resonance imaging (MRI) in assessing response to CRT in primary or recurrent vulvar cancer.
**Methods or Backround:** Patients with primary or recurrent vulvar cancer who received CRT and underwent pelvic MRI both at staging and at treatment completion were retrospectively enrolled. Response to therapy was assessed by two radiologists (with 5 and 1 year of experience in female pelvic imaging) according to Response Evaluation Criteria in Solid Tumors (RECIST) and compared with the current follow-up at our institution, consisting of clinical evaluation, positron emission/computed tomography (PET-CT), and ultrasound of inguinal lymph nodes.
**Results or Findings:** In total, 15 patients who fulfilled the inclusion criteria were included in the study. Regarding overall evaluation of local tumour and regional lymph nodes, MRI assessed by the more experienced radiologist provided an accuracy of 73.3%, with sensitivity and specificity of 70% and 80%, respectively. When analysing local disease and lymph nodes separately, MRI accuracy increased to 80% in both cases, with a significant improvement in both sensitivity and specificity (77.8% and 83.3% in local tumour assessment, respectively; 70% and 100% in nodal assessment, respectively). MRI images evaluated by the less experienced radiologist yielded lower accuracy in both overall and local assessment (60% and 66.7%, respectively); no change was recorded for nodal evaluation (80%).
**Conclusion:** Our results suggest that MRI can be a valuable tool in assessing CRT response, particularly by noninvasively identifying small-volume residual disease and guiding further investigation during follow-up.
**Limitations:** The small sample size was an identified limitation of this study.
**Ethics committee approval:** -
**Funding for this study:** No funding was received for this study.
**Author Disclosures:**
Angelica Marra: Nothing to disclose
Edoardo Andreani: Nothing to disclose
Riccardo Manfredi: Nothing to disclose
Benedetta Gui: Nothing to disclose
Valentina Lancellotta: Nothing to disclose
Evis Sala: Nothing to disclose
Salvatore Persiani: Nothing to disclose
Luca Russo: Nothing to disclose
Miriam Dolciami: Nothing to disclose

**14:00-15:30 Research Stage 2**

### Research Presentation Session: Head and Neck

### RPS 1008

### Temporal bone, oropharynx and nasopharynx

**Moderator**
R. Saat; Tallinn/EE

### RPS 1008-2

**MR imaging of endolymphatic hydrops in Meniere's disease: feasibility at 1.5Tesla**

*A. Ben Lakhal*, S. Boukriba, W. Frikha, E. Azouz, H. Hafsi, H. Mizouni; Tunis/TN
*(amine.ben.lakhal@outlook.com)*

**Purpose:** To show the feasibility of the diagnosis of endolymphatic hydrops in Meniere's disease using the delayed gadolinium enhanced 3D FLAIR sequence on a 1.5T MRI.
**Methods or Backround:** Meniere's disease is a condition of the inner ear that causes three main symptoms: episodic vertigo, hearing loss and tinnitus. Its pathological substrate is endolymphatic hydrops (EH). Many published studies have shown the visibility of EH on 3T MRI. Recently, some studies have been published to show the feasibility of this diagnosis using 1.5T MRI. This is an interesting proposition, especially in countries like ours (Tunisia) where only 6% of MRIs are 3T. And thus, we conducted this study. It was a multi-centric, prospective study, from January 2022 to October 2022. It included 35 patients who fulfilled the 2015 Barany Society criteria for Meniere's disease (27 definite and 8 probable). They underwent an imaging of the internal auditory canal 4 hours after IV administration of a double-dose of gadolinium (0.2mmol/kg) using a 3D FLAIR sequence with a fixed 120° flip angle.
**Results or Findings:** Visualisation of endolymphatic hydrops was possible in 33 patients (94%). For the other two: in one case the imaging analysis was poor due to motion artifacts and in the other, no hydrops was seen despite clinically significant disease.
**Conclusion:** With the administration of a double-dose of gadolinium, at the cost of an extra 4 minutes (average sequence time was 10 minutes on 1.5T compared to the 6 minute average on 3T), it is possible to diagnose endolymphatic hydrops using a 1.5T magnet. This finding facilitates access to imaging and in the long-run will improve both patient-care and treatment results.
**Limitations:** An identified limitation was that we did not compare our findings on 1.5T to imaging on 3T.
**Ethics committee approval:** Our study was approved by the ethics committee of the La Rabta Teaching Hospital.
**Funding for this study:** No conflicts of interest to declare.
**Author Disclosures:**
Eya Azouz: Nothing to disclose
Seifeddine Boukriba: Nothing to disclose
Hassen Hafsi: Nothing to disclose
Amine Ben Lakhal: Nothing to disclose
Habiba Mizouni: Nothing to disclose
Wassim Frikha: Nothing to disclose

### RPS 1008-3

**Quality and efficiency of AI-based, automated cochlear reconstructions in high-resolution temporal bone CT before cochlear implantation**

*M. Kopp*, M. Wetzl, R. Heiß, M. Wiesmüller, M. Zeilinger, V. Thimsen, J. Hornung, M. Uder, M. S. May; Erlangen/DE

**Purpose:** Cochlear implant (CI) planning requires optimal high-resolution CT imaging for evaluation of the cochlear length and width. The dedicated reformatted cochlear reconstructions (CR) are currently aquired manually, which is accompanied by additional time effort. This study compares the image quality (IQ) and time effort between manual (m) and automated (a) CR provided by an AI algorithm trained for anatomic landmark detection.
**Methods or Backround:** We retrospectively included 100 patients with temporal bone CT (50 patients in each group). The IQ of aCR versus mCR was rated on a 3-point Likert scale (1-insufficient, 2-diagnostic for anatomic overview, 3-optimal for CI implantation) by two readers. Furthermore, we measured the time effort for aCR and mCR.
**Results or Findings:** The missing rate for aCR was 12% and 6% for mCR. Median IQ was comparable between both groups for both sides (2 (2-3) versus 3 (1-3); $p > 0.05$). Prevalence of optimal image quality (=3) was 50% (aCR)

and 61% (mCR). The mean time effort for aCR and mCR was 73 versus 167 seconds (+228%).
**Conclusion:** IQ of AI-based aCR is statistically comparable to mCR and much more time efficient. For aCR and mCR we had relevant missing rates, which lead to additional manual reconstruction efforts. However, optimal IQ is more prevalent when mCR is performed. Consequently, optimal CI planning still requires manually reformatted CT reconstructions.
**Limitations:** Comparable to other IQ studies, this study is also based on the subjective cochlea image evaluation of two readers. Also, time measurments were performed manually. A digital time stamp function was unavailable.
**Ethics committee approval:** No information provided by the submitter.
**Funding for this study:** Not applicable.
**Author Disclosures:**
Marco Wiesmüller: Speaker: Siemens Healthcare GmbH
Matthias Stefan May: Speaker: Siemens Healthcare GmbH
Markus Kopp: Speaker: Infervision GmbH Speaker: Siemens Healthcare GmbH
Vivian Thimsen: Nothing to disclose
Martin Zeilinger: Nothing to disclose
Matthias Wetzl: Speaker: Siemens Healthcare GmbH
Michael Uder: Nothing to disclose
Joachim Hornung: Nothing to disclose
Rafael Heiß: Speaker: Siemens Healthcare GmbH

### RPS 1008-4

**Photon-counting CT allows better visualisation of temporal bone structures in comparison with current generation multi-detector CT**
*R. Hermans*, L. Boomgaert, L. Cockmartin, J. Binst, R. De Stefanis, H. Bosmans; Leuven/BE
*(robert.hermans@uzleuven.be)*

**Purpose:** To compare photon-counting CT (PCCT) and multi-detector CT (MDCT) for visualisation of temporal bone anatomical structures.
**Methods or Backround:** Thirty-six exams of temporal bones without pathology were collected from consecutive patients on a MDCT (Siemens Somatom Force) and another 35 exams on a PCCT scanner (Siemens Naeotom Alpha). Two radiologists independently scored visibility of 14 structures for the MDCT and PCCT dataset, using a 5-point Likert scale, with a two month wash-out period. For MDCT, the acquisition parameters were: 110 kV, 64 x 0.6 mm, pitch 0.85, quality ref. mAs 150, 1s rotation time; for PCCT: 120 kV, 144 x 0.2 mm, pitch 0.35, IQ level 75, 0.5s rotation time. Patient doses were reported as dose length product values (DLP). Visual grading scores were compared using a Mann-Whitney U test for each structure. Visual grading characteristic (VGC) analysis was used to compare overall anatomy visualisation in both scanners; ordinal regression quantified the odds of better visibility in PCCT versus MDCT.
**Results or Findings:** Substantial agreement was found between readers (intraclass-correlation coefficient 0.67 and 0.60 for MDCT and PCCT respectively.). All structures were scored higher for PCCT ($p<0.0001$), except for Arnold's canal ($p=0.12$). The area under the VGC curve was 0.73 (95% CI, 0.70-0.76), indicating a significantly better visualisation on PCCT. Ordinal regression showed the odds for better visualisation are 4.9 times higher (95% CI, 3.9-6.1) in PCCT ($p<0.0001$). Average (range) of DLP was 95 (79-127) mGy*cm for MDCT, and 74 (50-95) mGy*cm for PCCT ($p<0.001$).
**Conclusion:** PCCT provides a better depiction of temporal bone anatomy than MDCT, at a lower radiation dose.
**Limitations:** The MDCT and PCCT studies were judged in separate sessions in a non-blinded manner. Some degree of observer bias cannot be excluded.
**Ethics committee approval:** Ethics committee approval reference number: S65765
**Funding for this study:** No funding was received for this study.
**Author Disclosures:**
Robert Hermans: Nothing to disclose
Hilde Bosmans: Shareholder: Qaelum NV Research/Grant Support: Siemens AG, General Electric, Agfa-Gevaert Group
Rashèl De Stefanis: Nothing to disclose
Lukas Boomgaert: Nothing to disclose
Lesley Cockmartin: Nothing to disclose
Joke Binst: Nothing to disclose

### RPS 1008-5

**Texture analysis in otosclerosis screening**
*V. J. Ruiz García*, V. Márquez Pérez, S. Sanchez Paniagua Martín, J. Perez Lara, B. Asenjo, F. Sendra Portero, A. E. Perez Lara; Malaga/ES
*(vicentejavierruizgarcia@gmail.com)*

**Purpose:** To determine if texture analysis allows otosclerosis to be accurately identified in CT images of the petrous bone, using machine learning (ML) techniques for this purpose. To compare the accuracy of the artificial intelligence algorithm with the diagnostic precision of a radiologist specialised in head and neck, and general radiologists.
**Methods or Backround:** 50 patients with imaging-proven fenestral otosclerosis on CT scan and pathological audiometry were retrospectively selected, also 50 cases of healthy controls with normal findings on CT scan. Using 3D slicer we segmented the fissula ante fenestram and first-level statistics were obtained. Subsequently, a ML algorithm was created for detecting cases with otosclerosis based on radiomic data.
**Results or Findings:** Radiomic analysis demonstrated different CT features between otosclerosis patients and healthy controls. With the AI algorithms generated we have managed to predict the otosclerosis with 100% accuracy. On the other hand, the accuracy shown by the AI algorithm is significantly higher than the accuracy of general radiologists.
**Conclusion:** AI algorithms with texture analysis allows 100% detection of otosclerosis cases without false positives. In addition, AI algorithm has a higher accuracy than general radiologists. The use of this application will be of greater interest in centres without head and neck specialists.
**Limitations:** No limitations were identified.
**Ethics committee approval:** -
**Funding for this study:** No funding was received for this study.
**Author Disclosures:**
Javier Perez Lara: Nothing to disclose
Almudena Esther Perez Lara: Nothing to disclose
Sara Sanchez Paniagua Martín: Nothing to disclose
Francisco Sendra Portero: Nothing to disclose
Virginia Márquez Pérez: Nothing to disclose
Vicente Javier Ruiz García: Nothing to disclose
Beatriz Asenjo: Nothing to disclose

### RPS 1008-6

**Image quality and radiation dose of photon-counting paranasal sinuses and temporal bone CT: a comparison with dual-source and dual-layer CT**
*N. Mesropyan*, A. Isaak, D. Kravchenko, L. Bischoff, Y. C. Layer, T. Dell, U. I. Attenberger, J. A. Luetkens, D. Kütting; Bonn/DE

**Purpose:** This experimental study aimed to compare the image quality (IQ) of paranasal sinuses and temporal bone CT using different radiation dose settings on current high-end CT systems: dual-layer spectral, dual-source, and photon-counting.
**Methods or Backround:** A cadaveric human specimen was used for this experiment. Temporal bone imaging was performed with the following parameters: 120kV and A (high-dose):140-100mAs; B (middle-dose): 90-60mAs; C (low-dose): 50-25mAs; D (ultra-low-dose): 20-10mAs. Similarly, for paranasal sinuses CT: 100kV and A: 100-80mAs; B: 70-50mAs; C: 40-25mAs; D: 20-10mAs. IQ of important anatomic landmarks (temporal bone: facial nerve canal, cochlea, tympanic tegmen, malleus; paranasal sinuses: uncinate process, nasolacrimal duct, cribriform plate, maxillary ostium) was assessed using Likert-grading scale (1: non-diagnostic to 5: excellent). Dose-length product (DLP), CT dose index and scan length were retrieved and compared. Wilcoxon test, Friedman followed by Dunn's multiple comparison test were used for statistical analysis.
**Results or Findings:** For temporal bone, photon-counting CT provided excellent-to-good IQ up to low-dose scans for all anatomical landmarks, which was superior to dual-layer (excellent-to-sufficient), followed by dual-source CT (good-to-poor), $P<0.001$. Photon-counting CT had significantly better IQ compared to spectral CT in ultra-low-dose settings ($P<0.001$). For paranasal sinuses CT, no significant differences in IQ were found between all CT systems using high- and middle-dose scans ($P=0.81$). In low- and ultra-low-dose settings IQ were similar by photon-counting and dual-layer (C: $P=0.17$; D: $P=0.99$) and superior to that of dual-source CT (C: $P<0.05$). Photon-counting CT allows for reduction of DLP compared to spectral CT by paranasal sinuses (e.g., 16.8 vs. 111 by 100mAs, 1.7 vs. 11 by 10mAs).
**Conclusion:** Photon-counting CT allows for drastic radiation dose reduction while maintaining good IQ up to ultra-low-dose scans, which is also superior to other evaluated CT systems.
**Limitations:** An identified limitation was that this was an experimental study in a single human cadaveric specimen.
**Ethics committee approval:** -
**Funding for this study:** No funding was recevied for this study.
**Author Disclosures:**
Julian Alexander Luetkens: Nothing to disclose
Tatjana Dell: Nothing to disclose
Narine Mesropyan: Nothing to disclose
Alexander Isaak: Nothing to disclose
Leon Bischoff: Nothing to disclose
Yannik Christian Layer: Nothing to disclose
Dmitrij Kravchenko: Nothing to disclose
Ulrike I. Attenberger: Nothing to disclose
Daniel Kütting: Nothing to disclose

### RPS 1008-7

**Computed tomography-based radiomics in oropharyngeal cancer patients treated with radiotherapy: a promising tool for outcome modelling**

*S. Volpe*, F. Colombo, A. Gaeta, F. Mastroleo, M. Zaffaroni, M. G. Vincini, S. Raimondi, D. Alterio, B. A. Jereczek-Fossa; Milan/IT
*(stefania.volpe@ieo.it)*

**Purpose:** This study aims to build a predictive model to test the correlation between CT-based radiomic features, clinical outcomes (overall survival – OS, local progression free survival – LPFS) and immunobiological markers (e.g. HPV status, neutrophil/lymphocyte ratio) in patients with oropharyngeal squamous cell cancers (OPSCCs).
**Methods or Backround:** Consecutive OPSCCs patients treated with curative-intent radiotherapy (RT) at a single cancer centre from January 2005 to December 2017 were considered. Clinical data, tumour characteristics and pre-RT blood count parameters were analysed. The gross tumour volume of the primary tumour was segmented on non-contrast enhanced simulation scans and radiomic features were extracted using Pyradiomics v3.01. Features with limited redundancy were selected by Cox-LASSO regression models. Clinical model, radiomic model and clinical-radiomic model were evaluated and compared using the C-index. A stratified analysis on the subgroup with available HPV status was performed.
**Results or Findings:** One hundred and five patients were included (median age 59 years) with a median follow-up of 65.6 months. HPV status was available in 70/105 cases, 63 of which were positive (90%). At cross-validation, the combined clinical-radiomic model resulted the best performing one for the OS (C-index 0.86, 3 radiomics features) and the LPFS (C-index 0.80, 5 radiomics features) prediction. These results were confirmed also in the HPV+ subgroup analysis, either for the OS (C-index 0.89, 4 radiomics features) and the LPFS (C-index 0.72, 4 radiomics features) prediction.
**Conclusion:** CT-based radiomics features represent a reliable non-invasive tool for outcome prediction in OPSCC patients. The integration of radiomic analysis and clinical predictive factors can represent a concrete step along the road of precision medicine. Further validation with external dataset should be performed to confirm the promising role of the clinical-radiomic model.
**Limitations:** Identified limitations were: (1) this was a retrospective study, and (2) there was no external validation.
**Ethics committee approval:** -
**Funding for this study:** No funding was received for this study.
**Author Disclosures:**
Federico Mastroleo: Nothing to disclose
Aurora Gaeta: Nothing to disclose
Maria Giulia Vincini: Nothing to disclose
Sara Raimondi: Nothing to disclose
Barbara Alicja Jereczek-Fossa: Nothing to disclose
Stefania Volpe: Nothing to disclose
Francesca Colombo: Nothing to disclose
Mattia Zaffaroni: Nothing to disclose
Daniela Alterio: Nothing to disclose

### RPS 1008-8

**Integrating primary tumour and lymph node imaging features based on multi-sequence MRI to predict HPV-related p16 status with oropharyngeal squamous cell carcinoma**

*Q. Li*, S. Xiang; Shanghai/CN

**Purpose:** To develop a multi-sequence MRI radiomics model intgrating PT and LN imaging features for prediction of p16 status in OPSCC.
**Methods or Backround:** 141 patients with histopathological-confirmed OPSCC from two different institutions were enrolled. The HPV statuses were determined by p16 IHC analysis. A total of 2092 radiomics features were initially computed and extracted based on CE-T1WI and T2WI. The SVM classifier was employed to build the machine learning based classification models depending on p16 status. The models were validated in the testing cohort. AUC of ROC curve was computed to assess the performance of each model.
**Results or Findings:** In the testing cohort, fusion models yield better performance compared with models based on sole PT/LN (CE-T1WI: 0.80 vs. 0.71/0.73, T2WI: 0.74 vs. 0.64/0.71). Models based on multi-sequence outperformed single CE-T1WI/T2WI models (PT: 0.74 vs. 0.71/0.64, LN: 0.78 vs. 0.73/0.71). Finally, the PT-LN fusion model based on multi-sequence yielded best classification performance with a highest AUC of 0.91 for prediction of p16 expression. The differences between the final models and the other eight models were significant ($P<0.05$).
**Conclusion:** The results demonstrated that (1) the PT-LN fusion radiomics models improved the classification performance of sole PT or LN for prediction of p16 status; (2) the radiomics models based on multi-sequence outperformed single-sequence models in predicting the p16 status; (3) the PT-LN fusion model based on multi-sequence MRI radiomics features could serve as a noninvasive method to reflect the molecular information of OPSCC, which can assist oncologists in their clinical decision-making.
**Limitations:** Identified limitations were: (1) that additional improvement in inferring tumour HPV status may be achieved when combining radiomics with clinical characteristics, and (2) advanced MRI techniques, such as DCE and DWI were not performed in this study.
**Ethics committee approval:** -
**Funding for this study:** No funding was received for this study.
**Author Disclosures:**
Qiao Li: Nothing to disclose
Shiyu Xiang: Nothing to disclose

**14:00-15:30** **Research Stage 3**

### Research Presentation Session: Musculoskeletal

### RPS 1010

### Musculoskeletal: muscle, tendon and nerves

**Moderator**
F. Zaottini; Genoa/IT
*(federico.zaottini.fz@gmail.com)*

### RPS 1010-2

**Analysis of stiffness of rectus femoris in female elite soccer players by shear-wave elastography: a preliminary study**

*C. Urtasun Iriarte*[1], M. B. Barrio Piqueras[1], P. Nieto Moreno[2], B. Álvarez de Sierra García[2]; [1]Pamplona/ES, [2]Madrid/ES

**Purpose:** In this study, we explored the reliability of shear-wave elastography (SWE) for assessment of the rectus femoris muscle stiffness and its relationship with leg dominance in female elite soccer players.
**Methods or Backround:** This study was conducted on 20 healthy female individuals. Two examiners measured mean shear-wave velocity values of the proximal and distal rectus femoris muscles on both extremities using a 9L4 (4-9 MHz) transducer and a LogiqS8 ultrasound system (GE). Elasticity images were acquired by the tissue imaging quantification technique (GE). Reliability of SWE measurements was assessed through the intraclass correlation coefficient (ICC). Association between the dominant leg and SWE values was assessed by Fisher's exact test.
**Results or Findings:** The mean age of the subjects was 23.3 ± 4.48 years, with 78.9% of the sample being right -footed. Proximal and distal rectus femoris measurements on both the left and right legs showed a high interobserver ICC (0.98 and 0.94 respectively for the left leg, and 0.97 and 0.98 for the right). No significant association was found between the dominant leg and SWE values ($p=0.26$).
**Conclusion:** SWE is a reliable and repeatable technique for rectus femoris stiffness measurements according to interobserver ICC values in women soccer players.
**Limitations:** An identified limitation was the sample size.
**Ethics committee approval:** No information provided by the submitter.
**Funding for this study:** No funding was received for this study.
**Author Disclosures:**
Patricia Nieto Moreno: Nothing to disclose
Cesar Urtasun Iriarte: Nothing to disclose
Miguel Barrio Barrio Piqueras: Nothing to disclose
Beatriz Álvarez de Sierra García: Nothing to disclose

### RPS 1010-3

**A comparison between Q-Dixon MRI and high-speed T2-corrected multi-echo MR spectroscopic methods for quantifying thigh muscle fat content in chronic dialysis patients**

*W. Zhang*, L. Wang, Y. Yuan, X. Cheng; Beijing/CN
*(zwsgoforit@qq.com)*

**Purpose:** The purpose of this study was to compare quantitative Dixon (Q-Dixon) MRI and high-speed T2-corrected multi-echo (HISTO) MR spectroscopy (MRS) for quantifying the fat content of thigh muscles in chronic dialysis patients.
**Methods or Backround:** This study enrolled 40 adult chronic dialysis patients (mean age, 58.6 years; range, 89.1-29.7 years; 28 males). Proton density fat fraction (PDFF) was measured with 6-point Q-Dixon sequence and HISTO MRS within the upper-middle part of the left quadriceps femoris. The intraclass correlation coefficients (ICC) were calculated to evaluate the interobserver and intraobserver variability. Scatterplots, Bland-Altman plots and Spearman correlation coefficients were used to examine the relationship between Q-

Dixon and HISTO PDFF. The comparison between Q-Dixon and HISTO PDFF was investigated by Wilcoxon signed rank test.
**Results or Findings:** Interobserver and intraobserver agreement of Q-Dixon PDFF were both excellent (ICC: 0.934, 0.925). Q-Dixon PDFF of quadriceps femoris was positively correlated with HISTO PDFF (r=0.911, P＜0.001). Bland-Altman analysis revealed good agreement between Q-Dixon PDFF and HISTO PDFF, with an average difference in quadriceps femoris of 0.25%. The difference between Q-Dixon PDFF and HISTO PDFF was statistically insignificant (Z=0.241, P=0.125).
**Conclusion:** The 6-point Q-Dixon MRI exhibits good correlation and consistency with the HISTO MRS for fat quantitative measurement in the thigh. The high correlation of Q-Dixon MRI with HISTO MRS supports the idea that Q-Dixon MRI might become a reliable alternative to HISTO MRS in clinical assessment and radiological quantitative evaluation of thigh muscle fat infiltration.
**Limitations:** One important limitation was that this was a retrospective study with a rather small number of participants. Another limitation was that subjects of moderate and severe muscle fat filtration (Goutallier Grade 3 and 4) in the thigh were not acquired.
**Ethics committee approval:** -
**Funding for this study:** Funding was received from: Beijing Hospitals Authority Clinical Medicine, Development of Special Funding Support (code: ZYLX202107).
**Author Disclosures:**
Yi Yuan: Nothing to disclose
Wenshuang Zhang: Nothing to disclose
Xiaoguang Cheng: Nothing to disclose
Ling Wang: Nothing to disclose

### RPS 1010-4

**Suitability of dual-layer spectral-CT muscle fat quantification for assessing muscle quality and frailty**

*N. F. Schubert*, J. Erley, G. Campbell, P. Szwargulski, M. Breuer, J. Yamamura, I. Molwitz; Hamburg/DE

**Purpose:** To evaluate the suitability of dual-layer detector spectral-CT (dlsCT) fat quantification of the skeletal muscle to serve as a parameter of muscle quality and frailty.
**Methods or Backround:** In this prospective study, 22 patients with end-stage liver disease and an available abdominal dlsCT (IQon Spectral-CT, Philips, Best, Netherlands) within the last 3 months were included after ethics committee approval. Muscle fat fraction (FF) was assessed by three-material decomposition for muscle tissue, iodine, and fat at the height of the third lumbar vertebra (L3) for the whole abdominal muscle group. Muscle radiodensity attenuation (MRA) and the skeletal muscle index (SMI) were determined based on the whole abdominal muscle area at L3. Muscle quality was measured by phase angle in bioelectrical impedance analysis (BIA) as reference technique. Clinical muscle function was evaluated by the liver frailty index (LFI) consisting of grip-strength, chair-rise test, and balance tests. For statistics receiver operating characteristic (ROC)-curves and linear regression models were applied.
**Results or Findings:** FF performed better (area under the curve 0.75) than the SMI (0.43) or MRA (0.39) regarding the BIA phase-angle measurements. FF showed a slight predictability for the LFI ($r2=0.314$; $P=0.04$). No predictability was found for the MRA ($r2=0.141$, $P=0.086$) or the SMI ($r2=0.047$; $P=0.334$) to the LFI.
**Conclusion:** The diagnostic accuracy of the dlsCT muscle FF to assess BIA phase angle as an established parameter of muscle quality was better than that of the SMI or MRA. In comparison to single-energy CT parameters of muscle mass and quality, the dlsCT FF may be a superior parameter to predict frailty in patients with end-stage liver disease.
**Limitations:** BIA phase angle is known to be easily influenced by the hydration and nutritional status of patients.
**Ethics committee approval:** This study was approved by the ethics committee of Ärztekammer Hamburg, Germany (PV7006-4406-BO).
**Funding for this study:** This study was funded by the Hamburg Research Center for Medical Technology (04fmthh2020).
**Author Disclosures:**
Isabel Molwitz: Nothing to disclose
Graeme Campbell: Nothing to disclose
Patryk Szwargulski: Nothing to disclose
Jin Yamamura: Nothing to disclose
Miriam Breuer: Nothing to disclose
Jennifer Erley: Nothing to disclose
Niklas Ferdinand Schubert: Nothing to disclose

### RPS 1010-5

**Use of whole-body magnetic resonance and FIT 3D body scanner in the study of fat and muscle mass**

*F. P. Papa*, R. Faletti, A. Santonocito, S. Salto, F. Giorgino, F. Menchini, D. Tore, M. Gatti, P. Fonio; Turin/IT
*(francescop.papa@gmail.com)*

**Purpose:** The aim of the present study was to develop and validate a new method of investigation of the adipose and muscle mass that allows the integration of the data coming from the 3D scanner with the WB-MRI data, in order to obtain a more rapid and anatomically well-defined quantification of the muscle and adipose mass.
**Methods or Backround:** Sixteen volunteers were first tested with the body scanner (ProScanner, model 5.0 - Fit3D Inc.,) and then with WB-MRI (Philips 1.5 Tesla model). For the WB-MRI test, the volunteer was placed prone on the table. WB-MRI was done in a single imaging session. Segmentation of tissues was obtained through the open-source 3D Slicer research software and exported in .nrrd format to be included in a neural network.
**Results or Findings:** The difference between the fat mass values obtained on the body scanner and the WB-MRI was not statistically significant (p value of 0.9374). However the WB-MRI, compared to the 3D scanner, made it possible to distinguish between visceral and subcutaneous fat. On the contrary, the difference between lean mass detected by the body scanner and the muscle mass detected by WB-MRI was statistically significant ($p < 0.0001$).
**Conclusion:** The present study represents a feasibility study for a new method of body composition analysis by integrating data from the body scanner and the results of the whole-body magnetic resonance.
**Limitations:** The main limitation of the study is related to the small number of the population. Therefore it will be necessary to increase the sample size to include new patients in order to validate the efficacy of the protocol.
**Ethics committee approval:** No information provided by the submitter.
**Funding for this study:** No funding was received for this work.
**Author Disclosures:**
Sara Salto: Nothing to disclose
Francesco Pio Papa: Nothing to disclose
Marco Gatti: Nothing to disclose
Francesca Menchini: Nothing to disclose
Fabio Giorgino: Nothing to disclose
Riccardo Faletti: Nothing to disclose
Ambra Santonocito: Nothing to disclose
Davide Tore: Nothing to disclose
Paolo Fonio: Nothing to disclose

### RPS 1010-6

**A comparison of STIR, DWI, and T2-DIXON for muscle oedema in neuromuscular myopathies and myositis**

*G. Fichera*, F. Labella, R. Motta, R. Stramare, C. Giraudo; Padua/IT
*(giulia.fichera@studenti.unipd.it)*

**Purpose:** To assess muscle oedema in patients affected by neuromuscular myopathy and myositis with short tau inversion recovery (STIR), diffusion weighted imaging (DWI), and T2-DIXON.
**Methods or Backround:** Adults referring to our tertiary centre for neuromuscular myopathy or myositis who underwent an MR of the legs including STIR, DWI, and T2 DIXON were included. Muscle oedema was assessed in the calf muscles of each patient according to a 5-point scale (0 absent – 5 severe). For each sequence the quality of the images has been evaluated using a 10-point scale (0 very low quality – 10 excellent quality). ADCmin and ADCmean were extracted from the muscle with the highest grade of oedema on STIR. The ANOVA was used to compare all sequences for oedema and image quality; the Spearman coefficient was used to assess the relationship among ADC values and muscle oedema on STIR and T2 DIXON; the independent t-test was used to assess potential differences in ADC values between patients with myopathy or myositis (significance level $p<0.05$ for all analyses).
**Results or Findings:** Thirty-eight patients were examined (13 females; 48±19 years old); eight with myositis. Regarding the oedema a significant difference occurred between DWI and the other sequences (DWI 2.09±0.8, STIR 1.56±1 and T2 Dixon 1.66±1; $p<0.05$ each). The T2 DIXON sequence showed the highest average level of quality (T2 DIXON 7.8; STIR 6.8; and DWI 5.3; $p<0.001$, each). No correlation occurred among ADC values and oedema ($p>0.05$, each). Patients with myositis had significantly lower values of ADCmin (836±169 vs. 1025±220 x10-6 mm2/s, p=0.022).
**Conclusion:** The sequence T2 DIXON demonstrated high quality for muscle assessment while DWI, despite its tendency to overestimate oedema, could be especially useful for a quantitative assessment.
**Limitations:** No limitations were identified.
**Ethics committee approval:** No information provided by the submitter.
**Funding for this study:** No funding was received for this study.

Thursday

**Author Disclosures:**
Giulia Fichera: Nothing to disclose
Raffaella Motta: Nothing to disclose
Roberto Stramare: Nothing to disclose
Francesco Labella: Nothing to disclose
Chiara Giraudo: Nothing to disclose

### RPS 1010-7

**Role of diffusion tensor and diffusion weighted imaging in inflammatory myopathies**

*S. Agarwal*[1], S. Saran[2], V. S. Pai[2], A. Baweja[2]; [1]Jaipur/IN, [2]Rishikesh/IN

**Purpose:** We have explored the clinical feasibility of diffusion tensor (DTI) and diffusion weighted imaging (DWI) in evaluating anisotropic diffusion characteristics in dermatomyositis or polymyositis patients.
**Methods or Backround:** 24 patients with known dermatomyositis or polymyositis and 20 healthy volunteers were enrolled in this study. DTI, DWI and conventional MR sequences were performed on all subjects. Apparent diffusion coefficient (ADC), fractional anisotropy (FA), and three Eigen values were obtained from six selected muscles (vastus medialis, vastus lateralis, gracilis, biceps femoris, adductor magnus, and semimembranosus) and were compared between the inflammatory myopathy group and the healthy group. The extent of muscle oedema (no; minor <33.3%; 33.3-66.6%; major >66.6%), signal intensity (normal; low; moderate; high), fatty infiltration (no fat; fatty streaks; muscle>fat; muscle=fat; muscle<fat; muscle totally replaced by fat) were evaluated in STIR and T1W sequences. Skin and fascial oedema were also evaluated for every case. One way analysis of variance and student's t-test were used for statistical analysis with a significance of $p<0.05$.
**Results or Findings:** For each of the six measured muscles, FA values for patients with oedematous muscles were similar to those for healthy control subjects with normal muscles. The ADC and Eigen ($\lambda 1$, $\lambda 2$, and $\lambda 3$) values of oedematous muscles were significantly higher than those for patients with unaffected muscles and healthy control subjects ($p<0.05$ for all). Unaffected muscles of patients and healthy control subjects were similar in terms of all parameters. All the cases with major extent of muscle oedema had skin and fascial oedema.
**Conclusion:** DTI and DWI are clinically feasible for imaging thigh muscles and quantitatively evaluating oedematous muscles of patients with dermatomyositis or polymyositis.
**Limitations:** Patients with inflammatory myopathies were confirmed by biopsy, however biopsy was not carried out for healthy control patients.
**Ethics committee approval:** -
**Funding for this study:** No funding was received for this study.
**Author Disclosures:**
Sonal Saran: Nothing to disclose
Venkatesh Srisnivasa Pai: Nothing to disclose
Srishti Agarwal: Nothing to disclose
Ashish Baweja: Nothing to disclose

### RPS 1010-8

**Quantitative evaluation of sarcopenia before and after treatment with computed tomography in metastatic renal cell carcinoma patients treated with nivolumab and its relationship with prognosis**

*E. Özkan*, M. Köksal, Y. Açıkgöz, Ö. F. Kuzu, E. Algın; Ankara/TR
*(erdemozkan5454@gmail.com)*

**Purpose:** The aim of the study is to quantitatively evaluate sarcopenia with pre- and post-treatment computed tomography (CT) in metastatic renal cell carcinoma (RCC) patients using nivolumab, and to show the relationship of findings with prognosis and response to treatment.
**Methods or Backround:** Abdominal CT scans of 50 patients who were treated with nivolumab for the diagnosis of metastatic RCC in Ankara City Hospital between 2019-2022, before and after nivolumab, were analysed. Response to treatment was evaluated according to iRECIST criteria. The assessment of sarcopenia before and after treatment was made by measuring the skeletal muscle area (SMA) at the level of the L3 vertebra. In addition to the clinical and demographic data of the patients, overall survival and progression-free survival analyses were performed. The effects of the presence of sarcopenia before the treatment and the decrease in muscle mass during the treatment on the objective response to treatment and survival were investigated.
**Results or Findings:** Before the treatment, 30 patients were in the sarcopenic and 20 nonsarcopenic categories, and a statistically significant correlation was found between the presence of sarcopenia and multiple organ metastasis ($p=0.003$). It was determined that patients in the sarcopenic group and those in the group whose SMI values changed negatively during the process developed a statistically worse response to treatment ($p=0.027$ and $p=0.021$, respectively). Overall survival and progression-free survival were statistically significantly lower in the group of patients with sarcopenia before treatment and in the group of patients who developed a negative change in SMI during the treatment period.
**Conclusion:** Presence of sarcopenia is an important prognostic factor affecting treatment response and survival in patients with metastatic RCC who are treated with immunotherapy.
**Limitations:** Indentified limitations were that this was a single centre and retrospective study.
**Ethics committee approval:** The retrospective study was approved by the institutional review board.
**Funding for this study:** No funding was received for this study.
**Author Disclosures:**
Efnan Algın: Nothing to disclose
Erdem Özkan: Nothing to disclose
Yusuf Açıkgöz: Nothing to disclose
Murathan Köksal: Nothing to disclose
Ömer Faruk Kuzu: Nothing to disclose

### RPS 1010-9

**Opportunistic CT assessment of sarcopenia: a key role in prognosis and follow up of different clinical pathologies**

*G. Annarumma*, V. Chianca, A. Ponsiglione, L. Pace, R. Cuocolo, G. Fortunato, M. Carbone, A. Rizzo; Salerno/IT

**Purpose:** To provide an overview of the pathophysiology and diagnostic criteria of sarcopenia and to assess the opportunistic imaging tools currently available.
**Methods or Backround:** Sarcopenia is represented by mass and strength muscle loss and an impairment of function characterised by histological alterations of normal muscle structure. Pathophysiological mechanisms concern inflammatory cytokines and muscle protein degradation. Sarcopenia increases the risk of falls, fractures, cancer and organic diseases with a worsening quality of life in older people.
**Results or Findings:** BIA is the most used non-imaging diagnostic method to test lean, fat mass and body water content. DEXA is usually recommended to assess skeletal muscle index and appendicular lean mass index. US measures muscle thickness, cross-sectional area, echo intensity and fascicle length. It is helpful for bedridden patients because it is fast, cheap, dynamic and without radiation. MRI gives information on muscle mass, elasticity, oedema and fibrous substitution. It is an alternative method to CT because it isn't based on ionising radiationit, but it has high costs and it takes a long time for image acquisition. Cross-sectional areas of muscle at the third or fourth lumbar vertebra allows for evaluation of fatty infiltration and muscle mass during CT scanning. Artificial intelligence automatically estimates sarcopenia degree, but ROI should be accurately marked to avoid common pitfalls due to the algorithm's inability to distinguish visceral adipose tissue from subcutaneous adipose tissue. CT opportunistic screening can drive customised treatments and predict therapeutic complications or post-surgery prolonged hospitalisation.
**Conclusion:** Sarcopenia has a fundamental prognostic role in several organic diseases. MRI and CT are the best imaging methods to obtain a detailed description of muscle mass and degree of sarcopenia. AI reduces the processing time of large data, allowing for easier and faster opportunistic CT screening that predicts mortality and overall survival, without additional radiation and costs.
**Limitations:** No limitations were identified.
**Ethics committee approval:** No information provided by the submitter.
**Funding for this study:** No funding was received for this study.
**Author Disclosures:**
Leonardo Pace: Nothing to disclose
Gaspare Fortunato: Nothing to disclose
Gerardo Annarumma: Nothing to disclose
Mattia Carbone: Nothing to disclose
Vito Chianca: Nothing to disclose
Angelo Rizzo: Nothing to disclose
Andrea Ponsiglione: Nothing to disclose
Renato Cuocolo: Nothing to disclose

### RPS 1010-10

**Radiofrequency for the treatment of lower limb muscle injuries: diagnostic imaging in follow-up and response to treatment**

*S. Pasetto*; El Vendrell/ES
*(s.pasetto@gmail.com)*

**Purpose:** To study the clinical response of radiofrequency for the treatment of traumatic muscle injuries. To compare the results and the effectiveness of the treatment by means of radiofrequency or the conventional classic treatment for traumatic muscle injuries.
**Methods or Backround:** Radiofrequency therapy stimulates the natural mechanisms of healing and tissue recovery non-invasively. We used a specific form of high frequency currents to achieve both a bio-stimulation and/or thermal effect, by which the cell metabolism is activated restoring balance, and increased vascularisation and the internal temperature in the tissues of the skeletal muscle apparatus, which causes a further acceleration in the healing process.

**Results or Findings:** This was a prospective study, with a group of cases and a group of controls, using patients diagnosed with lower limb muscle strain injuries. Cases were treated by local radiofrequency. Controls were treated with classic conventional treatment consisting of rest, anti-inflammatories, compressive bandages and cold application to the damaged area (RICE method). We analysed clinical and ultrasoud image results after 2, 4 and 12 weeks from treatment, comparing the results of both procedures. The results showed that pain and disability yielded up to two weeks from the RF treatment, while patients treated with the RICE method recovered to a normal function in the controls by the fourth week. In the case group, the quantity of free fluid was not evident up to two weeks from the treatment; in control patients haematomas could persist until 12 weeks from treatment.
**Conclusion:** RF technology has been demonstrated clinically effective in the treatment of muscular injuries, diminishing the time of recovery and avoiding the fibrous scars, all this in comparison to the conventional classic treatment.
**Limitations:** An identified limitation was the small sample size.
**Ethics committee approval:** No information provided by the submitter.
**Funding for this study:** No funding to declare.
**Author Disclosures:**
Stefano Pasetto: Nothing to disclose

**14:00-15:30** **Research Stage 4**

### Research Presentation Session: Neuro

### RPS 1011

### CNS metastases, meningiomas and other neoplasms

**Moderator**
N. Anzalone; Milan/IT
*(anzalone.nicoletta@hsr.it)*

### RPS 1011-2

**Application of thin section contrast-enhanced fluid-attenuated inversion recovery sequence (CE-FLAIR) in differentiating the 'big three' brain tumours**
*Y. C. Lee*, S. Suh, I. Ryoo, H. N. Jung, L. Joo; Seoul/KR

**Purpose:** Despite the well-established radiologic findings, brain lymphomas, high grade gliomas, and metastases are occasionally not easily distinguishable with each other, using conventional and advanced imaging sequences. The differentiation of these tumours is clinically significant and valuable, due to their variable treatment options, clinical course, and pre-operative planning. The purpose of this study is to evaluate image findings of CE-FLAIR sequence in the three brain tumour groups and identify imaging features that characterise each of the three tumours.
**Methods or Backround:** Among 408 patients with brain tumours who underwent stereotaxic biopsy and/or surgery between December 2017 and September 2022, sixty patients with high-grade glioma, brain metastasis, or brain lymphoma were included. The stereotaxic MR sequence consisted of T1-weighted images (T1WI) and FLAIR sequence, before and after administrating gadolinium contrast agents with respective subtraction images. The imaging findings of the tumours were analysed, including volumetric evaluation via semi-automatic segmentation.
**Results or Findings:** The brain lymphomas showed the highest mean signal intensity (SI) and standard deviation values on contrast-enhanced T1WI (CE-T1WI) among the three tumour groups, whereas they showed the lowest mean SI and standard deviation values on CE-FLAIR sequence. The sequential volume change from CE-T1WI to CE-FLAIR was notably larger in metastases and lymphomas with 20.85% and 23.51%, respectively, compared to 9.61% in high grade gliomas. The maximal diameter change was most prominent in lymphomas with 6.53% increase, among the three groups. All of these results were statistically significant.
**Conclusion:** The thin section CE-FLAIR sequence is helpful in differentiating the three diagnostically challenging brain tumours: brain lymphomas, high grade gliomas, and metastasis.
**Limitations:** Limitations include the retrospective study design with small sample size in a single institution, limited variability on contrast agents and sequences, and human error in segmentation process.
**Ethics committee approval:** No information provided by the submitter.
**Funding for this study:** No funding was received for this study.
**Author Disclosures:**
Hye Na Jung: Nothing to disclose
Inseon Ryoo: Nothing to disclose
Yoon Chul Lee: Nothing to disclose
Sangil Suh: Nothing to disclose
Leehi Joo: Nothing to disclose

### RPS 1011-3

**Cranial nerve involvement: a rare presentation of CNS Lymphoma**
*A. D. Patkar*, D. P. Patkar, M. R. Verma, A. Narang, G. Ahuja, G. U. Harshe; Mumbai/IN
*(abhishekpatkar@gmail.com)*

**Purpose:** To study the involvement of multiple cranial nerves as a sequelae to primary CNS Lymphomas.
To highlight the importance of considering neurolymphomatosis as a key differential of cranial and spinal nerve thickening with enhancement.
**Methods or Backround:** Primary CNS lymphomas are generally of non-Hodgkin's type, which generally present as parenchymal lesions, with occasional leptomeningeal involvement. They originate in the brain, spinal cord, meninges, or eyes and seldom spread outside the CNS. Cranial nerve involvement, although rare, is not uncommon. We checked the MRI findings in five such patients who presented with neurolymphomatosis due to non-Hodgkin's lymphoma. The cases were studied to highlight the involvement of cranial and spinal nerves as the initial presentation of primary CNS lymphoma, its imaging features at presentation and over the course of the disease. Express consent was taken from all patients. Sequences used were: T1, T2, FLAIR, DWI, SWAN, FIESTA, post-contrast 3D T1, ASL, post-contrast DSC perfusion and spectroscopy.
**Results or Findings:** Clinically, all five patients presented with symptoms of cranial nerve involvement, mostly diplopia and facial palsy. Parenchymal lesions were seen in three of the patients in the first scan, however they showed no obvious clinical sign of parenchymal involvement. The cranial nerves involved include optic, oculomotor, trigeminal, facial, vestibulocochlear, glossopharyngeal, vagus, and accessory nerves, of which the trigeminal and VII–VIII nerve complex was most commonly involved. Following chemotherapy, abnormal nerve enhancement subsided in nearly all cases.
**Conclusion:** Multiple cranial and spinal nerve involvement can be a presenting feature of primary CNS lymphoma. Initial MRI presentation of enhanced and thickened nerves can lead to a number of confusing differentials including sarcoidosis, more so, in the absence of typical parenchymal lesions.
**Limitations:** No limitations were identified.
**Ethics committee approval:** No information provided by the submitter.
**Funding for this study:** No funding was received for this study.
**Author Disclosures:**
Gayatri Ulhas Harshe: Nothing to disclose
Aarushi Narang: Nothing to disclose
Deepak P. Patkar: Nothing to disclose
Mitusha Rakesh Verma: Nothing to disclose
Abhishek Deepak Patkar: Nothing to disclose
Gauri Ahuja: Nothing to disclose

### RPS 1011-4

**Virtual non-contrast images improve differentiation between vascular enhancement and calcifications in stereotactic planning CT examinations of cystic intracranial tumours**
*C. Nelles*, K. R. Laukamp, C. Zäske, N. Grosse Hokamp, S. Lennartz, C. Kabbasch, M. Schlamann, E. Celik, D. Zopfs; Cologne/DE

**Purpose:** To evaluate the diagnostic utility of spectral-detector CT (SDCT) derived virtual non-contrast images (VNC) for distinction between peri-cystic vascular enhancement and calcifications in contrast-enhanced stereotactic planning examinations of patients with cystic intracranial tumours.
**Methods or Backround:** A total of 48 patients with cystic intracranial tumours who received stereotactic SDCT scans between 02/2017 and 02/2020 were retrospectively included. Two separate hyperattenuating structures along the cyst margins in each patient were defined as either contrast enhancement or calcification, respectively, using MRI examinations as a reference. ROI-based quantitative analysis was performed in conventional images (CI) and VNC. In a subjective analysis, two radiologists diagnosed the predefined hyperattenuating structures as either vascular enhancement or calcification using CI and the combination of CI and VNC. Diagnostic confidence, image noise and removal of iodine in VNC were rated on 5-point Likert scales. Additionally, a potential diagnostic benefit of VNC was assessed.
**Results or Findings:** Attenuation values in CI were higher compared to VNC across all measured ROI (all $p<0.01$). CNR between calcification and white matter was significantly higher compared to CNR between vascular enhancement and white matter in VNC (2.6 vs. 1.3, $p<0.01$), while there was no significant difference in CI. In the qualitative analysis, diagnostic accuracy was significantly higher using both VNC and CI compared to using CI alone (4 [4-5] vs. 3 [2-3]; p<0,01). Image noise was rated lower in VNC compared to CI (5 [4-5] vs. 4 [3-5]; p<0,01). An additional diagnostic benefit of VNC was denoted in 84.4% of all cases.
**Conclusion:** SDCT-derived VNC images facilitate differentiation between peri-cystic vascular contrast enhancement and calcifications in stereotactic planning examinations of patients with cystic intracranial tumours.
**Limitations:** Identified limitations were: (1) the retrospective and monocentre study design with limited sample size, and (2) VNC attenuation measurements are prone to both longitudinal and inter-scanner variation.

**Ethics committee approval:** No information provided by the submitter.
**Funding for this study:** No funding was received for this study.
**Author Disclosures:**
Simon Lennartz: Nothing to disclose
Christoph Kabbasch: Nothing to disclose
Kai Roman Laukamp: Nothing to disclose
Christian Nelles: Nothing to disclose
David Zopfs: Research/Grant Support: Philips Healthcare Speaker: Philips Healthcare
Erkan Celik: Nothing to disclose
Charlotte Zäske: Nothing to disclose
Nils Große Hokamp: Speaker: Philips Healthcare Research/Grant Support: Philips Healthcare Consultant: Bristol-Myers Squibb
Marc Schlamann: Nothing to disclose

### RPS 1011-5

**Intra-/inter-observer agreement for detection of brain metastases using contrast-enhanced 3D T1-weighted turbo spin echo MRI at 1.5T and 3T**
*C. Xue*, G. Lo, K. K. Wong, A. S. Lai, W. H. R. Yung, J. Yuan; Hong Kong/HK
*(cindy.xue21@gmail.com)*

**Purpose:** The superiority of contrast-enhanced 3D-T1W-TSE over 3D-GRE in brain metastases (BM) detection has been well established at 3T, while its performance at 1.5T is still lacking in literature due to the possible lower SNR. This study aims to compare the inter-observer agreement in BM detectability using 3D-T1W-TSE at 1.5T and 3T.
**Methods or Backround:** 23 BM patients who underwent a 3T diagnostic scan using the dedicated volumetric head array coil and then a 1.5T (frameless brain radiosurgery treatment) planning scan using surface array coils within 14 days were retrospectively enrolled. The same contrast agent (CA) and registration protocol were used. Patients underwent no intervention between the two scans. Two radiologists independently detected BMs on 1.5T (isotropic 1.0mm voxel) and 3T (isotropic 0.9mm voxel) 3D-T1W-TSE MRI datasets with an interval of >30 days. Cohen's kappa and percent agreement were calculated to measure the intra-/inter-observer agreement in BM detection. BM detection agreement between 1.5T and 3T 3D-T1W-TSE were compared using McNemar's chi-squared test.
**Results or Findings:** A total of 79-102 BMs were detected on 1.5T planning-MRI, and 80-110 BMs on 3T diagnostic-MRI, with a percent agreement of 83% between them by both radiologists. The Cohen's kappa was substantial (0.72 [0.66-0.79]) between 1.5T and 3T by combining both radiologists' readings. The percent agreement between readers on 1.5T planning-MRI (80.4%) was insignificantly higher (p=0.185) than on 3T diagnostic-MRI (78.3%). The Cohen's kappa was 0.66 (0.58-0.75) for 1.5T planning-MRI and 0.69 (0.6-0.78) for 3T diagnostic-MRI.
**Conclusion:** Substantial intra-/inter-observer BM detectability was achieved on 1.5T planning-MRI compared to 3T diagnostic-MRI, suggesting the non-inferior BM detectability using 3D-T1W-TSE at 1.5T compared to 3T.
**Limitations:** The small sample size and single-centre retrospective study design were major limitations.
**Ethics committee approval:** This study was approved by the hospital research ethics committee. Written patient consent form was waived due to the retrospective nature.
**Funding for this study:** This study was supported by hospital research project REC-2018-06.
**Author Disclosures:**
Wai Hung Raymond Yung: Nothing to disclose
Jing Yuan: Nothing to disclose
Andrew SH Lai: Nothing to disclose
Gladys Lo: Nothing to disclose
Cindy Xue: Nothing to disclose
Ka Kin Wong: Nothing to disclose

### RPS 1011-6

**Comparison between post-contrast T1-weighted thin-slice 2D spin echo and 3D SPACE sequences in detection of brain metastases at 1.5T and 3T**
*A. Rulseh*, Z. Ryznarova, J. Vymazal; Prague/CZ

**Purpose:** The accurate detection of metastatic brain lesions (MBL) before radiotherapy is critical. Although spin echo (SE) is superior to gradient echo sequences in detecting small MBL, thin-slice whole-brain coverage is time-consuming. The sampling perfection with application optimised contrasts using different flip angle evolution (SPACE) sequences shares many advantages with SE, but with faster acquisition and greater resolution while achieving whole-brain coverage.
**Methods or Backround:** Fifty-six patients with MBL were included and underwent a standard protocol (1.5T, n=37; 3T, n=19), including post-contrast T1-weighted SE and SPACE (post-contrast order: SE first, n=26; SPACE first, n=30). Rating was performed by three raters in two sessions >6 weeks apart; images were de-identified and order randomised, only SE or SPACE per-subject per-session. The true number of MBL was determined using all available imaging including follow-up. Intraclass correlations were determined; consistency for intra-rater (SE vs. SPACE) and agreement for inter-rater (same sequence). A paired t-test was used to evaluate post-contrast sequence order.
**Results or Findings:** A total of 135 MBL were identified (mean/subject 2.41, SD 6.4). Relatively fewer lesions were identified on the first post-contrast sequence (SE/SPACE), however the difference was not significant (p=0.08). Intra-rater consistency (SE vs. SPACE) was excellent (ICC: R1, 0.984; R2, 0.971; R3, 0.946), as was inter-rater agreement, with ICC values of 0.984 and 0.969 for SE and SPACE sequences, respectively. Finally, agreement between individual sequences and the true number of lesions was excellent (SE ICC: R1, 0.981; R2, 0.973; R3, 0.977; SPACE ICC: R1, 0.984; R2, 0.971; R3, 0.965).
**Conclusion:** 3D T1-weighted SPACE sequences are not inferior to standard thin-slice SE in detection of MBL. All three experienced raters reached excellent consistency between SE and SPACE and agreement with ground truth.
**Limitations:** Not applicable.
**Ethics committee approval:** No information provided by the submitter.
**Funding for this study:** Funding was received from the Ministry of Health CZ, IG204303.
**Author Disclosures:**
Zuzana Ryznarova: Nothing to disclose
Josef Vymazal: Nothing to disclose
Aaron Rulseh: Nothing to disclose

### RPS 1011-7

**Deep learning based radiological longitudinal volumetric evaluation of brain metastases after stereotactic radiosurgery**
Y. Hammer, Y. Shoshan, W. Najjar, *L. Joskowicz*; Jerusalem/IL
*(josko@cs.huji.ac.il)*

**Purpose:** Clinical decisions on stereotactic radiosurgery (SRS), an indication for the treatment of brain tumours and metastases, are based on the comparison of the irradiated lesions' sizes in the pre- (baseline) and post- (follow-up) treatment MRI scans. The purpose of this study was to evaluate the performance of a novel fully automatic method for the accurate and reliable detection and volumetric evaluation of brain metastases on pairs of baseline and follow-up brain scans.
**Methods or Backround:** The method utilises a novel simultaneous multi-channel 3D U-Net classifier for the detection and segmentation of brain metastases in the follow-up MRI. We retrospectively collected 157 pairs of MRI scans from patients who underwent SRS at the Hadassah University Medical Center. An expert neurosurgeon manually delineated a total of 1137 metastases (469 baseline, 668 follow-up) in the MRI scans using the ITK-Snap software. The number of brain metastases with diameters 0-5mm, 5-10mm and > 10mm was 128, 380, and 629, respectively. The deep learning model was trained, validated and tested with 139 pairs (980 metastases), 8 pairs (46 metastases), and 10 pairs (111 metastases). The computed test set results were then compared to their respective ground truth delineations.
**Results or Findings:** Our method achieves a mean brain metastases detection precision (aka specificity) and recall (sensitivity) (std) of 0.87 (0.22) and 0.72 (0.40) for brain metastases with diameters > 5mm, 0.96 (0.12) and 0.85 (0.32) > 10mm, and 0.78 (0.33) and 0.56 (0.42) for all sizes. The metastases segmentation Dice scores are 0.91 (0.09), 0.92 (0.09) and 0.87 (0.14) for the same metastases sizes, all above the observer variability of 0.81 (0.13).
**Conclusion:** Automatic brain metastases detection and volumetric quantification may help improve the evaluation of treatment response.
**Limitations:** Identified limitations were (1) the use of single observer annotation, and (2) that this was a single centre study.
**Ethics committee approval:** No information provided by the submitter.
**Funding for this study:** No funding was received for this study.
**Author Disclosures:**
Leo Joskowicz: Consultant: HighRAD
Yonny Hammer: Nothing to disclose
Yigal Shoshan: Nothing to disclose
Wenad Najjar: Nothing to disclose

### RPS 1011-8

**Diagnostic accuracy of MRI techniques for treatment response evaluation in patients with brain metastasis: a systematic review and meta-analysis**
W. Teunissen[1], *C. W. Govaerts*[2], M. Kramer[2], J. Labrecque[1], M. Smits[1], L. Dirven[3], A. Van Der Hoorn[2]; [1]Rotterdam/NL, [2]Groningen/NL, [3]Leiden/NL
*(c.w.govaerts@umcg.nl)*

**Purpose:** To assess the diagnostic accuracy of advanced MRI-techniques for differentiating tumour progression and treatment-induced abnormalities in brain metastasis.
**Methods or Backround:** Treatment response evaluation after focal radiation in brain metastasis is performed with contrast-enhanced T1-weighted MRI. This can however be complicated by treatment-induced abnormalities. The diagnostic accuracy of advanced MRI-techniques in this context is unclear. We performed a systematic review and meta-analysis. Study selection and data extraction were performed by two authors independently. We used a bivariate random effects model to pool the data and an independent cohort of DSC-perfusion to externally validate the diagnostic accuracy of reported thresholds.
**Results or Findings:** 50 contingency tables could be made from 37 of 53 identified studies, comprising 1989 lesions. Anatomical MRI (16 studies, 726 lesions): pooled sensitivity and specificity of 79% and 76% respectively. DCE perfusion (4 studies, 114 lesions): pooled sensitivity and specificity of 74% and 92% respectively. DSC perfusion (12 studies, 418 lesions): pooled sensitivity and specificity of 83% and 78% respectively. Diffusion weighted imaging (7 studies, 288 lesions): pooled sensitivity and specificity of 67% and 79% respectively. MRS (4 studies, 54 lesions): pooled sensitivity and specificity of 80% and 78% respectively. Combined techniques (6 studies, 375 lesions): pooled sensitivity and specificity of 84% and 88% respectively. External validation of DSC showed a lower sensitivity and a higher specificity for the reported cut-off values included in this meta-analysis.
**Conclusion:** Combined perfusion/diffusion techniques resulted in the highest diagnostic accuracy for differentiating between tumour progression and treatment-induced abnormalities. External validation of DSC perfusion showed similar sensitivity and specificity to those reported at thresholds of 2.0-2.1. This work has been published as: Teunissen WHT et al. (2022) Diagnostic accuracy of MRI techniques for treatment response evaluation in patients with brain metastasis: A systematic review and meta-analysis. Radiother Oncol 177:121-133. doi: 10.1016/j.radonc.2022.10.026
**Limitations:** Quality control showed a high risk of bias and heterogeneity in many studies, reflecting inconsistencies with patient selection and follow-up.
**Ethics committee approval:** The external validation aspect of the study was reviewed by the Erasmus MC Medical Ethics Committee and all patients had either provided written informed consent or were already deceased at the time of inclusion.
**Funding for this study:** Funding was received from "Leading the change" (80-85009-98-2008-NVvR).
**Author Disclosures:**
Chris Wouter Govaerts: Nothing to disclose
Miranda Kramer: Nothing to disclose
Wouter Teunissen: Nothing to disclose
Anouk Van Der Hoorn: Nothing to disclose
Marion Smits: Speaker: AuntMinnie (paid to institution) Speaker: GE Healthcare (paid to institution) Consultant: Bracco (paid to institution)
Jeremy Labrecque: Nothing to disclose
Linda Dirven: Nothing to disclose

### RPS 1011-9

### Delayed FDG-PET CT in differentiating recurrent metastatic disease from radiation necrosis following cerebral stereotactic radiosurgery

*B. M. Ciavarra*, A. Krayyem, H. Alhasan, M. K. Gule-Monroe, M. Chen, C. Chung, S. Ferguson, J. Li, J. M. Johnson; Houston, TX/US
*(Bronson.M.Ciavarra@uth.tmc.edu)*

**Purpose:** Delayed 18F-FDG PET was utilised to compare 1-hour and 5-hour imaging post-injection in patients with metastatic brain cancer treated with radiation who developed MRI findings suspicious for recurrent disease.
**Methods or Backround:** Radiographic follow-up and/or pathology, identified 23 patients with radiation necrosis (RN) and 18 patients with progressive disease (PD). Maximum standard uptake values (SUV) were calculated for suspicious areas of MR enhancement (lesion) and compared to normal appearing brain (background) at both time points.
**Results or Findings:** Mean L/B (lesion/background) for PD at the early time point (1.30+0.61) was significantly higher than L/B for the early time point for RN (0.94+0.30), (p=0.0299). Additionally, the mean L/B for PD at the later time point (1.89+0.52) was significantly higher than L/B for the later time point for RN (1.11+1.06), (p=0.0092). L/B ratios in PD cases showed significantly higher SUV at the delayed time point compared to early time points (p=0.0497). L/B ratios in cases with RN were not significantly different between early and delayed time points (p=0.161). Notably, there were two PD patients who had a L/B ratio < 1 on the initial time point who revealed a L/B ratio > 1 on the delayed time point.
**Conclusion:** Contrary to conventional teaching, significant hypermetabolism was associated with cases of proven RN with a mean SUV of 8.75. Thus, the presence of increased metabolism associated with suspicious enhancement alone is not specific for PD. However, the addition of delayed FDG imaging improves overall study accuracy and reduces FP and FN. This was evidenced by 61% of PD patients on initial time point, and 72% of PD patients on delayed time point with L/B > 1.
**Limitations:** Limitations include a lack of algorithms that provide comprehensive MRI and PET metrics.
**Ethics committee approval:** This study was approved by the IRB of UT MDACC.
**Funding for this study:** Not applicable.
**Author Disclosures:**
Maria K Gule-Monroe: Nothing to disclose
Jason Michael Johnson: Grant Recipient: Blue Earth Diagnostics Consultant: Kura Oncology Consultant: InformAI
Jing Li: Nothing to disclose
Caroline Chung: Nothing to disclose
Melissa Chen: Nothing to disclose
Hamza Alhasan: Nothing to disclose
Sherise Ferguson: Nothing to disclose
Bronson Michael Ciavarra: Nothing to disclose
Apollo Krayyem: Nothing to disclose

### RPS 1011-10

### Assessment of ultrafast quantitative T2 imaging in differentiating meningioma WHO grades preoperatively

*Z. Li*[1], Y. Yang[2], Y. Zhang[1], X. Wang[1], Y. Zhuang[3], Y. Zhang[1], Z. Chen[2], J. Cheng[1], J. Bao[1]; [1]Zhengzhou/CN, [2]Xiamen/CN, [3]Rochester, NY/US
*(lizongye2021@163.com)*

**Purpose:** We assess the ability of a novel single-shot quantitative T2 magnetic resonance imaging (MRI) sequence and apparent diffusion coefficient (ADC) maps in predicting the WHO grade in meningiomas.
**Methods or Backround:** We enrolled 69 meningioma patients (59 with WHO 1, 10 with WHO 2 and 3) who underwent preoperative MRI examinations. Two independent neuroradiologists delineated regions of interest (ROI) on each slice, with the final results relying on their consensus. ROI was defined as tumour parenchyma that was significantly enhanced on contrast-enhanced T1-weighted imaging with the exclusion of large vessels and necrosis. The histogram parameters for each ROI were extracted, including mean, median, maximum, minimum, ten percentiles (P10), 90 percentiles (P90), interquartile range, range; entropy, skewness, kurtosis, uniformity and variance. Shapiro-Wilk test, independent t-test, Mann-Whitney U test, binary logistic regression, and receiver operating characteristic (ROC) analyses were performed. A value of p＜0.05 was considered to be the significance level.
**Results or Findings:** The mean, median, minimum and P10 of ADC maps were significantly higher in LGM patients than in HGM patients (p=0.010, 0.008, 0.013 and 0.002, respectively). However, histogram parameters based on T2 maps showed no significant difference between LGM and HGM. The mean, median, minimum and P10 based on ADC maps received AUCs of 0.757, 0.766, 0.747 and 0.811, respectively. Moreover, the combination of T2 kurtosis and ADC P10 obtained an AUC of 0.843.
**Conclusion:** We demonstrated that the ADC could effectively differentiate the WHO grade of meningiomas. ADC P10 proved to be the best predictor among all histogram parameters of ADC. While histogram analysis of T2 maps did not receive satisfactory results, the combination of T2 kurtosis and ADC P10 proved to be the best predictor.
**Limitations:** Not applicable.
**Ethics committee approval:** No information provided by the submitter.
**Funding for this study:** Not applicable.
**Author Disclosures:**
Yijie Yang: Nothing to disclose
Jianfeng Bao: Nothing to disclose
Xiao Wang: Nothing to disclose
Jingliang Cheng: Nothing to disclose
Zongye Li: Nothing to disclose
Zhong Chen: Nothing to disclose
Yuchuan Zhuang: Nothing to disclose
Yue Zhang: Nothing to disclose
Yong Zhang: Nothing to disclose

### RPS 1011-11

### Differentiation between spinal meningoma-schwannoma: quantitative signal intensity analysis based on T2 weighted magnetic resonance imaging

*Y. C. Güneş*, K. Ercan, S. Duran, E. Öztürk; Ankara/TR
*(gunesyasincelal@gmail.com)*

**Purpose:** To investigate the value of quantitative signal intensity (SI) measurements for the discrimination of spinal meningioma-schwannoma.
**Methods or Backround:** Neural foraminal extension, dural tail sign, and cystic tumoural changes are one of the MRI features which are used to discriminate between spinal meningioma and schwannoma. It is challenging to determine the correct diagnosis when using conventional MR, even though specific imaging features are given for the discrimination. Quantitative SI assessment gives promising results for tissue characterisation of some tumours. A total of 40 patients (histopathologically proven 20 meningioma, 20 schwannoma) with spinal 3-tesla (3T) MR were included in the study. Tumour signal intensity (TSI), paraspinal muscle signal intensity (PMSI), and tumour-fat signal intensity ratio (TFSIR) were calculated in axial T2-weighted images. TSI was calculated to pass through the outer contours of the whole lesion with free-hand ROI method. PMSI was calculated with a mean value of three different circular ROI on paraspinal muscles passing through the biggest dimension. TFSIR is the

Thursday ratio of TSI and PMSI. ROIs of the tumour and fat in all subjects were set by two radiologists.
**Results or Findings:** ICC values of two researchers TSI (r=0.983, 95% CI [0.967–0.994]), PMSI (r=0.981, 95% CI [0.964–0.993]), and TFSIR ratio [r=0.984, 95% CI [0.967–0.991]). Only TFSIR was statistically significant (p=0.003). The TFSIR of schwannomas and meningiomas were 0.68 ± 0.23 , 0.49 ± 0.16 (radiologist 1). The AUC was 0.780 (0.603–0.877) from ROC analysis. The cut-off value of TFSIR 0.631 (sensitivity: 60.0%, specificity: 88% [radiologist 1]).
**Conclusion:** Quantitative SI measurement is a useful method for discriminating spinal meningiomas from schwannomas. Tumour fat signal ratio (TFSIR) could be a novel predictive marker with excellent inter-rater reliability.
**Limitations:** The small sample size was an identified limitation.
**Ethics committee approval:** No information provided by the submitter.
**Funding for this study:** No funding was received for this study.
**Author Disclosures:**
Karabekir Ercan: Nothing to disclose
Semra Duran: Nothing to disclose
Yasin Celal Güneş: Nothing to disclose
Ebru Öztürk: Nothing to disclose

**16:00-17:30** **Research Stage 1**

### Research Presentation Session: Artificial Intelligence & Machine Learning & Imaging Informatics

### RPS 1105

### Radiomics in abdominal imaging and beyond

**Moderator**
M. Starmans; Rotterdam/NL
*(m.starmans@erasmusmc.nl)*

### RPS 1105-2

**What can radiogenomics offer to the radiologist: results from a systematic review**
*K. B. Holst*[1], A. R. Arildskov[1], F. J. Bruun[1], L. H. Hasselbach[1], S. H. Rasmussen[1], C. Borras[2], R. Mirón Mombiela[1]; [1]Herlev/DK, [2]Valencia/ES

**Purpose:** Radiomics refers to extraction of data from clinical images and is motivated by the concept that biomedical images contain information that reflects the underlying pathophysiology. It has been used in the field of oncology for example for outcome prediction and treatment response. We here present the results from a published systematic review focusing on studies using genetics to validate their radiomics models and outcomes and assess their contribution to the radiomics field.
**Methods or Backround:** All original research with the words radiomics and genomics in English and performed in humans up to 31.01.2022 were identified on Medline and Embase. The quality of the studies was assessed with Radiomic Quality Score (RQS) and the Cochrane recommendation for Quality Assessment on Diagnostic Accuracy Studies 2.
**Results or Findings:** 45 studies were included in our systematic review, and more than 50% were published in the last two years. The studies had a mean RQS of 12, and the studied tumours were very diverse. Up to 83% investigated the prognosis as the main outcome, with the rest focusing on response to treatment and risk assessment. Most applied either transcriptomics (54%) and/or genetics (35%) for genetic validation.
**Conclusion:** We present an emerging association between radiological features and genomic/molecular expression with the ability to explain underlying disease mechanisms and enhance prognostic assessment, risk assessment, and treatment response in cancer patients.
**Limitations:** Despite the rapidly increasing number of publications, there is still no real clinical use of the developed radiomics signatures so far. This is not surprising due to the low level of evidence and many biases.
**Ethics committee approval:** No information provided by the submitter.
**Funding for this study:** No funding was received for this study.
**Author Disclosures:**
Kristine Bærentz Holst: Nothing to disclose
Anne Rix Arildskov: Nothing to disclose
Rebeca Mirón Mombiela: Nothing to disclose
Sine Hvid Rasmussen: Nothing to disclose
Frederik Jager Bruun: Nothing to disclose
Consuelo Borras: Nothing to disclose
Lotte Harries Hasselbach: Nothing to disclose

### RPS 1105-3

**Impact of ComBat harmonisation on MRI radiomics-based tissue classification: the results of a dual-centre study**
*D. Leithner*, P. Gibbs, M. E. Mayerhöfer; New York, NY/US

**Purpose:** The purpose of this study was to determine whether ComBat harmonisation improves MRI radiomics-based tissue classification for datasets obtained with MRI scanners and protocols of different vendors.
**Methods or Backround:** One hundred patients who had undergone non-contrast-enhanced, high-resolution T1w 3D GRE Dixon MRI (two scanners/vendors; 50 patients each) were retrospectively included. Volumes of interest were placed in three visually inseparable tissues on Dixon-water images: the liver, the spleen, and skeletal muscle. After resampling to 2x2x2 $mm^3$ voxels, gray-level histogram (GLH), co-occurrence matrix (GLCM), run-length matrix (GLRLM), and size-zone matrix (GLSZM) radiomic features were extracted. Tissue classification was performed on pooled data from both centers/scanners, (1) without harmonisation, (2) after ComBat harmonisation with empirical Bayes estimation (ComBat-B), and (3) after ComBat harmonisation without empirical Bayes estimation (ComBat-WB). A multi-layer perceptron neural network with a random 70/30% split into training and test data and 10 iterations was used to distinguish between the three tissue types, separately for each dataset and each radiomic feature class.
**Results or Findings:** Mean tissue classification accuracies (based on 10 iterations each) for unharmonised, ComBat-B-harmonised, and ComBat-WB-harmonised test data were: 47.3, 55.1, 57.5% for GLH; 42.3, 65.3, 71.7% for GLCM; 45.3, 78.3, 78.3% for GLRLM; and 48.4, 81.1, 89.4% for GLSZM. Accuracies were significantly higher for both ComBat-B- and ComBat-WB-harmonsed data than for unharmonised data for all feature classes (at P<0.001, respectively). For GLCM and GLSZM, ComBat-WB harmonisation provided higher accuracies than ComBat-B harmonisation (P<0.001).
**Conclusion:** ComBat harmonisation, especially when applied without empirical Bayes estimation, may be useful for multi-centre MRI radiomics studies that use pooled data from different scanners/vendors.
**Limitations:** ComBat was tested for a single pulse sequence (T1w 3D GRE Dixon), and one device per centre/vendor.
**Ethics committee approval:** The local institutional review board approved this study.
**Funding for this study:** This research was funded in part through the NIH/NCI Cancer Center Support Grant P30 CA008748.
**Author Disclosures:**
Marius E. Mayerhöfer: Speaker: Siemens, GE, BMS
Doris Leithner: Nothing to disclose
Peter Gibbs: Nothing to disclose

### RPS 1105-4

**Robust prediction of the characteristics of aggressiveness of pancreatic neuroendocrine neoplasms (PanNEN) based on CT radiomic features**
*J. Mushtaq*, M. Mori, D. Palumbo, V. Andreasi, S. Partelli, A. del Vecchio, M. Falconi, C. Fiorino, F. De Cobelli; Milan/IT

**Purpose:** To predict tumour grade (G1 vs. G2/3), distant metastasis (M+), metastatic lymph nodes (N+), and microvascular invasion (VI) of pancreatic neuroendocrine neoplasms (PanNEN) based on preoperative CT radiomic features (RFs), using a machine learning approach to ensure methodological robustness and reproducibility.
**Methods or Backround:** This retrospective study included 101 patients who underwent surgery for PanNEN; the entire population was divided into training (n=70) and validation (n=31) cohorts. After tumour segmentation on contrast-enhanced CT, RFs were extracted from unenhanced CT images using a previously validated methodology. Furthermore, using minimum redundancy and a bootstrap-based machine learning approach, conventional radiological and clinical features were combined with RFs in multivariate logistic regression models. Models were trained and validated for each endpoint using only RFs (RF_model) and both (radiomic and clinicoradiological) features (COMB_model).
**Results or Findings:** Twenty-five patients had G2/G3 tumours, 37 N+, 14 M+, and 38 had VI. Few independent radiomic and clinicoradiological features were identified from a total of 182 RFs initially extracted. The resulting models for M+ and G showed moderate to high performances: area under the curve (AUC) for training/validation cohorts were 0.85/0.77 (RF_model) and 0.81/0.81 (COMB_model) for M+ and 0.67/0.72 and 0.68/0.70 for G. Only the COMB model could be built for N+ and VI, with N+ performing worse (AUC=0.72/0.61) than VI (0.82/0.75). The negative predictive value was moderately high (≥0.75) for all endpoints.
**Conclusion:** A reliable presurgical prediction of the histological characteristics of PanNENs was achieved by combining a small number of radiomic and clinicoradiological features. The proposed methodology guarantees high model's generalisability.
**Limitations:** The study's limitations include its retrospective nature and the relatively small number of events observed. External validation is also required.

**Ethics committee approval:** Data were collected within the context of an Ethics Committee approved study (06/INT/2022) in patients who had signed an institutional procedure specific informed consent.
**Funding for this study:** The study was supported by an AIRC grant (IG 2019-23015).
**Author Disclosures:**
Claudio Fiorino: Nothing to disclose
Junaid Mushtaq: Nothing to disclose
Valentina Andreasi: Nothing to disclose
Diego Palumbo: Nothing to disclose
Antonella del Vecchio: Nothing to disclose
Massimo Falconi: Nothing to disclose
Francesco De Cobelli: Nothing to disclose
Martina Mori: Nothing to disclose
Stefano Partelli: Nothing to disclose

### RPS 1105-5

**Development and validation of a radiomics-based decision-making supporting tool to improve the management and outcome of pancreatic cancer patients**

*A. Mokhtari*[1], R. Casale[1], Z. Paquier[1], T. Guiot[1], Z. Salahuddin[2], H. C. Woodruff[2], P. Lambin[2], J-L. Van Laethem[1], M. A. Bali[1]; [1]Brussels/BE, [2]Maastricht/NL

**Purpose:** The aims of the study were to identify reliable clinical and radiomics features to build machine learning models predicting progression-free survival (PFS) and overall survival (OS) using pretreatment CT.
**Methods or Backround:** Pretreatment contrast-enhancement CT scanner of 253 pathology-confirmed pancreatic adenocarcinoma (PDAC) patients were retrospectively (between 2010 and 2019) analysed. Demographic, clinical, and survival data were collected from medical records. OS was used to stratify patients into a long or short survival group (OS≥10 months, OS<10 months). For PFS, patients were stratified into a long or short PFS group (PFS≥3 months, PFS<3 months). Lesions were semi-manually segmented using MIM 6.9.0 software (Cleveland, US) and radiomics features were extracted using Oncoradiomics research software (Liège, BE). A fourth-step method was applied to select features the most reproducible, repeatable, and the less redundant. Two third of patients were randomly assigned to training-validation dataset and the remaining third for testing. Based on Random Forest (RF), different machine learning models were trained and tested to predict OS and PFS. Model performances were assessed using receiver operating characteristic (ROC) curves and associated area under the curve (AUC). The AUCs were compared using DeLong test. Significance was set at p<0.05.
**Results or Findings:** 171 radiomics features were extracted. 6 different subgroups of features were selected (clinical & radiomics features, clinical features and radiomics features; both for OS and PFS). Subsequently, 6 RF models were trained and tested. The clinical & radiomics model was the most predictive for both OS (AUC=0.75) and PFS (AUC=0.66). Other models reached lower AUCs.
**Conclusion:** Radiomics is an emergent methodology which can be used to predict survival outcomes in PDAC. Combination of clinical and radiomics features reached better performances.
**Limitations:** Heterogeneity CT scanner acquisition protocol. Retrospective cohort.
**Ethics committee approval:** Comité d'éthique – Hopital Erasme (HUB); Comité d'éthique – Institut Jules Bordet (HUB)
**Funding for this study:** Grant "Les amis de Bordet".
**Author Disclosures:**
Zelda Paquier: Nothing to disclose
Ayoub Mokhtari: Nothing to disclose
Maria Antonietta Bali: Nothing to disclose
Jean-Luc Van Laethem: Nothing to disclose
Roberto Casale: Nothing to disclose
Thomas Guiot: Nothing to disclose
Zohaib Salahuddin: Nothing to disclose
Henry Christian Woodruff: Nothing to disclose
Philippe Lambin: Nothing to disclose

### RPS 1105-6

**Dynamic change of radiomic features in course of neoadjuvant chemotherapy predicts histological response of pancreatic adenocarcinoma**

*F. Prato*, D. Palumbo, M. Mori, G. Belfiori, S. Crippa, A. del Vecchio, M. Falconi, C. Fiorino, F. De Cobelli; Milan/IT
*(francesco.prato1996@libero.it)*

**Purpose:** Considering the high rate of recurrence in pancreatic adenocarcinoma patients undergoing primary surgery, neoadjuvant chemotherapy (nCT) has been proposed as the first line treatment for all patients (including upfront resectable ones). However, evaluation of disease response after nCT represents a major issue undermining the chance for an optimal therapeutic path; in this respect, we aimed to build a model to provide precise evaluation of nCT response using delta radiomics, a novel tool to analyse tissue variation at different timepoints.
**Methods or Backround:** After exclusion, 83 patients who underwent duodenocefalopancreasectomy after nCT between 01/2016 and 12/2020 were enrolled in this retrospective, monocentric study. 182 radiomics features (RFs) were extracted from the late arterial phase of both pre- and post-nCT CT scans. Most significant ΔRFs were selected using minimum redundancy, robustness against delineation uncertainty and machine learning bootstrap-based method; then, a multivariable Cox regression analysis was made for each endpoint: (1) Recurrence, (2) Tumor Regression Grade (TRG) and (3) N2 (metastases in ≥4 regional lymph-nodes).
**Results or Findings:** Patients present a median overall survival of 21 months (IQR:14-31). Forty-eight patients experienced disease recurrence (57.8%), fourteen patients revealed to be N2 (16.8%) and 63 (75.9%) had a poor or minimal regression grade (TRG 0-1). Regarding N2, the resulting radiomic model showed moderate-to-high performances (AUC 0.82, p=0.001) with a considerable NPV (93.9%). Concerning other endpoints, good performance was found for Recurrence (AUC 0.70, p=0.004) and TRG (AUC 0.72, p=0.008). For all endpoints, the negative predictive value was excellent (≥0.75).
**Conclusion:** Our model may help to better adapt treatment of upfront and borderline resectable neoadjuvated patients, by providing crucial information about biological response to nCT. Validation cohort is already planned.
**Limitations:** Single centre, monocentric study. Validation cohort is needed and already planned.
**Ethics committee approval:** 06/INT/2022
**Funding for this study:** No funding was received for this study.
**Author Disclosures:**
Claudio Fiorino: Nothing to disclose
Diego Palumbo: Nothing to disclose
Antonella del Vecchio: Nothing to disclose
Massimo Falconi: Nothing to disclose
Francesco De Cobelli: Nothing to disclose
Martina Mori: Nothing to disclose
Giulio Belfiori: Nothing to disclose
Francesco Prato: Nothing to disclose
Stefano Crippa: Nothing to disclose

### RPS 1105-7

**Transformation of the histopathological growth pattern of colorectal liver metastases after chemotherapy predicted by a CT-based radiomics models**

*S. Wei*, J. Cheng, Y. Wang; Beijing/CN

**Purpose:** To investigate the transformation of histopathological growth pattern (HGP) of colorectal liver metastases (CRLMs) before and after chemotherapy by constructing a CT-based radiomics model.
**Methods or Backround:** Patients with histopathological confirmed CRLMs were identified by multidetector CT (MDCT) between January 2007 and July 2022. The HGPs of CRLMs were classified as either pure desmoplastic HGP (pdHGP) or non-pdHGP. Radiomics analysis was performed on the tumour-liver interface (TLI) zone on the CT images of the pre- and post-contrast (arterial(A) and portal venous (PV)) phases. Logistic regression analyses and receiver operating characteristic (ROC) curves were used to evaluate the model performance. Furthermore, by using the predicting model, the pre-chemotherapy HGP was diagnosed for patients who underwent chemotherapy before resection according to pretreatment CT images.
**Results or Findings:** Among 181 patients (chemo-naive of 106 and post-chemotherapy of 75 patients), there were 299 CRLMs (90 pdHGP and 200 non-pdHGP) were used to construct the predicting model. The prevalence of pdHGP was 24% and 45.8% in chemo naive and post-chemotherapy, respectively, and with significant differences (P=0.039). The 3 phases fused and PVP radiomics signature demonstrated equally good predictive performance in distinguishing between pdHGP or non-pdHGPs (the AUC of the fused phases were 0.908, 0.884 and 0.731, the AUC of the PVP were 0.906, 0.877 and 0.713 in the training, internal validation and external validation group, respectively). By using the PVP radiomic model, the HGP type could be seen for lesions before and after chemotherapy, with the pdHGP from 35.4% to 44.7%.
**Conclusion:** PdHGP could be more prevalent in post-chemotherapy CRLMs, which could be explained by HGP transformation testified by a radiomics HGP predicting model derived from MDCT images.
**Limitations:** (1) Retrospective study, small cases. (2) Not evaluated other rare HGP.
**Ethics committee approval:** This observational, retrospective study was approved by the institutional review boards (IRBs) of all institutions, the waivers of informed consent were obtained.
**Funding for this study:** License Number: 2019PHE058
**Author Disclosures:**
Jin Cheng: Nothing to disclose
Shengcai Wei: Nothing to disclose
YI Wang: Nothing to disclose

### RPS 1105-8

**Development and external validation of a non-invasive imaging biomarker to estimate the microsatellite instability status of gastric cancer and its prognostic value**

*H. Zhao*; Xian/CN
*(kindergirl@sina.cn)*

**Purpose:** This study aimed to develop and validate a non-invasive imaging biomarker for MSI assessment in GC and explore its prognostic value.
**Methods or Backround:** We retrospectively recruited 396 GC patients with pretreatment CT images from a single centre and a public database and divided them into an original cohort (n=356) and an external validation cohort (n=40). The SMOTE algorithm was used to generate a balanced training cohort (n=192) and the independent radiomics model, clinical model, and radiomics-clinic combined model were constructed for determining MSI status. The models' discrimination, calibration, clinical usefulness, and prognosis significance were evaluated by AUC, calibration, decision curve analyses, and Kaplan-Meier curve analysis, respectively.
**Results or Findings:** The radiomics-clinic combined model derived from clinical and quantitative CT-based "Radscore" exhibited the best discriminatory abilities of MSI status in all cohorts, with AUCs of 0.8363 (95% CI, 0.7800-0.8925) in the training cohort, 0.8343 (95% CI, 0.6881-0.9805) in the external validation cohort, and 0.7503 (95% CI, 0.6816-0.8189) in the original cohort, respectively. Meanwhile, the combined model demonstrated goodness of fitness, higher clinical net benefits, and significant positive integrated discrimination improvement compared with any independent model. While it showed no significant overall survival- or progression-free survival-based risk stratification ability (p>0.05).
**Conclusion:** The radiomics-clinic combined model could be a potential non-invasive biomarker for MSI status in GC, which help clinical decision-making, nevertheless, provided limited prognostic ability.
**Limitations:** First, as a retrospective study, the generalisability of our diagnosing model needs to be further explored in a multicentre prospective study with a larger cohort. Second, 2D segmentation was implemented on a single slice rather than three-dimensional (3D) segmentation covering the entire tumour.
**Ethics committee approval:** This study was approved by our Institutional Review Board and the written informed consent was waived due to the retrospective nature.
**Funding for this study:** National Natural Science Foundation of China (Grant No. 81671682, 81971615).
**Author Disclosures:**
Huiping Zhao: Nothing to disclose

### RPS 1105-9

**CT-based radiomics for prediction of programmed death ligand 1 (PD-L1) expression in urothelial carcinoma**

C. Lee, S-B. Hong, *S. Koh*, N. Lee, S. Kim; Busan/KR

**Purpose:** To develop a prediction model that can differentiate positive programmed death ligand 1 (PD-L1) expression from negative PD-L1 expression through radiomics-based investigation of computed tomography (CT) images in patients with urothelial carcinoma.
**Methods or Backround:** Sixty-four patients with urothelial carcinoma who underwent PD-L1 immunohistochemistry were retrospectively reviewed. CT images obtained 90 s after contrast media administration were selected for radiomic extraction. For the enhancing mass that appeared to be cancerous on CT, 1691 radiomic features were extracted with manually segmented regions of interest. Univariate and multivariate logistic regression analyses were performed to identify significant radiomic features for predicting the PD-L1 expression rate. A comprehensive model with significant radiomic features was obtained using logistic regression analysis. For the radiomics-based model, a receiver operating characteristic (ROC) analysis was performed.
**Results or Findings:** Among the 64 patients, 14 were included in the PD-L1 positive group. In logistic regression analysis, wavelet-LLH_glszm_SmallAreaEmphasis (odds ratio: 15.5838, p < 0.001) and wavelet-LLH_firstorder_TotalEnergy (odds ratio: 0.0058, p = 0.006) were significant radiomic features for predicting the PD-L1 positive group. Radiomics signature was -7.6007 + 15.5838 [wavelet-LLH_glszm_SmallAreaEmphasis] + 0.0058 [wavelet-LLH_firstorder_TotalEnergy]. The area under the ROC curve model representing the radiomics signature for differentiating the PD-L1 immunohistochemical positive group from the other group was 0.89. The sensitivity, specificity, positive likelihood ratio, and negative likelihood ratio of the prediction model were 92.86%, 82.00%, 5.16, and 0.09, respectively.
**Conclusion:** We developed a radiomics prediction model derived from contrast-enhanced CT, which may be a potential non-invasive tool for diagnosing PD-L1 positivity in patients with urothelial cancer.
**Limitations:** This is retrospective study. The study population included patients with urothelial carcinoma; however, the sites of each mass were diverse. Validation through the control for the signature equation was not performed.
**Ethics committee approval:** Pusan National University Institutional Review Board
**Funding for this study:** Nothing to declare.
**Author Disclosures:**
Suk Kim: Nothing to disclose
Seung-Baek Hong: Nothing to disclose
Sungho Koh: Nothing to disclose
Namkyung Lee: Nothing to disclose
Changmu Lee: Nothing to disclose

### RPS 1105-10

**Use of radiomics in the differential diagnosis of adrenal location neuroblastoma and wilms tumours in children**

*I. Ö. Koska*[1], H. N. Özcan[2], A. A. Tan[2], B. Beydoğan[2], G. Tufan Özer[2], B. Oğuz[2], M. Haliloğlu[2]; [1]Izmir/TR, [2]Ankara/TR
*(ozgurkoska@icloud.com)*

**Purpose:** Neuroblastoma and Wilms tumour are common intra-abdominal tumours in childhood. It is not always possible to distinguish large neuroblastoma from Wilms tumour with imaging methods. In this study, our aim is to differentiate between Wilms tumour and neuroblastoma located in the adrenal location by using radiomics methods.
**Methods or Backround:** CT scans of 30 patients with Wilms tumour and 30 neuroblastoma were evaluated using radiomics methods. The patients were segmented by 2 different radiologists. Pyradromics package with original images, Wavelet and LoG (laplace of Gaussian) transformed versions were used. Robust and non-redundant features were selected. Combined feature set (CFS) and extended feature set (EFS) were obtained. The features were projected into 2D using t-SNE method. Model training was done with support vector machine, XGBoost and Random Forest methods.
**Results or Findings:** The mean accuracy of the support vector machine model was found to be 0.84 for all features, and the standard deviation was 0.078. The performance of the XGBoost model was also found to be similar and stable in 5 features (average accuracy 0.82, standard deviation 0.097). In the random forest model, slightly different statistical values were obtained for these features, with the mean accuracy 0.83 in MRMR, standard deviation 0.08. Higher mean cross validation values and less dispersion were obtained in all features with the support vector machine.
**Conclusion:** Some neuroblastoma and Wilms tumours are difficult to distinguish with classical imaging methods, and this may delay the treatment that the patient should receive. Radiomics is promising in diagnosing adrenal masses that cannot be differentiated from neuroblastoma and Wilms tumour.
**Limitations:** Small sample size was the main limitation of our study. A second limitation was that all the patients were examined in a single centre, therefore data diversity was limited.
**Ethics committee approval:** Hacettepe University Ethical Board
**Funding for this study:** No funding was received for this study.
**Author Disclosures:**
Gözde Tufan Özer: Nothing to disclose
Berna Oğuz: Nothing to disclose
Mithat Haliloğlu: Nothing to disclose
Ilker Özgür Koska: Nothing to disclose
Beyza Beydoğan: Nothing to disclose
Hatice Nursun Özcan: Nothing to disclose
Aziz Anıl Tan: Nothing to disclose

### RPS 1105-11

**Optimised automated workflow for radiomic feature extraction from static and dynamic Magnetic Resonance Imaging (MRI) of soft tissue sarcoma**

*E. Duran-Sierra*, R. F. Valenzuela, M. Canjirathinkal, C. Costelloe, H. Moradi, J. Madewell, W. Murphy, B. Amini; Houston, TX/US
*(eduran3@mdanderson.org)*

**Purpose:** The purpose of this study was to develop a high-throughput vendor-neutral software solution for cost-efficient image processing workflow and radiomic feature extraction.
**Methods or Backround:** A Python-based software application was developed for image processing and feature extraction from conventional and advanced MRI sequences. The application uses contour files generated using a best-in-class application (MIM) and performs automated radiomic feature extraction. A separate perfusion subroutine processes dynamic contrast-enhanced images. The application automatically populates a relational database. This workflow solution was validated using a real-world dataset of 25 sarcoma patients imaged at multiple time points during treatment. A performance comparison was performed against an open-source software application (3D Slicer) and four commercial software applications available at our institution (MIM, AW-GE, Syngo, and DynaCAD).
**Results or Findings:** A total of 225 volumetric contour files and corresponding MRI series were processed using our application. The application performed automated extraction of 107 radiomic features from multiple MRI sequences originating from our installed fleet of 40 magnets (from two manufacturers). The perfusion subroutine estimated seven semiquantitative features extracted from

Thursday time-intensity curves. Commercial applications could not process the same number of contours due to limitations on the types of sequences supported and features pertinent to the scanner manufacturer. Syngo was able to process perfusion data originating only from Siemen's magnets. DynaCAD's license-related constraints limited the diffusion and perfusion processing to a reduced number of MRI scanners. All four commercial applications were limited in the number of radiomic and perfusion features they could process compared to our software application.
**Conclusion:** Our optimised application provides an automated, robust, vendor-neutral, high-throughput workflow for MRI data processing and feature extraction.
**Limitations:** The small sample size of 25 patients was identitied as a limitation.
**Ethics committee approval:** No information provided by the submitter.
**Funding for this study:** Funding was provided by John S. Dunn, Sr. Distinguished Chair in diagnostic imaging as well as the M.R. Evelyn Hudson Foundation Endowed Professorship.
**Author Disclosures:**
Elvis Duran-Sierra: Nothing to disclose
Behrang Amini: Nothing to disclose
Colleen Costelloe: Nothing to disclose
Heerod Moradi: Nothing to disclose
Raul Fernando Valenzuela: Nothing to disclose
William Murphy: Nothing to disclose
Mathew Canjirathinkal: Nothing to disclose
John Madewell: Nothing to disclose

**16:00-17:30** **Research Stage 2**

### Research Presentation Session: Chest

### RPS 1104

### Pulmonary nodules and more: oncologic imaging

**Moderator**
L. Beer; Vienna/AT
*(Lucian.beer@meduniwien.ac.at)*
**Author Disclosures:**
Lucian Beer: Research Grant/Support: Bayer, MDS, Novartis

### RPS 1104-2

**Extent and sources of disagreement between expert mesothelioma radiologists reporting therapeutic response using mRECIST 1.1 criteria: a multicentre retrospective cohort study**
*G. Cowell*, C. Noble, D. Stobo, A. Kidd, S. Tsim, K. Blyth; Glasgow/UK
*(gordon.cowell@ggc.scot.nhs.uk)*

**Purpose:** Malignant Pleural Mesothelioma (MPM) therapeutics need accurate radiological response classification. The concentric growth pattern of MPM violates the spherical growth assumptions required for unidirectional tumour measurement, but reliance persists. We evaluated extent and sources of discordant response classification.
**Methods or Backround:** We performed a multi-centre retrospective cohort study, involving 172 chemotherapy-treated MPM patients from 3 UK centres. Baseline and response CTs reporting was undertaken using mRECIST 1.1 by 2 expert MPM radiologists, both contributing to the Scottish National Mesothelioma MDT, +/- a third in discordant classifications (progressive disease (PD), stable disease (SD), partial disease response (PR)). Interrater reliability (IRR) was assessed using Cohen's kappa. A root cause analysis identified themes in discordant cases.
**Results or Findings:** 112/172 patients (65.1%) had concordant classification by both reviewers. Of 60/172 (34.9%) discordant cases, 50/172 patients (29.1%) crossed one classification group (PR versus SD, SD versus PD), whilst 10 patients (8.9%) crossed two (PR versus PD). IRR was moderate (kappa = 0.456, 0.346-0.566). Among discordant cases spanning 2 groups, 7 (70%) reviewed wrong imaging, 2 (20%) incorrectly applied mRECIST and 1 (10%) had a missed finding. Among discordant cases spanning 1 group, 24 (48%) had differing interpretation, 14 (28%) had <10% response difference, 4 (8%) incorrectly applied mRECIST, 4 (8%) had missed findings, 2 (4%) had recording errors, and 2 (4%) reviewed wrong imaging.
**Conclusion:** mRECIST response classification is inconsistent even among expert MPM radiologists. Multiple disagreement sources include human processing errors and response assessment criteria that oversimplify complex tumour morphology. Greater automation minimising human error, maximising consistency and enabling practical volumetry deployment is essential.
**Limitations:** Performance assessment in prospective clinical settings is required.
**Ethics committee approval:** No information provided by the submitter.
**Funding for this study:** This analysis is based on the PRISM study (Prediction of Chemoresistance based on Somatic Copy Number Variation in Mesothelioma): Funding Ref MPG16-7.
**Author Disclosures:**
David Stobo: Nothing to disclose
Selina Tsim: Nothing to disclose
Gordon Cowell: Nothing to disclose
Colin Noble: Nothing to disclose
Andrew Kidd: Nothing to disclose
Kevin Blyth: Nothing to disclose

### RPS 1104-3

**Can we trust AI? A comparison of AI software tools for automated detection, quantification and categorisation of pulmonary nodules in the HANSE LCS trial**
*R. Kondrashova*[1], T. F. Kaireit[1], J. Sperl[1], K. May[2], S. Stiebeler[3], J. Barkhausen[2], F. Wacker[1], S. Dettmer[1], J. Vogel-Claussen[1]; [1]Hannover/DE, [2]Lübeck/DE, [3]Grosshansdorf/DE

**Purpose:** To evaluate and compare the performance of two AI-based software tools for detection, quantification and categorisation of pulmonary nodules in a lung cancer screening (LCS) program in Northern Germany (HANSE trial).
**Methods or Backround:** Patient management depends on the assigned Lung Imaging Reporting and Data System (Lung-RADS) category, which is based on reliable detection and volumetry of pulmonary nodules. If a different Lung-RADS grade is assigned to the same subject by an alternative software, the patient may be treated differently. In our study 946 low-dose CT examinations in the HANSE LCS trial were analysed by two AI software tools regarding the lung nodule categorisation, quantification and detection and compared to the final radiologist read. The relationship between detected nodule volumes by both software tools was assessed by Pearson correlation (r) and tested for significance using Wilcoxon signed-rank test. The consistency of Lung-RADS classifications was evaluated by Cohen's kappa (κ) and percentual agreement.
**Results or Findings:** 88% and 66% of all (solid, semi-solid and ground glas) lung nodules (volume≥34mm3) were detected by Software tool 1 (S1) and Software tool 2 (S2), respectively. Although, the derived volumes of true positive nodules were strongly correlated (r>0.95), the volume derived by S2 was significantly higher than by S1 (P<0.0001, mean difference: 6mm3). Moderate percentual agreement (>54%) between S1 and S2 was found in the assignment of Lung-RADS classification (κ=0.41).
**Conclusion:** Significant nodule volume differences between AI software tools result in different Lung-RADS scores, which may cause altered participant management. Therefore, high performance and agreement of accredited AI software tools is necessary in a future national LCS program.
**Limitations:** S1 is used in the HANSE study and might influence the final decision of the radiologist (gold standard).
**Ethics committee approval:** No information provided by the submitter.
**Funding for this study:** No funding was received for this study.
**Author Disclosures:**
Katharina May: Nothing to disclose
Rimma Kondrashova: Nothing to disclose
Till F Kaireit: Nothing to disclose
Jens Vogel-Claussen: Nothing to disclose
Sabine Dettmer: Nothing to disclose
Susanne Stiebeler: Nothing to disclose
Jonathan Sperl: Employee: Siemens Healthineers
Jörg Barkhausen: Nothing to disclose
Frank Wacker: Nothing to disclose

### RPS 1104-4

**Risk assessment of persistent incidental pulmonary subsolid nodules to guide the appropriate surveillance interval and endpoint**
*M. Liu*, X. Liang, R. Zheng, S. Yang, M. Li, L. Zhang; Beijing/CN
*(liumengwen2021@163.com)*

**Purpose:** The longitudinal risks of pulmonary subsolid nodules (SSNs) growth for different nodule consistency, multiplicity, and size were assessed, to explore the appropriate surveillance interval and endpoint for the follow-up of SSNs.
**Methods or Backround:** HRCT data of SSNs from May 2005 to August 2022 were reviewed. The effect of nodule consistency, size, and multiplicity on growth was explored. The absolute risk of SSNs growth in each follow-up year was assessed by the immediate risk (IR) and cumulative risk (CR) using the life-table method.
**Results or Findings:** A total of 892 SSNs were included in this study, of which 833 were pure ground glass nodules (pGGNs), and 59 were part-solid nodules (PSNs). The growing and stable SSNs have significant differences in consistency and size of nodules (P<.001), but not in multiplicity (P=.55). For pGGNs≤6.6 mm, the IR of nodule growth remained below 5.00% for 14 years, and the CRs of two or three years were above 5.00% in the first 8 or 9 years. For pGGNs of 6.6 to 8.8 mm and larger than 8.8 mm, the IR remained above

5.00% for the first 2-7 years. The IR of PSN growth reached the highest in the fourth year (44.00%) and then decreased with only one or no nodule growth per year.
**Conclusion:** For pGGN≤6.6 mm, the follow-up can be done every 2 or 3 years for at least 8 years, for pGGN of 6.6 to 8.8 mm and larger than 8.8 mm, at least 7 years of annual repeat follow-up is required, and for PSN, continuous annual repeat follow-up is required.
**Limitations:** Not applicable
**Ethics committee approval:** This retrospective study was approved by the Institutional Review Board of Cancer Hospital Chinese Academy of Medical Sciences (approval number: NCC2017G-093), and the requirement for informed consent was waived.
**Funding for this study:** Funding was received from the CAMS Innovation Fund for Medical Sciences (2022-I2M-C&T-B-076), CAMS Innovation Fund for Medical Sciences (2021-I2M-C&T-B-061), Beijing Municipal Natural Science Foundation (7184238), and the National Natural Science Foundation of China (81701692).
**Author Disclosures:**
Rongshou Zheng: Nothing to disclose
Li Zhang: Nothing to disclose
Xin Liang: Nothing to disclose
Meng Li: Nothing to disclose
Shouxin Yang: Nothing to disclose
Mengwen Liu: Nothing to disclose

### RPS 1104-5

**Diagnostic performance of a deep learning-based method in the differential diagnosis of benign and malignant subcentimeter solid pulmonary nodules**

*J. Liu*[1], L. Qi[1], Y. Wang[1], S. Cheng[1], F. Li[1], J. Chen[1], Z. Zhou[1], Y. Yu[2], J. Wang[1]; [1]Beijing/CN, [2]Hong Kong/CN
*(sjz-ljn2008@163.com)*

**Purpose:** The aim of this study was to assess the diagnostic performance of a deep learning model for subcentimeter solid pulmonary nodules (SSPNs) by comparing it with radiologists' diagnoses using chest computed tomography (CT).
**Methods or Backround:** In 200 retrospectively collected SSPNs (100 malignant), malignancy was confirmed pathologically, and benignity was confirmed by follow-up CT or pathological analysis. CT images were fed into the deep learning model to obtain the probability of malignancy for each nodule (0–100%). According to the diagnostic results, the nodules were classified as benign, malignant, and indeterminate. The diagnostic performance of the model was compared with that of the radiologists. Moreover, all nodules were further divided into the 3–6-, 6–8-, and 8–10-mm groups by the nodule size. For each group, the diagnostic results of the model were compared with those of the radiologists.
**Results or Findings:** There was a statistically significant difference between the model and the radiologists' diagnoses (p < 0.001). The model reported more benign and malignant results and less indeterminate results. The accuracy of the model was higher than that of the radiologists (71.5% vs 38.5%, p < 0.001). Analysis of the three groups also showed that the model achieved higher diagnostic accuracy than the radiologists' approach and provided less indeterminate results than the radiologists' approach.
**Conclusion:** The deep learning-based method presents better diagnostic performance than radiologists in differentiating SSPNs, with higher diagnostic accuracy and certainty. This performance is not affected by nodule size and may aid radiologists in making more reasonable diagnoses, especially for nodules < 8 mm.
**Limitations:** The following limitations were identified: single-centre study, lack of comparisons with other studies.
**Ethics committee approval:** Ethics committee approval was obtained.
**Funding for this study:** Funding was received via the National Natural Science Foundation of China (grant 81971616) and the Beijing Natural Science Foundation (grant 7222148).
**Author Disclosures:**
Yizhou Yu: Nothing to disclose
Jianwei Wang: Nothing to disclose
Yawen Wang: Nothing to disclose
Linlin Qi: Nothing to disclose
Jianing Liu: Nothing to disclose
Jiaqi Chen: Nothing to disclose
Cancer Hospital Chinese Academy of Medical Sciences Sainan Cheng: Nothing to disclose
Zhen Zhou: Nothing to disclose
Fenglan Li: Nothing to disclose

### RPS 1104-6

**Spectral CT for the evaluation of pulmonary subsolid nodules: impact of quantitative reconstruction methods on measurement accuracy and image quality**

*L. Ding*[1], X. Li[1], J. Lin[1], S. Deng[1], M. Chen[1], W. Deng[2], Y. Xu[1], C. Yan[1]; [1]Guangzhou/CN, [2]Shanghai/CN
*(dingli_00@163.com)*

**Purpose:** The aim of this study was to evaluate the image quality and measurement accuracy of pulmonary subsolid nodules on spectral CT quantitative reconstruction images and polyenergetic-images with iterative reconstructions.
**Methods or Backround:** This prospective study recruited 270 patients who underwent dual-layer spectral CT scan for lung nodule follow-up. All CT examinations with subsolid nodules [pure ground-glass nodules (GGNs) or part-solid nodules] were reconstructed with electron density mapping (EDM), virtual monoenergetic imaging at different keV levels, hybrid and model–based iterative reconstruction (IR) algorithms. The objective image noise, signal-to-noise ratio, contrast-to-noise ratio, diameter, and volume of GGNs were measured for quantitative analysis. The overall image quality, image noise and visualisation of nodules were subjectively rated by two thoracic radiologists on a five-point scale (1 = unacceptable, 5 = excellent).
**Results or Findings:** A total of 198 subsolid nodules were identified, including 179 pure GGNs, and 19 part-solid nodules. Based on the objective analysis, EDM had the highest signal-to-noise ratio (164.71±133.60; p < 0.001) and contrast-to-noise ratio (227.97±161.96; p < 0.001) among all reconstructed image sets. Compared to hybrid IR, EDM resulted in consistently increased diameters (8.35±3.30 vs 7.94±3.22) and volumes (219.62±337.17 vs 208.84±318.26) for GGNs. Furthermore, EDM had a superior subjective rating score for the visualisation of GGNs compared with other reconstructed images (p < 0.001).
**Conclusion:** Quantitative spectral CT functional imaging enabled improved image quality and lesion conspicuity for the evaluation of lung subsolid nodules compared with IR algorithms.
**Limitations:** Not applicable
**Ethics committee approval:** This study was approved by the medical ethics committee of Nanfang hospital of Southern Medical University: NFEC-2021-371.
**Funding for this study:** Funding was provided by the National Natural Science Foundation of China (82271987and 82271939).
**Author Disclosures:**
Mingwang Chen: Nothing to disclose
Yikai Xu: Nothing to disclose
Chenggong Yan: Nothing to disclose
Jie Lin: Nothing to disclose
Shuting Deng: Nothing to disclose
Weiwei Deng: Nothing to disclose
Xiaomei Li: Nothing to disclose
Li Ding: Nothing to disclose

### RPS 1104-7

**Volumetric classification of pulmonary nodules in lung cancer screening: the influence of reconstruction parameters**

Y. Mao[1], M. Vonder[1], D. Han[1], M. Dorrius[1], J. Yi[2], G. de Bock[1], Z. Ye[3], M. Oudkerk[1], *H. L. Lancaster*[1]; [1]Groningen/NL, [2]Seoul/KR, [3]Tianjin/CN
*(h.l.lancaster@umcg.nl)*

**Purpose:** Previous studies have explored manual or semi-automatic volumetric pulmonary nodule measurement variability between different reconstruction settings, but no data are available from fully-automatic AI reading. Our aim was to evaluate the impact of reconstruction parameters on volumetric nodule classification in a LDCT lung cancer baseline screening dataset.
**Methods or Backround:** 359 individuals with at least one non-calcified solid-component nodule on their baseline LDCT scans in a Chinese lung cancer screening were included. Volumetric nodule measurements were performed for each scan with two medium-soft (D45f/1mm, and D45f/2mm) and one soft kernel (B30f/2mm), using an AI-based software (AVIEW LCS, v1.0.39, Coreline Soft). The largest non-calcified solid-component nodule per scan was determined. The classification was based on 100 mm3 NELSON-plus/EUPS protocol threshold. A separate agreement analysis was done for small-nodule classification, defined as cases with 30-100 mm3 as the largest solid nodule or the largest solid-component of subsolid nodule. Agreement between different slice thicknesses (D45f/1mm vs D45f/2mm) and kernels (B30f/2mm vs D45f/2mm) was evaluated by statistical analysis with Cohen's Kappa.
**Results or Findings:** The nodule classification based on a 100 mm3 threshold showed a 91.1% agreement ($\kappa$ = 0.80) between different slice thicknesses and between different kernels. The small-nodule classification showed a 72.2% agreement ($\kappa$ = 0.16) between slice thicknesses and a 66.5% agreement ($\kappa$ = 0.21) between different kernels.

Thursday

**Conclusion:** AI volumetric nodule classification generally showed suboptimal agreement between convolution kernels and slice thicknesses. For small nodules <100mm3 relevant to new nodule detection, results show strict consistency in reconstruction parameters is required for volume CT.
**Limitations:** The generalisability of our results might be limited by CT vendor specific characteristics.
**Ethics committee approval:** No information provided by the submitter.
**Funding for this study:** Funding for this study was provided by the Royal Netherlands Academy of Arts and Sciences (grant PSA_SA_BD_01) as well as the China Scholarship Council (CSC no. 202008440409).
**Author Disclosures:**
Monique Dorrius: Nothing to disclose
Harriet Louise Lancaster: Nothing to disclose
Geertruida de Bock: Nothing to disclose
Zhaoxiang Ye: Nothing to disclose
Yifei Mao: Nothing to disclose
Matthijs Oudkerk: Nothing to disclose
Jaeyoun Yi: Nothing to disclose
Marleen Vonder: Nothing to disclose
Daiwei Han: Nothing to disclose

### RPS 1104-8

**Tumour volume doubling time in lung cancer: a systematic review and meta-analysis**

*B. Jiang*[1], D. Han[2], M. Heuvelmans[2], C. van der Aalst[1], H. J. de Koning[1], M. Oudkerk[2]; [1]Rotterdam/NL, [2]Groningen/NL
*(beibei.jiang.research@outlook.com)*

**Purpose:** Tumour growth patterns have important implications for prognostication, determining screening intervals, and treatment decisions. Nevertheless, these have not been well described in lung cancer. The aim of our study was to characterise growth patterns of primary tumours in lung cancer and identify correlations for rapid and indolent growth patterns.
**Methods or Backround:** We are performing a systematic literature review of Medline, EMBASE, and web of science databases starting from 2004 until April 2022. We have identified studies reporting tumour growth or volumetric measured tumour volume doubling time (VDT) of pathologically confirmed primary lung cancer without intervention, and abstracted data to calculate pooled VDT and find correlations of growth patterns (rapid defined as VDT ≤ 400 days, indolent as VDT > 400 days, non-growth or shrinkage). Pooled mean VDT was calculated using a random-effects model.
**Results or Findings:** We have identified 28 studies, including 2682 patients with primary lung cancer. Pooled overall mean VDT was 222 days (95% CI 168 to 276 days, I2=92.5%), with 64.4% classified as rapid and 35.6% as indolent growth. In subgroup analyses, pooled mean VDT of adenocarcinoma, squamous cell, small cell, and other lung cancer were 315, 135, 71, and 192 days, respectively, and pooled mean VDT of non-solid, part-solid, and solid nodule types were 721, 513, and 267 days, respectively. The most consistent correlations of indolent tumour growth included subsolid type and adenocarcinoma histology.
**Conclusion:** The pooled mean VDT of primary lung cancer is approximately 222 days and around one third is indolent; the subsolid type and adenocarcinoma demonstrated the most consistent correlations.
**Limitations:** The remaining data analysis was limited by small study sample sizes (range 27-149 cases) and the heterogeneity of material and methods of the studies.
**Ethics committee approval:** No information provided by the submitter.
**Funding for this study:** No funding was received for this study.
**Author Disclosures:**
Beibei Jiang: Nothing to disclose
Harry J. de Koning: Nothing to disclose
Matthijs Oudkerk: Nothing to disclose
Marjolein Heuvelmans: Nothing to disclose
Daiwei Han: Nothing to disclose
Carlijn van der Aalst: Nothing to disclose

### RPS 1104-9

**Preoperative risk factors for early recurrence in patients with pathological stage IIIA-N2 non-small cell lung cancer after upfront surgery: prognostic value of new N stage subcategories**

*N-E. Oh*, J. Choe; Seoul/KR
*(naeunoh.rad@gmail.com)*

**Purpose:** To evaluate the preoperative risk factors in patients with IIIA-N2 non-small cell lung cancer (NSCLC) who underwent upfront surgery and evaluate the prognostic value of new N stage subcategories.
**Methods or Backround:** Patients with pathologic stage IIIA-N2 who underwent upfront surgery in a single tertiary centre from January 2015 to April 2021 were retrospectively reviewed. Each patient's clinical N (cN) stage was assigned to one of six categories (cN0, N1a, N1b, N2a1, N2a2, and N2b). Cox regression analysis was used to identify the significant prognostic factors for recurrence-free survival (RFS) and overall survival (OS).
**Results or Findings:** A total of 403 patients (mean age, 62.4±10.1, 178 women [44.2%]) were analysed. 30 patients (7.4%) recurred within 6 months. Multivariable analysis demonstrated that subcategories of clinical N staging (Hazard ratio [HR] for cN1, cN2a1, cN2a2, cN2b compared with cN0: 1.58, 1.11, 1.50 and 1.90; P=0.015, 0.636, 0.029 and 0.002, respectively) and maximum lymph node (LN) size at N1 station (≥12mm, HR: 1.67;P=0.002) were independent risk factors for RFS. Subcategories of pathological N2b stage (compared with N2a1; HR, 4.50; P<0.001) and early recurrence within 6 months (HR: 5.61; P<0.001) were independent prognostic factors for OS after adjustment. Regarding preoperative risk factors, clinical N2b and N2a2 stages (vs N0, HR: 1.74; P=0.009) and maximum LN size at N1 station (≥12mm, HR: 1.82, P=0.005) were also independent prognostic factors for OS after adjustment.
**Conclusion:** Assessing new N stage subcategories and reporting the maximum size of LN metastasis reflecting metastatic burden on preoperative CT may serve as important prognostic factors predicting early postoperative recurrence in stage IIIA-N2 patients.
**Limitations:** This was a retrospective study in a single centre, which can cause selection bias.
**Ethics committee approval:** The study was approved by the Institutional Review Board at Asan Medical Center, which waived the requirement for informed consent due to the retrospective nature of the study.
**Funding for this study:** No funding was received for this study.
**Author Disclosures:**
Na-Eun Oh: Nothing to disclose
Jooae Choe: Nothing to disclose

### RPS 1104-10

**One-stop shop percutaneous CT-guided multibiopsies in lung cancer patients: feasibility and safety**

*S. Ventura Díaz*, L. Gorospe Sarasua, M. Á. Gómez Bermejo, A. Michael-Fernández, A. González-Huete, E. Antolinos-Macho, A. Salgado Parente, A. Benito-Berlinches, P. Garrido-López; Madrid/ES
*(sofi9417vd@gmail.com)*

**Purpose:** To demonstrate that percutaneous CT-guided multibiopsies (PCTMB) performed in a single interventional procedure ("one-stop shop") in lung cancer patients with more than one lung lesion (including bilateral lesions) or with suspected oligometastatic extrapulmonary disease is clinically feasible, safe, and may accelerate correct diagnosis and staging, thus optimising the diagnostic and therapeutic management. We also suggest some tips and tricks for the interventional thoracic radiologist.
**Methods or Backround:** This was a pilot case-control study where we extracted clinical and demographic data, duration and type of procedure, histology, complications, antiplatelet/anticoagulant treatment, and length of hospital stay after the procedure. We compare the results with a control of 35 patients.
**Results or Findings:** Thirty cases (83.3% male, median age 72 years) underwent one-stop shop PCTMB between 2013 and 2022. Two patients had 2 ipsilateral lung lesions, 4 bilateral lung lesions (one in each lung), and 24 one pulmonary lesion and at least one extrapulmonary lesion. The median time to diagnostic histology was 4 days (0-20 days). Eight patients (27%) developed pneumothorax (similar to the control group [24%]), and only one required chest tube. No other major complications occurred. The median duration of the PTCMB procedure was 36 minutes, just 16 minutes more than in the control group. The hospital length of stay after the procedure was 18 hours, more than in the control group (6 hours).
**Conclusion:** One-stop shop PCTMB in lung cancer patients with more than one lung lesion or with suspected oligometastatic extrapulmonary disease is feasible, safe, and may expedite the diagnostic and staging process of these patients.
**Limitations:** This study was unicentric and retrospective.
**Ethics committee approval:** No information provided by the submitter.
**Funding for this study:** No funding was received for this study.
**Author Disclosures:**
Elisa Antolinos-Macho: Nothing to disclose
Miguel Ángel Gómez Bermejo: Nothing to disclose
Amparo Benito-Berlinches: Nothing to disclose
Sofía Ventura Díaz: Nothing to disclose
Luis Gorospe Sarasua: Nothing to disclose
Alba Salgado Parente: Nothing to disclose
Pilar Garrido-López: Nothing to disclose
Antonio Michael-Fernández: Nothing to disclose
Abel González-Huete: Nothing to disclose

**RPS 1104-11**
**Washout CT can discriminate pulmonary hamartoma from neuroendocrine neoplasms: simplicity in the radiomics-era**
A. Perrella, G. Bagnacci, N. Di Meglio, *V. Di Martino*, L. Luzzi, M. A. Mazzei, L. Volterrani; Siena/IT
*(dimartino7@student.unisi.it)*

**Purpose:** Solitary pulmonary nodules (SPNs) with a round shape represent an everlasting problem for radiologists, especially in the era of CT screening for lung cancer. The challenge gets even harder when malignant nodules show similar characteristics to benign ones. Hamartomas (HAs) and neuroendocrine neoplasms (NENs) share morphological features thus it is often impossible to discriminate between them by imaging. Anyway, making this differential diagnosis is crucial since HAs are invariably benign, whereas NENs have to be considered malignant and therefore need to be evaluated for surgical excision. The purpose of this study was to develop a simple method to discriminate pulmonary "fat-poor" HAs and NENs at contrast-enhanced CT (CECT).
**Methods or Backround:** Between September 2015 and December 2021 a total of 95 patients with a histologically proven diagnosis of lung NENs (74) and HAs (21) and for which a preoperative CECT scan has been performed, were identified through a review of our pathological and radiological databases. Among these, 55 cases (18 HAs and 37 NENs), studied with biphasic CECT, were ultimately selected and reviewed by three radiologists of differing experience levels, analysing their morphological features and characteristics of enhancement. The enhancement analysis was performed by placing an ROI within the lesion in non-contrast (NCp), post-contrast (PCp, 55-65seconds after intravenous contrast injection), and delayed phases (Dp, 180-300 seconds). 35 patients who underwent 18 FDG-PET/CT was evaluated in a secondary analysis. A Mann-Whitney test was used to analyse mean differences.
**Results or Findings:** HU values were significantly different between NENs and HAs in the PCp (p<0,001). In NCp and Dp attenuation values did not show significant differences in the two groups. Differences in values of HUs in PCp and Dp allowed us to discriminate between NENs and HAs.
**Conclusion:** Wash-out analysis, ΔHU (PCp-Dp), can perfectly discriminate pulmonary "fat-poor" hamartomas from neuroendocrine neoplasms.
**Limitations:** The retrospective nature of the study was identified as a limitation.
**Ethics committee approval:** No information provided by the submitter.
**Funding for this study:** No funding was received for this study.
**Author Disclosures:**
Maria Antonietta Mazzei: Nothing to disclose
Vito Di Martino: Nothing to disclose
Luca Luzzi: Nothing to disclose
Armando Perrella: Nothing to disclose
Nunzia Di Meglio: Nothing to disclose
Luca Volterrani: Nothing to disclose
Giulio Bagnacci: Nothing to disclose

**16:00-17:30** **Research Stage 3**

### Research Presentation Session: Interventional Radiology

### RPS 1109

### Interventional oncology (part 1)

**Moderator**
F. Wolf; Vienna/AT
*(florian.wolf@meduniwien.ac.at)*

**RPS 1109-2**
**Liver venous deprivation for volumetric and functional hypertrophy in a single centre: evaluation of outcomes and comparison of transhepatic and transjugular approaches for hepatic vein embolisation**
*D. Santangelo*, A. Della Corte, F. Ratti, C. Canevari, F. Cipriani, S. Gusmini, L. Aldrighetti, F. De Cobelli; Milan/IT
*(santangelo.domenico@hsr.it)*

**Purpose:** Liver venous deprivation (LVD), i.e. simultaneous portal and hepatic vein embolisation, has shown great potential for rapid liver hypertrophy in view of major hepatectomy; both transhepatic (TH) and transjugular (TJ) approaches for hepatic vein embolisation have been described, but no comparative data are available. The aim of this study is to evaluate safety and efficacy of LVD in a single centre, as well as to compare TH and TJ approaches.
**Methods or Backround:** Twenty-seven patients (June 2019-September 2022) who underwent LVD for future liver remnant (FLR) hypertrophy were retrospectively analysed (n=16 TH, n=11 TJ). Patients underwent pre- and post-procedural CT-scan and mTc-mebrofenin hepatobiliary scintigraphy to calculate volumetric/functional degree of hypertrophy (vDH, fDH).
**Results or Findings:** Pre-procedural FLR (FLR) was 27.74±7.84cc. Baseline FLR function was 2.14±0.68%/min/m2. Fourteen patients (51.85%) had undergone chemotherapy before the procedure. No significant differences in volume, function, and clinical characteristics were observed among TH and TJ groups at baseline analysis. Post-procedural complications occurred in n=2 patients (7.4%), developing left portal branch thrombosis. No cases of permanent off-target embolisation occurred. DH was 43.33±26.95% at 5-15 days and 53.3±32.74% at 25-35 days. fDH was 54.85±28.4%. Nineteen (70.4%) patients underwent scheduled surgery. Drop-outs were due to insufficient hypertrophy (n=1, 3.7%), disease progression (n=6, 22.2%), and inadequate performance status (n=1, 3.7%). Time between LVD and surgery was 41.06±23.18 days. DH and post-procedural complications did not differ in TH and TJ groups (p=0.247 and 0.658). No cases of post-hepatectomy liver failure were observed.
**Conclusion:** LVD is a safe and effective technique that may allow earlier surgery expanding the possibilities of radical oncological treatments. TH and TJ approach for hepatic vein embolisation seem equally safe and effective.
**Limitations:** Limited sample size.
**Ethics committee approval:** LEATUM - 64/INT/2021
**Funding for this study:** No funding was received for this study.
**Author Disclosures:**
Angelo Della Corte: Nothing to disclose
Luca Aldrighetti: Nothing to disclose
Simone Gusmini: Nothing to disclose
Federica Cipriani: Nothing to disclose
Francesco De Cobelli: Nothing to disclose
Carla Canevari: Nothing to disclose
Francesca Ratti: Nothing to disclose
Domenico Santangelo: Nothing to disclose

**RPS 1109-3**
**The role of MRI-based radiomics in prediction of local tumour progression after microwave ablation of colorectal liver metastases**
A. Della Corte, M. Mori, *F. Calabrese*, A. Pellegrini, D. Santangelo, A. del Vecchio, C. Fiorino, L. Aldrighetti, F. De Cobelli; Milan/IT

**Purpose:** The aim of this study is to evaluate feasibility and predictive performance of MRI radiomic features (RF) analysis for prediction of local tumour progression-free survival (LTPFS) after microwave ablation (MWA) of colorectal liver metastases (CLM).
**Methods or Backround:** Patients with oligometastatic colorectal cancer who underwent MWA for CLM (September 2015-January 2021) with a preprocedural Gadoxetic acid MRI were analysed. Preoperative clinical and radiological data were retrospectively recovered. CLMs were manually segmented on T2 and T1-Hepatobiliary phase (T1-HBP) MRI sequences by two observers to test interobserver variability with Intra-Class Correlation Coefficients (ICC). RF were extracted from both contours according to IBSI (Image Biomarker Standardisation Initiative) guidelines using the SPAARC software. Out of 182 RF, 148 resulted robust on T1-HBP and 141 on T2 (ICC>0.7). Cox multivariate analysis was performed to obtain clinical, radiomic and mixed predictive models of LTPFS.
**Results or Findings:** Fifty-four patients (76 CLM) were evaluated. Median follow-up was 14 months. LTP was observed in 19/76 CLM (25%). A clinical model including minimal ablation margins, intra-segment recurrence and tumour grade was able to predict LTPFS with moderately high discrimination (AUC=0.89, p=0.0001). Pure radiomic models also predicted LTPFS (T1-HBP: AUC=0.83, p=0.0003; T2: AUC=0.79, p=0.001). Mixed clinical-radiomic models resulted in the best performances: T1-HBP-based model included minimal ablation margins, tumour regression grade and 10th percentile of signal intensity (AUC=0.98, p=0.0001); T2-based model included minimal ablation margins, tumour regression grade and tumour flatness (AUC=0.95, p=0.0003).
**Conclusion:** MRI-based radiomic analysis of CLM is a feasible technique, with a prognostic value for recurrence after ablation. Mixed clinical-radiomic models for prediction of LTPFS performed better than pure clinical or radiomic models.
**Limitations:** Retrospective study; limited sample size.
**Ethics committee approval:** 59/INT/2017
**Funding for this study:** No funding was received for this study.
**Author Disclosures:**
Angelo Della Corte: Nothing to disclose
Claudio Fiorino: Nothing to disclose
Luca Aldrighetti: Nothing to disclose
Antonella del Vecchio: Nothing to disclose
Alessandro Pellegrini: Nothing to disclose
Francesco De Cobelli: Nothing to disclose
Martina Mori: Nothing to disclose
Francesca Calabrese: Nothing to disclose
Domenico Santangelo: Nothing to disclose

**RPS 1109-4**

**Comparison between Liver Venous Deprivation (LVD) and mini-invasive ALPPS for functional and volumetric growth of the future liver remnant**

A. Della Corte, *D. Santangelo*, F. Ratti, C. Canevari, F. Cipriani, S. Gusmini, L. Aldrighetti, F. De Cobelli; Milan/IT
*(santangelo.domenico@hsr.it)*

**Purpose:** To compare safety and efficacy of liver venous deprivation (LVD) and mini-invasive ALPPS.
**Methods or Backround:** Fifteen patients (June 2017-February 2022) who underwent LVD (simultaneous portal and hepatic vein embolisation) or mini-invasive ALPPS (laparoscopic partial liver transection followed by portal vein embolisation) for future liver remnant (FLR) hypertrophy were retrospectively analysed. All patients underwent pre- and post-procedural evaluation of FLR volume and function by post-processing of CT images and mTc mebrofenin hepatobiliary scintigraphy to allow calculation of kinetic growth rate (KGR).
**Results or Findings:** Eight patients underwent LVD, 7 patients underwent mini-invasive ALPPS. Higher rates of patients with Klatskin tumour were observed in LVD group (4/8, 50% vs 0/7, 0%); higher rates of cirrhotic patients in ALPPS group (3/7, 42.9% vs 0/8, 0%). Baseline FLR volume (28.8±7.6%) and function (1.9±0.6%/min/m2) were homogeneous (p=0.4 and 0.3, respectively). Length of stay was comparable (p=0.14), without post-procedural complications. Functional and volumetric KGR did not significantly differ (p=0.9). Surgery was completed in 12/15 patients (80%), of which 6/7 (85.7%) in ALPPS group and 6/8 (75%) in LVD group. Drop-out from surgery was due to insufficient growth (n=1, ALPPS) and extrahepatic progression (n=2, LVD). Time to surgery (58.6±57 days) was homogeneous (p=0.52). No significant differences were observed in term of intraoperative blood loss (p=0.8) and length of stay (p=0.34). No cases of post-hepatectomy liver failure were observed.
**Conclusion:** LVD and mini-invasive ALPPS are both safe and effective techniques, without significant differences in ability to promote volumetric and functional growth of the FLR in this exploratory cohort.
**Limitations:** Limited sample size.
**Ethics committee approval:** LEATUM - 64/INT/2021
**Funding for this study:** No funding was received for this study.
**Author Disclosures:**
Angelo Della Corte: Nothing to disclose
Luca Aldrighetti: Nothing to disclose
Simone Gusmini: Nothing to disclose
Federica Cipriani: Nothing to disclose
Francesco De Cobelli: Nothing to disclose
Carla Canevari: Nothing to disclose
Francesca Ratti: Nothing to disclose
Domenico Santangelo: Nothing to disclose

**RPS 1109-5**

**Radiomic features extraction before colorectal liver metastases ablation: tumour borders matter**

*F. Menchini*, M. Calandri, C. Gazzera, S. Tibaldi, G. Risi, V. Cignini, P. Fonio, V. Giannini; Turin/IT

**Purpose:** To investigate the potential role of radiomic features in predicting the local tumour progression (LTP) after thermal ablations of colon-rectal liver metastases (CRLM), focusing on the relationship between the ablated zone (AZ) and the surrounding parenchyma (border) and their prognostic role.
**Methods or Backround:** This retrospective multicentre study enrolled 38 patients that undergone to thermal ablations of CRCM performed by radiofrequencies or microwaves devices. The hepatic metastases were segmented by ITKsnap software (version 4.7) on the venous-portal phase of the abdomen CT-scan (acquired no longer than 1 month prior the procedure). A volume of surrounding liver parenchyma, with a width of 1 cm outward and 3 mm inward the lesion, was automatically generated: we defined this as "border".
**Results or Findings:** 71 radiomics features were extrapolated by an in-house image biomarkers software. We studied the features' distribution in the "lesions" and "borders" groups, comparing the lesions with LTP to the ones with no LTP. In the "borders" group 2 features reached statical significance (Mann-Whitney U test and Kruskal-Wallis test, p value <0.05) and 13 ones in the border group with an ablation margin >5mm.
**Conclusion:** Two different patterns of radiomic features expression were identified in the LTP/no LTP groups, in the "border" area. These findings underline the potentiality of surrounding liver parenchyma features as predictors of outcome after thermal ablations of CRLM.
**Limitations:** Limited sample of patients (38 patients, 57 lesions). Interoperator variability in the lesion's manual segmentation. External validation data are not available (ongoing).
**Ethics committee approval:** No information provided by the submitter.
**Funding for this study:** No fundings was received for this work.
**Author Disclosures:**
Valentina Giannini: Nothing to disclose
Marco Calandri: Nothing to disclose
Valentina Cignini: Nothing to disclose
Carlo Gazzera: Nothing to disclose
Gaetano Risi: Nothing to disclose
Paolo Fonio: Nothing to disclose
Francesca Menchini: Nothing to disclose
Stefano Tibaldi: Nothing to disclose

**RPS 1109-6**

**Comparison of Gd-EOB-DTPA-enhanced MRI and 99mTc-mebrofenin hepatobiliary scintigraphy to measure regional hepatic function after hypertrophy induction by radioembolisation**

M. P. Fabritius[1], O. Öcal[1], B. Garlipp[2], *F. Grawe*[1], M. Pech[3], M. Ingenerf[1], M. Seidensticker[1], S. Deniz[1], M. Ümütlü[1]; [1]Munich/DE, [2]Berlin/DE, [3]Magdeburg/DE

**Purpose:** To compare Gd-EOB-DTPA-enhanced MRI and 99mTc-labelled mebrofenin hepatobiliary scintigraphy (HBS) as imaging-based liver function tests after unilateral radioembolisation (RE) in patients with primary or secondary liver malignancies.
**Methods or Backround:** Twenty-three patients with primary or secondary liver malignancies who underwent Gd-EOB-DTPA-enhanced MRI within a prospective study (REVoluTion) were evaluated. REVoluTion was a prospective open-label, non-randomised, therapy-optimising study of patients undergoing right-sided or sequential RE for contralateral liver hypertrophy at a single centre in Germany. MRI and hepatobiliary scintigraphy were performed before radioembolisation (baseline) and 6 weeks after (follow-up). This exploratory subanalysis compared liver enhancement on hepatobiliary phase normalised to the spleen (liver to spleen ratio, LSR) and the muscle (liver to muscle ration, LMR) on MRI with mebrofenin uptake on HBS for the total liver (TL) and separately for the right (RLL) and left liver lobe (LLL).
**Results or Findings:** Mebrofenin uptake at baseline and follow up each correlated significantly with LSR and LMR for TL (LSR: baseline: r2=0.635, p<0.001; follow-up: r2=0.462, p=0.013. LMR: r2=0.473, p=0.011; r2=0.679, p<0.001) and RLL (LSR: r2=0.597, p=0.001; r2=0.483, p=0.010. LMR: r2=0.353, p=0.049; r2=0.644, p<0.001). Regarding the LLL, mebrofenin uptake correlated significantly with LMR (r2=0.464, p=0.013; r2=0.540, p=0.004), whereas with LSR a significant correlation was only seen at follow-up (r2=0.349, p=0.051; r2=0.359, p=0.046).
**Conclusion:** LSR and LMR correlate with mebrofenin uptake in HBS. This study indicates that Gd-EOB-DTPA-enhanced MRI and 99mTc-labelled mebrofenin HBS may equally be used to assess increase in contralateral liver lobe function after unilateral radioembolisation.
**Limitations:** Small study.
**Ethics committee approval:** No information provided by the submitter.
**Funding for this study:** No funding was received for this study.
**Author Disclosures:**
Matthias Philipp Fabritius: Nothing to disclose
Max Seidensticker: Nothing to disclose
Sinan Deniz: Nothing to disclose
Freba Grawe: Nothing to disclose
Maciej Pech: Nothing to disclose
Maria Ingenerf: Nothing to disclose
Muzaffer Ümütlü: Nothing to disclose
Benjamin Garlipp: Nothing to disclose
Osman Öcal: Nothing to disclose

**RPS 1109-7**

**Evaluation of prognostic scores using CT/MRI morphological parameters in HCC patients undergoing TARE**

*A. B. Beeskow*, A. Schindler, S. Ebel, T. Denecke, D. Seehofer, K. Steinhoff, O. Sabri, T. Berg, F. van Boemmel; Leipzig/DE
*(anne.paets@medizin.uni-leipzig.de)*

**Purpose:** To evaluate existing scoring systems using morphological parameters in patients with hepatocellular carcinoma (HCC) undergoing transarterial radioembolisation (TARE) regarding to survival and therapy response.
**Methods or Backround:** CT and MRI scans of 161 patients with HCC prior to 90Y-radioembolisation (TARE) were evaluated retrospectively in terms of macrovascular invasion, portal vein thrombosis, multifocality, and tumour volume, using the prognostic scoring systems MELD, Child-Pugh, BCLC, Okuda, CLIP, HAP and ALBI score, which have been evaluated in patients treated with TARE. ROC analysis was performed regarding to survival after 3 and 6 months as well as tumour response after 3 months (mRECIST).
**Results or Findings:** Scores were evaluated using image aspects (tumour size/volume, presence of portal vein thrombosis, multifocality, and ascites) in CT and MRI. In addition, the paraclinical parameters bilirubin and AFP were analysed. Preliminary results of ROC analysis showed best prediction

Thursday concerning 3- and 6-months survival by CLIP score (AUC=0.69 and AUC=0.69) and tumour response after 3 months by ALBI score (AUC=0.69).
**Conclusion:** Analysis showed a moderate prediction between the evaluated scores and 3- and 6-months survival and tumour response after 3 months. Thus, it might be necessary to develope new scores for patient selection prior to TARE considering interventional aspects and dosimetry.
**Limitations:** Limitations are the retrospective design and the small sample size. Furthermore, dosimetry was not considered.
**Ethics committee approval:** No information provided by the submitter.
**Funding for this study:** No funding was received for this study.
**Author Disclosures:**
Aaron Schindler: Nothing to disclose
Timm Denecke: Nothing to disclose
Florian van Boemmel: Nothing to disclose
Anne Bettina Beeskow: Nothing to disclose
Sebastian Ebel: Nothing to disclose
Osama Sabri: Nothing to disclose
Karen Steinhoff: Nothing to disclose
Daniel Seehofer: Nothing to disclose
Thomas Berg: Nothing to disclose

### RPS 1109-8

**The role of shear wave elastograph in predicting microwave ablation treatment efficacy in parathyroid adenomas**

*F. E. Urfali*, B. Aydin, G. B. Cansu, M. Korkmaz; Kütahya/TR
*(furkan.e.u@hotmail.com)*

**Purpose:** We aimed to evaluate the role of shear wave elastography (SWE) in predicting the duration and effectiveness of microwave ablation (MWA) therapy in parathyroid adenomas.
**Methods or Backround:** Between March 2021-October 2022, a total of 33 patients underwent US-guided MWA. Before the procedure, blood parameters (PTH, Ca), US findings of adenom, SWE (m/s) values were obtained from the archives. The generator mean watt and procedure time were recorded. Blood parameters and US were evaluated in the clinical controls of the patients in the first month after the procedure.
**Results or Findings:** A very high (r: 0.936, p<0.001) positive correlation was found between procedure time and adenoma size, and a moderate positive correlation (r: 0.574, p<0.001) with adenoma SWE values. A high positive correlation(r: 0.783, p<0.001) was found between mean generator watt between adenoma SWE values. There was no significant difference adenoma SWE values (r: 0.263, p>0.05) and adenoma size (r: 0.348, p>0.05) between PTH levels after the procedure. A moderate negative correlation (r: 578, p<0.001) was found between adenoma SWE values and adenoma volume reduction in the first month controls. Transient vocal cord paralysis occurred in 1 patient, and short-term local pain occurred in 4 patients after the procedure. Our procedure success was 97% for the first session, and because PTH levels did not decrease in one of our patients, the second session was performed.
**Conclusion:** We found higher mean watts during the procedure in stiff nodules and limited volume reduction at first month US controls. This may be due to the carbonisation effect of high temperature values. Hydrodissection with colloidal fluids will be appropriate as the processing time will be longer in stiff and large nodules. There was no significant relationship between adenoma stiffness and procedural success and postoperative PTH values.
**Limitations:** Long-term follow-up results of microwave ablation of parathyroid adenoma are unknown.
**Ethics committee approval:** This study was approved by the institutional review board at our hospital. Desicion number: e-41997688-050.99-65247.
**Funding for this study:** No funding was received for this study.
**Author Disclosures:**
Güven Barış Cansu: Nothing to disclose
Mehmet Korkmaz: Nothing to disclose
Furkan Ertürk Urfali: Nothing to disclose
Bünyamin Aydin: Nothing to disclose

### RPS 1109-9

**Clinical application of transcatheter arterial catheterisation dual cone-beam CT (CBCT) in the diagnosis and treatment of hepatocellular carcinoma (HCC)**

*W. Guan*[1], W. Wang[2], L. Zheng[1]; [1]Henan Province/CN, [2]Beijing/CN
*(15039482@qq.com)*

**Purpose:** To explore the clinical application value of dual cone-beam CT (CBCT) and MRI in the early diagnosis of hepatocellular carcinoma (HCC).
**Methods or Backround:** From March to June 2022, a total of 68 patients admitted to Henan Cancer Hospital with liver mass lesions on MRI images and confirmed by the pathologically were included in this study. Preoperative MRI scanning and real-time dual CBCT scanning at the beginning of transcatheter arterial chemoembolisation (TACE) treatment were performed in all patients. Using lipiodol-CT within one week after TACE treatment as the gold standard for diagnosis of HCC, accuracy, sensitively, specificity and area under the curve were used to assess the performance and clinical application value of dual CBCT images and MRI images in intrahepatic lesions.
**Results or Findings:** A total of sixty-eight patients were enrolled, including 61 males and 7 females, ranging in age from 34 to 82 years old, with an average age of 58 years old. Taking the lipiodol CT results as the "gold standard", a total of 302 intrahepatic lesions were detected in 68 patients with HCC. The sensitivity of lesions detected by preoperative MRI images was 80.46%, the accuracy and the precision were 100%. The sensitivity of lesions detected by dual CBCT images was 100%, the accuracy was 100%, the accuracy and the precision were 100%. Sensitivity and area under the curve (AUC) of lesions detected by dual CBCT images were superior to those by MRI images.
**Conclusion:** Compared with MRI images, dual CBCT by transcatheter arterial catheterisation showed good reliability and clinical application value to detect intrahepatic lesions in the early diagnosis and detection rate of HCC and could assist surgeons to achieve the goal of precise TACE treatment.
**Limitations:** Not applicable.
**Ethics committee approval:** Not applicable
**Funding for this study:** No funding was received for this study.
**Author Disclosures:**
Weiwei Guan: Nothing to disclose
Lin Zheng: Nothing to disclose
Wenxin Wang: Nothing to disclose

### RPS 1109-10

**Obscure hepatocellular adenomas not candidate for surgery: percutaneous thermal ablation after tagging with ethiodized oil**

*A. Inzerillo*[1], C. Allimant[2], V. Schembri[2], B. Guiu[2]; [1]Palermo/IT, [2]Montpellier/FR
*(inzerilloagostino@gmail.com)*

**Purpose:** Percutaneous thermal ablation (PTA) for hepatocellular adenoma (HCA) has been increasingly used in recent decades due to its lower rate of complications and morbidity. Certain HCAs are poorly visualised on ultrasound (US) or unenhanced computed tomography (CT). To overcome these problems, we describe a technique that allows persistent tagging of the tumour with intra-arterial injection of ethiolised oil to enhance visibility of adenoma, increasing the rates of successfully treated tumours.
**Methods or Backround:** This retrospective study included all patients who underwent PTA for HCA at our institution from November 2014 to April 2022. Inclusion criterion was PTA treatment using preprocedural intra-arterial Lipiodol injection for tumour tagging in poorly visible adenomas for US or non-contrast CT. We included 7 patients (5 women and 2 men; mean age 44 years) with 9 HCAs.
**Results or Findings:** Lipiodol retention by the lesion was always achieved. All the nine lesions (100 %) were completely covered by the ablation zone with ablative margin at the first PTA session without complications. There were no instances of local tumour progression or haemorrhage over a mean imaging follow-up period of 21 months.
**Conclusion:** PTA after intra-arterial iodided-oil injection is a useful, effective, and feasible therapeutic option for the treatment of challenging HCAs that are invisible on US images or unenhanced CT. This well-known technique for the treatment of HCC, which we describe for the first time for challenging HCA, may be an additional weapon in the arsenal of the multidisciplinary liver team. We suggest PTA for HCAs that are less than 3 cm in size, after a multidisciplinary discussion in a specialised liver cancer centre.
**Limitations:** Major limitation of this study includes the small sample size.
**Ethics committee approval:** No information provided by the submitter.
**Funding for this study:** No funding for this study.
**Author Disclosures:**
Agostino Inzerillo: Nothing to disclose
Boris Guiu: Nothing to disclose
Valentina Schembri: Nothing to disclose
Carole Allimant: Nothing to disclose

### RPS 1109-11

**Perfusion CT for early prediction of treatment response of cryoablation in renal cell carcinoma**

*O. Lamid-Ochir*[1], M. Miyazaki[2], T. Nakajima[3], H. Tokue[3], A. Tumenjargal[1]; [1]Ulaanbaatar/MN, [2]Kawagoe/JP, [3]Maebashi/JP
*(oyunbold132@yahoo.com)*

**Purpose:** To evaluate the quantitative analysis of perfusion CT (pCT) for assessment of early treatment response after cryoablation in renal tumours.
**Methods or Backround:** A total of 54 patients with renal tumours who had treated by CT-guided percutaneous cryoablation in our institution from June 2014 to August 2016. Twenty-seven patients were selected, who underwent pCT before and after within 1 week, 1, 3, 6 months, and 1-year treatment. Perfusion parameters including arterial flow perfusion (AFP) as tumour maximum, minimum, average, and average changes, and tumour diameter, freezing time, treatment cycles, and progression-free-survival were analysed and compared. The effects of treatment response were evaluated by RECIST standards version 1.1 as a non-responder and responder.

**Results or Findings:** Twenty-seven patients (22 men, 5 women: age, 68 ± 12 y.o.) were evaluated as quantitative analysis with pCT. Five of the 27 patients were non-responders, and 22 patients were responders. The mean AFP values were significantly different between non-responder (46.6±25.8) and responder (25.1 ± 6.1) at 1 month after cryoablation ($p<0.05$). ROC analysis demonstrated that tumour average (AFP) at 1 month had the cut-off of perfusion value (29.95) and area under curve (0.94), with sensitivity and specificity of 100% and 84.2%, respectively ($p<0.05$). Percent of progression free-survival was 100% (cut-off < 29.95) and 66.7% (cut-off >29.95) for 32 months observation.
**Conclusion:** pCT was able to evaluate and predict the therapeutic effects of cryoablation at 1 month after treatment. It can offer both morphologic and functional evaluation, providing a quantitative assessment of residual tumour vascularisation after treatment.
**Limitations:** It was a retrospective study with a small number of patients.
**Ethics committee approval:** No information provided by the submitter.
**Funding for this study:** No funding was received for this study.
**Author Disclosures:**
Takahito Nakajima: Nothing to disclose
Masaya Miyazaki: Nothing to disclose
Hiroyuki Tokue: Nothing to disclose
Oyunbold Lamid-Ochir: Nothing to disclose
Amartuvshin Tumenjargal: Nothing to disclose

### RPS 1109-12

**Radioembolisation in liver metastases of neuroendocrine tumours (NELM): prognostic factors for overall survival and (hepatic) progression-free survival – anything new?**

*M. Ingenerf*, F. Grawe, J. Rübenthaler, M. P. Fabritius, L. Beyer, J. Ricke, C. Auernhammer, M. Seidensticker, C. Schmid-Tannwald; Munich/DE

**Purpose:** To evaluate prognostic clinical and imaging parameters in patients with NELM for better outcome after transarterial radioembolisation (TARE).
**Methods or Backround:** 47 patients with NELM who underwent TARE and preinterventional liver MRI including diffusion weighted imaging (DWI) and 68Ga-DOTATATE PET/CT were included. Apparent diffusion coefficient (ADC) and standardised uptake value (SUV) of three liver metastases, and of tumour-free spleen and liver were measured. Tumour-to-spleen (T/S) and tumour-to-liver ratio (T/L) ratios (with possible combinations of SUVmax/SUVmax, SUVmax/SUVmean, and SUVmean/SUVmean) were calculated. Clinical parameters including hepatic tumour-burden, extrahepatic metastases, prior therapies, Chromogranin A (CgA), Ki-67 and bilirubin levels were assessed. Outcome parameters were OS, PFS and HPFS.
**Results or Findings:** Median OS, HPFS and PFS was 49.6, 28.3 and 13.1 months (m) respectively. Low Ki-67 (≤5%), low hepatic tumour burden (<10%), absence of extrahepatic metastases and increased Tmean/Lmax ratio were significant prognostic factors of both, longer OS and HPFS in multivariable cox regression. In addition, low baseline CgA was significantly associated with longer HPFS. Tmean/Lmax > 1.9 yielded a median OS of 69 vs. 33 m ($p<0.04$), and a median HPFS of 30 vs. 19m ($p=0.09$). Higher baseline SUVmax was the only variable significant for longer PFS in the multivariable model. SUVmax > 28 resulted in a median PFS of 16.9 m (vs. 6.5 m, $p=0.001$).
**Conclusion:** High preinterventional Tmean/Lmax ratios, and high SUVmax seem to have prognostic value in patients with NELM undergoing TARE and could play a supplemental role in patient selection and management besides conventional variables such as tumour burden.
**Limitations:** The informative value is limited due to the retrospective design and the small study population due to the rarity of NET.
**Ethics committee approval:** Local ethics committee of the university hospital LMU Munich
**Funding for this study:** No funding was received for this study.
**Author Disclosures:**
Matthias Philipp Fabritius: Nothing to disclose
Max Seidensticker: Nothing to disclose
Freba Grawe: Nothing to disclose
Maria Ingenerf: Nothing to disclose
Johannes Rübenthaler: Nothing to disclose
Christine Schmid-Tannwald: Nothing to disclose
Christoph Auernhammer: Nothing to disclose
Leonie Beyer: Nothing to disclose
Jens Ricke: Nothing to disclose

### RPS 1109-13

**Interventional oncological treatment of breast cancer liver metastases (BCLM): monocentric long-term evaluation on thermal ablation and local chemotherapy**

*T. J. Vogl*, J. Freichel, T. Gruber-Rouh, N-E. A. N-E. Mohammed; Frankfurt/DE
*(t.vogl@em.uni-frankfurt.de)*

**Purpose:** To retrospectively evaluate the development and technological progress in local oncological treatments of breast cancer liver metastasis (BCLM) using thermoablative techniques like laser-induced thermal therapy (LITT), microwave ablation (MWA) and transarterial chemoembolisation (TACE) in a multimodal application.
**Methods or Backround:** This monocentric study includes 1,136 patients (range, 28-87y) and is based on data generated over more than 26 years (from 1993 to 2020). Patients were divided into 5 different groups: LITT+TACE included 215 patients, LITT alone 212 patients, MWA alone 17 patients, MWA+TACE 143 patients, and TACE alone 549 patients. Therapy results were evaluated using survival rates of Kaplan-Meier estimator, Cox proportional hazard regression and log-rank test.
**Results or Findings:** Median survival for the LITT group is 2.2 years, for LITT+TACE 2.1 years. For LITT alone treatments 1-/3-/5-year survival rates were 80%, 37%, 22%, for combined LITT+TACE treatments 76%, 34% and 15%. Median survival in the MWA group was 5.6 years, for MWA+TACE 2.4 years. In the MWA group the 1-/3-/5-year survival rates were 89%, 89%, 89%. Combined MWA+TACE showed values of 77%, 38% and 22%. TACE as monotherapy had a median survival of 0.8 years. 1-/3-/5-year survival rates were 37%, 8% and 4%. Cox regression analysis showed that the different treatment methods are statistically significant predictors for patient survival.
**Conclusion:** Treatments with MWA and combined MWA+TACE resulted in best median survival rates. Patients treated with MWA or LITT or combined with TACE showed significantly higher survival times vs. TACE as monotherapy.
**Limitations:** No limitations.
**Ethics committee approval:** No information provided by the submitter.
**Funding for this study:** No funding was received for this study.
**Author Disclosures:**
Thomas J. Vogl: Nothing to disclose
Tatjana Gruber-Rouh: Nothing to disclose
Nour-Eldin Abdelrehim Nour-Eldin Mohammed: Nothing to disclose
Jason Freichel: Nothing to disclose

**16:00-17:30** **Research Stage 4**

### Research Presentation Session: Cardiac

### RPS 1103

### Cardiovascular CT: from morphology to function

**Moderator**
R. Vliegenthart; Groningen/NL
*(r.vliegenthart@umcg.nl)*
**Author Disclosures:**
Rozemarijn Vliegenthart: Research Grant/Support: Institutional research grants, Siemens Healthineers; Speaker: Bayer, Siemens Healthineers

### RPS 1103-2

**Coronary artery calcium density and volume are influenced by lesion distribution and image reconstruction parameters**

*B. Foldyna*[1], S. Basmagi[2], F. Aghapour Zangeneh[3], K. Doktorov[2], A. Matveeva[2], T. Denecke[4], R. Gohmann[4], M. Gutberlet[4], L. Lehmkuhl[2]; [1]Boston, MA/US, [2]Bad Neustadt/DE, [3]Mannheim/DE, [4]Leipzig/DE
*(BFOLDYNA@mgh.harvard.edu)*

**Purpose:** Computed tomography (CT)-derived coronary artery calcium (CAC) density predicts cardiovascular events independent of CAC volume. However, whether CAC distribution (i.e., number of calcified lesions relative to CAC volume) and CT image reconstruction parameters influence CAC density is unknown. Therefore, the primary objective of this study was to learn how CAC distribution, slice thickness, and increment influence CAC density.
**Methods or Backround:** One-hundred-twenty patients received clinically indicated non-contrast ECG-gated cardiac CT using a study-specific prospectively defined CAC scoring protocol with 1-, 3-, and 5-mm thick image reconstructions, with/without 20% increment. We related CAC density with volume in patients with CAC>0, adjusting for the number of calcified lesions and clinical parameters associated with CAC density in univariable analysis.

Thursday

We further analysed the association between image reconstruction parameters and CAC density, volume, and the number of calcified lesions.
**Results or Findings:** Overall, 75/120 (63%) patients (66% men, 63±11 years) presented with 244 calcified lesions. CAC density strongly correlated with CAC volume (rho=0.62; p<0.001) and moderately with number of lesions (rho=0.32; p=0.005). However, CAC density decreased with increasing lesion number at a given CAC volume (beta coefficient: -58.9; 95%CI: -84.7–-33.1; p<0.001), adjusted for age, body-mass-index, smoking, and hypercholesterolaemia. Greater slice thickness was related to fewer detected lesions (327, 244, and 204), lower CAC density (276.8 (243.3–330.5), 227.6 (197.0–268.6), and 193.5 (166.7–232.1) HU), and lower CAC volume (50.9 (25.5–121.5), 43.3 (18.9–105.5), and 31.4 (13.7–97.0) mm3) at 1-, 3-, and 5-mm images, respectively. Image increment did not significantly affect CAC measures.
**Conclusion:** CAC distribution and slice thickness strongly influence CAC density and volume, possibly confounding their prognostic value and adjusting for lesion distribution and CT reconstruction parameters if necessary.
**Limitations:** The cross-sectional study design was identified as a limitation.
**Ethics committee approval:** This study was approved by the institutional review board.
**Funding for this study:** Not applicable
**Author Disclosures:**
Lukas Lehmkuhl: Nothing to disclose
Robin Gohmann: Nothing to disclose
Timm Denecke: Nothing to disclose
Said Basmagi: Nothing to disclose
Foroud Aghapour Zangeneh: Nothing to disclose
Matthias Gutberlet: Nothing to disclose
Kalin Doktorov: Nothing to disclose
Borek Foldyna: Research/Grant Support: Unrelated research support through institution from AstraZeneca, MedTrace, Eli Lilly
Anna Matveeva: Nothing to disclose

### RPS 1103-3

**Impact of CT reconstruction algorithms on coronary calcium score**
B. Longere[1], *M. Blain*[2], V. Chevance[2], S. Mule[2], E. Reizine[2], H. Kobeiter[3], A. Luciani[2], V. Tacher[2]; [1]Lille/FR, [2]Paris/FR, [3]Créteil/FR

**Purpose:** Coronary artery calcium score (CAC) is a powerful biomarker of cardiovascular risk. A non-zero CAC is associated with a 5 to 7-fold increase in relative risk of a cardiovascular event. CAC measurement described by Agatston relies on filtered back projection (FBP). Several manufacturers propose new coronary calcium score (CAC) reconstruction including iterative reconstruction. Our goal was to assess the impact of reconstruction algorithms on CAC values and cardiovascular risk stratification.
**Methods or Backround:** This bicentric prospective study included 198 consecutive patients referred for coronary CTA or CAC measurement between January and June 2022. Raw data were reconstructed using four algorithms: 1) FBP (gold-standard), 2) 20% of partial model-based iterative reconstruction (pMBIR20), 3) 70% of pMBIR (pMBIR70), and 4) deep-learning image reconstruction (DLIR). CAC was measured on each reconstruction by two radiologists.
**Results or Findings:** Median CAC was 35 (IQR 0-263) with FBP. Other reconstruction algorithms were responsible for lower CAC values (p<.001). For CAC above 10 the use of different reconstruction algorithms did not modify patients classification. For CAC under 11 (n=79; 41.8%), other reconstructions than FBP significantly impacted categorisation (p<.01). Indeed, pMBIR20, pMBIR70, and DLIR downgraded 5/79 (6.3%), 8/79 (10.1%), and 12/79 (15.2%) patients from CAC 1–10 to zero, respectively. Excellent inter-observer agreement was achieved for CAC measurements for all reconstructions (intraclass correlation coefficient >.99).
**Conclusion:** As compared with FBP, other reconstructions underestimate CAC measurements and therefore nulled lower scores in 6.3% of patients demonstrating CAC 1—10, thus jeopardising the "power of zero".
**Limitations:** Only one CT manufacturer's reconstructions and acquisitions were evaluated.
**Ethics committee approval:** IRB#CRM-2204-288
**Funding for this study:** B.L. is a recipient of the 2021 Alain Rahmouni SFR-CERF research grant provided by the French Society of Radiology together with the French Academic College of Radiology.
**Author Disclosures:**
Vania Tacher: Nothing to disclose
Benjamin Longere: Nothing to disclose
Alain Luciani: Nothing to disclose
Edouard Reizine: Nothing to disclose
Sebastien Mule: Nothing to disclose
Virgile Chevance: Nothing to disclose
Maxime Blain: Nothing to disclose
Hicham Kobeiter: Nothing to disclose

### RPS 1103-4

**Atherosclerotic plaque inflammation in fatal myocardial infarction: non-invasive assessment with computed tomography**
*D. C. Rotzinger*[1], V. Magnin[1], A. van der Wal[2], S. Grabherr[1], S. Qanadli[1], K. Michaud[1]; [1]Lausanne/CH, [2]Amsterdam/NL
*(david.rotzinger@chuv.ch)*

**Purpose:** To evaluate the diagnostic ability of multiphase post-mortem CT angiography (MPMCTA) to detect atherosclerotic plaque enhancement (APE) as a surrogate marker of plaque inflammation.
**Methods or Backround:** The study was conducted in patients with fatal coronary occlusion obtained from autopsies in whom the culprit lesion and the ground truth for plaque inflammation was obtained by histopathological examination. Thirty-five patients were included (12 women, 34%; median [IQR] age, 52 [11] years). Two blinded radiologists assessed CT images in consensus for APE in the culprit lesion based on non-contrast and contrast-enhanced CT. Two forensic pathologists assessed histological samples in consensus and rated inflammatory signs semi-quantitatively. Cases with concurrent vasa vasorum density increase, intraplaque, and periadventitial inflammation were deemed positive for plaque inflammation. Finally, we correlated radiology and pathology findings.
**Results or Findings:** All culprit plaques had histological evidence of plaque erosion or rupture, and we found intraplaque mononuclear cells in 34, periadventitial mononuclear cells in 30, and increased vasa vasorum density in 39 cases. Thirty (85.7%) cases had all plaque inflammation patterns. APE at MPMCT was reported in 21 (60%) cases and resulted in a positive predictive value of 95.2% (77.3–99.2%) and a negative predictive value 28.6% (17–43.9%). Semi-quantitative histological ratings indicated significantly higher levels of intraplaque inflammation (p=0.024) and vasa vasorum density (p=0.032) in plaques with APE. On the other hand, adventitial inflammation was not significantly different in CT negative vs positive plaques (p=0.211).
**Conclusion:** On MPMCTA, contrast enhancement in fatal coronary plaque rupture or erosion was prevalent and provided a high PPV to non-invasively detect plaque inflammation.
**Limitations:** Our study lacks a quantitative analysis, which was challenging to implement due to the plaques' small size.
**Ethics committee approval:** The CER-VD, Canton of Vaud, Switzerland, approved this study.
**Funding for this study:** The authors received no funding.
**Author Disclosures:**
Virginie Magnin: Nothing to disclose
Allard van der Wal: Nothing to disclose
David Christian Rotzinger: Nothing to disclose
Katarzyna Michaud: Nothing to disclose
Silke Grabherr: Nothing to disclose
Salah Qanadli: Nothing to disclose

### RPS 1103-5

**Association of invasive and noninvasive coronary flow with major adverse cardiovascular events: multicentre trial**
*A. J. Vázquez Mézquita*, F. Biavati, M. Dewey; Berlin/DE

**Purpose:** To compare invasive coronary angiography quantitative flow ratio (QFR) with computed tomography fractional flow reserve (CT-FFR) to predict major adverse cardiovascular events (MACE).
**Methods or Backround:** Overall, 90 out of 3561 patients in a multicentre trial of ICA and CT, had MACE until 3.5 years of follow-up (52 in the ICA and 38 in the CT group). Among patients with MACE, complete images could be processed for double-blind analysis of QFR and CT-FFR in 83 of the 90 MACE patients (ICA=46, CT=37) which were statistically matched based on age, gender, and angina type with an equal number of 83 patients without MACE in both randomisation groups.
**Results or Findings:** QFR showed a higher accuracy compared to CT-FFR to predict MACE (AUC=0.76, 95% CI 0.66-0.86 vs AUC=0.68, 95% CI 0.56-0.81, p<0.001). A coronary flow threshold of 0.8 for QFR showed a higher association with MACE than a threshold of 0.75 (HR: 4.1, 95% CI: 2.2 - 7.5 vs HR: 3.4, 95% CI: 1.8 - 6.3, p<0.001). In contrast to CT-FFR, a threshold of 0.75 was more predictive of MACE compared to a threshold of 0.8 (HR, 2.8, 95% CI: 1.6 -4.8 vs HR: 2.0, 95% CI: 1.1- 3.7, p<0.001).
**Conclusion:** QFR showed a better prediction of MACE than CT-FFR until 3.5 years in a contemporary randomised trial.
**Limitations:** This study showed a low number of MACE during the 3.5-year follow-up.
**Ethics committee approval:** The trial was approved and reviewed by the local ethics committee at the coordinating centre, by the German Federal Office for Radiation Protection, as well as other local and national ethics committees.
**Funding for this study:** This work was funded by the German Research Foundation through the graduate program BIOQIC (GRK2260, project-ID: 289347353), the priority program SPP-Radiomics (SPP2177, project-ID: 402688427) and the DISCHARGE project (603266-2, HEALTH-2012.2.4.-2) funded by the FP7 Program of the European Commission.

**Author Disclosures:**
Marc Dewey: Other: Institutional master research agreements
Aldo Javier Vázquez Mézquita: Nothing to disclose
Federico Biavati: Nothing to disclose

### RPS 1103-6

**Optimised window settings for virtual monoenergetic imaging in dual-layer spectral coronary computed tomography angiography**

*L. R. M. Lanzafame*[1], G. Ascenti[1], C. Booz[2], S. Mazziotti[1], A. Blandino[1], T. D'Angelo[1]; [1]Messina/IT, [2]Frankfurt/DE
*(ludovicalanzafame@gmail.com)*

**Purpose:** The aim of this study was to identify the optimal window setting for displaying virtual monoenergetic reconstructions (MonoE) in dual-layer spectral Coronary Computed Tomography Angiography (CCTA).
**Methods or Backround:** Twenty-five patients who underwent a dual-layer spectral Coronary Computed Tomography Angiography were retrospectively evaluated. Conventional, Virtual Monoenergetic Images (MonoE) at 70 and 40 keV datasets were reconstructed. Firstly, contrast-to-noise ratio (CNR) was determined to evaluate objective image quality. Secondly, two blinded observers manually identified the best window setting (B-W/L) for MonoE 70 and MonoE 40 datasets. Subsequently, these values were correlated with aortic attenuation by means of linear regression analysis to obtain optimised W/L setting (O-W/L). Thirdly, subjective image quality (vascular contrast, blooming, stenosis demarcation and overall impression) was assessed using 5-point Likert scale.
**Results or Findings:** MonoE 40 obtained higher CNR values compared both to conventional and MonoE 70 (all p <0.0001). B-W/L were assessed to 1090/240 for MonoE 70 and 3120/760 for MonoE 40. O-W/L resulted in 1070/235 for MonoE 70 and 3065/755 for MonoE 40. MonoE 40 O-W/L also collected the best scores in subjective image quality for each parameter evaluated compared to conventional dataset (all p <0.008).
**Conclusion:** Optimisation of virtual monoenergetic imaging requires adjustments of W/L settings. Our results suggest a W/L setting of 1070/235 for MonoE 70 and 3065/755 for MonoE 40.
**Limitations:** This study has been performed on a relatively small cohort of patients. Additionally, our results are confined to similar acquisition protocols and a single vendor DECT-scanner.
**Ethics committee approval:** No information provided by the submitter.
**Funding for this study:** This study received no funding.
**Author Disclosures:**
Christian Booz: Speaker: Siemens
Alfredo Blandino: Nothing to disclose
Giorgio Ascenti: Speaker: Bracco Speaker: Philips
Silvio Mazziotti: Nothing to disclose
Ludovica Rosa Maria Lanzafame: Nothing to disclose
Tommaso D'Angelo: Speaker: Bracco Speaker: Philips

### RPS 1103-7

**Predicting successful crossing through chronic total occlusion of native coronary lesions within three guidewires**

*A. Zhang*, B. Yang; Shenyang/CN

**Purpose:** The purpose of this study is to select a new endpoint and establish a new scoring system, which includes the qualitative and quantitative characteristics of the lesion obtained from coronary computed tomography angiography (CCTA) to predict the efficiency of chronic total occlusion percutaneous coronary intervention (CTO-PCI).
**Methods or Backround:** This study analysed clinical, morphological, and histological characteristics of 267 CTO lesions in 252 patients (mean age 59.0 [52.0–67.0] years, 80.1% male), which were recruited from a single centre. The primary endpoint is successful crossing of the lesion within three guidewires. The new prediction model is generated by factors determined by multivariate analysis. An appropriate integer score is assigned to each independent predictor, then all scores are summed up and the difficulty level is established.
**Results or Findings:** 72.3% of the lesions reached the endpoint. Independent predictors included blunt proximal stump, proximal and distal branches, occluded segmental curvature >45 °, length ≥ 19.95, and calcified plaque volume ratio ≥ 0.125. As the score increased from 0 to 5, the success rate decreased from 98.83% to 12.50%.
**Conclusion:** The model can accurately predict the probability of successfully passing within three guidewires, and can be used for difficulty classification.
**Limitations:** This is a single-centre retrospective study, and the selection of experimental subjects will be biased. The selected experimental endpoint is not seen before, and the selection and use of guidewire will be somewhat different in different regions and hospitals.
**Ethics committee approval:** Y（2022）119
**Funding for this study:** Funding was received from the Key research and development project of Liaoning Province of China, Grant/Award Number:2020JH2/10300119 as well as the "Selecting the Best Candidates by Opening Competition Mechanism" Science and Technology Program of Liaoning Province of China, Grant/Award Number: 2022JH1/10400004.
**Author Disclosures:**
Anxiaonan Zhang: Nothing to disclose
Bengqiang Yang: Nothing to disclose

### RPS 1103-8

**Diagnostic performance of dynamic regadenoson stress cardiac CT perfusion imaging for the detection of haemodynamically significant coronary artery disease**

C. Urtasun Iriarte, *A. Ezponda Casajus*, M. B. Barrio Piqueras, C. Mbongo, M. Jiménez Vázquez, F. Caballeros Lam, N. Salterain González, J. J. Gavira Gómez, G. Bastarrika Alemañ; Pamplona/ES
*(aezponda@unav.es)*

**Purpose:** The aim of this study was to determine the diagnostic performance of dynamic stress CT myocardial perfusion imaging (CT-MPI) in addition to coronary CT angiography (CCTA) to detect haemodynamically significant coronary artery disease (CAD) compared to invasive coronary angiography (ICA) and fractional flow reserve (FFR).
**Methods or Backround:** Between December 2019 and October 2022, 104 consecutive patients with suspected or known CAD underwent CCTA and dynamic stress CT-MPI using regadenoson as a vasodilator agent. The presence of haemodynamically significant obstruction was determined by CCTA (CAD-RADS ≥3) and CT-MPI (myocardial blow flow relative to remote myocardium < 0.85) compared to CCTA alone, using angiographic severity and an FFR of ≤0.80 as reference.
**Results or Findings:** A total of 104 patients (82 males, mean age 64.4-9.9 years, 16 with prior stent implantation) were evaluated. CCTA and CT-MPI were positive in 47 (45.2%) and 30 (28.8%) patients, respectively. ICA was performed in 31 patients (22 males, 63.8-11.4 years old; 90% with CAD-RADS ≥3). CCTA showed a per-patient sensitivity of 100%; specificity of 50%, negative predictive value (NPV) of 100% and positive predictive value (PPV) of 89.3%. CCTA with CT-MPI increased the specificity (85.7%) and PPV (96.2%). Receiver operating curve (ROC) analysis showed improved discrimination accuracy for CCTA with CT-MPI (0.92; CI: 0.74-1; p=0.002) compared with CCTA alone (0.75; CI: 0.48-1; p=0.061).
**Conclusion:** Dynamic regadenoson stress CT-MPI offers incremental diagnostic value over CCTA alone for the identification of haemodynamically significant CAD.
**Limitations:** No limitations were identified.
**Ethics committee approval:** No information provided by the submitter.
**Funding for this study:** No funding was received for this study.
**Author Disclosures:**
Carmen Mbongo: Nothing to disclose
Cesar Urtasun Iriarte: Nothing to disclose
Miguel Barrio Barrio Piqueras: Nothing to disclose
Nahikari Salterain González: Nothing to disclose
Juan José Gavira Gómez: Nothing to disclose
Fanny Caballeros Lam: Nothing to disclose
Ana Ezponda Casajus: Nothing to disclose
Gorka Bastarrika Alemañ: Nothing to disclose
Marcos Jiménez Vázquez: Nothing to disclose

### RPS 1103-9

**Dynamic myocardial perfusion CT: impact of different reconstruction algorithm**

*A. Senatieri*[1], G. Muscogiuri[1], G. Chierchia[1], P. Marra[2], E. Faietti[2], P. A. Bonaffini[2], S. Sironi[2]; [1]Milan/IT, [2]Bergamo/IT

**Purpose:** To evaluate the impact of different degrees of adaptive statistical iterative reconstruction (ASiR-V) algorithm on the reproducibility of myocardial blood flow (MBF) values in dynamic myocardial perfusion CT (CTP).
**Methods or Backround:** Twenty-two patients underwent dynamic CTP in a 10-month period, which were post-processed using ASiR-V 100%, 80%, 60%, 40%, 20%, 0%. In every patient, MBF values were analysed for every myocardial segment for every different degree of ASiR-V algorithm. Moreover, in ten patients relative MBF has been calculated for every different degree of ASiR-V algorithm. Obtained data underwent statistical analysis with Wilcoxon and Friedman tests. Later, CTP acquisition image quality was evaluated through a four-point Likert score for every different degree of the ASiR-V algorithm. The data obtained underwent statistical analysis using a paired samples t-test. A p value of less than 0,01 was considered significant.
**Results or Findings:** Considering 100% ASiR-V as gold standard for CTP reconstruction, no statistically significant difference has been demonstrated between MBF values obtained analysing images reconstructed using ASiR-V from 0% to 80% (p>0,01) and MBF values obtained using ASiR-V 100%. Relative MBF calculated showed no significant differences between values obtained using an ASiR-V algorithm from 0% to 80% and values obtained using a 100% ASiR-V algorithm. A progressive increase of image quality has been noticed for images reconstructed through 0% to 60% ASiR-V (p<0,01), although no difference has been noticed between 80% and 100% ASiR-V (p>0,01).

Thursday

**Conclusion:** CTP-obtained MBF values are not influenced by different degrees of ASiR-V algorithm used and ASiR-V proved to progressively increase image quality due to reduction of image noise. Moreover, relative MBF is preserved with every degree of ASiR-V algorithm used.
**Limitations:** The small sample size was identified as a limitation.
**Ethics committee approval:** No information provided by the submitter.
**Funding for this study:** No funding was received for this study.
**Author Disclosures:**
Sandro Sironi: Nothing to disclose
Paolo Marra: Nothing to disclose
Pietro Andrea Bonaffini: Nothing to disclose
Gregorio Chierchia: Nothing to disclose
Alberto Senatieri: Nothing to disclose
Elena Faietti: Nothing to disclose
Giuseppe Muscogiuri: Nothing to disclose

### RPS 1103-10

**50% dose reduction in dynamic myocardial CT perfusion by skipping beat acquisitions is feasible**

*O. Sliwicka*[1], L. J. Oostveen[1], Z. Swiderska - Chadaj[2], K. Michielsen[1], J. Gommers[1], J. Habets[1], I. Sechopoulos[1]; [1]Nijmegen/NL, [2]Warsaw/PL
*(olga.sliwicka@radboudumc.nl)*

**Purpose:** To investigate the feasibility for dose reduction in dynamic myocardial CT perfusion (CTP) resulting from acquisitions involving periodic skipping of heartbeats.
**Methods or Backround:** Dynamic CTP datasets of 38 patients (15 positive for ischaemia at invasive coronary angiography) acquired with a 320-slice CT system (Aquilion ONE PRISM, Canon Medical Systems), reconstructed using a deep-learning-based algorithm (AiCE Cardiac) were retrieved. From these, new datasets were created with every 4th, 3rd, or 2nd-beat removed (Skip4, Skip3, Skip2, respectively). Seven radiologists evaluated the post-processed images and perfusion maps to indicate presence and probability of perfusion deficit. The area under the ROC curve (AUC) and intraclass correlation coefficient (ICC) were computed. The mean blood flow (MBF) in 16 myocardial segments for each skipped beat sequence was normalised by the respective MBF value for the full-dose acquisitions, averaged across patients, and analysed with ANOVA. Quantitatively, cases were assessed as positive if at least one segment had an MBF<1.0. The number of maps with perfusion defect corresponding to the stenosis on ICA according to the American Heart Association segment model and number of non-diagnostic MBF maps (>4 ml/g/min) were noted.
**Results or Findings:** No difference was detected in the AUC across datasets (p value=0.44) with good observer agreement (ICC=0.8), and in relative normalised MBF (average MBF (mean absolute error)) Skip4: 1.02 (0.11); Skip3: 1.03 (0.17); Skip2: 1.06 (0.20), p value=0.66). 12 out of 15 (80%) perfusion maps showed hypoperfused regions corresponding to the coronary change and three (20%) maps had non-diagnostic MBFs across all reconstructions.
**Conclusion:** Skipping every second beat acquisition does not decrease diagnostic performance in dynamic myocardial CTP with the consequent reduction of 50% in the radiation dose.
**Limitations:** Patient's coronary dominance was not included in the calculations.
**Ethics committee approval:** A waiver was obtained.
**Funding for this study:** No funding was received for this study.
**Author Disclosures:**
Olga Sliwicka: Nothing to disclose
Luuk J. Oostveen: Nothing to disclose
Zaneta Swiderska - Chadaj: Employee: Crop Science Bayer Research/Grant Support: Centre for Priority Research Area Artificial Intelligence and Robotics of Warsaw University of Technology within the Excellence Initiative: Research University (IDUB) programme. Research/Grant Support: Foundation for Polish Science (FNP)
Jessie Gommers: Nothing to disclose
Koen Michielsen: Nothing to disclose
Ioannis Sechopoulos: Research/Grant Support: Siemens Healthcare, Canon Medical, ScreenPoint Medical, Sectra Benelux, Volpara Healthcare, Lunit, iCAD Speaker: Siemens Healthcare
Jesse Habets: Nothing to disclose

### RPS 1103-11

**Local biomechanical properties and MSCT elasticity of the dilated ascending aorta**

*V. V. Saushkin*, Y. Varlamova, A. Skosyrsky, D. Panfilov, B. Kozlov, S. Sazonova; Tomsk/RU
*(saushkin.vv@outlook.com)*

**Purpose:** The aim of the study was to determine the association of biomechanical properties of the dilated ascending aorta (AA) with elasticity indices obtained by MSCT data.
**Methods or Backround:** The study included 24 patients (aged 61±9 years; 13 men) with dilated AA (AA diameter > 50mm). All patients underwent ECG-gated CTA. CTA datasets were reformatted into 10-phases. We measure AA diameters, cross-sectional areas and aortic segments length in systole and diastole. Additionally, we calculated circular strain (Cst), longitudinal strain (Lst), volume strain (Vst), compliance (com), and distensibility (dis) of AA. Further, all patients underwent AA replacement. Circular (cir) and longitudinal (lon) samples were collected from the resected AA. The obtained AA samples were used for failure testing. For all samples we obtained the following: Young's modulus (YM); failure stress (FS); tensile stress (TS); elongation(E); strain(ST).
**Results or Findings:** It was found that the biomechanical properties of the longitudinal and circular AA samples significantly differ from each other: YM 2,6 (2,02-3,33) vs 3,2 (2,37-3,72) MPa, p<0,02; FS 2,14 (1,36-2,87) vs 3,59 (1,94-4,81) N, P<0,03; TS 0,19 (0,13-0,32) vs 0,34 (0,27-0,45) MPa, p<0,02; E 6,9 (4,8-8,9) vs 8,3 (6,3-10,8)mm, p<0,005; ST 4,5 (2,9-5,7) vs 4,8 (3,9-6,3)%, p<0,04. We found a correlation between AA elasticity (MSCT) and the biomechanical properties of the AA samples. A moderate positive correlation was found between Cst and YM(lon) (r=0,41, p=0.004), Lst and YM(lon) (r=0,401, p=0,05), Lst and YM(cir) (r=0,48,p=0.02), Vst and YM(cir) (r=0,48, p=0,03), Dis and ST(cir) (r=0,52, p=0,01)m between Vst and TS(cir) (r-0,46, p=0,03), Vst and ST(cir) (r=-0,54, p=0.009). A strong positive correlation between Dis and E(cir) (r=0,63, p=0,001) and a strong negative correlation between Vst and FS(cir) (r=-0,61, p<0,002) were found.
**Conclusion:** This study showed the strongest correlations between TS(cir) and Vst, and E(cir) and dis. Circular samples are stronger and have more elastic properties that longitudinal samples. MSCT is a promising method to assess aortic elastic properties.
**Limitations:** No limitations were identified.
**Ethics committee approval:** This work was approved by an ethics committee.
**Funding for this study:** This study was supported through a grant from the Russian Science Foundation (project № 21-15-00160).
**Author Disclosures:**
Anatoliy Skosyrsky: Nothing to disclose
Svetlana Sazonova: Nothing to disclose
Yulia Varlamova: Nothing to disclose
Dmitriy Panfilov: Nothing to disclose
Viktor V. Saushkin: Nothing to disclose
Boris Kozlov: Nothing to disclose

### Friday, March 3

08:00-09:00 Research Stage 1

### Research Presentation Session: Abdominal Viscera & GI Tract

### RPS 1201

### What's new in LI-RADS and pre-liver transplant imaging

**Moderator**
A. Torregrosa Andrés; Valencia/ES
*(asunsuso@gmail.com)*

### RPS 1201-2

**Portosystemic shunts before liver transplantation: correlation between total shunt area and early graft dysfunction**

*A. Vanzulli*, G. Gorga, L. Centonze, M. Giavarini, F. Rizzetto, L. A. Carbonaro, A. Vanzulli; Milan/IT
*(andrea.vanzulli@unimi.it)*

**Purpose:** Spontaneous portosystemic shunts (SPSS) are the consequences of an abnormally increased portal venous pressure due to liver cirrhosis. The impact of SPSSs on natural history of cirrhotic patients was recently evaluated through the measurement of total shunt area (TSA), which allows a comprehensive assessment of SPSSs extension identifying a direct correlation of higher TSA with lower patient survival. Our purpose is to analyse the clinical impact of the TSA on the development of early allograft dysfunction (EAD), and graft and patient survival following LT.
**Methods or Backround:** Preoperative imaging of 346 cirrhotic patients undergoing primary LT between 2015 and 2020 were retrospectively revised, recording the size and anatomy of each SPSS to calculate TSA. The impact of TSA and selected patient and donor characteristics on the development of EAD, AKI, and clinically relevant complications was evaluated through univariate and multivariate logistic regression, whereas their effect on graft and patient survival was investigated through Cox regression analysis.
**Results or Findings:** A TSA exceeding 78.54 mm2 resulted as an independent risk factor for the development of EAD (odds ratio [OR]: 2.327; P = 0.003), grade 3 AKI (OR: 2.093; P = 0.041), and clinically relevant complications (OR: 1.962; P = 0.015). Moreover, higher TSA was significantly related to early graft and patient survivals, emerging as an independent risk factor for 12-mo graft loss (hazard ratio: 3.877; P = 0.007) and patient death (hazard ratio: 2.682; P = 0.018).
**Conclusion:** Higher TSA emerged as a significant risk factor for worse postoperative outcomes following LT, supporting the need for careful haemodynamic assessment and management of patients presenting multiple/larger shunts.
**Limitations:** The main limitation of this study is represented by its retrospective nature.
**Ethics committee approval:** This retrospective single-institution cohort study was formally approved by local Institutional Review Board.
**Funding for this study:** No funding was received for this study.
**Author Disclosures:**
Leonardo Centonze: Nothing to disclose
Francesco Rizzetto: Nothing to disclose
Luca Alessandro Carbonaro: Nothing to disclose
Angelo Vanzulli: Nothing to disclose
Andrea Vanzulli: Nothing to disclose
Matteo Giavarini: Nothing to disclose
Giovanna Gorga: Nothing to disclose

### RPS 1201-3

**Implementation of LI-RADS diagnostic algorithm with subtracted images**

*X. X. Tong*, I. Mercante, F. Crimì, E. Quaia, F. Vernuccio; Padova/IT
*(xiaoxiao.tong@studenti.unipd.it)*

**Purpose:** LI-RADS algorithm suggests optional use of subtraction images for the diagnosis of hepatocellular carcinoma (HCC). This study aims to investigate the role of subtraction images for the detection of LI-RADS major features, observation and categorisation in patients at risk of HCC.
**Methods or Backround:** This retrospective study included 34 high-risk patients (mean age ± SD: 61 ± 11 years old) with at least one untreated and non-LR-1 observation imaged with contrast-enhanced MRI between April 2015 and May 2019. A maximum of 3 lesions were considered per patient. Reference standard was based on pathology, upgrade to LR-5 and size stability/reduction at 2-year follow-up. Major features were analysed on conventional and subtraction images and observation categorisation was reported.
**Results or Findings:** Overall 54 observations (mean size ± SD: 22 ± 18 mm) were analysed, of which 23 (42.6%) were 10-19 mm in size. Of the 25 observations with arterial phase hyperenhancement (APHE) but without washout (WO) on conventional images, 6 (24%) demonstrated APHE and WO in the subtracted images with category upgrade from LR-4 to LR-5 and all of them were proven HCC. Of the 15 observations without APHE, 4 (27%) demonstrated APHE and WO in the subtracted images with category upgrade from LR-4 to LR-5 and all of them were proven HCC. Overall, 21 observations showed APHE and WO on subtracted images; only 1 (5%) of these observations was proven benign at follow-up, but was categorised as LR-4 both on conventional and subtracted images. There was no category downgrade from LR-5 to LR-4.
**Conclusion:** Subtracted images improve the detection of LI-RADS major features and observation categorisation compared to conventional images.
**Limitations:** Identified limitations were: (1) the small study population, (2) the retrospective, single centre nature of the study, (3) that there was no inter and intraobserver agreement, and (4) there was no assessment of accuracy.
**Ethics committee approval:** No information provided by the submitter.
**Funding for this study:** No funding was received for this study.
**Author Disclosures:**
Federica Vernuccio: Nothing to disclose
Irene Mercante: Nothing to disclose
Xiao Xiao Tong: Nothing to disclose
Filippo Crimì: Nothing to disclose
Emilio Quaia: Nothing to disclose

### RPS 1201-4

**Standardised radiological reporting: are we practicing LI-RADS?**

*A. N. Zafar*[1], N. Uddin[2], Z. S. Faruqui[2], S. Q. Ahmad[1]; [1]Rawalpindi/PK, [2]Lahore/PK
*(aasmarad@gmail.com)*

**Purpose:** Liver imaging reporting and data system (LI-RADS) is an effective way of communicating probability of malignancy in at-risk patients. We aimed to determine the practice of using LI-RADS in our country.
**Methods or Backround:** An online questionnaire was sent to residents and qualified radiologists in different cities. The percentage of those practicing LI-RADS, data regarding workplace, experience and highest degree were recorded.
**Results or Findings:** Almost equal numbers of qualified and resident radiologists participated (50.8% versus 49.2%); a total of 252 participants. Their place of work was recorded as government institution in 34.5% (87/252), 76 (30.2%) were in private set-up, 59 (23.4%) in teaching institutions and 19 (19%) in semi-government set up. Out of 252, the majority were not reporting liver masses according to LI-RADS (66.7%). The main reason was reported as lack of knowledge by 24.4% (41/168); 5.9% (10/168) found it difficult; 14.9% (25/168) did not state any reason and 47% (79/168) did not answer. Their experience ranged from 0.5 to 38 years; highest qualification being FRCR (Fellow of Royal College of Radiologists) in 3/168 (1.8%), followed by FCPS (Fellow College of Physicians and Surgeons Pakistan) in 82/168 (48.8%), DMRD (Diploma in Medical Radio-diagnosis) in 2/168 (1.2%), MCPS (Member College of Physicians and Surgeons) 6/168 (3.6%) and MD (doctor of medicine) in 3/168 (1.8%). Amongst 80 participants who were reporting according to LI-RADS, 31 were consultants and 49 were trainees with experience ranging between 0.5 to 35 years. Highest qualification was FRCR in 4/31 (12.9%), followed by FCPS in 26/31 (83.9%) and DMRD in 1/31 (3.2%).
**Conclusion:** There is a lack of standardised reporting terminology by the majority of radiologists in our country. It is imperative to spread awareness regarding LI-RADS to improve therapeutic management.
**Limitations:** Inexperienced residents, who were not yet exposed to cross-sectional imaging were included.
**Ethics committee approval:** No information provided by the submitter.
**Funding for this study:** No funding was received for this study.
**Author Disclosures:**
Zia Salman Faruqui: Nothing to disclose
Saqib Qayyum Ahmad: Nothing to disclose
Aasma Nudrat Zafar: Nothing to disclose
Najam Uddin: Nothing to disclose

### RPS 1201-5

**Optimising imaging assessment of liver transplant candidates: a comparative study of CT versus MRI at a large UK liver transplant centre**

*C. R. A. Corallo*, A. Lory, J. Zhong, A. R. Laverty, J. Bell, J. A. Guthrie, R. Albazaz; Leeds/UK
*(Carmelo.corallo@doctors.org.uk)*

**Purpose:** Currently no national guidelines in liver transplant imaging assessment exists in the UK despite a national system of organ allocation. Multiphase CT recently replaced contrast enhanced MRI as modality of choice in our centre to improve vascular assessment as the main surgical priority. The aim of this retrospective study is to assess the performance of each modality, including hepatocellular carcinoma (HCC) detection, vascular evaluation & overall compliance to UK liver transplant criteria (UKTC).
**Methods or Backround:** Adults assessed for liver transplantation underwent MRI in 2018 & CT in 2020, with the aim of identifying HCC and any vascular issues that may complicate transplantation. Patients were identified using departmental radiology information system search with data collected from patient pathway manager.
**Results or Findings:** 91 adults had MRI with 45 lesions of average size 17.5 mm (5-50mm); 20 indeterminate, 6 probable HCC, 17 definite HCC & 2 treated HCC. Vascular assessment was inconclusive in 14/91 cases requiring further assessment with CT. 35/91 proceeded to liver transplant, with histological discrepancies found in 2/35 (5%). None were outside UKTC. 138 adults had CT demonstrating 15 lesions of average size 16.5mm (5.6-38mm); 10 indeterminate, 4 probable HCC and 1 definite HCC. Vascular assessment was conclusive in all cases. MRI was necessary for liver lesion characterisation in 15 cases. 50/138 underwent liver transplant with histological discrepancies found in 5/50 (10%). One patient with cholangiocarcinoma was outside UKTC.
**Conclusion:** CT (with MRI for problem solving) is a reliable technique for liver transplant assessment, with greater accuracy for vascular evaluation than MRI. It is expected that a minority of patients will be outside UKTC with either CT or MRI assessment.
**Limitations:** Biases of a retrospective study.
**Ethics committee approval:** No information provided by the submitter.
**Funding for this study:** No funding was received for this study.
**Author Disclosures:**
James Ashley Guthrie: Nothing to disclose
Adam Robert Laverty: Nothing to disclose
Alexander Lory: Nothing to disclose
Jim Zhong: Nothing to disclose
Carmelo Ranieri Alessandro Corallo: Nothing to disclose
Joshua Bell: Nothing to disclose
Raneem Albazaz: Nothing to disclose

### RPS 1201-6

**Application of LI-RADS standardised reporting recommendations on CT and MRI radiology reports in clinical practice**
*M. Milazzo*, F. C. Sorrentino, R. Cannella, G. Brancatelli; Palermo/IT
*(monicamilazzo92@gmail.com)*

**Purpose:** The LI-RADS encourages the use of standardised reporting and defines requirements to improve consistency and reporting standards. This study evaluates the application of LI-RADS reporting recommendations in CT and MRI reports from clinical practice.
**Methods or Backround:** This retrospective study included radiology reports obtained between January 2018 and May 2022 using the LI-RADS algorithm. Reports were reviewed to assess the timing (during day or nightshift), priority (ordinary or urgent), experience of the radiologists (subspecialised abdominal radiologists or general radiologists), and categorisation of the observations (described individually or in aggregate). The description of location, size, major and ancillary features of the observations, other hepatic or extrahepatic findings, and impression with final observation diagnosis or management recommendations were recorded and compared by using the Pearson $\chi^2$ or Fisher's exact test.
**Results or Findings:** A total of 973 reports provided by 28 different radiologists were included (720 reports from subspecialised abdominal radiologists and 253 from general radiologists). According to the highest category, the LR-5, LR-M, and LR-TIV were described in 247 (25.4%), 31 (3.2%), and 53 (5.4%) reports, respectively. Reports from subspecialised abdominal radiologists more frequently provided ancillary features (27.0% vs. 9.9%; p<0.001) and impression with detailed diagnosis or management recommendations (67.5% vs 57.7%; p=0.005), while reports from general radiologists more frequently described hepatic vascular findings (96.8% vs. 91.7%; p=0.006) and extrahepatic findings (99.2% vs. 94.7%; p=0.002). No differences were observed according to the timing or priority of the reports.
**Conclusion:** Findings and impression reporting are affected by the radiologists' experience in liver imaging, with general radiologists less commonly reporting ancillary features and final diagnosis or management recommendations in the impressions.
**Limitations:** Indentified limitations were: (1) this was a single-centre study, and (2) there was no assessment of the diagnostic performance.
**Ethics committee approval:** No information provided by the submitter.
**Funding for this study:** No funding was received for this study.
**Author Disclosures:**
Giuseppe Brancatelli: Nothing to disclose
Roberto Cannella: Other: Bracco, Bayer: support for attending meetings.
Francesca Chiara Sorrentino: Nothing to disclose
Monica Milazzo: Nothing to disclose

### RPS 1201-7

**Effect of portal vein variations and segment 4 artery origin on the right/left lobe volume ratio and remnant liver volume in right-lobe living donor liver transplantation**
A. Durur Karakaya, *H. Özen Atalay*, L. Oğuzkurt; Istanbul/TR

**Purpose:** In this study, we aimed to investigate the effects of portal vein (PV) variations and segment 4 artery origin on right/left lobe volume ratio and remnant liver volume (RLV) in preoperative evaluation of living donor candidates for right-lobe transplantation.
**Methods or Backround:** A total number of 293 adult right-lobe living donor candidates between 2019-2022 were included. Triphasic abdominal computed tomography images were evaluated at a workstation; right/left lobe volumes were calculated. PV variations and the segment 4 artery origin were determined. Portal vein (PV) variations were classified into four categories.
**Results or Findings:** In 293 cases, 219 (74.7%) type 1, 40 (13.5%) type 2, 28 (9.5%) type 3 and 4 (1.3%) type 4 PV were detected. Segment 4 artery originated from LHA in 185 (63.1%), RHA in 93 (31.8%), celiac trunk in 12 (4.1%), from other origins in 3 cases (1%). The ratio of right/left lobe volume in cases with type 1 PV was found to be significantly lower than cases with type 2 and type 3 PV. While the rate of RLV lower than 30% of total liver volume was 42.8% in cases with type 3 PV, this rate was found as 24.6% and 20% in cases with type 1 and type 2 PV, respectively. There was no statistically significant effect of segment 4 artery origin on the RLV.
**Conclusion:** Right/left lobe volume ratio is variable according to PV types. It is recommended that donors with type 3 PV should be evaluated in more detail in terms of prevention of remnant liver insufficiency. The origin of the segment 4 artery has no effect on the right/left lobe volume ratio of the liver.
**Limitations:** Our retrospective study included a limited number of patients from a single institution.
**Ethics committee approval:** This study was approved by Koc University Biomedical Research Ethics Committee, Istanbul/Turkey.
**Funding for this study:** Not applicable.
**Author Disclosures:**
Afak Durur Karakaya: Nothing to disclose
Hande Özen Atalay: Nothing to disclose
Levent Oğuzkurt: Nothing to disclose

### RPS 1201-8

**Preprocedural liver and spleen volumes in patients with hepatocellular carcinoma: correlation with outcomes**
*A. T. Tran*[1], S. Belfrage-Maher[1], H. Abbouchie[1], N. Raju[1], A. N. Murphy[2], K. Lim[1], D. G. Ranatunga[1], M. Goodwin[1], N. Kutaiba[1]; [1]Heidelberg, Victoria/AU, [2]Melbourne, Victoria/AU
*(anthonytutran@gmail.com)*

**Purpose:** Advancements in surveillance and diagnosis have led to earlier treatment of hepatocellular carcinoma (HCC) but has not reduced the rate of late recurrence. Our study aims to assess whether preprocedural liver volume (LV), spleen volume (SV) and spleen-to-liver volume ratio (SLVR) were correlated with mortality in HCC patients.
**Methods or Backround:** HCC patients who had curative treatment (surgery or ablation) with 2 years of complete response were identified from a prospective Australian hospital database. The patients' preprocedural CT scans were used to calculate LV and SV using a semi-automated software. Statistical correlation with multiple regression analysis was used to correlate the SV and SLVR with mortality. Other variables used for the analysis included: recurrence of HCC, gender, age at therapy, tumour size and cirrhosis.
**Results or Findings:** Seventy-six patients with mean (± SD) age 63.1 ± 8.6 years were included. There was late recurrence of HCC in nearly half of the patients (45%) and 26% were deceased at the end of follow-up. The mean (± SD) LV of all patients was 1,779 mls ± 596 mls and was significantly correlated with mortality (r2=0.37, p=0.043). The mean (± SD) SV of all patients was 446 mls ± 265 mls and was not significantly correlated with mortality (r2=0.37, p=0.076). The mean (± SD) SLVR of all patients was 0.26 ± 16 and was significantly correlated with mortality (r2=0.37, p=0.012).
**Conclusion:** Preprocedural LV and SVLR was found to be significantly correlated with mortality in HCC patients. Preprocedural SV showed a trend towards statistical correlation. Preprocedural CT volumetric analysis is feasible and can provide prognostic information.
**Limitations:** Identified limitations were: (1) the small sample size, and (2) that cause of death was not investigated.
**Ethics committee approval:** This study was approved by the Austin Health (Heidelberg, Victoria, Australia) Ethics Committee.
**Funding for this study:** No funding was received for this study.

**Author Disclosures:**
Nihal Raju: Nothing to disclose
Sebastian Belfrage-Maher: Nothing to disclose
Mark Goodwin: Nothing to disclose
Anthony Tu Tran: Nothing to disclose
Numan Kutaiba: Nothing to disclose
Dinesh Gerard Ranatunga: Nothing to disclose
Hussein Abbouchie: Nothing to disclose
Alexandra N Murphy: Nothing to disclose
Kelvin Lim: Nothing to disclose

**08:00-09:00** **Research Stage 2**

### Research Presentation Session: Artificial Intelligence & Machine Learning & Imaging Informatics

### RPS 1205

### Using AI for quality control in radiography

**Moderator**
L. Hammerstrøm; Oslo/NO
*(linham@oslomet.no)*

### RPS 1205-2

**Predicting a radiograph's diagnostic quality before its acquisition: an artificial intelligence approach using depth imaging in a cadaver study**

*M. M. Sieren*, F. Leal dos Reis, M. Laufer, D. Mairhöfer, T. Käster, E. Barth, T. Martinetz, J. Barkhausen, A. Bischof; Lübeck/DE
*(malte.sieren@uksh.de)*

**Purpose:** The alignment of the patient's anatomy in front of the radiographic tube is essential for the diagnostic quality of the resulting radiograph. Depth camera (time-of-flight; ToF) images paired with artificial intelligence (AI) offer the potential to capture the anatomy prior to radiation exposure and predict the resulting image quality. Thus, the technology could prevent low-quality radiographs and repeated radiation exposure. This study aimed to demonstrate the feasibility of AI-assisted quality prediction of radiographs of the upper ankle joint in a cadaver study.
**Methods or Backround:** Pairs of ToF images and quality-assessed radiographs are needed to train the AI algorithm. Using an anatomical cadaver of the ankle joint in varying angulations, 123 image data pairs were acquired. One radiologist semi-quantitatively assessed the quality of the radiographs as 1 (excellent), 2 (acceptable), and 3 (unacceptable). The dataset was split ten times in an 80:20 ratio (training/test group) to train an EfficientNet-B0 convolutional neural network. A deviation of ≤0.5 from the radiologist's assessment was considered a match. Statistics included accuracy, Pearson correlation coefficient, mean absolute error and mean squared error. Values are given as mean±standard deviation.
**Results or Findings:** The algorithm achieved a prediction accuracy of 87%±0.05 and a Pearson correlation coefficient of 0.86±0.06. The mean absolute error of the semi-quantitative assessment was 0.14±0.05, and the mean squared error was 0.12±0.05.
**Conclusion:** This study demonstrates the feasibility of quality prediction on radiographs of the upper ankle joint based on a depth imaging technique before radiation exposure occurs. In perspective, this assistance system could improve the quality of radiographs and reduce repeated radiation exposures due to repetitions of inadequate radiographs.
**Limitations:** Identified limitations were: (1) the small sample size, and (2) the experimental setup.
**Ethics committee approval:** This study was approved by the ethics committee of the University of Lübeck, file reference: 20-277.
**Funding for this study:** Funding was received from the Federal Ministry for Economic Affairs and Climate Action, grant number: 01MK20012.
**Author Disclosures:**
Thomas Käster: Founder: Pattern Recognition Company GmbH
Thomas Martinetz: Nothing to disclose
Manuel Laufer: Nothing to disclose
Arpad Bischof: Nothing to disclose
Erhardt Barth: Nothing to disclose
Jörg Barkhausen: Nothing to disclose
Malte Maria Sieren: Nothing to disclose
Dominik Mairhöfer: Nothing to disclose
Fabio Leal dos Reis: Nothing to disclose

### RPS 1205-3

**Can AI help us in acquiring good skeletal images? Towards automated quality assessment of 2D skeletal X-ray imaging using AI-assisted pose estimation**

*O. Hertgers*[1], K. Hergaarden[1], S. Challiui[1], A. Bubberman[1], M. Brueck[2], N. Wieberneit[2], T. Harder[2], J. von Berg[2], H. J. Lamb[1]; [1]Leiden/NL, [2]Hamburg/DE
*(ohertgers@gmail.com)*

**Purpose:** Improper patient positioning leads to suboptimal image quality and in turn to retakes. Automated feedback on image quality by AI-assisted pose estimation of joints on 2D X-ray could reduce this. We compare CNN-based pose estimation to expert annotations of positioning.
**Methods or Backround:** We retrospectively sampled 500 lateral and 500 anterior-posterior ankle-joint X-rays from our academic hospital. Images were labelled by three readers (radiographers and radiology resident). Incomplete annotations, paediatric and cases with obscured joint spaces were excluded. Six quality labels were used, with an overall image quality label. A previously trained CNN-based pose estimation was used to derive medial-lateral (ML), cranial-caudal (CC) and flexion angles of the joint. The overall image quality, the quality labels and the estimated angles were compared to determine which individual labels and estimated angles were statistically most predictive for the overall image quality.
**Results or Findings:** For the lateral and AP cases respectively, 3.1% and 4.6% of the cases were excluded according to the criteria listed above. Regarding 'overall image quality', reader-pairs were in substantial agreement, with Cohens kappa ranging 0.7-0.8. The annotated label 'medial-lateral rotation' could explain the overall image quality with 84.5% accuracy for lateral respectively 93.8% for AP view. The medial-lateral rotation quality was predicted by the CNN with an AUC of 0.88 for lateral images and 0.82 for AP. When using this estimated angle to predict the overall image quality, this could be done with an AUC of 0.79 and 0.81 respectively.
**Conclusion:** AI-assisted pose estimation can play a role for correct positioning of ankle joints. We have shown that ML-rotation is a good marker for overall image quality. This feature, and the overall image quality, was predicted with good confidence by the pose estimation algorithm.
**Limitations:** An identified limitation was the single-site validation.
**Ethics committee approval:** A waiver of consent was obtained from the local ethics committee.
**Funding for this study:** No funding was received for this study.
**Author Disclosures:**
Omar Hertgers: Nothing to disclose
K.F.M. Hergaarden: Nothing to disclose
Adriaan Bubberman: Nothing to disclose
Tim Harder: Employee: Philips Research Hamburg
Nataly Wieberneit: Employee: Philips Research Hamburg
Jens von Berg: Employee: Philips Research Hamburg
Matthias Brueck: Employee: Philips Research Hamburg
Hildo J. Lamb: Nothing to disclose
Samir Challiui: Nothing to disclose

### RPS 1205-4

**Automated quality control of chest X-rays**

*I. A. Selby*, E. González Solares, A. Breger, M. Roberts, L. Escudero Sanchez, J. Babar, N. Walton, E. Sala, C-B. Schönlieb; Cambridge/UK
*(ias49@cam.ac.uk)*

**Purpose:** Shortcut learning and reliance on confounding features have harmed the ability of COVID-19 chest X-ray (CXR) artificial intelligence models to generalise. We present an automated quality control pipeline aiming to rapidly clean CXR data by automatically standardising or rejecting images, whilst providing labels to identify confounding features, such as pacemakers and radiographic projection.
**Methods or Backround:** Almost 68,000 CXRs were curated from 5 datasets covering 39 centres in 7 countries. Confounding factors were selected by a multidisciplinary team of radiologists, mathematicians and data scientists. Tools were developed using classical statistical techniques and convolutional neural networks (CNNs). Two datasets were used for development, with three held-back for testing.
**Results or Findings:** In the first stage, a CNN checks and corrects greyscale inversion (sensitivity/specificity: 94%/98%) and image quality is scored. In the second, image annotations are read and removed, image padding is cropped, and aspect ratio is used to exclude CXRs with less than two thirds of the lungs included. In the final step, image rotation is quantified using a CNN (88%/95%) and 90º rotation is corrected. Annotations are used to label radiographic projection with a CNN utilised in cases of failure (90%/92%). CNNs are used to label the presence of a pacemaker (76%/80%) and exclude lateral views (89%/99%).

**Conclusion:** All the tools demonstrate excellent performance on external datasets, showing their ability to generalise to multiple demographics, locations, equipment, and settings. The proposed pipeline can be used for rapid quality control of large CXR data sets, allowing developers to produce generalisable, ethically-sound models that are invariant to confounding and shortcuts.
**Limitations:** Not applicable.
**Ethics committee approval:** Original approval obtained in April 2020, with 'substantial' amendments approved on 9th August 2021. Approval was provided by the London-Brent Research Ethics Committee, and the Health Research Authority (HRA) and Health and Care Research Wales (HCRW) (IRAS ID: 282705, REC No.: 20/HRA/2504, R\&D No.: A095585).
**Funding for this study:** Funding was received from the following sources: EU/EFPIA IMI2 Joint Undertaking: DRAGON (101005122); NIHR Cambridge Biomedical Research Centre (BRC-1215-20014); CRUK National Cancer Imaging Translational Accelerator (C22479/A28667); Cambridge Mathematics of Information in Healthcare (EP/T017961/1); Intel Corporation.
**Author Disclosures:**
Carola-Bibiane Schönlieb: Nothing to disclose
Eduardo González Solares: Nothing to disclose
Michael Roberts: Employee: AstraZeneca
Lorena Escudero Sanchez: Nothing to disclose
Evis Sala: Consultant: Amazon Speaker: Siemens Speaker: GSK Shareholder: Lucida Medical
Anna Breger: Nothing to disclose
Ian Andrew Selby: Nothing to disclose
Judith Babar: Nothing to disclose
Nicholas Walton: Nothing to disclose

### RPS 1205-5

**Detecting patient mix-ups in chest radiographs: an artificial intelligence approach**

*L. Berkel*[1], A. Saalbach[2], H. Schulz[2], M. P. Heinrich[1], J. Barkhausen[1], M. M. Sieren[1]; [1]Lübeck/DE, [2]Hamburg/DE
*(lennart.berkel@uksh.de)*

**Purpose:** Radiographs are essential in numerous diagnostic workflows for initial diagnosis and follow-up. However, patient mix-ups can be a source of error, especially in the demanding working environment of intensive care medicine. These mix-ups are often difficult for the radiologist to recognize but can lead to significant treatment errors. This study aimed to develop an artificial intelligence algorithm that detects patient mix-ups on follow-up chest radiographs from an intensive care unit.
**Methods or Backround:** A Siamese neural network, pre-trained on the NIH dataset, was employed to generate low-dimensional image descriptors for image comparison and patient verification. To investigate the feasibility and the generalisability of detecting mix-ups, 5752 chest radiographs from 2218 patients over a two-month timespan were selected from the institution's picture archive. In a case study, every image was taken as a query image and compared to the remainder of the dataset to verify the patient's identity. For evaluation, the precision was computed for patients with a single image, multiple images, and both patient groups. Additionally, different similarity thresholds were considered to facilitate the detection of new patients.
**Results or Findings:** Low similarity thresholds facilitated the detection of new patients. With an increasing threshold, a precision of 94.9% could be reported for patients with multiple images. A mid-level threshold resulted in the highest average precision for both patient groups (90.0%).
**Conclusion:** Our algorithm can reliably identify existing and new patients in a representative dataset taken from the clinical routine. Therefore, it provides promising options to detect patient mix-ups. This functionality could be employed, for example, to alert radiologists of patient mix-ups during the reporting process and thus avoid diagnostic errors.
**Limitations:** An identified limitation was that this was a single centre study.
**Ethics committee approval:** Funding was received from the ethics committee of the University of Lübeck, file reference: 20-277.
**Funding for this study:** Funding was received from the Federal Ministry for Economic Affairs and Climate Action, grant number: 01MK20012.
**Author Disclosures:**
Heinrich Schulz: Author: Employee
Lennart Berkel: Nothing to disclose
Axel Saalbach: Author: Employee
Matthias Paul Heinrich: Nothing to disclose
Jörg Barkhausen: Nothing to disclose
Malte Maria Sieren: Nothing to disclose

### RPS 1205-6

**How does AI feedback impact student and qualified radiographers' decision making?**

*C. Rainey*[1], C. Hughes[1], R. Bond[2], J. McConnell[3], S. L. McFadden[1]; [1]L/Derry/UK, [2]Belfast/UK, [3]Leeds/UK
*(c.rainey@ulster.ac.uk)*

**Purpose:** As artificial intelligence (AI) becomes more integrated into healthcare it is imperative to deploy this technology responsibly. This research attempts to add insight into the effect of forms of AI feedback on the interpretation accuracy of student and qualified radiographers, highlighting optimal aspects of AI feedback.
**Methods or Backround:** A study was designed in Qualtrics®, deployed at ECR 2021 and promoted via social media (Twitter®/LinkedIn®), resulting in 94 respondents. Each participant was presented with a random selection of three musculoskeletal examinations from a dataset of 21. Diagnostic accuracy under four conditions (AI correct, AI incorrect, pathological and non-pathological examinations) was determined by comparison with a known ground truth diagnosis from three to five reporting radiographers and/or radiologists. ANOVA, t-tests and descriptive statistics were used to describe the data.
**Results or Findings:** There is a statistically significant difference in diagnostic accuracy of all participants before any AI feedback and after provision of binary diagnosis, when the AI is correct (p=0.007). In pathological cases there was a statistically significant decrease in accuracy following presentation of the heatmap (p=0.015) and a subsequent increase in accuracy when the binary diagnosis was provided (p=0.013).
**Conclusion:** Heatmap feedback was detrimental to participant accuracy in pathological cases and did not have a positive impact on accuracy in any condition. Binary diagnosis was beneficial to both groups across all of the four conditions with a significant impact on accuracy when the AI was correct and in pathological cases. Methods of explainability should be developed with cognisance of the impact on the end-user.
**Limitations:** Participants were analysed as two broad groups. Impact of years of professional experience on interaction with the AI will be investigated.
**Ethics committee approval:** This study was approved by the UU Nursing and Health research filter committee, approval number FCNUR-20-035.
**Funding for this study:** Funding was received from the College of Radiographers Industry Partnership award Scheme (CoRIPS) and Ulster University.
**Author Disclosures:**
Jonathan McConnell: Nothing to disclose
Clare Rainey: Nothing to disclose
Ciara Hughes: Nothing to disclose
Raymond Bond: Nothing to disclose
Sonyia Lorraine McFadden: Nothing to disclose

### RPS 1205-7

**Quality assessment compliance using a combination of deep learning, NLP and limited human intervention: feasibility for real time institutional deployment**

H. Mahajan, V. Mahajan, *V. K. Venugopal*, R. Takhar, S. Pundir, S. Gupta, B. Bahl, P. Mehta; New Delhi/IN
*(vasanthdrv@gmail.com)*

**Purpose:** To evaluate the performance of Oxipit "ChestEye Quality" tool for quality assurance purposes in chest X-ray reporting on a retrospective data sample.
**Methods or Backround:** A retrospective sample of anonymised chest X-ray studies from the time period of 06.01.2016 - 01.08.2021 (n=3892) from an Indian medical institution was selected. The studies were processed by the solution which identified the cases with the most potential for a missed finding, based on the output of the solution and the results of the automatic evaluation of the radiologist's final report, using a custom-made chest X-ray report natural language processing tool. A radiologist evaluated the cases flagged by the software to determine if there was a clinically significant radiological finding detectable in the image which was not appropriately described in the radiologist report. The final performance metrics were evaluated to quantify the number of cases with significant radiological findings missed in the radiologist's report and flagged by the solution.
**Results or Findings:** A total of 34 studies (0.87 %) were evaluated as containing clinically significant radiological findings detectable in the image on retrospective review which were not appropriately described in the final radiologist report. A total of 20 studies contained nodular opacities, 11 studies contained an area of consolidation, one study contained a pneumothorax, one study contained malposition of central venous line and one study contained a pleural effusion.
**Conclusion:** The results indicate that AI-based software used for quality assurance purposes is able to detect studies where a clinically significant radiological finding is not appropriately described by the reporting radiologist. In a prospective setting, this could help to prevent a significant number of radiological errors.

**Limitations:** More studies in the same field are required to confirm the findings.
**Ethics committee approval:** A waiver was obtained for the use of retrospective deidentified data.
**Funding for this study:** No funding was received for this study.
**Author Disclosures:**
Salil Gupta: Employee: CARPL.ai
Shivam Pundir: Employee: CARPL.ai
Bhanushree Bahl: Employee: CARPL.ai
Parv Mehta: Employee: CARPL.ai
Rohit Takhar: Employee: CARPL.ai
Harsh Mahajan: Other: Research Collaboration with Oxipit.ai, Predible Health. Other: Director at Mahajan Imaging. Other: Research Collaboration with Koninklijke Philips NV research, Lunit.ai, Subtle Medical, General Electric, Qure.ai, Quibim.
Vasantha Kumar Venugopal: Other: Research Collaboration with Quibim, Qure.ai, Lunit.ai, Oxipit.ai, General Electric, Koninklijke Philips NV research, Predible Health, Subtle Medical. Consultant: CARPL.ai.
Vidur Mahajan: Other: Research Collaboration with Quibim, Oxipit.ai, Qure.ai, General Electric. CEO: CARPL.ai. Other: Research Collaboration with Lunit.ai, Predible Health, Subtle Medical, Koninklijke Philips NV research

### RPS 1205-8

**Whole-body magnetic resonance imaging in the large population-based German national cohort study (NAKO): use of an automated image quality assessment for the prediction of perceived image quality**

*C. Schuppert*[1], R. von Krüchten[2], J. G. Hirsch[3], D. C. Hoinkiss[3], R. Kaaks[1], F. Bamberg[2], H-U. Kauczor[1], M. Günther[3], C. L. Schlett[2]; [1]Heidelberg/DE, [2]Freiburg/DE, [3]Bremen/DE

**Purpose:** To investigate the influence of protocol repetitions in magnetic resonance imaging (MRI) on the perceived image quality, and to examine the applicability of automated image quality assessment for the prediction of the subjectively preferred acquisition.
**Methods or Backround:** Our analysis included all participants in the prospective, multi-centre German national cohort (NAKO) study who underwent whole-body MRI from 2014 to 2016 (n=11,347; all currently available data). Radiologic technologists (RTs) were instructed to repeat any of the 12 examination protocols for reasons of setup errors and/or subjectively unsatisfactory image quality, and select one acquisition from the resultant set to be used subsequently. Up to 11 quantitative image quality parameters were automatically derived from all acquisitions. Inferential statistics involved paired t-tests, signed-rank tests, and receiver operating characteristic (ROC) curves.
**Results or Findings:** Repetitions in 12% (n=1,365) of participants yielded 2,342 sets of two or more acquisitions, in 1,377 (58.8%) of which the repetitions were performed without setup changes. For this stratified sample, RTs selected a repetition over the baseline acquisition in 1,112 instances (80.8%, range across protocols: 74-100%). Image quality parameters commonly showed statistically significant differences between the selected and discarded acquisitions, e.g., "sharpness", "signal-to-noise ratio" and "structured noise average" (all p<.001) in the neurological protocols T1w-3D-MPRAGE and 2D-FLAIR. Many were also retrospectively predictive for the selection of an acquisition, e.g., providing an area under the ROC curve of 0.651 and 0.732 (all p<.001) for the combination of the three aforementioned parameters in the same protocols.
**Conclusion:** MRI protocol repetitions predominantly improved image quality as perceived by RTs in this large cohort study. Automated image quality assessment demonstrated a good predictive ability for their decision and may complement or even replace manual selection.
**Limitations:** An identifed limitation was that RTs were not blinded (most importantly).
**Ethics committee approval:** The scientific advisory board and the ethics advisory board of the NAKO approved this study.
**Funding for this study:** No funding was received for this study.
**Author Disclosures:**
Christopher L. Schlett: Nothing to disclose
Daniel C. Hoinkiss: Nothing to disclose
Hans-Ulrich Kauczor: Nothing to disclose
Rudolf Kaaks: Nothing to disclose
Fabian Bamberg: Nothing to disclose
Jochen G. Hirsch: Nothing to disclose
Matthias Günther: Nothing to disclose
Christopher Schuppert: Nothing to disclose
Ricarda von Krüchten: Nothing to disclose

**08:00-09:00** **Research Stage 3**

### Research Presentation Session: Neuro

### RPS 1211

### Vessel wall and neurovascular imaging

**Moderator**
J. Hendrikse; Utrecht/NL

### RPS 1211-2

**Automatic differentation of ruptured and unruptured intracranial aneurysms on computed tomography angiography based on deep learning and radiomics**

*J. Feng*[1], R. Zeng[1], Y. Geng[2], Q. Chen[2], Q. Zheng[1], T. Deng[1], L. Lv[1], B. Xue[1], C. Li[1]; [1]Chongqing/CN, [2]Beijing/CN
*(junbangfeng@163.com)*

**Purpose:** Rupture of intracranial aneurysm is very dangerous and has a high probability of death and disability. In this study, deep learning and radiomics techniques were used to automatically segment and extract the characteristics of aneurysms, and models were established to classify ruptured and unruptured status.
**Methods or Backround:** A total of 1007 aneurysm patients from two hospitals were included. Aneurysm identification, segmentation and morphological features extraction were automatically perfomed with a 3-dimensional CNN network. Radiomic features were additionally computed via a pyradiomics package. After, dimensionality reduction, classification models including support vector machines (SVM), random forests (RF), and multilayer perceptron (MLP) were trained in 898 aneurysms of hospital 1 and tested in the external dataset of hospital 2 (253 aneurysms). Model performance was evaluated via area under the curve (AUC) index of receiver operating characteristics and compared with the DeLong test.
**Results or Findings:** The 3-dimensional CNN automatically segmented aneurysms and calculated 21 morphological features. The pyradiomics obtained 14 radiomics characteristics. After dimensionality reduction, 13 features were found associated with aneurysm rupture. The AUCs of SVM, RF and MLP on the training dataset and external test dataset were 0.86, 0.85, 0.90, 0.85, 0.88, 0.86, respectively. DeLong test showed that there were no significant differences in the performance among three models in the external test dataset.
**Conclusion:** In this study, we established three classification models which can distinguish between ruptured and unruptured aneurysms accurately via radiomic shape and morphological features. Automatic segmentation and morphological measurements of aneurysms were performed by our artificial intelligence system.
**Limitations:** No limitations were identified.
**Ethics committee approval:** This study was approved by the Chongqing University Central Hospital.
**Funding for this study:** This study was supported by the Chongqing Science and Health Joint Medical Research Project (2022QNXM013).
**Author Disclosures:**
Zheng Qingqing Qingqing Zheng: Nothing to disclose
Bo Xue: Nothing to disclose
Chuanming Li: Nothing to disclose
Yayuan Geng: Nothing to disclose
Junbang Feng: Nothing to disclose
Lei Lv: Nothing to disclose
Qiang Chen: Nothing to disclose
Rong Zeng: Nothing to disclose
Tie Deng: Nothing to disclose

### RPS 1211-3

**Flow and velocity in intracranial arteries of patients with arteriovenous malformations measured with phase-contrast magnetic resonance imaging**

*M. Correia de Verdier*, E. Ronne-Engström, L. Borota, J. Wikström; Uppsala/SE
*(maria.correiadeverdier@radiol.uu.se)*

**Purpose:** The aim of this study was to evaluate phase-contrast MRI (PC-MRI) measured flow and velocity in feeding arteries of arteriovenous malformations (AVMs) before and, when possible, also after treatment. Furthermore, we compared these measurements to the corresponding measurements in healthy controls to identify which parameter has the best potential to discriminate normal from pathological.
**Methods or Backround:** Highest flow (HF), lowest flow (LF), mean flow (MF), peak systolic velocity (PSV), end-diastolic velocity (EDV) and mean velocity (MV) were measured in feeding arteries in patients with intracranial AVMs using 2D PC-MRI at 3 T. Measurements were compared to previously reported values in healthy individuals. Values in patients above the 95th percentile in the healthy cohort were categorised as pathological. Nidus volume was measured using 3D time-of-flight (TOF) MR angiography.
**Results or Findings:** Ten patients with diagnosed AVMs were examined with PC-MRI. Of these, three patients also underwent follow-up PC-MRI after treatment. We found correlations between nidus volume and flow and velocity parameters (HF, MF, PSV, EDV and MV). Higher velocities (PSV, EDV and MV) were seen in patients with a nidus larger or equal to 5.7 cm3 compared to healthy controls. After treatment, there was a decrease in flow and velocity (all measured parameters). After treatment, velocities (PSV, EDV and MV) were no longer abnormal compared to healthy controls.
**Conclusion:** Pathological velocities can be identified in patients with a large nidus. After treatment, a decrease in flow and velocity is observed and velocities are normalised.
**Limitations:** The main limitation is the small and heterogeneous patient population, which underlines the relatively rare pathology of intracranial AVMs.
**Ethics committee approval:** No information provided by the submitter.
**Funding for this study:** Funding was received from The Swedish Stroke Association and Uppsala County Council.
**Author Disclosures:**
Ljubisa Borota: Nothing to disclose
Johan Wikström: Nothing to disclose
Elisabeth Ronne-Engström: Nothing to disclose
Maria Correia de Verdier: Nothing to disclose

### RPS 1211-4
**Vascular steal evidenced by early diffusion imaging of brain arteriovenous malformations**
*F. O. O. Ordoñez Rivas*, L. A. Medrano Danés, O. N. López Razo, C. A. Casas Murillo, M. Mercado; Monterrey/MX
*(frankfoor_18@hotmail.com)*

**Purpose:** This study aims to assess the microstructural changes in brain tissue conditioned by the theft phenomenon in patients with cerebral AVMs.
**Methods or Backround:** The ADC technique is a valuable tool in the quantitative study of the microstructural changes in the brain parenchyma. A common cause of these changes in some pathologies is chronic hypoperfusion. However, no studies have been conducted using this MRI technique to study the steal phenomenon in patients with AVM. Using the ADC technique, we analysed MRI studies of 17 patients between 10 and 60 years of age with a diagnosis of cerebral AVM and 27 studies of healthy patients, also between 20 and 60 years of age. The location, size and degree of AVMs according to the Spetzler-Martin classification were established and the respective ADC values of the brain parenchyma in both the affected and contralateral hemispheres were calculated.
**Results or Findings:** The areas that showed the most significant increase in ADC values were adjacent to the AVM nest. A statistically significant correlation (0.630) was found between the degree of AVM according to the Spetzler-Martin classification and the pattern of extension of the theft phenomenon ($p=0.180$) in the 10-20 years age group, but not in the 20-60 years age group ($p=0.813$).
**Conclusion:** Quantifiable changes in ADC values were observed, even when not visualized with structural MRI techniques. Therefore, this is a feasible, practical, economical, and non-invasive alternative to detect, delimit and quantify microstructural changes in the brain parenchyma conditioned by chronic hypoperfusion in patients with cerebral AVM.
**Limitations:** This is a retrospective study, the sample size was small, and sub-group analysis was even smaller.
**Ethics committee approval:** No information provided by the submitter.
**Funding for this study:** Not applicable.
**Author Disclosures:**
Luis Adiel Medrano Danés: Nothing to disclose
Claudio Alberto Casas Murillo: Nothing to disclose
Mariana Mercado: Nothing to disclose
Olga Nidia López Razo: Nothing to disclose
Franklin Orlando Ordoñez Ordoñez Rivas: Nothing to disclose

### RPS 1211-5
**Usefulness of ASL smearing artefact in differentiating aggressive from benign intracranial dural arteriovenous fistulas**
*H. K. Kamepalli*[1], V. S. V. G. Kalaparti[1], S. k. Kannath[2], B. Thomas[2], C. Kesavadas[2]; [1]Thiruvananthapuram/IN, [2]Kerala/IN
*(haki.kamepalli@gmail.com)*

**Purpose:** To exploit the arterial spin labelling (ASL), a non-invasive imaging technique to diagnose and classify intracranial dural arteriovenous fistula (DAVF) into benign and aggressive lesions based on the demonstration of the smearing artefact along with CVR (cortical venous reflex).
**Methods or Backround:** DSA (digital subtraction angiography) is the gold standard to diagnose and classify DAVF, however it is invasive and large amounts of radiation dose and contrast are unavoidable. Hence ASL was used in the assessment of suspected DAVF cases. This is a retrospective study and a total of 42 patients were included.
**Results or Findings:** A total of 43 and 51 fistulous sites were detected on ASL and DSA respectively. Smearing artefact is able to differentiate simple and aggressive types of DAVF with a sensitivity of 93% ($p<0.01$) while CVR has a sensitivity of 96.6 % ($p<0.05$). Similarly, each of them could demonstrate the aggressive fistula in 75% of the cases. When both of these techniques are combined, the sensitivity of differentiation increased to 96.88% with an accuracy of 86.05%. Moderate (k- 0.70) and perfect (k-1) interobserver agreement could be achieved in the interpretation of CVR and smearing artefact images respectively.
**Conclusion:** By demonstrating this artefact, overall sensitivity in identifying aggressive fistulas can be increased with perfect interobserver agreement. For those individuals who have been treated for DAVF but had their suspicion for recurrence on the lower side, it is possible to follow up with MRI along with ASL.
**Limitations:** Spatial resolution obtained may not be acceptable at times. Due to the limited temporal resolution, flow direction cannot be determined. Small amount of signal change may be challenging, to locate shunts with low flow rates.
**Ethics committee approval:** No information provided by the submitter.
**Funding for this study:** No financial disclosures involved in this study.
**Author Disclosures:**
Viswanadh Sri Venkata Ganesh Kalaparti: Nothing to disclose
Bejoy Thomas: Nothing to disclose
Chandrasekharan Kesavadas: Nothing to disclose
Hari Kishore Kamepalli: Nothing to disclose
Santosh kumar Kannath: Nothing to disclose

### RPS 1211-6
**The influence of contralateral circulation on the computational fluid dynamics of intracranial arteries: comparison between the simulated versus measured flow velocities**
*S. Oh*, S. T. Park, Y. Song; Seoul/KR
*(127321@schmc.ac.kr)*

**Purpose:** To evaluate the influence of contralateral circulation on computational fluid dynamics (CFD) of intracranial arteries, in comparison with the measured values using phase contrast MR angiography (PCMRA).
**Methods or Backround:** In 16 patients who performed both TOF MA angiography and PCMRA, 23 unilateral anterior circulation models without proximal stenosis were enrolled. CFD was performed with the inflow boundary condition of a pulsatile flow of internal carotid artery (ICA), obtained from PCMRA. Outflow boundary condition was given as atmospheric pressure. Simulated flow velocities of middle cerebral artery (MCA) and anterior cerebral artery (ACA) from CFD were compared with the measured velocity values from PCMRA.
**Results or Findings:** The velocities of MCA (mean difference: 32.59mm/sec) were shown to be more accurately simulated on CFD than the values of ACA (mean difference: 64.97mm/sec). In 4 models with severe stenosis/occlusion in the contralateral ICA, the simulated ACA velocities were significantly lower (<50%) than the measured values from PCMRA. Models from patients who had similar diameter of both ACAs are more likely to accurately simulate ACA velocities on CFD.
**Conclusion:** In CFD simulation of ACA flow velocity using unilateral anterior circulation model, the simulated values can be significantly lower than the measured values when there is severe stenosis or occlusion in the contralateral ICA. It may be necessary to consider the flow condition of the contralateral circulation in CFD of intracranial arteries.
**Limitations:** This study has a retrospective study design with a small sample size. There are intrinsic limitations in measuring intracranial arterial velocities using PC-MRI (partial volume effect, intravoxel phase dispersion, displacement artefact etc.). Intracranial arteries in posterior circulation were not analysed despite high rupture risk of posterior circulation aneurysms. This study was designed with underlying assumptions of laminar flow and Newtonian incompressible flow for blood flow.
**Ethics committee approval:** No information provided by the submitter.
**Funding for this study:** No funding was receied for this study.
**Author Disclosures:**
Sujeong Oh: Nothing to disclose
Sung Tae Park: Nothing to disclose
Yunsun Song: Nothing to disclose

**08:00-09:00** **Research Stage 4**

### Research Presentation Session: Radiographers

### RPS 1214

### Patient-centered care in radiography

**Moderators**
E. Kjelle; Kongsberg/NO
*(elin.kjelle@hbv.no)*
F. Macri; Geneva/CH

### RPS 1214-3

**Can communication tools help radiographers to overcome the challenges they encounter in communicating with a range of patients?**

S. Quirke, *A. England*, N. Moore, M. F. F. McEntee; Cork/IE
*(aengland@ucc.ie)*

**Purpose:** Many of the tasks performed by radiographers rely on effective communication with patients. This study looks at the experiences of radiographers' communicating with patients to determine what communication skills they believe they have, what challenges they encounter and if they have any familiarity with communication tools. It also assesses their willingness to accept further training on communication and utilise recognised communication tools like AIDET, which stands for "acknowledge, introduce, duration, explanation and thank you".
**Methods or Backround:** Data was collected using an online survey on the Microsoft (MS) forms platform. The survey consisted of 43 questions: 13 qualitative 'open-ended' questions and 30 'close-ended' quantitative questions. The survey gathered responses from the 25/03/22-14/05/22. The qualitative data from the online survey responses were analysed using descriptive statistics and thematic analysis. Cross distribution analysis, basic percentages, and graphic bar charts were used for quantitative data analysis.
**Results or Findings:** This survey was completed by a total of 109 radiographers. For the results five themes emerged (1) expectations, (2) education, (3) improvements, (4) errors, and (5) communication tools. The quantitative analysis found that 83.6% of radiographers had not received additional training in patient communication post qualification. According to 88.5% of participants, AIDET could be a helpful training tool for radiographers' patient interactions.
**Conclusion:** Radiographers do not believe they have received adequate training for communicating with patients and would like to receive additional training and education in communicating with patients. AIDET could be a valuable communication tool and could be used as a guide for student radiographers commencing their career working with people or for international radiographers whom English is not their native language.
**Limitations:** Survey questions were self-reported and may not have fully reflected professional practice.
**Ethics committee approval:** University College Cork Social Research Ethics Committee
**Funding for this study:** No funding was received for this study.
**Author Disclosures:**
Mark F. F. McEntee: Nothing to disclose
Niamh Moore: Nothing to disclose
Sonya Quirke: Nothing to disclose
Andrew England: Board Member: EFRS

### RPS 1214-4

**Ready patient one: could a virtual experience help as a preparatory tool prior to MRI scanning?**

*D. Hudson*[1], C. Heales[2]; [1]London/UK, [2]Exeter/UK

**Purpose:** The aims of this feasibility study were to assess how realistic a virtual scan experience was for participants and whether this helped manage emotional control associated with having an MRI.
**Methods or Backround:** Despite developments in MRI which have helped improve the patient experience, the procedure remains a source of anxiety for many, which can be expressed as claustrophobia due to the physicality of the scan equipment. The outcome of which can be movement, reduced scan quality or even inability to undergo a scan. Traditional forms of patient preparation only go so far in representing what to expect from a scan, and so virtual reality (VR) has emerged as a potential means of providing enhanced support prior to a scan. Fifteen participants volunteered to undergo a VR session comprising of two virtual scan experiences. Measures to assess their demand and resource appraisal of the experience and that of having a MRI were obtained throughout, along with more general feedback on its realism, usefulness and application.
**Results or Findings:** All participants managed to complete two virtual head scans within VR. Restriction was the dominant concern regarding claustrophobia and was shown to significantly reduce following the exposures. Demand-resource evaluation scores showed six participants to initially be in a state of 'threat', with all moving to, or lowering a state of 'challenge' following exposure, in particular after the second. Confidence to cope also significantly improved following exposure to the virtual experience. The virtual experience was considered realistic by users with some areas for improvement highlighted.
**Conclusion:** Results suggest VR does suitably represent reality and elicit an emotional response that can be improved through exposure, thereby suggesting such tools as beneficial in preparation for an MRI scan.
**Limitations:** Thys study had a small sample size.
**Ethics committee approval:** This study has received the psychology research ethics approval from the University of Exeter and the organisational approval from InHealth.
**Funding for this study:** No funding was received for this study.
**Author Disclosures:**
3rd co-author Christine Heales: Author: University of Exeter
Darren Hudson: Author: InHealth

### RPS 1214-5

**Evaluating radiographers' experiences of imaging examinations for patients with dementia**

K. Browne, *A. England*, N. Moore, R. Young, A. Owen, M. F. F. McEntee; Cork/IE
*(aengland@ucc.ie)*

**Purpose:** Patients with dementia present frequently to the radiology department for imaging. Stigmatisation around dementia may contribute to negative interactions during imaging examinations of these patients, leading to frustration for both patients and radiographers. The study aimed to investigate the radiographers' perspectives on specific factors impacting interactions during imaging patients with dementia.
**Methods or Backround:** An interaction form was developed and deployed in a single acute care hospital in Ireland. Radiographers were requested to complete the interaction form each time they were directly involved in examinations for patients known to have dementia. The form consisted of 16 closed quantitative items and one open question exploring aspects of the examination and factors contributing to the radiographer-patient interaction.
**Results or Findings:** Thirty-three interaction forms were completed. The majority (58%, n=19) involved general radiography examinations, with 27% (n=9) from CT examinations. Radiographers reported: 69% of patients appeared distressed when undergoing the imaging examination, 84% of examinations were reported to take longer than expected, and 27% of examinations required repeat imaging. The use of distractive and communicative techniques to make the patient feel more comfortable, along with presence of carers were reported to support the efficient completion of examinations.
**Conclusion:** The challenges identified appear to impact on patient distress levels, and can lead to repeat imaging, extended examination times, or the rebooking of examinations. This study highlights the need for interventions to support and improve quality of care for patients with dementia attending the radiology department.
**Limitations:** Thirty-three interaction forms were completed and this study captured experiences from a single centre, excluding MRI. Further work is required to increase knowledge regarding radiographer-patient interactions in those with a known diagnosis of dementia.
**Ethics committee approval:** This study was approved by the University College Cork Social Research Ethics Committee.
**Funding for this study:** No funding was received for this study.
**Author Disclosures:**
Mark F. F. McEntee: Nothing to disclose
Niamh Moore: Nothing to disclose
Rena Young: Nothing to disclose
Katie Browne: Nothing to disclose
Andrew Owen: Nothing to disclose
Andrew England: Board Member: EFRS

### RPS 1214-6

**Effect of relaxing music in patients during ECG GATED myocardial perfusion scintigraphy**

*C. A. P. Aguiar*, S. I. Rodrigues, A. F. C. L. Abrantes, L. P. V. Ribeiro, J. Rosas, R. P. P. Almeida, J. Pinheiro; Faro/PT

**Purpose:** Previous studies suggest that relaxing music has a positive effect on human behaviour, modifying vital signs, decreasing anxiety levels, and alleviating emotional distress. Furthermore, music has been shown to alter discomfort perception by distraction. The main aim of this study was to verify whether there was a significant effect on heart rate (HR) and HR variability due to listening to relaxing music during ECG GATED Myocardial Perfusion Scintigraphy (MPS) imaging procedures.

**Methods or Backround:** In the phase 1, stress examination procedures were considered. In the control group, 30 patients were placed in a “no music group” and did not receive headphones or any type of music. In the experimental group, 27 patients underwent to the ECG GATED MPS with relaxing music played through headphones. In the phase 2, rest examination procedures were considered using 30 patients in the experimental group and 27 patients in the control group. Patients’ HR were recorded in both scenarios.
**Results or Findings:** In phase 1, statistically significant differences were found in the HR of patients in the experimental group (t=3.028; p=0.005), translated into a decrease in the mean HR. In the remaining situations, no significant differences were observed although the HR and HR variability were slightly lower in the experimental groups in both phases.
**Conclusion:** In this study, the effect of relaxing music on patients was verified, as the HR decreased or stabilised in most patients subjected to music, especially in the phase 1 (stress examination). Thus relaxing music could provide benefits to both patient and radiographers, and may be considered within the routine of these procedures.
**Limitations:** Sample size, only one setting, and different types of relaxing music have not been tested.
**Ethics committee approval:** Ethics committee approved the study and written informed consent was delivered to the participants.
**Funding for this study:** No funding was received for this work.
**Author Disclosures:**
António Fernando Caldeira Lagem Abrantes: Nothing to disclose
Rui Pedro Pereira Almeida: Nothing to disclose
Sónia Isabel Rodrigues: Nothing to disclose
Joao Pinheiro: Nothing to disclose
Joana Rosas: Nothing to disclose
Carina Alexandra Pires Aguiar: Nothing to disclose
Luís Pedro Vieira Ribeiro: Nothing to disclose

### RPS 1214-7

**Perceptions of annual surveillance with MRI in women with a hereditary risk of breast cancer: a phenomenographic study**

*A-S. P. Sjöqvist*[1], J. Gardling[2], A. Holst-Hansson[2], S. Zackrisson[1], A. Bolejko[1]; [1]Malmö/SE, [2]Lund/SE
*(ann-sofi.sjoqvist@med.lu.se)*

**Purpose:** The aim of the study was to describe perceptions of annual surveillance by MRI of women with hereditary risk of breast cancer. The specific questions were to study perceptions of inclusion and participation in the surveillance program, along with perceptions of care during MRI examination.
**Methods or Backround:** In Sweden, women with heredity for breast cancer (BC) are offered yearly surveillance with magnetic resonance imaging (MRI) for early detection of the disease. This approach might favour prognosis. However, it is little known how these women perceive participation in surveillance by MRI. Individual interviews with 14 women in annual surveillance with MRI were conducted. A semi-structured interview guide was used and phenomenography was used as the research method. Phenomenography aims to explain how people perceive the world in real, experienced situations. Comprehension of the relation between different perceptions and the description by categories of how they relate to each other in a hierarchical order aims to explain the perception of the phenomena.
**Results or Findings:** Three categories emerged: `considering own risk of developing BC´, `surrendering to surveillance´ and `living in a cycle´. These categories describe the outcome space of women`s perceptions of participation in the surveillance program by MRI and their perceptions of care during examination.
**Conclusion:** The results of this study’s expected utility are to raise awareness among radiographers and professionals within clinical genetics, genetic oncology and primary care who meet these women with heredity for BC. The knowledge that these women experience `living life in a cycle´ discloses further research to investigate women’s lived experience in relation to annual surveillance.
**Limitations:** This study is performed in two radiology departments in the south of Sweden and might not be representative nationwide.
**Ethics committee approval:** Ethical Review Authority Sweden D-2019-06348
**Funding for this study:** Malmö Cancer Fundation
**Author Disclosures:**
Ann-Sofi Pia Sjöqvist: Nothing to disclose
Annette Holst-Hansson: Nothing to disclose
Anetta Bolejko: Nothing to disclose
Sophia Zackrisson: Other: Siemens Healthcare AG, Pfizer, BayerAG.
Jenny Gardling: Nothing to disclose

### RPS 1214-8

**Carers and Comforters: a survey of radiographers and radiation therapists' knowledge and experience**

R. Simon, *A. England*, N. Moore, R. Young, M. F. F. McEntee; Cork/IE
*(aengland@ucc.ie)*

**Purpose:** Carers and Comforters are those persons who are knowingly and willingly exposed to ionising radiation in support of an individual undergoing a radiation exposure. Justification for the exposure rests with the practitioner who is responsible for assessing the situation and determining that the exposure incurred by the carer and comforter is of benefit and that the risks are relayed to the carer or comforter.
**Methods or Backround:** An online survey was distributed in Europe via social media, exploring registered radiographers' and radiation therapists’ (or equivalent) knowledge and experience of working with carers and comforters.
**Results or Findings:** In total there were 89 responses to. the survey but only 86 responses met the inclusion criteria.The respondents indicated that they had limited knowledge of the legislation and the governing authorities for carers and comforters in their country. Despite respondents indicating that they frequently dealt with carers and comforters in the radiography or the radiation therapy department, only 56% of the respondents were aware of a local policy and just 40% used a consent form. Information was always provided by 57% of respondents. Despite this they indicated that the radiation risk was always understood by only 10% of carers and comforters but was sometimes understood in 71% of cases. Respondents selected information as the best way to achieve better understanding and to improve the management of carers and comforters.
**Conclusion:** To ensure that radiographers and radiation therapists are compliant with legislation and can fulfil their responsibilities to carers and comforters, there needs to be a straightforward process for staff to follow.
**Limitations:** Responses were self-reported and thus professional practices may differ from those reported.
**Ethics committee approval:** University College Cork Social Research Ethics Committee
**Funding for this study:** None.
**Author Disclosures:**
Mark F. F. McEntee: Nothing to disclose
Niamh Moore: Nothing to disclose
Rena Young: Nothing to disclose
Rachel Simon: Nothing to disclose
Andrew England: Board Member: EFRS

### RPS 1214-9

**Implementation of radiographer abnormality detection system (RADS) in the emergency department (ED): a Singapore experience**

J. W. L. Ooi , *F. Luo*; Singapore/SG

**Purpose:** The evolvement of the education curriculum of an undergraduate degree and accreditation from the Allied Health Professional Council (AHPC) of Singapore, topped with shortages of radiologist and inexperienced ED clinicians are catalysts to the development of the radiographer abnormality detection system (RADS) in Singapore. Besides career advancement for radiographers, RADS aims to provide a timely indication to the referrer with a succinct description of the location, type and number of the abnormalities present and is not a replacement for the definitive radiologist report. It is hopeful that this sharing will assist future RADS adoption at other radiology centres.
**Methods or Backround:** This review shares the implementation of RADS at the ED of a public healthcare institution in Singapore. The new service involves a group of qualified radiographers providing round-the-clock image commenting services. The Lewin's four theories (field theory, group dynamics, action research, and the 3-step model) will be used to share our experience for this planned initiative.
**Results or Findings:** Identification of potential barriers helps balance out the driving and restraining forces for an effective change plan. Understanding group dynamics allows change initiators to engage relevant stakeholders and involve them in the planning of the change initiative. Evaluation of the implementation can be carried out with consideration given to feedback received and openness to modification as well as willingness to learn and to enhance performance. The 3-step model focuses on motivating, bringing about and sustaining the change.
**Conclusion:** Diversification of professional skills and practices such as RADS enhances service flexibility and patient centricity. While expectations of health system change vary among stakeholder groups, Lewin found that successful organisational change may be planned.
**Limitations:** The single institutional review may limit generalisation and biases of its findings.
**Ethics committee approval:** No information provided by the submitter.
**Funding for this study:** No funding was received for this study.
**Author Disclosures:**
Jolene Wei Ling Ooi : Nothing to disclose
Felicia Luo: Nothing to disclose

**RPS 1214-10**

**Drivers for low-value imaging in Norway: a qualitative study**

*I. Ø. Brandsæter*, E. R. Andersen, B. M. Hofmann, E. Kjelle; Gjøvik/NO
*(Ingrid.o.brandsater@ntnu.no)*

**Purpose:** The objective of this study was to identify the drivers for low-value imaging in Norway.
**Methods or Backround:** Semi-structured interviews were conducted among representatives from general practitioners, specialists working in hospitals, radiologists, radiographers, managers of radiological departments, and the health authorities. Framework analysis was used to analyse the data.
**Results or Findings:** Twenty-seven participants were included in the study. Several drivers were identified and divided into two themes: healthcare system and culture, and referral and referral assessment. Drivers identified include good access to imaging, economic incentives, communication challenges, defensive medicine, expectations from both patients and referrers, poor referral quality and time constraints. The drivers affect each other and fortify their effect.
**Conclusion:** This study identified several drivers for low-value imaging in Norway. Knowledge about the drivers and their interaction is important when making measures to reduce low-value imaging and free resources for high-value imaging.
**Limitations:** Patients and some referrer groups were not included, thus lacking some important perspectives on drivers of low-value imaging. The interviews were conducted digitally, depriving positive aspects of a face-to-face interview. However, a digital solution gave the participants the opportunity to participate from their workspace.
**Ethics committee approval:** The study was approved by the Norwegian Centre for Research Data: approval number 475812.
**Funding for this study:** This project received financial support from the Research Council of Norway (project number 302503).
**Author Disclosures:**
Ingrid Øfsti Brandsæter: Nothing to disclose
Bjørn Morten Hofmann: Nothing to disclose
Elin Kjelle: Nothing to disclose
Eivind R. Andersen: Nothing to disclose

**10:30-12:00** **Research Stage 1**

### Research Presentation Session: Abdominal Viscera & GI Tract

### RPS 1301

### MRI in malignant and benign anorectal disease

**Moderator**
I. Santiago; Lisbon/PT

**RPS 1301-2**

**Prediction of neoadjuvant chemoradiotherapy response and analysis of tumour microenvironment in locally advanced rectal cancer based on multimodal radiomics**

*Q. Zhao*, H. Zhang, X. Zhao; Beijing/CN
*(zq_pumc@163.com)*

**Purpose:** The study aimed to develop a model predicting the neoadjuvant chemoradiotherapy (NCRT) response in patients with locally-advanced rectal cancer (LARC) based on the baseline MRI radiomics and tumour microenvironment analysis.
**Methods or Backround:** This retrospective study enrolled 180 patients with LARC (126 in the training and 54 in the validation cohort). Two radiologists manually delineated the tumours in T2-weighted images of baseline rectal MRI and extracted 1026 radiomic features from each tumour. The pathological response was evaluated according to the Dowrak tumour regression grade (TRG) system, with good response defined as TRG 3-4 and poor response defined as TRG 0-2. Tumour-stroma ratio (TSR) was estimated, and immunohistochemical analysis of CD3, CD8 and HIF-αwas performed using sections of colonoscopy biopsy specimens. Six machine-learning algorithms were performed to construct models to predict pathological responses, and the performance was compared.
**Results or Findings:** There are 98 good and 82 poor responders. Increased TSR (HR=3.458, P<0.001) was the only clinical risk factor for poor response. A total of nine radiomic features showed significant association with tumour response and were selected to calculate the rad score. The random forest algorithm performed best in the training cohort (AUC: 0.9833, P=0.002) and the validation cohort (AUC: 0.8224, P=0.036). Patients with good response had significantly higher expression of CD3 (P=0.042) and CD8 (P=0.035) and lower expression of HIF-α (P=0.021).
**Conclusion:** We developed a model for NCRT response prediction in LARC patients. The tumour microenvironment plays an essential role in tumour regression.
**Limitations:** The study is limited by being a retrospective study conducted in a single centre.
**Ethics committee approval:** The ethics committee of Cancer Hospital, Chinese Academy of Medical Sciences approved the study and waived informed consent.
**Funding for this study:** The study is funded by the Beijing Hope Run Special Fund of Cancer Foundation of China (LC2021A12) and the Special Research Fund for Central Universities, Peking Union Medical College (3332021098).
**Author Disclosures:**
Xinming Zhao: Nothing to disclose
Qing Zhao: Nothing to disclose
Hongmei Zhang: Nothing to disclose

**RPS 1301-3**

**Feasibility of three-dimension chemical exchange saturation transfer MRI for predicting tumour and node staging in rectal adenocarcinoma: an exploration of the optimal ROI measurement**

*Y. Pei*, X. Wang, W. Li, W. Liu; Changsha/CN
*(tjpyg027@163.com)*

**Purpose:** The study aimed to investigate the feasibility of predicting tumour (T) and node (N) staging with amide proton transfer weighted-signal intensity (APTw-SI) and magnetisation transfer (MT) for rectal adenocarcinoma (RA) using a three-dimensional fast spin echo-based chemical exchange saturation transfer (3D-CEST) technique and to explore the optimal ROI measurement for determinating the TN staging in RA patients.
**Methods or Backround:** Fifty-eight RA patients confirmed with pathologically TN staging underwent 3D-CEST and DWI. APTw-SI, MT and ADC values were measured using three ROI approaches (ss-ROI: single slice ROI; ts-ROI: three-slice ROI; and wt-ROI: whole-tumour ROI) on CEST and DW images to analyse the performance of TN staging (T staging: T1-2 vs T3-4; N staging: N- vs N+) with the areas under the curve (AUC) and determine the optimal ROI strategy.
**Results or Findings:** There were no significant differences in the TN staging with APTw-SI and ADC (all P>0.05) but MT values (all P<0.05) using three different kinds of ROIs. Furthermore, AUCs of MT values with ss-ROI were 0.860 (95% CI: 0.743 - 0.937) for T staging and 0.852 (95% CI: 0.735 - 0.932) for N staging, showing similar performance with ts-ROI (T staging: 0.856 [95% CI: 0.739 - 0.934], N staging: 0.831 [95% CI: 0.710 - 0.917]) and wt-ROI (T staging: 0.833 [95% CI: 0.712 - 0.918], N staging: 0.848 [95% CI: 0.729 - 0.929]) (all p>0.05).
**Conclusion:** MT values with ss-ROI have a good predictive performance on TN staging for RA patients, which is easier implemented to ts-ROI and wt-ROI on 3D-CEST and could be used in future clinical diagnosis.
**Limitations:** The study is limited by the relatively small sample size.
**Ethics committee approval:** The study is approved by the Medical Ethics Committee of Xiangya Hospital, Central South University (approval number: 2021111101).
**Funding for this study:** The study is funded by the Natural Science Foundation of Hunan Province (grant number: 2022JJ30950, Changsha, China).
**Author Disclosures:**
Wenguang Liu: Nothing to disclose
Xiao Wang: Nothing to disclose
Wenzheng Li: Nothing to disclose
Yigang Pei: Nothing to disclose

**RPS 1301-4**

**Diagnostic accuracy of MRI in the locoregional staging of the locally advanced rectal cancer (LARC)**

*M. Trovato*, R. Faletti, E. Territo, S. Vola, D. Velkobroda, M. Gatti, C. Dianzani, G. Salmè, P. Fonio; Turin/IT
*(dott.martinatrovato@gmail.com)*

**Purpose:** This research aimed to evaluate the diagnostic accuracy of MRI in the locoregional staging of the LARC by comparing it with anatomopathological reports and analysing the agreement between operators in the reporting.
**Methods or Backround:** Locoregional staging through MRI imaging plays a crucial role in locally advanced rectal cancer (LARC) since therapeutic decisions, and patient prognosis depends on it. 189 patients diagnosed with LARC underwent chemoradiotherapy followed by surgery at the A.O.U. City of Health and Science of Turin were selected. The final sample included 65 patients who underwent MRI staging and restaging and have been followed up for a period of 30 to 60 months.
**Results or Findings:** The diagnostic accuracy given by comparison with pathological analysis was found to be high for the dichotomous variables (vascular and mesorectal invasion, satellite tumour nodes and metastases)

and lower for the non-dichotomous variables (local extension and lymph node involvement). The RT-IC interval is a risk factor for exitus (p=0.0008) and for local recurrence (p=0.02). The risk of exitus (22-23.6), calculated with the Kaplan Meier curve, the Cox regression and the EPPY calculation, and the risk of local recurrence (4.3-5) were obtained for the RT-IC interval> 12 weeks. The "downstaging" variable has a low predictive power of exitus (AUC=0.69) and no predictive power of local recurrence. If both "RT-IC interval> 12 weeks" and "downstaging" risk factors are present, the risk of death rises to 33 (5-206).
**Conclusion:** MRI is a fundamental exam for the locoregional staging of LARC, and the evaluation must be performed following dedicated protocols by expert operators. The results of the report guide the therapeutic choices and influence the prognosis. The study confirmed that the RT-IC interval must be less than 12 weeks to avoid adverse events.
**Limitations:** No limitations were identified.
**Ethics committee approval:** No information provided by the submitter.
**Funding for this study:** No funding was received for this study.
**Author Disclosures:**
Chiara Dianzani: Nothing to disclose
Giulio Salmè: Nothing to disclose
Marco Gatti: Nothing to disclose
Dani Velkobroda: Nothing to disclose
Martina Trovato: Nothing to disclose
Riccardo Faletti: Nothing to disclose
Elena Territo: Nothing to disclose
Paolo Fonio: Nothing to disclose
Simone Vola: Nothing to disclose

### RPS 1301-5

**Training and external validation of pre-treatment FDG PET-CT-based models for outcome prediction in anal squamous cell carcinoma**
*R. Frood*[1], J. Mercer[2], P. Brown[3], A. Appelt[1], H. Mistry[2], R. Kochhar[2], A. Scarsbrook[1]; [1]Leeds/UK, [2]Manchester/UK, [3]York/UK

**Purpose:** The incidence of anal squamous cell carcinoma (ASCC) is increasing worldwide, with a significant proportion of patients treated with curative intent having a recurrence. The ability to accurately predict progression-free survival (PFS) and overall survival (OS) could help guide personalised treatment strategies. The study aimed to train and externally test radiomic/clinical feature-derived time-to-event prediction models.
**Methods or Backround:** Consecutive patients with ASCC treated with curative intent at two large tertiary referral centres undergoing baseline FDG PET-CT were included. Radiomic feature extraction was performed using LIFEx software on pre-treatment PET-CT. Two distinct predictive models for PFS and OS were trained and tuned at each centre, with the best-performing model externally tested on the other centres' patient cohorts.
**Results or Findings:** 187 patients were included from centre 1 (mean age 61.6±11.5 years, median follow-up 30 months, PFS events=57/187, OS events=46/187), and 257 patients were included from centre 2 (mean age 62.6±12.3 years, median follow-up 35 months, PFS events=70/257, OS events=54/257). The predictive ability of PET-CT-derived radiomic features diminished when the influence of metabolic tumour volume (MTV) was accounted for. The best-performing model for PFS and OS was achieved using a cox regression model based on age and MTV with a training c-index of 0.7 and an external testing c-index of 0.7 (standard error=0.4).
**Conclusion:** An externally validated model combining patient age and MTV shows potential for predicting PFS and OS in ASCC patients.
**Limitations:** The study is limited by being a retrospective study with only two centres included.
**Ethics committee approval:** No information provided by the submitter.
**Funding for this study:** No specific funding was received for this study.
**Author Disclosures:**
Joe Mercer: Nothing to disclose
Rohit Kochhar: Nothing to disclose
Hitesh Mistry: Nothing to disclose
Peter Brown: Nothing to disclose
Ane Appelt: Nothing to disclose
Andrew Scarsbrook: Nothing to disclose
Russell Frood: Nothing to disclose

### RPS 1301-6

**Largest mesorectal lymph node evaluation for detection of nodal metastases in locally advanced rectal cancer after chemoradiotherapy**
*G. Cabrelle*[1], A. Schillaci[1], C. Zanon[1], F. Vernuccio[1], A. Pepe[1], E. Quaia[1], S. Pucciarelli[1], C. Campi[2], F. Crimì[1]; [1]Padua/IT, [2]Genova/IT

**Purpose:** The research aimed to compare the accuracy of different dimensional criteria of the largest mesorectal lymph node in detecting regional nodal metastases after chemoradiotherapy (CT-RT) in patients affected by locally advanced rectal carcinoma (LARC).
**Methods or Backround:** 139 patients with LARC having MRI-scan before and after CT-RT were retrospectively enrolled. MRI after CT-RT were evaluated independently by two radiologists, blinded to the histological results. Short-, long-axis and third craniocaudal axis of the largest mesorectal lymph node were measured. The number of loco-regional lymph nodes with a short axis≥5mm (ESGAR method) was recorded. Patients were divided into the first cohort of 100 cases and a validation cohort of 39 patients. At first, an agreement test was performed for each type of measurement. After that, using as reference standard the histopathology, ROC curves were drawn for the short-, long-axis and volume of the largest lymph node to identify the best cut-off at Youden's index analysis. The accuracy of these two methods and of the ESGAR method was compared with the McNemar test, considering histopathological results. The accuracy results were retested on the validation cohort.
**Results or Findings:** On the 100 patients cohort, the agreement among readers was high for three methods (K-long-axis=0.80; K-volume=0.85; K-ESGAR=0.88) and low for the short-axis (K=0.11). With a cut-off of 95mm3 for the volume and of 5mm for the long-axis, accuracies of 69% and 63% were obtained, compared to the 65% accuracy of the ESGAR method. At the McNemar test, no significant differences were found. In the validation cohort, the data of accuracy were confirmed for the different methods (77%,74% vs 67%).
**Conclusion:** The largest lymph node's volume or long-axis after CT-RT in LARC is not inferior to the ESGAR method and could provide a faster evaluation for nodal status.
**Limitations:** The study has a limitation by being a single-centre study.
**Ethics committee approval:** This study was approved by the ethics committee.
**Funding for this study:** No funding was received for this study.
**Author Disclosures:**
Federica Vernuccio: Nothing to disclose
Alessio Schillaci: Nothing to disclose
Alessia Pepe: Nothing to disclose
Giulio Cabrelle: Nothing to disclose
Salvatore Pucciarelli: Nothing to disclose
Chiara Zanon: Nothing to disclose
Filippo Crimì: Nothing to disclose
Emilio Quaia: Nothing to disclose
Cristina Campi: Nothing to disclose

### RPS 1301-7

**The role of deep learning in the evaluation of response to neoadjuvant chemoradiotherapy with MRI in rectal cancer**
*E. Cingoz*, G. Ertaş, G. Kaval, S. Azamat, Ş. Karaman, N. Dagoglu Sakin, B. Kulle, M. Buyuk, M. G. Kartal; Istanbul/TR
*(edacanipek@hotmail.com)*

**Purpose:** Preoperative prediction of complete response to neoadjuvant chemoradiotherapy in patients with rectum cancer is of paramount importance due to the possibility of non-operative management, averting a major surgery with certain complications. A prediction tool with high accuracy is therefore required.
**Methods or Backround:** Patients with Stage 2 and 3 rectum cancer who received neoadjuvant chemoradiotherapy were included in the study. Each tumour was manually segmented, followed by the scoring of pre-treatment and post-treatment magnetic resonance (MR) images to detect the tumour regression grade. The data from the segmentation were processed with deep learning algorithms to obtain a model that may predict complete response with high accuracy and compared with the diagnostic power of MR tumour regression grade (mrTRG).
**Results or Findings:** 59 patients were enrolled (training dataset: 47 cases, test dataset: 12 cases). Two novel deep-learning models were developed. The performance of the models revealed satisfactory results, similar to the performance of mrTRG with an accuracy approximating 90%.
**Conclusion:** Deep learning is a promising tool to predict complete responders among patients with rectal cancer receiving neoadjuvant chemoradiotherapy with similar accuracy to mrTRG. Large datasets are needed to assess the potential for clinical use of deep-learning algorithms. Thus, the evaluation of rectal MRI supported by deep-learning models may have a high potential as an imaging biomarker of complete clinical response. Such a technique can spare patients the "watch and wait" protocol and may also enable them to avoid a major surgery with a substantial rate of morbidity and even mortality.
**Limitations:** Limitations of the study include its retrospective design and the relatively small sample size.
**Ethics committee approval:** The ethics committee approval can be found under file number 2022/73.
**Funding for this study:** No funding was received for this study.
**Author Disclosures:**
Nergiz Dagoglu Sakin: Nothing to disclose
Gökhan Ertaş: Nothing to disclose
Burak Kulle: Nothing to disclose
Merve Gulbiz Kartal: Nothing to disclose

Eda Cingoz: Nothing to disclose
Şule Karaman: Nothing to disclose
Sena Azamat: Nothing to disclose
Gizem Kaval: Nothing to disclose
Melek Buyuk: Nothing to disclose

### RPS 1301-8

**Can T2W texture analysis of the primary tumour differentiate nodal status after neoadjuvant chemoradiation in rectal cancer?**

D. van der Reijd[1], R. A. P. Dijkhoff[2], S. G. Drago[3], J. J. van Griethuysen[1], D. M. J. Lambregts[1], F. C. Bakers[2], R. G. H. Beets-Tan[1], *M. Maas*[1]; [1]Amsterdam/NL, [2]Maastricht/NL, [3]Monza/IT
*(moniquemaas@live.nl)*

**Purpose:** The morphological assessment of lymph nodes after chemoradiation remains a challenge. The aim of this study is to investigate if T2 weighted (T2W) texture analyses of the primary tumour derived from baseline and restaging MRI, can differentiate ypN0 from ypN+ after neoadjuvant chemoradiation (CRT) in rectal cancer.
**Methods or Backround:** 170 rectal cancer patients underwent baseline and restaging MRI after neoadjuvant CRT. A volume of interest was manually delineated around the primary tumour on pre-CRT T2W-MRI and around the tumour remnant or fibrotic scar on post-CRT T2W-MRI. 10 histogram texture features were extracted with the use of PyRadiomics, and delta features were calculated by comparing pre- with post-CRT features. Histopathological evaluation after resection classified patients as ypN0 or ypN+. Patients without signs of recurrence after >24 months of follow-up in the Watch&Wait program were also classified as ypN0. Means were compared with independent t-tests and a significance level of $p \leq 0.05$ was applied.
**Results or Findings:** 42/170 patients (25%) had ypN+. On pre-CRT MRI, only skewness was significantly higher in ypN+ patients (1.03 vs 0.776, p=0.028). On post-MRI, 5/10 features were significantly higher in ypN+, including Mean (5.29 vs 4.19, p=0.005), Median (4.97 vs 3.86,p=0.005), Minimum (-0.756 vs -1.31,p=0.014), 10th percentile (2.24 vs 1.26,p=0.001) and 90th percentile (8.70 vs 7.47,p=0.022). For the delta features (absolute difference and percentages), 7/20 showed a significantly smaller difference in ypN+ compared to ypN0. Additionally, Delta-Skewness was positive in ypN0 and negative in ypN+ (0.217 vs -0.0969,p=0.035).
**Conclusion:** T2W texture analysis derived from post-CRT MRI seem to have a higher discriminative value for ypN-status compared to pre-CRT MRI. Texture features relating to signal intensities had higher values in ypN+ patients in the primary tumour after CRT.
**Limitations:** Pre-CRT nodal status was not considerate.
**Ethics committee approval:** No information provided by the submitter.
**Funding for this study:** No funding was received for this study.
**Author Disclosures:**
Denise van der Reijd: Nothing to disclose
Monique Maas: Nothing to disclose
Joost J.M. van Griethuysen: Nothing to disclose
Silvia Girolama Drago: Nothing to disclose
Frans C.H. Bakers: Nothing to disclose
Doenja Marina Johanna Lambregts: Nothing to disclose
Regina G. H. Beets-Tan: Nothing to disclose
Rebecca Alexandra Paola Dijkhoff: Nothing to disclose

### RPS 1301-9

**Tumour regrowth in patients under the “watch-and-wait” programme in rectal cancer, the radiology view: our experience in ten years**

*L. M. Florentin*, G. Dulcich, G. Rossi, J. L. Savluk; Buenos Aires/AR
*(lautaro.florentin@hospitalitaliano.org.ar)*

**Purpose:** The study aimed to review the "watch and wait” (WW) programme from a radiological point of view and to describe tumoral regrowth patterns in patients under WW studied with magnetic resonance imaging (MRI) between January 1, 2012, to the present.
**Methods or Backround:** MRI has become a fundamental tool in diagnosing and following up on rectal cancer patients. Initial staging is aimed at detecting patients with locally advanced tumours. Those who achieve a complete response (CR) after neoadjuvant can decide for WW. WW includes digital rectal examination, endoscopy and MRI. 95% of tumour regrowth develops at the tumoral bed. It includes recurrence within the wall and/or mesorectum, iliac, and obturator lymph nodes. High-resolution T2 and diffusion with ADC map are essential for early regrowth detection. 41 patients with staging MRI and at least one MRI after CR were included.
**Results or Findings:** 31 patients presented low rectal cancer (75,6%). Nine had a middle rectal tumour and one in the anal canal. 13 patients (31,7%) were staged as T3: 57,1% were T3b; 12 patients (29%) as T2; 3 (7%) as T1; and 2 were T4. Lymph nodes were involved in 53,6% of cases. Most patients (75,6%) didn’t have radiological regrowth evidence. 10 had tumour regrowth. All were presented within the first two years after CR. One-third was among the first six months and others between 18 to 24 months. Regrowth was most detected at the rectal wall (mural) (70%), one patient showed extramural (mesorectal) regrowth, and two had both. Seven patients could access a surgical treatment. Transanal resection and ultralow anterior resection were the most used (three patients each). One patient undergoes Miles’ procedure.
**Conclusion:** MRI is a fundamental tool in rectal cancer. Radiologists should recognise different patterns of tumour regrowth.
**Limitations:** Not applicable.
**Ethics committee approval:** The study was approved by the Institutional Ethics Committee.
**Funding for this study:** No funding was received for this study.
**Author Disclosures:**
Lautaro Manuel Florentin: Nothing to disclose
Gustavo Rossi: Nothing to disclose
Jesica Lorena Savluk: Nothing to disclose
Gonzalo Dulcich: Nothing to disclose

### RPS 1301-10

**The clinical value of MR defecography: what additional knowledge is given by the radiologist to the surgeon? Preliminary results**

*A. Colarieti*, A. Stuto, F. Sardanelli; Milan/IT
*(anna.colarieti@gmail.com)*

**Purpose:** To measure the clinical value of magnetic resonance defecography (MRD) by quantifying the satisfaction of the surgeon before and after surgery.
**Methods or Backround:** Sixty-six patients with pelvic floor disorders, aged 27-79 years (60 ± 11, mean ± standard deviation), 55 females and 11 males, underwent 1.5-T MRD from 2020 to 2022. Examinations were performed using surface, phased array coils, at rest (axial/sagittal/coronal T2-weighted sequences), and during squeezing, straining, and evacuation (dynamic sagittal T2-weighted sequences); the original prospective report by the radiologist on duty was taken into consideration in this analysis. One radiologist evaluated the MRD and reported them according to usual clinical practice. The chief of colorectal surgery department assessed his satisfaction regarding the quality of the images (1=insufficient, 2=sufficient, 3=good, 4=optimal) and, in the cases that underwent surgery (n=22) the grade of concordance with the surgical evidence (-2=useless, 1=partially useless, 0=indifferent, 1=useful, 2=extremely useful).
**Results or Findings:** The radiologist was able to evaluate the images and determine an MRD-based diagnosis for 64/66 patients (97%); for 2/66 patients (3%) a diagnosis was not provided due to poor-quality images caused by low patient's compliance. For 59/64 patients (92%), the radiological diagnosis fitted with the clinical one. According to the surgeon, image quality of the 66 examinations was: optimal for 31 (47%), good for 15 (23%), sufficient for 11 (16%), and insufficient for 9 (14%). Surgery was performed for 22/66 patients. For 19/22, MRD was judged to be extremely useful and concordant with the surgical outcomes (85%), for 1/22 useful (5%), for 1/22 indifferent (5%), and for 1/22 useless (5%).
**Conclusion:** MRD provides images with optimal or good quality in the majority of cases and demonstrates a high capability to identify disorders of the pelvic floor, adding value in the preoperative surgical setting.
**Limitations:** The limitations of this study are that it is a single-institution study.
**Ethics committee approval:** Ethics committee request in preparation.
**Funding for this study:** No funding was received for this study.
**Author Disclosures:**
Anna Colarieti: Nothing to disclose
Francesco Sardanelli: Nothing to disclose
Angelo Stuto: Nothing to disclose

### RPS 1301-11

**The additive value of diffusion tensor imaging in the determination of peri-anal fistula activity**

M. M. M. Hasan, *M. Elmansy*, M. A. Gad, M. M. Mahmoud, M. Elhawary; Mansoura/EG

**Purpose:** The study aimed to elucidate the role of diffusion tensor magnetic resonance imaging (DT-MRI) in evaluating the activity state of the perianal fistula.
**Methods or Backround:** We retrospectively reviewed the data of 30 patients diagnosed with perianal fistula who underwent an MRI diffusion study before the surgical intervention. According to the presence of pus intraoperatively, patients were allocated into two groups: the active group (10 patients) and the inactive group (20 patients). Apparent diffusion coefficient (ADC) and fractional anisotropy (FA) were calculated for the fistulous area and the normal surrounding puborectalis as a control area.
**Results or Findings:** FA and ADC measurements showed a significant decrease in association with the active disease when measured at the fistula site, and this was not observed in the normal surrounding tissues. The area under the curve was 0.985 and 0.730 for the ADC and FA, respectively, when cut-off values of 1 x 10-3 mm2/sec and 0.621 were applied, respectively. Adding the FA to the ACD increased the specificity to 95 % in determining the active fistula.

**Conclusion:** DT-MRI could be used as a reliable diagnostic tool to differentiate patients with active perianal fistula disease from inactive ones and to predict the postoperative outcome.
**Limitations:** This study is limited by a relatively small sample size.
**Ethics committee approval:** The ethics committee approval can be found under file number R.21.01.1179.R1.
**Funding for this study:** Not applicable.
**Author Disclosures:**
Mohamed Mostafa Mahmoud: Nothing to disclose
Mohamed Mohsen Mohamed Hasan: Nothing to disclose
Mohammed Elhawary: Nothing to disclose
Mona Ali Gad: Nothing to disclose
Mostafa Elmansy: Nothing to disclose

### RPS 1301-12

**Preoperative magnetic resonance imaging of anal fistulas with scrotal extension**
D. Vo Tan, *C. C. Phan*, L. Nguyen, T. Ngjuyen, N. Nguyen, T. Nguyen;
Ho Chi Minh City/VN
*(chien.pc@umc.edu.vn)*

**Purpose:** We decide to conduct this magnetic resonance imaging (MRI) study to elucidate more clearly the characteristics and rules applicable to anal fistulas with scrotal extensions on MRI and correlate with the surgical findings.
**Methods or Backround:** We conducted a retrospective study in 150 consecutive patients with anal fistulas extending into the scrotum, who were diagnosed and underwent surgery at University Medical Center, Ho Chi Minh City, between 2017 and April 2022. MRI findings were evaluated and compared with surgical findings using Cohen's kappa coefficient (k) with a 95% confidence interval.
**Results or Findings:** 150 patients (mean age 37.6±10.9 years) with 166 fistulas, including 150 anal fistulas with scrotal extension. Most fistulas were low transsphincteric fistulas, with 120 patients (80.0%). There was a strong agreement for primary tract classification and detecting the location of internal openings between MRI and surgical findings with k=0.83 (0.78–0.87) and k=0.89 (0.85–0.93) ($p<0.001$), respectively. There is a significant correlation between the location of internal openings and the type of fistula ($p<0.05$). Low transsphincteric fistulas were predominant in the anterior group (103/122 patients vs 10/19 patients), while in the posterior group, it was more common in the high transsphincteric fistulas (7/19 patients vs 14/122 patients), and the intersphincteric fistulas (1/19 patients vs. 5/122 patients); and the suprasphincteric fistulas were only seen in the posterior group (two patients).
**Conclusion:** Anal fistula with scrotal extension are mostly low transsphincteric fistulas with an anterior internal opening which was an exception to Goodsall's rule.
**Limitations:** The study is limited by its retrospective design.
**Ethics committee approval:** The study received the ethics committee's approval.
**Funding for this study:** No funding was received for this study.
**Author Disclosures:**
Thien Ngjuyen: Nothing to disclose
Linh Nguyen: Nothing to disclose
Chien Cong Phan: Nothing to disclose
Duc Vo Tan: Nothing to disclose
Truc Nguyen: Nothing to disclose
Nam Nguyen: Nothing to disclose

**10:30-12:00 Research Stage 2**

### Research Presentation Session: Interventional Radiology

### RPS 1309

### Ablative technique and treatment assessment

**Moderator**
D. Filippiadis; Athens/GR
*(dfilippiadis@yahoo.gr)*

### RPS 1309-2

**Retrospective single-centre study to evaluate the use of targeted radiofrequency ablation (t-RFA) followed by vertebroplasty in difficult-to-reach metastatic spinal lesions**
*C. Pusceddu*; Cagliari/IT
*(clapusceddu@gmail.com)*

**Purpose:** The study aimed to assess the effectiveness of a navigational radiofrequency ablation device with concurrent vertebral augmentation in treating posterior vertebral body metastatic lesions, which are technically challenging to access. Primary outcomes were the evaluation of pain palliation and radiologic assessment of local tumour control.
**Methods or Backround:** Thirty-five patients with 41 vertebral spinal metastases involving the posterior vertebral body underwent computed tomography-guided percutaneous targeted radiofrequency ablation with a navigational radiofrequency ablation device associated with vertebral augmentation. Twenty-one patients (60%) had one or two metastatic lesions (Group A), and fourteen (40%) patients had multiple (>2) vertebral lesions (Group B). Changes in pain severity were evaluated by a visual analogue scale (VAS). Metastatic lesions were evaluated in terms of local radiological control.
**Results or Findings:** The procedure was technically successful in all the treated vertebrae. Among the symptomatic patients, the mean VAS score dropped from 5.7(95% CI 4.9-6.5) before tRFA and to 0.9(95% CI 0.4-1.3) after tRFA ($p < 0.001$). The mean decrease in VAS score between baseline and one-week follow-up was 4.8 (95% CI 4.2-5.4). VAS decrease over one week and one year following radiofrequency ablation was similar, suggesting that pain relief was immediate and durable. Neither patients with 1-2 vertebral metastases, nor those with multiple lesions, showed radiological signs of local progression or recurrence of the tumour in the index vertebrae during a median follow-up of 19 months (4-46 months) and 10 months (4-37 months), respectively.
**Conclusion:** t-RFA device and vertebral augmentation can be used in obtaining local tumour control with immediate and durable pain relief, providing effective treatment in the multimodality management of difficult-to-reach spinal metastases.
**Limitations:** This study has several limitations due to its retrospective nature and the small number of patients.
**Ethics committee approval:** The IRB of the cancer hospital approved this study.
**Funding for this study:** No funding was provided for this study.
**Author Disclosures:**
Claudio Pusceddu: Nothing to disclose

### RPS 1309-3

**Safety and efficacy of interventional radiology management of osteoblastomas: ten years of experience using RFA and MRGFUS**
*J. Daffinà*, F. Arrigoni, L. Zugaro, C. Masciocchi; L'Aquila/IT
*(daffinajulia@gmail.com)*

**Purpose:** Osteoblastoma (OB) is a rare benign bone tumour typically observed in young patients, which frequently causes discomfort and pain. Minimally invasive interventional radiology techniques such as CT-guided RFA and MRgFUS are now highly recommended for a vast scale of lesions. It frequently involves the spine, thus requiring highly complex surgery.
**Methods or Backround:** We retrospectively selected 23 osteoblastoma treated in our department. Of these 23, nine were treated using MRgFUS, and 14 with CT-guided RFA. Because of the necessity of a histological diagnosis, a bone biopsy was executed contextually to the ablation procedure, whereas when MRgFUS was chosen, the biopsy preceded treatment.
**Results or Findings:** Complete success in terms of pain relief was achieved in all patients. Additional treatments were not required in any patients. There were no complications. During follow-up, neither complications nor pathological findings related to the treatment were observed.

**Conclusion:** Considering the high success rate, the reduced hospital stay, and the lower risk of complications, these minimally invasive procedures should be suggested as an alternative to surgery in symptomatic patients that want to opt for an alternative procedure.
**Limitations:** We will further extend our sample size by prospectively including patients.
**Ethics committee approval:** No information provided by the submitter.
**Funding for this study:** No funding was received for this study.
**Author Disclosures:**
Francesco Arrigoni: Nothing to disclose
Julia Daffinà: Nothing to disclose
Carlo Masciocchi: Nothing to disclose
Luigi Zugaro: Nothing to disclose

### RPS 1309-4

**12-month clinical outcomes of MRI-guided transurethral ultrasound ablation for localised prostate cancer**

*E. H. M. Yli-Pietilä*[1], M. Anttinen[1], P. Mäkelä[1], P. Nurminen[1], P. Doerwald[2], H. E. Pärssinen[1], P. Taimen[1], R. Blanco Sequeiros[1], P. J Boström[1]; [1]Turku/FI, [2]Hamburg/DE
*(eemil.yli-pietila@tyks.fi)*

**Purpose:** MRI-guided transurethral ultrasound ablation (TULSA) is a novel minimally invasive therapy option for the treatment of localised prostate cancer (PCa). The objective of this study was to evaluate the safety and efficacy of TULSA for treating organ-confined PCa.
**Methods or Backround:** This prospective, single-centre, phase-two-study (NCT03814252) enrolled men with MRI-visible, biopsy-proven clinically-significant PCa (CSPCa), high-volume ISUP 1 or ≥ISUP 2 (high-volume ISUP 1 as determined on biopsies [>2 positive cancer core or ≥50% cancer in a core]). Patients received either whole-gland or focal TULSA, depending on their preference and disease characteristics. Adverse events (AEs, Clavien-Dindo), PSA, uroflowmetry, and QoL-questionnaires were recorded at three-month intervals, biopsies and mp-MRI at six and 12 months.
**Results or Findings:** 60 TULSA treatments were performed in men with ISUP 1/2/3/4 12%/62%/18%/8%, median [IQR] age 66 yr [61–72], PSA 6.9 ng/ml [4.5–9.7], volume 31 ml [25–41]. A substantial proportion had unfavourable intermediate-to-high risk but refused/were ineligible for surgery/radiation. As primary treatment, 13 (22%) received whole-gland ablation. AEs included 15 Grade 2 and 3 Grade 3. 15 repeat treatments were performed. 45 patients have completed 1-year follow-up, and 39 completed 1-year biopsy. At 1 year, median (IQR) PSA was 0.81 ng/ml [0.5-1.2], 28/39 (72%) patients were free of in-field CSPCa, and 5/39 (13%) had out-of-field CSPCa. The median average flow rate and Qmax remained stable, and post-void residual volume improved from 51 ml [14–103] to 24 ml [0–76]. 15/21 men (71%) remained potent at 12 months (IIEF-Q2≥2). Median IPSS and IPSS-QoL scores improved from 6 [4–13] and 2 [1-3] at baseline to 5 [2–9] and 1 [0-2] by 1 year.
**Conclusion:** TULSA offers promising oncological outcomes for treating localised PCa with minimal impact on patients' quality of life.
**Limitations:** The study is limited by small sample size and short-term follow-up.
**Ethics committee approval:** The ethics committee approval was granted, and informed consent was obtained from all study participants.
**Funding for this study:** The study was investigator-initiated.
**Author Disclosures:**
Heikki Elias Pärssinen: Nothing to disclose
Peter J Boström: Speaker: Speaker at a webinar sponsored by Profound Medical Inc
Pouya Doerwald: Employee: Employed at Profound Medical and receives salary wage
Pertti Nurminen: Nothing to disclose
Eemil Hannu Mikkel Yli-Pietilä: Nothing to disclose
Pietari Mäkelä: Nothing to disclose
Mikael Anttinen: Speaker: Honorarium speaker at a webinar sponsored by Profound Medical Inc
Pekka Taimen: Nothing to disclose
Roberto Blanco Sequeiros: Nothing to disclose

### RPS 1309-5

**Tips and tricks for interventional ablation treatment (RFA and MRgFUS) of osteoid osteomas in uncommon sites**

*J. Daffinà*, F. Arrigoni, L. Zugaro, C. Masciocchi; L'Aquila/IT
*(daffinajulia@gmail.com)*

**Purpose:** Over the past ten years, we have treated over 130 osteoid osteomas (OO) cases with interventional radiology procedures. Most OO have typical appearance and location (proximal epiphysis and diaphysis of the femur, tibial and humeral diaphysis, etc.); in sporadic cases, they can occur in some uncommon locations that make both diagnosis and treatment challenging. The purpose of our study is to review out-of-routine cases and tips and tricks for avoiding common pitfalls.
**Methods or Backround:** Retrospective review of all the treatment ablations (RFA and MRgFUS) performed in our institution for painful osteoid osteomas (OO) through the PACS system. We documented all the data, including complications, hospitalisation, procedural time and relapse.
**Results or Findings:** 11 cases of unusual OO locations were observed in our experience; they include the extremities' small bones, the patella and the scapula. The treatment of these atypical locations is linked to several difficulties compared to long bones diaphyseal OOs, in particular, due to the reduced stability of the needle within the lesion as well as the positioning of the patient. However, the procedural time was not significantly longer than the routine ones. Furthermore, previous surgery and the presence of bone grafts can alter RFA thermal dynamics, thus increasing the complexity of the procedure. Their superficial location and the presence of saddle or gliding joints make it arduous to maintain the RFA needle stable to reduce its movement during treatment. Moreover, a superficial location can require an "hydrodissection" to protect the skin from thermal injury. No major complications were recorded.
**Conclusion:** With specific procedural precautions, RFA and MRgFUS are confirmed as effective and safe treatments of OO, even in uncommon and challenging locations.
**Limitations:** No limitations were observed.
**Ethics committee approval:** No information provided by the submitter.
**Funding for this study:** No funding was provided for this study.
**Author Disclosures:**
Francesco Arrigoni: Nothing to disclose
Julia Daffinà: Nothing to disclose
Carlo Masciocchi: Nothing to disclose
Luigi Zugaro: Nothing to disclose

### RPS 1309-6

**Percutaneous thermal ablation with high-power microwave (MWA) of small renal masses (SRMs): feasibility and safety**

*L. V. Sciacqua*, A. Vanzulli, V. Ascenti, G. Carrafiello; Milan/IT
*(lucilla.sciacqua@unimi.it)*

**Purpose:** The study aimed to evaluate the feasibility and safety of high-power microwave ablation (MWA) in patients with SRMs.
**Methods or Backround:** We retrospectively evaluated 14 patients aged 61-83 with SRMs who underwent MWA at our institute in the last two years because they were deemed ineligible for nephrectomy (which still represents the gold standard for renal cancer) due to their advanced age or chronic renal failure. Monorenal patients with Von-Hippel-Lindau syndrome (VHL) were also enrolled in the study. The procedure is performed under sedation and local anaesthesia with lidocaine. The power of the microwave is 150W. Clinical response was evaluated one month after treatment with either computed tomography (CT) or magnetic resonance imaging (MRI).
**Results or Findings:** The mean lesion size is 3.3cm (range 3-3.8cm) with an average ablation time of 4min 22sec (range 2-9min). The proper positioning of the treatment antenna establishes technical success, achieved in 100% of cases. In all patients, areas of necrosis imputable to complete ablation were observed at follow-up scans performed immediately after treatment and at a one-month timepoint. No significant complications have been reported. Peri-procedural complications (i.e., perilesional haematomas) were reported in two out of 14 patients immediately after needle insertion and 21 days after the procedure, respectively. All these minor complications are classified as grade 1 in the CIRSE classification of adverse events.
**Conclusion:** Many studies have demonstrated that percutaneous ablation is safe and effective for treating localised renal tumours and allows for significant sparing of healthy renal parenchyma compared to PN. Nonetheless, further investigation is needed to confirm its feasibility and efficacy for treating renal cancer with high-power MWA.
**Limitations:** The study is limited by the number of patients.
**Ethics committee approval:** Not applicable.
**Funding for this study:** No funding has been required.
**Author Disclosures:**
Lucilla Violetta Sciacqua: Author: Nothing to discole
Velio Ascenti: Author: Nothing to disclose
Andrea Vanzulli: Author: Nothing to disclose
Gianpaolo Carrafiello: Author: Nothing to disclose

### RPS 1309-7

**Percutaneous cryoablation of hepatic tumours: indications, effectiveness and safety**

*L. Cea*, G. Pacella, C. Altomare, F. Andresciani, C. Bernetti, M. T. Bitonti, U. Ferrari, B. Beomonte Zobel, R. F. Grasso; Rome/IT
*(laura.cea@unicampus.it)*

**Purpose:** The study aimed to evaluate the efficacy and safety of percutaneous cryoablation for the treatment of primitive and secondary liver lesions.
**Methods or Backround:** Ten percutaneous cryoablation procedures, with ultrasound and/or CT guidance, were retrospectively examined. Cryoablation was chosen in case of the lesion in proximity to vulnerable anatomical structures using, in some cases, protection techniques (hydro-dissection and aero-dissection). Average volumes (cm³) of the ice balls and the necrotic cavity of the first and last available CT scan for each patient were calculated for the lesions treated using CT images, and comparisons were performed. The average difference between standard deviation and percentage difference was calculated for each comparison; the local rate of disease progression (LTP) was also evaluated.
**Results or Findings:** Ten lesions were included, selected from primary and secondary tumours. The average ice-ball volume was 21.2±21.7 cm³ (range 4.5-76.7 cm³). At the first follow-up, the average volume of the necrotic cavity was 11.8±12.4 cm³. At the last imaging follow-up examination, the mean volume of the necrotic cavity of ablate lesions was 3.2±4.2 cm³. The average difference between the volume of the necrotic cavity at the first follow-up and at the last follow-up was 8.5±4.1 cm³ with a reduction %ΔV of 72.5%. In an average follow-up period of 14.6±15.1 months, local disease progression was observed in only 2 out of 10 patients (20%). No complications were observed in all procedures.
**Conclusion:** In our experience, cryoablation can be considered an effective and safe percutaneous ablation technique for treating primary or secondary liver malignancies.
**Limitations:** Patient selection is fundamental. Further prospective studies with a larger number of patients are necessary.
**Ethics committee approval:** No information provided by the submitter.
**Funding for this study:** No funding was received for this study.
**Author Disclosures:**
Bruno Beomonte Zobel: Nothing to disclose
Rosario Francesco Grasso: Nothing to disclose
Maria Teresa Bitonti: Nothing to disclose
Ugo Ferrari: Nothing to disclose
Flavio Andresciani: Nothing to disclose
Giuseppina Pacella: Nothing to disclose
Caterina Bernetti: Nothing to disclose
Carlo Altomare: Nothing to disclose
Laura Cea: Nothing to disclose

### RPS 1309-8

**Interventional treatment in small-sized hepatocellular carcinoma (HCC) using microwave ablation (MWA): evaluation of local response and survival**

*T. J. Vogl*, H. Adwan, J. Trojan, N. N. N. Naguib, T. Gruber-Rouh; Frankfurt/DE
*(t.vogl@em.uni-frankfurt.de)*

**Purpose:** The study aimed to retrospectively evaluate the safety and efficacy of CT-guided percutaneous microwave ablation (MWA) of small-sized (≤2cm) hepatocellular carcinoma (HCC) regarding therapy response and survival rates.
**Methods or Backround:** In 87 patients (16 females, 71 males; mean: 63.8±10.8 years) with 130 small-sized HCC lesions, CT-guided MWA was performed. All cases were evaluated according to the axial diameter of the tumour, the volume of the post-ablation zone, technical success, complete ablation, therapy response, overall survival (OS), and progression-free survival. Contrast-enhanced MR imaging was performed post-ablation for evaluation of response to MWA.
**Results or Findings:** The mean axial diameter of the tumour was 1.4cm (range: 0.5-2cm), and the mean post-ablation volume was 32.6cm³. A technical success rate of 100% was achieved in all ablations (130/130). The complete ablation rate was 97.7% (127/130) of all tumours. The rate of local tumour progression (LTP) was 4.6% (4/87), and the rate of distant intrahepatic recurrence was 36.8% (32/87). 1-, 3-, and 5-year OS rates were 94.3%, 66.4% and 53.8%, respectively. The 1-, 3-, and 5-year PFS rates were 70.5%, 46.4% and 33%, respectively. No peri-procedural deaths were reported.
**Conclusion:** CT-guided MWA seems to be an effective and safe treatment for small-sized HCC with long OS time, low LTP rate and a low rate of complications.
**Limitations:** No limitations were recognised.
**Ethics committee approval:** Not applicable.
**Funding for this study:** No funding was received.
**Author Disclosures:**
Nagy Naguib Naeem Naguib: Nothing to disclose
Jörg Trojan: Nothing to disclose
Thomas J. Vogl: Nothing to disclose
Tatjana Gruber-Rouh: Nothing to disclose
Hamzah Adwan: Nothing to disclose

### RPS 1309-9

**Clinical and technical outcomes of intraoperative microwave thermal ablation of liver malignancies**

*R. Muglia*, D. Pinelli, P. Marra, L. Dulcetta, F. S. Carbone, M. Colledan, S. Sironi; Bergamo/IT
*(r.7@hotmail.it)*

**Purpose:** The study aimed to evaluate clinical and technical outcomes of intraoperative (laparoscopic/laparotomic) thermal ablation of liver malignancies performed with microwave technology.
**Methods or Backround:** We retrospectively evaluated 29 patients (M:F=23:6, mean age 65yrs) treated in our institute for liver malignancies with intraoperative microwave thermal ablation from July 1, 2017, to September 30, 2022. The percutaneous ultrasound-guided approach was excluded due to the suboptimal nodule visibility or harmful location, while liver resection for deep position or adherences. Data about liver and nodule pathology, surgical approach, nodule number and dimensions, technical success, complications and hospital length stay were collected. Technical success was intended as the absence of locoregional malignancies at follow-up CT/MRIs. Patients were followed-up on average for 17.5 months (σ=13.6).
**Results or Findings:** All patients but two were cirrhotic (93%), 14/29 (48%) had a single nodule, 11/29 (38%) had two, 4/29 had three (14%); 22/29 (75%) were treated in laparoscopy, 7/29 (25%) with a laparotomic approach. We ablated 48 nodules, 45 (94%) HCCs and 3 (6%) colorectal cancer metastases, of 17x17mm (mean value, σ=8.3), for 6 minutes (mean value, σ=4.5) with 100W of power. 44/48 nodules (92%) were treated successfully; the other 4/48 (8%, HCCs) presented residual disease on postprocedural CT/MRI and underwent subsequent chemo/radioembolisation. Only one complication was encountered (2%), a biliary fistula treated with glue embolisation. The average hospital stay was five days (σ=6.4).
**Conclusion:** Laparoscopic/laparotomic intraoperative liver ablation with microwaves is a fast and feasible procedure for a significant proportion of patients, unsuitable for percutaneous approach or hepatic resection, and burdened by rare recurrences and complications.
**Limitations:** We acknowledge the possibility of biases in patients' selection due to the study's retrospective nature, leading to cohort inhomogeneity. Moreover, our relatively small population prevented us from going beyond a descriptive statistical analysis.
**Ethics committee approval:** No information provided by the submitter.
**Funding for this study:** No funding was received for this study.
**Author Disclosures:**
Sandro Sironi: Nothing to disclose
Paolo Marra: Nothing to disclose
Francesco Saverio Carbone: Nothing to disclose
Domenico Pinelli: Nothing to disclose
Riccardo Muglia: Nothing to disclose
Michele Colledan: Nothing to disclose
Ludovico Dulcetta: Nothing to disclose

### RPS 1309-10

**Percutaneous and intraoperative microwave ablation of liver malignancies with a novel system working at 150W: assessment of safety, efficacy and ablation zone**

*D. Pastena*, A. Della Corte, F. De Cobelli, S. Gusmini, L. Aldrighetti, F. Ratti, F. Cipriani, D. Palumbo, E. Di Gaeta; Milan/IT
*(Davidepastena13@gmail.com)*

**Purpose:** Microwave ablation (MWA) is an effective treatment for selected cases of liver malignancies. Introducing a system that delivers ablation power up to 150W has led to potentially larger ablation zones (AZs) in a shorter time. In our study, we aimed to analyse technique efficacy, AZs features and safety profile of MWA performed with a novel system working at 150W.
**Methods or Backround:** From May to September 2022, 14 consecutive patients underwent ultrasound-guided percutaneous or intraoperative MWA of 27 liver tumours with a 2450MHz/150W microwave generator (Emprint HP, Medtronic). Contrast-enhanced CT scan was performed one month after ablation to assess complete ablation, minimal ablation margins, AZ and sphericity index (SI).
**Results or Findings:** The mean lesion size was 16.2±9.4mm (5-40mm), including 3 nodules >30 mm (31, 35 and 40 mm, respectively). Mean ablation times were 231.1±97.7s (90-480s). Of the 27 ablated tumours (n=16 colorectal metastases, n=5 gastric metastases, n=3 intrahepatic cholangiocarcinoma, n=3 hepatocellular carcinoma), seven were treated percutaneously, 16 during laparoscopy and four during laparotomy. No intra- or periprocedural major complications were observed. At the CT scan performed one month, complete ablation was observed in all tumours (technique efficacy=100%), achieving AZs of 21.2±10cm2 (6.9-42.9cm2), a SI of 0.84±0.11 (0.5-0.97). Minimal ablation margins exceeded 5mm in all cases; in particular, in 22 tumours (81.5%), minimal margins exceeded 1 cm. On follow-up imaging, peripheral portal thrombosis along the track ablation route was observed in two patients (14.3%), not leading to clinical sequelae (grade 2).

**Conclusion:** Microwave ablation performed at 150W is a safe and effective technique also for larger liver lesions, achieving predictable spherical ablation zones.
**Limitations:** The study is limited by its sample size.
**Ethics committee approval:** The ethics committee approval is available under number MWA-01: 59/INT/2017.
**Funding for this study:** No funding was received for this study.
**Author Disclosures:**
Davide Pastena: Nothing to disclose
Angelo Della Corte: Nothing to disclose
Luca Aldrighetti: Nothing to disclose
Diego Palumbo: Nothing to disclose
Simone Gusmini: Nothing to disclose
Federica Cipriani: Nothing to disclose
Francesco De Cobelli: Nothing to disclose
Ettore Di Gaeta: Nothing to disclose
Francesca Ratti: Nothing to disclose

### RPS 1309-11

**Magnetic resonance imaging evaluation after percutaneous thermal ablation for malignant lung tumours: a tertiary centre experience**
*B. M. Suwita*, I. O. Akanbi, N. Zahran, E. Blana, P. Dalal; Harefield/UK
*(benedicta.mutiara.s@gmail.com)*

**Purpose:** The study aimed to evaluate whether MRI follow-up from day one and one month can predict tumour recurrence in 12 or 24 months.
**Methods or Backround:** A retrospective study examining patients who had CT-guided lung ablation between 2018 and 2020. Before undergoing ablation, all patients signed informed consent to use their imaging for research purposes anonymously. Our department performed MRI follow-ups on day one, and 1, 3, 6, 9, 12 months and then every six months after ablation. We evaluated MRI results on day one, one month, 12-month, and 24-month for contrast enhancement inside the ablation zone, which indicated tumour viability. Differences in contrast enhancement across the timeline (day one, 1-month, 12-month and 24-month) were calculated using Cochran’s Q test, followed by post-hoc analysis using McNemar’s test.
**Results or Findings:** Out of 111 patients, 27 were excluded due to not having MRI follow-up. Eighty-four patients (22 primary and 62 secondary tumours) with 237 lung lesions were analysed. Each patient had median 2 lung lesions (range 1–14 lesions) with a median size 13mm (range 3–90mm). Eighteen (7.6%) lesions were ablated using cryoablation, 93 (39.2%) RFA, and 126 (53.2%) MWA. There were statistically significant differences in contrast enhancement inside the ablation zone across the timeline ($p<0.001$). Post-hoc analysis showed a significant difference between 1-month and 12-month ($p<0.001$), 1-month and 24-month ($p<0.001$), 12-month and 24-month ($p<0.001$), and 1-day and 24-month ($p<0.001$). No significant difference between day one and 1-month ($p=0.167$).
**Conclusion:** MRI follow-up on day one and 1-month could not predict tumour recurrence for 12-month and 24-month. Our results suggest that routine MRI follow-up can be effectively performed on 1-month, 12-month, and 24-month after lung ablation.
**Limitations:** Not all lesions were histology-proven; some were decided by multidisciplinary-team meetings based on the clinical history and imaging appearance.
**Ethics committee approval:** No information provided by the submitter.
**Funding for this study:** No funding was provided for the study.
**Author Disclosures:**
Benedicta Mutiara Suwita: Nothing to disclose
Isaac Olukayode Akanbi: Nothing to disclose
Nahla Zahran: Nothing to disclose
Paras Dalal: Nothing to disclose
Eirini Blana: Nothing to disclose

**10:30-12:00 Research Stage 3**

### Research Presentation Session: Neuro

### RPS 1311

### Degenerative diseases of the brain

**Moderator**
A. Majos; Lodz/PL
*(agata.majos@umed.lodz.pl)*

### RPS 1311-2

**Role of high-resolution ultrasound in migraine**
*L. Tovt*, M. Pansecchi, F. Zaottini, R. Picasso, U. Viglino, F. Pistoia, S. Sanguinetti, C. Martinoli; Genoa/IT
*(lucatovt@yahoo.it)*

**Purpose:** Our study aims to evaluate if high-resolution ultrasound (HRUS) can visualise the greater occipital nerve (GON) along its entire course. Furthermore, the study aims to identify pathological nerve alterations in patients suffering from migraine headache (MH) and candidates for surgery.
**Methods or Backround:** In patients with untreatable MH symptoms, excellent results are achieved through surgical GON neurolysis and focal occipital artery ligation. All patients candidates for surgery were examined using a 22-5MHz probe. The GON size superficial to obliquus capitis inferior, at the level of trapezius fascia perforation, the occipital artery diameter at the crossing point over the GON and the presence of GON alterations were recorded and compared to sex and age-matched healthy volunteer group. Two observers measured the nerve in healthy volunteers, and their agreement was evaluated with ICC statistics. The statistical significance of the measures between the volunteer and healthy group was evaluated with Student's t-test.
**Results or Findings:** 25 patients, 14 female (mean age=43; mean BMI=21) and 11 male (mean age=51; mean BMI=24) and 25 matched healthy volunteers were enrolled. The GON has been identified in all subjects with the high inter-observer agreement. The HRSU pathological findings, such as nerve enlargement in the piercing point of trapezius fascia or perineural fibrosis, were found in 50% of patients. All the findings were confirmed during surgery. No significant differences in the anatomical parameters investigated between healthy subjects and patients ($p=0,05$) were found.
**Conclusion:** The study showed that HRUS is reliable to depict the anatomical course of GON and to assess the point of the possible neurovascular conflict. HRUS can also demonstrate pathological nerve changes in a subgroup of patients.
**Limitations:** The study is limited by the small sample, and by the fact that GON nerve conduction studies are unavailable.
**Ethics committee approval:** Not applicable.
**Funding for this study:** No funding was received for this study.
**Author Disclosures:**
Federico Zaottini: Nothing to disclose
Carlo Martinoli: Nothing to disclose
Luca Tovt: Nothing to disclose
Umberto Viglino: Nothing to disclose
Michelle Pansecchi: Nothing to disclose
Sara Sanguinetti: Nothing to disclose
Riccardo Picasso: Nothing to disclose
Federico Pistoia: Nothing to disclose

### RPS 1311-3

**Large-scale functional network connectivity mediates the associations between serum lipids and cognitive function in patients with type 2 diabetes**
*W. Zhang*, B. Zhang; Nanjing/CN

**Purpose:** Patients with type 2 diabetes (T2D) often have altered lipid metabolism and cognitive decline. In this study, we explored the large-scale functional network connectivity alterations and their potential relationships with serum lipids and cognitive performance in T2D patients.
**Methods or Backround:** A total of 147 age-, sex- and education-matched participants were included, divided into T2D with mild cognitive impairment (T2MCI), T2D with normal cognition (T2NC) and healthy control subjects. Each participant underwent resting-state functional MRI scans and cognitive and peripheral blood biochemistry assessments. Large-scale internetwork FCs were calculated using independent component analysis. Partial correlation and mediation analyses were conducted to test for potential associations between lipid metabolism, internetwork FC, and cognitive performance.
**Results or Findings:** Compared with healthy control, the inter- and intra-network FCs were reduced in either T2MCI or T2NC. We found significant differences in inter-network FC among the three groups: between ventral attention and medial visual network and between left frontoparietal and posterior visual network. In T2D patients, lower inter-network FCs were

Friday correlated with higher serum total cholesterol, high-density lipoprotein cholesterol, and low-density lipoprotein cholesterol while correlated with worse cognitive performance. More importantly, the relationship between lipid metabolism scores and cognitive scores was mediated by the inter-network FC scores.
**Conclusion:** Our results suggested the possibility of improving cognitive function through regulating serum lipids in diabetic patients.
**Limitations:** This study is a cross-sectional study that cannot reveal causality. A longitudinal lipid-specific intervention study is needed to establish the direction of causality.
**Ethics committee approval:** All participants provided informed consent. The study was in accordance with the Declaration of Helsinki and registered at Clinicaltrials.gov (NCT02738671).
**Funding for this study:** This work was supported by the Jiangsu Funding Programme for Excellent Postdoctoral Talent and funding for clinical trials from the Affiliated Drum Tower Hospital, Medical School of Nanjing University.
**Author Disclosures:**
Bing Zhang: Nothing to disclose
Wen Zhang: Nothing to disclose

### RPS 1311-4

**Patterns of brain structure-function associations specific to cognitively normal individuals, patients with mild cognitive impairment, and dementia**

*S. Meribout*[1], Y. Statsenko[2], T. Habuza[2], T. M. E. Almansoori[2], K. Neidl-Van Gorkom[2], J. George Gelovani[2], M. Ljubisavljevic[2]; [1]Constantine/DZ, [2]Al Ain/AE
*(s-meribout@hotmail.com)*

**Purpose:** Brain morphology and cognitive performance undergo age-related changes which differ in the onset and progression rate between individuals with and without neurodegeneration. Hypothetically, the association of brain structure with function (SFA) also differs between normal and accelerated ageing. Our objective was to find SFA features specific to normal ageing, mild cognitive impairment (MCI) and dementia.
**Methods or Backround:** From the Alzheimer’s Disease Neuroimaging Initiative Database, we collected T1-weighted MRI images and cognitive test results of 1,302 subjects and constructed regression models predicting functional performance in cognitive tests from brain radiomics. The models were trained separately in each study cohort. We used impurity-based variable importance for ranking the features according to their predictive potential. The model performance metrics were the mean absolute error with adjustment to the range of values (MAE/range).
**Results or Findings:** We showed that structural determinants of cognitive performance differ among distinct cognitive tests and study cohorts. This can be explained by the involvement of different areas in the cognitive tests. Variance in age- and disease-related brain atrophy also account for different patterns of brain SFA in normal ageing and pathology. MMSE results can be predicted much more accurately than other tests: MAE/range is 4.5±0.23 in the cognitively normal examinees. The prediction of ADAS-cog also has credible performance (5.04±0.22). The RAVLT, TMT and DSST score predictions are significantly less accurate (10.62±0.5, 10.57±0.68 and 10.81±0.51; $p<0.001$).
**Conclusion:** In pathological ageing, neuronal loss differs among distinct cell groups and brain regions. Consequently, the SFA has pathology-specific features and can serve as a diagnostic marker of early MCI or dementia.
**Limitations:** Not applicable.
**Ethics committee approval:** No information provided by the submitter.
**Funding for this study:** Not applicable.
**Author Disclosures:**
Juri George Gelovani: Nothing to disclose
Klaus Neidl-Van Gorkom: Nothing to disclose
Taleb Mohamed Ebrahim Almansoori: Nothing to disclose
Milos Ljubisavljevic: Nothing to disclose
Sarah Meribout: Nothing to disclose
Tetiana Habuza: Nothing to disclose
Yauhen Statsenko: Nothing to disclose

### RPS 1311-5

**Free water alterations of deep grey matters over one to two years in small vessel disease**

*Y. Sun*, X. Han, Y. Hu, Y. Zhou; Shanghai/CN
*(cjs1119@hotmail.com)*

**Purpose:** The study aimed to evaluate the inflammation-related characteristics of the deep grey matter (DGM) using free-water (FW) mapping in small vessel disease (SVD) over one to two years.
**Methods or Backround:** Sixty-seven SVD patients with no cognitive impairment (NCI), 103 with mild cognitive impairment (VaMCI), and 21 healthy controls underwent brain MRI scans and neuropsychological evaluations at baseline. Of these, 28 VaMCI and 23 NCI participated in the follow-up study one to two years later. FW values within DGM, including the bilateral caudate nucleus, globus pallidus, putamen, substantia nigra, red nucleus, and dentate nucleus, were calculated for each individual. FW values in the thalamus were calculated in the subregion, including anterior nuclei, median nuclei, lateral nuclei (TL-LT), pulvinar (TL-PLV), and internal medullary lamina (TL-IML). Cross-sectional group comparisons were performed with one-way ANOVA. In longitudinal comparison, temporal and cross-sectional differences were studied using mixed factorial ANOVA analysis. Partial correlations were used to assess the relationship between the FW changes and cognitive function changes.
**Results or Findings:** Significantly higher FW values (index of neuroinflammation) were found in DGM at baseline in VaMCI and NCI compared to healthy controls. In longitudinal comparison, the group effect resulted in greater FW values in the left TL-PLV, bilateral TL-LT, and TL-IML for VaMCI compared with NCI. Furthermore, we observed significant negative associations between the FW value changes in the left TL-PLV and the right TL-LT and MoCA scores changes in VaMCI over one to two years.
**Conclusion:** Our results support the presence of elevated FW at the preclinical stage, which remains persistent during the early course of the illness. The FW changes might be the biomarkers for the mechanism of cognitive decline in the evolution of SVD.
**Limitations:** Only 51 SVD patients were included in the follow-up.
**Ethics committee approval:** The current study was approved by the Research Ethics Committee of the Renji Hospital, School of Medicine, Shanghai Jiao Tong University. Written informed consent was obtained from each subject before participation. All procedures were carried out in accordance with institutional guidelines.
**Funding for this study:** The study is funded by the National Natural Science Foundation of China (81901693).
**Author Disclosures:**
Xu Han: Nothing to disclose
Ying Hu: Nothing to disclose
Yan Zhou: Nothing to disclose
Yawen Sun: Nothing to disclose

### RPS 1311-6

**Machine learning-based perivascular space quantification in Alzheimer's disease**

A. Decker, P. Scheyhing, J. Harten, N. Zimmermann, D. Paech, A. Schneider, A. Radbruch, A. Effland, *K. Deike-Hofmann*; Bonn/DE

**Purpose:** Dysfunction of brain clearance via perivascular spaces (PVS) was suggested to play a key role in protein-accumulation-dementias (PADs) such as Alzheimer’s disease (AD) due to reduced clearance of beta-amyloid (Aβ) and other toxic metabolites. Here, we aimed to assess the PVS volume in patients with subjective cognitive decline (SCD), mild cognitive impairment (MCI) or mild AD compared to a cognitively healthy reference group (RG).
**Methods or Backround:** A machine-learning algorithm was trained to segment the PVS in the semiovale centre (CSO) on T2w MRI scans (n=894) of the DZNE Longitudinal Cognitive Impairment and Dementia Trial (DELCODE). Multivariate linear regression analysis adjusted for the patient's age was performed to correlate the PVS volume fraction (VF) with clinical data, including AD stage and cognitive function.
**Results or Findings:** Mild AD and MCI, but not SCD, patients had a significantly higher PVS VF than the RG ($p_{AD}=0.004$, $p_{MCI}=0.001$). Furthermore, already healthy study participants who developed MCI within 4.5 years after baseline revealed a higher PVS VF compared to those who stayed cognitively healthy. Cognitive function, as well as cognitive decline, were negatively correlated with the PVS VF ($p<0.005$ each).
**Conclusion:** PVS quantity in the CSO represents a potential imaging biomarker for cognitive function and decline in AD. Furthermore, the here shown very early changes of PVS volume in the AD continuum might hint towards a contribution of PVS to the pathogenesis of AD.
**Limitations:** Limitations include the moderate interrater agreement, and the image quality of the T2-weighted MRI data was not ideal for exact PVS delineation in every participant. Furthermore, we found a considerable interindividual variability of the PVS VF independent of the AD disease stage.
**Ethics committee approval:** The study was approved by the DELCODE steering committee.
**Funding for this study:** No funding was received for this study.
**Author Disclosures:**
Andreas Decker: Nothing to disclose
Nadine Zimmermann: Nothing to disclose
Katerina Deike-Hofmann: Shareholder: relios.vision GmbH Founder: relios.vision GmbH
Anja Schneider: Nothing to disclose
Daniel Paech: Founder: relios.vision GmbH Shareholder: relios.vision GmbH
Paul Scheyhing: Nothing to disclose
Alexander Effland: Founder: relios.vision GmbH Shareholder: relios.vision GmbH
Alexander Radbruch: Founder: relios.vision GmbH Shareholder: relios.vision GmbH
Julia Harten: Nothing to disclose

### RPS 1311-7
**Cross-modal imaging correlates of the substantia nigra in Parkinson's disease**
*L. J. Y. Lim*, Q. Lyu, S. Hartono, M. P. Lee, Q. Fan, E. K. Tan, L. Chan; Singapore/SG
*(e0008419@u.nus.edu)*

**Purpose:** Capabilities for high-resolution quantitative midbrain NM- and iron-sensitive MRI techniques have been recently developed for Parkinson's disease (PD) evaluation. The echogenic substantia nigra (SN) on transcranial ultrasound (TCS) is known to be associated with Parkinson's disease. MRI cross-modal correlates to TCS imaging data are unknown. We aim to evaluate correlates between SN echogenicity on TCS and neuromelanin/iron quantitative MRI measures in a case-control cohort of PD patients.
**Methods or Backround:** PD patients recruited from the Movement Disorders Clinic and age-matched healthy controls underwent brain MRI on a 3T scanner and TCS using established methods. From these, quantitative sonographic, neuromelanin and quantitative susceptibility mapping (QSM) measures were obtained. The size of the SN echogenicity on TCS, and the size and quantitative values of the SN mask on neuromelanin-sensitive and QSM MRI sequences were recorded. Two-sided parametric procedures, t-test and Pearson's correlation were used to compare the cross-modal quantitative imaging measures to identify significant correlates.
**Results or Findings:** 124 subjects were included in the final analyses. Our findings demonstrated a negative correlation between sizes of SN echogenic area to SN mask on neuromelanin ($p<0.001$, $r=-0.3$) and QSM ($p<0.0001$, $r=-0.4$). SN echogenic area positively correlated to SN susceptibility value after thresholding ($p<0.005$, $r=0.3$), whereas there was no significant correlation to neuromelanin signal intensity value.
**Conclusion:** The echogenic SN size on TCS correlated negatively with SN mask size on neuromelanin and QSM MRI, which was stronger for QSM. The susceptibility value on QSM also correlated positively with echogenic SN size on TCS. Stronger correlations between SN echogenicity and iron are found than neuromelanin quantitative MRI measures in PD.
**Limitations:** Full midbrain volumetric co-registration may allow the exact localisation of pathology and cross-modal imaging surrogates.
**Ethics committee approval:** Approval from the institutional ethics review board and informed consent from all subjects were obtained.
**Funding for this study:** This study was funded by the National Medical Research Council, Singapore.
**Author Disclosures:**
Leon Jian Ying Lim: Nothing to disclose
Ling Chan: Nothing to disclose
Qiqi Lyu: Nothing to disclose
Eng King Tan: Nothing to disclose
Septian Hartono: Nothing to disclose
Qiao Fan: Nothing to disclose
Moi Pin Lee: Nothing to disclose

### RPS 1311-8
**Neuromelanine-MRI using 2D gradient echo and deep learning: considerations for improving the visualisation of substantia nigra and locus coeruleus**
*S. Abo Seada*, A. van der Eerden, A. Boon, J. Hernandez-Tamames; Rotterdam/NL
*(s.aboseada@erasmusmc.nl)*

**Purpose:** The study aimed to improve neuromelanine-MRI imaging protocol for simultaneously visualising the SN and LC, using readily available deep-learning reconstruction.
**Methods or Backround:** Neuromelanine-MRI is an MR contrast for assessing neuromelanine (NM) content in the substantia nigra (SN) and locus coeruleus (LC), with diagnostic value for atypical parkinsonisms and possibly schizophrenia. Using a 2D MT-weighted gradient-echo sequence (0.4x0.4x3mm, TE=7.5ms, TR=340ms) in healthy volunteers, we varied several acquisition parameters and reconstruction methods to improve simultaneous SN and LC imaging. Using SPGR-signal calculations and relaxometry, optimal flip-angle was determined, and acquisition parameters affecting SNR were varied (partial echo [TE=4ms], anisotropic-resolution [0.4x0.7mm] and averages).
**Results or Findings:** The optimal flip-angle was 37 and 29 degrees for SN and LC, respectively. However, at low flip-angles, the resulting high signal of adjacent CSF renders visualising the LC challenging, and a flip-angle of 50 degrees was preferable in-vivo. Acquiring full vs partial echoes in single-average experiments increases SNR by 20% for both isotropic and anisotropic acquisition. When using multiple averages, full echoes increase SNR by 15% for a 13% time increase, and full echoes reduce susceptibility artefacts. Isotropic resolution (using three averages) performs similarly to anisotropic resolution (using 5 averages) similar in scan time (7min). Anisotropic resolution improves SNR but shows partial volume effects of the dot-like LC along the long axis of the voxel, while isotropic imaging depicts dots better in phantoms and in-vivo. Vendor-supplied deep-learning denoises isotropic-resolution images, but it cannot resolve partial volume effects from anisotropic-resolution data.
**Conclusion:** Flip-angle should be optimised relative to TR to avoid CSF signal. We recommend acquiring full echoes with the isotropic resolution for improved depiction and using deep-learning denoising to increase SNR (7:28s).
**Limitations:** 2D sequences were tested on three healthy volunteers.
**Ethics committee approval:** The ethics committee of the METC Erasmus MC, Rotterdam, gave their consent.
**Funding for this study:** SAS and AvdE received funding from Parkinson NL.
**Author Disclosures:**
Agnita Boon: Nothing to disclose
Juan Hernandez-Tamames: Nothing to disclose
Samy Abo Seada: Nothing to disclose
Anke van der Eerden: Nothing to disclose

### RPS 1311-9
**Composite iron and neuromelanin MRI quantitative marker matches expert Parkinson's disease MR visual nigrosome-1 classification and correlates with disease severity**
*L. L. Chan*, S. Hartono, R. Chen, W. Lee, P. Y. Teh, W. Hou, W. P. Tham, E. K. Tan; Singapore/SG
*(ling2chanSGH@gmail.com)*

**Purpose:** High-resolution nigral iron and neuromelanin-sensitive (NMS) MRI are useful radiological biomarkers in Parkinson's disease (PD), but diagnostic performance is variable in clinical practice. Susceptibility map-weighted imaging (SMwI) from varied scanners by expert raters has shown excellent diagnostic performance, but its diagnostic performance in non-expert raters, against NMS visual assessment by expert raters and quantitative MR parameters, is unknown. In a case-control cohort study, we compared the performance of susceptibility map-weighted imaging (SMwI) against NMS in the radiological classification of PD across raters of different expertise and extracted quantitative parameters.
**Methods or Backround:** 191 participants underwent clinical motor assessment and brain MRI using NMS and multi-echo susceptibility-weighted sequences. Quantitative susceptibility mapping (QSM) and SMwI were reconstructed. Radiological assessment of the substantia nigra (SN) by experts and non-expert raters using SMwI and NMS, quantitative parameters from SN masks were evaluated.
**Results or Findings:** Inter-rater agreement across raters for radiological nigrosome-1 assessment was substantial on SMwI ($\kappa=0.74$) and fair for hyperintensity loss on NMS (0.37), and excellent across all quantitative MR parameters (>0.85). Radiological PD classification using SMwI (accuracy >90%) was superior to NMS (75-<90%) across raters. A composite NMS-QSM quantitative marker achieved excellent classification performance (AUC 94%). A correlation between quantitative MRI parameters and clinical severity ($r=-0.4$, $p<0.0001$) was found.
**Conclusion:** SMwI was superior to NMS in the radiological classification of PD, and rater expertise affected performance. Matching the diagnostic performance of a composite NMS-QSM quantitative marker to that of expert raters has the potential for wide-scale objective evaluation and disease tracking in PD.
**Limitations:** Automated SN segmentation could further facilitate workflow, reduce variability and allow entire SN volume evaluation. Our manual SN segmentation was completed within 10 minutes, with the excellent inter-rater agreement.
**Ethics committee approval:** Not applicable.
**Funding for this study:** This study was funded by the Singapore National Medical Research Council.
**Author Disclosures:**
Robert Chen: Nothing to disclose
Ling Ling Chan: Nothing to disclose
Peik Yen Teh: Nothing to disclose
Eng King Tan: Nothing to disclose
Wei Ping Tham: Nothing to disclose
Weiling Lee: Nothing to disclose
Septian Hartono: Nothing to disclose
Wenlu Hou: Nothing to disclose

### RPS 1311-10
**Evaluation of cerebral volume changes in patients with tremor treated by MRgFUS thalamotomy**
*F. Bruno*, L. Pertici, C. Fagotti, G. Caldarelli, A. Catalucci, S. Martino, S. Mancini, C. Masciocchi, A. Splendiani; L'Aquila/IT
*(federico.bruno.1988@gmail.com)*

**Purpose:** Numerous studies have used imaging techniques to demonstrate the presence of structural and functional brain alterations in patients with tremor, trying to extract useful diagnostic and prognostic information. This is the first study to evaluate the changes in brain cortical and subcortical volumes in patients with essential tremor and Parkinson's disease after MRgFUS

thalamotomy. The secondary study's aim was to correlate volume changes with the patient's clinical features and treatment outcomes.
**Methods or Backround:** For this pilot retrospective study, we enrolled 31 patients (15 ET, mean age of 70.86 years) eligible for unilateral Vim thalamotomy. Clinical evaluation included tremor severity assessment using the FTM scale and cognitive assessment using the MoCA score. MRI data were acquired with a 3T scanner, using a dedicated 32-channel coil and acquiring a volumetric sequence T1 3D IR FSPGR, before treatment and one year after MRgFUS thalamotomy. Image processing and volume data extraction were conducted with dedicated software.
**Results or Findings:** Volumetric analysis showed a significant reduction ($p<0.05$) of the left thalamus one year after treating patients with ET. Other significant results were found on the same side in the other nuclei of the basal ganglia and the cerebellar cortex. In confronting the two groups (ET, PD), no significant differences were found in terms of age, FTM, MoCA scores, and brain volumes. Similarly, no significant correlations were found between the FTM and MoCA scores and the brain volumes before the treatment.
**Conclusion:** The preliminary results of this study highlight possible functional differences between essential tremor and Parkinson's in response to MRgFUS treatment. Further studies are needed to explore the actual prognostic value.
**Limitations:** The study is limited by the sample size.
**Ethics committee approval:** The local IRB gave the ethics approval.
**Funding for this study:** No funding was received for this study.
**Author Disclosures:**
Giulia Caldarelli: Nothing to disclose
Alessandra Splendiani: Nothing to disclose
Carlo Masciocchi: Nothing to disclose
Sara Mancini: Nothing to disclose
Leonardo Pertici: Nothing to disclose
Saverio Martino: Nothing to disclose
Cristina Fagotti: Nothing to disclose
Alessia Catalucci: Nothing to disclose
Federico Bruno: Nothing to disclose

**10:30-12:00 Research Stage 4**

### Research Presentation Session: Artificial Intelligence & Machine Learning & Imaging Informatics

### RPS 1305

### Opportunistic screening and body composition analysis using AI

**Moderator**
O. Arponen; Tampere/FI
*(otso.arponen@tuni.fi)*

### RPS 1305-2

**Aortic aneurysm volume and maximum diameter measurements per segment: full automation by artificial intelligence (AI)**
E. Guillo[1], A. Heraud[2], T. J. Postiglione[1], M. A. Bartoli[3], D. Fabre[1], S. Haulon[1], R. Ardon[1], G. d'Assignies[1], *A. A. Azarine*[1]; [1]Paris/FR, [2]Neuilly/FR, [3]Marseille/FR
*(drazarine@gmail.com)*

**Purpose:** The study aimed to evaluate a fully automatic method measuring outer to outer aortic wall maximum diameters and volume for each aortic segment.
**Methods or Backround:** On 350 CTA scans, three to nine expert clinicians performed diameter measurements and manual segmentations on all aortic segments. These scans originated from two French aortic centres and contained preoperative (38%) and postoperative (62%) cases with endovascular aortic repairs, aneurysms (70%) and dissections (11%). Another database of 1150 CTAs (similar variability and annotations) served to train an algorithm for automatic diameter and volume measurements. Automatic diameter measurements were compared to experts' median measurements. Volume estimation was compared using DICE and volume similarity (|V1-V2|/(2*(V1+V2)).
**Results or Findings:** Results are reported for aortic sections: ascending, arch, descend thoracic, suprarenal, infrarenal, right and left iliac, with 95% confidence intervals in brackets. Absolute median error w.r.t experts median measurement are (mm): 1.42[1.27, 1.58]; 2.18 [1.86, 2.5]; 1.68 [1.56, 1.87]; 1.57 [1.42, 1.74]; 1.78 [1.64, 2.0]; 1.47 [1.29, 1.68]; 1.48 : [1.34, 1.61] respectively. Median Dice coefficients are 0.87 [0.85, 0.88]; 0.80 [0.78, 0.81]; 0.9 [0.88, 0.9]; 0.8 [0.78, 0.81]; 0.90 [0.88, 0.9]; 0.74 [0.71, 0.75]; 0.73 [0.7, 0.76] respectively. Median volume similarity is 0.93 [0.92, 0.94], 0.9 [0.87, 0.92], 0.94 [0.93, 0.94], 0.9 [0.88, 0.92], 0.94 [0.93, 0.95], 0.8 [0.77, 0.82], 0.82 [0.8, 0.85] respectively. The median Dice coefficient and median volume similarity for the whole aorta are 0.95 [0.94, 0.95] and 0.98 [0.98, 0.99], respectively.
**Conclusion:** Automatic measurements of maximum aortic diameters are precise, in the median, to less than 2.5mm, with respect to experts. Automatic volume estimations for all sections are above 0.9 volume similarity. With its perfect reproducibility, this tool is a reliable solution for assisting clinical practice.
**Limitations:** The study is designed as a retrospective study.
**Ethics committee approval:** Review Boards of Groupe Hospitalier Paris-Saint-Joseph approved this study (IRB 00012157).
**Funding for this study:** No funding was received for this study.
**Author Disclosures:**
Michel A. Bartoli: Nothing to disclose
Dominique Fabre: Nothing to disclose
Stéphan Haulon: Nothing to disclose
Gaspard d'Assignies: Founder: Incepto-Medical
Thomas J Postiglione Thomas J. Postiglione: Nothing to disclose
Alexandre Heraud: Nothing to disclose
Roberto Ardon: Employee: Incepto-Medical
Arshid A. Azarine: Nothing to disclose
Enora Guillo: Nothing to disclose

### RPS 1305-3

**Deep learning for L3 body composition in paediatric CTs: a feasibility study**
*R. De Angelis*, K. Brou Boni, N. Coquelet, P. Simoni, M. A. Bali; Brussels/BE
*(riccardo.deangelis@bordet.be)*

**Purpose:** Body composition in terms of superficial abdominal fat (SAT), visceral abdominal fat (VAT), and skeletal muscle (SM) based on L3-centered computer tomography (CT) has been shown to be linked to outcomes in various clinical scenarios. Deep learning (DL) has recently become a promising tool to substitute standard semi-automatic methods, thereby fastening body composition and limiting potential inter-radiologists variability. However, its applicability in paediatric settings remains limited due to the low number of CTs performed in children. Our study investigates to what extent DL networks trained on adult CTs are reliable for the body composition of paediatric CTs.
**Methods or Backround:** A pre-trained convolutional neural network (ResNet-9 architecture) was trained on 40 adult CTs segmented by an experienced radiologist. Semi-segmentation of SAT, VAT and SM was performed using the syngo.via software (Siemens Heathineers, DE). The trained network was then used to generate a segmentation map of 13 paediatric CTs of children aged between four weeks and nine years from a retrospective anonymised database. Body composition similarity between the semi-automatic approach and the DL was reported in terms of the Dice similarity coefficient (DSC).
**Results or Findings:** The mean DSC were 0.93±0.01 for nine evaluated children. DL failed to assess four segmentations due to the presence of artefacts (e.g. the presence of oral contrast in native CT).
**Conclusion:** Our study shows that DL networks trained on adult CTs are promising for the body composition of paediatric CTs. This mitigates the need to have specific paediatric training. However, further investigations are needed with a more significant number of CTs and age stratification.
**Limitations:** The study is limited by the Low number of paediatric CTs, wide range of ages, and lack of interobserver comparison.
**Ethics committee approval:** No information provided by the submitter.
**Funding for this study:** Not applicable.
**Author Disclosures:**
Riccardo De Angelis: Nothing to disclose
Paolo Simoni: Nothing to disclose
Maria Antonietta Bali: Nothing to disclose
Nicolas Coquelet: Nothing to disclose
Kevin Brou Boni: Nothing to disclose

### RPS 1305-4

**Automated deep learning-based segmentation of abdominal adipose tissue on whole-body MRI in a population-based study of adolescents**
*T. Wu*[1], S. Estrada[2], R. Van Gils[1], R. Su[1], V. Jaddoe[1], E. Oei[1], S. Klein[1]; [1]Rotterdam/NL, [2]Bonn/DE
*(w.tong.1@erasmusmc.nl)*

**Purpose:** The study aimed to evaluate an automated deep-learning method for segmenting subcutaneous adipose tissue (SAT) and visceral adipose tissue (VAT) volumes on whole-body MRI scans in adolescents.
**Methods or Backround:** This study was conducted within Generation R Study, a population-based cohort study. 3,041 whole-body Dixon MRIs from adolescents aged 13 years were used for abdominal adipose tissue segmentation. A previously published Competitive Dense Fully Convolutional Network (CDFNet) was retrained on 62 individuals. Segmentation accuracy was assessed via the Dice score and volumetric similarity (VS) on eight individuals. Furthermore, a subset of 504 automated segmentations was

Friday evaluated visually by two experienced observers scoring under- and over-segmentation rate (scale 0-3), using intraclass correlation coefficient (ICC) to assess inter-observer agreement.
**Results or Findings:** The mean Dice score for SAT and VAT was 0.95 and 0.85, and VS for SAT and VAT was 0.98 and 0.93. The mean under- and over-segmentation rates of the two observers and the agreement between them were 2.92 (ICC 0.931) and 2.99 (ICC 0.922) for SAT, and 2.99 (ICC 0.956) and 2.95 (ICC 0.778) for VAT respectively, indicating excellent segmentation quality across individuals. The correlation coefficients of VAT with SAT, fat mass and BMI were 0.851, 0.801 and 0.698, respectively.
**Conclusion:** We retrained and evaluated a deep-learning method to accurately and automatically extract abdominal SAT and VAT volumes from whole-body MRIs of adolescents. These volumes will be used to study determinants of body composition in the Generation R Study.
**Limitations:** In longitudinal studies of children and adolescents such as Generation R, the CDFNet model must be retrained and validated for each follow-up time point representing different ages.
**Ethics committee approval:** The medical ethics committee of the Erasmus MC, University Medical Center in Rotterdam approved this study. The approval can be found under number MEC-2012-165-NL40020.078.12.
**Funding for this study:** TW is supported by China Scholarship Council (CSC) PhD Fellowship.
**Author Disclosures:**
Stefan Klein: Nothing to disclose
Edwin Oei: Nothing to disclose
Santiago Estrada: Nothing to disclose
Ruisheng Su: Nothing to disclose
Vincent Jaddoe: Nothing to disclose
Renza Van Gils: Nothing to disclose
Tong Wu: Nothing to disclose

### RPS 1305-5

### MRI-based artificial intelligence (AI) for abdominal adipose tissue segmentation: a meta-analysis

*N. Andjelic*[1], J. T. Heverhagen[2], M. Mitrakovic[2], K. Daneshvar Ghorbani[2]; [1]Sremska Kamenica/RS, [2]Bern/CH
*(nikola.andjelic92@gmail.com)*

**Purpose:** Manual segmentation of subcutaneous and visceral fat tissue on MRI to calculate body composition parameters is difficult. AI allows for a chance to automate this operation. Through systematic review and meta-analysis, we assessed the available AI segmentation solutions based on MRI and their role in body composition analysis.
**Methods or Backround:** We systematically searched PubMed (MEDLINE), Embase and Scopus. We extracted the studies about using MRI-based AI models for subcutaneous (SAT) and visceral adipose tissue (VAT) segmentation, published from January 1, 2011, to October 20, 2022. Studies that used CT and other imaging modalities were excluded. The reported dice similarity coefficient (DSC) of AI models was used for meta-analysis.
**Results or Findings:** A total of 213 studies were identified. Among them, 26 could be included in the systematic review, of which eight were included in the meta-analysis. Machine-learning models for SAT and VAT segmentation showed a combined DSC of 0.971 (95 %CI 0.962–0.980) and 0.960 (95 %CI 0.951–0.969), respectively. Considerable heterogeneity of included studies was observed.
**Conclusion:** MRI-based AI models are promising tools for automated subcutaneous and visceral adipose tissue segmentation. More definitive guidelines are necessary before their integration into the routine clinical workflow.
**Limitations:** Caution should be applied when interpreting results, for they could be skewed by significant heterogeneity between the studies. Also, segmentations were extracted from non-identical abdominal levels; therefore, the models would perform differently and gain different results across these levels. The review was focused solely on MRI-based models, ignoring other imaging modalities.
**Ethics committee approval:** No information provided by the submitter.
**Funding for this study:** No funding was received for this study.
**Author Disclosures:**
Johannes T. Heverhagen: Nothing to disclose
Milena Mitrakovic: Nothing to disclose
Keivan Daneshvar Ghorbani: Nothing to disclose
Nikola Andjelic: Nothing to disclose

### RPS 1305-6

### Abdominal adipose tissue segmentation and fat percentage calculation by using dual-neural network model

*B. Wen*[1], Y. Yuan[2], Y. Ao[2], L. Qiu[2], Z. Hu[1], D. Liang[1], X. Liu[1], H. Zheng[1], N. Zhang[1]; [1]Shenzhen/CN, [2]Yibin/CN

**Purpose:** This study designs a convolutional neural network-based dual-model system for fully automatically segmenting abdominal adipose tissue regions on computed tomography (CT) images. This system aims to assist radiologists to quickly and accurately determine the obesity status of patients by automatically segmenting abdominal adipose tissue and calculating its proportion.
**Methods or Backround:** In current clinical practice, an expert quantifies the fat tissue manually, which is time-consuming. Thus, designing a computer-aided method is essential. We prepared eight patients (2198 2D CT images) for model training; an expert radiologist finished delineating adipose tissue areas in the image. All images were resized, intensity values were normalised, and multiple quantitative metrics were calculated to evaluate the segmentation performance. We trained two models for different segmentation tasks, one is a three-class model that completes the segmentation of subcutaneous adipose tissue(SAT) and visceral adipose tissue(VAT), and the other is a simpler binary classification for segmenting the abdominal region. Accurately finding these three ROIs can calculate values closer to the actual fat percentage.
**Results or Findings:** Splitting SAT and VAT with mean-iu=0.972,dice=0.980,acc=0.986; predicting the abdominal region with mean-iu=0.982,dice=0.983, acc=0.997. We calculated the fat percentage of one of the patients; the fat percentage calculated using the annotated image is 49.90%, and using the model-predicted image calculation result is 50.63%. The results prove that the effect of model segmentation is reliable.
**Conclusion:** We successfully propose a CNN-based dual-model automatic segmentation method with high accuracy and stable performance. According to this method, we can further design and apply it to the clinical diagnosis of the fat status of the patient's body.
**Limitations:** Our model needs more clinical data to train to improve its robustness, and labelling patient data is a considerable workload. In future research, a method may be proposed to overcome this problem.
**Ethics committee approval:** No information provided by the submitter.
**Funding for this study:** Not applicable.
**Author Disclosures:**
Yongsheng Ao: Nothing to disclose
Xin Liu: Nothing to disclose
Hairong Zheng: Nothing to disclose
Lihua Qiu: Nothing to disclose
Yizhi Yuan: Nothing to disclose
Dong Liang: Nothing to disclose
Na Zhang: Nothing to disclose
Binbin Wen: Nothing to disclose
Zhanli Hu: Nothing to disclose

### RPS 1305-7

### Concordance between artificial intelligence (AI) computed tomography-based and bioimpedance-based analysis of body composition in a prospective study

*U. Fehrenbach*, N. L. Beetz, W. Wienbrandt, C. Maier, B. Globke, F. Tacke, T. C. Walter-Rittel, T. A. Auer, D. Geisel; Berlin/DE

**Purpose:** Body composition as an important indicator of patients’ frailty is increasingly recognised. The aim of this prospective trial is to compare body composition parameters obtained from the established bio impedance analysis (BIA) and computed tomography (CT) using an AI-based segmentation method.
**Methods or Backround:** We prospectively included 210 randomly selected patients who underwent contrast-enhanced CT imaging of the abdomen. Before the CT scan, all patients underwent BIA. For CT body composition analysis, an AI-based single-slice segmentation tool was used at the level of L3 (automated detection and segmentation). The BIA-based parameters body fat mass (BFM) and skeletal muscle mass (SMM), and the CT-parameters subcutaneous and visceral adipose tissue area (SATA and VATA) and skeletal muscle area (SMA) were obtained. Body fat percentage (BFP) and skeletal muscle index (SMI) were calculated by normalising the corresponding BIA and CT parameters to weight (BFP) or height (SMI). Parameters representing muscle or fat tissue were gender-specifically evaluated.
**Results or Findings:** Parameters representing fat and muscle tissue, BIA-based BFM and CT-based SATA+VATA, as well as BIA-based SMM and CT-based SMA, showed a strong correlation in female (fat: r=0.95; muscle: r=0.72) and male (fat: r=0.91; muscle: r=0.71) patients (p<0.001). Regression analysis was statistically significant and showed that BFP-CT and LSMI-CT could statistically significantly predict BFP-BIA and SMI-BIA for female (BFP: b1=5.29; CI95 [4.62-5.95]; SMI: b1=0.11; CI95 [0.09-0.13]) and male (BFP: b1=4.47; CI95 [3.97-4.97]; SMI: b1=0.09; CI95 [0.08-0.11]) patients (p<0.001).
**Conclusion:** AI-based CT body composition parameters strongly correlate with BIA and allow quantification of body fat percentage and skeletal muscle index comparable to the existing gold standard. Thus, an additional BIA appears unnecessary in patients who received an abdominal CT scan as part of their diagnostics.
**Limitations:** The study's main limitation is its small patient cohort with various diseases.
**Ethics committee approval:** The study was approved by Charité's institutional ethics committee (EA4/162/21).
**Funding for this study:** No funding was received for this study.
**Author Disclosures:**
Nick Lasse Beetz: Nothing to disclose

Thula Cannon Walter-Rittel: Nothing to disclose
Frank Tacke: Nothing to disclose
Brigitta Globke: Nothing to disclose
Christoph Maier: Nothing to disclose
Uli Fehrenbach: Grant Recipient: ESGAR, ESR, ECIO Speaker: Bayer, Siemens, IPSEN, Asahi Intecc, GE,
Dominik Geisel: Nothing to disclose
William Wienbrandt: Nothing to disclose
Timo Alexander Auer: Nothing to disclose

### RPS 1305-8

**Automatically derived CT-based body composition biomarkers correlate with overall survival in non-metastatic and metastatic non-small cell lung cancer patients**

*R. Hosch*, J. Haubold, S. Koitka, J. Keyl, K. Darwiche, S. Bölükbas, M. Stuschke, M. Schuler, F. Nensa; Essen/DE
*(rene.hosch@uk-essen.de)*

**Purpose:** Recently, the interest in body composition analysis (BCA) in oncology has increased. With deep learning advances for medical imaging, using CT-based BCA biomarkers could contribute to oncological staging. We investigated the impact of BCA biomarkers on the overall survival (OS) of metastatic (M1) and non-metastatic (M0) non-small cell lung cancer (NSCLC) patients.
**Methods or Backround:** Baseline CT thorax scans of 1914 NSCLC patients (783 female, 1131 male) diagnosed between 2015 and 2021 within +-60 days from the initial diagnosis were included. The collective consists of 1175 M0 and 739 M1 patients. Subsequently, BCA parameters were acquired using an in-house automated deep learning-based system and combined into the following three features: sarcopenia (muscle/bone), myosteatosis (inter- and intramuscular fat/total fat), and cardiac ((epicardial fat+paracardial fat)/total fat). The resulting biomarkers were analysed using the Kaplan-Meier method and multivariate cox-regression adjusted for age and gender.
**Results or Findings:** In both groups, preliminary Kaplan-Meier analyses indicated higher OS rates for NSCLC patients with a higher muscle-to-bone ratio ($pM0 \leq 0.001$, $pM1=0.003$), lower skeletal muscular fatty infiltration ($pM0 \leq 0.0001$, $pM1 \leq 0.001$), and lower cardiac fat ($pM0 \leq 0.001$, $pM1 \leq 0.001$). Additionally, the adjusted Cox regression indicated that sarcopenia ($p \leq 0.0001$), myosteatosis ($p \leq 0.0001$), and cardiac ($p \leq 0.001$) features were significant for non-metastatic patients. Moreover, these findings were applied to metastatic patients for sarcopenia ($p \leq 0.0001$), myosteatosis ($p \leq 0.001$), and cardiac fat ($p=0.032$).
**Conclusion:** BCA features are important biomarkers regarding the OS of NSCLC patients and could contribute valuable information during the clinical staging process.
**Limitations:** More clinical variables, such as laboratory or ECOG, should be added for future analysis. A multi-centre study would be desirable for further validation.
**Ethics committee approval:** This study was performed in adherence to all guidelines defined by the approving institutional review board of the investigating hospital. The Institutional Review Board waived written informed consent due to the study's retrospective nature. Complete anonymisation of all data was performed before inclusion in the study.
**Funding for this study:** No funding was received for this study.
**Author Disclosures:**
Julius Keyl: Nothing to disclose
Martin Stuschke: Nothing to disclose
Johannes Haubold: Nothing to disclose
Servet Bölükbas: Nothing to disclose
Sven Koitka: Nothing to disclose
Martin Schuler: Nothing to disclose
Kaid Darwiche: Nothing to disclose
René Hosch: Nothing to disclose
Felix Nensa: Nothing to disclose

### RPS 1305-9

**AI-based quantification of muscle volume in CT: an evaluation of different field of view**

*P. Borrelli*[1], T. Ying[2], O. Enqvist[3], J. Ulén[3], R. Kaboteh[1], E. Trägårdh[4], H. Kjölhede[2], L. Edenbrandt[1]; [1]Göteborg/SE, [2]Gothenburg/SE, [3]Malmö/SE, [4]Lund/SE
*(pablo.borrelli@vgregion.se)*

**Purpose:** Sarcopenia is associated with cancer-specific survival and complications. Skeletal muscle volume on CT is therefore clinically relevant to quantify. Manual quantification is time-consuming and, therefore, most commonly limited to only a single slice, for example, at the level of the third lumbar (L3) vertebra or the twelfth thoracic (Th12) vertebra. With AI-based methods, a 3D muscular volume can be segmented in less than a minute. This study aimed to assess how AI-based single-slice measurements or limited 3D volumes correlated with muscle volumes of the complete torso.
**Methods or Backround:** Skeletal muscle was automatically segmented by an AI-based method in CT from 998 patients (173 female/825 men) who had undergone CT of the entire torso for different clinical indications. Pre-defined sub-volumes and areas were calculated from the total torso segmentations. Thoracic muscle – volume from the top of the sternum (also automatically segmented) and 25cm in caudal direction; abdominal muscle – volume from the tip of the sacrum and 25cm in cranial direction; L3 and Th12 muscle areas. Linear regression analysis was used to assess the relations between the sub-volumes/areas and the total torso volume of muscle.
**Results or Findings:** The relationship between the thoracic and torso muscle volumes was significantly higher ($r2=0.89$) than between the single slice area of Th12 and torso volume ($r2=0.77$). The relationship between the abdominal and torso muscle volumes was significantly higher ($r2=0.94$) than between the single slice area of L12 and torso volume ($r2=0.83$).
**Conclusion:** Sub-volume of muscle provides a significantly more accurate estimation of total torso volume than single-slice measurements. AI-based 3D segmentations of skeletal muscle should replace manual single-slice measurements.
**Limitations:** The study is limited by the torso measurements used as substitute for whole-body measurements of muscle volume.
**Ethics committee approval:** Not applicable.
**Funding for this study:** No funding was received for this study.
**Author Disclosures:**
Thomas Ying: Nothing to disclose
Henrik Kjölhede: Nothing to disclose
Reza Kaboteh: Nothing to disclose
Pablo Borrelli: Nothing to disclose
Elin Trägårdh: Nothing to disclose
Olof Enqvist: Nothing to disclose
Johannes Ulén: Nothing to disclose
Lars Edenbrandt: Nothing to disclose

### RPS 1305-10

**Application of AI-software on CT-images for the evaluation of sarcopenia in patients with genitourinary tumours**

*A. Borrelli*, M. Pecoraro, M. Bicchetti, E. Messina, C. Catalano, V. Panebianco; Rome/IT
*(antonella.borrelli@uniroma1.it)*

**Purpose:** The study aimed to confirm the role of sarcopenia, assessed using an AI-software, as a prognostic predictor of response to therapy in genitourinary tumours and to correlate sarcopenia with treatment-related toxicity and oncological outcomes.
**Methods or Backround:** We retrospectively reviewed the medical records of 138 patients with a confirmed genitourinary cancer diagnosis before any anti-cancer treatment. Demographic characteristics were collected: gender, age, comorbidity, height, weight, BMI and radiological stage at diagnosis. An AI-powered software was used to quantify muscle mass by assessing the skeletal muscle cross-sectional area (SMA-L3) to obtain the skeletal muscle index (SMI). A multivariate logistic regression model was implemented to determine radiological and clinical features and body composition variables with an independent effect on sarcopenia and patient prognosis. Kaplan-Maier curves regression analysis was used to assess the association between sarcopenia and oncological outcomes.
**Results or Findings:** According to medical record data availability, a final cohort of 121 patients was enrolled. We included patients with prostate cancer ($n=16$), kidney cancer ($n=33$), urothelial carcinoma ($n=56$) and testicular cancer ($n=4$). The regression models showed how pre-treatment low SMA and low SMI ($p=0.001$ and $<0.001$, respectively) were associated with a higher probability of developing sarcopenia. Other predictor factors were represented by performance status and comorbidities. Sarcopenia at diagnosis has been shown to correlate with reduced progression-free survival and overall survival ($p=0,01$).
**Conclusion:** CT-based AI-powered models allow automated segmentation of body composition and aid sarcopenia diagnosis. Our results may suggest promoting standardised pathways to associate radiological staging of cancers with the analysis of the sarcopenia status and potentially direct patients to specific clinical assessment for personalised nutritional therapies, which might improve outcomes and quality of life.
**Limitations:** The study is limited by being a retrospective study and having a small sample size.
**Ethics committee approval:** The ethics committee was approved by the institutional review board.
**Funding for this study:** No funding was received for this study.
**Author Disclosures:**
Emanuele Messina: Nothing to disclose
Marco Bicchetti: Nothing to disclose
Valeria Panebianco: Nothing to disclose
Antonella Borrelli: Nothing to disclose
Martina Pecoraro: Nothing to disclose
Carlo Catalano: Nothing to disclose

12:30-13:30 Research Stage 1

### Research Presentation Session: Breast

### RPS 1402

### Radiomics-enhanced MRI

**Moderator**
B. Bennani-Baiti; Vienna/AT

### RPS 1402-2

**MRI-based radiomics approach predicts tumour recurrence in ER+/Her2- early breast cancer**

*G. Piccolo*[1], A. Delli Pizzi[1], R. Luberti[1], M. C. Torrione[1], M. Muzi[2], M. Caulo[1]; [1]Chieti/IT, [2]Ortona/IT
*(picc.gae@gmail.com)*

**Purpose:** Oncotype Dx is a genetic assay providing a recurrence score (RS) correlated with the risk of cancer recurrence and adjuvant treatment response in breast carcinoma. We investigated the ability of an MRI-based radiomics approach to predict the risk of tumour recurrence in breast cancer.
**Methods or Backround:** A total of 62 patients with biopsy-proved ER+/HER2- early breast cancer who underwent pretreatment MRI and Oncotype Dx were included. An RS>25 was considered discriminant between low-intermediate and high risk of tumour recurrence. Two readers segmented each tumour. Radiomics features were extracted from the tumour and the peritumoural tissues. Partial least square (PLS) regression was used as the multivariate machine-learning algorithm. PLS ßweights of radiomics features included the 5% of features with the largest ß-weights in magnitude (top 5%). Leave-one-out nested cross-validation (nCV) was used to achieve hyperparameter optimisation and evaluate the generalisable performance of the procedure. The diagnostic performance of the radiomics model was assessed through receiver operating characteristic (ROC) analysis. A null hypothesis probability threshold of 5% was chosen ($p<=0.05$).
**Results or Findings:** The nCV framework delivered an AUC of 0.76 ($p=1.1\cdot10$-3). The 47 features included in the top 5% were balanced between T and TST (23 and 24, respectively). Moreover, 33/47 (70%) were texture-related and 25/47 (53%) derived from high-resolution images (1 mm).
**Conclusion:** After a prospective evaluation in more extensive clinical trials, a radiomics-based machine learning approach may non-invasively identify patients who are more likely to benefit from adjuvant therapy.
**Limitations:** Limitations included: 1) the relatively low number of patients and lack of a validation cohort; 2) this was a retrospective single-center study; and 3) we only analysed dynamic contrast-enhanced MRI images, thereby excluding T2-weighted or diffusion-weighted images.
**Ethics committee approval:** No information provided by the submitter.
**Funding for this study:** No funding was received for this study.
**Author Disclosures:**
Andrea Delli Pizzi: Author: Author
Marzia Muzi: Author: Author
Gaetano Piccolo: Author: Presenter
Maria Concetta Torrione: Author: Author
Massimo Caulo: Author: Author
Riccardo Luberti: Author: Author

### RPS 1402-3

**Combining ultrafast MRI sequence with artificial intelligence (AI) for breast cancer detection**

B. Lokaj[1], A. Djema[1], K. Kinkel[2], C. Lovis[1], *J. Schmid*[1]; [1]Geneva/CH, [2]La Chaux-de-Fonds/CH
*(jerome.schmid@hesge.ch)*

**Purpose:** While having a higher sensitivity than mammography, multiparametric breast MRI has higher costs, longer acquisition times and limited availability. Therefore, abbreviated protocols and shorter sequences like ultrafast 4D are investigated. Some kinetic parameters derived from 4D ultrafast sequences have shown significant discrimination between benign and malignant lesions but possibly discard valuable image descriptors. AI can potentially provide valuable diagnostic support by handling this high-dimensional data.
**Methods or Backround:** From 4D ultrafast MRI examinations (n=415 lesions) collected from Clinique des Grangettes-Hirslanden with a Philips Ingenia 3T, two kinds of data were generated from the lesion segmentations – using 3D segmentations of the last subtracted phase (type A) or 3D MIP stacks (Type B). The performance of ResNet-50 and DenseNet-121 models were assessed on each kind of data.
**Results or Findings:** DenseNet-121 performed well (e.g. type A: accuracy=0.85, AUC=0.91, sensitivity=0.73, specificity=0.88), and was generally better than ResNet-50. Regardless of the model architecture, the data type did not really influence performances. Our results improved previous deep-learning classification studies involving MIP-like data (e.g., AUC improvement from 0.811 to 0.884).
**Conclusion:** Preliminary investigations show promising results despite the reduction of data dimensionality (single subtracted phase or MIP reconstructions). It is expected that the AI approaches fully exploiting the sequence 4D nature will improve classification performances.
**Limitations:** Further investigation of AI models is necessary to fully account for 4D data, which remains a technical challenge. We have yet to gather and process our multi-centre and multi-vendor data fully.
**Ethics committee approval:** This study was approved by Geneva State Ethics Committee (CCER). The approval can be found under the BASEC-ID 2019-00716.
**Funding for this study:** This work is part of the SUBREAM project funded by Swiss Cancer Research (Project n°KFS-5460-08-2021-R).
**Author Disclosures:**
Christian Lovis: Nothing to disclose
Amal Djema: Nothing to disclose
Jerome Schmid: Nothing to disclose
Belinda Lokaj: Nothing to disclose
Karen Kinkel: Nothing to disclose

### RPS 1402-4

**Radiomics for ultrafast dynamic contrast-enhanced MRI to predict prognostic biomarkers and subtypes of breast cancer: compared to conventional MRI in a single-centre prospective cohort**

*J-H. Jeong*[1], B. K. Seo[1], S-H. Lee[1], J. T. Lee[1], K. R. Cho[2], O. Woo[2], S. E. Song[2]; [1]Ansan/KR, [2]Seoul/KR
*(dwwfdg@gmail.com)*

**Purpose:** Ultrafast dynamic contrast-enhanced (DCE) MRI can detect early enhancement lesions before breast parenchymal enhancement, improving lesion conspicuity and shortening scan times. Few studies have investigated the predictive performance of ultrafast MRI-based radiomics in oncologic images. We investigated radiomics-based prediction of prognostic biomarkers and subtypes of invasive breast cancer using ultrafast DCE-MRI compared to conventional DCE-MRI.
**Methods or Backround:** This prospective study enrolled 116 patients who underwent both ultrafast and conventional MRI for breast cancer diagnosis. Two radiologists performed three-dimensional tumour segmentation independently on MRI (R1: 24 years' experience breast subspecialist, R2: radiology resident). We extracted 65 radiomic features from ultrafast and conventional images before and after contrast injection, and 3 kinetic parameters from ultrafast imaging. The accuracy of predicting prognostic biomarkers of breast cancer was analysed: estrogen receptor(ER), progesterone receptor(PR), human epidermal growth factor receptor 2 (HER2), and subtypes.
**Results or Findings:** The accuracy of R1 and R2 using ultrafast vs. conventional DCE-MRI radiomics to predict prognostic factors was as follows: ER, 71.3% vs. 64.4%; PR, 72.4% vs. 69.5%; HER2, 76.0% vs. 67.7%; and subtype, 72.8% vs. 67.2% for R1, and ER, 68.6% vs. 60.8%; PR, 71.0% vs. 68.4%; HER2, 70.0% vs. 68.9%; and subtype, 72.5% vs. 70.7% for R2. The performance using both radiomic features and kinetic parameters on ultrafast imaging improved ER, PR, and subtype prediction in both radiologists: ER, 74.7%; PR, 73.8%; and subtype, 76.4% for R1 and ER, 71.2%; PR, 74.3%; and subtype, 74.9% for R2.
**Conclusion:** The performance of ultrafast MRI-based radiomics was comparable to that of conventional MRI regardless of the radiologist's experience. In particular, adding the ultrafast MRI kinetic parameters improved the performance of radiomics-based hormone receptors and subtypes prediction.
**Limitations:** The small number of patients was a limitation.
**Ethics committee approval:** This study was approved by the Korea University Ansan Hospital Institutional Review Board.
**Funding for this study:** Funding was received from the Korean government (NRF-2021R1A2C1010565).
**Author Disclosures:**
Okhee Woo: Nothing to disclose
Bo Kyoung Seo: Nothing to disclose
Ju-Hyun Jeong: Nothing to disclose
Sung Eun Song: Nothing to disclose
Jeong Taek Lee: Nothing to disclose
Seung-Hak Lee: Nothing to disclose
Kyu Ran Cho: Nothing to disclose

**RPS 1402-5**

**Role of ultrafast dynamic contrast-enhanced MRI: prognostic imaging markers of breast cancer**

*S. Choe*, K. Kim, C. Hwang, J. Seo, Y. Kim; Daejeon/KR

**Purpose:** The purpose of this study was to determine whether ultrafast dynamic contrast-enhanced (DCE) MRI-derived early kinetic parameters using compressed sensing are associated with clinicopathological prognostic factors for breast cancer.
**Methods or Backround:** We evaluated 103 consecutive women with breast cancer (85 invasive carcinomas, 18 carcinomas in situ) who underwent both ultrafast DCE-MRI using compressed sensing (temporal resolution, 5-6 seconds; spatial resolution, 0.96×0.96×1.5mm) between January 2020 and December 2021. DCE-derived early kinetic parameters (time to enhancement (TTE) and maximum slope (MS)) were evaluated in breast lesions by two radiologists using a software programme and were correlated with clinicopathologic prognostic factors. The Mann-Whitney-U-test, linear regression model and independent t-test analysis were used.
**Results or Findings:** Ultrafast DCE-MRI-derived early kinetic parameters showed a strong relationship with some breast cancer characteristics, especially tumour size, histologic grade, axillary LN metastasis, and molecular subtypes. The TTE was significantly short in tumors larger than 2cm (p=0.001), invasive carcinoma than DCIS (p=0.003), higher histologic grade (p=0.029), positive axillary LN metastasis (p=0.029), ER-negative tumour (p=0.044), and Her-2 type&TNBC type (p=0.05). The MS was significantly larger in tumours larger than 2cm (p=0.008) and invasive carcinoma than in carcinoma in situ (p=0.007). The interobserver reliability between reviewers in evaluating TTE and MS was very good (k=0.95, 0.82).
**Conclusion:** Ultrafast DCE-MRI-derived parameters (TTE and MR) are strongly associated with some clinicopathologic prognostic factors and have a potential role in identifying highly aggressive breast cancer before invasive treatment.
**Limitations:** Our study has several limitations. First, it was a retrospective study in a single institution, which may have led to selection bias. Second, MRI examinations were performed after the biopsy procedure. So, post-biopsy changes or artefacts could have affected the MR signal. Third, the generalisability of our research is unclear due to the limited number of patients involved.
**Ethics committee approval:** Not applicable.
**Funding for this study:** No funding was received for this study.
**Author Disclosures:**
Keumwon Kim: Nothing to disclose
Jaeyoung Seo: Nothing to disclose
Youngjoong Kim: Nothing to disclose
Seoyeon Choe: Nothing to disclose
Cheolmog Hwang: Nothing to disclose

**RPS 1402-6**

**Analysis of radiomic features in the MRI assessment of the response to neoadjuvant chemotherapy in patients with triple-negative breast cancer**

*N. Damiani*, F. Galioto, M. Durando, G. Bartoli, V. Rossetti, E. Regini, I. Landolfi, P. Fonio, O. Rampado; Turin/IT
*(nic.dam91@gmail.com)*

**Purpose:** The study aimed to investigate the potential correlation between radiomic features and complete pathological response (pCR) to neoadjuvant chemotherapy (NAC) of triple-negative breast cancer (TNBC) at early, middle and end treatment breast MRI.
**Methods or Backround:** We conducted a retrospective analysis including 41 patients with newly diagnosed TNBC, who underwent dynamic breast contrast-enhanced (DCE) MRI and DWI before, during and post-NAC, 29 of whom had pCR. Using commercial software (Pyradiomics platform), after the segmentation of the lesions, 110 radiomic parameters were extracted. Radiomic metrics were evaluated in the fifth DCE sequence and then in the highest b-value (b:1000) DWI sequence. The variables evaluated were: pCR, complete radiological response, disease recurrence and exitus. A univariate analysis was performed using SRH test, and the statistical significance was set at p<0.05. For pCR and complete radiological response, a multivariate analysis was conducted with the elaboration of a correlation matrix. Possible models with multiple logistic regression were implemented, and ROC curves and the areas under curves (AUC) were calculated.
**Results or Findings:** We identified several features helpful in discriminating between pCR and no-pCR. The logistic regression model allowed us to improve these performances and extended the number of features predicting the characteristics of the lesion. From the multivariate analysis, we obtained predictive models derived from the combination of two or more features associated or not with the pre-therapy ki-67 index with AUC values between 0.84-0.95.
**Conclusion:** Our preliminary study suggests that MRI radiomic features could be helpful for predicting pCR to NAC in TNBC.
**Limitations:** Considering the small sample analysed, large prospective studies will be required to confirm the statistical significance of these findings.
**Ethics committee approval:** No information provided by the submitter.
**Funding for this study:** No funding was received for this study.
**Author Disclosures:**
Manuela Durando: Nothing to disclose
Nicolò Damiani: Nothing to disclose
Veronica Rossetti: Nothing to disclose
Osvaldo Rampado: Nothing to disclose
Paolo Fonio: Nothing to disclose
Elisa Regini: Nothing to disclose
Ilenia Landolfi: Nothing to disclose
Germana Bartoli: Nothing to disclose
Francesca Galioto: Nothing to disclose

**RPS 1402-7**

**AI-based anomaly detection identifies precursors of future lesions in breast MRI of high-risk women: a feasibility study**

*B. Burger*, M. Bernathova, P. Seeböck, C. Singer, P. Clauser, P. Baltzer, T. H. Helbich, G. Langs; Vienna/AT
*(bianca.burger@meduniwien.ac.at)*

**Purpose:** Breast magnetic resonance imaging (MRI) has been proposed as an additional screening test for women at high risk of breast cancer in whom mammography alone has poor sensitivity. In our study we tested the applicability of an AI-based anomaly detection algorithm for prediction of future lesions at MRI in high-risk women. Such unsupervised learning algorithms would avoid the need for annotated examples of lesions for training.
**Methods or Backround:** We trained a generative adversarial network on retrospectively collected dynamic contrast-enhanced MRI of 33 high-risk women who participated in a screening program but did not develop BC. The resulting model captured normal longitudinal variability of breast tissue appearance during follow-up. An anomaly score was defined as the deviation of observed breast MRI from the normal variability. We evaluated the association between this anomaly score calculated for breast MRI exams without lesions (BIRADS 1) and the emergence of lesions at a later time point in 31 women. Associations were analyzed by receiver operator characteristic (ROC) curves on local image patches (n=2x128), and logistic regression on the level of the entire breast MRI exam.
**Results or Findings:** The local patch-level anomaly score is a good predictor for future lesion emergence (mean area under the ROC curve 0.804). An exam-level summary score is significantly associated with the emergence of a lesion at any location at a later time point (p=0.045).
**Conclusion:** AI-based anomaly detection is a promising personalised screening tool for breast MRI in high-risk women. Applications include automatic preselection of negative screens for workload reduction and identification of precursors of lesions.
**Limitations:** Evaluation on additional subjects is required to underpin the findings of this feasibility study.
**Ethics committee approval:** No information provided by the submitter.
**Funding for this study:** Funding was received from the Comprehensive Cancer Center Forschungsförderung der Initiative Krebsforschung, MedUni Wien.
**Author Disclosures:**
Christian Singer: Nothing to disclose
Georg Langs: Founder: contextflow GmbH Speaker: Novartis Shareholder: contextflow GmbH
Philipp Seeböck: Nothing to disclose
Thomas H. Helbich: Nothing to disclose
Maria Bernathova: Nothing to disclose
Bianca Burger: Nothing to disclose
Pascal Baltzer: Nothing to disclose
Paola Clauser: Nothing to disclose

**RPS 1402-8**

**Preoperative assessment of axillary lymph-node involvement in breast cancer: a hybrid 18F-FDG PET/MRI radiomics and machine-learning study**

*V. Romeo*[1], P. Kapetas[2], P. Clauser[2], S. Rasul[2], R. Cuocolo[3], M. Caruso[1], T. H. Helbich[2], P. A. Baltzer[2], K. Pinker-Domenig[4]; [1]Naples/IT, [2]Vienna/AT, [3]Salerno/IT, [4]New York, NY/US
*(valeria.romeo@unina.it)*

**Purpose:** The study aimed to apply radiomics and machine learning (ML) to 18F-FDG PET/MRI images of breast cancer (BC) patients for the non-invasive prediction of axillary lymph node (LN) involvement.
**Methods or Backround:** In this prospective study, 103 patients with 114 BC lesions (negative LN=43, positive LN=71) underwent simultaneous 18F-FDG PET/MRI of the breast. Histology was used for LN standard or reference. Tumour size and quantitative diffusion (ADCmean), perfusion (mean transit time, plasma flow, the volume of distribution), and PET (SUVmax) parameters of BC lesions were first calculated. After image normalisation and discretisation, radiomics features (RF) were extracted from BC lesions on DCE-MRI, ADC, T2-w and PET images. The population was then split into a training (67%, negative LN=28, positive LN=45) and a test set (33%, negative

Friday

LN=14, positive LN=23). RF and quantitative parameters were selected through a multi-step process. Finally, a support vector machine (SVM) ML classifier was used to predict axillary LN involvement (presence/absence).
**Results or Findings:** A total of 4424 RF/quantitative parameters were extracted. After the selection process, only RF (n=14) extracted from DCE-MRI, PET, T2-w and ADC images were selected for the subsequent analysis. The SVM classifier obtained 74% and 73% accuracy in the training and test set, respectively. AUC, sensitivity, specificity, PPV and NPV in the test set were 0.76, 65%, 86%, 88% and 60%, respectively.
**Conclusion:** Radiomics and ML showed promises for the non-invasive assessment of axillary LN involvement in BC.
**Limitations:** The study is limited by being performed at a single institution and its sample size.
**Ethics committee approval:** Not applicable.
**Funding for this study:** This study received the following funding: (1) H2020 - Research and Innovation Framework Programme, PHC-11-2015 # 667211-2, (2) Jubiläumsfonds of the Austrian National Bank # Nr: 18207, and (3) Nr. 17186, the NIH/NCI Cancer Center Support Grant (P30 CA008748), the Breast Cancer Research Foundation and Susan G. Komen.
**Author Disclosures:**
Pascal A.T. Baltzer: Speaker: siemens Healthineers
Katja Pinker-Domenig: Consultant: Vara/Merantix Healthcare GmbH
Consultant: AURA Health Technologies GmbH
Valeria Romeo: Nothing to disclose
Thomas H. Helbich: Speaker: Guerbet Speaker: siemens Healthineers
Speaker: novomed
Sazan Rasul: Nothing to disclose
Panagiotis Kapetas: Nothing to disclose
Renato Cuocolo: Nothing to disclose
Martina Caruso: Nothing to disclose
Paola Clauser: Speaker: Siemens healthineers

**12:30-13:30** **Research Stage 2**

### Research Presentation Session: Interventional Radiology

### RPS 1409

### Neuroradiological interventions

**Moderator**
S. Duvnjak; Odense/DK
*(duvnjak.stevo@gmail.com)*

### RPS 1409-2

**Aneurysm wall enhancement of coiled intracranial aneurysms is associated with aneurysm remnants**
*S. L. Leber*, E. M. Hassler, W. Renner, J. Igrec, J. Steiner, M. Feichtinger, M. Pohl, H. Deutschmann, G. Reishofer; Graz/AT
*(stefan.leber@medunigraz.at)*

**Purpose:** Wall enhancement of untreated intracranial aneurysms (AWE) is suspected to be associated with aneurysm instability. The significance of AWE or enhancement of the aneurysm cavity (ACE) in coiled intracranial aneurysms is discussed controversially in the literature, as either healing mechanisms or adverse inflammatory processes might be the cause. Since studies on this topic are scarce, we aimed to compare these phenomena between coiled aneurysms with complete occlusion and coiled aneurysms with remnants.
**Methods or Backround:** We included 60 patients after coil embolisation for ruptured or unruptured intracranial aneurysms in our study and evaluated aneurysms for remnants, AWE and ACE with magnetic resonance vessel wall imaging at 3 Tesla. We then dichotomised our cohort with regards to occlusion state and compared the incidence of AWE and ACE of completely occluded aneurysms (n=22) to aneurysms with remnants (n=38) by chi square test.
**Results or Findings:** Our analysis shows a significantly higher incidence of AWE in coiled aneurysms with remnants compared to completely occluded aneurysms (p= 0.036). This phenomenon seems to be more frequent in coiled aneurysms with larger remnants. The incidence of enhancement of the aneurysm cavity did not differ significantly between both groups (p= 0.712).
**Conclusion:** Our study provides novel insights into enhancement patterns of coiled intracranial aneurysms. Our data supports the hypothesis that AWE of coiled aneurysms is associated with aneurysm recurrence rather than a phenomenon of aneurysm healing alone.
**Limitations:** Although our results are genuine, our data from this retrospective study has to be interpreted carefully. Comparisons between independent cohorts and prospective studies are needed for validation.
**Ethics committee approval:** The study was approved by the IRB of the Medical University of Graz.
**Funding for this study:** No funding was received for this study.
**Author Disclosures:**
Jakob Steiner: Nothing to disclose
Wilfried Renner: Nothing to disclose
Eva Maria Hassler: Nothing to disclose
Hannes Deutschmann: Nothing to disclose
Maximilian Pohl: Nothing to disclose
Stefan L. Leber: Nothing to disclose
Mira Feichtinger: Nothing to disclose
Jasminka Igrec: Nothing to disclose
Gernot Reishofer: Nothing to disclose

### RPS 1409-3

**Comparative analysis of CT angiography and CT perfusion to DSA for collateral status in patients with acute large vessel ischaemic stroke and to correlate the collateral status with clinical outcome**
*H. K. Kamepalli*[1], V. S. V. G. Kalaparti[2], K. Santhosh Kumar[1], J. Er[1], C. Kesavadas[1]; [1]Kerala/IN, [2]Thiruvananthapuram/IN
*(haki.kamepalli@gmail.com)*

**Purpose:** The objective of this study was to compare the collateral status of CTA single phase, CTA triple phase, and CT perfusion to digital subtraction angiography in acute ischaemic stroke secondary to intracranial large vessel occlusion and to correlate these collaterals scores with 90-day functional outcome (modified Rankin Scale).
**Methods or Backround:** Good collateral flow is associated with better clinical outcome and may help for proper patient selection. Retrospective and prospective acute ischaemic stroke patients who underwent CTA, CT perfusion, and DSA before endovascular treatment (EVT) for occlusion of intracranial ICA, M1 and/or M2 segments were included. 150 patients total were included and the modified American Society of Interventional and Therapeutic Neuroradiology score (mASITN/SIR) was used to assess collateral status. Imaging assessments were related to the modified Rankin Scale.
**Results or Findings:** Higher mASITN/SIR score on CT single phase (Odds ratio :1.7) and triple phase CT angio (Odds ratio:2.2) are associated with better functional outcome (mRS score ≤ 2). Although collateral score of CT perfusion has more concordance (0.42) with DSA compared with triple phase (0.38) and single phase (0.16) CT angiography, it is not correlating with functional outcome. Similarly, better inter-observer agreement is seen with CT single phase (0.72) and CT triple phase (0.62), compared with CT perfusion (0.49) & DSA (0.42).
**Conclusion:** Collateral score evaluated on 3-phase and single CTA was associated with better 90-day functional outcome compared with CT perfusion and DSA and, moreso, with triple phase CTA, apart from inter-observer agreement. So CTA, especially triple phase CT angio, is better than CT perfusion and DSA to predict better functional outcome.
**Limitations:** Confounding factors like diabetes, hypertension, and IV thrombolysis, which may influence the outcome, are not equally distributed between the groups. There was also selection bias because of the exclusion of subjects with MRI screening.
**Ethics committee approval:** No information provided by the submitter.
**Funding for this study:** No funding was received for this study.
**Author Disclosures:**
Viswanadh Sri Venkata Ganesh Kalaparti: Nothing to disclose
Chandrasekharan Kesavadas: Nothing to disclose
Hari Kishore Kamepalli: Nothing to disclose
Kannath Santhosh Kumar: Nothing to disclose
Jayadevan Er: Nothing to disclose

### RPS 1409-4

**autoTICI: automated TICI scoring in ischaemic stroke patients**
*M. van der Sluijs*, R. Su, S. A.P. Cornelissen, T. van Walsum, A. Van Der Lugt, M. C. R. Investigators; Rotterdam/NL
*(p.vandersluijs@erasmusmc.nl)*

**Purpose:** Extended Thrombolysis in Cerebral Infarction (eTICI) score is used in Digital Subtraction Angiography to quantify reperfusion grade in endovascular treated (EVT) ischaemic stroke patients. In a recent paper, an automatic TICI score (autoTICI), which quantifies the ratio of reperfused pixels after EVT, proved to have a comparable clinical outcome prediction concerning dichotomised modified Rankin Scale (mRS) at 90 days. We aim to study the performance of AutoTICI in a larger sample size, including M2 occlusions.
**Methods or Backround:** Patients in the MR CLEAN Registry with an ICA, M1 and M2 occlusion were selected if both anterior-posterior (AP) and lateral views were present in pre- and post-EVT DSAs with more than 6 frames. Outcomes were correlated with eTICI and ordinal/binary regression analyses of 90-day mRS score. The influence of occlusion location on the relation of autoTICI with outcome was determined using an interaction term.
**Results or Findings:** In total, 708 of 3180 patients were included. AutoTICI correlated moderately with eTICI (ρ=0.57) and was an independent predictor of mRS (acOR 1.12 95%CI, 1.05-1.21) per 10% increase. The AUC predicting functional independence (mRS 0-2) was comparable with eTICI (0.64 vs 0.65,

Friday p=0.65). We found significant interaction of autoTICI with the occlusion location on the mRS scale, in the AP and lateral view. The effect of autoTICI on mRS in M1/M2 occlusions is reduced in AP, while increased in lateral view.
**Conclusion:** AutoTICI proves to have similar predictable applications as eTICI when adjusting for prognostic factors. The effect size of autoTICI score depends on the occlusion location.
**Limitations:** Exclusion of large proportion of patients due to no availability of required DSA sequences, which could potentially lead to selection bias.
**Ethics committee approval:** This study was approved by the Central medical ethics committee Erasmus Medical Center Rotterdam: MEC-2014–235.
**Funding for this study:** Funding was received from the TWIN Foundation and the Health-Holland and Philips Healthcare, Q-Maestro project Grant EMCLSH19006.
**Author Disclosures:**
Theo van Walsum: Nothing to disclose
Ruisheng Su: Nothing to disclose
Aad Van Der Lugt: Nothing to disclose
MR CLEAN Registry Investigators: Nothing to disclose
Sandra A.P. Cornelissen: Nothing to disclose
Matthijs van der Sluijs: Nothing to disclose

### RPS 1409-5

**Safety and angiographic efficacy of rescue intra-arterial thrombolysis for improvement of final TICI score after thrombectomy**

*V. Opancina*[1], M. Muto[2], F. Giordano[2], G. Leone[2], M. Muto[2], E. Spina[2], P. Candelaresi[2]; [1]Kragujevac/RS, [2]Naples/IT

**Purpose:** The main objective of this study was to examine the safety and angiographic efficacy of rescue intra-arterial thrombolysis for improvement of final TICI score after thrombectomy.
**Methods or Backround:** Endovascular thrombectomy is the standard treatment in selected patients with acute ischaemic stroke and large vessel occlusion, but continuous improvement in angiographic and clinical outcome is still needed. Intra-arterial thrombolysis has been tested as a possible rescue tool in unsuccessful thrombectomy, or as an adjuvant therapy after the endovascular procedure, to pursue complete recanalisation. The study was designed as a cross-sectional study, performed at Stroke Unit, AORN Antonio Cardarelli, Napoli, from June-July 2022. The study included all patients, 18 and older, who were diagnosed with stroke and underwent unsuccessful thrombectomy, defined as TICI score ≤2b. The patients were treated with intra-arterial Alteplase administration (up to 15 mg), with the aim of improving recanalisation. The main outcome was final TICI score, while independent and cofounding variables included: socio-demographic data, clinical manifestations, CT findings, and angiographic findings. Secondary outcomes were mortality and modified-Rankin-Scale scores at 3 months. The impact of these variables on the main outcome was investigated by descriptive statistical analysis and logistic regression.
**Results or Findings:** Of 150 screened patients, 10 received intra-arterial Alteplase . An improvement of final TICI score was achieved in 7/10 patients (70%), which was associated with mRS ≤2 (aOR, 3.1; 95% CI, 1.4 to 6.9). Also, a major effect of intra-arterial Alteplase was observed for distally migrated emboli. None of the patients experienced any symptomatic haemorrhagic transformation or other major bleeding.
**Conclusion:** Our study shows a high rate of improvement of final TICI score, encouraging the development of randomised controlled trials of rescue intra-arterial thrombolysis in unsatisfactory angiographic results.
**Limitations:** The small sample size was a limitation.
**Ethics committee approval:** The study was approved by the Ethics Comittee of AORN Antonio Cardarelli, Napoli.
**Funding for this study:** No funding was received for this study.
**Author Disclosures:**
Valentina Opancina: Nothing to disclose
Paolo Candelaresi: Nothing to disclose
Mario Muto: Nothing to disclose
Giuseppe Leone: Nothing to disclose
Massimo Muto: Nothing to disclose
Flavio Giordano: Nothing to disclose
Emanuele Spina: Nothing to disclose

### RPS 1409-6

**Middle meningeal artery embolisation as treatment for chronic subdural haematoma: efficacy and methods**

*A. Salcuni*, A. Di Ruzza, F. Ricchetti, L. Milonia, C. Cirelli, C. Gaudino, M. Iacobucci, F. Biraschi; Rome/IT

**Purpose:** To report the experience of a single centre in the endovascular treatment of chronic subdural haematoma (SDH).
**Methods or Backround:** A retrospective analysis of patients with SDH, undergone to both neurosurgical and endovascular treatment from 2020 and 2022, was performed. In agreement with neurosurgical colleagues, the asymptomatic or paucisymptomatic patients without indication for surgical treatment or with an unsatisfactory result after surgical treatment were addressed to endovascular treatment. All patients underwent a neuroradiological examination at least 30 days after the procedure. Patients without neuroradiological improvement of the SDH or who required neurosurgical or endovascular re-treatment were considered as failures.
**Results or Findings:** A total of 51 SDH were treated by embolisation of the middle meningeal artery (MMA). Ten patients underwent embolisation after neurosurgical treatment (28%), while the remaining patients (78%) were treated during steroid therapy. Five patients were excluded from the endovascular treatment due to the presence of dangerous anastomoses between the intra- and extra-cranial circulation. A complete embolisation of the target arterial branches was achieved in 46 SDH. Among these 46 SDH, polyvinyl alcohol hydrogel particles were used in 35 subjects and embolising material based on ethylene vinyl alcohol copolymer in 11 patients.
**Conclusion:** MMA embolisation seems to represent a safe and effective approach in the treatment of SDH, without significant differences in clinical improvement, complications, and need for re-intervention when compared to conventional treatment. From our experience and given the curative effect of endovascular treatment in SDH, a primary role of embolisation of the MMA seems to emerge, especially in patients who are paucisymptomatic or have contraindications to surgery.
**Limitations:** Not very representative statistical sample and not standardized endovascular approach.
**Ethics committee approval:** -
**Funding for this study:** No funding was received for this study
**Author Disclosures:**
Francesca Ricchetti: Nothing to disclose
Luca Milonia: Nothing to disclose
Marta Iacobucci: Nothing to disclose
Francesco Biraschi: Nothing to disclose
Andrea Salcuni: Nothing to disclose
Alberto Di Ruzza: Nothing to disclose
Chiara Gaudino: Nothing to disclose
Carlo Cirelli: Nothing to disclose

### RPS 1409-7

**Large culprit plaque and more carotid calcified plaques are associated with low radial expansion rate of carotid artery stent by high-resolution magnetic resonance vessel wall imaging**

*M. Y. Sun*; Beijing/CN

**Purpose:** The radial expansion rate of the carotid artery stenting (CAS) has not been well researched. This study investigated the association between carotid plaque imaging features and the radial expansion rate of CAS.
**Methods or Backround:** A total of 89 patients who were treated with CAS for atherosclerotic carotid stenosis were retrospectively collected and categorized into the low radial expansion rate and high radial expansion rate groups of CAS based on the calculations of the carotid angiography. Patients underwent high-resolution magnetic resonance vessel wall imaging (HR-VWI) and quantitative plaque morphology and composition analysis. The clinical records and HR-VWI imaging features were compared between the two groups. Logistic regression analyses were performed to determine the independent factors associated with the low radial expansion rate of CAS.
**Results or Findings:** Among 89 patients, 46 (51.7%) developed a low radial expansion rate of CAS. The logistic regression analysis detected Max wall thickness (OR,1.75; 95% CI, 1.02-3.00, P=.04), Max circumference score of calcification (OR,2.79, 95% CI 1.06 - 7.37, P=.04), and Max area percentage of calcification (OR 1.20, 95% CI 1.04 - 1.39, P=0.01) as predictors of the low radial expansion rate of CAS. In addition, the combination of three factors increased the area under the curve to 0.90.
**Conclusion:** Large culprit plaque with larger circumference degree and area of calcification were independently associated with a low radial expansion rate of CAS. Preoperative HR-VWI plaque evaluation is important for a favorable CAS outcome.
**Limitations:** No limitations were identified.
**Ethics committee approval:** 2022135X
**Funding for this study:** Funding was received from the Natural Science Foundation of Beijing Municipality (grant No. 7222047) and the International Medical Foundation of China (grant No. Z-2014-07-2101).
**Author Disclosures:**
Meng Yu Sun: Nothing to disclose

**12:30-13:30 Research Stage 3**

### Research Presentation Session: Oncologic Imaging

### RPS 1416

### Multicentric research in oncological imaging

**Moderator**
D. Regge; Turin/IT
**Author Disclosures:**
Daniele Regge: Advisory Board: Health Triage; Consultant: Radmetrix

### RPS 1416-2

**Diagnostic value of dynamic contrast-enhanced MRI for the detection of clinically significant prostate cancer in a multi-reader study: preliminary results from the PI-CAI consortium**

*J. J. Twilt*[1], A. Saha[1], J. Bosma[1], D. Yakar[2], M. Elschot[3], J. Veltman[4], J. J. Futterer[1], H. Huisman[1], M. De Rooij[1]; [1]Nijmegen/NL, [2]Groningen/NL, [3]Trondheim/NO, [4]Almelo/NL

**Purpose:** The study aimed to compare the detection performance of ISUP grade group (GG) ≥ 2 using bi-parametric (bp)MRI, and multi-parametric (mp)MRI (which includes dynamic contrast IV administration), in an international reader study.
**Methods or Backround:** This retrospective reader study uses a cohort of 400 prostate mpMRI exams acquired between 2012–2021 at three Dutch and one Norwegian centres. Patients suspected of GG≥2 cancers and without prior prostate treatment or prior GG≥2 findings in an MRI-first approach are included. 65 radiologists (18 countries, 43 centres) with 1–23 years (median: 8) prostate reading experience were enlisted. Readers and cases are divided into blocks of 100 cases. For each case, readers assessed bpMRI and mpMRI in sequence to mimic the clinical routine. Suspected GG≥2 cancer findings were assigned a PI-RADS 3-5 score. Additionally, a patient-level suspicion score (0-100) of harbouring GG≥2 was indicated. Multi-reader multi-case (MRMC) analysis was used to compare the patient-level added value of mpMRI.
**Results or Findings:** Preliminary results from the first 14 readers with 2–15 years (median: 9) of experience indicate that overall, there is little improvement in GG≥2 detections between bpMRI and mpMRI readings with AUROCs of 0.857 (95% CI: 0.83, 0.89) and 0.860 (95% CI: 0.83, 0.89), respectively. For individual readers, absolute differences in AUROC ranged between 0.00–0.03 (95% CI: 0.00, 0.01).
**Conclusion:** MRI assessments in bpMRI had similar GG≥2 detections to mpMRI assessments at a per-case level. Multivariable influencers such as experience, workflow, image quality and protocol familiarity must be evaluated.
**Limitations:** Preliminary results are limited by the sample size. mpMRI readings of the original data were used to guide histologic verification. 13 out of 14 readers had high expertise as per 2020 ESUR/ESUI consensus statements.
**Ethics committee approval:** The study was performed in accordance with institutional review board requirements, and all institutional review boards waived the need for informed consent for the retrospective reuse of anonymised clinical data.
**Funding for this study:** The study was funded by the EU Horizon 2020: ProCAncer-I (grant number 952159).
**Author Disclosures:**
Joeran Bosma: Nothing to disclose
Henkjan Huisman: Grant Recipient: Siemens Healthineers, Health Holland, NWO
Anindo Saha: Nothing to disclose
Matthijs Elschot: Nothing to disclose
Derya Yakar: Grant Recipient: Siemens Healthineers, Health Holland, NWO Research/Grant Support: MDPI Life Journal Consultant: Astellas
Jurgen J. Futterer: Grant Recipient: Siemens Healthineers, Health Holland, NWO
Jeroen Veltman: Nothing to disclose
Maarten De Rooij: Nothing to disclose
Jasper Jonathan Twilt: Nothing to disclose

### RPS 1416-3

**Nationwide accuracy of CT for local staging of colon cancer**

*J. Shkurti*[1], R. G. H. Beets-Tan[1], J. Nederend[2]; [1]Amsterdam/NL, [2]Eindhoven/NL
*(j.shkurti@nki.nl)*

**Purpose:** Neoadjuvant chemotherapy for high-risk colon cancer has shown improved patient outcomes. Hence, accurate clinical staging becomes essential in selecting patients for neoadjuvant chemotherapy. Current Dutch guidelines recommend chemotherapy for pT4Nx and/or pTxN+ cancer. The aim of our study is to determine the accuracy of CT in detecting high-risk colon cancer patients.
**Methods or Backround:** Patients aged ≥ 18 years diagnosed with non-metastatic primary colon cancer in 2005-2020, who underwent primary surgical resection without neoadjuvant chemotherapy, were selected from the Netherlands Cancer Registry. Sensitivity and specificity analyses were conducted for the prediction of T3-T4 and N1-N2 stages. Additionally, the proportion of patients within a specific clinical stage eligible for adjuvant chemotherapy according to Dutch guidelines was calculated.
**Results or Findings:** A total of 44,489 patients were included. The median age was 71 years, 50% were female. Tumour location was right-sided in 46% of the patients, left-sided in 44% and transverse colon in 8%. Histology was adenocarcinoma in 86%, mucinous in 12%, and signet cell in 1%. The sensitivity and specificity of CT to discriminate T3-T4 from T1-T2 tumours were 80% and 76%, respectively, with PPV 92%, NPV 51%, and accuracy 79%. Similarly, the sensitivity and specificity of CT to detect N1-N2 were 62% and 70%, respectively, with PPV 60%, NPV 72%, and accuracy 67%. The proportion of patients that were possibly eligible for adjuvant chemotherapy was 30% of T2N0 (2,118/7,048), 37% of T3N0 (3,126/8,487), 64% of T4N0 (690/1,075), 56% of T2N+ (863/1,549), 60% of T3N+ (4,148/6,969), and 78% of T4N+ (2,359/3,021).
**Conclusion:** The nationwide accuracy of CT for T and N staging was comparable to the literature. Selecting patients for neoadjuvant chemotherapy based on CT following current Dutch guidelines can lead to significant overtreatment depending on the clinical stage.
**Limitations:** The study is limited by its retrospective design.
**Ethics committee approval:** No information provided by the submitter.
**Funding for this study:** No funding was received for this study.
**Author Disclosures:**
Jona Shkurti: Nothing to disclose
Regina G. H. Beets-Tan: Nothing to disclose
Joost Nederend: Nothing to disclose

### RPS 1416-4

**MRI-guided active surveillance without rebiopsies in patients with ISUP grade group 1 and 2 prostate cancer: the PROMM-AS study**

*B. Valentin*, J. P. Radtke, M. Boschheidgen, C. Arsov, G. Antoch, P. Albers, L. Schimmöller; Düsseldorf/DE

**Purpose:** The study aimed to assess the ability of mpMRI to risk stratify men with ISUP grade group (GG) 1 and 2 prostate cancer (PC) on AS to reduce guideline-mandated biopsy and to predict ISUP GG upgrading.
**Methods or Backround:** In this prospective single-centre cohort study, patients with ISUP GG 1 and 2 PC after MRI/TRUS fusion-guided biopsy were included, and the two-year outcome of a mpMRI-guided AS protocol (PROMM-AS) were analysed. All men underwent mpMRI 12 months after inclusion. In the case of stable mpMRI according to PRECISE criteria, rebiopsy was deferred, and the follow-up mpMRI after 24 months was performed. In the case of mpMRI progression or at the end of the study, a follow-up MRI/TRUS fusion-guided biopsy was indicated. Sensitivity, specificity, positive (PPV) and negative predictive values (NPV) for ISUP GG upgrading on MRI were calculated, and regression analyses were performed.
**Results or Findings:** In total, 101 men (60 with GG1 and 41 with GG2 PC) were allocated. Histopathological progression occurred in 29 men. In the GG1 subgroup, 18 men had progression, whereas 11 men progressed in the GG2 subgroup. Sensitivity, specificity, PPV and NPV for PRECISE were 94%, 64%, 81% and 88% in the GG1 and 91%, 50%, 91% and 50% in the GG2 subgroup. On regression analysis, higher PRECISE scores (4-5), initial PI-RADS, PSA, age and prostate volume were significant predictors of histological progression in ISUP GG1 and higher PRECISE score, initial PI-RADS and previous negative biopsy in ISUP GG2 PC.
**Conclusion:** MRI-guided monitoring of men on AS, including PRECISE criteria, avoids unnecessary follow-up biopsies in 88% of men with ISUP GG1 and sufficiently predicts GG upgrading over a follow-up period of two years in both ISUP GG1 and GG2.
**Limitations:** The study is limited by being a single-centre study and with limited follow-up time.
**Ethics committee approval:** The institutional review board approved the study (ID 2017034168).
**Funding for this study:** No funding was received for this study.
**Author Disclosures:**
Birte Valentin: Nothing to disclose
Christian Arsov: Nothing to disclose
Jan Philipp Radtke: Nothing to disclose
Peter Albers: Nothing to disclose
Matthias Boschheidgen: Nothing to disclose
Lars Schimmöller: Nothing to disclose
Gerald Antoch: Nothing to disclose

### RPS 1416-5

**CT radiomic prognostic features in non-small cell lung cancer (NSCLC) in a prospective multicentre cohort: is there added value?**

R. O'Shea, T. Manickavasagar, *C. Horst*, D. J. Hughes, A. Bille, E. Karapanagiotou, G. Cook, V. Goh; London/UK
*(carolyn.horst@gmail.com)*

**Purpose:** Published data suggest that radiomic signatures in operable NSCLC alone may be associated with outcome, but value-added against clinical parameters is debated. The study aimed to develop a model of overall survival (OS) in NSCLC using clinical variables and to evaluate prognostic quantitative features extracted from preoperative CT.
**Methods or Backround:** Two publicly-available datasets were used for model development (n=598). REPLICA, a prospective, operable NSCLC cohort from 35 hospitals, was used for model validation (n=410). CT was acquired with varying protocols (90-120kVp, varying mA, 0.5-5mm slice thickness). Tumour segmentation was automated using a U-net neural network model trained on publicly available datasets. Cox regression models were fitted to preselected clinical predictors (age, sex, tumour and nodal stage) and radiomic features (tumour volume, energy and elongation). Prognostic significance was estimated by bootstrapping, and models' two-year OS discriminations were compared using the DeLong test. To assess clinical utility, high-sensitivity two-year mortality (≥0.90) classifiers were generated.
**Results or Findings:** REPLICA participants were most commonly male (54%, 223/410) with stage III adenocarcinoma (61%, 249/410). Both models demonstrated significant prognostic discrimination (clinical C-index: 0.60, P<.001; clinicoradiomic C-index: 0.63, P<.001). Clinicoradiomic two-year OS discrimination was superior to clinical (AUC: 0.66 vs 0.63, P<.03). High-sensitivity classifiers performed similarly using clinicoradiomic (sensitivity: 0.99, specificity: 0.11) and clinical model scores (sensitivity: 0.98, specificity: 0.07).
**Conclusion:** This study confirmed the prognostic value of age, sex and pathological stage in NSCLC. Tumour volume, energy and elongation offered a modest performance increase in a heterogeneous multicentre validation dataset reflective of the population, but this was small at a clinically relevant sensitivity threshold.
**Limitations:** 43 participants were excluded from the REPLICA cohort (43/453, 9%) due to inaccessible imaging.
**Ethics committee approval:** The research ethics committee approval as part of the Guy's Cancer Cohort.
**Funding for this study:** The study was funded by the UKRI London Medical Imaging and AI Centre. Other funding is disclosed in the disclosure of the authors.
**Author Disclosures:**
Eleni Karapanagiotou: Nothing to disclose
Andrea Bille: Nothing to disclose
Gary Cook: Nothing to disclose
Thubeena Manickavasagar: Research/Grant Support: Research/Grant Support: National Institute for Health Research Biomedical Research Centre at Guy's & St Thomas' Hospitals and King's College London, Research/Grant Support: Cancer Research UK National Cancer Imaging Translational Accelerator, Research/Grant Support: UK Research & Innovation London Medical Imaging and Artificial Intelligence Centre, Research/Grant Support: Wellcome/Engineering and Physical Sciences Research Council Centre for Medical Engineering at King's College London
Vicky Goh: Research/Grant Support: Research/Grant Support: National Institute for Health Research Biomedical Research Centre at Guy's & St Thomas' Hospitals and King's College London, Research/Grant Support: Cancer Research UK National Cancer Imaging Translational Accelerator, Research/Grant Support: UK Research & Innovation London Medical Imaging and Artificial Intelligence Centre, Research/Grant Support: Wellcome/Engineering and Physical Sciences Research Council Centre for Medical Engineering at King's College London
Carolyn Horst: Research/Grant Support: NIHR Academic Clinical Fellowship
Robert O'Shea: Research/Grant Support: National Institute for Health Research Biomedical Research Centre at Guy's & St Thomas' Hospitals and King's College London Research/Grant Support: Cancer Research UK National Cancer Imaging Translational Accelerator Research/Grant Support: UK Research & Innovation London Medical Imaging and Artificial Intelligence Centre Research/Grant Support: Wellcome/Engineering and Physical Sciences Research Council Centre for Medical Engineering at King's College London
Daniel Johnathan Hughes: Nothing to disclose

### RPS 1416-6

**CT radiomic predictors of PD-L1 status in non-small cell lung cancer (NSCLC): external validation in a prospective multicentre cohort**

R. O'Shea, *T. Manickavasagar*, C. Horst, D. J. Hughes, D. Nonaka, A. Bille, E. Karapanagiotou, G. Cook, V. Goh; London/UK

**Purpose:** A previous study has suggested that CT radiomics may identify PD-L1 status in operable NSCLC. As PD-L1 is a target for immunotherapy treatment, CT radiomics may assist in tailored and effective onward treatment. This study evaluated previously proposed predictive features for PD-L1 status in a geographically distinct multicentre cohort.
**Methods or Backround:** Imaging and histopathology were from the REPLICA cohort, a prospective cohort of operable NSCLC from 35 institutions (n=408). PD-L1 was measured from tumour biopsies, and positivity defined through thresholds of 1% and 50% expression, respectively. Radiomic features were extracted from preoperative CT. Following recent recommendations, analysis was limited to four previously identified radiomic predictors of PD-L1 expression (kurtosis, cluster tendency, size-zone matrix non-uniformity, and normalised run-length matrix nonuniformity). Univariate associations were measured using area-under-the-receiver-operator-characteristic (AUC) and confidence intervals estimated by bootstrapping.
**Results or Findings:** Participants were predominantly male (221/408, 54%) with stage III lung cancer (247/408, 61%) and adenocarcinoma subtype (267/409, 65%). 127 participants had PD-L1 expression between 1-50% (127/408, 31%); 42 had >50% expression (42/408, 10%). No examined radiomic feature discriminated PD-L1 expression at any threshold. The greatest discrimination was demonstrated by kurtosis at the 50% threshold but remained poor (AUC: 0.55, 95% CI: [0.45-0.65], P>.05).
**Conclusion:** Although previously identified radiomic features discriminated PD-L1 expression in a single-institution cohort of advanced disease, they were not generalisable to our multicentre cohort of operable disease. These findings may be explained by either 1) limited radiomic sensitivity to PD-L1 expression in operable disease or 2) low generalisability of the evaluated model to a heterogeneous multicentre cohort.
**Limitations:** Two participants were excluded due to unavailable histology, and 43 participants were excluded due to inaccessible imaging.
**Ethics committee approval:** The Research Ethics Committee approval as part of Guy's Cancer Cohort.
**Funding for this study:** The study was funded by the UKRI London Medical Imaging and AI Centre.
**Author Disclosures:**
Eleni Karapanagiotou: Nothing to disclose
Andrea Bille: Nothing to disclose
Gary Cook: Nothing to disclose
Thubeena Manickavasagar: Research/Grant Support: UK Research & Innovation London Medical Imaging and Artificial Intelligence Centre; Wellcome/Engineering and Physical Sciences Research Council Centre for Medical Engineering at King's College London [WT 203148/Z/16/Z]; National Institute for Health Research Biomedical Research Centre at Guy's & St Thomas' Hospitals and King's College London; Cancer Research UK National Cancer Imaging Translational Accelerator [C1519/A28682].
Vicky Goh: Research/Grant Support: UK Research & Innovation London Medical Imaging and Artificial Intelligence Centre; Wellcome/Engineering and Physical Sciences Research Council Centre for Medical Engineering at King's College London [WT 203148/Z/16/Z]; National Institute for Health Research Biomedical Research Centre at Guy's & St Thomas' Hospitals and King's College London; Cancer Research UK National Cancer Imaging Translational Accelerator [C1519/A28682].
Carolyn Horst: Research/Grant Support: NIHR Academic Clinical Fellow
Robert O'Shea: Research/Grant Support: UK Research & Innovation London Medical Imaging and Artificial Intelligence Centre; Wellcome/Engineering and Physical Sciences Research Council Centre for Medical Engineering at King's College London [WT 203148/Z/16/Z]; National Institute for Health Research Biomedical Research Centre at Guy's & St Thomas' Hospitals and King's College London; Cancer Research UK National Cancer Imaging Translational Accelerator.
Daniel Johnathan Hughes: Nothing to disclose
Daisuke Nonaka: Nothing to disclose

### RPS 1416-7

**CT-based radiomics of colorectal liver metastases after chemotherapy: does it predict the tumour regression grade?**

*A. Ammirabile*[1], M. Savino[2], F. Fiz[3], S. Langella[4], T. M. M. Gallo[4], G. Torzilli[1], L. S. Politi[1], F. Leva[2], L. Viganò[1]; [1]Pieve Emanuele, Milan/IT, [2]Milan/IT, [3]Genoa/IT, [4]Turin/IT
*(angela.ammirabile@humanitas.it)*

**Purpose:** The standard colorectal liver metastases (CRLM) treatment is surgery with perioperative chemotherapy. Response to preoperative chemotherapy is the strongest predictor of survival, but there is a major discordance between its radiologic (RECIST criteria) and pathologic evaluation (tumour regression grade, TRG). This multi-centric retrospective study investigates whether the radiomic features of CRLM from preoperative computed tomography (CT) provide a reliable prediction of TRG.
**Methods or Backround:** All consecutive patients undergoing liver resection for CRLM after chemotherapy at the Humanitas IRCCS, Rozzano (2017-2022) and the Mauriziano Hospital, Turin (2018-2020) were considered. In patients with multiple CRLM, the largest one was analysed. Two VOIs were drawn on the portal phase of the preoperative post-chemotherapy CT: the tumour (tumour-VOI) and a 5mm rim of peritumoural tissue (margin-VOI). Radiomic features were extracted using the LifeX software. Multivariate analysis was

Friday performed to identify predictors of response (TRG1-3). Three models were compared: clinical, clinical/tumour-VOI radiomics, and clinical/tumour-Margin-VOI radiomics.
**Results or Findings:** 169 patients were included (median age 61 years). All TRG1-3 patients (31%) displayed a response at imaging, but 65% of patients displaying a radiological response did not have a pathologic one (TRG4-5). At cross-validation, the clinical model for prediction of TRG1-3 showed an AUC=0.656, the clinical/tumour-VOI radiomics model an AUC=0.738, and the clinical/tumour-margin-VOI radiomics model an AUC=0.759. The final model retained six clinical variables, three tumour-VOI features (CRLM volume, GLRLM_SRLGE, GLRLM_GLNU) and three margin-VOI features (sphericity, GLZLM_SZE, GLZLM_GLNU).
**Conclusion:** CT-based textural features of CRLM guarantee an adequate non-invasive assessment of TRG. Radiomic indexes from both tumour and peritumoural tissue predicted pathologic response, while RECIST criteria had limited value. Radiomics integrated and did not replace the standard clinical predictors of TRG.
**Limitations:** The study is limited by the following factors: a retrospective study, a small number of patients, and lack of external validation.
**Ethics committee approval:** The study was approved by the local IRB under protocol number 83/20.
**Funding for this study:** This study is funded by the AIRC grant that can be found under the number 2019–23822.
**Author Disclosures:**
Teresa Maria Maria Gallo: Nothing to disclose
Matteo Savino: Nothing to disclose
Francesca Leva: Nothing to disclose
Angela Ammirabile: Nothing to disclose
Letterio Salvatore Politi: Nothing to disclose
Serena Langella: Nothing to disclose
Guido Torzilli: Nothing to disclose
Francesco Fiz: Nothing to disclose
Luca Viganò: Grant Recipient: Principal Investigator

**12:30-13:30 Research Stage 4**

### Research Presentation Session: Physics in Medical Imaging

### RPS 1413

### New approaches in patient dose assessment, optimisation and quality assurance

**Moderator**
J. S. Andersson; Umeå/SE
*(jonas.s.andersson@regionvasterbotten.se)*

### RPS 1413-2

**Automatic image quality parameter determination for pulmonary embolism CT scans**
L. v. Haren[1], C. v. Pul[2], E. J. I. Hoeijmakers[1], E. Laupman[2], M. van der Vlies[2], B. Martens[1], B. M. F. Hendriks[1], *C. R. Jeukens*[1]; [1]Maastricht/NL, [2]Veldhoven/NL
*(cecile.jeukens@mumc.nl)*

**Purpose:** To develop an algorithm that automatically determines noise and contrast-related image quality (IQ) metrics for quality control purposes in pulmonary embolism (PE) CT scans, and to assess agreement between objective IQ metrics and subjective IQ scoring by radiologists.
**Methods or Backround:** In two hospitals, two datasets of 50 consecutive clinical PE CT scans were retrospectively collected, including repeated scans, having a lower IQ. We developed an algorithm based on lungs and pulmonary vessels segmentation using a U-net and K-means clustering. In these 3D segmentations, five IQ metrics were calculated: noise and signal-to-noise ratio in lungs, mean signal in vessels, and contrast and the contrast-to-noise ratio between lungs and vessels. Additionally, five noise IQ metrics used in the literature were calculated. In each hospital, two radiologists scored noise, contrast attenuation of the pulmonary arteries and diagnostic confidence separately using a 5-point Likert scale (1=poor to 5=excellent). Additionally, the presence of lung pathology (PE, lesions, emphysema, effusion) was recorded, as this may influence the automatic segmentation and calculated IQ scores. Regression analysis was performed to assess the correlation between IQ metrics and Likert scores, reporting adjusted R2 and significance.
**Results or Findings:** The algorithm was able to automatically calculate all IQ metrics for all scans. Multiple significant correlations were found between IQ metrics and all three different Likert scores. For diagnostic confidence strongest correlations were observed for contrast (R2= 0.45/0.21) and vessel signal metrics (R2= 0.32/0.22). Results were consistent between both hospitals: overall best and least-performing IQ metrics were similar. Lung pathology did not significantly influence IQ scores.
**Conclusion:** It is feasible to determine objective IQ metrics that correlate with subjective IQ based on an automatic algorithm in PE CT scans. This opens possibilities for continuous image quality monitoring in clinical practice.
**Limitations:** Not applicable.
**Ethics committee approval:** No information provided by the submitter.
**Funding for this study:** No funding was received for this study.
**Author Disclosures:**
Eva Janna Ina Hoeijmakers: Nothing to disclose
Lisan van Haren: Nothing to disclose
Mark van der Vlies: Nothing to disclose
Bibi Martens: Nothing to disclose
Emmeline Laupman: Nothing to disclose
Cécile RLPN Jeukens: Nothing to disclose
Carola van Pul: Nothing to disclose
Babs M F Hendriks: Nothing to disclose

### RPS 1413-3

**Utilisation of x-ray CT dataset to detect, characterise, and quantify the local motion**
*J. Hsieh*; Brookfield, WI/US

**Purpose:** Automatic detection and characterisation of motion are key to ensure good image quality. In the past, the topic has been extensively investigated for x-ray computed tomography. Methods to combat patient motion include the use of gating devices, reducing the patient scan time, and the development of advanced algorithms. Because physiologically-induced motion is complex and often varies spatially and temporally, existing approaches often rely on CT operators to detect local motion artefacts and initiate appropriate corrective actions.
**Methods or Backround:** We propose a data consistency-metric (DCM) for motion detection. The DCM is based on the observation that the size and shape of small rigid objects, such as blood vessels or lung nodules, do not change significantly over the data acquisition period. Based on the characteristics of the tomographic reconstruction, it can be shown that the magnitude of the background circular integrals (BCI) surrounding these objects is small. Therefore, the differential signal for a stationary object between the back-projected intensity and its BCI exhibits linear relationship with respect to the projection view. The “goodness of fit” of the differential signal, therefore, represents the level of motion present at each local region, and thus provides a good 3D motion map for the reconstructed volume.
**Results or Findings:** A mathematical model is first presented in the paper to establish a theoretical foundation. Extensive computer simulations are then utilised to confirm the validity of the theory. The consistency-metric was tested using low-dose clinical datasets to demonstrate its efficacy and robustness.
**Conclusion:** The efficacy of the proposed approach is clearly demonstrated, and future expansion to the algorithm is discussed. This study clearly demonstrates a methodology to automatically characterise patient motion using CT data.
**Limitations:** Further extensive evaluation with clinical dataset is needed.
**Ethics committee approval:** -
**Funding for this study:** No funding was received for this study.
**Author Disclosures:**
Jiang Hsieh: Consultant: Arineta, Ltd. Consultant: GE Healthcare

### RPS 1413-4

**Theory, application, and tools for quality management in medical physics**
*H. Sundström*; Östersund/SE
*(henriksun@gmail.com)*

**Purpose:** Insight into the theory and application of quality management (QM), quality assurance (QA), and quality control (QC) in medical physics and how digital tools combined with novel ways of working with manufacturer's QC programs is reshaping the future possibilities of quality and safety in radiology.
**Methods or Backround:** In this work, we have performed systematic audits of the radiology services in Region Jämtland Härjedalen (Sweden) using a dose monitoring system (REMbox, Dicom Port AB) and a novel IT-solution for QA of CT scanners from GE Healthcare (autoQA®, Dicom Port AB).
**Results or Findings:** Dose audit through use of the REMbox software has enabled early discovery of malfunctioning equipment, of need for optimization, and easy comparison of dose levels for all modalities. For example, a spike in radiation dose was observed from a remote x-ray site (Ysio Max, Siemens Healthineers) followed by examinations with zero registered dose. Registration and analysis of these events helped to quickly restore a broken KAP-meter as well as provide a basis for targeted education for the on-site staff. The use of AutoQA has enabled real-time QA of computed tomography calibrations, audit and history of patient protocols and remote access to error logs. Using autoQA during the Covid-19 pandemic, we have performed systematic review of daily calibrations and protocol changes through remote access. This novel way of

Friday working has enabled quick feedback to the department with the purpose of upholding basic radiation safety in cases such as failed calibrations and mistakes in revision of examination protocols.
**Conclusion:** Systematic work on the QM, QA, and QC level through digital tools offers new possibilities in medical physics to improve results, efficacy, and basic radiation safety.
**Limitations:** A single manufacturer's software was used for evaluation.
**Ethics committee approval:** -
**Funding for this study:** No funding was received for this study.
**Author Disclosures:**
Henrik Sundström: Nothing to disclose

### RPS 1413-5

**Remote and automated quality control using advanced image quality metrics in radiography and mammography: pilot results of an IAEA research project**

*V. Tsapaki*[1], O. Ciraj-Bjelac[1], R. Ruggeri[2], P. R. Costa[3], I. Fitton[4], C-I. Tsougos[5], C. Pesznyak[6], C. Kleefeld[7], J. Hsiu Ding Wong[8], J. Patricio Castillo Lopez[9], I. A. Tsalafoutas[10], U. Zdesar[11], A. Y. A. Mohamed[12]; [1]Vienna/AT, [2]Rio Negro/AR, [3]São Paulo/BR, [4]Paris/FR, [5]Larissa/GR, [6]Budapest/HU, [7]Galway/IE, [8]Kuala Lumpur/MY, [9]Mexico City/MX, [10]Doha/QA, [11]Ljubljana/SI, [12]Gezira/EG
*(v.tsapaki@iaea.org)*

**Purpose:** The objective of this work is to contribute to objective assessment and evaluation of image quality in digital radiography and mammography in diverse clinical settings through a daily/weekly remote/automated quality control (QC) methodology recently published by the International Atomic Energy Agency (IAEA).
**Methods or Backround:** The methodology is being evaluated under an IAEA Coordinated Research Project, launched in November 2021. Eleven countries (Argentina, Brazil, France, Greece, Hungary, Ireland, Mexico, Malaysia, Qatar, Slovenia and Sudan) agreed on a common workplan: 1) phantom construction, 2) staff training to implement daily/weekly QC testing, 3) pilot study in various digital modalities for 6 months, including comparison with similar available solution.
**Results or Findings:** The participants have not depicted any major challenges either in purchasing the material or in construction. Construction costs ranged from 15 to 133 euros per phantom. Eighty-nine phantoms were constructed so far and weekly QC testing was performed in 35 radiography and 23 mammography units. To facilitate implementation of methodology in hospitals, training material and video tutorials were produced in English, French, Greek, Hungarian and Spanish. Pilot comparison to 3 commercial automated solutions in one x-ray radiography unit show that the commercial phantoms/software are not sensitive enough to detect subtle changes in image quality. On the contrary, using the IAEA phantom and methodology, the scores followed trends theoretically expected.
**Conclusion:** The IAEA methodology is straightforward. The phantoms are easily fabricated and consist of low-cost material, readily available. It is also sensitive enough to detect subtle changes in image quality and, as such, presents an important asset in QC in digital radiology.
**Limitations:** This is a pilot survey. More data are needed.
**Ethics committee approval:** -
**Funding for this study:** Funding was received from IAEA research project E24025, "Advanced Tools for Quality and Dosimetry of Digital Imaging in Radiology."
**Author Disclosures:**
Ioannis A Tsalafoutas: Nothing to disclose
Paulo Roberto Costa: Nothing to disclose
Ricardo Ruggeri: Nothing to disclose
Christoph Kleefeld: Nothing to disclose
Virginia Tsapaki: Nothing to disclose
Isabelle Fitton: Nothing to disclose
Ahmed Yassen Ali Mohamed: Nothing to disclose
Jorge Patricio Castillo Lopez: Nothing to disclose
Olivera Ciraj-Bjelac: Nothing to disclose
Urban Zdesar: Nothing to disclose
Jeannie Hsiu Ding Wong: Nothing to disclose
Csilla Pesznyak: Nothing to disclose
Chrysovalantis-Ioannis Tsougos: Nothing to disclose

### RPS 1413-6

**CT tube current and tube voltage modulation to minimise the patient's radiation risk**

*E. Baader*, L. Klein, S. Sawall, J. Maier, M. Kachelrieß; Heidelberg/DE
*(edith.baader@dkfz.de)*

**Purpose:** To develop risk TCTVM, a combined tube current and tube voltage modulation technique which minimises the patient radiation risk while keeping image quality constant.
**Methods or Backround:** In diagnostic CT, tube current modulation (TCM) aims at reducing patient dose. Actual implementations, however, minimise the mAs-product of the scan. This mAsTCM typically yields a patient dose reduction but never a patient dose or risk minimisation. Recently, we had proposed riskTCM. It minimises the patient risk by means of minimising the effective dose, that is calculated in a patient-specific way [Moed. Phys. 49(7):4391-4403]. Compared to mAsTCM, riskTCM reduces the effective dose on average between 10% and 30%, depending on the body region. Here, we propose riskTCTVM, an extension of riskTCM that performs a tube voltage modulation (TVM) in addition to a TCM. Both methods were evaluated in a simulation study based on clinical CT data. Noisy polychromatic forward projections were calculated to simulate a scanner with TCM and TVM. The effective dose required to obtain a specified image quality in terms of image noise was determined for each patient. In riskTCTVM, the tube voltage was restricted to modulate between 70 and 150 kV.
**Results or Findings:** Compared to riskTCM, our proposed modulation technique shows significant reductions of the effective dose.
**Conclusion:** Modulating the tube voltage additionally to the tube current offers potential further reduction of the radiation risk.
**Limitations:** The proposed modulation technique was tested in a simulation study only.
**Ethics committee approval:** No information provided by the submitter.
**Funding for this study:** This work was supported by the Helmholtz International Graduate School for Cancer Research, Heidelberg, Germany.
**Author Disclosures:**
Laura Klein: Nothing to disclose
Edith Baader: Nothing to disclose
Marc Kachelrieß: Nothing to disclose
Stefan Sawall: Nothing to disclose
Joscha Maier: Nothing to disclose

### RPS 1413-7

**Validation of a Monte Carlo (MC) simulated cone-beam CT (CBCT) radiation transport model encompassing automatic exposure control (AEC): towards patient-specific dosimetry**

*N. McWilliams*, S. Cournane, J. McCavana, L. Leon-Vintro; Dublin/IE
*(nina.mcwilliams@ucdconnect.ie)*

**Purpose:** A number of recent developments in the area of CBCT have led to an increased use of CBCT in clinical practice. However, approaches to establishing patient dosimetry in CBCT are still under development. Replication of system-specific complexities such as automatic exposure control (AEC), rotational geometries, and scatter contributions make simulations of CBCT radiation doses challenging. The aim of this study was to model the radiation transport of a Siemens Artis Q CBCT C-arm interventional radiology (IR) system using Monte Carlo (MC) simulations, taking into account the system's AEC modulation.
**Methods or Backround:** CBCT acquisitions employ AEC modulation over the 200 degree acquisition, controlling the kVp and mA, to maintain a constant detector dose for each projection. TOPAS, a MC GEANT4 simulation toolkit, was used to simulate the most clinically used abdominal CBCT protocol. MC simulations were performed using the beam's energy spectrum, generated using SpekPy. The system's geometry and filtration were taken from manufacturer specifications. Cross-sectional dose distributions and percentage depth distributions (PDDs) were obtained from simulations and experimentally measured for a range of uniform and non-uniform phantoms.
**Results or Findings:** The Siemens Artis Q system and CBCT acquisitions were successfully modelled incorporating the AEC functionality. The number of photons simulated, per projection, was 10^8 histories which corresponded to a statistical uncertainty estimate of < 4%. A comparison of simulated doses with measured doses will be presented for all phantoms and all investigated conditions.
**Conclusion:** This study presents a TOPAS MC model of a Siemens Artis Q C-arm CBCT system for establishing dose distributions in phantoms, taking into account AEC functionality. Future directions of this study will focus on the development of patient-specific CBCT dosimetry simulations.
**Limitations:** This was a single-centre study.
**Ethics committee approval:** Full ethical approval for this study has been granted by St Vincent's University Hospital and University College Dublin.
**Funding for this study:** Funding was received from the Irish Research Council's Employment-Based Postgraduate Scholarship.
**Author Disclosures:**
Seán Cournane: Nothing to disclose
Nina McWilliams: Nothing to disclose
Jackie McCavana: Nothing to disclose
Luis Leon-Vintro: Nothing to disclose

### RPS 1413-8

**Cone-beam CT with collimated beam and exotic trajectory for quantitative imaging**

*A. Sarno*[1], G. Mettivier[1], P. Cardarelli[2], S. Pardi[1], M. C. MOLLO[1], R. de Asmundis[1], I. Vanore[1], P. Mauriello[1], P. Russo[1]; [1]Naples/IT, [2]Ferrara/IT
*(sarno@na.infn.it)*

**Purpose:** To investigate an innovative cone beam CT (CBCT) solution with exotic scanning geometry (ExoCT) for the reduction of scatter and cone artifacts toward its use in quantitative imaging evaluations.
**Methods or Backround:** The proposed geometry relies on a beam collimation in axial direction for scatter suppression and on a source oscillating geometry to compensate for the narrower field of view (FOV) and to reduce the cone artifacts due to the constraint of circular scanning in conventional CBCT. The ExoCT innovative geometry was investigated via Monte Carlo simulations and confirmed by preliminary measurements on a prototype scanner. Investigations focused on the reduction of the scatter-to-primary ratio (SPR) on projections and enhancement of image quality in phantom studies.
**Results or Findings:** SPR in ExoCT was 3 times lower than conventional CBCT, on average, for a fixed FOV (160-mm diameter cylindrical phantom, W/Al spectrum 80 kV). This resulted in a conspicuous reduction of cupping artifacts in the reconstructed 3D images as well as in an increase of 2.3 times the contrast-to-noise ratio of a simulated soft lesion. Conspicuity of the air gaps in a Defrise phantom was reduced for remote portion of the FOV in CBCT and resulted almost unchanged for the ExoCT geometry.
**Conclusion:** Comparison between image quality of ExoCT and CBCT showed that the first permits to increase the CNR and to reduce the cupping artifacts in phantom images, also producing a conspicuous reduction of cone-angle related artifacts, which affects image quality for remote portions of the FOV.
**Limitations:** Analyses were limited to in-silico studies and measurements on prototype scanner.
**Ethics committee approval:** -
**Funding for this study:** No funding was received for this study
**Author Disclosures:**
Riccardo de Asmundis: Nothing to disclose
Maximilian Carlos MOLLO: Nothing to disclose
Silvio Pardi: Nothing to disclose
Immacolata Vanore: Nothing to disclose
Paolo Mauriello: Nothing to disclose
Giovanni Mettivier: Nothing to disclose
Paolo Russo: Nothing to disclose
Antonio Sarno: Nothing to disclose
Paolo Cardarelli: Nothing to disclose

**14:00-15:30 Research Stage 1**

### Research Presentation Session: Chest

### RPS 1504

### Imaging of the airways and ILDs

**Moderator**
C. de Margerie-Mellon; Paris/FR
*(constance.de-margerie@aphp.fr)*
**Author Disclosures:**
Constance De Margerie-Mellon: Consultant: Boerhinger Ingelheim, Pfizer, Gilead Science, Bracco

### RPS 1504-2

**Evaluation of quantitative airway-artery dimensions and ratios of normal CT scans: a study from birth to adulthood**

Q. Lv, *Y. Chen*, E-R. Andrinopoulou, J-P. Charbonnier, P. Ciet, H. A. W. M. Tiddens; Rotterdam/NL

**Purpose:** To investigate age-related changes of quantitative airway-artery (AA) parameters derived from chest computed tomography (CT).
**Methods or Backround:** 1160 normal chest CT scans of participants aged 0-24 years were collected by the Normal Chest CT Study Group. Airway-artery (AA) analysis of suitable CTs was executed fully automatically (LungQ, version 2.1.0.1, Thirona, Nijmegen, Netherlands). Requirements for LungQ are volumetric acquisition and slice thickness ≤1.5mm. LungQ segments the bronchial tree, identifies generation starting from the segmental bronchi (G0) and higher generations (G1-G10), and measures for each AA-pair: diameters of airway outer (Aout), airway lumen (Alumen), and adjacent artery (A), and airway wall thickness (Awt). Next, AA-ratios (Aout/A, Alumen/A, and Awt/A) and number of AA-pairs are computed. Mixed-effects models were used to investigate the effect of age, gender, total lung volume, and iodine contrast on AA-outcomes. A p-value less than 0.05 was considered significant.
**Results or Findings:** 390 inspiratory CT scans met LungQ requirements (161 females, 229 males). Mean (SD) age was 12.3 (5.3) years. In total, 45208 AA-pairs were measured. The mixed-effects models showed that both Aout and Alumen increased 0.02mm (all $p<0.02$) per year, but Awt, Aout/A, Alumen/A, and Awt/A did not change with age. Aout was on average 0.09mm higher in males than females ($p<0.03$). No significant differences in other AA-parameters were observed between genders. The use of contrast led to a greater Awt ($p<0.001$), lower Alumen, Aout/A, Alumen/A, and Awt/A (all $p<0.001$), and did not influence Aout.
**Conclusion:** On normal chest CTs, AA-ratios do not change significantly from childhood into young adulthood. These results show that the AA-ratios as measured by the automatic AA-analysis can be used to diagnose and monitor airway dilatation and airway wall thickness.
**Limitations:** Mixed case.
**Ethics committee approval:** No information provided by the submitter.
**Funding for this study:** Funding was received from a PPP grant.
**Author Disclosures:**
Jean-Paul Charbonnier: Shareholder: Thirona Employee: Thirona
Eleni-Rosalina Andrinopoulou: Nothing to disclose
Pierluigi Ciet: Consultant: Vertex and Chiesi Pharmaceuticals
Qianting Lv: Nothing to disclose
Harm A W M Tiddens: CEO: Chief medical officer of Thirona
Yuxin Chen: Nothing to disclose

### RPS 1504-3

**Small airways disease and emphysema in pre-COPD: a cross-sectional study**

*S. Verleden*, J. Hendriks, C. Mai, A. Snoeckx, S. Koljenovic, P. Van Schil, V. Verplancke, J. Kwakkel-van-Erp, T. Lapperre; Antwerp/BE
*(stijn.verleden@uza.be)*

**Purpose:** Small airway disease is an important pathophysiological feature of COPD. Recently, pre-COPD has been proposed as a potential precursor stage of COPD. Pre-COPD is defined by abnormal pulmonary function or emphysema on CT in the absence of airflow obstruction. In this study, we investigate small airway involvement in pre-COPD using CT and microCT.
**Methods or Backround:** We collected surgical excision specimens (lobes from lobectomy or lungs from transplantation) from patients with pre-COPD (n=9), COPD GOLD I (n=5), GOLD II (n=6), GOLD III/IV (n=7), and controls (n=7, ever and never smokers). Lung specimens were cannulated, air-inflated, fixed in liquid nitrogen fumes and analysed using both CT (visual scoring of emphysema) and microCT (number and morphology of the small airways).
**Results or Findings:** The degree of emphysema was significantly higher in the pre-COPD group compared to controls, while there was no difference between pre-COPD and COPD. The number of terminal bronchioles (TB)/mL was decreased in pre-COPD ($p=0.001$), GOLDI ($p=0.0023$), GOLDII ($p<0.0001$), and GOLDIII/IV ($p<0.0001$) compared to controls. In addition, the number of alveolar attachments was lower in pre-COPD and COPD groups compared to control. We did not find any differences in the small airway number and morphology between the pre-COPD and GOLD I group. The % of emphysema on CT showed a strong correlation with the number of TB/mL.
**Conclusion:** In patients with pre-COPD, the number of small airways has diminished by 35% compared to controls, and associated with the degree of emphysema. Lungs of patients with pre-COPD therefore already show significant remodeling and small airway loss in the absence of physiologic airway obstruction. The exact aetiology of pre-COPD needs to be investigated.
**Limitations:** Single-centre study. Limited number of patients. No information about disease evolution.
**Ethics committee approval:** This study was approved by EDGE 001649.
**Funding for this study:** Funding was received from BOF DOC-Pro and the Funding University Hospital Antwerp.
**Author Disclosures:**
Paul Van Schil: Nothing to disclose
Senada Koljenovic: Nothing to disclose
Annemiek Snoeckx: Nothing to disclose
Stijn Verleden: Consultant: Sanofi
Cindy Mai: Nothing to disclose
Veronique Verplancke: Nothing to disclose
Johanna Kwakkel-van-Erp: Nothing to disclose
Jeroen Hendriks: Nothing to disclose
Therese Lapperre: Nothing to disclose

### RPS 1504-4

**Interstitial lung abnormalities in low-dose CT screening population: risk factors for the development of clinically significant interstitial lung disease**

*W. Jeong*, B-D. Nam, J. H. Hwang; Seoul/KR

**Purpose:** To investigate the natural course of interstitial lung abnormality (ILA) and determine the risk factors for the development of clinically significant interstitial lung disease (ILD).

**Methods or Backround:** We retrieved 36,891 low-dose chest CT records from January 2003 to May 2021 using the terms representing ILA. After the review of the presence of ILA, 102 patients (0.3%) were included. Their clinical findings, results of pulmonary function test (PFT), and initial and follow-up CT findings were analysed including visual analysis and AI-based quantitative analysis. We compared findings between the patients who were clinically diagnosed with ILD and those who were not.
**Results or Findings:** Among 102 patients, 23 patients (22%) were diagnosed with clinically significant ILD and 79 patients (78%) were not. On CT analysis, subpleural fibrotic ILA was more frequently identified (43% vs. 19%, p=0.004) in patients with clinically diagnosed ILD. Progression on follow-up CT was also more frequently seen in patients with clinically diagnosed ILD (77% vs. 30%, p<0.001). On logistic regression analysis, respiratory event (OR 5.56, 95% CI, p=0.0223) and progression on follow-up CT (OR 4.07, 95%, p=0.0498) were significant parameters for the clinical diagnosis of ILD.
**Conclusion:** ILA was identified in the chest low-dose CT screening population with a frequency of 0.3%, approximately one-fifth of which eventually developed clinically significant ILD. In patients with ILA, respiratory events and progression on follow-up CT were revealed as significant risk factors for the development of clinically significant ILD.
**Limitations:** This study is retrospective design and the number of patients was small. Not all patients performed follow-up PFTs and CTs. Data may be missing because the cohort was recruited using the search terms representing ILA.
**Ethics committee approval:** No information provided by the submitter.
**Funding for this study:** No funding was received for this study.
**Author Disclosures:**
Jung Hwa Hwang: Author: lead author
Wonjong Jeong: Author: first author
Bo-Da Nam: Author: corresponding author

### RPS 1504-5

**General population bronchial parameters: results from the ImaLife study**

*I. Dudurych*[1], M. de Bruijne[2], R. Vliegenthart[1]; [1]Groningen/NL, [2]Rotterdam/NL

**Purpose:** Evaluation of reference computed tomography (CT)-derived bronchial parameters for men and women in a cohort of the general population in the North of Netherlands.
**Methods or Backround:** Underlying physiological differences between men and women may contribute to differences in bronchial parameters (BP) and their changes with time. Establishing reference CT-derived BP in the general population could be useful for detection and monitoring of disease. We collected low-dose thoracic CT scans of participants in a large-scale, single-centre study in the North of Netherlands (Imaging in Lifelines). Automated software calculated BP for a subset of participants. We investigated the following BP: luminal area (LA), wall area percent (WA%), wall thickness (WT), total airway count (TAC), total airway volume (TAV), and square root of the wall thickness of a hypothetical airway with internal perimeter of 10mm (Pi10). Student's t-test was used to evaluate BP differences between male and female participants, one-way ANOVA was used for differences between ages binned into 10-year groups. Using multivariate linear regression, we assessed the influence of gender, age, weight, and total lung volume (TLV) on BP.
**Results or Findings:** 282 (53.3% male) participants were included. All bronchial parameters apart from WA% were higher in men compared to women (p>0.05). Significant differences of BPs between age groups were measured, with higher BPs in older groups. Multivariate regression analysis showed a significant influence of gender on Pi10, TAC, and WT after correction for age, weight, and TLV.
**Conclusion:** CT-derived bronchial parameters correlate with gender and age. Correction for these factors would be important in the use of bronchial parameters in the detection and monitoring of pulmonary disease.
**Limitations:** Currently, smoking and disease history was unavailable for analysis, and will be included in the future.
**Ethics committee approval:** This study was approved by the Institutional Review Board. The ImaLife study was registered with the Dutch Central Committee on Research Involving Human Subjects (https://www.toetsingonline.nl, Identifier:NL58592.042.16).
**Funding for this study:** Funding was received from IMDI/ZonMW.
**Author Disclosures:**
Marleen de Bruijne: Nothing to disclose
Ivan Dudurych: Nothing to disclose
Rozemarijn Vliegenthart: Research/Grant Support: Institutional research grant from Siemens Healthineers

### RPS 1504-6

**Additional value of lung ultra-high-resolution photon-counting CT imaging for multidisciplinary classification of idiopathic pulmonary fibrosis: first-in-human results**

*S. A. Si-Mohamed*, S. Boccalini, M. Nasser, D. Gamondes, R. Diesler, H. Lacombe, L. Boussel, V. Cottin, P. Douek; Lyon/FR

**Purpose:** The study aimed to compare the image quality between lung ultra-high-resolution (UHR) spectral photon-counting CT (SPCCT) and an energy-integrating detector CT (EID-CT) for usual interstitial pneumonia (UIP) classification and diagnosis assessed by a multidisciplinary discussion (MDD).
**Methods or Backround:** In this prospective board-approved study with informed consent, participants with idiopathic interstitial pneumonia (IIP) underwent both non-contrast lung CT on an SPCCT and an EID-CT (Philips Healthcare). Three lung radiologists independently assessed the diagnostic quality of fibrotic features using a five-point score (1: insufficient, 5: excellent). They assessed the pattern into typical UIP, probable UIP, indeterminate for UIP and most consistent with non-idiopathic pulmonary fibrosis (IPF) and its confidence on a four-point score (1: none, i.e., <50%, 2: moderate, i.e., 50-70%, 3: probable, i.e., 70-90%, 4: certain, i.e., >90%). An MDD assessed a final clinical diagnostic according to a six-point confidence score (1: certain for unclassifiable, 2: high for unclassifiable, 3: low for unclassifiable, 4: low for IPF, 5: high for IPF, 6: certain for IPF).
**Results or Findings:** Twenty consecutive participants (13(65%) male, 70±11-years) were enrolled. Scores of quality for fibrotic features were greater with SPCCT images (P values<0.05). The proportion of improvement with SPCCT images for the pattern confidence was 80%, with a certain confidence for 55% of the cases, while it was achieved only for 10% of the cases with EID-DLCT images. The proportion of improvement with SPCCT images for the multidisciplinary diagnostic confidence was 45%, with a certain confidence for 75% of the cases while it was achieved only for 40% of cases with EID-DLCT images. Any case was rated low confidence, contrary to five cases with EID-DLCT images.
**Conclusion:** Lung UHR SPCCT imaging outperformed EID-CT imaging in classifying IIP after an MDD.
**Limitations:** The number of patients was limited.
**Ethics committee approval:** The ethics committee approval can be found under number 2019-A02945-52.
**Funding for this study:** The study is funded by the European Union H2020. The funding can be found under approval number 2019-A02945-52.
**Author Disclosures:**
Salim Aymeric Si-Mohamed: Nothing to disclose
Rémi Diesler: Nothing to disclose
Delphine Gamondes: Nothing to disclose
Loïc Boussel: Nothing to disclose
Philippe Douek: Nothing to disclose
Hugo Lacombe: Nothing to disclose
Sara Boccalini: Nothing to disclose
Vincent Cottin: Nothing to disclose
Mohammad Nasser: Nothing to disclose

### RPS 1504-7

**AI-based small pulmonary artery and vein quantification are associated with lung function impairment in Chronic Obstructive Pulmonary Disease**

*A. K. Kwee*[1], J-P. Charbonnier[2], L. Gallardo Estrella[2], F. A. A. Mohamed Hoesein[1], S. Humphries[3], D. Lynch[3], P. de Jong[1]; [1]Utrecht/NL, [2]Nijmegen/NL, [3]Denver, CO/US

**Purpose:** Innovative quantitative CT analysis enables assessment of small peripheral pulmonary vessels. This feasibility study aimed to investigate the association between quantitative pulmonary arterial and venous biomarkers, pulmonary function, and COPD severity.
**Methods or Backround:** The AI-based vascular quantification platform LungQ (Thirona, the Netherlands) was used to quantify small arteries and veins (diameter <1mm) in 261 inspiratory CT scans from the COPDGene study, including never-smokers with normal pulmonary function and smokers with COPD (GOLD 0-IV and PRISm). The quantitative biomarkers included small artery and vein volume and branch count, all normalised for body height. The relationship of the vascular biomarkers with pulmonary function (FEV1% predicted, GOLD stage) was assessed with and without correction for CT-quantified emphysema (15th percentile), airway wall thickness (Pi10), age, height, BMI, smoking status (current/former), and pack-years smoked by using linear regression.
**Results or Findings:** Subjects with COPD GOLD IV (n=37) compared to never smokers (n=37) had a higher vein count (4254.4±883 vs. 3375.9±459; p<.001) and a lower artery count (5578.5±1040 vs. 5655.3±994; p<.001). Multivariate linear regression analysis showed a positive association between FEV1%predicted and artery count (β=0.388; p<.001) and artery volume (β=0.330; p<.001). Negative associations were found between FEV1% predicted and vein count (β=-0.494; p<.001) and vein volume (β=-0.437; p<.001). For total vessel volume and branch count, these associations were weaker or not significant.

Friday

**Conclusion:** Increased pulmonary vein count and volume are associated with a lower lung function and more severe COPD, whereas increased artery count and volume are associated with a higher lung function. Arteries and veins biomarkers contributed independently to lung function, even after correcting for emphysema and Pi10. Analysis of total vasculature showed less associations, highlighting the importance of artery-vein separation to gain a deeper understanding of the pathophysiology.
**Limitations:** Not applicable.
**Ethics committee approval:** -
**Funding for this study:** Not applicable.
**Author Disclosures:**
Jean-Paul Charbonnier: Employee: Thirona
David Lynch: Nothing to disclose
Anastasia K.A.L. Kwee: Nothing to disclose
Leticia Gallardo Estrella: Employee: Thirona
Pim de Jong: Nothing to disclose
Firdaus A. A. Mohamed Hoesein: Nothing to disclose
Stephen Humphries: Nothing to disclose

### RPS 1504-8

**The effect of SilverBeam filter on cyst scores in patients with cystic lung diseases**

*C. Steveson*[1], J. Schuzer[2], S. Rollison[3], J. Moss[3], M. Y. Chen[3]; [1]Adelaide/AU, [2]Wheaton, IL/US, [3]Bethesda, MD/US

**Purpose:** The aim of this study is to investigate the effect of a new silver beam filter on quantitative cyst scores in chest CT scans.
**Methods or Backround:** With institutional ethics approval, 32 consecutive patients with lymphangioleiomyomatosis (LAM) underwent chest CT at low radiation dose with the standard filter on a 320 detector row CT scanner with the following scan parameters: Helical scan, 0.5mm x 80 detector rows, 120kV, 20mA, 0.5s rotation speed, and standard pitch. A low dose scan with SilverBeam filter was performed with the following same parameters: Helical scan, 0.5mm x 80 detector rows, 120kV, 80mA, 0.5s rotation speed, and standard pitch. Each scan was reconstructed using a soft tissue kernel with deep learning reconstruction (AiCE) techniques. Cystic lung disease can be quantified as a cyst score (% of lungs affected by cysts). Cyst scores were quantified by semi-automated software. Signal to noise ratio was calculated for each reconstruction. Data was analysed by linear correlation, paired t-test, and Bland-Altman.
**Results or Findings:** Patients averaged 48.9 years (range 23-68yrs), 100% were female with mean BMI 26.1 ±6.7 kg/m2. The mean radiation dose was 0.33v ±0.03mSv for the Standard scan vs 0.33 ±0.05mSv for the SilverBeam scan. The mean cyst score was 7.46±9.22 for standard scan and 7.25±9.14 for SilverBeam scan, representing a difference of 0.21. Linear correlation coefficient was excellent at 0.98 ($p<0.001$). Signal-to-noise for standard images was comparable to SilverBeam images (2.92vs. 3.07, respectively, $p=0.51$).
**Conclusion:** SilverBeam filter scans have an excellent correlation with standard filter scans for chest CT at low radiation doses.
**Limitations:** Single Center Study.
**Ethics committee approval:** No information provided by the submitter.
**Funding for this study:** No funding was received for this study.
**Author Disclosures:**
Marcus Y Chen: Nothing to disclose
Chloe Steveson: Employee: Canon Medical Systems Corporation
John Schuzer: Employee: Canon Medical Research Unit
Joel Moss: Nothing to disclose
Shirley Rollison: Nothing to disclose

### RPS 1504-9

**Quantitative computed tomography assessment of interstitial lung disease associated with connective tissue disease**

*H. Guo*, H. Zhang; Lanzhou/CN

**Purpose:** To determine whether Quantitative CT (QCT) assessment parameters can identify extensive or limited lung disease in patients with CTD-ILD.
**Methods or Backround:** This retrospective study enrolled 93 CTD-ILD patients with complete clinical data. A computerised integrated index (CII) was developed based on five densitometric measures: mean lung attenuation (MLA), skewness, kurtosis, lung volume, and SD, which were all QCT parameters. Semi-quantitative CT (SQCT) included Goh and other visual scores. The correlation among QCT, SQCT, and PFT were analysed, the ability of each parameter to identify the severity of CTD-ILD was evaluated.
**Results or Findings:** 93 patients were included in the study, including 20 males and 73 females, with an average age of (53.66±12.79) years. MLA and SD were positively correlated with SQCT and kurtosis and skewness and CII were negatively correlated with SQCT, while lung volume was not correlated with SQCT. CII, kurtosis, skewness, and lung volume were positively correlated with FVC%, TLC%, VC%, FEV1% and FEV1/FVC, while SD and MLA were negatively correlated with them. CII, kurtosis, and skewness were positively correlated with DLco%, SD was negatively correlated with DLco%, but lung volume and MLA were not correlated with DLco%. There were significant differences in QCT, SQCT, FVC%, TLC%, VC%, FEV1% and DLco% in limited/extensive lung disease, while there was no significant difference in FEV1/FVC. Among all parameters, kurtosis had the strongest diagnostic ability for limited/extensive lung disease; the optimal kurtosis value was 3.421 (AUC=0.882). The next one was skewness; the best skewness value was 1.714(AUC=0.864). CII ranked third with the best cut-off value of -0.064(AUC=0.838).
**Conclusion:** QCT of CTD-ILD patients had a good correlation with SQCT and PFT, and QCT had the ability to identify the limited/extensive lung disease of CTD-ILD patients.
**Limitations:** Not applicable.
**Ethics committee approval:** -
**Funding for this study:** Not applicable.
**Author Disclosures:**
Honghong Guo: Nothing to disclose
Hao Zhang: Nothing to disclose

### RPS 1504-10

**Prognostic implications of clinical imaging and blood biomarkers on progression in fibrotic interstitial lung diseases using quantitative CT analysis**

K. Akbari, *J. Wasmayr*, D. Lang, B. Lamprecht, F. Fellner; Linz/AT
*(johanneswasmayr@gmx.at)*

**Purpose:** We recently showed that visually and semi-quantitatively evaluated honeycombing and traction bronchiectasis extent in baseline HRCT, together with clinical features and peripheral blood monocyte count (MON), could prognosticate progression in fibrotic interstitial lung disease (ILD) patients. In this study, we aimed to compare these results using an artificial intelligence (AI)-based quantitative CT analysis approach.
**Methods or Backround:** HRCT scans of a total of 140 patients were evaluated for the extent of ILD-specific findings by the AI-based CT analysis tool (SEARCH Lung CT, contextflow) using a percentage of total lung volume (<1%-100%). Outcomes were compared to our previous scoring model of 0-6 lung fields using percentage accordance, Cohen's Kappa, and Spearman Rank correlation. Univariate and multivariate analyses were performed to establish the impact of baseline AI-CT-based biomarkers on one-year progression (≥10% loss in forced vital capacity, ≥15% loss in diffusion capacity for carbon monoxide, lung transplant or death).
**Results or Findings:** A high correlation in outcome congruence was found for ground glass opacities and emphysema. In univariate analysis, reticulation and honeycombing significantly correlated with progression. Multivariate analysis confirmed significant interaction between the extent of reticular abnormalities and progression. Univariate and multivariate analysis of MON showed a similar significant correlation with progression. However, the extent of traction bronchiectasis, identified as a major HRCT variable in the previous analysis, was not yet available for AI-based CT analysis.
**Conclusion:** Ground glass opacities and emphysema showed the most significant accordance when comparing the AI-based and visual HRCT scoring approaches. In addition, the extent of reticulation and honeycombing in the quantitative CT analysis, as well as elevated blood monocyte count, predicted disease progression within one year. Results concerning traction bronchiectasis extent are still to be evaluated.
**Limitations:** The study is done in a retrospective setting with a small sample size.
**Ethics committee approval:** The ethics committee approval was received by Johannes Kepler University, Linz, Austria.
**Funding for this study:** No funding was received for this study.
**Author Disclosures:**
Kaveh Akbari: Nothing to disclose
David Lang: Nothing to disclose
Johannes Wasmayr: Nothing to disclose
Franz Fellner: Nothing to disclose
Bernd Lamprecht: Nothing to disclose

### RPS 1504-11

**Minimal clinically important difference (MCID) for quantitative analysis of progressive pulmonary fibrosis on high-resolution computed tomography (HRCT)**

*L. Iaccarino*, G. Cicchetti, G. Sgalla, A. Del Ciello, L. Calandriello, A. Farchione, L. Richeldi, A. R. Larici; Rome/IT
*(ludovica.iaccarino@gmail.com)*

**Purpose:** The study aimed to validate the visual semi-quantitative scoring of pulmonary fibrosis on high-resolution computed tomography (HRCT) scans against clinical parameters of ILD progression to identify the minimal clinically significant difference (MCID) of fibrotic extent over 12 months.
**Methods or Backround:** Patients with an ILD diagnosis were prospectively enrolled. Patients underwent spirometry, carbon monoxide diffusion capacity (DLCO) tests, 6-minute walk tests (6MWTs), and HRCT scan at baseline and follow-up. A thoracic radiologist estimated the total extent of ILD changes, expressed as an overall percentage of the entire lung volume.
**Results or Findings:** 35 patients (median age 73 years) with an ILD diagnosis were included. 80% of patients had IPF, while the remaining ones (20%) had non-IPF ILDs. 12-months changes in ILD extent significantly correlates with FVC (r=-0.412; p=0.046) and Shortness of Breath (SOB) scores (r=0.504; p=0.012). Patients with progressive disease (n=21) had a mean ILD extent of 33.8%, as compared to 22.9% of non-progressive patients (n=14) (mean difference 10.9%; p=0.042). An MCID for a total ILD extent of 10% was estimated after approximating the nearest 5%, consistently with the visual scoring method. Time to meaningful disease progression was significantly lower in patients with more than a 10% increase in ILD extent at 12 months (log-rank p=0.007).
**Conclusion:** The visual evaluation of fibrotic extent on HRCT is a valid and responsive measure of disease progression in ILD patients. A 12-month increase of ILD extent greater than 10% on HRCT represents a clinically meaningful difference that could help in the discrimination of progressive fibrosing ILDs (PF-ILDs).
**Limitations:** The study is limited by the sample size, the large majority of IPF patients, and the required application of the validated automatic method for the quantification of fibrotic abnormalities on HRCT scans (quantitative CT).
**Ethics committee approval:** No information provided by the submitter.
**Funding for this study:** No funding was received for this study.
**Author Disclosures:**
Ludovica Iaccarino: Nothing to disclose
Giacomo Sgalla: Nothing to disclose
Annemilia Del Ciello: Nothing to disclose
Luca Richeldi: Nothing to disclose
Anna Rita Larici: Nothing to disclose
Giuseppe Cicchetti: Nothing to disclose
Lucio Calandriello: Nothing to disclose
Alessandra Farchione: Nothing to disclose

### RPS 1504-12

**Are pre-existing interstitial lung abnormalities (ILAs) a risk factor for the development of fibrotic changes during follow-up of Covid-19 patients?**
*D. Colombi*[1], M. Petrini[1], N. Morelli[1], N. Sverzellati[2], E. Michieletti[1]; [1]Piacenza, PC/IT, [2]Parma/IT
*(colombidavide@gmail.com)*

**Purpose:** To investigate if pre-existing interstitial lung abnormalities (ILAs) are predictors for the development of fibrotic-like changes during follow-up of patients with Covid-19 pneumonia.
**Methods or Backround:** We selected patients, admitted to the emergency department (ED) of our hospital between March and December 2020, with pandemic chest computed tomography (CT) typical pattern (alone or associated with other pathology), positive nasal-pharyngeal swab for SARS-CoV-2, pre-pandemic chest CT, and follow-up CT for Covid-19 performed at least 6 months after pandemic CT. Pre-pandemic CT was evaluated for the presence of ILAs, pandemic CT for the visual extent of Covid-19 pneumonia expressed as percentage of the whole lung volume, and follow-up CT for the development of fibrotic-like changes defined as reticular lesions, bronchial dilatation, and subpleural cystic lesions. Multivariate logistic regression analysis was used to assess the relationship between the development of fibrotic-like changes at follow-up CT and pre-existing ILAs.
**Results or Findings:** 32 patients (median age 67 years, IQR 63-74 years; 19/32, 59% males) were included in the study. Pre-existing ILAs were identified in 7/32 (22%) patients, while fibrotic-like changes at follow-up CT in 10/32 (31%) cases. At multivariate logistic regression analysis, platelet count at ED admission (OR 0.97, 95% CI 0.95-0.99, P=0.03), glutamic pyruvic transaminase levels at ED admission (OR 1.04, 95% CI 1.00-1.07, P=0.03), and pre-existing ILAs (OR 8.75, 95% CI 1.00-91.2, P=0.04) were significant predictors of fibrotic-changes development.
**Conclusion:** Pre-existing ILAs are predictors of fibrotic-like changes development at more than 6 months follow-up CT of Covid-19 patients.
**Limitations:** Small sample. Retrospective study from a single hospital.
**Ethics committee approval:** -
**Funding for this study:** No funding was received for this study.
**Author Disclosures:**
Emanuele Michieletti: Nothing to disclose
Marcello Petrini: Nothing to disclose
Davide Colombi: Nothing to disclose
Nicola Sverzellati: Nothing to disclose
Nicola Morelli: Nothing to disclose

**14:00-15:30 Research Stage 2**

### Research Presentation Session: Genitourinary

### RPS 1507

### AI in genitourinary imaging: will it improve your performance?

**Moderator**
R. Cuocolo; Naples/IT

### RPS 1507-2

**Predicting the recurrence risk of renal cell carcinoma after nephrectomy: potential role of CT radiomics for adjuvant treatment decisions**
*D. Deniffel*[1], K. McAlpine[2], F. N. Harder[2], R. Jain[2], G. M. Healy[3], E. Salinas-Miranda[2], T. van der Kwast[2], A. Finelli[2], M. Haider[2]; [1]Munich/DE, [2]Toronto, ON/CA, [3]Dublin/IE
*(dominik.deniffel@googlemail.com)*

**Purpose:** Previous trial results suggest that only a small number of patients with non-metastatic renal cell carcinoma (RCC) benefit from adjuvant therapy, highlighting the need for improved patient selection. We assessed whether the addition of CT-based radiomics to established clinico-pathological biomarkers improved recurrence risk prediction for adjuvant treatment decisions.
**Methods or Backround:** This retrospective study included 453 patients with non-metastatic RCC undergoing nephrectomy. Cox models were trained to predict disease-free survival (DFS) using postoperative biomarkers (age, stage, tumor size, grade) with and without radiomics selected on preoperative CT. Models were assessed using C-statistic, calibration, and decision curve analyses (repeated 10-fold cross-validation).
**Results or Findings:** At multivariable analysis, one of four selected radiomic features (wavelet-HHL_glcm_ClusterShade) was prognostic for DFS with an adjusted hazard ratio (HR) of 0.44 (95% CI:0.22–0.88; p=0.02), along with AJCC stage group (III versus I, HR 2.90, 95% CI:1.47–5.75; p=0.002), Fuhrman grade 4 (versus grade 1, HR 8.90, 95% CI:2.42–32.71; p=0.001), age (per 10 years HR 1.29, 95% CI:1.03–1.61; p=0.03), and tumour size (per cm HR 1.13, 95% CI:1.04–1.22; p=0.003). Discriminatory ability of the combined clinical-radiomic model (C=0.80) was superior to that of the clinical base model (C=0.78; p<0.001). Decision curve analysis revealed a net benefit of the combined model when used for adjuvant treatment decisions. At a threshold probability of ≥25% for disease recurrence within 5 years, using the combined versus the clinical model was equivalent to treating 9 additional patients (per 1000 assessed) who would recur without treatment (i.e.,true-positive predictions) with no increase in false-positive predictions.
**Conclusion:** Adding CT-based radiomic features to established prognostic biomarkers improved postoperative recurrence risk assessment and may help guide decisions regarding adjuvant therapy.
**Limitations:** Retrospective study, lack of external validation.
**Ethics committee approval:** This study was approved by the Institutional Research Ethics Board.
**Funding for this study:** Funding was received from the Ontario Institute for Cancer Research and Deutsche Forschungsgemeinschaft (DFG).
**Author Disclosures:**
Kristen McAlpine: Nothing to disclose
Theodorus van der Kwast: Nothing to disclose
Antonio Finelli: Nothing to disclose
Rahi Jain: Nothing to disclose
Dominik Deniffel: Nothing to disclose
Gerard Michael Healy: Nothing to disclose
Masoom Haider: Nothing to disclose
Felix N. Harder: Nothing to disclose
Emmanuel Salinas-Miranda: Nothing to disclose

### RPS 1507-3

**Radiomics analysis on CT images for evaluation of kidney function in ADPKD**
*C. Cipri*, L. Larosa, L. M. Minordi, L. Calvaruso, P. M. Ferraro, L. Boldrini, H. E. Tran, L. Natale; Rome/IT
*(c.cipri@hotmail.it)*

**Purpose:** We aim to assess the capacity of radiomic features based on computed tomography to predict kidney function among patients with autosomal dominant polycystic kidney disease (ADPKD) for determination of total kidney volume (TKV).
**Methods or Backround:** We retrospectively selected 58 patients with ADPKD who underwent CT examination in 2021, of which 31 patients with eGFR ≥ 60 mL/min/1.73 m2 (class 0) and 27 with eGFR < 60 mL/min/1.73 m2 (class 1).

For each CT scan, an expert radiologist generated a region of interest (ROI) segmentation for cystic kidney compounds, obtaining 58 ROIs from which 217 radiomic features were extracted using a dedicated software. Three different logistic regression models were built: a height-adjusted TKV (Ht-TKV) model, a radiomic model based on the most statistically significative radiomic feature from univariate analysis (F_cm.sum.var), and a model from the combination of the above. Area under the curve (AUC) of the receiver operating characteristic (ROC) and accuracy were used to evaluate models' performance in discriminating between the two eGFR classes. We finally performed an internal 3-fold cross-validation (CV).
**Results or Findings:** The Ht-TKV, radiomic, and combined models presented, respectively, an AUC (95% confidence interval) of 0.79 (0.67-0.91), 0.84 (0.73-0.94), 0.85 (0.75-0.95), confirmed by the CV. Mean (standard deviation) values of the accuracy over CV iterations were 0.67(0.09), 0.78(0.08), and 0.79(0.08) for the three models.
**Conclusion:** Based on the quantitative results, we demonstrated that the Ht-TKV-radiomic combined model based on CT images from polycystic kidneys resulted in the most effective prediction of baseline kidney function in our cohort.
**Limitations:** Further studies will be needed to confirm the role of radiomics in ADPKD management, through implementation of a model extension to predict kidney function slope.
**Ethics committee approval:** No information provided by the submitter.
**Funding for this study:** No funding was received for this study.
**Author Disclosures:**
Luca Boldrini: Nothing to disclose
Luigi Larosa: Nothing to disclose
Luigi Natale: Nothing to disclose
Pietro Manuel Ferraro: Nothing to disclose
Huong Elena Tran: Nothing to disclose
Carla Cipri: Nothing to disclose
Luca Calvaruso: Nothing to disclose
Laura Maria Minordi: Nothing to disclose

### RPS 1507-4

### The role of radiomics analysis in the assessment of renal nodules on CT

*A. Zannotti*, F. Ventura, G. Giacomelli, E. Tola, A. Cimadamore, A. Borgheresi, A. Agostini, A. B. Galosi, A. Giovagnoni; Ancona/IT

**Purpose:** To develop a radiomics model for the characterisation of renal nodules on CT.
**Methods or Backround:** Patients who underwent surgical resection of the renal nodule, with preoperative CT (LightSpeed VCT, GE) with contrast agent (Iopamidol 370; 1.5 ml/kg) and availability of a histopathological report, were retrospectively included. Renal lesions were segmented by two radiologists in consensus, in the arterial phase on the axial section with greater diameter. The radiomics analysis was performed with validated software (PyRadiomics on Syngo.via Frontier, Siemens) by applying a bin-width of 4 (analysis with ImageJ, NIH). The data were normalised with 1x1x1 resampling and a filter was applied (LogSigma 1.5). The extracted features were analysed with univariate and multivariate analysis (endpoint: clear cell carcinoma, RCC).
**Results or Findings:** 45 patients were included (mean age 56 years; 32/45 RCC). Multivariate analysis selected two features (from first order and GLRLM groups, respectively) as independent factors associated with RCC ($p < 0.001$).
**Conclusion:** Radiomics analysis is feasible and effective in the characterisation of renal lesions on CT.
**Limitations:** Limited number of patients included in the study. Single center retrospective study.
**Ethics committee approval:** No information provided by the submitter.
**Funding for this study:** No funding was received for this study.
**Author Disclosures:**
Alice Zannotti: Nothing to disclose
Fiammetta Ventura: Nothing to disclose
Gloria Giacomelli: Nothing to disclose
Andrea Agostini: Nothing to disclose
Elisabetta Tola: Nothing to disclose
Alessia Cimadamore: Nothing to disclose
Andrea Benedetto Galosi: Nothing to disclose
Alessandra Borgheresi: Nothing to disclose
Andrea Giovagnoni: Nothing to disclose

### RPS 1507-5

### Deep learning algorithm performs similarly to radiologists in the assessment of prostate volume on MRI

*E. Thimansson*, J. Bengtsson, E. Baubeta, J. Engman, D. Flondell Sité, A. Bjartell, S. Zackrisson; Malmö/SE

**Purpose:** Prostate volume (PV) in combination with prostate-specific antigen (PSA) yields PSA density, which is an increasingly important biomarker. Calculating PV from MRI is a time-consuming, radiologist-dependent task. The aim of this study was to assess whether a deep learning algorithm can replace PI-RADS 2.1 based ellipsoid formula (EF) for calculating PV.
**Methods or Backround:** Eight different measures of PV were retrospectively collected for each of 124 patients who underwent radical prostatectomy and preoperative MRI of the prostate (multicentre and multi-scanner MRI´s 1,5 and 3 T). Agreement between volumes obtained from the deep learning algorithm (PVDL) and ellipsoid formula by two radiologists (PVEF1 and PVEF2) was evaluated against the reference standard PV obtained by manual planimetry by an expert radiologist (PVMPE). A sensitivity analysis was performed using a prostatectomy specimen as the reference standard. Inter-reader agreement was evaluated between the radiologists using the ellipsoid formula and between the expert and inexperienced radiologists performing manual planimetry.
**Results or Findings:** PVDL showed better agreement and precision than PVEF1 and PVEF2 using the reference standard PVMPE (mean difference [95% limits of agreement] PVDL: -0.33 [-10.80; 10.14], PVEF1: -3.83 [-19.55; 11.89], PVEF2: -3.05 [-18.55; 12.45]) or the PV determined based on specimen weight (PVDL: -4.22 [-22.52; 14.07], PVEF1: -7.89 [-30.50; 14.73], PVEF2: -6.97 [-30.13; 16.18]). Inter-reader agreement was excellent between the two experienced radiologists using the ellipsoid formula and was good between expert and inexperienced radiologists performing manual planimetry.
**Conclusion:** A commercially available deep learning algorithm performs similarly to radiologists in the assessment of prostate volume on MRI.
**Limitations:** Public datasets with reference standard were included in training of the algorithm when performance test could not be performed.
**Ethics committee approval:** This study was approved by the local ethics review committee at Lund University (entry no. 2014-886) and the Swedish Ethical Review Authority (entry no. 2019-03674).
**Funding for this study:** Funding was received from the Government funding progam ALF, Region Skåne, Sweden and the Stig and Ragna Gorthon Foundation, Helsingborg, Sweden.
**Author Disclosures:**
Erik Baubeta: Nothing to disclose
Despina Flondell Sité: Nothing to disclose
Anders Bjartell: Research/Grant Support: AB has received grants/research supports from Ferring, Bayer and Merck.
Erik Thimansson: Speaker: Speaker fees recieved from Siemens Healthineers
Johan Bengtsson: Nothing to disclose
Sophia Zackrisson: Speaker: SZ has received speaker fees from Siemens Healthcare AB and Pfizer AB.
Jonatan Engman: Nothing to disclose

### RPS 1507-6

### Development of radiomic models for improving the detection of clinically significant cancers among PI-RADS 4 and 5 lesions detected on 3T multiparametric MRI studies

*P. A. Bonaffini*[1], P. N. Franco[1], A. Corsi[1], E. De Bernardi[2], M. Occhipinti[3], G. Muscogiuri[4], M. Roscigno[1], L. F. Da Pozzo[1], S. Sironi[1]; [1]Bergamo/IT, [2]Monza/IT, [3]Liege/BE, [4]Milan/IT
*(pa.bonaffini@gmail.com)*

**Purpose:** Although the Prostate Imaging-Reporting and Data System (PI-RADS) criteria have high diagnostic accuracy, lesions scored as PI-RADS 4 and 5 may include false positives. This study aims to identify radiomic features that may support the detection of clinically significant tumours among PI-RADS 4/5 lesions.
**Methods or Backround:** Patients undergoing a 3T scan multiparametric MRI (mpMRI) for clinical suspicion of prostate cancer or during active surveillance were retrospectively enrolled. Pathological findings from fusion MRI-targeted biopsies served as ground truth. Clinical (age, PSA, PSA-density) and MRI conventional parameters (prostate volume, mean ADC) were collected. Lesions were manually contoured on itk-SNAP on axial T2-weighted images and ADC maps; volumes of interest were also obtained. Radiomic features were extracted with Pyradiomics. Clinical and radiomic features best correlating with final histopathological results were selected. Diagnostic values were assessed on validation samples.
**Results or Findings:** The final cohort included 99 patients (median age 69 years, IQR 59-79) and 111 PI-RADS 4/5 lesions. At pathology, 79 lesions (71%) were identified as clinically significant cancers (Gleason score ≥ 7). The best predictive clinical (PSA density) and radiomic multivariate zone-ignorant model (including 2 features from T2 and 1 from ADC) showed the following diagnostic values: sensitivity 79%, specificity 80%, positive predictive value (PPV) 91%, negative predictive value (NPV) 63%, accuracy 79%. The most accurate radiomic multivariate model (2 T2 features, 1 ADC) considering exclusively peripheral zone lesions showed even more promising values: sensitivity 86%, specificity 80%, PPV 93%, NPV 70%, accuracy 84%.
**Conclusion:** MRI-based radiomic features analysis can potentially improve the accuracy of subjective mpMRI assessment in discriminating clinically significant cancers among PI-RADS 4/5 lesions.
**Limitations:** Limited sample size. Lack of surgical correlation.
**Ethics committee approval:** This study was conducted following the Declaration of Helsinki, and approved by the local Papa Giovanni XXIII IRB (code ‘mpMR e Sorveglianza Attiva’ - 07/06/2018).
**Funding for this study:** No funding was received for this study.

**Author Disclosures:**
Sandro Sironi: Nothing to disclose
Mariaelena Occhipinti: Nothing to disclose
Marco Roscigno: Nothing to disclose
Pietro Andrea Bonaffini: Nothing to disclose
Andrea Corsi: Nothing to disclose
Paolo Niccolò Franco: Nothing to disclose
Luigi Filippo Da Pozzo: Nothing to disclose
Giuseppe Muscogiuri: Nothing to disclose
Elisabetta De Bernardi: Nothing to disclose

### RPS 1507-7

**Deep learning algorithm for tumour segmentation and classification of aggressiveness in patients with prostate cancer**

*S. Hong*[1], S. H. Kim[1], B. Yoo[2], J. Y. Kim[1]; [1]Busan/KR, [2]Seoul/KR
*(fatale27@naver.com)*

**Purpose:** To investigate the feasibility of a deep learning algorithm (DLA) based on apparent diffusion coefficient (ADC) maps for segmentation and classification (Gleason score [GS] 6 versus ≥7) of aggressiveness in patients with prostate cancer (PCa).
**Methods or Backround:** 149 consecutive patients who had undergone 3T-MRI and been pathologically diagnosed with PCa through radical prostatectomy were initially collected between October 2018 and March 2022. To develop the DLA, labeling of axial ADC images (b2000) was performed by two radiologists. The labelled data (148 images for GS6, 580 images for GS7) were applied for tumour segmentation using a convolutional neural network (CNN). For classification, a total of 93 images for GS6 and 372 images for GS7 were used after filtering. For external validation, 22 consecutive patients (25 images for GS6, 70 images for GS7) representing 5 different MR machines were recruited. The ground truth for tumour segmentation and classification were pathologic topographic maps and GS, respectively.
**Results or Findings:** In terms of segmentation and classification, U-Net and DenseNet were used for CNN, respectively. The gland Dice scores for internal and external validation were 0.951 and 0.9413, respectively, while the corresponding tumour scores were 0.822 and 0.7776, respectively. As for classification, the accuracies of internal and external validation were 73 and 75%, respectively. Specifically, for external validation, diagnostic predictive values of 48, 84, 52, and 82% (sensitivity, specificity, positive predictive value, and negative predictive value, respectively) were estimated for GS6, while those for GS7 were 84, 48, 82, and 52%, respectively.
**Conclusion:** Tumour segmentation and classification of PCa by a DLA developed based on ADC maps (b2000) alone is feasible.
**Limitations:** Tumour segmentation was conducted on a two-dimensional-image basis and only GS7 tumours were included in the CSC group.
**Ethics committee approval:** This retrospective study was approved by the institutional review board, and informed consent from patients was waived.
**Funding for this study:** No funding was received for this study.
**Author Disclosures:**
Joo Yeon Kim, Inje University College of Medicine, Haeundae Paik Hospital: Nothing to disclose
Byeongcheol Yoo: Employee: Deepnoid, Seoul, Korea
Seung Ho Kim: Nothing to disclose
Sujin Hong: Nothing to disclose

### RPS 1507-8

**External validation of a radiomics model for the assessment of extraprostatic extension (EPE) of prostate cancer (PCa)**

*G. Giacomelli*, E. Tola, F. Ventura, T. Valeri, L. Pierpaoli, A. Borgheresi, A. Agostini, A. B. Galosi, A. Giovagnoni; Ancona/IT

**Purpose:** To internally train and validate on an external cohort a radiomics model for the prediction of extraprostatic extension (EPE) of prostate cancer (PCa) in MRI.
**Methods or Backround:** We included patients who underwent robotic radical prostatectomy (2018–2021) with preoperative MRI (1.5T, PI-RADS protocol: Siemens Aera in the training cohort, Philips Achieva in the validation cohort) and available pathological reports; patients who underwent hormone therapy and with intraparenchymal index (IL) lesions were excluded. On small Field-of-View (FOV) axial T2 images, two readers in consensus placed two circular Regions-Of-Interest (ROI, diameter 15 mm), the first over the index lesion, capsule, and periprostatic fat, and the second one subtracting the lesion. The radiomics analysis was performed with validated software (PyRadiomics on Syngo.viaFrontier, Siemens). The extracted features were analysed with univariate and multivariate analysis.
**Results or Findings:** 25 patients (mean age 65 years, 15 EPE) and 20 patients (mean age 66 years, 6 EPE) were included in the training and validation cohorts, respectively. The model demonstrated good performance in terms of sensitivity (>65%), specificity (>80%), and positive predictive value (>70%) for the presence of EPE in both cohorts.
**Conclusion:** The proposed radiomics model is valid with acceptable diagnostic performance in an external
cohort of validation.
**Limitations:** Limited number of patients included in the study. Retrospective study.
**Ethics committee approval:** No information provided by the submitter.
**Funding for this study:** No funding was received for this study.
**Author Disclosures:**
Fiammetta Ventura: Nothing to disclose
Tommaso Valeri: Nothing to disclose
Gloria Giacomelli: Nothing to disclose
Luca Pierpaoli: Nothing to disclose
Andrea Agostini: Nothing to disclose
Elisabetta Tola: Nothing to disclose
Andrea Benedetto Galosi: Nothing to disclose
Alessandra Borgheresi: Nothing to disclose
Andrea Giovagnoni: Nothing to disclose

### RPS 1507-9

**Prediction of clinically significant prostate cancer in patients under active surveillance: performance of a fully-automated AI-algorithm for lesion detection and classification**

*B. Oerther*[1], H. Engel[1], C. L. Schlett[1], R. Grimm[2], H. von Busch[2], A. Sigle[1], C. Gratzke[1], F. Bamberg[1], M. Benndorf[1]; [1]Freiburg im Breisgau/DE, [2]Erlangen/DE

**Purpose:** Multiparametric MRI (MpMRI) of the prostate has been established as a fundamental tool in the diagnostic workup of prostate cancer (PCa) and improves detection of aggressive tumour subtypes. As numbers of individuals under active surveillance (AS) increase and treatment is required in cases of tumour progression, we evaluated whether an automated AI-driven algorithm can detect clinically significant PCa (csPCa) in patients under AS.
**Methods or Backround:** Consecutive patients under active surveillance who received mpMRI according to the PI-RADSv2.1 protocol and subsequent MR-guided ultrasound fusion (targeted and extensive systematic) biopsy between 2017 and 2020 were retrospectively analysed. Diagnostic performance of a clinically certified AI-driven algorithm was evaluated on both lesion level and patient level regarding the detection of csPCa.
**Results or Findings:** Analysis of 56 patients resulted in a total of 93 target lesions. Patient level sensitivity and specificity of the AI algorithm were 92.5% (37/40) and 31% (5/16) for the detection of ISUP ≥ 1, and 96.4% (27/28) / 25% (7/28) for the detection of ISUP ≥ 2, respectively. The only case of csPCa missed by the AI harbored only one Gleason 7a core out of 47 systematic biopsy cores (previous and subsequent biopsies rendered only non-csPCa).
**Conclusion:** AI-augmented lesion detection and PI-RADS scoring provide a robust tool for detecting progression to clinically significant PCa in patients under AS. Integration in the clinical workflow can serve as reassurance for the reader and streamline reporting, hence improving efficiency and diagnostic confidence.
**Limitations:** AI performance was tested on and acquired from a rather small cohort in a retrospective single-institution environment. Thus, results need to be verified in a larger prospective cohort, ideally in a multi-centre setting.
**Ethics committee approval:** No information provided by the submitter.
**Funding for this study:** No funding was received for this study.
**Author Disclosures:**
Benedict Oerther: Nothing to disclose
August Sigle: Nothing to disclose
Christopher L. Schlett: Nothing to disclose
Robert Grimm: Employee: Siemens Healthineers
Hannes Engel: Nothing to disclose
Matthias Benndorf: Nothing to disclose
Fabian Bamberg: Nothing to disclose
Christian Gratzke: Nothing to disclose
Heinrich von Busch: Employee: Siemens Healthineers

### RPS 1507-10

**Computed tomography image-based radiomic analysis helps differentiate ovarian clear-cell carcinoma from other types of epithelial ovarian cancer**

*J. Ren*, Y-L. He, L. Mao, X. Liu, Y. Li, X. Li, Z. Y. Jin, H-D. Xue; Beijing/CN
*(renjing703@163.com)*

**Purpose:** To develop and validate an integrated clinical-radiomic signature model that preoperatively identifies ovarian clear-cell carcinoma (OCCC) in patients with epithelial ovarian cancer (EOC).
**Methods or Backround:** Two hundred and eighty-two consecutive patients who were newly diagnosed with EOC between January 2016 and May 2021 were divided into a training set (n=225) and testing set (n=57) based on the time of surgery. Seven clinical characteristics were collected and 1218 radiomic features were extracted from each delineated tumor on portal venous-phase computed tomography images. The radiomic signature was built using F-test-based feature selection method and logistic regression algorithm within the 10-fold cross-validation procedure. Next, the clinical model (using clinical

Friday characteristics) and integrated model (using both radiomic signature and clinical characteristics) were constructed using the same procedure. Two senior and three junior radiologists independently interpreted the images in testing set with and without integrated model assistance. The diagnostic performances of the predictive models, radiologists, and radiologists aided by the integrated model were evaluated and compared.
**Results or Findings:** The integrated model showed better diagnostic performance (area under the curve [AUC]: 0.863) than the clinical model (AUC: 0.792) or radiomic signature (AUC: 0.781); the accuracy, sensitivity, and specificity were 77.0%, 64.3%, and 80.0%, respectively. Although no significant difference was found between the diagnostic performance of the integrated model and that of the radiologists, the diagnostic sensitivity of radiologists could be significantly improved with the integrated model assistance.
**Conclusion:** The integrated model shows promise in aiding the accurate pretreatment diagnosis of OCCC among patients with EOC.
**Limitations:** First, it was a retrospective single-centre study. Second, only clinical and radiomic features were considered when constructing our predictive models.
**Ethics committee approval:** This study was approved by the PUMCH Institution Review Board.
**Funding for this study:** Funding was received from the Natural Science Foundation of China (grant No. 81901829).
**Author Disclosures:**
Yong-Lan He: Nothing to disclose
Li Mao: Employee: AI Lab, Deepwise Healthcare, China
Zheng Yu Jin: Nothing to disclose
Hua-Dan Xue: Nothing to disclose
Jing Ren: Nothing to disclose
Xiuli Li: Employee: AI Lab, Deepwise Healthcare, China
Xinyu Liu: Nothing to disclose
Yuan Li: Nothing to disclose

### RPS 1507-11

**CT radiomics model to predict platinum sensitivity in epithelial ovarian carcinoma**
*M. He*, R. Singh, M. Wang, E. Y. P. Lee; Hong Kong/CN
*(u3008315@connect.hku.hk)*

**Purpose:** To assess the value of radiomics model based on contrast-enhanced CT (ceCT) in predicting platinum-based chemotherapy response of epithelial ovarian carcinoma (EOC).
**Methods or Backround:** Consecutive patients with histologically confirmed EOC with pre-treatment ceCT were retrospectively recruited from 2 centres. All patients underwent standard platinum-based chemotherapy before or after optimal cytoreduction. Platinum sensitivity was based on treatment response assessment, with refractory disease or resistant disease defined as disease progression while on platinum-based chemotherapy or disease recurrence within 6 months after completion of platinum-based chemotherapy, respectively; otherwise, they were considered as platinum sensitive. The whole volume of primary tumour was manually delineated on each slice of the baseline ceCT images. Radiomics features were extracted using the open-source package PyRadiomics (version 3.0). All patients were randomly divided into a training set and testing set with a ratio of 3:1. Synthetic minority over-sampling technique (SMOTE) was applied to minimise class biases. The extracted features were reduced using principal component analysis (PCA). Then, Random Forest (RF) classifier with repeated stratified 10-fold cross-validation was applied to build the model.
**Results or Findings:** Two hundred and thirty-eight EOC patients (51.6 ± 9.3 years) were recruited. The proposed RF model yielded classification metrics: area under the curve (0.762), sensitivity (80.0%), specificity (74.5%), and accuracy (80.0%) in testing set in predicting platinum sensitivity.
**Conclusion:** The proposed CT radiomics model could be useful in predicting platinum sensitivity in EOC with potential in guiding personalised treatment in EOC.
**Limitations:** The cohort was relatively small and heavily skewed towards platinum-sensitive group, but distribution was compatible with the reported prevalence of platinum sensitivity in EOC.
**Ethics committee approval:** This study was approved by HKU/HA HKW IRB (Reference No. UW 20-251), IRB of Sun Yat-sen University Cancer Centre (Reference No. YB2018-52).
**Funding for this study:** This work was supported by Health and Medical Research Fund (HMRF No. 08192106), Hong Kong.
**Author Disclosures:**
Rahul Singh: Nothing to disclose
Mandi Wang: Nothing to disclose
Elaine Yuen Phin Lee: Nothing to disclose
Mengge He: Nothing to disclose

### RPS 1507-12

**MRI- and histologic-molecular-based radiogenomics nomogram for preoperative assessment of risk classes in endometrial cancer**
*V. Celli*, R. V. Ninkova, M. Gennarini, V. Miceli, A. Cupertino, M. Dolciami, C. Catalano, L. Manganaro; Rome/IT
*(veronicacelli93@libero.it)*

**Purpose:** High- and low-risk endometrial carcinoma (EC) differ in whether or not lymphadenectomy is performed. We aimed to develop an MRI-based radiogenomics model able to preoperatively assess lymph-vascular space invasion (LVSI) and discriminate between low-risk and high-risk EC according to ESMO-ESGO-ESTRO 2020 guidelines, which include molecular risk classification proposed by “ProMisE”.
**Methods or Backround:** This is a retrospective, multicentric study that included 64 women with EC who underwent 3T MRI before hysterectomy. Radiomics features were extracted from T2WI images and apparent diffusion coefficient maps (ADC) after manual segmentation of the gross tumor volume. We constructed a multiple logistic regression approach from the most relevant radiomics features to distinguish between low- and high-risk classes under the ESMO-ESGO-ESTRO 2020 guidelines. A similar approach was taken to assess LVSI. Model diagnostic performance was assessed via ROC curves, accuracy, sensitivity, and specificity on both the training and test sets.
**Results or Findings:** A total of 240 radiomics features were extracted; to avoid model's overfitting, the features were ultimately reduced to 7 for risk class dataset and to 26 for LVSI; the LVSI predictive model used a single feature from ADC as predictor, the risk class model used 2 features as predictors from both ADC and T2WI. Low-risk predictive model showed AUC of 0.74 with accuracy, sensitivity, and specificity of 0.74, 0.76, 0.94; LVSI model showed AUC of 0.59 with accuracy, sensitivity, and specificity of 0.60, 0.50, 0.61.
**Conclusion:** MRI-based radiogenomics models are useful for preoperative EC risk-stratification and may facilitate therapeutic management.
**Limitations:** The small group of patients limits the number of radiomics features considered in the radiogenomic model in order to avoid model overfit.
**Ethics committee approval:** -
**Funding for this study:** This research received no external funding.
**Author Disclosures:**
Marco Gennarini: Nothing to disclose
Valentina Miceli: Nothing to disclose
Miriam Dolciami: Nothing to disclose
Angelica Cupertino: Nothing to disclose
Roberta Valerieva Ninkova: Nothing to disclose
Veronica Celli: Nothing to disclose
Carlo Catalano: Nothing to disclose
Lucia Manganaro: Nothing to disclose

**14:00-15:30 Research Stage 3**

### Research Presentation Session: Musculoskeletal

### RPS 1510

### Musculoskeletal: shoulder, pelvis and hip

**Moderator**
R. Sutter; Zurich/CH
**Author Disclosures:**
Reto Sutter: Author: Breitenseher Publisher; Other: Balgrist University Hospital has an academic research collaboration with Siemens Healthineers, Balzano Informatik and Bayer

### RPS 1510-2

**The incidence rate of internal rotation of the arm during shoulder MRI at Jordanian imaging centres**
*M. A. Ayasrah*; Irbid/JO
*(mohd.sakran@gmail.com)*

**Purpose:** This retrospective study was conducted to assess the prevalence rate of arm internal rotation during routine MRI exams digitally by using the evaluation tool of Picture and Archiving and Communication system (PCAS) in various MRI centres in Jordan.
**Methods or Backround:** A total of 58 valid shoulder MRI cases were recruited for the study from 12 MRI centres which participated. Using PACS viewer software, the prevalence rate of humeral head rotation during shoulder MRI, which reflected suboptimal factors that may endanger diagnostic accuracy, was digitally assessed.
**Results or Findings:** Nearly 1/3rd of the shoulder MRI (37%) conducted in the period of the study were in internal rotation of the arm. The range of rotation for the internal rotation humeral head for the reference volunteers was from 44.2°to 23.1. The range of motion was 80.7°.
**Conclusion:** The results of this study showed that 1/3rd of shoulder MRI examinations conducted at Jordanian imaging centres were performed in the suboptimal arm internal rotation position, which constituted a high prevalence rate. This might jeopardise the diagnostic accuracy of shoulder MRI and increase unnecessary healthcare costs.
**Limitations:** The results represent the MRI facilities in Jordan.
**Ethics committee approval:** Approval of the Institutional Review Board of Jordan University of Science and Technology (127/136/2020) was obtained.
**Funding for this study:** Not applicable.
**Author Disclosures:**
Mohammad Ahmad Ayasrah: Nothing to disclose

### RPS 1510-3

**Imaging of rotator cuff pathologies: a magnetic resonance imaging and ultrasonography correlation**
*M. M. Zurale*, A. A. Patil; Kolhapur/IN
*(mrinz18@gmail.com)*

**Purpose:** The study aimed to compare the diagnostic accuracy of ultrasonography in diagnosing rotator cuff pathologies by comparing its findings with MR imaging performed simultaneously on the same patient. Furthermore, the objective was to evaluate the other imaging findings associated with different types of rotator cuff pathologies in ultrasound and MRI.
**Methods or Backround:** This is a prospective study that included 50 patients. The inclusion criteria were (1) a history of pain in the shoulder and restriction of movements and (2) a history of trauma. The exclusion criteria were (1) a previous history of surgery or prosthesis and (2) patients with pacemakers, metallic implants or claustrophobia.
**Results or Findings:** On comparing USG with MRI for supraspinatus pathologies, diagnostic accuracy ranged between 92 to 100%. On comparing USG with MRI for subscapularis pathologies, diagnostic accuracy ranged between 88 to 100%. On comparing USG with MRI for infraspinatus pathologies, diagnostic accuracy ranged between 86 to 98%. Diagnostic accuracy for detecting full-thickness tear with retraction on USG compared to MRI was 98% for the supraspinatus tendon, 100% for the subscapularis tendon and 98% for the infraspinatus tendon. Diagnostic accuracy for detecting subacromial subdeltoid bursitis and subcoracoid bursitis on USG compared to MRI was 98% and 100%, respectively. Diagnostic accuracy for detecting subacromial impingement of the supraspinatus tendon and subcoracoid impingement of the subscapularis tendon was found to be 96% and 96%, respectively, with USG having the added advantage of the ease of dynamic assessment.
**Conclusion:** The diagnostic accuracy of ultrasound is comparable to MRI in evaluating rotator cuff pathologies and has the added value of dynamic evaluation for impingement. Modality choice for evaluating rotator cuff pathology should be based on several factors like availability, patient preference and clinical information being sought.
**Limitations:** No limitations were identified.
**Ethics committee approval:** The study has been approved by the institutional ethics committee at Apple Saraswati Multispecialty Hospital, Kolhapur, India.
**Funding for this study:** No funding was received for this study.
**Author Disclosures:**
Ashish Anandrao Patil: Nothing to disclose
Mrinmayi Mahesh Zurale: Nothing to disclose

### RPS 1510-4

**Biceps tendon tear at elbow: flexed elbow, abducted shoulder, forearm supinated (FABS) reconstruction from 3D Elbow MRI: inter-reader reliability and diagnostic performance assessment**
*V. A. Aggarwal*, U. Thakur, P. P. Gowda, Y. Xi, A. Chhabra; Dallas, TX/US
*(vikram.aggarwal@utsouthwestern.edu)*

**Purpose:** We aimed to digitally reconstruct flexed, abduction, and supination (FABS) virtual imaging from 3D isotropic elbow MRI and determine inter-reader and diagnostic performance in identifying biceps pathology.
**Methods or Backround:** In this case-control study, two fellowship-trained musculoskeletal radiologists and four musculoskeletal radiology fellows independently evaluated 2D and 3D MRIs of the elbows with FABS reconstructions, blinded to the clinical findings and final diagnosis. Each recorded a binary result as to whether they believed the tendon was intact and if both heads were visible, along with assigning a categorical value to the type of tear (partial, complete, near-complete tears, and myotendinous strain) and the extent of retraction in centimetres where applicable. Kappa and interclass correlation (ICC) were reported with 95% confidence intervals. Areas under the receiver operating curve (AUC) were reported.
**Results or Findings:** FABS reconstructions were obtained in all cases with majority showing good-excellent quality. With respect to tendon intactness, and type of tear, Kappa values were 0.66 (0.53-0.78) and 0.55 (0.43-0.66), respectively. For the extent of retraction, the ICC was 0.85 (0.79-0.91) when including the tendons with and without retraction, and 0.78 (0.61-0.91) when only including tendons with retraction. For tear vs. no tear, AUC values were 0.90 (0.81-0.98), p value of 7.89 e-19; 0.92 (0.84-1.00), 1.54 e-24; 0.90 (0.81-0.98), 7.89 e-19; 0.96 (0.91-1.01), 3.29 e-67; 0.89 (0.80-0.97), 4.66e-18; 0.82 (0.74-0.89), 1.66e-15.
**Conclusion:** Digital reconstruction of FABS positioning allows excellent assessment of individual biceps head tears and retraction with high diagnostic performance across both senior expert and junior fellow readers.
**Limitations:** Reconstructions were difficult to standardize as they relied on previously collected, unstandardized MRIs. Furthermore, previous MRIs were limited to those from our institution only. Finally, our readers had different levels of training.
**Ethics committee approval:** This study was approved by the IRB.
**Funding for this study:** No funding was received for this study.
**Author Disclosures:**
Avneesh Chhabra: Shareholder: Jaypee and Wolters Speaker: Siemens Research/Grant Support: Image biopsy lab Inc. Consultant: ICON Medical and Treace Medical Concepts, Inc.
Vikram Aditya Aggarwal: Nothing to disclose
Uma Thakur: Nothing to disclose
Prajwal P Gowda: Nothing to disclose
Yin Xi: Nothing to disclose

### RPS 1510-5

**Ultrasound examination of the shoulder shows good results in distinguishing between partial or complete rotator cuff tears according to Snyder's classification**
*C. Fanciullo*[1], M. Porta[1], S. La Marca[2], A. Aliprandi[1]; [1]Monza/IT, [2]Milan/IT

**Purpose:** Snyder's classification allows an immediate distinction between partial (articular A or bursal B) and complete (C) lesions of the rotator cuff. The aim of our work is to demonstrate the validity of this classification in ultrasound and compare it with the shoulder arthro-MRI.
**Methods or Backround:** From June 2021 to January 2022, 25 patients underwent a shoulder ultrasound (US) according to the ESSR guidelines, and subsequently an arthro-MRI for various diagnostic purposes. Two double-blind operators assigned A1-4, B1-4, or C1-4 values, depending on the extent of the lesion, in both US and MRI and then the interobserver correlation and agreement between US and MRI were calculated using the MR evaluation in agreement among readers as a gold standard.
**Results or Findings:** Very good US-RM agreement was obtained for both readers (k = 0.64, k = 0.72) considering all individual lesions; perfect agreement considering only partial A-B or complete C lesions without considering subcategories 1-4; and a moderate agreement between the two readers in US (k = 0.48).
**Conclusion:** Snyder's classification can be used in ultrasound by ensuring a correct distinction between partial or complete rotator cuff injuries, thus providing a precise surgical indication.
**Limitations:** Intrinsic to musculoskeletal ultrasound accuracy is the issue of observer variability. Moreover, the US-MR agreement was perfect only considering distinction between partial A-B vs. complete C lesion without considering subcategories 1-4.
**Ethics committee approval:** No information provided by the submitter.
**Funding for this study:** No funding was received for this study.
**Author Disclosures:**
Salvatore La Marca: Nothing to disclose
Cristiana Fanciullo: Nothing to disclose
Marco Porta: Nothing to disclose
Alberto Aliprandi: Nothing to disclose

### RPS 1510-6

**Application of diffusion microstructure imaging in musculoskeletal radiology: translation from head to shoulders**
A. Rau, P. M. Jungmann, T. D. Diallo, M. Reisert, E. Kellner, M. Eisenblaetter, F. Bamberg, *M. Jung*; Freiburg/DE

**Purpose:** Quantitative MRI techniques, such as diffusion microstructure imaging (DMI), are increasingly applied for advanced tissue characterisation. We determined its value in the rotator cuff (RC) muscle imaging by studying DMI parameters' association with isometric strength and a fat fraction (FF).
**Methods or Backround:** Healthy individuals prospectively underwent 3T-MRI of the shoulder using DMI and chemical shift encoding-based water-fat imaging. RC muscles were segmented, and quantitative MRI metrics (V-ISO – free fluid, V-intra – compartment inside of muscle fibres, V-extra – compartment outside of muscle fibres, and FF) were extracted. Isometric shoulder strength was quantified using specific clinical tests. Sex-related differences were assessed with student´s t. The association of DMI metrics, FF, and strength was tested. A factorial two-way ANOVA was performed to compare the main effects of sex and external/internal strength-ratio and their interaction effects on quantitative imaging parameters ratios of infraspinatus/subscapularis.

**Results or Findings:** Among 22 participants (mean age: 26.7±3.1 years, 50% female, mean BMI: 22.6±1.9 kg/m2), the FF of the individual RC muscles did not correlate with strength or DMI parameters (all p>0.05). Subjects with higher V-intra (r=0.57 to 0.87, p<0.01) and lower V-ISO (r=-0.6 to -0.88, p<0.01) had higher internal and external rotation strength. Moreover, V-intra was higher, and V-ISO was lower in all RC muscles in males compared to female subjects (all p<0.01). There was a sex-independent association of external/internal strength-ratio with the ratio of V-extra of infraspinatus/subscapularis (p=0.02).
**Conclusion:** Quantitative DMI parameters may provide incremental information about muscular function and microstructure in young athletes and may serve as a potential quantitative imaging biomarker.
**Limitations:** Small sample size. Further adaptation of the DMI model to muscle tissue is needed.
**Ethics committee approval:** This prospective study was approved by the ethics committee of the University of Freiburg, Germany (EK:1446/21).
**Funding for this study:** The study is funded by the German Society of Musculoskeletal Radiology (DGMSR).
**Author Disclosures:**
Marco Reisert: Nothing to disclose
Alexander Rau: Nothing to disclose
Matthias Jung: Nothing to disclose
Fabian Bamberg: Nothing to disclose
Elias Kellner: Nothing to disclose
Pia M Jungmann: Nothing to disclose
Michel Eisenblaetter: Nothing to disclose
Thierno D. Diallo: Nothing to disclose

### RPS 1510-7

### Arthro-MRI of the shoulder: indications, diagnostic accuracy and structured report

*S. Gómez Peña*, L. Escudero González, A. Crespo Rodríguez, M. J. Moreno Casado; Madrid/ES
*(sagompe@gmail.com)*

**Purpose:** The purpose of this study was to evaluate the radiological diagnostic accuracy of the shoulder MR arthrography (arthro-MRI) compared to arthroscopy as the reference standard.
**Methods or Backround:** Shoulder arthro-MRIs performed in our centre in the last five years were reviewed. Patient records were reviewed to collect the following data: age, sex, the motive for the study, previous MRIs, previous surgeries and subsequent arthroscopy or surgery. Surgical and imaging reports, as well as imaging tests, were reviewed. The imaging findings were grouped into the following categories: labral affectation (SLAP, soft-tissue Bankart, paralabral cyst, GARD, POLPSA...), bone lesions (bony Bankart and glenoid fractures, Hill-Sachs, other fractures...), rotator cuff and biceps affectation, glenohumeral ligament injuries, osteoarthritis, adhesive capsulitis and anatomical variants.
**Results or Findings:** A total sample of 126 imaging tests was obtained (83 males and 36 females), of which five patients subsequently underwent surgery (four Latarjet and one thoracic surgery). Three patients underwent a capsulotomy, and 28 underwent arthroscopy. In this group of 28 patients, 78.6% had labral affectation, 53% had bone affectation (four bony Bankarts, and nine Hill-Sachs lesions), and 39.3% had rotator cuff affectation, mainly of the supraspinatus tendon. The remaining pathology and anatomical variants were less frequent.
**Conclusion:** Shoulder arthro-MRI correlates well with arthroscopy and surgery. Possible measures to adopt in arthro-MRI are the systematic inclusion of the ABER projection and the use of a structured report. Structured reports decrease the likelihood of missing less significant radiological findings and facilitate clinical-surgical decision-making and investigation.
**Limitations:** The study is limited by being a retrospective study and including a limited number of patients.
**Ethics committee approval:** No information provided by the submitter.
**Funding for this study:** No funding was provided for this study.
**Author Disclosures:**
Sara Gómez Peña: Nothing to disclose
Ana Crespo Rodríguez: Nothing to disclose
Laura Escudero González: Nothing to disclose
Mª Jose Moreno Casado: Nothing to disclose

### RPS 1510-8

### Hip and shoulder MR arthrography: challenges and pitfalls

*D. E. Salazar Chiriboga*, N. Rodriguez Ramirez, L. Fernandez Vila, J. M. López-Arcas Calleja, J. Carrascoso Arranz, J. Fernandez Jara; Madrid/ES
*(danedusalazar@gmail.com)*

**Purpose:** The study aimed to review the different approaches in the injection techniques for an arthrogram MR of the hip and shoulder, discuss the most common challenges the radiologist can encounter during the procedure and describe how we can overcome some of those difficulties, and outline the most common pitfalls in the radiological interpretation of these studies.
**Methods or Backround:** Analysis of cases performed the past year in three health institutions in Madrid (one public hospital and two private hospitals), each with different workflows (overall +1000 cases). Expert discussion and analysis of the different methods used and review of the most common challenges the radiologist encountered during the procedures. Literature review on the most common pitfalls encountered in the radiological interpretation.
**Results or Findings:** Image-guided injection with ultrasound is a radiation-free modality and is encouraged to use in interventional procedures. Fluoroscopy is still widely used and has some indications and advantages in specific scenarios. Preprocedural planning is mandatory for obtaining efficient results. The most common pitfalls in the radiological interpretation were anatomical variants that simulate pathology.
**Conclusion:** MR arthrography can be done using different types of image-guided injection techniques, and the radiologist must be able to select the best option available according to the patient clinical features and the facilities at his disposal. MR arthrography requires preprocedural planning from the radiologist, including an optimal time to explain the procedure to the patient. Radiologists need to be aware of the anatomical variants present during the radiological interpretation of an MR arthrography of the hip and shoulder and not to mistake those with pathological findings.
**Limitations:** Not applicable.
**Ethics committee approval:** Not applicable
**Funding for this study:** This study received no specific grant from any funding agency in the public, commercial, or not-for-profit sectors.
**Author Disclosures:**
Luis Fernandez Vila: Nothing to disclose
Nicolas Rodriguez Ramirez: Nothing to disclose
Javier Fernandez Jara: Nothing to disclose
José María López-Arcas Calleja: Nothing to disclose
Javier Carrascoso Arranz: Nothing to disclose
Danilo Eduardo Salazar Chiriboga: Nothing to disclose

### RPS 1510-9

### Avascular necrosis of the femoral head: which MRI parameters and findings are more decisive in determining the prognosis at the time of diagnosis?

*B. Yavuz*, T. Cankurtaran, E. Cakmak; Ankara/TR
*(drbusrayavuz@gmail.com)*

**Purpose:** Avascular necrosis of the femoral head (AVNFH) is a disease of the hip that predisposes to osteoarthritis and joint replacement if not diagnosed in the early period. MRI is the gold standard for the diagnosis of AVNFH. Radiological staging of AVNFH has an important role in the diagnosis of the disease, treatment planning, and follow-up.
**Methods or Backround:** A retrospective review was performed on 25 patients (29 hips) diagnosed with AVNFH between January 2014 and September 2021. The hips were classified according to the Kerboul, ARCO, and JIC classification by two independent radiologists. Presence of the other MRI findings (double line sign, bone marrow edema, cyst like changes, synovial effusion) were collected. The percent volume of necrotic area and the combined necrotic angle were measured and calculated. The patients were grouped as those who developed collapse and those who did not. In this way, we investigated whether the MRI parameters based on the classification systems and MRI findings of AVNFH have prognostic value at the time of diagnosis.
**Results or Findings:** Interobserver reliability was evaluated using the kappa and ICC. Interobserver reliability was best for the JIC classification (mean, 0.89) and worst for the Modified Kerboul Grading (mean, 0.49). The success of the percent volume of necrosis and the combined necrotic angle in predicting the development of collapse were compared using the ROC curve. The percent volume of necrosis was found to be more predictive than the combined necrotic angle (0.79 vs 0.74).
**Conclusion:** Our study suggests the performance of MRI parameters in the development of collapse.
**Limitations:** The sample size in our study is small to compare the classifications. Also, we measured MRI parameters manually.
**Ethics committee approval:** This retrospective study was approved by the local ethics commission.
**Funding for this study:** No funding was received for this study.
**Author Disclosures:**
Busra Yavuz: Nothing to disclose
Eda Cakmak: Nothing to disclose
Tijen Cankurtaran: Nothing to disclose

### RPS 1510-10

**Frequency of absolute femoral retroversion of patients with SCFE after in-situ pinning**

*T. D. Lerch*[1], A. Kiapour[2], F. Schmaranzer[1], Y-j. Kim[2], E. Novais[2]; [1]Bern/CH, [2]Boston, MA/US

**Purpose:** The most common treatment for patients with Slipped-capital-femoral-epiphyses (SCFE) is in-situ pinning. Mean femoral version (FV) and frequency of femoral retroversion were investigated.
**Methods or Backround:** A retrospective IRB-approved controlled single-centre study of 112 hips of SCFE patients treated between 2000 and 2017 was performed. We reviewed our institutional database for patients with a diagnosis of unilateral SCFE (age 10-30 years) who were treated for SCFE. All included patients underwent postoperative pelvic CT scan (including the distal femoral condyles) after treatment with in-situ pinning (56 hips). Mean age was 13 years at SCFE diagnosis and 16 years at imaging. Mild SCFE was present in 19 hips(34%), moderate SCFE in 14 hips(25%), and severe SCFE in 23 hips(41%). Contralateral side was used as a control group (56 hips). FV was measured using the Murphy method and absolute femoral retroversion was defined FV<0°.
**Results or Findings:** Mean FV of SCFE patients treated with in-situ pinning (56 hips) was significantly (p<0.001) lower (-2.5±17°) compared to contralateral side (19±14°). Frequency of absolute femoral retroversion (FV<0°) was significantly (p<0.001) higher for SCFE patients (55%, 31 hips) compared to contralateral side (5%, 3 hips). Mean FV of mild SCFE was 0.3±15° (range -34-23°); 8 hips (42%) had absolute femoral retroversion(FV<0°). Mean FV of moderate was -2.8±10° (range -18-19°); 9 hips (64%) had absolute femoral retroversion(FV<0°). Mean FV of severe SCFE was -4.7±22° (range -36-61°); 14 hips (61%) had absolute femoral retroversion(FV<0°).
**Conclusion:** More than half of SCFE patients treated with in-situ pinning had absolute femoral retroversion on postoperative CT scans, ranging from 42% with mild SCFE to 67% with severe SCFE. This could help for evaluation of subsequent surgery (e.g., derotation femoral osteotomy). Postoperative CT could help to detect femoral retroversion after in-situ pinning.
**Limitations:** Radiation exposure of CT scans.
**Ethics committee approval:** No information provided by the submitter.
**Funding for this study:** No funding was received for this study.
**Author Disclosures:**
Till Dominic Lerch: Nothing to disclose
Ata Kiapour: Nothing to disclose
Florian Schmaranzer: Nothing to disclose
Eduardo Novais: Nothing to disclose
Young-jo Kim: Nothing to disclose

### RPS 1510-11

**Ligamentum teres oedema: a new marker in the symptomatic cases of femoroacetabular impingement (FAI)**

*A. Mushtak*, S. Intakhab Alam; Doha/QA
*(adamrads94@gmail.com)*

**Purpose:** The study aimed to introduce ligamentum teres oedema as a novel, non-invasive, reliable and sensitive 'marker' of hip micro-instability in symptomatic femoral acetabular impingement (FAI) and to equip the radiologist with the tools to diagnose/suggest the possibility of FAI confidently.
**Methods or Backround:** A retrospective study was conducted spanning the years 2014 to 2020. Imaging data was extracted from PACS, and clinical data from CERNER database. All patients with ligamentum teres oedema on hip MRI (done between 2014-2022) were included. Patients with a history suggestive of preceding trauma or pain after trauma were excluded. Ligamentum teres oedema was assessed on oblique axial PD- Fat-sat and STIR coronal sequences. The alpha angle, centre-edge angle of Wiberg, and angle of humeral head retroversion were assessed.
**Results or Findings:** Amongst the patients included, 52.7% (n=29) were males, and 47.3 % (n=26) were females. Both hip joints of each patient were considered as two unique individual hip joints in the study, thereby providing a total of 110 hip joints. Out of 55 patients with ligamentum teres oedema, one patient had isolated right-sided FAI, six patients had isolated left-sided FAI, and 48 patients (96 hip joints) had either FAI or ADDH. These seven patients (13 %) with unilateral FAI had a contralateral absence of FAI/ADDH (6.5% of false positives) despite the presence of ligamentum teres oedema on MRI. The rest of the patients (103 hip-joints: 93.5% positive predictive value) had findings of either FAI or DDH. Amongst the 93.5% positive cases (i.e. with ligamentum oedema and FAI/ADDH), 55.3 % had Cam, 8.7% Pincer, 25.2% mixed type FAI respectively, while 7.7% had ADDH.
**Conclusion:** Ligamentum teres oedema is a 'novel' marker in the diagnosis of femoroacetabular impingement (FAI), with significant clinical and radiological implications in detection and management.
**Limitations:** The study is limited by being a retrospective study.
**Ethics committee approval:** Not applicable.
**Funding for this study:** No funding was received for this study.
**Author Disclosures:**
Syed Intakhab Alam: Nothing to disclose
Adam Mushtak: Nothing to disclose

### RPS 1510-12

**Comparison between dedicated MRI and symphyseal fluoroscopic guided contrast agent injection in the diagnosis of cleft sign and pelvic ring instability in athletic groin pain**

*N. Holl*[1], J. S. Gerhardt[1], T. Tischer[1], R. Lenz[1], J. Krüger[2], A. Arevalo-Hernandez[1], M-A. Weber[1]; [1]Rostock/DE, [2]Berlin/DE

**Purpose:** The study aimed to compare dedicated MRI with targeted fluoroscopy-guided symphyseal contrast agent injection regarding the assessment of symphyseal cleft sign and pelvic ring instability in men with athletic groin pain.
**Methods or Backround:** Until present 66 athletic men (mean age: 32 years; 58 soccer players) were included. All patients were referred to our clinic by an experienced surgeon after an initial clinical examination using a standardised procedure. Symphyseal cleft injection of a contrast agent was performed with fluoroscopic guidance. Standing single-leg stance radiography and dedicated 3 Tesla MRI was performed. The presence of superior cleft sign, secondary cleft sign and osteitis pubis were recorded.
**Results or Findings:** Symphyseal bone marrow oedema was present in 50 patients, bilaterally in 41 patients. In MRI, no clefts were diagnosed in 14 patients, isolated superior cleft signs were in 13, isolated secondary cleft signs in 15 patients, and in 18 patients, both cleft signs were present simultaneously (combined injuries). For fluoroscopic examinations, we saw no cleft injuries in 24 patients, isolated superior cleft signs in 10, isolated secondary cleft in 21 patients and 11 combined injuries. In 7 cases, a combined cleft sign was observed in MRI, but only an isolated secondary cleft sign was visible in symphysography. Pelvic ring instability was observed in 25 patients and was linked to a cleft sign in 23 patients (7 superior cleft sign, 8 secondary cleft sign, 6 combined clefts and 2 atypical cleft injuries).
**Conclusion:** Dedicated 3 Tesla MRI outmatches symphysography for purely diagnostic purposes of superior and secondary clefts. Additional symphysography may be important for the therapeutic injection of corticosteroids and local anaesthetics. Microtearing at the prepubic aponeurotic complex is a prerequisite for the development of anterior pelvic ring instability.
**Limitations:** The study is limited by its patient selection.
**Ethics committee approval:** The study was approved by the Ethical Committee of Rostock University (approval No. A 2020-0040).
**Funding for this study:** No funding was received for this study.
**Author Disclosures:**
Andres Arevalo-Hernandez: Nothing to disclose
Judith Sarah Gerhardt: Nothing to disclose
Robert Lenz: Nothing to disclose
Jens Krüger: Nothing to disclose
Norman Holl: Nothing to disclose
Marc-André Weber: Nothing to disclose
Thomas Tischer: Nothing to disclose

**14:00-15:30** **Research Stage 4**

### Research Presentation Session: Vascular

### RPS 1515

### Intracranial vessels and carotid plaque imaging

**Moderator**
A. Van der Lugt; Rotterdam/NL

### RPS 1515-2

**Morphological atlas of the three-dimensional vascular network architecture in the entire developing and adult mouse brain at ultra-high resolution**

*J. Bisschop*[1], A. Miettinen[2], P. Monnier[1], A. Bonnin[3], M. Schmeltz[3], V. Novak[3], J. Vogel[4], M. Stampanoni[3], T. Wälchli[4]; [1]Toronto, ON/CA, [2]Jyväskylä/FI, [3]Villigen/CH, [4]Zürich/CH

**Purpose:** The establishment of cerebrovascular networks is crucial during brain development, acquires relative quiescence in the healthy adult brain, and plays critical roles during the initiation and progression of various CNS pathologies. Quantitative analysis of cerebrovascular morphology along the arterio-venous axis is important to understand better the three-dimensional brain vascular network across development, adulthood, and disease. However, computational analysis and quantification of vascular network datasets in individual brain regions remain challenging. We present a morphological atlas of the 3D vascular network architecture in the entire developing and adult mouse brain at ultra-high resolution.
**Methods or Backround:** We developed a pipeline including resin-based vascular corrosion casting, scanning electron microscopy, synchrotron radiation/desktop-µCT imaging and 3D-computational reconstruction facilitating quantitative analysis of various vascular network parameters. Registration to the Allen Mouse Brain Atlas coordinate system enabled detailed comparisons of anatomical regions between the early postnatal stage of development and adulthood. We anticipate this method could be applied to additional mouse organs and human samples.
**Results or Findings:** We visualised and quantified the entire cerebrovasculature in 19 animals (n=9 postnatal day 10 and n=10 adult wildtype mice). Network parameters vascular volume fraction, branchpoint density, vessel diameter, -length, -tortuosity, -directionality and extravascular distance were obtained at both developmental stages, subdivided into capillaries and non-capillaries. Quantitative findings were interpreted in the context of biological functions of anatomical regions, e.g. brain vascular networks in grey vs white matter structures or correlated with the known neuronal density of a specific brain region.
**Conclusion:** Using vascular corrosion casting, SRµCT-imaging and 3D-computational image analysis, we quantified cerebrovascular architecture to provide a morphological vascular atlas of the developing and adult mouse brain at a previously unmet micrometre resolution.
**Limitations:** The study was invasive, labour-intense, and unperfused vessels are not addressable.
**Ethics committee approval:** All the animal experiments were performed in accordance with governmental, institutional (University of Zurich) and ARRIVE guidelines and had been approved by the Veterinary Office of the Canton of Zurich (license number 173/2010).
**Funding for this study:** The study was funded by the Swiss Cancer Research and OPO-HOPE foundations.
**Author Disclosures:**
Jeroen Bisschop: Nothing to disclose
Philippe Monnier: Nothing to disclose
Anne Bonnin: Nothing to disclose
Johannes Vogel: Nothing to disclose
Thomas Wälchli: Nothing to disclose
Vladimir Novak: Nothing to disclose
Arttu Miettinen: Nothing to disclose
Marco Stampanoni: Nothing to disclose
Margaux Schmeltz: Nothing to disclose

### RPS 1515-3

**Radiological evaluation of middle meningeal artery by 3D rotational angiography**
*L. Sarı*, T. F. Yılmaz, I. I. I. Öz; Istanbul/TR

**Purpose:** The middle meningeal artery (MMA) is the most important dural artery and supplies more than two-thirds of the cranial dura. This study aimed to evaluate the topographic features and branches of the middle meningeal artery (MMA) by three-dimensional rotational angiography (3DRA) and describe its variations' radiological classification based on previous clinical examples of cadaver studies and literature.
**Methods or Backround:** We consecutively evaluated 87 cerebral hemispheres, including 32 females and 49 right sides, between May 2020 and December 2021, who had undergone unilateral or bilateral cerebral and carotid artery 3D rotational angiography imaging by CCA injection and between 18 and–76-years-old. Maximum intensity projection images with 10–30 mm slice thickness were used for the morphological evaluations and measurements of MMA and its foramen, canal, and branches.
**Results or Findings:** The diameters of the common carotid artery, internal carotid artery, and external carotid artery were significantly smaller in females than in men (p= 0.021, 0.021, and <0.001, respectively). According to the branching pattern, the most common pattern of the MMA was Type Ia (49,4%). The ophthalmic artery completely originated from MMA in the 1 (1.1%) cerebral hemisphere. The MMA arises from the ophthalmic artery in 2 cerebral hemispheres (2.3%) and the ophthalmic artery and internal maxillary artery in 2 cerebral hemispheres (2.3%).
**Conclusion:** It was observed that the branching pattern may show differences when compared to the cadaver studies with a radiologic evaluation with 3D-RA.
**Limitations:** The different rates of variation among different populations may be due to ethnicity, and we think this has an impact on the findings of our research.
**Ethics committee approval:** The study was approved by the Bezmialem Vakif University Local Ethics Committee (File Number: (23.08.2021/29088).
**Funding for this study:** No funding was received for this study.
**Author Disclosures:**
Ibrahim Ilker Ilker Öz: Nothing to disclose
Lütfullah Sarı: Nothing to disclose
Temel Fatih Yılmaz: Nothing to disclose

### RPS 1515-4

**Carotid plaques measured by carotid doppler ultrasonography are stronger and more accurate prognostic parameters than carotid intima-media thickness**
*D. J. Petrovic*; Belgrade/RS
*(dusanpetrovic736@gmail.com)*

**Purpose:** The objective of the survey was to evaluate the independence of carotid plaque thickness and PF CC-IMT mean in cardiovascular risk prediction and risk stratification.
**Methods or Backround:** 188 respondents between the age of 46 and 87 were divided in two groups (I group - 94 respondents without plaques with CIMT assessment and II group 94 respondents with carotid plaques; 124 men and 64 women; mean age ± SD, 63.8 ± 4) were prospectively examined by the carotid ultra-sound Doppler (carotid measurements included plaque thickness PT - non-stenotic plaques (carotid stenosis <50%), and stenotic culprit plaques (carotid stenosis ≥50%), mean CIMT and maximum CIMT). Subjects were followed for a period of 36 months from the inclusion in the study (regular control examinations). Data were recorded on new cases of mortality (CV mortality) and adverse CV events (myocardial infarction - MI, surgical or endovascular revascularization - coronary or stroke).
**Results or Findings:** In this study, CIMT values varied between 0.62 and 1.43mm (mean CIMT=1.21 ± 0.2) while 52 patients had non-stenotic (14 respondents plaque ulceration, 22 type 2 diabetes mellitus, 38 arterial hypertension) and 38 patients had stenotic culprit plaques (17 respondents plaque ulceration, 20 type 2 diabetes mellitus, 31 arterial hypertension). After 36 months of follow-up, 76 vascular events were noted (MI, TIA, stroke, and cardiovascular angioplasty or surgery) in this period.
**Conclusion:** Respondents with carotid plaques had higher cardiovascular events occurrence (p<0.01, high statistical difference). Carotid plaques as a parameter have higher predictive vascular event value importance than CIMT. Of note, stenotic plaques, the presence of ulceration on the free surface of the plaque, type 2 diabetes mellitus and hypertension were connected with the highest events occurrence.
**Limitations:** Ultrasound was used as only a diagnostical method.
**Ethics committee approval:** This study was approved.
**Funding for this study:** No funding was received for this study.
**Author Disclosures:**
Dusan Jordan Petrovic: Nothing to disclose

### RPS 1515-5

**3D-Arterial Analysis Software and CEUS in the assessment of severity and vulnerability of carotid atherosclerotic plaque in comparison with CTA, MRA and histology**
D. Fresilli, N. Di Leo, G. T. Lucarelli, G. Del Gaudio, V. Dolcetti, *P. Pacini*, C. Catalano, V. D'Andrea, V. Cantisani; Rome/IT

**Purpose:** The study aimed to evaluate the accuracy of ultrasonographic 3D-Arterial Analysis and CEUS in assessing the severity and vulnerability of carotid plaques.
**Methods or Backround:** 48 carotid plaques were enrolled with the following criteria: (1) asymptomatic stenosis of carotid artery >60% but <100%; or (2) recent transient ischemic attack or ischemic stroke and ipsilateral carotid stenosis >50%. All the patients underwent endarterectomy, and gross and histology evaluation was performed to grade the plaque. 3D-Arterial Analysis provided a colour map to evaluate plaque vulnerability and a 3D volumetric stenosis evaluation. Its diagnostic performance has been compared to histological examination and MRA for plaque vulnerability and to CEUS and CTA for stenosis grading.
**Results or Findings:** 35 vulnerable plaques at histological examination were identified with at least one of the following criteria: fibrous cap < 200 µm, presence of lipid core, intra-plaque haemorrhage, leukocyte recruitment or angiogenesis. 3D-Arterial Analysis software, CEUS, MRA and CTA were able to detect 34, 32, 31 and 30 of these 35 vulnerable plaques, respectively, with 97%, 91%, 89% and 86% of sensitivity and 44%, 89%, 89% and 89% of specificity. CTA has identified 40 severe stenoses, of which 39 were correctly evaluated by 3D-Arterial Analysis software, 37 by CEUS and 36 by MRA with a sensitivity of 98%, 93% and 90%, respectively and specificity of 50%, 100%, and 100% respectively.
**Conclusion:** 3D-Arterial Analysis software and CEUS seem effective tools to assess plaque's vulnerability and stenosis severity, providing useful information for surgery planning. Multicenter prospective evaluation is warranted to clarify the role of US multiparametric evaluation.

**Limitations:** The study limitations were regarding patient selection (small population size, single centre site, only surgical candidates) and the inability to evaluate all plaques (highly calcified and very long plaques).
**Ethics committee approval:** No information provided by the submitter.
**Funding for this study:** No funding was received for this study.
**Author Disclosures:**
Daniele Fresilli: Nothing to disclose
Giovanni Del Gaudio: Nothing to disclose
Patrizia Pacini: Nothing to disclose
Vito D'Andrea: Nothing to disclose
Nicola Di Leo: Nothing to disclose
Giuseppe Tiziano Lucarelli: Nothing to disclose
Vincenzo Dolcetti: Nothing to disclose
Vito Cantisani: Nothing to disclose
Carlo Catalano: Nothing to disclose

### RPS 1515-6

**Evaluation of carotid bifurcation plaque vulnerability: comparing MRI and ultrasound-based modalities**

*F. Rea De Falco*, M. Krepuska; Zürich/CH
*(federica.readefalco@gmail.com)*

**Purpose:** Vulnerable plaques are important risk factors for the development of TIA and ischemic stroke. The vulnerability of carotid artery plaques can be evaluated using MRI. The value of plaque evaluation using ultrasound remains under study. We aimed to evaluate the congruency of ultrasound compared to MRI for the prediction of plaque vulnerability.
**Methods or Backround:** In a retrospective analysis, a cohort of stroke/TIA admitted to the University Hospital Zürich fulfilled the criteria of a combined special protocol of plaque visualisation utilising MRI/ultrasound and CTA of the carotid bifurcation due to suspected unstable plaques within the 24 hours after admission. The hallmark of carotid plaque characteristics, such as haemorrhage, ulceration, lipid-rich necrotic core, thin/ruptured fibrous cap, and degree of stenosis of 11 patients (6 male, 5 female, mean age 73 years) were categorised by two blinded investigators.
**Results or Findings:** Four plaques were classified as vulnerable on MRI, and five plaques were suspected to be unstable on ultrasound. One patient was considered uncertain. Six of patients' MRI and ultrasonographic techniques were in agreement and classified vulnerable.
**Conclusion:** Through a retrospective analysis using the multimedia approach in a regular stroke workup setting, we demonstrate that ultrasound of the carotid bifurcation may be a suitable tool to exclude plaque vulnerability ( true negatives).
**Limitations:** The small sample size was the major limiting factor, but due to time restraints of the blinded testers, it was important to begin data collection to assess the importance of Carotid Ultrasound in the exclusion of Plaque Instability.
**Ethics committee approval:** No information provided by the submitter.
**Funding for this study:** No funding was received for this study.
**Author Disclosures:**
Miklós Krepuska: Nothing to disclose
Federica Rea De Falco: Nothing to disclose

### RPS 1515-7

**Association between perivascular fat density and high-risk carotid plaque**

*M. S. Beşler*, M. B. Karadenizli, R. S. Ökten; Ankara/TR
*(msbesler@gmail.com)*

**Purpose:** The aim of our study was to investigate the relationship between perivascular fat density (PFD) measured in carotid computed tomography angiography (CTA) and vulnerable carotid plaque.
**Methods or Backround:** A total of 867 patients who underwent carotid CTA between June 1, 2021, and December 1, 2021, were analyzed retrospectively (234 male, median age [IQR:61-75]). History of acute ischemia or transient ischemic attack within six months before or after CTA acquisition of 374 patients included in the study was evaluated with diffusion-weighted MRI and anamnesis. PFD and plaque density were measured on CTA images.
**Results or Findings:** Cerebrovascular symptoms were observed in 135 of 374 adult patients. Maximum plaque thickness, stenosis ratio, ulceration and PFD were statistically significantly higher in the symptomatic patient group compared to the asymptomatic group, while plaque density was lower ($p<0.05$). PFD was statistically significantly correlated with plaque density and plaque thickness, respectively ($r = -0.188$, 0.215;$p<0.001$). PFD (OR:1.038, 95% CI:1.023-1.053) and plaque density (OR:0.985, 95% CI:0.972-0.999) were found to be independent predictors for symptomatic patients in the multivariable model. The optimal cut-off value for PFD was estimated as -74 HU for predicting symptomatic patients (AUC:0.753, 95% CI:0.699-0.808, $p<0.001$). Interobserver agreement was found to be almost perfect for PFD (ICC: 0.818[95% CI:0.770-0.856]) and plaque density measurements (ICC: 0.853[95% CI:0.807-0.887]).
**Conclusion:** To the best of our knowledge, the current study is the first in the literature to evaluate carotid plaque density and PFD together in the prediction of cerebrovascular ischemic events. In conclusion, PFD and plaque density measured on carotid CTA images could be evaluated as independent predictors for cerebrovascular ischemic events.
**Limitations:** Perivascular adipose tissue and plaque characteristics were not evaluated histopathologically. Prospective studies with follow-ups longer than six months can be done in the future.
**Ethics committee approval:** No information provided by the submitter.
**Funding for this study:** No funding was received for this study.
**Author Disclosures:**
Muhammed Said Beşler: Nothing to disclose
Rıza Sarper Ökten: Nothing to disclose
Mustafa Burak Karadenizli: Nothing to disclose

### RPS 1515-8

**Feasibility of using ultra-low-dose contrast medium: image quality evaluation of carotid artery CT angiography with dual-layer detector CT in virtual monoenergetic images (VMIs) at 40Kev**

*J. Chen*, S. Dong, X. Zhang, L. Fan, L. Shiyuan; Shanghai/CN
*(jieqiong_cjq@yeah.net)*

**Purpose:** The study aimed to investigate the benefit of virtual monoenergetic imaging (VMI) on dual-layer detector CT (DLCT) in contrast medium (CM) reduction for carotid artery CTA.
**Methods or Backround:** 64 patients who underwent carotid artery CTA from August 2022 to October 2022 on a DLCT (Spectral CT 7500, Philips Healthcare, Best, Netherlands) were randomly divided into ultra-low-dose CM group (n=32, 320mgI/ml Loversol, 0.43ml/kg) and routine dose CM group (n=32, 320mgI/ml Loversol, 1.09ml/kg). VMI 40keV of the ultra-low-dose group and conventional images of the routine dose group were reconstructed. The image quality of the two groups was objectively evaluated with CT attenuation and CNR of the carotid artery. Two independent radiologists determined subjective scores with a 5-point scale, analysing the contrast, the edge sharpness and diagnostic confidence of the carotid artery and its branches. Non-parametric tests were used to compare the objective results and subjective scores between the two groups.
**Results or Findings:** The CM dose in the ultra-low-dose group was reduced by 60% compared to that in the routine dose group. The VMI 40Kev had even better objective image quality than conventional images. CT attenuation of the carotid artery was higher in the VMI 40Kev (695.38±171.19 ) than in conventional images (330.01±71.83) ($P<0.05$). CNR numbers of the carotid artery were higher in the VMI 40Kev (65.17±68.88) than in conventional images (37.92±14.83) ($P<0.05$). Differences were found between the two types of images in subjective scores for all arteries ($p<0.05$). Especially for the display of parts of the carotid artery's branches, subjective scores were higher in the VMI 40Kev (2.77±1.08) than in conventional images (2.05±0.97) ($P<0.05$).
**Conclusion:** VMI 40Kev on DLCT allows the usage of only 40% contrast medium dose for carotid artery CTA with comparable image quality to routine scans.
**Limitations:** The study is limited by the lack of validation about the carotid artery disease diagnosis.
**Ethics committee approval:** No information provided by the submitter.
**Funding for this study:** No funding was received for this study.
**Author Disclosures:**
Liu Shiyuan: Nothing to disclose
Shuwen Dong: Nothing to disclose
Li Fan: Nothing to disclose
Xiaohui Zhang: Nothing to disclose
Jieqiong Chen: Nothing to disclose

### RPS 1515-9

**MPI/FLI/CTA multi-modality imaging of atherosclerotic plaque with plexind1-targeted nanoparticles**

*M. Ma*; Beijing/CN
*(ma_ming_rui@163.com)*

**Purpose:** M1 macrophage is one of important components which contribute to the progression of atherosclerotic plaques. Previous research of our team showed that plexinD1 was expressed more in atherosclerotic plaques at vascular bifurcation than those at straight site and co-localized with M1 macrophages. This study aims to develop a novel multimodal imaging platform by designing a nanoprobe targeting plexinD1 to detect and monitor bifurcation lesions and provide a detecting method for animal research.
**Methods or Backround:** A novel multimodal imaging agent, A12-Fe3O4-Cy7, was constructed by coupling superparamagnetic iron oxide nanoparticles（SPIONs) with cyanine 7 N-hydroxysuccin imide ester（Cy7）and nanobody A12 which could target plexinD1 specifically. The physical and biological properties were evaluated. To assess A12-Fe3O4-Cy7 recognition and monitoring for plaque, high-fat-fed 10-week and 20-week ApoE-/- atherosclerotic mouse models were imaged using MPI and FLI, and both 3D images were fused with CTA. Histological confirmation is performed.

Friday

**Results or Findings:** The A12-Fe3O4-Cy7 was of favourable stability, biocompatibility（concentration< 200ug/ml) and targeting effect. It could detect, locate, and monitor atherosclerotic plaques at the bifurcation of the carotid artery in ApoE-/- mice accurately and sensitively, showing significantly higher MPI and FLI signals both in vivo and ex vivo compared with both themselves before nanoprobe injection and two control groups(n=3,p<0.05). FLI is more sensitive than MPI to detect plaques of high-fat-fed 10-week ApoE-/- mouse. The above was further confirmed histologically.
**Conclusion:** A12-Fe3O4-Cy7 might serve as a method to detect bifurcation plaques by targeting plexind1 in vivo.
**Limitations:** MPI is tested only in mouse models presently, and it requires a combination with CT to provide anatomical information. Besides, there was no intervention on plexind1, so the mechanism of plexind1 in plaque occurrence and progression at the bifurcation is not illustrated clearly, which is worth exploring in the future.
**Ethics committee approval:** No information provided by the submitter.
**Funding for this study:** Funding provided by the National Natural Science Foundation of China under Grant 81827808.
**Author Disclosures:**
Mingrui Ma: Nothing to disclose

### RPS 1515-10

**Intracranial arteriosclerosis and dementia**

*T. C. van den Beukel*[1], U. Siebert[2], F. Wolters[1], W. Spiering[3], P. de Jong[3], D. Bos[1]; [1]Rotterdam/NL, [2]Boston, MA/US, [3]Utrecht/NL
*(tim@vdbeukel.net)*

**Purpose:** Increasing evidence suggests that vascular factors are important in the etiology of dementia, but the role of intracranial arteriosclerosis remains unclear.
**Methods or Backround:** From the population-based Rotterdam Study, 2200 non-demented, stroke-free persons (51% women, mean age 70 years) underwent CT scanning, on which the presence and severity (based on volume tertiles in persons with calcification) of intracranial carotid artery calcification (ICAC), ICAC subtype (atherosclerotic or internal elastic lamina [IEL]) and vertebrobasilar artery calcification (VBAC) were assessed. All participants were followed from the CT date to the date of incident dementia (n=213), death, loss-to-follow-up, or January 1st 2020. The association of calcification with dementia was calculated using Cox models adjusted for confounders.
**Results or Findings:** During a median follow-up time of 13.4 years, 261 cases of dementia occurred, of which 19 were preceded by stroke. The presence of ICAC (hazard ratio [HR]: 1.53 [95%CI 1.01-2.32]) increased dementia risk, with a similar impact for the atherosclerotic subtype specifically. For IEL ICAC, we found particularly severe calcifications increased dementia risk (HR: 2.08 [95%CI 1.17-3.71]). Also, we found that primarily severe VBAC (HR for third vs first tertile: 2.07 [95%CI 1.04-4.10]) increased dementia risk. Similar risks were observed for clinical Alzheimer's disease.
**Conclusion:** Intracranial arteriosclerosis increases dementia risk independently of stroke. The mechanisms underlying these associations, such as increased cerebral pulsatility or small vessel damage, probably differ according to the subtype of arteriosclerosis.
**Limitations:** Our study involved mostly older white participants free of dementia baseline, limiting generalizability to other ethnicities, and younger or more sick populations. Some of the effects observed might have occurred through stroke acting as a meditator on the pathway from arteriosclerosis to dementia, although this is unlikely due to the small number of strokes preceding dementia.
**Ethics committee approval:** No information provided by the submitter.
**Funding for this study:** No funding was received for this study.
**Author Disclosures:**
Wilko Spiering: Nothing to disclose
Pim de Jong: Nothing to disclose
Uwe Siebert: Nothing to disclose
Daniel Bos: Grant Recipient: Dr. Bos is supported by a grant (A2017424F) from the BrightFocus Foundation.
Tim Cornelis van den Beukel: Nothing to disclose
Frank Wolters: Nothing to disclose

### RPS 1515-11

**The significance of femoral plaque characteristics in restenosis after carotid artery stenting (CAS)**

*M. Philippovich*, B. B. Nyárády, Á. Szőnyi, D. T. Nguyen, E. Dósa; Budapest/HU

**Purpose:** Although the association between vulnerable lesions and cardiovascular events is well established, little is known about their relationship to in-stent restenosis (ISR) after CAS. To address this issue, we initiated a prospective, non-randomised study to examine femoral plaques in patients who will undergo CAS and who will be followed longitudinally for the development of early ISR.
**Methods or Backround:** The final analysis included 257 patients (149 men; median age: 70 [IQR, 62–76] years) who underwent CAS (2012–2017) for significant (≥70%) internal carotid artery (ICA) stenosis. A quantitative scale was used to classify the size of femoral plaques on ultrasound images: grade 1, small plaques (<20mm2); grade 2, moderate plaques (20–50mm2); and grade 3, large plaques (>50mm2). The morphology of femoral plaques was graded in terms of echogenicity as echolucent–predominantly echolucent, 1; echogenic–predominantly echogenic, 2; sclerotic, 3; or calcified, 4. The surface of femoral plaques was categorised as smooth, irregular, or ulcerated. For each patient, the morphology of the largest femoral plaque was determined. Patients had carotid duplex ultrasound scans at 6 weeks, 6, 12, and 24 months after CAS.
**Results or Findings:** During the 44.7 (IQR, 21.7–61.4) months of follow-up, 28 patients (10.9%) had an ICA ISR ≥50%. Neither the size of femoral plaques (grade 1, P=0.691; grade 2, P=0.451; grade 3, P=0.238) nor the surface characteristics of femoral plaques (smooth, P=0.177; irregular, P=0.318; ulcerated, P=0.946) were significantly different between patients with and without ICA ISR. Echolucent–predominantly echolucent (GSM: <25) femoral lesions were independent predictors of ICA ISR (adjusted OR, 4.71; 95% CI, 2.39–7.89; P<001).
**Conclusion:** Ultrasound assessment of the echogenicity of femoral plaques before CAS may help identify patients at higher risk of carotid restenosis.
**Limitations:** The study is limited by its small sample size.
**Ethics committee approval:** The ethical approval can be found under number 132/2012.
**Funding for this study:** No funding was received for this study.
**Author Disclosures:**
Ádám Szőnyi: Nothing to disclose
Balázs Bence Nyárády: Nothing to disclose
Edit Dósa: Nothing to disclose
Dat Tin Nguyen: Nothing to disclose
Márton Philippovich: Nothing to disclose

### RPS 1515-12

**Is there an association between loss of protective aorta-carotid impedance mismatch and migraine in middle-aged women with ischemic stroke?**

B. J. Te Kiefte, M. Ali, G. Holswilder, N. van der Weerd, K. Linstra, J. Westenberg, G. Terwindt, M. Wermer, *H. J. Lamb*; Leiden/NL
*(h.j.lamb@lumc.nl)*

**Purpose:** In healthy subjects, the carotid artery has higher stiffness than the aortic arch, resulting in a protective aorta-carotid mismatch. Age-related aortic stiffening is known to cause aorta-carotid impedance mismatch, allowing pulsatile energy into the microcirculation of the brain. The aim of this study was to investigate whether loss of protective aorta-carotid impedance mismatch is associated with the presence of migraine in middle-aged women with ischemic stroke.
**Methods or Backround:** A total of 26 women (42-58y; mean 51y) with ischemic stroke (13 with a history of migraine) were included in this study. Patients underwent 3T MRI examinations to assess pulse wave velocity (PWV) in the aortic arch and carotid artery. PWV was determined using high-temporal 2D phase contrast MRI. Aortic arch PWV (PWVaorta) was assessed in the aorta at the level of the pulmonary trunk, with a single acquisition plane transecting the ascending and proximal descending aorta. Carotid artery PWV (PWVcarotid) was assessed by two consecutive acquisitions perpendicular to the left carotid artery (proximal in the common carotid artery and just below the petrous part of the internal carotid artery). Loss of protective aorta-carotid impedance mismatch was defined as PWVcarotid/PWVaorta < 1,0 (PWVratio).
**Results or Findings:** Only nine subjects (35%) had a PWVratio < 1.0 (4 with a history of migraine). Mean PWVaorta was 7.1 in women without migraine versus 8.0 in women with migraine. Mean PWVcarotid was 7.7 versus 9.2, and mean PWVratio 1.1 versus 1.2, respectively.
**Conclusion:** We did not find a relationship between loss of protective aorta-carotid impedance mismatch and migraine in middle-aged women with ischemic stroke in this small population.
**Limitations:** This study only used a small population without the direct comparison with healthy controls.
**Ethics committee approval:** No information provided by the submitter.
**Funding for this study:** This study was part of the CREW-MIST trial.
**Author Disclosures:**
Marieke Wermer: Nothing to disclose
Gisela Terwindt: Nothing to disclose
Mariam Ali: Nothing to disclose
Ghislaine Holswilder: Nothing to disclose
Bastiaan J.C. Te Kiefte: Nothing to disclose
Jos Westenberg: Nothing to disclose
Nelleke van der Weerd: Nothing to disclose
Katie Linstra: Nothing to disclose
Hildo J. Lamb: Nothing to disclose

**16:00-17:30** **Research Stage 1**

### Research Presentation Session: Artificial Intelligence & Machine Learning & Imaging Informatics

### RPS 1605

### Impact of AI on mammography screening

**Moderator**
M. Dietzel; Erlangen/DE
**Author Disclosures:**
Matthias Dietzel: CEO: radiologie weiterbildung de (GbR) school of radiology; Founder: radiologie weiterbildung de (GbR) school of radiology

### RPS 1605-2

**Real-world breast cancer screening performance with digital breast tomosynthesis before and after implementation of an artificial intelligence detection system**

*K. J. Schilling*[1], J. Shisler[2], J. W. Hoffmeister[3]; [1]Boca Raton, FL/US, [2]Jupiter, FL/US, [3]Nashua, NH/US
*(kschilling@bocaradiology.com)*

**Purpose:** To compare radiologists' breast cancer screening performance before and after implementation of an artificial intelligence (AI) detection system with digital breast tomosynthesis (DBT).
**Methods or Backround:** A retrospective analysis of mammography medical outcomes audit data was conducted with nine dedicated breast radiologists reading DBT screening exams at four sites during two distinct time periods. Data was collected from March 1, 2018 to February 29, 2020, prior to installation of AI ("pre-AI"), and from March 1, 2020 to February 28, 2022, with concurrent use of a deep learning AI detection system (ProFound AI, iCAD, Nashua, NH) ("post-AI"). Co-primary endpoints were cancer detection rate (CDR) per 1000 screen exams and recall rate. Secondary endpoints included positive predictive value for cancer screening (ppv1), sensitivity, and specificity. Estimates of performance pre-AI and post-AI and differences were obtained pooled across radiologists using Fisher exact test of independence.
**Results or Findings:** Performance rates were compared for women screened pre-AI (n=54,440, 383 cancers) and post-AI (n=48,742, 399 cancers). CDR improved from 5.77 (95%CI:5.17,6.44) to 7.08 (95%CI:6.37,7.86), an increase of 1.31 (95%CI:0.33,2.29,p=0.0047). Recall rate decreased from 6.97% (95%CI:6.76,7.19) to 6.96% (95% CI:6.74,7.19), a difference of -0.01% (95%CI:-0.32,0.30,p=0.4696). Ppv1 improved from 8.28% (95%CI:7.44,9.197) to 10.17% (95%CI:9.20,11.24), an increase of 1.90% (95%CI:0.55,3.24,p=0.0031). Sensitivity increased from 81.98% (95%CI:77.81,85.52) to 86.47% (95%CI:82.74,89.49), a difference of 4.48% (95%CI:-0.62,9.59,p=0.0523). Specificity increased from 93.56% (95%CI:93.35,93.77) to 93.70% (95%CI:93.48,93.91), a difference of 0.14% (95%CI:-0.16,0.44,p=0.1854).
**Conclusion:** Interpretation of screening DBT exams by dedicated breast radiologists after implementation of AI resulted in an increase in CDR, ppv1, and sensitivity without a change in recall rate or specificity. There is improvement of cancer detection with use of an AI detection system in clinical practice.
**Limitations:** Retrospective, single institution, single vendor.
**Ethics committee approval:** No information provided by the submitter.
**Funding for this study:** No funding was received for this study.
**Author Disclosures:**
Jeffrey W Hoffmeister: Shareholder: iCAD, Inc. Employee: iCAD, Inc.
Kathy Jean Schilling: Advisory Board: iCAD
Julie Shisler: Consultant: iCAD, Inc.

### RPS 1605-3

**AI in mammography screening: retrospective results from two different CE-marked AI systems**

M. Larsen[1], C. Flåt Aglen[1], M. A. Martiniussen[2], M. B. Bergan[1], *S. Hofvind*[1]; [1]Oslo/NO, [2]Kalnes/NO
*(sshh@kreftregisteret.no)*

**Purpose:** To compare the number of cancers selected by two different AI systems when the threshold for flagging cases is equal to the consensus rate in a standard screening setting in BreastScreen Norway.
**Methods or Backround:** Digital mammograms performed between 2009 and 2018 at one breast centre in BreastScreen Norway were analysed with two different CE-marked AI systems (A and B). A total of 59,246 examinations had AI results from systems A and B, including 363 screen-detected and 94 interval cancers. Each examination had a continuous risk score from system A and system B. The threshold for flagging examinations was set to 10%, which corresponded to the consensus rate in the study sample (positive interpretation score by either or both radiologists).
**Results or Findings:** System A flagged 92% (n=335/363) of the screen-detected and 40% (n=38/94) of the interval cancers. System B flagged 87% (n=315/363) of the screen-detected and 40% (n=38/94) of the interval cancers. A total of 84% of the screen-detected cancers were flagged by both system A and system B, as were 28 interval cancers. Among the 28 screen-detected cancers not flagged with system A, 64% were selected by only one of the two radiologists, and among the 48 screen-detected cancers not flagged with B, 44% were selected by only one radiologist.
**Conclusion:** System A flagged a higher number of screen-detected cancers than system B. For interval cancers, both systems flagged the same number of cases. However, the systems did not flag the exact same cases. Cases from a larger cohort of screened women have to be analysed to see if similar results will be observed.
**Limitations:** Not applicable.
**Ethics committee approval:** No information provided by the submitter.
**Funding for this study:** Not applicable.
**Author Disclosures:**
Camilla Flåt Aglen: Nothing to disclose
Marie Burns Bergan: Nothing to disclose
Marit Almenning Martiniussen: Nothing to disclose
Solveig Hofvind: Nothing to disclose
Marthe Larsen: Nothing to disclose

### RPS 1605-4

**AI-decision support for double reading in breast cancer screening with digital mammography: a pseudo-prospective evaluation**

K. Hamm[1], *A-K. Brehl*[2], D. Hellingman[3], B. Vetter[1], T. Jordan[1], C. Entrup[4], M. Engelke[5], B. Schubotz[1]; [1]Chemnitz/DE, [2]Nijmegen/NL, [3]Forchheim/DE, [4]Koblenz/DE, [5]Hamburg/DE
*(anne-kathrin.brehl@screenpointmed.com)*

**Purpose:** To evaluate the added value of artificial intelligence (AI) as decision support in a region of the German breast cancer screening program with independent double reading and consensus.
**Methods or Backround:** Between January and September 2022, a cohort of 21.030 digital mammography screening exams (including 118 screen-detected cancers) was double read with AI support by 3 readers. The AI system (Transpara, ScreenPoint Medical) provides a risk category (low, intermediate, or elevated) for each exam, indicating the likelihood of malignancy. Readers consulted AI whenever they were uncertain whether to forward an exam to consensus. Moreover, AI was used as decision support during consensus. The screening performance was compared to the performance of a historic cohort before the implementation of AI, which included 22.656 screening exams (of those, 94 cancers) recorded between January and September 2021 and read by the same 3 readers. AI categories were retrospectively retrieved. The amount of cancers per risk category was calculated. Screening performance was evaluated in terms of CDR, recall rate (RR), consensus workload, and positive predictive value (PPV).
**Results or Findings:** Overall, 99.1% (117/118) of screen-detected cancers were classified by AI as being intermediate or elevated risk. Implementation of AI as decision support led to a 26.8% increase in CDR (no AI: 4.1/1000; with AI: 5.6/1000, p = .028), along with a slight increase in RR (no AI: 2.2%, with AI: 2.5%, p = .04) and PPV (no AI: 18.7%, with AI: 22.3%, p = .04).
**Conclusion:** The implementation of AI-based decision support in a double reading setting significantly improved the quality of breast cancer screening in terms of cancer detection.
**Limitations:** Prospective results are needed to conclude the effect of AI as decision-support in screening.
**Ethics committee approval:** No information provided by the submitter.
**Funding for this study:** No funding was received for this study.
**Author Disclosures:**
Birgit Schubotz: Nothing to disclose
Torsten Jordan: Nothing to disclose
Daan Hellingman: Employee: Siemens
Martin Engelke: Nothing to disclose
Klaus Hamm: Nothing to disclose
Christian Entrup: Nothing to disclose
Bert Vetter: Nothing to disclose
Anne-Kathrin Brehl: Employee: Screenpoint Medical

### RPS 1605-5

**Role of automation bias in artificial intelligence for mammography**

*T. M. Dratsch*[1], X. Chen[1], M. Rezazade Mehrizi[2], R. Klöckner[3], M. Puesken[1], B. Baeßler[4], D. Maintz[1], D. Pinto dos Santos[1]; [1]Cologne/DE, [2]Amsterdam/NL, [3]Lübeck/DE, [4]Würzburg/DE

**Purpose:** The goal of this study was to explore whether and to what degree automation bias can affect inexperienced and experienced radiologists when reading mammograms aided by a purported artificial intelligence system.

Friday

**Methods or Backround:** A prospective experiment was designed in which twenty-two radiologists were asked to read 50 mammograms and provide their BI-RADS assessments assisted by a purported AI system. The purported AI system was set up in such a way that occasionally wrong BI-RADS predictions of varying degree would be presented to the readers alongside fake heatmaps.
**Results or Findings:** Performance for both inexperienced and experienced radiologists was significantly worse for the cases in which the purported AI-based system suggested an incorrect BI-RADS category, $F(1, 20)=159.44$, $p<.001$, $\eta 2p=.889$. Time to read one mammogram significantly decreased over the course of the study, $F(4, 68)=27.80$, $p<.001$, $\eta 2p=.621$. Inexperienced radiologists were significantly more likely to follow the suggestions of the purported AI when it incorrectly suggested a higher BI-RADS category than the actual ground truth (errors of commission), $t(20)=2.28$, $p=.034$, $d=0.97$.
**Conclusion:** Overall, our results suggest that, opposed to the widespread view that the combination of AI and human readers will enhance overall diagnostic accuracy, automation bias and other effects of human-machine interaction need to be considered to ensure safe deployment and improved diagnostic performance when combining AI and radiologists.
**Limitations:** We did not investigate the performance of radiologists without the use of the purported AI-based system and we only focussed on mammograms that are categorised with a standardised system (BI-RADS).
**Ethics committee approval:** This study was approved by the Faculty of Medicine and University Hospital Cologne, University of Cologne.
**Funding for this study:** This study was funded by a grant from the German Ministry of Health (BMG) as part of the project EVA-KI (ZMVI1-2520DAT03B).
**Author Disclosures:**
Daniel Pinto dos Santos: Nothing to disclose
David Maintz: Nothing to disclose
Bettina Baeßler: Nothing to disclose
Michael Puesken: Nothing to disclose
Roman Klöckner: Nothing to disclose
Mohammad Rezazade Mehrizi: Nothing to disclose
Thomas Markus Dratsch: Nothing to disclose
Xue Chen: Nothing to disclose

### RPS 1605-6

**Comparing level of automation and effects on outcome metrics of three innovative ways to implement AI in breast cancer screening**

C. Oberije[1], C. F. de Vries[2], G. Lip[2], N. Sharma[3], J. James[4], E. Ambrozay[5], *J. Nash*[1], A. Ng[1], P. Kecskemethy[1]; [1]London/UK, [2]Aberdeen/UK, [3]Leeds/UK, [4]Nottingham/UK, [5]Budapest/HU
*(jonathan.nash@kheironmed.com)*

**Purpose:** To assess differential effects on outcomes of three independent second reader (IR) implementations.
**Methods or Backround:** Data from two retrospective breast screening studies (N=112,596) were used to simulate outcome metrics and workload savings for different IR implementations, where AI serves as Reader-2: IR1: in all cases; IR2: only when AI and Reader-1 opinions agree 'recall' or 'no recall'; IR3: when AI and Reader-1 opinions agree 'no recall' only; otherwise, the historical Reader-2 (R2) opinion is used. Across IR1-3, human-AI interaction is only involved at arbitration for IR1.
**Results or Findings:** The screening performances of all implementations were comparable to human double reading (DR) with recall rates of: 4.73%, 4.75%, and 4.86% versus 5.02% for DR, and cancer detection of: 7.68/1000, 7.68/1000, and 7.71/1000 versus 7.76/1000 for DR. The simulations yielded arbitration rates of 12.16%, 1.62%, and 2.74%, while R2 was required to read 0%, 12.16%, and 50.76% of screens, resulting in human workload savings of 30.0-44.7%, 44.1-43.9%, and 23.7-24.3%, assuming arbitration time is 1-4x longer than R1-2.
**Conclusion:** The mode of incorporation of AI becomes more conservative from IR1 to IR3, resulting in different outcome trade-offs. While IR1 yielded an increased arbitration rate, it offered the highest workload savings opportunity. IR2 and IR3 observed a decreased arbitration rate and lower but more stable workload savings. The simulated outcomes of IR2-3 are more likely to hold in real practice as there is no human-AI interaction. However, given AI's promising performance, IR1's human-AI interaction during arbitration may present an opportunity for increased cancer detection, which may be suppressed in IR2-3. While all implementations require training and monitoring to ensure performance, the varying modes offer possibilities to meet the needs of specific screening units.
**Limitations:** Workflows are simulated.
**Ethics committee approval:** No information provided by the submitter.
**Funding for this study:** Funding was received from Kheiron Medical Technologies.
**Author Disclosures:**
Cary Oberije: Employee: Kheiron Medical Technologies
Nisha Sharma: Nothing to disclose
Gerald Lip: Nothing to disclose
Annie Ng: Employee: Kheiron Medical Technologies
Eva Ambrozay: Consultant: Kheiron Medical Technologies
Clarisse Florence de Vries: Nothing to disclose
Peter Kecskemethy: CEO: Kheiron Medical Technologies
Jonathan Nash: Employee: Kheiron Medical Technologies
Jonathan James: Nothing to disclose

### RPS 1605-7

**Ensuring consistent performance in changing clinical deployments: automatic recalibration of AI for breast cancer screening using real-time monitoring and unsupervised prediction alignment**

*M. Bernhardt*[1], G. Khara[1], N. Sharma[2], J. James[3], E. Ambrozay[4], J. Yearsley[1], P. Kecskemethy[1], T. Rijken[1], B. Glocker[1]; [1]London/UK, [2]Leeds/UK, [3]Nottingham/UK, [4]Budapest/HU
*(mb121@ic.ac.uk)*

**Purpose:** To evaluate the effectiveness of a novel automatic recalibration method for AI breast cancer detection models using real-time monitoring and prediction alignment to preserve the expected test performance under changes in image acquisition.
**Methods or Backround:** AI models are sensitive to changes in image acquisition, such as hardware changes or software updates, leading to possible drifts in the AI's sensitivity/specificity performance. We developed and evaluated an automatic recalibration method, unsupervised prediction alignment (UPA), that requires no ground truth annotations and only limited amounts of example images from the shifted data distribution. For this evaluation, we trained a mammography-based breast cancer detection model on 49,137 images acquired primarily on Hologic devices. The operating point was pre-calibrated using a Hologic-only validation set to perform at an equal sensitivity/specificity level of 82%. Three scenarios were simulated: (1) deployment of AI at a new site with previously unseen devices; (2) device replacement at a site where AI is already deployed; (3) updates to the device software resulting in changes in image characteristics.
**Results or Findings:** In all scenarios, the classification performance in terms of AUC was consistently high (~0.90), but a substantial sensitivity/specificity drift (up to 50%) was observed before recalibration of the AI system optimised for Hologic. Applying UPA successfully recovered the expected sensitivity/specificity performance for new, previously unseen devices (IMS Giotto, GE, Siemens), requiring only 500-1,000 unannotated cases for automatic recalibration, corresponding to data collected within one week at typical screening sites.
**Conclusion:** Real-time monitoring with automatic recalibration of AI can be an effective safety net for clinical deployment, preserving the expected sensitivity/specificity performance across a variety of scenarios causing image acquisition shifts.
**Limitations:** Not representative of commercially available AI systems.
**Ethics committee approval:** No information provided by the submitter.
**Funding for this study:** Funding was received from Kheiron Medical Technologies.
**Author Disclosures:**
Nisha Sharma: Nothing to disclose
Galvin Khara: Employee: Kheiron Medical Technologies
Melanie Bernhardt: Employee: Kheiron Medical Technologies
Ben Glocker: Employee: Kheiron Medical Technologies
Eva Ambrozay: Consultant: Kheiron Medical Technologies
Peter Kecskemethy: CEO: Kheiron Medical Technologies
Tobias Rijken: Founder: Kheiron Medical Technologies
Joseph Yearsley: Employee: Kheiron Medical Technologies
Jonathan James: Nothing to disclose

### RPS 1605-8

**Workload reduction of digital breast tomosynthesis screening using artificial intelligence and synthetic mammography**

*V. Dahlblom*, M. Dustler, S. Zackrisson, A. Tingberg; Malmö/SE
*(victor.dahlblom@med.lu.se)*

**Purpose:** A synthetic mammogram (SM), equivalent to digital mammography (DM), can be generated from digital breast tomosynthesis (DBT). To achieve the higher sensitivity of DBT, a time-consuming reading is necessary. However, SM is faster to read and might be sufficient in many cases. This study investigates using artificial intelligence (AI) to stratify examinations for reading either SM or DBT to minimise workload and maximise accuracy.
**Methods or Backround:** This is a retrospective study based on double-read paired DM and one-view DBT from the Malmö Breast Tomosynthesis Screening Trial. DBT examinations were analysed with the cancer-detection AI system ScreenPoint Transpara 1.7, resulting in a score (1-10). For low-risk examinations (score 1-6), SM reading was simulated by assuming equality with

Friday

DM reading. For high-risk examinations (score 7-10), the DBT reading results were used.
**Results or Findings:** By reading the DBT of 22% (3267/14772) of the cases with highest risk, and SM for the rest, 124 cancers were detected. That is, 31% (29/95) more cancers than with DM double reading, and 92% (124/135) of the cancers detectable with full DM and DBT double reading.
**Conclusion:** In a DBT-based screening programme, AI could be used to select high-risk cases where reading of DBT is valuable, while SM is sufficient for low-risk cases. Substantially more cancers could be detected compared to DM only, with only a limited increase in reading workload. Prospective studies are necessary.
**Limitations:** SM results have been approximated with DM results. Interaction between readers and AI was not studied. Single-centre study with a single vendor. Single-view DBT was used.
**Ethics committee approval:** No information provided by the submitter.
**Funding for this study:** Funding was received from the Swedish Governmental Funding for Clinical Research and Allmänna Sjukhusets i Malmö Stiftelse för bekämpande av cancer.
**Author Disclosures:**
Anders Tingberg: Research/Grant Support: Siemens Healthineers
Victor Dahlblom: Nothing to disclose
Magnus Dustler: Speaker: Siemens Healthineers
Sophia Zackrisson: Speaker: Siemens Healthineers

### RPS 1605-9

**Artificial intelligence (AI) and mammographic extremely dense breasts in BreastScreen Norway: could AI-based screening be an alternative to screening with MRI?**

*H. W. Koch*[1], M. Larsen[2], H. Bartsch[3], S. Hofvind[2]; [1]Stavanger/NO, [2]Oslo/NO, [3]Bergen/NO

**Purpose:** About 1 in 10 women have mammographic extremely dense breasts. These women have 3-6 times higher risk of developing breast cancer than women with fatty breasts, and also have reduced sensitivity due to tumours being masked by dense tissue. Since March 2022, the European Society of Breast Imaging recommends screening women with mammographic extremely dense breasts with MRI every 2-4 years. However, high costs and limited availability of MRI make it necessary to consider other options. The aim of this study was to investigate the performance of an AI system in reading mammograms from women with mammographic extremely dense breasts, compared to independent double reading by radiologists.
**Methods or Backround:** In this retrospective study, the study sample included 67 screen-detected cancers, 38 interval cancers, and 2403 negative screening examinations among women with mammographic extremely dense breasts collected from BreastScreen Norway between 2010 and 2018. An AI system (Transpara v.1.7.0) scored the examinations from 1-10, based on the risk of malignancy. Mammographic density was quantified by VolparaDensity v.1.5.0. When calculating sensitivity for independent double reading, interval cancers were considered false negative interpretation results.
**Results or Findings:** All 67 screen-detected cancers and 50.0% (19/38) of the interval cancers had an AI score of 10. Among women with a negative screening outcome, 16.1% (386/2403) had an AI score of 10. By setting AI score 10 as the threshold for a true positive screening examination, sensitivity of the AI system reached 81.9% compared to 63.8% for independent double reading.
**Conclusion:** Our results indicate promising performance of the AI system for women with mammographic extremely dense breasts. The cost of an increased number of false positive cases selected for consensus needs to be further explored in prospective studies.
**Limitations:** Retrospective study. Cancer-enriched dataset.
**Ethics committee approval:** No information provided by the submitter.
**Funding for this study:** No funding was received for this study.
**Author Disclosures:**
Henrik Wethe Koch: Nothing to disclose
Hauke Bartsch: Nothing to disclose
Solveig Hofvind: Nothing to disclose
Marthe Larsen: Nothing to disclose

### RPS 1605-10

**Exploring the relationships between an AI algorithm and human readers in a national breast screening external quality assurance scheme**

A. Taib, J. James, I. Darker, *Y. Chen*; Nottingham/UK

**Purpose:** This study applied a national external quality assurance (EQA) scheme for breast cancer screening to a commercially available Artificial Intelligence (AI) algorithm to explore if there was concordance between incorrectly labelled mammograms by AI and human readers.
**Methods or Backround:** Three EQA test sets were assessed by the algorithm. Mammogram cases were examined at breast level and the highest score for a feature was used to determine if a breast was recalled or returned to screening by the AI algorithm according to a pre-determined threshold. Scores ranged from 0 (lowest level of suspicion) to 100 (highest level of suspicion). AI results were compared to readings by a set of humans also taking part in the EQA test sets. Sensitivity and specificity were calculated based on biopsy-proven pathological outcomes or normal follow-up at the next screening round.
**Results or Findings:** 1,868 humans took part in at least one of the EQA test sets and therefore were included in the study. The cases consisted of 265 normal, 9 benign, and 86 malignant breasts. There was no significant difference in the mean AUC for the AI algorithm and humans (0.940 vs 0.954, respectively, $z = -1.207$, $p = 0.228$). Fifty-six percent (n=5) of the false negative AI cases were answered correctly by most humans (error rate for case <50%). Sixty percent (n=9) of the false positive AI cases were answered correctly by most humans (error rate for case <50%).
**Conclusion:** The AUC was similar between humans and the algorithm. However, the questions answered incorrectly according to pathology differed between AI and humans. This merits further investigation.
**Limitations:** The interaction between AI and human readers was not assessed.
**Ethics committee approval:** No information provided by the submitter.
**Funding for this study:** Funding was received from Lunit, which had no level of involvement in the study.
**Author Disclosures:**
Adnan Taib: Nothing to disclose
Iain Darker: Nothing to disclose
Jonathan James: Nothing to disclose
Yan Chen: Nothing to disclose

### RPS 1605-11

**Algorithmic transparency and interpretability measures improve radiologists' performance in BI-RADS 4 classification**

*F. Jungmann*, S. Ziegelmayer, F. Lohöfer, S. Metz, C. Müller-Leisse, M. Engelmaier, M. Makowski, G. Kaissis, R. Braren; Munich/DE

**Purpose:** Objective: to evaluate the perception of different types of AI-based assistance and the interaction of radiologists with the algorithm's predictions and certainty measures.
**Methods or Backround:** In this retrospective observer study, four radiologists were asked to classify breast imaging-reporting and data system 4 (BI-RADS4) lesions (n=101 benign, n=99 malignant). The effects of different types of AI-based assistance (occlusion-based interpretability map, classification, and certainty) on the radiologists' performance (sensitivity, specificity, questionnaire) were measured. The influence of the Big Five personality traits was analysed using Pearson correlation.
**Results or Findings:** Diagnostic accuracy was significantly improved by AI-based assistance (increase of 2.8 % ± 2.3 %, 95 %-CI 1.5 % to 4.0 %, $p = 0.045$) and trust in the algorithm was generated primarily by the certainty of the prediction (100% of participants). Different human-AI interactions were observed, ranging from nearly no interaction to humanisation of the algorithm. High scores in neuroticism were correlated with higher persuasibility (Pearson's $r = 0.98$, $p = 0.02$), whilst higher consciousness and change of accuracy showed an inverse correlation (Pearson's $r = -0.96$, $p = 0.04$).
**Conclusion:** Trust in the algorithm's performance was mostly dependent on the certainty of the predictions in combination with a plausible heatmap. Human-AI interaction varied widely and was influenced by personality traits.
**Limitations:** Small cohort size, influence of different algorithmic performances not tested, order bias, demand characteristics bias.
**Ethics committee approval:** No information provided by the submitter.
**Funding for this study:** Not applicable.
**Author Disclosures:**
Rickmer Braren: Nothing to disclose
Christina Müller-Leisse: Nothing to disclose
Sebastian Ziegelmayer: Nothing to disclose
Friederike Jungmann: Nothing to disclose
Stephan Metz: Nothing to disclose
Marcus Makowski: Nothing to disclose
Georgios Kaissis: Nothing to disclose
Fabian Lohöfer: Nothing to disclose
Maximilian Engelmaier: Nothing to disclose

### RPS 1605-12

**Sample size requirements for reliable assessment of subgroup performance and generalisability of AI algorithms for BI-RADS density prediction**

G. Khara[1], *H. Trivedi*[2], R. Patel[1], A. Ng[1], C. Oberije[1], A. Heroux[1], J. Nash[1], B. Glocker[1], P. Kecskemethy[1]; [1]London/UK, [2]Atlanta, GA/US
*(hari.trivedi@emory.edu)*

**Purpose:** To evaluate the effect of sample size on real-world generalisation for an AI BI-RADS density model.
**Methods or Backround:** A BI-RADS density AI model was trained on 85,597 and tested on 20,602 studies, from a representative, ethnically, and demographically diverse dataset collected at a US breast cancer centre across multiple sites. Performance was assessed against historical reader BI-RADS

Friday opinions, to reflect real-world workflows in a traditional screening program, on a range of sample sizes (from 100 to 15k), including stratification on the extreme dense (type D) subgroup. The uncertainty in subsample performance is captured by the width of the 95% confidence intervals (CI).
**Results or Findings:** A:B:C:D BI-RADS density categories represent a 11:41:42:6 ratio in each dataset. The mean 4-category accuracy on 100, 1k, and 15k subsampled populations is 81.7%, 81.4%, and 81.4%, with increasingly tighter CI widths of 6.3%, 2.1%, and 0.4%, respectively. For the type D subgroup, we observe an accuracy of 79.8%, 79.7%, and 79.2%, with 4x larger CI widths of 20.2%, 8.7%, and 1.9%, respectively. Uncertainty reduced as larger sample sizes were used. Previous work considered 2% CI widths to be sufficient, which is demonstrated with a sample size of 1k on the overall sample versus 15k for the type D subgroup.
**Conclusion:** It is important to use representative, real-world, large-scale sample sizes when assessing AI for BI-RADS density classification to ensure results translate to real-world environments and populations. It is especially important for BI-RADS density classification where inter-rater variability is high and some density types are less represented.
**Limitations:** Results may not be representative of other AI systems.
**Ethics committee approval:** This study was approved by eIRB Study ID: STUDY00000673.
**Funding for this study:** Funding was received from Kheiron Medical Technologies.
**Author Disclosures:**
Cary Oberije: Employee: Kheiron Medical Technologies
Galvin Khara: Employee: Kheiron Medical Technologies
Ravi Patel: Employee: Kheiron Medical Technologies
Ben Glocker: Employee: Kheiron Medical Technologies
Hari Trivedi: Nothing to disclose
Peter Kecskemethy: CEO: Kheiron Medical Technologies
Adam Heroux: Employee: Kheiron Medical Technologies
Annie Ng: Employee: Kheiron Medical Technologies
Jonathan Nash: Employee: Kheiron Medical Technologies

**16:00-17:30** **Research Stage 2**

### Research Presentation Session: Head and Neck

### RPS 1608

### Salivary glands, orbit, head and neck

**Moderator**
K. Markiet; Gdansk/PL
*(karolina.markiet@gmail.com)*

### RPS 1608-2

**Diagnostic accuracy of multiparametric ultrasound in the differential diagnosis of parotid lesions compared with MRI examination**

*V. Dolcetti*, G. Del Gaudio, P. Pacini, C. Solito, C. Di Bella, C. Catalano, V. Cantisani; Rome/IT
*(vincenzodolcetti@gmail.com)*

**Purpose:** To evaluate the diagnostic accuracy of multiparametric ultrasound (B-mode, eco-colour Doppler, elastosonography and CEUS) in the characterisation of the parotid gland formations, compared to MRI, with particular interest in the perfusional study.
**Methods or Backround:** 126 patients with parotid lesions were enrolled prospectively. The ultrasound evaluation was carried out by integrating B-mode ultrasound with eco-colour Doppler (CDUS), elastosonography with strain ratio (SE) and CEUS. All patients underwent FNAC or histological examination, which was considered as the gold standard. In 60 patients who had performed pre-operative MRI, a further retrospective comparison was made with the results previously obtained from the ultrasound evaluation.
**Results or Findings:** CDUS, SE and CEUS in differentiating between benign and malignant neoplasms showed a sensitivity value of 73%, 77% and 86% respectively, and a specificity value of 78%, 80% and 97% respectively. CDUS, SE and CEUS showed diagnostic accuracy of 78%, 78% and 90% respectively. The agreement with MRI was excellent (k = 0.87), in particular to CEUS performed on benign lesions. Furthermore, MRI showed diagnostic accuracy similar to multiparametric ultrasound (91%). SE [Strain Ratio (SR)> 3] revealed poor diagnostic accuracy (78%) due to the low SR values that characterise non-Hodgkin's lymphomas. In the differential diagnosis between pleomorphic adenoma and Warthin tumour, the SE (SR cut-off: 2.5) instead showed high sensitivity (93%) and good accuracy (85%). These limits are partially overcome by MRI.
**Conclusion:** Multiparametric ultrasound can represent a valid diagnostic integration and could sometimes be considered as an alternative to MRI in the evaluation of parotid lesions.
**Limitations:** Multiparametric ultrasound shows overall good diagnostic accuracy in the differential diagnosis between benign lesions, but appears less accurate in distinguishing benign from malignant ones. Further studies on larger samples are needed to prove it.
**Ethics committee approval:** No information provided by the submitter.
**Funding for this study:** No funding was received for this study.
**Author Disclosures:**
Giovanni Del Gaudio: Nothing to disclose
Patrizia Pacini: Nothing to disclose
Chiara Di Bella: Nothing to disclose
Vincenzo Dolcetti: Nothing to disclose
Carmen Solito: Nothing to disclose
Vito Cantisani: Nothing to disclose
Carlo Catalano: Nothing to disclose

### RPS 1608-3

**Role of diffusion-tensor imaging in the evaluation of parotid masses**

*A. El-Badrawy*[1], D. K. Khedr[1], E. Abdel Salam[1], N. Elbadrawy[2]; [1]Mansoura/EG, [2]Dakahlia/EG
*(adelelbadrawy@hotmail.com)*

**Purpose:** This study aimed to determine the value of diffusion-tensor imaging (DTI) in evaluating parotid gland tumours.
**Methods or Backround:** This study was conducted on 42 patients (aged 8-76 years: mean 45 years) who presented with parotid gland swelling. They underwent diffusion-tensor imaging obtained using a single-shot echoplanar imaging sequence at 1.5T scanner. The fractional anisotropy (FA) and the mean diffusivity (MD) value of the salivary gland tumours was calculated and correlated with pathological findings.
**Results or Findings:** All cases are confirmed pathologically. Our study showed a significant difference (P=0.001) in MD and FA of both malignant and benign tumours. ADC values for malignant and benign tumours were 1.2+0.45×10-3mm2/s and 1.75+0.66×10-3mm2/s respectively. The FA values for malignant and benign tumours were 0.45+0.07 and 0.18+0.05, respectively.
**Conclusion:** Diffusion-tensor imaging is a non-invasive, very good and promising discriminator for the characterisation and differentiation of benign and malignant parotid gland tumours.
**Limitations:** The limitation of our study is a small number of cases.
**Ethics committee approval:** The study was approved by the institutional research ethics review committee (Mansoura University/Faculty of Medicine/ Egypt). IRB reference number is “R.22.10.1887”.
**Funding for this study:** This manuscript is made by the authors' work without receiving any funding.
**Author Disclosures:**
Eman Abdel Salam: Nothing to disclose
Doaa Khedr Khedr: Nothing to disclose
Nada Elbadrawy: Nothing to disclose
Adel El-Badrawy: Nothing to disclose

### RPS 1608-4

**The effect of HBO treatment on post-radiation salivary glands by MRI T2 mapping**

*K. Cankar*, J. Vidmar, I. Serša, Z. Finderle; Ljubljana/SI
*(ksenija.cankar@mf.uni-lj.si)*

**Purpose:** To evaluate the effect of hyperbaric oxygenation (HBO) therapy on the salivary gland tissue in patients with head and neck tumours after radiation therapy.
**Methods or Backround:** HBO effects were monitored by using the T2 mapping method on a 3T MRI clinical system. Twenty patients treated with radiation doses from 50 to 80. Gy were included as well as 20 gender- and age-matched controls. The initial measurements were performed before the first HBO therapy (40.2±20 months after radiation therapy), and the second after 20 daily HBO therapies at 2.5ATA (absolute atmosphere) in which patients breathed 100% oxygen for 90 minutes each day. Complementary to MRI, salivary flow, buffer capacity, and pH were also determined.
**Results or Findings:** The mean T2 values obtained from T2 maps on the radiation side decreased from 121±20 msec to 113±16msec at the end of HBO therapy (p=0.002), but no change was observed on the contralateral side. In addition, a negative Pearson correlation coefficient was observed between the mean T2 value in the parotid gland on the radiation side and unstimulated saliva pH (R=-0.647, p=0.004), as also stimulated salivary flow (R=-0.592, p=0.01).
**Conclusion:** T2 mapping of salivary glands can be used to reliably quantify their response to HBO therapy; hence it has the potential to be a complementary diagnostic tool in the assessment of salivary gland status in patients with hyposalivation.

**Limitations:** In clinical settings, scan time is rather short (e.g. ten minutes per scan) and the only option to increase resolution is to increase sensitivity. This can be achieved most efficiently by using optimised multichannel RF probes and higher field MRI magnets.
**Ethics committee approval:** The study was independently reviewed and approved by the Ethical Committee of the National Ministry of Health (Approval number 0120-659/2016/6).
**Funding for this study:** This work was supported by Grant No. P3-0019, Ministry of Higher Education, Science and Technology, Slovenia.
**Author Disclosures:**
Ksenija Cankar: Nothing to disclose
Jernej Vidmar: Nothing to disclose
Igor Serša: Nothing to disclose
Zare Finderle: Nothing to disclose

### RPS 1608-5

**Dynamic contrast-enhanced (DCE) 3Tesla MRI for orbital lesion characterisation**

*E. O'Shaughnessy*, C. Le Cossec, N. Mambour, A. Lecoeuvre, J. Savatovsky, M. Zmuda, L. Duron, A. Lecler; Paris/FR

**Purpose:** Characterising orbital lesions remains challenging clinically or with imaging. The goal of this study was to determine the diagnostic performance of dynamic contrast-enhanced MRI at 3Tesla when characterising various orbital lesions.
**Methods or Backround:** This IRB-approved prospective single-centre study enrolled participants presenting with an orbital lesion undergoing a 3Tesla MRI prior to surgery from December 2015 to May 2021. Morphological, diffusion-weighted and DCE MRI were assessed by two readers blinded to all data. An univariate analysis followed by a multivariable analysis were performed to distinguish benign from malignant lesions and orbital inflammations from lymphoma.
**Results or Findings:** 131 participants (66/131 (50%) women and 65/131 (50%) men, mean age 52 ± 17 years [19-88]) were enrolled. Univariate analysis showed higher Ktrans and Kep medians in malignant versus benign lesion (1.1 min-1 versus 0.65 min-1, p=0.03 and 2.1 min-1 versus 1.1 min-1, p=0.01, respectively) and in lymphoma versus orbital inflammation (1.2 min-1 versus 0.6 min-1, p=0.001 and 2.3 min-1 versus 1.1 min-1, p<0.001, respectively). The best performing multivariable model in distinguishing malignant versus benign lesions included parameters from dynamic contrast-enhanced (DCE), Apparent Diffusion Coefficient (ADC) and morphology, and reached an AUC of 0.81 [0.67-0.96], yielding a sensitivity of 0.82 [0.55-1] and a specificity of 0.81 [0.65-0.96]. This same model reached an AUC of 0.84 [0.65-0.1], yielding a sensitivity of 0.83 [0.5-1] and a specificity of 0.85 [0.62-1] in distinguishing lymphoma from orbital inflammation.
**Conclusion:** Dynamic contrast-enhanced MRI might be valuable when characterising orbital lesions, either alone or in combination with morphological and diffusion weighted imaging.
**Limitations:** This is a single-centre study including a relatively low number of patients. Orbital lesions remain rare, thus enrolling patients is challenging. Multicentric prospective studies would be valuable.
**Ethics committee approval:** Approved by an institutional research ethics board, and adherence to the tenets of the Declaration of Helsinki. Our study follows the Standards for Reporting of Diagnostic Accuracy Studies (STARD) guidelines.
**Funding for this study:** Public funding was received for this study.
**Author Disclosures:**
Loïc Duron: Nothing to disclose
Chloé Le Cossec: Nothing to disclose
Mathieu Zmuda: Nothing to disclose
Natasha Mambour: Nothing to disclose
Emma O'Shaughnessy: Nothing to disclose
Adrien Lecoeuvre: Nothing to disclose
Julien Savatovsky: Nothing to disclose
Augustin Lecler: Nothing to disclose

### RPS 1608-6

**Imaging features of epidermoid cysts located in the floor of the mouth: a narrative review of literature**

*N. Basla*, D. Sfondrini, M. F. Achilli, M. Catalano, F. Sanvito, S. Marelli, M. Benazzo, M. G. Sala, L. Preda; Pavia/IT

**Purpose:** To discuss, through a review of literature, the modality of presentation of epidermoid cysts in the head and neck district with particular emphasis on radiological imaging, especially MRI.
**Methods or Backround:** All the articles reporting epidermoid cysts located in the oral cavity were searched using PubMed/Medline. For each article, age and sex, onset time of symptoms, location and size, imaging features (US, CT, MRI) and surgical access were analysed.
**Results or Findings:** Our search yielded 35 articles including 39 lesions in 38 patients, mostly young adults, without significant gender prevalence. This is a rare and uncommon localisation. Regarding the first diagnostic approach, US and CT were performed mostly (15 and 14 cases respectively). MRI was performed in only 9 cases. Considering the following diagnostic steps, US and MRI were both performed in 16 cases and CT in 25 cases. The imaging features of the lesions were found to be quite overlapping in all patients and in all the diagnostic methods.
**Conclusion:** In the diagnosis of epidermoid cysts of the oral cavity, MRI should be performed as a second level imaging modality because it is crucial for lesion characterisation and for the relationship with muscular structures of the floor of the mouth. This latter aspect is essential for surgical planning. The radiological report should describe the relationship between the mass and mylohyoid and geniohyoid muscles as this is decisive for the choice of surgical approach. When MRI is not available or is contraindicated, CT can be useful.
**Limitations:** Not all the articles mentioned diagnostic imaging of investigation. Discarded were all the cases of epidermoid cysts not located in the head and neck region and those that weren't defined as epidermoid cysts by a histopathological diagnosis.
**Ethics committee approval:** No information provided by the submitter.
**Funding for this study:** The authors have no funding sources to disclose.
**Author Disclosures:**
Domenico Sfondrini: Nothing to disclose
Marina Francesca Achilli: Nothing to disclose
Michele Catalano: Nothing to disclose
Maria Gabriella Sala: Nothing to disclose
Nicoletta Basla: Nothing to disclose
Lorenzo Preda: Nothing to disclose
Marco Benazzo: Nothing to disclose
Francesco Sanvito: Nothing to disclose
Stefano Marelli: Nothing to disclose

### RPS 1608-7

**Perimarginal lymph nodes: topography and predictive role of computed tomography**

*E. Radin*, J. De Groodt, F. Degrassi, C. Marrocchio, L. Calderan, A. V. Marcuzzo, G. Tirelli, M. A. A. Cova; Trieste/IT
*(ERIK.RADIN2@studenti.units.it)*

**Purpose:** Perimarginal lymph nodes (PMNs) could represent an underrated site for metastatic disease in oral cavity squamous cell cancer. Our study aimed to identify an anatomical volume, in which detecting PMNs in preoperative imaging, determining whether their features on contrast-enhanced CT (CECT) images could be used to diagnose them as pathological.
**Methods or Backround:** We retrospectively analysed 50 consecutive patients diagnosed with oral cavity squamous cell carcinoma who had undergone preoperative CECT and surgical excision of the primary tumour and neck dissection, including level IB. According to anatomical-surgical landmarks, two different size volumes (V1 and V2) and the number of PMNs detected within each were established. Thus we compared the radiological and histopathological findings to verify the accuracy of counting and categorising them as pathological.
**Results or Findings:** The number of PMNs detected in V1 and removed by ENT surgeons is the same in 11 out of 41 cases (27%), while in V2 the number reaches 36% (18 out of 50). The difference between PMNs removed and those detected in V1 was statistically significant (p<0.001), while the comparison with V2 was not (p=0.487). CECT predicts PMNs metastasis with a sensitivity of 54.4% and a specificity of 65.6%. Cohen's kappa coefficient (κ) was 0.175 and the agreement between data reached a value of 62.22%.
**Conclusion:** The number of PMNs we identified in V2 is more similar to that of PMNs surgically removed in the same patient with no statistical differences in the series, and the reliability of this volume is statistically higher than the one represented by V1. Preoperative CECT has a low sensibility and specificity to determinate metastatic involvement of PMNs, thus reinforcing the indication of their prophylactic removal in oral cavity cancer.
**Limitations:** No limitations were identified.
**Ethics committee approval:** No information provided by the submitter.
**Funding for this study:** No funding was received for this study.
**Author Disclosures:**
Erik Radin: Nothing to disclose
Alberto Vito Marcuzzo: Nothing to disclose
Giancarlo Tirelli: Nothing to disclose
Cristina Marrocchio: Nothing to disclose
Jasmina De Groodt: Nothing to disclose
Loretta Calderan: Nothing to disclose
Maria Assunta A. Cova: Nothing to disclose
Ferruccio Degrassi: Nothing to disclose

**RPS 1608-8**

**To evaluate the role of various MR sequences in the evaluation of adult traumatic brachial plexus injuries**

*A. Kumar*, S. Gamanagatti, C. Sathya, R. Sharma, A. S. Bhalla, J. Kumar; New Dehli/IN
*(DRATINKUMAR@GMAIL.COM)*

**Purpose:** To evaluate the role of various MR sequences in the evaluation of adult traumatic brachial plexus injuries.
**Methods or Backround:** A prospective study was carried out over a 2-year period wherein MRI was done in 91 patients with clinical and electrophysiological evidence of traumatic brachial plexopathy. MR neurography was performed on a 1.5-T MR unit using conventional 2D STIR, 3D SPACE STIR, and DWN sequences. For each patient, MIP and MPR images were generated from the three-dimensional dataset of the SPACE STIR sequence acquired in the coronal plane. The diagnostic quality and abnormal MR findings obtained in the 3D SPACE STIR sequence were compared with conventional STIR sequence and diffusion-weighted neurography. Surgical findings were available for comparison in 49 patients.
**Results or Findings:** 3D SPACE STIR sequence was found to be better than DWN and conventional 2D STIR sequence for demonstrating the postganglionic nerve roots, with an excellent diagnostic quality in 98.24% nerve roots assessed, compared to 90.76% by DWN and 86.59% by STIR. 3D SPACE STIR and conventional coronal STIR sequence showed abnormality in 62% nerve roots assessed, followed by DWN in 57.8%. The sensitivity of SPACE STIR, coronal STIR, and DWN in picking up the abnormal postganglionic injury was 98%, 96%, and 87.7%, respectively. However, the specificity of all three sequences was 100% when compared with intraoperative findings.
**Conclusion:** 3D SPACE STIR sequence was comparable to conventional 2D STIR in picking up the abnormality of the postganglionic plexus, but can provide better anatomical demonstration of brachial plexus with the advantages of MIP and MPR images. DWN also has excellent correlation with the STIR sequences and can help increase the confidence level.
**Limitations:** Operative correlation was available in only about half of the patients.
**Ethics committee approval:** This study was approved by the Institutional ethics committee.
**Funding for this study:** No funding was received for this study.
**Author Disclosures:**
Shivanand Gamanagatti: Nothing to disclose
Ashu Seith Bhalla: Nothing to disclose
C. Sathya: Nothing to disclose
Atin Kumar: Nothing to disclose
Jyoti Kumar: Nothing to disclose
Raju Sharma: Nothing to disclose

**RPS 1608-9**

**Standardisation of dual-energy CT iodine uptake of head and neck: definition of reference values in a big data cohort**

*I. Yel*, A. Gökduman, M. Dimitrova, S. Mahmoudi, L. S. Alizadeh, T. J. Vogl, C. Booz; Frankfurt/DE

**Purpose:** Despite a considerable amount of literature on dual-source CT iodine uptake of the head and neck, the physiologic iodine uptake of organs in this region has not yet been defined. Therefore we aimed to establish reference values for the iodine uptake of healthy organs to facilitate clinical application in the future.
**Methods or Backround:** Consecutive venous DECT scans of head and neck were reviewed and unremarkable exams were included (n=617; mean age=55years; 214w/403m). 35 ROI measurements were performed in 16 anatomical regions. Iodine uptake was compared among different organs/tissues, and subgroup analysis was performed (male vs female/young vs middle-aged vs old/normal weight vs overweight vs obese).
**Results or Findings:** Overall mean iodine uptake values ranged between 0.5 and 9.4 mg/dl. Women showed higher iodine concentration in the cervical vessels and for the parotid gland, masseter muscle, submandibular glands, sublingual glands, palatine tonsils, tongue body, thyroid gland, and the sternocleidomastoid muscle than men ($p \le 0.04$). With increasing age, intravascular iodine concentrations increased as well as the iodine uptake for the cerebellum and the thyroid gland, while values for the tongue and palatine tonsils were lower compared to younger subjects ($p \le 0.03$). Iodine concentrations for the parotid glands and the sternocleidomastoid muscles decreased with higher BMI ($p \le 0.004$), while normal weight patients showed higher iodine values inside the jugular veins, the other cervical glands, and tonsils compared to patients with higher BMI ($p \le 0.04$).
**Conclusion:** Physiologic iodine uptake values of cervical organs and tissues show gender-, age-, and BMI-related differences, which should be considered in the clinical routine of head and neck DECT.
**Limitations:** Due to biodynamical and size changes with age, not every structure could be included. Scans and post-processing were performed using one manufacturer, restricting a generalisation of our results for other systems.
**Ethics committee approval:** Approved by local ethics committee
**Funding for this study:** No funding was received for this study.
**Author Disclosures:**
Christian Booz: Nothing to disclose
Ibrahim Yel: Speaker: Siemens Healthineers
Mirela Dimitrova: Nothing to disclose
Aynur Gökduman: Nothing to disclose
Thomas J. Vogl: Nothing to disclose
Scherwin Mahmoudi: Nothing to disclose
Leona Soraja Alizadeh: Nothing to disclose

**RPS 1608-10**

**Role of dual-energy CT in the evaluation of jaw tumours and cysts**

*D. Justine*, A. S. Bhalla, S. Manchanda, A. R. Choudhary, D. Mishra, A. R. Mriddha; New Delhi/IN

**Purpose:** Jaw lesions exhibit a wide range of pathologic features and aggressive potential, frequently with similar imaging characteristics. Although characteristic features exist based on radiographs, CT and MRI, many are challenging to differentiate without biopsy. We believe dual-energy CT (DECT), with its rapid imaging time and material decomposition algorithms, has significant potential to address this issue.
**Methods or Backround:** Contrast-enhanced DECT was performed in 57 patients with a known histopathological diagnosis. Iodine concentration (IC), water concentration (WC), and normalised iodine concentration (NIC) were the quantitative parameters obtained. We used a one-way ANOVA/Kruskal-Wallis H test as we compared >2 independent groups. If significant differences were found, a series of independent t-tests or Mann-Whitney U tests were done. Receiver operating characteristic (ROC) curve analysis was used to calculate the diagnostic performance.
**Results or Findings:** Ameloblastomas were the most common pathology (n = 20), and in non-ameloblastomas it was central giant cell granulomas (CGCGs), (n = 11), followed by odontogenic keratocysts (OKCs). CGCGs showed a higher IC, WC and NIC than ameloblastomas ($p<0.05$). An IC threshold of 31.35×100µg/cm3 had the maximum sensitivity (81.8%) and specificity (65%). Between ameloblastomas and OKCs, the former had a higher mean IC, WC and NIC ($p<0.001$). Also, ameloblastomas had higher IC and lower WC compared to other non-ameloblastomas. A quantitative differentiation of CGCGs (higher IC, WC, NIC), ameloblastomas (moderate IC, WC, NIC), OKCs (lower IC, WC, NIC), and other non-ameloblastomas (lower IC and higher WC) is thus conceivable.
**Conclusion:** DECT imaging offers a more precise and reliable way to differentiate between jaw tumours. It can serve as a single imaging modality for simultaneous quantitative and morphological evaluation, obviating the need for additional studies.
**Limitations:** Small sample size and heterogeneous lesions within the other non-ameloblastoma group.
**Ethics committee approval:** The study was approved by the Institute Ethics Committee for postgraduate research, AIIMS, New Delhi w.e.f. 26-8-2020 and the study was conducted prospectively from August 2020 to April 2022. Informed consent was obtained from all patients who were included in the study.
**Funding for this study:** No funding was received for this study.
**Author Disclosures:**
Asit Ranjan Mriddha: Nothing to disclose
Smita Manchanda: Nothing to disclose
Ajoy Roy Choudhary: Nothing to disclose
Ashu Seith Bhalla: Nothing to disclose
Deepak Justine: Nothing to disclose
Deepika Mishra: Nothing to disclose

**RPS 1608-11**

**Assessment of osseous microstructure in the temporomandibular joint using a photon-counting-detector CT system: a cadaveric study with comparison to micro CT and energy-integrating-detector CT**

*M. S. May*, M. Wiesmüller, M. Kopp, R. Heiß, M. Wetzl, C. Treutlein, M. Uder; Erlangen/DE
*(matthias.may@uk-erlangen.de)*

**Purpose:** Imaging the temporomandibular joints (TMJ) and their adjacent soft tissue structures essentially support the clinical diagnosis of temporomandibular joint disorders. Photon counting detectors (PCD) have the potential to improve spatial resolution in clinical imaging. The aim of this study was to evaluate this new technique in comparison with energy-integrating detectors (EID) with ultra-high-resolution collimation, the current in-vivo gold standard, and the ex-vivo gold standard micro CT (mCT).
**Methods or Backround:** Eight cadaveric models of the TMJ from four human donors were included in this prospective study. Each cadaveric model was examined on a PCD, EID, and mCT system and reconstructed with the maximum spatial resolution for each modality (PCD: 0.14x0.14x0.25mm; EID: 0.13x0.13x0.4mm; mCT: 0.04x0.04x0.04mm). The following four trabecular microstructure parameters were calculated by manual segmentation and standardised thresholding using dedicated software: bone volume fraction (BV/TV), mean trabecular thickness (Tb.Th), mean maximum trabecular thickness (Tb.Thmax), and mean trabecular separation (Tb.Sp). Subjective image quality (overall, artifacts, resolution) was assessed on a four-point Likert scale.
**Results or Findings:** All trabecular parameters in mCT were significantly better than PCD and EID (all p<0.01). Tb.Th and Tb.Thmax values acquired with PCD were significantly lower compared to EID (p=0.01 and p=0.04, respectively). PCD also delivered a higher BV/TV ratio and a lower Tb.Sp value compared to EID, but differences were non-significant (p=0.08 and p=0.05). Overall image quality was best in mCT (all p<0.05) and better for PCD than for EID (p=0.04). Image artefacts were comparable between all three techniques. Spatial resolution was rated significantly higher for PCD compared to EID (p≤0.02), and best for mCT (p<0.01).
**Conclusion:** CT-microstructure visualisation of the TMJ in PCD is closer to the ex-vivo gold-standard mCT compared to EID.
**Limitations:** PCD availability is limited so far.
**Ethics committee approval:** No information provided by the submitter.
**Funding for this study:** No funding was received for this study.
**Author Disclosures:**
Marco Wiesmüller: Speaker: Siemens Healthcare GmbH
Matthias Stefan May: Speaker: Siemens Healthcare GmbH
Markus Kopp: Speaker: Siemens Healthcare GmbH
Matthias Wetzl: Speaker: Siemens Healthcare GmbH
Michael Uder: Nothing to disclose
Rafael Heiß: Speaker: Siemens Healthcare GmbH
Christoph Treutlein: Speaker: Siemens Healthcare GmbH

### RPS 1608-12

**Incidental head and neck findings on positron emission tomography-computed tomography (PET-CT) in patients with cancers**

*A. El-Badrawy*, N. Elbadrawy; Mansoura/EG
*(adelelbadrawy@hotmail.com)*

**Purpose:** The purpose of this retrospective study was to assess the frequencies and clinical significance of incidental head and neck findings on PET/CT in patients with cancers.
**Methods or Backround:** A total of 534 patients with malignancy underwent PET/CT were evaluated. Foci with an abnormally increased FDG uptake and/or CT abnormalities unrelated to the patient's disease were recorded. The diagnosis of these lesions was confirmed histopathologically or on follow-up imaging.
**Results or Findings:** Incidental findings within the head and neck on PET-CT were detected on 145/534 (27%). These findings were normal variants, inflammation, benign lesions or malignant tumour. Inflammations underwent proper therapy. Benign lesions had recommendations for treatment or follow-up, according to their nature. Incidental synchronous primary malignant tumours were detected on 5/534 (0.93%). They were thyroid (3/534) and parotid carcinomas (2/534).
**Conclusion:** Incidental head and neck findings are likely on PET-CT of patients with head and neck cancer. Some of these findings are important and warrant further investigation to ensure adequate management.
**Limitations:** Limitations of our study include a retrospective of our analysis and a shortage of long-term follow-up.
**Ethics committee approval:** The study was approved by the institutional research ethics review committee (Mansoura University/Faculty of Medicine/Egypt). IRB reference number is R.22.05.1707.
**Funding for this study:** This manuscript is made by the authors' works without receiving any funding.
**Author Disclosures:**
Nada Elbadrawy: Nothing to disclose
Adel El-Badrawy: Nothing to disclose

**16:00-17:30 Research Stage 3**

### Research Presentation Session: Musculoskeletal

### RPS 1610

### Musculoskeletal: wrist/hand and foot/ankle

**Moderator**
R. Prada González; Vigo/ES
*(rachelprad@gmail.com)*

### RPS 1610-2

**MRI in cases of wrist joint pain**

*R. Rastogi*, A. K. Singh, V. Pratap; Moradabad/IN
*(rajulrst@yahoo.co.in)*

**Purpose:** The wrist joint is a commonly used joint in day-to-day activities and hence is not only susceptible to various pathologies but is also the cause of significant morbidity in cases of wrist pain. Although magnetic resonance imaging (MRI) is the usual imaging investigation preferred for evaluation of a painful wrist joint, its inherent limitations in the form of high cost, limited availability, higher scan time and lack of comparison with the contralateral side has prompted us to design a study comparing the role of HRUS with MRI in such cases.
**Methods or Backround:** Eighty patients with a painful wrist joint were evaluated using HRUS and MRI. The two imaging modalities were compared not only in detecting various findings related to a painful wrist joint, such as joint effusion, synovitis, synovial hypertrophy, tenosynovitis, rice bodies, bone erosions, etc., but also in diagnosing the final group of disease. Appropriate statistical tests were then used to analyse the results.
**Results or Findings:** Our study revealed that HRUS performs similarly to MRI in the detection of joint effusion, synovitis, synovial hypertrophy, tenosynovitis, rice bodies, etc., but provides very poor evidence in the detection of bone pathologies, especially marrow oedema or chronic fractures. In our study, HRUS was equivalent to MRI in the final diagnosis in 67.5% of cases, was inferior to MRI in 30% of cases and was superior to MRI in 2.5% of cases.
**Conclusion:** Since HRUS has a high accuracy in detecting pathologies in cases of wrist joint pain, it should be used as the first imaging modality. However, patients who receive an equivocal diagnosis or who require surgical planning need MRI of the wrist joint.
**Limitations:** The small sample size identified as a limitation to this study.
**Ethics committee approval:** No information provided by the submitter.
**Funding for this study:** No funding was received for this study.
**Author Disclosures:**
Rajul Rastogi: Nothing to disclose
Abhishek Kumar Singh: Nothing to disclose
Vijai Pratap: Nothing to disclose

### RPS 1610-3

**Clinical and radiological predictors of delayed union or non-union of scaphoid fractures**

*G. Folco*, C. B. Monti, D. Capra, A. Zagarella, M. Gallazzi, F. Sardanelli; Milan/IT

**Purpose:** The purpose of our study was to review the role of different clinical and radiological variables from X-ray images of the hand in patients with scaphoid fractures on potential delayed union or non-union.
**Methods or Backround:** We retrospectively reviewed all patients who underwent at least one X-ray examination of the hand at our institution for a scaphoid fracture, excluding those who were referred for surgical treatment. Patients' clinical variables such as age and sex, and radiological data such as fracture dislocation site on the scaphoid bone, articular involvement and side, were collected to then construct a multivariate model to predict delayed union or non-union of those fractures.
**Results or Findings:** Our final population was composed of 105 patients, 75 (71%) of whom were male with a median age of 39 years (interquartile range: 27–63 years). Overall, 26 (25%) patients developed a delayed union or non-union of their scaphoid fracture. At multiple regression, fracture displacement (p<0.001), and patients' age (p=0.046) and sex (p=0.038) yielded a significant impact on delayed union or non-union, with an overall R-squared of 0.479 for such predictive modelling. Site fracture on the scaphoid bone displayed a borderline significance of delayed union or non-union rates (p=0.079).
**Conclusion:** A higher age, male gender, fracture dislocation, or fracture sites other than the middle third of the scaphoid bone, were predictors of higher rates of delayed union or non-union of scaphoid fractures. Such data can be considered when planning clinical pathways for patients presenting with scaphoid fractures.

**Limitations:** Single-centre, retrospective study on a small patient cohort.
**Ethics committee approval:** Not applicable.
**Funding for this study:** No funding was received for this study.
**Author Disclosures:**
Mauro Gallazzi: Nothing to disclose
Francesco Sardanelli: Advisory Board: Bayer Research/Grant Support: Bracco
Caterina Beatrice Monti: Nothing to disclose
Gianluca Folco: Nothing to disclose
Andrea Zagarella: Nothing to disclose
Davide Capra: Nothing to disclose

### RPS 1610-4

**Reliability of radioulnar and carpal alignment measurements in the wrist between radiographs and 3D imaging**

*D. Ferreira Branco*, C. Bouvet, M. Hamard, J. Y. Beaulieu, P-A. A. Poletti, S. Boudabbous; Geneva/CH
*(david.ferreira.branco@gmail.com)*

**Purpose:** Conventional radiography is the first modality in examining the radiocarpal joint in the event of trauma or instability. This study sought to determine the reliability of cone beam computer tomography (CBCT) and scout view in evaluating carpal alignment compared with conventional radiographs in order to assess the influence of wrist kinematics on regular measurements.
**Methods or Backround:** We prospectively recruited 305 patients who successively underwent plain radiography and CBCT. 51 patients with prior acute unilateral wrist trauma were eligible for entry into the study (mean age 39 years). Three blinded readers performed measurements separately. Axial method and bony axial lines defined previously in the literature were applied, with three categories of measurements performed, consisting of distal radioulnar, radiocarpal and radio/carpometacarpal measurements. Intraclass correlation coefficients (ICCs) for paired t-test were calculated to assess inter and intraobserver agreements.
**Results or Findings:** Interobserver agreement was very high (>0.94) for all modalities. Intraobserver reliability between scout view and CBCT was almost perfect for all measurements. Intraobserver reliability between radiograph and scout view/CBCT was perfect for distal radioulnar measurements, substantial for radiocarpal and moderate for radio/carpometacarpal measurements.
**Conclusion:** In absence of strict position control between two imaging acquisitions, only distal radioulnar measurements were shown to be perfectly reliable when using these two imaging methods, which is suggestive of a minor relevance of ulnar/radial deviation. Excepting for some angles, radiocarpal and radiocarpometacarpal measurements were shown to differ according to wrist position, being thus more sensitive to flexion/extension of the wrist.
**Limitations:** Inclusion of traumatic wrists does not allow conclusions to be drawn about angle values. Differences in experience among observers did not enable us to ensure that reliability depended only on imaging modality. Manual reconstruction of CBCT imaging was thought likely to underestimate interobserver reliability.
**Ethics committee approval:** No information provided by the submitter.
**Funding for this study:** No funding was received for this study.
**Author Disclosures:**
Jean Yves Beaulieu: Nothing to disclose
Sana Boudabbous: Nothing to disclose
David Ferreira Branco: Nothing to disclose
Pierre-Alexandre Aloïs Poletti: Nothing to disclose
Cindy Bouvet: Nothing to disclose
Marion Hamard: Nothing to disclose

### RPS 1610-5

**The value of ultrasound and magnetic resonance imaging in the evaluation of carpal tunnel syndrome**

*T. T. Nguyen*, N. Q. Vo, N. T. Hoang; Hue/VN
*(ntthao@hueuni.edu.vn)*

**Purpose:** The aim of this study was to determine the US and MRI parameters in the diagnosis of idiopathic carpal tunnel syndrome (CTS).
**Methods or Backround:** This study comprised 39 wrists in 24 symptomatic CTS patients who underwent clinical, electrophysiological, US and MRI evaluations. The control group was made up of 10 wrists in six healthy volunteers, for comparison purposes. Diagnosis of CTS is based on clinical and nerve conduction studies conducted according to the American Association of Neuromuscular & Electrodiagnostic Medicine (AANEM) guidelines. Measurements of the median nerve included cross-sectional area (CSA) at three different levels: the distal radioulnar joint (CSA1), the inlet (pisiform, CSA2), and the outlet (hook of hamate, CSA3) of the carpal tunnel, the ratio CSA2 and CSA1 (R-CSA), the difference between CSA2 and CSA1 (Delta S), the flattening ratio of the median nerve at the inlet and the outlet. Data were analysed using the software package IBM SPSS Statistics for Windows version 20.0.
**Results or Findings:** Significant differences were found between CTS patients and controls for CSA of the median nerve on both US and MRI. A cut-off value of 9.5 mm2 on US and 10.9 mm2 on MRI for the CSA at the inlet of the carpal tunnel had the highest sensitivity and specificity. Using this cut-off, MRI and US have the sensitivity and specificity of 97.4% and 95.2%, 80%, and 97.4%, respectively. We suggest using median nerve CSA at the pisiform, 15.5mm2 on US and 15.9 mm2 on MRI as the criteria for severe CTS.
**Conclusion:** Median nerve CSA at the inlet of the carpal tunnel is the most valuable parameter in diagnosing CTS on US and MRI.
**Limitations:** Only a small number of participants was included.
**Ethics committee approval:** This study was approved by the ethics committee of the University of Medicine and Pharmacy, Hue University, Vietnam (No. H2020/159).
**Funding for this study:** This study was supported by Hue University through the Core Research Program (No. NCM. DHH.2020.09).
**Author Disclosures:**
Ngoc Thanh Hoang: Nothing to disclose
Thanh Thao Nguyen: Nothing to disclose
Nhu Quynh Vo: Nothing to disclose

### RPS 1610-6

**The importance of stress-MRI in complex pulley lesions of the fingers**

*F. Schellhammer*[1], A. Vantorre[2]; [1]Cologne/DE, [2]Frankfurt/DE

**Purpose:** Bowstringing in devastating pulley lesions can be easily seen on dynamic MRI. However, competence of nonruptured A3-pulley can hardly be estimated. Pain adjusted stress-MRI might be a tool in further research.
**Methods or Backround:** 16 high-level sports climbers with clinical signs of complex pulley lesion of the fingers received standard MRI in stretched finger position as well as an additional MRI in bend finger position fixing the stamp of a syringe between the tip of the injured finger and the thumb. The climbers were told not to cross the pain barrier. Sagittal T1 SE, T2 TSE and PD TSE fat sat sequences were obtained.
**Results or Findings:** Stress-MRI disclosed bowstringing in two cases with a liquid filled gap between the plantar plate of the PIJ and the flexor tendon of 4 and 4.2 mm respectively. In nonruptured A3-pulley this gap was 0.8 on average (range: 0 – 2 mm). Gaps were seen best on the PD-weighted images. In standard MRI one lesion of the A3-pulley was detected.
**Conclusion:** The competence of the elastic A3-pulley is of major importance for therapeutic decision making in complex pulley lesions. Stress-MRI is required to achieve better understanding of this pulley. In high-level sports climbers a gap between the plantar plate of the PIJ and the flexor tendon of up to 2 mm seems to be acceptable. A larger gap as well as bowstringing indicates a rupture of the A3-pulley.
**Limitations:** This study only examined a small number of very specific patients.
**Ethics committee approval:** No information provided by the submitter.
**Funding for this study:** We did not receive funding for the study presented.
**Author Disclosures:**
Frank Schellhammer: Nothing to disclose
Andreas Vantorre: Nothing to disclose

### RPS 1610-7

**Deep learning-based evaluation and quantification of real-time MRI for the assessment of active wrist motion in health and disease**

*L. M. Wilms*, K. L. Radke, S. Nebelung, D. B. B. Abrar, M. Frenken, A. Müller-Lutz, G. Antoch; Düsseldorf/DE

**Purpose:** This study aims to investigate the clinical transferability of a standardised real-time MRI technique with a downstream fully automatic deep learning-based clinical evaluation method during active radioulnar abduction in patients with scapholunate (SL) ligament injuries.
**Methods or Backround:** Using a technical framework based on real-time MRI and algorithm-based image post-processing, 20 bilateral wrists of ten partial (SLpartial) or completely (SLcomplete) SL-deficient patients were imaged during continuous active radioulnar abduction using a 1.5-T MRI scanner, a custom-made motion device, and at a temporal resolution of 95 ms/image. After CNN-based automatic segmentation of the scaphoid and lunate (300 images/wrist), SL gap widths were determined as a function of wrist position across the range of motion. The healthy wrist of each patient served as a reference standard. For statistical analysis, a paired Student's t-test was used.
**Results or Findings:** Across the entire range of motion, SL gap widths increased significantly as a function of SL injury (ΔSL gap widths: 1.81±0.49 mm [complete]; 0.78±0.38 mm [partial]; p=0.006), particularly at maximum radial abduction, and displayed distinctly different motion patterns and trajectories.
**Conclusion:** By means of optimised real-time MRI, standardised active radioulnar motion, and advanced post-processing, configurational changes of the proximal carpus under motion can be visualised and quantified. Merging real-time wrist MRI and the introduced technical framework thus provides a powerful diagnostic tool for dynamic quantification of wrist function.
**Limitations:** The FLASH sequence was only acquired in the form of single 2D images, thereby potentially missing more complex carpal instability patterns that involve rotational, translational, and deviational instability.

**Ethics committee approval:** The study was conducted according to the guidelines of the Declaration of Helsinki and approved by the Institutional Review Board (or Ethics Committee) of the Medical Faculty, University of Dusseldorf, Germany (protocol code study 2019-590 and date of approval: 28.01.2020).
**Funding for this study:** This research was funded by "Elterninitiative Kinderkrebsklinik e. V.", L.M.W., D.B.A., M.F. and S.N. were supported by the local Research Committee of the Medical Faulty of Heinrich-Heine-University Düsseldorf. S.N. was additionally supported by grants from the "Deutsche Forschungsgemeinschaft" (DFG) (NE 2136/3-1).
**Author Disclosures:**
Sven Nebelung: Nothing to disclose
Karl Ludger Radke: Nothing to disclose
Lena Marie Wilms: Nothing to disclose
Gerald Antoch: Nothing to disclose
Anja Müller-Lutz: Nothing to disclose
Miriam Frenken: Nothing to disclose
Daniel B. Benjamin Abrar: Nothing to disclose

### RPS 1610-8

**Deep learning MRI reconstruction for turbo spin echo hand and wrist imaging: a comparison of image quality, visualisation of anatomy, and detection of common pathologies with standard imaging**
J. Herrmann, S. Afat, S. Gassenmaier, H. Almansour, *S. Werner*; Tübingen/DE

**Purpose:** The purpose of this study was to evaluate a deep learning (DL)-reconstruction for turbo spin echo (TSE) sequences of the hand and wrist concerning image quality, visualisation of anatomy, diagnostic performance, and common pathologies.
**Methods or Backround:** 21 patients (mean age: 43±19 [19-85] years, 10 men) were prospectively enrolled in this study between October 2020 and June 2021. Besides the standard MRI examination including TSE sequences reconstructed with a GRAPPA reconstruction (TSE_S), prospectively undersampled TSE sequences reconstructed with a DL-reconstruction (TSE_DL) were acquired. Two experienced MSK-imaging radiologists qualitatively evaluated the images concerning image quality, noise, edge sharpness, artifacts, and diagnostic confidence, as well as the delineation of anatomical structures using a five-point Likert scale and assessed common pathologies. Wilcoxon signed rank test and kappa statistics were performed to compare the sequences.
**Results or Findings:** Overall image quality, artefacts, delineation of anatomical structures, and diagnostic confidence of TSE_DL were rated to be comparable to TSE_S ($p>0.05$). Additionally, TSE_DL showed decreased image noise (4.90, median 5, IQR 5–5) compared to TSE_S (4.52, median 5, IQR 4–5, $p<0.05$) and improved edge sharpness (TSE_DL: 4.10, median 4, IQR 3.5–5; TSE_S: 3.57, median 4, IQR 3–4; $p<0.05$). Inter- and intrareader agreement was substantial to almost perfect ($\kappa$ = 0.632-1.000) for the detection of common pathologies.
**Conclusion:** Compared to TSE_S, TSE_DL provided decreased noise and increased edge sharpness, equal image quality, delineation of anatomical structures, detection of pathologies, and diagnostic confidence. As TSE_DL reduces examination time by more than 60%, it may increase patient comfort and throughput.
**Limitations:** First, the small sample size and monocentric design, proved to be a limitation of this study. Second, the acquisition parameters for TSE_S and TSE_DL were slightly inhomogeneous between the 1.5 T and 3 T scanners. MRI scanners of a single vendor were used for this study.
**Ethics committee approval:** This study was approved by the ethics committee of the Eberhard Karls University of Tübingen.
**Funding for this study:** No funding was received for this study.
**Author Disclosures:**
Judith Herrmann: Nothing to disclose
Saif Afat: Nothing to disclose
Haidara Almansour: Nothing to disclose
Sebastian Gassenmaier: Nothing to disclose
Sebastian Werner: Nothing to disclose

### RPS 1610-9

**Ultrasound-guided thread release for decompression of the cubital tunnel: a post mortem study**
*S. Wirth*[1], P. Sorgo[2], G. Bodner[1], J. Streicher[2], S. A. Jengojan[1]; [1]Vienna/AT, [2]Krems an der Donau/AT

**Purpose:** Ultrasound-guided thread release (UGTR) is a promising new technique for the treatment of peripheral nerve compression syndromes. The purpose of this study is to demonstrate the suitability of UGTR for releasing the cubital tunnel without damaging adjacent structures (vessels, tendons, muscles, nerves).
**Methods or Backround:** In consideration of more efficient and minimally invasive treatments, ultrasound-guided procedures are increasingly becoming an alternative to open surgery. In UGTR, a cutting thread is looped around the Osbourne's ligament under ultrasound-guidance and the cubital tunnel is released using the thread as a saw. The interventions were performed on 9 softly embalmed anatomic specimens. Prior to the interventions, the threads were placed in hematoxylin-eosin stain to improve tracing of the thread track. The outcome, complications and limitations were assessed via subsequent postinterventional dissections by an anatomist.
**Results or Findings:** In all interventions the Osborne's ligament was completely transected without any damage to the surrounding nerves, blood vessels, tendons or muscles.
**Conclusion:** UGTR is shown to be a safe and effective alternative for the release of the cubital tunnel in anatomic specimens.
**Limitations:** This study was performed on cadaveric arms, therefore clinical results can potentially differ from our results.
**Ethics committee approval:** This study has been submitted to and approved by the Commission of Scientific Integrity and Ethics of the Karl Landsteiner University of Health Sciences.
**Funding for this study:** No funding was required.
**Author Disclosures:**
Gerd Bodner: Nothing to disclose
Johannes Streicher: Nothing to disclose
Sophia Wirth: Nothing to disclose
Philipp Sorgo: Nothing to disclose
Suren Armeni Jengojan: Nothing to disclose

### RPS 1610-10

**Correlation between elastography and MRI findings in calcaneal tendon lesions**
*A. R. Cavalcante*, A. A. P. Semblano, R. V. de Almeida, R. M. Rogerio, Y. S. Silveira, W. Cunha de Assis, Z. N. G. Costa; Belém/BR
*(alanrcavalcante@gmail.com)*

**Purpose:** Evaluation of the Achilles tendon (AT) by elastography, correlating with findings from ultrasound and magnetic resonance imaging (MRI), in the assessment of AT injuries.
**Methods or Backround:** Cross sectional study in a private service of the Amazon basin between 1st January and 31st December 2021. Patients were evaluated with elastography images obtained by the GE Logiq S8 ultrasound device, equipped with Shear-wave technology of the same device. Mode B, Power/Colour Doppler were also analysed. Then an MRI assessment was performed to determine correlation.
**Results or Findings:** Pathological process changes the physiological structure of normal tissue in several ways, including rigidity, density and elasticity of that structure, and such changes in consistency can be detected by elastography. Elastography findings demonstrate that AT is hard in about 93% of cases in healthy tendons, while areas of softening can be found in half of tendinopathy cases due to microlesions. This leads to a series of degenerative changes that soften and weaken the tendon, eventually leading to spontaneous intrasubstantial rupture. In cases of partial or complete AT injury, the finding is characteristic on ultrasound and MRI. Elastography however, can corroborate the findings or even be useful in cases of minor injuries or ruptures not visualised by common ultrasound in B-mode; and with accessibility and lower cost than magnetic resonance imaging.
**Conclusion:** Elastography is a simple, reproducible an low-cost technique when compared to other imaging methods such as magnetic resonance. It shows good accuracy, like MRI, for the diagnosis of small tendon injuries of the calcaneus. Elastography appears a promising potential method for early diagnosis of lesions, as well as for therapeutic guidance and as a follow-up tool.
**Limitations:** 83 patients with suspicion of AT lesions were assessed.
**Ethics committee approval:** No information provided by the submitter.
**Funding for this study:** Private funding was received for this study.
**Author Disclosures:**
Rafaela Veras de Almeida: Nothing to disclose
Yan Santos Silveira: Nothing to disclose
Willie Cunha de Assis: Nothing to disclose
Ricardo Mendes Rogerio: Nothing to disclose
Aluizio Augusto Pereira Semblano: Nothing to disclose
Alan Rodrigues Cavalcante: Nothing to disclose
Zózimo Neto Gomes Costa: Nothing to disclose

### RPS 1610-11

**The role of selective injury to the superior and inferior band of the anterior talofibular ligament on ankle stability: a high-resolution US study**
*R. Picasso*, F. Zaottini, F. Pistoia, L. Tovt, M. Pansecchi, G. Marcenaro, C. Martinoli; Genoa/IT
*(riccardo.picasso@gmail.com)*

**Purpose:** Recent studies detailed the anatomy of the anterior talofibular ligament (ATFL), which usually consists of an upper and a lower band. The present study aimed to evaluate the potential of high-resolution ultrasound (US) in identifying the anatomic configuration of the ATFL and assessing the impact of selective band injury on ankle stability.

**Methods or Backround:** The study population was recruited from patients occurring in the emergency department after an inversion ankle injury. Fifteen days after trauma, patients were evaluated with US and lateral ankle ligaments, including the superior and inferior ATFL bands, the calcaneofibular and the posterior talofibular, and were graduated from 0 (normal) to 3 (complete tear). Ankle stability was assessed: I) utilising clinical examination and the extrapolation of a US instability constant (IC)=(A-B)/A, where A is the distance between the fibular tip and the talus during ankle plantarflexion/ inversion and B during dorsiflexion/eversion; ii) through the Cumberland ankle instability tool. Linear regression and Mann-Whitney U-tests were used to investigate the correlation between ligament injury and ankle instability.
**Results or Findings:** 40 patients were recruited. In 32 cases US enable to recognise two ATFL bands, whereas, in the remaining eight cases, only one band was identified. Injury to the inferior band was demonstrated to correlate with ankle instability as assessed through the IC (p<.001), whereas no correlation was found between the degree of injury of the superior band and any measurement of ankle instability.
**Conclusion:** US enabled the characterisation of the anatomic configuration of the ATFL. Ankle sprain determining injury to the inferior band of the ATFL results in more severe ankle instability than injuries involving only the superior band of the ATFL.
**Limitations:** The study is limited by the low number of patients and the lack of arthroscopic confirmation of US findings.
**Ethics committee approval:** Not applicable.
**Funding for this study:** No funding was received for this study.
**Author Disclosures:**
Giovanni Marcenaro: Nothing to disclose
Federico Zaottini: Nothing to disclose
Carlo Martinoli: Nothing to disclose
Luca Tovt: Nothing to disclose
Michelle Pansecchi: Nothing to disclose
Riccardo Picasso: Nothing to disclose
Federico Pistoia: Nothing to disclose

### RPS 1610-12

**Accuracy of clinical diagnosis, imaging methods, and biopsy in ankle and foot tumours and pseudotumours**

*F. Ruiz Santiago*, B. Moraleda Cabrera; Granada/ES
*(ferusan12@gmail.com)*

**Purpose:** This work in progress presents the experience of our institution in managing tumours and pseudotumours of the ankle and foot to review the usefulness of different imaging methods in diagnosing ankle and foot tumours.
**Methods or Backround:** 57 patients with pathological or microbiological diagnosis by surgery and/or imaging-guided biopsy. Of these, four cases were infectious, or inflammatory processes, 53 were diagnosed as tumours or pseudotumours of the ankle and foot, 34 from the soft tissues, and 19 from the bones. Clinical diagnosis and imaging accuracy diagnosis were assessed, with up to two differential diagnoses, matching with the final pathological or surgical diagnosis. The biopsy sensitivity, specificity, and accuracy were calculated
**Results or Findings:** Global accuracy numbers were: 28.8 % clinical; 58.6 % US; 71.7 % MRI; 88.5% CT; and 34.3% radiography, for a global imaging accuracy of 73.6%. In soft tissue masses numbers were: 17.6% clinical; 59.3 % US; 63.3 % MRI; 71.4% CT, for a global imaging accuracy of 61.8%. In bone tumors numbers were: 26.3% clinical; 87.5% MRI; 94.7 % CT; 70.6% radiography, for a global imaging accuracy of 94.7%. A biopsy was performed in 38 cases, 20 ultrasounds, and 18 CT-guided, with 29 true positives, 4 false negatives, and 5 true negatives. Global accuracy was 88.9 % and 90% in CT and ultrasound-guided biopsies.
**Conclusion:** Imaging accuracy overwhelms clinical diagnosis and must be mandatory before any interventional procedure. The imaging-guided biopsy is an accurate complementary tool when doubts persist about the nature or malignancy of the tumour.
**Limitations:** This is a retrospective work but intended to reflect normalised daily clinical practice.
**Ethics committee approval:** This study complies with local ethics committee approval (TFG-EITM-2020)
**Funding for this study:** No funding was received for this study.
**Author Disclosures:**
Fernando Ruiz Santiago: Nothing to disclose
Beatriz Moraleda Cabrera: Nothing to disclose

### RPS 1610-13

**Diagnostic performance comparison of conventional radiography to magnetic resonance imaging for suspected osteomyelitis of the extremities: a multireader study**

*P. P. Gowda*[1], M. Guirguis[1], H. Archer[1], O. Ashikyan[1], P. Pezeshk[1], D. Hoang[2], Y. Xi[1], A. Chhabra[1]; [1]Dallas, TX/US, [2]Houston, TX/US
*(Prajwal.Gowda@UTSouthwestern.edu)*

**Purpose:** The purpose of this study was to determine whether MRI provides improved diagnostic accuracy compared to radiography for the diagnosis of extremity osteomyelitis (OM) with multireader analysis.
**Methods or Backround:** In this cross-sectional study, three musculoskeletal fellowship trained radiologists evaluated cases of suspected OM in two rounds - first using radiographs (XR), then again four weeks later with conventional MRI. Previously described radiologic signs for OM were recorded. Each reader recorded individual findings on both modalities and rendered a binary diagnosis along with certainty of final diagnosis on a confidence scale of 1-5. This was compared with the pathology-proven diagnosis of OM and no OM to determine diagnostic performance. Intraclass correlation (ICC) and Conger's Kappa were used for statistics.
**Results or Findings:** XR and MRIs of 213 pathology proven cases were included in this study, with 66 being negative for both OM and abscess, 49 diagnosed with both soft tissue abscess and OM, 30 positives for OM only, and 68 with soft tissue abscess. 139 were males (age = 51.1 +/- 14.7 years, mean +/-SD) and 74 females (age = 51.3 +/- 13.6 years, mean +/-SD) with bones of interest in the upper and lower extremities in 29 and 184 cases, respectively. MRI showed significantly higher sensitivity and NPV than XR (P<0.001 for both metrics). Conger's Kappa for OM diagnosis was 0.62 and 0.74 on XR and MRI, respectively. Reader confidence improved slightly from 4.54 to 4.57 when MRI was used.
**Conclusion:** MRI is a diagnostically more effective imaging modality than XR for finding extremity osteomyelitis with better interreader reliability.
**Limitations:** This study did not explore the utility of DWI in the diagnosis of OM, which may have a great deal of potential. Furthermore, we did not evaluate cases of other differential diagnoses.
**Ethics committee approval:** The IRB approved this study and waived patient consent.
**Funding for this study:** No funding was received for this study.
**Author Disclosures:**
Diana Hoang: Nothing to disclose
Avneesh Chhabra: Advisory Board: Image Biopsy Lab Inc. Other: Jaypee, Wolters, Siemens, Research/Grant Support: Image Biopsy Lab Inc. Consultant: ICON Medical and TREACE Medical Concepts Inc.
Parham Pezeshk: Consultant: Image Biopsy Lab Inc.
Oganes Ashikyan: Consultant: Image Biopsy Lab Inc.
Holden Archer: Nothing to disclose
Mina Guirguis: Nothing to disclose
Prajwal P Gowda: Nothing to disclose
Yin Xi: Nothing to disclose

**16:00-17:30** **Research Stage 4**

### Research Presentation Session: Cardiac

### RPS 1603

### Risk stratification using cardiovascular imaging

**Moderator**
M. Williams; Edinburgh/UK
**Author Disclosures:**
Michelle Claire Williams: Speaker: Canon Medical Systems, Siemens Healthineers and Novartis

### RPS 1603-2

**Cardiac phenotypes in arterial hypertension: a magnetic resonance study of the Hamburg City Health Cohort**

*J. Erley*, C. Jahnke, H. Chen, K. Muellerleile, E. Cavus, G. K. Lund, S. Blankenberg, G. Adam, E. Tahir; Hamburg/DE
*(j.erley@uke.de)*

**Purpose:** The aim of this study was to analyse gender-specific phenotypes of cardiac structure and function in subjects with AHT and controls using magnetic resonance imaging (CMR).
**Methods or Backround:** The Hamburg City Health Study (HCHS) is a population-based, prospective cohort study, conducted by the University Medical Center Hamburg. Individuals between 45 and 74 years of age underwent CMR (3T, Magnetom Skyra, Siemens). Subjects with AHT (blood pressure ≥ 140/90 mmHg or ≥ 1 antihypertensive medication) and controls were investigated. Participants with known cardiac disease were excluded. Generalised mixed linear models were conducted, including confounders (age, body mass index, origin, diabetes mellitus, smoking, hyperlipoproteinemia, atrial fibrillation, and medication).
**Results or Findings:** 1984 subjects were analysed (1381 (69.6%) with AHT and 603 controls). 48.6% of controls and 42.4% of subjects with AHT were female. Females showed a 2.4% [95% confidence interval: 1.7-3.1] higher LV ejection fraction (LVEF) and 4.3% [3.3- 5.2] higher RVEF (all p<0.001) than males. End-diastolic volume index (EDVi), end-systolic volume index (ESVi), and stroke volume index (SVi) were lower in females than in males (p<0.001). AHT resulted in a 1.1-1.6% [0.2-2.8] higher EF (p=0.013-0.017), higher SVi, and lower EDVi/ ESVi in males and females likewise. Late gadolinium enhancement (LGE) was less likely detected in females (odds ratio (OR): -0.9 [-1.4 to -0.5], p<0.001). AHT did not significantly increase the chances of LGE presence (OR: 0.4 [0.0 to 0.9], p=0.072).
**Conclusion:** Cardiac structural and functional parameters, assessed with CMR, were influenced by gender and AHT in the HCHS-cohort after adjusting for confounders. However, the effect of gender was more pronounced.
**Limitations:** Patients were not grouped according to duration of AHT, no follow-up available, possible selection bias (more male subjects participated in study and agreed with contrast agent administration).
**Ethics committee approval:** This study was approved by the local ethics committee of the medical association in Hamburg.
**Funding for this study:** Not applicable.
**Author Disclosures:**
Stefan Blankenberg: Nothing to disclose
Charlotte Jahnke: Nothing to disclose
Ersin Cavus: Nothing to disclose
Gunnar K Lund: Nothing to disclose
Jennifer Erley: Nothing to disclose
Gerhard Adam: Nothing to disclose
Kai Muellerleile: Nothing to disclose
Hang Chen: Nothing to disclose
Enver Tahir: Nothing to disclose

### RPS 1603-3

**Impact of smoking as a risk factor in patients with suspected coronary artery disease in the DISCHARGE trial**

*A. J. Vázquez Mézquita*[1], M. Mancone[2], L. I. Birtolo[2], M. Dewey[1]; [1]Berlin/DE, [2]Rome/IT

**Purpose:** To investigate the impact of smoking in patients with suspected coronary artery undergoing coronary computed tomography (CT) or invasive coronary angiography (ICA).
**Methods or Backround:** The influence of smoking on the efficacy and safety of CT and ICA is unknown. This is a pre-specified subgroup analysis of a pragmatic, multicenter, randomized DISCHARGE study of CT vs. ICA which includes 3561 patients with suspected CAD conducted at 26 centers in 16 European countries. The primary endpoint was major adverse cardiovascular events (MACE: cardiovascular death, nonfatal myocardial infarction, or stroke). Key secondary endpoints were an expanded MACE composite (including transient ischemia attack or major procedure-related complication) and major procedure-related complications.
**Results or Findings:** No significant interaction between CT and ICA in regard to MACE was found (p=0.85). In the CT group, non-fatal stroke as a composite of MACE ( HR: 0.07 95% CI 0.01 – 0.55; p=0.033; p-interaction p=0.026) was less common in non-smokers compared with ICA at a median follow-up of 3.5 years. In current smokers, CT strategy is associated with fewer expanded MACE ( HR: 0.38, 95% CI 0.17 – 0.88, p=0.023; p-interaction p= 0.81).
**Conclusion:** MACE was similar between CT and ICA. A CT-first strategy is associated with fewer non-fatal strokes as composite of MACE in never-smokers and with fewer expanded MACE in current smokers.
**Limitations:** We could not systematically identify silent events. The smoking data were compiled based on the self-reporting system.
**Ethics committee approval:** The trial was approved by the local ethics committee at the coordinating center, by the German Federal Office for Radiation Protection, as well as other local and national ethics committees.
**Funding for this study:** This work was funded by the German Research Foundation through the graduate program BIOQIC (GRK2260, project-ID: 289347353), the priority program SPP-Radiomics (SPP2177, project-ID: 402688427), and the DISCHARGE project (603266-2, HEALTH-2012.2.4.-2) funded by the FP7 Program of the European Commission.
**Author Disclosures:**
Lucia Ilaria Birtolo: Nothing to disclose
Massimo Mancone: Nothing to disclose
Marc Dewey: Other: ESR Executive Council
Aldo Javier Vázquez Mézquita: Nothing to disclose

### RPS 1603-4

**Diagnostic and prognostic value of stress CMR imaging to evaluate chronic coronary syndromes: a twenty-year meta-analysis**

*C. Mantini*[1], G. Bisaccia[1], M. Y. Khanji[2], A. Cipriani[3], A. Fedorowski[4], S. Gallina[1], S. E. Petersen[2], C. Bucciarelli-Ducci[2], F. Ricci[1]; [1]Chieti/IT, [2]London/UK, [3]Padova/IT, [4]Stockholm/SE
*(cesare.mantini@gmail.com)*

**Purpose:** We aim to provide a contemporary quantitative data synthesis, by systematic review and meta-analysis of studies published over the last twenty years, on diagnostic accuracy and prognostic value of stress cardiovascular magnetic resonance (CMR) in patients with chronic coronary syndromes (CCS).
**Methods or Backround:** We measured pooled diagnostic indicators, including diagnostic odds ratio (DOR), sensitivity, specificity, area under the curve (AUC), and summary effect size indicators, including odds ratios (ORs) and cumulative annualised event rates (AERs) for all-cause death, cardiovascular death, and major adverse cardiac events (MACE).
**Results or Findings:** We identified 33 diagnostic studies pooling 7,815 individuals (mean age 62 years, 62% males) and 31 prognostic studies pooling 67,080 patients (mean age 62 years; 57% males; 381,357 person-years). Stress CMR yielded a pooled DOR of 26.4 (95%CI:10.6-65.9), a sensitivity of 81% (95%CI:68-89), a specificity of 86% (95%CI:75-93%), and an AUC of 0.84 (95%CI:0.77-0.89) for the detection of functionally obstructive CAD with fractional flow reserve as the reference test. 3T imaging yielded higher diagnostic accuracy achieving a DOR of 33.2. Presence of stress-inducible ischaemia was associated with higher all-cause mortality (OR:2.0;95%CI:1.7-2.3), CV mortality (OR:6.4;95%CI:4.5-9.1), and increased risk of MACE (OR: 5.3 95%CI:4.0-7.0). AERs for cardiovascular death and MACE were <1% in patients without stress-inducible ischaemia.
**Conclusion:** Stress CMR imaging yields optimum diagnostic accuracy and delivers robust prognostic information in patients with CCS and known or suspected CAD, particularly at 3T magnetic field. While presence of inducible ischaemia portends excess mortality and increased risk of MACE, a negative stress CMR heralds very low risk of future cardiovascular events, with a warranty period of at least 3.5 years.
**Limitations:** No limitations were identified.
**Ethics committee approval:** No information provided by the submitter.
**Funding for this study:** No funding was received for this study.
**Author Disclosures:**
Mohammed Y Khanji: Nothing to disclose
Artur Fedorowski: Nothing to disclose
Chiara Bucciarelli-Ducci: Nothing to disclose
Cesare Mantini: Nothing to disclose
Sabina Gallina: Nothing to disclose
Alberto Cipriani: Nothing to disclose
Steffen Erhard Petersen: Nothing to disclose
Giandomenico Bisaccia: Nothing to disclose
Fabrizio Ricci: Nothing to disclose

### RPS 1603-5

**Coronary computed tomography angiography scan-guided compared to angiography-guided planning for coronary artery bypass graft strategy: a single-centre preliminary analysis**

*E. Sfriso*, I. Franzese, D. Ruggiero, M. G. Belgrano, E. Mazzaro, M. A. A. Cova; Trieste/IT
*(enrico.sfriso95@gmail.com)*

**Purpose:** Invasive coronary angiography (ICA) is considered the gold standard for assessing coronary artery (ICA) disease. However, computed tomography angiography (CCTA) has arisen as a non-invasive tool capable of providing deeper information on CA anatomy and wall. We hypothesised that CCTA would improve the appropriateness and selection of vessels in coronary artery bypass graft (CABG) operations.
**Methods or Backround:** A single-centre blinded retrospective pilot study was performed, using a case-crossover design. Between 2014 and 2022, 15 patients with a preoperative high quality CCTA who underwent a standard ICA-guided CABG were enrolled. CCTAs were performed with PHILIPSiCT256 at the same Institution. Each case was retrospectively evaluated by a blinded cardiac surgeon to plan CABG, first ICA-guided and after CCTA-guided. A radiologist was always present to assess CCTA and to perform multiplanar and 3D reconstruction using the software Aquarius-iNtuition(TeraRecon).
**Results or Findings:** All patients received left anterior descending artery(LAD) graft. Concordance between ICA-planned number and operation-reported number of graft was 40%; for the CCTA it was 33.3%. Concordance between ICA and CCTA was 73.3%. The Bland-Altman plot showed good agreement between intraoperative diameter measures and values predicted using CCTA, especially for LAD (limits of agreement: ICA, -0.45 and 0.28 versus CCTA, -0.39 and 0.22). The Lin's Concordance Correlation Coefficient also confirmed similar value for LAD diameter between intraoperative measurement and predicted ICA value (0.63 [IC95%, 0.20-0.85]) or CCTA one (0.62[IC95%, 0.19-

Friday

0.85]). Surgeons also suggested that CCTA better identified depth of myocardial bridge.
**Conclusion:** The preliminary results and suggestions by the “New Heart-Team” agreed that the CCTA provides an effective CABG planning tool more consistent with real coronary anatomy. Thus, CCTA is likely to become a routine tool for optimal CABG planning if further studies confirm preliminary results.
**Limitations:** Not applicable.
**Ethics committee approval:** No information provided by the submitter.
**Funding for this study:** Not applicable.
**Author Disclosures:**
Enrico Sfriso: Nothing to disclose
Manuel Gianvalerio Belgrano: Nothing to disclose
Ilaria Franzese: Nothing to disclose
Enzo Mazzaro: Nothing to disclose
Maria Assunta A. Cova: Nothing to disclose
Danilo Ruggiero: Nothing to disclose

### RPS 1603-6

**CT coronary angiography in follow-up of children with Kawasaki disease having coronary artery abnormalities: a new promising paradigm**

*M. Singhal*, R. Pilania, A. Thangaraj, S. Singh; Chandigarh/IN

**Purpose:** Aim of the study was to evaluate the role of follow-up computed tomography coronary angiography (CTCA) in children with Kawasaki Disease (KD) having coronary artery abnormalities (CCAs) at a tertiary care centre in Chandigarh, India.
**Methods or Backround:** CTCA was carried out on 128-slice, dual-source CT scanner Definition Flash (Siemens, Erlangen, Germany). Follow-up CTCA was performed in 15/257 children.
**Results or Findings:** Median age at diagnosis: 48 months [range 4-96 months]. Median interval between the first and second CTCA examination: 37 months [range 6-85 months]. Findings of CTCA at presentation: 21 aneurysms and 11 dilatations: left main coronary artery (LCA)–5 aneurysm and 3 dilatation; left anterior descending artery (LAD)– 9 aneurysm, 1 dilatation; right coronary artery (RCA)–7 aneurysm and 1 dilatation and left circumflex artery (LCx) dilatation in 6 patients. Giant aneurysms: 6 patients (LAD – 5; RCA-3). Thrombosis and complex aneurysms: 3 patients each. Follow-up CTCA was normalised in 5/15 (33.3%) patients. Remaining 10 patients showed persistent (albeit regressed/remodelled) coronary artery aneurysms: LCA-5; LAD-8; RCA-7; LCx-2. Two patients showed mural calcifications. Stenosis: two patients revealed long segment stenosis in LAD with mural calcification, one developed increased stenosis up to 80% of LAD, and one patient had stenosis on follow up, which wasn’t noticed in the initial CTCA. One patient developed thrombus in fusiform aneurysm of LAD after 42 months. None of these abnormalities were detected on 2DE.
**Conclusion:** Follow-up CTCA in KD explicitly demonstrates complications like thrombosis, stenosis, and calcifications in comparison to 2DE. This is a new development and has potential to be the modality of choice for follow-up of children with CAAs of KD.
**Limitations:** Single institution study and needs validation from other centres.
**Ethics committee approval:** No information provided by the submitter.
**Funding for this study:** No funding was received for this study.
**Author Disclosures:**
Manphool Singhal: Nothing to disclose
Surjit Singh: Nothing to disclose
Abarna Thangaraj: Nothing to disclose
Rakesh Pilania: Nothing to disclose

### RPS 1603-7

**Use of radiological procedures in cohort of children with congenital heart disease by diagnosis**

*S. Afroz*[1], H. M. Olerud[1], U. S. Thevathas[1], B. H. Østerås[2], A. Nordsteien[1], G. Dohlen[2]; [1]Drammen/NO, [2]Oslo/NO
*(Susmita.Afroz@usn.no)*

**Purpose:** To count the frequency of radiological examinations for pediatric patients with congenital heart disease stratified by diagnosis to facilitate awareness of radiation dose burden.
**Methods or Backround:** 7954 x-ray, 1390 interventional radiology, and 235 CT examinations for 1031 congenital heart disease patients with 17 different main diagnoses following the recommendation of ICD-10 were collected from RIS. All patients were born between 2000 and 2020. Most common diagnoses are reported with the median, IQR, and maximum number of CT, interventional, and plain x-ray examinations.
**Results or Findings:** High number of examinations reported for anomalous pulmonary venous connection (PAPVR) with the median (IQR;Maximum): CT 2(0-5;5), interventional 2(2-5;9), and x-ray 11(10-101;104) and pulmonary atresia (PA): CT 1(0-2;5), interventional 1(1-3;5), and x-ray 16(12-30;57). Low number of examinations was reported for atrial septal defect (ASD): CT 0(0-0;4), interventional 0 (0-1;4), x-ray 0(0-3;110), patent ductus arteriosus (PDA): CT 0(0-0;3), interventional 1(0-1;5), x-ray 2(0-3;56), coarctation of the aorta (COA): CT 0(0-0;2), interventional 0(0-1;3), x-ray 0 (0-8;30) and ventricular septal defect (VSD): CT 0(0-0;5), interventional 1(1-1;4), x-ray 6 (3-10;54).
**Conclusion:** The results show that the diagnoses PAPVR and PA having a large number of examinations will cause more radiation burden to this young patient group than ASD, PDA, COA, or VSD. This finding is useful for clinicians to plan efficient disease management with a focus on radiation protection.
**Limitations:** Patients with a very high number of examinations are yet to be investigated. Multiple diagnoses were not taken into consideration.
**Ethics committee approval:** This study was approved by the Regional Committees for Medical and Health Research Ethics (REK).
**Funding for this study:** HARMONIC is funded by EU for five years (June 2019 – June 2024) under Grant agreement No 847707.
**Author Disclosures:**
Susmita Afroz: Author: The HARMONIC project (Health effects of cArdiac fluoRoscopy and MOderN radiotherapy in paediatriCs) has received funding from the Euratom research training programme 2014 – 2018 under grant agreement No 847707. This presentation reflects only the author’s view and the European Commission is not responsible for any use that may be made of the information content
Anita Nordsteien: Author: The HARMONIC project (Health effects of cArdiac fluoRoscopy and MOderN radiotherapy in paediatriCs) has received funding from the Euratom research training programme 2014 – 2018 under grant agreement No 847707. This presentation reflects only the author’s view and the European Commission is not responsible for any use that may be made of the information content
Hilde Merete Olerud: Author: The HARMONIC project (Health effects of cArdiac fluoRoscopy and MOderN radiotherapy in paediatriCs) has received funding from the Euratom research training programme 2014 – 2018 under grant agreement No 847707. This presentation reflects only the author’s view and the European Commission is not responsible for any use that may be made of the information content
Gaute Dohlen: Author: The HARMONIC project (Health effects of cArdiac fluoRoscopy and MOderN radiotherapy in paediatriCs) has received funding from the Euratom research training programme 2014 – 2018 under grant agreement No 847707. This presentation reflects only the author’s view and the European Commission is not responsible for any use that may be made of the information content
Bjørn Helge Østerås: Author: The HARMONIC project (Health effects of cArdiac fluoRoscopy and MOderN radiotherapy in paediatriCs) has received funding from the Euratom research training programme 2014 – 2018 under grant agreement No 847707. This presentation reflects only the author’s view and the European Commission is not responsible for any use that may be made of the information content
Utheya Salini Thevathas: Author: The HARMONIC project (Health effects of cArdiac fluoRoscopy and MOderN radiotherapy in paediatriCs) has received funding from the Euratom research training programme 2014 – 2018 under grant agreement No 847707. This presentation reflects only the author’s view and the European Commission is not responsible for any use that may be made of the information content

### RPS 1603-8

**4D flow and atrial imaging biomarkers in ischaemic cryptogenic stroke's pathogenesis: a preliminary analysis**

*M-D. Ippoliti*, C. Di Manna, S. Vicini, D. M. Bellini, M. Rengo, I. Carbone; Rome/IT

**Purpose:** To assess the presence of Left Atria Disfunction (LAD) in terms of impaired atrial wall contraction and intracavitary blood flow's turbulence by using the advanced Cardiac Magnetic Resonance (CMR) technique 4D flow in patients with cryptogenic stroke compared to control groups.
**Methods or Backround:** Prospective, multicentre study. Patients with cryptogenic stroke, cardioembolic stroke, lacunar stroke, and fibrillating were enrolled. Each patient underwent a CMR 4D flow study. Pressure gradient, mean and peak WSS, peak velocity, and flow were analysed with Arterys software (V19.03).
**Results or Findings:** Currently, 35 of 100 estimated patients were enrolled (15 with cryptogenic stroke, 9 fibrillating, 6 with lacunar stroke, 5 with cardioembolic stroke). The preliminary analysis was developed by performing a comparative and a ROC-curve analysis. Notwithstanding the absence of statistically-significant differences among groups, slightly higher values of pressure gradient, mean WSS, and peak velocity were found in patients with cryptogenic stroke (6,6 ± 4,7 vs 4,6 ± 3,2 mmHg for pressure gradient, 11,7 ± 1,0 vs 10,4 ± 1,2 cPa for mean WSS and 121,33 ± 46,20 vs 101,61 ± 43,2 cm/sec for peak velocity). Flow was basically lower in those patients (1,50 ± 0,86 vs 1,18 ± 0,91 l/min). Among the LAD parameters considered, peak velocity has the higher diagnostic accuracy for the identification of cryptogenic stroke patients (AUC=0,636).
**Conclusion:** The existence of specific imaging biomarkers, deriving from the left atrium blood flow’s analysis with CMR 4Dflow, could be predictive for the development of cryptogenic ischaemic stroke in the absence of other identifiable ischaemic pathology risk factors.

**Limitations:** The comparative analysis' statistical significance was strongly affected by the small sample size of each group. Hopefully, these limitations will be overcome by completing the analysis on the whole study sample.
**Ethics committee approval:** No information provided by the submitter.
**Funding for this study:** No funding was received for this study.
**Author Disclosures:**
Iacopo Carbone: Nothing to disclose
Simone Vicini: Nothing to disclose
Marco Rengo: Nothing to disclose
Davide Maria Bellini: Nothing to disclose
Caterina Di Manna: Nothing to disclose
Maria-Dea Ippoliti: Nothing to disclose

### RPS 1603-9

**Diabetic peripheral neuropathy is associated with aggravated subclinical LV dysfunction in patients with type 2 diabetes mellitus assessed using CMR feature tracking**
*X-M. Li*, W-F. Yan, J. Li, Z-G. Yang; Chengdu/CN

**Purpose:** This study aims to investigate the association between subclinical left ventricular (LV) myocardial dysfunction and diabetic peripheral neuropathy (DPN) in patients with T2DM and no history of cardiovascular events.
**Methods or Backround:** One hundred ninety-two patients with T2DM (131 patients without DPN and 61 patients with DPN) and 65 healthy individuals underwent cardiac magnetic resonance (CMR) examination with cine sequences. The LV global strain parameters, including radial, circumferential and longitudinal peak strain (PS), and peak systolic and diastolic strain rates (PSSR and PDSR, respectively), were evaluated using CMR feature tracking.
**Results or Findings:** LV longitudinal PS was reduced significantly in the following order: controls, patients with T2DM without DPN, and patients with DPN ($p < 0.001$). LV circumferential PS and radial PSSR were decreased in patients with T2DM presenting with DPN (p = 0.003 and 0.027, respectively) but were preserved in patients without DPN; LV radial PS, longitudinal PSSR, and PDSR in three directions were decreased significantly in both patient groups (all $p < 0.05$), and they were not significantly different between the patient groups (all $p > 0.05$). In multivariate linear regression analysis, DPN ($\beta$ = 0.990, p = 0. 035), retinopathy ($\beta$ = 1.389, p = 0.037), chronic kidney disease ($\beta$ = 1.529, p = 0.008), and HDL levels ($\beta$ = -1.549, p = 0.009) were independently associated with LV longitudinal PS.
**Conclusion:** DPN was associated with aggravated subclinical LV dysfunction in T2DM patients, suggesting the need to screen cardiovascular dysfunction aimed at implementing extensive interventions in this cohort of patients.
**Limitations:** A single-centre study with relatively limited sample size. Stress test was not performed, and subclinical coronary ischaemic disease could not be excluded.
**Ethics committee approval:** This study was approved by the Biomedical Research Ethics Committee of West China Hospital.
**Funding for this study:** Funding was received from the National Natural Science Foundation of China (81771897, 81771887 and 81471722).
**Author Disclosures:**
Jiang Li: Nothing to disclose
Wei-Feng Yan: Nothing to disclose
Xue-Ming Li: Nothing to disclose
Zhi-Gang Yang: Nothing to disclose

### RPS 1603-10

**Cardiac involvement in non-cirrhotic portal hypertension: MRI detects myocardial fibrosis and oedema similar to compensated cirrhosis**
*A. Isaak*, J. Chang, N. Mesropyan, D. Kravchenko, L. Bischoff, C. Strassburg, U. I. Attenberger, M. Praktiknjo, J. A. Luetkens; Bonn/DE
*(alexander.isaak@ukbonn.de)*

**Purpose:** The exact role of portal hypertension in cirrhotic cardiomyopathy remains unclear, and it is uncertain whether cardiac abnormalities also occur in noncirrhotic portal hypertension (NCPH). This MRI study aimed to evaluate the presence of subclinical myocardial dysfunction, oedema, and fibrosis in NCPH.
**Methods or Backround:** In this prospective single-centre study conducted from November 2018 to February 2022, participants underwent multiparametric abdominal and cardiac MRI, including cardiac function, myocardial oedema assessment, late gadolinium enhancement (LGE), and abdominal and cardiac mapping.
**Results or Findings:** A total of 111 participants were included (44 participants with NCPH [48±15 years; 23 women], 47 cirrhotic controls, 20 healthy controls). The cirrhosis group was dichotomised (Child A vs Child B/C). NCPH participants demonstrated a more hyperdynamic circulation compared to healthy controls (cardiac index: 3.7±0.6 L/min/m² vs 3.2±0.8 L/min/m², P=0.004; global longitudinal strain: 27.3±4.6% vs 24.6±3.5%, P=0.022). The extent of abnormalities indicating myocardial fibrosis and oedema in NCPH was comparable to Child A cirrhosis (e.g., LGE presence: 32% vs 33% vs 69%, P=0.004; combined T1 and T2 elevations: 46% vs 27% vs 69%, P=0.017; NCPH vs Child A vs Child B/C). Correlations between splenic T1 and myocardial T1 values were found (r=0.41; P=0.007). Splenic T1 values were associated with the presence of LGE (odds ratio, 1.010; 95%CI: 1.002, 1.019; P=0.013).
**Conclusion:** MRI parameters of myocardial hypercontractility, fibrosis, and oedema were observed in participants with NCPH and associated with splenic parameters, indicating a specific portal hypertensive cardiomyopathy.
**Limitations:** Sample size, no invasive reference standard.
**Ethics committee approval:** No information provided by the submitter.
**Funding for this study:** A.I. was supported by a university grant (BONFOR and Deutsche Forschungsgemeinschaft; EXC2151 – 390873048).
**Author Disclosures:**
Julian Alexander Luetkens: Nothing to disclose
Narine Mesropyan: Nothing to disclose
Christian Strassburg: Nothing to disclose
Alexander Isaak: Research/Grant Support: BONFOR and DFG (University grant)
Leon Bischoff: Nothing to disclose
Dmitrij Kravchenko: Nothing to disclose
Ulrike I. Attenberger: Nothing to disclose
Johannes Chang: Nothing to disclose
Michael Praktiknjo: Nothing to disclose

### Saturday, March 4

08:00-09:00 **Research Stage 1**

### Research Presentation Session: Artificial Intelligence & Machine Learning & Imaging Informatics

### RPS 1705

### AI-powered lung nodule detection

**Moderator**
B. S. Kelly; Dublin/IE
*(brendanskelly@me.com)*

### RPS 1705-2

**Screening malignant pulmonary nodules from chest CT images using muti-scale supervised contrastive learning**

X. Xu[1], Y. Wei[1], Z. Xue[1], J. Zheng[1], Y. Zhan[1], X. Zhou[1], Z. Ding[2], *F. Shi*[1], D. Shen[1]; [1]Shanghai/CN, [2]Hangzhou/CN
*(feng.shi@uii-ai.com)*

**Purpose:** To develop a deep learning model for automatic benign-malignant diagnosis of pulmonary nodules considering the complicated surrounding contextual environments on computed tomography (CT) images.
**Methods or Backround:** We propose a supervised contrastive learning framework to effectively incorporate the multi-scale 3D contextual information of nodules. First, nodules from coarse, medium, and fine scales of a 3D CT scan are employed to learn pairwise relationships. Moreover, to guarantee consistency, a supervised contrastive loss is introduced to encourage the information polling of multiple scales of the same class and punishing those from different classes, which effectively augments the classification performance.
**Results or Findings:** We employ a dataset with pathologically proven labels, containing a total of 1,226 nodules, where 556 are benign and 670 are malignant. Training and testing data are split on the patient level with a ratio of 8:2. The results of our experiments demonstrate that our model achieves 93.9% accuracy and 97.3% area under the receiver operating curve (AUC). In contrast, the ablation study shows the single scale model only achieves inferior performance with 90.6% accuracy and 95.7% AUC. Furthermore, the experiment shows that, when using only 25% of annotated nodules, our model can still obtain 89.4% accuracy, improving by 2.9% from the state-of-the-art contrastive learning model SimCLR.
**Conclusion:** Our study demonstrates that contextual environments from different scales provide useful information in classifying pulmonary nodules. Therefore, leveraging the relationship between different scales for prediction and clustering of the pulmonary nodules belonging to the same class can effectively improve the robustness and accuracy of a deep learning model. Experimental results suggest that our proposed framework can achieve plausible performance, especially with limited annotated samples.
**Limitations:** Further classifying malignant nodules into different pathological levels.
**Ethics committee approval:** The IRB of Affiliated Hangzhou First People's Hospital, Zhejiang University School of Medicine, China, approved this study.
**Funding for this study:** Funding was received from the National Science and Technology Innovation 2030-Major Project (2021ZD0111103).
**Author Disclosures:**
Yiqiang Zhan: Nothing to disclose
Feng Shi: Nothing to disclose
Ying Wei: Nothing to disclose
Dinggang Shen: Nothing to disclose
Zhong Xue: Nothing to disclose
Xiang Zhou: Nothing to disclose
Jie Zheng Jie Zheng: Nothing to disclose
Zhongxiang Ding: Nothing to disclose
Xiaoxian Xu: Nothing to disclose

### RPS 1705-3

**AI shows promise for future use as a first-read filter to accurately rule out benign lung nodules detected at baseline in CT lung cancer screening**

*H. L. Lancaster*[1], M. A. Heuvelmans[1], J. Yi[2], M. Oudkerk[1]; [1]Groningen/NL, [2]Seoul/KR
*(h.l.lancaster@umcg.nl)*

**Purpose:** Lung cancer screening (LCS) is on the brink of worldwide implementation with the ultimate goal of reducing lung cancer mortality. However, effectiveness could be hindered due to a lack of resources and mounting pressures as a result of increasing workload. Artificial intelligence (AI) could offer a solution if used a first-read filter to accurately stratify and rule out benign lung nodules.
**Methods or Backround:** Post CAD-speed and nodule-segmentation parameter updates, the AI-prototype (AVIEW_LCS_v1.1.39.14, CorelineSoft) performed an independent analysis of 283 baseline ultra-LDCT scans. Nodules were stratified according to the NELSON-plus-protocol; <100 mm3 as negative and ≥100 mm3 as indeterminate/positive; as 100mm3 is accepted as the upper limit for benign nodule growth. A consensus reference read was performed by experienced radiologists, and nodule-volume discrepancies reported as negative-misclassifications (NM [classified by AI as <100 mm3 but ≥100 mm3 by consensus]) and positive misclassifications (PM [classified as ≥100 mm3 by AI but <100 mm3 by consensus]). Results of v 1.1.39.14 were compared to those of an earlier version (v 1.0.34).
**Results or Findings:** Preliminary results were as follows; 1502 nodules were detected, of which 1019 were solid-non-calcified and 404 part-solid. AI v 1.1.39.14 had 44 discrepancies with consensus, 3 NMs and 41 PMs, all of which less than reported in v 1.0.34. In 7 out of 8 NMs reported in v 1.0.34, v 1.1.39.14 correctly classified the nodule, and 1 nodule remained a NM albeit now being classified as solid instead of part-solid. 2 new NMs were identified in v 1.1.39.14.
**Conclusion:** Our preliminary results show this updated AI-prototype achieved excellent results in terms of negative predictive value, which would be beneficial in LDCT LCS for ruling-out benign nodules at baseline.
**Limitations:** Aforementioned results are from a dataset where cases had ≥1 nodule present. Validation will be performed in a LCS-dataset where cases of no-nodules are also present.
**Ethics committee approval:** No information provided by the submitter.
**Funding for this study:** No funding was received for this study.
**Author Disclosures:**
Marjolein A. Heuvelmans: Nothing to disclose
Harriet Louise Lancaster: Nothing to disclose
Matthijs Oudkerk: Shareholder: Institute for Diagnostic Accuracy
Jaeyoun Yi: Shareholder: Coreline Soft

### RPS 1705-4

**Baseline whole-lung CT features deriving from deep learning and radiomics: prediction of benign and malignant pulmonary ground glass nodules**

*W. Huang*[1], Z. TaoHu[1], L. Fan[2]; [1]Weifang/CN, [2]Shanghai/CN

**Purpose:** The aim of this study was to develop and validate a model for the prediction of benign and malignant GGNs based on whole-lung baseline CT features deriving from deep learning and radiomics.
**Methods or Backround:** This retrospective study included 383 GGNs from 2 centres, confirmed by pathology. A total of 268 nodules were randomly divided into the training (n=161) and internal validation (n=107) cohort, and another 115 nodules were used as an external validation cohort. Clinical and morphological features (CMF) of GGNs at baseline chest CT were evaluated, and the whole-lung radiomics features (WLRF) were extracted simultaneously. The general neural network was used to adjust the weight ratio of each radiomics feature intelligently, and the CMF was combined with WLRF to build prediction model 1 (CMF+WLRF). Besides, baseline whole-lung CT image features (WLIF) are further assisted and extracted using the convolutional neural network. Then, WLIF was combined with CMF and WLRF to build Model 2 (CMF+WLIF) and Model 3 (CMF+WLRF+WLIF). The area under the receiver operator characteristic curve (AUC) was used to compare the prediction performance among the three models. The DeLong test was used to compare AUCs between models.
**Results or Findings:** The AUCs of model 1, model 2, and model 3 on the internal validation cohort were 0.83, 0.83, and 0.85, respectively. The AUCs of model 1, model 2, and model 3 on the external validation cohort were 0.80, 0.81, and 0.83, respectively.
**Conclusion:** The whole-lung baseline CT features to predict the benign and malignant GGNs was feasible and the model combining CMF, WLRF, and WLIF showed the best performance.
**Limitations:** The number of GGNs is not large enough. Since this was a multicentre study, the CT scanning and reconstruction parameters differed, affecting the models' performance.
**Ethics committee approval:** This study was approved by the institutional review committee of the Second Affiliated Hospital of Naval Medical University, and patients' informed consent was waived because it was a retrospective study.
**Funding for this study:** Funding for this study was received from the National Natural Science Foundation of China (81871321, 81930049, 82171926).
**Author Disclosures:**
Zhou TaoHu: Nothing to disclose
Li Fan: Nothing to disclose
Wenjun Huang: Nothing to disclose

### RPS 1705-5

**Using an artificial intelligence algorithm[5] to improve radiologists' performance in detecting pulmonary nodules in chest-CT scans: a multireader multicase study**

S. Mandal, D. Robert, R. Chouhan, P. Vanapalli, V. Challa, S. Sathyamurthy, A. Chouksey, P. Putha, *A. Modi*; Bangalore/IN
*(ankit.modi@qure.ai)*

**Purpose:** Nodules are the most common findings in early-stage lung cancer chest-CT scans. Various types of observer error and their medico-legal consequences have been reported. This study evaluates an AI algorithm's effect on radiologists' nodule detection performance.
**Methods or Backround:** 262 NLST images were randomly selected. Ground truth was established by a radiologist with more than 20 years of experience. Eight radiologists with varying experience (2–7 years ) interpreted the images in both unaided and aided session separated by at least four weeks. True positive was defined as a marking which had an overlap with ground truth boundary. Time to read a scan was captured during all the readings. Data was analysed using a two-way mixed effects ANOVA to estimate the difference in nodule-level sensitivity of readers between aided and unaided detection.
**Results or Findings:** 189 of the 262 scans had at least one nodule as per ground truth. 299 nodules were present in these 189 scans. The average number of nodules per each nodule-containing scan was 1.58. When AI was used, reader sensitivity increased from 83.4% to 90.0% (6.6% difference, 95% CI: 4.7% - 8.5%, p-value < 0.00001). Readers correctly identified an average of approximately 40 more nodules. The average number of false positives per image (FPPI) when unaided was about 0.298 and 0.169 when AI was used. The median time to complete one scan was 5.81 and 10.67 minutes with and without AI respectively.
**Conclusion:** AI has the potential to improve radiologists' nodule detection performance, reduce reading time, and reduce missed nodules.
**Limitations:** This was a pilot study; it is not intended to be generalisable across a wider reader population. There was only one evaluator. Only binary ratings from readers were obtained.
**Ethics committee approval:** No information provided by the submitter.
**Funding for this study:** Not applicable
**Author Disclosures:**
Dennis Robert: Employee: Qure.ai
Rohit Chouhan: Employee: Qure.ai
Preetham Putha: Founder: Qure.ai
Ankit Modi: Founder: Qure.ai
Saigopal Sathyamurthy: Employee: Qure.ai
Souvik Mandal: Employee: Qure.ai
Vikash Challa: Employee: Qure.ai
Amit Chouksey: Employee: Qure.ai
Prakash Vanapalli: Employee: Qure.ai

### RPS 1705-6

**Improving computer-aided malignancy estimation of pulmonary nodules using unannotated chest CT data**

*S. S. Chennamsetty*[1], S. Vaidya S[2], A. Chunduru[2], K. C. Kaluva[1], V. Saxena[3], B. Aggarwal[3], C. H. Nomura[4], Z. Udwadi[5], M. Pereira[5]; [1]Bangalore/IN, [2]Bengaluru/IN, [3]New Delhi/IN, [4]São Paulo/BR, [5]Mumbai/IN
*(saisaketh.c@nference.net)*

**Purpose:** The aim of this study was to evaluate the performance of a computer-aided malignancy estimation model using a self-supervised deep learning model, and compare it with the Pan-Canadian Early Detection of Lung Cancer (PanCan) model for estimation of malignancy risk of pulmonary nodules.
**Methods or Backround:** We developed a self-supervised learning-based deep learning pipeline for malignancy estimation of pulmonary nodules from chest CT imaging. In the initial learning phase, we trained a Resnet-50 CNN using Bootstrap Your Own Latent methodology using 9062 3D nodule patches obtained from 3063 patients in LIDC-IDRI and NLST datasets. No malignancy labels were required for this step. For the downstream task, we fine-tuned our model on nodules from 1074 patients from multiple institutions, against their respective biopsy-proven malignancy status. We compared against the PanCan model, which requires additional clinical data and nodule characteristics for predicting the malignancy status. The test dataset contained 189 patients from an independent centre not part of the training or validation set.
**Results or Findings:** The test dataset consisted of 189 patients (23 primary malignancy, 133 metastatic malignancy, 19 benign and 14 infection). We measured and compared the sensitivity, specificity and area under the curve (AUC) for our system versus the PanCan model on this dataset. Our system achieves an AUC of 0.78, whereas the PanCan model achieves an AUC of 0.7.
**Conclusion:** Self-supervision-based learning (SSL) pipelines are an effective way of training deep learning models when obtaining labelled data is a tedious process. Using an SSL-based pipeline, we achieve better results over the PanCan model, without the need for patient information.
**Limitations:** Testing on large scale multi centred data can help prove the generalisability.
**Ethics committee approval:** No information provided by the submitter.
**Funding for this study:** Funding was received from Predible, Supported by AstraZeneca.
**Author Disclosures:**
Vivek Saxena: Nothing to disclose
Melvika Pereira: Nothing to disclose
Suthirth Vaidya S: Employee: Nference inc.
Cesar Higa Nomura: Nothing to disclose
Krishna Chaitanya Kaluva: Employee: Nference inc.
Sai Saketh Chennamsetty: Employee: Nference inc.
Zarir Udwadi: Nothing to disclose
Bharat Aggarwal: Nothing to disclose
Abhijith Chunduru: Employee: Nference inc.

### RPS 1705-7

**The effect of applying an uncertainty estimation method on the performance of a deep learning model for nodule malignancy risk estimation**

*D. Peeters*[1], N. Alves[1], K. V. Venkadesh[1], R. Dinnessen[1], Z. Saghir[2], C. M. Schaefer-Prokop[3], R. Vliegenthart[4], M. Prokop[1], C. Jacobs[1]; [1]Nijmegen/NL, [2]Hellerup/DK, [3]Amersfoort/NL, [4]Groningen/NL

**Purpose:** Artificial Intelligence (AI) algorithms often lack uncertainty estimation for classification tasks. Uncertainty estimation may however be an important requirement for clinical adoption of AI algorithms. In this study, we integrate a method for uncertainty estimation into a previously developed AI algorithm and investigate the performance when applying different uncertainty thresholds.
**Methods or Backround:** We used a retrospective external validation dataset from the Danish Lung Cancer Screening Trial, containing 818 benign and 65 malignant nodules. Our previously developed AI algorithm for nodule malignancy risk estimation was extended with a method for measuring the prediction uncertainty. The uncertainty score (UnS) was calculated by measuring the standard deviation over 20 different predictions of an ensemble of AI models. Two UnS thresholds at the 90th and 95th percentile were applied to retain 90% and 95% of all cases as certain, respectively. For these scenarios, we calculated the area under the ROC curve (AUC) for certain and uncertain cases, and for the full set of nodules.
**Results or Findings:** On the full set of 883 nodules, the AUC of the AI risk score was 0.932. For the 90th and 95th percentile, the AUC of the AI risk score for certain cases was 0.934 and 0.935, respectively, and for the uncertain cases was 0.710 and 0.688, respectively.
**Conclusion:** In this retrospective dataset, we demonstrate that integrating an uncertainty estimation method into a deep learning-based nodule malignancy risk estimation algorithm slightly increased the performance on certain cases. The AI performance is substantially worse on uncertain cases and therefore in need of human visual review.
**Limitations:** This study is a retrospective analysis on data from one single lung cancer screening trial. More external validation is needed.
**Ethics committee approval:** No information provided by the submitter.
**Funding for this study:** Funding for this study was received from the Dutch Cancer Society.
**Author Disclosures:**
Natalia Alves: Nothing to disclose
Renate Dinnessen: Nothing to disclose
Cornelia M. Schaefer-Prokop: Nothing to disclose
Dre Peeters: Nothing to disclose
Mathias Prokop: Research/Grant Support: Research grant and royalties to institution from MeVis Medical Solutions, Bremen, Germany
Kiran Vaidhya Venkadesh: Nothing to disclose
Zaigham Saghir: Nothing to disclose
Rozemarijn Vliegenthart: Nothing to disclose
Colin Jacobs: Research/Grant Support: Research grant and royalties to institution from MeVis Medical Solutions, Bremen, Germany

### RPS 1705-8

**Deploying a dual-AI algorithm in routine clinical workflow to detect missed findings on thoraco-abdominal CT**

*F. U-H. Chowdhury*[1], S. Karthik[1], M. Darby[1], R. Albazaz[1], D. J. M. Tolan[1], S. Burbidge[1], S. Swift[1], Y. Pfeffer[2], J. Balcombe[2]; [1]Leeds/UK, [2]Rechovot/IL

**Purpose:** Missed pulmonary nodules and dilated thoracic/abdominal aorta may lead to worsened patient outcomes. Computer aided detection (CAD) devices generate a large number of alerts, many of which are redundant. Filtration of CAD outputs using natural language processing (NLP) analysis of radiologist reports alerts only to unreported findings.
**Methods or Backround:** Real-time computer vision analysis of 8947 thoraco-abdominal CT scans and NLP analysis of corresponding radiologist reports, to detect missed pulmonary nodules > 6 mm, and dilated aorta (thoracic > 45 mm, abdominal > 35 mm). Outputs sent to reporting radiologist for review.

Saturday

**Results or Findings:** Positive imaging findings identified in 2424/8947 (27.1%) cases. 1988/2424 (82.0%) were already reported as per NLP. 436/8947 (4.9%) cases with unreported findings as per NLP were alerted to radiologists, who approved as actionable findings 32/373 missed nodules and 7/63 missed dilated aortas. Estimated total radiologist review time was 291 minutes (436 alerts x 40 seconds review time per alert). Overall, in 39/8497 (0.44%) of cases an actionable missed finding was detected, at a cost of 7.5 minutes (291/39) of radiologist time each.
**Conclusion:** A dual algorithm combining computer vision analysis of thoracic-abdominal CT and NLP report analysis outputted a small proportion (4.9%) of scans for review, with relatively high probability (8.9%) of actionable missed nodule or missed dilated aorta. The high yield and small volume output may justify implementation of the dual AI algorithm in the clinical workflow to improve detection of actionable pulmonary nodules and dilated aortas with minimal increased radiologist workload.
**Limitations:** Longterm follow-up of actionable findings is being awaited.
**Ethics committee approval:** No information provided by the submitter.
**Funding for this study:** Funding was received from the Leeds Hospitals Charity (£5000) for technical implementation.
**Author Disclosures:**
Sarah Swift: Nothing to disclose
Damian John Michael Tolan: Nothing to disclose
Shishir Karthik: Nothing to disclose
Jonathan Balcombe: Board Member: Chief Medical Officer
Fahmid Ul-Haque Chowdhury: Nothing to disclose
Raneem Albazaz: Nothing to disclose
Michael Darby: Nothing to disclose
Simon Burbidge: Nothing to disclose
Yitzi Pfeffer: Board Member: Chief Technical Officer

### RPS 1705-9

**Automatic detection and classification of lesion changes in longitudinal studies by bipartite graph matching**
S. Rochman, R. Lederman, J. Sosna, *L. Joskowicz*; Jerusalem/IL
*(josko@cs.huji.ac.il)*

**Purpose:** Radiological follow-up of cancer patients requires the quantitative comparison of current and prior volumetric scans, which is time-consuming and requires expertise. This study evaluated the performance of a novel automatic method for the detection and classification of lesion changes in longitudinal studies.
**Methods or Backround:** The method simultaneously analyses lesions in consecutive pairs of scans. It inputs the lesion segmentations in each scan (obtained manually or automatically) and outputs the matching lesions and the lesion changes. It automatically registers the prior and current lesions and matches them based on their spatial location and proximity with a model-based bipartite graph technique. Lesion changes are then classified into three types: first, lesions appearing in both scans (existing), second, only in the current scan (new), third, only in the prior scan (disappeared). The method was evaluated on two datasets. The lung metastases dataset consists of 48 pairs chest CT scans from 17 patients with a total of 691 lesions (10.6 ±10.1 lesions/scan). The liver metastases dataset consists of 160 pairs of abdominal CECT scans from 49 patients with a total of 1692 lesions (11.2 ±9.0 lesions/scan). Ground truth manual lesion segmentations, lesion matching and lesion changes types were obtained from an expert radiologist. Accuracy was evaluated by comparing the ground truth and the computed lesions' bipartite graphs.
**Results or Findings:** Our method correctly classifies the existing, new, and disappeared lesion changes types for the lung and liver metastases: 389/391 (99.5%), 141/142 (99.3%), 96/96 (100%), and 1165/1244 (93.6%), 704/722 (97.5%), 453/476 (95.2%), with 394/395 (99.7%) and 1252/1318(95.0%) correct matching edges, respectively.
**Conclusion:** Computer-aided lesion matching and analysis of lesion in longitudinal studies may improve quantitative follow-up and evaluation of disease status, treatment efficacy and response to therapy.
**Limitations:** There was single-observer annotation and data was obtained from only one centre.
**Ethics committee approval:** No information provided by the submitter.
**Funding for this study:** No funding was received for this study.
**Author Disclosures:**
Richard Lederman: Consultant: HighRAD Ltd
Leo Joskowicz: Consultant: HighRAD Ltd
Shalom Rochman: Nothing to disclose
Jacob Sosna: Consultant: HighRAD Ltd

**08:00-09:00** **Research Stage 2**

### Research Presentation Session: Breast

### RPS 1702

### B3 lesions: addressing the uncertain malignant potential management of elevated risk (high-risk) breast lesions

**Moderator**
N. Sharma; Leeds/UK
**Author Disclosures:**
Nisha Sharma: Advisory Board: hologic, BD

### RPS 1702-2

**Upgrade rates of non-malignant breast papillary lesions diagnosed on core needle biopsy: a meta-analysis of 5271 lesions**
*M. Cao*[1], A. Cozzi[2], L. Rossi[2], M. Curti[2], F. Del Grande[2], S. Schiaffino[2]; [1]Milan/IT, [2]Lugano/CH
*(mengmengcao3@gmail.com)*

**Purpose:** To perform a systematic review and meta-analysis of the upgrade rates to in situ and invasive cancer of non-malignant breast papillary lesions diagnosed on core needle biopsy.
**Methods or Backround:** Following the PRISMA checklists, the PubMed and EMBASE databases were searched for articles published up to December 31, 2021, reporting the upgrade to in situ or invasive cancer of non-malignant breast papillary lesions diagnosed on core needle biopsy, with surgical pathology and/or follow-up as reference standard. Articles were selected in two rounds (title and abstract, then full-text) by two independent readers. Random-effects meta-analyses of single proportions with the Freeman-Tukey transformation were performed on included articles.
**Results or Findings:** A total of 63 studies (5009 women, 5271 non-malignant papillary lesions) from 13 countries published between 1999 and 2021 were included in quantitative synthesis. The overall summary upgrade rate to malignancy was 11.1% (95%CI 9.1–13.5%), whereas the overall summary upgrade rate to in situ cancer was 8.5% (95%CI 5.7–12.1%) and the overall summary upgrade rate to invasive cancer was 2.2% (95%CI 0.7–3.3%). Among 1987 lesions specified to be without atypia (from 27 studies) the summary upgrade rate to malignancy was 3.6% (95%CI 2.6–6.8%), the summary upgrade rate to in situ cancer was 3.5% (95%CI 1.9–5.6%), and the summary upgrade rate to invasive cancer was 0.1% (95%CI 0.0–0.4%). Among 626 papillary lesions with atypia (from 25 studies) the summary upgrade rate to malignancy was 37.9% (95%CI 24.8–50.7%), the summary upgrade rate to in situ cancer was 22.4% (95%CI 13.0–30.9%), and the summary upgrade rate to invasive cancer was 4.6% (95%CI 0.7–7.9%).
**Conclusion:** The upgrade of core needle biopsy-diagnosed breast papillary lesions to in situ cancer is four- to 35-fold more frequent than the upgrade to invasive cancer.
**Limitations:** This study represents a preliminary analysis without meta-regressions.
**Ethics committee approval:** No information provided by the submitter.
**Funding for this study:** No funding was received for this study.
**Author Disclosures:**
Andrea Cozzi: Nothing to disclose
Mengmeng Cao: Nothing to disclose
Simone Schiaffino: Nothing to disclose
Filippo Del Grande: Nothing to disclose
Lorenzo Rossi: Nothing to disclose
Marco Curti: Nothing to disclose

### RPS 1702-3

**Breast lesions of uncertain malignant potential (B3): 19 years of monocentric experience**
*C. Bellini*[1], G. Bicchierai[2], A. Franconeri[2], D. De Benedetto[3], F. Di Naro[3], J. Nori[2], V. Miele[3]; [1]Scandicci/IT, [2]Castelfiorentino/IT, [3]Florence/IT
*(1chiarabellini@gmail.com)*

**Purpose:** The purpose of this study was to assess the general trend and outcome of breast lesions of uncertain malignant potential (B3) in the last years, evaluating clinical and radiological predictive factors of malignancy.
**Methods or Backround:** We retrospectively included 1182 women with a histological diagnosis of B3 from 2000 to 2019. Each year B3 frequencies and outcomes were reported. Positive Predictive Value (PPV) of sex (male, female), age (<45, 45-75, >75 years old), clinical suspicion (palpable: benign, equivocal, malignant; not palpable), American College Radiology – ACR breast density, radiological findings (mass, microcalcifications, architectural distortion), size (<10, 10-20, >20 mm), radiological suspicion (BI-RADS), Core

Saturday

Biopsy procedure (Needle Core Biopsy – NCB, Vacuum-Assisted Biopsy – VAB), guidance (ultrasound, stereotactic/tomosynthesis, magnetic resonance imaging, freehand), needle (8G, 11G, 14G) and histological subtype were evaluated in 778 women with histological surgical specimen after Open Excision (OE) or Vacuum-Assisted Excision (VAE), or proof of stability on follow-up.
**Results or Findings:** The average frequency of B3 on total biopsies was 3.77% (range: 1.88%-6.06%), with an upward trend over the years. The overall malignancy rate of B3 lesions was 20.4% (10% carcinoma in situ and 10.4% invasive). The most significant predictors of malignancy (PPV) included age > 75 years old (48.8%), malignant palpable lesion (72.7%), B ACR breast density (29.6%), mass associated with microcalcifications (34.9%), size >20 mm (33.3%), BI-RADS 5 (35.9%), NCB procedure (27.1%), 14G (27.9%) and atypical ductal hyperplasia – ADH (40.9%).
**Conclusion:** The percentage of B3 lesions has increased over the years, with an overall malignancy rate of 21.9%. ADH had the highest association with malignancy among all histological subtypes. Clinical and radiological factors proved useful outcome predictors.
**Limitations:** This was a retrospective study.
**Ethics committee approval:** No information provided by the submitter.
**Funding for this study:** Not applicable
**Author Disclosures:**
Andrea Franconeri: Nothing to disclose
Giulia Bicchierai: Nothing to disclose
Jacopo Nori: Nothing to disclose
Vittorio Miele: Nothing to disclose
Chiara Bellini: Nothing to disclose
Federica Di Naro: Nothing to disclose
Diego De Benedetto: Nothing to disclose

### RPS 1702-4

**B3 lesions detected at Ultrasound Core-Needle biopsy: could we safely avoid surgery?**

*A. Biondo*, N. Damiani, G. Bartoli, E. Regini, I. Castellano, A. Pittaro, L. Bergamasco, M. Durando, P. Fonio; Turin/IT
*(andrea.biondo25@gmail.com)*

**Purpose:** The purpose of this study was to evaluate the upgrade to malignancy after surgical excision of breast lesions of uncertain malignant potential (B3) detected at ultrasound core-needle biopsy (US-CNB).
**Methods or Backround:** A retrospective study including consecutive patients who underwent US-CNB classified as B3 between January 2018 and January 2022 in our Breast Imaging Service was performed. Exclusion criteria were vacuum-assisted biopsy (VAB)-proven B3 lesions, lesions referred to follow-up rather than surgery after multidisciplinary evaluation and those not already excised. All patients included were classified according to clinical and radiological findings (size, ultrasonographic features and BI-RADS categories).The US-CNB outcome was compared to pathology, considered the gold standard, in order to evaluate the upgrade to malignancy after surgery. Uni- and multivariate analysis was performed on the data.
**Results or Findings:** During the study period, 414 patients received the diagnosis of B3 lesions: 175/414 performing VAB and 239/414 by US-CNB. Of those, 91/239 were sent to follow-up and 148/239 (35,7%) to surgery, only 132 with final pathology available. Among these 132 patients (average age 48,2 years), B3 lesions (average size 15,2 mm, range 4-70 mm) were mostly hypoechoic masses (87%) with circumscribed margins (73%), BI-RADS category 3 (71,2%). US-CNB histologic results were as follows: 28 (21,2%) papillary lesions (PL), 21 (16%) atypical ductal hyperplasia (ADH), 27 (20,5%) radial scar (RS/CSL), 28 (21,2%) fibroepithelial lesions (FE),13 (9,8%) flat epithelial atypia (FEA), 9 (6,8%) lobular neoplasia (LN), 3 (2,3%) PASH and 3 (2,3%) phyllodes tumours (PT). 41% of the overall B3-classified lesions had atypia. After surgical excision, 13/132 (9,8%) B3 lesions were upgraded to cancer (11 in situ, 2 invasive), predominantly ADH (7/13; 53,8%) and RS/CSL (4/13; 3%). Patients' age >66years (OR=3,63; p=0.008), BI-RADS category 4 (OR=5,41; p=0.04) and ADH-US-CNB outcome (OR=11,3; p<0.001) were associated with a higher risk of malignancy.
**Conclusion:** Our findings suggest that US-CNB-diagnosed B3 lesions demonstrate a good reliability to pathology and a low upgrade-to-malignancy rate, enforcing the choice to "safely" avoid unnecessary excisions, but paying attention to ADH lesions, BI-RADS category 4 and older patients .
**Limitations:** This was a retrospective study.
**Ethics committee approval:** No information provided by the submitter.
**Funding for this study:** No funding was received for this study.
**Author Disclosures:**
Manuela Durando: Nothing to disclose
Nicolò Damiani: Nothing to disclose
Elisa Regini: Nothing to disclose
Alessandra Pittaro: Nothing to disclose
Isabella Castellano: Nothing to disclose
Laura Bergamasco: Nothing to disclose
Germana Bartoli: Nothing to disclose
Paolo Fonio: Nothing to disclose
Andrea Biondo: Nothing to disclose

### RPS 1702-5

**Ultrasound-guided vacuum-assisted excision (VAE): management of breast lesions with imaging-histology discordance**

*S. Marziali*[1], C. Depretto[2], L. Rabiolo[2], E. D'Ascoli[2], S. Carriero[2], G. Irmici[2], C. De Berardinis[2], G. Della Pepa[2], G. P. Scaperrotta[2];
[1]San Donato Milanese/IT, [2]Milan/IT
*(Sara.Marziali@unimi.it)*

**Purpose:** Core biopsy is a reliable approach for diagnosing suspicious breast findings. However, false negative results occur in 1.6-6% of cases due to insufficient biopsy samples or closely linked anatomopathological diseases. Imaging-histology discordance is defined as a lesion presenting with imaging features suspicious for malignancy but benign pathology at percutaneous breast biopsy: such cases eventually undergo a more invasive surgical excisional biopsy to collect a larger histology sample. This study aims to evaluate the efficacy of ultrasound-guided vacuum-assisted excision (VAE) as an alternative treatment for discordant breast lesions.
**Methods or Backround:** VAE was used to perform a percutaneous ultrasound-guided excision of discordant breast lesions between 01/2015 and 04/2018. Lesions with a mean diameter of 10.3 mm (range: 3-30 mm) and suspicious ultrasound imaging (BI-RADS≥4) but non-malignant histopathology were classified as B1 (n = 18), B2 (n = 109) and B3 (n = 32). We evaluated the upgrade rate (UR), the technical success (TS), and technical effectiveness (TE) of the VAE, as well as the delayed false negative findings (DR), with radiological follow-up over the next two years. There were only seven minor complications (4.4%): haematomas, seromas and liponecrosis.
**Results or Findings:** 159 consecutive discordant breast lesions underwent VAE. The post-VAE histological outcomes were: B1 (n = 7), B2 (n = 117), B3 (n = 24) and B5 (n = 11), of which five CDI (2 associated to DCIS) and six DCIS, all surgically radicalised. The UR was 6.9%, TS 100%, TE 87.9%, DR 0%. The complications were minimal.
**Conclusion:** The study indicates that VAE excision is a viable alternative to surgery for discordant lesions; compared to surgical excision, it offers lower costs, simplified management, it is better tolerated, and has less cosmetic impact on patients.
**Limitations:** The operator dependency of US-guided biopsy was identified as a limitation.
**Ethics committee approval:** This single-centre retrospective study was approved by the institutional review board and informed consent was waived.
**Funding for this study:** No funding was received for this study.
**Author Disclosures:**
Claudia De Berardinis: Nothing to disclose
Elisa D'Ascoli: Nothing to disclose
Sara Marziali: Nothing to disclose
Gianmarco Della Pepa: Nothing to disclose
Serena Carriero: Nothing to disclose
Gianfranco Paride Scaperrotta: Nothing to disclose
Catherine Depretto: Nothing to disclose
Giovanni Irmici: Nothing to disclose
Lidia Rabiolo: Nothing to disclose

### RPS 1702-6

**Single-centre evaluation of percutaneous vacuum-assisted excision (VAE) of breast lesions of uncertain malignant potential (B3 lesions) as an alternative to open surgical biopsy**

G. Bicchierai, *F. Pugliese*, D. De Benedetto, F. Di Naro, V. Miele, J. Nori; Florence/IT
*(francescapugliese28@gmail.com)*

**Purpose:** The aim of this study was to assess the effectiveness of using VAE to manage some selected B3 lesions in a prospective single-centre study approved by the ethics committee of our university hospital by quantifying the number of B3 lesions undergoing VAE, the malignant upgrade rate and the complications encountered.

**Methods or Backround:** All B3 lesions diagnosed between 01/2020 and 12/2021 were identified. Information was obtained on the initial biopsy and final histology, method of VAE image guidance, needle size, number of cores and complications. The exclusion criteria were: size> 20 mm, papillary lesions with atypia, spindle cell tumours, fibroepithelial lesions and presence of cancer in the breast at the time of diagnosis, according to the NHSBSP guidelines. The final decision to participation in the study was made at a breast multidisciplinary meeting.
**Results or Findings:** In total, 234 B3 lesions were diagnosed, 74 (31.6%) met the inclusion criteria and underwent VAE. VAE was performed under x-ray (57/74) or ultrasound guidance (17/74), taking an average of 19.5 cores with a 8-G needle. Five cases (6.7%) were upgraded to a malignant diagnosis following VAE, 3 ADH lesions and 2 LIN. Only minor complications were recorded i.e. haematomas and bleeding, in 7 patients (9.5%). No new lesions or malignancy has occurred at the site of the VAE with an average mammographic follow-up of 21 months.
**Conclusion:** VAE could be a safe and effective pathway for the management of some selected B3 lesions, reducing the number of open surgical procedures.
**Limitations:** The main limitations of the study are represented by the small number of lesions included, even if the number of cases is not particularly low for a single-centre, and by an average follow-up time that is still relatively short.
**Ethics committee approval:** No information provided by the submitter.
**Funding for this study:** Not applicable
**Author Disclosures:**
Giulia Bicchierai: Nothing to disclose
Federica Di Naro: Nothing to disclose
Francesca Pugliese: Nothing to disclose
AOU Careggi Diego De Benedetto: Nothing to disclose
Jacopo Nori: Nothing to disclose
Vittorio Miele: Nothing to disclose

### RPS 1702-7

**Performance of a multivariable model to predict upgrade risk of atypical ductal hyperplasia diagnosed on vacuum assisted breast biopsy: decision support tool to avoid unnecessary surgeries**

*R. Rella*[1], M. Conti[1], P. Belli[1], C. Rossati[2], F. Morciano[1], J. Nunnari[1], O. Tommasini[1], F. Fornasa[3], G. Romanucci[2]; [1]Rome/IT, [2]Verona/IT, [3]San Bonifacio/IT
*(rossella.rella@gmail.com)*

**Purpose:** To develop an imaging and histopathological-based multivariable model to predict malignant upgrade at surgical excision in patients with atypical ductal hyperplasia (ADH) diagnosed on stereotactic/tomosynthesis-guided vacuum assisted breast biopsy (VABB).
**Methods or Backround:** In three Italian centres, the authors retrospectively reviewed the institutional database to identify patients with ADH diagnosed by VABB. Inclusion criteria: surgical excision of the lesion or availability of radiologic follow-up (FUP)≥24 months. Risk factors for ADH upgrade were identified by multivariable logistic regression analysis with stepwise backward selection of variables (only variables that showed association with the outcome at a p-value<0.100 were included). The discriminatory power of the model was calculated through the Area Under the Receiver Operating Characteristic Curve (ROC AUC). The Hosmer-Lemeshow test was used to assess model calibration.
**Results or Findings:** 112 ADH included: 91/112 (81.3%) underwent surgical excision and 20 proved malignant; 21/112 (18.7%) sent to FUP and one developed breast cancer (BC) after eight years. The overall upgrade rate was 18.7% (12.5% to ductal carcinoma in situ and 6.2% to invasive BC). Patients' age (odds ratio [OR]=2.54, P=0.038), synchronous BC (OR=31.82, P=0.039), BI-RADS category (OR=5.60, P=0.042) and number of foci of ADH on biopsy (OR=7.48, P=0.002) were associated with a higher risk of malignancy underestimation and selected as risk factors in the score definition. Our model had an AUC=0.85 (95%CI 0.76-0.94). An estimated 22.3% of benign surgeries could be avoided with 100% sensitivity according to the fitted model.
**Conclusion:** Our multivariate model showed a high diagnostic performance in decreasing unnecessary surgeries and seemed a promising decision support tool for management of ADH.
**Limitations:** A limitation of our study is its retrospective design - thus, it will be useful to validate these findings in a prospective cohort.
**Ethics committee approval:** No information provided by the submitter.
**Funding for this study:** This research received no external funding.
**Author Disclosures:**
Francesca Morciano: Nothing to disclose
Josè Nunnari: Nothing to disclose
Rossella Rella: Nothing to disclose
Oscar Tommasini: Nothing to disclose
Francesca Fornasa: Nothing to disclose
Marco Conti: Nothing to disclose
Giovanna Romanucci: Nothing to disclose
Claudia Rossati: Nothing to disclose
Paolo Belli: Nothing to disclose

### RPS 1702-8

**Radio-histological analysis of B3 lesions with atypical ductal hyperplasia: development of a breast cancer risk assessment model**

*S. Zanni*, A. Pecchi, R. Battista, B. Canossi, A. Drago, P. Torricelli, G. Tazzioli, L. Cortesi, R. Cuoghi Costantini; Modena/IT

**Purpose:** This study presents an exploratory retrospective analysis of histologically confirmed B3 breast lesions with atypical ductal hyperplasia (ADH) to identify risk factors for the upgrade of lesions to B5.
**Methods or Backround:** We selected 206 patients, with a core needle biopsy or vacuum assisted breast biopsy (CNB/VABB) resulting in a B3 lesion with ADH in the 2014-2019 period. Breast density and appearance of lesions at mammograms were classified with BI-RADS system; histological findings in addition to ADH were noted. 107/206 patients subsequently underwent surgery; the results were classified as confirmed benign lesion or lesion upgraded to carcinoma. 99/206 patients were subjected to annual follow-up to detect eventual development of cancer.
**Results or Findings:** 48/107 (44.86%) of patients that underwent surgery had a diagnosis of B5, with ductal carcinoma in situ (66.67%) as the most common finding. We highlighted a significant direct relationship between BI-RADS scoring and prevalence of patients with malignancy at surgery (p=0.01), and between upgraded B5 lesions and higher breast density (45.65% with "c" pattern). The use of VABB in B3 patients did not reduce underestimation of malignancy, which was not related to the presence of residual microcalcifications post procedure. 4.17% of patients in follow-up received a diagnosis of malignancy.
**Conclusion:** Higher breast density and higher BI-RADS scores represent predictors of upgrade to malignancy in B3 patients with ADH. Residual microcalcifications after VABB cannot be a selective criterion to surgical biopsy after a histological diagnosis of ADH.
**Limitations:** Possible limitations are due to the fact that this study is a retrospective one, and that there is an accuracy difference between CNB and stereotactic VABB, since the former method is more operator-dependent.
**Ethics committee approval:** No information provided by the submitter.
**Funding for this study:** The study did not receive external funding.
**Author Disclosures:**
Barbara Canossi: Nothing to disclose
Pietro Torricelli: Nothing to disclose
Annarita Pecchi: Nothing to disclose
Sara Zanni: Nothing to disclose
Riccardo Cuoghi Costantini: Nothing to disclose
Laura Cortesi: Nothing to disclose
Giovanni Tazzioli: Nothing to disclose
Rachele Battista: Nothing to disclose
Antonella Drago: Nothing to disclose

### RPS 1702-9

**Architectural distortions of the breast in tomosynthesis versus synthetic mammography and ultrasound: histopathological correlation**

G. Romanucci[1], *C. Rossati*[1], R. Rella[2], F. Fornasa[1]; [1]Verona, VR/IT, [2]Rome/IT

**Purpose:** The purpose of this study was to compare histological results of architectural distortions (AD) when they are detected by tomosynthesis (DBT) versus synthetic mammography (synt2D) or ultrasound (US).
**Methods or Backround:** Data from Patients with AD who had undergone US-CNB or DBT-VAB between September 2019 and August 2021 were retrospectively reviewed.
**Results or Findings:** The DBT-VAB group had 111/123 AD (90,2%) and 26/111 (23,4%) were DBT only. On histology we found the following: 3/26 (11,5%) B2, 18/26 (69,2%) B3, 5/26 (19,2%) B5. 85/111 (76,5%) were DBT and synt2D. On histology we found: 16/85 (18,8%) B2, 47/85 (55,2%) B3 and the other two (4,2%) were upgraded after surgical biopsy, 22/85 B5 (23,7%). The US-CNB group had 12/123 AD (9,7%), 3/12 (25%) were DBT and visible in US, on histology we found one B3 (33,3%) upgraded to cancer after surgical biopsy and two B5 (66,6%); 9/12 (75%) AD were visible in all methodologies, on histology we found three B2 (33,3%), two B3 (22,2%) of which one was upgraded to cancer after surgical biopsy and four B5 (44,4%). PPV for AD that were only DBT was 19.2%, PPV for lesions detectable via DBT and synt2D was 28,2%. 14/68 (20,5%) B3 lesions underwent surgical biopsy, of which 4/14 (28,5%) were upgraded to B5 and 54/68 underwent follow-up, of which 53 had negative follow-up and one had a new cancer diagnosis in a different breast quadrant.
**Conclusion:** ADs that are only DBT show a lower rate of malignancy outcome, but the rate of malignancy is not low enough to avoid biopsy. In all groups, an US correlation is shown to be a malignancy predictor. ADs, especially when only DBT, are often found to be B3 lesions at histology and have the risk of B5 upgrading after surgical biopsy.
**Limitations:** This was a retrospective singlecentre study.
**Ethics committee approval:** No information provided by the submitter.

**Funding for this study:** No funding was received for this study.
**Author Disclosures:**
Rossella Rella: Nothing to disclose
Francesca Fornasa: Nothing to disclose
Giovanna Romanucci: Nothing to disclose
Claudia Rossati: Nothing to disclose

**08:00-09:00** **Research Stage 3**

### Research Presentation Session: Neuro

### RPS 1711

### Prenatal and paediatric neuroimaging

**Moderator**
E. Vázquez; Barcelona/ES
*(elida.vazquez@vallhebron.cat)*

### RPS 1711-2

**In utero tractography of the sensorimotor tracts in fetuses with intraventricular haemorrhage (IVH): feasibility and added prognostic value**

*E. Hadi*[1], T. Dorittke[2], P. Kienast[2], A. Hershko Klement[3], G. Kasprian[2]; [1]Ramat Gan/IL, [2]Vienna/AT, [3]Jerusalem/IL
*(efrat.hadi@gmail.com)*

**Purpose:** Diffusion tensor imaging (DTI) provides information about the exact location and extent of white matter involvement in preterm neonates with IVH. Alterations in corticospinal tract (CST) characteristics may be correlated with postnatal cerebral palsy. However, the prognosis of prenatal IVH is difficult to predict. In this retrospective cohort study, the feasibility, imaging characteristics, and prognostic value of CST tractography in fetuses with IVH were assessed.
**Methods or Backround:** The left and right CSTs were reconstructed, based on an in utero DTI sequence using a multi-region of interest (ROI). Two ROI were located at the level of the posterior limb of the internal capsula and the crus cerebri. One rater blinded to structural MRI-evaluated morphology and integrity of the CSTs. Further, internal capsule/CST anisotropy and FA values were determined. Postnatal motor function data were obtained from the parents by telephone interviews.
**Results or Findings:** 39 fetuses were included. 53% demonstrated parenchymal involvement on T2-weighted sequences. CST involvement was significantly correlated with the presence of T2-w parenchymal damage (p=0.02). CST asymmetry at the level of the internal capsula was more common among fetuses who showed parenchymal involvement, but the difference was not statistically significant. Postnatal motor impairments - were distributed significantly different between cases with a CST tractographic involvement (CSTI) and those without CSTI (p=0.01); only 6.3% of those lacking CSTI developed motor impairment compared to 53.8% of those with CSTI.
**Conclusion:** Fetal CST tractography is feasible, has prognostic value and may facilitate parental counselling in cases of prenatal IVH. Tractographic involvement of the CST (CSTI) indicates a more than 50% risk for cerebral palsy, whereas over 90% of cases without CSTI show normal motor development.
**Limitations:** There was a small retrospective cohort and a small number of cases with motor outcomes.
**Ethics committee approval:** No information provided by the submitter.
**Funding for this study:** No funding was received for this study.
**Author Disclosures:**
Tim Dorittke: Nothing to disclose
Efrat Hadi: Nothing to disclose
Patric Kienast: Nothing to disclose
Anat Hershko Klement: Nothing to disclose
Gregor Kasprian: Nothing to disclose

### RPS 1711-3

**Prenatal DTI phenotypes in partial callosal agenesis**

B. Sigl, *P. Kienast*, M. Stümpflen, M. Diogo, D. Prayer, G. Kasprian; Vienna/AT
*(pat.kienast@gmail.com)*

**Purpose:** Partial corpus callosum agenesis (pCCA) is one of the most common developmental malformations of the human brain. Different phenotypes of pCCA with respect to morphology as well as connectivity have not yet been studied systematically. The aim of this study is to distinguish different pCCA-phenotypes and to evaluate the value of DTI imaging.
**Methods or Backround:** 26 prenatal MRI (GW 22+0 to 35+3; Philips Ingenia 1.5T, sense cardiac coil) in pCCA were evaluated. T2-weighted images (TR=10975ms/TE = 140ms/resolution = 0.9 x 1.37 x 3 mm3) were evaluated by two radiologists for the presence of genu, corpus, and splenium, and width of brain tissue medially to the corpora of the lateral ventricles as a possible correlate of alternative pathways. In DTI (TE = 2613/ TE = 90/ resolution = 0.94 x 0.94 x 3.00 mm3/ 16 directions/ b-value= 700s/ mm2), forceps minor, forceps major, the CC-body and Probst and Sigmoid bundles were analysed.
**Results or Findings:** DTI visualised callosal fiber portions in 77% of cases, with the forceps minor representing the largest share with 42.5% (corpus 27.5%/ forceps major 30%). Probst and/or Sigmoid bundle were present more often in cases with low or no callosal fiber count. Furthermore, DTI imaging detected 13 (32.5%) fibre tracts of the CC that could not be visualised on T2 imaging. In all cases an unequivocal detection of Probst (n=18 patients) and Sigmoid (n=8 patients) bundles was only possible by tractography. Increased brain tissue medial to the lateral ventricles, suggested the presence of these alternative pathways in 47.6% of cases.
**Conclusion:** Using morphological imaging and DTI in utero different phenotypes of pCCA could be distinguished: pCCA with no alternative pathways, Probst bundle, Sigmoid bundle, Probst and Sigmoid bundle. These alternative fibre tracts, in particular, cannot be adequately assessed in standard protocols. The forceps minor, which is formed in most cases, seems to be particularly robust in its genesis.
**Limitations:** The small case number was identified as a limiation.
**Ethics committee approval:** No information provided by the submitter.
**Funding for this study:** No funding was received for this study.
**Author Disclosures:**
Benjamin Sigl: Nothing to disclose
Patric Kienast: Nothing to disclose
Marianna Diogo: Nothing to disclose
Daniela Prayer: Nothing to disclose
Gregor Kasprian: Nothing to disclose
Marlene Stümpflen: Nothing to disclose

### RPS 1711-4

**MRI biomarkers for brain maturation in preterm infants after administration of melatonin**

*C. Asteggiano*[1], L. Mazzocchi[1], S. Aversa[2], S. Longo[1], L. Chiapparini[3], A. Simoncelli[3], S. Cociglio[1], S. Orcesi[1], A. Pichiecchio[1]; [1]Pavia/IT, [2]Brescia/IT, [3]Milan/IT

**Purpose:** Melatonin (ME) has a neuroprotective function in animal models and term infants affected by hypoxic-ischaemic encephalopathy. Early administration of ME appears to promote brain maturation with its antioxidant effect. The study aims to examine the neuroprotective effects of ME per OS in preterm infants and to identify reliable potential biomarkers of brain maturation.
**Methods or Backround:** In our prospective multicentre trial, we enrolled 60 preterm newborns in two cohorts: the melatonin group and the placebo group. In the ME group, ME was administered per OS starting at 96 hours from birth. 52 preterm infants underwent MRI on 1.5T at TEA (39 – 41 weeks) in spontaneous postprandial sleep without sedation. The MRI protocol included T1w and T2w volumetric brain sequences and 20-direction DTI, with a total acquisition time of 20 minutes. The sequences were evaluated both qualitatively by applying a validated cumulative maturity score and quantitatively by extrapolating the T1/T2 ratio. To assess the T1/T2 ratio, one neuroradiologist drew 4 ROIs around the pyramidal bundles (PB) at the posterior limb of the internal capsule and at the optic radiation (OR), just lateral to the atrium of the lateral ventricles, bilaterally. We then extrapolated the quantitative values from all the ROIs.
**Results or Findings:** We did not find a statistical significance in either the qualitative score or the T1/T2 ratio between the ME and the placebo group. However, we found a very high correlation (PEARSON test: o.89) between T1/T2 ratio values obtained from PB and OR ROIs: higher values in PB correspond to higher values in OR.
**Conclusion:** Since PB physiologically myelinates earlier than OR during brain maturation, higher T1/T2 values in PB and OR may suggest a better and earlier myelination process in preterm infants.
**Limitations:** Not applicable
**Ethics committee approval:** No information provided by the submitter.
**Funding for this study:** Not applicable
**Author Disclosures:**
Salvatore Aversa: Nothing to disclose
Simona Orcesi: Nothing to disclose
Laura Mazzocchi: Nothing to disclose
Luisa Chiapparini: Nothing to disclose
Anna Simoncelli: Nothing to disclose
Sara Cociglio: Nothing to disclose
Stefania Longo: Nothing to disclose
Anna Pichiecchio: Nothing to disclose
Carlo Asteggiano: Nothing to disclose

### RPS 1711-5

**Potential value of quantitative Magnetic Resonance Imaging (MRI) for neurologic outcome prediction in neonates formerly born extremely premature**

V. Schmidbauer, *J. Leinkauf*, M. S. Yildirim, H. Bektasli, K. Goeral, M. Stümpflen, D. Prayer, G. Kasprian; Vienna/AT
*(n11917163@students.meduniwien.ac.at)*

**Purpose:** Extremely preterm infants are at risk for neurodevelopmental delay. This study aims to identify quantitative MR-based biomarkers for the prediction of future neurologic outcomes in neonates born <28 weeks of gestation.
**Methods or Backround:** Apparent diffusion coefficients (ADC), fractional anisotropy (FA), and T1-/T2-relaxation times (T1R/T2R) of the brainstem and the posterior limb of the internal capsule (PLIC) were determined at term-equivalent ages in a sample of 33 extremely preterm infants. Cognitive, language, and motor outcome scores were collected at 12 months corrected-age. Pearson's correlation analyses detected relationships between outcome data and quantitative metrics. Moreover, stepwise regression procedures were conducted to estimate the future neurologic performance using MR-based measures.
**Results or Findings:** Significant correlations were observed between cognitive outcome and MR-based biomarkers: T2R (r=0.412; p=0.017) and ADC (r=-0.401; p=0.021) (medulla oblongata). Furthermore, significant correlations were found between motor outcomes and quantitative metrics: T1R [pontine tegmentum (r=0.346; p=0.049), midbrain (r=0.415; p=0.016), right PLIC (r=0.513; p=0.002), and left PLIC (r=0.504; p=0.003)]; T2R [right PLIC (r=0.405; p=0.019)], ADC [medulla oblongata (r=-0.408; p=0.018) and pontine tegmentum (r=-0.414; p=0.017)], and FA [pontine tegmentum (r=-0.352; p=0.045)]. T2R/ADC (medulla oblongata) [cognitive outcomes (R2=0.296; p=0.037)] and T1R (right PLIC)/ADC (medulla oblongata) [motor outcomes (R2=0.405; p=0.009)] revealed predictive potential for future neurologic development.
**Conclusion:** There are relationships between neurologic performance at one year corrected-age and relaxometry-/DTI-based measures determined by neuroimaging near term. Both modalities reveal prognostic value for the prediction of cognitive and motor outcomes. Thus, quantitative MRI represents a promising approach with which to estimate neurologic development in neonates with a preterm history.
**Limitations:** Small sample size was identified as a limitation of this study.
**Ethics committee approval:** This study was approved by the ethics committee of the Medical University of Vienna.
**Funding for this study:** No funding was received for this study.
**Author Disclosures:**
Victor Schmidbauer: Nothing to disclose
Mehmet Salih Yildirim: Nothing to disclose
Joel Leinkauf: Nothing to disclose
Daniela Prayer: Nothing to disclose
Katharina Goeral: Nothing to disclose
Gregor Kasprian: Nothing to disclose
Marlene Stümpflen: Nothing to disclose
Hatice Bektasli: Nothing to disclose

### RPS 1711-6

**MRI evaluation of brain in children with developmental delay**

*R. Kalaiarasan*, V. P. Rathnasabapathy, P. Anand; Chennai/IN
*(ramyakalaiarasan90@gmail.com)*

**Purpose:** Developmental delay is termed as gross or significant delay in more than one developmental domain. MRI is the best modality to evaluate such children for early diagnosis and treatment, helps in counselling parents regarding their outcome and risk of recurrence in siblings. The main aims are to study the most common MRI findings in patients with developmental delay and to further categorise the abnormal MRI based on its findings and to help the clinician in diagnosing the cause of developmental delay and proceed to further treatment.
**Methods or Backround:** An observational and descriptive study of MRI of the Brain in 100 pediatric patients referred to department of Radio-diagnosis in Chettinad Hospital and research institute for a duration of 2 years from December 2014-december 2016 from Paediatric department for the cause of developmental delay. The patients were diagnosed with developmental delay upon taking a detailed history. Both sexes were included in the study.
**Results or Findings:** Normal MRI Findings were seen in 36% cases and abnormal findings were seen in 64% (64 cases). Furthermore, the aetiological factors were classified as traumatic/neurovascular (30%), congenital/ developmental (12%), metabolic/degenerative (8%), neoplastic (4%) and non-specific (10%). The most common features observed in the study were PVL, gliosis with volume loss and thinning of the corpus callosum. Common anatomical abnormalities are corpus callosum agenesis and nodular heterotopia.
**Conclusion:** MRI brain study is an effective tool in identifying causative factors in developmental delay and aids the clinician with proper diagnosis, treatment and counselling of the parents.
**Limitations:** Advanced MRI techniques such as diffusion tensor imaging, MR spectroscopy, functional MRI and tractography yield better results, particularly in patients with structurally normal brain - this was not done in this study.
**Ethics committee approval:** No information provided by the submitter.
**Funding for this study:** This study was self-funded.
**Author Disclosures:**
Ramya Kalaiarasan: Nothing to disclose
Vijay Prabhu Rathnasabapathy: Nothing to disclose
Prem Anand: Nothing to disclose

### RPS 1711-7

**T2-weighted Turbo-Spin-Echo PROPELLER acquisition with compressed sensing for rapid and motion-robust brain imaging in infants and children**

*B. D. Wichtmann*[1], C. Katemann[2], M. Mezger[1], Y. C. Layer[1], O. Weber[2], J. A. Luetkens[1], U. I. Attenberger[1], A. Radbruch[1], D. Paech[1]; [1]Bonn/DE, [2]Hamburg/DE
*(barbara.wichtmann@ukbonn.de)*

**Purpose:** Enabling faster and motion-robust magnetic resonance imaging (MRI) of the brain in infants and children with a new compressed sensing (CS) accelerated turbo-spin-echo (TSE) T2-weighted-sequence using the PROPELLER-technique (periodically rotated overlapping parallel lines with enhanced reconstruction, henceforth named csMVXD) compared to the conventional, CS-accelerated Cartesian TSE T2-weighted-sequence (csT2).
**Methods or Backround:** This prospective study included 22 patients (age: 5.7+/- 14.1 years, 10 male, 12 female) undergoing a clinically indicated MRI-examination of the brain on a 3T-scanner (Ingenia ElitionX, Philips Healthcare, Best, the Netherlands). The csT2-sequence (TE/TR: 90/[6316-7369]ms, flip angle 90°, acquired voxel size: 0.55 x 0.55 x 2 mm, reconstructed voxel size: 0.18 x 0.18 x 2 mm; CS-factor 2) and csMVXD-sequence (TE/TR:100/[6107-7968] ms, flip angle 90°, acquired voxel size: 0.55 x 0.55 x 2 mm and reconstructed voxel size: 0.41 x 041 x 2 mm; CS-factor 2) were acquired. Two blinded radiologists qualitatively rated both sequences on a 5-point-Likert-scale from 1-5 (nondiagnostic-excellent) for artefacts, image sharpness, basal ganglia delineation, lesion conspicuity, and overall image quality. Based on the image intensity distribution (mean, standard deviation (sd)), the apparent contrast-to-noise ratio of gray/white matter (GM/WM) (aCNR=(mean(GM)-mean(WM))/sd(muscle)) and apparent signal-to-noise ratio of GM (aSNR=mean(GM)/sd(muscle)) were calculated. Statistical analysis was performed using the Wilcoxon signed rank test and paired sample t-test. Interrater reliability of qualitative image assessment was evaluated using intraclass correlation coefficient (ICC) estimates.
**Results or Findings:** The average acquisition time of the csMVXD-sequence (211seconds) was 23% shorter than that of the csT2-sequence (276seconds; p<0.001). Excluding metal artefacts, the csMVXD-sequence significantly reduced (motion-)artefacts (p<0.001), increased image sharpness (p<0.001), basal ganglia delineation (p<0.001), lesion conspicuity (p<0.001), and overall image quality (p<0.001). Interobserver reliability for qualitative scoring was good (ICC=0.87). Quantitative analysis yielded a slightly higher aCNR (8.35+/-5.75vs.6.7+/-4.6; p=0.006) and aSNR (25.9+/-9.8vs.20.5+/-5.8; p=0.003) for the csT2-sequence.
**Conclusion:** The csMVXD-sequence enables faster and motion-robust imaging of the brain in infants and children compared to the conventional csT2-sequence, potentially reducing the rate of non-diagnostic scans.
**Limitations:** The csMVXD-sequence exhibited a slightly reduced aCNR/aSNR without affecting overall image quality. Metal artefacts, when present, were prominent in both sequences, though slightly more pronounced in the csMVXD-sequence.
**Ethics committee approval:** No information provided by the submitter.
**Funding for this study:** No funding was received for this study.
**Author Disclosures:**
Julian Alexander Luetkens: Speaker: J.A.L. received payments for lectures from Philips Healthcare.
Daniel Paech: Nothing to disclose
Barbara Daria Wichtmann: Speaker: B.D.W. received payments for lectures from Philips Healthcare.
Yannik Christian Layer: Research/Grant Support: Y.C.L is supported by a research grant from Siemens Healthcare GmbH.
Oliver Weber: Employee: Philips GmbH
Madeleine Mezger: Nothing to disclose
Ulrike I. Attenberger: Nothing to disclose
Christoph Katemann: Employee: Philips GmbH
Alexander Radbruch: Nothing to disclose

### RPS 1711-8
**1H MR spectroscopy of the brain reveals distinct metabolic abnormalities in progressive myoclonus epilepsy type 1 (EPM1)**
*J. Hakumäki*, J. Hyppönen, M. Könönen, P. Koskenkorva, E. Mervaala, R. Vanninen, R. Kälviäinen; Kuopio/FI
*(juhana.hakumaki@uef.fi)*

**Purpose:** Progressive myoclonic epilepsy type-1 (EPM1) is a rare autosomal recessive neurodegenerative disorder. It presents in early childhood with stimulus-induced myoclonus and tonic-clonic seizures and later ataxia, intention tremor, dysarthria, incoordination, and variable neuropsychiatric and cognitive decline. The disease lacks clear findings on MRI, although voxel-based morphometry reveals slight reductions in cortical and thalamic gray matter, and slightly reduced cerebellar volume, indicating neurodegeneration. Only MRS of the brainstem has been performed, which revealed lower than normal NAA/Cr ratios.
**Methods or Backround:** Our patients (n=18) underwent MR spectroscopy as part of a larger national study cohort. Normal volunteers (n=10) were studied as controls. MRI and 2D MR spectroscopy were performed on a 1.5T Siemens-Avanto scanner. The 2D spectroscopic volume was placed to encompass the basal ganglia, including thalamic nuclei, both insular cortices, and occipital lobes with both grey and white matter. An echo time of 135 was chosen for clean delineation of metabolites and ratios for Cho, NAA and Lac against Cre.
**Results or Findings:** As a neurodegenerative marker, NAA/Cr was significantly ($p < 0.01$-$0.05$) reduced in multiple basal ganglia, insula, and occipital white+grey matter in patients. Furthermore, significantly elevated Lac/Cr-ratios were observed bilaterally in thalami, insula and the occipital lobes. The Cho/Cr ratios were mostly unaffected. The Lac/Cr-ratio in these areas correlated strongly ($R = 0.571^{*}$-$0.873^{**}$) with declined neurocognitive performance.
**Conclusion:** MRS reveals distinct cerebral metabolic disturbances in EPM1 patients indicating neurodegeneration as supported by MRI studies. Previous neurophysiology indicates that EPM1 is also characterised by cerebral motor dysfunction, potentially originating at a modulatory level. Our findings and correlations in this spectroscopy study clearly lend themselves to this idea, helping to delineate future research targets.
**Limitations:** The study population was relatively small, due to the rarity of the disease.
**Ethics committee approval:** This study was approved by the research ethics committee of the hospital district.
**Funding for this study:** Funding was received from Kuopio University Hospital and from the University of Eastern Finland.
**Author Disclosures:**
Esa Mervaala: Nothing to disclose
Reetta Kälviäinen: Nothing to disclose
Juhana Hakumäki: Nothing to disclose
Jelena Hyppönen: Nothing to disclose
Mervi Könönen: Nothing to disclose
Ritva Vanninen: Nothing to disclose
Päivi Koskenkorva: Nothing to disclose

**08:00-09:00** **Research Stage 4**

### Research Presentation Session: Cardiac

### RPS 1703
### Valvular heart disease

**Moderator**
M. Hrabak Paar; Zagreb/HR
*(maja.hrabak.paar@mef.hr)*

### RPS 1703-2
**Mitral valve prolapse associated with mitral annular disjunction - easy to overlook anatomical feature of a potentially fatal disease**
*K. Sienkiewicz*, J. Fijalkowska, K. Dorniak, J. Pienkowska, D. K. Galaska, J. Niemierko, M. Tuzimek, A. Glińska, E. Szurowska; Gdansk/PL

**Purpose:** Mitral annular disjunction (MAD) is a feature of arrhythmogenic mitral valve prolapse (AMVP). MAD is a structural abnormality that manifests as a separation between the mitral valve annulus and the left ventricle. MAD is visible only in systole and undetectable in diastole, therefore it is easy to overlook.
**Methods or Backround:** In order to establish features of MAD and if they correlate with late gadolinium enhancement (LGE), we retrospectively analysed our database from 2018-2022 and found 109 patients in whom MAD was indentified in CMR. The cut-off value for MAD in our study was at 2 mm length of the disjunction zone and its extent along mitral annulus was evaluated in the three typical long axis planes. In all patients LGE was assessed.
**Results or Findings:** The majority of patients were women, 71 (65%) vs 38 (35%) men. Women were younger than men, (41.8 yo vs 49.5 yo, respectively). 95 (87%) of patients had bileaflet MVP. The average length of the MAD was 6.6 mm in the posterior, 6.3 mm in the inferior and 5.6 mm in the lateral wall. In 52 (47%) patients LGE was found in the basal posterior segment, in 16 (30%) LGE in was also present in the posterior papillary muscle and in 4 (3.6%) isolated papillary muscle LGE was noted. No statistically significant correlation between LGE and MAD length, localisation or its extent along mitral annulus was found.
**Conclusion:** In conclusion, MAD was most commonly found in young women with bileaflet MVP and it was the most prominent in the posterior wall. In almost half of the patients LGE was found in the locus that is considered typical of AMVP. No statistically significant correlation between MAD features and LGE was notable
**Limitations:** LGE could be postinfective in some cases.
**Ethics committee approval:** Written consent for MRI was obtained from each subject.
**Funding for this study:** written consent for MRI in each subject
**Author Disclosures:**
Katarzyna Sienkiewicz: Nothing to disclose
Joanna Pienkowska: Nothing to disclose
Jadwiga Fijalkowska: Nothing to disclose
Magdalena Tuzimek: Nothing to disclose
Julia Niemierko: Nothing to disclose
Karolina Dorniak: Nothing to disclose
Edyta Szurowska: Nothing to disclose
Dorota Kulawiak Galaska: Nothing to disclose
Anna Glińska: Nothing to disclose

### RPS 1703-3
**The role of cardiac magnetic resonance imaging with late gadolinium enhancement in predicting arrhythmic risk in mitral valve prolapse: a systematic review and meta-analysis**
*A. Santonocito*, F. P. Papa, D. Tore, M. Visciano, K. Rocco, S. Salto, F. Ullo, R. Faletti, M. Gatti; Turin/IT
*(santonocitoambra@gmail.com)*

**Purpose:** The aim of this study was to evaluate the role of cardiac magnetic resonance imaging with late gadolinium enhancement (LGE-CMR) in arrhythmic risk stratification of patients with mitral valve prolapse (MVP).
**Methods or Backround:** EMBASE, PubMed/MEDLINE, and CENTRAL were searched for studies reporting the impact of LGE-CMR in arrhythmic risk stratification of MVP patiens (distinguish patients with and without complex arrhythmias – cVA and no-cVA). The primary outcome was the presence of cVA. A random effects model was used to analyse, pool, and plot the logarithm of the odds ratio (LogOR) and the diagnostic performance measurements across studies.
**Results or Findings:** Eight studies (870 patients, 130 with LGE) were included in the final analysis. The presence of LGE was strongly associated with cVA (logOR: 2.18, 95%CI: 1.05-3.31). The pooled sensitivity and specificity for LGE in identifying MVP patients with cVA were 74% (95% CI: 48–90%) and 78% (95% CI: 65-87%), whereas the unconditional positive and negative predictive value were 73% (95% CI: 66–81%) and 75 (95% CI: 67–84%). The per-patient analysis revealed a +LR and –LR were 3.4 (95% CI: 2.0–5.6) and 0.34 (95% CI: 0.15 – 0.75), with DOR equal to 10 (95% CI: 3–31) (i.e., with an estimated pre-test probability of cVA of 27%, a positive LGE could increase the post-test probability to 55% and a negative LGE can decrease the post-test probability to 11%). The area under the HSROC curve was 0.83 (95% CI: 0.79–0.86).
**Conclusion:** LGE identifies MVP patients at risk for cVA, albeit with moderate sensitivity, specificity and predictive value.
**Limitations:** Only the LGE value was studied as a predictor of arrhythmia, not the other CMR parameters. Almost all the studies included are retrospective cohort studies and many of them are small in size.
**Ethics committee approval:** No information provided by the submitter.
**Funding for this study:** No funding was received for this study.
**Author Disclosures:**
Sara Salto: Nothing to disclose
Francesco Pio Papa: Nothing to disclose
Marco Gatti: Nothing to disclose
Federica Ullo: Nothing to disclose
Riccardo Faletti: Nothing to disclose
Katia Rocco: Nothing to disclose
Ambra Santonocito: Nothing to disclose
Davide Tore: Nothing to disclose
Michele Visciano: Nothing to disclose

### RPS 1703-4

**Dark-PAP sign: a new prognostic marker in patients with preserved left ventricular ejection fraction**

*C. De Gori*, M. L. Parisella, G. Aringhieri, D. Cioni, L. Faggioni, R. Lencioni, E. Neri, G. D. Aquaro; Pisa/IT
*(degoricarmelo@hotmail.it)*

**Purpose:** We sought to evaluate the prognosis-related role of papillary muscle abnormalities in patients with ventricular arrhythmias, preserved LVEF without a definite diagnosis of cardiac disease.
**Methods or Backround:** 391 patients with >500/24h premature ventricular complexes and/or with non-sustained ventricular tachycardia (NSVT), preserved LVEF and no history of cardiac disease were enrolled. Different features of papillary muscles were considered: supernumerary muscles, papillary thickness, the attachment, late gadolinium enhancement (LGE). Dark-PAP was defined as end-systolic signal hypointensity of both papillary muscles in early post-contrast cine images. Mitral valve prolapse, mitral annular disjunction (MAD) and myocardial LGE were considered.
**Results or Findings:** Dark-PAP sign was found in 79 (20%) of patients (more frequently in females). It was associated with a higher prevalence of mitral valve prolapse and MAD. During a median follow-up of 2534 days, 22 hard cardiac events occurred. At Kaplan-Meier curve analysis patients with Dark-PAP sign were at higher risk of events than those without (p<0.0001). Dark-PAP sign was significantly associated with hard cardiac events in all the multivariate models. Dark-PAP sign improved prognostic estimation when added to NSVT (p=0.0006), to LGE of LV walls (p=0.005) and to a model including NSVT+LGE (p=0.014). Dark-PAP sign allowed a significant net reclassification when added to NSVT (NRI 0.30, p =0.03), to LGE (NRI 0.25, p =0.04), and to NSVT + LGE (NRI 0.32, p =0.02).
**Conclusion:** Dark-PAP sign is a novel prognostic marker in patients with ventricular arrhythmias and preserved ejection fraction.
**Limitations:** Firstly, we performed all the CMR using 1.5T machines and with a high dose of 0.5 molar Gd-based contrast agent (0.2 mmol/Kg) and acquired cine-SSFP immediately after injection. Secondly, first pass perfusion imaging could provide further information on myocardial perfusion of lateral wall and even of papillary muscles.
**Ethics committee approval:** No information provided by the submitter.
**Funding for this study:** Funding for this study was provided through institutional funding by Fondazione G. Monasterio, Pisa, Italy.
**Author Disclosures:**
Maria Luisa Parisella: Nothing to disclose
Emanuele Neri: Nothing to disclose
Giacomo Aringhieri: Nothing to disclose
Carmelo De Gori: Nothing to disclose
Giovanni Donato Aquaro: Nothing to disclose
Riccardo Lencioni: Nothing to disclose
Lorenzo Faggioni: Nothing to disclose
Dania Cioni: Nothing to disclose

### RPS 1703-5

**Fully automated valvular blood flow assessment from 4D flow MRI, including automated cardiac valve tracking and transvalvular velocity mapping**

*T. in de Braekt*[1], J-P. Aben[2], M. Maussen[2], H. van den Bosch[1], P. Houthuizen[1], A. Roest[3], P. van den Boogaard[3], H. J. Lamb[3], J. Westenberg[3]; [1]Eindhoven/NL, [2]Maastricht/NL, [3]Leiden/NL
*(thomas.idb@hotmail.com)*

**Purpose:** Cardiac valvular flow quantification from 4D flow MRI is a valuable diagnostic method for assessing valve function and intracardiac haemodynamics, but analysis involves time-consuming manual valvular segmentation. The purpose was to compare 4D flow MRI with automated valve tracking and novel automated segmentation to manual valve segmentation in patients with corrected atrioventricular septum defect (AVSD) and healthy volunteers.
**Methods or Backround:** Data was retrospectively collected from 8 patients with corrected AVSD (mean age: 20 ± 8 years) and 23 healthy volunteers (mean age: 26 ± 14 years). Free breathing whole-heart 4D flow MRI was acquired at 3T without respiratory gating. Fully manual, fully automated and manually corrected automated segmentation of all cardiac valves was performed using CAAS MR Solutions (Pie Medical Imaging). Net forward volumes (NFVs) were calculated for all valves. Analysis times and NFVs were compared by Wilcoxon signed-rank test. NFV variation was calculated as the mean NFV of all valves over the standard deviation of differences among all four valves. Interobserver variability was tested by intraclass correlation coefficients (ICCs).
**Results or Findings:** Interobserver agreement for fully automated versus fully manual segmentation was excellent for NFV (ICC ≥ 0.97). No differences in NFV variation over cardiac valves was observed for fully automated versus fully manual segmentation (10.4% [IQR, 7.3%-16.0%] vs 9.6% [IQR, 6.9% - 12.9%], respectively; p = 0.164). Analysis time for all subjects was shorter for manually corrected automated versus fully manual segmentation (4.1 minutes [IQR, 2.9 - 4.9 minutes] vs 9.4 minutes [IQR, 7.8 - 10.5 minutes], respectively; p < 0.001).
**Conclusion:** Fully automated segmentation does not compromise quantification of NFV in patients with corrected AVSD and healthy volunteers. Valvular flow quantification with 4D flow MRI benefits from automated valve segmentation by reducing post-processing analysis time.
**Limitations:** Not applicable
**Ethics committee approval:** Not applicable
**Funding for this study:** Not applicable
**Author Disclosures:**
Arno Roest: Nothing to disclose
Marc Maussen: Nothing to disclose
Pieter van den Boogaard: Nothing to disclose
Jos Westenberg: Nothing to disclose
Jean-Paul Aben: Nothing to disclose
Thomas in de Braekt: Nothing to disclose
Hildo J. Lamb: Nothing to disclose
Patrick Houthuizen: Nothing to disclose
Harrie van den Bosch: Nothing to disclose

### RPS 1703-6

**Comparison of non-enhanced flow evaluation in MRI using 4D flow sequence versus 2D flow sequence in repaired Tetralogy of Fallot patients**

*J. Champollion*, K. Warin-Fresse; Nantes/FR
*(radiologiechampollion@gmail.com)*

**Purpose:** 4D flow analysis has already been evaluated as a reliable way of flow quantification in repaired Tetralogy of Fallot (rTOF) patients follow-up, but thus has been mostly done under the use of contrast agents or without clear information about its use. We aim to evaluate the reliability of quantitative 4D flow analysis in rTOF patients without the use of contrast agents, compared to classic 2D flow.
**Methods or Backround:** 24 patients were included. Routine cardiac MRI in those patients included 4D flow and 2D flow. Data analysis involved quantitative measurement of flow in aorta and pulmonary valve on both 4D and 2D flux sequences. Additionally, ventricular volumes were measured to test internal consistency with indirect regurgitation evaluation. Pulmonary and aortic regurgitant fraction has been calculated and used for statistical analysis.
**Results or Findings:** Good agreement has been shown between 4D flow regurgitant fraction and 2D flow direct regurgitant fraction for the aortic (r=0.93, p < 0.0001) and the pulmonary (r = 0.93, p < 0.0001) method.
**Conclusion:** Non-enhanced 4D flow MRI seems to be a reliable method of quantitative flow in rTOF patients. 4D-MR is presented as a precise tool of cardiac exploration, and should lead to a time gain compared with classic 2D sequences. Since no contrast agent is used, long-term gadolinium brain deposits could be avoided.
**Limitations:** Few patients were included and the study was done in a restrospective manner.
**Ethics committee approval:** No information provided by the submitter.
**Funding for this study:** No funding was received for this study.
**Author Disclosures:**
Karine Warin-Fresse: Nothing to disclose
Jean Champollion: Nothing to disclose

### RPS 1703-7

**Aliased flow signal planimetry by cardiovascular magnetic resonance imaging for grading aortic stenosis severity: a prospective pilot study**

*V. Di Mascio*[1], C. Mantini[1], M. Olivieri[1], F. Ricci[2], M. Caulo[1]; [1]Pescara/IT, [2]Chieti/IT
*(valeria.dimascio@virgilio.it)*

**Purpose:** We tested the diagnostic accuracy and precision of aliased orifice area planimetry (AOAcmr), a new, simple, non-invasive technique for grading of AS (aortic stenosis) severity by low-VENC phase-contrast cardiovascular magnetic resonance (CMR) imaging.
**Methods or Backround:** Twenty-two consecutive patients with mild, moderate or severe AS and 6 age- and sex-matched healthy controls had TTE and CMR examinations on the same day. We performed analysis of agreement and correlation among (i) AOAcmr, (ii) geometric orifice area (GOAcmr) by direct CMR planimetry, (iii) EOAecho by TTE-continuity equation, and (iv) the 'gold standard' multimodality EOA (EOAhybrid) obtained by substituting CMR LVOT area into Doppler continuity equation.
**Results or Findings:** There was excellent pairwise positive linear correlation among AOAcmr, EOAhybrid, GOAcmr and EOAecho (p<0.001); AOAcmr had the highest correlation with EOAhybrid ($R2 = 0.985$, p<0.001). There was good agreement between methods, with the lowest bias (0.019) for the comparison between AOAcmr and EOAhybrid. AOAcmr yielded excellent intra- and interrater reliability (intraclass correlation coefficient: 0.997 and 0.998, respectively).
**Conclusion:** Aliased orifice area planimetry by 2D phase contrast imaging is a simple, reproducible, accurate 'one-stop shop' CMR method for grading AS,

Saturday potentially useful when echocardiographic severity assessment is inconclusive or discordant. Larger studies are warranted to confirm and validate these promising preliminary results.
**Limitations:** The study population was small.
**Ethics committee approval:** No information provided by the submitter.
**Funding for this study:** No funding was received for this work.
**Author Disclosures:**
Cesare Mantini: Nothing to disclose
Valeria Di Mascio: Nothing to disclose
Marzia Olivieri: Nothing to disclose
Massimo Caulo: Nothing to disclose
Fabrizio Ricci: Nothing to disclose

### RPS 1703-8

**Ultra-high resolution and K-edge imaging of prosthetic heart valve with spectral photon counting CT: a phantom study**

*S. Boccalini*[1], C. Mayard[1], H. Lacombe[2], M. Villien[3], S. A. Si-Mohamed[1], M. Pozzi[1], P. Douek[1]; [1]Bron/FR, [2]Lyon/FR, [3]Suresnes/FR
*(sara.boccalini@yahoo.com)*

**Purpose:** To compare cardiac prosthetic valves image quality on Spectral Photon Counting CT (SPCCT) and dual-energy CT (DECT).
**Methods or Backround:** Two prosthetic metallic valves (SJM™ 25 mm, 17 mm) and three biological valves (Trifecta 23 mm, 25 mm, Epic™ 27 mm) placed in a tube containing iodine contrast (~350 HU) inside a thoracic phantom were scanned on a DECT and SPCCT (x3 each). Four valves were scanned also with two simulated valvular lesions (< 2 mm), with and without an outer extension ring of the thoracic phantom. SJM-25, SJM-17 were scanned again with parameters adapted for Tungsten-K-edge imaging. Thickness and blooming artefacts of all metallic parts were calculated. The voxels with densities <3xSD of the contrast media in volumetric ROIs were calculated as an estimate of dark streak artefacts. Conspicuity and sharpness of the different parts of the valves and the lesions were subjectively scored by three readers on ultra-high resolution conventional images and Tungsten-K-edge images.
**Results or Findings:** With SPCCT, metallic structures were thinner, with less blooming and dark streak artefacts (11 (IQ=6) vs 40 (IQ=13)% of voxels, p<0.01). On subjective analysis, some structures were visible or clearly visible only with SPCCT. SPCCT yielded better scores for all parts except for the leaflets of biological valves, hardly visible anyhow. Also, the lesions were easier to identify and sharper with SPCCT (p<0.02), although scores remained low (2 (IQ=2) and 2(IQ=2)). Adding the extension ring resulted in reduced scores. As expected, only the leaflets were visible on Tungsten-K-edge imaging and they had less blooming and metallic artefact as compared to conventional SPPCT (p<0.01).
**Conclusion:** With SPCCT objective and subjective image quality prosthetic cardiac valves is improved. Nevertheless, millimetric lesions remain a challenge. Tungsten-K-edge imaging allowed for an even greater reduction of artefacts.
**Limitations:** These were static phantom experiments.
**Ethics committee approval:** No information provided by the submitter.
**Funding for this study:** Funding was received from Hospices Civils de Lyon.
**Author Disclosures:**
Marjorie Villien: Employee: Philips
Salim Aymeric Si-Mohamed: Nothing to disclose
Matteo Pozzi: Nothing to disclose
Philippe Douek: Nothing to disclose
Hugo Lacombe: Research/Grant Support: Philips
Sara Boccalini: Nothing to disclose
Charles Mayard: Nothing to disclose

### RPS 1703-9

**Deep learning-based prediction of tricuspid regurgitation from routine contrast-enhanced CT chest**

*L. D. Hahn*, M. D. LeComte, J. H. Kim, D. Gupta, M. E. Bodenstein, P. Kim, E. Weihe, A. Hsiao; San Diego, CA/US
*(ldhahn@gmail.com)*

**Purpose:** Moderate to severe tricuspid regurgitation is associated with increased cardiac mortality. Most cases of tricuspid regurgitation are secondary to conditions that cause right atrial/ventricular enlargement and tricuspid annular dilation, features which are commonly observed on CT. We hypothesised that a deep learning algorithm may be able to identify patients with moderate to severe tricuspid regurgitation based on routine CT or CTA of the chest.
**Methods or Backround:** We retrospectively identified 1043 unique patients who underwent routine (non-ECG-gated) contrast-enhanced CT or CTA chest and echocardiography within one day between 2016 and 2022. 225 patients (21.6%) had at least moderate tricuspid regurgitation. We developed a deep learning algorithm leveraging the encoding layers of a previously developed segmentation algorithm that segments the four cardiac chambers. 693 studies were used for training, 100 for validation, and 250 as an independent test set.
**Results or Findings:** The algorithm demonstrated a sensitivity of 0.92 and specificity of 0.80 for the validation set. Sensitivity was 0.93 and specificity of 0.89 was for the test set. Area under the receiver operating characteristic curve (AUROC), varying the algorithm probability threshold for tricuspid regurgitation, was 0.93 for the validation set and 0.96 for the test set. By comparison, the AUROC for the test set was 0.85 using a cut-off for right atrial volume, 0.79 for tricuspid annulus size, and 0.79 for right ventricular volume.
**Conclusion:** A deep learning algorithm, trained with the encoding layers of a segmentation algorithm, can accurately identify the presence of moderate to severe tricuspid regurgitation on chest CT more effectively than chamber segmentation. Application of such an algorithm may help improve detection of patients with clinically significant tricuspid regurgitation.
**Limitations:** The main limitation was the single institution, retrospective study design.
**Ethics committee approval:** This study constitutes NIH exempt human subjects research (category 4: secondary collection of data).
**Funding for this study:** No funding was received for this study.
**Author Disclosures:**
Lewis Dirk Hahn: Grant Recipient: Radiological Society of North America
Elizabeth Weihe: Nothing to disclose
Paul Kim: Research/Grant Support: Circle CVI Research/Grant Support: General Electric
Mark Eugene Bodenstein: Nothing to disclose
Jessica H Kim: Nothing to disclose
Diviya Gupta: Nothing to disclose
Albert Hsiao: Research/Grant Support: Bayer AG Research/Grant Support: GE Healthcare Founder: Arterys
Matthew David LeComte: Nothing to disclose

**10:30-12:00 Research Stage 1**

### Research Presentation Session: Artificial Intelligence & Machine Learning & Imaging Informatics

### RPS 1805

### Recent developments and implementations strategies for AI

**Moderator**
P. M. A. van Ooijen; Groningen/NL
*(p.m.a.van.ooijen@umcg.nl)*
**Author Disclosures:**
Peter M. A. Van Ooijen: Advisory Board: MedicalPHIT, ContextFlow; Board Member: EuSoMII; Speaker: Novartis, Bayer, Bracco, Siemens

### RPS 1805-2

**Democratising AI: specialty society support for multicentre research and model validation in Artificial Intelligence**

*B. Allen*[1], K. Dreyer[2], K. Schmidt[3], L. Brink[3], C. Wald[4]; [1]Birmingham, AL/US, [2]Boston, MA/US, [3]Reston, VA/US, [4]Nahant, MA/US
*(bibb@mac.com)*

**Purpose:** Processes developed at the American College of Radiology (ACR) support collaborations in artificial intelligence (AI) research across multiple institutions using federated learning (FL) techniques and multicentre AI model validation. Initial results indicate medical specialty societies can facilitate these activities with improved model performance and generalisability, and mitigation of unintended bias.
**Methods or Backround:** Widespread clinical deployment of AI algorithms developed at single institutions or without multisite validation may be subject to unintended bias and/or a lack of generalisability of performance. Furthermore, many commercial AI products have been cleared for clinical use by regulatory agencies without multisite validation. Robust multicentre AI development and validation of AI software may mitigate these challenges but are not routinely performed. Newly developed ACR processes/infrastructure support a diverse variety of institutions in AI research using distributed data solutions. The development of hybrid central/distributed research data registries and applications within the ACR's AI-LAB enable multicentre AI training (FL) and validation without a need for extensive local data science capabilities at the participating sites.
**Results or Findings:** Although FL requires attention to data preparation, which can be challenging particularly for smaller institutions and/or those without robust data science resources, the results of our initial pilots using FL and distributed validation across a variety of institution types are similar to other federated learning studies in the literature. We outline ways to mitigate limitations and demonstrate that, with specialty society support, any institution can meaningfully participate in AI research and development.
**Conclusion:** Medical specialty societies such as ACR can support FL and distributed validation allowing a diverse variety of institutions to meaningfully participate in AI research and development thereby mitigating bias and improving generalisability in AI models.
**Limitations:** Entities other than medical specialty societies could provide similar services.
**Ethics committee approval:** No information provided by the submitter.
**Funding for this study:** No funding was received for this study.
**Author Disclosures:**
Christoph Wald: Other: Chair ACR Commission On Informatics
Kendall Schmidt: Nothing to disclose
Laura Brink: Nothing to disclose
Bibb Allen: Other: Chief Medical Officer ACR Data Science Institute
Keith Dreyer: Other: Chief Science Officer ACR Data Science Institute

### RPS 1805-3

**AI-based method for detection of mislabelled CT studies in clinical trials**
*L. Edenbrandt*, T. Gillberg, O. Enqvist; Malmö/SE
*(lars.edenbrandt@gmail.com)*

**Purpose:** In clinical trials, diagnostic images are commonly de-identified and uploaded to a central repository for further analysis. The manual step of replacing the patient identification number with the correct trial identification number is critical. Mislabelling studies may lead to false study results. The purpose was to develop an automated artificial intelligence (AI)-based method to detect if pairs of de-identified CT studies have been obtained from the same patient or not.
**Methods or Backround:** The first step of the AI-based method is to segment bones in the two CT studies to be compared using Organ Finder (SliceVault AB, Malmö, Sweden). The SurfaceNets method is used to extract meshes describing the bone surfaces. The meshes from the two studies are aligned using the Iterative Closest Point algorithm (ICP) to put them in a common coordinate system. The percentage of vertices in the smaller mesh with residuals below a predefined threshold is used as a measure of similarity. Two CT studies are classified as being obtained from the same patient if this percentage is at least 95% in three of the six hip, scapulae, and clavicle bones. The AI-based method was applied to CT studies covering the torso and obtained from patients with lymphoma. Each CT study was compared to all the other studies.
**Results or Findings:** All the 49 pairs of CT from the same patients as well as all the 2.096 pairs of CT from different patients were correctly classified by the AI-based method.
**Conclusion:** Mislabelled CT studies in clinical trials can be detected using an AI-based method, which compares the shape of bone structures.
**Limitations:** The present version of Organ Finder cannot segment separate vertebrae, but when that is included the AI-based method can be updated.
**Ethics committee approval:** No information provided by the submitter.
**Funding for this study:** No funding was received for this study.
**Author Disclosures:**
Olof Enqvist: Nothing to disclose
Tony Gillberg: Nothing to disclose
Lars Edenbrandt: Nothing to disclose

### RPS 1805-4

**Potential risk of off-label use of commercially available AI-based software for radiology**
*K. G. van Leeuwen*[1], D. M. Hedderich[2], S. Schalekamp[1]; [1]Nijmegen/NL, [2]Munich/DE
*(kicky.vanleeuwen@radboudumc.nl)*

**Purpose:** The aim of this study was to analyse potential discrepancies between the claims and disclaimers of the intended purpose statements of CE-marked AI-based software for radiology.
**Methods or Backround:** In March 2022, we asked all vendors listed on www.AIforRadiology.com (n=87) to verify or submit the intended purpose according to European clearance for their products (n=191). Any new additions were included until September 26th 2022 (n=12)). Claims and disclaimers were extracted from the statements. Potential conflicts of claims and disclaimers were flagged.
**Results or Findings:** We received the intended purpose statements for 157 of the 203 products. Of those, 36 were excluded as they provided too little information to analyse. The included products were certified under the current medical device regulations (class IIa = 24, class IIb = 9) and former Medical Device Directive (class I = 45, class IIa = 39, class IIb = 3). Of the 121 included statements 56 held disclaimers. For 13 of these products the claims and disclaimers were flagged to contradict each other. Potential discrepant disclaimer statements were e.g. 'act per the standard of care' (n=7) and 'not for diagnostic use' (n=6), while claiming to aid in the diagnosis, triaging or risk scoring of clinical conditions.
**Conclusion:** Potential discrepancies in claims and disclaimers were found for a substantial number of AI-tools bearing the risk that users of the AI software misunderstand the permitted use-cases which may lead to off-label use.
**Limitations:** Not all intended purpose statements received were of sufficient quality to use for analysis. The definition of what information the intended purpose should contain is not clearly specified under the MDR making it hard to objectively assess or compare.
**Ethics committee approval:** No information provided by the submitter.
**Funding for this study:** No funding was received for this study.
**Author Disclosures:**
Kicky Gerhilde van Leeuwen: Employee: Scarlet
Steven Schalekamp: Nothing to disclose
Dennis M Hedderich: Nothing to disclose

### RPS 1805-5

**Leveraging the Clinical Production PACS Platform for AI algorithm evaluation and retraining – preliminary work**
*P. Korfiatis*[1], Z. Kelm[1], A. Chan[1], T. Kline[1], M. Lin[2], W. Holler[3], N. Moily[2], R. Hartman[1]; [1]Rochester, AL/US, [2]San Diego, CA/US, [3]Berlin/DE

**Purpose:** To enable translation of the algorithms into clinical production and support their adoption, it is important to assess the algorithm's impact on the real-world workflow. For algorithms performing quantification, the time burden of algorithm output correction is an important component of the algorithm's utility. When this correction involves radiologist interaction, this time burden can be minimised by using the clinical viewer and tools to which they are accustomed. In this study, we focused on segmentation of hepatic metastases.
**Methods or Backround:** A pipeline (Figure 1) was created incorporating clinical image viewing tools for evaluating the performance of an AI algorithm. During the first step, the algorithm would query and retrieve appropriate imaging based on the IRB number of the study. The imaging dataset will include preliminary liver tumour segmentations. Following this step, the exams were assigned to the participating radiologists. A custom view was created to allow access to the assigned cases. During the segmentation evaluation, the radiologist used the image analysis toolset that is natively available in the clinical viewer to revise the AI-generated segmentation and a FHIR questionnaire to capture feedback and record time spent (Figure 2).
**Results or Findings:** The validation pipeline was applied to a cohort of 60 patients. The Dice recorded was 0.79 (0.13). Capturing the effect of accuracy and the time penalty for correction of the AI output during algorithm validation can inform algorithm design, including AI retraining. This can be crucial in radiology workflows, where the time required to interact with the AI output can be a factor that affects utilisation (figure 3).
**Conclusion:** The proposed approach can capture the real-life performance and impact of AI algorithm in clinical workflows.
**Limitations:** Further research should involve a larger scale evaluation, applied in more model tasks.
**Ethics committee approval:** This research was approved by the IRB.
**Funding for this study:** No funding was received for this study.
**Author Disclosures:**
Alex Chan: Nothing to disclose
Wolfgang Holler: Employee: Visage
Panagiotis Korfiatis: Nothing to disclose
MingDe Lin: Employee: Visage
Nagaraj Moily: Employee: Visage
Zachary Kelm: Nothing to disclose
Robert Hartman: Nothing to disclose
Timothy Kline: Nothing to disclose

### RPS 1805-6

**Lifelong nnU-Net: a framework for standardised medical continual learning**
*C. Gonzalez*[1], A. Ranem[1], D. Pinto dos Santos[2], A. Othman[3], A. Mukhopadhyay[1]; [1]Darmstadt/DE, [2]Cologne/DE, [3]Mainz/DE
*(camila.gonzalez@gris.informatik.tu-darmstadt.de)*

**Purpose:** Most research and product development in medical AI focuses on designing static systems for a dynamic clinical world. This often leads to abrupt drops in performance post-deployment as acquisition standards and patient populations change. Continual learning is becoming a viable alternative and raising the interest of medical image researchers and regulatory bodies alike.
**Methods or Backround:** We present Lifelong nnU-Net, an open-source framework that places state-of-the-art continual segmentation at the hands of researchers and clinicians. Equipped with all necessary modules for training and testing continual models, we lower the barrier for evaluating new solutions in a continual fashion. Five continual learning methods are currently implemented: rehearsal, EWC, LwF, Riemannian Walk and MiB.
**Results or Findings:** We evaluate all methods for the use cases of prostate and hippocampus segmentation in MRIs. For each anatomy, we train a patch-based 3D nnU-Net for 250 epochs per dataset in a system with NVIDIA Tesla T4 GPUs. Rehearsal training performs best, maintaining a Dice of at least 85.94±0.76% for prostate and 88.17±3.63% for hippocampus. EWC is a good alternative, reliably reducing the amount of forgetting, though this comes at the cost of a loss of model plasticity. While the sequential model has a Dice of 91.91 ±0.38% in the last prostate task, it decreases to 87.79±0.83% for EWC. For hippocampus, this behavior is more pronounced, with the Dice of the last task falling from 90.92±1.08% to 31.93±6.09%.
**Conclusion:** With Lifelong nnU-Net, we present a first reproducible benchmark for accelerating continual learning research in medical applications.
**Limitations:** Particularly when rehearsal training is not a viable option, it is currently challenging to prevent forgetting without reducing model plasticity.
**Ethics committee approval:** No information provided by the submitter.
**Funding for this study:** This work was supported by the German Bundesministerium für Gesundheit (BMG) with grant EVA-KI [ZMVI1-727 2520DAT03A].
**Author Disclosures:**
Daniel Pinto dos Santos: Consultant: Cook Medical Consultant: Amboss GmbH
Camila Gonzalez: Nothing to disclose
Amin Ranem: Nothing to disclose
Ahmed Othman: Nothing to disclose
Anirban Mukhopadhyay: Nothing to disclose

### RPS 1805-7

**Evaluation of a generic registration algorithm for lesion matching in follow-up CT**

*J. H. Moltz*[1], S. Heldmann[2], A. Hering[3]; [1]Bremen/DE, [2]Lübeck/DE, [3]Nijmegen/NL
*(jan.moltz@mevis.fraunhofer.de)*

**Purpose:** Assessing therapy response and tumour progression based on CT images is a significant part of radiological routine work. Software support that facilitates image comparison may speed up reading and avoid mistakes. This study evaluates the accuracy of a general-purpose whole-body image registration algorithm to match lesions in follow-up CT pairs, using a public dataset.
**Methods or Backround:** The registration algorithm was developed independently of the purpose of this study and the used dataset. It uses a three-step approach: (1) a translational alignment, (2) a rigid registration to refine the alignment and account for major changes in patient position or anatomy, and (3) a deformable registration to find a locally accurate mapping of anatomical landmarks such as lesions. We used the deep longitudinal study dataset, which defines 7776 follow-up lesion pairs within the DeepLesion dataset. For each pair, we applied the registration to the prior lesion centre and compared the result to the given current lesion centre. As no segmentations are available, we used the long and short axis diameters to estimate whether the result is within the lesion.
**Results or Findings:** The median distance between predicted and given lesion centre was 3.1 mm (IQR 1.8 - 5.4 mm). The distance was below the long and short axis radius in 92% and 86%, respectively. Registration accuracy was highest in the lungs and pelvis and lowest in kidney and bones.
**Conclusion:** Our generic registration algorithm is able to locate corresponding lesions with high accuracy. It can be directly used for cursor synchronisation in viewers, making image comparison more efficient. It is also a good basis for automating lesion measurements in follow-up or detecting and highlighting change.
**Limitations:** CTs were cropped 30 mm above and below a lesion. Registration may perform differently on full CTs.
**Ethics committee approval:** No information provided by the submitter.
**Funding for this study:** No funding was received for this study.
**Author Disclosures:**
Alessa Hering: Nothing to disclose
Stefan Heldmann: Nothing to disclose
Jan Hendrik Moltz: Nothing to disclose

### RPS 1805-8

**Improving scalability of semi-automated medical segmentations using uncertainty estimations**

*L. M. Nunez*, M. Visentin, Z. Arya, J. Wade, C. Nucifor, J. Connell, M. Kelly; Oxford/UK
*(luis.nunez@perspectum.com)*

**Purpose:** Automated organ segmentations typically require time-consuming manual review and editing if they are to be used for clinical decision-making. Here we evaluate the utility of uncertainty estimation from a U-Net liver segmentation to confine editing to regions at high risk of error and its impact on case review time.
**Methods or Backround:** Using a dataset of 425 liver MRIs, spanning 1.5T and 3T Siemens, GE, and Philips scanners, a Monte Carlo Dropout U-Net was trained to output both the liver segmentation mask and the spatial uncertainty map. Patches (30x30 pixels regions) with an average uncertainty above 0.4 in the map were considered at high risk of error in the segmentation mask. To test the ability of the patches to flag segmentation errors, 10 automated segmentations were edited by two operators and the review time for each case was recorded. After a week, the blinded operators were asked to edit the same 10 cases, but only in the regions flagged by the patches.
**Results or Findings:** The average Dice score between the ground truths and the 10 automated segmentations corrected with patches versus without was 0.9661±0.0097 and 0.9660±0092, respectively. The flagged patches covered 97% of the area edited by both operators when patches were not used. The patch-based approach reduced the time needed for manual edits by 34% (p=0.002) from 7.2 min/case to 4.8 min/case.
**Conclusion:** We showed uncertainty estimates could be used to flag regions with a high risk of error in automated medical segmentations and reduce the time needed for manual correction while maintaining high-quality segmentations. This approach is important for optimising a robust medical imaging segmentation service.
**Limitations:** Further investigation is needed on a more representative sample as the current one is too small.
**Ethics committee approval:** No information provided by the submitter.
**Funding for this study:** Not applicable
**Author Disclosures:**
John Connell: Employee: Perspectum Ltd.
Luis Miguel Nunez: Employee: Perspectum Ltd.
Marco Visentin: Employee: Perspectum Ltd.
Charlie Nucifor: Employee: Perspectum Ltd.
Matt Kelly: Employee: Perspectum Ltd.
Joe Wade: Employee: Perspectum Ltd.
Zobair Arya: Employee: Perspectum Ltd.

### RPS 1805-9

**Semi-supervised learning method for abdominal multi-organ segmentation**

*W. Xu*, C. Chen, S. Wang, R. Zhang; Beijing/CN
*(xweixin@infervision.com)*

**Purpose:** This study aims to build a DL-based model with a small amount of annotated training data to segment 13 abdominal organs effectively.
**Methods or Backround:** In this study, 2300 cases (300 cases with a pix-level mask containing 13 abdominal organs) from more than 20 medical groups are used. Among them, 2050 cases (50 labelled cases and 2000 unlabelled cases) are used for the training, 50 annotated cases are used for the validation and 200 annotated cases are used for the final test. Our proposed method is a whole-volume-based coarse-to-fine framework. In this framework, two 3DResUNet networks are cascaded to realise the relocation of small organs (left and right adrenal glands, gallbladder, and oesophagus) more precisely. And 9 large organs (liver, left and right kidneys, spleen, stomach, aorta, inferior vena cava, pancreas, and duodenum) are divided into 3 groups for fine segmentation since that relative position information can be captured. Moreover, we incorporate a pseudo-labelling learning approach on 2000 unlabelled cases to improve the model's performance.
**Results or Findings:** For the validation phase, the mean Dice Similarity Coefficient (DSC) and Normalised Surface Dice (NSD) values of 13 abdominal organs segmentation are 0.8767 and 0.9316, respectively. And the average running time of every case is 19.56 seconds, the max GPU memory is 2657 MB. Similarly, for the test phase, the mean DSC and NSD values are 0.8774 and 0.9358, respectively.
**Conclusion:** These experimental results show that our proposed DL-based model could effectively realise abdominal multi-organ segmentation with few annotated data and has the potential to support radiologists as a CAD system.
**Limitations:** All of the data used in this study are collected from public datasets and the model will be tested on real clinical datasets from hospitals.
**Ethics committee approval:** No information provided by the submitter.
**Funding for this study:** Not applicable
**Author Disclosures:**
Weixin Xu: Nothing to disclose
Cancan Chen: Nothing to disclose
Rongguo Zhang: Nothing to disclose
Shaokang Wang: Nothing to disclose

### RPS 1805-10

**A study in contrasts: validating the optimal MRI contrast phase for deep learning liver segmentation**

*M. Gross*[1], S. Huber[2], S. Arora[2], T. Ze'evi[2], S. Haider[3], A. S. Kücükkaya[1], M. Strazzabosco[2], J. Chapiro[2], J. A. Onofrey[2]; [1]Berlin/DE, [2]New Haven, CT/US, [3]Munich/DE
*(moritz.gross@charite.de)*

**Purpose:** In recent years different automated liver segmentation methods for magnetic resonance imaging (MRI) have been proposed using different contrast phases and their combinations. The aim of this head-to-head comparison was to validate the optimal contrast phase for deep learning liver segmentation on multiphasic contrast-enhanced MRI.
**Methods or Backround:** This study included 586 multiphasic contrast-enhanced MRI series with pre-contrast, arterial-, portal-venous, and delayed phase sequences. Imaging was acquired using a standard institutional imaging protocol. All images were co-registered and livers were manually segmented. Fully convolutional neural networks were trained to automatically segment the liver using the same 3D U-Net-derived architecture on single contrast phases (Pre-Net, Arterial-Net, Portal-Venous-Net, Delayed-Net) and their possible multi-phasic combinations (six 2-Phase-Nets, four 3-Phase-Nets, and one 4-Phase-Net) resulting in a total of 15 models. The dataset was randomly split into 70/15/15% for training/validation/testing sets and segmentation accuracy was quantified by the Dice Similarity Coefficient (DSC). A Friedmann's test was used for pairwise performance comparisons between the developed segmentation models.
**Results or Findings:** The portal-venous-net attained the highest DSC in the test set (mean±SD: 0.970±0.017) and significantly outperformed the other segmentation models (<0.001 for all segmentation models except the 2-Phase-Net trained on the combination of arterial- and portal-venous phase: 0.966±0.025, p=0.1372). The mean runtime for automated segmentation was 0.35 (±0.16) seconds.
**Conclusion:** This study demonstrated that the portal-venous phase is the optimal contrast phase for deep learning liver segmentation on contrast-enhanced MRI. The portal-venous-net yielded state-of-the-art liver segmentation performance and fast processing times required for clinical practice. Furthermore, no extensive image preprocessing such as co-registration was required and artefacts on other imaging sequences did not affect segmentation performance.
**Limitations:** We evaluated T1-weighted contrast-enhanced MRI sequences. Future work will include other imaging sequences.
**Ethics committee approval:** No information provided by the submitter.
**Funding for this study:** Funding was received through from the NIH (Grant P30 KD034989).
**Author Disclosures:**
Julius Chapiro: Nothing to disclose
Stefan Haider: Nothing to disclose
Steffen Huber: Nothing to disclose
Sandeep Arora: Nothing to disclose
Mario Strazzabosco: Nothing to disclose
Ahmet Said Kücükkaya: Nothing to disclose
Tal Ze'evi: Nothing to disclose
John Aaron Onofrey: Nothing to disclose
Moritz Gross: Nothing to disclose

### RPS 1805-11

**Fully automated liver segmentation for liver volumetry and extraction of imaging biomarkers: a study with internal and external validation**

*M. Gross*[1], S. Huber[2], S. Arora[2], S. Haider[3], F. Michallek[1], V. Vilgrain[4], M. Dewey[1], J. Chapiro[2], J. A. Onofrey[2]; [1]Berlin/DE, [2]New Haven, CT/US, [3]Munich/DE, [4]Clichy/FR
*(moritz.gross@charite.de)*

**Purpose:** The purpose of this study was to develop and evaluate a deep convolutional neural network (DCNN) for automated liver segmentation, volumetry, and radiomic feature extraction using portal-venous phase contrast-enhanced magnetic resonance imaging (MRI).
**Methods or Backround:** This study included 470 patients from an institutional hepatocellular carcinoma database with T1-weighted portal-venous MRI. For model development and evaluation, the data was randomly split into 70/15/15% for training, validation, and internal testing sets, respectively. From a transatlantic collaborative institution, 20 de-identified scans were made available for external testing. A 3D-DCNN based on the U-Net was trained to automatically segment the liver using manual segmentations as ground truth. Segmentation accuracy was quantified by the Dice Similarity Coefficient (DSC). Significance between the internal and external test sets was assessed by the Mann-Whitney U test. Liver volumes were calculated from the segmentations. Agreement of liver volumetry and extracted radiomic features between the manual and automated segmentations was assessed by the intraclass correlation coefficient (ICC).
**Results or Findings:** Segmentation accuracy (mean±SD) of the 3D-DCNN was similarly high in the internal (0.97±0.2) and external (0.96±0.03) test set (p=0.25). Agreement [95% CI] (ICC significance) of liver volumetry was 0.99 [1.0, 1.0] (p<0.0001) and 0.97 [0.93, 0.99] (p<0.0001) in the internal and external test set, respectively. Radiomic features derived from automated liver segmentations demonstrated an ICC (mean±SD) of 0.98±0.04 (range: 0.80-1.00) for the internal test set and 0.94±0.10 (range: 0.16-1.00) for the external test set compared to manual segmentations.
**Conclusion:** Automated liver segmentation demonstrates robust and generalisable segmentation and volumetry performance on both internal and external MRI data and can be used for reproducible radiomic feature extraction.
**Limitations:** The proposed liver segmentation method relies on contrast-enhanced MRI and DSC does not capture the relevance of inaccurate segmentations.
**Ethics committee approval:** No information provided by the submitter.
**Funding for this study:** Funding was received via the NIH (Grant P30 KD034989).
**Author Disclosures:**
Julius Chapiro: Nothing to disclose
Stefan Haider: Nothing to disclose
Steffen Huber: Nothing to disclose
Sandeep Arora: Nothing to disclose
Marc Dewey: Nothing to disclose
Florian Michallek: Nothing to disclose
Valérie Vilgrain: Nothing to disclose
John Aaron Onofrey: Nothing to disclose
Moritz Gross: Nothing to disclose

### RPS 1805-12

**Organ Finder - a new AI-based organ segmentation tool for CT**

*L. Edenbrandt*[1], O. Enqvist[1], M. Larsson[1], J. Ulén[1], E. Trägårdh[2]; [1]Malmö/SE, [2]Lund/SE
*(lars.edenbrandt@gmail.com)*

**Purpose:** Automated organ segmentation in computed tomography (CT) has proved to be a vital component in many artificial intelligence-based tools for image analysis. A limitation with a recently presented organ segmentation tool (RECOMIA) is that only CT without contrast was used during training. The aim of this study was to develop a new tool (Organ Finder 2.0) for organ segmentation in CT using both non-contrast and contrast studies.
**Methods or Backround:** A total of 1.171 CT studies from seven different publicly available CT databases were retrospectively included. Twenty CT-studies were used as test set and the remaining 1.151 were used to train a convolutional neural network. 22 different organs were studied. Professional annotators segmented a total of 5.826 organs and segmentation quality was assured manually for each of these organs.
**Results or Findings:** Organ Finder showed high agreement with manual segmentations in the test set. The average Dice index over all organs was 0.94 and the same high performance was found for four different subgroups of the test set based on presence or absence of intravenous and oral contrast. The performance of Organ Finder was significantly better than that of RECOMIA.
**Conclusion:** An AI-based tool can be used to accurately segment organs in both contrast and non-contrast CT studies. The results indicate that the size of a training set in the order of a thousand examples and high-quality manual segmentations should be used to handle common variations in the appearance of a CT in clinical routine.
**Limitations:** A limitation with Organ Finder is that only 22 organs are included. Future work will include adding soft tissue organs, for example, the gastrointestinal tract, heart, muscle, and prostate.
**Ethics committee approval:** No information provided by the submitter.
**Funding for this study:** No funding was received for this study.
**Author Disclosures:**
Måns Larsson: Nothing to disclose
Elin Trägårdh: Nothing to disclose
Olof Enqvist: Nothing to disclose
Johannes Ulén: Nothing to disclose
Lars Edenbrandt: Nothing to disclose

### RPS 1805-13

**Validation of a deep learning neuroblastoma detection and segmentation tool in MR images**

*D. Veiga Canuto*, L. Cerdà Alberich, A. Gomis Maya, A. Jimenez-Pastor, A. Alberich-Bayarri, L. Marti-Bonmati; Valencia/ES

**Purpose:** To assess the accuracy of a fully automatic nnU-Net CNN algorithm to identify and segment primary neuroblastoma tumours in T2 weighted MR images in children.
**Methods or Backround:** A large multicentre imaging repository of patients from different European institutions with neuroblastic tumours (PRIMAGE) was used to validate the performance of a previously trained tool based on the nnU-Net architecture, to identify and delineate primary neuroblastoma tumours. A dataset of 490 patients with an MR T2 weighted exam was selected (456 at diagnosis and 33 after chemotherapy). This dataset was completely independent from the one used to train and test the model. The nnU-Net segmentation mask was manually edited by an expert radiology and the Dice Similarity Coefficient (DSC), a modified version of the False Positive Rate and the False Negative Rate were calculated.
**Results or Findings:** All cases were successfully segmented by the AI solution. The mean DSC obtained from the comparison of the automatically obtained masks with the masks resulting after a manual edition was 0.99. Sub-analyses were performed regarding the timepoint (diagnosis vs treatment), tumour location and magnetic field strength with no significant differences.
**Conclusion:** The automatic nnU-Net pipeline was able to locate the lesions and segment the tumour on the T2 weighted images in all cases. No statistically significant differences were found between the CNN and manual editing. The semi-automatic approach with minor manual editing of the deep learning segmentation increases radiologist's confidence in the solution and reduces significantly their required involvement in this task.

**Limitations:** Tumours may contact with extensive lymph nodes, making their differentiation difficult, which can lead to errors in the segmentation performed by the net.
**Ethics committee approval:** The ethics committee for investigation with medicinal products of the University and Polytechnic La Fe Hospital approved this study (ethic code: 2018/0228, 27 March 2019).
**Funding for this study:** Funding for this study was received from PRIMAGE, Horizon 2020|RIA project (Topic SC1-DTH-07-2018), grant agreement 826494.
**Author Disclosures:**
Armando Gomis Maya: Nothing to disclose
Leonor Cerdà Alberich: Nothing to disclose
Luis Marti-Bonmati: Grant Recipient: PRIMAGE
Ana Jimenez-Pastor: Nothing to disclose
Diana Veiga Canuto: Nothing to disclose
Angel Alberich-Bayarri: Shareholder: Quibim CEO: Quibim Founder: Quibim

**10:30-12:00** **Research Stage 2**

### Research Presentation Session: Emergency Imaging

### RPS 1817

### Emergency radiology: new frontiers and practice issues

**Moderator**
S. Wirth; Villingen-Schwenningen/DE

### RPS 1817-2

**The value of deep learning reconstruction algorithm in CT whole brain perfusion in patients with acute ischaemic stroke**
*L. Lei*, X. Guo, H. Wang, J. Ma, S. W. Yue; Zhengzhou/CN

**Purpose:** To evaluate the influence of deep learning image reconstruction (DLIR) algorithm on the image quality and perfusion parameters of brain CT perfusion (CTP) scanning in patients with acute ischaemic stroke and its diagnostic value for acute cerebral infarction.
**Methods or Backround:** A total of 47 patients with acute ischaemic stroke undergoing CTP examination within 24 hours of symptom onset were collected prospectively. Datasets were reconstructed with filtered back projection (FBP), adaptive statistical iterative reconstruction-Veo (ASIR-V) (40%, 80%) and DLIR (low, medium, high; TrueFidelity™). We evaluated the diagnostic accuracy of different reconstruction algorithms by detecting cerebral infarction, referring to the brain CT or MRI reviewed within one week of onset, and calculated perfusion parameters such as CBF, CBV, Tmax, MTT and TTP in grey matter and white matter of the frontal, temporal and parietal lobes on the contralateral side. CT values and SD were measured at the same site as where the perfusion parameters were collected and the SNR and CNR were calculated. Subjective evaluation was completed by two senior radiologists according to the Abels' scoring system.
**Results or Findings:** The diagnostic accuracy of CTP images with 6 reconstruction algorithms for detecting acute cerebral infarction was 85.1% (40/47). The longest diameter of core infarction measured by DLIR-H images was closest to final infarct size. No statistically significant difference in perfusion parameters and CT values was found among the 6 groups ($P>0.05$), while the image noise gradually decreased as the strength levels increased whether ASIR-V or DLIR, CNR and SNR gradually increased (all $P<0.001$). All images could meet the diagnostic requirements with scores greater than six points.
**Conclusion:** Although FBP and ASIR-V could meet diagnostic requirements, DLIR images could effectively improve image quality and DLIR-H is the most sensitive for lesion detection.
**Limitations:** Not applicable.
**Ethics committee approval:** No information provided by the submitter.
**Funding for this study:** Not applicable.
**Author Disclosures:**
Jinping Ma: Nothing to disclose
Hui Wang: Nothing to disclose
Xiaoxu Guo: Nothing to disclose
Song Wei Yue: Nothing to disclose
Limin Lei: Nothing to disclose

### RPS 1817-3

**Quantitative assessment of the correlation between pulmonary perfusion defect index and pulmonary embolism risk stratification using dual-layer detector spectral CT**
*Y. Zhou*[1], Z. Wang[1], S. Dong[2]; [1]Zhengzhou/CN, [2]Beijing/CN
*(1178033775@qq.com)*

**Purpose:** To investigate iodine density (ID) map combined with effective atomic number (Zeff) map using dual-layer detector spectral CT (DLCT) for quantitative evaluation of the correlation between pulmonary perfusion defect index (PPDI) and clinical risk stratification of acute pulmonary embolism.
**Methods or Backround:** This study retrospectively enrolled 75 patients with acute pulmonary embolism (PE) who underwent CTPA using DLCT. The clinical risk stratification was conducted according to the ESC 2019 guidelines. Spectral data were post-processed to ID map, Zeff map and fusion of ID and Zeff (ID-Zeff map). Two radiologists independently recorded the area and number of pulmonary lobes occupied by low perfusion areas separately and evaluated the difference of pulmonary perfusion defect areas in each group. The difference in PPDI was evaluated according to the risk stratification for each group. The ROC curve and the area under the curve (AUC) were drawn to evaluate the diagnostic efficiency. T-test and Mann-Whitney U test were used for quantitative data, and Chi-square test was used for qualitative data.
**Results or Findings:** The number of perfusion defect areas detected in ID map, Zeff map and ID-Zeff map were 154, 198 and 214, respectively. Between ID and ID-Zeff map, the average number of low perfusion areas (2.05±0.35 vs. 2.85±0.52p <0.001) and percentage of perfusion defect areas in each pulmonary lobe (22.45%±5.04% vs. 31.44%±9.24%, $p<0.001$) were lower in ID-Zeff. The low risk group was significantly lower than medium risk group in three groups (all $p<0.001$). All of the three groups can effectively distinguish low/intermediate risk. Furthermore, the ID-Zeff map showed the highest AUC among the three groups (AUC=0.896).
**Conclusion:** ID-Zeff fusion map can improve the identification of hypoperfusion areas. The PPDI based on ID-Zeff map can accurately predict the clinical risk stratification of acute pulmonary embolism.
**Limitations:** Not applicable.
**Ethics committee approval:** No information provided by the submitter.
**Funding for this study:** Not applicable.
**Author Disclosures:**
Shushan Dong: Nothing to disclose
Zhihao Wang: Nothing to disclose
Yuhan Zhou: Nothing to disclose

### RPS 1817-4

**Role of CT in evaluation of left ventricular wall in out-of-hospital cardiac arrest patients treated with ECMO**
*A. Pelle*, C. Ruggieri, S. Veglia, M. Zanierato, D. Di Cuonzo, G. Ciccone, G. Limerutti; Turin/IT

**Purpose:** Out-of-hospital cardiac arrest (OHCA) is burdened by high mortality, especially when refractory (RCA). In selected cases, extra-corporeal life support therapies (ECLS) may be used, including extra-corporeal membrane oxygenation (EMCO). CT in the initial phase is becoming increasingly important, due to its ability to ensure the position of ECMO cannulas and to look for extra-cardiac causes of CA and ECMO-related complications. However, few studies have proved the ability of CT to detect abnormal left ventricular wall findings. This study examined left ventricular wall findings in CT after ECLS and those of echocardiography and coronary-angiography.
**Methods or Backround:** We evaluated OHCA patients treated with ECLS between January 2021 and May 2022, who underwent CT, echocardiography and coronary-angiography. We reviewed CT images paying attention to left ventricular wall enhancement, in short and long axis, divided into seven areas; these findings were classified as follows: homogeneous enhancement (HE), segmental defect (SD), total defect (DT). These segments were subsequently divided into three groups depending on territories of the major coronary arteries (right, circumflex and left anterior descending). A territory was rated as abnormal when at least one segment had altered enhancement in CT, was hypokinetic on echocardiography, and its tributary coronary was occluded on coronary-angiography.
**Results or Findings:** A total of 13 patients (median age 51 years) were eligible. All underwent CT and echocardiography; only 7 underwent coronary-angiography. Concordance between CT and echocardiography was high (sensitivity 84%, specificity 85.7%). Good correlation was found between CT and coronary-angiography (sensitivity 100%, specificity 75%).
**Conclusion:** Left ventricular wall findings on CT in RCA patients treated with ECLS are a good predictor of coronary artery disease and improve diagnostic performance. These results need further validation due to our limited sample size and lack of studies.
**Limitations:** Identified limitations were: (1) the small sample size and (2) the lack of literature.
**Ethics committee approval:** No information provided by the submitter.
**Funding for this study:** No funding was received for this study.

**Author Disclosures:**
Daniela Di Cuonzo: Nothing to disclose
Simona Veglia: Nothing to disclose
Chiara Ruggieri: Nothing to disclose
Giovannino Ciccone: Nothing to disclose
Marinella Zanierato: Nothing to disclose
Andrea Pelle: Nothing to disclose
Giorgio Limerutti: Nothing to disclose

### RPS 1817-5

**Positive rate and quality assessment of CT pulmonary angiography in sickle cell disease: a case–control study**

*A. H. Yusuf*; Manama/BH

**Purpose:** Pulmonary complications of sickle cell disease are common and can be clinically indistinguishable from pulmonary embolisms which can lead to the overuse of computed tomography (CT) pulmonary angiography. The diagnostic performance of CT pulmonary angiography improves by maximising its quality. Little is known about the positive rate and quality of CT pulmonary angiography in sickle cell disease.
**Methods or Backround:** In this retrospective study we examined the positive rate and mean contrast enhancement of the pulmonary artery in CT pulmonary angiography studies that were performed to rule out pulmonary embolisms in sickle cell disease. A control group was selected for comparison.
**Results or Findings:** The study included 480 patients, including 240 sickle cell disease patients and a similar number of control patients. In total, 19 patients had a pulmonary embolism, yielding a positive rate of 4.0%. Patients with sickle cell disease had a positive rate comparable to that of the control group (4.2% vs. 3.8%; P=0.08). However, patients with sickle cell disease had a significantly lower contrast enhancement than those in the control group (266.1±90.5 Hu vs. 342.2±116.1 Hu; P< 0.01). In particular, a quarter (25.4%) of sickle cell disease patients had suboptimal scans. The linear regression model revealed that sickle cell disease patients had a lower contrast enhancement of the pulmonary artery compared to the control group.
**Conclusion:** This study showed a relatively low positive rate of CT pulmonary angiography for both sickle cell disease patients and the control group. However, sickle cell disease was significantly associated with suboptimal image quality with inadequate pulmonary arterial contrast enhancement.
**Limitations:** The retrospective nature of the study is an important limitation. In addition, there was no subjective assessment of the scans.
**Ethics committee approval:** This study was approved by the ethical review committee of Salmaniya Medical Complex (Bahrain).
**Funding for this study:** No funding was received for this study.
**Author Disclosures:**
Ali Hassan Yusuf: Nothing to disclose

### RPS 1817-6

**Survey of emergency radiology organisation in Swiss and and Nordic country hospitals**

*A. Platon*[1], M. T. Nummela[2], J. B. Dormagen[3], F. V. Bensch[2], F. Thoren[4], L. L. Nagel[5], H. Teisen[6], P-A. A. Poletti[1]; [1]Geneva/CH, [2]Helsinki/FI, [3]Oslo/NO, [4]Göteborg/SE, [5]Aarhus/DK, [6]Svendborg/DK
*(Alexandra.Platon@hcuge.ch)*

**Purpose:** To assess the organisation of emergency radiology (ER) in hospitals in Switzerland and Nordic countries (Denmark, Finland, Norway, Sweden).
**Methods or Backround:** An online survey, with multiple-choice and open questions, was sent to radiologists in charge of ER in five university and five county hospitals in each country, on behalf of the Swiss Society of Emergency Radiology and of the Nordic Forum for Trauma and Emergency Radiology.
**Results or Findings:** The vast majority (98%) of hospitals answered the survey. A dedicated ER unit was found in 62% of university and 41% of county hospitals. 65% of university and 32% of county hospitals have an attending radiologist dedicated to ER. 93% of university and 63% of county hospitals have a CT-scanner dedicated to ER. In 69% of university and 77% of county hospitals, emergency radiologists provide the interpretation of neuro-CT examinations during on-call time. In 58% of university and 90% of county hospitals, general radiologists are also on-call for paediatric radiology. Teleradiology is performed in 42% of university and in 68% of county hospitals. MRI is used for both neuro and bodily emergencies in 73% of university and 55% of county hospitals. Sonography for non-traumatic emergencies is performed by radiologists in 77% of university and 77% of county hospitals.
**Conclusion:** The survey describes the current organisation and practices of ER in Swiss and Nordic hospitals. The collected data can serve as a basis of comparison for further surveys.
**Limitations:** An identified limitation was that the hospitals were of various sizes.
**Ethics committee approval:** No information provided by the submitter.
**Funding for this study:** No funding was received for this study.
**Author Disclosures:**
Mari T. Nummela: Nothing to disclose
Alexandra Platon: Nothing to disclose
Henrik Teisen: Nothing to disclose
Johann Baptist Dormagen: Nothing to disclose
Pierre-Alexandre Aloïs Poletti: Nothing to disclose
Lise Loft Nagel: Nothing to disclose
Frank Volker Bensch: Nothing to disclose
Fredrik Thoren: Nothing to disclose

### RPS 1817-7

**Impact of the acute pancreatitis diagnostic ordering pattern on emergency radiology department performance**

*J. Vukmirovic*, D. Markovic, T. B. Plojović, L. Sedlar, D. Sekulic, B. Jovandić, D. Vasin, D. Mašulović; Belgrade/RS
*(jelica.doskovic@gmail.com)*

**Purpose:** Prompt and correct diagnosis of acute pancreatitis is essential in the emergency department. Inconsistency of current diagnostic pathways led us to further investigate.
**Methods or Backround:** This retrospective study analysed 190 patients with a confirmed diagnosis of acute pancreatitis from the emergency radiology department at the Clinical Centre of Serbia, over the period January to October 2022. Department performance is evaluated based on confirmed diagnosis and complications, pre-existing knowledge of laboratory results (leukocytes, pancreatic enzymes), clinical findings, exam duration, and need for abdominal CT scan.
**Results or Findings:** All patient underwent abdominal ultrasonography, of which 43 had atypical clinical findings, 126 were without prior laboratory results, 51 had only leukocyte count and 13 had both pancreatic enzyme and leukocyte results. Ultrasonography is relatively sensitive (67%) but not specific without laboratory and clinical findings. Diagnostic ordering pattern had no statistically significant impact on sensitivity and duration of exam, except in the group of patients with atypical symptoms and no pre-exam laboratory results. Initial CT scan was carried out in 91 cases with a sensitivity of 92%, while 43 patients had a follow-up CT for complications with, 96% sensitivity. Results of CT scans proved to be independent of previous findings in all groups, but the call for it could be avoided in a small but significant group if the level of pancreatic enzymes was known. Ultrasonography proved to be on par with CT for the follow-up examinations (sensitivity 86%).
**Conclusion:** Ultrasonography is a necessary initial and reliable follow-up exam. A prior physical exam and complete laboratory results raise its sensitivity and shorten duration in patients with atypical symptoms. Further research should be done on its cost-effectiveness in the emergency department.
**Limitations:** Identified limitations were the relatively small sample and the single centre experience.
**Ethics committee approval:** No information provided by the submitter.
**Funding for this study:** No funding was received for this study.
**Author Disclosures:**
Danijela Sekulic: Nothing to disclose
Danilo Markovic: Nothing to disclose
Ljubica Sedlar: Nothing to disclose
Dragan Vasin: Nothing to disclose
Tarik Binasa Plojović: Nothing to disclose
Biljana Jovandić: Nothing to disclose
Jelica Vukmirovic: Nothing to disclose
Dragan Mašulović: Nothing to disclose

### RPS 1817-8

**Dosimetric studies on the smart-ortho clinical protocol**

E. M. L. Vaccara, F. Levrero, *C. Martinoli*, M. Dordolo, G. Pellecchia, L. Ridolfi, F. Zaottini, R. Picasso, G. Bianchi; Genoa/IT
*(carlo.martinoli@unige.it)*

**Purpose:** In the emergency department we recently installed the radiological equipment, Multitom RAX (Siemens), that extends the anatomical coverage by acquiring up to four images (smart-ortho) and composing them into a single image. During the acceptance tests, dosimetric and geometric checks were carried out. The objective of this work is to verify the patient dose for that protocol, to then be compared with the tabulated value of the diagnostic reference level (DRL) specific for the AP lumbar spine of the ISTISAN 20/22 reports (DAP=1.5-4.5 Gycm2), as well as to participate in multicentre research projects aimed at creating new national DRLs.
**Methods or Backround:** The image quality on an anthropomorphic phantom of the chest abdomen and the dose accuracy has been verified by two methods: for the measurement of the dose-area product, we used the new DAP check camera (Radcal), recently calibrated, and for the extended dose profiles we used the GAFCHROMIC XR-QA2 calibrated at fixed energy. Within a couple of months, we should have a statistically significant number of patients for the smart-ortho protocol. The data will be analysed with the dose management software, Gray Detector.

Saturday

**Results or Findings:** The absence of artefacts was verified with the phantom acquired in automatic mode. The overall anatomical coverage of the specific protocol is 113 cm with three adjacent overlapping images of (2.4±0.5) cm and 131 cm with four overlapping images of (6±1) cm. The relative error of the dose-area product reported on the individual images is equal to 11% compared to the values measured by the Radcal camera. The DAP value of the smart-ortho protocol is equal to the sum of the DAPs of the images.
**Conclusion:** The dose management software will allow evaluation of the risk-benefit ratio of the method for better clinical use.
**Limitations:** No limitations were identified.
**Ethics committee approval:** No information provided by the submitter.
**Funding for this study:** No funding was received for this study.
**Author Disclosures:**
Gennaro Bianchi: Nothing to disclose
Federico Zaottini: Nothing to disclose
Carlo Martinoli: Nothing to disclose
Maurizio Dordolo: Nothing to disclose
Elena Maria Luisa Vaccara: Nothing to disclose
Fabrizio Levrero: Nothing to disclose
Laura Ridolfi: Nothing to disclose
Gianluca Pellecchia: Nothing to disclose
Riccardo Picasso: Nothing to disclose

### RPS 1817-9

**Utility of gadolinium-based contrast agent for the MRI diagnosis of tonsillar abscesses**

*J-P. T. Vierula*, J. Hirvonen, J. Nurminen, M. J. Nyman, J. Heikkinen, K. Mattila; Turku/FI
*(jptvie@gmail.com)*

**Purpose:** MRI has excellent diagnostic accuracy for deep neck abscesses, but scan protocols are long and an intravenous gadolinium-based contrast agent is commonly required. We assessed a short (5 min) scan protocol including only T2- and diffusion-weighted (DWI) sequences for diagnostic accuracy of tonsillar abscesses.
**Methods or Backround:** Three board-certified radiologists retrospectively analysed MRI images from 52 patients with acute tonsillar infections (13 tonsillitis with no abscess, 19 tonsillar abscesses, and 20 deeply extending abscesses) for the presence of an abscess and extension into parapharyngeal or retropharyngeal spaces. Surgical findings were used as reference standard for abscesses. Data were analysed in two separate sessions: first with only axial T2-weighted Dixon and DWI images and subsequently, with additional T1-weighted postcontrast Dixon images. Readers were blinded to clinical and surgical data. Interobserver agreement was calculated using Fleiss' Kappa.
**Results or Findings:** Overall diagnostic accuracy for abscesses across all readers was good to excellent using the short protocol: sensitivity 0.93, specificity 0.85, positive predictive value (PPV) 0.95, negative predictive value (NPV) 0.80, accuracy 0.91. Adding T1-weighted postcontrast images: sensitivity 0.98, specificity 0.85, PPV 0.95, NPV 0.94, accuracy 0.95. Postcontrast images improved accuracy for deep extension from 0.85 to 0.87, specificity from 0.92 to 0.97, interobserver Kappa from 0.70 to 0.82 for abscesses and from 0.50 to 0.66 for deep extension.
**Conclusion:** Even a short MRI protocol without postcontrast images has good to excellent diagnostic accuracy for tonsillar abscesses, outperforming previously published estimates for contrast-enhanced CT. Adding postcontrast images reduced false negatives for abscesses and false positives for deep extension, and improved radiologist agreement. Using a contrast agent is a trade-off between shorter scan times and slightly improved accuracy.
**Limitations:** The limited availability of emergency MRI was an identified limitation.
**Ethics committee approval:** No information provided by the submitter.
**Funding for this study:** Funding was received from the Sigrid Jusélius Foundation.
**Author Disclosures:**
Jaakko Heikkinen: Nothing to disclose
Jussi Hirvonen: Nothing to disclose
Jari-Pekka Tapani Vierula: Nothing to disclose
Mikko Juhani Nyman: Nothing to disclose
Kimmo Mattila: Nothing to disclose
Janne Nurminen: Nothing to disclose

### RPS 1817-10

**Emergency cranial CT scan "without relevant findings": should clinical CT requests be substantially optimised?**

*E. Otón González*, M. S. Canales, N. I. Casado Alarcón, H. Ortiz Mayoral, E. Cotillo Ramos, J. M. Plasencia Martinez, J. M. Garcia Santos; Murcia/ES

**Purpose:** To analyse the cranial CT yield in a general hospital radiology department (GHED) in non-trauma emergencies.
**Methods or Backround:** Non-trauma cranial CTs performed in our GHED between October 2017 to January 2022 were included. Those with known malignant tumours or chronic hydrocephalus, and repeated CTs within the following three months, were excluded. We considered as "relevant findings" radiology reports of acute ischaemia/haemorrhages, intracranial masses, unknown hydrocephalus, or brain oedema. The association between the clinical CT request and 1) the relevant imaging findings, and 2) the CT findings' relevance on the final diagnosis were assessed by Chi2 test.
**Results or Findings:** We included 499 patients, 237 (47.5%) men, median age 63 (44-78) years. Relevant findings were significantly more frequent in patients with than without: motor deficit (30/85 [35%], 80/414 [19%]; P=0.001); speech deficit (37/89 [42%], 73/410 [18%]; P<0.001); postural instability or gait disorder (25/77 [33%], 85/42 [20%]; P=0.02); seizures (13/34 [38%], 97/465 [21%]; P=0.018); cognitive impairment (21/59 [36%], 89/440 [20%]; P=0.008); and acute mental status impairment (37/113 [33%], 73/385 [19%]; P=0.002). On the contrary, differences were not significant in patients with or without: sensory deficit (14/63 [22%], 96/439 [22%]; P=0.971); visual deficit (11/55 [20%], 98/442 [22%]; P=0.714); headache (28/148 [19%], 82/351 [23%]; P=0.274); dizziness (28/122 [23%], 82/337 [22%]; P=0.781); vertigo (8/50 [16%], 102/449 [23%]; P=0.277); syncope/presyncope (13/59 [22%], 97/440 [22%]; P=0.998); delirium/other psychiatric symptoms (7/39 [18%], 103/460 [22%]; P=0.520); nausea/vomiting (10/64 [16%], 98/432 [23%]; P=0.200); neck pain (7/26 [27%], 103/472 [22%]; P=0.542); fever (5/33 [15%], 105/466 [22%]; P=0.322); hypertensive crisis (4/15 [27%], 106/488 [22%]; P=0.661); and lower limbs weakness (6/33 [18%], 104/466 [22%]; P=0.580). The latter constituted 44.5% (222/499) of all cranial CT requests. The same significant associations were found between the CT request and the CT findings' relevance on the final diagnosis at discharge.
**Conclusion:** The cranial CT yield in our GHED suggests that there is substantial room for improvement; action is needed for clinical request optimisation.
**Limitations:** The evolution data are not yet available.
**Ethics committee approval:** Code CETI 02/2022.
**Funding for this study:** No funding was received for this study.
**Author Disclosures:**
Elena Otón González: Nothing to disclose
Juana María Plasencia Martinez: Nothing to disclose
Jose Maria Garcia Santos: Nothing to disclose
Marta Sánchez Canales: Nothing to disclose
Estefanía Cotillo Ramos: Nothing to disclose
Nuria Isabel Casado Alarcón: Nothing to disclose
Herminia Ortiz Mayoral: Nothing to disclose

### RPS 1817-11

**The use of dual and triple rule out computerised tomography angiography by using diagnostic low-dose contrast material and radiation in acute chest pain**

*A. K. Imrek*, M. Hızal, Y. Yılmazsoy, Z. Coşgun; Bolu/TR
*(dr.ahmetimrek@gmail.com)*

**Purpose:** Our aim is to investigate the feasibility of dual and triple rule out CT angiography, by reducing the radiation dose by using relatively high milliampere-seconds with less contrast agent and low kilovoltage, without affecting the diagnostic value.
**Methods or Backround:** We acquired dual/triple rule out CT angiographies from the Izzet Baysal Training and Research Hospital between April 2021 and February 2022, using both standard contrast amount and standard-dose, and low-volume contrast medium and low radiation dose for the thorax or thoracoabdomen examinations. A total of 93 patients with standard-dose and 91 patients with low-dose were included in the study.
**Results or Findings:** Signal-to-noise ratio and contrast-to-noise ratio were calculated separately. There was no significant difference in CNR values between the two groups in the aorta, pulmonary and coronary arteries, however, a significant difference was found in SNR values. According to the subjective image quality evaluation, no significant difference was found between the standard and low-dose patients. In the low-dose group, radiation dose was reduced by 63.80% and the contrast material by 31.5%, compared to the standard-dose group.
**Conclusion:** In our study, we have shown that dual/triple rule out CT angiography can be performed with low-dose radiation and low-volume contrast medium. Although its routine use is not always appropriate, low-dose CT angiography may be preferred in emergency situations where kidney function tests cannot be performed, or in patients with borderline renal function tests or in an at risk group.

**Limitations:** Cardiac rhythm regulating drugs could not be administered due to the fact that the patients came from the emergency department and we did not have suitable physical conditions.
**Ethics committee approval:** This study was approved by Bolu Abant Izzet Baysal University Clinical Research Ethics Committee (approval date: 27/04/2021, decision number: 2021/87).
**Funding for this study:** Not applicable.
**Author Disclosures:**
Yunus Yılmazsoy: Nothing to disclose
Zeliha Coşgun: Nothing to disclose
Mustafa Hızal: Nothing to disclose
Ahmet Kerem Imrek: Nothing to disclose

**10:30-12:00** **Research Stage 3**

### Research Presentation Session: Neuro

### RPS 1811

### Stroke imaging and neurovascular interventions

**Moderator**
S. Haller; Geneva/CH
**Author Disclosures:**
Sven Haller: Advisory Board: EPAD; Consultant: SPINEART, WYSS

### RPS 1811-2

**Left atrial appendage thrombus detection using extended carotid CT angiography in acute stroke**

*S. I. Antal*, N. Szabó, Z. T. Kincses; Szeged/HU
*(szabolcs.antal96@gmail.com)*

**Purpose:** Our objective is to assess the percentage of ischaemic stroke that can be attributed to left atrial appendage (LAA) thrombi detectable by CTA. From the acquired database we try to establish the LAA morphologic features that have the highest risk for developing thrombi.
**Methods or Backround:** Current guidelines recommend transthoracic echocardiography (TTE) for routine screening of cardiac emboli, however the visualisation of the LAA where the thrombi are commonly found is poor. Transoesophageal echocardiography (TOE) would provide better detectability of LAA thrombus, but it is a time-consuming and semi-invasive method. Extending non-gated carotid CTA examination to the LAA could reliably detect thrombi, and could aid treatment and secondary prevention.
**Results or Findings:** We examined 129 acute stroke patients' (aged 70.6 ± 11.22 years) CTA scans that were extended 4 cm below the carina to visualise the LAA. We detected LAA thrombus in five patients (3.87%), two of them had atrial fibrillation. 19.38% of all patients (25 cases) had recently discovered or previously known atrial fibrillation. Windsack and cauliflower morphologies were the most common LAA morphologies associated with filling defect.
**Conclusion:** Our extended CTA scans reliably detected LAA thrombi even in cases where TTE did not, and showed that 3 patients' LAA thrombus would have been untreated based on ECG monitoring and TEE. We also showed that the benefits of CTA outweigh the disadvantages arising from the slight amount of excess radiation.
**Limitations:** Limitations include not scanning with ECG-gating (resulting in motion artefacts), and not repeating the scans several minutes after contrast agent administration (possible false positives).
**Ethics committee approval:** No information provided by the submitter.
**Funding for this study:** The study was supported by a GINOP-2.3.2-15-2016-00034 grant, an EFOP-3.6.1-16-2016-00008 grant, by a Horizon 2020 grant (H2020-MSCA-RISE-2016 734718), NAP 2.0 (2017-1.2.1-NKP-2017-00002) and the National Brain Research Program (KTIA_13_NAP-A-II/20).
**Author Disclosures:**
Nikoletta Szabó: Nothing to disclose
Zsigmond Tamas Kincses: Nothing to disclose
Szabolcs István Antal: Nothing to disclose

### RPS 1811-3

**Posterior circulation stroke: prognostic value and predictive power of two evaluation systems on angio-CT: BATMAN score and PC-CS**

*G. Salmè*[1], A. Carisio[2], S. Veglia[2], A. Krainik[3], S. Grand[3], D. Di Cuonzo[2], A. N. A. Serafini[2], G. Ciccone[2], G. Limerutti[2]; [1]Savigliano/IT, [2]Turin/IT, [3]Grenoble/FR
*(salme.giulio@gmail.com)*

**Purpose:** Compare two different angio-CT scores to perform intracranial vessels evaluation in posterior circle acute ischaemic stroke: BATMAN score and PC-CS.
**Methods or Backround:** A retrospective analysis of 60 patients with a diagnosis of acute ischaemic stroke was performed: 33 patients came from Centre Hospitalier Grenoble Alpes – CHUGA, 27 from A.O.U. Città della Salute e della Scienza, Mollette, Turin. We analysed system scores feasibility and compared interobserver agreement, correlation between these system scores and prognosis.
**Results or Findings:** Time necessary to apply score is quite similar between an expert radiologist and a fellow (90-100 s). We found good interobserver agreement. These score systems expressed a good correlation with discharge prognosis (p value: 0.034 for both scores) and a 3 months to discharge prognosis (BATMAN score p value: 0.0012; PC-CS p value: 0.0035). We did not find correlation in comparison with other scores, such TICI score, NIHSS and TOAST classification.
**Conclusion:** BATMAN and PC-CS are feasible and easy to apply, in a short time; these are crucial in times of related morbidities. They express a strong correlation with prognosis and lead operators to improve treatment in patients who show a better long-time prognosis.
**Limitations:** This is a retrospective study that includes a small population, matching the low prevalence of posterior circle acute ischaemic stroke that allows timely treatment.
**Ethics committee approval:** No information provided by the submitter.
**Funding for this study:** No funding was required for this retrospective study.
**Author Disclosures:**
Giulio Salmè: Nothing to disclose
Andrea Carisio: Nothing to disclose
Simona Veglia: Nothing to disclose
Alexandre Krainik: Nothing to disclose
Giovannino Ciccone: Nothing to disclose
Daniela Di Cuonzo: Nothing to disclose
Alessandro Niccolò Annibale Serafini: Nothing to disclose
Sylvie Grand: Nothing to disclose
Giorgio Limerutti: Nothing to disclose

### RPS 1811-4

**Mechanical thrombectomy beyond the circle of Willis: efficacy and safety of different techniques for M2 occlusions**

*R. Gigli*, I. Valente, A. M. Alexandre, G. Garignano, A. Pedicelli; Rome/IT
*(riccardogigli93@gmail.com)*

**Purpose:** M2 segment occlusions represent one third of non-lacunar ischaemic stroke and can lead to permanent neurological deficits. Various techniques are available for mechanical thrombectomy beyond the circle of Willis, but data evaluating their effectiveness and safety are lacking.
**Methods or Backround:** A retrospective review of patients with ischaemic stroke undergoing mechanical thrombectomy for M2 occlusions from 13 centres in North American and Europe was performed. The primary outcome was a 90-day modified Rankin Scale and reperfusion rates across stent-retriever, direct aspiration and combined techniques.
**Results or Findings:** There were 465 patients with M2 occlusions who underwent mechanical thrombectomy. Stent-retriever alone was used in 133 (28.6%), direct aspiration alone in 93 (20.0%) and the combined technique in 239 (51.4%) patients. Successful reperfusion was achieved with the combined technique in 198 (82.2%), with stent-retriever alone in 112 (84.2%) and with direct aspiration alone in 62 (66.7%). Intraprocedural subarachnoid hemorrhages (iSAH) were 36 (7.7%) and were more likely to occur in patients treated with the stent-retrievers and combined technique. Good clinical outcome was achieved in 260 (61.8%) patients; 59 (14.0%) patients died. Older age, higher baseline NIHSS, parenchymal haemorrhage and iSAH were associated with poor outcome while successful recanalisation and higher baseline ASPECTS were associated with good outcome. No differences were found among the three techniques in terms of clinical outcome.
**Conclusion:** Stent-retrievers and a combined approach for M2 occlusions seem more effective than direct aspiration, but with higher rates of iSAH. This leads to no detectable difference in clinical outcome at 3 months.
**Limitations:** Reperfusion grade and complications were self-adjudicated. Interventionalist discretion could have favoured a specific technique. 89 patients were excluded from this study due to lack of 90-day mRS data, which may have biased the results.
**Ethics committee approval:** No information provided by the submitter.
**Funding for this study:** No funding was received for this study.
**Author Disclosures:**
Iacopo Valente: Nothing to disclose
Andrea Maria Alexandre: Nothing to disclose
Giuseppe Garignano: Nothing to disclose
Riccardo Gigli: Nothing to disclose
Alessandro Pedicelli: Nothing to disclose

### RPS 1811-5

**Cerebral arterial air emboli on post-endovascular treatment dual-energy CT are associated with poor short- and long-term clinical outcomes**

I. Huijberts[1], *F. M. E. Pinckaers*[1], W. H. van Zwam[1], H. D. Boogaarts[2], R. van Oostenbrugge[1], A. Jacobi-Postma[1]; [1]Maastricht/NL, [2]Nijmegen/NL
*(floor.pinckaers@mumc.nl)*

**Purpose:** To determine the incidence of cerebral arterial air emboli (CAAE) on immediate post-endovascular treatment (EVT) dual-energy CT (DECT) and describe its association with clinical outcomes.
**Methods or Backround:** EVT records of patients treated in the Maastricht UMC+ from 2010 to 2019 were screened. Exclusion criteria included intracerebral haemorrhage on post-EVT DECT. In the ipsilateral middle cerebral artery (MCA)-territory, circular and linear (length≥1.5*width) CAAE were counted. Clinical data were collected from prospective stroke records. The modified Rankin Scale (mRS) at 90 days was the primary outcome measure. Multivariable linear, logistic, and ordinal regression was used as appropriate to analyse the association between each outcome measure and (1) linear and (2) isolated circular CAAE. Adjustments were based on univariate analyses.
**Results or Findings:** Out of a total of 651 EVT-records, 402 patients were included. In 65 patients (16%), at least one linear CAAE was found in the ipsilateral MCA-territory. 17 patients (4%) showed isolated circular CAAE. Multivariable regression analyses showed a significant association between both the presence and the amount of linear CAAE in the ipsilateral MCA-territory and the mRS at 90 days (presence: adjusted (a)cOR: 3.24, 95%CI: 1.79-5.85; amount: 1.29, 95%CI: 1.14-1.46), NIHSS at 24-48h (presence: aβ: 4.02, 95%CI: 1.72-6.32; amount: aβ: 0.82, 95%CI: 0.37-1.27), mortality at 90 days (presence: aOR: 3.17, 95%CI: 1.4-7.19; amount: aOR: 1.24, 95%CI: 1.07-1.43) and stroke progression (presence: aOR: 3.66, 95%CI: 1.75-7.67; amount: aOR: 1.28, 95%CI: 1.11-1.47). Isolated circular CAAE did not have a significant effect on any outcome measure on multivariable analyses.
**Conclusion:** CAAE may be found frequently on post-EVT CT imaging. The presence and the amount of linear CAAE, but not circular CAAE, are associated with unfavourable short- and long-term clinical outcomes.
**Limitations:** A limitation is our single-centre setup.
**Ethics committee approval:** The ethics committee of the Maastricht University Medical Centre+, Maastricht, the Netherlands, approved this study (MED-2020-1456). The need for individual patient consent has been waived.
**Funding for this study:** Not applicable.
**Author Disclosures:**
Wim H. van Zwam: Other: DSMB member of the Anaconda's Solonda study, In Extremis study and Philips' We-Trust study (funding paid to the institution) Consultant: Stryker, Nicolab and Cerenobus (fees paid to the institution)
Hieronymus D. Boogaarts: Nothing to disclose
Robbert van Oostenbrugge: Nothing to disclose
A.A. Jacobi-Postma: Research/Grant Support: Siemens Healthineers and Bayer Healthcare (paid to the institution)
Florentina Maria Egidius Pinckaers: Nothing to disclose
Ilse Huijberts: Nothing to disclose

### RPS 1811-6

**The relationship between CT perfusion and post-endovascular therapy contrast extravasation on dual-energy CT in acute ischaemic stroke patients**

*M. M. Q. Robbe*, F. M. E. Pinckaers, R. van Oostenbrugge, W. van Zwam, A. Jacobi-Postma; Maastricht/NL

**Purpose:** The purpose of this study was to investigate the relationship between CTP derived parameters and CE on dual-energy CT (DECT) immediately after EVT.
**Methods or Backround:** In acute ischaemic stroke patients, CT perfusion (CTP) is used for the estimation of ischaemia (as core) and hypoxia (as penumbra). Both could possibly lead to blood brain barrier disruption (BBBD), which could result in contrast extravasation (CE) during endovascular stroke therapy (EVT). As such, CE might be a surrogate marker for BBBD. All patients treated with EVT in our center from 2010 up and including 2019 were screened. Included patients had an anterior large vessel occlusion and received both CTP at baseline and DECT within 3 hours post-EVT. The CTP core and penumbra thresholds were set at rCBF<20% and Tmax>6s, respectively. The Alberta stroke programme early CT score (ASPECTS) was used to score penumbra and core on CTP (forming a CTP-ASPECTScore and CTP-ASPECTScore+penumbra), and to score CE on iodine maps of DECT (forming a CE-ASPECTS). The relationship between CTP-ASPECTScore and CTP-ASPECTScore+penumbra, and CE-ASPECTS was assessed using Spearman's rank correlation. A delta ASPECTScore-CE (CTP-ASPECTScore minus CE-ASPECTS) was calculated.
**Results or Findings:** Out of a total of 654 EVT records, 109 patients were included. The median CTP-ASPECTScore+penumbra, CTP-ASPECTScore and CE-ASPECTS were 2 (IQR 1-4), 7 (IQR 4-10), and 6 (IQR 3-8) respectively. The correlation between CTP-ASPECTScore+penumbra and CE was 0.15 (p=0.13), and the correlation between CTP-ASPECTScore and CE was 0.23 (p=0.02). The median ASPECTScore-CE was 0 (IQR -2-2) indicating that the amount of CTP-ASPECTScore is not structurally lower or higher than the amount of CE-ASPECTS.
**Conclusion:** We found a weak but significant correlation between CTP-ASPECTScore and CE-ASPECTS, indicating core tissue might not result in CE, and CE might not always be caused by core.
**Limitations:** This was a single-centre study.
**Ethics committee approval:** This study was approved by the ethics committee of the Maastricht University Medical Centre+, Maastricht, The Netherlands (MEC-2020-1456). The need for individual patient consent was waived.
**Funding for this study:** Not applicable
**Author Disclosures:**
Magretha Maria Quirien Robbe: Nothing to disclose
Wim van Zwam: Consultant: Stryker, Nicolab, and Cerenovus (paid to the institution) Other: DSMB member of Philips We-Trust study, Anaconda's Solonda study, and In Extremis study (paid to the institution)
Robbert van Oostenbrugge: Nothing to disclose
A.A. Jacobi-Postma: Research/Grant Support: Research/Grant Support: Siemens Healthcare and Bayer Healthcare
Florentina Maria Egidius Pinckaers: Nothing to disclose

### RPS 1811-7

**Prognostic implications of intracranial haemorrhage on dual-energy CT immediately following endovascular treatment of acute ischaemic stroke**

*F. M. E. Pinckaers*[1], M. M. Q. Robbe[1], S. G. Olthuis[1], H. D. Boogaarts[2], W. H. van Zwam[1], R. van Oostenbrugge[1], A. Postma[1]; [1]Maastricht/NL, [2]Nijmegen/NL
*(floor.pinckaers@mumc.nl)*

**Purpose:** To describe the occurrence and prognostic relevance of intracerebral haemorrhage (ICH) on dual-energy CT (DECT) within 3h after endovascular treatment (EVT).
**Methods or Backround:** EVT records in the MUMC+ from 2010 to 2019 were screened. DECTs were evaluated for ICH according to the Heidelberg criteria. Each ICH patient was classified into a single Heidelberg class (HC) by the following order of severity: (HC-2) parenchymal haematoma (PH)2, (HC-1) haemorrhagic infarction (HI)1, HI2 and PH1, (HC-3) remote PH, intraventricular haemorrhage and subarachnoid haemorrhage. Symptomatic ICH (sICH) was likewise scored according to the Heidelberg criteria. Clinical data were gathered from prospective records. The primary outcome measure was the modified Rankin Scale (mRS) score at 90 days. Conventional tests, in which each HC was compared to patients without ICH, were performed on a single imputed dataset.
**Results or Findings:** Out of 651 records, 478 patients were included. 76 (15.9%) patients showed ICH on DECT, of which 18 were classified as HC-1, 6 as HC-2, and 52 as HC-3. All HCs were significantly associated with the NIHSS at 24-48 h (p=.002, p<.001 and p=.008, respectively). Both asymptomatic (a) ICH and sICH of HC-1 were associated with the mRS (p=0.004 and p=0.03, respectively). aICH of HC-1 was also associated with mortality at 90 days (p=0.001), whereas sICH was not (p=0.2). All ICHs in HC-2 were symptomatic, and the association to both mRS and mortality was significant (p<.001). In HC-3, only sICH was associated with the mRS and mortality (p=0.001 and p=0.02, respectively).
**Conclusion:** Post-EVT ICH is a frequent finding and is associated with both short- and long-term outcomes. These results suggest that a routine post-EVT DECT may have additional value in acute stroke management.
**Limitations:** Limitations included our single-centre setup.
**Ethics committee approval:** This study was approved by the ethics committee of the Maastricht University Medical Centre, Maastricht, The Netherlands (MEC-2020-1456). The need for individual patient consent has been waived.
**Funding for this study:** Not applicable
**Author Disclosures:**
Susanne G.H. Olthuis: Nothing to disclose
Alida Postma: Research/Grant Support: Siemens Healthineers and Bayer Healthcare (paid to the institution)
Magretha Maria Quirien Robbe: Nothing to disclose
Wim H. van Zwam: Other: DSMB member of the Anaconda's Solonda study, In Extremis study and Philips' We-Trust study Consultant: Stryker, Nicolab and Cerenobus (paid to the institution)
Hieronymus D. Boogaarts: Nothing to disclose
Robbert van Oostenbrugge: Nothing to disclose
Florentina Maria Egidius Pinckaers: Nothing to disclose

### RPS 1811-8

**Diagnostic accuracy of dual-energy computed tomography in the diagnosis of the neurological complications after endovascular treatment of acute ischaemic stroke: systematic review and meta-analysis**

*N. Yahia*[1], A. Mouffokes[2], N. Yasen[3], A. Elrosasy[4], K. Philip[5], A. E. Ali[6]; [1]Zagazig/EG, [2]Oran/DZ, [3]Cairo/EG, [4]Helwan/EG, [5]Sohag/EG, [6]Chicago, IL/US
*(nohayahiaradio@gmail.com)*

**Purpose:** To investigate the diagnostic performance of dual-energy computed tomography (DECT) in the detection of neurological complications after endovascular therapy (EVT) of acute ischaemic stroke (AIS).
**Methods or Backround:** We searched PubMed, EBSCO, Web of Science, Scopus, and ScienceDirect databases for the relevant published studies. We selected studies that estimate the validity of DECT in the detection of complications after EVT for AIS. A quality assessment of bias and applicability was conducted using the Quality of Diagnostic Accuracy Studies-2 Tool (QUADAS II). A meta-analysis was performed to calculate mean estimates of sensitivity, specificity, positive likelihood ratio (PLR) and negative likelihood ratio (NLR) for each detected complication. The summary receiver operating characteristic (sROC) curve was utilised to get the Cochran Q index and the area under the curve (AUC).
**Results or Findings:** Of the 19 studies, 18 studies were included in the quantitative synthesis. The pooled overall sensitivity and specificity of DECT in the detection of intracerebral haemorrhage (ICH) were 67.6% (95% CI: 63.3-71.8%) and 95.5% (95% CI: 94.2-96.5%), while in the detection of ischaemia these were 86.4% (95% CI: 83.2-89.2%) and 90.6% (95% CI: 87.1-93.5%), respectively. Heterogeneity between studies was present (Cochran-Q: 0.909, AUC: 0.963) and (Cochran-Q: 0.893, AUC: 0.952) for the detection of ICH and ischaemia in the sROC curve, respectively.
**Conclusion:** DECT shows high accuracy and specificity in the detection of neurological complications post-endovascular AIS treatment. Further prospective studies with a larger sample size and standardised reference tests are recommended to support and validate these findings.
**Limitations:** Considerable and substantial heterogeneity was detected between studies in pooled estimates of the diagnostic performance of DECT in the detection of ICH and cerebral ischaemia, respectively.
**Ethics committee approval:** It is a systematic review and meta-analysis. It was registered at PROSPERO.
**Funding for this study:** No funding was received for this study.
**Author Disclosures:**
Ahmed E. Ali: Nothing to disclose
Adel Mouffokes: Nothing to disclose
Kerollos Philip: Nothing to disclose
Noha Yahia: Nothing to disclose
Amr Elrosasy: Nothing to disclose
Noha Yasen: Nothing to disclose

### RPS 1811-9

**Can we rely on CTA findings to predict infarct volume after stroke?**

*M. J. Rodriguez*, M. Perez Akly, C. H. Besada; Buenos Aires/AR
*(matiasr1991@gmail.com)*

**Purpose:** Multiple clinical and radiological variables are involved in the neurologic outcome of patients with ischaemic stroke. Computed tomography angiography (CTA) is indicated in patients who may be candidates for endovascular therapy. Different radiological findings, such as the degree of leptomeningeal collaterals and the length, density or extension of the thrombus, have been described as predictors of neurological prognosis after a stroke with proximal arterial occlusion. The purpose of this research is to evaluate the best radiological predictors of final infarct volume in patients with proximal vessel oclussion using CTA.
**Methods or Backround:** We performed an observational study. We included adult patients with ischaemic stroke with proximal vessel occlusion diagnosed with CTA evaluated from 2009 to 2019. We measured the thrombus length and the density in non-contrast CT. We recorded collateral status, and the extent of occlusion using the clot burden score. Then, we measured the final infarct volume in a control CT and analysed the relationship between these factors and the final infarct size.
**Results or Findings:** We included 54 patients with ischaemic stroke caused by proximal vessel obstruction, 41 (75%) were female. The average age was 82 years old. Almost 60% of the strokes compromised the right hemisphere and the most affected vessel was the middle cerebral artery (M1) (40.7%). We found a good association between leptomeningeal collateral status (p=0.03) and clot burden score (p=0.01) with the final infarct volume. Thrombus length and density did not correlate with final infarct volume.
**Conclusion:** Leptomeningeal collateral status and clot burden score may assist in predicting the final infarct volume in patients with ischaemic stroke. These CTA findings could determine the selection of patients for mechanical thrombectomy and those for conservative treatment.
**Limitations:** An identified limitation was that this was a single centre study
**Ethics committee approval:** Ethics committee approval was received from CEPI.
**Funding for this study:** No funding was received for this study.
**Author Disclosures:**
Manuel Perez Akly: Nothing to disclose
Cristina Hilda Besada: Nothing to disclose
Matias Javier Rodriguez: Nothing to disclose

### RPS 1811-10

**Endovascular therapy of symptomatic intracranial arterial stenosis with the new generation Credo stent with Heal technology**

*M-S. Schüngel*, S. Schob, R. Brill, M. Skalej; Halle (Saale)/DE
*(marie-sophie.schuengel@uk-halle.de)*

**Purpose:** In the post-SAMMPRIS-era, the adequate therapy of intracranial arterial stenosis (ICAS) remains a matter of debate. The appropriate instant of time – whether to treat or not to treat electively – is still discussed controversially. So far, devices for cerebral angioplasty were mostly used off-label. The novel Credo Heal stent was invented for the treatment of ICAS additionally coming along with an anti-thrombotic coating. However, studies investigating the clinical application are lacking for the moment.
**Methods or Backround:** All patients from our neurovascular centre who underwent endovascular therapy of symptomatic ICAS using the Credo Heal stent were included. For each patient, the case history, medical regimen, technical data, periprocedural adverse events and short-term outcomes were reviewed.
**Results or Findings:** Eleven consecutive patients suffering from symptomatic ICAS were successfully treated with the Credo Heal. Endovascular procedures were performed electively as well as in the setting of acute stroke. Technical failure of the device did not occur in any case. Periprocedural clinical complications were observed in two cases: one thrombus formation and one case of vessel rupture after PTA. Excepting the latter case, sufficient vessel reconstruction (remaining grade of stenosis <20%) after stenting was observed in all cases.
**Conclusion:** The Credo Heal as a novel stent designed for the endovascular therapy of ICAS efforts sufficient treatment of cerebral stenotic lesions. Still, ICAS remains a severe disease and especially the treatment in the setting of acute stroke inherits an increased risk of periinterventional and early postinterventional adverse events.
**Limitations:** The study only involves a small patient collective and short term follow-up results due to the novelty of the device. Multicentric experiences comprising an enlarged treatment group and long-term results are warranted.
**Ethics committee approval:** No information provided by the submitter.
**Funding for this study:** No funding was received for this study.
**Author Disclosures:**
Martin Skalej: Nothing to disclose
Richard Brill: Nothing to disclose
Stefan Schob: Nothing to disclose
Marie-Sophie Schüngel: Nothing to disclose

### RPS 1811-11

**Black-blood imaging of coiled aneurysms reflects angiographic coiling features and aneurysmal micro-canalisation**

*C. Godi*[1], F. Destro[2], P. Garofalo[2], E. Tombetti[1], A. Ambrosi[1], A. Iadanza[1], C. Michelozzi[1], A. Falini[1], N. E. Anzalone[1]; [1]Milan/IT, [2]Cagliari/IT
*(godi.claudia@hsr.it)*

**Purpose:** Black-blood (BB) imaging is particularly suitable for evaluating vessel wall abnormalities, and can demonstrate signs of wall instability in untreated cerebral aneurysms. Initial studies in endovascularly treated aneurysms showed that BB-enhancement is frequent, but its biological meaning is controversial. Hypothesising a relationship of BB-enhancement with incomplete occlusion status and coil packing density at DSA, we compared BB-findings and post-treatment DSA.
**Methods or Backround:** We analysed the subjects undergoing 3T MRI with BB-imaging between January 2017 and October 2020 after a previous aneurysmal coiling. Coil packing density and aneurysmal occlusion status (according to the modified Raymond-Roy Classification) were determined on post-treatment DSA. The presence of aneurysmal pre-contrast BB-signal was scored. BB-enhancement was classified as follows: first, enhancement at the neck; second, intrasaccular/intra-coil enhancement and third, peripheral enhancement. Statistical analyses were performed applying ANOVA and generalised linear mixed-effect model (GLMM; significant p-values <0.05).
**Results or Findings:** 48 aneurysms from 44 patients were identified. Pre-contrast BB signal was observed in the 50% of the aneurysms, and significantly associated with baseline aneurysm size, but not with angiographic features of occlusion status or coil packing density. BB-enhancement was detectable in 31 aneurysms (65%). Angiographically incomplete aneurysmal occlusion and reduced coil packing density associated with enhancement at the neck and periphery of the aneurysm.
**Conclusion:** BB-enhancement, a frequent finding in coiled aneurysms, is associated with incomplete aneurysmal occlusion and reduced coil packing density at DSA, likely reflecting incompletely suppressed flow within the aneurysm.

**Limitations:** The study was limited by sample size constraints as well as by the fact that the definition of site of BB enhancement is time-consuming and not straightforward.
**Ethics committee approval:** No information provided by the submitter.
**Funding for this study:** No funding was received for this study.
**Author Disclosures:**
Andrea Falini: Nothing to disclose
Nicoletta Emanuela Anzalone: Nothing to disclose
Francesco Destro: Nothing to disclose
Claudia Godi: Nothing to disclose
Enrico Tombetti: Nothing to disclose
Paolo Garofalo: Nothing to disclose
Caterina Michelozzi: Nothing to disclose
Alessandro Ambrosi: Nothing to disclose
Antonella Iadanza: Nothing to disclose

### RPS 1811-12
**Indirect flow diversion for off-centred bifurcation aneurysms and distant small-vessel aneurysms: a retrospective proof of concept study from five neurovascular centres**
S. Schob, *M-S. Schüngel*, R. Brill, M. Skalej; Halle (Saale)/DE
*(marie-sophie.schuengel@uk-halle.de)*

**Purpose:** Off-centred bifurcation aneurysms and aneurysms arising from peripheral vessel segments oftentimes cannot be treated via implanting a flow diverting stent (FDS) at the neck level for technical reasons. In those, indirect flow diversion - a FDS deployed in the main artery proximal to the parent vessel of the aneurysm - can be a viable treatment strategy.
**Methods or Backround:** Clinical data, aneurysm characteristics, anti-platelet medication, follow-up results and associated complications were recorded.
**Results or Findings:** A total of17 patients from five neurovascular centres were included. The average distance between the FDS and the aneurysm was 1.65mm. In 88.2%, aneurysm perfusion was reduced immediately after implantation. Delayed opacification (OKM A3: 11.8%), reduction in size (OKM B1-3: 29.4%) and occlusion (D1: 47.1%) were observable at the last follow-up. Any procedural related adverse events in the early phase and in the late subacute phase were not observed in any case.
**Conclusion:** Our study suggests indirect flow diversion as a viable approach for aneurysms arising from bifurcations involving small, efferent branches and aneurysms arising from a peripheral portion of small cerebral arteries. The therapeutic effect is based on the progressive deconstruction of the aneurysm, and potentially its parent vessel. However, careful evaluation of the individual collateral situation is important for treatment success in order to prevent thrombo-embolic adverse events. Still, disconnecting the Circle of Willis with flow diverting technology should be considered a functionally significant strategy for aneurysm treatment.
**Limitations:** The study enrolled a small patient cohort only and a variety of different haemodynamic devices. Validation with larger studies and long term outcomes is warranted.
**Ethics committee approval:** No information provided by the submitter.
**Funding for this study:** No funding was received for this study.
**Author Disclosures:**
Martin Skalej: Nothing to disclose
Richard Brill: Nothing to disclose
Stefan Schob: Nothing to disclose
Marie-Sophie Schüngel: Nothing to disclose

**10:30-12:00** **Research Stage 4**

### Research Presentation Session: Oncologic Imaging

### RPS 1816
### Advances in MRI for oncological imaging

**Moderator**
H.-P. Schlemmer; Heidelberg/DE
**Author Disclosures:**
Heinz-Peter Schlemmer: Advisory Board: Bayer Healthcare

### RPS 1816-2
**Response evaluation of cancer therapeutics (RESPECT-MI) in metastatic breast cancer (MBC): preliminary data from a whole body magnetic resonance imaging (WB-MRI) study**
*R. Maggioni*, P. Pricolo, A. Colombo, P. Summers, G. Petralia; Milan/IT

**Purpose:** To assess whether WB-MRI allows an earlier identification of progressive disease (PD) in comparison to CT scans in patients with MBC.
**Methods or Backround:** The inclusion criteria were: patients with MBC and at least one bone metastasis (with or without soft tissue metastases) and beginning of a new systemic treatment. All patients underwent assessments by CT scans and WB-MRI every 12 weeks until week 96, then every 24 weeks until clinical or radiological PD was evident. A bone scan or fluorodeoxyglucose (FDG)-positron emission tomography (PET) was performed at the baseline and when PD was evident. The reporting radiologist of the WB-MRI was blinded to the results of the CT scans and the reporting radiologist of the CT scans was blinded to the results of the WB-MRI. Neither treating clinicians nor patients were blinded at any point.
**Results or Findings:** A total of 39 patients were recruited between September 2019 and July 2022, but only 31 patients in the trial for at least 24 weeks were considered for preliminary data. Of these 31 participants, 10 patients were excluded for methodological errors and 3 patients were excluded for drop-out. Of the remaining 18 participants, 9 patients were stable or in response to therapy, while 9 patients had PD at imaging, identified with WB-MRI only in 4 participants (44.4%) and with both CT scans and WB-MRI in 5 participants (55.56%); none had PD at CT scans only.
**Conclusion:** WB-MRI enabled identification of PD before CT scans in 44.4% of patients with MBC. Early identification of PD is important to avoid delays in shifting to the subsequent treatment.
**Limitations:** More data are needed.
**Ethics committee approval:** The local ethics committee approved this monocentric prospective study and waived the necessity for specific consent. All patients provided written informed consent for the performance of the WB-MRI, as well as for the use of their clinical and medical information for research and scientific dissemination purposes.
**Funding for this study:** Funding was received from the IEO (European Institute of Oncology).
**Author Disclosures:**
Roberta Maggioni: Nothing to disclose
Giuseppe Petralia: Nothing to disclose
Paul Summers: Owner: QMRI Tech
Paola Pricolo: Nothing to disclose
Alberto Colombo: Nothing to disclose

### RPS 1816-3
**MRI proton density fat fraction for estimation of tumour grade in steatotic hepatocellular carcinoma**
*D. Kurt*[1], P. A. Kupczyk[1], C. Endler[1], J. A. Luetkens[1], G. M. Kukuk[2], U. I. Attenberger[1], C. C. Pieper[1]; [1]Bonn/DE, [2]Graubünden/CH

**Purpose:** Image-based detection of intralesional fat in focal liver lesions has been established in diagnostic guidelines as a feature indicative of hepatocellular carcinoma (HCC) and associated with a favuorable prognosis. Given recent advances in MRI-based fat quantification techniques, we investigated a possible relationship between intralesional fat content and histologic tumour grade in steatotic HCCs.
**Methods or Backround:** Patients with histopathologically confirmed HCC and prior MRI with proton density fat fraction (PDFF) mapping were retrospectively identified. Intralesional fat of HCCs was assessed using a ROI-based analysis and the median fat fraction of steatotic HCCs was compared between tumour grades G1-3 with nonparametric testing. ROC analysis was performed in case of statistically significant differences ($p<0.05$). Subgroup analysis was conducted for patients with and without liver steatosis.
**Results or Findings:** A total of 57 patients with steatotic HCCs (total of 62 lesions) were eligible for analysis. The median fat fraction was significantly higher for G1 lesions (median [interquartile range], 7.9% [6.0–10.7%]) than for G2 (4.4% [3.2–6.6%]; P=.001) and G3 lesions (4.7% [2.8–7.8%]; P=.036). PDFF was a good discriminator between G1 and G2/3 lesions (AUC .81; cut-off 5.8%, sensitivity 83%, specificity 68%). In patients with liver steatosis, intralesional fat content was higher than in the overall sample, with PDFF performing better in distinguishing between G1 and G2/3 lesions (AUC .92; cut-off 8.8%, sensitivity 83%, specificity 91%).
**Conclusion:** Quantification of intralesional fat using MRI PDFF mapping allows distinction between well and less differentiated steatotic HCCs. Therefore, further investigation of intratumoural fat content as a potential prognostic indicator of treatment response is encouraged.
**Limitations:** Identified limitations were: (1) selection bias, (2) sampling bias, (3) sample size, (4) the use of two different MRI field strengths, and (5) the threshold of 2.2% for PDFF as a definition of steatotic HCCs was empirical and extrapolated from the literature.
**Ethics committee approval:** No information provided by the submitter.
**Funding for this study:** No funding was received for this study.
**Author Disclosures:**
Patrick Arthur Kupczyk: Nothing to disclose
Julian Alexander Luetkens: Nothing to disclose
Christoph Endler: Nothing to disclose
Guido Matthias Kukuk: Nothing to disclose
Ulrike I. Attenberger: Nothing to disclose
Claus Christian Pieper: Nothing to disclose
Darius Kurt: Nothing to disclose

### RPS 1816-4

**Impact of 18F-FDG PET/MRI on therapeutic management of women with newly diagnosed breast cancer: results from double-center trial**

*J. Kirchner*[1], L. Umutlu[2], N-M. Bruckmann[1], J. Morawitz[1], K. Jannusch[1], K. Herrmann[2], G. Antoch[1], C. Buchbender[1]; [1]Düsseldorf/DE, [2]Essen/DE
*(Julian.Kirchner@med.uni-duesseldorf.de)*

**Purpose:** To investigate whether 18F-FDG-PET/MRI in addition to (guideline-recommended) conventional staging leads to changes in therapeutic management in patients with newly diagnosed breast cancer and compare the diagnostic accuracy of 18F-FDG-PET/MRI to conventional staging for determining the UICC-stage.
**Methods or Backround:** In this prospective study a total of 208 women with newly diagnosed, therapy-naive breast cancer were enrolled. All patients underwent conventional staging as recommended by the current ESMO-guideline and a whole-body 18F-FDG-PET/MRI with a dedicated breast examination. A multidisciplinary tumour board served to determine two different therapy recommendations for each patient, (1) based on conventional staging alone and (2) based on the combined assessment of conventional staging and 18F-FDG-PET/MRI examinations. Major changes in therapy recommendations and differences between the conventional staging algorithm and 18F-FDG-PET/MRI for determining the correct UICC-stage were evaluated.
**Results or Findings:** Major changes in therapeutic management based on the combined assessment of conventional staging and 18F-FDG-PET/MRI were detected in 5 out of 208 patients, amounting to changes in therapeutic management in 2.4% (95% CI 0.78%; 5.2%) of the study population. In determining the UICC-stage the guideline-based staging algorithm and 18F-FDG-PET/MRI were concordant in 135 of 208 (64.9%; 95% CI: 58%; 71.4%) patients. The conventional guideline algorithm correctly determined the UICC stage in 130 of 208 (62.5%; 95% CI: 55.5%; 69.1%) patients, and 18F-FDG-PET/MRI correctly determined the UICC stage in 170 of 208 (81.9 %; 95% CI: 75.8%; 86.7%) patients.
**Conclusion:** Despite the diagnostic superiority of 18F-FDG-PET/MRI over conventional staging in determining the correct UICC-stage, the current (guideline-recommended) conventional staging algorithm is sufficient for adequate therapeutic management of patients with newly diagnosed breast cancer and 18F-FDG-PET/MRI does not have an impact on patient management.
**Limitations:** No long-term patient outcome was assessed.
**Ethics committee approval:** No information provided by the submitter.
**Funding for this study:** Funding was received from Deutsche Forschungsgemeinschaft (BU3075/2-1; BU3075/2-2; & KI2434/1-2).
**Author Disclosures:**
Ken Herrmann: Nothing to disclose
Christian Buchbender: Nothing to disclose
Lale Umutlu: Nothing to disclose
Julian Kirchner: Nothing to disclose
Gerald Antoch: Nothing to disclose
Janna Morawitz: Nothing to disclose
Kai Jannusch: Nothing to disclose
Nils-Martin Bruckmann: Nothing to disclose

### RPS 1816-5

**The value of quantitative MR elastography-based stiffness for assessing the microvascular invasion grade in hepatocellular carcinoma**

L. Zhang, M. Li, *J. Wang*; Guangzhou/CN

**Purpose: Purpose:** to evaluate the potential diagnostic value of MR elastography (MRE)-based stiffness to noninvasively predict the microvascular invasion (MVI) grade in hepatocellular carcinoma (HCC).
**Methods or Backround:** Methods: 185 patients with histopathology-proven HCC who underwent MRI and MRE examinations before hepatectomy were retrospectively enrolled. According to the three-tiered MVI grading system, the MVI was divided into negative-MVI (n=89) and positive-MVI (n=96) groups, and the latter group was categorised into mild-MVI (n=49) and severe-MVI (n=47) subgroups. Logistic regression and area under the receiver operating characteristic curve (AUC) analyses were used to determine the predictors associated with MVI grade and analyse their performances, respectively.
**Results or Findings:** Results: among the 185 patients, tumour size ≥50 mm (p=0.031), tumour stiffness (TS)/liver stiffness (LS) >1.47 (p=0.001), TS >4.33 kPa (p<0.001), and non-smooth tumour margin (p=0.006) were significant independent predictors for positive-MVI. Further analysing the subgroups, tumour size ≥50 mm (p<0.001), TS >5.35 kPa (p=0.001) and AFP level >400 ng/mL (p=0.044) were independently associated with severe-MVI. The models incorporating MRE and clinical-radiological features together performed better for evaluating positive-MVI (AUC: 0.846) and severe-MVI (AUC: 0.802) than the models using clinical-radiological predictors alone (AUC: positive-/severe-MVI, 0.737/0.743).
**Conclusion:** MRE-based stiffness was an independent predictor for both the positive-MVI and severe-MVI.
**Limitations:** First, this was a retrospective single-centre study, so bias in the patient population and pathological specimens was inevitable. Second, we excluded HCC tumours < 2 cm. Therefore, the conclusions of this study may not be generalisable to all HCCs. Finally, we only conducted a single-institute study and did not validate the positive-MVI and severe-MVI models, so these models require further external validation to examine their transferability to different populations.
**Ethics committee approval:** No information provided by the submitter.
**Funding for this study:** Funding was received from the National Natural Science Foundation of China, grant number: 91959118 (JW).
**Author Disclosures:**
Jin Wang: Nothing to disclose
Lina Zhang: Nothing to disclose
Mengsi Li: Nothing to disclose

### RPS 1816-6

**Clinical impact of whole-body MRI as a problem solving tool in patients with high risk colorectal cancer**

J. Willemse, M. Lahaye, N. Kok, A. Aalbers, G. L. Beets, C. Rijsemus, M. Maas, R. G. H. Beets-Tan, *D. M. J. Lambregts*; Amsterdam/NL

**Purpose:** To investigate the clinical impact of whole-body MRI including DWI (DWI-MRI) as a problem solving tool in patients with high risk primary or (suspected) recurrent colorectal cancer (CRC).
**Methods or Backround:** Retrospective study of n=59 CRC patients who underwent second line DWI-MRI following routine routine CT (n=31), PET-CT (n=1) or both (n=17) because of high risk primary CRC (with suspected disseminated disease) or suspected recurrent CRC during FU. Two readers in consensus interpreted the clinical imaging reports of all (PET-)CT and DWI-MRIs and converted the free text reports of each described lesion into a confidence score (0=definitely benign to 4=definitely malignant). In case of any discrepant findings ((PET-)CT score 0-2 converted to DWI-MRI score 3-4; (PET-)CT score 2-4 converted to DWI-MRI score 0-1) or extra findings (DWI-MRI score 2-4 not identified on (PET-)CT), this was categorised as "impact". Cases with no discrepant/extra findings were classified as "no impact". Results were compared with histology or clinical follow-up as a standard of reference.
**Results or Findings:** DWI-MRI had impact in 28/59 (47%) and no impact in 31/59 (53%) cases. In 10/28 impact cases, DWI-MRI found extra lesions (16 in total); in 12/28 there were discrepant findings (total 17 lesions); in 6/28 both discrepant and extra findings occurred (total 15 lesions). Of the 48 discrepant/extra lesions, DWI-MRI was confirmed to be correct in 28 (58%) and incorrect in 7 (15%); in 13 (27%) no standard of reference was available. Discrepant and extra lesions mainly concerned peritoneal depositions (19), lymph nodes (7) and hepatic lesions (6).
**Conclusion:** Addition of DWI-MRI to routine CT and/or PET/CT had an impact in approximately half of the studied cases and should thus be considered as a problem solving tool in patients with high risk or suspected recurrent CRC.
**Limitations:** An identified limitation was the retrospective nature of this study.
**Ethics committee approval:** No information provided by the submitter.
**Funding for this study:** Not applicable.
**Author Disclosures:**
Max Lahaye: Nothing to disclose
Monique Maas: Nothing to disclose
Doenja Marina Johanna Lambregts: Nothing to disclose
Charlotte Rijsemus: Nothing to disclose
Jeroen Willemse: Nothing to disclose
Niels Kok: Nothing to disclose
Regina G. H. Beets-Tan: Nothing to disclose
Geerard L. Beets: Nothing to disclose
Arend Aalbers: Nothing to disclose

### RPS 1816-7

**Diffusion-weighted MRI after radiofrequency ablation for hepatocellular carcinoma**

*H. Albalshy*; Shebeen El-Kom/EG
*(hebaelbalshy@gmail.com)*

**Purpose:** To evaluate the role of diffusion-weighted (DW) MRI in patients with hepatocellular carcinoma (HCC) treated by radiofrequency ablation (RFA).
**Methods or Backround:** This prospective study was carried out on 30 patients with HCC. The results of DW MRI were compared with the triphasic CT results. An MRI follow-up scan was performed two weeks after RFA. Both included in-phase/out-phase sequences, T2-weighted imaging (WI), and DWI. Analysis was carried out for the size and the enhancement pattern of the treated lesions confirmed on subtraction images, followed by calculation of the maximum transverse dimension of the enhancing tumour tissue after RFA. The mean ADC of the focal lesion was calculated by positioning multiple regions of interest over the tumour.
**Results or Findings:** Abdominal triphasic spiral CT for 30 patients showed post-treatment adequately managed lesions in 18 (60%) patients and residual lesions (not adequately manages lesions) in 12 (40%) patients. On comparing the DWI findings with abdominal CT findings after RFA, there were 11 (91.7%) true residual lesions and 18 (100%) true adequately managed lesions. According to DW MRI, the mean ADC value in residual lesions was significantly lower than the mean ADC value in ablated lesions (P<0.001). The mean ADC value in patients with residual lesions was 0.98 ± 0.09×103 mm²/s whereas the mean ADC value in patients with adequately managed lesions was 1.33 ± 0.16×103 mm²/s
**Conclusion:** DWI is a promising tool in the evaluation of the post-RFA HCC and can provide information about molecular tissue characteristics.
**Limitations:** Despite using the breath-hold examination technique, some patients had to be excluded because of motion artefacts.
**Ethics committee approval:** By ethical committee of Menofia faculty of medicine.
**Funding for this study:** Not applicable.
**Author Disclosures:**
Heba Albalshy: Nothing to disclose

### RPS 1816-8

**ADC measurement in whole body MRI for multiple myeloma: a three b-values vs. two b-values DWI comparison**

*M. Zerunian*, B. Masci, M. Polici, F. Pucciarelli, A. Del Gaudio, N. Ubaldi, D. De Santis, D. Caruso, A. Laghi; Rome/IT
*(marta.zerunian@gmail.com)*

**Purpose:** To assess apparent diffusion coefficient (ADC) values in whole body magnetic resonance imaging (WB-MRI) reconstructed from diffusion-weighted imaging (DWI) with three b-values (50-500-1000s/mm2) compared with two b values (50-1000s/mm2).
**Methods or Backround:** Thirty-seven consecutive patients (20 female, 57±14 years) with a diagnosis of multiple myeloma who underwent WB-MRI (1.5T) from March 2021 to May 2022 were retrospectively included. Exclusion criteria were: contraindications to MRI and severe motion artefacts on DWI. WB-MRI protocol included axial DWI sequences acquired with three b-values (50-500-1000s/mm2); then, ADC maps were reconstructed with both three b-values (ADC3b) and two b-values (ADC2b) excluding b500 s/mm2. A radiologist evaluated average ADC values of focal bone marrow lesions by placing regions of interest (ROIs, area 1cm2) in both ADC3b and ADC2b; a focal bone lesion was considered when ADC values were in the range 700–1400 µm2/sec according to MY-RADS. Then, ROIs were placed in normal bone marrow in comparison in both ADC maps to also assess healthy tissue. P<0.05 was considered significant.
**Results or Findings:** A total of 278 bone lesions and 251 healthy ROIs were assessed. The objective analysis showed significant differences of mean ADC values between bone lesions in ADC3b and ADC2b maps (960.5±215.5 µm2/sec vs. 935.1±216.5 µm2/sec, P<0.0001) and in healthy bone marrow ROIs (616.2± 294.4 µm2/sec vs. 602.9± 292.4 µm2/sec, P<0.0001). Despite significant differences almost all of the analysed ROIs were in the same range of ADC values for bone lesions/healthy tissue; however, for borderline values (650-750 µm2/sec) ADC3b showed higher values considered as bone lesions 7/278 ROIs (>700µm2/sec) compared with ADC2b (<700µm2/sec), which were then confirmed as pathological bone lesions by a panel of three expert radiologists who assessed the entire WB-MRI protocol.
**Conclusion:** ADC2b values are comparable with ADC3b, except for borderline values (650-750 µm2/sec).
**Limitations:** An identified limitation was the small sample population.
**Ethics committee approval:** This study was approved by the local institutional review board; written informed consent obtained from all participants.
**Funding for this study:** No funding was received for this study.
**Author Disclosures:**
Damiano Caruso: Nothing to disclose
Benedetta Masci: Nothing to disclose
Francesco Pucciarelli: Nothing to disclose
Marta Zerunian: Nothing to disclose
Nicolò Ubaldi: Nothing to disclose
Domenico De Santis: Nothing to disclose
Andrea Laghi: Nothing to disclose
Antonella Del Gaudio: Nothing to disclose
Michela Polici: Nothing to disclose

### RPS 1816-9

**Is bone marrow fat fraction a surrogate of marrow cellularity? A quantitative whole spine T2 Dixon study**

A. Belassein[1], *C. Mourad*[2], S. Aubry[1], P. Omoumi[3]; [1]Besançon/FR, [2]Beirut/LB, [3]Lausanne/CH

**Purpose:** To correlate bone marrow fat fraction (BMFF) assessed by T2-weighted Dixon MRI with bone marrow cellularity at biopsy.
**Methods or Backround:** This is an IRB-approved bicentric retrospective study. Patients with multiple myeloma for whom TSE T2-weighted Dixon sequences and bone marrow biopsy were available (study group) were included. A control group consisted of age- and sex-matched subjects who had TSE T2-weighted Dixon of the spine. One radiologist calculated fat fractions (FF) from regions of interest (ROI) on water-only and fat-only Dixon images in vertebral bodies (one cervical, dorsal, lumbar, and sacral) that did not contain a focal lesion. In every patient, bone marrow fat fraction (BMFF) was calculated by averaging all FF measurements. Myeloma patients were classified according to their bone marrow infiltration patterns. Bone marrow cellularity, bone marrow plasma cell percentage (BMPC%) and prognosis scores were obtained by reviewing medical records.
**Results or Findings:** There were 59 MM patients (mean age 66.76 years ± 11.57 [SD]; 38 men) and 55 control subjects (mean age 64.09 years ± 9.92 [SD]; 33 men). There was a significant difference in BMFF between myeloma patients and control subjects (p<0.0001). BMFF was negatively correlated with BMPC% (ρ=-0.67; p<0.0001) and marrow cellularity (ρ=-0.79; p<0.0001). This negative correlation was present with variegated or diffuse pattern (ρ=-0.54; p=0.0243) and multifocal pattern (ρ=-0.66; p<0.0001) but not in MM patients with normal MRI (ρ=-0.48; p=0.1601).
**Conclusion:** BMFF may be used as a surrogate of marrow cellularity in MM patients with abnormal MRI. Its potential use as a biomarker for therapeutic follow-up remains to be determined.
**Limitations:** Identified limitations were: (1) this was a retrospective study, (2) bone marrow biopsy/aspirate was not obtained from all vertebral bodies, and (3) bone marrow biopsy/aspirate was not obtained from the control group.
**Ethics committee approval:** No information provided by the submitter.
**Funding for this study:** No funding was received for this study.
**Author Disclosures:**
Abigael Belassein: Nothing to disclose
Charbel Mourad: Nothing to disclose
Patrick Omoumi: Nothing to disclose
Sébastien Aubry: Nothing to disclose

### RPS 1816-10

**EMVI detection in locally advanced rectal cancer after pCRT with T2, DWI and contrast-enhanced sequences: a comparison**

*R. Angelone*, F. Crimì, Q. R. Bao, A. Corso, G. Cabrelle, F. Vernuccio, G. Spolverato, S. Pucciarelli, E. Quaia; Padua/IT
*(raimondo.angelone@studenti.unipd.it)*

**Purpose:** The aim of this study was to determine the accuracy of three state-of-the-art MRI sequences for the detection of extramural venous invasion (EMVI) in locally advanced rectal cancer (LARC) patients after preoperative chemoradiotherapy (pCRT)
**Methods or Backround:** This retrospective study included 103 patients (median age 66 years old [43-84]) surgically treated with pCRT for LARC and submitted to preoperative contrast-enhanced pelvic MRI after pCRT. T2-weighted, DWI and contrast enhanced sequences were evaluated by two radiologists with expertise in abdominal imaging, blinded to clinical and histopathological data. Patients were scored according to the probability of EMVI presence on each sequence using a grading score ranging from 0 (no evidence of EMVI) to 4 (strong evidence of EMVI). Results from 0 to 2 were ranked as EMVI negative and from 3 to 4 as EMVI positive. ROC curves were drawn for each technique, using histopathological results as reference standard.
**Results or Findings:** T2-weighted, DWI and contrast enhanced sequences demonstrated an area under the ROC curve (AUC) respectively of 0.610 (95% CI: 0.509-0.704), 0.729 (95% CI: 0.633-0.812), and 0.624, (95% CI: 0.523-0.718). The AUC of DWI sequence was significantly higher than that of T2-weighted (p=0.0494) and contrast enhanced (p=0.0315) sequences.
**Conclusion:** DWI is more accurate than T2-weighted and contrast enhanced sequences for the identification of EMVI following pCRT in LARC patients.
**Limitations:** This is a single-centre retrospective study, but we achieved a large study population analysing a cohort of patients assessed during an 11-year timespan. Being a retrospective study, we didn't achieve a perfect pathologic-radiologic matching, but the accurate reporting of the position of the EMVI in the MRI images and histopathological examination allowed for good matching of the data.
**Ethics committee approval:** Data were processed in aggregated and anonymised form. Patients' identity can not be retrieved from the study. Data were collected during normal clinical practice. Personal data were treated in compliance with the Regulation (EU) 2016/679.
**Funding for this study:** Open access funding provided by Università degli Studi di Padova within the CRUI-CARE Agreement.
**Author Disclosures:**
Federica Vernuccio: Nothing to disclose
Giulio Cabrelle: Nothing to disclose
Quoc Riccardo Bao: Nothing to disclose
Salvatore Pucciarelli: Nothing to disclose
Raimondo Angelone: Nothing to disclose
Filippo Crimì: Nothing to disclose
Emilio Quaia: Nothing to disclose
Gaya Spolverato: Nothing to disclose
Antonio Corso: Nothing to disclose

### RPS 1816-11
**Correlation between MRI, surgical and pathological peritoneal cancer index for colorectal cancer patients undergoing cytoreductive surgery and HIPEC**
C. Rijsemus, N. Kok, A. Aalbers, P. Snaebjornsson, *D. M. J. Lambregts*, R. G. H. Beets-Tan, M. Lahaye; Amsterdam/NL

**Purpose:** CRS and hyperthermic intraperitoneal chemotherapy (CRS-HIPEC) patients are selected based on the extent and location of peritoneal metastases (PM) reflected as the peritoneal cancer index (PCI). However, practice shows that lesions considered malignant by the surgeon during surgery and consequently resected may not contain viable tumour tissue at histopathology. DW-MRI is a noninvasive way to determine PM. Therefore, this study aims to investigate if the PCI determined by DW-MRI (mriPCI) correlates better with the pPCI (pathological PCI) than the sPCI (surgical PCI).
**Methods or Backround:** Patients with PM from CRC who underwent CRS-HIPEC and an MRI preoperatively were included. All initial MRI, surgical and pathological reports of patients were reviewed for the PCI. When a report did not describe the PCI, the findings were analysed by a blinded medical specialist to extract the PCI from the initial reports.
**Results or Findings:** In this study, 61 patients were included. MRI outperformed the surgeon in 31/62 patients and the surgeon outperformed the MRI in 17/62 patients. The mriPCI was similar to sPCI in 13/62 patients. The surgeon scored more regions in all patients together (206) compared to the radiologist (121) and pathologist (142). The intraclass correlation between the sPCI and pPCI is 0.94 (0.77- 0.98), between mriPCI and pPCI 0.88 (0.79- 0.93) and between sPCI and mriPCI 0.81 (0.64- 0.89).
**Conclusion:** In more than 70% of the patients, MRI had similar results or outperformed the surgeon for the extent of the PM. The agreement between the radiologist and the surgeon/pathologist was good. This means that MRI is a good noninvasive alternative to select patients for CRS-HIPEC. However, surgical inspection remains superior to MRI, especially in patients with miliary disease, explaining the higher ICC.
**Limitations:** Identified limitations were (1) the retrospective design, and (2) the sample size.
**Ethics committee approval:** Reference: IRBd20-266.
**Funding for this study:** No funding was received for this study.
**Author Disclosures:**
Max Lahaye: Nothing to disclose
Petur Snaebjornsson: Nothing to disclose
Doenja Marina Johanna Lambregts: Nothing to disclose
Charlotte Rijsemus: Nothing to disclose
Niels Kok: Nothing to disclose
Regina G. H. Beets-Tan: Nothing to disclose
Arend Aalbers: Nothing to disclose

### RPS 1816-12
**Efficiency of diffusion tensor imaging in the diagnosis of prostate cancer**
*K. Y. Karakoishin*[1], Z. J. Zholdybay[1], A. Ainakulova[1], J. Owen[2]; [1]Almaty/KZ, [2]Lexington, KY/US
*(kanat.karakoishin@yandex.ru)*

**Purpose:** Prostate cancer is one of the most common cancers in the world. One of the components of multiparametric MRI in the diagnosis of prostate cancer is DWI. However, the diagnosis of low-grade prostate cancer is still not high. Diffusion tensor imaging can provide information about the direction and magnitude of diffusion anisotropy in cancer-suspicious areas of the prostate. The aim of the study is to demonstrate the capabilities of DTI in the diagnosis of prostate cancer.
**Methods or Backround:** A total of 25 patients with suspected prostate cancer aged 48-80 years underwent multiparametric MRI including DTI. The obtained T2WI, DTI sequences were compared with each other and in accordance with the anatomical structure of the prostate gland. Prostate cancer-suspicious areas were marked as ROIs in which fractional anisotropy (FA) and mean diffusivity (MD) were measured on DTI maps. The presence or absence of prostate cancer was confirmed by a subsequent 12-point transrectal biopsy under ultrasound guidance.
**Results or Findings:** We found that cancer-suspicious areas of the prostate had high FA and low MD compared to unaltered areas. Fisher's test for FA of the central and transient zones: 0.00231, p=0.002, odds ratio: 18.667; for FA of the peripheral zone: 0.00002, p<0.001, odds ratio 70.000; for MD of the central and transient zones: 0.10759, p=0.108, odds ratio: 3.974; for MD of the peripheral zone: 0.00409, p=0.004, odds ratio: 13.000. The AUC for FA of the central and transient zones: 0.895 [0.733÷1000], p<0.001; for FA of the peripheral zone: 0.933 [0.856÷1000], p<0.001.
**Conclusion:** The results indicate the high efficiency of DTI in the diagnosis of prostate cancer.
**Limitations:** Further studies are needed to include more patients, as well as correlation analyses with ADC maps.
**Ethics committee approval:** The ethics committee of the Research Institute of Fundamental and Applied Medicine, named after B. Atchabarov.
**Funding for this study:** No funding was received for this study.
**Author Disclosures:**
Zhamilya J. Zholdybay: Nothing to disclose
Joseph Owen: Nothing to disclose
Akmaral Ainakulova: Nothing to disclose
Kanat Yessenkululy Karakoishin: Nothing to disclose

**12:30-13:30** **Research Stage 1**

### Research Presentation Session: Chest

### RPS 1904
### MRI in thoracic imaging

**Moderator**
J. E. Wildberger; Maastricht/NL
*(j.wildberger@mumc.nl)*

### RPS 1904-2
**Comparison of capability for predicting therapeutic effect of chemoradiotherapy on non-small cell lung cancer among chemical exchange saturation transfer (CEST) imaging, DWI and FDG-PET/CT**
*Y. Ohno*[1], M. Yui[2], K. Yamamoto[2], T. Yoshikawa[3], D. Takenaka[3], H. Nagata[1], T. Ueda[1], Y. Ozawa[1], H. Toyama[1]; [1]Toyoake/JP, [2]Otawara/JP, [3]Akashi/JP
*(yohno@fujita-hu.ac.jp)*

**Purpose:** To directly and prospectively compare the capability of chemical exchange saturation transfer (CEST) imaging, DWI and FDG-PET/CT for predicting the therapeutic effect of chemoradiotherapy on stage III non-small cell lung cancer (NSCLC) patients.
**Methods or Backround:** Eighty-four consecutive stage III NSCLC patients underwent CEST imaging, DWI, FDG-PET/CT and CRT. In accordance with RECIST guidelines, all patients were divided into responders (n=65) and non-responders (n=19). Magnetisation transfer ratio asymmetry (MTRasym) on CEST imaging was calculated from z-spectra at 3.5ppm, after which an MTRasym map was computationally generated. To evaluate the capability for differentiation between the two groups, MTRasym, ADC and SUVmax at primary lesion were assessed by means of ROI measurements. Student's t-test was performed to determine the difference for each index between responders and non-responders. Each threshold value determined from ROC analysis results was used to compare progression-free survival (PFS) and overall survival (OS) for the two groups as assessed by each method by using the Kaplan-Meier method followed by log rank test. Finally, a Cox proportional hazards regression analysis was used to estimate hazard ratios (HRs) for gender, age, performance status (PS), histological subtype, tumour stage, and all indexes.
**Results or Findings:** All indexes showed significant differences between responders and non-responders (p<0.0001). For each index, PFS and OS of responders were significantly longer than those of non-responders (p<0.05). MTRasym at 3.5ppm (HR=0.70, p=0.002) and SUVmax (HR=1.41, p=0.0004) were identified as significant predictors for PFS. MTRasym at 3.5ppm (HR=0.76, p=0.04) and tumour staging (HR=0.57, p=0.02) were also significant predictors for OS.
**Conclusion:** CEST imaging is equally or more useful than DWI and FDG-PET/CT for predicting the therapeutic effect of CRT on stage III NSCLC patients.
**Limitations:** The limited study sample was an identified limitation.
**Ethics committee approval:** This study was approved by the IRBs of Kobe University and Fujita Health University Hospitals.
**Funding for this study:** A research grant was received from Canon Medical Systems Corporation.
**Author Disclosures:**
Yoshiyuki Ozawa: Nothing to disclose
Kaori Yamamoto: Employee: Canon Medical Systems Corporation
Hiroshi Toyama: Research/Grant Support: Canon Medical Systems Corporation
Takahiro Ueda: Nothing to disclose
Daisuke Takenaka: Nothing to disclose
Hiroyuki Nagata: Research/Grant Support: Canon Medical Systems Corporation
Masao Yui: Employee: Canon Medical Systems Corporation
Takeshi Yoshikawa: Research/Grant Support: Canon Medical Systems Corporation
Yoshiharu Ohno: Research/Grant Support: Canon Medical Systems Corporation

### RPS 1904-3

**Foetal MRI radiomics predict gestational age based on foetal lung shape and texture**

*F. Prayer*, M. Watzenböck, D. Prayer, B. Ulm, G. Kasprian; Vienna/AT

**Purpose:** To investigate the feasibility of gestational age prediction based on in-vivo foetal MRI lung shape and texture radiomics features.
**Methods or Backround:** T2-weighted coronal foetal MRI images of foetuses with normal lung development at gestation weeks (GW) 24, 27, 30 and 33 were retrospectively identified. Cases were excluded due to incomplete lung representation, presence of motion artefacts or twin pregnancy. Foetal whole lung segmentation masks were obtained manually using ITK-Snap. Radiomics features (n=67) previously shown to be robust against test-retest image acquisitions were extracted from lung masks using pyradiomics. Gestational age in weeks was predicted based on radiomics features using elastic-net regression. Prediction accuracy against gestational age calculated from foetal ultrasound was assessed by leave-one-out cross-validation. R-squared and Pearson correlation between predicted and observed values were used as validation metrics. Data analysis was performed using Python 3.7.1.
**Results or Findings:** Out of 2680 cases, in-vivo foetal MRI data from 148 fetuses (48 at GW 24, 36 at GW 27, 30 at GW 30, and 34 at GW 33) were included. Predicted gestational age showed an excellent correlation with observed gestational age (R-squared = 0.85, Pearson r = 0,92, p < 0.001), indicating a high predictive accuracy.
**Conclusion:** Non-invasive in-vivo foetal MRI-based prediction of gestational age based on whole lung radiomics is feasible using routinely acquired T2-weighted images. In the future, the proposed technique can be used to generate gestational age-specific foetal MRI lung radiomics signatures to improve the prenatal detection of abnormal lung development.
**Limitations:** Retrospective data analysis performed at a single centre.
**Ethics committee approval:** No information provided by the submitter.
**Funding for this study:** This project received funding from the Seed Grant funding programme of the European Society of Radiology (ESR) in collaboration with the European Institute for Biomedical Imaging Research (EIBIR), kindly supported by an unrestricted, non-exclusive grant from GE Healthcare.
**Author Disclosures:**
Florian Prayer: Grant Recipient: This project has received funding from the Seed Grant funding programme of the European Society of Radiology (ESR) in collaboration with the European Institute for Biomedical Imaging Research (EIBIR) kindly supported by an unrestricted, non-exclusive grant from GE Healthcare.
Barbara Ulm: Nothing to disclose
Daniela Prayer: Nothing to disclose
Gregor Kasprian: Nothing to disclose
Martin Watzenböck: Nothing to disclose

### RPS 1904-4

**Reproducibility and feasibility of RM-FC score in a heterogeneous bronchiectasis population using a rapid and easy-to-perform MRI protocol**

C. C. Giannessi, *C. Moroni*, D. Cozzi, E. Cavigli, A. Bindi, S. Luvarà, G. Basile, L. Macconi, V. Miele; Florence/IT
*(chiaramoroni73@gmail.com)*

**Purpose:** Magnetic resonance (MR) has become a powerful diagnostic tool in young bronchiectasic patients both with and without cystic fibrosis (CF), though burdened by the need for a rapid and reproducible score to compare examinations and identify the entity of bronchial involvement. We retrospectively applied CF-RM score (a visual score based on presence and severity of bronchial-pulmonary involvement) in order to assess its interobserver reproducibility and to evaluate its correlation with main functional index of airway obstruction (FEV1).
**Methods or Backround:** We retrospectively evaluated 17 outpatients with a bronchoscopic diagnosis of chronic bronchiectasis who had undergone lung MR in the last year. The MR protocol consisted of the following sequences: a multishot TSE T2w in the axial, coronal and sagittal plane, an axial T1w in-and-out of phase and an axial DWIw. This protocol is time-saving and easily performed also on dyspnoic patients. RM-FC score was applied by two physicians (a trainee and a thoracic expert) and was correlated with FEV1.
**Results or Findings:** We found good reproducibility between the two physicians (r=0.8). CF-MR score in the overall population demonstrated weak correlation with the FEV1 but showed a strong correlation (r>0.8) when only the more severe cases were considered, identified by a FC-RM threshold of 25.
**Conclusion:** In our experience we found FC-RM score a reproducible, easy to apply tool, able to estimate the bronchial compromise in bronchiectasis patients, regardless of their etiology, especially in those with more severe bronchial compromise.
**Limitations:** We found a weaker correlation between MR-FC score and FEV1 in less severe cases. This result might be related to the lack of clear identification of air trapping in our MR images, preventing a real assessment of the severity of compromise in patients with fewer bronchial findings.
**Ethics committee approval:** No information provided by the submitter.
**Funding for this study:** No funding was received for this study.
**Author Disclosures:**
Alessandra Bindi: Nothing to disclose
Gregorio Basile: Nothing to disclose
Caterina Caterina Giannessi: Nothing to disclose
Vittorio Miele: Nothing to disclose
Silvia Luvarà: Nothing to disclose
Letizia Macconi: Nothing to disclose
Edoardo Cavigli: Nothing to disclose
Chiara Moroni: Nothing to disclose
Diletta Cozzi: Nothing to disclose

### RPS 1904-5

**Magnetic resonance imaging of pulmonary and paranasal sinus abnormalities in children with primary ciliary dyskinesia compared to children with cystic fibrosis**

*L. Wucherpfennig*[1], F. Wuennemann[2], S. Hämmerling[1], J-P. Schenk[1], H-U. Kauczor[1], M. Mall[3], M. O. Wielpütz[1], O. Sommerburg[1]; [1]Heidelberg/DE, [2]Wiesbaden/DE, [3]Berlin/DE
*(lena.wucherpfennig@med.uni-heidelberg.de)*

**Purpose:** Primary ciliary dyskinesia (PCD) and cystic fibrosis (CF) are characterised by inherited impaired mucociliary clearance leading to chronic progressive lung disease as well as chronic rhinosinusitis (CRS). However, comparative magnetic resonance imaging (MRI) studies regarding pulmonary and paranasal sinus abnormalities and their association in children with PCD and with CF, are lacking.
**Methods or Backround:** A total of 18 children with PCD (mean age 8.5±5.8 years, range 0-18 years) and 36 age-matched children with CF (mean age 8.5±5.8 years, range 0-18 years) underwent same-session chest and paranasal sinus MRI. Pulmonary and paranasal sinus abnormalities were assessed using validated chest MRI scores and previously evaluated CRS-MRI scores.
**Results or Findings:** Chest MRI scores were similar in children with PCD and in children with CF (P=0.420-0.601), while consolidations were more prevalent in children with PCD (P<0.05). In children with PCD as well as in children with CF, chest MRI global scores correlated with FEV1% (r=-0.512 to -0.687, P<0.05) and chest MRI perfusion scores with LCI (r=0.489-0.568, P<0.05). CRS-MRI sum scores, especially mucopyoceles, were lower in children with PCD than in children with CF (P<0.05), while opacification of mastoid cells was more prevalent in children with PCD (P<0.001). CRS-MRI sum scores did not correlate with chest MRI scores in children with PCD (P=0.641-0.863), while it correlated with MRI morphology scores in children with CF (P<0.01).
**Conclusion:** MRI detects differences in chest and paranasal sinus abnormalities between children with PCD and children with CF. Our data support the role of same-session chest and paranasal sinus MRI as a sensitive non-invasive modality for disease monitoring in PCD and CF.
**Limitations:** Due to rarity of PCD, the number of patients was moderate.
**Ethics committee approval:** This study was approved by the institutional ethics committee (S-211/2011, S-370/2011).
**Funding for this study:** This study was supported by grants from the German Federal Ministry of Education and Research (82DZL004A1, 82DZL009B1).
**Author Disclosures:**
Olaf Sommerburg: Nothing to disclose
Mark O. Wielpütz: Grant Recipient: This study was supported by grants from the German Federal Ministry of Education and Research (BMBF) (82DZL004A1, 82DZL009B1). Funders had no involvement in the collection, analysis and interpretation of data, in the writing of the report and in the decision to submit.
Lena Wucherpfennig: Nothing to disclose
Susanne Hämmerling: Nothing to disclose
Felix Wuennemann: Nothing to disclose
Hans-Ulrich Kauczor: Nothing to disclose
Marcus Mall: Nothing to disclose
Jens-Peter Schenk: Nothing to disclose

### RPS 1904-6

**Whole-body MRI: a one-stop shop for staging lung cancer**

*A. Antony*, S. H. Chandrashekhara, R. Sharma, A. S. Bhalla, D. Kandasamy, S. Shamim, P. Singh Malik, S. Kumar Arava; New Delhi/IN

**Purpose:** The second most common cancer and the leading cause of cancer-related deaths worldwide is lung cancer. Lung cancer's stage plays an imperative role in determining its outcome. Current staging pathways with 18F-FDG PET/CT have several limitations, which delay treatment initiation. WB-MRI with diffusion weighted imaging (DWI) is a non-invasive, radiation-free imaging modality for evaluating primary and metastatic lesions. Our study examined the role of WB-MRI for the staging of lung cancer to reduce the delay in treatment initiation.

**Methods or Backround:** A whole-body MRI with diffusion weighted imaging (DWI) using 1.5 T and 18F-FDG PET/CT was performed on 31 patients with biopsy-proven lung cancer, which were staged independently by two radiologists and one nuclear medicine physician. The diagnostic accuracy of conventional WB-MRI without DWI and WB-MRI with DWI was calculated and compared considering 18F-FDG PET/CT as the gold standard. We evaluated the agreement between WB-MRI and 18F-FDG PET/CT.
**Results or Findings:** WB-MRI was superior in terms of delineating fat planes to 18F-FDG PET/CT. In addition, WB-MRI with DWI provided better diagnostic accuracy than WB-MRI without DWI in N and M staging. When compared to 18F-FDG PET/CT, which failed to detect CNS metastases, WB-MRI identified the presence of metastases to the brain in both cases. Contrast administration was not found to increase the accuracy of lung cancer staging. There was a good correlation between WB-MRI and 18F-FDG PET/CT in terms of overall TNM staging.
**Conclusion:** WB-MRI with DWI is an ideal radiation-free imaging tool for the staging of lung cancer with good diagnostic accuracy which will result in earlier initiation of treatment.
**Limitations:** The study had a small sample size and was a single-centre study. Pathological confirmation of suspected nodes was not available.
**Ethics committee approval:** No information provided by the submitter.
**Funding for this study:** No funding was received for this study.
**Author Disclosures:**
Ajith Antony: Nothing to disclose
Ashu Seith Bhalla: Nothing to disclose
S.H. Chandrashekhara: Nothing to disclose
S.A. Shamim: Nothing to disclose
Sudhir Kumar Arava: Nothing to disclose
Prabhat Singh Malik: Nothing to disclose
Raju Sharma: Nothing to disclose
Devasenathipathy Kandasamy: Nothing to disclose

**12:30-13:30 Research Stage 2**

### Research Presentation Session: Hybrid, Molecular and Translational Imaging

### RPS 1906

### Insights into pathophysiology with MRI and PET

**Moderator**
F. A. Gallagher; Cambridge/UK
*(fag1000@cam.ac.uk)*
**Author Disclosures:**
Ferdia Aidan Gallagher: Advisory Board: EIBIR; Board Member: World Molecular Imaging Society; Consultant: AstraZeneca; Grant Recepient: GlaxoSmithKline; Research Grant/Support: GE Healthcare

### RPS 1906-2

**[68Ga]Ga-Pentixafor-PET/MRI for treatment response assessment in mantle cell lymphoma: comparison between changes in CXCR4 expression and lesion size**
*M. E. Mayerhöfer*[1], M. Raderer[2], M. Hacker[2], M. Weber[2], L. Nics[2], D. Leithner[1], H-J. Wester[3], A. Haug[2]; [1]New York, NY/US, [2]Vienna/AT, [3]Munich/DE

**Purpose:** CXCR4 imaging with [68Ga]Ga-Pentixafor-PET has shown promise for assessment of haematologic malignancies, in particular before treatment. The aim of this exploratory study was to compare CXCR4 imaging with [68Ga]Ga-Pentixafor-PET to anatomic MRI for treatment response assessment in patients with mantle cell lymphoma (MCL).
**Methods or Backround:** Twenty-two post-treatment [68Ga]Ga-Pentixafor-PET/MRI scans of 16 patients (7 women and 9 men; mean age, 69.9±7.9) with a total of 67 target lesions on baseline PET/MRI were analysed. Rates of complete remission per lesion and per scan, according to MRI (based on lesion size) and [68Ga]Ga-Pentixafor-PET (based on SUV decrease to lower than liver and blood pool uptake) were compared using McNemar tests. T-tests and Pearson correlation coefficients (r) were used to compare rates of change in lesion diameter products (DP) on MRI, and standardised uptake values (SUVmax, SUVmean) on PET, relative to baseline.
**Results or Findings:** On post-treatment PET/MRI, more lesions were rated as CR based on [68Ga]Ga-Pentixafor-PET criteria (58/89) than based on MRI criteria (43/89) (P=0.012). Complete remission after treatment was observed more frequently on [68Ga]Ga-Pentixafor-PET (11/22 scans, 50.0%) than on MRI (5/22 scans, 22.7%) (P=0.031). Rates of change did not differ significantly between lesion DP (-69.20±34.62%) and SUVmax (-64.59±50.78%, P=0.22), or DP and SUVmean (-60.15±64.58, P=0.064). Correlations were strong between DP and SUVmax (r=0.71, P<0.001) and DP and SUVmean (r=0.73, P<0.001).
**Conclusion:** In patients with mantle cell lymphoma, [68Ga]Ga-Pentixafor-PET may be useful for treatment response assessment. Specifically, [68Ga]Ga-Pentixafor-PET may detect complete remission status earlier than anatomic MRI using lesion size criteria.
**Limitations:** The cohort size was limited due to the exploratory study design.
**Ethics committee approval:** No information provided by the submitter.
**Funding for this study:** This research was funded in part through the NIH/NCI Cancer Center Support Grant P30 CA008748.
**Author Disclosures:**
Alexander Haug: Other: Pentixapharm
Marius E. Mayerhöfer: Speaker: Siemens Healthneers Speaker: Brsitol Myers Squibb Speaker: GE Healthcare
Lukas Nics: Nothing to disclose
Hans-Jürgen Wester: Nothing to disclose
Markus Raderer: Speaker: Celgene, Ipsen, Novartis, and Eisai
Markus Hacker: Nothing to disclose
Doris Leithner: Nothing to disclose
Michael Weber: Nothing to disclose

### RPS 1906-3

**[18F]FDG PET/MRI in children suffering from lymphoma: does MRI contrast media make a difference?**
K. Jannusch, J. Morawitz, K. Herrmann, L. Umutlu, G. Antoch, J. Kirchner, *N-M. Bruckmann*; Düsseldorf/DE

**Purpose:** To answer the question if paediatric lymphoma patients profit from an MRI contrast agent application at [18F]FDG PET/MRI staging.
**Methods or Backround:** 105 [18F]FDG-PET/MRI datasets of 32 children (mean age 14 years ± 3 years) with newly diagnosed lymphoma were included. Two reading protocols were analysed: (i) PET/MRI-1 comprising unenhanced T2w and/or T1w and diffusion-weighted imaging in combination with a [18F]FDG-PET dataset, and (ii) PET/MRI-2 comprising an additional contrast enhanced T1w sequence. For each examination at both reading protocols tumour stage was determined following the revised IPNHLSS (International-Pediatric-NHL-Staging-System). Afterwards, lesion-based analysis was performed to elaborate differences between both reading protocols. Follow-up, prior examinations as well as histopathology served as reference standard.
**Results or Findings:** In patient based analysis PET/MRI-1 and PET/MRI-2 determined correct IPNHLSS tumour stage at 90/105 (86%) paediatrics. In region based analysis both reading protocols correctly detected 119/127 (94%) lymphoma-affected regions. There were no statistically significant differences between PET/MRI-1 and PET/MRI-2 reading protocols according to patient based and region based analysis. Sensitivity, specificity, positive predictive value, negative predictive value and diagnostic accuracy for PET/MRI-1 and PET/MRI-2 reading protocol were 94%, 97%, 90%, 99%, 97%, respectively.
**Conclusion:** No beneficial effect of MRI contrast agent application at [18F]FDG-PET/MRI staging of paediatric lymphoma patients was found. Thus, switching to an contrast-free [18F]FDG-PET/MRI-protocol should be considered for all paediatric lymphoma patients.
**Limitations:** Histopathological sampling for subtype determination was available in all patients but in accordance with current ethical and clinical guidelines not every detected lesion could be sampled. As already published in numerous previous studies on hybrid imaging, a modified reference standard was applied.
**Ethics committee approval:** All procedures performed were in accordance with the ethical standards of the institutional research committee of the University Duisburg-Essen and with the principles of the 1964 Declaration of Helsinki and its later amendments.
**Funding for this study:** No funding was received for this study.
**Author Disclosures:**
Ken Herrmann: Nothing to disclose
Lale Umutlu: Nothing to disclose
Julian Kirchner: Nothing to disclose
Gerald Antoch: Nothing to disclose
Janna Morawitz: Nothing to disclose
Kai Jannusch: Nothing to disclose
Nils-Martin Bruckmann: Nothing to disclose

### RPS 1906-4

**Correlation between imaging markers derived from PET/MRI and invasive acquired biomarkers in newly diagnosed breast cancer**
*K. Jannusch*, J. Morawitz, N-M. Bruckmann, K. Herrmann, L. Umutlu, G. Antoch, J. Kirchner; Düsseldorf/DE

**Purpose:** The aim of this study was to evaluate the diagnostic potential of data derived from [18F]FDG-PET/MRI compared with invasive acquired biomarkers in newly diagnosed breast cancer.
**Methods or Backround:** A total of 136 women with newly diagnosed breast cancer (54 ± 11 years) were included. All underwent a dedicated [18F]FDG-

PET/MRI for initial staging. A tumour-adapted volume of interest was placed in the primaries and different bone marrow regions to assess PET/MRI data. Breast-cancer tumour grading and immunohistochemical biomarkers, disseminated tumour cells (DTC) as well as molecular subtypes of each women were assessed after ultrasound guided biopsy of the primaries and bone marrow aspiration. Correlations between SUV / ADC values and tumour grading / immunohistochemical biomarkers / DTC / molecular subtypes were calculated.
**Results or Findings:** Oestrogen-receptor expression was found in 99/137 (73%) and progesterone-receptor expression in 87/136 (64%) of the women. Both show significant inverse SUVmax correlation (ER: r=0.29, p<0.01; PR: r=0.25, p<0.01). HER2-receptor expression (34/136, 25%) showed no significant correlation with SUV and ADC. Ki67 (mean %: 44±27) showed a significant positive correlation with SUVmean (r=0.2, p<0.05). Molecular subtype correlates significantly positive with SUVmax and SUVmean (r=0.28, r=0.33, p's< 0.01). No correlation between tumour grading /DTC´s and SUV / ADC could be found.
**Conclusion:** The present data show a correlation between glucose metabolism and oestrogen-receptor, progesterone-receptor, Ki67 and molecular subtype and no correlation towards DTC´s. Therefore, 18F-FDG-PET/MRI may be used as a predictive tool for individual immunohistochemical biomarkers whereas it cannot replace bone marrow aspiration for the detection of DTC.
**Limitations:** Histopathological sampling was derived from a core-needle biopsy and cellularity / immunohistochemical markers may be underrepresented.
**Ethics committee approval:** All procedures performed were in accordance with the ethical standards of the institutional research committee of the University Duisburg-Essen (study number 17-7396-BO) and Düsseldorf (study number 6040R) and with the principles of the 1964 Declaration of Helsinki and its later amendments.
**Funding for this study:** Funding was received from Deutsche Forschungsgemeinschaft (BU3075/2-1 and KI2434/1-2).
**Author Disclosures:**
Ken Herrmann: Nothing to disclose
Lale Umutlu: Nothing to disclose
Julian Kirchner: Nothing to disclose
Gerald Antoch: Nothing to disclose
Janna Morawitz: Nothing to disclose
Kai Jannusch: Nothing to disclose
Nils-Martin Bruckmann: Nothing to disclose

### RPS 1906-5

### Myocardial blood flow and reserve in patients with acute coronary syndrome: comparison with cardiac magnetic resonance

*O. V. Mochula*, A. Maltseva, A. Mochula, V. Ryabov, K. W. Zavadovsky; Tomsk/RU
*(mochula.olga@gmail.com)*

**Purpose:** To assess quantitative global parameters of myocardial perfusion detected by dynamic SPECT, in patients with acute coronary syndrome; and to compare with contrast-enhanced cardiac magnetic resonance.
**Methods or Backround:** All ACS patients underwent dynamic SPECT and CMR in a period of 7±2 days after admission to the hospital. Contrast-enhanced CMR was conducted on the 1.5-Tesla scanner with an assessment of myocardial oedema (ME), microvascular obstruction (MVO) and infarct size (IS). Stress myocardial blood flow (MBF) and myocardial flow reserve (MFR) was detected by using a cardiac hybrid system with cadmium-zinc-telluride.
**Results or Findings:** The study included 59 patients (42 men, age 60.2±9.8 years). On the first step, based on ECG, patients were divided: ST segment Elevation Myocardial Infarction (STEMI), n=34; Non-ST segment Elevation Myocardial Infarction (NSTEMI), n=25. Stress MBF and MFR were significantly decreased in STEMI group (p<0.05): 0.76(0.62;1.2)vs 1.45(0.85;1.69) ml/min/g, 1.69(1.09;2.22)vs 2.11(1.78;2.63), respectively. On the second step, based on CMR, patients were divided: Ischemic type of myocardial injury, n=42; Non-ischemic type of myocardial injury, n=17. Stress MBF was significantly decreased in the first group: 0.76(0.62;1.39)vs 1.41(0.85;1.74) ml/min/g. On the third step, based on CMR, patients were divided: Transmural damage of myocardium (n=35); Non-transmural damage of myocardium (n=24). Stress MBF was significantly decreased in the first group: 1.75(1.08;2.19)vs 2.1(1.61;2.9) ml/min/g. The Spearman correlation showed that stress MBF had strong negative relationships with ME (ρ=-0.52), MVO (ρ=-0.57), IS (ρ=-0.61).
**Conclusion:** The analysis of quantitative global parameters of myocardial perfusion showed that such an approach allows for the identification of myocardial flow disturbances. Dynamic SPECT can be used to assess the severity of the disease, risk stratification and possibly prognosis in ACS patients.
**Limitations:** An identified limitation was the small size of the study population.
**Ethics committee approval:** No information provided by the submitter.
**Funding for this study:** This study was supported by a grant from the Russian Science Foundation (project N 21-75-00051, https://rscf.ru/project/21-75-00051/).
**Author Disclosures:**
Olga Vital'evna Mochula: Nothing to disclose
Andrei Mochula: Nothing to disclose
Konstantin W Zavadovsky: Nothing to disclose
Vyacheslav Ryabov: Nothing to disclose
Alina Maltseva: Nothing to disclose

### RPS 1906-6

### Development of realistic MRI phantoms to improve sequence design, signal quantification and reproducibility

P. Barkawitz[1], F. B. Schwarz[1], I. Hernandez-Giron[2], M. M. Wagner[1], M. Scheel[1], B. Hamm[1], C. Schalley[1], M. Makowski[3], *P. Jahnke*[1]; [1]Berlin/DE, [2]Leiden/NL, [3]Munich/DE

**Purpose:** To develop highly realistic phantoms simulating human anatomy and pathology for use in sequence design, validation and quantitative MR imaging.
**Methods or Backround:** We investigated an inkjet approach using gadolinium-doped ink and hydrogel print substrates to create realistic anthropomorphic phantoms for T1-weighted MR imaging. Our investigation encompassed agarose and polyacrylamide hydrogels, which were modified by coupling diethylenetriaminepentaacetic acid (DTPA) anchor molecules to bind printed gadolinium ions. We created line pair phantoms and anthropomorphic phantoms replicating patient anatomy and pathology, including different neck and liver tumours. T1 mapping values and the modulation transfer function (MTF) were analysed to evaluate phantom stability.
**Results or Findings:** Inkjet printing of gadolinium-doped ink to hydrogel substrates enabled production of highly realistic anthropomorphic phantoms with anatomical and tumour details for T1-weighted MR imaging. Hydrogel extension by a synthetically modified DTPA molecule completely restricted diffusion of printed gadolinium ions. Phantom stability was demonstrated over a period of eighteen months by reproducible T1 mapping values and MTF results. The neck and abdomen phantoms we created demonstrated flexibility of the method in producing patient- and disease-specific phantoms for use in clinical MR scanners.
**Conclusion:** Inkjet printing using gadolinium-doped ink and DTPA-coupled hydrogels enables creation of highly realistic MR phantoms. The method we present provides novel reference tools for the development, improvement and validation of sequence designs and quantitative MRI.
**Limitations:** The method is currently limited to T1-weighted imaging and future work will focus on phantom application in development, validation and improvement of MR sequences, quantitative MRI, and in particular, AI.
**Ethics committee approval:** This study was approved by the ethics committee of Charité - Universitätsmedizin Berlin.
**Funding for this study:** Funding was received from DFG grant JA2890/1-1.
**Author Disclosures:**
Bernd Hamm: Nothing to disclose
Philip Barkawitz: Nothing to disclose
Moritz Moritz Wagner: Nothing to disclose
Paul Jahnke: Patent Holder: EP3135199A1, US9924919B2, US10182786B2, DE202015104282U1, EP3556432A1, WO2019201745A1 Employee: PhantomX GmbH Founder: PhantomX GmbH
Christoph Schalley: Nothing to disclose
Felix Benjamin Schwarz: Founder: PhantomX GmbH Employee: PhantomX GmbH
Irene Hernandez-Giron: Nothing to disclose
Michael Scheel: Patent Holder: EP3135199A1, US9924919B2, US10182786B2, DE202015104282U1
Marcus Makowski: Nothing to disclose

### RPS 1906-7

### Reducing contrast agents' residuals in hospital wastewater: the GREENWATER study

*M. Zanardo*, L. Asmundo, D. Capra, R. Cardani, A. Colarieti, V. Magni, C. B. Monti, L. V. Renna, F. Sardanelli; Milan/IT
*(moreno.zanardo90@gmail.com)*

**Purpose:** The GREENWATER study aims to appraise the extent of retrievable iodinated contrast agents (ICAs) and gadolinium-based contrast agents (GBCAs) from patients' urine collected after CT and MRI examinations. Our current purpose is to provide a first glance on this initial experience.
**Methods or Backround:** After ethics committee approval, in this prospective, observational, single-centre study we enrolled outpatients aged ≥18 years, scheduled to perform a contrast-enhanced CT or MRI for any clinical indication, asking them to collect post-examination urine in dedicated canisters prolonging their hospital stay up to one hour after injection.
**Results or Findings:** After the first three months of enrolment, acceptance rate was 126/130 (96%, 95% confidence interval, CI, 94–100%). Patients' median age was 59 years (interquartile range, IQR, 47–73 years), 65 males (50%). Overall, 74 patients underwent MRI and 56 CT. In particular, 58 (45%)

Saturday patients were referred to imaging for cardiac indications, 48 (36%) for neurological indications, and 24 (18%) for other reasons. The median volume of iodine injected per patient was 22.2 g (IQR 19.2−26.0 g), whereas the median volume of gadolinium injected per patient was 1.2 mol (IQR 1.0−1.6 mol). The median volume of collected urine was 100 mL (IQR 70−144 mL).
**Conclusion:** The patients' acceptance rate was very high (over 90%), indicating a high patients' "green" awareness and interest for a sustainable radiology. Urine samples displayed sufficient volumes to allow patient-by-patient analyses for building a model to predict the amount of iodine and gadolinium retrievable using this approach.
**Limitations:** Chemical data from urine samples is not yet available, as analyses will be performed in bulk by March 2023.
**Ethics committee approval:** No information provided by the submitter.
**Funding for this study:** Funding was received from Bracco Imaging.
**Author Disclosures:**
Anna Colarieti: Nothing to disclose
Francesco Sardanelli: Research/Grant Support: Bracco
Caterina Beatrice Monti: Nothing to disclose
Laura Valentina Renna: Nothing to disclose
Moreno Zanardo: Nothing to disclose
Rosanna Cardani: Nothing to disclose
Veronica Magni: Nothing to disclose
Luigi Asmundo: Nothing to disclose
Davide Capra: Nothing to disclose

**12:30-13:30 Research Stage 3**

### Research Presentation Session: Musculoskeletal

### RPS 1910

### Musculoskeletal: arthritis, cartilage

**Moderator**
A. S. Gersing; Munich/DE

### RPS 1910-2

**Knee diameter and cross-section area measurements in MRI as new promising methods of chondromalacia diagnosis: a pilot study**
*D. A. Sieron*[1], K. Szyluk[2], D. Lukoszek[3], I. Jabłońska[4], A. Sieroń[5], A. Christe[1]; [1]Bern/CH, [2]Piekary Śląskie/PL, [3]Hanusek/PL, [4]Wysowa-Zdrój/PL, [5]Czestochowa/PL

**Purpose:** Chondromalacia often affects the knee joint. Risk factors for the development of cartilage degenerative changes include overweight, female gender and age. The use of radiological parameters to assess the knee joint is rarely reported in the scientific literature.
**Methods or Backround:** The study involved 324 patients, including 159 (49%) women and 165 (51%) men, with an age range between 8-87 years (mean: 45.1 ± 20.9). The study group had a body mass index (BMI) in the range of 14.3-47.3 (mean: 27.7 ± 5.02). A 1.5 Tesla and 3.0 Tesla (T) MRI scanner was used to assess the cartilage of the knee joint using the Outerbridge scale. The radiological parameters analysed were the Insall-Salvati index, knee surface area, knee AP (antero-posterior) maximal diameter, and knee SD (sinistro-dexter) maximal diameter.
**Results or Findings:** Parameters such as the knee surface area, knee AP maximal diameter and knee SD maximal diameter showed a significant correlation with Outerbridge Scale ($p<0.014$). The age of the patients showed a significant correlation with each knee parameter ($p<0.004$). Results of knee AP and SD maximal diameter measurements strongly depended on BMI level.
**Conclusion:** A significant relationship was found between the knee surface area, knee AP maximal diameter, and knee SD maximal diameter, and the advancement of chondromalacic changes in the knee joint, age and BMI.
**Limitations:** One of the major limitations of the study is the lack of an asymptomatic control group. In addition, information such as thickening of the knee joint bursa, Hoffa's pad, and fluid in the joint cavity, which can significantly affect radiographic findings, were not included in the analysis.
**Ethics committee approval:** No information provided by the submitter.
**Funding for this study:** This research received no external funding.
**Author Disclosures:**
Aleksander Sieroń: Nothing to disclose
Dominik Aleksander Sieron: Nothing to disclose
Dawid Lukoszek: Nothing to disclose
Izabela Jabłońska: Nothing to disclose
Karol Szyluk: Nothing to disclose
Andreas Christe: Nothing to disclose

### RPS 1910-3

**Preliminary evaluation of articular cartilage of the knee joint with T2 mapping protocol on 0.3 Tesla MRI in patients without cartilage lesion at arthroscopic evaluation: a starting point**
*M. C. Faugno*, A. Marino, G. Argento, A. Laghi; Rome/IT
*(mariacarlafaugno@gmail.com)*

**Purpose:** The aim of this study is to evaluate the reliability of T2-mapping on the evaluation of knee cartilage at low field magnet (0.3T) MRI and to compare it to arthroscopy.
**Methods or Backround:** A preliminary prospective study was conducted on 12 patients with planned arthroscopic surgery. MRI examination was performed before arthroscopy and images were analysed with dedicated software. Two radiologists quantitatively assessed the distribution of cartilage T2 relaxation time on each articular surface of the knee joint (14 regions of interest were positioned). Arthroscopic knee surgery was performed by a specialised knee orthopedic surgeon who was blinded to the MRI data.
**Results or Findings:** We evaluated a total of 168 regions of interest and arithmetic mean ±SD of T2 relaxation time was calculated. The average T2-relaxation time of the 168 regions was of 43±8 msec. Analysis performed on single regions showed slightly higher values at the level of the femoral condyles in the anterior portion (medial condyle 46±6 and lateral condyle 46±11); and slightly higher values in the medial patellar site compared to lateral (medial 41±6 and lateral 40±4). All cartilage surfaces were considered free of lesions and therefore arthroscopically classified as degree 0 according to Outerbridge classification; therefore the preliminary values found on a low-field MRI showed cartilage surface free of lesions and therefore healthy.
**Conclusion:** In our preliminary study we analysed a sample of patients considered arthroscopically free of cartilage lesions. We discovered a range of T2 relaxation values that could be considered as a starting point for the future evaluation of patients with knee chondropathy on low field MRI.
**Limitations:** Small population sample. Monocentric study.
**Ethics committee approval:** No information provided by the submitter.
**Funding for this study:** No funding was received for this study.
**Author Disclosures:**
Antonio Marino: Nothing to disclose
Andrea Laghi: Nothing to disclose
Maria Carla Faugno: Nothing to disclose
Giuseppe Argento: Nothing to disclose

### RPS 1910-4

**Quantitative T2 maps of knee articular cartilage estimated with a dictionary-based approach**
*J. M. Coelho*[1], T. T. Fernandes[2], L. Nogueira[1], A. Oliveira[1], R. G. Nunes[2]; [1]Porto/PT, [2]Lisbon/PT

**Purpose:** The T2 relaxation time is sensitive to cartilage degradation marked by loss of collagen integrity and increasing water content. Typically, quantitative T2 maps are created using multi-echo spin-echo (MESE) sequences followed by exponential fitting, but dictionary approaches produce more precise T2 values, matching the data to pre-computed echo modulation curves (EMC). This study compares T2 maps calculated with exponential fitting and EMC matching of the knee articular cartilage (KAC).
**Methods or Backround:** 10 healthy volunteers (18-40 yrs) recruited using the KOOS form to assess prior knee injuries. T2 maps of femoral and tibial KAC were obtained using a 3-T MRI Philips Achieva scanner, with a 30 slice sagittal MESE sequence with parameters: TR=2140 ms, 10 echoes (5.9:5.9:59 ms), thickness 2.5mm, 220x204 matrix, 15cm field of view, scan 9:15 min. Segmentation of eight weight-bearing KAC regions performed on three consecutive central slices of each condyle. T2 maps estimated in Python with EMC and mono-exponential (MonoExp) fit, superimposed on the MESE images to extract mean values for each segmented section. The statistical difference between T2 dictionary and monoexponential maps was evaluated with a Wilcoxon signed-rank test using SPSS (version 28, IBM).
**Results or Findings:** Global mean EMC T2 values were lower (39.6ms, sd=4.3ms) compared with the mono-exponential fit (40.8ms, sd=3.8) ($Z = -2.521$; $p < 0.05$). Mean EMC/MonoExp T2 for lateral condyle was 40.6/41.1ms and for the medial was 38.6/40.4ms, respectively.
**Conclusion:** Dictionary T2 values were closer to those found in the literature for non-MESE sequences. Quantitative T2 maps of the KAC calculated with EMC presented lower values compared with the mono-exponential fitting.
**Limitations:** The small sample size. Applying the dictionary methodology requires detailed pulse sequence knowledge.
**Ethics committee approval:** No information provided by the submitter.
**Funding for this study:** Fundação para a Ciência e a Tecnologia (FCT) - (grants 2020.05080.BD and UIDB/50009/2020).
**Author Disclosures:**
Luisa Nogueira: Nothing to disclose
António Oliveira: Nothing to disclose
Tiago T. Fernandes: Nothing to disclose
Rita G. Nunes: Nothing to disclose
José Manuel Coelho: Nothing to disclose

### RPS 1910-5

**T2 mapping application for the diagnostics of chondromalacia after lateral patellar dislocation**

*E. Voronkova*, P. Menshchikov, I. Melnikov, A. Manzhurtsev, M. Ublinskiy, D. Vorobyev, D. Kupriyanov, O. Bozhko, T. Akhadov; Moscow/RU

**Purpose:** Chondromalacia is a pathology of the patella cartilage that often occurs after lateral patellar dislocation (LPD). The aim of the study is to examine short-term consequences of the first-time LPD on patellar cartilage condition in teenagers using T2 mapping.
**Methods or Backround:** The study includes 77 patients (15.1 ± 1.8 years) with different stages of chondromalacia caused by first time LPD and 48 healthy volunteers (14.7 ± 2.2 years). All research participants underwent MRI including T2 mapping (TSE, 6 TE from 13 to 78ms, voxel size 0.4×0.4×3 mm). T2 values were calculated in six regions: deep, intermediate, superficial layers and medial, lateral parts.
**Results or Findings:** In the lateral part of the cartilage, an increase in T2 values was found for both the mild and severe chondromalacia group in the deep and intermediate layers compared to the control group. In the medial part, an increase in T2 values is observed only for the severe group in the deep layer, while T2 in the mild chondromalacia group either doesn't change (deep and intermediate layers) or decreases (superficial layer).
**Conclusion:** The study showed a principal difference in T2 changes between medial and lateral cartilage facet. Although the medial part usually suffers first after LPD, the absence of T2 changes for the mild group may indicate completed reparative processes, while the decrease in T2 indicates that the reparation is still ongoing. Elevated T2 in the lateral part are a sign of metabolic problems and increased load due to medial patellofemoral ligament injury.
**Limitations:** The main limitation of our study is the lack of surgical confirmation of chondromalacia.
**Ethics committee approval:** The study was approved by CRIEPST ethic committee.
**Funding for this study:** This work is supported by a RSF 21-75-00068 grant.
**Author Disclosures:**
Petr Menshchikov: Nothing to disclose
Maxim Ublinskiy: Nothing to disclose
Olga Bozhko: Nothing to disclose
Andrei Manzhurtsev: Nothing to disclose
Denis Vorobyev: Nothing to disclose
Elena Voronkova: Nothing to disclose
Dmitry Kupriyanov: Nothing to disclose
Tolibjohn Akhadov: Nothing to disclose
Ilya Melnikov: Nothing to disclose

### RPS 1910-6

**New look for the old disease: a suggested new classification for tibiofemoral osteoarthritis**

*M. E. Abd El Bagi*, M. J. Alsalman, O. Abdelkareem, H. Al Jawad, H. Al Rehaily, N. Ozair, M. Aljarie, J. Altwalah, A. Mohamed; Riyadh/SA
*(drm_bagi@hotmail.com)*

**Purpose:** To suggest a new classification for tibiofemoral osteoarthritis.
**Methods or Backround:** Review of the previous radiographic classifications of knee osteoarthritis.
**Results or Findings:** Osteoarthritis affects 7% of the world's population. So far, there are 25 classifications. Kellgren and Lawrence's classification was suggested in 1957 is still the most commonly used radiographic classification. However, Kellgren and Lawrence questioned their own grading of grades 1 and 2. Other workers noted that, in grade 4, further deterioration cannot be classified and longitudinal studies cannot be assessed. The classification was also questioned by other authors because it relies mainly on the presence of osteophytes. Our community has a very high prevalence of tibiofemoral osteoarthritis. We noticed many cases of severe disease without osteophyte formation. The classification does not accommodate room for those who are described as grade 4 but who continue to show deterioration on radiographs. There is no suggested referral pathway to help reduce the long waiting time that these patients suffer. There is no score for loose bodies affecting the referral pathway.
**Conclusion:** We propose a simplified structured classification, whether osteophytes are present or absent. We add a stratified grading for osteoarthritis severity to follow up deterioration. Our classification indicates the possible clinical pathway that may help the first-line referral physician to avoid multiple referrals. Our classification caters for secondary osteoarthritis needing attention to the cause, particularly early referral of a rheumatology clinic. We included an item for the presence of loose bodies which necessitate a fast-track referral.
**Limitations:** Our classification does not include MRI or arthroscopic correlation.
**Ethics committee approval:** No information provided by the submitter.
**Funding for this study:** There was no funding for this study.
**Author Disclosures:**
Jumanah Altwalah: Nothing to disclose
Nabil Ozair: Nothing to disclose
Mohammed Jassim Alsalman: Nothing to disclose
Ahmed Mohamed: Nothing to disclose
Mohamed Elmutasim Abd El Bagi: Nothing to disclose
Mohammed Aljarie: Nothing to disclose
Omar Abdelkareem: Nothing to disclose
Hussain Al Jawad: Nothing to disclose
Haniyya Al Rehaily: Nothing to disclose

### RPS 1910-7

**The role of MR in the recognition and identification of the main periprosthetic complications of total hip arthroplasty (THA)**

*F. Molea*[1], A. Aprato[1], L. D'Ambrosio[2], F. Arabia[1], G. L. Desi[1], S. Artiaco[1], T. Robba[1]; [1]Turin/IT, [2]Orbassano/IT
*(francesca.molea@gmail.com)*

**Purpose:** To investigate the use of magnetic resonance (MR) for the detection and characterisation of peri-prosthetic complications of total hip arthroplasty (THA).
**Methods or Backround:** We evaluated 114 patients, median age 67 years, undergoing THA with different indications (coxarthrosis 76.3%, neoplasia 9.6%, trauma 7%, dysplasia 3.5%, other 3.5%) who underwent MRI during follow-up without any symptoms (65.8%); or for the presence of a suspected clinic for complications (metallosis 8.8%, pseudotumour 1.8%, aseptic loosening 5.3%, recurrent dislocations 13.3%, peri-prosthetic infection 2.7%, neoplastic recurrence 1.8%). All patients with symptoms suspected of complications had an imaging finding alteration of the periprosthetic tissues. We selected the following signs as complication imaging findings: prosthetic rupture, access route, simple or debris effusion, septate synovitis, pericapsular inflammation, bone oedema, osteolysis, fracture, periostitis, pericapsular oedema, muscle oedema, calcifications/heterotopic ossifications, trochanteric bursitis, sinus tract, air bubbles. We tried to derive from these data which were the most frequent and validated associations between imaging and clinic.
**Results or Findings:** From the analysis of these data emerged the finding that the most significant associations, between the most frequent complications and the imaging findings, are the following: for metallosis simple effusion (80%), debris effusion (80%), septate synovitis (30%), oedema bone (60%), osteolysis (60%), muscle atrophy (30%). For periprosthetic infections: access route (66.7%), simple effusion (66.7%), debris effusion (33.3%), septate synovitis (66.7%), pericapsular inflammation (66.7%), bone oedema (66.7%), osteolysis (66.7%), periostitis (33.3%), pericapsular oedema (66.7%), muscle oedema (66.7%), muscle atrophy (66.7%), sinus tract (33%), air bubbles (33%), heterotopic calcifications/ossifications (66.7%). For aseptic loosening: access route harvest (16.7%), simple effusion (66.7%), debris effusion (66.7%), bone oedema (83.3%), osteolysis (83.3%), edema pericaspular (33.3%), muscle oedema (33.3%), muscle atrophy (83.3%). 39 of these patients underwent surgical revision with histological confirmation of the suspected finding on imaging.
**Conclusion:** MR has been shown to be valid in recognising and characterising the main features related to the most frequent peri-prosthetic complications of THA.
**Limitations:** No limitations were identified.
**Ethics committee approval:** No information provided by the submitter.
**Funding for this study:** No funding was received for this study.
**Author Disclosures:**
Alessandro Aprato: Nothing to disclose
Stefano Artiaco: Nothing to disclose
Francesca Molea: Nothing to disclose
Federica Arabia: Nothing to disclose
Lorenzo D'Ambrosio Co-author: Nothing to disclose
Gian Luca Desi: Nothing to disclose
Tiziana Robba: Nothing to disclose

### RPS 1910-8

**Artifact reduction using virtual monoenergetic images and iterative metal artifact reduction for hip prosthesis associated artifacts in photon-counting CT**

*Y. C. Layer*, N. Mesropyan, A. Isaak, P. A. Kupczyk, T. Dell, J. A. Luetkens, U. I. Attenberger, D. Kütting; Bonn/DE
*(Yannik.Layer@ukbonn.de)*

**Purpose:** Aim of this study was to assess the impact of virtual monoenergetic images (VMI) in combination and comparison with iterative metal artifact reduction (IMAR) on hip prosthesis associated artifacts in photon-counting CT (PCCT).
**Methods or Backround:** For this retrospective study 33 scans with hip prosthesis associated artifacts acquired during clinical routine on a PCCT from 08/2022-09/2022 were analysed. VMI were reconstructed for 100-190 keV (10 keV steps) with and without IMAR and compared to polychromatic images. Artifact extent and assessment of adjacent soft tissue were rated by two

Saturday radiologists using a 5-point Likert grading scale for qualitative assessment. Quantitative assessment was performed using ROIs measuring attenuation and standard deviation in most pronounced hypodense and hyperdense artifacts, artifact-impaired bone, muscle, vessels, bladder and artifact-free corresponding tissue. An adjusted attenuation was calculated as difference between artifact-impaired tissue and corresponding tissue without artifacts for quantification of artifacts.
**Results or Findings:** Qualitative assessment for all investigated image reconstructions significantly improved compared to polychromatic images (PI). VMI of 100 keV with combination of IMAR achieved best results (e.g. diagnostic quality of the bladder: Median PI: 1 (Range: 1-3); VMI100kev+IMAR: 5 (3-5); $p < 0.05$). In quantitative assessment VMI of 100keV with IMAR provided best artifact reduction with an adjusted attenuation closest to 0 (e. g. bone: PI: 302.78; VMI100keV+IMAR: 51.18; $p < 0.05$).
**Conclusion:** The combination of VMI and IMAR significantly reduces hip prosthesis associated artifacts in PCCT and effectively improves the diagnostic quality of the surrounding tissue. VMI of 100 keV in combination with IMAR showed best results and are recommended to support scans of patients with hip replacement.
**Limitations:** The study design is retrospective and single-centred, comprising a small cohort.
**Ethics committee approval:** No information provided by the submitter.
**Funding for this study:** Y.C.Layer is supported by a research grant from Siemens Healthcare.
**Author Disclosures:**
Patrick Arthur Kupczyk: Nothing to disclose
Julian Alexander Luetkens: Nothing to disclose
Tatjana Dell: Nothing to disclose
Narine Mesropyan: Nothing to disclose
Alexander Isaak: Nothing to disclose
Yannik Christian Layer: Research/Grant Support: Yannik Christian Layer is supported by a research grant from Siemens Healthcare GmbH.
Ulrike I. Attenberger: Nothing to disclose
Daniel Kütting: Nothing to disclose

### RPS 1910-9

**Metal artifact reduction in photon-counting detector CT: quantitative evaluation of artifact reduction techniques**

S. Skornitzke[1], V. Mergen[2], T. D. Do[1], J. Biederer[1], H. Alkadhi[2], W. Stiller[1], T. Frauenfelder[2], H-U. Kauczor[1], *A. Euler*[2]; [1]Heidelberg/DE, [2]Zurich/CH

**Purpose:** To quantitatively compare different metal artifact reduction techniques for clinical photon-counting detector CT (PCD-CT).
**Methods or Backround:** A multi-energy phantom was scanned on a first-generation dual-source PCD-CT with four different central inserts: water-equivalent plastic, aluminum, steel, and titanium. Acquisitions were performed at 120kVp and 140kVp. Images were reconstructed with different spectral reconstruction settings (virtual monoenergetic images (VMI) from 110keV to 150keV, T3D, none); different quantum iterative reconstruction levels (QIR-3, QIR-off); two reconstruction kernels (Br36, Br56); and iterative metal artifact reduction (iMAR). A metal artifact quantification algorithm was used to calculate voxel-based percentages of metal artifacts and mean CT numbers of an adjacent water-equivalent insert. Repeated measures analysis of variances was performed for statistical analysis.
**Results or Findings:** Metal artifacts were strongest for steel (up to 67% of voxels), followed by titanium (up to 24%), and minor for aluminum (up to 3%). VMI, QIR, and iMAR had a significant impact on artifact reduction (all $p< 0.05$). Depending on the metal, average artifacts were reduced to 0.6-2.5% of voxels using iMAR, to 1.2-11.2% using VMI, and to 0.7-1.7% when combining iMAR and VMI. Average artifacts were 7.7% and 11% for QIR-off and QIR-3, respectively. Maximum reduction was found using VMI at 150keV with iMAR leading to a total artifact elimination for aluminum and titanium and residual artifacts of 0.1% for steel. Tube potential and reconstruction kernel did not show a significant impact on artifact reduction ($p>0.064$). CT numbers were significantly affected by iMAR, VMI, tube potential and reconstruction kernel (all $p<0.0001$), while QIR did not show a significant effect ($p=0.4715$).
**Conclusion:** Metal artifact reduction with PCD-CT was strongest by combining iMAR and VMI at 150 keV, while reconstruction kernel and tube potential had limited impact.
**Limitations:** Phantom study.
**Ethics committee approval:** No information provided by the submitter.
**Funding for this study:** There was no funding for this study.
**Author Disclosures:**
Thuy Duong Do: Nothing to disclose
Thomas Frauenfelder: Nothing to disclose
Victor Mergen: Nothing to disclose
Wolfram Stiller: Nothing to disclose
Jürgen Biederer: Nothing to disclose
Hans-Ulrich Kauczor: Nothing to disclose
Stephan Skornitzke: Nothing to disclose
Andre Euler: Nothing to disclose
Hatem Alkadhi: Nothing to disclose

**12:30-13:30 Research Stage 4**

### Research Presentation Session: Physics in Medical Imaging

### RPS 1913

### Image quality and patient dose in breast CT and other breast imaging techniques

**Moderator**
M. Sans Merce; Geneva/CH
*(marta.sansmerce@hcuge.ch)*

### RPS 1913-2

**Cascaded linear system-based model for simulation and optimisation of a dedicated breast CT system**

*J. J. Pautasso*, S. A. M. Tunissen, K. Michielsen, I. Sechopoulos; Nijmegen/NL

**Purpose:** Optimisation of the image acquisition parameters of new imaging devices is crucial for their optimal clinical introduction. In this work, a parallel-cascaded model (PCM) to predict the output image quality of a new dedicated breast CT (bCT) system was developed and validated.
**Methods or Backround:** The PCM predicts the pre-sampled modulation transfer function (MTF) and the normalised noise power spectrum (NNPS) by modelling each step in the image formation process. For validation, the characteristics of a bCT system were replicated, with the spectrum set to 49 kV+1.42 mm Al filtration with tube currents set to 20, 32, 40, 50, and 64 mA. The attenuation of an average breast (14 cm diameter) was represented using 2 mm Al + 0.257 mm Cu. The percentage difference between measurement and PCM prediction was quantified for the MTF and the mean percentage error (MPE) was calculated for the NNPS, both at 1 and 2 lp/mm. In addition, the PCM-predicted and bCT-acquired noise images were compared in terms of kurtosis and skewness.
**Results or Findings:** The difference in the MTF was -5.4% and 7.6% at 1 lp/mm and 2 lp/mm, respectively. The MPE (min, max) of the NNPS was -5.5% (-10.2%, 0.1%) and 15.7% (10.9%, 21.0%), at those same two frequencies. Kurtosis was -0.001 (-0.004, 0.003) for the PCM-predicted and 0.006 (-0.019, 0.017) for the bCT-acquired projection images. Moreover, skewness was 0.002 (-0.01, 0.01) and 0.04 (0.02, 0.06), respectively.
**Conclusion:** A parallel-cascaded model capable of predicting the image quality performance of a bCT system has been developed. Future steps involve parameter optimisation for the development of a new bCT image acquisition technique.
**Limitations:** The model inaccuracy is related to the veiling glare effect, which is not yet included.
**Ethics committee approval:** No information provided by the submitter.
**Funding for this study:** ERC Grant 864929
**Author Disclosures:**
Juan José Pautasso: Nothing to disclose
Sjoerd A. M. Tunissen: Nothing to disclose
Koen Michielsen: Nothing to disclose
Ioannis Sechopoulos: Speaker: Siemens Healthcare Research/Grant Support: Siemens Healthcare, Canon Medical, ScreenPoint Medical, Sectra Benelux, Volpara Healthcare, Lunit, iCAD

### RPS 1913-3

**How digital breast tomosynthesis could catch up with full-field digital mammography in microcalcification visibility**

*M. Radicke*, M. Beister, J. Freudenberger, M. Herbst, F. Lueck, R. Nanke, L. Ritschl, J. Wicklein, S. Kappler; Forchheim/DE
*(marcus.radicke@siemens-healthineers.com)*

**Purpose:** Digital breast tomosynthesis (DBT) enables significantly higher cancer detection rates compared to full-field digital mammography (FFDM) without compromising the recall rate. However, nowadays established tomosynthesis system concepts still tend to be inferior to FFDM regarding microcalcification reproduction.
**Methods or Backround:** The reduction of in-plane resolution which causes the lower detectability of microcalcifications is mostly due to tube-motion-induced focal spot blurring. To restrict this effect the velocity of the tube movement is kept low, resulting in longer acquisition times which, on contrary, may induce artifacts due to patient motion.
**Results or Findings:** We present a research prototype based on a novel x-ray tube that has an adaptive focal spot position. The focal spot position can be controlled via electromagnetic fields such that the external tube motion during each x-ray pulse is compensated via the internal focal spot deflection. By this, focus blur is basically removed, in-plane resolution is decoupled from acquisition speed and pulse length and becomes equivalent to FFDM, even in fast wide-angle tomosynthesis scans. In addition to the higher spatial resolution within the reconstructed images we also explored a new image reconstruction framework. Here, noise texture of DBT slices and of the synthetic mammogram were improved by a combination of deep-learning and dual domain filtering. The reconstruction framework additionally includes a fast-reader-mode using the concept of hybrid-depth-resolution which enhances calcification-visibility by adjusting their depth appearance.
**Conclusion:** The presented research prototype is an approach to unite high soft lesion detectability with an FFDM-like calcification visibility combined in a fast workflow.
**Limitations:** This prototype is part of a research project and not commercially available.
**Ethics committee approval:** No information provided by the submitter.
**Funding for this study:** Not applicable
**Author Disclosures:**
Julia Wicklein: Employee: Siemens Healthcare GmbH Shareholder: Siemens Healthcare GmbH
Magdalena Herbst: Shareholder: Siemens Healthcare GmbH Employee: Siemens Healthcare GmbH
Steffen Kappler: Shareholder: Siemens Healthcare GmbH Employee: Siemens Healthcare GmbH
Jörg Freudenberger: Shareholder: Siemens Healthcare GmbH Employee: Siemens Healthcare GmbH
Marcel Beister: Shareholder: Siemens Healthcare GmbH Employee: Siemens Healthcare GmbH
Ferdinand Lueck: Shareholder: Siemens Healthcare GmbH Shareholder: Siemens Healthcare GmbH Employee: Siemens Healthcare GmbH
Marcus Radicke: Shareholder: Siemens Healthcare GmbH Employee: Siemens Healthcare GmbH
Ludwig Ritschl: Employee: Siemens Healthcare GmbH Shareholder: Siemens Healthcare GmbH
Ralf Nanke: Employee: Siemens Healthcare GmbH Shareholder: Siemens Healthcare GmbH

### RPS 1913-4

**Breast glandular dose of a clinical photon-counting spiral breast CT system: Monte Carlo simulation study**

*O. V. V. Ivashchenko*[1], K. Michielsen[2], N. Voormolen[3], I. Hernandez-Giron[3], J. Heemskerk[3], I. Sechopoulos[2]; [1]Groningen/NL, [2]Nijmegen/NL, [3]Leiden/NL
*(o.v.ivashchenko@umcg.nl)*

**Purpose:** To investigate the glandular dose resulting from a clinical photon-counting spiral breast CT (SBCT) scanner using the manufacturer-recommended acquisition settings.
**Methods or Backround:** The x-ray spectrum and output of an AB-CT SBCT scanner were determined using empirical measurements, breast phantoms, and an established spectrum model. The manufacturer acquisition geometry was replicated in a Geant4-based breast dosimetry Monte Carlo (MC) simulator. The MC-model was validated by comparing simulated and measured air kerma in a standard CTDI-head phantom using various exposure levels. The mean (MGD) and normalised glandular dose (DgNct) were estimated using manufacturer-recommended settings (60 kV, 32 mA, no AEC, 0.97 pitch, 2 s rotation), 4.5 mm steps in the spiral trajectory, and 2*10^6 photons per projection. A range of phantom sizes (8-16 cm max diameter (Dmax), 0.5 and 0.75 diameter/length ratio, 1.45 mm skin layer, 0.273 mm isotropic voxels) and glandular densities (1/5/14.3/25 %) were used, representing a realistic patient population.
**Results or Findings:** The x-ray spectrum has a first half-value layer of 3.0 mm Al. The simulated air kerma of the CTDI-head phantom was within -1% and -4.2% of the measurements for the central and peripheral rods, respectively. The MGD for the lowest density (1%) breasts range from 7.2 mGy (Dmax=8 cm) to 4.6 mGy (Dmax=16 cm), while DgNct values varied from 0.32-0.26 mGy/mGy air kerma. Within the same breast size, DgNct and MGD decreases by 10% when glandularity increases from 1% to 25%.
**Conclusion:** SBCT acquisitions evaluated in this work result in MGDs that vary between 4.3-7.2 mGy, depending on the imaged breast characteristics. We observed an inverse dose-size dependence, likely related to the fixed tube current rate of the SBCT scanner.
**Limitations:** Phantom study
**Ethics committee approval:** No information provided by the submitter.
**Funding for this study:** Not applicable
**Author Disclosures:**
Oleksandra V. V Ivashchenko: Nothing to disclose
Koen Michielsen: Nothing to disclose
Irene Hernandez-Giron: Nothing to disclose
Ioannis Sechopoulos: Other: Research agreements with Siemens Healthcare, Canon Medical, Screenpoint Medical, Sectra Benelux, Volpara Solutions, Lunit Inc., and iCAD. Speaker bureau for Siemens Healthcare. Scientific advisory board of Koning Corp.
Jan Heemskerk: Nothing to disclose
Nora Voormolen: Nothing to disclose

### RPS 1913-5

**Refraction beats attenuation in breast CT**

*M. Rawlik*[1], A. Pereira[1], Z. Wang[2], L. Romano[1], R. Kubik-Huch[3], Z. Varga[1], A. Boss[1], M. Stampanoni[1]; [1]Zurich/CH, [2]Beijing/CN, [3]Baden-Dättwil/CH

**Purpose:** To assess the benefit of using refraction of x-rays in CT imaging of the breast.
**Methods or Backround:** Attenuation-based imaging is fundamentally limited by the dose: modern detectors are close to 100% efficient in counting single photons. Detecting refraction, the deflection of the x-rays by the tissue, in addition to attenuation, has long been speculated to cause a breakthrough for dose efficiency of CT. We have constructed a refraction-sensitive grating-interferometry CT system based on a conventional 70kVp x-ray source, commercially-available gratings and a photon-counting detector. We imaged a human breast specimen from an autopsy fixed in formaldehyde and quantitatively compared the quality of the volumes reconstructed from the simultaneously acquired attenuation and refraction signal, as well as a combination thereof. We considered the dose necessary to reach a contrast-to-noise-ratio of 5 bewteen adipose and glandular tissue.
**Results or Findings:** We found the benefit of refraction-based imaging to increase with the sharpness of the reconstruction kernel. The combination of both the attenuation and refraction signals from our system was more dose-efficient for kernels sharper than 263 um, which required a dose of 16 mGy, or higher, on our system. We estimated that the technology can ultimately reduce the dose by a factor 2-3, while maintaining the image quality.
**Conclusion:** Grating-interferometry CT can be more dose-efficient than conventional CT in a clinical setting. The technology is already ripe for clinical breast CT; as it develops, it will make other applications significantly more dose-efficient.
**Limitations:** To date, a single fixed specimen was imaged. The study will continue with imaging of fresh mastectomy specimens.
**Ethics committee approval:** Ethical agreement KEK-2012_554
**Funding for this study:** SNF R'Equip 206021_189662 (SiDRY), ETH-Research Commission Grant ETH-12 20-2, ETH Doc.Mobility Fellowship, Promedica Stiftung Chur, SNF Sinergia CRSII5 183568, PHRT Pioneer Project 2021-612 CLARINET, Swisslos Fund of Aargau.
**Author Disclosures:**
Zhentian Wang: Consultant: GratXray AG
Andreas Boss: Board Member: GratXray AG
Zsuzsanna Varga: Nothing to disclose
Alexandre Pereira: Nothing to disclose
Marco Stampanoni: Founder: GratXray AG Board Member: GratXray AG
Michał Rawlik: Other: GratXray AG
Rahel Kubik-Huch: Nothing to disclose
Lucia Romano: Other: GratXray AG

### RPS 1913-6

**Dose measurement and comparison between mammography and breast CT**

*D. Cester*, S. Shim, A. Boss; Zurich/CH

**Purpose:** Dedicated spiral breast-CT systems with photon counting detectors could be included in screening programs for breast cancer; however, concerns about dose exposure must first be addressed. The objective of this work was to investigate and compare the breast dose for a mammography device and a spiral breast-CT, depending on different breast size and density.
**Methods or Backround:** 3D-printed phantoms of three different sizes and filled with four mixtures were used to simulate different breast densities. The phantoms were scanned on a conventional mammography device and on a breast-CT system with standard clinical protocols; the surface dose was measured with MOSFET dosimeters. In addition, a Monte Carlo simulation was performed to validate the mammography measurements. The values for the surface dose and the calculated average absorbed dose were compared across the two modalities.
**Results or Findings:** The surface dose and the calculated average absorbed dose showed similar results in the comparison between the mammography and the breast-CT measurements, displaying a higher dependence of the dose values on the phantom size and density in the mammography case. In terms of absolute dose values, the breast-CT scan resulted in similar or lower dose particularly in case of small phantoms or high glandularity.
**Conclusion:** Depending on breast size and glandularity, the dose values for a breast-CT scan can be similar or even lower than those of a conventional mammography scan.
**Limitations:** Technical constraints limited this study to only three phantom sizes. Only a reduced number of points could be directly measure, and only on the surface of the phantoms. In addition, the agreement between the simulations and the measurements was not optimal for conventional mammography with small phantom size.
**Ethics committee approval:** No information provided by the submitter.
**Funding for this study:** Resources for this study were provided by the host institute.

**Author Disclosures:**
Andreas Boss: Nothing to disclose
Davide Cester: Nothing to disclose
Sojin Shim: Nothing to disclose

### RPS 1913-7

**Low contrast detectability assessment in digital breast tomosynthesis: an innovative statistical approach for an absolute quantitative evaluation**

*R. Villa*[1], N. Paruccini[1], C. Ingraito[2], N. Oberhofer[3], C. Pasquali[1], E. De Ponti[1]; [1]Monza/IT, [2]Milan/IT, [3]Bolzano/IT
*(raffaele.vill@gmail.com)*

**Purpose:** Low contrast detectability (LCD) is an important aspect of image quality; unfortunately, its assessment is not trivial in digital breast tomosynthesis, where there is not a widely accepted commercial phantom as the Artinis CDMAM phantom in digital mammography. Therefore, the aim of this work is to propose an innovative and simplified method for threshold contrast evaluation in digital breast tomosynthesis (DBT).
**Methods or Backround:** Based on a statistical approach and implemented in MATLAB, the method determines contrast detail threshold curves from a homogenous region of a tomographic acquisition. Absolute values for low contrast thresholds (LCth) have been derived using a glandular step wedge (from 0% to 100% glandularity), included in the Tissue-Equivalent-Phantom for Mammography (CIRS,USA), making them ideally independent from specific acquisition parameters and employed equipment. The proposed method was applied to mammographic DBT acquisitions from 5 equipment of 3 manufacturers, characterised by different detection systems and acquisition modalities. Besides evaluating LCth dependencies on acquisition parameters, results were compared with the human observer detection of semi-spherical masses of different diameters (from 4.75 to 0.9mm) of 75% Glandularity.
**Results or Findings:** LCD curves show the expected dependencies from acquisition parameters, including Average Glandular Dose. Furthermore, while LCth expressed in PV cannot be compared between different x-ray spectrum and equipment, the conversion in terms of Glandularity allows an absolute quantification. Comparison with observer detection of semi-spherical masses gives a good agreement with the methods in the various considered conditions.
**Conclusion:** The statistical method gives interesting results in terms of absolute quantification of LCth. Thus, the current method could be applied in quality assurance programme and acceptance tests.
**Limitations:** A deeper investigation should be performed to include a wider range of equipment, in order to define typical or expected values.
**Ethics committee approval:** Not required.
**Funding for this study:** No funding was received for this study.
**Author Disclosures:**
Nadia Oberhofer: Nothing to disclose
Elena De Ponti: Nothing to disclose
Raffaele Villa: Nothing to disclose
Nicoletta Paruccini: Nothing to disclose
Claudia Pasquali: Nothing to disclose
Chiara Ingraito: Nothing to disclose

### RPS 1913-8

**On the limitations of using PMMA phantoms to assess image quality in dual-energy contrast-enhanced mammography**

*K. Houbrechts*, R. Massera, N. Marshall, H. Bosmans; Leuven/BE
*(katrien.houbrechts@uzleuven.be)*

**Purpose:** To investigate the possibility of using polymethyl methacrylate (PMMA) as an alternative for breast tissue simulating material, when assessing technical image quality in dual-energy contrast-enhanced mammography (CEM).
**Methods or Backround:** Monte Carlo code (MC-GPU v1.5b) was used to simulate CEM images, for the low energy (LE) and high energy (HE) spectra used by the Siemens Revelation system. Breast simulating phantoms with a range of thicknesses and glandularities were simulated and the LE attenuation equivalence of PMMA was estimated. A test object containing a 100% glandular insert and four inserts with different iodine concentrations was simulated, first with the breast simulating material and then with the equivalent PMMA thickness. The weighting factor required to minimise the glandular insert signal in the subtracted CEM image was established along with signal difference to noise ratio (SDNR) for the iodine inserts.
**Results or Findings:** Large differences were seen between the required weighting factors for PMMA and breast simulating material; e.g. cancelling the glandular insert in a 50 mm thick 30% glandular breast required a weighting factor of 0.26, versus a factor of -0.05 for the PMMA equivalent phantom (43.5 mm thickness). SDNR measured in PMMA compared to breast simulating backgrounds varied as a function of iodine concentration; e.g. SDNR differences in 50 mm thick 30% glandular background versus 43.5 mm PMMA background were -8±2% for 0.5 mg/cm² iodine and -2±1% for pure iodine (0.26 as weighting factor).
**Conclusion:** Background cancellation efficiency, quantified as residual signal of a glandular insert, cannot be investigated in a PMMA background. SDNR measured in PMMA phantoms may not predict the SDNR in patient CEM images and must be interpreted with caution.
**Limitations:** A simple weighted subtraction is applied. Simulations should be supported with measurements.
**Ethics committee approval:** No information provided by the submitter.
**Funding for this study:** KULeuven PhD grant.
**Author Disclosures:**
Nicholas Marshall: Nothing to disclose
Rodrigo Massera: Nothing to disclose
Hilde Bosmans: Nothing to disclose
Katrien Houbrechts: Nothing to disclose

### RPS 1913-9

**Study of innovative approaches for 3D printed anthropomorphic phantoms for 2D and 3D x-ray breast imaging**

K. Bliznakova[1], *A. Sarno*[2], G. Mettivier[2], T. Georgiev[1], N. Dukov[1], N. Okkalidis[3], Z. Bliznakov[1], P. Russo[2]; [1]Varna/BG, [2]Naples/IT, [3]Thessaloniki/GR
*(sarno@na.infn.it)*

**Purpose:** To investigate new materials and 3D printing methodologies for manufacturing breast phantoms for investigations in x-ray breast imaging.
**Methods or Backround:** We investigated, in terms of Hounsfield Unit (HU) and reproduced complexity of anatomical background in acquired CT images, breast phantoms manufactured via Fused Deposition Modelling (FDM) 3D printing technology with variable extrusion rates, this meant to tune density of the included tissues and breast phantoms manufactured via inkjet printers. They were produced from breast models derived from both breast images acquired via MRI scanners and a dedicated breast CT scanner. Images of the produced phantoms were acquired via a whole body CT at 120 kV and at 70 kV and reconstructed voxel size was 0.9 mm × 0.9 mm with a slice thickness 0.5 mm.
**Results or Findings:** The HU of all manufactured phantoms (both FDM and inkjet produced) resulted close to the expected ones, for both the simulated adipose and glandular breast tissues. The reproduced parenchymal anatomical noise reflected that in the clinical images; however, in the case of MRI derived breast models, it resulted slightly lower than in images for breast CT dedicated derived phantoms. This may be due by the lower image definition in the first technology.
**Conclusion:** We demonstrated that low budget phantoms produced via variable extrusion rate FDM and via conventional inkjet printers permit a fine breast reproduction of both breast parenchymal complexity and HU in acquired CT images.
**Limitations:** Phantoms were produced for few breast models and evaluations made exclusively at 70 kV and 120 kV
**Ethics committee approval:** No information provided by the submitter.
**Funding for this study:** This project has received funding from the European Union’s H2020 research and innovation programme under the Marie Skłodowska-Curie GA No 101008020
**Author Disclosures:**
Kristina Bliznakova: Nothing to disclose
Zhivko Bliznakov: Nothing to disclose
Tihomir Georgiev: Nothing to disclose
Nikolay Dukov: Nothing to disclose
Giovanni Mettivier: Nothing to disclose
Paolo Russo: Nothing to disclose
Antonio Sarno: Nothing to disclose
Nikoforos Okkalidis: Nothing to disclose

**14:00-15:30** **Research Stage 1**

### Research Presentation Session: Abdominal Viscera & GI Tract

### RPS 2001

### GI tract imaging: biomarker, technique and performance developments

**Moderator**
D. J. M. Tolan; Leeds/UK
*(damian.tolan@nhs.net)*
**Author Disclosures:**
Damian John Michael Tolan: Speaker: Guerbet

### RPS 2001-2

**Abdominal computed tomography findings in patients with COVID-19**
*E. Kaba*, G. Burakgazi, F. Beyazal Çeliker, S. Mahmutoğlu, S. Sekmen, L. Sağır, H. Er; Rize/TR
*(esatkaba04@gmail.com)*

**Purpose:** In this study, we aimed to compare the intra-abdominal solid organs and intra-abdominal major vascular structures in the abdominal computed tomography (CT) obtained from the COVID-19 patients and the healthy group.
**Methods or Backround:** This study was conducted retrospectively between the patients who applied to our hospital with abdominal symptoms within three months after COVID-19 infection between March 2020 and March 2021 and the control group. 100 COVID-19 patients and 80 control healthy groups were included. Abdominal CT findings, laboratory values and demographic data of COVID-19 patients were compared with the control group.
**Results or Findings:** In abdominal CT, dilated bowel loop was observed in four patients, and perforation was observed in two patients. Cholelithiasis was found in eight patients, intrahepatic bile duct dilatation in two patients, hepatomegaly in 36 patients, and splenomegaly in 12 patients. Abdominal pain was the most common of the abdominal symptoms. Portal vein diameter and left renal artery diameter were found to be significantly lower in the patient group (p=0.018, p=0.015). Patients with and without hepatomegaly and patients with and without splenomegaly were compared in the patient group. Portal vein diameters in splenomegaly patients and ALT values in hepatomegaly patients were found to be statistically significantly higher (p=0.00, p=0.047). The time elapsed since Covid-19 PCR positivity was compared with the vascular diameters detected by abdominal CT, and no statistically significant finding was found.
**Conclusion:** Evaluation of intra-abdominal solid organs in abdominal CT and measuring the diameters of abdominal main vascular structures with COVID-19 patients may be useful for diagnosis and treatment.
**Limitations:** The study is limited by its retrospective design and by being a single-centre study.
**Ethics committee approval:** The ethics approval was obtained by Recep Tayyip Erdoğan University (approval number: 11/11/2021, 2021-194).
**Funding for this study:** The authors have no relevant conflicts of interest or financial support to declare.
**Author Disclosures:**
Sudem Mahmutoğlu Colak: Nothing to disclose
Hüseyin Er: Nothing to disclose
Lütfullah Sağır: Nothing to disclose
Fatma Beyazal Çeliker: Nothing to disclose
Gülen Burakgazi: Nothing to disclose
Sümeyye Sekmen: Nothing to disclose
Esat Kaba: Nothing to disclose

### RPS 2001-3

**Diagnostic performance of contrast-enhanced CT in loco-regional staging and evaluation of retroperitoneal surgical margins of colon cancer patients**
*I. Bianco*, C. Maino, T. P. Giandola, C. R. G. L. O. M. Talei Franzesi, P. Marra, D. Ippolito, S. Sironi; Milan/IT

**Purpose:** To evaluate diagnostic accuracy of preoperative contrast-enhanced CT (CECT) in local staging of colon cancer compared with histopathological data as standard of reference.
**Methods or Backround:** We retrospectively enrolled 75 patients who underwent CECT before surgical resection. Images were reviewed randomly and blindly by a radiologist with 15 years' experience in abdominal imaging to localise and stage the tumour. Each lesion was classified into T2, T3, and T4 according to WHO classification system. The following parameters were collected: tumour bulging, fat stranding, lateroconal fascia invasion, enlarged vessels, the axial short diameter of the biggest node, shape, enhancement pattern, intranodal necrosis and nodes' cluster. Sensitivity, specificity, positive predictive value (PPV), negative predictive value (NPV) and diagnostic accuracy were computed by using contingency tables and ROC curves.
**Results or Findings:** The most frequent tumour location was the right colon (55%), followed by sigmoid (26%), left colon (14%) and transverse (5%). Sensitivity was highest for T3 lesions, reaching a value of 69%, whereas specificity was highest for T2 (90%) and T4 (82.6%) lesions. The PPV was intermediate for all lesion categories, reaching a maximum value of 50%, while the NPV was acceptable for all lesion categories, with a maximum of 84.4% for T2 lesions. Overall, diagnostic accuracy reached optimal values for T2 [92% (85.2-99.1)] and T4 [76% (59.5-93.4)] lesions, while modest for T3 lesions [69% (55.3-82.9)]. The lateroconal fascia invasion and enlarged vessels resulted as independent predictor factors (OR=3.292 and OR=2.651) for T staging, while nodes' cluster and dimension as independent predictors factors of N staging (OR=3.798 and OR=1.083).
**Conclusion:** CT has proven to be a useful tool in the evaluation of T staging, offering good diagnostic performance in particular for T2 and T4 lesions.
**Limitations:** Not applicable.
**Ethics committee approval:** No information provided by the submitter.
**Funding for this study:** Not applicable.
**Author Disclosures:**
Sandro Sironi: Nothing to disclose
Cesare Maino: Nothing to disclose
Paolo Marra: Nothing to disclose
Cammillo Roberto Giovanni Leopoldo Oreste Massimiliano Talei Franzesi: Nothing to disclose
Ilaria Bianco: Nothing to disclose
Teresa Paola Giandola: Nothing to disclose
Davide Ippolito: Nothing to disclose

### RPS 2001-4

**Diagnostic accuracy of computed tomography in preoperative staging of colon cancer patients: the impact of the readers' experience**
*I. Bianco*[1], T. P. Giandola[1], M. Ragusi[1], C. Maino[1], P. A. Bonaffini[1], D. Ippolito[2], S. Sironi[3]; [1]Milan/IT, [2]Monza/IT, [3]Bergamo/IT

**Purpose:** To assess agreement and diagnostic accuracy between different radiologists in the detection of specific features related to the T and N stage in colon cancer patients.
**Methods or Backround:** We enrolled 75 patients who underwent preoperative contrast-enhanced computed tomography (CECT) and surgical resection for colon cancer. Pathological data were used as standard of reference. Images were reviewed blindly by 3 radiologists with different experience in abdominal imaging: the most experienced one (R1), an abdominal radiologist (R2) and a general radiologist (R3). Radiologists were asked to localise the tumour and evaluate following parameters: location, number of lesions, parietal thickening, tumour bulging, perivisceral fat stranding, lateroconal fascia invasion, vessel enlargement (> 2 mm), nodes appearance (shape, enhancement, presence of clusters). Reliability analysis was assessed with κ statistic or interclass correlation coefficient (ICC)
**Results or Findings:** Tumour location, number of lesions and circumferential thickening showed an almost perfect concordance (k=0.853, k=0.800 and k=0.671, respectively). T staging demonstrated an overall moderate agreement (k=0.531), which was only considered fair for the T3 group (k=0.402). Overall agreement was moderate in the evaluation of bulging (k=0.478), perivisceral inhomogeneity (k=0.490), and lateroconal fascia invasion (k=0.436). Moderate to substantial agreement existed for nodal enhancement (k=0.431), shape (k=0.484), short axis (ICC=0.732) and intranodal necrosis (k=0.606), while the evaluation of nodes' cluster was fair (k=0.358). When comparing CT findings between R1 and R3 and between R2 and R3 all parameters showed reduced reliability for nodes (k=0.287-0.477) and lesion characteristics (k=0.288-0.545), except for tumour location and tumour focality (k=0.710-0.825)
**Conclusion:** Despite CT represents a reliable diagnostic tool for assessment of colon cancer patients, reader's experience remains one of the most important factors associated with correct evaluation of specific features in staging T and N parameters.
**Limitations:** Not applicable.
**Ethics committee approval:** No information provided by the submitter.
**Funding for this study:** Not applicable.
**Author Disclosures:**
Sandro Sironi: Nothing to disclose
Cesare Maino: Nothing to disclose
Pietro Andrea Bonaffini: Nothing to disclose
Maria Ragusi: Nothing to disclose
Ilaria Bianco: Nothing to disclose
Teresa Paola Giandola: Nothing to disclose
Davide Ippolito: Nothing to disclose

### RPS 2001-5

**Using structured templates or free text style in reporting CT staging on colon cancer: a national survey**

R. Dam Andersen, M. R. V. Pedersen, L. Hesseldal, *S. R. Rafaelsen*; Vejle/DK
*(soeren.rafael.rafaelsen@rsyd.dk)*

**Purpose:** The survey aimed to evaluate the use of CT free text reports and structured report templates when it comes to staging CT imaging of colon cancer.
**Methods or Backround:** A multiple-choice questionnaire survey was conducted over four weeks. Online questionnaire software was used, and a link was shared with medical doctors with help from the Danish Colorectal Cancer Group (DCCG) and a social media radiologist group.
**Results or Findings:** Clinicians preferred the template style (95%), whereas the support for template reports was less among the radiologist (76%). All female responders preferred the template style, and this was only true for 84% of the male responders. Furthermore, the survey showed a slightly deficient level of national CT reporting quality. Only seven out of thirteen questions and sub-questions concerning CT report quality achieved an approval rate of more than 85%. The colorectal cancer multidisciplinary team consultants who always or usually work with template-style reporting of CT scans of colon cancer tend to be more satisfied with the quality and content of the reports compared to those who rarely use or read template reports.
**Conclusion:** The following indicators were insufficiently reported: tumour invasion growth, number of hepatic metastasis, segment location of hepatic metastasis and retroperitoneal lymph node involvement. Nearly all participants found relevant information easily accessible in the template reports group.
**Limitations:** Missing response rate.
**Ethics committee approval:** No information provided by the submitter.
**Funding for this study:** No funding was provided for this study.
**Author Disclosures:**
Laura Hesseldal: Nothing to disclose
Malene Roland Vils Pedersen: Nothing to disclose
Rasmus Dam Andersen: Nothing to disclose
Sören R. Rafaelsen: Nothing to disclose

### RPS 2001-6

**CT radiomic features of colorectal cancer liver metastasis for predicting upcoming disease progression after initial systemic chemotherapy**

*S. Song*, H. Kim, B. Kim, J-I. Choi; Seoul/KR
*(leontiusk@gmail.com)*

**Purpose:** This study aims to investigate whether CT radiomic analysis of CRLM can predict early disease progression during the course of initial systemic chemotherapy. Early prediction of poor response to an initial chemotherapeutic agent can be helpful for appropriate and quick replacement of the chemotherapeutic regimen.
**Methods or Backround:** We enrolled 134 patients who underwent systemic chemotherapy with FORFIRI or FOLFOX regimen for CRLM from January 2017 to December 2020. We divided patients into two groups; the training set and validate set. We reviewed CT images of each patient and selected CT scans which were taken just prior to disease progression (early progression; EP). We also selected baseline CT scans for chemotherapy, after which the patient showed partial/complete response or stable disease according to RECIST 1.1 (no progression; NP). The largest CRLMs of each patient were selected and manually segmented by volume of interest on the portal venous phase. The training was performed using the supporting vector machine, and radiomic features were extracted. The best subset of significant features was selected by L1-based feature selection, and a radiomic classification model was built. Performance of training and validation sets were yielded using the AUC curve.
**Results or Findings:** 107 radiomic features were extracted from the training set, and 14 were selected for the prediction model. The prediction model yielded an AUC of 0.76 and an accuracy of 74.1% for the training set and an AUC of 0.71 and an accuracy of 64.6% for the validation set.
**Conclusion:** Radiomic analysis of CT scans for CRLM is a potential predictive tool for early prediction of disease progression. Radiomic analysis of CRLM can warn of early progression within two or three months and provide beneficial information to prepare alternative chemotherapeutic regimens.
**Limitations:** This is a single-centre study.
**Ethics committee approval:** Not applicable.
**Funding for this study:** This research received no specific grant.
**Author Disclosures:**
Sangkeun Song: Nothing to disclose
Hokun Kim: Nothing to disclose
Bohyun Kim: Nothing to disclose
Joon-Il Choi: Nothing to disclose

### RPS 2001-7

**Computed tomography measured sarcopenia as a risk factor for complications after abdominal wall hernia repair**

*A. N. Vázquez Tobías*; Silao/MX
*(dr.natanael.vazquez@gmail.com)*

**Purpose:** Abdominal wall hernias are highly prevalent in the general population. The incidence of complications after surgical repair is reported to be as high as 20%. Sarcopenia is a recently identified risk factor for the development of postsurgical complications. Computed Tomography (CT) is an accessible and reproducible method to identify sarcopenia.
**Methods or Backround:** We studied a sample of patients who underwent surgical repair of abdominal wall hernia. The presurgical CT scan was analysed using the software “CoreSlicer” to obtain measurements of area (PMA) and density of the psoas muscles and the Psoas Muscle Index (PMI). The statistical analysis was performed using “Epi Info” to determine the risk of postsurgical complications in patients with sarcopenia.
**Results or Findings:** 96 patients were included in the study, in which we found a statistically significative increase in the risk of developing complications when sarcopenia was determined by PMA and PMI ($p<0.05$), specifically for surgical wound infection, and recurrence.
**Conclusion:** This study showed that CT measured sarcopenia is a risk factor for the development of complications in patients who underwent surgical repair for abdominal wall hernia. We believe that every patient undergoing this procedure should be screened using this method in the initial approach. New studies are needed to determine if nutritional and physiotherapeutic interventions to improve this condition have an impact on the prognosis.
**Limitations:** The retrospective nature of this study calls for newer prospective studies to confirm the findings.
**Ethics committee approval:** No information provided by the submitter.
**Funding for this study:** No funding was received for this study.
**Author Disclosures:**
Adolfo Natanael Vázquez Tobías: Nothing to disclose

### RPS 2001-8

**Dual-energy CT muscle fat quantification as a new follow-up parameter and predictor of survival in intensive-care patients**

*J. Erley*, K. Roedl, N. F. Schubert, G. de Heer, C. Burdelski, E. Tahir, J. Yamamura, G. Adam, I. Molwitz; Hamburg/DE
*(j.erley@uke.de)*

**Purpose:** The study aimed to evaluate changes in body composition and their impact on survival using a new dual-energy computed tomography (DECT) material decomposition parameter in immobilised intensive care unit (ICU) patients.
**Methods or Backround:** All ICU patients receiving two DECT scans (SOMATOM Force, Siemens, Germany) within a minimum interval of 10 days were retrospectively included from November 2019 to September 2022. DECT fat fraction (DECT FF) was determined by material decomposition for iodine, soft tissue, and fat in the posterior paraspinal muscle at the height of the third lumbar vertebra (L3). Waist circumference (WC), skeletal muscle index (SMI), muscle radiodensity attenuation (MRA) and subcutaneous/visceral adipose tissue area (SAT, VAT) were measured. Days until hospital dismissal or death after ICU admission were noted. Wilcoxon signed-rank test, intraclass correlation coefficients (ICC) and multiparametric Cox-regression survival analysis (including body composition parameters, gender, age, body mass index, sequential organ failure assessment (SOFA) score at ICU admission) were employed.
**Results or Findings:** 82 patients were included (43% female; age 60±13 years). 48 patients (59%) died 76±89 days after ICU admission. SMI decreased ($-4.3\pm6.5 cm^2/m^2$, $p<0.001$) from CT1 to CT2 (mean 31±27 days), as did MRA (-3.1±6.3HU, $p<0.001$) and VAT ($-8.4\pm33.7 cm^2$, $p=0.033$). DECT FF increased (+6.0±6.2 %, $p<0.001$). WC and SAT did not significantly change. DECT-FF (hazard ratio: 3.4 [95% confidence interval 1.3-9.2], $p=0.016$) was the only parameter next to age and SOFA-score able to predict in-hospital mortality. Interobserver variability of DECT FF measurements was excellent (ICC 0.98-0.99).
**Conclusion:** DECT FF increased in immobilised ICU patients while SMI, MRA and VAT decreased. DECT FF was the only body composition parameter able to predict in-hospital mortality.
**Limitations:** This is a single-centre, retrospective study with a small patient cohort and variable time between CT exams.
**Ethics committee approval:** The study has been approved with a waiver of consent by the local ethics committee of the medical association in Hamburg.
**Funding for this study:** Not applicable.
**Author Disclosures:**
Christoph Burdelski: Nothing to disclose
Isabel Molwitz: Nothing to disclose
Geraldine de Heer: Nothing to disclose
Kevin Roedl: Nothing to disclose
Jin Yamamura: Nothing to disclose
Jennifer Erley: Nothing to disclose

Niklas Ferdinand Schubert: Nothing to disclose
Gerhard Adam: Nothing to disclose
Enver Tahir: Nothing to disclose

### RPS 2001-9

**Bolus tracking and lean body weight-based contrast material dose for acquiring portal venous phase CT studies of the abdomen: preliminary results**

*R. Valletta*[1], M. Bonatti[1], V. Vingiani[1], F. Lombardo[2], T. Gorgatti[1], F. Ferro[1]; [1]Bolzano/IT, [2]Negrar/IT
*(riccardo.valletta@gmail.com)*

**Purpose:** To evaluate the potential advantages and limits of using a bolus tracking technique and a lean body weight-based contrast material dose for acquiring portal venous phase CT scans of the abdomen.
**Methods or Backround:** IRB-approved prospective study, patients gave their informed consent. In the period August 2022-September 2022 we randomly selected 38 oncologic patients (20M/18F; mean age 63y) scheduled for a portal venous phase abdominal CT on our third generation Dual Source scanner who performed the same examination using standard protocol within the previous 12 months. Standard protocol: 0.6gI/Kg body weight contrast material and 70" fixed delay acquisition. Experimental protocol: 0.7gI/Kg lean body weight (measured with commercially available impedance balance) contrast material, and bolus tracking ROI on the VII segment (trigger: 50 HU). Liver, portal vein, kidneys' cortex, and spleen attenuation were measured using round ROIs and normalized to paraspinal muscles, both on current and previous CT.
**Results or Findings:** Median contrast dose (370mgI/Kg contrast material) was 97ml using the experimental protocol and 110ml using the standard one (p=0.0001, Wilcoxon test). Median acquisition delay using the experimental protocol was 69.5” (range 56-90”). Median normalised portal vein attenuation was 3.29 using the experimental protocol and 2.90 using the standard one (p=0.0103, Wilcoxon test). No statistically significant differences between experimental and standard protocols were found in liver, kidneys' cortex, and spleen normalised attenuations (p>0.05).
**Conclusion:** Using a lean body weight-based contrast material dose and a bolus tracking technique enables to significantly reduce the administered contrast dose with no significant changes in parenchymal organs enhancement.
**Limitations:** Preliminary results; limited sample.
**Ethics committee approval:** No information provided by the submitter.
**Funding for this study:** No funding was received for this study.
**Author Disclosures:**
Matteo Bonatti: Nothing to disclose
Fabio Lombardo: Nothing to disclose
Tommaso Gorgatti: Nothing to disclose
Riccardo Valletta: Nothing to disclose
Federica Ferro: Nothing to disclose
Vincenzo Vingiani: Nothing to disclose

### RPS 2001-10

**Tailored scan delay in single-pass contrast-enhanced abdominal multi-detector CT: effects of either a fixed rate or a fixed injection duration on vascular enhancement**

*L. Camera*, V. D'Ambrosio, L. Pinto, A. Paludi, R. Liuzzi, R. De Cicco, A. Ponsiglione, S. Maurea, A. Brunetti; Naples/IT
*(camera@unina.it)*

**Purpose:** To evaluate the effects on vascular enhancement of either a fixed rate (FR) or a fixed injection duration (FID) in single-pass (SP) contrast-enhanced abdominal multi-detector CT (CE-MDCT).
**Methods or Backround:** 100 patients (55M; 45F; aged 20-83 years) with non-traumatic acute abdominal pain underwent a SP CE-MDCT performed after i.v. injection of 1.7cc/Kg of a non ionic iodinated contrast-media (370 mgI/ml). In all patients CE-MDCT was acquired by a SP protocol characterised by a scan delay tailored around a monophasic contrast injection performed with either a FID of 55 sec (group A; n=50; 51 ± 17 yrs; BMI = 22.7 ± 4.2) or a FR of 2cc/sec (group B; n= 50; 53 ± 19 yrs; BMI 23.4 ± 4.4). In both groups patients were further stratified according to total body weight (Kg) as follows: 40-60 (L); 61-80 (M); 81-100 (H). Signal- (SNR) and contrast-to-noise ratios (CNR) were calculated for the liver and for both abdominal aorta (AA) and main portal vein (MPV) using the psoas muscles as reference tissues. Statistical analysis was performed by Mann-Whitney test (p<0.05).
**Results or Findings:** Whereas no significant differences in vascular enhancement were observed in the L (n=40) as well as the M (n=48) sub-groups, both SNR as well as CNR were significantly higher in Group A for AA (26.0 ± 3.4 vs 19.0 ± 3.8 and 24.5 ± 4.3 vs 16.4 ± 4.8) and MPV (20.3 ± 2.8 vs 16.3 ± 2.8 and 16..9 ± 2.3.0 vs 13.5 ± 1.8) in the H sub-groups (n= 12), respectively.
**Conclusion:** As far as vascular enhancement is concerned a FID results in an overall better image quality than a FR in SP CE-MDCT.
**Limitations:** Non consecutive patients. Small series except for IBD.
**Ethics committee approval:** Approval obtained by ethical committee "Carlo Romano" of University "Federico II" of Naples in 2021.
**Funding for this study:** No funding was received for this study.
**Author Disclosures:**
Rossella De Cicco: Nothing to disclose
Luigi Camera: Nothing to disclose
Raffaele Liuzzi: Nothing to disclose
Lorenzo Pinto: Nothing to disclose
Adriana Paludi: Nothing to disclose
Andrea Ponsiglione: Nothing to disclose
Vincenzo D'Ambrosio: Nothing to disclose
Arturo Brunetti: Nothing to disclose
Simone Maurea: Nothing to disclose

### RPS 2001-11

**Low-volume bowel preparation in CT colonography does not affect the quality of colon cleansing: a randomised non-inferiority trial**

*A. Onori*, S. Vicini, D. M. Bellini, M. Rengo, I. Carbone; Rome/IT
*(alexonori@yahoo.com)*

**Purpose:** The study aimed to determine whether the quality of a low-volume reduced bowel preparation (LV-RBP) for CT colonography (CTC) is non-inferior to a standard full-volume reduced bowel preparation (FV-RBP) regimen.
**Methods or Backround:** In this randomised controlled trial, consecutive participants referred for CTC were randomly assigned to receive LV-RBP (52.5g of PMF104 in 500mL of water) or FV-RBP (105g of PMF104 in 1000mL of water). Images were independently reviewed by five readers, who rated bowel preparation’s quality using a four-point scale (0-3; best-worst) on a per-colonic segment basis. The primary outcome was the non-inferiority of LV-RBP to FV-RBP in the proportion of segments that scored 0 (non-inferiority margin of 10%). Non-inferiority in residual fluids and colonic distension, superiority in lesions detection rates and patient acceptability were secondary outcomes.
**Results or Findings:** 110 participants (mean age 65±14 years; 74 women) were allocated to LV-RBP (n=55) or FV-RBP (n=55) arms. There were 92% of segments that scored 0 in colon cleansing quality in LV-RBP and 94% in FV-RBP for prone scans and 94% vs 92% as for supine scans. The risk difference was -2.1 (95%CI: -5.9 to 1.7) and 1.5 (95%CI: -2.4 to 5.4) for prone and supine positions, respectively. Residual fluids and colonic distension were also non-inferior in LV-RBP. LV-RBP was associated with fewer evacuations during preparation (7±5 vs 10±6; p=0.002). There was almost perfect agreement for the overall quality of colon cleansing and volume of residual fluids scoring among all readers (ICC=0.90 [95% CI 0.88-0.91] and ICC=0.88 [95% CI 0.87-0.90] in LV-RBP and in FV-RBP arm, respectively). Patient’s discomfort related to preparation was mild by most participants for both regimens (median VAS score (0-10): 2 vs 2; p=0.64).
**Conclusion:** LV-RBP for CTC demonstrated a non-inferior quality of colon cleansing, with improved gastrointestinal tolerability compared to FV-RVP.
**Limitations:** Not applicable.
**Ethics committee approval:** Not applicable.
**Funding for this study:** Not applicable.
**Author Disclosures:**
Alessandro Onori: Nothing to disclose
Iacopo Carbone: Nothing to disclose
Simone Vicini: Nothing to disclose
Marco Rengo: Nothing to disclose
Davide Maria Bellini: Nothing to disclose

### RPS 2001-12

**Reliability and diagnostic accuracy of a fast MR-enterography protocol for the assessment of Crohn's Disease activity**

*V. Caliendo*, V. Patanè, G. Vacca, M. Del Canto, V. Nardone, S. Cappabianca, R. Grassi, A. Reginelli; Naples/IT
*(vale.caliendo@gmail.com)*

**Purpose:** We aim to investigate the reliability and the diagnostic accuracy of a fast MRE protocol (T2w HASTE, T2w b-SSFP, DWI) versus the standard one (T2w HASTE, T2w b-SSFP, DWI, contrast-enhanced T1w GRE fs) for the assessment of Crohn's Disease (CD) activity.
**Methods or Backround:** 67 consecutive CD patients (39 men and 28 women, mean age: 39 years old) underwent MRE with the standard protocol and subsequently performed endoscopy within 7 days, over an 18-month period. One radiologist with >5 years of experience in GI imaging reviewed MRE images on the selected fast protocol at first, and then on the standard one. 74 bowel segments were assessed to establish disease activity. MRE analysis was blinded to patient data and endoscopy results. As primary endpoint, the reliability of the abbreviated protocol vs. the standard one was evaluated with Chronbach Alpha Test and Intraclass Coefficient Correlation. As secondary endpoint, the correlation between MRE activity and endoscopic activity was tested with multivariate binary logistic regression. ROC analysis was performed to evaluate diagnostic accuracy of the quantitative parameters.
**Results or Findings:** Statistical analysis revealed a homogeneous evaluation of MRE findings between the two protocols, with overall excellent reliability

Saturday

(ICC: 0.92-1; Chronbach Alpha: 0.91-1) for all the features tested and good reliability for stenosis (ICC: 0.84). Both protocols showed perfect correlation with endoscopy, with edema and DWI being the most accurate features in predicting endoscopic activity (sensitivity: 88.9%; specificity: 96.4%; PPV: 94.6%, AUC: 0.976).
**Conclusion:** The proposed fast MRE protocol was non-inferior to the conventional one in the assessment of CD activity using endoscopy as reference standard.
**Limitations:** Retrospective, single-centre study.
**Ethics committee approval:** No information provided by the submitter.
**Funding for this study:** No funding was received for this study.
**Author Disclosures:**
Mariateresa Del Canto: Nothing to disclose
Giovanna Vacca: Nothing to disclose
Salvatore Cappabianca: Nothing to disclose
Valentina Caliendo: Nothing to disclose
Valerio Nardone: Nothing to disclose
Roberto Grassi: Nothing to disclose
Vittorio Patanè: Nothing to disclose
Alfonso Reginelli: Nothing to disclose

**14:00-15:30** **Research Stage 2**

### Research Presentation Session: Breast

### RPS 2002

### Advanced applications in MRI of the breast and contrast-enhanced mammography (CEM)

**Moderator**
G. Esen; Istanbul/TR
*(gulesenicten@gmail.com)*

### RPS 2002-2

**Completely automated analysis in breast proton MR spectroscopy: can it be done?**
D. Javor[1], *B. Bennani-Baiti*[1], P. Baltzer[2]; [1]Krems/AT, [2]Vienna/AT

**Purpose:** The study aimed to assess how fully automated software compares against the human expert interpretation of breast 1H-MRS spectra in diagnosing breast lesions.
**Methods or Backround:** Multiparametric contrast-enhanced breast MRI and single-voxel proton MR spectroscopy (1H MRS) of 74 consecutive patients were acquired on a 3T PET-MRI scanner and automatically imported into and analysed by the SpecTec-ULR 1.1 software (LifeTec Solutions GmbH). Additionally, all ensuing spectra were independently analysed and interpreted by two blinded radiologists. Histopathology +/> 24 months of imaging follow-up served as the reference standard. Non-parametric Spearman correlation coefficients for all measured parameters (SNR and integral of tCho), Passing and Bablok regression and receiver operating characteristics (ROC) analysis were calculated to assess diagnostic test performance as well as to compare automated vs manual reading.
**Results or Findings:** Based on 117 spectra of 74 patients, the AUC for tCho SNR and integrals ranged from 0.768 to 0.814 and from 0.721 to 0.784 to distinguish benign from malignant tissue, respectively. Neither displayed significant differences between measurements (automated vs human expert readers, p>0.05), in line with the results from the univariate Spearman rank correlation coefficients and the Passing and Bablok regression analysis.
**Conclusion:** This pilot study demonstrates that 1H-MRS data from breast MRI can be automatically exported and interpreted without human interaction by the SpecTec-ULR 1.1 software. The diagnostic performance of this software was not inferior to human expert readers.
**Limitations:** This is only a pilot study that needs to be confirmed in a prospective multi-centre trial.
**Ethics committee approval:** Not applicable.
**Funding for this study:** This study was partially funded by the Austrian National Bank (ID 17186).
**Author Disclosures:**
Barbara Bennani-Baiti: Nothing to disclose
Domagoj Javor: Nothing to disclose
Pascal Baltzer: Nothing to disclose

### RPS 2002-3

**Advanced DWI sequences in breast MRI, comprehensive comparison of improved sequences and ultra-high b-values: what is the perfect match?**
*D. Hausmann*[1], I. Todorski[1], A. Pindur[1], E. Weiland[2], T. Benkert[2], L. Bosshard[3], M. Prummer[3], R. Kubik-Huch[1]; [1]Baden/CH, [2]Erlangen/DE, [3]Zurich/CH
*(daniel.hausmann@ksb.ch)*

**Purpose:** The study aimed to compare two diffusion-weighted imaging (DWI) techniques in breast MRI with a standard DWI concerning image quality and ADC.
**Methods or Backround:** 40 patients (malignancy (n=20)) were included. In addition to the standard sequence (s-DWI)) with two measured b-values (b50, b800) and three extrapolated (e) b-values (e-b1500-, e-b2000-, and e-b2500-values), two prototypical sequences (3D zoomed (z)-DWI with SPAIR fat saturation (fs) (3D z-DWI) and DWI with improved IR fs (IR m-b1500 DWI)) were applied. 3D z-DWI was acquired with the same measured b-values (b50 and b800) and the same the e-b-values. In IR m-b1500 DWI b50 and b1500 were measured, and e-b2000 and e-b2500 were mathematically extrapolated. Three readers analysed three b-values (m-b1500/e-b1500, e-b2000 and e-b2500) for each DWI regarding scan preference and image quality parameters according to Likert scales. ADCs were measured in all 20 lesions.
**Results or Findings:** 3D z-DWI was the most preferred sequence (54%), followed by IR m-b1500 DWI (46 %). B1500 was significantly preferred over b2000 in 3D z-DWI and IR m-b1500 DWI (p=0.001, p=0.002). Lesion detection was not significantly different between the sequences and b-values (p=0.174). There was a non-significant trend toward lower values in IR m-b1500 DWI (ADC: 0.80 (±0.06) x 10-3mm2/s) compared to s-DWI (p=0.090) and 3D z-DWI (p=0.110).
**Conclusion:** Across all b-values, a significant improvement of the image quality and especially a reduction of image artefacts is achieved by 3D z-DWI + IR m-b1500 DWI. 3D z-DWI with a calculated b1500 is the recommended combination taking into account the scan preference, but also the examination time and ADC calculation.
**Limitations:** The study is limited by a small collective, subjective image quality analysis and 1.5T examinations.
**Ethics committee approval:** The ethics committee of Northwestern- and Central Switzerland (ID: 2020-00408) approved this study.
**Funding for this study:** No funding was provided for this study.
**Author Disclosures:**
Elisabeth Weiland: Employee: Employee at Siemens Healthineers
Michael Prummer: Nothing to disclose
Alexandra Pindur: Nothing to disclose
Inga Todorski: Nothing to disclose
Lars Bosshard: Nothing to disclose
Rahel Kubik-Huch: Nothing to disclose
Thomas Benkert: Employee: Employee at Siemens Healthineers
Daniel Hausmann: Equipment Support Recipient: Sequences were provided for research purposes at no cost

### RPS 2002-4

**Repeatability of normal fibroglandular total sodium concentration measurements at 3T**
*O. Arponen*, J. Kaggie, M. McLean, M. Nanaa, G. C. Baxter, M. J. Graves, R. Manavaki, F. J. Gilbert; Cambridge/UK
*(otso.arponen@tuni.fi)*

**Purpose:** Sodium-(23Na)-MRI may be able to differentiate malignant breast tumours from benign tumours and normal fibroglandular tissue (FGT). Furthermore, the total tissue sodium concentration (TSC) provides information about the cellularity of tumours, suggesting that the TSC could be used as an imaging biomarker of therapy response.
**Methods or Backround:** The local ethics committee approved this prospective study, and the volunteers gave written informed consent. Ten normal volunteers (24-65 years) were scanned in the prone position with a dual-tuned bilateral Na/H breast coil (Rapid Biomedical, Rimpar, Germany) on a 3T system (MR750, GE Healthcare, Waukesha, WI, USA). 23Na-MRI was performed using a 3D cones UTE trajectory; scan time for the sequence was approximately nine minutes. T1-/T2-weighted proton images were acquired. Two radiologists (with more than four years of experience) drew large freehand region-of-interests (ROIs) covering as much FGT as possible and small ROIs covering the area with the highest signal on the sodium images at the level of the nipple while referencing the T1W-/T2W-images. For calibration, ROIs were drawn on a phantom with a sodium concentration of 80mM. B1 correction was applied, and both breasts were used independently in the analyses.
**Results or Findings:** One volunteer was excluded because the phantom was poorly visible. The mean TSC using LROI vs SROI methods were 53.6±18.2mmol/l vs 69.5±24.4mmol/ (observer 1; P=0.033) and 53.1±16.4mmol 62.3±16.4mmol/l (observers 2; P>0.05) (the independent Student T-test). The SMICC for LROI was 0.800 (95% confidence interval (CI): 0.542-0.920) and for SROI 0.738 (95% CI: 0.425-0.893) (the single measures Intraclass Correlation Coefficient).

**Conclusion:** Although TSC measurements are reproducible, the ROI sampling method may affect the concentration values. This warrants the future evaluation of the reproducibility of TSC values in patients with breast cancer.
**Limitations:** The study is limited by its sample size.
**Ethics committee approval:** The CUH Research Ethics Committee approval was obtained.
**Funding for this study:** Not applicable.
**Author Disclosures:**
Otso Arponen: Nothing to disclose
Roido Manavaki: Nothing to disclose
Fiona J. Gilbert: Nothing to disclose
Muzna Nanaa: Nothing to disclose
Mary McLean: Nothing to disclose
Martin J Graves: Nothing to disclose
Joshua Kaggie: Nothing to disclose
Gabrielle Carmen Baxter: Nothing to disclose

### RPS 2002-5

### First results of novel deep-learning accelerated T2-weighted TSE imaging in 3T breast MRI

*C. Wilpert*, S. Hannah, M. Reisert, A. Rau, C. Neubauer, F. Bamberg, M. Windfuhr-Blum, M. Benndorf, J. Neubauer; Freiburg im Breisgau/DE

**Purpose:** The study aimed to compare the novel deep-learning accelerated T2-weighted TSE imaging (DL-T2) to a standard T2 TSE sequence for breast MRI with an evaluation of overall image quality features in intra-individual comparison.
**Methods or Backround:** Prospective pseudo-anonymized blinded study on 101 women with a mean age of 52 years (range: 24–81) who underwent 3T breast MRI with a standard breast imaging protocol and a novel DL reconstructed T2 sequence. Imaging parameters for DL-T2 and T2 were set identically with a spatial resolution of 0.6x0.6x3.0mm. Quantitative analysis (SNR) was performed ROI based. Two radiologists rated images with Likert Scales for qualitative analysis with intra-individual comparisons.
**Results or Findings:** The mean acquisition time was 01:50min for DL-T2 compared to 04:29min for standard T2 ($p < .001$). There was a statistically significant difference in SNR in DL-T2 and T2 in intra-individual comparison (52±25 vs. 37±17, $p < .001$). Image quality was improved for DL-T2 with excellent quality in 84/101 cases compared to 7/101 cases for standard T2 ($p < .001$). With exception of bone marrow, which was improved in standard T2 (3.9±0.4 vs. 3.1±0.4, $p <.001$), the visibility of all anatomic regions was improved in DL-T2 compared to standard T2 ($p< .001$). When lesions were visible, lesion conspicuity was rated higher in DL-T2. No additional artifacts were observed for DL-T2.
**Conclusion:** DL-T2 for breast imaging results in a reduction of acquisition time of 41% compared to standard T2. Overall, the image quality features of DL-T2 were improved compared to standard T2.
**Limitations:** Only a small cohort was assessed.
**Ethics committee approval:** The ethics committee approval can be found under number EK 22-1185.
**Funding for this study:** Internal funding was provided for this study.
**Author Disclosures:**
Caroline Wilpert: Nothing to disclose
Jakob Neubauer: Nothing to disclose
Marco Reisert: Nothing to disclose
Alexander Rau: Nothing to disclose
Claudia Neubauer: Nothing to disclose
Matthias Benndorf: Nothing to disclose
Fabian Bamberg: Nothing to disclose
Marisa Windfuhr-Blum: Nothing to disclose
Schneider Hannah: Nothing to disclose

### RPS 2002-6

### Low-dose CT and MRI-based texture and perfusion analysis for correlations with prognostic biomarkers and survival outcomes in patients with breast cancer: a single-centre prospective cohort study

*H. Y. Kim*[1], B. K. Seo[1], J. Y. Lee[2], K. R. Cho[3], O. Woo[3], S. E. Song[3]; [1]Ansan/KR, [2]Goyang-si/KR, [3]Seoul/KR

**Purpose:** Computerised texture and perfusion analysis using CT and MRI can assess tumour heterogeneity and angiogenesis. There are limited data comparing CT and MRI in predicting breast cancer prognosis. This study explored the association of texture and perfusion parameters with histological biomarkers and survival outcomes using concurrently performed CT and MRI in breast cancer patients.
**Methods or Backround:** 147 patients with invasive breast cancer underwent dynamic contrast-enhanced breast MRI and low-dose perfusion CT before treatment. We analysed texture and perfusion features and compared those imaging parameters with histological biomarkers and subtypes. Patients were dichotomised into high and low groups according to the median value of each CT and MRI parameter. The Kaplan-Meier method was used to estimate progression-free survival (PFS). We performed survival analysis using the Cox proportional hazards model.
**Results or Findings:** Out of 54 texture and perfusion parameters, entropy (texture irregularity) on T2- and postcontrast T1-weighted MRI and postcontrast CT, and perfusion (mL/min per 100 mL) on CT were significantly associated with hormone receptors, human epidermal growth factor receptor 2, and molecular subtypes ($P<0.05$). Patients with high entropy on postcontrast CT showed worse PFS than patients with low entropy on postcontrast imaging ($P=0.05$), whereas entropy on postcontrast T1-weighted MRI was not associated with PFS ($P=0.30$). In particular, high entropy on postcontrast CT showed a significant negative impact on the PFS in patients with high Ki67 ($P=0.046$).
**Conclusion:** Low-dose CT texture and perfusion analysis were comparable to MRI, and the texture irregularity of postcontrast CT could be a feasible parameter to predict PFS in breast cancer patients.
**Limitations:** The small number of patients and two-dimensional texture analysis have limitations.
**Ethics committee approval:** Not applicable.
**Funding for this study:** The study is funded by the Korean government (No. NRF-2021R1A2C1010565).
**Author Disclosures:**
Okhee Woo: Nothing to disclose
Hyo Young Kim: Nothing to disclose
Bo Kyoung Seo: Nothing to disclose
Sung Eun Song: Nothing to disclose
Ji Young Lee: Nothing to disclose
Kyu Ran Cho: Nothing to disclose

### RPS 2002-7

### Clinical feasibility of virtual monochromatic dual-layer computed tomographic imaging for preoperative staging for breast carcinoma: comparison with breast MRI

*B. d. Yeo*, W. H. Kim, H. J. Kim; Daegu/KR
*(dong2737@naver.com)*

**Purpose:** Dual-layer CT (DL-CT) can create virtual monochromatic images (VMIs) at different monochromatic x-ray energies, and VMIs at low keV are known to have a high contrast-noise ratio. We aimed to evaluate the clinical feasibility of chest DL-CT with contrast enhancement to assess breast cancer extent for preoperative tumour staging in comparison with breast MRI.
**Methods or Backround:** A total of 153 breast cancer patients underwent DL-CT for initial work-up. Virtual monochromatic images (40–200 keV) were generated, and MIP reconstruction with low energy (40 keV) images was performed for both breast areas. Two radiologists reviewed DL-CT and breast MRI in consensus without knowledge of index cancer information, including laterality and size. The detection rate of index cancer was compared between DL-CT and breast MRI, and correlation and agreement of tumor size between two imaging modalities was evaluated.
**Results or Findings:** Among index cancer, 85% (131/155) was found in DL-CT, and 88% (137/155) was found in breast MRI. The absolute difference of mean tumour size between DL-CT (23.2mm) and breast MRI (24.4mm) was 1.2mm. DL-CT and breast MRI was highly correlated (correlation coefficient = 0.841, $P < .001$) and had an excellent agreement in tumour size assessment (intraclass correlation coefficient = 0.811 [95% CI = 0.739, 0.863]. Breast MRI showed a slightly higher tendency of overestimation compared to chest DL-CT.
**Conclusion:** Chest DL-CT was feasible to assess breast cancer extent for preoperative tumour staging with an excellent agreement in tumour size compared to breast MRI.
**Limitations:** First, we excluded patients who underwent neoadjuvant chemotherapy, and this could make selection bias. Second, results may vary depending on the reconstruction skill.
**Ethics committee approval:** No information provided by the submitter.
**Funding for this study:** Nothing to disclose.
**Author Disclosures:**
Hye Jung Kim: Nothing to disclose
Bok dong Yeo: Nothing to disclose
Won Hwa Kim: Nothing to disclose

### RPS 2002-8

### Background parenchymal enhancement on contrast-enhanced mammography: a preliminary analysis of associations with breast density and patients' characteristics

*V. Magni*[1], A. Cozzi[2], G. Muscogiuri[1], A. Benedek[2], F. Sardanelli[1]; [1]Milan/IT, [2]San Donato Milanese/IT
*(veronica.magni@unimi.it)*

**Purpose:** The study aimed to evaluate the association of background parenchymal enhancement (BPE) on contrast-enhanced mammography (CEM) with breast density (BD), menstrual status, and age.
**Methods or Backround:** This retrospective analysis included CEM performed for second-level assessment of suspicious mammographic findings. According to the Breast Imaging Reporting and Data System (BI-RADS) scales, three

Saturday readers independently and blindly evaluated BPE on recombined CEM images and BD on low-energy CEM images. Inter-rater reliability was estimated using Fleiss κ. Correlations of BPE with BD, menopause, and age were assessed using Kendall's τB and Spearman's ρ. Multivariable binary logistic regression was performed, dichotomising BD and BPE as low (a/b BI-RADS categories, minimal/mild BPE) and high (c/d BI-RADS categories, moderate/marked BPE).
**Results or Findings:** A total of 116 women (median age 57.4 years, interquartile range 51.4–67.2, 86/116 in menopause) were included. BD was classified as a in 16/116 (13.8%), as b in 64/116 (55.2%), as c in 30/116 (25.9%), and as d in 6/116 (5.2%), with moderate inter-rater reliability (κ=0.55; 95% confidence interval [CI] 0.46–0.61). BPE was minimal in 63/116 (54.3%), mild in 36/116 (31.0%), moderate in 10/116 (8.6%), marked in 7/116 (6.0%), with substantial inter-rater reliability (κ=0.64; 95% CI 0.57–0.72). A moderate positive correlation was found between BPE and BD (ρ=0.36, p<0.001), while moderate negative correlations were found with menopause (ρ=-0.40, p<0.001) and age (τB=-0.31, p<0.001). At multivariable regression, high BD was strongly associated with high BPE (odds ratio [OR] 5.37, 95% CI 1.65–17.55, p=0.005), whereas menopause showed a strong negative association with high BPE (OR 0.13, 95% CI 0.04–0.43, p=0.001).
**Conclusion:** BPE on CEM is associated with well-established breast cancer risk factors, being higher in women with higher BD, premenopausal status, and younger age.
**Limitations:** Single-centre preliminary analysis.
**Ethics committee approval:** The ethics committee of IRCCS Ospedale San Raffaele, Milan, Italy (protocol code CESM; approved May 10th, 2018) approved the study.
**Funding for this study:** Unconditional grant from GE Healthcare was received.
**Author Disclosures:**
Andrea Cozzi: Nothing to disclose
Giulia Muscogiuri: Nothing to disclose
Francesco Sardanelli: Speaker: Bayer Advisory Board: Bracco Speaker: Bracco Advisory Board: DeepTrace Technologies S.R.L Grant Recipient: GE Healthcare Speaker: GE Healthcare
Veronica Magni: Nothing to disclose
Adrienn Benedek: Nothing to disclose

### RPS 2002-9

**Reducing contrast volume in contrast-enhanced mammography (CEM) in breast cancer patients: a randomised-controlled feasibility study**

*T. van Nijnatten*[1], L. Neeter[1], P. Nelemans[1], S. Gommers[1], S. Vanwetswinkel[1], C. Mihl[1], M. Smidt[1], J. E. Wildberger[1], M. Lobbes[2]; [1]Maastricht/NL, [2]Zuyderland/NL
*(Thiemo.nijnatten@mumc.nl)*

**Purpose:** The current study aimed to investigate the diagnostic accuracy of contrast-enhanced mammography (CEM) in terms of maximum tumour diameter measurements and degree of lesion enhancement when reducing the volume of contrast administration in breast cancer patients.
**Methods or Backround:** A prospective randomised-controlled feasibility study was performed in patients with a recent breast cancer diagnosis, including clinical CEM as a diagnostic workup. Patients were randomised to undergo a second ('experimental') CEM using a reduced volume of iodinated contrast agent: 80%, 60% or 40% of the initial volume of the clinical CEM. Three readers reviewed all cases to determine the maximum tumour diameter and degree of lesion enhancement: no, possible, weak, moderate or strong. Tumour size measurement per lesion on clinical and experimental CEM exam per reader was analysed by using the Wilcoxon-signed rank test. Interobserver agreement among the three readers regarding the degree of lesion enhancement was calculated by quadratic weighted k coefficient for clinical and experimental CEM, respectively.
**Results or Findings:** Ten breast cancer patients were included, with 13 enhancing cancers, and all patients underwent both clinical and experimental CEM. Tumour size measurements among clinical and experimental CEM per reader were not significantly different (reader 1: mean tumour diameter: 24 vs 25mm, p=0.687; reader 2: mean tumour diameter 23 vs 20mm, p=0.647; reader 3: mean tumour diameter: 29 vs 21mm, p=0.155). Interobserver agreement differed among the readers on clinical CEM compared to experimental CEM (reader 1 vs reader 3: 0.593 to 0.122; reader 2 vs reader 3: 0.546 to 0.734).
**Conclusion:** Reducing the volume of iodinated contrast agents in CEM does not seem to affect tumour diameter measurements, although interobserver agreement regarding the degree of lesion enhancement differed.
**Limitations:** Small sample size in a feasibility study.
**Ethics committee approval:** This prospective randomised-controlled feasibility study was approved by the medical ethical committee of Maastricht University Medical Center + (project 162048).
**Funding for this study:** The funding for this study was provided by Bayer.
**Author Disclosures:**
Marjolein Smidt: Nothing to disclose
Sigrid Vanwetswinkel: Nothing to disclose
Lidewij Neeter: Nothing to disclose
Casper Mihl: Speaker: Bayer
Patty Nelemans: Nothing to disclose
Marc Lobbes: Research/Grant Support: GE Healthcare Advisory Board: Hologic, Tromp Medical, Bayer, Guerbet Speaker: GE Healthcare, Tromp Medical, Bayer, Guerbet
Joachim E. Wildberger: Research/Grant Support: Agfa, Bard, Bayer, Cook, GE Healthcare, Philips, Optimed, Radiomics, Siemens Speaker: Bayer, Siemens
Suzanne Gommers: Nothing to disclose
Thiemo van Nijnatten: Speaker: GE Healthcare

### RPS 2002-10

**Contrast-enhanced mammography (CEM) as an alternative to breast MRI for screening of women at increased risk for breast cancer: preliminary results**

*G. Gennaro*[1], E. Baldan[1], P. Belli[2], D. Bernardi[3], E. Bezzon[1], G. Ciriello[1], V. Iotti[4], I. Polico[1], F. Caumo[1]; [1]Padova/IT, [2]Rome/IT, [3]Rozzano/IT, [4]Reggio Emilia/IT
*(gisella.gennaro@iov.veneto.it)*

**Purpose:** The study aimed to compare the performance of CEM and breast MRI for screening women at increased risk for breast cancer.
**Methods or Backround:** This preliminary retrospective analysis included 106 of 500 women at increased risk for breast cancer who prospectively underwent both CEM and MRI between March 2019 and October 2022. Low-energy (LE) CEM as a surrogate of mammography, CEM and MRI were reviewed by six independent radiologists. The ground truth was obtained from histology and/or at least a one-year follow-up and used to build the individual receiver operating characteristic (ROC) curves for each modality. The comparison between modality pairs was obtained from the differences between the areas under the average ROC curves (AUCs). P<0.05 was considered statistically significant.
**Results or Findings:** The mean age was 55 (53-74). Of 106 patients, 23/106 (22%) were at intermediate risk, and 83/106 (78%) were at high risk. Fifty-one lesions were diagnosed either by CEM or MRI or both, 22/51 (43%) malignant and 29/51 (57%) benign. The mean AUCs for CEM were significantly better than LE-CEM (LE-CEM: 0.796; CEM: 0.954; p=0.0156), while the AUC difference between CEM and MRI was not statistically significant (CEM; 0.954; MRI=0.924; p=0.3673).
**Conclusion:** CEM confirms its superiority vs LE-CEM (i.e. mammography) and its non-inferiority vs breast MRI. Thereby, CEM might be an alternative for screening women at increased risk for breast cancer.
**Limitations:** The study is limited by a preliminary dataset.
**Ethics committee approval:** The ethics committee approval can be found under the number CESM2018.
**Funding for this study:** The study is funded, and more information can be found under grant number RSF-2017-00000562.
**Author Disclosures:**
Valentina Iotti: Nothing to disclose
Francesca Caumo: Nothing to disclose
Enrica Baldan: Nothing to disclose
Elisabetta Bezzon: Nothing to disclose
Daniela Bernardi: Nothing to disclose
Gisella Gennaro: Nothing to disclose
Ilaria Polico: Nothing to disclose
Giovanna Ciriello: Nothing to disclose
Paolo Belli: Nothing to disclose

### RPS 2002-11

**Contrast-enhanced mammography (CEM) enhancing asymmetry: a single-centre first case analysis**

*G. Migliaro*[1], G. Bicchierai[1], F. Di Naro[1], D. De Benedetto[1], F. Amato[2], J. Nori[1], V. Miele[1]; [1]Florence/IT, [2]Agrigento/IT
*(giuliano.migliaro@gmail.com)*

**Purpose:** The study aimed to define which kind of lesions the enhancing asymmetry (EAs), termed in the BI-RADS lexicon for CEM, is related with.
**Methods or Backround:** 3359 CEM exams, executed at AOUC Careggi in Florence, Italy, between 2019 and 2021, were retrospectively assessed by two radiologists. For each one of the EAs found, we described the size, the enhancing conspicuity (degree of enhancement relative to background described as low, moderate, or high), and whether there was a corresponding finding in the traditional radiology images (US or mammography), the biopsy results when performed, including any follow-up exams and the presence of BPE of normal breast tissue (minimal, mild, moderate, marked).
**Results or Findings:** The population comprised 65 women (mean age 56,75 years, median age 52 years). 37 underwent CEM for a preoperative staging assessment, and 28 for a problem-solving examination. Among 65 EAs, after biopsy, 18/65 (27,69%) resulted as B5 lesions, 4/65 (6,15%) as B3 lesions, and 43/65 (66,15%) were negative or benign either after biopsy or 12 months follow-up. Using the Fisher test, we examined if one among enhancing conspicuity, BPE, or a corresponding US or mammography sign, linked to a higher rate of B5 lesions. EAs with higher enhancing conspicuity (from moderate to high) correlate with a higher risk of B5 lesions (p: 0,00236), while there was no statistic correlation with BPE grade or traditional radiology sign.
**Conclusion:** EAs can relate both with benign and tumoural lesions, and they need to be assessed as the other CEM descriptors, moreover, since EAs with higher enhancing conspicuity correlate with a higher risk of B5 lesions.
**Limitations:** It is a single-centre retrospective study based on a restrained population.
**Ethics committee approval:** No information provided by the submitter.
**Funding for this study:** This research received no external funding.
**Author Disclosures:**
Giulia Bicchierai: Nothing to disclose
AOU Careggi Diego De Benedetto: Nothing to disclose
Jacopo Nori: Nothing to disclose
Vittorio Miele: Nothing to disclose
Giuliano Migliaro: Nothing to disclose
Federica Di Naro: Nothing to disclose
Francesco Amato: Nothing to disclose

### RPS 2002-12

**The role of contrast-enhanced mammography in the preoperative evaluation of invasive lobular carcinoma of the breast**

*E. Giannotti*[1], Y. Chen[2], G. Bicchierai[3], J. Nori[3], D. De Benedetto[3], U. Lalji[4], T. van Nijnatten[4], J. James[2]; [1]Cambridge/UK, [2]Nottingham/UK, [3]Florence/IT, [4]Maastricht/NL

**Purpose:** The study aimed to assess the performance of contrast-enhanced mammography (CEM) in the preoperative staging of breast invasive lobular carcinoma (ILC).
**Methods or Backround:** Women with an ILC who had undergone CEM (2013-2021) were included in this multicentre, multivendor retrospective study. Index lesion size and multifocality were recorded for 2D mammography (FFDM), CEM, and, when available, MRI. Comparison with histological data was made for the women treated by primary surgical excision. Pearson correlation coefficients, analysis of variance (ANOVA), and odds ratios (OR) were used to assess differences with a significance level of .05.
**Results or Findings:** 137 women from three centres (median age 58.5 (34-85)) were included in the study. 49.6% (68/137) presented symptomatically 50.4% (69/137) were screen-detected. 84% (115/137) underwent primary surgical excision. CEM's diagnostic accuracy was greater than FFDM (74.6% vs 50.4% OR=2.88, p=.0002). No tumour was seen in 22 FFDM. There was a significantly higher positive correlation between histological size at surgery and CEM size compared with FFDM size (CC r=.626 vs r=.442, p=.033). 60.9% (70/115) were found to have multi-focal disease following surgery. The sensitivity of CEM for detecting multifocal disease was 70% (49/70), which was significantly higher than FFDM 20% (14/70) (OR=9.33, p<0.0001). 29 underwent both CEM and MRI. The mean lesion size on MRI was 27.1mm and was significantly greater than the mean histological size of 20.6mm (Z=-2.275, p=.023). The mean CEM size for these women was 22.1mm and was equivalent to the histological size (Z=-0.674, p=.511).
**Conclusion:** CEM is a useful tool for staging women with ILC, and it provides superior local staging information for lesion size and multifocality compared to FFDM. MRI overestimated lesion size compared to CEM, CEM could be an alternative to breast MRI.
**Limitations:** The study is limited by being a retrospective study.
**Ethics committee approval:** Not applicable.
**Funding for this study:** No funding was provided for this study.
**Author Disclosures:**
Diego De Benedetto: Nothing to disclose
Giulia Bicchierai: Nothing to disclose
Jacopo Nori: Nothing to disclose
Elisabetta Giannotti: Nothing to disclose
Thiemo van Nijnatten: Nothing to disclose
Ulrich Lalji: Nothing to disclose
Jonathan James: Nothing to disclose
Yan Chen: Nothing to disclose

### RPS 2002-13

**Comparing CESM and CE-MRI in the evaluation of enhancing secondary lesions detected by CESM**

I. Ş. S. Örgüç, *Ç. R. Açar*; Manisa/TR

**Purpose:** The presence of secondary malignant lesions has an important role in the management of the patient and the decision on surgical treatment type. CE-MRI is the gold standard in detecting secondary malignant lesions. CESM is an imaging modality with functional properties, and its performance is comparable with CE-MRI. The aim of this study is to compare the efficiency of CESM and CE-MRI in enhancing secondary lesions detected by CESM.
**Methods or Backround:** We retrospectively reviewed 105 patients with CESM and CE-MRI examinations and pathologically proven breast malignancy. We detected 61 enhancing secondary lesions in addition to primary malignant lesions by CESM. We compared these findings with CE-MRI findings.
**Results or Findings:** Among secondary enhancing lesions, 40 (65.57%) were malign, and 21 (34.42%) were benign. 25 multifocal, nine multicentric and six bilateral malignancies were diagnosed. In the detection of malignancy in the enhancing secondary lesions, CESM had 97.50% sensitivity, 71.42% specificity, 86.66% positive predictive value (PPV), 93.75% negative predictive value (NPV); CE-MRI had 100% sensitivity, 66.66% specificity, 91.11% PPV, 100% NPV. AUC were 0.845 for CESM and 0.833 for CE-MRI. CE-MRI showed higher sensitivity but lower specificity than CESM.
**Conclusion:** The diagnostic performance of CESM is comparable with the CE-MRI in the evaluation of enhancing secondary lesions.
**Limitations:** Our sample size was limited.
**Ethics committee approval:** Our study has been approved by Celal Bayar University ethics commitee.
**Funding for this study:** No funding to declare.
**Author Disclosures:**
Ihsan Şebnem Sebnem Örgüç: Nothing to disclose
Çağdaş Rıza Açar: Nothing to disclose

**14:00-15:30** | **Research Stage 3**

### Research Presentation Session: Interventional Radiology

### RPS 2009

### Interventional oncology (part 2)

**Moderator**
S. Boudabbous; Geneva/CH
*(sanaboudabous@yahoo.fr)*

### RPS 2009-2

**Are histomorphological patterns a predictor for survival in uveal melanoma patients with hepatic metastases undergoing hepatic artery infusion chemotherapy?**

*H. Steinberg*, S. Ting, S. Zensen, J. M. Ludwig, Y. Li , H. Richly, J. Grueneisen, J. Theysohn, B. M. Schaarschmidt; Essen/DE

**Purpose:** Hepatic artery infusion chemotherapy (HAIC) is an established palliative treatment option in patients with hepatic metastases from uveal melanoma. The purpose of this study was to evaluate the predictive value of two histomorphological patterns (spindle cell vs epitheloid) for overall survival (OS) in patients undergoing HAIC.
**Methods or Backround:** We performed a retrospective analysis on patients with hepatic metastases that underwent liver biopsy prior to HAIC. Histomorphological patterns of these metastases were analysed by a board certified pathologist and were classified as either predominant spindle cell or epitheloid pattern. Mean overall survival (OS) between both patterns was analysed using Kaplan-Meier curves and the log-rank test. p<0.05 indicated statistical significance.
**Results or Findings:** 60 uveal melanoma patients (29 female, 31 male, mean age: 61.6 ± 12.1 years) with hepatic metastases were analysed. In 73.3% (44/60) patients, an epitheloid pattern, in 21.7% (13/60) a spindle cell pattern and in 5% (3/60) other patterns were found. Mean survival in both groups was comparable (epitheloid pattern - OS: 19.2 months, 95% CI, 14.5 to 23.9; spindle cell pattern - OS: 19.9 months, 95% CI, 10.3 to 29.6). No significant differences between both patterns were detected by the log-rank test ($\chi^2(2)=0.22$, p=0.881).
**Conclusion:** In patients with hepatic metastases from uveal melanoma undergoing HAIC, histomorphological patterns are not a suitable predictor for survival.
**Limitations:** Due its retrospective nature and its small sample size, the result of the study is limited and selection bias might be possible.
**Ethics committee approval:** The local institutional review board approved this study and the demand to obtain informed consent was waived (20-9749-BO).
**Funding for this study:** No funding was received for this study.
**Author Disclosures:**
Jens Theysohn: Other: Boston Scientific
Johannes Grueneisen: Nothing to disclose
Yan Li : Nothing to disclose
Benedikt Michael Schaarschmidt: Research/Grant Support: PharmaCept
Johannes Maximilian Ludwig: Nothing to disclose
Sebastian Zensen: Nothing to disclose
Saskia Ting: Nothing to disclose
Heike Richly: Nothing to disclose
Hannah Steinberg: Nothing to disclose

### RPS 2009-3

**Combination of TARE and extrahepatic TACE to treat HCC with extrahepatic artery supply: a case series**

L. C. Pescatori, A. Galletto Pregliasco, H. Derbel, A. Luciani, S. Mule, H. Kobeiter, V. Tacher, *L. Saccenti*; Créteil/FR

**Purpose:** The aim of this study was to report safety, feasibility and tumour response rate of hepatocellular carcinoma (HCC) presenting with an intrahepatic and extrahepatic arterial supply treated by combined trans-arterial radioembolisation (TARE) through the intrahepatic arteries and trans-arterial chemoembolisation (TACE) through an extrahepatic feeding artery (EHFA).
**Methods or Backround:** This single centre feasibility study included all patients with HCC showing both intrahepatic and extrahepatic arterial supply on multiphase CT treated between 2018 and 2021. Demographic and clinical characteristics were obtained, including age, gender, presence of cirrhosis, and previous treatments. Technical and clinical results were recorded. The tumour response was evaluated using mRECIST method.
**Results or Findings:** Nine patients (8 men, mean age 62 [SD ±11]) were included, 7 with liver cirrhosis, 5 patients previously treated by TARE, 2 by TACE and 1 had contralateral lobectomy. Mean lesion diameter was 77mm(SD±37). Eight patients had a single lesion, 3 had portal vein invasion (2 patients VP1 and 1 VP3). The EHFA was a branch of the diaphragmatic artery in 6 cases, of the adrenal artery in 2 cases and of the left gastric artery in 1 case. The mean diameter of the part treated with TACE was 49mm(SD±15.5). The ratio between the whole lesion and the mean diameter of the part treated with TACE was 1.6(SD±0.4). Mean follow up was 22 months (SD±13). Five patients had further treatment: 2 percutaneous ablation and 3 chemotherapy. At the end of follow up, 5 patients were still alive: 1 patient was in progression disease, 1 patient had stable response, 1 patient partial response, 2 patients had complete response.
**Conclusion:** Combination of TARE and extrahepatic TACE seems feasible, safe and efficient. Further studies are needed to validate these preliminary results
**Limitations:** Few patients. Retrospective study.
**Ethics committee approval:** No information provided by the submitter.
**Funding for this study:** No funding was received for this study.
**Author Disclosures:**
Vania Tacher: Nothing to disclose
Laetitia Saccenti: Nothing to disclose
Haytham Derbel: Nothing to disclose
Lorenzo Carlo Pescatori: Nothing to disclose
Alain Luciani: Nothing to disclose
Athena Galletto Pregliasco: Nothing to disclose
Sebastien Mule: Nothing to disclose
Hicham Kobeiter: Nothing to disclose

### RPS 2009-4

**Factors impacting survival after transarterial radioembolisation in hepatocellular carcinoma: results from the prospective CIRT study**

*T. K. K. Helmberger*[1], D. Arnold[2], M. Ronot[3], B. Sangro[4], N. Schaefer[5], H. Pereira[3], B. Zeka[6], N. De Jong[6], F. Kolligs[7]; [1]Munich/DE, [2]Hamburg/DE, [3]Paris/FR, [4]Pamplona/ES, [5]Lausanne/CH, [6]Vienna/AT, [7]Berlin/DE
*(Thomas.Helmberger@muenchen-klinik.de)*

**Purpose:** Trans-arterial radioembolisation (TARE) with Yttrium-90 resin microspheres is an established treatment option for patients with hepatocellular carcinoma (HCC). However, optimising treatment application and patient selection remains challenging. We report here on prognostic factors for effectiveness outcomes, including dosing methods, of the HCC cohort collected in the prospective observational study CIRSE Registry for SIR-Spheres Therapy (CIRT).
**Methods or Backround:** We analysed 422 HCC patients enrolled between Jan 2015 and Dec 2017 and followed up for 24 months. Patient characteristics and treatment-related data were collected at baseline; adverse events and time to event data (overall survival [OS], progression-free survival [PFS] and hepatic PFS) at every 3-month follow-up. We used the multivariable Cox proportional-hazard model and propensity score matching to identify independent prognostic factors for effectiveness outcomes.
**Results or Findings:** The median OS was 16.5 months, the median PFS was 6.1 months, and the median hPFS was 6.7 months. Dosimetry with partition model resulted in improved OS compared to body surface area-based approaches in a multivariable analysis (HR 0.65 [95% CI 0.46-0.92], p=0.0144), which was confirmed in the exact matching propensity score analysis (HR 0.56 (95% CI 0.35-0.89) p=0.0136). Other independent prognostic factors for overall survival were Eastern Cooperative Oncology Group (ECOG) >0 (p=0.0018), presence of ascites (p=0.0152), right-sided tumours (p=0.0002), the presence of lobar portal vein thrombosis (PVT, p=0.0378) and main PVT, (p=0.0028), ALBI grade 2 (p=0.0043) and 3 (p=0.0014).
**Conclusion:** This prospective observational dataset shows that using TARE with partition model dosimetry can significantly benefit survival outcomes of the patients.
**Limitations:** The observational design and lack of source document verification should be considered when interpreting the results.
**Ethics committee approval:** This study has been approved by applicable regulatory bodies and/or ethics committees in Germany, Switzerland, Turkey, Italy, Spain, Israel, France and Belgium as applicable for observational studies.
**Funding for this study:** This study was funded by an unconditional research grant from Sirtex Medical Europe, GmbH.
**Author Disclosures:**
Maxime Ronot: Nothing to disclose
Helena Pereira: Nothing to disclose
Niklaus Schaefer: Nothing to disclose
Dirk Arnold: Nothing to disclose
Bruno Sangro: Nothing to disclose
Bleranda Zeka: Nothing to disclose
Niels De Jong: Nothing to disclose
Thomas K. Karl Helmberger: Nothing to disclose
Frank Kolligs: Nothing to disclose

### RPS 2009-5

**CT hepatic arteriography with hybrid angio-CT system during TACE: a strategy to overcome respiratory motion in patients without breath-hold**

*H. Kim*, H. Jung; Iksan-si/KR

**Purpose:** To compare the difference in imaging qualities according to whether or not respiratory breath hold was performed in CT hepatic arteriography with hybrid angio-CT system during TACE.
**Methods or Backround:** From February 2019 to September 2019, 49 TACE procedures for HCC using hybrid angio-CT system in single-institution were included. The imaging qualities of CT hepatic arteriography for TACE planning were compared between patients with respiration hold (hold, n=31) and patients without respiration hold (breathing, n=18). CT hepatic arteriography images of 105 HCCs (33 in breathing group, 72 in hold group) were reviewed by two radiologists and classified by ordinal scoring system (for tumour identification, tumour feeder vessel identification) and binary scoring system (for breath motion artifact). Data were analysed by Chi-square test or Fisher's exact test.
**Results or Findings:** Comparison of tumour identification and tumour vessel identification were performed in breathing and hold groups for each HCC. Excellent tumour identification was recorded in 40 of 72 (55.6%) hold group and 14 of 33 (42.4%) breathing group. Tumour feeder vessel identification was optimal in 36 of 72 (50%) hold group and 17 of 33 (51.5%) breathing group. There were no significant differences in both tumour identification (p=0.44) and tumour feeder vessel identification (p=0.95). Analysis of breath motion artifact was performed for 49 CT hepatic arteriography images. There were only two cases presenting with respiratory motion artifact (one for each hold and breathing groups, respectively). There was no significant difference between CT hepatic arteriography with respiratory hold and without respiratory hold (p=0.61).
**Conclusion:** Respiratory motion did not degrade image qualities of CT hepatic arteriography with hybrid angio-CT system for intraprocedural TACE planning.
**Limitations:** This study is retrospective and limited to single-centre.
**Ethics committee approval:** No information provided by the submitter.
**Funding for this study:** We received no specific grant from any funding agency.
**Author Disclosures:**
Hyunseok Jung: Nothing to disclose
Hyojoo Kim: Nothing to disclose

### RPS 2009-6

**Selective internal radiation therapy (SIRT) trends for hepatocellular carcinoma, cholangiocarcinoma and liver metastasis: a total population analysis from 2006 to 2020 in Germany**

*S. Thoduka*[1], A. Pfestroff[1], N. Eisenmenger[2], T. Paulus[3], A. H. Mahnken[1], M. Luster[1], N. Abolmaali[3]; [1]Marburg/DE, [2]Hürth/DE, [3]Bochum/DE
*(smita.thoduka@gmail.com)*

**Purpose:** To investigate trends of selective internal radiation therapy (SIRT) for hepatocellular carcinoma (HCC), cholangiocarcinoma (CCC) and liver metastasis in Germany.
**Methods or Backround:** We analysed the nationwide German hospital billing database from 2006 to 2019 for diagnosis of HCC, CCC or liver metastasis in combination with SIRT. For analyses of SIRT on the hospital level, we used the reimbursement INFO tool based on German hospitals' quality reports from 2008 to 2020. Linear regression analysis was performed to detect changes over time.
**Results or Findings:** We included a total of 13981 SIRT procedures. The annual numbers increased from 99 in 2006 to 1605 in 2015 (p<0.001; increase by 1521%) decreasing to 1175 cases in 2019 (p<0.001). In 2008, 6 of 21 hospitals (28.6%) performed more than 20 SIRTs per year, which increased to 19 of 59 (32.2%) in 2020. The share of SIRT for HCC increased from 29.8% in 2006 to 44.7% in 2019 (p<0.001) and for CCC from 0% in 2006 to 9.5% in 2019 (p<0.001) while the share of SIRT for liver metastasis decreased from

Saturday

70.2% in 2006 to 45.7% in 2019 (p<0.001). In-hospital mortality was 0.2% after SIRT procedure. Gastritis (2.73%), liver failure (0.39%) and sepsis (0.32%) were the most common in-hospital complications reported.
**Conclusion:** We observed an increase of SIRT procedures in Germany with the number of hospitals offering the procedure going up from 21 in 2008 to 59 in 2020. While treatment of liver metastasis remains the most common indication, SIRT for HCC and CCC increased significantly over the last years. The mortality and complication rates show SIRT is a relatively safe procedure.
**Limitations:** Retrospective design. Lack of clinical information in databases.
**Ethics committee approval:** The data presented in this study was obtained in accordance with the World Health Association Declaration of Helsinki in its latest version. Analysed data was completely anonymised and derived from established databases with rigorous data protection measures. Therefore, an additional ethics statement was not required.
**Funding for this study:** No funding was received for this study.
**Author Disclosures:**
Tobias Paulus: Nothing to disclose
Andreas Pfestroff: Nothing to disclose
Nasreddin Abolmaali: Nothing to disclose
Smita Thoduka: Nothing to disclose
Nicole Eisenmenger: Nothing to disclose
Markus Luster: Nothing to disclose
Andreas H. Mahnken: Nothing to disclose

### RPS 2009-7

**Tumour enhancement analysis on post-procedural Cone Beam CT after DEB-TACE and TAE for hepatocellular carcinoma: evaluation of response and progression**

A. Della Corte, *S. Parisi*, C. Sorce, L. Augello, G. Guazzarotti, D. Palumbo, S. Gusmini, F. De Cobelli; Milan/IT

**Purpose:** The aim of this study is to quantitively evaluate tumour enhancement on post-procedural CBCT in patients treated with DEB-TACE or TAE and its ability to predict treatment response and progression.
**Methods or Backround:** From February 2020 to July 2022, 23 consecutive patients underwent DEB-TACE (n=10) or TAE (n=13) of 38 HCCs. Patients with complete embolisation, post-procedural CBCT, post-procedural intra-tumour contrast retention were included. Mean density on post-procedural CBCT (mHU*) was calculated by manual tumour contouring. Qualitative analysis included enhancement pattern (homogeneous, inhomogeneous, rim) and evidence of afferent vessels contrast stagnation. mRecist criteria were used to evaluate treatment response on first follow-up CT scan 1 month after the procedure.
**Results or Findings:** Thirteen patients with 17 HCCs met inclusion criteria; six underwent TAE (n=9 lesions, 52.94%) and 7 DEB-TACE (n=8, 47.05%). mHU* did not significantly differ among TAE and DEB-TACE groups (p=0.34) and was not influenced by enhancement pattern (p=0.64). On follow-up CT, 12/17 lesions (70.6%) showed complete response -6/9 following TAE and 6/8 after DEB-TACE. mHU* was significantly higher in those with complete response (p=0.03) and statistically significant in the DEB-TACE group (p=0.046). In lesions with complete response after a median follow-up of 9.2 months, 6/12 (50%) showed progression on target lesions, 4 treated with TAE and 2 with DEB-TACE; mean time to progression was 10.5 and 10 months, respectively. No difference in mHU* was observed in terms of progression (p=0.87); homogeneous enhancement pattern was more frequent in lesions without progression (p=0.05).
**Conclusion:** In patients with HCC treated with DEB-TACE or TAE, no significant differences were observed in efficacy and tumour enhancement. Post-procedural enhancement on CBCT may reflect relative intra-tumoural bead accumulation and be valuable in predicting response.
**Limitations:** Limited sample size.
**Ethics committee approval:** San Raffaele Ethics Committee approval n. 64/INT/2021
**Funding for this study:** No funding was received.
**Author Disclosures:**
Giorgia Guazzarotti: Nothing to disclose
Angelo Della Corte: Nothing to disclose
Diego Palumbo: Nothing to disclose
Simone Gusmini: Nothing to disclose
Carlo Sorce: Nothing to disclose
Francesco De Cobelli: Nothing to disclose
Sergio Parisi: Nothing to disclose
Luigi Augello: Nothing to disclose

### RPS 2009-8

**DEB-TACE with LUMI and SeQure anti-reflux microcatheter for the treatment of HCC**

*G. Pellegrino*, V. Ascenti, C. Lanza, S. Carriero, P. Biondetti, A. M. Ierardi, S. A. Angileri, G. Carrafiello; Milan/IT
*(gspp.pllgrn@gmail.com)*

**Purpose:** To evaluate safety and efficacy of a DEB-TACE treatment with LUMI in combination with an anti-reflux microcatheter (SeQure®, Guebert, Villepinte, France) for the treatment of HCC.
**Methods or Backround:** We performed an observational longitudinal prospective study involving 11 patients with HCC who underwent DEB-TACE with DC Bead LUMI and SeQure microcatheter between July and October of 2022. We evaluated as primary outcomes: number of treated lesions, dose of DC Beads LUMI and doxorubicin, percentage of tumour coverage. Technical success was defined as the correct positioning of the microcatheter as assessed through pre-imaging, clinical success was defined using the mRECIST version 1.1 criteria for HCC. To evaluate the safety we considered intra-procedural and up to one-month post-procedural adverse effects, and all-cause mortality within 30 days of the procedure.
**Results or Findings:** The procedure was performed with ≤ one vial (2 ml) of DC Bead LUMI loaded with 50 mg of Doxorubicin. The tumour coverage was of 80-100% in 68% of cases. A high dilution (1:20) has been obtained adding 40 ml of contrast (Xenetix 350 mg/ml) to the beads. A 2.4 / 2.7 / 2.8 F SeQure microcatheter has been used. We observed a 1 month tumour/whole liver overall response as CR in 69.9/58.9%, PR in 14.3/12.6%, SD in 11.1/12.6%, PD in 4.7/15.9%. No non-target embolisation (NTE) events, or post-procedural complications occurred. In two patients, the CECT at approximately one month after the procedure showed a partial response with residual disease in the surroundings of the treated area and required additional treatment.
**Conclusion:** The combined usage of DEB-TACE with LUMI and SeQure microcatheter may prevent non-target embolisation events, potentially increasing the efficacy of the treatment and reducing the peri-procedural complications.
**Limitations:** Monocentric, small population, different operators.
**Ethics committee approval:** No information provided by the submitter.
**Funding for this study:** No funding was received for this study.
**Author Disclosures:**
Salvatore Alessio Angileri: Nothing to disclose
Anna Maria Ierardi: Nothing to disclose
Velio Ascenti: Nothing to disclose
Pierpaolo Biondetti: Nothing to disclose
Serena Carriero: Nothing to disclose
Gianpaolo Carrafiello: Nothing to disclose
Giuseppe Pellegrino: Nothing to disclose
Carolina Lanza: Nothing to disclose

### RPS 2009-9

**Difference between c-TACE and degradable starch microspheres (DSM-TACE) in the treatment of intermediate HCC: experience from a tertiary centre**

*C. Lanza*, S. A. Angileri, S. Carriero, P. Biondetti, G. Pellegrino, V. Ascenti, A. M. Ierardi, G. Carrafiello; Milan/IT

**Purpose:** To evaluate the difference between c-TACE and degradable starch microspheres (DSM-TACE) in the treatment of intermediate HCC.
**Methods or Backround:** We retrospectively analysed all patients with intermediate HCC treated with TACE between January 2021 and October 2022. We divided the patients into 2 groups: group 1 treated with c-TACE and group 2 treated with degradable starch microspheres (DSM-TACE). Primary outcome measures evaluated: number of treated lesions, dose of lipiodol, dose of Embocept, dose of chemotherapy, time of procedures and costs. The efficacy assessment was based on response rate in target lesion(s), according to mRECIST criteria. The progression-free survival (PFS), as well as overall survival (OS), were also calculated. Safety assessment was based on the recording of the adverse events, classified according to CIRSE classification.
**Results or Findings:** 40 patients were retrospectively analysed. We divided the patients into 2 groups: group 1 treated with c-TACE (n=20) and group 2 treated with DSM-TACE (n=20). 17 of the total (42,5%) did not receive any previous treatment before TACE and 23 (57,5%) of the total received previous treatment. No statistical significant differences in terms of efficacy, and in terms of progression-free survival (PFS), as well as overall survival (OS) were observed between the two groups. (p=0.05). Group 1 was associated with a major rate of peri-procedural complications, according to CIRSE classification (p<0.05). Group 2, due to the multiple procedures needed, was associated with higher costs and major risks associated with the procedure. (p<0.05).
**Conclusion:** The clinical efficacy observed between the two groups was equivalent. Group 1 was associated with a major rate of peri-procedural complications, and group 2 was associated with higher procedural costs and major risks for multiple procedures needed.

**Limitations:** Retrospective study. Small cohort.
**Ethics committee approval:** No information provided by the submitter.
**Funding for this study:** No funding was received for this study.
**Author Disclosures:**
Salvatore Alessio Angileri: Nothing to disclose
Anna Maria Ierardi: Nothing to disclose
Velio Ascenti: Nothing to disclose
Pierpaolo Biondetti: Nothing to disclose
Serena Carriero: Nothing to disclose
Gianpaolo Carrafiello: Nothing to disclose
Giuseppe Pellegrino: Nothing to disclose
Carolina Lanza: Nothing to disclose

### RPS 2009-10

**Transpulmonary chemoembolisation (TPCE) of unresectable lung metastases: tumour response and survival time**

*T. J. Vogl*, L. Hammann, H. Adwan, T. Gruber-Rouh; Frankfurt a. Main/DE
*(t.vogl@em.uni-frankfurt.de)*

**Purpose:** To retrospectively evaluate tumour response and patient survival after palliative treatment of pulmonary metastases using transpulmonary chemoembolisation (TPCE) in an interventional oncological setting.
**Methods or Backround:** 223 patients (138 women and 85 men; mean: 59.3 ± 11.9 years) with unresectable lung metastases and not responding to systemic chemotherapy received repetitive TPCE (mean number of sessions 4.7 ± 3.7) between January 1990 and May 2021. Patients had predominant lung metastases often with bilateral lung involvement. Origin of the metastases was either colorectal carcinoma (n=139) or breast cancer (n=84). Tumour-supplying vessels were catheterised selectively in order to locally apply the chemotherapeutic agents combined with lipiodol and microspheres. The response was assessed according to the revised RECIST criteria.
**Results or Findings:** Due to incomplete data regarding the RECIST criteria only 183 cases were evaluated. Partial response was achieved in 4.9% (n=9), stable disease in 62.3% (n=114), and progressive disease in 32.8% (n=60) of the patients. The mean and median overall survival time were 23.5 and 14 months, respectively. Only a low rate of side effects and no major complications were documented.
**Conclusion:** TPCE is a well-tolerated procedure for palliative treatment of unresectable lung metastases. It has the potential to improve local tumour control and to prolong survival.
**Limitations:** Retrospective study design. Incomplete data set (only 183 of 223 patients could be evaluated according to the RECIST criteria).
**Ethics committee approval:** No information provided by the submitter.
**Funding for this study:** No funding was received for this study.
**Author Disclosures:**
Lars Hammann: Nothing to disclose
Thomas J. Vogl: Nothing to disclose
Tatjana Gruber-Rouh: Nothing to disclose
Hamzah Adwan: Nothing to disclose

### RPS 2009-11

**What have we learned in more than 10 years of ablation of osteoid osteoma using Magnetic Resonance-guided Focused Ultrasound Surgery (MRgFUS)?**

*F. Arrigoni*, C. Acanfora, J. Daffinà, L. Zugaro, A. Barile, C. Masciocchi; L'Aquila/IT

**Purpose:** We reviewed clinical and radiological results of all the ablative procedures of osteoid osteoma performed with Magnetic Resonance-guided Focused Ultrasound Surgery (MRgFUS) in our institution over the last 10 years, inquiring on the reasons of both failures and successful treatments.
**Methods or Backround:** We retrospectively evaluated the outcomes of MRgFUS treatments of osteoid osteoma performed from 2012 to 2022. For all patients, we collected the clinical and radiological follow-up studies. In case of recurrent lesions, we investigated for the reason of failure, also evaluating the results of the second RFA performed in all cases in our institution.
**Results or Findings:** Since 2012, we have treated 63 osteoid osteomas, but during and after the recent pandemic period, we have recorded a decrease in the number of treatments. For all patients, clinical and radiological follow-up studies were available. We recorded 5 cases of relapse of the lesion due to wrong selection criteria and technical limitation. No complications were recorded.
**Conclusion:** This is probably the largest series of osteoid osteomas treated with MRgFUS. Our results confirm the validity of MRgFUS as treatment of choice of osteoid osteoma located on the bone surface. This technique is minimally invasive, safe, and effective. Specific expertise is, however, always required.
**Limitations:** Monocentric study. No control group.
**Ethics committee approval:** No information provided by the submitter.
**Funding for this study:** No funding was received for this study.
**Author Disclosures:**
Francesco Arrigoni: Nothing to disclose
Julia Daffinà: Nothing to disclose
Carlo Masciocchi: Nothing to disclose
Antonio Barile: Nothing to disclose
Luigi Zugaro: Nothing to disclose
Chiara Acanfora: Nothing to disclose

### RPS 2009-12

**Combination therapy of lipiodol based transarterial embolisation and microwave ablation versus microwave ablation as monotherapy for early-stage hepatocellular carcinoma: a comparative study**

*H. Adwan*, M. Adwan, T. Vogl; Frankfurt a. Main/DE

**Purpose:** To compare the efficacy and safety of the combination therapy of lipiodol based transarterial embolisation (TAE) and microwave ablation (MWA) with MWA as monotherapy in treating early hepatocellular carcinoma (HCC).
**Methods or Backround:** This retrospective study enrolled 49 patients in the TAE-MWA group (12W and 37M; mean age: 63±9.5 years) with 55 tumours, and 63 patients in the MWA group (18W and 45M; mean age: 66±10.4 years) with 67 tumours. Patients' cases were evaluated according to pre-ablation diameter of tumours, complications, complete ablation, local recurrence (LR), intrahepatic distant recurrence (IDR), overall survival (OS), and progression-free survival (PFS).
**Results or Findings:** The mean tumour diameter was 1.9 cm in the TAE-MWA group, and 2 cm in the MWA group (p-value:0.4). Initial complete ablation was achieved in all tumours in the TAE-MWA group, and in 97% of the tumours in the MWA group (p-value:0.5). There were no treatment-related deaths or major complications in the whole patient cohort reported. The rates of LR were 5.5% and 7.5% in the MWA-TAE group and MWA-group, respectively, (p-value:0.73). The rates of IDR were 42.9% and 52.4% in the MWA-TAE group, and MWA-group, respectively, (p-value:0.42). The 1-, 2-, and 3-year OS rates were 97.7%, 85.1%, and 78.8% in the TAE-MWA group, and 91.9%, 71.4% and 59.8% in the MWA group, respectively, (p-value:0.004). The 1-, and 2-year PFS rates were 55.1%, and 44.6% in the TAE-MWA group, and 49.2%, and 29.6% in the MWA group, respectively, (p-value:0.18).
**Conclusion:** The combination therapy of MWA and TAE was superior to the monotherapy with MWA in the treatment of early HCC. Patients in the TAE-MWA group had significantly longer OS than the patients in the MWA group.
**Limitations:** Retrospective study.
**Ethics committee approval:** Ethical approval for this study was obtained.
**Funding for this study:** No funding was received for this study.
**Author Disclosures:**
Thomas Vogl: Nothing to disclose
Hamzah Adwan: Nothing to disclose
Moath Adwan: Nothing to disclose

**14:00-15:30** **Research Stage 4**

### Research Presentation Session: Cardiac

### RPS 2003

### Cardiovascular MRI: functional assessment

**Moderator**
M. Singhal; Chandigarh/IN

### RPS 2003-2

**Fetal cardiovascular MRI using Doppler ultrasound gating: Comparison with echocardiography for evaluation of cardiovascular anomalies**

*M. Dargahpour Barough*, B. Hergert, M. Tavares de Sousa, S. Zhang, J. Herrmann, J. M. Seliger, G. Adam, P. Bannas, B. Schönnagel; Hamburg/DE

**Purpose:** To evaluate the diagnostic performance of fetal cardiovascular MRI for assessment of fetal cardiovascular anomalies in comparison to echocardiography.
**Methods or Backround:** This prospective study included same-day fetal cardiovascular MRI and echocardiography of 30 fetuses (28-38 weeks gestational age). A fetal cardiovascular MRI was performed using Doppler ultrasound for cardiac gating at 3T. A multi-slice cine steady-state free-precession sequence covering the fetal heart and thoracic vessels was acquired in transversal orientation. MRI image quality was assessed in consensus by two radiologists using a 4-point scale (from 1=low to 4=high quality). Fetal cardiovascular anatomy and diameters were evaluated by MRI in a four-chamber view, left ventricular outflow tract (LVOT) view, right ventricular outflow tract (RVOT) view, and three-vessel view (3V) and compared to

Saturday prenatal echocardiography. Postnatal echocardiography served as the standard of reference.
**Results or Findings:** MRI image quality was good (mean score: 3.0; score 4 in 24%, score 3 in 55%, score 2 in 20%). One fetus was excluded due to inadequate MRI image quality caused by severe motion artefacts. Compared to echocardiography, MRI detected 15/17 (88%) of cardiovascular anomalies (Tetralogy of Fallot, AVSD, Ebstein`s anomaly, Coarctation, aortic valve stenosis, hypoplastic aortic arch). Quantitative assessment of cardiac and vessel diameters revealed no statistically significant differences between echocardiography and fetal cardiovascular MRI (all p<0.05).
**Conclusion:** Fetal cardiovascular MRI using Doppler ultrasound gating allowed reliable diagnosis of cardiovascular anomalies and may be a useful additional diagnostic tool in the prenatal assessment of fetal cardiovascular anomalies.
**Limitations:** Reduced quality of some CINE slices due to fetal motion and maternal respiratory artefacts. A limited number of study subjects.
**Ethics committee approval:** No information provided by the submitter.
**Funding for this study:** Funding was received from German research foundation (SCHO1564/2-1 and BA5893/6-1)
**Author Disclosures:**
Jan Moritz Seliger: Nothing to disclose
Manuela Tavares de Sousa: Nothing to disclose
Peter Bannas: Nothing to disclose
Björn Schönnagel: Nothing to disclose
Shuo Zhang: Nothing to disclose
Gerhard Adam: Nothing to disclose
Bettina Hergert: Nothing to disclose
Maryam Dargahpour Barough: Nothing to disclose
Jochen Herrmann: Nothing to disclose

### RPS 2003-3

**Atrial dimensions reference values on healthy subjects**
A. Lupi, R. Angelone, *M. Milone*, S. Zinato, F. Crimì, F. Vernuccio, G. Cabrelle, E. Quaia, A. Pepe; Padova/IT
*(maura.milone@studenti.unipd.it)*

**Purpose:** Steady-state free precession (SSFP) cine sequence of cardiovascular magnetic resonance (CMR) is the gold standard for the evaluation of ventricular size and function. It provides excellent blood-endocardium and epicardium-fat contrast and a good temporal resolution without diminishing image quality. Despite the wide use of CMR in clinical practice, there are currently few studies exploring the normal atrial ranges, specifically those of the right atrium (RA). Moreover, to our knowledge, there are no studies on the RA dimensions obtained with the monoplane method on the 4-chamber view. Normalized values are primarily influenced by gender and body surface area (BSA). Thus the aim of this study is to provide reference values of RA dimensions.
**Methods or Backround:** Fifty healthy volunteers (M:F=1:1, five subjects per gender and age decile, aged 20-70 years) underwent CMR. Atrial areas, absolute and indexed to BSA, and volumes for males and females were obtained using cvi42 software and categorized by age group. Data are reported as mean +/- sd. Differences among gender and age groups were evaluated.
**Results or Findings:** The study population was composed of 25 males and 25 females (mean age 44±14 and 43±14, respectively). RA surface was significantly higher in males (p<0.05), and the left atrial surface did not show significant differences among genders. When atrial areas were normalized to BSA, no gender differences were observed (overall mean 12 ± 2 cm2/m2 for both atria). Both right and left absolute atrial volumes were significantly higher in males, too (p<0.05). Neither atrial volume nor areas showed a significant correlation with age.
**Conclusion:** Atrial dimensions show significant variations among gender but do not vary after adjustment for BSA. References ranges are useful for clinical and research purposes for CMR interpretation and for avoiding misdiagnosis.
**Limitations:** No limitations were identified.
**Ethics committee approval:** No information provided by the submitter.
**Funding for this study:** Not applicable.
**Author Disclosures:**
Federica Vernuccio: Nothing to disclose
Stefania Zinato: Nothing to disclose
Alessia Pepe: Nothing to disclose
Amalia Lupi: Nothing to disclose
Giulio Cabrelle: Nothing to disclose
Raimondo Angelone: Nothing to disclose
Maura Milone: Nothing to disclose
Filippo Crimì: Nothing to disclose
Emilio Quaia: Nothing to disclose

### RPS 2003-4

**Heart failure with preserved ejection fraction in post-myocardial infarction patients: a myocardial MR tissue tracking study**
*H. Li*, Y. Zheng, X. Peng, H. Liu, Y. Li, Z. x. Tian, S. Jin, H. Huo, T. Liu; Shenyang/CN

**Purpose:** This study aimed to explore the value of cardiac magnetic resonance tissue tracking (CMR-TT) technology in evaluating heart failure with preserved ejection fraction (HFpEF) in patients with chronic myocardial infarction (CMI).
**Methods or Backround:** Between June 2016 and March 2022, 92 CMI patients (HFpEF-CMI,n=54; non-HF-CMI,n=38) and 40 healthy controls were enrolled in this retrospective study. Quantitative measurements of myocardial damage, such as biventricular myocardial strain parameters derived by CMR-TT and infarct size and transmurality by late gadolinium enhancement, were assessed. One-way analysis of variance, independent samples t-test, and rank sum test were used to compare myocardial impairment among groups. Pearson or Spearman correlation coefficient was used to measure correlations between left ventricular (LV) strains and clinical and functional parameters. Logistic regression analysis and receiver operating characteristic curve were performed to identify the best parameter for diagnosing HFpEF-CMI.
**Results or Findings:** HFpEF-CMI patients demonstrated significantly impaired LV strains and strain rates in all three directions (radial, circumferential and longitudinal) compared to non-HF-CMI patients and healthy controls (P<0.001 for all), whereas only global longitudinal strain was significantly impaired in HFpEF-CMI patients vs. controls for right ventricular strain parameters (P<0.001). LV strains showed moderate correlation with N-terminal pro-brain natriuretic peptide (radial, circumferential and longitudinal strain, R=-0.401, R=0.408, R=0.407, respectively, P<0.001 for all). LV strains in the three directions (radial, circumferential and longitudinal) (area under ROC curve [AUC]=0.707, 0.708, 0.731, respectively, P<0.01 for all) were discriminators for HFpEF-CMI and non-HF-CMI.
**Conclusion:** CMR-TT provides clinicians with useful additional imaging parameters to facilitate the assessment of CMI patients with HFpEF. LV strain parameters can detect early cardiac insufficiency in patients with HFpEF-CMI and have potential value for discriminating between HFpEF and non-HF patients post-CMI.
**Limitations:** No limitations were identified.
**Ethics committee approval:** No information provided by the submitter.
**Funding for this study:** Not applicable.
**Author Disclosures:**
Yue Zheng: Nothing to disclose
Hui Liu: Nothing to disclose
Han Li: Nothing to disclose
Yue Li: Nothing to disclose
Xin Peng: Nothing to disclose
Huaibi Huo: Nothing to disclose
Zhao xin Tian: Nothing to disclose
Shiqi Jin: Nothing to disclose
Ting Liu: Nothing to disclose

### RPS 2003-5

**MR 4D-flow derived left atrial acceleration factor for characterization of higher-grade diastolic dysfunction**
*C. Reiter*, U. Reiter, C. Kräuter, A. Schmidt, D. Scherr, M. Fuchsjäger, G. Reiter; Graz/AT
*(Clemens.Reiter@medunigraz.at)*

**Purpose:** Increased left atrial filling pressures are a hallmark feature of diastolic dysfunction of grade 2 and larger. Recently the left atrial acceleration factor (α) was introduced as an imaging-derived MR 4D-Flow biomarker for the detection of elevated left atrial pressure in a population of patients with pulmonary hypertension. The aim of this study was to evaluate α for the characterization of higher-grade diastolic dysfunction.
**Methods or Backround:** Thirty-four subjects with different grades of diastolic dysfunction evaluated by echocardiography (13/4/11/6 for grade 0/1/2/3, respectively) underwent cardiac MR at 3T, including time-resolved whole-heart 3-directional phase-contrast-imaging (4D-Flow). Left atrial peak systolic outflow-velocity (Ve) was evaluated from the mitral annulus, and peak systolic (Vs) and diastolic (Vd) inflow-velocities were evaluated at the orifice of the pulmonary veins from multiplanar reformatted 4D-Flow data with prototype software. α was calculated by α=Ve/[(Vs+Vd)/2]. Relationships between α and grades of diastolic dysfunction were analyzed by t-test, correlation- and ROC analysis.
**Results or Findings:** Mean α were 1.13±0.10, 1.26±0.21, 1.57±0.10, 3.07±0.97 for grades 0/1/2/3, respectively. α showed a strong correlation to grade of diastolic-dysfunction (r=0.89, p<0.001) and a significant difference was observed comparing patients with higher-grade diastolic dysfunction (grade 2 and 3) compared to grade 0 and 1 (1.16±0.14 vs. 2.1±1.63, p=0.003). Area-under-the-curve for detection of higher-grade diastolic dysfunction was 0.986 resulting in a sensitivity of 1.00 (95%-confidence-interval: 0.80-1.00) and a specificity of 0.94 (95%-confidence-interval: 0.71-1.00) with a cut-off value of 1.38.
**Conclusion:** The single parameter left atrial acceleration factor allows differentiation of low- vs. high-grade diastolic dysfunction.
**Limitations:** Small sample size.
**Ethics committee approval:** No information provided by the submitter.
**Funding for this study:** Funding was received from OeNB Anniversary Fund 17934.
**Author Disclosures:**
Ursula Reiter: Nothing to disclose
Gert Reiter: Employee: Siemens Healthcare Diagnostics GmbH
Michael Fuchsjäger: Nothing to disclose
Daniel Scherr: Nothing to disclose
Clemens Reiter: Nothing to disclose
Albrecht Schmidt: Nothing to disclose
Corina Kräuter: Nothing to disclose

### RPS 2003-6

**CMR global longitudinal strain predicts adverse outcomes in non-ischaemic dilated cardiomyopathy without myocardial scar**
*X. Xiang*, X. Chen, K. Zhao, Y. Song, S. Yu, S. Zhao; Beijing/CN
*(xiangxiaorui@live.com)*

**Purpose:** Despite late gadolinium enhancement (LGE) have already proved a strong association with adverse outcomes in non-ischaemic dilated cardiomyopathy (NIDCM) patients, for those who with negative LGE, mortality remains a clinical issue. This study therefore aimed to investigate the prognostic impact of cardiac magnetic resonance (CMR) feature tracking derived strain in NIDCM patients without myocardial scar.
**Methods or Backround:** Consecutive patients with NIDCM who underwent CMR between 2012 and 2014 were retrospectively evaluated, and those with positive LGE were excluded. Ventricular systolic dysfunction was measured as LV and RV global longitudinal strain (GLS) by CMR feature tracking. All patients were followed up for the combined endpoints (CE) including all-cause mortality and heart transplantation.
**Results or Findings:** A total of 171 patients with NIDCM were followed up for a median of 5.9 years, and 26 patients reached CE. Both LV and RV GLS were significantly associated with endpoints. Using separate multivariate COX analysis models, LV-GLS offered incremental prognostic value for endpoints beyond NYHA class and NT-proBNP (HR, 1.377; P<0.001). RV-GLS also exhibited an independent prognostic value in addition to NYHA class, NT-proBNP, and anti-arrhythmic medication (HR, 1.142; P=0.002). Furthermore, among patients of LVEF less than 35%, patients with a LVGLS>-12.92% had a higher rate of CE (log-rank, P<0.001).
**Conclusion:** CMR feature tracking derived biventricular GLS associated with adverse outcomes in NIDCM patients without myocardial scar.
**Limitations:** First, this was a single-centre study. Second, there was a degree of selection bias related to being able to undergo a CMR examination. Third, there was no genetic characterisation of study patients. Despite the limitations, this is the first study to assess the association between GLS and outcomes in NIDCM without scar.
**Ethics committee approval:** Ethical approval was obtained by the hospital's Institutional Review Board (IRB).
**Funding for this study:** This study was supported by Key Project of National Natural Science Foundation of China (No.81930044).
**Author Disclosures:**
Shiqin Yu: Nothing to disclose
Xiuyu Chen: Nothing to disclose
Yanyan Song: Nothing to disclose
Xiaorui Xiang: Nothing to disclose
Shihua Zhao: Nothing to disclose
Kankan Zhao: Nothing to disclose

### RPS 2003-7

**Cardiac magnetic resonance in patients with acute myocarditis: is there a role for myocardial strain?**
*S. Salto*[1], D. Tore[1], R. Faletti[1], M. Gatti[1], A. Biondo[1], E. Bozzo[1], I. Landolfi[1], A. Palmisano[2], P. Fonio[1]; [1]Turin/IT, [2]Milan/IT

**Purpose:** To evaluate the diagnostic role of myocardial strain measured by cardiac magnetic resonance imaging (CMR) in patients with acute myocarditis.
**Methods or Backround:** Multicenter prospective observational study on consecutive patients. Seventy-seven patients with CMR diagnosis of myocarditis according to the 2018 Lake Louise Criteria were enrolled. A group of 24 healthy volunteers without prior history of cardiovascular disease was used as a control group. 2D and 3D strain parameters (GRS, GCS, GLS, PSSR radial, circumferential and longitudinal, PDSR radial, circumferential and longitudinal) were calculated with feature tracking technique using commercial software (CVI42 5.13, Circle Cardiovasc. Img, Calgary, Canada). Patients' strain parameters (considered as a whole group and stratified according to ejection fraction <50% or >50%) were compared with controls. Mann-Whitney test, univariate (uBLR) and multivariate (mBLR) binary logistic regression analysis were used for statistical analysis and ROC curves were calculated.
**Results or Findings:** From the comparison between controls and patients (considered as a whole group and stratified according to EF), statistically significant differences emerged for all 2D strain parameters with p values always <0.0001. Multivariate analysis showed that with at least four altered 2D strain parameters, it is possible to discriminate between controls and patients with a sensitivity of 0.86, specificity of 1, PPV of 1, NPV of 0.67 and AUC of 0.97. A sensitivity of 0.78, specificity of 0.73, PPV of 0.91, NPV of 0.48 and AUC of 0.82 is achieved with at least two altered 3D strain parameters.
**Conclusion:** Myocardial strain evaluated with CMR showed an independent diagnostic value compared to the traditional Lake Louise criteria, and it may represent a new imaging biomarker for the diagnosis of acute myocarditis.
**Limitations:** Limited patient population.
**Ethics committee approval:** No information provided by the submitter.
**Funding for this study:** No funding was received for this work.
**Author Disclosures:**
Elena Bozzo: Nothing to disclose
Sara Salto: Nothing to disclose
Riccardo Faletti: Nothing to disclose
Anna Palmisano: Nothing to disclose
Marco Gatti: Nothing to disclose
Paolo Fonio: Nothing to disclose
Ilenia Landolfi: Nothing to disclose
Davide Tore: Nothing to disclose
Andrea Biondo: Nothing to disclose

### RPS 2003-8

**Follow-up with the cardiac magnetic resonance of patients with acute myocarditis: time evolution of strain parameters**
*A. Santonocito*[1], D. Tore[1], R. Faletti[1], M. Gatti[1], E. Bozzo[1], F. Menchini[1], F. Ullo[1], A. Esposito[2], P. Fonio[1]; [1]Turin/IT, [2]Milan/IT
*(santonocitoambra@gmail.com)*

**Purpose:** To evaluate the evolution of myocardial strain parameters measured by cardiac magnetic resonance imaging (CMR) in patients with acute myocarditis at one month and one-year follow-up.
**Methods or Backround:** Multicenter prospective observational study on 77 consecutive patients with CMR diagnosis of acute myocarditis according to 2018 Lake Louise Criteria. All patients underwent 3 CMR exams (within three weeks from the acute event, at one month and one year). A group of 24 healthy volunteers without prior history of cardiovascular disease was used as a control group. 2D and 3D strain parameters (GRS, GCS, GLS, PSSR radial, circumferential and longitudinal, PDSR radial, circumferential and longitudinal) were calculated with feature tracking technique using commercial software (CVI42 5.13, Circle Cardiovasc. Img, Calgary, Canada). Statistical analysis was performed with the Mann-Whitney test.
**Results or Findings:** In the comparison between acute and 1-month exams, a statistically significant improvement emerged for GRS 2D, PSSR RAD 2D, PSSR CIRC 2D, PSSR LONG 2D, PDSR RAD 2D, GCS 3D, and GLS 3D. In the comparison between acute and 1-year exams, a statistically significant improvement emerged for GRS 2D, GCS 2D, GCS, and PDSR LONG 3D. In the comparison between 1-year exams and healthy controls, statistically, significant differences remain for all 2D strain parameters with p values <0.0001, except for GLS (p>0.05).
**Conclusion:** Many cardiac strain parameters rapidly improve between the acute event and the 1-month follow-up, probably due to the resolution of oedema. Compared to healthy controls, many 2D strain parameters are still altered at 1-year follow-up. It is yet to be determined the clinical relevance of persisting strain alterations at long-term follow-up in patients with previous myocarditis.
**Limitations:** Limited patient population.
**Ethics committee approval:** The study was approved by the Ethics Committee.
**Funding for this study:** No funding was received for this work.
**Author Disclosures:**
Elena Bozzo: Nothing to disclose
Marco Gatti: Nothing to disclose
Francesca Menchini: Nothing to disclose
Antonio Esposito: Nothing to disclose
Federica Ullo: Nothing to disclose
Riccardo Faletti: Nothing to disclose
Ambra Santonocito: Nothing to disclose
Davide Tore: Nothing to disclose
Paolo Fonio: Nothing to disclose

### RPS 2003-9

**Myocardial strain measurements derived from MR feature-tracking: influence of sex, age, field strength and vendors on reference values**

*W. Yang*, J. Xu, M. Lu; Beijing/CN

**Purpose:** CMR-FT is a novel technique for assessing myocardial deformation and dysfunction. We aimed to characterise the normal reference values of myocardial strain derived from cardiovascular magnetic resonance feature tracking (CMR-FT) in four cardiac chambers and identify sources of variation affecting FT strains using a systematic review and meta-analysis of the CMR-FT literature.
**Methods or Backround:** PubMed, Embase, and Scopus were searched for myocardial strains of all four chambers measured by CMR-FT in healthy adults. The pooled means of all strain parameters were generated using random-effects hedges model. Subgroup analyses and meta-regressions were performed to identify the sources of variations.
**Results or Findings:** 33 studies with a total of 2,954 healthy subjects were included in the meta-analysis. The pooled means of left ventricular global longitudinal strain (LV-GLS), LV global radial strain and LV global circumferential strain were -18.8% (95%CI:-20.0%, -17.6%), 46.0% (95%CI:39.2%, 52.9%), and -22.2% (95%CI:-23.6%, -20.7%). The pooled means of left atrial global longitudinal strain (LA-GLS, corresponding to total strain, passive strain and active strain) were 35.0% (95%CI:28.8%, 41.2%), 21.3% (95%CI:16.5%, 26.2%) and 14.3% (95%CI:10.8%, 17.8%). The pooled means of right ventricular global longitudinal strain (RV-GLS) and right atrial global longitudinal total strain were -25.4% (95%CI:-27.2%, -23.5%) and -36.2% (95%CI:-53.9%, -18.6%). Meta-regression identified field strength (P<0.01, I2=98.9%) and FT vendor (P<0.01, I2=99.1%) as significant confounders explaining heterogeneity of LV-GLS. The variations of LA-GLS active were associated with regional distribution (P<0.01, I2=98.3%) and FT vendor (P=0.01, I2=98.5%). Also differences in FT vendor were attributed to variations of RV-GLS (P<0.01, I2=94.7%).
**Conclusion:** The normal reference values of CMR-FT strain parameters in all four cardiac chambers in healthy subjects were demonstrated. Differences in FT vendor contributed to the heterogeneity of LV-GLS, LA-GLSactive and RV-GLS, while sex, age and MR vendor showed no effect on the normal values of CMR-FT strain measurements.
**Limitations:** Not applicable.
**Ethics committee approval:** No information provided by the submitter.
**Funding for this study:** Not applicable.
**Author Disclosures:**
Minjie Lu: Nothing to disclose
Jing Xu: Nothing to disclose
Wenjing Yang: Nothing to disclose

### RPS 2003-10

**Correlations between genetic and cardiac MR features in patients with non-compacted myocardium**

E. Mershina, R. Myasnikov, *D. Filatova*, A. Meshkov, A. Kiseleva, V. Sinitsyn; Moscow/RU

**Purpose:** Non-compacted myocardium (NCM) is a form of cardiomyopathy with prominent heterogeneity of genetic features: mutations in genes encoding components of sarcomeric proteins (dominant), nucleus, cytoskeleton, mitochondria, ion channels and other structures can be identified. The purpose of this study was to compare the morphological and functional characteristics of NCM defined by cardiac MRI (CMR) with genetic biomarkers.
**Methods or Backround:** Forty-two patients were chosen from a registry of NCM patients who underwent genetic testing (250 pts). The selection was based on identified mutations in genes: MYH7 (20 pts, age 33.6±12.0 yrs), MYbp3c (11 pts, age 42.2± 9.4 yrs), TTN (11 pts, age 38.4±15.9 yrs). CMR was performed with a 1.5T scanner.
**Results or Findings:** Three phenotypes were distinguished: patients with and without NCM signs and patients with NCM-like syndrome. There was a statistically significant correlation between genetic factors and phenotype (p=0.034): the greatest number of patients with NCM was found in the MYH7 group (60%), there were no patients with normal phenotype; the minimum number was in the MYbp3c group (36%), there were four patients with normal phenotype. Indexed RV EDV/RV ESV were maximal in the MYH7 group (85.0/53.4 ml/m2) and minimal in the TTN group (65.7/36.8 ml/m2), p < 0.05. The ratio of papillary muscle mass to total myocardial mass was maximal in the MYH7 group (23.75%) and minimal in the TTN group (17.09%), p <0.05. Myocardial fibrosis was most frequently identified in patients with TTN mutations (36%). In patients with MYbp3C mutations, it was identified in 27% of cases, and in patients with MYH7 mutations - in 5% of cases.
**Conclusion:** Mutations in MYH7 are more likely to contribute to RV dysfunction and the development of NCM phenotype. These mutations are associated with impaired LV function. Myocardial fibrosis in patients with mutations in MYH7 is less frequent.
**Limitations:** No limitations were identified.
**Ethics committee approval:** No information provided by the submitter.
**Funding for this study:** Not applicable.
**Author Disclosures:**
Valentin Sinitsyn: Nothing to disclose
Anna Kiseleva: Nothing to disclose
Aleksei Meshkov: Nothing to disclose
Elena Mershina: Nothing to disclose
Daria Filatova: Nothing to disclose
Roman Myasnikov: Nothing to disclose

### RPS 2003-11

**Role of cardiac magnetic resonance derived right ventricular strain in early detection of arrhythmogenic right ventricular cardiomyopathy**

*J. Radhakrishnan*, A. Ayyappan, J. Valakada, V. Subbaih A; Trivandrum/IN

**Purpose:** To study the role of right ventricular strain analysis in early detection of the arrhythmogenic right ventricular cardiomyopathy in patients with non-sarcoid RVOT arrhythmias.
**Methods or Backround:** Cardiac MRI was performed on 42 patients with RVOT arrhythmias. Twenty-seven patients fulfilled the 2020 International diagnostic criteria for arrhythmogenic cardiomyopathy. Of the 15 patients who didn't fulfil the criteria for the diagnosis, 5 of the patients, on follow-up, developed abnormalities suggestive of ARVC – MRI false negative cases. Right ventricular strain analysis was carried out in all 42 cases on b-SSFP imaging sequences in the 4-chamber and short axis planes using cvi42, Circle Cardiovascular Imaging software. RV strain analysis of 30 normal subjects as a control group was conducted as well.
**Results or Findings:** There was a significant difference in the -mean radial strain of control group (29.57+/-2.7%) versus ARVC (14.38+/-1.7%) (p<0.01) -mean circumferential strain of control group (-15.42+/-1.19%) versus ARVC (-12.16+/-1.3%)(p<0.01) -mean longitudinal strain of control group (-26.43+/-2.29%) versus ARVC (-13.88+/-1.08%)(p,0.01). Based on the cut-off strain values derived from the control group, strain parameters were able to identify the MRI-negative ARVC cases with high sensitivity (100%) and specificity of 68.2%,
**Conclusion:** Conventional MRI assessment of RV function and volumes, in addition to the late gadolinium enhancement and regional dyskinesias, had a sensitivity of 84.37% in diagnosing ARVC. The addition of strain imaging parameters increased the sensitivity of MRI to 93%. There was an increase in positive predictive value by 11% with the addition of RV strain parameters to conventional imaging.
**Limitations:** Large-volume studies of normal populations are required to define the strain values of a normal heart. The RV endocardial contouring has its limitations owing to the inherently low thickness of the RV wall.
**Ethics committee approval:** No information provided by the submitter.
**Funding for this study:** No funding was received for this study.
**Author Disclosures:**
Jayakrishnan Radhakrishnan: Nothing to disclose
Jineesh Valakada: Nothing to disclose
Anoop Ayyappan: Nothing to disclose
Venkata Subbaih A: Nothing to disclose

### RPS 2003-12

**A novel and simple cardiac MRI score (PE²RT) predicts outcome in takotsubo syndrome**

*A. Isaak*, J. Bratz, D. Kravchenko, N. Mesropyan, L. Bischoff, U. I. Attenberger, D. Kütting, S. Zimmer, J. A. Luetkens; Bonn/DE

**Purpose:** To find simple cardiac MRI features that are associated with major adverse cardiac events (MACE) in takotsubo syndrome (TTS).
**Methods or Backround:** In this retrospective study (2007-2021), patients who underwent cardiac MRI and had a diagnosis of TTS were identified. Presence of TTS-associated complications was assessed (pericardial effusion [>5mm], pleural effusion [>20mm], right ventricular [RV] involvement, and ventricular thrombus). PE²RT score was calculated by the sum of these complications (one point for each complication; range 0-4). The relationship between PE²RT score and MACE (composite of cardiovascular death, hospitalisation with new-onset of acute or heart failure symptomatic, pacemaker implantation) on clinical follow-up was examined using univariable Cox regression analysis and Kaplan-Meier analysis.
**Results or Findings:** 79 patients with TTS (68±14 years; 71 women) were included. Median duration of follow-up was 18.8 months (IQR, 0.5-56 months). 15 patients (19%) developed MACE on follow-up. Most frequently observed complications were pericardial effusion (34/79 patients, 43%) and pleural effusion (25/79 patients, 32%), followed by RV involvement (17/79 patients, 22%), and intraventricular thrombus (7/79 patients, 9%). The median PE²RT score was higher in patients with MACE (2 [IQR, 2-3] vs. 1 [IQR, 0-1]; P<0.001). Besides different clinical and imaging parameters such as physical trigger, diabetes mellitus, and left ventricular ejection fraction, an association was observed between PE²RT score and MACE on univariable Cox regression analysis (per PE²RT point [0-4]: HR, 2.44; 95%CI, 1.62-3.68; P<0.001). Patients with PE²RT score ≥2 had higher risk of MACE on univariable Cox regression analysis (HR, 15.68; 95%CI, 3.51-70.10; P<0.001) and Kaplan-Meier analysis (log rank P<0.001).

**Conclusion:** Complications detected by cardiac MRI and summarised as PE²RT score are frequently observed in TTS and are associated with long-term occurrence of MACE.
**Limitations:** Sample size.
**Ethics committee approval:** Informed consent was waived due to retrospective design.
**Funding for this study:** A.I.: BONFOR and DFG, EXC2151–390873048.
**Author Disclosures:**
Julian Alexander Luetkens: Nothing to disclose
Sebastian Zimmer: Nothing to disclose
Narine Mesropyan: Nothing to disclose
Alexander Isaak: Grant Recipient: University grant (BONFOR and DFG; EXC2151 – 390873048)
Leon Bischoff: Nothing to disclose
Dmitrij Kravchenko: Nothing to disclose
Ulrike I. Attenberger: Nothing to disclose
Daniel Kütting: Nothing to disclose
Johanna Bratz: Nothing to disclose

**16:00-17:30** **Research Stage 1**

### Research Presentation Session: Artificial Intelligence & Machine Learning & Imaging Informatics

### RPS 2105

### AI in stroke and neurovascular imaging

**Moderator**
S. Bisdas; London/UK

### RPS 2105-2

**Evaluation of automated CT workflow support in acute ischaemic stroke**
*S. Temmen*, F. J. A. Meijer, R. Becks, K. G. van Leeuwen; Nijmegen/NL
*(sandertemmen@hotmail.com)*

**Purpose:** The automation platform (AP) is an AI-based tool to support CT imaging in the diagnostic work-up of acute cerebral stroke. We evaluated the AP's processing time and diagnostic performance of the large vessel occlusion (LVO) detection algorithm on conventional CT angiography (CTA) as compared to radiologists.
**Methods or Backround:** The AP's processing time was timed in a retrospective cohort of 60 patients presenting with acute stroke symptoms who received the stroke CT protocol (non-contrast CT, CTA and perfusion CT (CTP)) and compared to prospective timing of 21 CTs read and processed by 13 radiologists. The diagnostic performance of LVO detection was evaluated on a retrospective cohort of 100 acute stroke patients and compared to the reading of these CT studies by five observers.
**Results or Findings:** The mean processing time for the AP's LVO tool was 60 seconds (37-77s). Reading time was 238s/353s/554s for neuroradiologists/non-neuroradiologists/residents. The mean processing time of the AP's CTP tool was 196 seconds (145-255s) and 243s/349s/335s for neuroradiologists/non-neuroradiologists/residents. Sensitivity of the LVO detection algorithm was 52% and specificity was 99%. Sensitivity of radiologists ranged between 78-91% and specificity ranged between 97-100%. Sensitivity of AP increased to 77% when M2 occlusions were excluded.
**Conclusion:** The AP showed shorter processing time of CTA and CTP, but the diagnostic performance of LVO detection was substantially lower as compared to radiologists.
**Limitations:** The AP was not implemented in clinical practice and its duration was retrospectively timed and compared to prospective timing of radiologists. To thoroughly evaluate the performance of the AP, it should be applied to a larger and more heterogeneous study population. Lastly, it is important for end-users to realise the AP was not intended for diagnosing vessel occlusions other than LVOs in the anterior circulation.
**Ethics committee approval:** The study has been approved by the medical-ethical committee of our hospital and informed consent was waived because of the retrospective collection of the study data.
**Funding for this study:** Not applicable.
**Author Disclosures:**
Sander Temmen: Nothing to disclose
Ruud Becks: Nothing to disclose
Frederick Jan Anton Meijer: Speaker: Speaker Bureau Canon Medical Systems
Kicky Gerhilde van Leeuwen: Nothing to disclose

### RPS 2105-3

**Automated classification of ischemic stroke territory on diffusion-weighted MRI: collaboration of human knowledge and deep learning**
*I. Ö. Koska*, Y. K. K. Çetinoğlu, M. E. E. Uluç, O. Dicle, M. F. Gelal, M. A. Selver; Izmir/TR
*(ozgurkoska@icloud.com)*

**Purpose:** Our aim was building a stroke territory classifier model by using DWI and DL. We hypothesised that, relevant transformations that inject human knowledge may help the model to make more accurate predictions and explored this hypothesis by extensive experiments.
**Methods or Backround:** Retrospective analysis of DWI images of 271 consecutive acute ischemic stroke patients examined between January 2017 to April 2020 was conducted. Pretrained MobileNetV2 and EfficientNetB0 architectures were used to classify territorial subtype as middle cerebral artery, posterior circulation or watershed areas. Various input combinations using edge maps, thresholded versions and hard attention versions were explored. Effect of augmenting the three channel inputs of pretrained models on classification performance was analysed. ROC analyses and confusion matrix derived performance metrics of the models were reported.
**Results or Findings:** MobileNetV2 based model achieved 93% accuracy with native images. Building the three channels of the input with native image, bounding box crop, and cross sign crop, improved the accuracy to 94.4%. ROC-AUC were 0.98 for MCA and watershed infarct predictions and 0.99 for posterior circulation prediction with this model.
**Conclusion:** Modified pretrained models may be augmented with transformations of native images to provide more accurate classification of ischaemic stroke territory in DWI. Collaboration of human knowledge and deep learning may lead to better results.
**Limitations:** We could not include ACA vascular territory due to limited number of samples. However we could cover 80-85% of all major arterial stroke territories under 3 classes. On the other hand our models analysed each slice as a separate instance, however patient level predictions is more relevant to integrate into clinical workflows. Another limitation of our study was the limited variance of image acquisition conditions since it was a single centre study.
**Ethics committee approval:** Dokuz Eylül University Ethical Board.
**Funding for this study:** No funding was received for this study.
**Author Disclosures:**
Mustafa Alper Selver: Nothing to disclose
Yusuf Kenan KENAN Çetinoğlu: Nothing to disclose
Oğuz Dicle: Nothing to disclose
Muhsin Engin Engin Uluç: Nothing to disclose
Ilker Özgür Koska: Nothing to disclose
Mustafa Fazil Gelal: Nothing to disclose

### RPS 2105-4

**Generalisability of academically successful algorithms in stroke lesion segmentation**
M. Bauer[1], *S. Ingala*[1], J. Johansen[1], A. Bjørnerud[2], I. Digernes[2], A. Pai[1], T. Nome[2], C. Nome[2]; [1]Copenhagen/DK, [2]Oslo/NO
*(silvia.ingala@gmail.com)*

**Purpose:** To evaluate the use of academically successful AI algorithms against CE marked algorithms in the segmentation of stroke lesions.
**Methods or Backround:** Both nnUNet and Apollo were trained on the same in-house dataset consisting of 1447 scans, comprising of 395 infarcts, 164 tumours, 130 haemorrhages, and 790 normal scans with no abnormalities detected. Three MRI sequences were used to train the algorithms: DWI, SWI and FLAIR. Dataset A consisted of scans conformed to DWI space and then resampled to isotropic resolution. Similarly, dataset B and C were conformed to SWI and FLAIR space respectively before resampling to isotropic resolution. Only one fold per dataset was trained for the Apollo algorithm (3D UNet with domain-specific augmentation), whereas 5 folds for each model (3D UNet, 2D UNet and 3D UNet Cascade) were trained for the nnUNet algorithm. Predictions from the three models for both the Apollo and the nnUNet algorithms were then ensembled in the native space to obtain the final prediction. Testing data: 30 cases with infarctions consisting of manually segmented DWI/SWI/FLAIR MRIs were obtained from the Oslo University Hospital and the OSCAR study. Each of the 30 subjects consisted of pre-thrombectomy and post thrombectomy MRI scans, both of which were manually segmented by expert neuroradiologists.
**Results or Findings:** For pre-thrombectomy cases, a mean dice of 0.72 ± 0.18 was obtained for the Apollo algorithm, and 0.64 ± 0.29 was obtained for the nnUNet algorithm. For post-thrombectomy cases, a mean dice of 0.85 ± 0.12 was obtained for the Apollo algorithm, and 0.85 ± 0.14 was obtained for the nnUNet algorithm.
**Conclusion:** Academic success of machine learning algorithms that are computationally expensive do not necessarily translate into real-world success.
**Limitations:** No limitations were identified.
**Ethics committee approval:** No information provided by the submitter.
**Funding for this study:** Funded by Cerebriu A/S.

**Author Disclosures:**
Atle Bjørnerud: Nothing to disclose
Cecilie Nome: Nothing to disclose
Akshay Pai: Founder: Cerebriu A/S
Jacob Johansen: Nothing to disclose
Ingrid Digernes: Nothing to disclose
Marko Bauer: Nothing to disclose
Silvia Ingala: Nothing to disclose
Terje Nome: Nothing to disclose

### RPS 2105-5

**iSPAN: improved prediction of outcomes post thrombectomy with machine learning**

*B. S. Kelly*, P. Mathur, J. Duignan, A. Lawlor, R. P. Killeen, J. Thornton; Dublin/IE
*(brendanskelly@me.com)*

**Purpose:** Acute ischaemic stroke is a major cause of morbidity and mortality. Thrombectomy has improved patient outcomes but has associated complications. Our purpose was to use machine learning (ML) to predict which patients are most likely to have a Day90-mRS (modified Rankin Score) of 0-2.
**Methods or Backround:** This retrospective study included all patients aged over 18 years with an anterior circulation stroke treated by the National Thrombectomy Service from 2010 to 2020. We trained and tested several machine learning models to predict which patients would most benefit from thrombectomy and compared these to existing clinical prediction scores. We identified the most important features used by the best preforming model. A novel score (iSPAN) was derived by adding an optimised weighting of the most important features.
**Results or Findings:** 812 patients were included, 397 female, average age 73. The best performing clinical score and ML model were SPAN and XGBoost with a sensitivity specificity and accuracy of (0.967, 0.290, 0.628) and (0.783, 0.693, 0.738) respectively. A significant difference in accuracy was found overall and XGBoost was more accurate than the next best predictor (SPAN) (p< 0.0018). The most important features were Age, mTICI and total number of passes. Addition of 11 points for mTICI <3 and 3 points for >3 passes to the SPAN (iSPAN) achieved the best training accuracy. iSPAN achieved a test sensitivity, specificity and accuracy of 0.816, 0.661 and 0.738 and was not significantly more accurate than XGBoost (p>0.5).
**Conclusion:** iSPAN achieves better prediction accuracy than existing clinical scores and is easier to calculate and interpret than XGBoost without significant performance reduction.
**Limitations:** Selection bias. Single Centre.
**Ethics committee approval:** Beaumont Hospital.
**Funding for this study:** ICAT Programme, Wellcome Trust and the Health Research Board (203930/B/16/Z).
**Author Disclosures:**
Aonghus Lawlor: Nothing to disclose
John Thornton: Nothing to disclose
Ronan P Killeen: Nothing to disclose
Brendan S Kelly: Nothing to disclose
Prateek Mathur: Nothing to disclose
John Duignan: Nothing to disclose

### RPS 2105-6

**Predicting acute stroke occurrence based on weather systems to improve clinical resource allocation: a machine learning approach**

N. Santhanam, F. Siegel, *M. E. Maros*; Mannheim/DE

**Purpose:** Because of global warming, weather variations and fronts are becoming increasingly relevant stressors for the human body, which can cause cardiovascular stress and lead to acute cerebrovascular disease. Furthermore, extreme weather-related spikes in the emergency admission can overwhelm the healthcare providers. Therefore, we investigated whether machine learning-based models can be utilised to predict the number of daily stroke admissions based on current weather systems.
**Methods or Backround:** 7914 (male: 4244, 53.6%) patients diagnosed with cerebral infarction (2015/01-2021/12) were extracted from the local data integration centre. Weather parameters were downloaded from the German Weather Service. Complex weather features were also derived. Patient-level geospatial weather data were matched to admission time to the hospital. Autoregressive neural network (ANN) and boosted trees (XGB) were fitted using a stratified cross-validation setting to predict the total number of ischaemic stroke admissions per day as predictor value either as regression or multiclass classification models.
**Results or Findings:** The ANN model identified mean air temperature as a significantly negative predictor with 2.7% increased risk of stroke admission for each 1°C decrease in ambient temperature. This effect stayed significant to up to 5 days. The accuracy of the XGB classification model on test data was 61.5%. But the weather effect can be quantified as risk score from this model and should be adjusted for in patient level models.
**Conclusion:** We could demonstrate significant associations between complex weather systems and the number of stroke admissions, which can be potentially exploited for resource allocation and optimised therapy planning of neuro/radiological emergencies.
**Limitations:** Single centre retrospective design of a tertiary university clinic with a catchment area of >500.000 patients. Patient-level risk factors were not directly modelled.
**Ethics committee approval:** 2017-825R-MA & 2017-828R-MA.
**Funding for this study:** Medical-Informatics-Initiative (BMBF-MIRACUM:01ZZ1801E).
**Author Disclosures:**
Máté Elöd Maros: Nothing to disclose
Fabian Siegel: Nothing to disclose
Nandhini Santhanam: Nothing to disclose

### RPS 2105-7

**Automated MR carotid vessel wall segmentation with sparse annotation**

X. Li[1], J. Zhang[1], X. Ouyang[1], Y. Wei[1], Y. Zhang[1], Z. Xue[1], Z. Ding[2], *F. Shi*[1], D. Shen[1]; [1]Shanghai/CN, [2]Hangzhou/CN
*(feng.shi@uii-ai.com)*

**Purpose:** To overcome the sparse annotation problem where only limited 2D slices are annotated, and build a deep-learning model for automatic and accurate carotid vessel wall segmentation.
**Methods or Backround:** We propose a multi-modality transfer learning framework (MT-Net) to tackle this problem. Briefly, CTA has been widely used in diagnosing atherosclerosis. Our framework is designed to transfer anatomical knowledge of vessels from CTA to MR by jointly segmenting vessel lumens in CTA and MR, while only constraining available annotations in 2D MR slices. Furthermore, in the MR branch, the lumen results will be used to further guide the multi-channel network for final vessel wall segmentation.
**Results or Findings:** We adopted 50 MR images from the public COSMOS 2022 Challenge dataset, acquired from a 3T Philips scanner with an isotropic resolution of 0.6 mm. The in-house CTA dataset consists of 119 images obtained from GE Revolution scanner with resolutions ranging from 0.4 mm to 1.0 mm. Experiments show that the proposed MT-Net yields more robust vascular anatomy than the baseline ResUNet by improving the Dice Score from 0.628 to 0.906, and the centreline-Dice metric from 0.514 to 0.799. Further experiments indicate Dice Scores of 0.885 and 0.675 in segmenting lumen and wall areas, respectively, outperforming 0.804 and 0.500 produced by the state-of-the-art UNet++ network trained solely with MR data.
**Conclusion:** We show that CT annotations can aid MR segmentation, and sparse annotation can be efficiently used. Experimental results demonstrate satisfactory 3D lumen and vessel wall segmentations, which can aid future analysis of vessel plaque characteristics.
**Limitations:** The shape of vessel walls could be constrained during the segmentation, which will be our future work.
**Ethics committee approval:** IRB of Affiliated Hangzhou First People's Hospital, Zhejiang University School of Medicine, China.
**Funding for this study:** This study was funded in part by the National Natural Science Foundation of China (no. 81830056)
**Author Disclosures:**
Feng Shi: Nothing to disclose
Yuyao Zhang: Nothing to disclose
Ying Wei: Nothing to disclose
Dinggang Shen: Nothing to disclose
Xi Ouyang: Nothing to disclose
Zhong Xue: Nothing to disclose
Jiadong Zhang: Nothing to disclose
Zhongxiang Ding: Nothing to disclose
Xibao Li: Nothing to disclose

### RPS 2105-8

**A study of plaque detection by CNN-based model on carotid CTA images**

C. Chen, H. Zhang, J. Liu, *W. Xu*, R. Zhang; Beijing/CN
*(xweixin@infervision.com)*

**Purpose:** The study aims to propose a CNN-based (convolutional neutral network) model to detect calcified plaques, mixed plaques, and non-calcified plaques automatically on carotid CT angiography (CTA) images.
**Methods or Backround:** 1189 CTA images (538 calcified plaques, 381 mixed plaques, 149 non-calcified plaques) and 164 CTA images (173 calcified plaques, 113 mixed plaques, 48 non-calcified plaques) from 7 hospitals are used as training and internal validation cohort, and 103 CTA images (282 calcified plaques, 430 mixed plaques, 297 non-calcified plaques) from other 3 different hospitals were collected separately as external validation cohort. All plaques were annotated by consensus of three board-certified radiologists. Given plaques adhering to the inner wall of carotid artery, the vessel and part neighbouring tissues (including plaques) were precisely cropped from CTA images, noted as the pre-processing ROI (region of the interest), and other irrelevant regions were removed from original images. The model consisting of sparse convolution, residual block, drop block and UNet architecture was

Saturday developed on the ROIs, noted as SpResUnet. Finally, the cropped ROIs were resized to 448×192×344 as the model inputs, and data augmentation such as rotation, brightness and Gauss noise was also adapted for more robustness.
**Results or Findings:** The model evaluation focused on all predicted plaques. The recall, precision, and f1-score values of the SpResUnet model on internal validation cohort achieved 0.718, 0.725, and 0.721, respectively, and on external validation cohort were 0.868, 0.820, and 0.843, respectively.
**Conclusion:** This CNN-based model on carotid CTA images demonstrated good performance on internal and external validation cohort, which proved the real-world application prospect on the plaque detection of carotid CTA images.
**Limitations:** A prospective validation study will be required in the future.
**Ethics committee approval:** This study was approved by IRB, with a waiver of the written informed consent requirement.
**Funding for this study:** Not applicable.
**Author Disclosures:**
Jun Liu: Nothing to disclose
Huan Zhang: Nothing to disclose
Weixin Xu: Nothing to disclose
Cancan Chen: Nothing to disclose
Rongguo Zhang: Nothing to disclose

### RPS 2105-9

### Automated carotid artery segmentation learned from diameter annotations

*R. Camarasa*, H. Kervadec, D. Bos, M. De Bruijne; Rotterdam/NL
*(r.camarasa@erasmusmc.nl)*

**Purpose:** To develop a fully automated method to delineate the carotid artery lumen and wall on MR images which requires only diameter annotations for training.
**Methods or Backround:** AI based approaches for image segmentation typically require a large training set with voxelwise, manual annotations, which is a time consuming process. We leverage diameter annotations, inspired by the definition of the degree of stenosis, by combining them to anatomical knowledge about the carotid artery. This resulted in the optimisation of 3 objectives; 1) the location of the lumen centroid, 2) the diameters of the lumen and the outer wall, 3) the shape of the artery, and enabled us to train a neural network to predict the full delineation of the lumen and outer wall, circumventing the requirement of voxelwise annotations. We validated our method comparing to manual annotations on a public dataset of 3D Motion Sensitized Driven Equilibrium prepared Rapid Gradient Echo MR images of 24 patients from the Care II study with recent transient ischemic attack or stroke, using 4-fold cross-validation.
**Results or Findings:** Dice coefficient, a measure of the overlap between the predicted segmentation and manually annotated segmentation, was 0.815 ± 0.088 for the lumen and 0.584 ± 0.088 for the vessel wall. This was close to training with voxelwise manual annotations (lumen: 0.844 ± 0.082; wall: 0.709 ± 0.086).
**Conclusion:** Good segmentations of the carotid artery can be obtained by a neural network without extensively annotated training data.
**Limitations:** In this small pilot study, we used synthetic diameter annotations derived from full segmentations.
**Ethics committee approval:** No information provided by the submitter.
**Funding for this study:** This work was funded by Netherlands Organisation for Scientific Research (NWO) VICI project VI.C.182.042.
**Author Disclosures:**
Daniel Bos: Nothing to disclose
Marleen De Bruijne: Nothing to disclose
Robin Camarasa: Nothing to disclose
Hoel Kervadec: Nothing to disclose

### RPS 2105-10

### Performance of an AI-based automated identification of intracranial haemorrhage in real clinical practice

*A. Ayobi*[1], A. Bani-Sadr[2], Y. Chaibi[1], S. Quenet[1], M. Scudeler[1], M. Marx[1], F. Cotton[2]; [1]La Ciotat/FR, [2]Lyon/FR
*(angela.ayobi@avicenna.ai)*

**Purpose:** To evaluate the real-world performance of an FDA-cleared and CE-marked artificial intelligence (AI) based software for intracranial haemorrhage (ICH) detection on head non-contrast-CT (NCCT) scans at a large emergency teleradiology network.
**Methods or Backround:** A retrospective assessment of head NCCTs from patients admitted to several emergency departments over a time period of 2 weeks was performed. The data was received by a teleradiology company (Telediag, Lyon, France) for interpretation. CINA-ICH v1.0 (Avicenna.AI, La Ciotat, France) was integrated into the workflow for flagging suspected ICH findings immediately after NCCT reception. Only the data automatically processed by the device was selected. The results of the algorithm were compared to the final report of senior radiologists used as reference standard. Finally, an independent neuroradiologist reviewed the false positive and false negative cases and established the causes that could have influenced the device performance.
**Results or Findings:** A total of 300 NCCTs were included in the study. Of these, 2 post-contrast-NCCTs were excluded. The comparison of the device's outputs with the reference standard (298 cases with 39 positive ICH) yielded sensitivity of 97.4% [95%CI: 86.5%-99.9%], specificity of 96.1% [95%CI:93.%-98.1%], PPV of 79.2% and NPV of 99.6%. Only 1 ICH was missed (false negative) corresponding to a very subtle subarachnoid haemorrhage. Moreover, incorrect detection of ICH (10 false positives) was due to streak/motion artifacts (n=3), hyperintense venous sinus (n=2), parafalcorial meningioma (n=1), falx cerebri (n=1), chronic stroke area with no acute ICH (n=1) and indeterminate (n=2).
**Conclusion:** This study provides significant real-world data that demonstrate the potential of CINA-ICH to accurately identify life-threatening conditions such as ICH. Indeed, AI-automated ICH detection may have a positive effect on early diagnosis leading to an improvement in patient management and clinical workflow.
**Limitations:** Not applicable.
**Ethics committee approval:** No information provided by the submitter.
**Funding for this study:** Not applicable.
**Author Disclosures:**
Marlene Scudeler: Employee: Avicenna.AI, La Ciotat, France
Sarah Quenet: Employee: Avicenna.AI, La Ciotat, France
Michael Marx: Employee: Avicenna.AI, La Ciotat, France
Angela Ayobi: Employee: Avicenna.AI, La Ciotat, France
Alexandre Bani-Sadr: Nothing to disclose
François Cotton: Employee: Telediag, Lyon, France
Yasmina Chaibi: Employee: Avicenna.AI, La Ciotat, France

### RPS 2105-11

### Improving haemorrhage detection in sparse-view CTs via deep learning

*J. B. Thalhammer*, M. Schultheiss, F. Pfeiffer, D. Pfeiffer, F. Schaff; Munich/DE
*(johannes.thalhammer@tum.de)*

**Purpose:** For diagnosis of intracranial haemorrhage, a Cranial Computed Tomography (CCT) scan is usually performed. However, the increased health risk caused by radiation is a concern. The most important strategy to reduce this potential risk is to keep the radiation dose as low as possible consistent with the diagnostic task. Sparse-view CT is an effective way to reduce dose by reducing the total number of views acquired, albeit at the expense of image quality. Purpose of this study is to investigate possible benefits of deep learning algorithms applied to sparse-view CCTs.
**Methods or Backround:** We use a U-Net architecture to reduce artifacts from sparse-view CCTs, predicting fully sampled reconstructions from sparse-view ones. We evaluate the results by a haemorrhage classification network, trained on fully sampled CCTs to detect haemorrhages. The performance of the U-Net was compared to an analytical approach based on total variation (TV). To quantify the classification performance, we use the area under the receiver operator characteristic curve (AUC-ROC).
**Results or Findings:** The U-Net performed superior compared to TV with respect to quality and automated haemorrhage diagnosis. With the U-Net, the number of views can be reduced from 4096 views to 512 views with almost no decrease in classification performance (AUC-ROC decrease of 0.0013 compared to full-view) and to 256 views with a slight performance decrease (AUC-ROC decrease of 0.0076 compared to full-view).
**Conclusion:** Our results suggest that the classification accuracy of haemorrhages in sparse-view CCTs can be improved substantially by deep learning methods. This demonstrates the feasibility of rapid automated haemorrhage detection on sparse-view CCT data to assist radiologists in routine clinical practice.
**Limitations:** To produce sparse-view CTs, sinograms with a reduced number of views were created from the CT dataset.
**Ethics committee approval:** No information provided by the submitter.
**Funding for this study:** TUM Institute for Advanced Study.
**Author Disclosures:**
Manuel Schultheiss: Nothing to disclose
Johannes Benedikt Thalhammer: Nothing to disclose
Franz Pfeiffer: Nothing to disclose
Florian Schaff: Nothing to disclose
Daniela Pfeiffer: Nothing to disclose

**RPS 2105-12**

**Clinical outcome prediction in paediatric traumatic brain injuries using multiparametric artificial neural networks based on CT findings, GCS score, blood glucose, and Hb levels**

A. Gupta, H. Mahajan, *V. K. Venugopal*, V. Mahajan, S. Gupta, B. Bahl, R. Takhar, S. Pundir, P. Mehta; New Delhi/IN
*(vasanthdrv@gmail.com)*

**Purpose:** To use artificial neural networks to predict the 6 months clinical outcome for patients with traumatic brain injury (TBI) based on Glasgow Coma Scale (GCS) score, blood glucose levels, haemoglobin results, and CT scan findings.
**Methods or Backround:** A deep learning model was trained using the CT studies of patients of age 0-18 years (haemorrhage-intraventricular, intracerebral, epidural, subdural and subarachnoid, midline shift above 5mm and cistern integrity), GCS score (Eye Opening, Verbal Response & Motor Response); blood glucose levels and haemoglobin results. Data from 2020-2022 was collected. Favourable and unfavourable 6 month outcomes were used as primary outcomes, where unfavourable suggests death while favourable outcome suggests survival of the patient. The Deep Learning Model was trained on data for 2,400 data points and validated on 480 data points. An independent test set of 320 data points was reserved for final testing. The Artificial Neural Network was trained with binary cross-entropy loss function with Adam optimizer's Adaptive Moment Estimation back-propagation technique. The model consisted of 3 Fully connected layers with a Relu activation function and a final output layer with a sigmoid activation function. The model was trained with 16 inputs, normalised for continuous data and labels encoded for categorical data.
**Results or Findings:** For patients with a favourable outcome, the mean GCS score was found to be 12.29±1.22, for patients with unfavourable outcomes the mean GCS score was found to be 5.17±2.10. The ROC-AUC score for validation data was found to be 0.988 while the AUC score on the test dataset was 0.976.
**Conclusion:** Thus, Deep Learning-based AI solutions can accurately and precisely predict the favourable/unfavourable outcome for a paediatric patient and provide major assistance in inpatient care.
**Limitations:** Limited number of studies.
**Ethics committee approval:** Waiver obtained for the use of retrospective deidentified data.
**Funding for this study:** No funding was received for this study.
**Author Disclosures:**
Salil Gupta: Employee: CARPL.ai
Abhishek Gupta: Employee: CARPL.ai
Shivam Pundir: Employee: CARPL.ai
Bhanushree Bahl: Employee: CARPL.ai
Parv Mehta: Employee: CARPL.ai
Rohit Takhar: Employee: CARPL.ai
Harsh Mahajan: Other: Research Collaboration with Koninklijke Philips NV research, Oxipit.ai, Quibim, General Electric, Predible Health. Other: Director at Mahajan Imaging. Other: Research Collaboration with Subtle Medical, Qure.ai, Lunit.ai
Vasantha Kumar Venugopal: Other: Research Collaboration with General Electric, Predible Health, Koninklijke Philips NV research, Subtle Medical, Lunit.ai, Oxipit.ai, Quibim. Consultant: CARPL.ai. Other: Research Collaboration with Qure.ai
Vidur Mahajan: Other: Research Collaboration with Quibim, Subtle Medical, Koninklijke Philips NV research, Qure.ai, General Electric, Oxipit.ai, Predible Health CEO: CARPL.ai Other: Research Collaboration with Lunit.ai

**16:00-17:30** **Research Stage 2**

### Research Presentation Session: Genitourinary

### RPS 2107

### GU tract: multimodality imaging diagnosis

**Moderator**
A. Ljimani; Düsseldorf/DE
*(alexandra.ljimani@med.uni-duesseldorf.de)*

**RPS 2107-2**

**Does diffusion-weighted magnetic resonance imaging help in the detection of renal parenchymal disease and staging/prognostication in chronic kidney disease?**

*J. Khatana*; Chandigarh/IN
*(jkhatana67@gmail.com)*

**Purpose:** Diffusion-weighted imaging (DWI) in renal diseases is an upcoming modality, and its utility as an additional marker is yet to be proven. This study intended to find the relationship between apparent diffusion coefficient (ADC) values with renal function tests and stages of chronic kidney disease (CKD) to assess renal dysfunction and to label a cut-off for normal renal function and dysfunction.
**Methods or Backround:** A prospective diagnostic study was conducted on 120 patients: 60 with deranged renal function tests (RFT) and 60 with normal. DWI using a 1.5-Tesla MRI (at b-values of 0 and 500 s/mm2) was done. A region of interest of size 1-2 cm2 was placed on renal parenchyma in the region of the medulla, one each, over the superior, mid, and lower regions of the kidney separately. ADC values were recorded for renal parenchyma and compared.
**Results or Findings:** In patients with renal dysfunction, ADC values were significantly lower than in patients with normal function (1.75 ± 0.25 vs 2.28 ± 0.21 of the right kidney and 1.79 ± 0.17 vs 2.29 ± 0.21 of the left kidney [×10−3 mm2/s]; p = 0.001). ADC values of different stages of CKD showed a decreasing trend with increasing stages.
**Conclusion:** ADC values taken at all poles to get focal involvement of the kidney can be used to measure each kidney separately, and values can be individually correlated with the elevated renal parameters. The cut-off value of the mean ADC for individual kidneys was > 2.28 (×10−3 mm2/s) in normal renal function and < 2.00 (×10−3 mm2/s) in renal dysfunction.
**Limitations:** The sample size of patients was concise, and we could not categorize the patients with low ADC values in our study into acute or chronic renal disease.
**Ethics committee approval:** This study was approved by Ethics committee-SGRDIMSAR, Amritsar
**Funding for this study:** Funding was not required as the study was conducted in a charitable hospital.
**Author Disclosures:**
Jasmin Khatana: Nothing to disclose

**RPS 2107-3**

**Doppler micro vascularisation (MICROV) vs CEUS for characterisation of renal masses: a tertiary care referral university-hospital preliminary experience**

*E. Bertelli*, L. Lattavo, M. Vizzi, R. Ruzga, S. Pastacaldi, V. Valoriani, S. Agostini, V. Miele; Florence/IT
*(elena.bertelli3@gmail.com)*

**Purpose:** This study aimed to evaluate the possible role of MicroV in detecting lesional vascularisation of solid and cystic renal lesions, comparing it to CEUS.
**Methods or Backround:** 169 patients (January 2021-February 2022) underwent a MicroV evaluation before CEUS. Of these, 137 patients (160 lesions, 64 solid and 96 cystic) were enrolled in this monocentric retrospective observational study. For solid masses, both intralesional and peripheral vascularization were analyzed and quantified by MicroV, and then compared to peak enhancement at CEUS. Regarding cystic lesions, we evaluated the presence or absence of vascularization within the cystic wall and/or septa. MicroV results were compared with CEUS findings using Pearson's Chi-square test. An optimal size cut-off for renal lesions was determined with the Youden test.
**Results or Findings:** For solid lesions, a statistically significant correlation (p <0.05) was observed between MicroV parameters and peak enhancement at CEUS. The detection rate (DR) for lesional vascularization on MicroV was 87.5%, while if we consider only lesions larger than the optimal cut-off (14 mm), the DR increases to 98%. In cystic lesions, the MicroV showed a high specificity (93.9%) in predicting CEUS results and a high positive predictive value (84.2%). The concordance was 100% in Bosniak I lesions and 80% in

Bosniak IV, while it was lower for the other Bosniak categories. There was a statistically significant correlation ($p < 0.05$) between Bosniak grade and lesional vascularization at MicroV.
**Conclusion:** MicroV can not replace CEUS but could reduce its use, especially in solid lesions larger than 14 mm and in cysts classified as BIV, a goal particularly important in an Active Surveillance setting and a perspective of hospital savings.
**Limitations:** The study lacks CEUS quantitative evaluation through Time/Intensity curves and histopathological correlation.
It also includes a small number of BIII cystic lesions
**Ethics committee approval:** This study was approved by Comitato Etico Area Vasta Centro (CEAVC): 14660_oss
**Funding for this study:** No funding was received for this study.
**Author Disclosures:**
Lorenzo Lattavo: Nothing to disclose
Sandro Pastacaldi: Nothing to disclose
Michele Vizzi: Nothing to disclose
Vittorio Miele: Nothing to disclose
Elena Bertelli: Nothing to disclose
Ron Ruzga: Nothing to disclose
Vittoria Valoriani: Nothing to disclose
Simone Agostini: Nothing to disclose

### RPS 2107-4

**Reduced renal apparent diffusion coefficient after COVID-19-associated acute kidney injury**

*P. Eckerbom*[1], T. Luther[1], E. Cox[2], J. Weis[1], M. Hultström[1], M. Lipcsey[1], S. Francis[2], P. Liss[1], R. Frithiof[1]; [1]Uppsala/SE, [2]Nottingham/UK
*(per.eckerbom@akademiska.se)*

**Purpose:** The study aimed to investigate if there were any differences regarding renal blood flow, oxygenation or tissue characteristics among recovered patients, previously intensive care treated for severe COVID-19 without and with different grades of AKI.
**Methods or Backround:** 22 patients, previously intensive care treated for severe COVID-19 with respiratory failure, were included in the study. Eight of the patients had AKI grade 3; six had AKI grade 1, and eight did not have AKI during their hospitalization. The patients were scanned at 3T with sequences for phase contrast, ASL, BOLD, TRUST, DWI, T1 and T2, approximately five months after their first day of intensive care. All patients had a plasma creatinin within normal range within two years prior to hospitalization and had no history of renal disease.
**Results or Findings:** Apparent diffusion coefficient (ADC) was significantly reduced both in the cortex and in the medulla in the AKI grade 3 group compared to the no AKI group. Also, total and global perfusion and estimated glomerular filtration rate (eGFR) were significantly reduced in the AKI grade 3 group compared to the no AKI group. No significant differences in renal oxygenation, T1, T2 or renal volume were found between the groups.
**Conclusion:** On follow-up after intensive care treatment for severe COVID-19, patients that had high-grade AKI (AKI grade 3) during hospitalization had reduced cortical and medullary ADC, reduced total renal blood flow and reduced eGFR compared to patients that did not have AKI. Reduced ADC might indicate edema or fibrosis. No differences between the groups were found regarding T1, T2 or kidney size speaking against edema.
**Limitations:** The study included a relatively small number of patients, variation in follow-up time, and risk of bias due to comorbidities among the participants.
**Ethics committee approval:** This study was approved by Uppsala Regional Ethical Review Agency approval No. 2020-02697 with amendments No.2020-03629, 2020-05758 and 2021-02205.
**Funding for this study:** Several types of funding were received for this study.
**Author Disclosures:**
Robert Frithiof: Nothing to disclose
Tomas Luther: Nothing to disclose
Miklós Lipcsey: Nothing to disclose
Jan Weis: Nothing to disclose
Michael Hultström: Nothing to disclose
Eleanor Cox: Nothing to disclose
Per Liss: Nothing to disclose
Per Eckerbom: Nothing to disclose
Susan Francis: Nothing to disclose

### RPS 2107-5

**First experience with photon counting CT in the evaluation of urinary stone composition: a standardised phantom study**

*D. P. Overhoff*[1], S. Faby[2], R. Stoll[1], A. Hesse[3], H. U. Schmelz[1], M. Juergens[2], B. Schmidt[2], T. Nestler[1], S. Waldeck[1]; [1]Koblenz/DE, [2]Forchheim/DE, [3]Bonn/DE

**Purpose:** CT plays a vital role in diagnosis and treatment planning in urolithiasis. Photon counting detector (PCD)- CT inherits the spectral data and thus the information on the composition of urinary stones with every examination. This study evaluates the accuracy of a novel PCD-CT in analyzing the composition of urinary stones.
**Methods or Backround:** N=148 mixed urinary stones were examined ex-vivo in a phantom representing the abdomen using a PCD-CT (NAEOTOM Alpha, Siemens Healthineers, Forchheim, Germany). The datasets were analyzed with the syngo.CT DE Calculi Characterization prototype application (syngo.via VB60, Siemens Healthineers, Forchheim, Germany) regarding composition. As a standard of reference, the urinary stone composition was determined by IR spectroscopy and manually measured by water displacement method and calliper. The PCD-CT measurements were compared with the standard of reference, and the Pearson correlation coefficient and sensitivity/specificity were calculated.
**Results or Findings:** The mean volume of urinary stones was 165 mm3 (min 10 mm3 - max 6400 mm3) with a mean diameter of 6.4 mm (min 2.5mm - max 30.1mm) measured by water displacement method and calliper. 86.5% (n=128) were detected automatically, and 87.8% (n=130) of the urinary stones could be analyzed. The urinary stones that could not be analyzed were significantly smaller, with a mean= 3.7±1.0 mm (t-test 0.002). For the uric stones that were available for analysis, a significant correlation of 0.570 ($p < 0.0001$) was found between the standard of reference IR and PCD-CT. PCD-CT demonstrated a sensitivity of 60.0% and a specificity of 94.5% for all urinary stones.
**Conclusion:** The new PCD-CT technology yields the possibility to reliably distinguish the composition of urinary stones between uric and non-uric stones. A limitation seems to be the size of the stones with poorer detectability and the difference ability of smaller stones.
**Limitations:** This is an ex vivo study.
**Ethics committee approval:** No information provided by the submitter.
**Funding for this study:** No funding was received for this study.
**Author Disclosures:**
Rico Stoll: Nothing to disclose
Albrecht Hesse: Nothing to disclose
Bernhard Schmidt: Employee: Siemens Healthcare GmbH
Tim Nestler: Nothing to disclose
Markus Juergens: Employee: Siemens Healthcare GmbH
Daniel Pasqual Overhoff: Nothing to disclose
Stephan Waldeck: Nothing to disclose
Hans U. Schmelz: Nothing to disclose
Sebastian Faby: Employee: Siemens Healthcare GmbH

### RPS 2107-6

**The usefulness of corticomedullary-phase computed tomography urography in patients with suspected acute renal colic visiting the emergency department**

*S. Lee*, Y. S. S. Park, B. N. Park, J. Lee, J. W. Choi, K. A. A. Kim, C. H. Lee; Seoul/KR

**Purpose:** The study aimed to evaluate the sensitivity of corticomedullary-phase imaging for detecting urinary stones in patients with renal colic who visited the emergency department.
**Methods or Backround:** This retrospective study included 253 patients with suspected renal colic from two tertiary hospitals in South Korea who visited the emergency department and underwent computed tomography (CT) urography. Two radiologists blinded to the clinical history independently reviewed the corticomedullary-phase images. The sensitivity for identifying urinary stones was evaluated for each reviewer. After the initial evaluation, the images were re-evaluated based on patient history. The sensitivity of the re-evaluation was recorded.
**Results or Findings:** Of 253 patients, 150 (59%) had urinary stones. Among them, significant stones were observed in 138 patients (92%) and obstructive changes on CT in 124 patients (82.7%). For identifying significant urinary stones, the sensitivity was 98.6% (136/138) for both reviewers. For identifying significant urinary stones with urinary obstruction, the sensitivity was 99.2% (123/124) for reviewer 1 and 100% (124/124) for reviewer 2. Sensitivity for identifying significant stones increased from 98.6% to 100% for reviewer one and from 98.6% to 99.3% for reviewer two in the re-evaluation session.
**Conclusion:** Corticomedullary-phase CT urography was sensitive for diagnosing urolithiasis in patients with acute renal colic who visited the emergency department.

**Limitations:** First, it had a retrospective design and included a relatively small number of patients with urolithiasis. Second, the reviewers were aware of the purpose of our study; accordingly, the sensitivity might have been affected by the presumably meticulous inspection of the images to identify stones. Third, we analyzed the corticomedullary-phase image on CT urography, which has a slightly more rapid acquisition time than the portal venous-phase image, a commonly used single-phase enhanced CT image.
**Ethics committee approval:** No information provided by the submitter.
**Funding for this study:** This research received no specific grant from any funding agency.
**Author Disclosures:**
Jongmee Lee: Nothing to disclose
Chang Hee Lee: Nothing to disclose
Kyeong Ah Ah Kim: Nothing to disclose
Yang Shin Shin Park: Nothing to disclose
Bit Na Park: Nothing to disclose
Jae Woong Choi: Nothing to disclose
Seokyoung Lee: Nothing to disclose

### RPS 2107-7

### Using relative aldosterone secretion index in selected patients undergoing adrenal venous sampling may differentiate unilateral primary aldosteronism from bilateral disease

*A-C. Su*[1], K-H. Tu[1], T-W. Sheng[2], Y-C. Wong[1], L-J. Wang[2], C-C. Huang[2], C-B. Chang[1]; [1]Taoyuan/TW, [2]New Taipei/TW
*(fattytale@gmail.com)*

**Purpose:** Relative aldosterone secretion index (RASI) is unaffected by hormone values from the contralateral side. Thus, we believe it may help primary aldosteronism (PA) subtyping, especially in unilaterally successful adrenal venous sampling (AVS). We hypothesized in any successful side of AVS, having RASI < 1 suggests contralateral disease, owing to the effect of contralateral suppression, while RASI > 1, the possibilities are ipsilateral or bilateral diseases, and differentiation was further made by post-saline-infusion-test (SIT) aldosterone level, with < 10 ng/dL favouring bilateral disease and > 10 ng/dL for ipsilateral ones.
**Methods or Backround:** We retrospectively reviewed 37 patients who underwent AVS in our centre, with the main protocol being unstimulated bilateral simultaneous sampling. AVS Data were viewed as right and left sides, for each side had their own RASI independently. Diagnostic consistency between lateralization index (LI) and RASI in patients with bilateral successful cannulation was analyzed by Cohen's kappa coefficient. For patients who underwent surgical treatment, the diagnostic performance of LI and RASI were further validated by ROC curve and area under the curve.
**Results or Findings:** RASI and LI have high diagnostic consistency (k=0.738) and higher diagnostic agreement (k=0.797) after post-SIT aldosterone level was added for further differentiation. ROC curve was constructed using RASI and post-SIT results as a predictor to validate the post-adrenalectomy outcome, and the area under the curve was 0.989, indicating excellent diagnostic accuracy.
**Conclusion:** With proper cut-off value and assistance of post-SIT aldosterone level, RASI is a useful diagnostic index in AVS data interpretation, even in unilaterally successful AVS.
**Limitations:** This is a retrospective, single-centre study with a limited sample size and potential bias. Further confirmation of the cut-off value in a different protocol or with a larger sample size is needed.
**Ethics committee approval:** IRB No.: 202200360B0
**Funding for this study:** This research received no external funding.
**Author Disclosures:**
Chun-Bi Chang: Nothing to disclose
Chen-Chih Huang: Nothing to disclose
An-Chi Su: Nothing to disclose
Kun-Hua Tu: Nothing to disclose
Li-Jen Wang: Nothing to disclose
Yon-Cheong Wong: Nothing to disclose
Ting-Wen Sheng: Nothing to disclose

### RPS 2107-8

### Long-term follow-up results of multiparametric prostate MRI and prognostic value of PI-RADSv2

Ö. Önder, *Y. Yaraşir*, V. Gürler, M. Ayva, M. S. Yazici, B. Akdogan, M. Karçaaltincaba, M. N. Özmen, D. Akata; Ankara/TR
*(yasinyarasirmd@gmail.com)*

**Purpose:** The study aimed to reveal the prognostic value of PI-RADSv2 by calculating long-term clinically-significant prostate cancer(cs-PCa) diagnosis-free survival rates and evaluating the relationship between index PI-RADSv2 scores and National Comprehensive Cancer Network (NCCN) prostate cancer(PCa) risk groups.
**Methods or Backround:** A data set was created by retrospectively collecting the clinicoradiological and histopathological data of adult patients(n=2456) who underwent mp-MRI with the suspicion of PCa in between 2014-2021. Cases with at least one histopathological examination or at least one follow-up mp-MRI or at least one year of adequate clinical follow-up (prostate-specific antigen[PSA], digital rectal exam) and baseline PI-RADSv2 scores of 1-2(n=385) and 3(n=478) were filtered from the data-set and included in the cs-PCa diagnosis-free survival analysis. Kaplan-Meier test was used for survival analysis. Subsequently, 826 cases with a histopathological examination after mp-MRI were filtered, and distributions of index PI-RADSv2 scores and NCCN PCa risk groups were evaluated. Based on the NCCN guideline: very low, low and intermediate-favourable risk groups were classified as 'Favourable-PCa', while cases in the risk groups with intermediate-unfavourable, high and very high were grouped as 'Unfavourable-PCa'.
**Results or Findings:** The 3-year cs-PCa diagnosis-free survival rates were 96.5% and 90.9% in the cases with initial PI-RADSv2 scores of 1-2 and 3, respectively. While MRI-defined PSA density (mPSAD) significantly affects the survival for both subgroups, the presence of previous negative biopsy significantly affects survival only in the PIRADSv2 3 subgroup. Index PI-RADS scores were found inversely proportional to non-neoplastic biopsy rates and directly proportional to unfavourable-PCa rates in histopathologically-correlated cases.
**Conclusion:** The cs-PCa diagnosis-free survival rate is considerably high in cases with PI-RADS≤3 during long-term follow-up. This finding may strengthen the clinician's hand in patient management. The higher the index PI-RADSv2 score, the higher the probability of unfavourable-PCa.
**Limitations:** Single-center retrospective study. No standard follow-up protocol.
**Ethics committee approval:** No information provided by the submitter.
**Funding for this study:** No funding was received for this study.
**Author Disclosures:**
Bulent Akdogan: Nothing to disclose
Muşturay Karçaaltincaba: Nothing to disclose
Müjdat Ayva: Nothing to disclose
Deniz Akata: Nothing to disclose
Mustafa Nasuh Özmen: Nothing to disclose
Volkan Gürler: Nothing to disclose
Yasin Yaraşir: Nothing to disclose
Mustafa Sertac Yazici: Nothing to disclose
Ömer Önder: Nothing to disclose

### RPS 2107-9

### Single-centre experience of multiparametric prostate MRI and PI-RADS v2: exploring the learning curve

Ö. Önder, *V. Gürler*, Y. Yaraşir, M. S. Yazici, B. Akdogan, A. D. Karaosmanoğlu, M. Karçaaltincaba, M. N. Özmen, D. Akata; Ankara/TR
*(volkangurler91@gmail.com)*

**Purpose:** This study aimed to share our institutional multiparametric prostate-MRI(mp-MRI) experience by temporally evaluating the initial PI-RADSv2 score distribution and radiopathological correlation results over a 7-year period.
**Methods or Backround:** Radiopathological data of adult patients who underwent mp-MRI with the suspicion of prostate cancer (PCa) at our institution between 2014-2021 were collected retrospectively(n=2456). Only the initial mp-MRI results were considered. All mp-MRIs had been reported according to PI-RADS v2 by four readers, each with at least 10 years of experience in abdominal radiology. ISUP≥2 was considered clinically significant prostate cancer (cs-PCa). Histopathologically proven PCa patients whose time interval between initial MRI and biopsy exceeds one year were excluded from the radio pathological-correlation analysis. PI-RADSv2 score distribution and diagnostic performance metrics of radiopathological correlation were temporally evaluated for both the whole department and each reader.
**Results or Findings:** Of included 2456 patients, 783(31.9%) had a histopathological examination. As our institutional experience has grown over the years, the rate of PI-RADS 1-2 assigned cases increased (from 27.5% to 47.1%), and the rate of PI-RADS 4 cases decreased(from 24.4% to 14%). Although the PI-RADS 3 rate increased in the early period(from 36.5% to 42%), a sharp decline to 30.5% has been observed since 2019. Despite declining PI-RADS 4 and roughly stable PI-RADS 5 rates, cs-PCa and PCa detection rates in PI-RADS≥4 cases have increased significantly over the years, both on a readership basis and in general terms(from 39-54% to 55-74%). However, no significant change was observed in the cancer detection rates in PI-RADS≤3 cases, which shows that our learning curve has improved the positive predictive value but does not affect the negative predictive value much.
**Conclusion:** The temporal change in the PI-RADSv2 score distribution and radiopathological correlation results can reflect an institution's growing mp-MRI experience and demonstrate the learning curve.
**Limitations:** The study is a single-centre retrospective study.
**Ethics committee approval:** No information provided by the submitter.

**Funding for this study:** No funding was received for this study.
**Author Disclosures:**
Bulent Akdogan: Nothing to disclose
Muşturay Karçaaltincaba: Nothing to disclose
Deniz Akata: Nothing to disclose
Mustafa Nasuh Özmen: Nothing to disclose
Volkan Gürler: Nothing to disclose
Yasin Yaraşir: Nothing to disclose
Ali Devrim Karaosmanoğlu: Nothing to disclose
Mustafa Sertac Yazici: Nothing to disclose
Ömer Önder: Nothing to disclose

### RPS 2107-10

**Leiomyoma or sarcoma? MRI performance in the differential diagnosis of suspicious uterine masses**

*M. Bonatti*[1], R. Valletta[1], F. Lombardo[2], G. Negri[1], F. Ferro[1]; [1]Bolzano/IT, [2]Negrar/IT
*(matteobonatti@hotmail.com)*

**Purpose:** The study aimed to assess the diagnostic performance of MRI in distinguishing between leiomyomas and malignant/potentially malignant mesenchymal neoplasms in patients with rapidly enlarging/sonographically suspicious uterine masses.
**Methods or Backround:** IRB-approved retrospective study including 88 patients (51±11 years) who underwent MRI for rapidly enlarging/ sonographically suspicious uterine mass at our Institution between Jan2016 and Dec2021, followed by surgery or >12 months sonographic follow-up. Two radiologists (14 and 6 years of experience in gynaecological imaging) independently evaluated MRI images; discrepancies were solved by consensus. Qualitative image analysis included lesion's margins (sharp/ irregular), architecture (homogeneous/inhomogeneous), endometrial infiltration (yes/no), necrotic areas (yes/no), hemorrhagic areas (yes/no), and predominant signal intensity on T1-WI, T2-WI, CE T1-WI, DWI, and ADC map. Qualitative image analysis included lesion size, signal intensity on T2-WI and CE T1-weighted images normalized to myometrium, and signal intensity on DWI and ADC map normalized to the endometrium. Lesions were finally classified as benign, indeterminate, or malignant tumour.
**Results or Findings:** After surgery (52/88 patients) or follow-up (36/88 patients, 33±20 months), 83/88(94.3%) lesions were classified as benign and 5/88(5.7%) as malignant/potentially malignant (3 sarcomas and 2 STUMPs). The presence of necrotic areas, hyperintensity on DWI and higher lesion/endometrium DWI ratio were significantly associated with malignant/potentially malignant lesions (p=0.027, 0.008 and 0.015, respectively). DWI ratio showed an AUC of 0.816(95%CI 0.549-1.000) in identifying malignant/potentially malignant lesions, and a cut-off value of 0.82 led to 89% sensitivity and 80% specificity. The two readers identified malignant lesions with 95.5% accuracy (80.0% sensitivity, 96.4% specificity, 57.1 PPV, 93.3% NPV).
**Conclusion:** MRI has high accuracy in identifying malignant/potentially malignant myometrial masses, but in everyday practice, the positive predictive value is relatively low, given the low pre-test malignancy probability.
**Limitations:** This is a retrospective study with a relatively small patients population.
**Ethics committee approval:** Given this is a retrospective study, the need for informed consent has been waived.
**Funding for this study:** No funding was received for this study.
**Author Disclosures:**
Giovanni Negri: Nothing to disclose
Matteo Bonatti: Nothing to disclose
Fabio Lombardo: Nothing to disclose
Riccardo Valletta: Nothing to disclose
Federica Ferro: Nothing to disclose

### RPS 2107-11

**Comparison of surgeon's intraoperative assessment of residual tumour and postoperative findings on CT in patients with advanced stage epithelial ovarian cancer: a retrospective cohort study**

*A. G. Anghelone*, G. Avesani, C. Panico, I. Peters, A. Fagotti, R. Manfredi, E. Sala, B. Gui; Rome/IT
*(andreaanghelone@gmail.com)*

**Purpose:** The study aimed to compare the postoperative CT findings to the surgeon's intraoperative assessment and to determine their effect on survival.
**Methods or Backround:** Primary debulking surgery (PDS) for advanced epithelial ovarian cancer aims to resect all macroscopically visible lesions. Previous studies showed a discrepancy of 20-48% between the surgeon's intraoperative assessment of residual tumour (RT) and findings on postoperative computed tomography (CT) scans. Patients with radiographical lesions that were suspicious of malignancy had a worse prognosis. All patients with newly diagnosed FIGO stage III-IV ovarian cancer, subjected to complete or near complete PDS (RT < 2.5 mm) at our centre between June 2019 and June 2021 and with a CT within 50 days from PDS, were evaluated. CT scans were assessed using a 5-point scale, ranging from benign to malignant. Indeterminate results were omitted from further analyses.
**Results or Findings:** A total of 145 patients were identified. Clinical data and postoperative CT scans could be retrieved for 103 patients. Of these 103 patients, CT findings corresponded to the surgeon's intraoperative assessment in 76.7% of cases. In 14 patients (13.6%), CT findings were scored as indeterminate. Lesions that were deemed probably malignant or malignant were found in seven (6.8%) and three patients (2.9%), respectively, with a median lesion size of 24.5 mm (range 8.0-85.0 mm). After comparing radiologically concordant and discordant findings, no differences in disease-free or overall survival were seen.
**Conclusion:** In line with previous data, a discrepancy of 22.5% was found between the surgeon's intraoperative assessment and radiological postoperative CT scan on the presence of RT. Nonetheless, CT findings did not affect patient survival.
**Limitations:** This is a retrospective study with a limited number of patients.
**Ethics committee approval:** No information provided by the submitter.
**Funding for this study:** No funding was received for this study.
**Author Disclosures:**
Inge Peters: Nothing to disclose
Giacomo Avesani: Nothing to disclose
Andrea Giovanni Anghelone: Nothing to disclose
Benedetta Gui: Nothing to disclose
Riccardo Manfredi: Nothing to disclose
Evis Sala: Nothing to disclose
Camilla Panico: Nothing to disclose
Anna Fagotti: Nothing to disclose

### RPS 2107-12

**MR defecography: comparison of HMO system measurement between supine and lateral decubitus patient position**

*T. Chulroek *[1], A. Nunthanawanich[1], K. Sasiwimonphan[1], M. Harisinghani[2]; [1]Bangkok/TH, [2]Boston, MA/US
*(thitinan.c@chula.ac.th)*

**Purpose:** The study aimed to compare the measurement value and grading in dynamic magnetic resonance defecography (MRD) within closed-magnet system of pelvic floor dysfunction patients performed in supine versus lateral decubitus position using the H line, M line and organ prolapse (HMO) classification system.
**Methods or Backround:** Between 2017-2019, 100 patients with pelvic floor dysfunction underwent MRD during defecation in both supine and lateral decubitus positions. MR images were measured and graded by two blinded radiologists. Mean value of each HMO parameter and grading severity were compared between supine and lateral positions. Image quality (IQ) between two positions was also evaluated. Paired T-test and Wilcoxon ranked test were performed for significant difference. P<0.05 was considered as statistical significance.
**Results or Findings:** For HMO measurement, M-line, levator plate angle (LPA), urethral hypermobility (UH), uterine prolapse and peritoneocele had significantly higher mean values when measured in lateral decubitus position than in supine position. For grading, M-line, uterine prolapse and peritoneocele also had more grading severity in lateral decubitus than supine position with statistical significance. Only anterior rectocele had mean value and grading severity in supine more than lateral position (p = 0.003 and p = 0.005). IQ in supine was better than lateral decubitus position (p<0.001).
**Conclusion:** MRD in lateral decubitus showed more severe degree of pelvic floor disorder in most parameters based on the HMO grading system irrespective of inferior imaging quality.
**Limitations:** This is a non-randomised, retrospective study. No gold standard reference, such as a correlative surgical report, because most patients were treated by conservative treatment, and operative note did not report grading of pelvic organ descent.
**Ethics committee approval:** Institutional Review Board (IRB) and Health Insurance Portability and Accountability Act (HIPPA) approval were obtained and requirement for informed consent was waived by our hospital IRB.
**Funding for this study:** All authors declare that there is no conflict of interest. This study was unfunded.
**Author Disclosures:**
Thitinan Chulroek : Nothing to disclose
Auttapon Nunthanawanich: Nothing to disclose
Mukesh Harisinghani: Nothing to disclose
Kewalee Sasiwimonphan: Nothing to disclose

**16:00-17:30** **Research Stage 3**

### Research Presentation Session: Oncologic Imaging

### RPS 2116

### Quantification of tumour response with imaging

**Moderator**
J. Dinkel; Munich/DE
**Author Disclosures:**
Julien Dinkel: Advisory Board: Parexel, Böhringer

### RPS 2116-2

**Artificial Intelligence-based response prediction in patients with muscle-invasive bladder cancer treated with neoadjuvant immunotherapy**

*J. Greidanus*[1], S. Trebeschi[1], L. S. Mertens[1], R. G. H. Beets-Tan[1], D. Raggi[2], B. W. van Rhijn[1], M. van der Heijden[1], A. Necchi[2], T. N. Boellaard[1]; [1]Amsterdam/NL, [2]Milan/IT
*(j.greidanus@nki.nl)*

**Purpose:** Neoadjuvant immunotherapy showed promising results in muscle-invasive bladder cancer (MIBC). 42-46% of patients achieve a complete pathological response (pCR), urging the quest for predictive biomarkers. We hypothesised that Artificial Intelligence (AI) can automatically quantify predictive image features for immunotherapy response using multiparametric MRI (mpMRI).
**Methods or Backround:** We identified 98 MIBC patients (≥cT2N0 and cM0) who received neoadjuvant immunotherapy, followed by radical cystectomy in the PURE-01 trial (n=79) and NABUCCO trial (n=19). A mpMRI was acquired at baseline and after immunotherapy. A nnU-Net model was trained using T2-weighted, DWI b=1000, ADC-map, and DCE at 8 and 12 seconds. This model was used to automatically detect a tumour. Volumetric and radiomics analyses were performed to determine its predictive value for pCR at baseline and after immunotherapy. We trained our models on the PURE-01 dataset, with an internal validation set (PURE-01-test, n=15) and NABUCCO as external validation.
**Results or Findings:** AI-derived volumetric analysis did not predict pCR at baseline (p=0.36) but showed significant performance after immunotherapy (PURE-01-test: AUC=0.86, p=0.0003) and when comparing baseline-vs-after immunotherapy (PURE-01-test: AUC=0.79, p=0.0004). Only the comparison between baseline-vs-after immunotherapy was performant on the external validation set (AUC=0.67). Similar values were observed for the radiomics analysis. The AI algorithm underestimated the radiologist's ROI but outperformed it in predicting pCR.
**Conclusion:** This study showed a promising predictive performance of AI models using mpMRI features obtained after neoadjuvant immunotherapy in MIBC. In the future, this might enable patient selection for organ-sparing options. Predictions based on tumour features derived from the mpMRI images before neoadjuvant immunotherapy remain challenging.
**Limitations:** Due to the unique nature of this novel treatment strategy, the dataset is yet limited. Differences between scanners influence the results. CT scans should also be considered for inclusion.
**Ethics committee approval:** No information provided by the submitter.
**Funding for this study:** Funding was received from Dutch Uro-Oncology Study group (DUOS).
**Author Disclosures:**
Stefano Trebeschi: Nothing to disclose
Thierry N. Boellaard: Nothing to disclose
Joyce Greidanus: Nothing to disclose
Bas W.G. van Rhijn: Nothing to disclose
Daniele Raggi: Nothing to disclose
Laura S. Mertens: Nothing to disclose
Andrea Necchi: Research/Grant Support: MSD, AstraZeneca, Ipsen, Gilead Employee: Bayer Other: Travel, Accommodations, Expenses: Roche, MSD, AstraZeneca, Janssen, Rainer Therapeutics, Pfizer Consultant: MSD, Roche, Bayer, AstraZeneca, Clovis Oncology, Janssen, Incyte, Seattle Genetics/Astellas, Bristol-Myers Squibb, Rainier Therapeutics, Bycicle Therapeutics, GlaxoSmithKline, Basilea Pharmaceutica, Catalym Other: Honoraria: Roche, MSD, AstraZeneca, Janssen, Foundation Medicine, BMS, Astellas
Regina G. H. Beets-Tan: Nothing to disclose
Michiel van der Heijden: Research/Grant Support: Bristol-Myers Squibb, AstraZeneca and Roche (all paid to the Netherlands Cancer Institute). Consultant: Bristol-Myers Squibb, Merck, Merck Sharp & Dohme, Roche, AstraZeneca, Seattle Genetics and Janssen (all paid to the Netherlands Cancer Institute).

### RPS 2116-3

**Baseline and post-treatment advanced MRI of extremity soft-tissue non-myxoid undifferentiated pleomorphic sarcoma**

*R. F. Valenzuela*, B. Amini, M. Canjirathinkal, E. Duran-Sierra, W. Murphy, H. Idrees, J. Madewell, C. Costelloe; Houston, TX/US
*(raulvp@estigia.cl)*

**Purpose:** Undifferentiated pleomorphic sarcomas (UPS) post-therapeutically demonstrate hemosiderin deposition, granulation tissue formation, fibrosis, and calcification. To determine the value of advanced MRI with Diffusion-WI (DWI), Contrast-enhanced Susceptibility-Weighted-WI (SWI-CE), and Perfusion-WI (PWI/DCE) in UPS treatment response.
**Methods or Backround:** A Retrospective IRB-authorized study included 26 extremity non-myxoid UPS patients with imaging and resection from February 2021-October 2022. Volumetric tumour segmentation from DWI, SWI-CE, and PWI/DCE, was obtained at Baseline (BL), Post-Chemotherapy (PC), and Post-Radiation (PRd).
**Results or Findings:** Treatment-induced tumour necrosis (TIN) in surgical specimens separated cases into Good Responders (GR: TIN>90%, 13 patients), Partial-Responders (PR: 89-31%, eight patients), and Non-Responders (NR: <30%, five patients). Tumour Volume: GR increased by 60% at PC and 90% at PRd. GR baseline ADCmean was 1200, and PR-NR was 1500 x10-6 mm2/sec. At PRd, GR ADC increased by 45% and PR-NR by 30%. SWI patterns: GR: "complete ring" in 82%. PR: "incomplete ring" in 50%. NR: "globular" in 60%. "Complete ring" significantly correlated with GR (p=0.0000021) and "incomplete ring" with PR (p=0.1562). The globular pattern was associated with NR (p=0.1455). PWI/DCE: Type V was the most common time-intensity curve (TIC) at BL. GR: 100% type II TIC at PRd. Post-radiation PWI/DCE patterns: GR: "capsular-like" in 69% (p=6.7x10-5), PR: "unipolar" in 87% (p=0.00016). NR: "bipolar" in 60% (p=0.145).
**Conclusion:** RECIST/WHO criteria often cannot separate GR from PR-NR. Post radiation volumetric pseudoprogression, >40% ADC increase, SWI-CE "complete ring," PWI/DCE Type II TIC and "capsular-like" patterns correlate with >90% TIN. Suboptimal responders showed SWI "incomplete ring," TIC III/IV/V, and "unipolar" or "bipolar" enhancement. Advanced MRI is a valuable tool for UPS treatment assessment.
**Limitations:** Small sample.
**Ethics committee approval:** No information provided by the submitter.
**Funding for this study:** The John S. Dunn, Sr. Distinguished Chair in Diagnostic imaging. M.R Evelyn Hudson Foundation Endowed Professorship.
**Author Disclosures:**
Hajra Idrees: Nothing to disclose
Elvis Duran-Sierra: Nothing to disclose
Behrang Amini: Nothing to disclose
Colleen Costelloe: Nothing to disclose
Raul Fernando Valenzuela: Nothing to disclose
William Murphy: Nothing to disclose
Mathew Canjirathinkal: Nothing to disclose
John Madewell: Nothing to disclose

### RPS 2116-4

**Multi-graphical analysis of total-body 11C-MET dynamic PET in multiple myeloma patients**

*W. Gu*, Y. Wang, Y. Gu, Q. Ge, X. Yu, J. Li, L. Li, Y. Chen, Y. Zhou; Shanghai/CN
*(wenjian.gu@united-imaging.com)*

**Purpose:** This study proposes a multi-graphical analysis method using the Gjedde-Patlak (Patlak) plot with Logan plot that automatically identifies the time point when the reversible kinetics of tracer can be detected, in order to better describe the pharmacokinetic processes of tracers involving reversible uptake.
**Methods or Backround:** In quantitative dynamic PET study, reversible tracer kinetics in tissue are often neglected due to the limited PET scanning time or sensitivity, hindering the full analysis of tracer kinetics. In this study, 60-minute dynamic total-body 11C-MET PET/CT images of twelve multiple myeloma patients were obtained on a uEXPLORER PET-CT scanner (United Imaging Healthcare). Regions of interests (ROIs) of major organs and bone marrow were drawn. Conventional Patlak and multi-graphical model were applied and assessed using ROI-wise and voxel-wise analysis (parametric imaging).
**Results or Findings:** Multi-graphical method demonstrated better goodness-of-fit in ROI-wise analysis and higher parametric image quality. The normalised mean square error (NMSE) of multi-graphical method decreased by 70% compared to that of Patlak model (0.0014 and 0.0048, respectively). The NMSEs of all ROI fitting with multi-graphical method were significantly reduced compared to that of Patlak model. The average time when the 11C-MET reversible kinetic were detected of all ROIs was 24.33 minutes. The ROI reversible kinetic time points for bone marrow, liver, lung, spleen, kidney and muscle were 29.42, 29.52, 17.08, 23.67, 18.67 and 27.65 minutes, respectively. Based on the acquired time of split, the Ki image using Patlak plot of 5-24 minutes had better tumour-to-background ratio (TBR) compared to that of 5-60 minutes.

**Conclusion:** Our proposed method can automatically and effectively capture the splitting time point between irreversible and reversible kinetics of 11C-MET, improving 11C-MET PET kinetic model fitting and parametric imaging to achieve higher diagnostic value.
**Limitations:** No limitations were identified.
**Ethics committee approval:** This study has been approved by the ethics committee of Renji Hospital with written informed consent waived.
**Funding for this study:** No funding was received for this study.
**Author Disclosures:**
Qi Ge: Nothing to disclose
Lianghua Li: Nothing to disclose
Yihan Wang: Nothing to disclose
Yumei Chen: Nothing to disclose
Wenjian Gu: Nothing to disclose
Jiajin Li: Nothing to disclose
Yun Zhou: Nothing to disclose
Yue Gu: Nothing to disclose
Xiaofeng Yu: Nothing to disclose

### RPS 2116-5

**First in-human total-body 68Ga-FAPI-04 PET kinetic modeling and parametric imaging in pancreatic and gastric cancer patient studies**

*X. Yang*, R. Chen, Y. L. Ng, Y. Chen, X. Yu, C. Zheng, G. Huang, J. Liu, Y. Zhou; Shanghai/CN
*(xinlan.yang@united-imaging.com)*

**Purpose:** 68Ga-FAPI-04 PET has been used in clinical oncology for diagnosis and treatment. Parametric images generated from dynamic total-body PET, a powerful method for quantification of tracer uptake, can provide improved performance compared to standardised uptake value (SUV) imaging in clinical diagnosis. However, the knowledge of total-body kinetic modelling and parametric imaging of 68Ga-FAPI-04 is limited.
**Methods or Backround:** Dynamic total-body 68Ga-FAPI-04 PET/CT (uEXPLORER, United Imaging Healthcare) was performed on thirteen patients. The region of interest (ROI) was drawn manually over normal organs and pathological lesions. The time activity curves (TACs) were fitted by three compartment models (reversible one-tissue compartment model, irreversible two-issue compartment model, reversible two-tissue compartment model (2T4K)), and multi-graphical analysis (Logan, Patlak, RE plots). The total distribution volumes (V_ts) were calculated from compartment models and Logan plot at ROI and voxel-wise level (parametric V_t image).
**Results or Findings:** The compartment model of 2T4K had the lowest Akaike Information Criterion and fitted well to the measured ROI TACs. The fitting of Logan and RE plots were better than Patlak. The V_t images showed less image noise and higher lesions conspicuity compared to the SUV images in visual assessment. Objective image quality assessment revealed that V_t (2T4K) images and V_t (Logan) images had 5.0-fold and 5.0-fold higher signal-to-noise ratio (SNR), and 3.6-fold and 4.1-fold higher contrast-to-noise ratio (CNR) compared to SUV images, respectively. In addition, no significant differences in SNR and CNR were observed between V_t(2T4K) images and V_t(Logan) images.
**Conclusion:** Total-body parametric imaging of 68Ga-FAPI-04 yielded superior quantification beyond SUV, which may serve as a promising imaging method for early lesion detection and therapeutic response assessment.
**Limitations:** The influence of V_t images on patients' clinical diagnosis and treatment decisions will be further investigated in the ongoing project.
**Ethics committee approval:** This study was approved by the ethics committee of Renji Hospital.
**Funding for this study:** No funding was received for this study.
**Author Disclosures:**
Xinlan Yang: Nothing to disclose
Yee Ling Ng: Nothing to disclose
Gang Huang: Nothing to disclose
Yun Zhou: Nothing to disclose
Yumei Chen: Nothing to disclose
Jianjun Liu: Nothing to disclose
Chaojie Zheng: Nothing to disclose
Ruohua Chen: Nothing to disclose
Xiaofeng Yu: Nothing to disclose

### RPS 2116-6

**Assessing response to therapy in patients with bone metastases from breast cancer using the apparent diffusion coefficient**

*F. Zugni*, A. Colombo, R. Maggioni, G. Petralia; Milan/IT
*(fabio.zugni@ieo.it)*

**Purpose:** To evaluate the potential of the apparent diffusion coefficient (ADC) value as a marker of response to therapy in patients with bone metastases from breast cancer (MBCa).
**Methods or Backround:** This retrospective study examined MBCa patients who underwent Whole-Body Diffusion MRI at 1.5 Tesla during first-line therapy. Up to five bone metastases per patient were identified for ADC measurement at baseline and after therapy. Changes in morphologic images (RECIST 1.1 criteria) were used as a per-patient reference standard for defining disease response/stability/progression, integrated in the absence of measurable disease by symptoms and tumour markers increase/decrease. We used paired and unpaired t-tests to evaluate the performance of ADC for differentiating disease response from non-response (either stability or progression), and used J-index to establish ADC cut-off values. In clinical practise, patients receive the same treatment as long as either stability or response of disease are observed (non-progression). Therefore, we also evaluated the performance of ADC for differentiating disease non-progression from progression.
**Results or Findings:** Eighty bone metastases from 22 patients were analysed. After therapy, ADC values and percentage ADC increase were higher in patients with disease response ($p<0.009$ and $p<0.009$). The cut-offs for identifying response were post-therapy ADC >1180 µm2/s and percentage ADC increase >49% compared to baseline (SE/SP: 80/67% and 80/100%, respectively). ADC values and percentage ADC increase were also higher in patients with non-progression ($p<0.001$ and $p=0.019$), with cut-offs for identifying non-progression of 1180 µm2/s and of percentage ADC increase >16%.
**Conclusion:** Measuring ADC changes of bone metastases after therapy allowed to identify patients with disease response and those with non-progression. Further research is encouraged for validating ADC as a marker of response in patients with bone-metastatic breast cancer.
**Limitations:** No funding was received for this study.
**Ethics committee approval:** No information provided by the submitter.
**Funding for this study:** None
**Author Disclosures:**
Roberta Maggioni: Nothing to disclose
Fabio Zugni: Nothing to disclose
Giuseppe Petralia: Nothing to disclose
Alberto Colombo: Nothing to disclose

### RPS 2116-7

**Implementation of a novel mini-invasive electromagnetic device for real-time tracking of prostate motion in extreme hypofractionated radiotherapy treatments**

*V. Pisoni*, D. Panizza, R. Lucchini, V. Faccenda, M. Daniotti, P. Caricato, S. Trivellato, S. Arcangeli; Monza/IT

**Purpose:** The aim of this study is to describe a novel mini-invasive electromagnetic (EM) device for real-time tracking of prostate motion (PM) in stereotactic body radiation therapy (SBRT) implemented in our department.
**Methods or Backround:** Thirteen prostate cancer patients underwent SBRT in 4 or 5 fractions with the EM device, consisting of a three-way catheter with an integrated transmitter. The planning target volume (PTV) was defined with an isotropic expansion of 2 mm from the prostate gland and seminal vesicles. Treatments were planned with a 10 MV volumetric modulated arc technique (VMAT) without a flattening filter (FFF). The patient's positioning and preparation were verified using cone-beam computed tomography (CBCT) images; the EM device was used throughout the duration of the treatment to verify and correct displacements greater than 2 mm.
**Results or Findings:** The PM never exceeded the established threshold in 31 of the 56 monitored fractions (55%). There were more than 2 mm movements in the remaining sessions, but only ten occurred during treatment delivery and required beam interruption. The average session duration was 10.2 minutes [5.5-22.7]; without the online tracking would have been reduced to 6.7 minutes, but the prostate would have been out of tolerance in 14% of the total delivery time, potentially resulting in the target missing and OAR damages.
**Conclusion:** The EM device resulted in a reliable system for monitoring PM in SBRT, with reduced incidence on session duration and excellent patient tolerability. These results also led to further dose escalation, allowing RT to be delivered to selected patients in a single session.
**Limitations:** Limitations for the study are: no rotational correction, manual beam on/off command, EM system calibrated only on one Linac and no backup strategy in case of machine breakdowns.
**Ethics committee approval:** No information provided by the submitter.
**Funding for this study:** Absent
**Author Disclosures:**
Raffaella Lucchini: Nothing to disclose
Martina Daniotti: Nothing to disclose
Sara Trivellato: Nothing to disclose
Paolo Caricato: Nothing to disclose
Stefano Arcangeli: Nothing to disclose
Valerio Pisoni: Nothing to disclose
Denis Panizza: Nothing to disclose
Valeria Faccenda: Nothing to disclose

**RPS 2116-8**

**Does Deauville score differ between standard-timepoint and delayed-timepoint scans on total-body 18F-FDG PET/CT scans?**

Y. G. Abdelhafez[1], A. Calabrò[2], T. Othman[1], J. Tuscano[1], L. Nardo[1], *F. Sen*[1]; [1]Sacramento, CA/US, [2]Brescia/IT
*(fsen@ucdavis.edu)*

**Purpose:** Deauville score (DS) relies on comparing lesion uptake to liver and blood pool. However, both tissues demonstrate tracer washout over time, which may lead to over-estimation of DSs. Furthermore, it is not known which lymph node group(s) or organ(s) would be more likely influenced by delayed imaging. We aim to evaluate DS changes for 18F-FDG total-body [TB]-PET/CT scans acquired at 2-hr compared to 1-hr post-injection.
**Methods or Backround:** Fifty-three 18F-FDG TB-PET/CT studies were obtained in 45 patients (58.9±16.2 yrs; 24 females) with lymphoma (HL=7; NHL=38). All the studies were acquired as dual-timepoint (TP) total-body scans at 1hr and 2hrs. Scans were reviewed by one radiologist and DSs were recorded for 1hr-TP and 2hr-TP for each site of pre-determined 28 sites.
**Results or Findings:** On study-basis (n=53), the average DSs were 4.43±0.15 and 4.47±0.15 (P=0.4) for 1hr-TP and 2hr-TP, respectively. On site-basis (n=1484), DSs were significantly higher for 2hr-TP compared to 1hr-TP (respectively, 1.57±1.3 vs. 1.55±1.3; P<0.001). DSs were identical for 1447 sites (kappa=0.93; 95%CI=0.91-0.95). While DSs were downstaged for 3 sites for 2hr-TP, 34 sites were upstaged: one site (spleen) from DS-1 to DS-4, 12 sites from DS-3 to DS-4 (spleen=6; bone marrow=2; nodes=3), and 21 sites from DS-4 to DS-5 (spleen=1; nodes=20).
**Conclusion:** DSs assessed on delayed 2-hr 18F-FDG TB-PET/CT scans were not significantly higher from standard 1-hr scans. However, 2.3% of sites may show a clinically meaningful change from DS≤3 to DS-4 (0.9%) or increased intensity from DS-4 to DS-5 (1.4%). Among different sites, spleen seems to be particularly susceptible to score changes. The overall direction of change needs further validation against solid reference standard (e.g., histopathology) and the prognostic implication is currently under investigation.
**Limitations:** Small cohort; lacking validation.
**Ethics committee approval:** No information provided by the submitter.
**Funding for this study:** US National Institutes of Health R01-CA249422;R01-CA206187.
**Author Disclosures:**
Fatma Sen: Nothing to disclose
Anna Calabrò: Nothing to disclose
Joseph Tuscano: Nothing to disclose
Tamer Othman: Nothing to disclose
Lorenzo Nardo: Research/Grant Support: NIH Investigator: United Imaging Healthcare
Yasser Gaber Abdelhafez: Nothing to disclose

**RPS 2116-9**

**Liver volumetry improves evaluation of treatment response to hepatic artery infusion chemotherapy compared to RECIST 1.1 criteria in uveal melanoma patients with liver metastases**

*S. Zensen*, G. L. Alatzides, H. Steinberg, A. Milosevic, J. M. Ludwig, J. T. Siveke, J. Theysohn, M. Forsting, B. M. Schaarschmidt; Essen/DE

**Purpose:** For local tumour control, liver metastases can be treated in uveal melanoma (UM) patients by hepatic artery infusion chemotherapy (HAIC). Due to the diffuse metastatic spread, short-term evaluation using the Response Evaluation Criteria in Solid Tumours (RECIST) 1.1 criteria is often challenging. Diffuse organ involvement often leads to liver enlargement, which can be easily detected by liver volumetry (LV). This study aims to compare RECIST 1.1 and LV for the evaluation of HAIC treatment response in UM patients with liver metastases.
**Methods or Backround:** In this observational retrospective cohort study, treatment response after first HAIC was evaluated in 143 patients (mean age 65.1±10.9 years, 54% female) treated by HAIC between 10/2013 and 12/2020 by RECIST 1.1 and LV on CT imaging performed before and after first HAIC using syngo.via (Siemens Healthineers, Germany). Different increases in liver volume were analysed to set a threshold to distinguish between stable disease (SD) and progressive disease (PD) in LV. Overall survival (OS) was calculated as the time from first HAIC to patient death and survival curves were compared using Kaplan-Meier test and log-rank test.
**Results or Findings:** In the overall population, median OS (mOS) was 13.5 months (95% CI 11.2-15.8months). In LV, a threshold of 10% increase in liver volume is suited to identify patients with significantly reduced OS (SD: 103/143patients, mOS 15.9months; PD: 40/143patients, 6.6months; p<0.001). Response to treatment can also be well evaluated using RECIST 1.1 (SD: 121/143patients, 14.6months; PD: 22/143patients, 8.5months; p=0.0023).
**Conclusion:** In UM patients with liver metastases, LV with a threshold for liver volume increase of 10% is effective to evaluate treatment failure and can be used as an add-on or even alternative to RECIST 1.1.
**Limitations:** Single-centre and retrospective study design.
**Ethics committee approval:** University of Duisburg-Essen.
**Funding for this study:** Not applicable
**Author Disclosures:**
Michael Forsting: Nothing to disclose
Jens Theysohn: Nothing to disclose
Georgios Luca Alatzides: Nothing to disclose
Benedikt Michael Schaarschmidt: Nothing to disclose
Johannes Maximilian Ludwig: Nothing to disclose
Hannah Steinberg: Nothing to disclose
Sebastian Zensen: Nothing to disclose
Jens T. Siveke: Nothing to disclose
Aleksandar Milosevic: Nothing to disclose

**RPS 2116-10**

**Impact of low skeletal muscle mass and quality on clinical outcomes in patients with head and neck cancer undergoing (chemo)radiation**

*G. Besutti*[1], E. Bonelli[2], F. Monelli[1], R. Farì[2], P. Ciammella[2], L. Braglia[2], M. Pellegrini[1], P. Pattacini[2]; [1]Modena/IT, [2]Reggio Emilia/IT
*(giulia.besutti@ausl.re.it)*

**Purpose:** The study aimed to explore the impact of low skeletal muscle mass and quality on survival outcomes and treatment tolerance in patients undergoing radical chemo-radiation therapy for head and neck cancer (HNC).
**Methods or Backround:** Pre-treatment CT scans of HNC patients undergoing (chemo)radiation therapy were retrospectively reviewed to obtain the L3-skeletal muscle index (L3-SMI), and density (L3-SMD) and intramuscular adipose tissue area (L3-IMAT). Low SMD and low SMI were defined according to previously reported thresholds, while high IMAT was defined using population-specific cut-point analysis. SMI, SMD and IMAT were also measured at the proximal thigh (PT) level and tested as continuous variables. Body composition parameters were evaluated in multivariate analyses for overall, progression-free, and cancer-specific survivals (OS, PFS, CSS), adjusted by age, sex, stage, diabetes, body mass index (BMI), and weight loss.
**Results or Findings:** 225 HNC patients were included. In multivariate analyses, L3-SMI was not significantly associated with survival, while poor muscle quality was negatively associated with all survivals (for OS: HR=1.88, 95%CI=1.09-3.23, p=0.022 and HR=2.04, 95%CI=1.27-3.27, p=0.003, for low L3-SMD and high L3-IMAT, respectively). Indices at the PT level, tested as continuous variables, showed that increasing PT-SMI and PT-SMD were significant protective factors for all survival outcomes (for OS: HR for one cm2/m2 increase in PT-SMI 0.96; 95%CI=0.94-0.98; p=0.001 and HR for one HU increase in PT-SMD 0.90; 95%CI=0.85-0.94; p<0.001, respectively).
**Conclusion:** Pre-treatment low muscle quality is a strong prognostic indicator of death risk in patients affected by HNC and undergoing (chemo)radiotherapy with curative intent.
**Limitations:** Retrospective design, no validated cut-off for all variables, PT indices available only in a subset of patients (n=130).
**Ethics committee approval:** This study was approved by COMITATO ETICO AREA VASTA EMILIA NORD.
**Funding for this study:** No funding was received for this study.
**Author Disclosures:**
Pierpaolo Pattacini: Nothing to disclose
Massimo Pellegrini: Nothing to disclose
Filippo Monelli: Nothing to disclose
Patrizia Ciammella: Nothing to disclose
Roberto Farì: Nothing to disclose
Efrem Bonelli: Nothing to disclose
Luca Braglia: Nothing to disclose
Giulia Besutti: Nothing to disclose

**RPS 2116-11**

**Visceral adiposity and sarcopenic obesity associates with impaired 3-month survival in frail older adults with cancer**

*A. Tolonen*, K. Lehtomäki, H. Kerminen, M. Bärlund, P. Österlund, O. Arponen; Tampere/FI

**Purpose:** High amount of visceral adipose tissue (VAT), assessed with computed tomography (CT), has been associated with inferior survival in adults with cancer. We retrospectively examined the association between high VAT index (VATI), sarcopenic obesity, and 3-month overall survival (OS) in patients ≥75 years with solid tumours at frailty risk (G8 screen ≤14/17).
**Methods or Backround:** Optimal VATI (=VAT area/patient's height^2) cut-offs for 3-month OS (69.3cm2/m2 for men and 61.4cm2/m2 for women) were obtained using receiver operator characteristics. Sarcopenic obesity was defined as low muscle mass (as in Tolonen et al. ECR 2022: #21291) combined with high body mass index (BMI) >25 or high VATI. Kaplan-Meier survival estimates, and Cox regression models were analysed.
**Results or Findings:** VAT values were determined for 37 females and 42 males (median age 80 years). 3-month OS-rates for high/low VATI were 58%/88% in the non-curative group (n=58; HR 4.3 [95% CI 1.3-14]) with median OS of 5.9 versus 9.8 months (HR 1.40 [0.8-2.5]). In univariate analysis, 3-month OS associated with combined low muscle mass and BMI>25 (3.81 [1.3-11]) or high VATI (HR 5.53 [1.8-17]), but BMI alone did not. In multivariable analysis, low muscle mass with high VATI remained significant

Saturday

(HR 8.91 [1.7-46]), when adjusted for sex (1.59 [0.3-8.3]), poor ECOG performance status (2.06 [0.6-7.5]), and frailty on Clinical Frailty Scale (2.66 [0.7-10]). No survival were found in the curative treatment group (n=21).
**Conclusion:** CT-determined high VAT combined with low muscle mass as in sarcopenic obesity are associated of impaired 3-month OS in non-curative patients and could thus guide treatment decisions.
**Limitations:** Small cohort.
**Ethics committee approval:** The study was approved by the local institutional review board at Tampere University Hospital (study numbers: R19628S and R20503S). Ethics Committee approval and written informed consent are not needed in single-institution register-based studies in Finland.
**Funding for this study:** Tampere University Hospital.
**Author Disclosures:**
Maarit Bärlund: Nothing to disclose
Otso Arponen: Nothing to disclose
Pia Österlund: Nothing to disclose
Hanna Kerminen: Nothing to disclose
Kaisa Lehtomäki: Nothing to disclose
Antti Tolonen: Nothing to disclose

**16:00-17:30** **Research Stage 4**

### Research Presentation Session: Cardiac

### RPS 2103

### Cardiac MRI: tissue characterisation

**Moderator**
I. Žuža; Rijeka/HR

### RPS 2103-2

**Reference values for 3-Tesla magnetic resonance imaging derived native myocardial T1 for Chinese children**
*W. Bai*, Y-K. Guo, H. Xu; Chengdu/CN

**Purpose:** The purpose of this study was to establish the percentile reference values for native T1 of left ventricle (LV) myocardium in healthy paediatric subjects for 3T MRI.
**Methods or Backround:** 174 healthy Chinese children (89 boys; age 6–18 years old) were prospectively recruited. T1 mapping images were acquired in three LV short axis slices using a modified Look-Locker inversion recovery (MOLLI) sequence from 3T MRI. The LV myocardial native T1 were measured using the American Heart Association 16-segment model and compared regionally. The percentile reference graphs of regional LV myocardium were generated for the age span with the lambda-mu-sigma method.
**Results or Findings:** The global native T1 value of LV myocardium was 1237.11 ± 33.61 ms. The native T1 of septal myocardium (1260.03 ± 36.15 ms) was significantly higher than that of the anterior (1228.83 ± 36.76 ms, p < 0.001), posterior (1226.10 ± 37.69 ms, p < 0.001), lateral (1221.61 ± 36.58 ms, p < 0.001) myocardium. Native T1 was negatively correlated with age (septal myocardium: r = -0.33, p < 0.001). The age-related percentile charts revealed that native T1 showed a downward trend with age, which decreased significantly before late puberty, and relatively steady in late puberty.
**Conclusion:** This study provided the native T1 reference ranges of LV myocardium in healthy Chinese children using the MOLLI acquisition with a 3T MRI and found that native T1 showed a downward trend with age but steady in late puberty.
**Limitations:** Children under 6 years of age were not included because they often require sedation or general anaesthesia to undergo the MRI examination and cannot hold their breath.
**Ethics committee approval:** Ethics Committee of West China Second University Hospital of Sichuan University (No.2022-105).
**Funding for this study:** This work was supported by the National Natural Science Foundation of China (No. 82120108015, 82271981).
**Author Disclosures:**
Huayan Xu: Nothing to disclose
Ying-Kun Guo: Nothing to disclose
Wei Bai: Nothing to disclose

### RPS 2103-3

**Cardiac magnetic resonance imaging normal values in the New-Zeeland white rabbit animal model**
*U. Reiter*, C. Reiter, C. Kräuter, Y. Tehlivets, L. Barones, V. Bubalo, M. Fuchsjäger, O. Tehlivets, G. Reiter; Graz/AT
*(ursula.reiter@medunigraz.at)*

**Purpose:** Rabbits are well recognised research models of human cardiovascular diseases. Cardiac magnetic resonance (CMR) normal data are, however, lacking. Aim of the study was to provide CMR normal values in New-Zeeland white rabbits using clinical routine equipment.
**Methods or Backround:** Twenty-five sedated male New-Zeeland white rabbits (body weight, 3.56±0.23 kg) underwent free-breathing, pulse-gated, contrast-enhanced CMR using a clinical 3T MR scanner and a 15-channel knee coil. The CMR protocol included cine imaging, pre- and post-contrast T1-mapping, T2-mapping and late-enhancement imaging. Body-surface area (BSA) was calculated from Meeh's formula. Left ventricular (LV), right ventricular (RV) function parameters, feature-tracking derived global circumferential (GCS) and longitudinal (GLS) strains, as well as inline calculated relaxation-time maps were evaluated by routine CMR software. Blood-haematocrit was measured prior to imaging to enable calculation of myocardial extracellular volume fraction (ECV). Means are given together with standard deviations; relationships between parameters were investigated by correlation analysis.
**Results or Findings:** Rabbits' heart rate was 153±21 beats/minute. No late-enhancement patterns were found, excluding unknown pericardial disease and/or focal myocardial fibrosis. LV and RV ejection fractions (EF) were 52±5% and 61±5%, respectively. Ventricular masses correlated significantly with BSA; correspondingly, BSA-normalized LV and RV masses were 13.7±1.3 g/m² and 5.9±1.0 g/m². LVGLS (-16±3%) correlated with LVEF (r=-0.60); LVGCS was -16±2%. RVGLS and RVGCS were -22±5% and -12±3%, respectively. Global LV myocardial T1 and T2 times were 1384±45 ms and 39±3 ms; calculated ECV was 32±3%.
**Conclusion:** CMR imaging of the rabbit model is feasible with clinical routine MR equipment. Functional and morphological reference values are given for male New-Zeeland white rabbits.
**Limitations:** Only male rabbits were studied. Small sample size.
**Ethics committee approval:** Austrian Federal Ministry of Education, Science and Research (TVG-GZ: 2021-0.370.442).
**Funding for this study:** FWF, Fonds zur Förderung der Wissenschaftlichen Forschung, Austria (GrantNr. P33672-B). OeNB Anniversary Fund (Grant No. 17934)
**Author Disclosures:**
Ursula Reiter: Nothing to disclose
Gert Reiter: Employee: Siemens Healthcare Diagnostics GmbH
Michael Fuchsjäger: Nothing to disclose
Oksana Tehlivets: Nothing to disclose
Vladimir Bubalo: Nothing to disclose
Clemens Reiter: Nothing to disclose
Lisa Barones: Nothing to disclose
Yuriy Tehlivets: Nothing to disclose
Corina Kräuter: Nothing to disclose

### RPS 2103-4

**Cardiac adaptation in competitive male and female rowers and characterisation of non-ischemic myocardial fibrosis**
*P. Harms*, A. Kisters, H. Chen, J. Erley, E. Cavus, G. K. Lund, K. Muellerleile, G. Adam, E. Tahir; Hamburg/DE
*(ph.harms@uke.de)*

**Purpose:** To study the cardiac adaptations in competitive male and female rowers and the morphology of non-ischemic myocardial fibrosis by cardiac MRI.
**Methods or Backround:** Nineteen male (33±13 years) and nine female rowers (30±10 years), and 20 sedentary controls underwent 3 Tesla CMR (Philips, Ingenia) including late gadolinium enhancement (LGE) imaging and T1 mapping. Subjects were recruited from local sports clubs. CMR data were analysed using CVi42 (Circle Cardiovascular Imaging).
**Results or Findings:** Male rowers had an increased left ventricular (LV) mass (68±12 vs. 54±8 g/m², P<0.05) and LV volume (105±14 vs. 75±12 ml/m², P<0.001) compared to controls, but LV ejection fraction (EF) was similar (P=0.764). Female rowers also had an increased LV mass (59±6 vs. 49±6 g/m², P<0.01) and higher LV volume (97±6 vs. 79±8 ml/m², P<0.0001) compared to controls, but LVEF was similar (P=0.528). Male (113±12 vs. 76±16 ml/m², P<0.0001) and female rowers (101±16 vs. 82±24 ml/m², P<0.05) had an increased right ventricular (RV) volume compared to controls. RVEF was similar between rowers and controls. Septal T1 relaxation times were similar between male (1207±24 ms, P=0.752) and female rowers (1248±27 ms, P=0.447) and controls. Focal non-ischemic myocardial fibrosis was detected in four male (21%), but in none of the female rowers and in age- and gender-matched controls. LGE+ athletes 1 and 2 had focal fibrosis of the basal inferoseptal LV wall at the right ventricular insertion point, LGE+ athletes 3 and 4 at the basal inferolateral wall.

**Conclusion:** In our small cohort of competitive male and female rowers the incidence of focal myocardial fibrosis was 14%. Both athlete groups showed signs of ventricular adaptation to endurance training compared to age- and gender-matched controls.
**Limitations:** A small sample size and self-reported training frequency represent a potential limitation.
**Ethics committee approval:** No information provided by the submitter.
**Funding for this study:** No funding was received for this study.
**Author Disclosures:**
Ersin Cavus: Nothing to disclose
Gunnar K Lund: Nothing to disclose
Phillip Harms: Nothing to disclose
Jennifer Erley: Nothing to disclose
Gerhard Adam: Nothing to disclose
Kai Muellerleile: Nothing to disclose
Anna Kisters: Nothing to disclose
Hang Chen: Nothing to disclose
Enver Tahir: Nothing to disclose

### RPS 2103-5

**Focal myocardial fibrosis in competitive male and female swimmers: presence, localisation and extent**

*A. S. Kisters*, P. Harms, H. Chen, J. Erley, M. K. Świderska, K. Müllerleile, G. Adam, E. Tahir; Hamburg/DE
*(annasophiekisters@gmail.com)*

**Purpose:** Myocardial fibrosis occurs in a variable frequency in asymptomatic athletes detected by late gadolinium enhancement (LGE) cardiac MRI (CMR). The purpose of this study was to examine and analyse the prevalence, localisation and extent of myocardial fibrosis in competitive swimmers.
**Methods or Backround:** Twelve female (35±8 years) and nineteen male swimmers (35±10 years) and 20 sedentary control subjects underwent 3 Tesla CMR, including late gadolinium enhancement (LGE) imaging. Subjects were recruited from local sports clubs. CMR data were analysed using CVi42 (Circle Cardiovascular Imaging). CMR parameters, normalised to body surface area, are given as the mean of two independent observers.
**Results or Findings:** Male swimmers had a higher left ventricular (LV) mass (69±16 vs. 54±8 g/m², P=0.031) and LV volume (96±13 vs. 75±12 ml/m², P=0.009) compared to controls. Also female swimmers had a similar LV mass (55±8 vs. 49±6 g/m², P=0.103) and higher LV volume (95±9 vs. 79±8 ml/m², P=0.004). Focal non-ischemic myocardial fibrosis was detected in five female (42%) and six male swimmers (32%). Four out of eleven LGE+ athletes (36%) demonstrated focal fibrosis of the basal inferolateral wall. No focal myocardial fibrosis was detected in age- and gender-matched controls.
**Conclusion:** In our small cohort of competitive male and female swimmers the incidence of focal myocardial fibrosis was 35%. Both athlete groups showed signs of ventricular adaptation to endurance training compared to age- and gender-matched controls. Focal non-ischemic myocardial fibrosis was present in the anterior, anteroseptal, inferior and inferolateral LV wall.
**Limitations:** Small number of study participants. The self-reported race time and race distances represent a potential limitation. Furthermore we cannot exclude a history of illicit drug use.
**Ethics committee approval:** No information provided by the submitter.
**Funding for this study:** Not applicable.
**Author Disclosures:**
Anna Sophie Kisters: Nothing to disclose
Kai Müllerleile: Nothing to disclose
Phillip Harms: Nothing to disclose
Jennifer Erley: Nothing to disclose
Gerhard Adam: Nothing to disclose
Hang Chen: Nothing to disclose
Enver Tahir: Nothing to disclose
Monika Katarzyna Świderska: Nothing to disclose

### RPS 2103-6

**Late gadolinium enhancement dispersion mapping for predicting arrhythmic events in patient with dilated cardiomyopathy**

*G. D. Aquaro*[1], C. De Gori[1], M. L. Parisella[2], L. Faggioni[1], L. Restivo[3], A. De Luca[3], G. Aringhieri[1], D. Cioni[1], E. Neri[1]; [1]Pisa/IT, [2]Fondi/IT, [3]Trieste/IT
*(aquarogd@gmail.com)*

**Purpose:** In non-ischemic dilated cardiomyopathy (DCM) prediction of malignant ventricular arrhythmias is very challenging. The late gadolinium enhancement (LGE) dispersion mapping is a radiomics method for the quantification of tissue heterogeneity through the Global Dispersion Score (GDS). The aim of this study is the prognostic role of GDS in DCM patients.
**Methods or Backround:** In this multicentre study 510 consecutive patients with non-ischemic DCM were enrolled. All patients underwent cardiac magnetic resonance imaging. LGE images were retrospectively analysed by measurement of GDS. A combined endpoint of sudden cardiac death, cardiac arrest and appropriate implantable cardioverter-defibrillator intervention was considered.
**Results or Findings:** Mean left ventricular ejection fraction (LVEF) was 35±11%. LVEF was >35% in 241 patients (47%). LGE was present in 225 patients (45%). Median extent of LGE was 12% of LV mass (interquartile range -IQR- 6-20%). Among patients with positive LGE, GDS was 0.14 (IQR 0.08-0.20). The whole population was divided in 3 groups: GDS=0, GDS>0 and ≤0.10 and GDS>0.10. During follow-up 81 patients had major events (8 SCD, 73 appropriate ICD interventions). At Kaplan-Meier analysis, patients with GDS>0.10 had worse prognosis than those with lower values of GDS (p<0.0001). At multivariate analysis, GDS>0.10 (HR 2.9, 95% CI 1.7-5, p=0.0002) was an independent predictor of events. Subgroup analysis confirmed the prognostic value of GDS regardless of LVEF > or ≤35% and LGE extent.
**Conclusion:** GDS is a useful marker to identify DCM patients at higher risk for major arrhythmic events regardless LVEF and extent of LGE.
**Limitations:** LGE dispersion mapping was evaluated with 2D approach.
**Ethics committee approval:** Ethical Committee of Pisa.
**Funding for this study:** Institutional funds of University of Pisa, Fondazione G. Monasterio, University of Trieste.
**Author Disclosures:**
Maria Luisa Parisella: Nothing to disclose
Antonio De Luca: Nothing to disclose
Emanuele Neri: Nothing to disclose
Luca Restivo: Nothing to disclose
Giacomo Aringhieri: Nothing to disclose
Carmelo De Gori: Nothing to disclose
Giovanni Donato Aquaro: Nothing to disclose
Lorenzo Faggioni: Nothing to disclose
Dania Cioni: Nothing to disclose

### RPS 2103-7

**Development and validation of an MRI-based nomogram for predicting adverse outcomes in non-ischaemic dilated cardiomyopathy**

*X. Xiang*[1], X. Lin[2], K. Zhao[1], S. Yu[1], X. Chen[1], S. Zhao[1]; [1]Beijing/CN, [2]Fuzhou/CN
*(xiangxiaorui@live.com)*

**Purpose:** Effective and specific risk evaluation for patients with non-ischaemic dilated cardiomyopathy (NIDCM) remains challenging. Thus, this study aimed to construct a novel MRI-based nomogram to predict outcomes in NIDCM.
**Methods or Backround:** A total of 335 consecutive NIDCM patients who underwent cardiac MRI were retrospectively enrolled. Comprehensive clinical evaluation and imaging investigation were obtained, including measurements of late gadolinium enhancement (LGE) and feature tracking (FT) strain. All patients were followed up for a composite endpoint of major adverse cardiac events (MACE) including all-cause mortality and heart transplantation. These patients were randomly divided into training and validation cohort (7:3). LASSO regression analysis were utilised to identify prognostic factors. A nomogram based on the selected predictive variables was built to predict MACE.
**Results or Findings:** MACE occurred in 93 out of 234 patients in the training cohort, and in 41 out of 101 patients in the validation cohort. Six variables including NYHA class, NT-proBNP, Beta-blocker medication, LGE presence, and LV global longitudinal strain (GLS) were found to be significantly associated with MACE and were used for constructing the nomogram. The nomogram achieved good discrimination with C-indexes of 0.80 and 0.82 in the training and validation cohorts, respectively. The calibration curve for 1-, 3-, and 5-year survival also showed high coherence between the predicted and actual probability of MACE. Decision curve analysis identified the model was clinically useful in predicting MACE.
**Conclusion:** This study presents a predictive model that incorporates MRI parameters and clinical risk factors, which can be conveniently used to facilitate the risk stratification in NIDCM.
**Limitations:** This is a retrospective, single-centre study and have some inevitable biases. This study lacks available data to consider the genetic signatures. And the real-world benefit of our model needs further verification.
**Ethics committee approval:** Ethical approval was obtained by the hospital’s Institutional Review Board.
**Funding for this study:** National Natural Science Foundation of China(No.81930044).
**Author Disclosures:**
Shiqin Yu: Nothing to disclose
Xiuyu Chen: Nothing to disclose
Xiaorui Xiang: Nothing to disclose
Shihua Zhao: Nothing to disclose
Kankan Zhao: Nothing to disclose
Xiaoqiang Lin: Nothing to disclose

**RPS 2103-8**

**Extracellular volume fraction as an MRI biomarker of cancer therapy cardiotoxicity: a systematic review and meta-analysis**

*G. Folco*, C. B. Monti, D. Capra, M. Zanardo, F. Silletta, F. Secchi, F. Sardanelli; Milan/IT
*(gianluca.folco@unimi.it)*

**Purpose:** To investigate the studies exploring the role of cardiac MRI-derived extracellular volume fraction (ECV) as a biomarker of cancer-therapy related cardiotoxicity.
**Methods or Backround:** In April 2022, we performed a systematic search on EMBASE and PubMed for articles on the use of MRI-derived ECV as a cancer therapy-related cardiotoxicity biomarker. Two blinded researchers performed screening of the retrieved articles, including only those reporting ECV values at least 3 months after cardiotoxic cancer treatment. Data extraction was performed for each article, including clinical and technical data, and ECV values. Pooled ECV was calculated using the random effects model and compared among different treatment regimens and among those who did or did not experience overt cardiac dysfunction, via post-hoc analyses. Meta-regression analyses were conducted to appraise which clinical or technical variables yielded a significant impact on ECV. The threshold for statistical significance was set at p≤0.050.
**Results or Findings:** Overall, 19 studies were included. Study population ranged from 9 to 236 patients, for a total of 1123 individuals. The average age of patients ranged from 12.5 to 74 years. Most studies included patients with breast and esophagus cancer, treated with anthracyclines and chest radiotherapy. Pooled ECV was 28.44% (95% confidence interval, CI, 26.85–30.03%) among subjects who had undergone cardiotoxic cancer treatments, versus 25.23% (95%CI 23.31–27.14%) among those who had not (p=0.003). Only magnetic field strength (p=0.006) and the sequence used for T1 mapping (p=0.026) yielded a significant impact on ECV.
**Conclusion:** A higher ECV in patients who underwent cardiotoxic treatment could reflect subclinical changes in the myocardial structure associated to cancer treatment, proposing ECV as an early biomarker of cardiotoxicity.
**Limitations:** Heterogeneity concerning both clinical and technical aspects of the included works.
**Ethics committee approval:** No information provided by the submitter.
**Funding for this study:** No funding was received for this study.
**Author Disclosures:**
Francesco Secchi: Nothing to disclose
Francesco Sardanelli: Advisory Board: Bayer Research/Grant Support: Bracco
Caterina Beatrice Monti: Nothing to disclose
Gianluca Folco: Nothing to disclose
Moreno Zanardo: Nothing to disclose
Francesco Silletta: Nothing to disclose
Davide Capra: Nothing to disclose

**RPS 2103-9**

**T1, T2, and ECV mapping derived from a single-slice measurements is often non-inferior compared to mean of mutiple views in diagnosing myocarditis: insights from the MyoRacer Trial**

*C. Lücke*[1], C. Wirsing[2], W. Rutschke[1], B. Foldyna[3], P. Seitz[1], R. F. Gohmann[1], K. Klingel[3], P. Lurz[1], M. Gutberlet[1]; [1]Leipzig/DE, [2]Ludwigsburg/DE, [3]Tübingen/DE
*(cluecke@gmx.de)*

**Purpose:** Mapping aids to diagnose myocarditis on cardiac MRI. First aim: to compare the diagnostic performance of single slice T1, ECV, and T2 mapping vs. mean, performed in two-chamber-(2CH), four-chamber-(4CH) views and midventricular short-axis-orientation (SA). Second aim: to find the slice orientation with the best diagnostic performance compared to endomyocardial biopsy (EMB).
**Methods or Backround:** This study is a sub study of the prospective MyoRacer trial (Lurz et al. 2016) with imaging parameters as published. Mapping was acquired as single slices in 2CH, 4CH and SA orientations and measured on adedicated cMR-workstation. The readers were blinded to the biopsy results. We compared the discriminative capacity of individual single-slice-mapping with the means of multi-slice measures using ROC analysis and biopsy as reference. Diagnostic performance was examined by using ROC-analysis, AUCs were compared.
**Results or Findings:** Among 88 included patients (20 (32%) women; 43±12y), 62 (70,4%) were EMB positive for myocarditis. AUC for T2 mapping were 0.696 for the mean, 0.693 for SA, 0.671 for 2CH, and 0.62 for 4CH and did not differ significantly p>0.125. AUC for T1 mapping were 0.667 for the mean, 0.667 for 4CH, 0.662 for the 2CH, and 0.583 for the SA orientation. The difference between mean and SA was statistically significant p=0.0256 (other differences p> 0.19). AUC for ECV calculation were 0.667 for the mean, 0.635 for SA, 0.679 for 2CH, and 0.629 for 4CH, not statistically significant (p>0.18).
**Conclusion:** Although the mean of three measurements shows the best diagnostic performance, even a single slice of native T1 or T2 Mapping or ECV-analysis delivers similar diagnostic accuracy in patients with suspected myocarditis. Long axis T1 mapping can however outperform a single slice T1 mapping in midventricular short axis orientation.
**Limitations:** Selection bias.
**Ethics committee approval:** No information provided by the submitter.
**Funding for this study:** No funding was received for this study.
**Author Disclosures:**
Karin Klingel: Nothing to disclose
Christian Lücke: Nothing to disclose
Matthias Gutberlet: Nothing to disclose
Patrick Seitz: Nothing to disclose
Borek Foldyna: Nothing to disclose
William Rutschke: Nothing to disclose
Clara Wirsing: Nothing to disclose
Philipp Lurz: Nothing to disclose
Robin F. Gohmann: Nothing to disclose

**RPS 2103-10**

**Erdheim-Chester disease cardiovascular involvement is associated with myocardial fibrosis and atrial dysfunction: a cardiac magnetic resonance (CMR) study.**

*D. Vignale*, A. Palmisano, C. Campochiaro, A. Tomelleri, A. Esposito; Milan/IT
*(vignale.davide@hsr.it)*

**Purpose:** The purpose is to characterise features of cardiovascular involvement in patients with Erdheim-Chester Disease (ECD) and to evaluate their association with myocardial fibrosis and cardiac function.
**Methods or Backround:** Among the 54 patients with biopsy-proven ECD referred to our institution between 2009 and 2021, we retrospectively enrolled 29 patients (59±12 years, 79% males) who underwent 1.5T CMR using a standardised protocol for qualitative and quantitative assessment of ECD localisations, evaluation of atrial and ventricular function, and assessment of myocardial fibrosis. Three patients did not receive contrast media.
**Results or Findings:** Cardiac involvement was present in 22/29 (76%) patients, with right atrioventricular groove as the most commonly affected site (22/22 [100%]; 19 [12–46] mL) followed by right atrial walls (14/22 [63%], 21 [6-38] mL). Involvement of thoracic aorta and superior vena cava was present in 17/29 (59%) and 11/29 (38%) patients, with volumes of 8 [6-9] and 8 [6-22] mL, respectively. Patients with cardiac involvement had slight diffuse edema (T2-ratio 2.1 [2.0-2.2] vs 1.7 [1.6-1.7], p<0.001). Patients with right atrioventricular groove involvement, encasing the right coronary artery, had higher fibrosis burden (3SD scar burden 7 [5-9] vs 1[1-2]%, p=0.003) localised in infero-septal (20/26 [77%] patients) and inferior (14/26 [54%] patients) mid-basal segments, with non-dense non-ischemic appearance except in two patients presenting with dense transmural fibrosis suggesting previous myocardial infarction. Patients with right atrial involvement had atrial dysfunction [ejection fraction 45 [30-60] vs 56 [52-69]%, p=0.03]. Mean biventricular volumes and function did not differ according to ECD localisations.
**Conclusion:** In ECD patients, right atrioventricular groove localisation is associated with downstream myocardial fibrosis, suggesting hemodynamic alterations due to coronary encasement. Atrial pseudomass impacts on contractility and is associated to atrial dysfunction.
**Limitations:** Relative small sample. No follow-up.
**Ethics committee approval:** No information provided by the submitter.
**Funding for this study:** No funding was received for this study.
**Author Disclosures:**
Davide Vignale: Nothing to disclose
Antonio Esposito: Nothing to disclose
Alessandro Tomelleri: Nothing to disclose
Anna Palmisano: Nothing to disclose
Campochiaro Corrado Campochiaro: Nothing to disclose

**RPS 2103-11**

**CMR-based assessment of myocardial injury in asymptomatic people living with human immunodeficiency virus: correlation with nadir CD4 count**

*X. Peng*, T. Liu; Shenyang/CN

**Purpose:** To evaluate the presence of subclinical myocardial injury in asymptomatic People Living with Human Immunodeficiency Virus (PLWH, HIV) by cardiac magnetic resonance (CMR) imaging and to explore the possible association between subclinical myocardial injury and HIV-related clinical characteristics.
**Methods or Backround:** 80 asymptomatic PLWH (age = 53(47-56) years, 90% male) and 50 healthy participants matched according to age and sex were included in this study. The CMR protocol at 3 tesla included cine imaging, native T1, T2, and T2* mapping of the left ventricular. The associations between CMR parameters and HIV-related clinical characteristics, including white cell counts, were evaluated by Spearman rank correlation and logistic regression.
**Results or Findings:** The left ventricular ejection fraction in asymptomatic PLWH was lower than that in healthy control subjects (60.6 ± 3.4% vs. 62.6 ± 3.0%, p = 0.001), but was still within normal range. PLWH revealed significantly higher native myocardial T1 values (1241 ± 29ms vs. 1189 ± 21ms, p < 0.001), native T2 values (40.7 ± 1.5ms vs. 37.9 ± 1.4ms, p < 0.001), but showed no difference in native T2* values (17.3ms [16.3-19.1ms] vs. 18.3ms [16.5-19.3ms], p = 0.201). In multivariable logistic regression analyses, nadir CD4+ T-cell count <500 cells/mm³ was associated with higher odds of elevated native T1 myocardial values (odds ratio, 6.12 [95% confidence interval, 1.07-34.91] p = 0.041) in PLWH.
**Conclusion:** Subclinical myocardial inflammation is present in asymptomatic PLWH. A lower nadir CD4 count is a risk factor for subclinical myocardial injury, which may serve as a biomarker for determining subclinical myocardial injury in PLWH.
**Limitations:** Not applicable.
**Ethics committee approval:** Not applicable.
**Funding for this study:** Not applicable.
**Author Disclosures:**
Xin Peng: Nothing to disclose
Ting Liu: Nothing to disclose

### RPS 2103-12

**Value of a short non-contrast cardiac magnetic resonance protocol compared to standard contrast-enhanced multiparametric cardiac magnetic resonance protocol in patients with MINOCA**

*D. Tore*[1], A. Palmisano[2], R. Cau[3], D. Vignale[2], M. Porcu[3], A. Esposito[2], R. Faletti[1], L. Saba[3], M. Gatti[1]; [1]Turin/IT, [2]Milan/IT, [3]Cagliari/IT

**Purpose:** To compare the diagnostic performance of a short cardiac MRI protocol without contrast media (ShMRI) to a standard comprehensive cardiac MRI protocol (StMRI) in patients with MINOCA.
**Methods or Backround:** Retrospective study on patients with a working diagnosis of MINOCA who were admitted to one of three participating hospitals between 01/2019 and 12/2020 and underwent a StMRI. An expert and a non-expert reader performed a blinded reading with the ShP (long axis cine images, T2w-STIR, T1 and T2 mapping). After at least three months from the ShMRI reading session, expert and non-expert reader in consensus evaluated the full StMRI (reference standard) obtaining the examinations in a random order. For both reading session, readers were asked to report: 1) diagnosis; 2) level of confidence in the diagnosis with the ShP; 3) number of myocardial segments involved and 4) functional parameters.
**Results or Findings:** 179 patients enrolled. The ShP lasted 21 min and the StP 45 min (p<0.0001). The diagnosis agreement with ShP and StP by expert and non-expert readers was 152/179=85% and 129/179=72% patients, respectively (p=0.005) and rose to 101/102 (99%) and 70/79 (87%), respectively (p=0.007) when the diagnostic confidence was good/very good. The ShP overestimated ejection fraction and underestimated cardiac volumes compared with the StP (p<0.01) and underestimated the number of segments involved by pathology (p=0.0008).
**Conclusion:** The ShP was found to be a debatable alternative to the StP in patients with MINOCA, because of insufficient concordance with the diagnosis obtained with the StMRI. Nevertheless, when an experienced reader reach a good or very good diagnostic confidence using the ShP, it may choose to stop the examination, reducing the length of the examination without affecting the final diagnosis.
**Limitations:** Retrospective study.
**Ethics committee approval:** No information provided by the submitter.
**Funding for this study:** No funding was received for this work.
**Author Disclosures:**
Luca Saba: Nothing to disclose
Davide Vignale: Nothing to disclose
Antonio Esposito: Nothing to disclose
Riccardo Cau: Nothing to disclose
Riccardo Faletti: Nothing to disclose
Anna Palmisano: Nothing to disclose
Marco Gatti: Nothing to disclose
Davide Tore: Nothing to disclose
Michele Porcu: Nothing to disclose

### Sunday, March 5

**08:00-09:00** **Research Stage 1**

### Research Presentation Session: Abdominal Viscera & GI Tract

### RPS 2201

### Advances in liver ultrasound

**Moderator**
C. Ewertsen; Copenhagen/DK

### RPS 2201-2

**Depth dependence of liver fat quantification with ultrasound attenuation imaging**

*G. Ferraioli*[1], A. Raimondi[1], A. De Silvestri[1], R. G. Barr[2]; [1]Pavia/IT, [2]Rootstown, OH/US
*(giovanna.ferraioli@unipv.it)*

**Purpose:** To determine whether there is a depth dependence when estimating the attenuation coefficient (AC) in liver
**Methods or Backround:** This retrospective study was performed with the Aplio i800 ultrasound (US) system equipped with the AC algorithm (ATI, Canon Medical Systems, Japan). ATI measurements were taken positioning the region of interest (ROI) upper edge at 2,3,4,5 cm below the liver capsule. The median value of 5 acquisition was used for the analysis. Univariate and multivariate (including sex, age, stiffness, skin-to-liver capsule distance) linear regression models were fitted using ATI value as dependent variable and the depth at which measurements were taken as independent variable.
**Results or Findings:** 63 individuals (34 females, mean age: 51+/-14 years; mean BMI: 28.3+/-4.3 kg/m2; skin-to-liver capsule distance: 2.1+/-0.4cm; liver stiffness: 5.46+/-2.01 kiloPascal) were studied. Steatosis assessed with B-mode US was present in 32 individuals. ATI values showed a decrease of 0.052 dB/cm/MHz per 1cm increase of the depth (coefficient: -0.052; 95% CI -0.062 to -0.042; p<0.001). Similar results were obtained with multivariate model (coefficient -0.049; 95%CI -0.060 to -0.038 p<0.001) and this decrease was significantly affected by the skin-to-liver capsule distance (p<0.001).
**Conclusion:** There is depth dependence in the measurement of the attenuation coefficient obtained in the liver. This can affect results dramatically. A standardised protocol needs to be developed to minimise differences in AC measurements and to reliably assess changes in serial measurements.
**Limitations:** This was a retrospective study.
**Ethics committee approval:** Fondazione IRCCS Policlinico San Matteo, Pavia, Italy
**Funding for this study:** No funding was received for this study.
**Author Disclosures:**
Ambra Raimondi: Nothing to disclose
Annalisa De Silvestri: Nothing to disclose
Giovanna Ferraioli: Speaker: Canon Medical Systems, Fujifilm, Mindray Ultrasound, Philips Ultrasound, Siemens Healthineers
Richard G Barr: Grant Recipient: Siemens Ultrasound, Philips Ultrasound, Mindray Ultrasound, Samsung Ultrasound Speaker: Philips Ultrasound, Mindray Ultrasound

### RPS 2201-3

**Performance of shear wave elastography as a non-invasive method to predict liver fibrosis demonstrated by biopsy**

*C. G. Sotomayor*, M. Marqués, C. Correa, C. Parra Albornoz, J. S. Casas, S. Yévenes Aravena, A. J. Sanhueza, F. Araya, G. Cardenas; Santiago/CL
*(camilosotomayor@uchile.cl)*

**Purpose:** To calculate the sensitivity and specificity of shear wave elastography (SWE) to predict liver fibrosis demonstrated by biopsy
**Methods or Backround:** We performed a cross-sectional study in consecutive adult patients (Clinical Hospital University of Chile, 03/2019–07/2022) with medical indication of liver biopsy, due to non-acute causes of liver disease, who underwent a contemporary (<24-hrs) SWE (Philips EPIC 7G System, ElastPQ technique). SWEs with 10 measurements and an interquartile range–to–median ratio (IQR/M) between consecutive acquisitions <30%, were judged as reliable. Liver stiffness (kPa) was used to estimate the degree of fibrosis (Metavir, F1-F4) according to Bedossa et al., and dichotomised for fibrosis (+/-; F1/F2-F4). The degree of fibrosis (Metavir, F0-F4) by biopsy was likewise dichotomised (+/-; F0-F1/F2-F4). Linear regression was used to determine the association between liver stiffness and the degree of fibrosis by biopsy. We then calculated the sensitivity and specificity of SWE to predict fibrosis (+/-) by biopsy. For statistical analyses (R version 3.2.3, R Foundation for Statistical Computing), a statistical significance level of P<0.05 (two-tailed) was used.
**Results or Findings:** 43 patients (52±14 years-old; 75% female) were studied. Mean liver stiffness was 8.9±3.7 kPa, estimated liver fibrosis was F1, 38%; F2, 31%; F3, 17%; F4, 15%, and liver fibrosis by biopsy was F0, 31%; F1, 21%; F2, 19%; F3, 17%, F4, 13%. Liver stiffness was significantly associated with the degree of fibrosis by biopsy (Std. β, 0.71; P<0.001). Sensitivity and specificity of SWE to predict fibrosis by biopsy was 85 and 76% (IQR/M <30%, n=43), and 93 and 92% (IQR/M <20%, n=29), respectively.
**Conclusion:** SWE is a non-invasive method with excellent sensitivity and specificity to predict liver fibrosis by biopsy when an IQR/M<20% is used as cut-off point to judge its reliability.
**Limitations:** This was a single-centre study.
**Ethics committee approval:** No information provided by the submitter.
**Funding for this study:** No funding was received for this study.
**Author Disclosures:**
Francisca Araya: Nothing to disclose
Mariana Marqués: Nothing to disclose
Juan Salvador Casas: Nothing to disclose
Claudio Correa: Nothing to disclose
Gonzalo Cardenas: Nothing to disclose
Camilo G. Sotomayor: Nothing to disclose
Alvaro Javier Sanhueza: Nothing to disclose
Sebastián Yévenes Aravena: Nothing to disclose
Carla Parra Albornoz: Nothing to disclose

### RPS 2201-4

**Assessment of liver stiffness changes with strain-wave elastography in post-long COVID 19 patients with no co-morbidities**

*Y. Aksu*[1], S. Çitil[2]; [1]Nevşehir/TR, [2]Kahramanmaraş/TR
*(dryusufaksu@hotmail.com)*

**Purpose:** We aimed to evaluate liver stiffness changes with strain-wave elastography in post-long Coronavirus 2019 disease (post-long COVID-19) patients with no co-morbidities.
**Methods or Backround:** This retrospective study was conducted between February 2021 and August 2022 in Kahramanmaraş Necip Fazıl City Hospital. Two groups were formed to compare strain-wave elastography and laboratory findings. Group 1 was post-long COVID-19 patients (n=91). Group 2 (n=84) comprised healthy subjects.
**Results or Findings:** The patient group mean age was 37 (47-56); and 39.5 (29-53.5)in the control group (p=0.110). The patient group mean body mass index was 27.54 (23.09-30.45) and 26.1 (22.92-31.23); in the control group (p=0.982). In the patient group, female /male patient ratio was s 57/34 and 44/40; in the control group (p=0.170). There was no significant difference between patients and control group in terms of demographic characteristics and BMI. In patient group mean liver strain ratio was 1.00 (0.61-1.67) and 0.72 (0.47-1.16) in control group (p=0.001). There was a significant difference between the patient and control group in terms of mean liver strain ratio (p=0.001). Table 1 summarises these findings. According to ROC analysis for predicting post-long COVID-19 patients with strain-wave elastography, the optimum cut-off value for liver strain ratio was >1.31 sensitivity 38.5% specitivity 86.9% [AUC: 0.646 P<0.001]. Box plot graphics demonstrate significant difference between mean liver strain ratio between patient and control group
**Conclusion:** Increased mean liver strain ratio was found in the patient group compared to the control group with no co-morbidities, laboratory imaging changes in post-long COVID-19 patients. This finding verified stiffness elevation in post-long COVID-19 patients.
**Limitations:** There was lack of liver biopsy.
**Ethics committee approval:** Kahramanmaraş Sütçü İmam University Medical Faculty Ethical Deanship funded this study (Approval no=2022/06-02).
**Funding for this study:** The section Limitations should be written in mixed case.
**Author Disclosures:**
Yusuf Aksu: Nothing to disclose
Serdal Çitil: Nothing to disclose

### RPS 2201-5

**Stiffness on shear wave elastography as a potential microenvironment biomarker for predicting tumour recurrence in hepatocellular carcinoma**

*X. Zhong*, H. Long, L. Chen, Y. Xie, X. Xie, M. Lin; Guangzhou/CN

**Purpose:** To explore the pathologic basis and prognostic value of tumor and liver stiffness measured by two-dimensional shear wave elastography (2D-SWE) for HCC patients after hepatic resection
**Methods or Backround:** A total of 201 patients with solitary resectable HCC were prospectively enrolled from February 2019 to February 2021. The stiffness of intratumoural tissue, peritumoural tissue, adjacent liver tissue and distant liver tissue were evaluated by 2D-SWE. The correlations between stiffness and pathological characteristics were analysed in 114 patients. The predictive values of stiffness for recurrence-free survival (RFS) were evaluated and Cutoff Finder was used to determine optimal cut-off stiffness values. Cox proportional hazards analysis was used to identify independent predictors of RFS and a nomogram was established for RFS prediction.

**Results or Findings:** Pathologically, intratumoural stiffness was associated with stroma proportion and microvascular invasion (MVI), while peritumoural stiffness was associated with tumour size, capsule and MVI. Adjacent liver stiffness was correlated with capsule and liver fibrosis stage, while distant liver stiffness was correlated with liver fibrosis stage. Peritumoural stiffness, adjacent liver stiffness and distant liver stiffness were all correlated to RFS (all $p<0.05$). Higher peritumoural stiffness (>49.4kPa) (HR=1.988, $p=0.007$) and higher adjacent liver stiffness (>31.2kPa) (HR=2.376, $p=0.023$) were significant independent predictors of worse RFS, along with tumour size and MVI. The nomogram based on these variables showed a C-index of 0.772.
**Conclusion:** Stiffness measured by 2D-SWE could be a useful biomarker of the tumour microenvironment and tumour invasiveness. Peritumoural stiffness and adjacent liver stiffness showed important values in predicting tumour recurrence after curative resection in HCC.
**Limitations:** 2D-SWE could not be applied in tumoors with a depth of more than 8cm.
**Ethics committee approval:** The ethics committee of the first affiliated hospital of Sun Yat-sen university.
**Funding for this study:** National Natural Science Foundation of China (No. 92059201).
**Author Disclosures:**
Haiyi Long: Nothing to disclose
Yuhua Xie: Nothing to disclose
Xiaoyan Xie: Nothing to disclose
Lili Chen: Nothing to disclose
Xian Zhong: Nothing to disclose
Manxia Lin: Nothing to disclose

### RPS 2201-6

**Comparison of quantitative microflow imaging and CEUS for detecting hepatic congestion in living donor liver transplantation**
*T. Han*, J. Shin, J. A. Hwang, D. Cha, T. W. Kang, W. K. Jeong; Seoul/KR
*(taewon1.han@samsung.com)*

**Purpose:** Hepatic congestion is an important factor for successful living donor liver transplantation outcome. The purpose of this study was to compare the accuracy of quantitative microflow imaging (MFI) and contrast-enhanced ultrasound (CEUS) in detecting hepatic congestion in living donor liver transplant grafts.
**Methods or Backround:** MFI and CEUS data conducted on the first post-operative day in patients who underwent LDLT between May 2022 to Sep 2022 were evaluated. 33 patients were finally included in the study. Two radiologists evaluated the quantitative vascular parameters on MFI, vascular index (VI) and CEUS findings. The VI (%) indicates the ratio of the pixels for the Doppler signal to those for the whole lesion and was calculated using the Image J software. Receiver operating characteristic curve analysis was performed to obtain cut-off values for determination of the VI for diagnosing hepatic congestion. The gold standard in diagnosing hepatic congestion is a subsequent computed tomography (CT) exam conducted in patients for detecting post-operative complications after LDLT. The McNemar test was used to compare the sensitivity and specificity of MFI and CEUS in the detection of hepatic congestion.
**Results or Findings:** CT demonstrated hepatic congestion in 45.5% (15 of 33) of patients about two weeks after a liver transplant. The cut-off value of VI on MFI was 12%. Quantitative MFI (80%, 12 of 15) demonstrated a higher sensitivity than CEUS (53.33%, 8 of 15) in detecting hepatic congestion. But there was no statistical significance between quantitative MFI and CEUS ($p>0.05$) in detecting hepatic congestion.
**Conclusion:** Quantitative analysis of MFI may prove to be beneficial in diagnosing hepatic congestion after LDLT.
**Limitations:** Exclusion of image due to motion artifact may have resulted in an overestimation of the diagnostic performance of MFI.
**Ethics committee approval:** No information provided by the submitter.
**Funding for this study:** No funding was received for this study.
**Author Disclosures:**
Woo Kyoung Jeong: Nothing to disclose
Dongik Cha: Nothing to disclose
Jeong Ah Hwang: Nothing to disclose
Taewon Han: Nothing to disclose
Tae Wook Kang: Nothing to disclose
Jaeseung Shin: Nothing to disclose

### RPS 2201-7

**Ultrasound screening of liver steatosis by innovative method of the attenuation coefficient measurement (ACM) steatometry**
*O. Dynnyk*, M. Zhayvoronok, S. Mostovyi, N. Kobyliak; Kyiv/UA
*(obdynnyk@gmail.com)*

**Purpose:** COVID-19 and liver steatosis are modern challenges: a pandemic within a pandemic. COVID-19 induces steatosis. Steatosis often debuts already at the stage of complications. The insidious nature of steatosis requires screening. The purpose is to implement the US screening of liver steatosis by the attenuation coefficient measurement (ACM) steatometry.
**Methods or Backround:** First group: in 2016-2018, 7,318 visitors to the US lab of both sexes aged 18-82 years underwent an examination of the liver in B-mode and quantitative staging of steatosis by ACM (dB/cm) of the Soneus P7 device (Ultrasign, Ukraine). Second group: 226 people aged 19-75 were examined from April to October 2022.
**Results or Findings:** In the first group, B-mode ultrasound detected steatosis in 1,317 people (18.0%): mild - 302 (4.1%), moderate - 893 (12.2%), severe - 122 (1.7%). According to ACM, steatosis was detected in 1819 people (24.8%): mild S1 in 962 (13.1%), medium S2 - 637 (8.7%), severe S3 - 220 (3.0%). In the second group, B-mode detected steatosis in 104 people (46%): mild - 35 (15.5%), moderate - 56 (24.8%), severe - 13 (5.7%). According to ACM, steatosis was detected in 114 people (50.4%): mild S1 - 44 (19.5%), medium S2 - 55 (24.3%), severe S3 -15 (6.6%). Sparring was found in 39 cases (17.3%): S1 – 2 (4.5%); S2 - 25 (45.5%); S3 – 12 (80%).
**Conclusion:** ACM has proven to be more sensitive than B-mode US to mild forms of liver steatosis and can be used for screening. The predominance of more severe forms of steatosis and sparring in the second group may indicate enforced changes in the lifestyle of the Ukrainian population during the COVID pandemic and the war (hyperalimentation of carbohydrates and hypodynamia).
**Limitations:** No limitations were identified.
**Ethics committee approval:** No information provided by the submitter.
**Funding for this study:** No funding was received for this study.
**Author Disclosures:**
Oleh Dynnyk: Nothing to disclose
Maxim Zhayvoronok: Nothing to disclose
Nazarii Kobyliak: Nothing to disclose
Serhii Mostovyi: Nothing to disclose

### RPS 2201-8

**Diagnostic performance of ultrasound-guided attenuation parameter (UGAP) for the quantification of hepatic steatosis in patients with biopsy-proven nonalcholic fatty liver disease: a prospective study**
*R. Cannella*, F. Agnello, G. Porrello, A. U. Spinello, G. Infantino, S. Petta, D. Cabibi, A. Taibbi, T. V. V. Bartolotta; Palermo/IT

**Purpose:** To assess the diagnostic performance and the reproducibility of Ultrasound-Guided Attenuation Parameter (UGAP) for the quantification of hepatic steatosis in patients with biopsy-proven nonalcholic fatty liver disease (NAFLD)
**Methods or Backround:** This prospective study included adult patients with NAFLD who underwent ultrasound examination with UGAP and liver biopsy within two months. UGAP measurements were performed by two independent and blinded radiologists. A total of 12 consecutive measurements were acquired in each patient which were repeated twice by the first radiologist. The median values were considered for the analysis. Hepatic steatosis was graded at liver biopsy as: (0) <5%; (1) 5-33%; (2) 33-66%; (3) >66%. Area under the ROC curve (AUC) with Youden index were calculated to determine the diagnostic performance with optimal cut-off. The intraclass correlation coefficient (ICC) was used to assess the inter- and intra-observer reproducibility.
**Results or Findings:** 52 patients (31 female, with mean age of 53.8±11.3 years) with NAFLD were prospectively enrolled. At histopathological analysis, 11/52 (21.2%) and 21/52 (40.4%) had grade 2 and 3 steatosis respectively. Median UGAP was 0.76 dB/cm/MHz (IQR: 0.05 dB/cm/MHz; IQR/median ratio: 5.7%). For the diagnosis of grade ≥2 steatosis, median UGAP had an AUC of 0.801 (95%CI: 0.662-0.901, $p<0.001$) while, for the grade 3 steatosis median, UGAP had an AUC of 0.863 (95%CI: 0.735-0.944, $p<0.001$). The optimal cut-off of >0.75 produced a sensitivity of 78.1% and a specificity of 82.3% for the diagnosis of grade ≥2 steatosis. The inter- and intra-observer reproducibility were excellent with an ICC of 0.93 (95%CI: 0.88-0.96) and 0.95 (95%CI: 0.91-0.97), respectively.
**Conclusion:** UGAP measurements provide a good performance for the diagnosis of moderate to severe steatosis in patient with NAFLD with and excellent reproducibility.
**Limitations:** This was a single-centre study. Lack of control group without NAFLD.
**Ethics committee approval:** No information provided by the submitter.
**Funding for this study:** Funding was provided by GE Healthcare
**Author Disclosures:**
Giorgia Porrello: Nothing to disclose
Adele Taibbi: Nothing to disclose
Daniela Cabibi: Nothing to disclose
Roberto Cannella: Other: Bracco, Bayer: support for attending meetings.
Francesco Agnello: Nothing to disclose
Giuseppe Infantino: Nothing to disclose
Salvatore Petta: Nothing to disclose
Alessandro Umberto Spinello: Nothing to disclose
Tommaso Vincenzo Vincenzo Bartolotta: Nothing to disclose

**08:00-09:00** **Research Stage 2**

### Research Presentation Session: Chest

### RPS 2204

### Thoracic imaging in COVID-19

**Moderator**
M. Silva; Parma/IT
*(mariosilvamed@gmail.com)*

### RPS 2204-2

**Pulmonary embolism and CT pulmonary angiography usage in Omicron COVID patients**

*J. Kang*, B. Gwyther, A. Rottenberg, Q. Omran, E. Avini, R. Patel, A. Whittington, H. Chana; London/UK
*(jungwoo.kang2@nhs.net)*

**Purpose:** The NHS has stopped mandatory COVID-testing of asymptomatic patients, partly due to reduced Omicron-COVID severity and vaccinations. Asymptomatic and nosocomial transmission are more prevalent in Omicron; therefore, this decision has significant risk to inpatients. Currently, little data is comparing the risk of pulmonary embolism(PE) in Omicron-COVID and non-COVID patients, which this study examines.
**Methods or Backround:** This retrospective cohort study examined all patients undergoing CT pulmonary angiogram (CTPA) between 24/01/2022-30/04/2022 in two UK hospitals. Data were collected on patients' age, sex, COVID status, CTPA results (COVID pneumonitis, PE), and COVID vulnerability and vaccination status in COVID patients as part of a larger study. Data was acquired regarding the number of COVID and non-COVID presentations to and admissions from the emergency department from 24/01/2022-23/04/2022. Differences in PE prevalence and association with mortality were analysed with logistic regression.
**Results or Findings:** Omicron COVID, compared to non-COVID patients, had a greater prevalence of PE in admitted patients (3.3% vs. 0.62%; OR=2.35; p<0.001). Omicron-COVID patients were more likely to have a CTPA than non-COVID patients (OR=3.37; p<0.001), with COVID patients accounting for 13.2% of all inpatient CTPAs despite only accounting for 1.9% of new admissions. CTPA yield was not significantly different between Omicron-COVID and non-COVID patient scans (11.4% vs. 13.3%; p=0.915). Presence of acute PE increased mortality in Omicron-COVID (AOR=3.90; 95% CI=1.18–12.93; p=0.026) but not in non-COVID patients (AOR=1.25; 95% CI=0.68–2.28; p=0.470).
**Conclusion:** Admission with Omicron infection is significantly associated with increased PE risk. Limiting Omicron nosocomial spread is necessary to reduce inpatient morbidity/mortality and demands on busy radiology departments.
**Limitations:** The main limitation is the reliance on diagnostic coding data for COVID status determination for total emergency department/inpatient numbers. Further, selection bias in analysing mortality did not include all inpatients.
**Ethics committee approval:** The audit committee and Research/Development department for service evaluation approval was obtained.
**Funding for this study:** Not applicable.
**Author Disclosures:**
Harmeet Chana: Nothing to disclose
Jungwoo Kang: Nothing to disclose
Ashley Whittington: Nothing to disclose
Elhaam Avini: Nothing to disclose
Rajiv Patel: Nothing to disclose
Qabass Omran: Nothing to disclose
Augustus Rottenberg: Nothing to disclose
Bethany Gwyther: Nothing to disclose

### RPS 2204-3

**Myocardial extracellular volume assessment at computed tomography in COVID-19 patients with regards to pulmonary embolism**

C. B. Monti, *F. Rizzetto*, M. Zanardo, D. Capra, G. Folco, F. Silletta, F. Secchi, F. Sardanelli; Milan/IT
*(Francesco.rizzetto@unimi.it)*

**Purpose:** Our purpose was to assess myocardial extracellular volume (ECV) in patients with novel coronavirus disease (COVID-19) with regard to the presence of pulmonary embolism (PE).
**Methods or Backround:** We retrospectively reviewed consecutive patients who underwent contrast-enhanced computed tomography (CT) for assessing PE and whose CT scans included at least one unenhanced and one venous phase acquisition performed at about 1 minute after contrast administration for evaluating the mediastinum. Included patients were also to have haematocrit values obtained at most one day from the CT scan. Regions of interest were placed in the myocardium and ventricular blood pool of both the unenhanced and venous phase scan, and ECV was subsequently calculated.
**Results or Findings:** Our final population counted 94 patients, 63 (67%) of whom were males, with a median age of 70 years (interquartile range, IQR, 56–76 years). 28 patients were diagnosed with PE at CT. Median haematocrit was 39% (IQR 35–43%). Median overall ECV was 31% (IQR 26–35%), whereas median ECV in patients with PE was 34% (IQR 29–38%), significantly higher than in those without PE (30%, IQR 25–34%, p=0.010).
**Conclusion:** Myocardial ECV is high in COVID-19 patients, possibly indicating a likelihood of underlying myocardial damage. The higher ECV in patients with PE may relate to ventricular overload rising from subsequent pulmonary hypertension.
**Limitations:** Single-centre analysis in a retrospective study. ECV was calculated using non-gated CT unenhanced and portal venous phase scans.
**Ethics committee approval:** Ethics Commitee of IRCCS Ospedale San Raffaele - Study Protocol CARDIORETRO
**Funding for this study:** No funding was required.
**Author Disclosures:**
Francesco Rizzetto: Nothing to disclose
Francesco Secchi: Nothing to disclose
Francesco Sardanelli: Advisory Board: Bayer Research/Grant Support: Bracco
Caterina Beatrice Monti: Nothing to disclose
Gianluca Folco: Nothing to disclose
Moreno Zanardo: Nothing to disclose
Francesco Silletta: Nothing to disclose
Davide Capra: Nothing to disclose

### RPS 2204-4

**Can radiomics act as a predictor of pulmonary fibrosis in COVID-19?**

*G. Fichera*, G. Minesso, E. Cocconcelli, R. Motta, R. Stramare, E. Balestro, C. Giraudo; Padova/IT
*(giulia.fichera@studenti.unipd.it)*

**Purpose:** To assess the predictive role of pulmonary fibrosis of radiomic features extracted from chest computed tomography (CT) patients with COVID-19.
**Methods or Backround:** Patients affected by COVID-19 who underwent a chest CT at diagnosis and had a clinical and radiological 12 months follow-up in our tertiary centre were assessed. One radiologist with five years of experience in thoracic imaging used an open-source software (3D Slicer) to semi-automatically segment and extract 33 radiomic features from the lung parenchyma of each enrolled patient. At follow-up, patients were considered as affected by signs of fibrosis according to clinical (e.g., shortness of breath, dry cough) and radiological findings (e.g., interstitial thickening, bronchiectasis). The factor analysis was applied to select highly correlating radiomic features. Their predictive role was investigated by logistic regression analysis with post-hoc Bonferroni correction to reduce the effect of repeated testing (p<0.05 as the significance level). In case of significant results, the diagnostic value of the variable/s was computed by applying receiver operating characteristic curves.
**Results or Findings:** One hundred twenty patients (43 females; mean age 61±13 years) matched the inclusion criteria and were examined. Twenty-two patients (18.3%) had clinical and radiological signs of fibrosis. The following highly correlating features were selected via the factor analysis: Idm, Imc2, autocorrelation, cluster shade, energy, and long-run low grey level emphasis. The radiomics model was significant (p=0.036; in particular, Imc2 p=0.036), but after the post hoc correction, none of the variables demonstrated to be a predictor of pulmonary fibrosis (p>0.05, each).
**Conclusion:** Radiomics seems not to have a role in predicting pulmonary fibrotic changes at 12 months in COVID-19 patients.
**Limitations:** No limitations were identified.
**Ethics committee approval:** No information provided by the submitter.
**Funding for this study:** No funding was provided for this study.
**Author Disclosures:**
Giulia Fichera: Nothing to disclose
Raffaella Motta: Nothing to disclose
Roberto Stramare: Nothing to disclose
Elisabetta Balestro: Nothing to disclose
Giacomo Minesso: Nothing to disclose
Elisabetta Cocconcelli: Nothing to disclose
Chiara Giraudo: Nothing to disclose

### RPS 2204-5

**Computer-aided simple triage (CAST) for COVID-19 pneumonia based on RSNA expert consensus statement and radiological finding evaluations in a multi-centre study**

*Y. Ohno*[1], T. Aoki[2], M. Endo[3], H. Koyama[4], H. Moriya[5], F. Okada[6], T. Higashino[7], N. Oyama-Manabe[8], J. Matsumoto[9]; [1]Toyoake/JP, [2]Kitakyushu/JP, [3]Chiba/JP, [4]Osaka/JP, [5]Fukushima-City/JP, [6]Oita/JP, [7]Himeji/JP, [8]Saitama/JP, [9]Kawasaki/JP
*(yohno@fujita-hu.ac.jp)*

**Purpose:** To determine the capability of machine learning (ML) based computer-aided simple triage (CAST) software based on RSNA expert consensus statement for diagnosis of COVID-19 pneumonia in a multicenter study.
**Methods or Backround:** 174 cases who underwent CT and PCR tests for COVID-19 were retrospectively included in this multicenter study. CT data were assessed by CAST and consensus from three independent board-certified chest radiologists. All cases were divided into three groups: positive cases (i.e. typical and indeterminant appearances), atypical appearance and negative for pneumonia (i.e. latter two groups were assessed as negative cases). To determine radiological finding evaluation capability with CAST, three other board-certified chest radiologists also assessed CAST results of radiological findings, including pulmonary emphysema, nodular lesion, consolidation, GGO, reticulation and honeycombing. They classified them into five criteria, respectively. To determine the capability of CAST, agreements between CAST and consensus were evaluated by kappa statistics with the $\chi 2$ test. Then, sensitivity (SE), specificity (SP) and accuracy (AC) between CAST and PCR were compared with those between consensus and PCR by McNemar's test. To determine overall radiological finding evaluation capability, each radiological finding accuracy of CAST was assessed in all readers.
**Results or Findings:** Agreement between CAST and consensus evaluations was determined as moderate ($\kappa=0.55$, $p<0.0001$). No significant difference of SE (88.5%) was observed between CAST and consensus, although SP (47.1%) and AC (67.8%) of CAST were significantly lower than those of consensus (SP: 79.3%, $p<0.0001$; AC: 83.9%, $p<0.0001$). Accuracies for radiological finding evaluation by CAST were determined as follows: reader A, 91.7-99.4%; reader B, 99.4-100%; reader C, 99.4-100%.
**Conclusion:** This multicenter study shows CAST is considered at least as valuable as radiologists for COVID-19 pneumonia triage with accurate radiological finding evaluations.
**Limitations:** No limitations were identified.
**Ethics committee approval:** IRB of Fujita Health University Hospital
**Funding for this study:** This study was funded by a research grant from Canon Medical Systems Corporation.
**Author Disclosures:**
Hiroshi Moriya: Nothing to disclose
Takatoshi Aoki: Nothing to disclose
Hisanobu Koyama: Nothing to disclose
Fumito Okada: Nothing to disclose
Noriko Oyama-Manabe: Nothing to disclose
Yoshiharu Ohno: Research/Grant Support: Canon Medical Systems Corporation
Junichi Matsumoto: Nothing to disclose
Masahiko Endo: Nothing to disclose
Takanori Higashino: Nothing to disclose

### RPS 2204-6

**Imaging burden associated with COVID-19 vaccination-related symptoms and complications**

*J. Krawczyk*, D. Amiras; London/UK
*(jejabig.psn@gmail.com)*

**Purpose:** To establish imaging burden associated with COVID-19 vaccination-related symptoms and complications.
**Methods or Backround:** Imperial College Healthcare NHS Trust services a population of 1.5-2 million. We searched the imaging database in our Institution across all modalities in the period of 1/1/20 - 1/10/22 using the 'vaccine' keyword as a search criterium in the request clinical indication to attempt to assess the imaging burden of potential complications from vaccination. Results related to other vaccines (e.g. pneumococcal/flu) or imaging reports where COVID vaccine-related symptoms were not the primary indicators were excluded.
**Results or Findings:** There were 1133 results of the search with the above criteria. 38 requests related to other non-COVID vaccines, and these were excluded. The vast majority of imaging requested due to COVID vaccine-related symptoms was reported as normal or not suggestive of any pathology resulting from the vaccine. Among described pathologies, reactive lymphadenopathy was by far the most common one, often resulting in follow-up imaging. Some reported symptoms, however, were linked to serious, life-threatening pathology and flare-ups of chronic conditions, with extensive imaging findings.
**Conclusion:** We have found a significant imaging burden because of imaging requests due to COVID-19 vaccine-related symptoms. However, in most cases, no pathology was demonstrated. To lessen the imaging burden, raised awareness on which complications warrant imaging and education on imaging features of COVID-19 vaccine-related complications are essential.
**Limitations:** The study was limited by the need to use search terms in the clinical indications; this may be omitted by the ordering clinicians, leading to an underestimation of the imaging burden.
**Ethics committee approval:** No information provided by the submitter.
**Funding for this study:** Not applicable.
**Author Disclosures:**
Jędrzej Krawczyk: Nothing to disclose
Dimitri Amiras: Nothing to disclose

### RPS 2204-7

**Can radiology residents be a reliable aid in diagnosing COVID-19 pneumonia? A study on the interobserver agreement between residents and radiologists in diagnosing COVID-19 pneumonia on CXR**

*M. Á. Gómez Bermejo*[1], P. Marazuela Garcia[2], N. A. Almeida Arostegui[1], A. Vicente Bartulos[1], J. J. Alarcon Rodriguez[1], A. Ureña Vacas[3], Á. Arribas Marcos[1], L. Gorospe Sarasua[1]; [1]Madrid/ES, [2]Toledo/ES, [3]Alicante/ES
*(miangelgomber@gmail.com)*

**Purpose:** To assess interobserver agreement between radiology residents and thoracic radiologists in diagnosing COVID-19 pneumonia with chest radiography (CXR).
**Methods or Backround:** We included patients with clinically suspected COVID-19 admitted to the emergency department of a tertiary hospital on 24 March 2020. Five thoracic radiologists and five radiology residents prospectively, independently, and blindly interpreted every patient's CXR. The CXR findings were reported using the Fleischner Society Glossary and classified as suspicious or not suspicious for COVID-19 pneumonia. The RT-PCR result was used as the reference standard. We used Cohen's kappa statistics to measure the interobserver agreement in diagnosing COVID-19 pneumonia and identifying each imaging finding.
**Results or Findings:** 257 patients were included. Interobserver agreement between residents and thoracic radiologists was good, with a Cohen kappa index of 0.74 (95%CI 0.62-0.86). The highest agreement was obtained for lung opacities (0.8; 95%CI 0.67-0.92). However, agreement in the detection of peri broncho vascular interstitial thickening was poor (0.29; 95%CI 0.17-0.41).
**Conclusion:** Our study suggests that radiology residents can diagnose COVID-19 pneumonia on CXR with slightly inferior accuracy but with a high degree of agreement with expert thoracic radiologists. This could help in emergency department overcrowding, as was the case during the COVID-19 pandemic.
**Limitations:** The study was performed on a single day and during peak-pandemic when the incidence was very high. The chest radiograph readers were only ten and of one single institution.
**Ethics committee approval:** No information provided by the submitter.
**Funding for this study:** No funding was received for this study.
**Author Disclosures:**
Nicolás Alejandro Almeida Arostegui: Nothing to disclose
Álvaro Arribas Marcos: Nothing to disclose
Miguel Ángel Gómez Bermejo: Nothing to disclose
Agustina Vicente Bartulos: Nothing to disclose
Jose Javier Alarcon Rodriguez: Nothing to disclose
Pablo Marazuela Garcia: Nothing to disclose
Almudena Ureña Vacas: Nothing to disclose
Luis Gorospe Sarasua: Nothing to disclose

### RPS 2204-8

**Quantitative CT analysis of COVID-19 pneumonia from the acute phase up to 24-months follow-up and correlation with functional respiratory parameters**

C. Romei[1], *S. C. Fanni*[1], F. Volpi[1], L. Colligiani[1], F. Pistelli[1], B. Bartholmai[2], L. Carrozzi[1], E. Neri[1], A. De Liperi[1]; [1]Pisa/IT, [2]Rochester/US

**Purpose:** This study aims to assess the CT changes in patients with COVID-19 pneumonia from baseline to 24-month follow-up and their correlations with functional parameters.
**Methods or Backround:** Patients hospitalised and who survived COVID-19 were recruited. All the patients underwent chest CT at baseline (T0) and 3 months after discharge (T3) with pulmonary function tests (PFTs). Patients with residual signs repeated CT at 12 (T12) and 24 (T24) months. CT underwent a texture analysis through CALIPER, which quantifies different patterns: percentage of normal parenchyma (Norm), ground glass (GG), reticulations (Ret), and vascular-related structures (VRS). Differences ($\Delta$) for each parameter between time points were calculated. ROC analyses were performed to test whether CALIPER parameters at T0 were associated with functional impairment at T3 and the persistence of lung abnormalities at T12.

Sunday

**Results or Findings:** The cohort was represented by 133 subjects at T0 and T3, 61 at T12, and 34 at T24. GG median were 8.44, 0.14, 0.13, and 0.12 at T0, T3, T12, and T24, while Ret were 2.79 at T0 and 0.14 at follow-up time-points. Δ for Ret were -3.91 between T3 and T0, -0.07 between T12 and T3, and -0.01 between T12 and T24. GG and VRS at T0 achieved AUCs of 0.73 as predictors of total lung capacity impairment at T3. Considering the persistence of abnormalities at T12, GG and VRS at T0 achieved AUCs of 0.71 and 0.72.
**Conclusion:** CALIPER quantified the CT patterns from baseline up to 24-months follow-up in COVID-19 pneumonia. Most of the resolution occurred at T3 and what persisted at T3 also persisted at T12 and T24, predominantly Ret. Baseline parameters were associated with functional impairment at T3 and persistence of abnormalities at T12.
**Limitations:** Small sample size in a monocentric study.
**Ethics committee approval:** No information provided by the submitter.
**Funding for this study:** No funding was required.
**Author Disclosures:**
Emanuele Neri: Nothing to disclose
Laura Carrozzi: Nothing to disclose
Chiara Romei: Nothing to disclose
Brian Bartholmai: Nothing to disclose
Salvatore Claudio Fanni: Nothing to disclose
Annalisa De Liperi: Nothing to disclose
Francesco Pistelli: Nothing to disclose
Leonardo Colligiani: Nothing to disclose
Federica Volpi: Nothing to disclose

### RPS 2204-9

**CT-based diagnosis of COVID-19: when is the use of an AI assistance system beneficial? An international multicentre multivendor study of human-machine interaction**
*J. Kottlors*, F. Meng, P. Fervers, A-I. Iuga, R. Shahzad, D. Maintz, T. Persigehl; Cologne/DE

**Purpose:** Artificial intelligence (AI) can distinguish COVID-19 from other non-COVID-19 pneumonia (nCP) based on the typical morphological appearance in computed tomography (CT). The present investigation aims to evaluate and identify cases where AI assistance for differentiating COVID-19 and nCP is beneficial.
**Methods or Backround:** This IRB-approved study focused on the evaluation of radiologists' performance without and with AI assistance by eight radiologists, resulting in a total of n = 2880 diagnostic decisions within a subgroup (n = 180; 50% COVID-19 / 50% nCP) from an international multicentre, multivendor chest CT dataset (n = 1591). To identify CT examinations in which AI assistance is particularly useful to achieve higher accuracy, we used subgroup- and multiple regression analyses with interaction effects depending on the aetiology of the pathogen, the complexity of the image findings, individual confidence score, and radiologists' reporting time.
**Results or Findings:** Without AI assistance, the diagnostic accuracy of the eight radiologists was 79.1% and increased with AI assistance to 82.3%. Significantly fewer errors occurred when using AI assistance (p<.05) in the subgroups with more complex cases. The same applies to cases with a priori lower confidence score and longer reporting time (p<.05). The radiologists' combination of low confidence and long reporting time predicts a benefit of using AI assistance.
**Conclusion:** AI assistance for radiological differentiation between COVID-19 and nCP in CT increases diagnostic accuracy, especially in cases with low confidence and simultaneously long reporting time. The widespread and targeted use of AI assistance in radiological reporting of chest CT examinations seems to be warranted.
**Limitations:** The study is retrospective - bidimensional decisions without including other diseases in the experimental setting.
**Ethics committee approval:** The study was conducted according to the guidelines of the Declaration of Helsinki and approved by the Ethics Committee of The First Hospital of Jilin University IRB: 2020-595, Wuhan First Hospital IRB: [2020]17, Ningbo hwamei Hospital, University of Chinese Academy of Sciences IRB: PJ-NBEY-KY-2020-194-01, Cologne University IRB: 20-1676.
**Funding for this study:** This study was funded by the BMBF as part of the University Medicine Network (Project RACOON, 01KX2021) and by the China International Medical Foundation, Imaging Research, SKY (Z-2014-07-2003-03).
**Author Disclosures:**
Rahil Shahzad: Employee: Philips Healthcare, no conflicts of interest.
David Maintz: Nothing to disclose
Philipp Fervers: Nothing to disclose
Fanyang Meng: Nothing to disclose
Jonathan Kottlors: Nothing to disclose
Andra-Iza Iuga: Nothing to disclose
Thorsten Persigehl: Nothing to disclose

**08:00-09:00** **Research Stage 3**

### Research Presentation Session: Musculoskeletal

### RPS 2210

### Musculoskeletal: body composition

**Moderator**
C. Messina; Milan/IT
**Author Disclosures:**
Carmelo Messina: Grant Recepient: Bracco, Abiogen, Fidia

### RPS 2210-2

**Opportunistic bone mineral density screening in overview scans using spectral detector technology**
*J. L. Hammel*, L. Birnbacher, L. Ushakov, M. Makowski, F. Pfeiffer, D. Pfeiffer; Munich/DE

**Purpose:** To check whether overview scans in spectral detector CT provide appropriate diagnostic information to calculate dual-energy x-ray absorptiometry (DEXA) equivalent areal bone mineral density (BMD) maps for opportunistic osteoporosis screening.
**Methods or Backround:** A 64-slice single-source dual-layer spectral CT scanner was used to acquire overview (scout) scan data of 52 patients, resulting in a total number of 208 vertebral bodies. Scout scans performed with a standard dose of 0.06 mGy were processed using a spectral dictionary denoising algorithm. Virtual monoenergetic images were used to determine the three-dimensional (3D) bone mineral density. A 3D convolutional network for image segmentation and labelling is applied for automated BMD quantification. The 3D spine mask is forward projected onto the detector plane to achieve a perfect registration of 2D and 3D BMD maps for correlation analysis.
**Results or Findings:** In a representative patient sample, a 6-fold increase in signal-to-noise ratio from 3.5 to 21.8 was achieved by using the spectral dictionary denoising method. The spectral 3D BMD and the corresponding areal BMD values ranged from 118 to 293 mg/cm**3 and 0.65 to 1.42 g/cm**2, respectively. 7, 23, and 22 subjects were diagnosed as osteoporotic, osteopenic or normal, according to patient-wide averaged areal BMD values. The correlation analysis between both methods showed a Pearson correlation coefficient of 0.67 with a p-value << 0.01 and a 95 % confidence interval of the slope of 148 ± 22.5.
**Conclusion:** Denoised material-specific BMD maps in projection space show a significant positive correlation to volume measurements enabling opportunistic osteoporosis screening in all patients with a fractional dose of a volume CT scan.
**Limitations:** The measurement of the BMD on overview scans gives only 2D information, in which trabecular and cortical bone cannot be quantified separately.
**Ethics committee approval:** The medical faculty approved this study.
**Funding for this study:** BMBF-GRK2274 and IAS supported this study.
**Author Disclosures:**
Johannes Leopold Hammel: Nothing to disclose
Lorenz Birnbacher: Nothing to disclose
Franz Pfeiffer: Nothing to disclose
Lev Ushakov: Nothing to disclose
Marcus Makowski: Nothing to disclose
Daniela Pfeiffer: Nothing to disclose

### RPS 2210-3

**Comparison between QCT-derived volumetric bone mineral density (BMD) and DXA-based areal BMD and trabecular bone ccore (TBS): a lumbar spine study**
*S. Fusco*, G. Irmici, F. Serpi, D. Albano, L. M. Sconfienza, C. Messina; Milan/IT
*(stefano.fusco@unimi.it)*

**Purpose:** Applying quantitative computed tomography (QCT) to chest/abdominal CT scans can provide valuable information for opportunistic screening for osteoporosis. We compared QCT volumetric bone mineral density (BMD) to areal BMD and trabecular bone score (TBS) obtained using dual-energy x-ray absorptiometry (DXA).
**Methods or Backround:** We retrospectively evaluated subjects who underwent a DXA scan and a chest/abdomen CT scan (six months was the maximum time lapse between the two examinations). We used the ''asynchronous'' calibration of the CT images to obtain BMD values from QCT (Mindways Software Inc., Austin, TX). T-score values were used for DXA diagnosis according to the World Health Organization (WHO) criteria. For QCT, the American College of Radiology ranges were used to diagnose normal, osteopenic or osteoporotic status.

**Results or Findings:** After applying the inclusion and exclusion criteria, our final sample comprised 105 subjects (87 women, mean age 69±11 years). Overall, 49 subjects (46,6%) presented at least one major fragility fracture (FF). QCT diagnosis was as follows: osteoporosis=59; osteopenia=36; normal status=10. DXA diagnosis was: osteoporosis=25; osteopenia=35; normal status=45. 38 subjects (36,2%) showed degraded microarchitecture according to TBS values. The correlation was moderate between BMD values by DXA and QCT (r=0.446) as well as between TBS and QCT (r=0.524), while it was good between DXA and TBS (r=0.621). ROC curves were calculated to assess the capability of discriminating between subjects with/without FF and showed the following area under the curve: 0.748 for QCT BMD, 0.575 for DXA BMD and 0.650 for TBS.
**Conclusion:** Opportunistic QCT diagnosed a higher prevalence of osteoporosis compared to DXA. TBS performed better than DXA BMD in discriminating between subjects with/without FF.
**Limitations:** The study is limited by being a retrospective study.
**Ethics committee approval:** Not applicable.
**Funding for this study:** No funding was received for this study.
**Author Disclosures:**
Stefano Fusco: Nothing to disclose
Carmelo Messina: Grant Recipient: Bracco, Abiogen
Luca Maria Sconfienza: Grant Recipient: Samsung Medison, Esaote, Abiogen, Pfizer, Novartis, Fidia, Biolive, Janssen Cilag, Bracco, MSD
Giovanni Irmici: Nothing to disclose
Francesca Serpi: Nothing to disclose
Domenico Albano: Grant Recipient: Bracco

### RPS 2210-4

**Clinical QCT-, DECT- and PCCT-based bone-volume fraction measurement: a pairwise and qualitative comparison of the three techniques**

*P. Wili*[1], D. Gascho[2], A. Dudle[1], D. Nanz[2], M. Thali[2], P. Zysset[1]; [1]Bern/CH, [2]Zurich/CH
*(patrik.wili@irm.uzh.ch)*

**Purpose:** Dual-energy and photon-counting computed tomography (DECT, PCCT) could open the door for opportunistic osteoporosis screening and a more accurate representation of the morphological structure of bone without the need for a calibration phantom or increased radiation dose. This study sought to explore qualitatively the potential of new CT technologies to evaluate bone-volume fraction and the pitfalls of quantitative CT (QCT)-based bone-density measurements.
**Methods or Backround:** Besides other morphological parameters of bone, volumetric bone mineral density (vBMD) or bone-volume fraction (BV/TV) are crucially important indicators of bone strength. While vBMD can be quantitatively measured by QCT using a reference phantom, DECT and PCCT are thought to enable a direct assessment of BV/TV. We aimed a pairwise and quantitative comparison of the three techniques using bovine trabecular bone specimens and to quantify the influence of elevated fat concentration in marrow on the prediction accuracy. With the help of a specially designed polymethyl-methacrylate sample holder, we successively replaced the marrow of each sample with three media: air, 0.9% NaCl and an adipose tissue surrogate to mimic different marrow conditions.
**Results or Findings:** Paired t-testing showed that elevated fat concentration in marrow has no significant effect on DECT-based BV/TV assessment (p-value=0.5), while it leads to a systematic underestimation of the bone density using QCT (p-value<0.001). Even more accurate differentiation of bone in its components seems feasible with PCCT. The evaluation of those images is ongoing and will be part of the presentation.
**Conclusion:** DECT allows robust bone density evaluation without increased radiation dose, while QCT is significantly biased through an altered fat concentration in the bone marrow and could lead to a false estimation of bone strength.
**Limitations:** The specimen holder doesn't mimic surrounding human body tissues contributing to beam hardening effects.
**Ethics committee approval:** No information provided by the submitter.
**Funding for this study:** Not applicable.
**Author Disclosures:**
Patrik Wili: Nothing to disclose
Philippe Zysset: Nothing to disclose
Alice Dudle: Nothing to disclose
Daniel Nanz: Nothing to disclose
Dominic Gascho: Nothing to disclose
Michael Thali: Nothing to disclose

### RPS 2210-5

**A clinical study assessing Ibx BH as a tool for opportunistic screening for osteoporosis and treatment decision-making from wrist radiographs**

*R. Meertens*[1], B. T. Lopez[2], B. Crone[2], M. Gundry[1], R. Lane[1], F. Manning[1]; [1]Exeter/UK, [2]Sedgefield/UK
*(r.m.meertens@exeter.ac.uk)*

**Purpose:** Osteoporosis is an increasing societal burden for an ageing population. This study investigated a physics-based modelling algorithm (Ibx_BH) utilising post-processing from wrist radiographs, facilitating opportunistic identification of those with poor bone health at an early stage. Aims were to i) predict risk of fragility fracture and treatment recommendations, based on FRAX assessment guidelines; and ii) calculate a probability of osteoporosis based on predicted dual x-ray absorptiometry (DXA) T-scores.
**Methods or Backround:** 265 participants over the age of 50 were recruited in this single-centre, non-randomised, prospective study. Participants underwent FRAX assessment, bilateral postero-anterior wrist radiographs, and DXA of the wrists, hips and lumbar spine.
**Results or Findings:** Area Under Receiver Operating Characteristic Curve analysis of osteoporosis prediction based on DXA T-scores at corresponding regions of interest at the ultra-distal and distal third of the radius produced 0.9484 (99%CI [0.9197, 0.9772]) and 0.9714 (99%CI [0.9487, 0.9941]), respectively. A risk prediction model based on Ibx_BH for prediction of FRAX treatment decision-making demonstrated a sensitivity of 0.97 (97.5%CI [0.85, 1]) and specificity 0.79 (97.5%CI [0.71, 0.87]). This is compared to FRAX without femoral neck aBMD that demonstrated a matched sensitivity 0.97 (97.5%CI [0.85, 1]) and specificity 0.60 (97.5%CI [0.50, 0.69]).
**Conclusion:** Ibx_BH applied at the wrist may provide a clinically useful risk prediction model of osteoporosis and FRAX-based treatment decisions, presenting the potential opportunistic identification of poor bone health during routine wrist radiographs at an earlier stage in the patient pathway.
**Limitations:** Further research is ongoing to investigate the application of Ibx_BH to other radiographic examinations commonly undertaken in people over 50 and to optimise the potential use of Ibx_BH in current clinical pathways based on multiple clinical sites.
**Ethics committee approval:** UK NHS Health Research Authority (Ref: 21/LO/0772)
**Funding for this study:** This study was funded by Ibex Innovations Ltd.
**Author Disclosures:**
Robyn Lane: Nothing to disclose
Ben Thomas Lopez: Employee: Ibex Innovations Ltd.
Fay Manning: Nothing to disclose
Robert Meertens: Nothing to disclose
Michael Gundry: Nothing to disclose
Ben Crone: Employee: Ibex Innovations Ltd.

### RPS 2210-6

**Fatty acids composition of the sacroiliac joint in axial spondyloarthritis: analysis using 3.0 T chemical shift-encoded MRI**

*M. Chen*, C. Cheng, C. Zou, G. Cheng; Shenzhen/CN
*(chenmin@bjmu.edu.cn)*

**Purpose:** The study aimed to investigate the bone marrow fatty acids (FAs) composition of the sacroiliac joint in patients with axSpA compared to controls using chemical shift-encoded MRI (CSE-MRI).
**Methods or Backround:** Patients with axial spondyloarthritis (axSpA) and non-SpA controls were prospectively included. Multiple gradient-echo CSE-MRI images of the sacroiliac joint were acquired on a 3.0T MRI scanner and post-processed for bone marrow FAs composition analysis. Regions of interest (ROIs) were manually placed on the subchondral bone with and without fat metaplasia. Intergroup comparisons were performed using t-tests.
**Results or Findings:** A total of 85 participants were analysed, including 48 axSpA (25 male, 23 female) and 37 non-SpA controls (18 male, 19 female). In axSpA, male patients had significantly higher monounsaturated FA compared to controls in areas with fat metaplasia in the sacrum (+12%) and in the ilium (+9%), significantly lower polyunsaturated FA in areas with fat metaplasia in the sacrum (-10%), and significantly lower saturated FA in areas without fat metaplasia in the sacrum (-6%). In females, patients with axSpA had significantly higher SFA in areas with fat metaplasia in the ilium (+7%) compared to controls.
**Conclusion:** Fatty acids composition of the sacroiliac joint alters in patients with axSpA and can be detected using CSE-MRI-based analysis.
**Limitations:** The study included only a small sample of axSpA patients and controls, and although several significant changes were found, these findings need to be validated in larger cohorts.
**Ethics committee approval:** This study was approved by institutional ethics committee and written informed consent was obtained from all participants.

**Funding for this study:** No funding was received for this study.
**Author Disclosures:**
Min Chen: Nothing to disclose
Guanxun Cheng: Nothing to disclose
Chao Zou: Nothing to disclose
Chuanli Cheng: Nothing to disclose

### RPS 2210-7

**Can biosensitive compositional MRI sequences tell us about early ageing?**

*M. Frenken*, L. Radke, L. M. Wilms, D. B. B. Abrar, A. Müller-Lutz; Düsseldorf/DE

**Purpose:** Analysis of the age dependence of cartilage tissue and its collagen-proteoglycan matrix using T1ρ-, T2-mapping and gagCEST (glycosaminoglycan chemical exchange saturation transfer) imaging in the metacarpophalangeal (MCP) joints of healthy volunteers.
**Methods or Backround:** 84 MCP joints of 21 volunteers without finger pain (right hand D1- D4, mean age 44 ± 24 years, range: 20-61 years) were examined with T1ρ-, T2-mapping, gagCEST imaging and T2 -weighted sequences with a 3T MR scanner, with approval of the local ethics committee after written informed consent was obtained. Only MCP joints without signs of osteoarthritis were analysed. A region-of-interest-based analysis of T1ρ-, T2-mapping and gagCEST was performed. Kendall-Tau (τ)-correlation between age and T1ρ-, T2-mapping and gagCEST was performed.
**Results or Findings:** T1ρ-mapping values increased significantly with age τ= 0.35, p< 0.001. T2-mapping values increased significantly with age τ= 0.29, p= 0.0052. GagCEST effects increased significantly with age τ= 0.25, p= 0.014.
**Conclusion:** All biosensitive compositional sequences (T1ρ-, T2-mapping, gagCEST) show significant age dependence, implying that cartilage tissue degradation and its collagen-proteoglycan matrix occurs before this is visible on standard morphological MRI and before irreversible damage occurs. Furthermore, this work demonstrates that the measurement of biosensitive MRI techniques is feasible even in small joints and that T1ρ-mapping, reflecting loss of proteoglycans, might be the best compositional sequence for predicting early cartilage ageing.
**Limitations:** Due to ethical reasons, a histological correlation was not performed.
**Ethics committee approval:** The ethics committee of the Heinrich-Heine-University approved this study.
**Funding for this study:** No specific funding for this study. The first author (MF) was supported by a grant from the Heinrich-Heine-University Düsseldorf.
**Author Disclosures:**
Ludger Radke: Nothing to disclose
Lena Marie Wilms: Nothing to disclose
Anja Müller-Lutz: Nothing to disclose
Miriam Frenken: Nothing to disclose
Daniel B. Benjamin Abrar: Nothing to disclose

### RPS 2210-8

**Dual-energy CT collagen density mapping of lower limb tendons in assessment on chronic tendinopathy: a proof-of-concept study**

*M. H. Lerchbaumer*, S. Kim, F. Halfter, T. Hees, C. Gwinner, B. Hamm, T. Diekhoff; Berlin/DE
*(markus.lerchbaumer@charite.de)*

**Purpose:** The study aimed to evaluate differences in collagen density detected by dual-energy computed tomography (DECT) of lower limb tendons with proven chronic tendinopathy in comparison to signal intensity (SI) on magnetic resonance imaging (MRI).
**Methods or Backround:** Preliminary data from a prospective study included 22 affected tendons in 17 patients (15 patellar tendons, 7 Achill tendons) who underwent bilateral DECT and MRI at the same time. Collagen density maps were reconstructed from the DECT datasets using three-material decomposition. Densities were measured in regions of interest (ROIs) placed in the affected area of localised structural damage (a) and whole tendon on a transversal plane (b) and compared to SI on PDfs-MRI in the same ROI. Normal values were assessed in the regular tendon (contralateral or non-affected area).
**Results or Findings:** Median collagen density was significantly lower in affected tendons compared to healthy tissue (a: 5.15 [IQR, 0-21.0] HU, b: 71.3 [IQR, 42.2-85.8] HU vs healthy 106.8 [IQR, 102.8-110.6] HU; all p<0.001), while MRI showed a corresponding higher SI (a: 71.3 [IQR, 42.3-85.8], b: 21.7 [IQR, 13.8-29.7] vs healthy 4.9 [IQR, 3.3-6.5]; all p<0.001). The collagen density of affected tendons showed a high negative correlation to SI on MRI for both measurements (a: -0.782, p<0.001; b: -0.776, p<0.001). Normal tendon tissue showed values of >100 HU with a corresponding SI of <10 on MRI for all tendons.
**Conclusion:** Collagen density in DECT of lower limb tendons can detect tendon changes corresponding so SI on MRI. Further research is needed to understand these changes and the role of DECT in follow-up or prognostics.
**Limitations:** The study is limited by showing the first results of a single-centre study and by the rare literature regarding DECT in assessing lower limb tendons.
**Ethics committee approval:** The ethics committee of Charité University Medicine approved this study.
**Funding for this study:** No funding was received for this study.
**Author Disclosures:**
Bernd Hamm: Nothing to disclose
Suchung Kim: Nothing to disclose
Torsten Diekhoff: Nothing to disclose
Finn Halfter: Nothing to disclose
Markus Herbert Lerchbaumer: Nothing to disclose
Tilman Hees: Nothing to disclose
Clemens Gwinner: Nothing to disclose

**08:00-09:00** **Research Stage 4**

### Research Presentation Session: Neuro

### RPS 2211

### Brain and spine trauma and haemorrhage

**Moderator**
A. Mazumder; London/UK

### RPS 2211-2

**Accuracy of the AI service for detection of intracranial haemorrhages on brain CT: results of a six-month multicentre clinical trial**

*A. N. Khoruzhaya*, K. M. Arzamasov, E. I. Kremneva, A. Vladzymyrskyy; Moscow/RU
*(khoruanna69@yandex.ru)*

**Purpose:** To evaluate the performance accuracy of the AI service for detecting intracranial haemorrhages (ICH) on brain CT during a six-month multicenter clinical trial.
**Methods or Backround:** Within the framework of the Moscow Experiment on Computer Vision in Radiology (ClinicalTrials.gov Identifier: NCT04489992), an AI service with declared metric values (sensitivity - 0.89, specificity - 0.96, ROC AUC - 0.96) was connected to the CT devices of 56 hospital medical organisations on 18th April 2022. Data on the results of processing brain CTs by the AI service were collected until 30th September 2022. 552 CT scans from these data were randomly selected and analysed by three radiologists with more than three years experience in neuroradiology. The radiologists evaluated the actual presence of ICH (0/1) and the result provided by the AI service (response threshold 0.75). According to the results of the radiologist assessment (Ground Truth), the signs of ICH contained 173 CT studies (31%). ROC AUC was calculated using the tool https://roc-analysis.mosmed.ai/.
**Results or Findings:** Full agreement in the evaluation of brain CT scans by radiologists and the AI service was achieved in 55% (301 CT); partial agreement in 20% (110). The number of false positive responses from the AI service was 24% (134), false negative 1% (7). Thus the sensitivity was 95.9%, the specificity was 64.6%, and AUC was 0.93.
**Conclusion:** In terms of sensitivity during the clinical trial, the AI service exceeded the declared values but significantly lost in specificity. The obtained values of diagnostic accuracy metrics indicate the possibility of its effective application in practice.
**Limitations:** There were no limitations.
**Ethics committee approval:** No information provided by the submitter.
**Funding for this study:** Programme of the Moscow Healthcare Department “Scientific Support of the Capital's Healthcare” for 2020–2022: AAAA-A21-121012290079-2 supported this study.
**Author Disclosures:**
Elena Igorevna Kremneva: Nothing to disclose
Anton Vladzymyrskyy: Nothing to disclose
Kirill Mihajlovich Arzamasov: Nothing to disclose
Anna Nikolaevna Khoruzhaya: Nothing to disclose

### RPS 2211-3

**Fibre-specific white matter abnormalities in moderate to severe traumatic brain injury**

*Y. Jia*, N. Bourke, P. Jenkins, D. Sharp, T. Parker; London/UK
*(yj7218@ic.ac.uk)*

**Purpose:** White-matter neurodegeneration contributes significantly to the pathophysiology of traumatic brain injury (TBI), but the ability to resolve tract-specific alterations in vivo, particularly in crossing fibre regions, is currently limited. Fixel-based analysis (FBA) is a novel diffusion MRI analysis technique that assesses specific white-matter fibre populations within a voxel (known as fixels). This study aimed to evaluate FBA's performance in identifying tract-

Sunday specific white-matter abnormalities in chronic TBI patients, especially within crossing fibre regions.
**Methods or Backround:** Diffusion-weighted imaging was acquired for adults with chronic moderate to severe TBI (N=29; median time since injury 22.6 months) and age and gender-matched healthy controls (N=17). Whole-brain and tract-of-interest analyses were performed to compare differences in white-matter connectivity represented by fixel-wise metrics (fibre density (FD), fibre bundle cross-section (FC), and combined FD and FC (FDC)) between groups. Analyses were adjusted for age, sex, and intracranial volume.
**Results or Findings:** Widespread reductions were found in all fixel-wise metrics in TBI patients. There were major differences in the scale and distribution of these reductions between metrics. Importantly, FBA was able to resolve the distinct effects of TBI in a crossing fibre region at both the voxel and tract level, with reduced fixel-wise metrics in the genu of the corpus callosum and relative sparing of overlapping fibres from the left superior longitudinal fasciculus.
**Conclusion:** FBA identified variabilities in white-matter abnormalities in TBI patients. Crucially, FBA was able to resolve TBI-related tract-specific alterations even in crossing fibre regions, supporting further exploration of fixel-wise metrics as more specific biomarkers of white-matter neurodegeneration in TBI.
**Limitations:** Fixel-wise metrics diagnostic and prognostic accuracy require validation.
**Ethics committee approval:** Approved by the West London and GTAC NRES Committee (14/LO/0067)
**Funding for this study:** Data collection: The NIHR Professorship (NIHR-RP-011–048) was awarded to D.J.S further support from the NIHR Clinical Research Facility and Biomedical Research Centre at Imperial College Healthcare NHS Trust.
**Author Disclosures:**
YiFan Jia: Nothing to disclose
David Sharp: Nothing to disclose
Thomas Parker: Nothing to disclose
Peter Jenkins: Nothing to disclose
Niall Bourke: Nothing to disclose

### RPS 2211-4

### Injury Patterns of the spine following blunt trauma: a per-segment analysis of spinal structures and their detection rates in CT and MRI

L. D. Grünewald, *V. Koch*, C. Booz, I. Yel, S. Martin, T. Vogl;
Frankfurt am Main/DE

**Purpose:** To provide a detailed analysis of injury patterns of the spine following blunt trauma, and to establish the role of supplementary MRI by evaluating discrepancies in the detection rates of damaged structures in CT and MRI.
**Methods or Backround:** 216 patients with blunt trauma to the spine, who underwent CT followed by supplementary MRI, were included in this study. All acquired CT and MRI images were interpreted blind to clinical symptoms and injury mechanisms. The interpretation was performed using a dedicated catalogue of typical findings associated with spinal trauma and assessed for spinal stability using the AO classification systems.
**Results or Findings:** Lesions to structures associated with spinal instability were present in 31.0% in the cervical spine, 12.3% in the thoracic spine, and 29.9% in the lumbar spine. In all spinal segments, MRI provided additional information regarding potentially unstable injuries. Novel information derived from supplementary MRI changed clinical management towards surgery in 3.6% of patients with injury to the cervical spine. No change in clinical management came from novel information on the thoracolumbar spine. Patients with injuries to the vertebral body, intervertebral disc or spinous process were significantly more likely to benefit from supplementary MRI.
**Conclusion:** Supplementary MRI in patients who suffered blunt trauma to the spine detected additional injury and unstable lesions in most patients and almost all injured spinal segments. This changed clinical management towards surgery in 3.6% of patients with injury to the cervical spine. No change in clinical management resulted from additional information in the thoracolumbar spine.
**Limitations:** Preselection bias towards patients with injuries to the spine
**Ethics committee approval:** No information provided by the submitter.
**Funding for this study:** No funding was received for this study.
**Author Disclosures:**
Simon Martin: Nothing to disclose
Christian Booz: Speaker: Siemens Healthineers
Ibrahim Yel: Speaker: Siemens Healthineers
Thomas Vogl: Nothing to disclose
Vitali Koch: Nothing to disclose
Leon David Grünewald: Nothing to disclose

### RPS 2211-5

### Peculiarities of the brain metabolism in young subjects in the chronic post-contusion phase: 1H MRS study

*Z. Z. Rozhkova*, V. Kholin, I. Karaban'; Kiev/UA
*(zinayida.rozhkova@mirumclinic.com)*

**Purpose:** We propose biomarkers for determining the severity of the mental deficit in young subjects in the chronic post-contusion phase.
**Methods or Backround:** Two groups of subjects were studied by MRI and MRS using a 1.5T Magnetom Sola (SIEMENS). 1-st(PG) consists of 29 patients (20-51yo,4 f,25 m) who underwent rehabilitation therapy after brain contusion that occurred 3-6 months ago, and the 2-nd (VG) -12 volunteers (23-40yo,5f,7m). Spectra are recorded in the prefrontal anterior cingulate lobe (PACL), internal capsule (IC), and in cerebral peduncles (CP) .
**Results or Findings:** From the spectra, the ratios: NAA/Cho, NAA/Cr, Cho/Cr, mIns/Cr, Lip/Cr, Lac/Cr, Glx/Cr are obtained. The content of cerebral metabolites and the ratios are significantly altered in regions that appeared normal on MRI. In pyramidal tracts (IC, CP) in PG we found significant reduction of NAA/Cr, Lip/Cr, Lac/Cr, Glx/Cr in comparison with VG. In CI: NAA/Cr=1.8285+-0.43(PG) vs 1.9370+-0.12(VG), in CP:NAA/Cr=2.1251+-0.49(PG) vs 1.8168+-0.21(VG). We observed a higher NAA/Cho ratio in PG, in PACL, and in IC, and in CP, which reflects both an increase of Cho and decrease of NAA. Alteration of Glx content in pyramidal tracts are more pronounced, than other metabolites:(7.31+-1.9), (11.6+-1.7) in PG, and VG. Glx/Cr decrease with the grade of affective behavior disorders. Significant correlation between the Lip/Cr and clinical status were found in pyramidal tracts, and in PACL. The ratio of mIns/Cr were reduced in PACL in PG in comparison with VG:(0.89+-0.14), (1.35+-0.32), respectively.
**Conclusion:** MRS is a very useful tool for evaluation of major changes in metabolite levels in various regions of the brain in the chronic phase after brain contusion. There is strong correlation between altered metabolite content and initial severity of brain injury after contusion, and also late clinical outcome.
**Limitations:** Our data are initial.
**Ethics committee approval:** No information provided by the submitter.
**Funding for this study:** Not applicable.
**Author Disclosures:**
Irina Karaban': Nothing to disclose
Zinayida Z Rozhkova: Nothing to disclose
Victor Kholin: Nothing to disclose

### RPS 2211-6

### Anticoagulation and risk of traumatic brain injury: overly generalised and poorly understood

*K. Kow*, H. Hassan, R-E. Chung, M. Majeed, K. S. Tsang; London/UK
*(kevin.kow.kkjh@doctors.org.uk)*

**Purpose:** To compare the presence and nature of anticoagulation and the likelihood of traumatic brain injury in patients presenting with head injury
**Methods or Backround:** All CT head requests for head injuries using NICE guidelines were analysed between October 2021 to January 2022. Patients who were stated to have anticoagulation were identified. Patients on multiple anticoagulants were excluded. The remaining were sub-divided based on class of anticoagulation. Findings were analysed to identify presence of extracranial or intracranial abnormalities, and compared to nature and dose of anticoagulation to identify trends linking various factors.
**Results or Findings:** 1514 CT heads were carried out under the NICE head injury pathway. Of these, 646 (47%) were stated as being on anticoagulation. six were excluded due to being on multiple forms of anticoagulation. Direct-acting oral anticoagulants (DOACs) were the most commonly used, followed by Warfarin, antiplatelets and low molecular weight heparin (LMWH) in descending order. There was no statistically significant association between anticoagulation and the presence of abnormal CT head findings. No specific type of anticoagulation shows significant association with the risk of intracranial injury except for Apixaban which demonstrated a statistically significant reduced likelihood of abnormal findings compared to non-anticoagulated patients ($p<0.05$).
**Conclusion:** Anticoagulation is increasingly prevalent in the UK and a key indication for urgent imaging; however, it does not necessarily increase chances of traumatic brain injury. Apixaban demonstrated significantly reduced likelihood of abnormal CT findings compared to non-anticoagulated patients. While this does not indicate that Apixaban is protective, it suggests that presence of anticoagulation therapy alone should not be a determinant of patient management. Not all forms of anticoagulation are equivalent and decisions should be based on type of anticoagulation rather than merely presence of it.
**Limitations:** Power calculations are pending.
**Ethics committee approval:** No information provided by the submitter.

**Funding for this study:** No funding was received for this study.
**Author Disclosures:**
Kai Sun Tsang: Nothing to disclose
Rui-En Chung: Nothing to disclose
Kevin Kow: Nothing to disclose
Muhammad Majeed: Nothing to disclose
Hussein Hassan: Nothing to disclose

### RPS 2211-7

**Longitudinal profile of axonal damage following poor grade subarachnoid haemorrhage: concordance of cerebral tau and diffusion tensor MRI**
*L. Lenhart*, C. Scherfler; Innsbruck/AT

**Purpose:** Elevated tau protein, a microtubule-stabilising protein assembling axonal bundles, was associated with poor long-term functional outcome in poor-grade aneurysmal subarachnoid hemorrhage (aSAH) patients. To investigate the extent of axonal damage, we quantified the spatial and temporal dimensions of microstructural integrity by using diffusion tensor imaging (DTI) and correlated the results with cerebral-microdialysis tau levels and functional outcome parameters of patients with poor-grade aSAH.
**Methods or Backround:** DTI at 3T was performed in poor-grade aSAH patients at three weeks (n=11) and 12 months (n=10) after ictus and compared with 12 age- and gender-matched healthy subjects using statistical parametric mapping (SPM). Mean DTI values within the 1-cm radius of the catheter tip were measured by region of interest analysis to determine its interaction with focal tau levels.
**Results or Findings:** SPM localised significant DTI abnormalities of voxel clusters surrounding the temporal horn and the lateral ventricles bilaterally at the three-week time-point and in the areas of the superior longitudinal fascicles at 12-month follow-up when compared to healthy subjects. Significant fractional anisotropy decreases without mean diffusivity increases at 12 months were also evident in the white matter compartment of the temporal lobe, middle cerebellar peduncle as well as the basal ganglia, and correlated with tau levels and parameters of functional outcome.
**Conclusion:** The study revealed widespread axonal damage that deteriorated further until the 12-month follow-up and was associated with poor functional and cognitive outcome, suggesting DTI as a surrogate marker to predict progression of axonal injury in patients following poor-grade aSAH.
**Limitations:** A potential limitation of this work was the small sample size.
**Ethics committee approval:** No information provided by the submitter.
**Funding for this study:** This study received funding from the Austrian Science Fund (FWF) under Project no. KLI375.
**Author Disclosures:**
Christoph Scherfler: Nothing to disclose
Lukas Lenhart: Nothing to disclose

**09:30-11:00** **Research Stage 1**

### Research Presentation Session: Breast

### RPS 2302

### Ways towards personalised breast screening

**Moderator**
V. Martinez de Vega; Madrid/ES
*(Vicente.martinez@quironsalud.es)*

### RPS 2302-2

**Health4Her: implementation of an alcohol brief intervention among women attending a BreastScreen service**
*D. J. Lockie*[1], M. E. Clemson[1], J. Grigg[2], D. Lubman[2]; [1]Ringwood East/AU, [2]Melbourne/AU
*(lockie.darren@gmail.com)*

**Purpose:** Alcohol is a major modifiable risk factor for breast cancer in women, yet awareness of this risk has remained surprisingly low and health information about it is still not systematically provided in healthcare environments. This study aims to understand the need for, acceptability and effectiveness of an alcohol brief intervention offered in a BreastScreen setting.
**Methods or Backround:** Phase I: BreastScreen clients (n=422) were surveyed to gain an understanding of their alcohol consumption levels, knowledge of breast cancer risk factors, information needs, and acceptability of alcohol health promotion. Phase II: an iPad-delivered animated intervention was offered to women after their mammogram. Women were randomised to receive four minutes of an alcohol brief intervention plus three minutes of lifestyle health promotion (active arm, n=279), or only lifestyle health promotion (control arm, n=279). Assessment at baseline and four weeks.
**Results or Findings:** At baseline, awareness of the alcohol/breast cancer link was low (<20%). At four weeks, there was a significantly greater increase in the proportion of active (45.6%, 95% CI 37.8, 53.4) rather than control participants (18.1%, 95% CI 10.2, 25.9) accurately identifying alcohol as a breast cancer risk factor (diff=27.6%, 95% CI 18.9, 36.2; p<0.001), as well as improving alcohol literacy more broadly. Over half of the participants (60%) reported that they shared the health information they had learned with family members/friends, and the BreastScreen setting was the most appropriate setting for receiving this information.
**Conclusion:** This study supports implementing alcohol brief interventions in the BreastScreen setting. Results demonstrate the benefits of this highly scaleable model of alcohol health promotion, with the potential to improve alcohol literacy and reduce harmful alcohol use among a large population of women who stand to benefit most.
**Limitations:** Not powered to investigate alcohol consumption.
**Ethics committee approval:** Eastern Health
**Funding for this study:** VicHealth and Eastern Health supported this study.
**Author Disclosures:**
Jasmin Grigg: Grant Recipient: NHMRC grants
Michelle Elizabeth Clemson: Grant Recipient: NHMRC grants
Dan Lubman: Grant Recipient: NHMRC grants
Darren J. Lockie: Grant Recipient: NHRMC grants

### RPS 2302-3

**A clinical risk model for personalised screening and prevention of breast cancer**
*M. Eriksson*[1], K. Czene[1], E. F. Conant[2], P. Hall[1]; [1]Stockholm/SE, [2]Philadelphia, PA/US

**Purpose:** Risk assessment of breast cancer using image-derived AI models has shown higher discriminatory performance than traditional lifestyle/familial-based risk models. The long-term performance of an image-derived AI risk model incorporating lifestyle and familial risk factors is yet to be investigated.
**Methods or Backround:** Based on a mammographic screening cohort conducted since 2010 in Sweden, ages 40-74, a nested case-control study was performed, including 1,661 incident breast cancers and 7,913 controls, matched on the year of study entry. The imaging-only AI model extracted mammographic features (density, microcalcifications, masses, left-right breast asymmetries of these features) and age from baseline mammograms. The lifestyle/familial-expanded model also included lifestyle and familial risk factors. Absolute risks were estimated using the two models and the clinical Tyrer-Cuzick model. Model performances were compared across the 10-year follow-up.
**Results or Findings:** The AUCs of the lifestyle/familial-expanded AI risk model ranged from 0.79 (95%CI 0.74-0.83) to 0.68 (95%CI 0.67-0.70) for breast cancers developed 1-10 years after study entry. The corresponding AUCs were 0.76 (95%CI 0.71-0.81) to 0.66 (95%CI 0.64-0.67) for the imaging-only AI risk model and 0.67 (95%CI 0.61-0.74) to 0.62 (95%CI 0.60-0.63) for Tyrer-Cuzick. The increased performance of the lifestyle/familial-expanded AI model was observed in multiple risk subgroups and cancer subtypes. Among the ~7% of women at high risk, the positive predictive value was 6.1% using the lifestyle/familial-expanded model compared to 5.7% using the imaging-only risk model, p<0.01, and Tyrer-Cuzick 5.2%, p<0.01.
**Conclusion:** The lifestyle/familial-expanded AI risk model showed the highest overall performance for both long-term and short-term risk assessment and multiple subgroups and cancer subtypes compared to the imaging-only model and Tyrer-Cuzick. Image-derived AI models can identify women who may benefit from personalised screening and/or risk-reducing intervention.
**Limitations:** The study is carried out in the Swedish screening setting.
**Ethics committee approval:** The Karolinska Institute ethics committee approved this study.
**Funding for this study:** The study is funded by Märit and Hans Rausing Initiative Against Breast Cancer.
**Author Disclosures:**
Mikael Eriksson: Patent Holder: iCAD
Kamila Czene: Nothing to disclose
Per Hall: Patent Holder: iCAD
Emily F. Conant: Advisory Board: iCAD

### RPS 2302-4

**Which women have the most significant benefit of digital breast tomosynthesis? An automatic density software analysis in the Malmö Breast Tomosynthesis Screening Trial**
*J. Olinder*, D. Förnvik, K. Johnson, S. Zackrisson; Malmö/SE
*(jakob.olinder@med.lu.se)*

**Purpose:** The study aimed to evaluate which women, based on breast density subgroups, had the greatest benefit of digital breast tomosynthesis (DBT) compared to digital mammography (DM) in the prospective Malmö Breast Tomosynthesis Screening Trial (MBTST).
**Methods or Backround:** The MBTST (n=14 848) compared one-view DBT and two-view DM with a consensus meeting before the recall. Breast density was assessed by the automatic software Laboratory for Individualized Breast Radiodensity Assessment (LIBRA). Sensitivity, specificity and cancer detection rates were compared between DBT and DM by breast density quintiles of per cent breast density (PBD). The association between breast density and cancer detection was analysed with logistic regression, adjusted for age <55 and ≥55 years and previous screening participation.
**Results or Findings:** In total, 14 730 exams were included in the analysis. Sensitivity was higher and specificity lower for DBT compared to DM for all density subgroups. The highest PBD quintile showed the largest difference in sensitivity and specificity: 81.1% (95% confidence interval (CI) 65.8-90.5) vs 43.2% (95% CI 28.7-59.1), p<0.001 and 95.5% (95% CI 94.7-96.2) vs 97.2% (95% CI 96.6-97.8), p<0.001, respectively. PBD quintile was positively associated with cancer detected with DBT, adjusted odds ratio 1.24 (95% CI 1.09-1.42, p=0.001).
**Conclusion:** Women with the highest breast density had, in terms of increased sensitivity, the greatest benefit of DBT compared to DM at the cost of slightly lower specificity. Breast density was positively associated with cancer detection with DBT. The greater benefit of DBT, compared to DM, for women in the densest breast category could be applicable in future risk-based breast cancer screening.
**Limitations:** The specificity of DM could be overestimated since DBT images were available at consensus meetings. The original trial was not powered for subanalyses.
**Ethics committee approval:** The Lund University Ethics Committee approval and written informed consent were obtained.
**Funding for this study:** The funding was provided by the ALF grant, Swedish Cancer Society.
**Author Disclosures:**
Kristin Johnson: Speaker: KJ has received speaker´s fee from Siemens Healthineers.
Daniel Förnvik: Nothing to disclose
Jakob Olinder: Nothing to disclose
Sophia Zackrisson: Speaker: SZ has received speaker's fees from: Siemens Healthcare AG, Pfizer, Bayer AG

### RPS 2302-5

**Risk-based breast screening (RIBBS) in young women: stratification of population and cancer detection rate (CDR) from recruitment**

*F. Caumo*, E. Baldan, E. Bezzon, G. Ciriello, A. Coran, C. Dal Bosco, I. Polico, D. Ruggieri, G. Gennaro; Padua/IT

**Purpose:** The study aimed to provide stratification and CDR from recruitment of a personalised breast screening programme for (45-49y) women.
**Methods or Backround:** 10,270 women (aged 45) were invited to the Veneto Institute of Oncology (IOV) to participate in a personalised breast screening. All women underwent two-view tomosynthesis (DBT), which was processed by validated software to calculate volumetric breast density (VBD). Women with mean VBD>25% received supplemental breast US. The Tyrer-Cuzick risk model, including VBD, was used to obtain the individual lifetime risk (LTR), which allowed women to be stratified into three risk profiles: low-risk (LR) (LTR≤17%), intermediate-risk (IR) (17%<LTR≤30%), high-risk (HR) (LTR>30%). The risk category was used to determine the periodicity of the subsequent screening event: biennial for LR women and annual for IR and HR women. CDRs were compared between density and risk categories. P-value<0.05 was considered statistically significant.
**Results or Findings:** 18.5% of women (1903/10270) had dense breasts and received supplemental US. Most of the women were at low-risk (5942/10270=57.9%), while the remaining 41.9% were respectively at intermediate (3452/10270=33.6%) and high-risk (876/10270=8.5%). Final stratification for the subsequent screening rounds was: 1y-DBT in 2711 women (26.4%), 1y-( DBT+US) in 1617 (15.7%), 2y-DBT in 5656 (55.1%), and 2y-(DBT+US) in 286 (2.8%). Overall CDR was 5.6/1000 with a non-significant difference between dense and non-dense breasts (dense: 7.9/1000;non-dense: 5.1/1000; p=0.1507) and a significant difference between LR and IR+HR women (LR: 3.0/1000; IR+HR: 9.2/1000; p<0.0001).
**Conclusion:** The increased probability of breast cancer in women at intermediate and high-risk confirms the need for personalised risk-based screening models.
**Limitations:** Only incident cancers.
**Ethics committee approval:** The approval can be found under the number RIBBS-2019.
**Funding for this study:** The study is funded by a regional grant (968 DGR 06_07_2018).
**Author Disclosures:**
Francesca Caumo: Nothing to disclose
Enrica Baldan: Nothing to disclose
Elisabetta Bezzon: Nothing to disclose
Gisella Gennaro: Nothing to disclose
Ilaria Polico: Nothing to disclose
Alessandro Coran: Nothing to disclose
Domenico Ruggieri: Nothing to disclose
Chiara Dal Bosco: Nothing to disclose
Giovanna Ciriello: Nothing to disclose

### RPS 2302-6

**Mammographic features of screen-detected breast tumours less than 10mm and risk of breast cancer death**

*N. Moshina*, M. Larsen, C. Flåt Aglen, S. Hofvind; Oslo/NO
*(namo@kreftregisteret.no)*

**Purpose:** The association between mammographic features and breast cancer-specific survival has not been entirely understood. To increase radiologists' attention to the deadly tumours on screening mammograms, more evidence is needed. We aimed to analyse the association of mammographic features and the risk of breast cancer death among women diagnosed and treated for invasive screen-detected breast cancer<10mm in diameter.
**Methods or Backround:** We included data from 5152 women aged 50-71 diagnosed with invasive breast cancer of <10 mm after participation in BreastScreen Norway, 1996-2020. Data on mammographic features (mass, spiculated mass, architectural distortion, asymmetric density, density with calcification and calcification alone), tumour diameter, and cause of death were obtained from the cancer registry. Cox regression was used to estimate hazard ratios (HR) with 95% confidence intervals (CI) for breast cancer death by mammographic features, adjusting for age and tumour diameter.
**Results or Findings:** The mean age at diagnosis was 60.8 (standard deviation, SD=5.8). The distribution of features was: mass 28.4%, spiculated mass 30.4%, architectural distortion 2.2%, asymmetric density 13.6%, density with calcification 7.0%, and calcification alone 18.4%. The number of breast cancer deaths was 106, and the mean time from diagnosis to death was 8.7 (SD=5.0) years. The adjusted HR for breast cancer death was 2.16 (95% CI: 1.15, 4.07) for calcification alone and 2.09 (95% CI: 1.13, 3.88) for density with calcification, using spiculated mass as reference.
**Conclusion:** Screen-detected invasive cancers of <10mm presenting as calcification alone and density with calcification were associated with a higher risk of breast cancer death than those presented as spiculated mass. Mammographic calcifications should be considered a factor associated with a poor prognosis in women with small screen-detected invasive cancers.
**Limitations:** None were identified.
**Ethics committee approval:** No information provided by the submitter.
**Funding for this study:** No funding was received for this study.
**Author Disclosures:**
Camilla Flåt Aglen: Nothing to disclose
Nataliia Moshina: Nothing to disclose
Solveig Hofvind: Nothing to disclose
Marthe Larsen: Nothing to disclose

### RPS 2302-7

**Recall, screen-detected and interval breast cancer for women prevalently screened with digital breast tomosynthesis vs digital mammography**

Å. S. Holen[1], M. B. Bergan[1], N. Moshina[1], *H. S. Aase*[2], S. Hofvind[1]; [1]Oslo/NO, [2]Bergen/NO

**Purpose:** In this cohort study, following the randomised controlled Tomosynthesis Trial in Bergen (To-Be), women were screened with digital breast tomosynthesis (DBT). We compared rates of recall, screen-detected and interval breast cancer for women prevalently screened with DBT with women prevalently screened with digital mammography (DM) in the screening round prior to the To-Be trial.
**Methods or Backround:** The study population included 4562 women prevalently screened with DBT in 2018-2019 and 4089 women prevalently screened with DM in 2014-2015 in Bergen, Norway. All women were followed for two years for interval breast cancer. We compared rates of recall, screen-detected and interval breast cancer for these two groups of women using descriptive statistics.
**Results or Findings:** The recall rate was 8.6% (95% confidence interval, CI: 7.7-9.4%) for women prevalently screened with DBT and 6.0% (95% CI: 5.3-6.8%) for DM. The rate of screen-detected breast cancer was 9.2 per 1000 screened (95% CI: 6.6-12.4/1000) for DBT compared to 7.8 per 1000 (95% CI: 5.6-11.0/1000) for DM. The rate of interval breast cancer was 1.5 per 1000 screened (95% CI: 0.6-3.1/1000) for DBT and 2.9 per 1000 (95% CI: 1.5-5.1/1000) for DM.
**Conclusion:** The recall rate was significantly higher for women prevalently screened with DBT compared to DM. The rate of screen-detected breast cancer was higher, while the rate of interval breast cancer was lower for women prevalently screened with DBT compared to DM. However, these rates did not reach statistically significant differences.
**Limitations:** A small trial population and few cancers might have hampered our ability to detect any significant differences between the groups.
**Ethics committee approval:** The Regional Committees for Medical and Health Research Ethics approved this study. Their approval can be found under the number 2015/424.
**Funding for this study:** The Research Council of Norway and the Norwegian Cancer Society funded this study.
**Author Disclosures:**
Hildegunn Siv Aase: Nothing to disclose
Nataliia Moshina: Nothing to disclose
Marie Burns Bergan: Nothing to disclose
Åsne Sørlien Holen: Nothing to disclose
Solveig Hofvind: Nothing to disclose

### RPS 2302-8

**Clinical feasibility of breast CT as an opportunistic screening modality: a prospective trial of 1614 patients in Switzerland**

*A. Landsmann*, J. Wieler, N. Berger, M. Marcon, A. Boss; Zurich/CH
*(annalandsmann@gmx.de)*

**Purpose:** This prospective single-centre trial evaluated the clinical feasibility of photon-counting breast CT (BCT) in breast cancer (BC) detection in an opportunistic screening cohort in Switzerland.
**Methods or Backround:** 1614 women were screened with BCT between July 2019 and August 2022. All patients signed informed consent. Women with dense breasts (1113) received supplemental ultrasound (sUS). Examinations were evaluated based on the BI-RADS catalogue. Cancer detection rate, positive predictive value (PPV), and false negative rate were calculated and compared to the fifth national monitoring report for Swiss Cancer Screening.
**Results or Findings:** 22 cancers (two carcinomas in situ and 20 invasive breast cancers) were detected with BCT and sUS, yielding a higher detection rate (1.4%) compared to MG with sUS (0.7%). Four cancers were only visible in sUS in women with dense breasts. The two carcinomas in situ presented as microcalcifications in BCT without correlating findings in sUS. PPV for BCT and MG was 66.6% and 8.4%, respectively. The false positive rate for BCT was 0.8%, compared to 7.4% in MG.
**Conclusion:** Breast CT can serve as an alternative screening method for BC detection with a higher cancer detection rate than mammography screening programmes, resulting in higher patient compliance in women with a fear of conventional mammography.
**Limitations:** Participating women did not receive a supplemental MG to compare with the gold-standard for BC screening.
**Ethics committee approval:** The approval was given by Kantonale Ethikkomission, Zurich, and can be found under the number and title BASEC-Nr. 2018-01694, "Imaging quality and potential clinical relevance of breast computed tomography in-vivo: a single-center, prospective study".
**Funding for this study:** No funding was received.
**Author Disclosures:**
Andreas Boss: Nothing to disclose
Jann Wieler: Nothing to disclose
Nicole Berger: Nothing to disclose
Anna Landsmann: Nothing to disclose
Magda Marcon: Nothing to disclose

### RPS 2302-9

**A survey by the European Society of Breast Imaging on breast imaging readers' preferences regarding quality assurance (QA) measures in screening and diagnostic mammography**

*E. Michalopoulou*[1], E. M. M. Fallenberg[2], P. Clauser[3], Y. Chen[1];
[1]Nottingham/UK, [2]Munich/DE, [3]Vienna/AT
*(Eleni.Michalopoulou@nottingham.ac.uk)*

**Purpose:** Although several QA documents are now recommended by European bodies or national organisations, the QA in mammography is quite heterogenous between countries and not always actively implemented across Europe. The European Society of Breast Imaging launched a survey among its members to collect information on breast imaging readers' preferences regarding QA measures in mammography.
**Methods or Backround:** An online survey consisting of 25 close- and open-ended questions was distributed to all EUSOBI members and national breast radiology bodies from Europe. The questions were designed to anonymously collect responders' demographic characteristics, information on their mammography workload and data about QA for mammography reporting in their country. Descriptive statistical tests, $\chi 2$ test and linear regression were conducted to analyse the data.
**Results or Findings:** A total of 251 breast imaging readers completed the survey from April to July 2022. Most of them were providing both breast screening and symptomatic services (54.6%) and were working in an academic hospital environment (33.9%). For most responders (53%), QA measures were established in their workplace, 27.1% of whom were working in a private setting. Although less than one-third (28.3%) had to participate in a performance test, the vast majority (75.7%) agreed that a mandatory test every two years would improve their skills.
**Conclusion:** QA measures were in place for all responders working in screening and diagnostic mammography in order to evaluate their breast imaging performance. Although there were substantial differences between countries, the importance of having QA in the workplace and implemented was widely acknowledged by breast imaging readers.
**Limitations:** The questionnaire was kept short of encouraging breast imaging readers' participation, preventing the capture of further information regarding radiology quality assurance and standards. Additionally, responders were not evenly distributed among European countries.
**Ethics committee approval:** The study was approved by the EUSOBI Board. The requirement for ethical approval was waived after discussion with the University of Nottingham research and development team because this study was deemed to represent a survey.
**Funding for this study:** Not applicable.
**Author Disclosures:**
Eva Maria M. Fallenberg: Nothing to disclose
Eleni Michalopoulou: Nothing to disclose
Yan Chen: Nothing to disclose
Paola Clauser: Nothing to disclose

### RPS 2302-10

**Effect of a translated invitation letter on mammography screening attendance among immigrants from Arabic-speaking countries: a randomised controlled trial**

*J. E. Thy*, S. Sagstad, Å. S. Holen, M. B. Bergan, S. Hofvind; Oslo/NO
*(joty@kreftregisteret.no)*

**Purpose:** Language has been identified as a barrier among immigrants to attend cancer screening, as well as other health care services in general. Several studies have shown substantially lower attendance in mammographic screening among immigrants vs non-immigrants, particularly among immigrants from non-Western countries compared to immigrants from Western countries. The main aim of this randomised controlled trial was to explore attendance to mammographic screening among immigrants from Arabic-speaking countries receiving an invitation in a major official language of their country of birth.
**Methods or Backround:** The study population included all women with a registered immigration date and birth country of Iraq, Lebanon, Syria, Palestine, Egypt, Sudan, Algeria, Tunisia, and Morocco (Arabic language), invited to BreastScreen Norway between April 5th, 2021 and June 30th, 2022 (n=2647). The women were digitally randomised to an intervention or a control group. The control group received standard postal invitations in Norwegian only. The intervention group received the same invitation in Norwegian and a translated version in Arabic.
**Results or Findings:** The attendance rate was 47.0% (628/1336) in the intervention group and 45.3% (594/1311) in the control group (p=0.381). There were no differences between the groups when stratified by age or years since immigration.
**Conclusion:** Translated invitation letters to mammography screening had no effect on the attendance rate among immigrants from Arabic-speaking countries.
**Limitations:** We assumed that the women understood the official language of their country of birth.
**Ethics committee approval:** Regional Committee for Medical and Health Research Ethics approved this study.
**Funding for this study:** The study was funded by the Norwegian Cancer Society.
**Author Disclosures:**
Marie Burns Bergan: Nothing to disclose
Åsne Sørlien Holen: Nothing to disclose
Silje Sagstad: Nothing to disclose
Solveig Hofvind: Nothing to disclose
Jonas E. Thy: Nothing to disclose

### RPS 2302-11

**Self-reported quality of life for women with screen-detected and symptomatic breast cancer**

*N. Moshina*, M. Larsen, Å. S. Holen, S. Hofvind; Oslo/NO
*(namo@kreftregisteret.no)*

**Purpose:** The evidence on quality of life for women with screen-detected vs symptomatic breast cancer is sparse. We aimed to explore the quality of life and quality-adjusted life years (QALY) among Norwegian women by detection mode, screen-detected vs symptomatic cancer.
**Methods or Backround:** This retrospective cross-sectional study included 1206 women with screen-detected breast cancer in BreastScreen Norway and 1021 women diagnosed outside the screening programme, aged 50-69 at diagnosis, 2006-2017. Self-reported data on quality of life score (visual analogue scale 0-100) and index values (0-1) were extracted from the EQ-5D-

5L questionnaire. Linear regression was used to analyse the association between quality of life score and detection mode, adjusting for treatment, relapse and sociodemographic factors. QALY was estimated by summing up the index values for each year between the third and the 14th year since diagnosis, assuming all women would survive.
**Results or Findings:** The mean age at diagnosis was 59.9 (standard deviation, SD=5.7) years, time since diagnosis to assessment 7.6 (SD=3.4) years, and quality of life score 66.2 (SD=21.0) for women with screen-detected cancer. The values were 57.3 (SD=6.2) years, 8.0 (SD=3.4) years and 61.0 (SD=21.8) for women with symptomatic cancer. Women with symptomatic cancer had a lower adjusted quality of life score, -5.1 (95% confidence interval, -6.8; -3.4), compared to women with screen-detected cancer. Women with screen-detected and symptomatic cancer would experience 9.7 and 9.3 QALY, respectively, between the third and the 14th year since diagnosis.
**Conclusion:** The findings add to the benefits of organised mammographic screening.
**Limitations:** No limitations were recognised.
**Ethics committee approval:** The study was approved by the Regional Committees for Medical and Health Research Ethics (N28484).
**Funding for this study:** The study was funded by a grant from the Dam Foundation via the Norwegian Women's Public Health Association.
**Author Disclosures:**
Nataliia Moshina: Nothing to disclose
Åsne Sørlien Holen: Nothing to disclose
Solveig Hofvind: Nothing to disclose
Marthe Larsen: Nothing to disclose

### RPS 2302-12

**Automated breast ultrasound in comparison to 2D mammography, digital breast tomosynthesis, hand-held ultrasound in the detection of breast cancer: a cohort of 4500 examinations**
*A. Vourtsis*, A. Kachulis, T. N. Sergentanis; Athens/GR
*(athinavourtsi@yahoo.gr)*

**Purpose:** This prospective study aimed to assess the detection rate of breast cancer on automated breast ultrasound (ABUS) vs 2D mammography, digital breast tomosynthesis (DBT) and hand-held breast ultrasound (HHUS) as a supplemental screening tool in women with dense breasts.
**Methods or Backround:** A total of 4500 examinations on women with heterogeneously dense or extremely dense breasts (aged 52.2±9.5 years, range: 25-93 years) were included in this study. 2D mammography in the MLO projection, DBT in MLO and CC projection, ABUS and HHUS were obtained. Two dedicated breast radiologists interpreted all examinations independently. The study has been approved by the local institutional review board.
**Results or Findings:** Eighty-eight cancers (67 invasive carcinomas and 21 DCIS) were identified in the study cohort; of them, 74/88 cancers were detected on mammography (54 invasive and 20 DCIS). ABUS identified 75/88 cancers (67 invasive and eight DCIS); among them, 13 invasive carcinomas were not visualised on mammography yielding an incremental cancer detection rate of 2.9 invasive cancers per 1000 examinations. Seventy-eight of 88 cancers were detected on DBT (59 invasive and 19 DCIS). ABUS detected eight invasive carcinomas that were not seen on DBT, whereas 12 cases of DCIS not visualised on ABUS were found on DBT. Notably, 27.0% (20/74) of cancers seen on mammography and 24.4% (19/78) seen on DBT were DCIS. An excellent agreement was observed between ABUS and HHUS.
**Conclusion:** This study highlights the positive impact of both ABUS and HHUS in the detectability of invasive breast cancers in women with dense breasts.
**Limitations:** Screening and diagnostic cases have been collectively included in this study.
**Ethics committee approval:** The IRB approval number is 219/08-Sep-2019.
**Funding for this study:** The study was funded by Invenia ABUS Research Grant Proposals ("Invenia CFP"), GE, Investigator Sponsored Research Data Form (grant number 16745041186).
**Author Disclosures:**
Theodoros N. Sergentanis: Nothing to disclose
Athina Vourtsis: Advisory Board: General Electric (GE) Advisory Board: Volpara Solutions Research/Grant Support: General Electric (GE) Consultant: Research collaborator with Screen Point B.V. Company and with Q.V.C.A. company Speaker: General Electric (GE)
Aspasia Kachulis: Nothing to disclose

### RPS 2302-13

**Automated breast ultrasound (ABUS) - acquisition and reading times, image quality, recall rates and positive predictive value (PPV) over time: first experience from BRAID trial**
*I. Allajbeu*, K. Morris, M. Nanaa, N. Payne, P. L. Moyle, K. Charnley, F. J. Gilbert; Cambridge/UK
*(ia359@cam.ac.uk)*

**Purpose:** The study aimed to investigate the progress in ABUS acquisition and reading times, image quality, recall rates and PPV in the first three years of the Breast Risk Adaptive Imaging for Density (BRAID) trial.
**Methods or Backround:** ABUS exams were performed as part of the BRAID trial, an ongoing multi-centre study investigating the impact of supplementary screening in the early detection of breast cancer for women with dense breasts. An audit was undertaken to assess ABUS image quality and acquisition time during the initial six months and after one year's experience. A random sample of 20 examinations for each time point was selected and assessed using an image quality checklist by a radiographer and radiologist with four years of experience in ABUS. In addition, reading times, recall rates and PPV were calculated over a three-year period.
**Results or Findings:** The average acquisition time was reduced significantly from 60 minutes to 30 minutes at six months and 20 minutes at one year, with more frequent use of two-view protocols. There was a significant reduction in reporting times from 423 seconds initially to 227 seconds after one year. Image quality improved from acceptable at set up to very good/optimal after one year, with a consistent reduction in technical artefacts. Recall rates for baseline examinations did not change over time at 5.26%, but second-round recalls were significantly lower at 3.2%. PPV of baseline examinations increased from 27.8 % in the first year to 33.3 % in the second year.
**Conclusion:** Our findings indicate that adequate training with systematic image quality checks and a learning curve is necessary to improve acquisition technique and reader performance.
**Limitations:** The ongoing study, missing data different readers and radiographers experience through sites.
**Ethics committee approval:** Not applicable.
**Funding for this study:** The study was funded by Cancer Research UK and GE.
**Author Disclosures:**
Kirsten Morris: Nothing to disclose
Penelope Laura Moyle: Nothing to disclose
Fiona J. Gilbert: Research/Grant Support: Hologic Research/Grant Support: Volpara Research/Grant Support: GE healthcare Research/Grant Support: Bayer
Kate Charnley: Nothing to disclose
Iris Allajbeu: Nothing to disclose
Muzna Nanaa: Nothing to disclose
Nicholas Payne: Nothing to disclose

**09:30-11:00** **Research Stage 2**

### Research Presentation Session: Genitourinary

### RPS 2307

### Prostate cancer: detection, surveillance and relapse

**Moderator**
T. Barrett; Cambridge/UK

### RPS 2307-2

**Accuracy of magnetic resonance imaging-ultrasound fusion-guided and systematic biopsy of the prostate**
*T. in de Braekt*[1], S. van Rooij[1], A. Daniels-Gooszen[1], W. Scheepens[1], S. Bosch[2], J. Nederend[1]; [1]Eindhoven/NL, [2]Veldhoven/NL
*(thomas.idb@hotmail.com)*

**Purpose:** Multiparametric magnetic resonance imaging (mp-MRI) of the prostate with subsequent targeted biopsy of suspicious lesions have become indispensable in the diagnostic workup of prostate cancer. The objective was to investigate the diagnostic accuracy of systematic-biopsies, targeted-biopsies and the combination of both.
**Methods or Backround:** All patients undergoing systematic biopsies and TRUS-fusion-targeted biopsies from January 2013 to June 2022 were included. MRI was evaluated according to PI-RADS. The biopsy threshold was PI-RADS category ≥3. Systematic biopsies consisted of 8-12 cores, depending on prostate volume. TRUS-fusion biopsies were performed using Hitachi Preirus HI RVS. Detection rates for prostate cancer and clinically significant prostate cancer (Gleason Score ≥3+4) were compared between biopsy types using McNemar's test.
**Results or Findings:** In total, 867 patients were included, of which 615 had cancer, and 434 had clinically significant cancer. Overall detection rates were: PI-RADS 3 48% (99/208), PI-RADS 4 72% (314/434) and PI-RADS 5 90% (202/225). Detection rates for clinically significant cancer were 21% (44/208), 53% (229/434) and 72% (161/225), respectively. The combination of biopsy methods detected the most cases of clinically significant prostate cancer (p<0.001). TRUS-fusion biopsies detected significantly more clinically significant prostate cancer than systematic biopsies alone (43.1% vs 40.3%, p=0.046). Compared to the combination biopsies, TRUS-fusion-biopsies missed cancer in 14% (88/615) and significant cancer in 14% (60/434). Significant cancers were missed in patients with PI-RADS 3 in 32% (14/44), PI-RADS 4 in 15% (35/229), and PI-RADS 5 in 7% (11/161).
**Conclusion:** The combination of systemic biopsies and TRUS-fusion biopsy provides the highest detection of clinically significant prostate cancer. In PI-RADS 5 lesions, TRUS-fusion biopsy misses only 7% of significant cancer. In these cases, systematic biopsies could possibly only be performed in case of a negative TRUS-fusion biopsy result.
**Limitations:** Not applicable.
**Ethics committee approval:** Not applicable.
**Funding for this study:** Not applicable.
**Author Disclosures:**
Sanne van Rooij: Nothing to disclose
Wout Scheepens: Nothing to disclose
Alette Daniels-Gooszen: Nothing to disclose
Steven Bosch: Nothing to disclose
Joost Nederend: Nothing to disclose
Thomas in de Braekt: Nothing to disclose

### RPS 2307-3

### Role of the PI-QUAL score for prostate MRI quality in the detection of clinically significant cancer in equivocal lesions

*A. E. Woernle*, L. Dickinson, V. Kasivisvanathan, A. Freeman, A. Haider, J. Hines, C. M. Moore, C. Allen, F. Giganti; London/UK
*(zchaaew@ucl.ac.uk)*

**Purpose:** PI-QUAL is a 1-to-5 scoring system to assess prostate MR image quality. PI-QUAL 1-3 means a scan of suboptimal diagnostic quality. PI-QUAL 4-5 indicates a scan of better diagnostic quality. We applied PI-QUAL to assess image quality in the number of equivocal lesions (i.e., Likert 3) from our one-stop clinic.
**Methods or Backround:** We included 757 consecutive patients referred for urgent assessment on a cancer pathway. Scans were initially reported by one of seven expert radiologists, and the clinical decision was made according to that report. All scans were then rescored by another expert radiologist, who applied PI-QUAL criteria. We looked at the histology of equivocal (Likert 3) lesions from MR-targeted biopsies and differentiated between scans of suboptimal diagnostic quality (PI-QUAL 1-3) vs optimal diagnostic quality (PI-QUAL 4-5). Clinically significant prostate cancer (csPCa) was defined as a Gleason score ≥ 7.
**Results or Findings:** 261/757 (34%) scans were PI-QUAL 1-3, 68 (26%) of which scored Likert 3/5. 496/757 (66%) scans were PI-QUAL 4-5, 103 (21%) of which scored Likert 3/5. For equivocal lesions, PI-QUAL 1-3 scans harboured csPCa in 7/38 (18%) cases, while PI-QUAL 4-5 scans harboured csPCa in 17/46 (37%) of cases. In the equivocal population, DWI was the sequence most affected by suboptimal image quality (58/171), followed by T2-WI (17/171) and DCE (10/171).
**Conclusion:** Images of suboptimal quality (PI-QUAL 1-3) result in a greater number of i) equivocal lesions and ii) unnecessary biopsies. Optimal image quality (PI-QUAL 4-5) leads to fewer i) equivocal lesions and ii) unnecessary biopsies.
**Limitations:** Different MR scanners were used (1.5T vs 3T). A single reader was assessing PI-QUAL.
**Ethics committee approval:** Institutional review board approval at UCLH was obtained, and the results included in this study are part of an audit of MR scans and patient data under standard clinical care.
**Funding for this study:** The study was funded by the North Central London Cancer Alliance.
**Author Disclosures:**
Alexandre Edward Woernle: Nothing to disclose
Caroline M Moore: Research/Grant Support: Supported by Medical Research Council, UKNIHR, Movember, Prostate Cancer UK, Cancer Research UK and the EAU Research Foundation
Alex Freeman: Nothing to disclose
Louise Dickinson: Nothing to disclose
Aiman Haider: Nothing to disclose
Clare Allen: Nothing to disclose
John Hines: Nothing to disclose
Veeru Kasivisvanathan: Research/Grant Support: Academic Clinical Lecturer funded by the UK National Institute for Health Research (NIHR)
Francesco Giganti: Research/Grant Support: 2020 Young Investigator Award funded by the Prostate Cancer Foundation / CRIS Cancer Foundation Research/Grant Support: Funded by the UCL Graduate Research Scholarship and the Brahm PhD scholarship in memory of Chris Adams

### RPS 2307-4

### Inter-reader variability of manual segmentation of prostate volume on T2-weighted MRI: what an impact on PSA density?

*A. Colarieti*[1], A. Venturi[2], M. Cressoni[1], M. Interlenghi[2], C. Salvatore[3], I. Castiglioni[1], F. Sardanelli[1]; [1]Milan/IT, [2]Merano/IT, [3]Pavia/IT
*(anna.colarieti@gmail.com)*

**Purpose:** PSA density (PSAd), based on prostate volume (PV), is a decision-making parameter for patients with suspected prostate cancer (PCa). We assessed the inter-reader variability between three radiologists in prostate manual segmentation of MRI and its impact on PSAd.
**Methods or Backround:** We retrospectively analysed 68 treatment-naïve patients (aged 66.2±6.9 years) with an increased PSA (7.59±4.80 ng/mL) who underwent 1.5-T multiparametric MRI for whom biopsy or follow-up was available. Three radiologists (R1, R2, R3) manually segmented slice-by-slice the gland on T2-weighted images using a segmentation tool of a research platform (TRACE4Reasearch, DeepTrace Technologies). Segmentation time was recorded. Dice score coefficient, Welch's t-test, and 95% confidence interval (CI) were used. A threshold of ≥0.15ng/mL2 for PSAd positivity was used.
**Results or Findings:** Of 68 patients, 38 had biopsy-confirmed PCa, and the remaining 30/68 had a negative biopsy or follow-up. Mean segmentation time varied between 4 (R1 and R2) and 7 min (R3) per patient. Mean PSA was 7.37±3.69ng/mL in positive and 7.87±5.97ng/mL in negative patients (p=0.69); PSAd based on the PV obtained by R1 was 0.12±0.07 and 0.12±0.07ng/mL2, respectively (p=0.74). Couples of readers R1-R3 and R2-R3 showed a significantly different number of segmented slices (p<0.001) and PV (p<0.001); the DSC for the PV varied from 0.871 to 0.890. These variations affected PSAd-based diagnosis of 2/68 (2.9%) patients (95% CI 0.4–10.2%), both PCa-positive, diagnosed as negative using the PV segmented by R1 and R2.
**Conclusion:** Manual segmentation of PV is a time-demanding task; inter-reader variability, despite relatively high DSCs, can change PSAd-based diagnosis of a non-negligible number of patients. Artificial intelligence-based methods for automatic prostate segmentation are welcome to save time and overcome inter-reader variability.
**Limitations:** This is a single-center study.
**Ethics committee approval:** Not applicable.
**Funding for this study:** No funding was received for this study.
**Author Disclosures:**
Anna Colarieti: Nothing to disclose
Isabella Castiglioni: Owner: Castiglioni declares to own DeepTrace Technologies S.R.L shares
Francesco Sardanelli: Nothing to disclose
Christian Salvatore: CEO: Salvatore declares to be CEO of DeepTrace Technologies S.R.L., a spinoff of Scuola Universitaria Superiore IUSS, Pavia, Italy
Massimo Cressoni: Nothing to disclose
Alessandro Venturi: Nothing to disclose
Matteo Interlenghi: Owner: Interlenghi declares to own DeepTrace Technologies S.R.L shares

### RPS 2307-5

### Diagnostic accuracy of PI-RADS 4 upgraded from PI-RADS 3 as a consequence of positive DCE MRI in prostate peripheral zone lesions. Towards two new subgroups: PI-RADS 3F and PI-RADS 3B

E. Messina, *M. Pecoraro*, M. Bicchetti, L. Laschena, C. Catalano, V. Panebianco; Rome/IT
*(pecoraro.martina1@gmail.com)*

**Purpose:** The primary aim of the study was to evaluate the diagnostic performance of MRI and PI-RADS score in detecting clinically significant prostate cancer (csPCa) in patients with peripheral lesions scored as PI-RADS 4 according to DWI/ADC patterns, regardless of contrast enhancement, and PI-RADS 4 up-graded from PI-RADS 3 as a consequence of positive DCE (PI-RADS 3up). Moreover, to determine clinical and radiological features associated with csPCa.
**Methods or Backround:** This observational prospective study enrolled 389 patients, directed to baseline multiparametric MRI and, if positive, to MRI-directed biopsy. MRI and PI-RADS score performance was assessed, differentiating the conventional (c)PI-RADS (PI-RADS 3up considered as PI-RADS 4) from the experimental (e)PI-RADS (PI-RADS 3up considered as PI-RADS 3). Univariate and multivariable analyses were implemented to determine features independently correlated to csPCa.
**Results or Findings:** Considering csPCa diagnosis, cPI-RADS and ePI-RADS show, respectively, an AUC of 0.78 and 0.82, a sensitivity of 98.5% and 90.9%, specificity of 44.7% and 67.7%, PPV of 47.8 % and 59.1 %, NPV of 98.3 % and 93.5 %, and an accuracy of 63.0% and 75.6%. In the multivariable logistic regression model, the variable showing independent correlation with csPCa were age (p<0.002), PSA density (p=0.03), DWI as best sequence (p<0.02), ePI-RADS (p=0.001) and cPI-RADS (p=0.02).
**Conclusion:** The upgrade of PI-RADS 3 lesions because of positive DCE does not show a positive impact on the overall diagnostic performance, and actually, it worsens it. DWI proved to have extremely higher diagnostic power compared to DCE.
**Limitations:** It is a monocentric study in a high-volume referral centre. Both readers were experienced, and all images showed good quality (PIQUAL ≥ 3); this could limit the reproducibility of our results.
**Ethics committee approval:** Not applicable.
**Funding for this study:** No funding was received for this study.
**Author Disclosures:**
Marco Bicchetti: Nothing to disclose
Valeria Panebianco: Nothing to disclose
Emanuele Messina: Nothing to disclose
Martina Pecoraro: Nothing to disclose
Ludovica Laschena: Nothing to disclose
Carlo Catalano: Nothing to disclose

### RPS 2307-6

**Improved diffusion-weighted imaging of the prostate: comparison of readout-segmented and zoomed single-shot imaging**

*M. Boschheidgen*[1], M. Klingebiel[1], E. Weiland[2], R. Strecker[2], L. Schimmöller[1], G. Antoch[1]; [1]Düsseldorf/DE, [2]Erlangen/DE
*(matthias.boschheidgen@med.uni-duesseldorf.de)*

**Purpose:** Diffusion-weighted imaging (DWI) is the most important sequence for the detection and grading of prostate cancer (PCa). New approaches like zoomed single-shot imaging (z-EPI) with advanced image processing (AdvProc) or multi-shot readout segmentation (rs-EPI) try to improve DWI quality. This study evaluates the objective and subjective image quality (IQ) of rs-EPI and z-EPI with and without AdvProc.
**Methods or Backround:** Fifty-six consecutive patients (67±8 years; median PSA 8.3ng/ml) with mp-MRI performed at 3Tesla between February and October 2019 and subsequently verified PCa by targeted plus systematic MRI/US-fusion biopsy were included in this retrospective single-centre cohort study. Rs-EPI and z-EPI were prospectively acquired in every patient. Signal intensities (SI) of PCa and benign tissue in ADC, b1000, and calculated high b-value images were analysed. The endpoints were signal-to-noise ratio (SNR), contrast-to-noise ratio (CNR), PCa contrast intensity (CI), and subjective IQ on a five-point scale evaluated by three blinded readers. Wilcoxon signed rank test, Friedman test and Cohen´s kappa coefficient was calculated.
**Results or Findings:** SNR, CNR, and PCa CI of z-EPI with and without AdvProc were superior to rs-EPI (p<0.01), whereas no significant differences were observed between z-EPI with and without AdvProc. Subjective IQ was significantly higher for z-EPI with AdvProc compared rs-EPI for ADC, b1000, and calculated high b-values (p<0.01). Compared to z-EPI without AdvProc, z-EPI with AdvProc was superior for ADC and calculated high b-values (p<0.01), but no significant differences were shown for b1000 images.
**Conclusion:** Advanced z-EPI with and without AdvProc was superior to rs-EPI regarding objective imaging parameters, and z-EPI with AdvProc was superior to rs-EPI regarding subjective imaging parameters for detecting PCa.
**Limitations:** Different DWI performance when comparing different scanners and its dependence on configuration and software version.
**Ethics committee approval:** Not applicable.
**Funding for this study:** No funding was received for this study.
**Author Disclosures:**
Elisabeth Weiland: Nothing to disclose
Maximilian Klingebiel: Nothing to disclose
Ralph Strecker: Nothing to disclose
Matthias Boschheidgen: Nothing to disclose
Lars Schimmöller: Nothing to disclose
Gerald Antoch: Nothing to disclose

### RPS 2307-7

**Predicting factors for extraprostatic extension in patients with prostate cancer using decision curve analysis**

J. Baek, S. H. Kim, *H. M. Kim*; Daegu/KR

**Purpose:** The study aimed to identify effective factors predicting extraprostatic extension (EPE) in patients with prostate cancer (PCa) using decision-curve analysis.
**Methods or Backround:** This retrospective cohort study recruited 898 consecutive patients with PCa treated with robotic-assisted laparoscopic radical prostatectomy. The patients were divided into an EPE and non-EPE group based on whole-mount histopathologic sections. Analysis of the histopathology (ISUP biopsy grade group) and MRI (PI-RADS v2.1 score [1-5] and Mehralivand EPE grades [0-3]) showed predictive factors for EPE. We also assessed the clinical usefulness of the prediction model based on decision-curve analysis.
**Results or Findings:** Of 898 patients, 235 patients (29.3%) had an EPE, and 565 patients (70.7%) had non-EPE. Multivariable logistic regression analysis showed biopsy ISUP grade group, PI-RADS v2.1 score and Mehralivand EPE grades as independent risk factors for EPE. Assessing the regression models, the best discrimination (area under the curve 0.879) was obtained using the basic model (age, serum PSA, prostate volume at MRI, positive biopsy core, clinical T stage and D'Amico risk group) plus Mehralivand EPE grades 3. Decision-curve analysis showed that combining Mehralivand EPE grades 3 with the basic model resulted in superior net benefits for the prediction of EPE.
**Conclusion:** Mehralivand EPE grades and PI-RADS v2.1 score to basic clinical and demographic information is potentially useful for predicting EPE in patients with PCa.
**Limitations:** Small sample size in a single research centre.
**Ethics committee approval:** Not applicable.
**Funding for this study:** No funding was provided for this study.
**Author Disclosures:**
See Hyung Kim: Nothing to disclose
John Baek: Nothing to disclose
Hyun Min Kim: Nothing to disclose

### RPS 2307-8

**Prediction of extraprostatic extension with TCL measurement: role of the zonal level of prostate cancer**

*S. Y. Park*, G. Y. Jeon, B. G. Park, J. Park; Daegu/KR

**Purpose:** In a previous study, positive basal core biopsy predicts pelvic lymph node metastasis in prostate cancer. However, there have been no reports of microscopic extraprostatic extension (EPE) along the prostate gland level. Therefore, this study aimed to investigate the probability of EPE along the zonal level of the prostate by measuring tumour contact length (TCL) on multiparametric magnetic resonance imaging (mpMRI).
**Methods or Backround:** The records of 308 patients with radical prostatectomy (RP) were identified. The levels of the tumour were categorised as apex, mid, and base, and we evaluated the correlation between TCL measured by MRI and microscopic EPE on pathologic specimens. Univariable and multivariable logistic regression analyses were performed to assess the association between tumour origin, index tumour diameter, TCL measured by MRI, and microscopic EPE in radical prostatectomy specimens.
**Results or Findings:** There were 45 apical cancers (21%), 87 mid-gland cancers (41%), and 82 base cancers (38%) in 214 patients. After RP, there were 18 patients with pT3 in the apex tumour (40.0%), 31 patients with mid-gland tumours (35.6%), and 50 patients with base tumours (61.0%). Multivariable analysis demonstrated that the zonal level of the tumour, especially the base level, was an independent predictive factor for EPE (p=0.039). ROC analysis for prediction of EPE with zonal level for base tumour (AUC 0.858) showed a 96% sensitivity and a 65.6% specificity at TCL of 15mm (J=0.448, p<0.001).
**Conclusion:** Our results indicate that base cancers are more likely to have EPE than the mid gland or apical cancer. Therefore, the origin of prostate cancer according to the zonal level may help make favourable treatment decisions and predict clinical prognosis.
**Limitations:** Retrospective design and the comparatively small number of apical cancer are major limitations.
**Ethics committee approval:** No information provided by the submitter.
**Funding for this study:** No funding was provided for this study.
**Author Disclosures:**
Byung Geon Park: Nothing to disclose
Ga Young Jeon: Nothing to disclose
Jongmin Park: Nothing to disclose
Seo Young Park: Nothing to disclose

### RPS 2307-9

**High-performance integrative mathematical models for pathological status prediction in a large series of PCa patients: added value of radiomics and mp-MRI**

G. Marvaso, L. J. Isaksson, M. Zaffaroni, M. G. Vincini, P. Summers, G. Musi, G. Petralia, B. A. Jereczek-Fossa *F. Mastroleo*; Milan/IT
*(fed.mastroleo@gmail.com)*

**Purpose:** The most common systems used to risk-stratify PCa patients have been repeatedly shown to have suboptimal prognostic and stratification performances. The aim of the present study is to test the ability of high-performance mathematical models employing clinical radiological and radiomics features to improve the accuracy of non-invasive prediction of pathological features of PCa.
**Methods or Backround:** A cohort of 949 patients who underwent mpMRI and prostatectomy at IEO between 2015 and 2018 was selected. The prostate gland was segmented with an internally-developed deep-learning segmentation algorithm. Gradient-boosted decision tree models were separately trained using clinical, radiological and/or radiomic features. The contribution of each feature category was assessed in the whole cohort and within different risk groups and PI-RADS categories through the cumulative SHAP value and the mean absolute error (MAE). A comparison regarding misclassified patients between the models and the clinical workflow was performed.
**Results or Findings:** The AUC performance of four models demonstrated that the model, including all variables, resulted in the best in most endpoints. Radiological features account for the largest contribution in improving the

Sunday predictive performance, while radiomics appear to bring a measurable boost, although small. SHAP subgroup analyses showed that, although the mean/median influence of radiomic features is low, their contribution to individual patients' prediction can be very high; moreover, MAE values resulted lower in low-risk and low-PIRADS classes. The best prediction model outperformed the naïve one in all the considered endpoints in terms of AUC, whereas the accuracies were comparable.
**Conclusion:** Our results highlight the potential benefit of mathematical models for pathological feature prediction in PCa. These results are of considerable interest in informing clinical decision-making and can provide valuable information for personalising therapy and to guide the clinical course.
**Limitations:** Not applicable.
**Ethics committee approval:** The ethics committee notification can be found under the number UID 2438.
**Funding for this study:** No funding was provided for this study.
**Author Disclosures:**
Federico Mastroleo: Nothing to disclose
Maria Giulia Vincini: Nothing to disclose
Gennaro Musi: Nothing to disclose
Giulia Marvaso: Nothing to disclose
Barbara Alicja Jereczek: Nothing to disclose
Giuseppe Petralia: Nothing to disclose
Paul Summers: Nothing to disclose
Lars Johannes Isaksson: Nothing to disclose
Mattia Zaffaroni: Nothing to disclose

### RPS 2307-10

**Predictive role of Node-RADS in patients with PCa candidates for radical prostatectomy with extended lymph node dissection: a comparative analysis with validated nomograms**

*L. Laschena*, S. Lucciola, M. L. Pisciotti, A. Dehghanpour, C. Catalano, V. Panebianco; Rome/IT
*(ludovica.laschena@uniroma1.it)*

**Purpose:** The study aimed to analyse the predictive value of preoperative Node-RADS determination at imaging for pelvic lymph nodes (LN) involvement in cases of prostate cancer (PCa) considered for radical prostatectomy (RP) with extended lymph node dissection (eLND) and to compare it with validate predictive nomograms.
**Methods or Backround:** 150 patients with a histological diagnosis of PCa (high risk or intermediate with an estimated risk for pN+ higher than 5% using the Briganti or 7% using the Gandaglia nomogram) submitted for RP with an ePLND from 2018 and 2021 were retrospectively examined. Node-RADS determination was performed in all cases using the preoperative MRI, performed by a radiologist blinded for pathologic results and compared with the MSKCC, Briganti 2012, Gandaglia 2017 and Gandaglia 2019 nomograms.
**Results or Findings:** LN involvement at final pathology (pN+) was found in 36/150 (24.0%) of the cases and the mean percentage of positive LNs in pN+ cases was 15.90±13.40. The mean number of PLNs removed at RP was similar (p=0.188) between pN0 (23.9 ± 8.0) and pN+ (25.3 ± 8.0) cases. Considering a Node-RADS 4-5 positive and a Node RADS 1-2 negative, the PPV was 100% and the NPV was 79.6%. A Node-RADS score 4-5 showed a lower sensitivity (0.167 vs 0.972, 1.000, 0.971, 0.960, respectively), a higher specificity (1.000 vs 0.079, 0.096, 0.138, 0.186, respectively) and a similar AUC (0.583 vs 0.591, 0.581, 0.574, 0.597, respectively) when compared to the above-mentioned nomogram.
**Conclusion:** Our evaluation suggests that the Node-RADS score, combining configuration to size determination, could improve specificity in terms of pathologic PLN prediction, but a very low sensitivity has also been described.
**Limitations:** The study is limited by being a retrospective analysis.
**Ethics committee approval:** The study was approved by the institutional review board.
**Funding for this study:** No funding was received for this study.
**Author Disclosures:**
Sara Lucciola: Nothing to disclose
Valeria Panebianco: Nothing to disclose
Martina Lucia Pisciotti: Nothing to disclose
Ailin Dehghanpour: Nothing to disclose
Ludovica Laschena: Nothing to disclose
Carlo Catalano: Nothing to disclose

### RPS 2307-11

**Clinical application of the PRECISE score for reporting magnetic resonance imaging in men on active surveillance for prostate cancer**

J. Aerts, C. Berquin, S. Hendrickx, N. Lumen, C. Van Praet, S. Verbeke, G. M. Villeirs, *P. J. L. De Visschere*; Ghent/BE
*(pieter.devisschere@ugent.be)*

**Purpose:** The PRECISE score has been developed to standardise prostate MRI reporting in men on active surveillance (AS) for prostate cancer (PCa). Our objective was to evaluate the feasibility of PRECISE scoring and to assess its accuracy and prognostic value.
**Methods or Backround:** All patients on AS for PCa with a baseline MRI at diagnosis and at least one repeat MRI between January 2008 and September 2022 were included in a prospective database. All MRI scans were retrospectively re-reported by an expert radiologist and appointed a PI-RADS (v2.1) and a PRECISE score. Clinically significant progression was defined by histopathological upgrading (on biopsy or radical prostatectomy) to Gleason grade group 3 or higher and/or evolution to stage T3 disease.
**Results or Findings:** 188 patients were included for analysis, with a total of 358 MRI scans (1-6 repeat MRI per patient). Median follow-up was 46 months (interquartile range 21-74). 93 (49.5%) patients stayed on AS, 86 (45.7%) switched to active treatment, 7 (3.7%) switched to watchful waiting, and two (1.2%) died of another cause. PRECISE 4-5 had a sensitivity, specificity, NPV and PPV of 78%, 70%, 90% and 49% for clinically significant progression. The four-year progression-free survival (PFS) was 91% vs 66% for PRECISE 1-3 vs 4-5 (p<0.001) and 81% vs 43% for the MRI performed within 18 months after PCa diagnosis (N=137).
**Conclusion:** Implementing PRECISE scoring for PCa patients on AS is feasible and offers prognostic value. Patients with PRECISE scores 4-5 MRI have a significantly higher risk of clinically significant progression after four years.
**Limitations:** The repeated PSA testing, prostate biopsies and follow-up MRI scans in the active surveillance programme were not standardised but performed at the discretion of the treating clinician.
**Ethics committee approval:** Not applicable.
**Funding for this study:** No funding was provided for this study.
**Author Disclosures:**
Camille Berquin: Nothing to disclose
Pieter Julien Luc De Visschere: Nothing to disclose
Sofie Verbeke: Nothing to disclose
Geert M. Villeirs: Nothing to disclose
Jan Aerts: Nothing to disclose
Nicolaas Lumen: Nothing to disclose
Charles Van Praet: Nothing to disclose
Sigi Hendrickx: Nothing to disclose

### RPS 2307-12

**Prostate imaging for recurrence reporting (PI-RR) assessment score: diagnostic accuracy, interobserver agreement among readers with variable experience, and correlation with PSA levels**

*P. N. Franco*, S. Santos, A. Garcia Baizan, M. Aymerich, L. Paredes Velásquez, M. Otero Garcia; Vigo/ES
*(francopaoloniccolo@gmail.com)*

**Purpose:** The prostate imaging for recurrence reporting (PI-RR) system has been recently proposed to promote uniformity in the MRI assessment of prostate cancer (PCa) local recurrence after radical prostatectomy (RP) and radiation therapy (RT). The aims of the present study are to evaluate PI-RR's diagnostic accuracy, assess the interobserver agreement among readers with variable experience, and correlate MRI findings with anatomopathological and laboratory parameters.
**Methods or Backround:** Consecutive patients who underwent a multiparametric MRI for suspicion of PCa local recurrence after primary treatment were retrospectively enrolled (October 2017-February 2020). PI-RR scores were independently assessed for each patient by five readers with a variable experience in prostate imaging (two senior and three junior radiologists). Biochemical data and histopathological features were collected. The reference standard was determined through biochemical, imaging, or histopathological follow-up data. Reader's diagnostic performance was assessed using contingency tables. Cohen's kappa coefficient (κ) and intraclass correlation coefficient (ICC) were calculated to measure interobserver agreement.
**Results or Findings:** The final cohort included 120 patients (median age, 72 years [IQR, 62-82]). Considering a PI-RR score≥ 3 as positive for recurrence, diagnostic values' ranges among the readers were: sensitivity 79-86%; specificity 64-86%; positive predictive value 95-98%; negative predictive value 33-46%; accuracy 79-87%. Regardless of readers' experience, the interobserver agreement was good or excellent (κ ranges across all readers: 0.52-0.77), and ICC was 0.8. PSA-velocity and trigger-PSA showed a statistically significant correlation with the presence of local recurrences at imaging.
**Conclusion:** PI-RR system is an effective tool for MRI assessment of PCa local recurrence. It may help radiologists to standardise the MRI interpretation and reporting in local PCa recurrences after RT and RP.
**Limitations:** Retrospective and single-centre nature. Inhomogeneous distribution of RT- and RP-treated patients.
**Ethics committee approval:** The local ethics committee formally approved this study.
**Funding for this study:** No funding was received for this study.
**Author Disclosures:**
Milagros Otero Garcia: Nothing to disclose
Sofia Santos: Nothing to disclose
Laura Paredes Velásquez: Nothing to disclose

Alejandra Garcia Baizan: Nothing to disclose
Paolo Niccolò Franco: Nothing to disclose
Maria Aymerich: Nothing to disclose

### RPS 2307-13

**Apparent diffusion coefficient for the prediction of biochemical recurrence after prostatectomy in patients with prostate cancer**

*R. Maggioni*, S. Alessi, A. Colombo, P. Summers, S. Pasi, A. Berenghi, G. Conti, S. Vignati, G. Petralia; Milan/IT

**Purpose:** The study aimed to assess the role of the apparent diffusion coefficient (ADC) for the prediction of biochemical recurrence (BCR) five years after robot-assisted radical prostatectomy (RARP) in patients with prostate cancer (PCa).
**Methods or Backround:** 1439 consecutive patients with PCa treated with RARP from 2012 to 2015 were identified. All patients had preoperatory multiparametric magnetic resonance imaging performed on the same scanner (Avanto Siemens Healtineers 1.5T), with different Prostate Imaging Reporting And Data System (PI-RADS) v2.1 lesions. Only index lesions involving the peripheral zone were considered. ADC values were calculated using three b-values (0-500-1000s/mm2). Performance of Multivariable Cox Models, including clinical (age, PSA, cT, ISUP, cN) and radiological (prostate volume, lesion size, ADC) variables, was assessed (C-index).
**Results or Findings:** 1207/1439 (84%) patients had index lesions in the peripheral zone, and 25% (306/1207) of them experienced BCR. ADC (HR, 95% CI: 0.22, 0.12-0.42) was an independent significant predictor of five-years BCR, and the multivariable model had a C-index=0.72. Using ADC cut-offs of 850µm2/s and 1100µm2/s, identified adjusting tertiles based on clinical evidence, patients with different BCR-free survival rates were significantly separated (p<0.001). Results remained consistent in sensitivity analyses after the exclusion of patients with positive surgical margins (220, 18%) and patients who performed “salvage” radiotherapy after surgery (97, 8%). Results were consistent also at subgroup analyses in patients with focal index lesions (PI-RADS≥3, 867, 72%) but not in patients with diffuse lesions (PI-RADS≤2, 340, 28%), as expected.
**Conclusion:** ADC value is a significant predictor of BCR five years after surgery. It could be used in the risk assessment of patients with PCa to improve treatment planning.
**Limitations:** Mean ADC value may vary according to the scanner and protocol. External validation is needed.
**Ethics committee approval:** The ethics committee approval can be found under the number UID_2425.
**Funding for this study:** No funding was provided for this study.
**Author Disclosures:**
Roberta Maggioni: Nothing to disclose
Sarah Alessi: Nothing to disclose
Giuseppe Petralia: Nothing to disclose
Guglielmo Conti: Nothing to disclose
Paul Summers: Owner: QMRI Tech
Silvano Vignati: Nothing to disclose
Alessandro Berenghi: Nothing to disclose
Alberto Colombo: Nothing to disclose
Sara Pasi: Nothing to disclose

**09:30-11:00 Research Stage 3**

### Research Presentation Session: Paediatric

### RPS 2312

### Paediatric abdominal imaging

**Moderator**
J.-N. Dacher; Rouen/FR
*(jndacher@gmail.com)*

### RPS 2312-2

**Correlation between quantitative MRI liver tissue characterisation and long-term medical outcome in native liver survivor patients with biliary atresia after Kasai portoenterostomy**

*M. Caruso*, A. Stanzione, F. Di Dato, C. Ricciardi, N. Pisani, M. De Giorgi, G. D. Paoli, R. Liuzzi, S. Maurea; Naples/IT

**Purpose:** The aim of this study was to correlate liver MRI quantitative findings of native liver survivor biliary atresia (BA) patients after Kasai portoenterostomy (KP) with medical outcome.
**Methods or Backround:** Thirty BA patients were retrospectively enrolled after KP (median follow-up = 15.2 years) and classified according to clinical and laboratory data into two groups: patients with ideal (Group 1; n=11) and non-ideal (Group 2; n=19) medical outcome. For MRI analysis, liver and spleen volumes (cm3) were measured using a semi-automatic method. Portal vein diameter (mm) was measured with callipers. For DWI analysis, mean, maximum and minimum signal intensities corresponding to liver tissue conventional ADC values were measured using ROI analysis; similarly, texture analysis of ADC map and T2-weighted images was performed.
**Results or Findings:** A significant difference in liver and spleen volumes was observed between the two groups. The liver volume was significantly (p=0.007) lower in patients of Group 2 (954.88 + 218.31 cm3) compared to that of patients of Group 1 (1140.94 + 134.62 cm3). Conversely, the spleen volume was significantly (p<0.001) higher in patients of Group 2 (555.49 + 263.92 cm3) compared to that of patients of Group 1 (231.83 + 70.97 cm3). No differences between the two groups were found in portal vein diameter, liver ADC mean, maximum and minimum values as well as in ADC and T2-weighted textural parameters.
**Conclusion:** Although significant quantitative morpho-volumetric liver and spleen changes occurred in BA patients with non-ideal medical outcome after KP, no significant microstructural liver abnormalities detectable by conventional DWI sequence and texture analysis of ADC maps and T2-weighted images were found compared to those with ideal medical outcome.
**Limitations:** Sample size was limited and data was gathered from a single institution.
**Ethics committee approval:** No information provided by the submitter.
**Funding for this study:** No funding was received for this study.
**Author Disclosures:**
Raffaele Liuzzi: Nothing to disclose
Gregorio Delli Paoli: Nothing to disclose
Arnaldo Stanzione: Nothing to disclose
Fabiola Di Dato: Nothing to disclose
Marco De Giorgi: Nothing to disclose
Noemi Pisani: Nothing to disclose
Martina Caruso: Nothing to disclose
Carlo Ricciardi: Nothing to disclose
Simone Maurea: Nothing to disclose

### RPS 2312-3

**Unmasking the spectrum of computed tomographic appearances of enteric duplication cysts in paediatric patients**

*I. Gupta*, M. Bagarhatta, A. Bhandari; Jaipur/IN
*(ishagupta2707@gmail.com)*

**Purpose:** The aim was to study the imaging appearances of enteric duplication cysts on computed tomography with histopathological correlation.
**Methods or Backround:** Enteric duplication cysts (EDCs) are rare congenital malformations of the digestive tract with an incidence is 1:4,500 births. They can be found anywhere along the gastrointestinal tract, can be associated with spinal defects and cardiac or urinary malformations and can have varied clinical presentations and complications. Radiology plays an important role in unmasking these masqueraders and establishing the diagnosis. The study was designed as a descriptive type of observational study. Included were pediatric patients diagnosed with enteric duplication cysts on CT scans after taking proper consent. The study was performed from August 2021 to March 2022.
**Results or Findings:** Over eight months, 21 patients were referred for CT evaluation based on suspicion of enteric duplication cysts on USG, out of which 18 were reported to be enteric duplication cysts on CT. Ileum was the most frequent site of involvement (41%), followed by the oesophagus (23%). CT diagnosis correlated with histopathology in 94% of cases and played a pivotal role in surgical planning.
**Conclusion:** EDCs are rare congenital malformations of the gastrointestinal tract with varied clinical presentations. Although ultrasound is the first line of investigation, a CT scan is crucial for depicting the exact location, extension, complications, associated anomalies and anatomical relationship with surrounding structures. As CT plays pivotal for diagnosis and surgical planning for patients with enteric duplication cysts, familiarity with the spectrum of imaging findings is of prime importance. This study also revealed the current occurrence, spectrum, and distribution of these lesions in the paediatric population at a state of art tertiary care hospital.
**Limitations:** Due to the rare occurrence of EDCs, the sample size of the study was small.
**Ethics committee approval:** Not applicable.
**Funding for this study:** No funding was provided for this study.
**Author Disclosures:**
Meenu Bagarhatta: Nothing to disclose
Isha Gupta: Nothing to disclose
Anu Bhandari: Nothing to disclose

### RPS 2312-4

**Doppler-ultrasound reference values after paediatric liver transplantation: a consecutive cohort study**

*M. V. Verhagen*, R. de Kleine, H. Groen, H. van der Doef, T. Kwee, R. de Haas; Groningen/NL
*(mvverhagen@gmail.com)*

**Purpose:** Doppler ultrasound (DUS) is the main imaging modality to evaluate vascular complications of paediatric liver transplants (LT). The current study aimed to determine reference values and their change over time.
**Methods or Backround:** A consecutive cohort of paediatric patients undergoing an LT were retrospectively included between 2015 and 2020. Timepoints for standardised DUS were: intraoperative and postoperative (day 0), days 1-7, months 1 and 3, and years 1 and 2. DUS measurements of the hepatic artery (HA), portal vein (PV), and hepatic vein(s) (HV) were included if there were no complications during two year follow-up. Measurements consisted of the following: peak systolic velocity (PSV) and resistive index (RI) for the HA, PSV for the PV, and venous pulsatility index (VPI) for the HV. Generalised estimating equations were used to analyse change over time.
**Results or Findings:** One hundred and twelve paediatric patients with 123 LTs were included (median age 3.3 years, interquartile range 0.7-10.1). Ninety-five HAs, 100 PVs, and 115 HVs without complications were included. Reference values for HA PSV and RI, PV PSV, and HV VPI were obtained for all timepoints (4043 included datapoints in total) and presented using 5th-95th percentiles and threshold values. All reference values changed significantly over time (p=0.032 to p<0.001).
**Conclusion:** DUS reference values of hepatic vessels in children after LT are presented, reference values change over time with specific vessel-dependent patterns. Timepoint-specific reference values improve interpretation of DUS values, and may help to better weigh their clinical significance.
**Limitations:** This was a retrospective study, although measurements were obtained according to a standardised protocol, some measurement variability may have occurred.
**Ethics committee approval:** No information provided by the submitter.
**Funding for this study:** Not applicable
**Author Disclosures:**
Ruben de Kleine: Nothing to disclose
Martijn V Verhagen: Nothing to disclose
Hubert van der Doef: Nothing to disclose
Robbert de Haas: Nothing to disclose
Henk Groen: Nothing to disclose
Thomas Kwee: Nothing to disclose

### RPS 2312-5

**Association of physical activity and screen time with DXA- and MRI-based body composition in adolescents, a population-based study**

*T. Wu*, J. Yang-Huang, M. W. Vernooij, M. Rodriguez-Ayllon, V. Jaddoe, H. Raat, S. Klein, E. Oei; Rotterdam/NL
*(w.tong.1@erasmusmc.nl)*

**Purpose:** This study aimed to assess the associations of physical activity and screen time with body composition in adolescents.
**Methods or Backround:** 3,258 children aged 13 years from the Generation R Study, a population-based cohort study, were studied cross-sectionally. Physical activity and screen time were assessed via self-report questionnaires. Body mass index (BMI), dual-energy x-ray absorptiometry-based measures (i.e., fat mass index (FMI), lean body mass index (LBMI) and android/gynoid fat mass ratio (A/G ratio)), and MRI-based measures (i.e., abdominal subcutaneous fat index, abdominal visceral fat index and abdominal visceral/subcutaneous fat mass ratio (V/S ratio)) were obtained. In order to quantify MRI-based measurements, a deep learning-based segmentation algorithm was employed.
**Results or Findings:** A higher physical activity level was associated with a lower FMI (Z-score difference (95% CI): -0.08 (-0.14, -0.01)), A/G ratio (-0.10 (-0.18, -0.03)) and abdominal visceral fat index (0.11 (-0.20, -0.02)), and a higher LBMI (0.15 (0.08, 0.22)). A higher screen time was associated with a higher BMI (0.02 (0.01, 0.04)), FMI (0.02 (0.01, 0.03), A/G ratio (0.03 (0.02, 0.04)), abdominal subcutaneous fat index (0.03 (0.02, 0.05)) and abdominal visceral fat index (0.03 (0.01, 0.05)), and a lower V/S ratio (-0.02 (-0.04, -0.01)).
**Conclusion:** In adolescents, higher physical activity and lower screen time are linked to lower levels of adiposity both at the general and visceral levels. This information may be used to develop targeted intervention and prevention strategies that promote healthy behavior among adolescents.
**Limitations:** Since the main analyses in our study were cross-sectional, we cannot determine causality. We therefore, additionally adjusted for the body composition outcomes at age 10 years to reduce the chance of reverse causation, and comparable results were found.
**Ethics committee approval:** This study was approved by the Medical Ethical Committee of the Erasmus MC, University Medical Centre in Rotterdam (MEC-2012-165-NL40020.078.12).
**Funding for this study:** TW is supported by the China Scholarship Council (CSC) PhD Fellowship.
**Author Disclosures:**
Stefan Klein: Nothing to disclose
Edwin Oei: Nothing to disclose
María Rodriguez-Ayllon: Nothing to disclose
Hein Raat: Nothing to disclose
Vincent Jaddoe: Nothing to disclose
Meike W. Vernooij: Nothing to disclose
Junwen Yang-Huang: Nothing to disclose
Tong Wu: Nothing to disclose

### RPS 2312-6

**1H MRS-detected liver triglyceride concentrations in children with cardiometabolic risk factors related to gestational size**

*J. Hakumäki*, H. Nordman, L. Hintikka, H. Huopio, J. Jääskeläinen; Kuopio/FI
*(juhana.hakumaki@uef.fi)*

**Purpose:** Birth size is known to affect cardiometabolic risk in children. Being small for gestational age (SGA) bears a higher risk for adverse cardiovascular health and obesity later in life. Being born large for gestational age (LGA) also presents a clear risk, at least for obesity. The catch-up growth experienced by many SGA children is thought to be particularly risk-inducive. Effective diagnostic procedures and interventions regarding cardiometabolic risk in LGA and SGA children are thus required. To this end, we investigated the usefulness of liver MR spectroscopy for detecting potential metabolic differences in the above pediatric groups.
**Methods or Backround:** 34 children aged six to eight years were recruited for single-voxel MR spectroscopy of the liver at 1.5T, performed during breath-triggering on a Siemens-Avanto system. The children were grouped into SGA (n=4), LGA (n=9) and offspring of GDM mothers (n=6). Normal for gestational age controls (NGA) were also studied as controls (n=15). Several anthropometric and laboratory parameters were also measured. Statistical analyses and comparisons were performed with IBM-SPSS.
**Results or Findings:** The SGA group showed significantly higher liver fat content, as calculated by the AOC of the methyl resonance at 0.9ppm, than NGA children or LGA children (P<0.01). This was observed similarly for the 1.3ppm methylene resonance (p<0.05). Liver choline levels did not differ between groups. The LGA group did not differ significantly from NGA. Interestingly there was a significant correlation between s-CRP and methyl -CH2- lipid concentration (p<0,01).
**Conclusion:** 1H MR spectroscopy of the liver may help identify children at risk for adverse cardiometabolic development, such as silent inflammation and abnormal liver fat accumulation. Further studies are warranted.
**Limitations:** Relatively small group sizes.
**Ethics committee approval:** Approved by the research ethics committee of the hospital district.
**Funding for this study:** The study is funded by the University of Eastern Finland and Kuopio University Hospital.
**Author Disclosures:**
Henrikki Nordman: Nothing to disclose
Leena Hintikka: Nothing to disclose
Hanna Huopio: Nothing to disclose
Juhana Hakumäki: Nothing to disclose
Jarmo Jääskeläinen: Nothing to disclose

### RPS 2312-7

**Paediatric gastrojejunal tube exchange: reaping the rewards of a multi-disciplinary service**

*D. Grant*, A. C. Lee, D. Fawkner-Corbett, A. Sharrard, R. Bradley; Oxford/UK

**Purpose:** Our paediatric gastrojejunal tube exchange service launched three years ago, as a multidisciplinary team comprising paediatric radiology, paediatric surgery, and specialist nursing. It has seen continued growth in patient numbers and patient/parental approval, and forms a core branch of the regional paediatric enteral feeding service, which extends beyond the hospital into the community. In this subset of typically complex patients requiring jejunal feeding, we have found that departmental fluoroscopic exchange has dramatically reduced overnight hospital stays and the need for risky general anaesthetic procedures. We hypothesise that it will also reduce patient radiation exposure.
**Methods or Backround:** An evaluation of all paediatric jejunal tube exchanges performed within our fluoroscopy room was undertaken. Patient demographics, procedure/screening time, and dose were recorded. These were compared to age-grouped patients who had undergone these procedures under general anaesthetic in theatre.
**Results or Findings:** 253 gastrojejunal/jejunal procedures have been performed in paediatric fluoroscopy, 87% of which have occurred since this service formally launched in October 2019. There was a significant (p<0.0001) reduction in radiation dose amongst children undergoing the procedure in fluoroscopy compared to theatres. 60% of patients spent no more than one hour in the department. No major complications were demonstrated.

**Conclusion:** Our gastrojejunal tube exchange service has shown great success. From a patient perspective, it has improved access to treatment and reduced need for anaesthetic risks. From an economic perspective, it has reduced bed occupancy and we have shown that it has resulted in significantly reduced radiation exposure.
**Limitations:** There were relatively fewer patients in the theatre comparison group (due in large part to the growing success of the service).
**Ethics committee approval:** This study was registered and approved for service evaluation within the trust, using grouped anonymised data, and was not felt to require separate ethics committee approval.
**Funding for this study:** No funding was received for this study.
**Author Disclosures:**
Alex CH Lee: Nothing to disclose
David Grant: Nothing to disclose
Ruth Bradley: Nothing to disclose
David Fawkner-Corbett: Nothing to disclose
Alison Sharrard: Nothing to disclose

### RPS 2312-8

**Diffusion tensor magnetic resonance imaging in the grading of liver fibrosis associated with congenital ductal plate malformations**

*M. Elhawary*, M. Elmansy, K. Mohamed Ali, E. Abdallah,
A. A. K. A. Abdel Razek, T. Barakat, A. Sarhan; Mansoura/EG

**Purpose:** A liver biopsy is still the standard method for diagnosing ductal plate malformations (DPM). However, it is an invasive tool. Magnetic resonance imaging (MRI) showed its accuracy in diagnosing such pathology. Herein, this study was conducted to elucidate the role of diffusion MRI parameters in predicting the degree of hepatic fibrosis.
**Methods or Backround:** This prospective study included 29 patients with DPM and 20 healthy controls. Both groups underwent diffusion tensor magnetic resonance imaging (DT-MRI). Its parameters were compared between patients and controls and then correlated with the degree of liver fibrosis in the patient group.
**Results or Findings:** All patients with DPM, whatever its type, expressed significantly lower hepatic apparent diffusion coefficient (ADC) compared to controls. However, fractional anisotropy (FA) showed no significant difference between them. The ADC value of 1.65 x 10-3 mm2/s had sensitivity and specificity of 82.1% and 90%, respectively, differentiating DPM patients from the healthy controls. It was evident that patients with higher fibrosis grades had significantly lower hepatic ADC, indicating a negative correlation between ADC and the grade of hepatic fibrosis r¬s= -0.901, p< 0.001.
**Conclusion:** DT-MRI showed good efficacy in the diagnosis of congenital DPM. Moreover, ADC could be applied to monitor the degree of liver fibrosis rather than the invasive liver biopsy.
**Limitations:** The study is limited by the relatively small sample size.
**Ethics committee approval:** The ethics committee approval can be found under the number MS.20.02.1036.
**Funding for this study:** Not applicable.
**Author Disclosures:**
Khadiga Mohamed Ali: Nothing to disclose
Amr Sarhan: Nothing to disclose
Tarik Barakat: Nothing to disclose
Mohammed Elhawary: Nothing to disclose
Ahmed Abdel Khalek Abdel Abdel Razek: Nothing to disclose
Mostafa Elmansy: Nothing to disclose
Ebtesam Abdallah: Nothing to disclose

### RPS 2312-9

**Accuracy of tranperineal ultrasonography vs genitogram in children wth ambguous genitalia: a pilot study**

*D. Bhalla*, M. Jana, B. Ravisandhiran; New Delhi/IN
*(deeksha.bhalla2@gmail.com)*

**Purpose:** The study aimed to evaluate the accuracy of transperineal ultrasonography (TPUS) vs genitogram in the mapping of internal organs in children with ambiguous genitalia.
**Methods or Backround:** Retrospective study of consecutive children who underwent both genitogram and TPUS for ambiguous genitalia at our institute between January to September 2022. A paediatric radiology fellow analysed both examinations in a blinded manner and documented the visualisation of the urethra, vagina/Mullerian remnants and anal canal on both examinations. Additionally, the presence and length of urogenital sinus(UGS) and rectal fistula with urethra/vagina were recorded. The agreement between the two modalities was calculated using kappa statistics.
**Results or Findings:** Thirty-seven examinations from 36 children (20 male, 16 female) were included in the final analysis. The urethra was identified in all 97.3% genitograms and all TPUS examinations. Mullerian remnants were seen in 59.4% TPUS examinations, while they were opacified in only 46% genitograms. The anal canal was identified in 94.5% TPUS and opacified in 16.2% genitograms. Urogenital sinus was seen in 67.5% TPUS, while only 43% genitograms had UGS opacification. Common cloaca was seen in 10.8% and 8.1% genitograms, while rectourethral fistula was seen in 5.4% TPUS and not identified in any genitogram. The kappa agreement between both modalities for identification and the length of the common channel was 0.49, while for the rectal fistulae it was 0.47.
**Conclusion:** Transperineal ultrasonography has a high sensitivity for the detection of Mullerian structures as well as urogenital and rectal fistulae. This should be used as an adjunct to genitogram in all cases with ambiguous genitalia.
**Limitations:** The limitation was the small sample size and the retrospective nature of the study.
**Ethics committee approval:** Not applicable.
**Funding for this study:** No funding was provided for this study.
**Author Disclosures:**
Bharathi Ravisandhiran: Nothing to disclose
Deeksha Bhalla: Nothing to disclose
Manisha Jana: Nothing to disclose

### RPS 2312-10

**Is screening for renal anomalies warranted in neonates with isolated single umbilical artery?**

*I. Al Shuaili*; Muscat/OM
*(dribtisam27@gmail.com)*

**Purpose:** The aim of this study was to determine the prevalence of renal anomalies in patients with an isolated single umbilical artery (SUA) in our population and to decide whether screening (performing a renal ultrasound and in case of abnormalities on the ultrasound performing a micturating cystourethrogram in infants with an isolated SUA is justified.
**Methods or Backround:** Retrospectively, all renal ultrasound examinations assessed at our hospital in the last 15 years, the period between September 2007 and November 2021 in neonates with SUA with or without associated anomalies were analysed. 113 infants were included. Infants with SUA and obvious coexistent congenital abnormalities were not included in the study. Our focus is on isolated SUA. In infants with abnormal renal ultrasonography, subsequent MCUG was assessed (if it was carried out).
**Results or Findings:** Only around 100 patients had SUA per our tertiary hospital. The prevalence of neonates with isolated SUA compared to non-isolated is high (80.5%). The prevalence of renal anomalies in isolated SUA is low (16.5) compared to the prevalence of renal anomalies in non-isolated SUA (45.5%). Consequently, there is no excess of significant renal malformations among infants with isolated SUA (16.5%, 15/91). The majority of abnormalities found on renal US were mild hydronephrosis (73%) without further consequences.
**Conclusion:** Only one case had a subsequent further investigation (MCUG), which turned out normal.
Our data suggest that it is not necessary to screen for renal anomalies in infants with a single umbilical artery without other anomalies seen at physical examination.
**Limitations:** There was only a small positive sample.
**Ethics committee approval:** No information provided by the submitter.
**Funding for this study:** No funding was received for this study.
**Author Disclosures:**
Ibtisam Al Shuaili: Nothing to disclose

### RPS 2312-11

**Shear wave elastography for assessing liver cirrhosis in infants with biliary atresia**

Y. Chen, J. Zhao, *Y. Zhu*; Shanghai/CN

**Purpose:** The study aimed to investigate the performance of shear wave elastography (SWE) in assessing liver cirrhosis in infants with biliary atresia (BA).
**Methods or Backround:** Between June 2019 to December 2021, a total of 76 consecutive infants with BA underwent surgery in our institute were retrospectively analysed. All infants underwent SWE evaluation within one week prior to surgery. Liver fibrosis was diagnosed according to intraoperative liver biopsy histopathology using Batts-Ludwig scoring system, and liver cirrhosis was defined as F4 stage. Mann-Whitney test was used to compare the SWE measurements of the infants with cirrhosis and non-cirrhosis. ROC analysis was employed to evaluate the performance of SWE in diagnosing liver cirrhosis.
**Results or Findings:** The biopsy histopathology confirmed 14 infants with fibrosis stage F0 (no fibrosis), 14 infants with fibrosis stage F1 (mild fibrosis), 14 infants with fibrosis stage F2 (moderate fibrosis), 20 infants with fibrosis stage F3 (severe fibrosis), and 14 infants with fibrosis stage F4 (cirrhosis). The median SWE measurement was 10.3 (IQR: 6.6-12.1) kPa, 10.5 (IQR: 8.6-16.5) kPa, 11.8 (IQR: 8.9-15.7) kPa, 12.5 (IQR: 8.5-16.9) kPa and 22.6 (IQR: 13.9-40.5) kPa for infants with fibrosis stage F0, F1, F2, F3 and F4, respectively. Compared with infants without cirrhosis, those with cirrhosis had significantly higher SWE measurements (22.6 [IQR: 13.9-40.5] kPa vs 10.8 [IQR: 8.3-15.3] kPa, P＜0.01). The AUC of SWE measurements for the diagnosis of liver cirrhosis was 0.871 (95% CI: 0.767-0.975), with the optimal cut-off value of 21.26 kPa showing a sensitivity of 67.0% and specificity of 93.2%, respectively.
**Conclusion:** For infants with BA, SWE enables non-invasive diagnosis of liver cirrhosis with high specificity. It would be a useful tool for the preoperative assessment and follow-up of BA.
**Limitations:** The retrospective design and small sample size were the limitations.
**Ethics committee approval:** The ethics committee of Xinhua Hospital, affiliated with the Shanghai Jiaotong University School of Medicine, approved this study. The approval can be found under the number XHEC-D 2015-160.
**Funding for this study:** No funding was provided for this study.
**Author Disclosures:**
Jing Zhao: Nothing to disclose
Yaqing Chen: Nothing to disclose
Yunkai Zhu: Nothing to disclose

**09:30-11:00** **Research Stage 4**

### Research Presentation Session: Vascular

### RPS 2315

### Vascular imaging: aorta

**Moderator**
H. von Tengg-Kobligk; Berne/CH

### RPS 2315-2

**Feasibility of abdominal aortic angiography utilising ultra-low-dose contrast medium based on low-level virtual monoenergetic imaging: a prospective study**

*S. Dong*, J. Chen, X. Zhang, S. Xu, L. Fan, L. Shiyuan, C. Yuanyuan; Shanghai/CN

**Purpose:** The study aimed to investigate the feasibility of ultra-low-dose contrast medium (CM) utilisation based on low-level virtual monoenergetic images (VMI) in abdominal aortic angiography.
**Methods or Backround:** Fifty-eight patients who underwent abdominal aortic CT angiography examination on a dual-layer detector CT (spectral CT 7500, Philips Healthcare, Best, Netherlands) were randomly divided into ultra-low-dose CM group (LDCM) (n=27) and normal dose CM group (NCCM) (n=31). CM injection amount in NCCM was set as body weight (Kg) × iodine concentration (mgI/ml) / CM concentration (mg/ml). In LDCM group, 25% CM dosage of NCCM was used, and VMI 40keV was reconstructed instead of conventional images (VMI-LDCM). For objective analysis, circular regions of interest (ROIs) were placed on the abdominal aorta at the level of bifurcation of the renal artery. The CT values, signal-to-noise ratios (SNRs), and contrast signal-to-noise ratios (CNRs) of the abdominal aorta were calculated and compared in two groups (NDCM and VMI-LDCM). Subjective assessments were performed by two radiologists independently (0-3 point grading score: 0 = non-diagnostic, 3= excellent angiographic images). T-test, Kruskal-Wallis Test and Cohen's kappa test were used for statistical analysis.
**Results or Findings:** The CNR of VMI-LDCM were significantly higher than NDCM group (p<0.01). There were no significant differences in subjective scoring for primary and secondary arterial branches between NDCM and VMI-LDCM images (p＞0.05), but there were significant differences in the third- and fourth-grade arterial branches (p<0.01). There was substantial agreement between the two independent readers.
**Conclusion:** The combination of VMI 40keV enabled 75% CM reduction in clinical abdominal aortic CTA scans and improved the imaging quality of small branch angiography.
**Limitations:** The relatively low number of included participants enabled assessment.
**Ethics committee approval:** Not applicable.
**Funding for this study:** No funding was received for this study.
**Author Disclosures:**
Liu Shiyuan: Nothing to disclose
Shuwen Dong: Nothing to disclose
Li Fan: Nothing to disclose
Shaochun Xu: Nothing to disclose
Cui Yuanyuan: Nothing to disclose
Xiaohui Zhang: Nothing to disclose
Jieqiong Chen: Nothing to disclose

### RPS 2315-3

**Automatic AI-based measurement of the thoracic aortic diameter in low-dose non-contrast chest CT: a validation study**

*I. Hamelink*, E-J. de Heide, G. J. Pelgrim, T. Kwee, P. M. A. Van Ooijen, G. de Bock, R. Vliegenthart; Groningen/NL

**Purpose:** Aortic diameters, as measured on a chest CT serve as a reliable tool for diagnosing thoracic aortic aneurysms. Such a task would be ideal for an AI application that automatically analyses chest CT scans. The aim of this study was to validate the performance of a commercial, fully automatic AI system for diameter assessment of the thoracic aorta.
**Methods or Backround:** In this study, 240 non-contrast non-gated low-dose chest CT scans of the Imaging in LifeLines (ImaLife) cohort stratified for age and aortic dilatation status were analysed for aortic diameters. Aortic diameters at eight guideline-compliant landmark positions were measured manually by two trained readers and with the AI algorithm. In 90 examinations, the diameters were manually reassessed for inter- and intra-reader variability. Manual and AI measurements were compared for the discrepancy.
**Results or Findings:** AI evaluation failed in 11 out of 240 cases due to incorrect segmentation. In the resulting 229 scans, a paired t-test showed no significant difference in maximum aortic diameter between manual and automatic measurements (P>0.05). Inter- and intra-reader variability showed the highest discrepancy (2.0±1.6mm and 1.2±1.0mm, respectively) for the measurements at the sinus of Valsalva, indicating a relatively low reproducibility of measurements at that site. Measurements at that site were ignored in further evaluation. For all other automatic measurements, the mean discrepancy was 1.3±2.4mm, compared to an inter- and intra-reader variability of respectively 1.3±1.2mm and 0.7±0.6mm.
**Conclusion:** AI software is able to generate results on non-contrast non-gated low-dose chest CT scans with discrepancies similar to manual measurements.
**Limitations:** CT data from this study is low-dose non-gated similar to lung cancer screening and therefore not the clinical standard.
**Ethics committee approval:** No information provided by the submitter.
**Funding for this study:** Not applicable.
**Author Disclosures:**
Iris Hamelink: Research/Grant Support: PhD funded by PUSH grant
Geertruida de Bock: Nothing to disclose
Peter M. A. Van Ooijen: Nothing to disclose
Rozemarijn Vliegenthart: Research/Grant Support: Institutional research grant from Siemens Healthineers
Thomas Kwee: Nothing to disclose
Erik-Jan de Heide: Nothing to disclose
Gert Jan Pelgrim: Nothing to disclose

### RPS 2315-4

**Deep-learning-based quantification of aortic macroangiopathy improves the prediction of cardiovascular mortality in the national lung screening trial (NLST)**

*A. Rau*[1], B. Wilhelm[1], L. Michel[1], M. Reisert[1], V. Raghu[2], H. Aerts[2], M. T. Lu[2], F. Bamberg[1], J. Weiß[1]; [1]Freiburg/DE, [2]Boston, MA/US
*(alexander.rau@uniklinik-freiburg.de)*

**Purpose:** Currently, maximum vessel diameter is the only measure from computed tomography (CT) used in clinical practice to assess cardiovascular disease (CV)-related alterations of the aorta due to time and equipment constraints; however, quantification of detailed macroangiopathic changes is feasible but labour-intensive.
**Methods or Backround:** We used a deep neuronal patchwork in CTs of the national lung screening trial to quantify macroangiopathic features of the aorta, e.g. maximum diameter, volume, and calcifications (reported in quartiles). The primary outcome was CV mortality. Harrel's c-index was used to compare the predictive value of the aortic features. Cox proportional hazards regression was employed for the association between macroangiopathic features and CV mortality.
**Results or Findings:** In 22,904 participants (mean age 61.4±50 years; 59.1% male), there were 1.7% (392/22,904) CV deaths over a follow-up of 6.6±1.1 years. Aortic volume and calcifications had a significantly higher predictive value for CV mortality than the maximum diameter (c-index 0.60 and 0.62 vs 0.58; p≤0.002). In univariable analysis, there were significant associations between all macroangiopathic features and CV mortality: maximum diameter (HR: 1.03 [1.02-1.04]; p<0.001), volume (HR: 1.00 [1.00-1.00]; p<0.001) and calcifications (HR2nd quartile: 1.76 [1.23-2.53]; HR3rd quartile: 2.29 [1.62-3.23]; HR4th quartile: 3.61 [2.60-5.01]; all p≤0.002). In multivariable models containing maximum diameter and either volume or calcifications plus baseline demographics and traditional CV risk factors, volume (aHR: 1.00 [1.00-1.00]; p=0.04) and the 3rd/4th calcification quartile remained significantly associated (HR3rd quartile: 1.45 [1.00-2.10]; HR4th quartile: 1.99 [1.37-2.88]; p≤0.04) with CV mortality whereas the association for maximum diameter was attenuated.
**Conclusion:** Deep-learning-based quantification of aortic volume and calcifications are independent predictors for CV mortality beyond maximum diameter and traditional CV risk factors in heavy smokers.
**Limitations:** The patchwork output could not be approved for all participants.

**Ethics committee approval:** Not applicable.
**Funding for this study:** No funding was provided for this study.
**Author Disclosures:**
Ben Wilhelm: Nothing to disclose
Marco Reisert: Nothing to disclose
Lea Michel: Nothing to disclose
Alexander Rau: Nothing to disclose
Hugo Aerts: Nothing to disclose
Fabian Bamberg: Nothing to disclose
Jakob Weiß: Nothing to disclose
Vineet Raghu: Nothing to disclose
Michael T. Lu: Nothing to disclose

### RPS 2315-5

**Quantitative 4D flow derived thoracic aortic normal values of 2D flow parameters in healthy volunteers**

*S. Ebel*[1], A. Kühn[1], B. Köhler[2], B. Behrendt[2], B. Riekena[1], B. Preim[2], T. Denecke[1], M. Grothoff[1], M. Gutberlet[1]; [1]Leipzig/DE, [2]Magdeburg/DE
*(sebastian.ebel@icloud.com)*

**Purpose:** The study aimed to utilise 4D flow to acquire normal values of “conventional 2D flow parameters” in healthy volunteers in order to replace multiple single 2D flow measurements with a single 4D flow acquisition.
**Methods or Backround:** A kt-GRAPPA5 accelerated 4D flow sequence was used. Flow volumes were assessed by forward (FFV), backward (BFV), and net flow volumes (NFV) [ml/heartbeat] and flow velocities by axial (VAX) and absolute velocity (VABS) [m/s] in 116 volunteers (58 females, 43±15 years). The aortic valve regurgitant fraction (RF) was calculated.
**Results or Findings:** The gender-neutral mean maximum FFV, BFV, NFV and RF in the ascending aorta were 93.5±14.8, 3.6±2.8, 89.9±0.6ml/heartbeat and 3.9±2.9%. There were significantly higher values in males regarding FFV, BFV, NFV and RF, but no gender dependency regarding VAX and VABS. Mean peak Vax was lower (1.01±0.31m/sec) than VABS (1.23±0.35 m/sec). We could determine normal ranges for all intended parameters.
**Conclusion:** This study provides quantitative 4D flow-derived thoracic aortic normal values of 2D flow parameters in healthy volunteers. FFV, BFV, NFV and Vax did not differ from normal values of single 2D flow acquisitions and could therefore replace time-consuming multiple single 2D flow acquisitions. The 4D flow-specific VABS should not be used interchangeably.
**Limitations:** Only one specific 4D flow sequence was evaluated.
**Ethics committee approval:** The local ethics board of the Medical Faculty of the University Leipzig approved this study. The approval can be found under the number AZ 443/16-ek.
**Funding for this study:** The study was supported by a DFG grant (GU 777/4-1 AOBJ 629068 and GR 4617/2-1 AOBJ 629069).
**Author Disclosures:**
Boris Riekena: Nothing to disclose
Bernhard Preim: Nothing to disclose
Timm Denecke: Nothing to disclose
Matthias Gutberlet: Nothing to disclose
Matthias Grothoff: Nothing to disclose
Benjamin Köhler: Nothing to disclose
Sebastian Ebel: Nothing to disclose
Alexander Kühn: Nothing to disclose
Benjamin Behrendt: Nothing to disclose

### RPS 2315-6

**Diagnostic accuracy of virtual non-iodine imaging in photon-counting detector CT angiography for endoleak detection after endovascular aortic repair**

*A. M. Turrion Gomollon*, M. Polacin, D. N. Nakhostin, T. Sartoretti, V. Mergen, H. Alkadhi, A. Euler; Zurich/CH
*(anamaria.turriongomollon@usz.ch)*

**Purpose:** The aim of this study was to compare diagnostic accuracy for endoleak detection after endovascular abdominal aortic aneurysm repair between a triple-phase CT with true non-contrast (TNC) or a dual-phase CT with virtual non-iodine images (VNI) on photon-counting detector CT (PCD-CT).
**Methods or Backround:** Adult patients after endovascular aortic aneurysm repair who received a triphasic examination (TNC, arterial, venous phase) on a first-generation dual-source PCD-CT between August 2021 and July 2022 were retrospectively included. Endoleak detection was evaluated by two blinded radiologists on two different readout sets (TNC-arterial-venous vs VNI-arterial-venous). VNI were reconstructed from the venous phase scans. The radiologic report with additional confirmation by an expert reader served as reference standard for endoleak presence. Image noise of TNC and VNI was assessed objectively calculating the noise power spectrum (NPS) in a phantom and subjectively by one radiologist in patients using a 5-point scale. Sensitivity, specificity and interreader agreement (Krippendorf α) were calculated.
**Results or Findings:** 110 patients with 41 endoleaks were included. Endoleak detection was comparable between both readout sets (sensitivity/specificity: 0.95/0.84 (TNC) vs. 0.95/0.86 (VNI) for reader 1 and 0.88/0.98 (TNC) vs. 0.88/0.94 (VNI) for reader 2). Interreader agreement for endoleak detection was substantial (TNC: 0.716, VNI: 0.756). Subjective image noise was comparable between TNC and VNI (4; [4, 5] vs 4; [4, 5], p=0.44). In the phantom, peak spatial frequency of the NPS was similar between TNC and VNI (fpeak=0.16/mm vs 0.16/mm). Objective image noise was higher in TNC (12.7 HU) as compared to VNI (11.5 HU).
**Conclusion:** Endoleak detection and image noise were comparable using virtual non-iodine images in dual-phase CT as compared to true non-contrast images in triple-phase CT. This offers the possibility to omit the TNC scan, consecutively reducing patients' radiation exposure.
**Limitations:** There was no outcome analysis.
**Ethics committee approval:** This study was approved by the cantonal ethics commitee Zurich.
**Funding for this study:** No funding was received for this study.
**Author Disclosures:**
Victor Mergen: Nothing to disclose
Thomas Sartoretti: Nothing to disclose
Dominik Nader Nakhostin: Nothing to disclose
Ana Maria Turrion Gomollon: Nothing to disclose
Malgorzata Polacin: Nothing to disclose
Andre Euler: Nothing to disclose
Hatem Alkadhi: Nothing to disclose

### RPS 2315-7

**The feasibility of virtual monoenergetic images at 200keV as non-contrast datasets for endoleak and aortic aneurysm sac calcification detection in follow-up after endovascular aortic repair**

*A. Streckenbach*, D. Altmeppen-Többen, M. Meyer; Hamburg/DE
*(a.streckenbach@uke.de)*

**Purpose:** Spectral CT enables the reconstruction of virtual monoenergetic images (VMI) and virtual non-contrast images (VNC) from contrast-enhanced CT scans. The purpose of this study was to assess the feasibility of VMI at 200keV for the detection of endoleaks after endovascular aortic repair (EVAR) and aortic aneurysm sac calcifications.
**Methods or Backround:** In this retrospective study 87 patients underwent a triple-phase spectral CT protocol for follow-up after EVAR using a dual-layer CT system. The protocol included an unenhanced phase (TNC), an arterial phase and a delayed phase - the latter two in spectral mode. From these datasets VNC and VMI datasets at 200keV were reconstructed from the arterial phase. Attenuation measurements and calcium scoring of the aortic aneurysm sac was performed on each dataset and the TNC datasets served as reference standard. The absolute difference between the TNC and VNC/200keV were compared employing the Wilcoxon signed rank test.
**Results or Findings:** Endoleak detection was correctly identified in all 33 patients (38%). Mean absolute attenuation differences of the aortic aneurysm sac between TNC and the VNC /200keV datasets was significantly different, with higher differences for the VNC datasets (VNC: 8.9±6.3HU and 200keV 6.7±4.8HU; p<0.01). Calcium score values were significantly lower for both VNC and 200keV datasets, when compared to TNC datasets (TNC: 3175±2415, VNC 2541±2211 and 200keV 2623±2313; p<0.03). Of note, mean absolute difference in calcium scoring between TNC and VNC datasets demonstrated significantly higher values, when compared to 200keV datasets (VNC: 137±202 and 200keV 97±161HU; p=0.02).
**Conclusion:** VMI at 200keV as non-contrast enable accurate endoleak detection in patients after EVAR. When compared to VNC datasets, 200keV datasets demonstrate significant better agreement with TNC datasets for aortic aneurysm sac calcium scoring.
**Limitations:** This study was limited by its small cohort. There were measurement inaccuracies as well as differences down to technical reasons, for example artifacts.
**Ethics committee approval:** No information provided by the submitter.
**Funding for this study:** No funding was received for this study.
**Author Disclosures:**
Danielle Altmeppen-Többen: Nothing to disclose
Mathias Meyer: Nothing to disclose
Anna Streckenbach: Nothing to disclose

### RPS 2315-8

**Novel biomarkers for endoleak in patients with abdominal aortic aneurysm (AAA) undergoing endovascular aortic repair (EVAR)**

*J. Izzi*, F. L. Serafini, A. Delli Pizzi, P. Simeone, A. Giammarino, B. Consorte, M. Villani, P. Lanuti, M. Caulo; Chieti/IT

**Purpose:** We aimed to evaluate the potential of peripheral blood circulating extracellular vesicles as early diagnostic biomarkers for endoleak development in patients affected by an abdominal aortic aneurysm (AAA) treated with endovascular aortic repair (EVAR).
**Methods or Backround:** We designed a monocentric prospective study focused on AAA eligible for EVAR. 22 patients were enrolled. For each patient, a peripheral blood sample was collected during every diagnostic step protocol:

(1) before CT angiography (CTA), at AAA diagnosis; (2) after one-month post-EVAR implantation, before follow-up CTA; (3) after 6 months from EVAR, before follow-up contrast-enhanced ultrasonography (CEUS); (4) after 12 months post-EVAR implantation, before follow-up CTA. At the one-month CTA, patients were divided into two groups: “endoleak” and “no endoleak”, depending on the presence of an endoleak, the most common long-term EVAR complication. Peripheral blood samples of each patient during every follow-up step were analysed by polychromatic flow cytometry.
**Results or Findings:** Patients developing the endoleak showed a significant decrease of activated platelets derived-EVs between the baseline condition and six months after EVAR intervention. Furthermore, one month after EVAR implantation, patients developing the endoleak showed higher concentrations of activated endothelial derived-EVs than patients who did not develop it, suggesting their great potential as a noninvasive and specific biomarker for early identification of EVAR complications.
**Conclusion:** Quantitatively and qualitatively, differences in EV populations were demonstrated in patients developing the endoleak compared to non-complicated patients. EVs derived from activated platelets have great potential as a noninvasive and specific biomarker for the early identification of endoleaks.
**Limitations:** The study is limited by the small population.
**Ethics committee approval:** The ethical committee of the University G. d’Annunzio, Chieti-Pescara, Italy approved the study. The approval can be found under the number 1804, with the date of approval of February 20, 2020.
**Funding for this study:** No funding was received for this study.
**Author Disclosures:**
Michela Villani: Nothing to disclose
Jacopo Izzi: Nothing to disclose
Andrea Delli Pizzi: Nothing to disclose
Pasquale Simeone: Nothing to disclose
Alberto Giammarino: Nothing to disclose
Paola Lanuti: Nothing to disclose
Francesco Lorenzo Serafini: Nothing to disclose
Massimo Caulo: Nothing to disclose
Bruno Consorte: Nothing to disclose

### RPS 2315-9

**The value of hyper-realistic rendering (HRR) in the diagnosis of endoleak**
B. Guo, *L. Peng*, C. Yan, H. Zhao, W. Liu, G. Zhang, W. Fu; Shanghai/CN

**Purpose:** Hyper-realistic rendering (HRR, United Imaging Healthcare, Shanghai, China) is an advanced 3D reconstruction technique that can produce photorealistic 3D CT images with lifelike surface details and improved display of complex spatial relationships. We aimed to evaluate the value of HRR in the diagnosis of endoleak.
**Methods or Backround:** This retrospective study included 33 patients with a confirmed diagnosis of endoleak (type I:19, type II:14). All the patients underwent both CT angiography (CTA) and digital subtraction angiography (DSA). The multiplanar reconstruction (MPR) and HRR from the same CTA examination were evaluated by two experienced vascular surgeons, and a consensus was reached. A diagnostic category (type I or type II endoleak) was assigned to each image, and the diagnostic confidence was rated according to a 5-point Likert scale (1=poor, 5=excellent). For those patients with type II endoleak, the readers were asked to identify the feeding vessels from the inferior mesenteric artery, internal iliac artery, and lumbar arteries. The diagnostic performance was compared between the MPR and HRR, using DSA as the reference standard.
**Results or Findings:** HRR outperformed MPR in terms of the classification of endoleak, showing higher sensitivity (HRR 0.96 vs MPR 0.64, $p<0.05$), specificity (HRR 0.87 vs MPR 0.68, $p<0.05$), AUC (HRR 0.92 vs MPR 0.66, $p<0.05$) and ratings of diagnostic confidence (HRR $4.82\pm0.39$ vs MPR $3.91\pm0.68$, $p<0.05$). For the detection of the feeding vessels, HRR achieved higher sensitivity (HRR 0.92 vs MPR 0.60, $p<0.05$) and AUC (HRR 0.93 vs MPR 0.71, $p<0.05$) on the per-vessel level, compared to MPR. No significant difference was found in specificity between the HRR and MPR ($p>0.05$).
**Conclusion:** The HRR could serve as a powerful technique for endoleak diagnosis.
**Limitations:** The study was performed as a single-centre study.
**Ethics committee approval:** The study was approved by the institutional review board (IRB), and the requirement for written informed consent was waived for its retrospective nature.
**Funding for this study:** The National Natural Science Foundation of China (grant number 82000436) funded this study.
**Author Disclosures:**
Haitong Zhao: Employee: United Imaging Healthcare
Weiguo Fu: Nothing to disclose
Guozhi Zhang: Employee: United Imaging Healthcare
Wenqing Liu: Employee: United Imaging Healthcare
Cheng Yan: Nothing to disclose
Liying Peng: Employee: United Imaging Healthcare
Baolei Guo: Nothing to disclose

### RPS 2315-10

**Added value of quantitative dynamic total-body FDG-PET in the assessment of disease activity of Takayasu’s arteritis**
*Y. Duan*[1], Y. L. Ng[2], M. Zhao[1], K. Zan[1], X. Cui[1], Y. Zhou[2], Z. Cheng[1], X. Wang[1]; [1]Ji Nan/CN, [2]Shanghai/CN
*(dyh8212@163.com)*

**Purpose:** The study aimed to evaluate the added value of quantitative dynamic total-body FDG-PET over static PET for the disease activity of Takayasu’s arteritis (TAK).
**Methods or Backround:** Nine active-stage TAK patients (21-63 years) and six age- and gender-matched (23-60 years) control participants were enrolled in this study. All patients underwent one-hour dynamic and delayed static total-body FDG PET/CT scans at 1-, 2- and 4-hour post-injection. Quantitative parameters, including maximum and mean standardised uptake values, tissue influx rate (Ki) and lesion-to-background ratios (LBRs) from static and dynamic PET were analysed. Spearman’s rank correlation and Wilcoxon signed-rank tests were performed.
**Results or Findings:** There were no differences between study groups in age, gender, weight, blood glucose, FDG injection dose and liver uptake (all $p>0.05$). For TAK patients, the median SUVmax on 1-, 2- and 4-hour PET were 2.63[IQR:2.47-3.39], 2.66[IQR:2.10-3.48] and 2.86[IQR:1.95-3.93], respectively (all $p>0.05$). The median LBR on 1-, 2- and 4-hour PET were 1.19[IQR:1.07-1.54], 1.46[IQR:1.28-2.35], and 1.83[IQR, 1.95-3.93], respectively (all $p<0.001$). The median of dynamic PET-derived Ki and LBR_Ki was 0.011[IQR:0.010-0.015] and 2.69[IQR:2.38-3.49], respectively. The LBR_Ki for TAK patients were significantly higher than LBR_SUV1h ($p<0.0001$), LBR_SUV2h ($p<0.0001$) and LBR_SUV4h ($p=0.0003$). An increasing trend of median LBR_Ki across 1-, 2- and 4-h PET was observed with the increment of 164.4%, 115.4% and 78.4%, respectively. The SUVmax, Ki, LBR_SUV1h, and LBR_Ki were significantly higher in TAK patients than in control cases ($p<0.001$, $p=0.001$, $p<0.001$ and $p<0.001$, respectively).
**Conclusion:** Quantitative LBR_Ki may be considered a more sensitive parameter than LBR_SUV. Dynamic FDG-PET is suggested to have great potential to quantitatively assess the disease activity of TAK more accurately than conventional static PET.
**Limitations:** No limitations were identified.
**Ethics committee approval:** This prospective study was approved by the institutional review board of our hospital.
**Funding for this study:** No funding was received for this study.
**Author Disclosures:**
Minjie Zhao: Nothing to disclose
Yanhua Duan: Nothing to disclose
Yee Ling Ng: Nothing to disclose
Xiao Cui: Nothing to disclose
Zhaoping Cheng: Nothing to disclose
Yun Zhou: Nothing to disclose
Ximing Wang: Nothing to disclose
Keyu Zan: Nothing to disclose

### RPS 2315-11

**Stent appearance in resected human arteries using a novel silicon-based photon-counting CT prototype**
*E. Verelst*[1], G. Van Gompel[1], K. H. Nieboer[1], J. De Mey[1], F. R. Van Den Bergh[1], D. Crotty[2], P. D. Deak[3], N. Buls[1]; [1]Brussels/BE, [2]Cork/IE, [3]Ittigen/CH
*(emma.verelst@vub.be)*

**Purpose:** The study aimed to evaluate stent appearance using a prototype silicon-based photon counting CT system (PCCT), compared with a standard-of-care energy-integrating-detector (EID) CT.
**Methods or Backround:** Three 20cm diameter phantoms were developed consisting of a 2% agar-agar-water mixture in which resected and stented (AdvantaV12, Atrium) human arteries were respectively embedded: a) carotid (d=5.5mm), b) femoral (d1=7mm, d2=5.8mm) and c) iliac (d=8.5mm). Helical scans were acquired using a prototype PCCT system (GE Healthcare, Milwaukee, USA) and a conventional EID CT system (GE Revolution Apex40, Milwaukee, USA) at 120kV, with 40mm collimation, p=0.9, 0.5s rotation time and a mAs selected to achieve a CTDIvol of 9mGy. Images were reconstructed with a bone kernel, 15cm field-of-view and slice thickness of 0.416mm (PCCT) and 0.625mm (EID). Stent appearance was evaluated by 1) assessment of stent diameter and difference with nominal diameter using a paired sample t-test, 2) subjective stent strut appearance evaluation by two vascular radiologists in consensus using a five-point Likert scale (5= very clear strut margin, 1= impossible to evaluate) and a Wilcoxon signed rank test.
**Results or Findings:** Overall evaluated stents, the mean error in stent diameter towards the nominal value was lower for PCCT (0.14mm ± 0.3mm), compared to EID CT (0.25mm ± 0.67mm), p=0.049. With an average score of 4.5 ± 0.6 (clear to very clear stent margin), the stent strut appearance was perceived better for the PCCT system compared to the EID CT system with an average score of 2.3 ± 1.3 (poor to moderate stent margin), p=0.003.

**Conclusion:** This study shows the potential of superior high spatial resolution with a new silicon-based prototype PCCT detector, allowing more accurate stent measurements and visualisation compared to conventional EID CT systems.
**Limitations:** This was an in-vitro phantom study.
**Ethics committee approval:** Ethical approval was obtained for using human arteries and their transportation to the Karolinska Institute in Stockholm, Sweden, from the ethical committee at VUB/UZ Brussel.
**Funding for this study:** No funding was received for this study.
**Author Disclosures:**
Koenraad Hans Nieboer: Nothing to disclose
Johan De Mey: Nothing to disclose
Emma Verelst: Nothing to disclose
Dominic Crotty: Employee: GE Healthcare
Gert Van Gompel: Nothing to disclose
Paul D Deak: Employee: GE Healthcare
Nico Buls: Nothing to disclose
Frans R.A. Van Den Bergh: Nothing to disclose

**11:30-12:30 Research Stage 1**

### Research Presentation Session: Artificial Intelligence & Machine Learning & Imaging Informatics

### RPS 2405

### Structured reporting and language processing with AI

**Moderator**
M. Huisman; Utrecht/NL
**Author Disclosures:**
Merel Huisman: Board Member: EuSoMII; Employee: Radboud UMC

### RPS 2405-2

**Development of standardised semantics, common data elements, and structured reports within clinical environment**
*N. Cihoric*, K. Nairz, B. Nemeth, H. Von Tengg-Kobligk; Bern/CH
*(nikola.cihoric@gmail.com)*

**Purpose:** Common data elements (CDE), structured reports (SR), and standardised semantics are promising to change radiology reporting. However, there is a lack of resources and engagement from the broader community and data about implementation within the clinical routine. Our goal was to develop a framework for the development of CDE, SR, and semantics for the clinical environment.
**Methods or Backround:** First, we develop a set of standardised procedures for the extraction of clinical knowledge and semantic resources. Afterward, we implemented the workflow for the development and validation of the CDE and SR within open-source software, and we started an integration project with the industry partners. We focused on a single disease, breast cancer.
**Results or Findings:** We develop a set of semantic resources, CDE, and SR, for reporting findings across breast cancer clinical pathways, from screening to treatment response and follow-up. Our CDE and SR are technology-agnostic and can be integrated by any RIS or PACS system.
**Conclusion:** The development of clinically validated CDE and SR coupled with standardised semantic resources is feasible and connected with reasonable efforts.
**Limitations:** This is a monocentric study, which included one disease entity. Broader evaluation in other institutions and other disease entities is mandated.
**Ethics committee approval:** Kantonale Ethikkommission, Murtenstrasse 31, Hörsaaltrakt Pathologie, Eingang 43A, Büro H372, 3010 Bern.
**Funding for this study:** The study is supported by the Swiss Innovation Agency - Innosuisse.
**Author Disclosures:**
Bence Nemeth: Nothing to disclose
Nikola Cihoric: Nothing to disclose
Knud Nairz: Nothing to disclose
Hendrik Von Tengg-Kobligk: Nothing to disclose

### RPS 2405-3

**Natural language processing algorithm trained to perform data labeling of acute rib fractures based on chest CT reports: a proof of concept study**
*N. Bar*, E. Borohovich, B. Rinott, R. Almog, A. Ilivitzki, E. Bercovich; Haifa/IL

**Purpose:** Radiology reports represent good candidates for natural language processing (NLP) algorithms since they tend to contain systematically structured data. We intend to harness an NLP system to automate data labeling of rib fractures, based on CT reports. Labeled data can then be used to train a deep learning algorithm to detect and characterise rib fractures in the original imaging raw data, a process which is normally limited by the need for manual data labeling by a radiologist.
**Methods or Backround:** The training dataset consisted of 1039 reports of trauma protocol CTA chest scans performed at Rambam Medical Center, a tertiary hospital in Israel. A semi-supervised learning model was used to detect textual descriptions of fractured ribs. We compared several models including a self-training classifier, an SGD classifier, and a label spreading model. Based on the intended use of the data, a specificity-driven approach was selected to choose the best model.
**Results or Findings:** The best model was the SGD classifier, which performed with a specificity of 0.98 and an F1 score of 0.8.
**Conclusion:** NLP is a powerful and effective tool enabling mass data labeling based on radiology reports. The output represents a real-life labeling standard which can then be used as training datasets for imaging deep learning algorithms.
**Limitations:** The study focused on one type of fractures and was conducted in a single centre.
**Ethics committee approval:** Rambam healthcare campus Helsinki Committee
**Funding for this study:** No funding was received for this study.
**Author Disclosures:**
Eyal Bercovich: Nothing to disclose
Bar Rinott: Nothing to disclose
Einat Borohovich: Nothing to disclose
Ronit Almog: Nothing to disclose
Nitai Bar: Nothing to disclose
Anat Ilivitzki: Nothing to disclose

### RPS 2405-4

**A knowledge-based framework for the development of a natural language processing (NLP) pipeline and the design of semantically interoperable structured reporting for radiology**
*N. Cihoric*, K. Nairz, B. Nemeth, H. Von Tengg-Kobligk; Bern/CH
*(nikola.cihoric@gmail.com)*

**Purpose:** To evaluate the feasibility of developing complex software specifications intended to be used as "software as a medical device" for the NLP pipeline coupled with chatbot and structured reporting in radiology.
**Methods or Backround:** We implemented a proprietary NLP processing software, developed clinical semantic resources for a chatbot technology, and open-source software for the development of the common data elements and structured reports paired with the HL7 FHIR application programming interface. Together with industry partners, we detailed software development documentation with a project management timeline. The first minimal viable product is planned for Q1 2023.
**Results or Findings:** Our efforts resulted in the specification of an integrated platform for the NLP pipeline with a module for developing and validating structured reports, a patient-oriented chatbot for acquiring anamnestic data, and an application for semantic data exchange. Within the pre-project phase, we installed NLP and structuring and validation software.
**Conclusion:** Planning productive, complex, knowledge-based projects in academic settings is feasible.
**Limitations:** This is a monocentric study. Broader evaluation in other institutions is mandated.
**Ethics committee approval:** Kantonale Ethikkommission, Murtenstrasse 31, Hörsaaltrakt Pathologie, Eingang 43A, Büro H372. 3010 Bern
**Funding for this study:** The study is supported by the Swiss Innovation Agency - Innosuisse.
**Author Disclosures:**
Bence Nemeth: Nothing to disclose
Nikola Cihoric: Nothing to disclose
Knud Nairz: Nothing to disclose
Hendrik Von Tengg-Kobligk: Nothing to disclose

### RPS 2405-5

**Extraction of tumoural response criteria from semi-structured CT-scan reports**
*V. Pohyer*, B. Rance, L. S. Fournier; Paris/FR
*(valentin.pohyer@hotmail.fr)*

**Purpose:** Evaluation of an extraction tool for RECIST tumor data from semi-structured CT-scan reports, development of an interactive visualisation tool.
**Methods or Backround:** The reports come from the data warehouse of the Hôpital Européen Georges Pompidou (HEGP) located in Paris, gathered from 15/01/2001 to 31/12/2021. We used a Python tool developed in-house to extract and normalise 20 items of interest with Natural Language Processing methods relying on 243 regular expressions. We evaluated the performance of the extraction compared to manual annotation of 100 randomly selected reports, as well as the evolution of the presence of the information over time. We developed a visualisation tool in the form of a R-Shiny web application.

**Results or Findings:** Among the 151,092 reports identified over the 21 year-period, 8,957 (5.9%) had an identifiable RECIST conclusion. The global performance of the extraction in terms of precision/recall was >95% for all items. The presence of items was variable: the sum of target lesions, and the categories of response for the target, non-target and new lesions were identified in 98%, 91%, 61% and 80% of the cases respectively. The treatment start date was harder to extract (9%), probably because the radiologists were unaware of this information, but also because of its variability. The content evolved during the study period with gain, loss and stability of items. This can be compared to an evolutionary process with pressurized selection, mutation and derivation.
**Conclusion:** The combination of a big-data approach with a visualisation tool allows radiologists to interrogate and use information extracted from routine patient care.
**Limitations:** Some items like anatomical locations are too variable to be found with regular expressions and require an artificial intelligence.
**Ethics committee approval:** No information provided by the submitter.
**Funding for this study:** Fonds Unique Interministériel (FUI), reference DOS0066427/00 (RIHDO project).
**Author Disclosures:**
Laure S. Fournier: Speaker: Bayer, Novartis, Janssen, Sanofi, General Electric Healthcare, Median technologies Other: Congress suport at Guerbet Other: Member of the scientific commitee of the institut Servier Research/Grant Support: Phillips, Evolucare, ArianaPharma, Siemens, General Electric Healthcare, Dassault Systems
Valentin Pohyer: Nothing to disclose
Bastien Rance: Research/Grant Support: TWINONCO BPI Project Other: Elsevier (Associated Editor of the International Journal of Medical Informatics)

### RPS 2405-6

**Automated structured reporting using a dialogue system based on speech recognition and natural language processing**

*T. Jorg*[1], B. Kämpgen[2], D. Feiler[3], C. Düber[1], P. Mildenberger[1], F. Jungmann[1]; [1]Mainz/DE, [2]Kaiserslautern/DE, [3]Munich/DE

**Purpose:** Structured reporting is the favorable form of radiology reporting. Nevertheless, it still has disadvantages. Reporting templates mostly consist of checkboxes and drop-down menus which require radiologists to use mouse and keyboard for completion. Filling in the templates is time-consuming and bears the risk of distraction from the actual image study. Therefore, better integration of speech recognition into structured reporting is needed. We developed a reporting-tool which can automatically transform dictated free-text into a structured report using natural language processing. Additionally, an integrated chat-bot gives the radiologist feedback if findings are missing.
**Methods or Backround:** The reporting-tool's graphical user interface has two parts: a (HTML)-representation of the to-be-filled structured reporting template, and a chat window for audio-visual communication between the radiologist and a structured reporting bot. The structured reporting-bot does natural language processing, dialogue management with a knowledge base, and natural language generation. The dialogue management determines the findings of the structured report and possible messages to the radiologist.
**Results or Findings:** The reporting-tool was evaluated for the CT retroperitoneum use case. A standard structured reporting template for urolithiasis was applied. The structured reporting-bot was trained with synonyms and messages from 83 examples formulated by a uroradiology expert. The reporting-tool was then tested using free-text reports from our archive (n=50). Inserting these reports showed that the reporting-tool fills in the findings correctly (F1-score: 0.89, precision: 0.96, recall: 0.83) and generates messages to the radiologist for missing findings. Users appreciated the tool and stated that there was less distraction from image studies compared to standard structured reporting. Operability was rated as user friendly and intuitively.
**Conclusion:** Our reporting-tool can be a useful aid in clinical routine.
**Limitations:** There is no integration in the Radiology information system yet.
**Ethics committee approval:** No information provided by the submitter.
**Funding for this study:** BMBF, Project: 16SV8321.
**Author Disclosures:**
Benedikt Kämpgen: Employee: Empolis Information Management
Christoph Düber: Nothing to disclose
Tobias Jorg: Nothing to disclose
Florian Jungmann: Nothing to disclose
Dennis Feiler: Employee: DFC-SYSTEMS GmbH
Peter Mildenberger: Nothing to disclose

### RPS 2405-7

**Retrospective structuring of radiological report databases by efficient on-site development of text-based transformers**

*S. Nowak*[1], D. Biesner[2], Y. C. Layer[1], M. Theis[1], H. Schneider[2], B. Wulff[2], U. I. Attenberger[1], R. Sifa[2], A. M. Sprinkart[1]; [1]Bonn/DE, [2]Sankt Augustin/DE

**Purpose:** Providing insights for on-site development of transformer-based structuring of free-text databases, investigating previously proposed labeling and pre-training strategies.
**Methods or Backround:** The mentioning of the following findings were classified by applying a system based on human-defined rules (termed "silver labels") in 93368 free-text German intensive care unit chest x-ray reports from 20912 patients: infiltrates, pleural effusions, congestion, pneumothorax, correct or incorrect position of the central venous catheter. Of all available reports, 18000 were additionally labeled manually in 197 hours (termed "gold labels") of which 10% were used for testing. Using masked-language-modeling a transformer was pre-trained on-site (Tmlm) and compared to a public, medically pre-trained transformer (Tmed). Training text classification on silver labels only, gold labels only and first with silver and then gold labels (hybrid training) was investigated for both transformers and varying numbers (N: 500, 1000, 2000, 3500, 7000, 14580) of gold-labelled reports. Macro-averaged F1-scores (MAF1) in % with 95% confidence intervals were determined.
**Results or Findings:** When utilising all gold-labeled reports, highest MAF1 was observed for Tmlm,gold (95.5 [94.5-96.3]). Differences between Tmlm, gold (N: 7000, 94.7 [93.5-95.7]) and Tmed, gold (91.5 [90.0-92.8]) were significant when using 7000 gold-labeled reports or less. When using 2000 or more gold-labeled reports, no significant improvement were observed for Tmlm, hybrid (N: 2000, 91.8 [90.4-93.2]) over Tmlm, gold (N: 2000, 91.4 [89.9-92.8]).
**Conclusion:** On-site pre-training of transformers with fine-tuning on manual annotations is an efficient way to unlock report databases for data-driven medicine.
**Limitations:** As the annotators did not have to interpret imaging, but were simply required to mark statements of the attending radiologist, we judged that annotation by supervised medical research assistants was sufficient and costly annotation by board-certified radiologists were not required.
**Ethics committee approval:** Institutional Review Board approval was obtained by the local Ethics Committees at the Medical Faculty of the Rheinische Friedrich-Wilhelms-Universität Bonn (AZ 411/21).
**Funding for this study:** Federal Ministry of Education and Research of Germany (grant no. 01|S18038B).
**Author Disclosures:**
Alois M. Sprinkart: Nothing to disclose
Yannik Christian Layer: Nothing to disclose
Sebastian Nowak: Nothing to disclose
Ulrike I. Attenberger: Nothing to disclose
Helen Schneider: Research/Grant Support: Is affiliated with the Competence Center for Machine Learning Rhine-Ruhr, which is funded by the Federal Ministry of Education and Research of Germany (grant no. 01|S18038B).
Maike Theis: Nothing to disclose
David Biesner: Research/Grant Support: Is affiliated with the Competence Center for Machine Learning Rhine-Ruhr, which is funded by the Federal Ministry of Education and Research of Germany (grant no. 01|S18038B).
Rafet Sifa: Research/Grant Support: Is affiliated with the Competence Center for Machine Learning Rhine-Ruhr, which is funded by the Federal Ministry of Education and Research of Germany (grant no. 01|S18038B).
Benjamin Wulff: Research/Grant Support: Is affiliated with the Competence Center for Machine Learning Rhine-Ruhr, which is funded by the Federal Ministry of Education and Research of Germany (grant no. 01|S18038B).

### RPS 2405-8

**Application of deep learning in generating structured radiology reports: a transformer-based technique**

S. A. R. Moezzi[1], A. Ghaedi[1], M. Rahmanian[1], Z. Mousavi[1], *A. Sami*[2]; [1]Shiraz/IR, [2]Edinburgh/UK
*(a.sami@napier.ac.uk)*

**Purpose:** Since radiology reports are written in free-text narrations, extraction of relative information for further analysis is difficult. In these circumstances, natural language processing (NLP) techniques can facilitate automatic information extraction and transformation of free-text formats to structured data. In recent years, deep learning (DL)-based models have been adapted for NLP experiments with promising results. Despite the significant potential of DL models based on artificial neural networks (ANN) and convolutional neural networks (CNN), the models face some limitations to implement in clinical practice. Transformers, another new DL architecture, have been increasingly applied to improve the process.

**Methods or Backround:** Therefore, in this study, we propose a transformer-based fine-grained named entity recognition (NER) architecture for clinical information extraction. We collected 88 abdominopelvic sonography reports in free-text formats and annotated them based on our developed information schema. The text-to-text transfer transformer model (T5) and Scifive, a pre-trained domain-specific adaptation of the T5 model, were applied for fine-tuning to extract entities and relations and transform the input into a structured format.
**Results or Findings:** Our transformer-based model in this study outperformed previously applied approaches such as ANN and CNN models based on ROUGE-1, ROUGE-2, ROUGE-L, and BLEU scores of 0.816, 0.668, 0.528, and 0.743, respectively, while providing an interpretable structured report.
**Conclusion:** Considering the increasing application of transformer-based NLP models in recent years, using a text-to-text transfer transformer model (T5) and a pre-trained domain-specific adaptation of T5 (Scifive) in this study has shown accepted performance for NER of radiology reports and translation of free-text records to the structured data.
**Limitations:** Although the model has shown perfect performance despite the relatively small corpus of reports used as the train set, more reports should be included to improve the model performance.
**Ethics committee approval:** No information provided by the submitter.
**Funding for this study:** No funding was received for this study.
**Author Disclosures:**
Seyed Ali Reza Moezzi: Nothing to disclose
Ashkan Sami: Nothing to disclose
Mojdeh Rahmanian: Nothing to disclose
Zahra Mousavi: Nothing to disclose
Abdolrahman Ghaedi: Nothing to disclose

**11:30-12:30 Research Stage 2**

### Research Presentation Session: Head and Neck

### RPS 2408

### Sinonasal and dental imaging

**Moderator**
L. Flygare; Umeå/SE
*(lennart.flygare@umu.se)*

### RPS 2408-2

**Volumetric analysis to assess response to neoadjuvant chemotherapy within 2 prospective phase 2 trials in sinonasal epithelial cancers**
*P. Rondi*, M. Ravanelli, I. Buffa, D. Mattavelli, P. Bossi, D. Farina; Brescia/IT
*(paolo.rondi92@gmail.com)*

**Purpose:** The aim of this study is to assess the value of volumetric analysis in predicting outcome of patients with sinonasal epithelial cancers treated by neoadjuvant chemotherapy (NAC) followed by surgery or chemo-radiotherapy within the prospective clinical trials SINTART-1 and SINTART-2.
**Methods or Backround:** Fifty patients treated within the SINTART-1 and -2 clinical trials (NAC followed by locoregional treatment in locally advanced sinonasal epithelial cancers) with pre-treatment, after 1 cycle of NAC, and at the best response (BR) magnetic resonance (MR) were selected. Tumours were measured uni-dimensionally (along three orthogonal vectors) and volumetrically (V). Uni-dimensional measurements were prospectively used to assess treatment response according to RECIST 1.1. Measurements were repeated by two radiologists to assess the interobserver variability. To speed up the manual segmentation of V, interpolation (VI) between non-contiguous slices (maximum of 5 slices manually segmented) was used. The percentage changes in tumour metrics were used in the survival analysis (overall survival, OS; disease-free survival, DFS).
**Results or Findings:** Volume was the most repeatable measure in pre-treatment studies and in terms of percentage variation during treatment. Correlation between manual V and VI was excellent. Differently from RECIST 1.1 and uni-dimensional measures, which did not provide any prognostic correlation, V change of more than 50% after 1 cycle of NAC (HR 0.33 and 0.31, p 0.02 and p 0.01, respectively) and at the BR (HR 0.33 and 0.41, p 0.01 and p 0.04, respectively) correlated with OS and DFS.
**Conclusion:** Volumetric assessment seems to be the most valuable tool to track response to NAC and to predict the outcome, outperforming RECIST 1.1. Tumour segmentation can be significantly speeded up by using interpolation methods.
**Limitations:** Relatively low sample size.
**Ethics committee approval:** No information provided by the submitter.
**Funding for this study:** No funding was received for this study.
**Author Disclosures:**
Irene Buffa: Nothing to disclose
Paolo Bossi: Nothing to disclose
Davide Farina: Nothing to disclose
Paolo Rondi: Nothing to disclose
Marco Ravanelli: Nothing to disclose
Davide Mattavelli: Nothing to disclose

### RPS 2408-3

**Retrospective analysis of dosimetric data from oral and maxollofacial examinations with dedicated CBCT**
*D. Dabli*, C. Louison, B. Lallemant, C. Reynaud, J-P. Beregi, J. Greffier; Nîmes/FR
*(Djamel.Dabli@chu-nimes.fr)*

**Purpose:** To establish local dosimetric reference levels for oral and maxillofacial examinations performed with dedicated cone-beam CT.
**Methods or Backround:** All oral and maxillofacial examinations of adult patients (≥18 years) performed on the dedicated Newtom 5G Cone beam CT (CBCT) equipement were included over a period of 5 consecutive months. Dosimetric indicators: CT dose index (CTDI) and dose length product (DLP) of these examinations were retrospectively collected and analysed. Local dosimetric reference levels (LDRL) were established for each examination in terms of mean ± standard deviation. For inner ear and sinus examination, the LDRL values with CBCT were compared to those of multi-slice CT (MSCT).
**Results or Findings:** 231 examinations were performed during the study period. Sinus examinations accounted for 38.1% of the total number of examinations, 23.3%, 23.2%, 13.1% and 4.3% accounted for facial massif, inner ear, salivary gland, dental panoramic examinations respectively. The mean age of all patients was 50.7±18.3 years and 52% of the patients were male. The highest mean CTDI and DLP values were observed for the inner ear examinations with a mean of 24.71±5.75 mGy and 169.61±34.13 mGy*cm respectively. The mean CTDI was 9.85±1.83, 9.52±2.29, 9.32±2.56 and 8.71±1.82 for the sinus, dental panoramic, salivary glands, facial massif examinations respectively. The mean DLP values were 146.38±31.08, 129.13±25.38, 132.26 ± 34.45 and 156.98±37.47 mGy*cm respectively. For inner ear, the mean CTDI and DLP were significantly (p<0.01) lower (-50.93% and 58.33%) than with MSCT. Opposite trend were found for sinus examination with CTDI and DLP significantly higher with CBCT (46.2%, 99.1% respectively).
**Conclusion:** The highest CTDI and DLP values with CBCT were for inner ear. The LDRL values were lower with CBCT than MSCT for inner ear and higher for sinus examinations.
**Limitations:** Monocentric study.
**Ethics committee approval:** No information provided by the submitter.
**Funding for this study:** No funding was received for this study.
**Author Disclosures:**
Jean-Paul Beregi: Nothing to disclose
Christophe Reynaud: Nothing to disclose
Camille Louison: Nothing to disclose
Benjamin Lallemant: Nothing to disclose
Joel Greffier: Nothing to disclose
Djamel Dabli: Nothing to disclose

### RPS 2408-4

**Use of virtual monoenergetic images and iterative metal artifact reduction for reduction of dental implant associated artifacts in photon-counting CT**
*Y. C. Layer*, N. Mesropyan, P. A. Kupczyk, J. A. Luetkens, A. Isaak, T. Dell, U. I. Attenberger, D. Kütting; Bonn/DE
*(Yannik.Layer@ukbonn.de)*

**Purpose:** Aim of this study was to assess the impact of photon-counting CT (PCCT) derived virtual monoenergetic images (VMI) and iterative metal artifact reduction (IMAR) on dental implant associated artifacts.
**Methods or Backround:** For this retrospective study 37 scans with dental implant associated artifacts acquired during clinical routine on a PCCT from 08/2022- 09/2022 were analysed. VMI were reconstructed for 90-190 keV (10 keV steps) with and without IMAR and compared to polychromatic images. Artifact extent and assessment of adjacent soft tissue were rated by two radiologists using a 5-point Likert grading scale for qualitative assessment. Quantitative assessment was performed using ROIs measuring attenuation and standard deviation in most pronounced hypodense and hyperdense artifacts, artifact-impaired soft tissue and artifact-free muscle tissue. An adjusted attenuation was calculated as difference between artifact-impaired soft tissue and muscle tissue without artifacts for quantification of artifacts.
**Results or Findings:** Qualitative assessment for all investigated image reconstructions significantly improved compared to polychromatic images (PI). VMI of 130 keV and 170 keV with combination of IMAR achieved the best results (e.g. diagnostic quality of soft palate: median PI: 1 (Range: 1-3); VMI130kev+IMAR: 3.5 (2-5); p < 0.05). In quantitative assessment VMI of 110keV with IMAR showed the best results with an adjusted attenuation closest to 0 (PI: 39.13 ± 35; VMI110keV+IMAR: -0.24 ± 14.34; p<0.05).

**Conclusion:** The combination of VMI and IMAR significantly reduces dental implant associated artifacts in PCCT and effectively improves the diagnostic quality of the craniomandibular region. VMI of 130 keV in combination with IMAR showed best results and are recommended to support head and neck scans with artifacts.
**Limitations:** Study design is retrospective and single-centred, investigating a small cohort.
**Ethics committee approval:** No information provided by the submitter.
**Funding for this study:** Y.C. Layer is supported by a research grant from Siemens Healthcare.
**Author Disclosures:**
Patrick Arthur Kupczyk: Nothing to disclose
Julian Alexander Luetkens: Nothing to disclose
Tatjana Dell: Nothing to disclose
Narine Mesropyan: Nothing to disclose
Alexander Isaak: Nothing to disclose
Yannik Christian Layer: Research/Grant Support: Yannik Christian Layer is supported by a research grant from Siemens Healthcare GmbH.
Ulrike I. Attenberger: Nothing to disclose
Daniel Kütting: Nothing to disclose

### RPS 2408-5

**Periodontal disease can be monitored through intraosseous oedema extent in dental MRI**

*J. Schwarting*, F. A. Probst, M. Griesbauer, T. Robl, E. Burian, B. Wiestler, T. Brunner, M. Folwaczny, M. Probst; Munich/DE
*(julian.schwarting@tum.de)*

**Purpose:** Periodontal disease can be visualised in 3T MRI with T2-weighted STIR and Fast Field Echo T1-weighted Black Bone sequences, where hyperintense oedema and bone loss correlate to the typical clinical findings periodontal pocket depth and gum bleeds. The aim of this study was to investigate the reversibility of imaging findings through standard treatment in the timespan of 3 months in correlation to the clinical examination status, in order to determine whether MRI imaging can be used to monitor disease activity.
**Methods or Backround:** 36 patients with generalised periodontitis were prospectively analysed and were imaged with 3D isotropic T2-weighted STIR and Fast Field Echo T1-weighted Black Bone sequences before and 3 months after standard therapy. Bone oedema depth was measured in 164 teeth in the STIR sequence before and after treatment. Results were compared with standardised clinical examinations according to the periodontal screening index (PSI).
**Results or Findings:** Periodontitis results in bone oedema adjacent to affected teeth with an average depth of 1.55 (2.35)mm. 3 months after therapy, mean oedema depth shrunk to 1.11 (2.08) mm, $p<.01$. Periodontal treatment reduced the frequency of sites with oedema from 53.3% to 38.8% ($p<0.01$). Bivariate regression analysis revealed strong linkage of the preoperative size of the osseous oedema with successful treatment (OR 1.74 95% CI: 1.12-2.71; $p=0.015$).
**Conclusion:** Periodontal osseous oedema in T2 STIR imaging can be used to monitor inflammation in patients with high-grade periodontal disease.
**Limitations:** Apart from primary inflammatory conditions, excessive functional stress can also cause osseous oedema and limit diagnostic accuracy. Intraosseous oedema can also occur as part of chronic inflammation or after endodontal or surgical treatment. In some cases, gingival inflammation can be difficult to distinguish from bone oedema through MR imaging.
**Ethics committee approval:** No information provided by the submitter.
**Funding for this study:** No external funding was used.
**Author Disclosures:**
Teresa Robl: Nothing to disclose
Monika Probst: Nothing to disclose
Magdalena Griesbauer: Nothing to disclose
Matthias Folwaczny: Nothing to disclose
Teresa Brunner: Nothing to disclose
Julian Schwarting: Nothing to disclose
Florian Andreas Probst: Nothing to disclose
Egon Burian: Nothing to disclose
Benedikt Wiestler: Nothing to disclose

### RPS 2408-6

**Iterative metal artifact reduction facilitates tumour assessment in head and neck CT compromised by dental hardware: analysis of appropriate reconstruction presets for optimised tumour visualisation**

*N. C. Bayerl*[1], M. S. May[1], B. Schmidt[2], C. Hofmann[2], J-P. Roth[1], M. Uder[1], S. Ellmann[1]; [1]Erlangen/DE, [2]Forchheim/DE

**Purpose:** The purpose of this study was to evaluate an iterative metal artifact reduction (iMAR) algorithm in CT imaging of oral and oropharyngeal cancer when tumour visualisation is compromised by metal artifacts. In addition, the most appropriate algorithm preset to optimise image quality was to be determined.
**Methods or Backround:** The raw data of 27 consecutive patients with contrast-enhanced neck CT and histologically confirmed oral or oropharyngeal cancer affected by metal artifacts from dental hardware, were post-processed using five iMAR levels with ascending intensities (levels 1/2/3/4/5) and one reconstruction without metal artifact reduction (filtered back projection; FBP). The images were evaluated subjectively by two blinded radiologists with 5 and 10 years of experience, who assessed tumour contrast and edge delineation on a 5-point Likert scale. Objective analysis was performed by determining the signal-to-noise ratio (SNR) and artifact index. Intergroup comparisons were performed using Friedman-ANOVA tests.
**Results or Findings:** iMAR reconstructions improved tumour assessment subjectively as well as objectively. Subjectively, both tumour contrast and edge delineation improved significantly with all iMAR levels compared to FBP reconstructions, reaching their maxima at iMAR levels 4 and 5 ($p<0.05$). The readers were in excellent agreement regarding their subjective assessment (Intraclass Correlation Coefficient ICC=0.84). Objectively, SNR and artifact index improved significantly under iMAR ($p<0.05$), each reaching the optimum at iMAR level 5.
**Conclusion:** iMAR levels 4 and 5 are the most appropriate presets for CT imaging of oral and oropharyngeal cancer in patients with dental hardware and significantly improve tumour assessment when images are affected by metal artifacts.
**Limitations:** This retrospective study included a small sample size. Also, there may be vendor-specific differences between metal artifact reduction algorithms that were not addressed by evaluating only one vendor's tool.
**Ethics committee approval:** Friedrich-Alexander-University of Erlangen-Nuremberg.
**Funding for this study:** No funding was received for this study.
**Author Disclosures:**
Matthias Stefan May: Speaker: Speakers bureau of the Siemens Healthcare GmbH
Stephan Ellmann: Nothing to disclose
Bernhard Schmidt: Employee: Siemens Healthcare GmbH
Jan-Peter Roth: Nothing to disclose
Michael Uder: Speaker: Speakers bureau of the Siemens Healthcare GmbH
Nadine Christina Bayerl: Nothing to disclose
Christian Hofmann: Employee: Siemens Healthcare GmbH

### RPS 2408-7

**The Cone Beam Computed Tomography practice, knowledge and medico-legal issues by Israeli dental clinicians**

R. Abdalla-Aslan[1], G. Almoznino[2], L. Moskovich[3], D. J. Aframian[2], *C. Nadler*[2]; [1]Haifa/IL, [2]Jerusalem/IL, [3]Nahariya and Jerusalem/IL
*(nadler@hadassah.org.il)*

**Purpose:** To assess Cone Beam Computed Tomography (CBCT) clinical use, knowledge and medico-legal issues by Israeli dental clinicians.
**Methods or Backround:** The use of CBCT is rapidly expanding in various dental fields. An anonymous questionnaire assessing clinical use (including requested output: cross sectional images (CSI), DICOM-files, interpretation report), knowledge, and medico-legal CBCT issues was distributed in conferences and via e-mails.
**Results or Findings:** Our survey included 387 dentists (M:F-1:1.5; mean age=44.2±12.0; ~38% response rate), of whom 58.1% were certified specialists. Most (93%) refer to CBCT, mainly to implants planning (27%) and impacted 3rd molars (24%). As the CBCT output, 31%, 42% and 9% of the responders requested CSI, CSI with DICOM-files and DICOM-files, respectively. Among responders who stated they always review the whole imaging volume, 38% never ask for DICOM-files, and only 36% always ask for DICOM-files. Most dentists (85.9%) had encountered incidental findings within a scan. The average knowledge test score was 68.1%. Higher scores and previous CBCT training correlated with younger age ($p=0.001$ and $p<0.001$, respectively). Only a third of the participants stated that they always review the whole imaging volume. 41.7% believed that the referring clinician should review the scan, while 57.1% thought that it should be a specialised radiologist. Most responders (70.7%) were interested in CBCT continuing-education (CE) course.
**Conclusion:** Different CBCT output are requested. Most dentists, although aware of the possibility of incidental findings, do not review the whole imaging volume. CBCT knowledge was better demonstrated by younger dentists, suggesting CE CBCT courses are mostly needed for senior dentists.
**Limitations:** The results of this study may be biased since responders were mostly experts, who voluntarily agreed to participate.
**Ethics committee approval:** The study was approved by our institutional review board (0726-18-HMO).
**Funding for this study:** No funding were allocated for this study.
**Author Disclosures:**
Liad Moskovich: Nothing to disclose
Galit Almoznino: Nothing to disclose
Chen Nadler: Nothing to disclose
Doron J. Aframian: Nothing to disclose
Ragda Abdalla-Aslan: Nothing to disclose

**RPS 2408-8**
**Assessability of the nasopalatine and inferior alveolar nerves with a dedicated dental MRI coil compared with CBCT**
*T. E. Lichtenstein*, A. Grandoch, J. Oeser, J. E. Zöller, N. Grosse Hokamp, J. Neugebauer; Cologne/DE

**Purpose:** A high risk of nerve injury is inherent in numerous oral and maxillofacial surgery procedures. Preoperative imaging can simplify the planning and execution of surgery and help to protect nerves. This study compared the assessability of nerves in the oral cavity using a 1.5 T magnetic resonance imaging (MRI) scanner with a dedicated dental signal amplification coil versus cone beam computed tomography (CBCT).
**Methods or Backround:** An additional MRI was obtained in 24 patients with routine CBCT examination. Six predefined criteria were used to evaluate the assessability of the inferior alveolar and nasopalatine nerves on CBCT and MRI using a special 4-channel dental coil.
**Results or Findings:** The assessability of the inferior alveolar nerve was significantly better in both the sagittal and axial planes, and that of the nasopalatine nerve was significantly better in the axial plane by MRI with the dental coil than by CBCT. In contrast, the assessability of the nasopalatinal nerve in the sagittal plane was significantly better on CBCT than on MRI. The overall assessability was not significantly different between the modalities.
**Conclusion:** The results of this feasibility study suggest that 1.5 T MRI with a special dental coil is equivalent, if not superior, to CBCT in imaging nerve structures of the stomatognathic system. However, MRI, contrary to CBCT, does not cause radiation exposure and allows visualisation of all relevant hard and soft tissues and therefore gives an advantage over the established technique.
**Limitations:** Study limitations include the use of a routine clinical imaging protocol and a 1.5 T MRI.
**Ethics committee approval:** No information provided by the submitter.
**Funding for this study:** No funding was received for this study.
**Author Disclosures:**
Joachim E. Zöller: Nothing to disclose
Jörg Neugebauer: Speaker: Dentsply Sirona
Thorsten Enno Lichtenstein: Nothing to disclose
Nils Große Hokamp: Research/Grant Support: Philips Speaker: Philips Consultant: Bristol Myers Squibb
Julia Oeser: Nothing to disclose
Andrea Grandoch: Nothing to disclose

**RPS 2408-9**
**Clinical validation of AI tools for automated tumour delineation on PET-CT**
*D. G. K. Petersen*, C. N. Ladefoged, F. L. Andersen, B. M. Fischer; Copenhagen/DK
*(david.gergely.kovacs.petersen@regionh.dk)*

**Purpose:** To test if openly available artificial intelligence (AI) is good enough for automated tumour delineation in clinical practice. This could allow for routine prognostic use of PET-CT biomarkers and alleviate the workload associated with tumour delineation prior to radiotherapy planning.
**Methods or Backround:** Patients scanned between January 1, 2014 and December 31, 2019 at Rigshospitalet were included retrospectively to train promising readily available AI models to predict the PET-positive tumour volume (GTV-PET) using PET-CT images as input. 1095 patients were included; 835 were used for training and validation, 196 for comparing methods and 64 for comparison to clinical performance. On the latter, six experienced nuclear medicine specialists delineated each case twice to measure clinical performance as basis for comparison to AI. Biomarker reproducibility using AI was evaluated in a Bland-Altman analysis.
**Results or Findings:** Five methods were selected based on a literature review for implementation and testing. The highest performing method, nnUNet (F. Insensee et al., doi: 10.1038/s41592-020-01008-z), achieved a DICE-coefficient of 0.80 [0.77:0.86]. We found no significant difference between clinical delineation performance (0.82 ± 0·20) and the AI method (p=0.12). Biomarkers were reliable with no considerable differences between AI and physicians' performance. For physicians as well as AI, the pattern of outliers showed that in rare cases, images alone do not provide sufficient information to generate the final clinical GTV-PET.
**Conclusion:** AI matches clinical performance and can be used reliably to support delineation of GTV-PET and for automated biomarker extraction.
**Limitations:** While we did not make any changes to the implemented methods, in-house data was used for training, marking further external validation desirable. Human supervision is necessary to account for contextual clinical information in rare cases.
**Ethics committee approval:** No information provided by the submitter.
**Funding for this study:** Louis-Hansen Fonden, Hartmann Fonden, Capital Region of Denmark.
**Author Disclosures:**
Barbara Malene Fischer: Nothing to disclose
Flemming Littrup Andersen: Nothing to disclose
Claes Nøhr Ladefoged: Nothing to disclose
David Gergely Kovacs Petersen: Nothing to disclose

**11:30-12:30** **Research Stage 3**

### Research Presentation Session: Oncologic Imaging

### RPS 2416

### Advances in CT for oncological imaging: dual-energy

**Moderator**
S. Mulé; Paris/FR
*(sebastien.mule@aphp.fr)*

**RPS 2416-2**
**Virtual non-calcium imaging for qualitative and quantitative assessment of bone marrow involvement in multiple myeloma: our experience with dual-energy computed tomography**
*A. Onori*, S. Ruggiero, C. Di Manna, D. M. Bellini, M. Rengo, I. Carbone; Rome/IT
*(alexonori@yahoo.com)*

**Purpose:** The aim of this study was to compare the diagnostic performance of virtual non-calcium imaging (VNCa) and Conventional Computed Tomography imaging (CCT) in the evaluation of bone marrow (BM) involvement in patients affected by multiple myeloma and to investigate the impact of VNCa values on patients' prognoses.
**Methods or Backround:** In this mono-centred retrospective study, consecutive patients with MM who underwent Dual Energy Computed Tomography (DECT) in the last 12 months were enrolled. Two readers randomly and independently reviewed two datasets of images per patient (VNCa and monochromatic 120kVp-like) for the presence of BM involvement. The number and dimension of BM lesions were compared on a per segment analysis. CT numbers of BM lesions and unaffected bone were measured on VNCa images. Results were stratified according to the disease status (stable or progression).
**Results or Findings:** Significantly more lesions (n=748) were identified on VNCa images compared to CCT (n=502) (p<0,0001). No significant differences were observed for lesions' dimensions (18,72±9,68 mm, p=0,6352). VNCa CT numbers showed a significant difference between lesions in patients with stable disease and lesions in patients with progressive disease (-143,67±202,63 HU vs 4,54±51,95 HU; p=0,0087). There was almost perfect agreement for both number and dimension of the lesions among readers (ICC = 0.93 [95% CI 0.84-0.71] and ICC = 0.89 [95% CI 0.75-0.96], respectively).
**Conclusion:** VNCa is superior to CCT in identifying BM involvement in MM and ROI-based analysis of the lesions could also be an interesting tool for its prognostic implications.
**Limitations:** Not applicable
**Ethics committee approval:** No information provided by the submitter.
**Funding for this study:** Not applicable
**Author Disclosures:**
Alessandro Onori: Nothing to disclose
Iacopo Carbone: Nothing to disclose
Marco Rengo: Nothing to disclose
Sergio Ruggiero: Nothing to disclose
Davide Maria Bellini: Nothing to disclose
Caterina Di Manna: Nothing to disclose

**RPS 2416-3**
**Improved visualisation of liver metastases in malignant melanoma patients using unenhanced dual-energy CT collagen imaging**
*M. Dimitrova*, C. Booz, T. Vogl, L. D. Grünewald, S. Martin, A. Gökduman, I. Yel; Frankfurt am Main/DE

**Purpose:** The aim of this study was to evaluate a novel dual-energy CT collagen imaging algorithm for coloured depiction of malignant melanoma liver metastases compared with standard CT image reconstruction.
**Methods or Backround:** In this retrospective study 42 malignant melanoma patients (71 hypodense liver lesions, mean age: 59 year, 19 women and 23

men) who had undergone clinically indicated third-generation dual-source DECT-staging scans between September 2019 and September 2022 were included. Three blinded board-certified radiologists independently evaluated conventional gray-scale dual-energy unenhanced CT series for the presence of melanoma liver metastasis. After four weeks, readers re-evaluated examinations using coloured dual-energy CT collagen maps based on material decomposition. Additional performed MRI analysed by two experienced board-certified radiologists, blinded to CT and clinical information, or biopsy served as a standard of reference. Sensitivity and specificity were the primary indices of diagnostic accuracy.
**Results or Findings:** A total of 101 lesions were evaluated in non-enhanced DECT images (41 metastases and 60 cysts). Colour-coded collagen maps yielded significantly higher overall sensitivity (84% vs 72%) and specificity (91% vs 83%) for depicting melanoma liver metastases compared with standard CT (all comparisons, P<.001). Collagen maps showed substantial interreader agreement (K = 0.82) while standard CT showed moderate interreader agreement (K = 0.69). DECT collagen imaging provided superior diagnostic confidence (mean scores 4.85 vs 2.94) and image quality (mean scores 4.50 vs 3.15) compared with standard CT images (all comparisons, P<.001).
**Conclusion:** DECT-based collagen imaging can be used to significantly improve diagnostic accuracy and confidence for the detection of liver metasases in patients with malignant melanoma in unenhanced CT images. Particularly patients with impaired renal function, hyperthyroidism, or allergies to iodine-containing contrast media may benefit from this novel imaging post-processing algorithm.
**Limitations:** This was a single-centre retrospective study.
**Ethics committee approval:** The IRB approved this study.
**Funding for this study:** No funding was received for this study.
**Author Disclosures:**
Simon Martin: Speaker: Siemens Healthineers
Christian Booz: Speaker: Siemens Healthineers
Ibrahim Yel: Speaker: Siemens Healthineers
Mirela Dimitrova: Nothing to disclose
Thomas Vogl: Nothing to disclose
Aynur Gökduman: Nothing to disclose
Leon David Grünewald: Nothing to disclose

### RPS 2416-4

**Quantitative parameters derived from contrast-enhanced dual layer spectral CT on predicting macrotrabecular-massive subtype in single hepatocellular carcinoma**

M. Li[1], A. Li[1], Z. Lina[1], J. Zhou[1], W. Deng[2], Z. Zhang[2], *J. Wang*[1]; [1]Guangzhou/CN, [2]Shanghai/CN

**Purpose:** The aim of this study was to evaluate the potential diagnostic performance of quantitative parameters obtained from contrast-enhanced Dual Layer Spectral CT (DLSCT) to non-invasively predicting macrotrabecular-massive (MTM) subtype in single hepatocellular carcinoma (HCC).
**Methods or Backround:** Fifty-seven histopathology proven HCC patients with preoperative DLSCT examinations (+/−MTM, n = 14/43) before liver resection were enrolled retrospectively from 06/2020 to 07/2021. The effective atomic number (Zeff) and the normalised iodine density (NID) values of tumour areas (without haemorrhage or necrosis regions) in both arterial phase (AP) and portal venous phase (PVP) were measured. NID was calculated by taking the ratio of iodine density between the tumour and the aorta of the same scan slice. Clinical and radiological features were analysed by logistic regression analysis to establish a clinical-radiological (CR) model. Area under the receiver operating characteristic curve (AUC) was used to assess the diagnostic performance of quantitative parameters, CR model and combination of them for predicting MTM-HCC subtype.
**Results or Findings:** Among four quantitative parameters, NID in PVP showed good performance in identifying positive-MTM subtype (AUC:0.79; 95% CI: 0.67-0.91). The multivariate analyses found that AFP level > 400 ng/mL, nonsmooth tumour margin and intratumour haemorrhage were independent risk factors for prediction of positive-MTM-HCC subtype, and the predictive CR model was constructed from the above three variables. Incorporating NID value in PVP obviously improved the performance of CR model (CR model, AUC: 0.82 [95% CI: 0.69-0.91], sensitivity, 64.3%, specificity, 90.7%; the combination model, AUC: 0.89; [95% CI: 0.78-0.96], sensitivity, 92.9%, specificity, 72.7%) (p=0.05).
**Conclusion:** NID value in PVP derived from DLSCT was a promising biomarker for identifying MTM subtype in single HCC. When combined with a CR model, NID in PVP could improve performance in predicting positive MTM-HCC subtype.
**Limitations:** Not applicable
**Ethics committee approval:** No information provided by the submitter.
**Funding for this study:** Not applicable
**Author Disclosures:**
Zhang Lina: Nothing to disclose
Jinhui Zhou: Nothing to disclose
Jin Wang: Nothing to disclose
Zhongping Zhang: Nothing to disclose
Mengsi Li: Nothing to disclose
Anqi Li: Nothing to disclose
Weiwei Deng: Nothing to disclose

### RPS 2416-5

**Soft reconstruction kernels improve image quality of HCC on a Photon-Counting Detector CT**

*D. Graafen*, L. Müller, M. C. Halfmann, F. Stoehr, C. Düber, Y. Yang, T. S. Emrich, R. Kloeckner; Mainz/DE
*(dirk.graafen@unimedizin-mainz.de)*

**Purpose:** Photon-Counting Detector CT (PCD-CT) has the potential to considerably improve abdominal oncologic imaging. In particular, imaging of hepatocellular carcinoma (HCC) might profit to a great extent, since a characteristic signal behaviour of the tumour allows for non-invasive diagnosis without histologic proof. The aim of this study was to investigate the influence of different reconstruction kernels on the image quality of triple-phase liver PCD-CT for imaging of HCC.
**Methods or Backround:** A total of 24 patients with viable HCC lesions were scanned on a first-generation PCD-CT. Virtual monoenergetic images at 50 keV were reconstructed using a regular body and quantum kernel, each with four different sharpness levels (Br36, Br40, Br44, Br48, Qr36, Qr40, Qr44, and Qr48). Quantitative image analysis included noise and contrast-to-noise ratio (CNR) of the lesions. Using a 5-point Likert scale, three raters performed a qualitative image analysis evaluating noise, contrast, lesion conspicuity, and overall quality.
**Results or Findings:** In all contrast phases, CNR was the highest, using kernels with a sharpness level of 36 (all p<0.05). Softer reconstruction kernels were also rated better regarding noise and image quality (all p<0.05). No significant differences were found in image contrast and lesion conspicuity. Comparing body and quantum kernels with equal sharpness levels, we found no difference in all image quality criteria.
**Conclusion:** Soft reconstruction kernels provide best CNR and overall image quality for the evaluation of HCC in triple-phase PCD-CT. Usage of quantum kernels with the potential of spectral post-processing shows no restrictions in image quality compared to regular body kernels and should therefore be preferred.
**Limitations:** The main limitation of the study is its single-centre design, featuring a moderate number of patients.
**Ethics committee approval:** No information provided by the submitter.
**Funding for this study:** No funding was received.
**Author Disclosures:**
Roman Kloeckner: Nothing to disclose
Dirk Graafen: Nothing to disclose
Fabian Stoehr: Nothing to disclose
Christoph Düber: Nothing to disclose
Tilman Stephan Emrich: Advisory Board: Siemens Healthineers
Moritz Christian Halfmann: Nothing to disclose
Lukas Müller: Nothing to disclose
Yang Yang: Nothing to disclose

### RPS 2416-6

**Rho/Z maps derived from dual-energy CT for improved liver lesion differentiation in malignant melanoma patients**

*M. Dimitrova*, I. Yel, T. Vogl, S. Martin, L. D. Grünewald, A. Gökduman, C. Booz; Frankfurt am Main/DE

**Purpose:** This study aims to evaluate the diagnostic accuracy of dual-energy computed tomography (DECT)-based Rho/Z-maps generated from non-enhanced images for differentiation of hypodense liver lesions in patients with diagnosed malignant melanoma compared with conventional CT value measurements.
**Methods or Backround:** 42 malignant melanoma patients were included in this retrospective study. All patients had undergone a routinely performed third-generation dual-source DECT scan for staging of malignant melanoma. Rho (electron density) value and Z (effective atomic number) values as well as Hounsfield units (HU) were measured in hypodense liver lesions. Values were compared, and diagnostic accuracy for differentiation was computed using receiver operating characteristic (ROC) curve analyses. Additional performed MRI or biopsy served as a standard of reference.
**Results or Findings:** A total of 101 lesions were evaluated in non-enhanced DECT images (41 metastases and 60 cysts). Z parameters (mean difference [MD], -2.141 ± 0.1814; percentage difference [PD],121%) showed the greatest difference between melanoma metastases and benign cysts, followed by Rho (MD, -27.40 ± 3.467; PD, 118%) and HU120kV (MD, -25.20 ± 2.828; PD, 94%). Furthermore, Z and Rho measurements (Z, 0.98; 95% CI, 0.88-1; Rho, 0.98;

95% CI, 0.85-1) showed higher AUC values for lesion differentiation than HU120kV (0.97; 95% CI, 0.81-1).
**Conclusion:** Compared with conventional CT measurements, DECT measurements allow for improved differentiation of liver metastases and cysts in patients with malignant melanoma. This is particularly beneficial for patients with impaired renal function, hyperthyroidism, or allergies to iodine-containing contrast media.
**Limitations:** This was a single-centre retrospective study.
**Ethics committee approval:** The IRB approved this study
**Funding for this study:** No funding was received for this study.
**Author Disclosures:**
Simon Martin: Speaker: Siemens Healthineers
Christian Booz: Speaker: Siemens Healthineers
Ibrahim Yel: Speaker: Siemens Healthineers
Mirela Dimitrova: Nothing to disclose
Thomas Vogl: Nothing to disclose
Aynur Gökduman: Nothing to disclose
Leon David Grünewald: Nothing to disclose

### RPS 2416-7

**Rho/Z maps derived from dual-energy CT for differentiation of hypodense liver lesions in patients with malignant melanoma**
*C. Booz*, I. Yel, A. Gökduman, L. D. Grünewald, S. Martin, L. S. Alizadeh, T. J. Vogl, M. Dimitrova; Frankfurt/DE

**Purpose:** This work aims to evaluate the diagnostic accuracy of dual-energy computed tomography (DECT)-based Rho/Z-maps for differentiation of hypodense liver lesions in patients with diagnosed malignant melanoma compared with conventional CT value measurements.
**Methods or Backround:** This retrospective study included 73 patients (mean age, 70 ± 13 years; 43 men and 30 women) suffering from malignant melanoma who had undergone third-generation dual-source DECT as part of tumour staging between December 2017 and December 2021. For this study, we measured Rho (electron density) and Z (effective atomic number) values as well as Hounsfield Units (HU) in hypodense liver lesions. Values were compared, and diagnostic accuracy for differentiation was computed using receiver operating characteristic (ROC) curve analyses. Additional performed MRI or biopsy served as a standard of reference.
**Results or Findings:** A total of 122 lesions (51 metastases and 71 cysts) in contrast-enhanced DECT images were evaluated. The greatest difference between measured values for melanoma metastases and benign cysts was observed for Z parameters (mean difference (MD), -1.613 ± 0.0921, 110%), followed by Rho (MD, -34.71 ± 3.318, 88%) and finally HU120kV (MD, -22.46 ± 3.007, 63%). Furthermore, the Z and Rho measurements showed an higher AUC value (Z, 0.992; 95% CI, 0.956-1; Rho, 0.908; 95% CI, 0.842-0.953) compared with HU120kV (0.829; 95% CI, 0.751-0.891) for lesion differentiation.
**Conclusion:** Rho and Z measurements derived from DECT allow for improved differentiation of liver metastases and cysts in patients with malignant melanoma compared with conventional CT value measurements. Therefore, if technically available, Rho/Z maps and corresponding measures should be applied in case of hypodense liver lesions in this patient group.
**Limitations:** Retrospective single-centre study design.
**Ethics committee approval:** The IRB approved this study.
**Funding for this study:** No funding was received for this study.
**Author Disclosures:**
Simon Martin: Speaker: Siemens Healthineers
Christian Booz: Speaker: Siemens Healthineers
Ibrahim Yel: Speaker: Siemens Healthineers
Mirela Dimitrova: Nothing to disclose
Aynur Gökduman: Nothing to disclose
Thomas J. Vogl: Nothing to disclose
Leona Soraja Alizadeh: Nothing to disclose
Leon David Grünewald: Nothing to disclose

### RPS 2416-8

**Dual-layer spectral detector CT-based extracellular volume fraction to predict synchronous liver metastases in colorectal cancer**
*W. Peng*, L. Wan, S. Dong, H. Zhang; Beijing/CN

**Purpose:** To explore whether the dual-layer spectral detector CT (DLCT)-based parameters, especially the extracellular volume fraction (ECVf) can predict synchronous liver metastases (SLM) in colorectal cancer (CRC).
**Methods or Backround:** This prospective study selected patients on suspicion of CRC between April 2021 and April 2022 consecutively to undergo DLCT (pre-contrast, venous, and equilibrium phase contrast-enhanced). Patients with confirmed CRC by biopsy were enrolled. Lesions' quantitative spectral parameters (including iodine concentration (IC), normalised IC (NIC), Z-effective value (ZV), and normalised ZV (NZV)) and mean attenuation on conventional 120-kVp images of each phase were extracted. ECVf based on IC (ECVf-iodine) and mean attenuation (ECVf-attenuation) were calculated separately based on the equilibrium phase. Abdominal enhanced CT or MRI were performed at the primary diagnosis and during a six-month follow-up to identify the status of SLM. Each parameter was compared between the SLM and non-SLM groups. Receiver-operating characteristic (ROC) curve analysis was performed to evaluate the predicting efficiency.
**Results or Findings:** A total of 207 patients (median age, 56 years; interquartile range, 42–76 years; 122 men) with 57 SLMs were enrolled. Parameters of IC, NIC, ZV, NZV, and mean attenuation showed no difference between the SLM group and non-SLM group (all P＞0.05). The ECVf-iodine was significantly higher in the SLM group (36.7 ± 6.7 %) than in the non-SLM group (28.3 ± 4.6 %, P < 0.05). Good correlation was seen between ECVf-iodine and ECVf-attenuation (r=0.94, P=0.006). The AUC of ECVf-iodine was 0.869 for predicting SLM (cut-off value, > 30.9 %), which was significantly higher than that of ECVf-attenuation (AUC, 0.731, P < 0.05).
**Conclusion:** The calculated ECVf-iodine from DLCT enabled an ideal predictive performance of synchronous liver metastases in colorectal cancer.
**Limitations:** Lack of external validation.
**Ethics committee approval:** Approved by the local ethics committee and written informed consent obtained from all subjects.
**Funding for this study:** National Natural Science Foundation of China(81971589).
**Author Disclosures:**
Shushan Dong: Nothing to disclose
Lijuan Wan: Nothing to disclose
Hongmei Zhang: Nothing to disclose
Wenjing Peng: Nothing to disclose

**11:30-12:30** **Research Stage 4**

### Research Presentation Session: Radiographers

### RPS 2414

### The future of the radiography profession

**Moderators**
M. Raissaki; Iraklion/GR
*(mraissaki@yahoo.gr)*
G. Unterhumer; Vienna/AT
*(gerold.unterhumer@fh-campuswien.ac.at)*

### RPS 2414-3

**Artificial intelligence impact on radiographers' activities and profession in Switzerland**
*M. Champendal*[1], S. E. de Labouchere[1], I. Gremion[1], S. S. Ghotra[1], S. Torre[2], R. Khine[3], L. Marmy[1], C. Malamateniou[2], C. S. d. Reis[1]; [1]Lausanne/CH, [2]London/UK, [3]Wycombe/UK
*(Melanie.Champendal@hesav.ch)*

**Purpose:** To explore the possible impact of AI on radiographers' activities and profession in Switzerland based on clinical radiographers, teaching-staff, students and physicians (radiologists/nuclearists/radio-oncologists) perspectives.
**Methods or Backround:** A previously validated survey in the UK was translated into French and German and disseminated by professional bodies (ASTRM/SSNM/SSRO/SSR) and social media using snowball sampling. The intended audience were Swiss radiographers (clinical practitioners, educators, researchers, students) and physicians working in radiology (radiologists, nuclearists, radio-oncologists). The survey had five main sections: i) demographics, ii) AI-knowledge, iii) skills, iv) confidence in AI, and v) perceptions about the impact of AI on clinical practice. Descriptive and association statistics and qualitative thematic analysis were conducted.
**Results or Findings:** 243 responses were collected (89% radiographers; 11% physicians). AI is being used in clinical practice by 43% of participants. A 63.8% of them did not feel confident with AI-terminology (63.8%). Radiography participants see AI as an opportunity (57.2%), while 18.5% as a threat. The opportunities were associated with streamlining repetitive tasks, minimising human errors, and increasing time allocated to patient-centred care, research, image quality and patient safety. The most important threats identified by the participants were a) a reduction on work positions (22.6%) and b) a decrease on the radiographers' expertise level due to automation bias (16.4%). Participants (68.3%) did not feel well trained/prepared to implement AI in their practice, highlighting the non-availability of specific training (87.6%) in the topic. 93% of the participants mentioned that AI education should be included at bachelor.
**Conclusion:** Although most participants perceive AI as an opportunity, lack of relation knowledge, educational provisions, and confidence were identified. Specific training on this topic needs to be implemented to improve practice and understanding on AI.

**Limitations:** Four national languages and different educational levels of participants.
**Ethics committee approval:** Approved by ASTRM-SSR-SSNM-SSRO.
**Funding for this study:** Not applicable
**Author Disclosures:**
Ricardo Khine: Nothing to disclose
Claudia Sa dos Reis: Nothing to disclose
Isabelle Gremion: Nothing to disclose
Stephanie Elaine de Labouchere: Nothing to disclose
Switinder Singh Ghotra: Nothing to disclose
Laurent Marmy: Nothing to disclose
Sofia Torre: Nothing to disclose
Mélanie Champendal: Nothing to disclose
Christina Malamateniou: Nothing to disclose

### RPS 2414-4

**Development of a new breast phantom: image characteristics at the swing angle of digital breast tomosynthesis (DBT)**

*N. Kakihana*[1], A. Takada[1], H. Inagawa[1], Y. Nagai[1], N. Ikeda[2], N. Nakagawa[2], A. Takeuchi[2], Y. Muramatsu[1]; [1]Kashiwa/JP, [2]Kyoto/JP
*(nkakihan@east.ncc.go.jp)*

**Purpose:** Breast compression changes the position, thickness and shape of mammary glands and lesions. The aim of this study was to verify the image characteristics of digital breast tomosynthesis (DBT) against the swing angle using a compressible breast phantom which is under development.
**Methods or Backround:** A new breast phantom developed consisted of several polyurethane resins. The phantom is radially arranged with simulated mammary glands. Ten simulated masses (10 mm to 2 mm, 2 mm-step) of two different x-ray absorption types were placed. A breast x-ray system (AMULET Innovality, FUJIFILM) was used to take images in HR-mode (swing angle:+/-20 degrees) and ST-mode (swing angle :+/-7.5 degrees). The phantom was rotated every 30 degrees and the compression pressure was 20 N to 140 N. The imaging conditions were 30 kV, i-AEC, W/Al and N-mode. The visual evaluation method was 1 point if the circular shape of the simulated mass was clearly visible, 0.5 points if it was unclear, and the full score was 10 points. The receivers were four certified radiological technologists in mammography with an average experience of 10.8 years.
**Results or Findings:** HR scores showed a significant difference between 20 N and 50 N of compression pressure (P<0.007). However, no significant difference was found above 50 N (P=1.0). ST scores showed a significant difference between compression pressures 50 N and 80 N (P<0.005), but not above 80 N (P=1.0).
**Conclusion:** Using a breast phantom under development, the effect of compression on DBT at different swing angles was examined.
**Limitations:** The results are based on a unique breast x-ray system with a prototype phantom.
**Ethics committee approval:** No information provided by the submitter.
**Funding for this study:** This research is conducted in collaboration with Kyoto Kagaku.
**Author Disclosures:**
Norie Nakagawa: Employee: Kyoto Kagaku Co., Ltd.
Yoshihisa Muramatsu: Nothing to disclose
Nariko Kakihana: Nothing to disclose
Yuichi Nagai: Nothing to disclose
Atsuko Takada: Nothing to disclose
Hikari Inagawa: Nothing to disclose
Nobutaka Ikeda: Employee: Kyoto Kagaku Co., Ltd.
Aiko Takeuchi: Employee: Kyoto Kagaku Co., Ltd.

### RPS 2414-5

**A comparison of perceived image quality between computer display monitors and augmented reality smart glasses**

*A. England*[1], J. D. Thompson[2], S. Dorey[3], S. Al-Islam[4], M. Long[1], C. Miaorino[1], M. F. F. McEntee[1]; [1]Cork/IE, [2]Barrow-in-Furness/UK, [3]Manchester/UK, [4]Hillingdon/UK
*(aengland@ucc.ie)*

**Purpose:** Augmented-reality (AR) smart glass provide an alternative to standard computer display monitors (CDM) and there are increasing reports of their use within medicine. AR glasses may provide an opportunity to improve visualisation during fluoroscopy and interventional radiology (IR) procedures when there can be difficulty in viewing intra-procedural images on a CDM. The aim of this study was to evaluate radiographer perception of image quality (IQ) in fluoroscopy and IR images with CDM and AR smart glasses.
**Methods or Backround:** 38 radiographers attending the European Congress of Radiology 2022 evaluated ten fluoroscopic-guided surgery and IR images on both a 2.3 megapixel CDM and a set of Epson Moverio BT-50 AR smart glasses in an ambient lighting environment. Participants provided oral responses for pre-defined IQ criteria. Mean IQ scores for were compared for AR glasses and CDM.
**Results or Findings:** Of the 38 participants, the mean age was 39±10 years. 23 (60.5%) participants required corrective glasses. Participants were from 12 different countries, the majority (9, 23.7%) from the United Kingdom/Northern Ireland. For nine out of ten images, the AR glasses demonstrated a statistically significant increase in perceived IQ when compared to the CDM. Improvements in perceived IQ for the AR glasses were on average (SD) 2.2 (0.9) points higher than for the CDM.
**Conclusion:** AR glasses appear to show improvements in perceived IQ when compared to a CDM. AR glasses could provide an option for improving the experiences of radiographers involved in image-guided procedures and should be subject to further clinical evaluations.
**Limitations:** Observers evaluated only static fluoroscopy images, further studies are needed to evaluate dynamic/cine fluoroscopy images.
**Ethics committee approval:** University College Cork Social Research Ethics Committee.
**Funding for this study:** No funding was received for this study.
**Author Disclosures:**
Shofiq Al-Islam: Nothing to disclose
Carla Miaorino: Nothing to disclose
Mark F. F. McEntee: Nothing to disclose
Shaun Dorey: Nothing to disclose
Maria Long: Nothing to disclose
Andrew England: Board Member: EFRS
John David Thompson: Nothing to disclose

### RPS 2414-6

**Potential radiological 3D printable materials for plain radiography phantom construction**

*M. V. L. Oliveira*[1], M. Savi[2], M. Andrade[2], F. A. P. Soares[2], C. Ubeda[3]; [1]Salvador/BR, [2]Florianópolis/BR, [3]Arica/CL
*(marcusradiology@gmail.com)*

**Purpose:** The aim of this work was to investigate 3D printable materials, considering their radiological properties by measuring their intensity pixel and contrast values to assess their suitability for plain radiography phantom construction.
**Methods or Backround:** 16 plastic materials with six printed setups (varying infill densities and different infill structures) were tested in a D.R. x-ray unit. To perform the experimental measurements of this study, the plastic materials were designed and printed using the same geometry of a cube (2 cm x 2 cm x 2 cm). In addition, a PMMA slab with the same thickness of cubes was used. In total, nine protocols (tube potential 50 kV,60kV,70kV, and time-current product 2.5mAs, 5mAs, and 10mAs) were selected to obtain the images. The digital images were evaluated using image J software by means of the plot profile tool and mean pixel value measurement. The standard ROI size was applied to evaluate all the materials.
**Results or Findings:** The 3D cubes made in Copper, Bronze, Tungsten, and Bismuth have shown high attenuation values at 50kV and 60 kV tube potential and at 2.5,5 mAs time-current product protocol. The plastic materials were not shown at 70kV and 10mAs, but both Tungsten and Bismuth. All cubes were shown when time-current products 2.5 mAs and 5 mAs were selected without tube potential influence.
**Conclusion:** The Tungsten and Bismuth plastic filaments had total attenuation of x-ray in all protocols. Other materials have presented the potential for Phantom construction.
**Limitations:** The influence of thickness was not evaluated.
**Ethics committee approval:** No information provided by the submitter.
**Funding for this study:** No funding was received for this study.
**Author Disclosures:**
Marco Andrade: Nothing to disclose
Flavio Augusto Penna Soares: Nothing to disclose
Carlos Ubeda: Nothing to disclose
Matheus Savi: Nothing to disclose
Marcus Vinicius Linhares Oliveira: Nothing to disclose

### RPS 2414-7

**Combining deep learning reconstruction with automatic tube voltage selection for radiation dose optimisation in chest and abdomen CT**

*S. D. Moerup*[1], H. Precht[1], S. J. Foley[2]; [1]Odense/DK, [2]Dublin/IE

**Purpose:** To test the impact of combining automatic tube voltage selection (ATVS), with deep learning image reconstruction (DLIR) for optimising radiation dose and image quality during chest and abdominal CT scans.
**Methods or Backround:** This single-centre study prospectively enrolled seventy-two patients (33 women and 39 men, mean age, 69 years) referred for chest and abdomen CT. All patients were scanned on a GE Revolution Apex CT scanner. Acquisition parameters included rotation time 0.5sec, pitch 0.986, ATCM interval 80-1300mA, ATVS, size adjusted noise index. All scans were reconstructed with 0.625mm, iterative reconstruction ASIR-V 40% and DLIR (TrueFidelity (TF)) low, medium and high. Quantitative image quality was evaluated using image noise and HU measurements within circular ROI (20mm) in the aorta, liver parenchyma and muscle tissue. Contrast to noise

Sunday ratio (CNR) and signal to noise (SNR) were calculated. Radiation dose was assessed by recording the CT dose index (CTDI) and dose-length product (DLP).
**Results or Findings:** In total 288 image sets and 864 ROI's were evaluated. In 54% of the patients the ATVS reduced the kV from standard 120. In 27 patients the ATVS increased the kV from the chest scan to the abdomen. Image noise reduced progressively between ASIR-V and each level of TrueFidelity (low: -4% (p=0.29), medium: -23% (p=0.008), high: -38% (p<0.001). Both CNR and SNR were improved (185% & 76%) by using DLIR high. Mean CTDI was 22.7mGy (range: 10-42.0mGy) and DLP of 484.9mGy.cm (ranging from 248.5-792.4mGy.cm). A reduction in radiation dose of 52% was achived compared to the departments other scanners (mean:1152mGy).
**Conclusion:** Deep learning reconstruction and automatic tube voltage selection make it possible to both improve image quality and reduce radiation dose for chest and abdomen CT
**Limitations:** The subjective analyses was not performed yet.
**Ethics committee approval:** No information provided by the submitter.
**Funding for this study:** No funding was received for this study.
**Author Disclosures:**
Helle Precht: Nothing to disclose
Svea Deppe Moerup: Nothing to disclose
Shane J Foley: Nothing to disclose

### RPS 2414-8

**3D virtual reality simulation user experience of novice radiography students**

M. O'Connor, J. Stowe, *J. Potočnik*, S. Murphy, L. Rainford; Dublin/IE
*(jaka.potocnik@ucd.ie)*

**Purpose:** An immersive three-dimensional (3D) virtual reality simulation tool was integrated into a stage one undergraduate radiography curriculum and user feedback retrieved.
**Methods or Backround:** The 3D virtual simulation tool manufactured by Virtual Medical Coaching Ltd was introduced to first year radiography students (n=105) in their first trimester. This technology guides students through a comprehensive process of learning anatomy, radiographic positioning and pathology. Subsequently, students undertook a radiography examination on a virtual patient in the VR suite using HTC Vive Pro™ headsets and hand controllers. Instant feedback was provided in the form of a radiographic image and written report. At the end of the trimester, an online survey was disseminated to students to gather user feedback. Thematic and descriptive statistical analyses were applied.
**Results or Findings:** A response rate of 79% (n=83) was achieved. Most respondents (58%) reported enjoying VR simulation, whilst some felt indifferent towards it (27%). Ninety-four percent would recommend this tool to other students. The mean length of time it took for students to feel comfortable using the technology was 60 minutes (10-240 min). Over half of the cohort (58%) desired more VR practice. Students attributed enhanced confidence in the areas of beam collimation (75%), anatomical marker placement (63%), centring of the x-ray tube (64%) and exposure parameter selection (56%) to their VR practice. Students advocated the use of VR in formative or low stakes assessments, but not in high stakes assessments. Issues flagged included technical glitches, inability to palpate patient and lack of constructive feedback.
**Conclusion:** 3D virtual radiography simulation is perceived by radiography students to be a valuable pedagogical tool in radiography education.
**Limitations:** Students were limited to four hours using the VR simulation tool, which may have impacted evaluation of this tool.
**Ethics committee approval:** No information provided by the submitter.
**Funding for this study:** No funding was received for this study.
**Author Disclosures:**
Michelle O'Connor: Nothing to disclose
Louise Rainford: Nothing to disclose
Shauna Murphy: Nothing to disclose
Jaka Potočnik: Nothing to disclose
John Stowe: Nothing to disclose

### RPS 2414-9

**The value of postpositive dual flow CCTA in the diagnosis of coronary artery AI**

*Y. Huang*; Guangxi/CN
*(75446846@qq.com)*

**Purpose:** To investigate the application value of postpositive dual flow CCTA in the diagnosis of coronary artery AI.
**Methods or Backround:** Patients CCTA used 64-row CT from May 2019 to May 2021, were selected and randomly divided into two groups. In all cases DSA were performed within 7 days. Group A used test bolus injection CCTA; group B used postpositive dual flow CCTA. Upload the two groups of CCTA images to the PACS, and use the "medical accurate" coronary artery AI image diagnosis system to automatically extract images for analysis and diagnosis. The degree of coronary stenosis can be divided into mild stenosis (<50%), moderate stenosis (50%-70%) and severe stenosis (>70%). With DSA results as the gold standard, the main observation indicators are: sensitivity, specificity, accuracy of coronary stenosis detection, ventricular function analysis, and statistical analysis of differences in results.
**Results or Findings:** The sensitivity, specificity and accuracy of AI in group A were 91.55%, 96.67% and 92.86% respectively; group B was 93.25%, 97.74%, 93.67% (P>0.05), with no statistical significance. There was no significant difference between the control group and the study group. Heart function analysis: there was no significant difference between group A and group B in the assessment of left ventricular function, and the value of group A in the assessment of right ventricular function was significantly lower than that of group B.
**Conclusion:** In the diagnosis of coronary artery AI, the postpositive dual flow CCTA does not affect the analysis of the degree of coronary stenosis. It solves the problem of unclear boundary between right ventricle and ventricular septum, improves the accuracy of automatic extraction of cardiac structure and automatic evaluation of cardiac function by AI, and has high application value.
**Limitations:** Not applicable.
**Ethics committee approval:** No information provided by the submitter.
**Funding for this study:** Not applicable.
**Author Disclosures:**
Yan Huang: Nothing to disclose

### RPS 2414-10

**Clinical application of DR dual energy subtraction technology combined with spiral CT bone 3D imaging technology and AI diagnostic technology in traumatic rib fractures**

*Y. Huang*; Guangxi/CN
*(75446846@qq.com)*

**Purpose:** To investigate the clinical application value of digital x-ray imaging (DR) combined with dual energy subtraction (DES), spiral CT bone three-dimensional imaging technology and AI diagnostic technology in the diagnosis of traumatic rib fracture.
**Methods or Backround:** 200 cases of traumatic rib fracture in our hospital from January 2019 to January 2022 were selected as the study subjects for examination. They were divided into 50 cases (group A) examined by conventional DR technology, 50 cases examined by DR combined with DES (group B), 50 cases examined by DR combined with DES and 50 cases examined by spiral CT bone 3D imaging technology (group C), and 50 cases examined by DR combined with DES, spiral CT bone 3D, and AI diagnostic technology (group D). After the examination, the group of junior diagnostic doctors was recorded as the control group, and the group of senior diagnostic doctors was recorded as the research group for diagnosis. The main observation indicators were: detection rate, number of misdiagnosis and missed diagnosis cases, diagnostic accuracy, and statistical analysis results.
**Results or Findings:** The detection rate and diagnostic accuracy rate in the study group of group A were higher than those in the control group, and the number of misdiagnosis and missed diagnosis cases were less than those in the control group (P<0.05), with statistical significance. There was no significant difference in the accuracy between the study group and the control group in group B (P>0.05).
**Conclusion:** DR combined with DES technology, spiral CT bone 3D imaging technology and AI diagnostic technology have better image quality performance and powerful post-processing function in the diagnosis of traumatic rib fracture, which increases the accuracy and detection rate of image diagnosis.
**Limitations:** Not applicable.
**Ethics committee approval:** Not applicable.
**Funding for this study:** Not applicable.
**Author Disclosures:**
Yan Huang: Nothing to disclose

13:00-14:30 Research Stage 1

### Research Presentation Session: Artificial Intelligence & Machine Learning & Imaging Informatics

### RPS 2505

### New frontiers for AI in prostate MRI

**Moderator**
F. Giganti; London/UK
**Author Disclosures:**
Francesco Giganti: Consultant: Lucida Medical; Investigator: Recipient of the 2020 Young Investigator Award (20YOUN15) funded by the Prostate Cancer Foundation / CRIS Cancer Foundation

### RPS 2505-2

**Can AI for prostate MRI generalise to multiple centres and scanners?**
*A. Shah*[1], N. Vasdev[2], P. Burn[3], A. W. Rix[4], M. Hinton[4], N. Moreira Da Silva[4], E. Sala[4]; [1]Hampshire/UK, [2]Stevenage/UK, [3]Taunton/UK, [4]Cambridge/UK

**Purpose:** The purpose of this study was to evaluate how AI-based software to support the analysis of prebiopsy MRI for prostate cancer can be used to generalise to routinely collected, sequentially referred patient data from multiple sites, MRI scanners, field strengths and protocols.
**Methods or Backround:** The methods used were the following: AI-based software (Lucida Medical, Prostate Intelligence v2.1) was developed using retrospective data from 459 patients from the PAIR-1 and PROSTATEx studies. Data included one 1.5T and four 3T scanners, with varying protocols. Data was partitioned into training (n=274, prevalence=0.28), development-validation (n=91, prevalence=0.33) and hold-out test (n=94, prevalence=0.35) sets. The software computes scores/ROIs to identify GS≥3+4 csPCa per patient and per lesion. Patients scored PI-RADS3/4/5 had csPCa confirmed by biopsy. PI-RADS1/2 patients/lesions that did not receive biopsy are assumed negative.
**Results or Findings:** The AI patient score is intended to support decisions on whether to perform a biopsy. At per patient threshold 3.5, the AI identified patients with csPCa with a sensitivity of 90% (95% CI 78-100%), a specificity of 63% (49-75%), an NPV of 92% (84-100%), and an AUC of 0.86 (0.76-0.93) using mpMRI hold-out test data. In a meta-analysis of twelve major studies (prevalence=0.37), radiologists identified patients with csPCa with sensitivity of 86%, a specificity of 42% and an NPV of 84%. The AI lesion score is intended to assist selecting biopsy targets. At per lesion threshold 2.5, the AI identified csPCa lesions with a sensitivity of 93% (95% CI 85-100%), a specificity of 48% (38-58%), an NPV of 93% (86-100%), and an AUC of 0.82 (0.74-0.9) on the same data (128 lesions, per lesion prevalence 0.34).
**Conclusion:** AI can generalise to support prostate cancer detection in diverse real-world settings including 1.5T and 3T scanners, with sensitivity and specificity comparable to expert radiologists in major studies.
**Limitations:** This study used data from standard-of-care biopsies at four centres and five scanners representing one scanner vendor. Work is continuing to add centres and scanners, and provide further validation.
**Ethics committee approval:** NHS HRA IRAS #278640
**Funding for this study:** Funding for this study was provided by Lucida Medical.
**Author Disclosures:**
Aarti Shah: Nothing to disclose
Antony William Rix: Founder: Lucida Medical CEO: Lucida Medical
Paul Burn: Nothing to disclose
Mark Hinton: Employee: Lucida Medical
Evis Sala: Founder: Lucida Medical Consultant: Lucida Medical
Nikhil Vasdev: Nothing to disclose
Nadia Moreira Da Silva: Employee: Lucida Medical

### RPS 2505-3

**Thin-slice prostate MRI enabled by deep learning image reconstruction**
*S. Gassenmaier*[1], V. Warm[1], D. Nickel[2], E. Weiland[2], J. Herrmann[1], H. Almansour[1], S. Afat[1]; [1]Tübingen/DE, [2]Erlangen/DE

**Purpose:** The aim of this study was to investigate the impact of a thin-slice deep learning accelerated T2-weighted (w) TSE imaging sequence (T2DLR) of the prostate as compared to conventional T2w TSE imaging (T2S).
**Methods or Backround:** Thirty patients were included in this prospective study at one university centre after obtaining written informed consent. T2S (3 mm slice thickness) was acquired first in three orthogonal planes, followed by thin-slice T2DLR (2 mm slice thickness) in axial plane. The acquisition time of axial conventional T2S was 4:12 min compared to 4:37 min of T2DLR. Imaging datasets were evaluated by two radiologists using a Likert scale ranging from 1 - 4, with 4 being the best regarding the following parameters: sharpness, lesion detectability, artefacts, overall image quality, and diagnostic confidence.
**Results or Findings:** The mean patient age was 68 ± 8 years. The sharpness of images and lesion detectability were rated superior in T2DLR with a median of 4 versus a median of 3 in T2S (p<0.001 for both readers). Overall image quality was also evaluated to be superior in T2DLR versus T2S, with a median of 4 versus 3 (p<0.001 for both readers).
**Conclusion:** Thin-slice T2DLR of the prostate provides a significant improvement of image quality without significant prolongation of acquisition time.
**Limitations:** Limitations of this study are primarily related to its small sample size.
**Ethics committee approval:** Informed consent was obtained from all participants.
**Funding for this study:** No funding was received for this study.
**Author Disclosures:**
Judith Herrmann: Nothing to disclose
Elisabeth Weiland: Employee: Siemens Healthcare GmbH
Saif Afat: Nothing to disclose
Haidara Almansour: Nothing to disclose
Dominik Nickel: Employee: Siemens Healthcare GmbH
Verena Warm: Nothing to disclose
Sebastian Gassenmaier: Nothing to disclose

### RPS 2505-4

**Prostate gland segmentation on prostate magnetic resonance images: an AI study using a U-net-based convolutional neural network**
*B. Ünverdi*, M. A. Özdemir, A. Onan, T. Acar; Izmir/TR
*(basakunverdi@hotmail.com)*

**Purpose:** The purpose of this study was to automatically segment prostate whole gland, transitional zone (TZ) and periferal zone (PZ) on MR images by using a U-net-based convolutional neural network (CNN).
**Methods or Backround:** A total number of 100 MRs between January and December 2020 were initially enrolled. T2 weighted axial DICOM images were converted to .nifti format on the MedSeg.ai platform. Then whole gland and TZ masks were traced manually. The remaining areas were segmented automatically by subtracting TZ masks from whole gland masks and subsequently PZ masks were obtained. A U-net based CNN algorithm with 7 depths was used to process each image. Data from 80 patients was used in training the algorithm and among these, 10 were randomly selected to perform validation. The remaining 20 were used in the testing process. Evaluation metrics applied to the test set are accuracy, mean and median Dice Similarity Coefficient (DSC), mean Hausdorff Distance (HSD), Mean Surface Distance (MSD), mean Relative Absolute Volume (RAV).
**Results or Findings:** Mean DSC of 0.91±0,03, 0.87±0,06, 0.70±0.16 and median DSC of 0.92, 0.90, 0.75 were obtained for whole gland, TZ and PZ segmentation respectively. Mean HSD was 8.58, 9.52, 18.78, MSD was 0.92, 0.84, 1.30 and mean RAV was 3.51, 9.87, 70.57 for the segmentation of aforementioned structures.
**Conclusion:** Our algorithm demonstrated favourable results for whole gland and TZ segmentation and it was superior to most of the examples from previous studies. PZ segmentation success rate was lower but in acceptable range compared to state of the art methods in deep learning. We have proved our algorithm can aid radiologists in prostate gland segmentation.
**Limitations:** This was a retrospective, single-centre study, no sequence other than T2 was observed and no 3T MR images were used.
**Ethics committee approval:** This study was approved by Izmir Bozyaka Education and the Research Hospital Ethics Committee.
**Funding for this study:** not applicable
**Author Disclosures:**
Mehmet Akif Özdemir: Nothing to disclose
Türker Acar: Nothing to disclose
Başak Ünverdi: Nothing to disclose
Aytuğ Onan: Nothing to disclose

### RPS 2505-5

**Deep learning-based algorithm for prostate cancer detection on multi-vendor MRI scans, with a focus on how annotator variability affects algorithm performance**
N. Erhart[1], L. Alberge[2], R. Ardon[2], G. Bouzillé[1], A. David[3], R. Mathieu[1], *G. d'Assignies*[2], L. Beuzit[1]; [1]Rennes/FR, [2]Paris/FR, [3]Nantes/FR

**Purpose:** The aim of this study was to develop a deep learning algorithm to detect and segment cancer risk lesions on biparametric prostate MRI from different manufacturers and with different magnetic fields and evaluate the impact of annotator variability on algorithm performance.
**Methods or Backround:** A cohort (n=1802) was selected from a prospective database of patients undergoing multiparametric prostate MRI for clinical or biological suspicion of prostate cancer, extension assessment or active surveillance of cancer. MRI scans were randomly divided into training (n=1628) and test (n=174) datasets. PI-RADS v2.1 assessment was reported by two to

Sunday three experienced radiologists. A deep-learning (DL) algorithm was designed and trained to predict cancer-risk lesions from biparametric MRI. To incorporate prior knowledge into the detection model, a first zonal segmentation model on the T2W sequences was created. The performance of the DL algorithm was assessed using receiver operating characteristics (ROC) analysis and free-response ROC. We also evaluated lesion spatial congruence variability between DL framework and annotators. Finally, inter-operator variability was analysed in the location and segmentation of the lesions using spatial congruence analysis.
**Results or Findings:** The lesion detection algorithm achieved an AUC of 0.87 (95% CI: 0.79-0.91) for PI-RADS ≥ 4 lesions, an AUC of 0.87 (95% CI:0.79-0.90) for PI-RADS ≥ 3 lesions and an AUC of 0.88 (95% CI: 0.83-0.92) for only consensual PI-RADS ≥ 4 lesions. The spatial congruence analysis reported an absolute error of maximum axial measurement between our algorithm and two readers of respectively 2.8 mm and 3.7 mm versus 2.1 mm for the inter-annotator variability.
**Conclusion:** Our algorithm achieves similar performances to the ones in the literature, on data originating from different magnetic fields and different MRI manufacturers. Non-consensual annotations had a small impact at the patient level but were significant at the lesion level.
**Limitations:** This was a retrospective and mono-centric study.
**Ethics committee approval:** The study was approved by the Ethical Committee of Rennes University Hospital (10th of February, 2020).
**Funding for this study:** Not applicable
**Author Disclosures:**
Léo Alberge: Employee: Incepto-Medical
Nora Erhart: Nothing to disclose
Romain Mathieu: Nothing to disclose
Gaspard d'Assignies: Founder: Incepto-Medical
Guillaume Bouzillé: Nothing to disclose
Roberto Ardon: Employee: Incepto-Medical
Luc Beuzit: Nothing to disclose
Arthur David: Nothing to disclose

### RPS 2505-6

### Developing a machine learning radiomics-based model to predict clinically significant prostate cancer on multi-parametric MRI

C. Gaudiano, M. M. Mottola, L. Bianchi, B. Corcioni, *A. Cattabriga*, M. A. Cocozza, A. Palmeri, R. Golfieri, A. Bevilacqua; Bologna/IT

**Purpose:** Identifying clinically significant prostate cancer (csPCa) is key in selecting patients for radical treatments. The risk of misdiagnosing prostate cancer (PCa) through multi-parametric MRI (mpMRI) is relevant, also using the updated PIRADS score. csPCa has many definitions, however patients with Gleason Grade Group (GG)≥3 have a significantly worse prognosis. This study aims to develop a machine learning model predicting csPCa patients (i.e, GG≥3) by mpMRI radiomic features and to analyse similarities between GG=1, GG=2, and GG≥3.
**Methods or Backround:** 106 patients with 120 PIRADS≥3 lesions at mpMRI underwent target and systematic biopsy, providing histologic diagnosis of PCa, including 62 GG<3 and 58 GG≥3. Radiomic features were generated locally from apparent diffusion coefficient and selected, using the least absolute shrinkage and selection operator and Wilcoxon rank-sum test (p<0.001), to finally achieve four features. These were used to analyse similarities among GGs, underwent data augmentation, and exploited to train a support vector machine classifier through 100 runs of three-fold cross validation, subsequently validated on a test set.
**Results or Findings:** csPCa was defined as any GG≥3 lesion at target prostate biopsy. Kruskal-Wallis and Wilcoxon rank-sum tests (p<0.001) were used for multiple and pairwise comparisons among GGs. Receiver operating characteristic (ROC) related metrics were used to assess classifier performance. Results and Limitations were the following: GG=1 and GG=2 resulted equivalent (p=0.26), whilst clear separations between either GG1, or GG2, and GG≥3 exist (p<10-6 ). On the test set, area under the curve=0.88 (95% CI, 0.68-0.94), with positive and negative predictive values being 84%. The features retain a histological interpretation.
**Conclusion:** Our model, exploiting mpMRI features, predicts patients with GG≥3 with high accuracy.
**Limitations:** Validating the model based on biopsy was identified as a major limitation.
**Ethics committee approval:** Ethics Committee code: 784/2021/Oss/AOUBo)
**Funding for this study:** This work received no funding.
**Author Disclosures:**
Rita Golfieri: Nothing to disclose
Margherita Margherita Mottola: Nothing to disclose
Lorenzo Bianchi: Nothing to disclose
Arrigo Cattabriga: Nothing to disclose
Antonino Palmeri: Nothing to disclose
Beniamino Corcioni: Nothing to disclose
Maria Adriana Cocozza: Nothing to disclose
Caterina Gaudiano: Nothing to disclose
Alessandro Bevilacqua: Nothing to disclose

### RPS 2505-7

### Prediction of clinically significant prostate cancer using machine learning models

*Ö. Önder*, V. Gürler, A. Azizova, M. Ayva, B. Bakirarar, M. S. Yazici, B. Akdogan, M. N. Özmen, D. Akata; Ankara/TR
*(omeronder@hacettepe.edu.tr)*

**Purpose:** To estimate the probability of clinically-significant prostate cancer (cs-PCa) via machine learning (ML) algorithms using clinical and radiological data.
**Methods or Backround:** Clinical, biochemical, radiological, and histopathological data of adult patients who underwent multiparametric prostate MRI at our institution between 2014-2021 were collected retrospectively. Exclusion criteria: (1) Patients with known prostate cancer (PCa); (2) Patients without a definitive histopathological diagnosis; (3) Histologically-proven PCa patients whose time interval between index MRI and biopsy exceeds one year. A data set was created by including 826 cases. ISUP≥2 was considered as cs-PCa. The added value of each variable in cs-PCa prediction was calculated with two different gain methods. Seven independent variables that contributed significantly to the prediction (Index PI-RADSv2 score, MRI-defined PSA density [mPSAD], age, free/total PSA ratio, previous negative biopsy, digital rectal examination, coexistent malignancy) were used in the analysis. logistic regression, naive Bayes, multilayer perceptron, support vector machine, J48, and hybrid model were used as ML classification methods. The dataset was tested using 10-fold cross-validation. All analyses were performed using the R-programming language with RWeka and e1071 packages.
**Results or Findings:** Of 826 included patients, 249 (30.1%) had cs-PCa. The two variables with the most significant effect on cs-PCa prediction were mPSAD and PI-RADSv2 score. Considering performance metrics, the hybrid model gave the best results among all the ML models with 97% specificity, 91.1% PPV, 88.2% NPV, and 89.9% classification accuracy. Compared to the diagnostic performance of PI-RADS v2 ([PPV range: 33.2%-44.8%] and [NPV range: 92.8%-95.5%]), all models had slightly lower NPV but prominently higher PPV.
**Conclusion:** ML algorithms showed promising performance in predicting the probability of cs-PCa. By using these models, PPV of PI-RADS v2 can be significantly improved while substantially maintaining its already high NPV.
**Limitations:** The limitations were that the study was a single-centred one and it had no external validation.
**Ethics committee approval:** No information provided by the submitter.
**Funding for this study:** No funding was received for this study
**Author Disclosures:**
Bulent Akdogan: Nothing to disclose
Müjdat Ayva: Nothing to disclose
Deniz Akata: Nothing to disclose
Mustafa Nasuh Özmen: Nothing to disclose
Volkan Gürler: Nothing to disclose
Aynur Azizova: Nothing to disclose
Mustafa Sertac Yazici: Nothing to disclose
Ömer Önder: Nothing to disclose
Batuhan Bakirarar: Nothing to disclose

### RPS 2505-8

### Development and validation of an explainable AI-CAD system to predict high-aggressive prostate cancer: a multicentre radiomics study based on biparametric MRI

*K. Rocco*[1], M. Gatti[1], D. Regge[1], R. Cuocolo[2], G. Nicoletti[1], A. Stanzione[3], D. Tore[1], R. Faletti[1], V. Giannini[1]; [1]Turin/IT, [2]Baronissi/IT, [3]Naples/IT

**Purpose:** The aim of this study was to develop a model to distinguish between low-aggressive [Grade group grading system (GG) <2] and high-aggressive (GG >3) prostate cancer (PCa) through a radiomics signature based on prostate biparametric MRI (bpMRI), to validate the model on a multi-centre, multi-vendor dataset and to provide an explanation for the signature.
**Methods or Backround:** This was a retrospective multicentre study. A radiologist with experience in prostate MRI manually segmented the lesions on T2w images and ADC maps. The presence of at least one confirmed PCa at subsequent target biopsy was mandatory. The radiomic signature was developed using two centres' datasets using feature selection methods and classifiers: minimum redundancy maximum relevance, affinity propagation (AP), features ranking using chi-squared tests, Mann-Whitney U test and stepwise generalised logistic regressor, decision tree, support vector machine, ensemble learner, naïve Bayes (NB) and generalised logistic regression. Then, the best-performing model was selected and a cross-validation strategy was adopted. The selected model and cut-offs were applied to the validation set.
**Results or Findings:** 283 patients with 299 lesions were included (construction set 175 PCas, validation set 124). The best performing combination was AP-NB (balanced accuracy 71,4%, sensitivity 69,4%, specificity 73,4%, PPV 76,9%, NPV 65,6% on training set; and 71.1%, 69.4%, 72.8%, 78.8%, 66.9% on validation set). The subset of features included seven features from ADC maps and three features from T2w images, "ADC-GLRLM-

Sunday

Run Length Non-Uniformity" and "ADC-GLRLM-Run entropy" being the most important.
**Conclusion:** Our radiomic model can automatically assign an aggressiveness class to suspicious lesions distinguishing PCas with a good prognosis and those with higher aggressiveness. This model may have a role in clinical decision-making to choose between active surveillance and biopsy for patients with newly diagnosed prostate lesions.
**Limitations:** This was a retrospective study.
**Ethics committee approval:** No information provided by the submitter.
**Funding for this study:** This work received an EU grant through the Horizon2020 programme.
**Author Disclosures:**
Valentina Giannini: Nothing to disclose
Marco Gatti: Nothing to disclose
Daniele Regge: Nothing to disclose
Riccardo Faletti: Nothing to disclose
Katia Rocco: Nothing to disclose
Giulia Nicoletti: Nothing to disclose
Arnaldo Stanzione: Nothing to disclose
Davide Tore: Nothing to disclose
Renato Cuocolo: Nothing to disclose

### RPS 2505-9
**Prediction of Gleason grade discordance by using machine learning methods**
*I. Loc*[1], K. Özbozduman[1], S. Durmaz[1], D. Cengiz[2], M. Kılıc[1], T. Esen[1], B. Unlu[1], M. Vural[1]; [1]Istanbul/TR, [2]Zeytinburnu/TR
*(iremz382@gmail.com)*

**Purpose:** Clinicians rely on the prostate biopsy Gleason grade (GG) to counsel patients on treatment options. In this study, we aim to determine the clinical variables that are relevant for GG upgrade at final pathology using statistics and investigate the predictability of GG upgrade using machine learning for a personalised treatment planning.
**Methods or Backround:** In total, 95 patients were diagnosed with prostate cancer (PCa) at MRI-guided in-bore biopsy and subject to radical prostatectomy (RP) subsequently. At final pathology, 61 patients had concordant GG, whereas 27 upgraded and 7 downgraded. Biopsy GG 1 patients made up 62.9% of the upgrading group. Clinical factors that can be used in prediction of GG upgrade at final pathology were evaluated by statistical and machine learning methods.
**Results or Findings:** Statistically significant clinical variables for GG upgrade at final pathology include biopsy GG group, number of total and positive biopsy cores. Increasing number of biopsy cores, particularly 3 at minimum, and increasing number of positive cores improved GG concordance. Combining biopsy data with pre-biopsy clinical variables, machine learning studies yielded 85.6% accuracy (sensitivity: 0.621 ± 0.013, specificity: 0.953 ± 0.003), significantly surpassing the baseline of 64% accuracy obtained by the biopsy. In addition, machine learning studies indicated that minimum ADC can be beneficial regarding the GG upgrade prediction, for the patients GG>1.
**Conclusion:** Incorporation of MRI-guided in-bore biopsy results, tumour size, number of biopsy cores and minimum ADC value obtained from mp-MRI can be useful to predict GG upgrade at final pathology.
**Limitations:** The potential limitations of this study are retrospective design, small sample size that affects both statistical and ML studies, and the possible increase in selection bias over 8 years of time.
**Ethics committee approval:** No information provided by the submitter.
**Funding for this study:** This research didn't receive any funding.
**Author Disclosures:**
Tarik Esen: Nothing to disclose
Kaan Özbozduman: Nothing to disclose
Irem Loc: Nothing to disclose
Duygu Cengiz: Nothing to disclose
Metin Vural: Nothing to disclose
Selahattin Durmaz: Nothing to disclose
Mert Kılıc: Nothing to disclose
Burcin Unlu: Nothing to disclose

**13:00-14:30** **Research Stage 2**

### Research Presentation Session: Interventional Radiology

### RPS 2509
### Peripheral embolisation

**Moderator**
T. Rodt; Lüneburg/DE
*(Thomas.Rodt@klinikum-lueneburg.de)*

### RPS 2509-2
**Digital subtraction angiography versus digital variance angiography for optimisation of radiation dose during image guidance of Prostatic Artery Embolisation (PAE)**
*L. S. Alizadeh*[1], T. Vogl[1], I. Yel[1], L. D. Grünewald[1], J. Kiss[2], K. Szigeti[2], S. Osváth[2], V. I. Óriás[2], C. Booz[1]; [1]Frankfurt/DE, [2]Budapest/HU

**Purpose:** Digital Variance Angiography (DVA) provided a higher image quality compared to standard Digital Subtraction Angiography (DSA) in previous retrospective trials. This study aims to compare the performance of DVA versus standard DSA in Prostatic Artery Embolisation (PAE) for radiation dose optimisation.
**Methods or Backround:** 96 angiographic acquisitions of 16 patients (mean age 73.72, SD 10.02, range 49 87) undergoing PAE at our institution were evaluated. The signal-to-noise ratio (SNR) of DSA and DVA pairs was compared using regions of interest between standard image protocols and optimised low-dose protocols. Clinical image quality was tested by three experienced interventional radiologists in a randomised, blinded trial using a 5-point Likert scale consisting of clinically relevant criteria. Fleiss' kappa test was used to determine interrater agreement.
**Results or Findings:** Optimised low-dose protocols DVA images still provided a 1.38 times higher SNR than DSA (median value, Q1 Q3 interval was 1.32 2 13). The visual evaluation indicated that DVA videos provided a similar to higher image quality compared to conventional DSA images since, in 73.7% of comparisons, evaluators preferred DVA over DSA. The interrater agreement was 83.1%, and Fleiss's kappa was 0.52 (p<0.001).
**Conclusion:** In the PAE setting, the quality reserve of DVA provides the opportunity for a significant reduction of radiation dose as an attempt to solve one of the significant issues related to PAE procedures. DVA imaging enhances, optimises SNR, even in low-dose acquisitions, and could be feasible for future contrast agent reduction.
**Limitations:** The study results are initial results from a single-centre prospective trial on a limited cohort.
**Ethics committee approval:** The study was approved by the Universities ethics board.
**Funding for this study:** The study was supported by the European Commission EIC Accelerator Pilot grant (968430 KMIT-ACC), the National Research, Development and Innovation Office of Hungary (NKFIA; NVKP-16-1-2016-0017 National Heart Program, and 2020-1.1.5-GYORSÍTÓSÁV-2021-00018).
**Author Disclosures:**
Christian Booz: Advisory Board: Siemens
Ibrahim Yel: Nothing to disclose
Thomas Vogl: Nothing to disclose
Krisztián Szigeti: Employee: Kinepict
Szabolcs Osváth: Employee: Kinepict
János Kiss: Employee: Kinepict
Leona Soraja Alizadeh: Nothing to disclose
Leon David Grünewald: Nothing to disclose
Viktor Imre Óriás: Employee: Kinepict

### RPS 2509-3
**Uterine artery embolisation for symptomatic uterine leiomyomata: 12-Year clinical and morphologic follow-up using UFS-Qol questionnaire and pelvic MR**
*F. Andresciani*, E. Vergantino, G. Pacella, C. Bernetti, L. Cea, U. Ferrari, A. Buoso, B. Beomonte Zobel, R. F. Grasso; Rome/IT
*(flavioandresciani@gmail.com)*

**Purpose:** The purpose of this study was to assess clinical outcomes after uterine artery embolisation (UAE) in the treatment of symptomatic uterine leiomyomata.
**Methods or Backround:** The purpose of this study was to assess clinical ouThe 12-year post-intervention outcomes were evaluated by asking the patients who underwent UAE in our Centre to fill out the standardised Uterine Fibroid Symptom and Health-Related Quality of Life Questionnaire (UFS-QOL). Moreover, satisfaction, pregnancy and reinterventions rate were evaluated.

All patients underwent contrast-enhanced MRI on a 1.5-Tesla scanner before UAE. The MR was performed 6 and 12 months after the procedure; pre-and post-procedural uterine volume, longest diameter and localisation of the dominant fibroid as well as the number of lesions, were compared to after uterine artery embolisation (UAE) in the treatment of symptomatic uterine leiomyomata.
**Results or Findings:** A total of 89 women were treated with UAE between September 2010 and October 2022. The questionnaire was mailed in March 2022. The mean follow-up was 65 months at a mean age of 45. Questionnaires were completed by 80/89 patients (89%). The remaining nine patients were contacted by telephone. The HRQOL and SSS scores were evaluated by UFS-QOL six months after UAE, displaying significant improvement after the procedure. Eighty-one out of 89 (91%) patients affirmed to be at least fairly satisfied with UAE. A mean 37% of reduction in uterine volume was found 12 months after the procedure.
**Conclusion:** After 12 years of follow-up, 91% of UAE- treated patients with symptomatic uterine leiomyomata affirmed to be at least fairly satisfied with UAE, confirming the efficacy of this procedure.
**Limitations:** No limitations.
**Ethics committee approval:** The study was approved by the institutional review board with permission to perform chart review and a waiver of written informed consent.
**Funding for this study:** Not applicable.
**Author Disclosures:**
Rosario Francesco Grasso: Nothing to disclose
Bruno Beomonte Zobel: Nothing to disclose
Flavio Andresciani: Nothing to disclose
Andrea Buoso: Nothing to disclose
Elva Vergantino: Nothing to disclose
Ugo Ferrari: Nothing to disclose
Giuseppina Pacella: Nothing to disclose
Caterina Bernetti: Nothing to disclose
Laura Cea: Nothing to disclose

### RPS 2509-4

**Transradial versus transfemoral Uterine Artery Embolisation: a prospective, non-blinded randomised clinical trial**

*A. A. A. Bessar*; Zagazig/EG
*(ahmedawadbessar@gmail.com)*

**Purpose:** The study aimed to compare between transradial (TRA) and transfemoral (TFA) approaches for uterine artery embolisation (UAE) in patients with either abnormal uterine bleeding and/or pelvic pain from uterine fibroids.
**Methods or Backround:** In this prospective clinical trial, consecutive patients from January 1, 2019, to June 30, 2021, were randomised in either TRA or TFA groups. Clinical data such as age, parity and body mass index were recorded. The recorded operative details included bilaterality of the puncture site and uterine artery (UA) catheterisation, duration of the procedure, consumption of analgesics and embolising particles and postoperative complications.
**Results or Findings:** A total of 42 ladies were included in this study (twenty-one in each group). The mean age for ladies in the TFA group is 43.86 ± 4.10 years old and 44.86 ± 5.14 years old in the TRA group. The mean amounts of vials used in the TFA and TRA groups were 2.10 ± 0.77 and 1.52 ± 0.75, respectively. In all TRA patients, bilateral uterine artery access was performed; however, bilateral uterine artery catheterization was successful in only 85.7% of TFA patients. However, total radiation dose, fluoroscopic and total procedural duration were lower in TRA patients, but there were no significant statistical differences between both groups.
**Conclusion:** Both techniques are comparable for UAE, but in the TRA group, there is more success in performing bilateral UA catheterization with less radiation dose and procedural time.
**Limitations:** There are no limitations for this study.
**Ethics committee approval:** The study was approved by IRB in Zagazig University school of medicine.
**Funding for this study:** No specific funding was received.
**Author Disclosures:**
Ahmed Awad Abdelaziz Bessar: Nothing to disclose

### RPS 2509-5

**Clinical value of pelvic parametric X-ray angiography (ccDVA) for image guidance in prostatic artery embolisation**

*L. S. Alizadeh*[1], J. Kiss[1], T. Vogl[1], M. Gyánó[2], V. I. Óriás[2], V. Koch[1], C. Booz[1]; [1]Frankfurt/DE, [2]Budapest/HU

**Purpose:** Colour-coded parametric Digital Variance Angiography (ccDVA) images can be calculated from raw angiographic image data and could provide potentially useful information in Prostatic artery embolisation (PAE). Our objective was to investigate the clinical value of parametric images calculated from raw image data compared to conventional Digital Subtraction Angiography (DSA).
**Methods or Backround:** We retrospectively examined the image series of 47 patients (mean age 67.47, ±9.76, range 46-82) undergoing PAE. We generated 128 arterial, selective internal iliac, parametric, ccDVA from the raw data of the angiography images. Three experienced interventional radiologists evaluated image quality for both image data using a 5-point-Likert scale, based on clinically relevant criteria (identification of dominant feeding arteries, stenoses, collaterals to the rectum or pubic areas) to determine whether the ccDVA provided additional clinical information compared to standard DSA angiograms.
**Results or Findings:** Subjective image quality was evaluated with mean scores of 4.16 (range3-5;±0.73), significantly higher for DA compared to mean scores of 3.43 (range2-5;±1.80) for standard DSA. The evaluators agreed that 1.) CDAs provided more information than the simple BW angiograms (87%); 2.) the image would be useful to select the dominant feeding artery (93%); 3.) the functional stenoses of the branches could also be determined (84%). The inter-rater agreement, Fleiss' kappa and significance were 1.) 81%, 0.19, p<0.05 2.) 85%, 0.32, p<0.01 and 3.) 79%, 0.11, p<0.05, respectively.
**Conclusion:** We can conclude that parametric delay images calculated from X-ray angiography series provide more information than standard angiograms and hold additional information about the branches of the internal iliac artery, although decision-making solely based on CDAs will require appropriate prospective safety assessment.
**Limitations:** The study is a single centre retrospective trial.
**Ethics committee approval:** The study was approved by the University ethics committee
**Funding for this study:** European Commission EIC Accelerator Pilot grant (968430 KMIT-ACC),(NKFIA; NVKP-16-1-2016-0017 National Heart Program, and 2020-1.1.5-GYORSÍTÓSÁV-2021-00018).
**Author Disclosures:**
Christian Booz: Advisory Board: Siemens
Thomas Vogl: Nothing to disclose
Vitali Koch: Nothing to disclose
János Kiss: Employee: Kinepict
Leona Soraja Alizadeh: Nothing to disclose
Marcell Gyánó: Employee: Kinepict
Viktor Imre Óriás: Employee: Kinepict

### RPS 2509-6

**Uterine artery embolisation (UAE) in patients with adenomyosis alone or with fibroids: MR features and clinical effectiveness**

*V. Bérczi*, K. G. Turtóczki, H. Cho, S. Dastaran, P. N. Kaposi, S. Várbíró, Z. Tömösváry, N. Ács, I. Kalina; Budapest/HU
*(Berczi@hotmail.com)*

**Purpose:** The study aimed to identify adenomyotic patients with or without concomitant fibroids who had undergone uterine artery embolisation; to assess the clinical effectiveness and MR feature changes.
**Methods or Backround:** Reports of all patients from our UAE database (>750 patients) - who had pre- and postprocedural MR available - were reviewed. MR images were analysed for location, differentiation, uterine layer involvement, extent, lesion volume, uterus volume, type of adenomyosis, signal intensity ratio of adenomyosis/myometrium and adenomyosis/striated muscle, contrast medium enhancement, nonperfused volume. Clinical effectiveness was assessed by the numerical analogue quality-of-life score (0 – unbearable symptoms, 100 – perfect quality of life). Statistical analysis: paired Wilcoxon and Fisher tests.
**Results or Findings:** Out of the 577 patients with both pre-and post-MRI available, pure adenomyosis (PA) were identified in 12 (12/577=2.1%) patients, and the total number of fibroids in addition to adenomyosis (APF) was 58 (58/577=10.1%). The 12 patients with PA these results were compared with 12 patients APF patients. Clinical follow-up data show symptom improvement in 95.8% (23/24); the median numerical analogue quality-of-life score before and after embolisation was 10 (range 0-50) and 70 (range 0-100) for the PA group (p=0.003) and 10 (range 0-90) and 100 (range 80-100) for the APF group (P=0.012); improvement was greater in the APF than in the PA group (p=0.015). In 1 patient (4.2%; 1/24) symptoms have not improved. The volume of adenomyotic lesion were pre-UAE: median 59 cm3, range 11-334 cm3, post-UAE: median 20 cm3, 6-442 cm3, p=0.105 for the PA group; pre-UAE: median 21 cm3, range 2-131 cm3, post-UAE: median 8 cm3, range 2-119 cm3; p=0.008 for the APF group. The other MR parameters tested had no significant predictive value.
**Conclusion:** UAE elicits significant clinical improvement in symptomatic pure adenomyosis.
**Limitations:** A small number of patients were included in this study.
**Ethics committee approval:** No information provided by the submitter.
**Funding for this study:** No funding was received for this study.
**Author Disclosures:**
Hyunsoo Cho: Nothing to disclose
Kolos György Turtóczki: Nothing to disclose
Ildikó Kalina: Nothing to disclose
Zoltán Tömösváry: Nothing to disclose
Sorour Dastaran: Nothing to disclose

Viktor Bérczi: Nothing to disclose
Szabolcs Várbíró: Nothing to disclose
Nándor Ács: Nothing to disclose
Pál Novák Kaposi: Nothing to disclose

### RPS 2509-7

**Early versus delayed bronchial artery embolization for non-massive hemoptysis**

*H. Kim*[1], J. Pak[1], Y. Jang[1], H. N. Lee[1], S-J. Park[2], S. Lee[3], Y. C. Cho[4]; [1]Cheonan-si, Chungcheongnam-do/KR, [2]Ansan-si, Gyeonggi-province/KR, [3]Pohang-si, Gyeongsangbuk-do/KR, [4]Gangneung-si, Gangwon-do/KR

**Purpose:** The aims of this study were to compare clinical outcomes of early versus delayed bronchial artery embolization (BAE) for non-massive hemoptysis and to investigate predictors of recurrent hemoptysis.
**Methods or Backround:** From March 2018 to February 2021, 138 consecutive patients (age: 65.5 ± 12.4 years; male, 67.4%) with non-massive hemoptysis underwent BAE. The enrolled patients were divided into an early embolisation (EE) group (within the first 24 hours, n = 79) and a delayed embolisation (DE) group (n = 59).
**Results or Findings:** The time-to-embolisation ranged between 0 and 15 days and was shorter in the EE group (0.5 ± 0.5 days) than in the DE group (4 ± 2.8 days, p < 0.001). The in-hospital clinical outcomes were not different between the two groups, except for hospital stay (EE group, 10.7 ± 10.4 days; DE group, 12.6 ± 11.2 days; p = 0.017). The recurrence-free survival in the EE group was significantly better than that in the DE group (p = 0.018). The time-to-embolization (hazard ratio (HR), 1.21; 95% confidence interval (CI), 1.04–1.42; p = 0.015) and aspergilloma (HR, 6.89; 95% CI, 2.08– 22.86; p = 0.002) were predictive factors for recurrent hemoptysis.
**Conclusion:** BAE is an effective and safe treatment modality for non-massive hemoptysis. An early interventional strategy should be considered in patients presenting with non-massive hemoptysis to reduce the length of hospital stay and early recurrence. A delayed time-to-embolisation and the presence of aspergilloma were independent risk factors for recurrent hemoptysis.
**Limitations:** First, this is a retrospective study. In addition, the follow-up period and embolic materials used for each patient were variable.
**Ethics committee approval:** The institutional review boards of all collaborating institutions approved this retrospective study.
**Funding for this study:** No funding was received for this study.
**Author Disclosures:**
Sung-Joon Park: Nothing to disclose
Juyoung Pak: Nothing to disclose
Youngsil Jang: Nothing to disclose
Hyoung Nam Lee: Nothing to disclose
Sangjoon Lee: Nothing to disclose
Youngjong Cho Cho: Nothing to disclose
Hyeji Kim: Nothing to disclose

### RPS 2509-8

**Transarterial embolisation of active arterial bleeding in COVID-19 patients: a multicentre study**

*T. A. Auer*[1], R. Kloeckner[2], P. Minko[3], M. Wildgruber[4], D. Pinto dos Santos[5], J. B. Hinrichs[6], G. F. Torsello[7], L. Müller[2], B. M. Schaarschmidt[8]; [1]Berlin/DE, [2]Mainz/DE, [3]Homburg/DE, [4]Münster/DE, [5]Frankfurt/DE, [6]Hannover/DE, [7]Göttingen/DE, [8]Essen/DE

**Purpose:** In critically ill COVID-19 patients, necessary anticoagulation is challenging due to accompanying coagulopathy. Therefore, the purpose of this study was to assess transarterial embolisation in COVID-19 patients with active arterial bleeding.
**Methods or Backround:** We retrospectively reviewed COVID-19 patients undergoing transarterial embolisation due to active arterial bleeding in nine tertiary hospitals in Germany. The Chi-square test and Fisher's exact test were used for testing the association between the categorical variables, p< 0.05 was considered statistically significant.
**Results or Findings:** In total, 53 COVID-19 patients (female: n=16, age: 57.3± 14.3 years) received 66 angiographies due to active arterial bleeding. In 20.8% (11/53), a second embolization was necessary, 3.8% (2/53) of patients needed an additional third embolization due to active arterial bleeding. Surgery was performed in 7.5% (4/53) of patients due to active bleeding after initial embolization. 58.5% (31/53) of patients had a severe course of COVID-19 infection necessitating ECMO therapy, and 86.8% (46/53) of patients received anticoagulation. Bleeding was visible during the angiography in 54 cases, and transarterial embolization was performed. 30 days mortality rate in patients with ECMO therapy was significantly higher than without ECMO therapy (ECMO patients: 45.2%; non-ECMO patients: 13.6%, p=0.02). Patients with anticoagulation did not have a worse outcome than those without anticoagulation (anticoagulation: 34.9%; no anticoagulation: 14.3%, p=0.43).
**Conclusion:** Transarterial embolization is a feasible, safe and effective procedure in COVID-19 patients with active arterial bleeding. Patients with ECMO therapy have significantly higher mortality than those without ECMO therapy. Treatment with anticoagulation could not be identified as a risk factor for higher mortality.
**Limitations:** Due to its retrospective and multicenter character, selection bias and differences in interventional techniques and experiences cannot be excluded
**Ethics committee approval:** No information provided by the submitter.
**Funding for this study:** No funding was received for this study.
**Author Disclosures:**
Giovanni Federico Torsello: Nothing to disclose
Daniel Pinto dos Santos: Nothing to disclose
Roman Kloeckner: Nothing to disclose
Peter Minko: Nothing to disclose
Benedikt Michael Schaarschmidt: Nothing to disclose
Moritz Wildgruber: Nothing to disclose
Timo Alexander Auer: Nothing to disclose
Lukas Müller: Nothing to disclose
Jan B Hinrichs: Nothing to disclose

### RPS 2509-9

**Role of multi-detector computed tomography angiography for pre-procedural planning and radiation dose reduction in bronchial artery embolization**

*B. S. Reddy*, P. Chatterjee, K. Sunder, R. Radhakrishnan, V. Mazumdar; Chennai, Tamil Nadu/IN
*(sudeepreddy2009@gmail.com)*

**Purpose:** The study aimed to evaluate prospectively bronchial and Non-Bronchial Systemic Arteries (NBSA) with MDCT Angiography (MDCTA) prior to bronchial artery embolization (BAE) in patients with hemoptysis and the amount of radiation dose reduction when BAE is performed with the knowledge of MDCTA.
**Methods or Backround:** This was a prospective study done for four years, from October 2018 to September 2022. MDCTA was performed in 80 patients (70 men, 10 women) with hemoptysis who were referred for BAE. Transverse, multiplanar and 3-Dimensional reconstruction images were analyzed to identify abnormal hypertrophied bronchial arteries and NBSA causing hemoptysis. Digital subtraction angiography (DSA) was performed with the knowledge of the findings of MDCTA. Selective arteriograms of abnormal bronchial arteries and NBSA were performed. DSA findings were compared with MDCTA. Embolization was done in all patients using Polyvinyl alcohol particles (300-500 µm), Gelfoam or coils. The total cumulative dose for a single BAE intervention ranges from 0.2–2.7 Gy as the standard range, and we compared this with our radiation dose parameters.
**Results or Findings:** MDCTA shows 96.2% sensitivity and 100% specificity in identifying abnormal bronchial arteries and 64.7% sensitivity and 100% specificity in identifying abnormal NBSA. There was 40.2% reduction in cumulative DAP, 30.7% reduction in cumulative air kerma and 77.1% reduction in contrast dose when BAE was performed with knowledge of MDCTA.
**Conclusion:** MDCTA allows rapid and detailed identification of abnormal bronchial arteries and NBSA using a variety of reformatted images and provides a precise road map which can be used to guide therapeutic arterial embolization procedures. This information may be helpful for Interventional radiologists in order to avoid thoracic aortography and attempt direct catheterization of arteries for occlusion, resulting in reducing examination time, contrast load and radiation dose.
**Limitations:** Our study was a single-centred study without external validation.
**Ethics committee approval:** No information provided by the submitter.
**Funding for this study:** Not applicable.
**Author Disclosures:**
Bhanu Sudeep Reddy: Nothing to disclose
Vishakha Mazumdar: Nothing to disclose
Pritam Chatterjee: Nothing to disclose
K Sunder: Nothing to disclose
Ravikumar Radhakrishnan: Nothing to disclose

**13:00-14:30** **Research Stage 3**

### Research Presentation Session: Neuro

### RPS 2511

### Imaging of primary brain neoplasms

**Moderator**
Z. Rumboldt; Rovinj-Rovigno/HR

### RPS 2511-2

**Pre-operative MRI in the prediction of posterior fossa ependymoma subtypes**

*G. Librizzi*[1], M. Rossetto[2], M. Mansour[3], G. Veronesi[1], F. Morra[2], V. Vitale[3], E. Cagliari[2], M. P. Gardiman[1], R. Manara[1]; [1]Padua/IT, [2]Venice/IT, [3]Vicenza/IT
*(gia.librizzi@gmail.com)*

**Purpose:** According to the most recent WHO Classification of Tumors of the Central Nervous System, histone H3 lysine 27 methylation status differentiates posterior fossa ependymomas into two subtypes (PFA and PFB, respectively) with different prognoses. This retrospective study investigates the possible role of pre-operative MRI in predicting posterior fossa ependymoma subtypes.
**Methods or Backround:** Patients with posterior fossa ependymomas treated between July 2009 and November 2021 were included. Pre-operative brain MRI exams were assessed blinded to the final diagnosis. A score was created to assign tumours either to PFA or PFB subtypes. The score comprised six variables (points): age (<10 years +2; 10-20 years 0; >20 years -2), brainstem displacement (lateral +1; anterior -1), cisternal extension (prepontine +2; cerebellopontine angle +1; no involvement -1), obex infiltration (absent +1; present-1), vessel encasement (present +1; absent -1), bilateral extension into the lateral recesses of the fourth ventricle (absent +1; present -1). Sensitivity, specificity and accuracy were measured. Student's t-test was performed for scores. P-value <0.05 was defined as statistically significant.
**Results or Findings:** There were 12 PFA and 7 PFB tumours. Mean scores were 3.3±3.8 for PFA and -4.2±1.9 for PFB, and the difference was significant (p=0.0002). Using -1 as the cut-off (PFA if ≥-1, PFB <-1), the score showed a sensitivity of 92.7%, a specificity of 85.7% and an accuracy of 89.5%.
**Conclusion:** Pre-operative MRI scores showed good sensitivity, accuracy and specificity in predicting posterior fossa ependymoma subtypes. Further studies are needed to validate our findings.
**Limitations:** The study included a small sample size, and had a retrospective design.
**Ethics committee approval:** No information provided by the submitter.
**Funding for this study:** Not applicable.
**Author Disclosures:**
Francesco Morra: Nothing to disclose
Valerio Vitale: Nothing to disclose
Giulia Veronesi: Nothing to disclose
Marta Rossetto: Nothing to disclose
Mariam Mansour: Nothing to disclose
Marina Paola Gardiman: Nothing to disclose
Renzo Manara: Nothing to disclose
Enrico Cagliari: Nothing to disclose
Giovanni Librizzi: Nothing to disclose

### RPS 2511-3

**Nomogram incorporating preoperative MRI-VASARI features for differentiating intracranial extraventricular ependymoma from glioblastoma multiforme**

*Y. Fu*[1], X. Wang[1], Y. Yao[2], M. Jiang[2], X. Yi[1], B. T. Chen[3]; [1]Changsha/CN, [2]Nanning/CN, [3]Duarte, CA/US
*(xiangyafy@csu.edu.cn)*

**Purpose:** The study aimed to develop and validate a nomogram based on MRI visually accessible rembrandt images (VASARI) features for preoperatively differentiating intracranial extraventricular ependymoma (IEE) from glioblastoma multiforme (GBM).
**Methods or Backround:** The clinical data and MRI-VASARI features of 114 patients with histopathologically confirmed IEE and 258 patients with histopathologically confirmed GBM in a multicenter cohort were retrospectively analyzed. Predictive models for differentiating IEE from GBM were built using a multivariate logistic regression method, and a nomogram was generated. The performance of the nomogram was assessed with respect to its calibration, discrimination, and clinical usefulness.
**Results or Findings:** The predictors identified in this study consisted of six VASARI features and four clinical features. Compared with the individual models, the combined model incorporating VASARI features has the highest area under curve (AUC) value (training set: 0.99 (CI: 0.98-1.00), validation set: 0.97 (CI: 0.94-1.00)) than that of the clinical model. The nomogram was well-calibrated and had significant clinical benefits according to the calibration curve and decision curve analysis.
**Conclusion:** The nomogram combining clinical and VASARI characteristics performed well for differentiating IEE from GBM preoperatively, and may help in the diagnosis and treatment of brain tumours.
**Limitations:** Our study was the retrospective data collection in a multi-centre study, and our sample size was still modest.
**Ethics committee approval:** The present study was approved by the Ethics Committee of the First Affiliated Hospital of Guangxi Medical University (IRB#:2022-KY-E-(236)
**Funding for this study:** Funding was received from the Natural Science Foundation of Hunan Province (2022JJ30979)
**Author Disclosures:**
Yan Fu: Nothing to disclose
Xueying Wang: Nothing to disclose
Yao Yao: Nothing to disclose
Bihong T Chen: Nothing to disclose
Muliang Jiang: Nothing to disclose
Xiaoping Yi: Nothing to disclose

### RPS 2511-4

**Semi-automated texture analysis to differentiate glioblastoma and central nervous system lymphoma**

*Y. Özbay*, A. Y. Altunbulak, H. I. Altunbulak, G. Çoban Çifçi; Ankara/TR
*(docofrad@gmail.com)*

**Purpose:** The study aimed to evaluate the diagnostic performance of semi-automated texture analysis, first-order, and higher-order texture parameters, based on fluid-attenuated inversion recovery (FLAIR) imaging, in differentiating glioblastoma (grade 4 IDH-wildtype, WHO 2021) and central nervous system lymphoma.
**Methods or Backround:** This IRB-approved retrospective study was conducted on 48 patients (mean age 48.76 ± 12.63 years) with glioblastoma, and 32 patients (mean age 51.78 ± 17.61 years) with central nervous system lymphoma. Texture analysis was performed using a semiautomated region-growing segmentation technique (syngo. via MM Radiomics Frontier, v1.2.6). Two raters segmented the tumours independently, on FLAIR images, and 1224 radiomic features were generated per patient.
**Results or Findings:** Following least absolute shrinkage and selection operator (LASSO) regularization and logistic regression analysis, 16 texture features of the first-order histogram analysis, grey-level co-occurrence matrix, grey-level run-length matrix, neighbouring grey-level dependence matrix features remained as significant contributors with the area under the curve 0.74–0.92, to differentiate the glioblastoma and central nervous system lymphoma.
**Conclusion:** Semi-automated texture analysis of FLAIR imaging can differentiate glioblastoma from central nervous system lymphoma and could be a promising tool for non-invasive distinction.
**Limitations:** Licensed semiautomated region-growing segmentation technique (syngo. via MM RAdiomics Frontier, v1.2.6) enables only one sequence segmentation without an overlay option, due to this limitation, we can only obtain the FLAIR sequence features.
**Ethics committee approval:** The study was approved by the Institutional Review Board.
**Funding for this study:** No funding was received for this study.
**Author Disclosures:**
Ahmet Yasir Altunbulak: Nothing to disclose
Gökçen Çoban Çifçi: Nothing to disclose
Halil Ibrahim Altunbulak: Nothing to disclose
Yakup Özbay: Nothing to disclose

### RPS 2511-5

**The application value of contrast-enhanced T2 FLAIR in the differential diagnosis of solid CNS lymphoma and high-grade adult diffuse glioma**

*H. Gu*[1], Z. Li[1], J. Yang[2], W. Xie[1], H. Bao[1], Q. Zhao[3], Q. Wang[1]; [1]Kunming/CN, [2]Baoshan/CN, [3]Qujing/CN

**Purpose:** The study aimed to investigate the value of contrast-enhanced T2 FLAIR (CE-T2 FLAIR) in the differential diagnosis of solid central nervous system lymphoma (CNSL) and high-grade adult diffuse glioma (HG-ADG).
**Methods or Backround:** A retrospective analysis of 53 lesions of solid CNSL and 14 lesions of solid HG-ADG confirmed by pathology/follow-up was performed. All patients underwent preoperative cranial MRI, including contrast-enhanced T1WI (CE-T1WI) and CE-T2 FLAIR. The characteristics of tumour enhancement, including the uniformity and degree of enhancement of the tumour parenchyma and the degree and extent of enhancement at the edge of the tumour parenchyma, were evaluated by visual score on CE-T1WI and CE-FLAIR, respectively. Then the visual scores of the enhancement features of the tumours in the CNSL and HG-ADG groups at CE-T1WI and CE-T2FLAR were compared separately. P<0.05 was considered statistically significant.
**Results or Findings:** On CE-T2 FLAIR, there was a statistical difference between the two groups in the degree of tumour parenchymal enhancement,

Sunday and the degree of enhancement of CNSL was lower than that of HG-ADG. Moreover, there was a statistical difference between the two groups in the degree and extent of tumour parenchymal marginal enhancement, and the degree and extent of CNSL were higher than that of HG-ADG. But there was no statistical difference between the two groups in the uniformity of tumour parenchymal enhancement. On CE-T1WI, all of the above enhancement features were not statistically different between the two groups.
**Conclusion:** The probability of obvious thin-layer enhancement at the edge of the tumour parenchyma of CNSL on CE-T2 FLAIR is high. This sign has great value in the differential diagnosis of solid CNSL and HG-ADG. This has important implications for guiding clinical treatment decisions.
**Limitations:** Not applicable.
**Ethics committee approval:** This study was approved by Special Fund of Yunnan Clinical Medical Research Center (No. 202102AA100067)
**Funding for this study:** Funding was received from Special Fund of Yunnan Clinical Medical Research Center (202102AA100067)
**Author Disclosures:**
Heyi Gu: Nothing to disclose
Jingyun Yang: Nothing to disclose
Wei Xie: Nothing to disclose
Zongfang Li: Nothing to disclose
Han Bao: Nothing to disclose
Qirui Zhao: Nothing to disclose
Qin Wang: Nothing to disclose

### RPS 2511-6
**Grade-4 IDH-wildtype and IDH-mutant astrocytomas: differentiation using semi-automated texture analysis**
*A. Y. Altunbulak*, Y. Özbay, H. I. Altunbulak, G. Çoban Çifçi; Ankara/TR
*(ahmet.altunbulak@gmail.com)*

**Purpose:** The study aimed to investigate the value of semi-automated texture analysis in the differentiation of grade-4 IDH-wildtype astrocytomas (glioblastoma) and grade-4 IDH-mutant astrocytomas (according to WHO-2021 criteria).
**Methods or Backround:** This IRB-approved retrospective analysis was performed on 18 IDH-mutant (patients mean age 35.0 ±11,74 years) and 52 IDH-wild types (patients mean age 47.68 ± 14.76 years) grade-4 gliomas. Texture analysis was carried out with postprocessing tools (syngo. via MM Radiomics Frontier, v1.2.6). Using a semiautomated region-growing segmentation method, two raters separately segmented the tumours. Totally 1224 radiomic features were produced from fluid-attenuated inversion recovery (FLAIR) imaging per one patient.
**Results or Findings:** Our preliminary texture analysis of the 70 patients with grade-4 gliomas demonstrated that the first-order histogram analysis, grey-level co-occurrence matrix, grey-level run-length matrix, grey-level size-zone matrix, neighbouring grey-level dependence matrix features based on the FLAIR images is promising to distinguish glioblastoma and grade-4 IDH-mutant astrocytomas (AUC: 0.76-0.94; accuracy > 83%).
**Conclusion:** Texture analysis can be used to differentiate glioblastoma from grade-4 IDH-mutant astrocytoma and could contribute to optimal patient management and surgical planning.
**Limitations:** There is no limitation for this study.
**Ethics committee approval:** The study was approved by the Institutional Review Board.
**Funding for this study:** No funding was received for this study.
**Author Disclosures:**
Ahmet Yasir Altunbulak: Nothing to disclose
Gökçen Çoban Çifçi: Nothing to disclose
Halil Ibrahim Altunbulak: Nothing to disclose
Yakup Özbay: Nothing to disclose

### RPS 2511-7
**Development of circulating TAMs as candidate biomarkers in glioma and new diagnostic tool to complement MRI in the management of gliomas**
*C. Giordano*[1], G. Guerri[1], M. F. Romano[2], L. Natale[1], E. Sala[1], S. Gaudino[1]; [1]Rome/IT, [2]Naples/IT
*(carolinagiordano91@gmail.com)*

**Purpose:** Glioblastoma (GB) is infiltrated by TAMs (tumour-associated macrophages). FKBP51s is emerging as a new TAM marker. We investigate the potential utility of combining conventional MRI and circulating TAM analysis using FKBP51s for the management of GB patients.
**Methods or Backround:** 49 GB patients. MRI conventional sequences were evaluated before and after contrast medium (TSE, T1, T1 post-gad and T2 sequences, FLAIR and SWI sequences). In the pre-op MR images, qualitative (tumour localization, gross intratumoral haemorrhage, ependymal impregnation, infiltration of the corpus callosum and midline shift) and quantitative analysis (measurement of tumour volumes, necrosis score, score intratumor susceptibility signal intensity (ITSS) and oedema measurement). In post-op MR images, the presence of a residual tumour was assessed, and where it was present, its volume was calculated. TAM analysis was conducted on peripheral blood mononuclear cells stained with CD14/CD163/PDL-1/FKBP51s and flow cytometry.
**Results or Findings:** A significant expansion of CD163 monocytes was measured. The PDL1/FKBP51s phenotype of circulating monocytes correlated with the MR tumour necrosis score. The CD163/FKBP51s phenotype was associated with the postoperative MRI diagnosis of the presence of a residual tumour. Moreover, a positive correlation was calculated between the proportion of CD163/FKBP51s monocytes and corpus callosum infiltration.
**Conclusion:** The association of MRI morphological data with specific TAM subtypes suggests a potential utility of combining MR with FKBP51s-based immunophenotyping of peripheral blood, to monitor GB patients.
**Limitations:** Ten patients of the study population were not at their first diagnoses but were relapsed patients who had already undergone treatments.
**Ethics committee approval:** Prot N. 0011966/21 del 31/03/2021
**Funding for this study:** No funding was received for this study.
**Author Disclosures:**
Carolina Giordano: Nothing to disclose
Giulia Guerri: Nothing to disclose
Simona Gaudino: Nothing to disclose
Maria Fiammetta Romano: Nothing to disclose
Luigi Natale: Nothing to disclose
Evis Sala: Nothing to disclose

### RPS 2511-8
**Prediction of survival in low-grade adult diffuse glioma patients using machine learning based on MRI radiomics features**
*V. Opancina*[1], M. Muto[2], R. Maksimović[3], S. Jankovic[1], G. Kvascev[3], N. Zdravkovic[1]; [1]Kragujevac/RS, [2]Naples/IT, [3]Belgrade/RS

**Purpose:** The aim was to investigate the feasibility of predicting the overall survival of patients with low-grade adult-diffuse gliomas using machine learning to offer an alternative method to non-invasively predict the prognoses of these patients.
**Methods or Backround:** The study was designed as a multicentric, retrospective cross-sectional study, performed in our institutions. The inclusion criteria were: patients 18 years or older; low-grade glioma seen in MR image between 2012-2022; surgical resection or biopsy; certain pathological diagnosis; MR imaging: T2wi, T2/FLAIR, T1c and DWI before the treatment. The regions of interest (ROIs) were manually segmented on the axial slice with the largest cross-section of the tumours by two authors blinded to histological results. Data were randomly divided into training and test cohorts. The variance method was used to calculate the variance of extracted imaging features. Logistic regression was used to establish the model. Receiver operating characteristic (ROC) curve analysis and decision curve analysis (DCA) were used to assess the performance of the models. ROC curve analysis was used to assess the established models according to the area under the curve (AUC), sensitivity, and specificity values and DCA to evaluate the application value of the models.
**Results or Findings:** A total of 1,620 features were extracted from T2w, T2/FLAIR, and T1c images. In the test cohort, the combined model performed the best out of all models. The AUCs of the T2w, T2/FLAIR, T1c, and combined models were 0.75, 0.79 and 0.88, respectively, and accuracies were 0.71, 0.74 and 0.82, respectively. The ROC curves and DCA showed that the combined model had the highest efficiency and most favourable clinical benefits.
**Conclusion:** The combined radiomics model based on multi-parameter MRI features provided a reliable non-invasive method for the prognostic prediction of diffuse low-grade gliomas.
**Limitations:** The study included a small sample.
**Ethics committee approval:** The study was approved by the Ethics Comittee of University of Belgrade, University of Kragujevac, AORN Antonio Cardarelli.
**Funding for this study:** No funding was received for this study.
**Author Disclosures:**
Nebojsa Zdravkovic: Nothing to disclose
Valentina Opancina: Nothing to disclose
Goran Kvascev: Nothing to disclose
Ružica Maksimović: Nothing to disclose
Mario Muto: Nothing to disclose
Slobodan Jankovic: Nothing to disclose

### RPS 2511-9
**IDHwt glioblastoma WHO Grade 4 tumours progress through a spectrum of imaging patterns as they develop**
*M-T. Lee*, K. Fatania, R. Gilliver, J. Start, R. Frood, S. Currie; Leeds/UK
*(mingtelee1@gmail.com)*

**Purpose:** The study aimed to examine imaging features common to the developing GBM. Hypothesis: Growing GBM undergo a spectrum of imaging changes.
**Methods or Backround:** Retrospective longitudinal observational study. Patients with pre-operative imaging diagnosed with IDHwt GBM were identified from a tertiary neuroscience centre database covering 01/01/2014–31/03/2022.

All imaging was reviewed by an experienced neuroradiologist. Patients with imaging that preceded the typical radiological appearance of GBM (TRA GBM) of a centrally necrotic, peripherally enhancing tumour with surrounding oedema were ascertained. Imaging patterns prior to TRA GBM were identified.
**Results or Findings:** 76/569 (13%) patients had imaging that preceded TRA GBM; 57 solitaries, 19 multiple lesions giving 84 lesions in total. At the time of tissue sampling, 20 lesions never had imaging showing TRA GBM; these were classed as 'non-TRA GBM'. At the time of initial imaging, 63% presented with seizure, and 89% had a cortical or cortical/subcortical lesion. Earliest imaging feature for the 84 lesions: T2 hyperintensity (n=17), CT hyperdensity (n=50), and T2 iso-intensity (n=17). When CT and MRI were available (n=40), all CT hyperdense lesions showed T2 iso-intensity, reduced diffusivity, and the following enhancement patterns: none=26%, nodular n=35%, solid 29% and patchy peripheral n=10%. Mean time to develop TRA GBM from T2 hyperintensity=139 days & from CT hyperdensity=69 days. All 20 non-TRA GBM showed CT hyperdensity (n=13) or T2 iso-intensity (n=7). 3/20 had tissue diagnoses when the only imaging obtained was CT which showed a hyperdense lesion.
**Conclusion:** GBM show a spectrum of imaging features as they develop, starting with T2 hyperintensity, progressing to CT hyperdensity, T2 iso-intensity, reduced diffusivity, and variable enhancement to TRA GBM. Red flags for non-TRA GBM include a cortical/subcortical hyperdense/T2 iso/low ADC lesion in a patient presenting with a seizure.
**Limitations:** This was a retrospective study that had one observer.
**Ethics committee approval:** Ethical approval received as follows: (IRAS ref: 277122, Enhancing understanding and prediction of cancer outcomes with baseline characteristics from routinely collected).
**Funding for this study:** Not applicable.
**Author Disclosures:**
Joanna Start: Nothing to disclose
Ruth Gilliver: Nothing to disclose
Ming-Te Lee: Nothing to disclose
Kavi Fatania: Nothing to disclose
Stuart Currie: Nothing to disclose
Russell Frood: Nothing to disclose

### RPS 2511-10

**Advanced diffusion imaging of gliomas**
*V. Sedlák*; Prague/CZ

**Purpose:** Advanced diffusion imaging using multiple b-values allows for the modelling of various tissue parameters, which might correlate with tumour composition, its microarchitecture and possibly its molecular makeup. Multi-shell diffusion imaging might be a useful adjunct to conventional MR protocol and provide new information when evaluating low and high-grade gliomas, including differentiation of true progression from pseudo-progression or IDH mutant from IDH wild-type gliomas.
**Methods or Backround:** In addition to standard neurooncology brain protocol, a multi-shell diffusion sequence including b-values from 90 to up to 3000 was performed, allowing for the reconstruction of multiple diffusion models, including ADC, DTI, DKI, NODDI or IVIM. Tumoral compartment masks (enhancing and non-enhancing tumour, necrosis, oedema) were extracted via semiautomated segmentation. In the next step, histograms of individual diffusion indices were extracted for each tumoral compartment. These were compared to histopathological and molecular features of the tumour.
**Results or Findings:** 50 patients have been included in the study so far. Preliminary analysis of data shows a significant correlation of used diffusion indices when 1) differentiating true progression from pseudo-progression, 2) differentiating low-grade gliomas from high-grade gliomas, 3) differentiating high-grade tumours from its mimics, including lymphoma, tumefactive demyelination. The final analysis of acquired data is still underway at the time of writing this abstract.
**Conclusion:** Advanced diffusion imaging with the utilisation of multiple b-values provides diagnostically useful information when added to standard contrast-enhanced brain MR protocol.
**Limitations:** Multi-shell diffusion acquisitions are usually lengthy, extending the imaging protocol. In certain cases, however, the acquisition of data for advanced diffusion models can be done in less than two minutes (e.g. certain kurtosis indices). A thorough reconstruction of diffusion data is currently also computationally demanding.
**Ethics committee approval:** No information provided by the submitter.
**Funding for this study:** Funded by the institution where the study was performed - Military University Hospital in Prague
**Author Disclosures:**
Vojtěch Sedlák: Nothing to disclose

### RPS 2511-11

**Scientific research and clinical application of the recently developed "Firefly imaging": a preoperative assessment of brain tumour blood vessels and intratumoral microbleeds**
*X. Cao*, Z. Chen, L. Zhang, J. Zhang; Shanghai/CN

**Purpose:** The study aimed to compare 3D contrast-enhanced T1WI flow-sensitive black-blood (CE-T1WI FSBB) and 3D contrast-enhanced T1WI (CE-T1WI) sequences in assessing blood vessels and microbleeds in preoperative brain tumours and investigate the correlation between visible vessels and microbleeds.
**Methods or Backround:** Patients with brain tumours underwent scans with a 3.0-T MRI system using a 32-element phased-array head coil. An intravenous injection of 10.0 ml gadolinium glutamate was used for post-contrast imaging. Three neuroradiologists counted the internal blood vessels of the brain tumours separately based on CE-T1WI FSBB and CE-T1WI, and they counted microbleeds in the tumour on CE-T1WI FSBB. A two-way random intraclass correlation coefficient (ICC) was used for inter-reader agreement. Further sub-analysis of consistency was carried out depending on histology and WHO grading. The linear regression analysis (with F-test) was used to study the correlation between intratumoral vessels and microbleeds based on CE-T1WI FSBB ($\alpha$=0.05).
**Results or Findings:** Inter-reader agreements for intratumoral vessel count on CE-T1WI FSBB (ICC=0.92) and CE-T1WI (ICC=0.93) were excellent. The agreement for intratumoral microbleed count on CE-T1WI FSBB (ICC=0.99) was also excellent. There were statistically significant differences in vessel counts using Mann-Whitney U -test: image readers could identify more vessels on CE-T1WI FSBB than on CE-T1WI, particularly for meningiomas, schwannomas, gliomas, and WHO grade I tumours. The intratumoral vessels had a significant positive effect on microbleeds (microbleeds =5.024 + 1.665 × vessels; F=11.51).
**Conclusion:** More intratumoral vessels could potentially be identified using 3D CE-T1WI FSBB compared to CE-T1WI, and the number of vessels showed a positive linear relationship with the number of microbleeds, which might suggest that brain tumours with abundant blood supply are more prone to microbleeds.
**Limitations:** There was no gold standard for histopathological verification.
**Ethics committee approval:** This study was approved by the Institutional Review Board of Huashan Hospital, Fudan University (KY2021-066).
**Funding for this study:** Funding was received from Shanghai Sailing Program (No.22YF1405000).
**Author Disclosures:**
Zhentao Chen: Nothing to disclose
Jun Zhang: Nothing to disclose
Lijun Zhang: Nothing to disclose
Xin Cao: Nothing to disclose

### RPS 2511-12

**Perfusion MRI in daily practice from a radiologist's perspective: results from the Dutch multicentre PERISCOPE project**
I. Huisman[1], *W. Teunissen*[2], L. Dirven[3], L. Berkemeier[1], A. Van Der Hoorn[1], M. Smits[2], C. Periscope[2]; [1]Groningen/NL, [2]Rotterdam/NL, [3]Leiden/NL

**Purpose:** Perfusion MRI (pMRI) is one of the evaluation techniques in brain tumour follow-up. However, there is no clear consensus on its added value and no proper insights into its impact on the clinical workflow. We investigated how time-consuming pMRI is for the radiologist and assessed the degree of added diagnostic certainty.
**Methods or Backround:** We performed onsite measurements and surveys in 11 neuro-oncological centres in the Netherlands during the reading of scans for brain tumour follow-up. The live timing of pMRI reading was measured with a stopwatch. A seven-point Likert scale (1=least confident) was used to quantify the degree of certainty of the radiologist on diagnosing scans with pMRI and asked what the certainty was if pMRI was not available. Statistically significant differences were assessed with a Wilcoxon Signed Rank test.
**Results or Findings:** Eighteen radiologists participated in this study, resulting in 72 pMRI assessments. In district hospitals (21 scans), the mean reading time was 2:28 (IQR:1:14-3:02) minutes; in university hospitals (51 scans), the mean reading time was 1:37 (IQR:0:23-2:41) minutes. DSC was the most commonly used pMRI method in this study (N=65) and took a mean reading time of 02:01(IQR:0:48-2:56) minutes. Diagnostic certainty was significantly lower ($p<0.001$) if pMRI was not available versus with available pMRI with a median Likert score of 4.71(±1.85) versus 5.34(±1.57), respectively.
**Conclusion:** This study provided new insights about the time investment of reading pMRI scans, which can be used to provide clear guidance on the cost-effective implementation of pMRI for brain tumour surveillance. Moreover, the results indicate that the pMRI adds value in terms of a higher degree of certainty from a radiologist's perspective.

**Limitations:** It is currently unclear if diagnostic certainty is associated with diagnostic accuracy of pMRI.
**Ethics committee approval:** No information provided by the submitter.
**Funding for this study:** Funding was received from “Leading the Change”(80-85009-98-2008-NVvR)
**Author Disclosures:**
Wouter Teunissen: Nothing to disclose
Lea Berkemeier: Nothing to disclose
Ilse Huisman: Nothing to disclose
Anouk Van Der Hoorn: Nothing to disclose
Marion Smits: Speaker: Speaker fees from GE Healthcare and AuntMinnie (paid to institution).
Consortium Periscope: Nothing to disclose
Linda Dirven: Nothing to disclose

### RPS 2511-13

**Diagnostic validity and reliability of brain tumour reporting and data system (BT-RADS) in the management of recurrent high-grade glioma**

*N. Yahia*[1], R. N. Ahmed[2]; [1]Zagazig/EG, [2]Mosul/IQ
*(nohayahiaradio@gmail.com)*

**Purpose:** The study aimed to estimate the diagnostic accuracy and assess the reliability of BT-RADS in the management of recurrent high-grade glioma.
**Methods or Backround:** This is a prospective single-centre study that enrolled 81 patients with operated and pathologically proven high-grade glioma. The patients were submitted to magnetic resonance imaging (MRI) with contrast for follow-up. Two readers independently evaluated and analyzed the MRI images and assigned the BT-RADS category for each patient. The histopathology was used as a standard reference test for BT-RADS categories 3, 4, and 5. However, neurological clinical evaluation was used as a reference standard for BT-RADS categories 1 and 2 to estimate the diagnostic performance of the BT-RADS for predicting recurrent high-grade glioma, according to the two readers. The inter-reader agreement was assessed using the intra-class correlation test.
**Results or Findings:** Based on the patient-by-patient analysis, the current study included 42 recurrent and 39 non-recurrent high-grade glioma cases. Based on the two readers, the BT-RADS showed a sensitivity, specificity, and accuracy of 90.5% to 92.9%, 76.9% to 84.6%, and 83.9% to 88.9%, respectively. Both readers agreed that BT-RADS 3b was the optimal cut-off for predicting recurrent high-grade glioma. The overall inter-reader agreement was excellent at 0.915 (0.871–0.945, P 0.001).
**Conclusion:** The BT-RADS is a promising diagnostic tool for predicting recurrent high-grade glioma with high diagnostic accuracy and reliability. BT-RADS helps neuro-oncologists in clinical decision-making for better patient outcomes.
**Limitations:** First, the study was in a single centre, so we recommend further prospective multicentric studies with a larger sample size. Second, images were analyzed by highly experienced neuro-radiologists that might affect the diagnostic performance of the BT-RADS.
**Ethics committee approval:** Reference number: 9559.
**Funding for this study:** No funding was received for this study.
**Author Disclosures:**
Rasha Nadeem Ahmed: Nothing to disclose
Noha Yahia: Nothing to disclose

**13:00-14:30 Research Stage 4**

### Research Presentation Session: Cardiac

### RPS 2503

### Artificial intelligence in cardiovascular imaging

**Moderator**
H. Cochet; Pessac/FR
**Author Disclosures:**
Hubert Cochet: Consultant: Fineheart, Guerbet; Founder: inHEART; Share Holder: inHEART; Speaker: Siemens Healthineers, Abbott, Biosense Webster, Biotronik

### RPS 2503-2

**DEMOCRAT: deep learning for contrast medium and radiation reduction in coronary CT**

*G. Tremamunno*, D. De Santis, T. Polidori, L. Pugliese, C. Santangeli, N. Ubaldi, G. Piccinni, D. Caruso, A. Laghi; Rome/IT
*(giuseppe.tremamunno@uniroma1.it)*

**Purpose:** To evaluate the impact of high-strength deep learning image reconstructions (DLIR-H) applied to 80-kVp coronary CT angiography (CCTA) on radiation dose, contrast medium, and image quality, compared with the conventional 100-kVp protocol.
**Methods or Backround:** From June to October 2022, clinically indicated CCTA of consecutive patients with BMI < 30 kg/m2 were prospectively included and randomly assigned (1:1 ratio) into three groups: group A (100 kVp, ASiR-V 50%, and iodine delivery rate [IDR] =1.6); group B (80 kVp, DLIR-H, and IDR =1.4), and group C (80 kVp, DLIR-H, and IDR =1.2). Radiation and contrast dose, objective image quality (vascular attenuation, image noise, contrast-to-noise ratio [CNR], signal-to-noise ratio [SNR]), and subjective image quality were compared among the three groups.
**Results or Findings:** 64 CCTA were evaluated. Group B and C significantly reduced radiation dose compared to group A (1.97 ±0.8 mSv and 1.99 ± 0.6 mSv, respectively, VS 3.2 ± 0.8 mSv; P <0.001). They also achieved significantly higher attenuation, SNR, CNR, and a subjective image quality score compared to group A (all P <0.001). All groups had comparable background noise, with group B showing lower noise only in the aorta and the circumflex artery compared to group A (P <0.05). Group B slightly outperformed group C in subjective and objective image quality; however, no statistical differences have been found. Group C was performed with the lowest contrast media dose (44.5 ± 3.6 mL) compared to groups A and B (54.4 ± 5.4mL and 52.6 ± 5.3 mL; P <0.001).
**Conclusion:** DLIR applied to 80kVp CCTA with 1.4 and 1.2 IDR significantly reduces radiation and contrast medium dose while improving image quality compared to conventional 100kVp 1.6 IDR protocol in non-obese patients.
**Limitations:** Diagnostic accuracy has not been evaluated.
**Ethics committee approval:** No information provided by the submitter.
**Funding for this study:** No funding was received for this study.
**Author Disclosures:**
Luca Pugliese: Nothing to disclose
Damiano Caruso: Nothing to disclose
Nicolò Ubaldi: Nothing to disclose
Domenico De Santis: Nothing to disclose
Giuseppe Tremamunno: Nothing to disclose
Curzio Santangeli: Nothing to disclose
Giulia Piccinni: Nothing to disclose
Tiziano Polidori: Nothing to disclose
Andrea Laghi: Nothing to disclose

### RPS 2503-3

**CT angiography after percutaneous coronary intervention: assessment of the image quality of deep learning image reconstruction on coronary artery in-stent restenosis**

Y. Wang, *W. Wu*, Y. Zhang; Zhengzhou/CN

**Purpose:** To evaluate the image quality of the deep learning image reconstruction (DLIR) algorithm on coronary artery in-stent restenosis (ISR) after percutaneous coronary intervention (PCI) compared with iterative reconstruction technique (ASiR-V).
**Methods or Backround:** 40 patients who were diagnosed ISR by coronary angiography (CAG) and underwent CT angiography after PCI were retrospectively involved in this study. All patients were examined on a 256 slice CT scanner with a prospective axial ECG-triggering protocol. Adaptive Statistical Iterative reconstruction-VEO Standard-Definition, (ASIR-V 50% SD, group A), high-definition (ASIR-V 50% HD, group B) convolution kernel, respectively] and medium level DLIR (DLIR-M, group C) were used for image reconstruction. Quantitative image assessment including the CT attenuation and image noise (SD) of the major coronary arteries, and aortic root (AO). Signal-to-noise ratio (SNR) and contrast-to-noise ratio (CNR) in coronary arteries were calculated. Qualitative image analysis was performed with ASiR-V and DLIR, by two experienced coronary CCTA radiologists. Images were assessed using a 5-point scale (5, excellent; and 1, not acceptable) with the focus on image noise, coronary wall definition, structure visibility, lumen interpretability, and image conspicuity.
**Results or Findings:** Image noise of the DLIR-M group was significantly lower and SNR as well as CNR were significantly higher than the ASIR-V 50% SD and ASIR-V 50% HD groups in AO. Image noise of DLIR-M group was significantly lower and SNR as well as CNR were significantly higher than the ASIR-V 50% SD group on the mean arteries. The DLIR-M group had the highest qualitative scores on image noise, coronary wall definition, structure visibility, lumen interpretability and image conspicuity.
**Conclusion:** Compared with ASIR-V, DLIR can significantly improve image quality DLIR reconstruction.
**Limitations:** CCTA with DLIR could improve the image quality of radiological examination after PCI for diagnosing ISR.
**Ethics committee approval:** No information provided by the submitter.
**Funding for this study:** No funding was received for this study.
**Author Disclosures:**
Wenjie Wu: Nothing to disclose
Yiran Wang: Nothing to disclose
Yonggao Zhang: Nothing to disclose

### RPS 2503-4

**A comparison between iterative reconstruction and deep learning algorithm on plaque volume quantification**

*G. Chierchia*[1], G. Muscogiuri[1], P. Marra[2], E. Çollaku[2], A. Senatieri[1], E. Faietti[2], P. A. Bonaffini[2], S. Sironi[2]; [1]Milan/IT, [2]Bergamo/IT
*(g.chierchia@campus.unimib.it)*

**Purpose:** Compare volumes of low-density, fibrofatty, and calcific plaques between images reconstructed with iterative reconstruction (ASIR50%-50%FBP) and with a deep learning algorithm (DLIR) on low, medium and high levels.
**Methods or Backround:** 33 patients with known CAD were selected, and multiplanar reconstruction was performed with ASIR 50% images, DLIR-low (DLIR-L), DLIR-medium (DLIR-M), and DLIR-high (DLIR-H). Volumes for low-density, mixed, calcified plaque were estimated with a post-processing software for each coronary segment per vessel, according to the American Heart Association (left anterior descending artery (LAD); left circumflex artery (LCX) right coronary artery (RCA)); also the total plaque burden was calculated. A $p<0.05$ was considered significant.
**Results or Findings:** The low-density plaque showed significantly lower p-values in DLIR reconstruction compared to ASIR 50% on proximal RCA (DLIR-L $p<0.01$, DLIR-M $p=0.017$, DLIR-H<0.01), proximal LAD (DLIR-L $p=0.039$, DLIR-M $p<0.01$, DLIR-H $p<0.01$), LCX (DLIR-L $p=0.02$, DLIR-M $p<0.01$, DLIR-H $p<0.01$), on whole vessel analysis on LAD (DLIR-L $p=0.01$, DLIR-M $p=0.01$, DLIR-H $p<0.01$), on RCA (DLIR-L $p=0.02$, DLIR-H $p=0.02$), on LCX for DLIR-M ($p=0.02$, DLIR-H $p<0.01$) and for low-density plaque burden (DLIR-L $p<0.01$, DLIR-M $p=0.01$, DLIR-H $p<0.01$). Also, mixed plaque volumes were found to be significantly lower on proximal RCA for DLIR-H ($p<0.01$), on the common left artery for DLIR-H ($p=0.04$), and proximal LAD for DLIR-M ($p=0.02$) and DLIR-H ($p<0.01$). Whole vessel analysis showed a significant difference for LAD (DLIR-M $p=0.04$, DLIR-H $p=0.01$) on the RCA (DLIR-H $p=0.04$) for plaque burden of mixed plaque (DLIR-H $p=0.01$). Calcific plaque volume was found to be significantly lower only in the distal LAD (DLIR-M $p=0.03$), but also the whole calcific burden was lower on DLIR-M reconstructions ($p=0.03$).
**Conclusion:** While the study was performed on a limited number of patients, it seems that deep learning reconstructions may play a role in plaque volume quantification of fibrofatty and mixed plaques. A study with larger numbers might be needed.
**Limitations:** A study with larger numbers might be needed.
**Ethics committee approval:** No information provided by the submitter.
**Funding for this study:** No funding was received for this study.
**Author Disclosures:**
Sandro Sironi: Nothing to disclose
Paolo Marra: Nothing to disclose
Pietro Andrea Bonaffini: Nothing to disclose
Gregorio Chierchia: Nothing to disclose
Elona Çollaku: Nothing to disclose
Alberto Senatieri: Nothing to disclose
Elena Faietti: Nothing to disclose
Giuseppe Muscogiuri: Nothing to disclose

### RPS 2503-5

**Analysis of a novel multitask, deep-learning algorithm for automated coronary artery disease classification: the impact of AI assistance on reader accuracy and variability**

*R. Farì*[1], M. van Assen[2], P. L. N. von Knebel Doeberitz[2], B. Bottcher[2], M. Schoebinger[3], G. Fung[3], C. N. De Cecco[2]; [1]Modena/IT, [2]Atlanta, GA/US, [3]Forchheim/DE
*(dott.roberto.fari@gmail.com)*

**Purpose:** This study aims to evaluate the impact of AI-assisted reading (AIAR) on the agreement between readers using a heterogeneous multivendor coronary CT angiography (CCTA) dataset composed of images with different quality.
**Methods or Backround:** A total of 623 patients who underwent clinical CCTA were included in this retrospective study. An expert reader (R1) analysed all CCTAs to assess CADRADS on a per-segment basis. Variability analysis was performed with a second expert reader (R2) who performed the same analysis on a randomly selected group of 274 patients. CADRADS scores were extracted from radiologist reports (RR) when available (362 patients). The algorithm used is a novel, fully-automated multitask AI algorithm which performs simultaneous detection, classification, and quantification of coronary lesions. A second AIAR was performed by R1 on 623 patients. R2 performed an AIAR in a subgroup of patients (n=30) where R1 disagreed with AI about obstructive vs non-obstructive disease.
**Results or Findings:** Spearman's correlation between R1 and R2's manual reading was 0.899 and increased to 0.949 when R1 used AI assistance. The Spearman's correlation between R1 and RR was 0.899 for manual reading and increased to 0.938 when R1 used AI assistance. In the subgroup of 30 patients with disagreement between R1 and AI, the agreement was low between human readers (R1 and R2 manual reading Spearman's correlation = 0.688). When both readers used AI assistance, the agreement between them greatly improved (R1 AIAR and R2 AIAR Spearman's correlation = 0.975).
**Conclusion:** The use of a fully automated DL algorithm assistance for CADRADS assessment in CCTA improved the agreement between readers, both in the general population and in cases with high variability.
**Limitations:** The limitations of this study are the retrospective perspective, the high number of CADRADS 0, and the only three partial readers.
**Ethics committee approval:** No information provided by the submitter.
**Funding for this study:** Funding was received from Siemens Healthineers.
**Author Disclosures:**
Marly van Assen: Other: Siemens Healthineers
Carlo Nicola De Cecco: Other: Siemens Healthineers
Roberto Farì: Other: Siemens Healthineers
George Fung: Other: Siemens Healthineers
Max Schoebinger: Other: Siemens Healthineers
Philipp Lorenzo Nikolau von Knebel Doeberitz: Other: Siemens Healthineers
Benjamin Bottcher: Other: Siemens Healthineers

### RPS 2503-6

**Impact of super resolution deep learning reconstruction with 1024 matrix in potential CAD-RADS 3 lesions: retrospective analysis of 50 cases**

*M. Ohana*[1], F. Tatsugami[2], A. Labani[1], W. Fukumoto[2], S. El Ghannudi[1], A. Taniguchi[3], K. Haioun[3], K. Awai[2], C. Roy[1]; [1]Strasbourg/FR, [2]Hiroshima/JP, [3]Otawara/JP
*(mickael.ohana@gmail.com)*

**Purpose:** CAD-RADS 3 lesion in Coronary CT Angiography (CCTA) is defined as an intermediate (50-69%) stenosis. Whether the use of a Super Resolution Deep Learning Reconstruction (SR-DLR) algorithm could modify the adjudication of these intermediate lesions, particularly when heavily calcified, is unknown. Our primary objective is therefore to compare the classification of potential CAD-RADS 3 lesions by expert readers when using conventional DLR with a 512 matrix (i.e. current reference standard) and SR-DLR with a 1024 matrix.
**Methods or Backround:** Fifty non-consecutive CCTA with potential intermediate stenosis – as based on the initial reading – acquired on a 4th generation 320-row scanner were retrospectively included and reconstructed using DLR (512 matrix) and SR-DLR (1024 matrix). The 100 datasets were anonymised and randomised. Four expert radiologists from two different institutions independently read all reconstructions and were asked to adjudicate the level of the stenosis. A statistical multivariate model was used to assess the implications of reconstruction, reader, stenosis localisation and stenosis type.
**Results or Findings:** Of the 50 potential intermediate stenosis, 29 were due to calcified plaques, 12 due to mixed plaques and 9 were due to non-calcified plaques. When merging all readings, SR-DLR was consistent with DLR in 66% of cases, downgraded the classification in 24% and upgraded it in 10%. Maximum discrepancy with DLR was observed in calcified plaques.

**Conclusion:** The use of SR-DLR with 1024 matrix significantly modifies the way expert readers adjudicate potential intermediate stenosis on CCTA. This effect tends towards downgrading of the lesion, and appears statistically more pronounced in calcified plaques.
**Limitations:** While not in the design of the present study, the lack of a gold standard (invasive coronary angiography, only available for five cases) limits the analysis of these results.
**Ethics committee approval:** This study was approved by the IRB of Strasbourg University.
**Funding for this study:** Not applicable
**Author Disclosures:**
Wataru Fukumoto: Nothing to disclose
Aissam Labani: Nothing to disclose
Soraya El Ghannudi: Nothing to disclose
Fuminari Tatsugami: Nothing to disclose
Karim Haioun: Employee: Canon Medical Systems Japan
Catherine Roy: Nothing to disclose
Kazuo Awai: Nothing to disclose
Akira Taniguchi: Employee: Canon Medical Systems Japan
Mickaël Ohana: Speaker: Boehringer Ingelheim Speaker: Canon Medical Systems Europe

### RPS 2503-7

**Machine learning prediction of obstructive coronary artery disease in PLWH: a new model based on HIV-specific factors.**

*H. Liu*, H. Huo, J. Zhou, X. Peng, H. Li, Y. Zheng, S. Jin, H. Ding, T. Liu; Shenyang City, Liaoning Province/CN

**Purpose:** The traditional models for evaluating the pretest probability of obstructive coronary artery disease (CAD) are only applicable to the general population. However, people living with HIV (PLWH) experience a higher CAD risk. We aim to develop a machine learning (ML) model to predict obstructive CAD (≥50% stenosis) on coronary CT angiography (CCTA) in PLWH.
**Methods or Backround:** We compared the ML model established by logistic regression with traditional models (CAD consortium basic, CAD consortium clinical and CONFIRM score) in a retrospective cohort of PLWH who underwent CCTA. In addition, coronary artery calcium score (CACS) was added to each model for comparison. Predictive performance of the above models was evaluated according to Delong et al.
**Results or Findings:** Overall, 163 PLWH (age 50±11 years, 83.4% males) were analysed, of whom 18 (11.0%) had obstructive CAD. The ML model (AUC of 0.847) had the highest discrimination for obstructive CAD compared with CAD consortium basic, CAD consortium clinical and CONFIRM score (AUC of 0.690, 0.779, and 0.809, respectively). The ML with CACs (AUC of 0.946,) demonstrated the best performance. P<0.05 for all comparisons.
**Conclusion:** The ML model incorporating cardiovascular risk factors and HIV-specific factors can more accurately estimate the pretest likelihood of obstructive CAD in PLWH than traditional models. And the best prediction was achieved with the addition of CACs to the ML model. In clinical practice, the use of such models can improve risk stratification in HIV populations and help guide downstream management.
**Limitations:** Not applicable
**Ethics committee approval:** Not applicable
**Funding for this study:** Not applicable
**Author Disclosures:**
Yue Zheng: Nothing to disclose
Hui Liu: Nothing to disclose
Han Li: Nothing to disclose
Haibo Ding: Nothing to disclose
Xin Peng: Nothing to disclose
Huaibi Huo: Nothing to disclose
Shiqi Jin: Nothing to disclose
Ting Liu: Nothing to disclose
Jie Zhou: Nothing to disclose

### RPS 2503-8

**CT-derived radiomic features of epicardial adipose tissue as a marker of coronary artery disease**

*N. Mobini*, C. B. Monti, D. Capra, F. Secchi, F. Sardanelli; Milan/IT

**Purpose:** Epicardial adipose tissue (EAT) has recently been recognised as a biomarker of cardiovascular disease, particularly coronary artery disease (CAD). In this study, we explored the feasibility of radiomic analysis derived from EAT of cardiac computed tomography (CT) in differentiating CAD from non-CAD patients.
**Methods or Backround:** We retrospectively reviewed consecutive patients who had undergone cardiac CT for CAD assessment at our institution. A total of 286 radiomic features were extracted using unenhanced CT from single-slice manual EAT segmentations. Five high-ranked features were retained through Fast Correlation Based Filter (FCBF) which is an entropy-based selection and reduces redundant, highly correlated features. Then, three classification models including Logistic Regression (LR), Random Forest (RF), and Support Vector Machine (SVM) were trained with stratified 10-fold cross-validation to predict the presence of CAD, derived from contrast-enhanced CT imaging. Average area under the receiver operating characteristics curve (AUC) was used to assess the diagnostic performance of the models.
**Results or Findings:** The study comprised 580 patients (217 women), with a median age of 65 years (interquartile range: 73–56), and CAD was reported in 228 (39%) of them at CT scan. High-ranked selected features included two clinical variables (age, sex), two first order features (maximum, mean absolute deviation), and one second order textural feature (GLCM-entropy). The developed CAD classification models demonstrated mean AUC values of 0.74 for the LR, 0.80 for the RF, and 0.76 for the SVM, respectively.
**Conclusion:** EAT radiomic features derived from a single, unenhanced CT slice, paired with age and sex, can discriminate between CAD and non-CAD patients with good performances.
**Limitations:** This was a single-centre study and external validation is needed.
**Ethics committee approval:** The study was approved by the local ethics committee.
**Funding for this study:** No funding was received for this study.
**Author Disclosures:**
Francesco Secchi: Nothing to disclose
Francesco Sardanelli: Speaker: bureau Advisory Board: Bayer Grant Recipient: bureau Advisory Board: General Electric Advisory Board: Bracco
Nazanin Mobini: Nothing to disclose
Caterina Beatrice Monti: Nothing to disclose
Davide Capra: Nothing to disclose

### RPS 2503-9

**Redefining hypertrophic cardiomyopathy using AI: a new personalised definition of inappropriate hypertrophy**

*H. Shiwani*[1], L. Lopes[1], G. Joy[1], S. Castelletti[2], C. Manisty[1], P. Kellman[3], G. Captur[1], J. Moon[1], R. Davies[1]; [1]London/UK, [2]Milan/IT, [3]Bethseda, MD/US

**Purpose:** The diagnosis of hypertrophic cardiomyopathy (HCM) with a uniform threshold of ≥15mm of maximal wall thickness (MWT) is biased leading to under-diagnosis in younger/smaller/females and over-diagnosis in older/larger/males. We have redefined pathological left ventricular hypertrophy (LVH) by calculating a MWT threshold adjusted for age, sex, and body-surface-area (BSA) facilitated by AI.
**Methods or Backround:** The reference range was derived from cardiac MRIs of 4,118 healthy UK Biobank participants. Cardiac MRIs from 43,329 UK Biobank participants, 258 local healthy subjects and 149 HCM patients composed the validation dataset. All scan analysis was automated using published AI algorithms to measure MWT. Multiple regression models were created using age, sex and BSA. The upper limit of normal was derived using the mean+1.96SD of the residual values.
**Results or Findings:** ~40% of variation in wall thickness is explained by sex, age, and body surface area (r2=0.39). The predicted MWT was calculated as 3.90*BSA+0.03*Age+2.06 (males) and 3.20*BSA+0.07*Age–0.34 (females). The upper limit was calculated as 1.3*Predicted MWT. The performance was equivalent between 15mm (sensitivity 91.9%, specificity 95.7%) and an adjusted threshold (sensitivity 90.6%, specificity 96.4%) across the UK Biobank, healthy volunteers and HCM patients. Across the UK Biobank, 15mm has a large male skew; 8% of males (1642/20,860) to 1% of females (212/22,379). With an adjusted threshold, there is no skew; 4% of males (735/20,860) and 4% of females (806/22,379) are classified with inappropriate hypertrophy.
**Conclusion:** 15mm has an inherent skew diagnosing fewer female/smaller/younger patients. There are segments of the population in which there may be under-diagnosis (5% women) or over-diagnosis (4% men). With an adjusted threshold, we rectify this whilst maintaining high specificity for HCM. This has implications for treatment, as current criteria may bias ICD (implantable cardioverter defibrillator) placement to prevent sudden cardiac death.
**Limitations:** A multi-ethnic international cohort of patients is required to validate these findings.
**Ethics committee approval:** No information provided by the submitter.
**Funding for this study:** No funding was received for this study.
**Author Disclosures:**
Peter Kellman: Nothing to disclose
Silvia Castelletti: Nothing to disclose
Rhodri Davies: Nothing to disclose
Charlotte Manisty: Nothing to disclose
Luis Lopes: Nothing to disclose
George Joy: Nothing to disclose
Hunain Shiwani: Nothing to disclose
James Moon: Nothing to disclose
Gabriella Captur: Nothing to disclose

### RPS 2503-10

**Automated detection and quantification of myocardial scar using AI-powered joint bright- and black-blood LGE imaging**

*A. Bustin*[1], I. Ribal[1], G. Montier[1], J-D. Maes[1], M. Montaudon[1], G. Dournes[1], P. Jais[1], M. Stuber[2], H. Cochet[1]; [1]Pessac/FR, [2]Lausanne/CH
*(aurelien.bustin@ihu-liryc.fr)*

**Purpose:** To introduce a framework for fully automated scar detection and quantification combining novel joint bright- and black-blood LGE imaging (SPOT) with artificial intelligence (AI)-powered analysis.
**Methods or Backround:** 300 patients (83 females, 55±15 years) with myocardial infarction underwent CMR at 1.5T (Siemens, Aera). Short-axis 2D whole-heart PSIR (reference) and SPOT images were collected under breath-hold 15 min post-injection. SPOT jointly collects bright-blood (wall visualization) and black-blood (scar visualization) images by combining inversion-recovery with T1-rho pulses. Two radiologists provided ground truth left ventricular (LV) wall and scar contours on both sequences using CVI42. A 2D U-net model was trained on a subset of 288 patients to automatically extract wall contours from bright-blood SPOT images. Contours were propagated to black-blood SPOT images and scar was extracted as voxels with a signal 12 standard deviation above the mean intensity in a region of interest automatically defined in the LV cavity. Total infarct mass, volume and transmurality were automatically measured. Bland-Altman analyses and correlation coefficients were used to assess inter-reader agreements in 12 patients.
**Results or Findings:** SPOT datasets were automatically processed in ~10s (manual: 17±7 min). As compared to PSIR, SPOT images showed higher inter-reader agreement in manual wall segmentation (R2=0.92, ICC=0.83 vs R2=0.79, ICC=0.58). There was a nonsignificant bias between manual and automated LV mass values (-2.5g, 95%CI: -20 to +15g, R2=0.92, P=0.35). The approach resulted in a fully automated quantification of scar mass with excellent correlation to manual measurements (R2=0.93, bias: -0.39g, 95%CI: -9.5 to +8.7g, P<0.01) and allowed for automated mapping of scar presence (89% accuracy) and transmurality (75% accuracy) across AHA segments.
**Conclusion:** The AI-powered SPOT imaging framework allows for fast, robust, and fully-automated detection and quantification of post-infarction scar.
**Limitations:** For this study, a 3D extension is warranted.
**Ethics committee approval:** No information provided by the submitter.
**Funding for this study:** Funding was received for this study. The grant number reads as follows: ANR-11-EQPX-0030, ANR-21-CE17-0034-01, ANR-10-IAHU04-LIRYC, and ERC-715093.
**Author Disclosures:**
Geraldine Montier: Nothing to disclose
Indra Ribal: Nothing to disclose
Aurelien Bustin: Nothing to disclose
Gaël Dournes: Nothing to disclose
Jean-David Maes: Nothing to disclose
Hubert Cochet: Nothing to disclose
Michel Montaudon: Nothing to disclose
Pierre Jais: Nothing to disclose
Matthias Stuber: Nothing to disclose

### RPS 2503-11

**Radiomics-based detection of acute myocardial infarction at non-contrast enhanced midventricular short-axis cine images**

*B. Vande Berg*[1], J. Bogaert[1], S. Tilborghs[1], A. Cernicanu[2], T. Dresselaers[1]; [1]Leuven/BE, [2]Eindhoven/NL

**Purpose:** Our goal is to assess the feasibility of detecting acute myocardial infarcts based on radiomics analysis of non-contrast midventricular short-axis (SAX) balanced steady-state free precession (bSSFP) cine images.
**Methods or Backround:** Non-contrast midventricular single slice SAX bSSFP images (Ingenia 1.5T) of 99 consecutive AMI patients and 49 control subjects were used. In 83 patients (84%) the SAX covered the infarcted myocardium as visualised at late gadolinium-enhanced CMR. End-diastolic (ED) and end-systolic (ES) SAX frames were manually and automatically (Neosoft suiteHEART®) segmented. Radiomic feature extraction was performed (PyRadiomics®; no prior filters; 25 bins, shape features excluded) followed by feature normalisation. Next, the top three features were selected based on pairwise correlation and optimal classification results (train/validate set) using a support vector machine approach. Finally, classification accuracy (CA) was assessed on the test data.
**Results or Findings:** The classification accuracy (CA) of visual analysis by a radiologist of the ED and ES images was respectively 65% and 78%. The combination of run-length, non-uniformity, cluster shade and median features yielded the most accurate machine learning algorithm for the evaluation of the manually segmented ES images (CA=0.846, F1-score=0.847, Precision=0.852, and Specificity=0.854). For ED, the top features were run-length, non-uniformity correlation and skewness, but this feature combination performed worse than the ES features, with lower CA and F1-score (0.769 and 0.770, respectively). In contrast, the manual and automated segmentation resulted in similar features and classification results.
**Conclusion:** Detection of AMI using radiomics features of non-contrast mid-ventricular SAX cine images is feasible, and more robust on ES than ED. Automated contouring did not influence the performance of the algorithm.
**Limitations:** This is a study with single-centre, single-slice (2D) data with a heterogeneous AMI group.
**Ethics committee approval:** The Research Ethics Committee UZ / KU Leuven granted favourable advice to the proposed study, as it was described in the protocol. The commission is of the opinion that from an ethical standpoint, there are no objections to the proposed study. The study was approved on October 12, 2021.
**Funding for this study:** No funding was received for this study.
**Author Disclosures:**
Tom Dresselaers: Nothing to disclose
Alexandru Cernicanu: Nothing to disclose
Baptiste Vande Berg: Nothing to disclose
Sofie Tilborghs: Nothing to disclose
Jan Bogaert: Nothing to disclose
